# Off-target effects of the lysosomal acid lipase inhibitors Lalistat-1 and Lalistat-2 on neutral lipid hydrolases

**DOI:** 10.1016/j.molmet.2022.101510

**Published:** 2022-04-30

**Authors:** Ivan Bradić, Katharina B. Kuentzel, Sophie Honeder, Gernot F. Grabner, Nemanja Vujić, Robert Zimmermann, Ruth Birner-Gruenberger, Dagmar Kratky

**Affiliations:** 1 Gottfried Schatz Research Center, Molecular Biology and Biochemistry, Medical University of Graz, Graz, Austria; 2 Diagnostic and Research Institute of Pathology, Medical University Graz, Graz, Austria; 3 Institute of Molecular Biosciences, University of Graz, Graz, Austria; 4 BioTechMed Graz, Graz, Austria; 5 Institute of Chemical Technologies and Analytics, TU Wien, Vienna, Austria

**Keywords:** LAL, Lipolysis, Lipid hydrolase activity, ATGL, HSL, MGL

## Abstract

**Objectives:**

Lysosomal acid lipase (LAL) is the key enzyme, which degrades neutral lipids at an acidic pH in lysosomes. The role of LAL in various cellular processes has mostly been studied in LAL-knockout mice, which share phenotypical characteristics with humans suffering from LAL deficiency. In vitro, the cell-specific functions of LAL have been commonly investigated by using the LAL inhibitors Lalistat-1 and Lalistat-2.

**Methods:**

We performed lipid hydrolase activity assays and serine hydrolase-specific activity-based labeling combined with quantitative proteomics to investigate potential off-target effects of Lalistat-1 and -2.

**Results:**

Pharmacological LAL inhibition but not genetic loss of LAL impairs isoproterenol-stimulated lipolysis as well as neutral triglyceride and cholesteryl ester hydrolase activities. Apart from LAL, Lalistat-1 and -2 also inhibit major cytosolic lipid hydrolases responsible for lipid degradation in primary cells at neutral pH through off-target effects. Their binding to the active center of the enzymes leads to a decrease in neutral lipid hydrolase activities in cells overexpressing the respective enzymes.

**Conclusions:**

Our findings are critically important since they demonstrate that commonly used concentrations of these inhibitors are not suitable to investigate the role of LAL-specific lipolysis in lysosomal function, signaling pathways, and autophagy. The interpretation of their effects on lipid metabolism should be taken with caution and the applied inhibitor concentrations in cell culture studies should not exceed 1 μM.

## Highlights

• Lysosomal acid lipase inhibitors Lalistat-1/2 interfere with neutral lipolysis.
• Lalistat-1/2 bind to active center of neutral lipid hydrolases.
• Off-target effects may bias results of acid versus neutral lipolysis studies.
• Lalistat-1/2 can only be used with caution at concentrations <1 μM.

## 1 Introduction

Lysosomal acid lipase (LAL) is the only known enzyme responsible for the degradation of cholesteryl esters (CE), triacylglycerols (TG) [1,2], diacylglycerols (DG) [2], and retinyl esters (RE) [3] in lysosomes at pH ∼4.5 (reviewed in [4]). In humans, LAL deficiency (LAL-D) alters intracellular cholesterol homeostasis [1] and causes ectopic lipid accumulation, alteration of plasma lipids, and premature mortality [5]. Depending on the level of residual enzymatic activity, LAL-D can be subclassified as Wolman disease (WD) or CE storage disease (CESD) [6,7]. Patients with a complete loss of LAL activity develop WD and die within the first year of life, whereas CESD patients may retain up to 12% residual enzymatic activity and survive into adulthood [5,[8], [9], [10]]. LAL-deficient (Lal−/−) mice develop a phenotype more similar to CESD than WD despite complete loss of LAL activity. These animals suffer from dyslipidemia, progressive hepatosplenomegaly, loss of white adipose tissue (WAT), and accumulation of neutral lipids in the liver, spleen, and small intestine [[11], [12], [13]].

In addition to Lal−/− mice and genetically manipulated cells, the pharmacological LAL inhibitors Lalistat-1 (L1) and Lalistat-2 (L2) were suggested as a suitable tool to study cell-specific consequences of LAL-D in vitro [[14], [15], [16], [17]]. L1 or L2 have been used in >50 scientific studies to investigate defective lysosomal lipid hydrolysis in various cellular processes such as autophagy and lipolysis [[18], [19], [20], [21], [22], [23], [24], [25], [26]]. In the clinical setting, L2 found its application to specifically measure LAL activity in human blood samples and cells with the goal of simpler and more cost-effective diagnosis of LAL-D [[27], [28], [29]]. L1 and L2 have been shown to competitively inhibit LAL activity without affecting the activities of the extracellular lipolytic enzymes human pancreatic lipase and bovine lipoprotein lipase [30,31]. Despite numerous claims of their high specificity for LAL, their effects on neutral intracellular lipases have never been studied to our knowledge. Lipases in the cytoplasm hydrolyze lipid droplet-associated TG at pH 7 in a process called neutral lipolysis [32]. Adipose triglyceride lipase (ATGL) together with its co-activator CGI-58 (also known as ABHD5) initiates TG hydrolysis to form DG and free fatty acid (FFA) [33,34]. The process is completed by hormone-sensitive lipase (HSL) and monoacylglycerol lipase (MGL) by consecutively hydrolyzing DG and monoacylglycerols (MG) to glycerol and FFA. HSL exhibits much broader substrate specificity than ATGL and degrades beside DG also TG, MG [35], CE [36], and RE [37]. Since the enzymes involved in neutral TG and CE hydrolysis degrade the same substrates as LAL only in different compartments of the cell and cross-communicate [4], meaningful conclusions depend on a high selectivity of L1 and L2 for LAL, while exerting minimal to no effect on the major neutral TG and CE hydrolases ATGL and HSL [38]. Otherwise, possible off-target effects of the inhibitors could yield misleading results and conclusions in studies investigating neutral and acid lipolysis.

In the present study, we demonstrate that L1 and L2 impair overall lipolysis. In addition to LAL, L2 inhibits HSL, MGL, ATGL, and other lipid hydrolases in mouse cells and tissues. L1 inhibited them as well but to a lesser extent. In addition, L1 and L2, inhibited human ATGL and HSL. Therefore, the impact of these inhibitors and the interpretation of their effects on lipid metabolism should be taken with caution.

## 2 Material and methods

### 2.1 Animals

Tissues and primary cells were isolated from Lal−/− [11], and atLal−/− mice and their corresponding WT littermates. atLal−/− mice were generated by crossing Lal-floxed mice (Lipa^tm1a(EUCOMM)Hmgu^) with transgenic mice expressing the Cre recombinase under the control of the adiponectin promoter [39]. All mice were backcrossed to a C57BL/6J background for at least 6 generations and maintained on a regular 12 h light/12 h dark cycle in a clean and temperature-controlled (22 °C ± 1 °C) environment with unlimited access to chow diet (Altromin 1324, Lage, Germany) and water. All animal experiments were performed according to the European Directive 2010/63/EU in compliance with national laws and approved by the Austrian Federal Ministry of Education, Science and Research, Vienna, Austria (BMWFW-66.010/0197-WF/V/3b/2017).

### 2.2 Isolation and differentiation of SVC

SVC were isolated as described previously [40] with minor modifications. Briefly, subcutaneous WAT (sWAT) from WT and atLal−/− mice was dissected and digested in medium containing 1.5 U/ml Collagenase D (Roche Diagnostics, Mannheim, Germany), 2.4 U/ml Dispase II (Sigma–Aldrich, St. Louis, MO), and 10 mM CaCl_2_ (Sigma–Aldrich, St. Louis, MO) at 37 °C for 40 min under continuous shaking. Tissue digestion was terminated by the addition of complete DMEM/F12 containing Glutamax (LifeTechnology, Carlsbad, CA) supplemented with 10% FBS and 1% penicillin/streptomycin. The cell suspension was filtered (70 μm) and centrifuged at 700×*g* for 10 min. Cells were grown until ∼80% confluency before being seeded for differentiation. Two days after SVC reached confluency (day 0), complete DMEM/F12 was replaced by an induction medium containing complete DMEM/F12 supplemented with 1 μM dexamethasone, 0.5 mM IBMX, 1 μg/ml insulin, and 1 μM rosiglitazone. The induction medium was replaced on day 3 with complete DMEM/F12 containing 1 μg/ml insulin. From day 5–7, cells were maintained in complete DMEM/F12. Throughout differentiation, cells isolated from WT mice were treated with 30 μM L2 (#1234569-09-5, Sigma–Aldrich, St. Louis, MO) or 0.02% DMSO as control at day 0, 3, and 5. On day 7, mature adipocytes were harvested for further analyses.

### 2.3 Lipolysis measurement in adipocytes

Lipolysis was measured after a 1 h pre-incubation of SVC fully differentiated into adipocytes in FBS-free DMEM/F12 supplemented with 2% FA-free BSA (Biowest, Nuaillé, France) as described previously [41]. Briefly, cells were stimulated with 10 μM isoproterenol (Merck, Darmstadt, Germany), and FA release was measured in the supernatant using the NEFA-HR kit (Fujifilm Wako Chemicals Europe, Neuss, Germany) according to the manufacturer's protocol.

### 2.4 Isolation and differentiation of BMDM

After cervical dislocation, tibia and femur of WT and Lal−/− mice were dissected and rinsed with PBS containing 1 mM EDTA. The bone marrow was then filtered (70 μm) and the cell suspension was centrifuged at 145×*g* for 5 min at room temperature. Red blood cells were lysed by addition of ACK buffer (145 mM NH_4_Cl, 10 mM KHCO_3_, 100 μM EDTA). Cells were seeded in cell culture plates and differentiated into macrophages in high-glucose DMEM (LifeTechnology, Carlsbad, CA) containing 10% conditioned L929 cell medium (#85011425, ECACC, UK Health Security Agency, Salisbury, UK), 10% lipoprotein-deficient serum, and 1% penicillin/streptomycin. On day 5 of differentiation, the medium was replaced with complete DMEM and a portion of the cells was treated with different concentrations (0.1 μM, 1 μM, 10 μM, 30 μM, and 100 μM) of either L1 (501,104-16-1, Tocris Bioscience, Bristol, UK), L2, or 0.02% DMSO as control for 20 h. The remaining cells were left untreated and used for inhibitor treatment of lysates. All experiments were performed on day 6 of differentiation.

### 2.5 Oil red O (ORO) staining

Cells were fixed in 10% formalin in PBS for 30 min. Lipids were stained with an ORO solution (0.25% ORO in 60% isopropyl alcohol) diluted 3:2 with dH_2_O for 45 min. Slides were embedded with Dako Faramont Aquaeus Mounting Medium (Agilent Technologies, Santa Clara, CA) and visualized on an Olympus BX63 microscope (Olympus, Shinjuku, Japan). Images were taken using an Olympus DP73 camera (Olympus, Shinjuku, Japan).

### 2.6 Lipid quantification in cells

Cells were lysed in RIPA buffer (150 mM NaCl, 1% Triton® X-100, 0.1% SDS, 50 mM Tris, 0.5% Na-deoxycholate, pH 8) supplemented with a protease inhibitor cocktail (P8340, 1:1,000; Sigma–Aldrich, St. Louis, MO)) by 2 × 10 s sonication on ice. Protein concentration was determined by a Lowry assay (Bio-Rad Laboratories, Hercules, CA). Concentrations of TG, TC, and free cholesterol (FC) were measured with enzymatic kits (DiaSys, Holzheim, Germany), whereas CE concentrations were calculated by subtracting FC from TC.

### 2.7 RNA isolation and quantitative real-time PCR (qRT-PCR)

Total RNA from tissues and cells was isolated with TriFast reagent (Peqlab, Erlangen, Germany) according to the manufacturer's protocol. Two micrograms of total RNA were reverse transcribed using the High Capacity cDNA Reverse Transcription Kit (Applied Biosystems, Carlsbad, CA). qRT-PCR was performed on a Bio-Rad CF X96 real-time PCR system (Bio-Rad Laboratories, Hercules, CA) using GoTaq® qPCR Mastermix (Promega, Fitchburg, WI). Samples were analyzed in duplicate and normalized to the expression of *cyclophilin A*. Primer sequences are listed in Table S1.

### 2.8 Lipase activity assays

Neutral (pH 7) and acid (pH 4.5) CEH and TGH activities were measured in cell and tissue lysates by using radioactively labeled substrates as described previously [42] with minor modifications. Briefly, tissues and cells were lysed in neutral lysis buffer (100 mM potassium phosphate, 1 mM dithiothreitol (Carl Roth, Karlsruhe, Germany), pH 7), sonicated twice on ice for 10 s, and centrifuged at 1,000×*g* and 4 °C for 10 min. To measure CE hydrolase activity, the substrate contained 200 μM cholesteryl oleate/sample, 0.04 μCi/sample cholesteryl [1–^14^C]-oleate (Amersham Biosciences, Piscataway, NJ, USA), and 455 μM mixed micelles of phosphatidylcholine and phosphatidylinositol (3:1). The substrate for the TG hydrolase activity assay contained 300 μM triolein/sample, 0.5 μCi/sample [9,10-^3^H(N)]-triolein (Perkin Elmer, Waltham, MA, USA), and 45 μM of above-mentioned mixed micelles. The substrates were emulsified either in acid citrate buffer (containing 54% of 100 mM citric acid monohydrate and 46% of 100 mM trisodium citrate, dehydrated, pH 4.2) or in 100 mM potassium phosphate buffer (pH 7) to measure acid and neutral lipase activities, respectively. In addition, 30 μM L2 or 0.02% DMSO (control) was added to lysates from mature adipocytes. Lysates from BMDM were treated with various concentrations of L1, L2 or DMSO (control), whereas tissue lysates and COS-7 cell lysates were exposed to 30 μM L1, L2 or DMSO (control). All lysates treated with L1 or L2 were pre-incubated with the respective inhibitors for 10 min before reaction initiation and activity assays were performed in the presence of the inhibitors.

Neutral MGH activity was measured in cell lysates using 1-oleoyl-rac-glycerol (M7765, Sigma–Aldrich, St. Louis, MO). Cells were sonicated twice for 10 s on ice in lysis buffer (0.25 M sucrose, 1 mM EDTA, 20 μM DTT, pH 7) containing 1 μg/ml pepstatin (2936.2, Carl Roth, Karlsruhe, Germany), 2 μg/ml antipain (2933.2, Carl Roth, Karlsruhe, Germany), and 20 μg/ml leupeptin (CN333, Lactan, Graz, Austria) and centrifuged at 1,000×*g* for 10 min at 4 °C. Protein concentrations were determined in the supernatant. Samples (10 μg protein of LacZ, HSL, and PNPLA6; 0.1 μg protein of MGL) were incubated with 1 mM 1-oleoyl-rac-glycerol in PBS (pH 7.4) containing 3% FA-free BSA (A6003, Sigma–Aldrich, St. Louis, MO) and 2.5 mM CHAPS (1479.1, Carl Roth, Karlsruhe, Germany) for 20 min (PNPLA6) or 1 h (LacZ, MGL, and HSL). To quantifiy MGH activity, glycerol release was measured using the Free Glycerol Reagent according to the manufacturer's protocol (F6428, Sigma–Aldrich, St. Louis, MO).

### 2.9 Overexpression of lipid hydrolases in COS-7 cells

The constructs used for overexpression of mouse and human ATGL and CGI-58 as well as mouse HSL have been previously described [33,34]. The sequences containing the complete open reading frame of human HSL (NM_010719.5; KpnI/XhoI), mouse MGL (NM_001166251.1; BamH1/XhoI), and mouse PNPLA6 (NM_001122818.2; XhoI/XbaI) were amplified by PCR from human or mouse cDNA using Advantage cDNA Polymerase Mix (BD Biosciences Clontech, Palo Alto, CA), respectively. cDNA was prepared from mRNA using SuperScript Reverse Transcriptase protocol (Thermo Fisher Scientific, Waltham, MA). The PCR products were ligated to compatible restriction sites of the eukaryotic expression vector pcDNA4/HisMaxC (Thermo Fisher Scientific).

Monkey kidney (SV 40 transformed) COS-7 cells were cultivated in high-glucose DMEM containing 10% FBS and 1% penicillin/streptomycin, and transfected with METAFECTENE® (Biontex, Munich, Germany) in 10 cm cell culture dishes at a 1:3 ratio of DNA to Metafectene (μg:μl) according to the manufacturer's protocol. Twenty-four hours after transfection, cells were harvested for lipase activity assays and western blotting experiments. To generate ATGL/CGI-58 overexpressing lysates, 25 μg of proteins from cells overexpressing ATGL or CGI-58 were mixed.

### 2.10 Western blotting experiments

COS-7 cells were harvested in lysis buffer containing 1 mM dithiothreitol and protease inhibitor cocktail (P8340, 1:1,000; Sigma–Aldrich, St. Louis, MO) and sonicated twice on ice for 10 s. Twenty-five micrograms of protein were separated by SDS-PAGE and transferred to a nitrocellulose membrane. The following primary anti-rabbit antibodies were incubated overnight at 4 °C: ATGL (#2138; 1:1,000, Cell Signaling Technologies, Danvers, MA), HSL (#4107; 1:1,000, Cell Signaling Technologies), 6X His tag (ab18184; 1:1,000, Abcam, Cambridge, UK), Calnexin (#2679T; 1:1,000, Cell Signaling Technologies), and anti-mouse CGI-58 (H00051099-M01; 1:1,000, Abnova, Taipei City, Taiwan). Two hours after incubation with secondary anti-rabbit (P0448, 1:2000, Agilent Technologies, Santa Clara, CA) or anti-mouse (P0260, 1:500, Agilent Technologies, Santa Clara, CA) antibodies, membranes were visualized using ECL (BioRad Laboratories, Hercules, CA) and the ChemiDoc™ Imaging System (BioRad Laboratories).

### 2.11 Activity-based probe (ABP) labeling and enrichment

BMDM were differentiated and treated as described above. On day 6, cells were incubated for 30 min with (probe) or without (no probe) 30 μM ABP C6 (hexyl 4-nitrophenyl (3-azidopropyl)phosphonate). The ABP C6 probe was synthesized as previously described [43,44]. Cells were washed with cold PBS and lysed by sonication (15 s) in lysis buffer (100 mM Tris–HCl pH 8.5, 1% SDS, 10 mM tris(2-carboxyethyl)phosphine, 30 mM N-ethylmaleimide, protease inhibitor cocktail (#P8340, 1:2,000; Sigma–Aldrich, Vienna, Austria)) and incubated at 95 °C for 10 min. Cell debris was removed by centrifugation at 14,000×*g* for 10 min at 4 °C. After protein estimation by bicinchoninic assay (#23225, Thermo Fisher Scientific, Vienna, Austria), 500 μg per sample was precipitated with acetone. Precipitates were redissolved in 100 mM Tris–HCl (pH 8.5) and 1% SDS and incubated with 1.2 nmol DBCO-TEV-Biotin strain-promoted click linker (PiChem, Grambach, Austria) [44] at 37 °C on an orbital shaker at 500 rpm overnight to ensure a click reaction between the ABP C6 probe and the biotin-containing linker. Amicon Ultra 3 kDa centrifugal filters were used to remove excess linker and reduce SDS concentration. The retentate was washed with 8 M urea in 100 mM Tris–HCl (pH 8.5), followed by 2 M urea in 100 mM Tris–HCl (pH 8.5). For enrichment, the retentate was incubated on streptavidin-agarose resin (#20347, Thermo Fisher Scientific, Vienna, Austria) in micro-spin columns on an overhead rotor for 5 h. The beads were subsequently washed with 2 M urea in 100 mM Tris–HCl (pH 8.5) and incubated overnight with 100 ng sequencing-grade modified trypsin (#V5111, Promega, Walldorf, Germany) in 100 mM Tris–HCl (pH 8.5). On-bead digested proteins were collected from the spin columns and desalted with 1% trifluoroacetic acid prior to LC-MS/MS measurement.

### 2.12 Liquid chromatography–mass spectrometry/mass spectrometry (LC-MS/MS), data analysis, and statistics

In-house packed 2-layered polystyrene-divinylbenzene, reversed-phase sulfonate (SDB-RPS) stage tips (#66886-U, Empore SPE disks, Supelco, Pennsylvania, USA) were used for desalting. Samples were loaded onto the stage tips, washed once with 0.2% trifluoroacetic acid and eluted with 5% NH_4_OH in 80% acetonitrile (ACN), followed by vacuum centrifugation until completely dry. Samples were redissolved in 16 μl of solvent A (0.1% formic acid in H_2_O), and 3 μl of each sample was chromatographically separated by nano-HPLC (Dionex Ultimate 3000, Thermo Fisher Scientific, Gmermany) on an Aurora Series UHPLC C18 column (250 mm × 75 μm, 1.6 μm) (IonOpticks, Middle Camberwell, VIC, Australia) at 40 °C and a flow rate of 0.4 μl/min with the following gradient: 0–8 min: 2% solvent B (0.1% formic acid in ACN); 8–68 min: 2–17% B; 68–98 min: 17–25% B; 98–108 min: 25–37% B; 108–118 min: 37–95% B; 118–128 min: 95% B; 128–129 min: 95–2% B; 129–139 min: 2% B. MS analysis was performed on a timsTOF Pro mass spectrometer (Bruker Daltonics, Bremen, Germany) operated in positive data-dependent parallel accumulation-serial fragmentation (PASEF) mode [45] with trapped ion mobility spectrometry (TIMS) enabled at 100% duty cycle (100 ms cycle time). Source capillary voltage was set to 4,500 V and the dry gas flow to 3 L/m at 180 °C.

MaxQuant version 2.0.3.0 [46] was used for database search and protein quantitation. Data analysis was performed with Perseus version 1.6.15.0 [47]. The UniProt *mus musculus* FASTA file (downloaded on 2021/08/17) including frequent protein contaminants (derived from the cRAP database) was used with a reverted decoy database. The false discovery rate (FDR) was set to 1% for peptide spectrum match, 1% for protein, and 1% for the site decoy fraction. Label-free quantitation (LFQ) was performed with a minimum of 2 ratio counts of razor and unique peptides, and match between runs was enabled with a retention time window of 0.7 min and an alignment window of 20 min. Peptide tolerance was set to ±20 for the first peptide search and ±10 for the main peptide search. N-Ethylmaleimide on cysteine was set as a fixed modification, while oxidation of methionine and acetylation at the N-terminus were set as variable modifications, with a maximum of 5 modifications allowed per peptide. A tryptic digest was chosen with a maximum of 2 missed cleavages allowed and a minimum peptide length of 7 amino acids.

From the MaxQuant protein search we obtained a list of 5,116 proteins with LFQ intensity values (representing protein abundance) that were further processed in Perseus. The protein matrix was reduced by removing contaminants. The list of proteins with the respective LFQ intensity values was further log2-transformed, resulting in invalid values for each missing value. The individual samples were then assigned to the appropriate groups as control, L1, L2, or according to the ABP C6 probe as treated (probe) or untreated (no probe). To verify serine hydrolase enrichment, the probe samples were compared with the no probe samples within one group (i.e., control, L1, L2). Each matrix was reduced to proteins identified in at least 3 (out of 4) individual samples in at least one group (e.g., probe or no probe), which resulted in a list of 4,578 proteins in the control samples, 4,456 proteins in L1 samples, and 4,301 proteins in L2 samples. The remaining missing values were imputed from the normal distribution with a downshift of 1.6 and a width of 0.4, and the normal distribution of the data was checked using log histograms (Fig. S3C). For statistical analysis, a two-sided t-test with a p-value of 0.05 and s0 of 1, and a permutation-based FDR of 5% (250 randomizations to correct for multi-testing) was performed and visualized as volcano plots (Fig. S3B). A list of proteins with the corresponding results of the statistical tests for each group is available in Tables S2-4. To identify enzymes targeted by L1 or L2, all serine hydrolases significantly enriched by the probe with a fold change higher than 3 in the control samples were used for statistical tests. LFQ intensities of L1 (probe) and L2 (probe) samples were normalized to control (probe) and unpaired two-tailed Student's t-tests were performed (Figure 4 and S4). Data are presented as mean +SD and significance levels were set at ∗ p < 0.05, ∗∗p ≤ 0.01, and ∗∗∗p ≤ 0.001 relative to controls.

The MS proteomics data have been deposited to the ProteomeXchange Consortium via the PRIDE [48] partner repository with the dataset identifier PXD030672.

### 2.13 Statistical analysis

Data are presented as mean ± SD. Statistical significance between groups was determined using the GraphPad Prism 5.01 software. Comparisons between 2 groups were performed using the unpaired 2-tailed Student's t-test, and comparisons between multiple groups were analyzed by 1-way ANOVA followed by the Bonferroni post-hoc test. For qRT-PCR results, the 2^−ΔΔCT^ method was used. Significance levels were set at ∗ p < 0.05, ∗∗p ≤ 0.01, and ∗∗∗p ≤ 0.001 relative to controls, ^#^p < 0.05, ^##^p ≤ 0.01, and ^###^p ≤ 0.001 between different genotypes, ^§^p < 0.05, ^§§^p ≤ 0.01, ^§§§^p ≤ 0.001 between different treatments, and ^$^ p < 0.05, ^$$^ p ≤ 0.01, and ^$$$^ p ≤ 0.001 for ATGL/CGI-58 o/e relative to ATGL o/e.

## 3 Results

### 3.1 Pharmacological inhibition of LAL reduces isoproterenol-stimulated lipolysis in mature adipocytes

**Figure 1:**
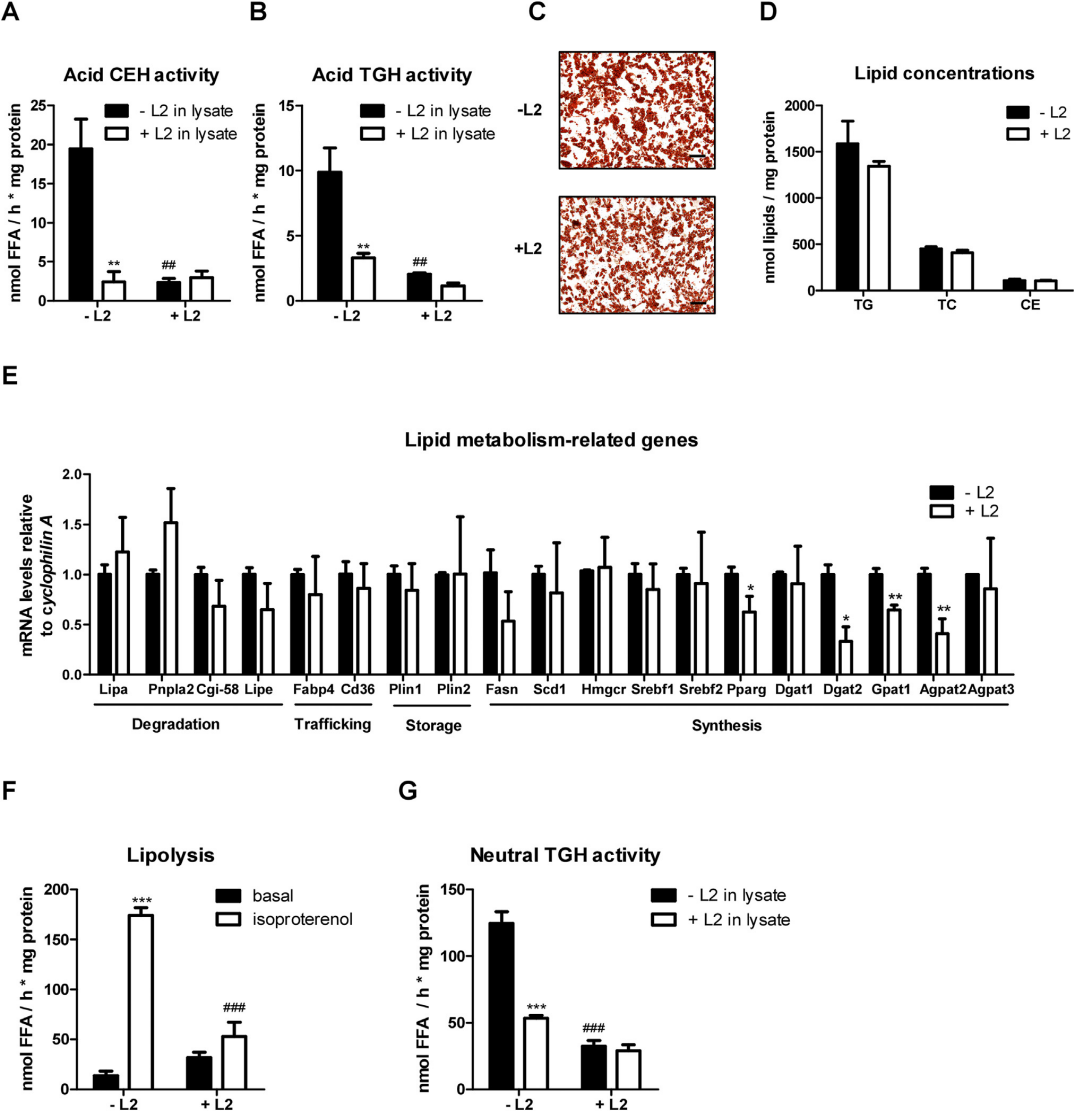
**Lalistat 2 (L2) does not affect adipocyte differentiation but reduces isoproterenol-stimulated lipolysis.** Stromal vascular cells were isolated from subcutaneous white adipose tissue of wild-type mice and differentiated into mature adipocytes in the absence (- L2) or presence of 30 μM Lalistat 2 (+ L2); 0.02% DMSO was used as control. (A) Acid (pH 4.5) cholesteryl ester hydrolase (CEH) and (B) triglyceride hydrolase activity (TGH) activity in lysates of adipocytes in the absence (- L2 in lysate) or presence (+ L2 in lysate) of 30 μM L2. (C) Lipid staining with oil red O (magnification, 10x; scale bar, 100 μm) and (D) quantification of TG, total cholesterol (TC), and CE concentrations. (E) mRNA expression of genes associated with lipid metabolism. (F) Basal and isoproterenol (10 μM) stimulated lipolysis in adipocytes determined as FA release. (G) Neutral (pH 7) TGH activity of adipocytes +/− 30 μM L2 in differentiation medium and lysates. Data (n = 3) represent mean + SD. The results are representative of two independent experiments. Statistically significant differences were calculated by (D, E) Student's t-test or (A, B, F, G) 1-way ANOVA followed by Bonferroni post-hoc test; ∗∗p ≤ 0.01, ∗∗∗p ≤ 0.001 for comparisons within the group (−/+ L2); ^##^p ≤ 0.01, ^###^p ≤ 0.001 for comparisons between the groups (−/+ L2 in lysate or basal/isoproterenol-stimulated).

LAL-D in mice is associated with massive loss of WAT [12,13]. To study the impact of pharmacological LAL inhibition on adipocyte differentiation and lipid homeostasis, we isolated stromal vascular cells (SVC) from subcutaneous WAT (sWAT) of wild-type (WT) mice and differentiated them into mature adipocytes in the absence or presence of 30 μM L2. We confirmed LAL inhibition in L2-treated mature adipocytes by reduced activities of acid (pH 4.5) CE hydrolase (CEH; 88%) and TG hydrolase (TGH; 79%) (Figure 1A and B). To exclude the possibility that LAL was not completely inhibited during cell differentiation, we simultaneously incubated cell lysates shortly before (10 min) and during the activity assays (60 min) with 30 μM L2 and observed no additional significant reduction in activity (Figure 1A and B). We chose 30 μM because the most commonly used L2 concentrations are reported in a range between 10 and 100 μM. Lipid staining with oil red O (Figure 1C) and comparable concentrations of TG, total cholesterol (TC), and CE (Figure 1D) revealed that inhibition of LAL in mature adipocytes did not affect lipid accumulation. mRNA expression of genes involved in lipid degradation (*Lipa* (encoding LAL)*, Pnpla2* (encoding ATGL)*, Cgi-58, Lipe* (encoding HSL)) and lipid transport (*Fabp4, Cd36*), and lipid storage (*Plin1* and *Plin2)*, remained unaltered upon L2 treatment (Figure 1E). Unchanged mRNA expression of *Fasn, Scd1, Hmgcr, Srebf1*, *Srebf2*, and *Agpat3,* but decreased expression of *Pparg, Dgat2, Gpat1, and Agpat2* showed a negative effect of L2 on lipid synthesis (Figure 1E). To investigate whether and to which extent LAL inhibition affects overall lipolysis, we measured the release of FFA in the basal state and after stimulating lipolysis with the non-selective β3-adrenergic receptor agonist isoproterenol [49]. Basal lipolysis remained unchanged in adipocytes differentiated with L2, whereas isoproterenol-stimulated lipolysis was reduced by 70% (Figure 1F), suggesting that L2 treatment also affected neutral lipase activity. Accordingly, a 74% decrease in neutral (pH 7) TGH activity indicated that L2 may exert LAL-independent effects and also inhibit neutral lipases (Figure 1G). To prevent a possible indirect effect of LAL inhibition on the activity of neutral lipases, we added L2 to the lysates, which resulted in a strong decrease in TGH activity by 66%, comparable to the in vitro treatment of the cells (Figure 1G).

### 3.2 L2 inhibits neutral lipid hydrolase activity in both WT and Lal−/− mature adipocytes

**Figure 2:**
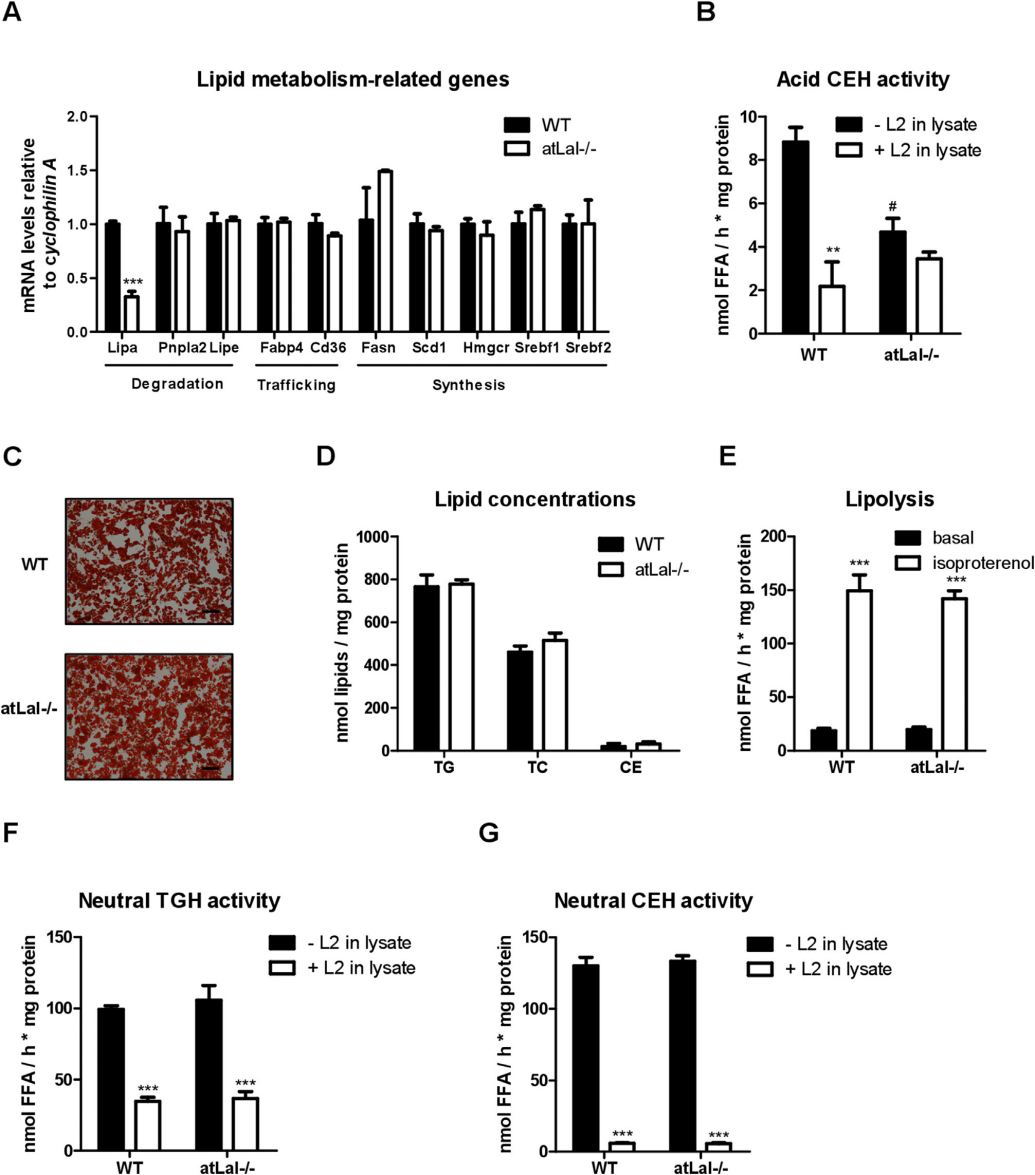
**Lalistat 2 (L2) inhibits neutral lipid hydrolase activity in adipocytes.** Stromal vascular cells were isolated from subcutaneous white adipose tissue of wild-type (WT) and adipocyte-specific (at) Lal−/− mice and differentiated into mature adipocytes. (A) mRNA expression of genes associated with lipid metabolism. (B) Acid (pH 4.5) cholesteryl ester hydrolase (CEH) activity in lysates of adipocytes in the absence (- L2 in lysate) or presence (+ L2 in lysate) of 30 μM L2. (C) Lipid staining with oil red O (magnification, 10x; scale bar, 100 μm) and (D) quantification of TG, total cholesterol (TC), and CE concentrations. (E) Basal and isoproterenol (10 μM) stimulated lipolysis in WT and Lal−/− adipocytes determined as free fatty acid release. Neutral (pH 7) (F) triglyceride hydrolase (TGH) and (G) cholesteryl ester hydrolase (CEH) activity in the absence (0.02% DMSO) or presence of 30 μM L2 in lysates. Data (n = 3) represent mean + SD. Statistically significant differences were calculated by (A, D) Student's t-test or (B, E, F, G) 1-way ANOVA followed by Bonferroni post-hoc test; ∗∗p ≤ 0.01, ∗∗∗p ≤ 0.001 for comparisons within the groups (−/+ L2); ^#^p < 0.05 for comparisons between the groups (−/+ L2 in lysate).

We next isolated SVC from sWAT of WT and adipocyte-specific Lal−/− (atLal−/−) mice (Lal−/− SVC) and differentiated them into mature adipocytes. Decreased *Lipa* mRNA expression (Figure 2A) and reduced acid CEH activity (Figure 2B) confirmed the loss of LAL in mature Lal−/− adipocytes. Consistent with the results of pharmacological inhibition of LAL, genetic loss of the enzyme in mature adipocytes failed to affect mRNA expression of genes involved in lipid metabolism (Figure 2A), lipid staining (Figure 2C), and lipid quantification (Figure 2D). In contrast to L2 treatment (Figure 1G), basal and isoproterenol-stimulated lipolysis were unchanged in Lal−/− adipocytes (Figure 2E). Unaltered lipolysis along with comparable neutral TGH and CEH activities in Lal−/− adipocytes (Figure 2F and G) corroborated that genetic loss of LAL did not affect neutral lipid hydrolysis. Upon addition of L2 to lysates from WT and Lal−/− adipocytes, we observed similarly decreased neutral TGH and CEH activities in cells of both genotypes, confirming that L2 has off-target effects on neutral lipases.

### 3.3 L1 and L2 inhibit neutral lipid hydrolases in bone marrow-derived macrophages (BMDM) in a dose-dependent manner

**Figure 3:**
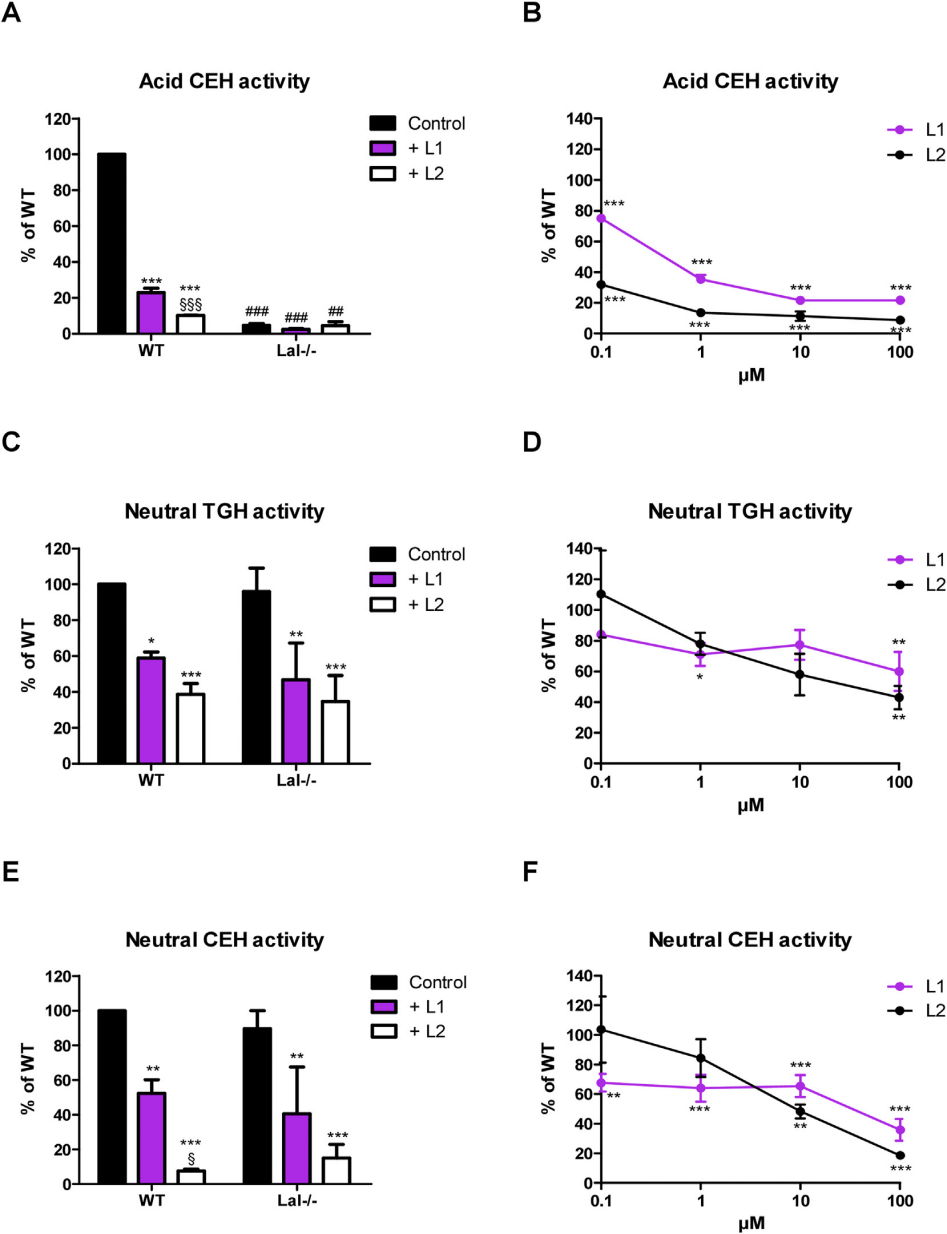
**Lalistat-1 (L1) and L2 inhibit neutral lipid hydrolases in bone marrow-derived macrophages (BMDM) in a dose-dependent manner.** Bone marrow from WT and Lal−/− mice was differentiated to macrophages for 6 days. (A) Acid (pH 4.5) cholesteryl ester hydrolase (CEH), (C) neutral (pH 7) triglyceride hydrolase (TGH), and (E) neutral CEH activities of BMDM treated in vitro with 30 μM L1, L2 or 0.02% DMSO (Control) for 20 h. Dose-dependent inhibition of L1 and L2 on (B) acid CEH, (D) neutral TGH, and (F) neutral CEH activity. Data (n = 3) represent mean ± SD. Lipid hydrolase activities were compared to untreated WT BMDM (arbitrarily set to 100%) and graphs represent the remaining hydrolase activity upon inhibitor treatment. Statistically significant differences were calculated by 1-way ANOVA followed by Bonferroni post-hoc test. ∗p < 0.05, ∗∗p ≤ 0.01, ∗∗∗p ≤ 0.001 for control versus L1 or L2 treatment within the same genotype; ^##^p ≤ 0.01, ^###^p ≤ 0.001 for comparing same treatment between different genotypes; ^§^p < 0.05, ^§§§^p ≤ 0.001 for L1 versus L2 treatment within the same genotype.

To determine whether the inhibition of neutral lipases by L2 is cell type independent, we performed lipase activity assays using WT and Lal−/− BMDM. In addition, we included L1 in the experiments. Treatment of WT BMDM with 30 μM L1 or L2 in vitro (20 h incubation in culture) or in lysates (10 min lysate pre-incubation and 60 min incubation during activity assays) revealed that both inhibitors drastically decreased acid CEH activity (Figure 3A and Fig. S1A). In vitro*,* L2 was a more effective acid CEH inhibitor than L1 (90% versus 77% inhibition) (Figure 3A), whereas L2 was less effective than L1 (81% vs 92%) when added to lysates (Fig. S1A). The impaired acid CEH activity in Lal−/− BMDM under the applied conditions (Figure 3A and Fig. S1A) indicated that acid CEH activity was exclusively catalyzed by LAL. Next, we investigated whether lower concentrations of L1 or L2 effectively inhibit acid CEH activity. Indeed, 10 μM L1 in vitro or in lysate was sufficient to reduce acid CEH activity by 89% and 79%, respectively (Figure 3B and Fig. S1B). Treatment of cells with 1 μM L2 already inhibited acid CEH activity by 84% (Figure 3B), whereas 10 and 100 μM L2 were required for 71% and 91% inhibition in lysates, respectively (Fig. S1B).

In WT and Lal−/− BMDM, 30 μM L1 or L2 reduced neutral TGH activity between 41% and 65% in vitro (Figure 3C). A further decrease in neutral TGH activity of ≥30% was also observed after addition of inhibitors to lysates (Fig. S1C). We then determined whether lower L1 and L2 concentrations could eliminate off-target effects on neutral TGH and CEH activities. Dose-dependent inhibition showed that both inhibitors reduced neutral TGH activity by >20% at 1 μM, regardless of the type of supplementation, and reached inhibition levels of >40% at concentrations between 10 and 100 μM (Figure 3D and Fig. S1D). Moreover, 30 μM L1 or L2 were highly effective in inhibiting neutral CEH activity in vitro (Figure 3E) and in lysates (Fig. S1E). Dose-dependent inhibition of neutral CEH activity reached significance in vitro at 0.1 μM L1 (32%) and at 10 μM L2 (52%), respectively (Figure 3F), whereas treatment of the lysate with 1 μM L1 or 0.1 μM L2 significantly reduced neutral CEH activity by 58% and 63%, respectively (Fig. S1F). Taken together, these results suggested that in vitro concentrations of L1 or L2 sufficient to inhibit the majority of LAL activity also affected the activity of neutral lipid hydrolases. L2 inhibits neutral lipase activity with an IC_50_ value of ∼10 μM. L1 reduced neutral TGH and CEH activities by ~35% already at 1 µM. This effect was even more pronounced when the inhibitors were added to lysates, with L1 and L2 inhibiting acid and neutral CEH activity to a similar extent. This suggests a narrow window for selective LAL inhibition of no more than 1 μM in cell experiments, with smaller effects on neutral lipases.

### 3.4 L1 and L2 inhibit mouse and human ATGL and HSL in overexpressing cells

**Figure 4:**
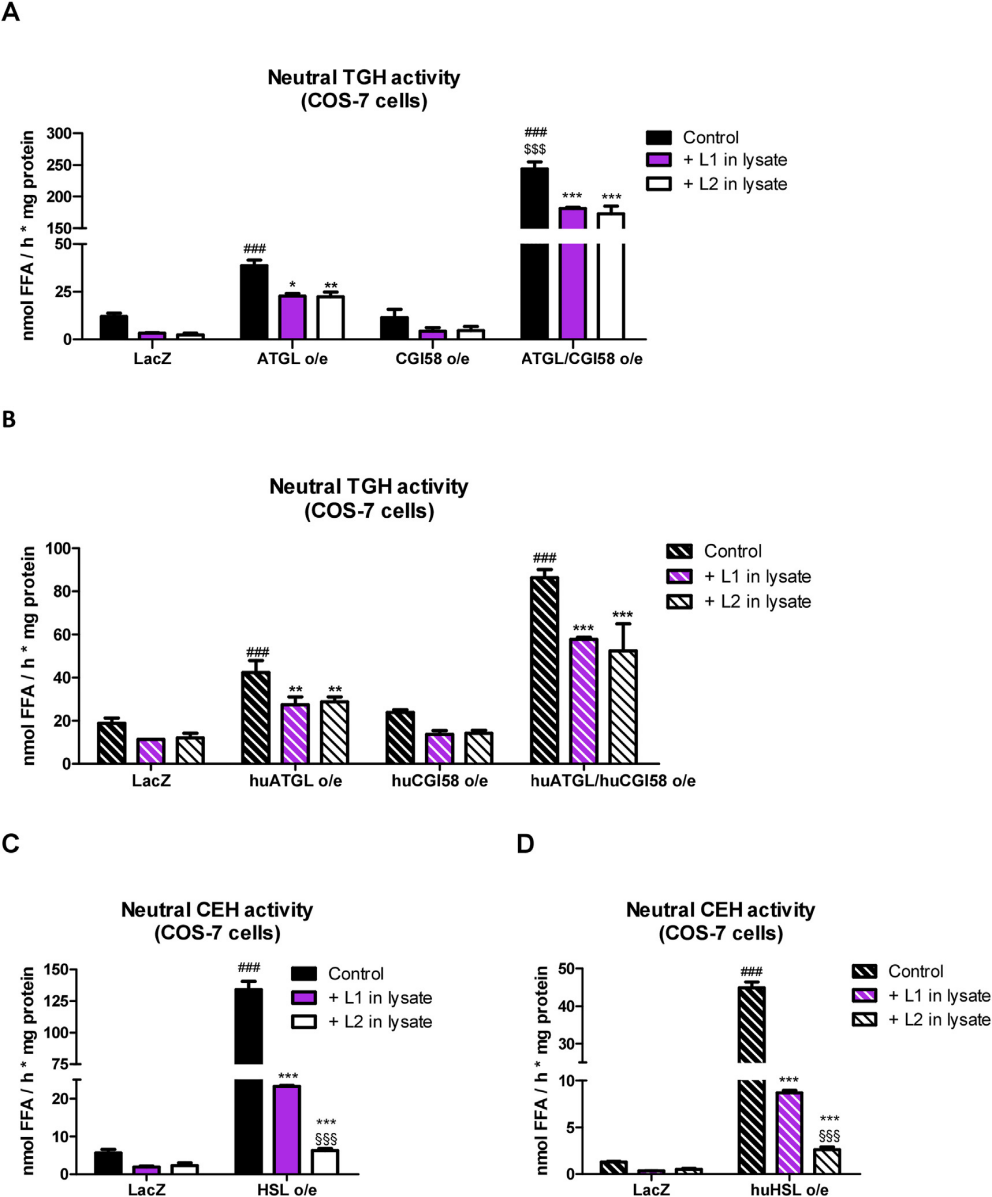
**L1 and L2 inhibit mouse and human ATGL and HSL.** Protein lysates of COS-7 cells were treated with 30 μM L1, L2 or 0.02% DMSO (Control). Neutral TGH activity in lysates of COS7 cells overexpressing (o/e) (A) mouse or (B) human ATGL, CGI-58, and ATGL/CGI-58. Neutral CEH activity in lysates of COS-7 cells o/e (C) mouse or (D) human HSL. Cells transfected with LacZ were used as control. Data (n = 3) represent mean +SD. Statistically significant differences were calculated by 1-way ANOVA followed by Bonferroni post-hoc test; ∗p < 0.05, ∗∗p ≤ 0.01, ∗∗∗p ≤ 0.001 for control versus L1 or L2 treatment within the same genotype/group; ^###^p ≤ 0.001 for comparing treatments in different genotypes/groups; ^§§§^p ≤ 0.001 for L1 versus L2 in the same genotype/group; ^$$$^ p ≤ 0.001 for comparing ATGL/CGI-58°/e to ATGL o/e.

Since the major lipid hydrolases that degrade TG and CE at neutral pH are ATGL and HSL [38], we overexpressed mouse and human (hu) ATGL (in absence and presence of its co-activator CGI-58) and HSL in COS-7 cells to directly examine whether they were inhibited by L1 and L2. Mouse ATGL overexpression resulted in a 3.2-fold increase in neutral TGH activity compared with LacZ-transfected cells, which further increased 6.3-fold by the combined overexpression of ATGL and CGI-58 (Figure 4A). Addition of 30 μM L1 or L2 to the cell lysates reduced neutral TGH activity by >26%, indicating that the inhibitors also impact ATGL activity (Figure 4A). Neutral and acid CEH activities remained unaffected by overexpression of the proteins (Fig. S2C and D), confirming that ATGL has no CEH activity. Consistent with these data, overexpression of huATGL and huATGL/huCGI58 led to a 2.3-fold and 4.6-fold increase in neutral TGH activity, which was inhibited by >32% after the addition of L1 or L2 (Figure 4B).

Mouse HSL overexpression (Fig. S2A and B) caused a 23.7-fold increase in neutral CEH activity compared with LacZ-transfected cells (Figure 4C), and neutral TGH activity was increased by 2.4-fold (Fig. S2E). Addition of 30 μM L1 or L2 reduced neutral CEH activity by 83% and 95%, respectively (Figure 4C), and TGH activity by 57% and 62%, respectively (Fig. S2E). As expected, both inhibitors decreased acid CEH activity by >99% in LacZ- and mouse HSL-transfected cells (Fig. S2F). Overexpression of huHSL resulted in a 34.3-fold increase in neutral CEH activity, which was inhibited by 80% and 94% by L1 or L2 treatment, respectively (Figure 4D).

In summary, these data demonstrated that L1 and L2 inhibit mouse and human ATGL and HSL.

### 3.5 L1 and L2 inhibit the activity of several neutral lipid hydrolases by binding to their active site

**Figure 5:**
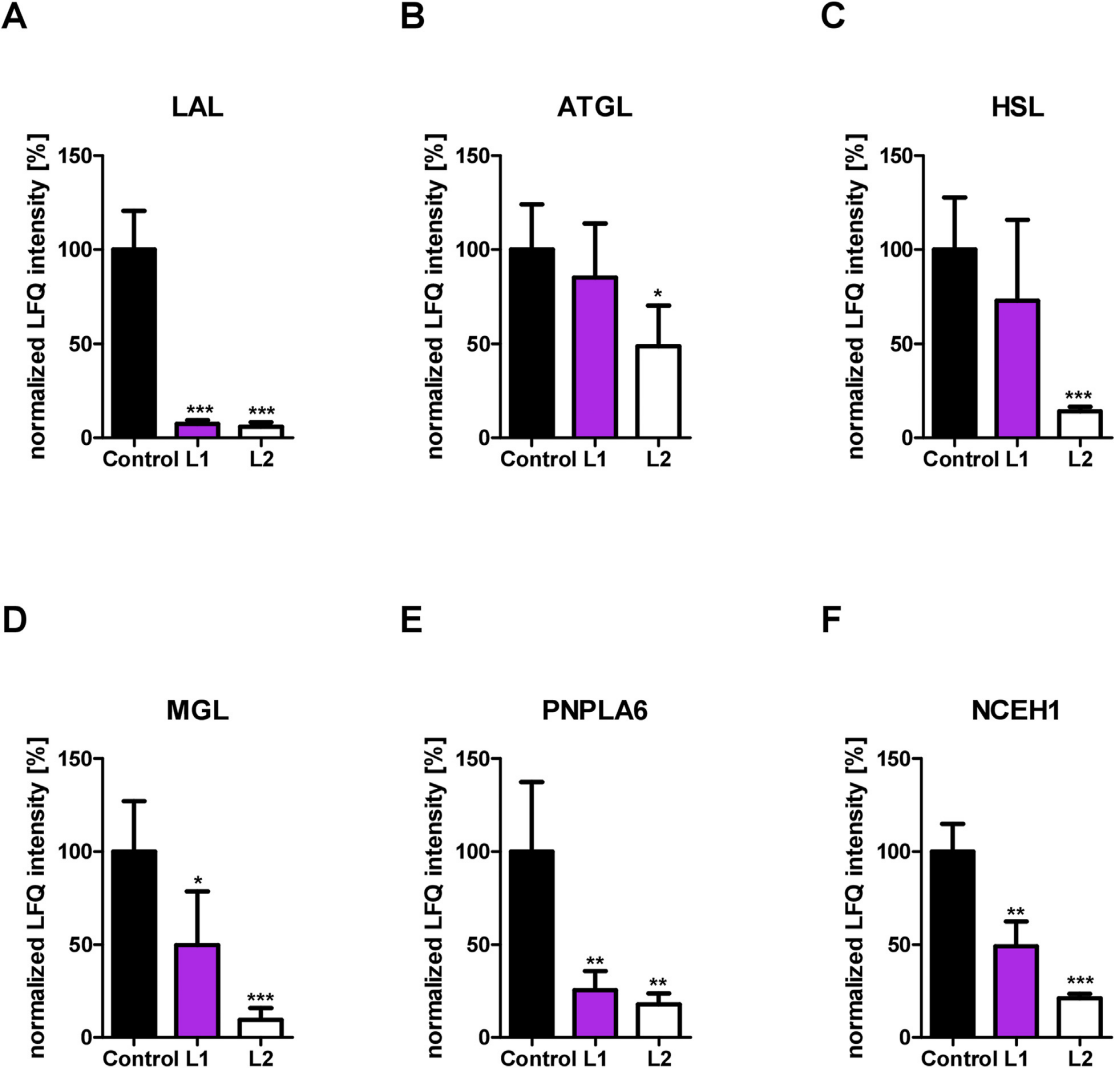
**L1 and L2 reduce activity-based probe binding of several lipid hydrolases.** Bone marrow from WT mice was differentiated into macrophages for 6 days. Bone marrow-derived macrophages were treated with 30 μM L1, L2 or 0.02% DMSO (Control) for 20 h and labeled with the ABP C6 probe to enrich active serine hydrolases for activity-based proteomic profiling (ABPP). LFQ intensity values (representing probe binding in ABPP experiments) derived from label-free quantitation of MS data were normalized to control (arbitrarily set to 100%). Abundance of ABP-bound (A) LAL, (B) ATGL, (C) HSL, (D) MGL, (E) PNPLA6, and (F) NCEH1. Data (n = 4) represent mean + SD. Statistically significant differences were calculated by two-tailed Student's t-test; ∗p < 0.05, ∗∗p ≤ 0.01, ∗∗∗p ≤ 0.001 for control versus L1 or L2 treatment.

Finally, we employed untargeted activity-based proteomic profiling of lipases in BMDM pretreated with/without 30 μM L1 or L2 using a serine hydrolase-directed probe (ABP C6) that efficiently and covalently binds to active lipases in their active sites [44]. ABP C6-labeled proteins were subsequently enriched and analyzed by quantitative mass spectrometry. Serine hydrolase enrichment was assessed by comparing ABP C6-treated (probe) with untreated samples (no probe) within each group (control, L1 treatment, L2 treatment). Principle component analysis (PCA) revealed separation based on probe treatment within the control and each inhibitor group (Fig. S3A). Comparison between probe and no probe samples resulted in specific enrichment of serine hydrolases (including several lipid hydrolases) in probe-treated samples in each group, as visualized by volcano plots (Fig. S3B) and listed in Tables S2-4. To determine whether the inhibitors interfered with binding of the ABP C6 probe to serine hydrolases, we compared the abundance of the serine hydrolases in L1-or L2-treated samples with control samples. After binding of an inhibitor to a serine hydrolase, the active site is occupied, interfering with the binding of ABP C6. This results in lower enrichment of inhibited compared to non-inhibited serine hydrolases, which can be identified in proteomic analysis as a decreased abundance of the respective serine hydrolase. We determined the extent of inhibition by L1 or L2 in all serine hydrolases significantly enriched in control samples with a fold change >3 (Fig. S3B) by comparing the protein abundance (LFQ intensity) in the probe samples in the absence (control) or presence of L1 or L2. Strikingly, this approach revealed that several serine hydrolases were targeted by L1 or L2. Treatment with both 30 μM L1 and L2 caused a reduction of LAL binding by the ABP C6 probe by >90% (Figure 5A). ABP-binding to HSL (Figure 5C) was also strongly reduced by L2 treatment by 86%, whereas ATGL appeared to be less affected (Figure 5B). In addition, two enzymes that can cleave MG (MGL and PNPLA6) were targeted by L2 (Figure 5D, E), resulting in reduced activity of 91% and 82%, respectively. We confirmed the inhibition of neutral MG hydrolase (MGH) activity in COS-7 cells overexpressing MGL, PNPLA6, or HSL with/without addition of L1 or L2. The 139-fold increase in neutral MGH activity in MGL overexpressing cell lysates was inhibited by L1 and L2 treatment by 33% and 59%, respectively (Fig. S4A). Overexpression of HSL or PNPLA6 increased neutral MGH activity by 5.0- and 3.8-fold, respectively (Fig. S4B). In agreement with the ABP results, treatment with L1 or L2 decreased neutral MGH activity by >57% and >32% in cells overexpressing HSL and PNPLA6, respectively, with higher inhibition by L2 (Fig. S4B).

With a decreased binding affinity to ABP by 79%, L2 also captured NCEH1 (Figure 5F), an enzyme important for CE hydrolysis in macrophages [50]. To a lesser extent, these trends were also observed with L1 treatment, which reduced the activity of these lipid hydrolases by ≥ 50%. Interestingly, several other serine hydrolases were also reduced in L1-and L2-treated samples: acyl-protein thioesterase 1 (LYPLA1), acyloxyacyl hydrolase (AOAH), alpha beta hydrolase domain containing 11 (ABHD11), non-lysosomal glucosylceramidase (GBA2), acyl-coenzyme A thioesterase 11 (ACOT11), phospholipase D1 (PLD1), phospholipase A2 group XV (PLA2G15), acylamino acid releasing enzyme (APEH), lysosomal pro-X-carboxypeptidase (PRCP) and tripeptidyl peptidase 2 (TPP2) (Fig. S5). Overall, L2 treatment reduced probe binding to a higher extent than L1 treatment in most enzymes (Figure 5, Fig. S5).

These data emphasize that neither L1 nor L2 binds exclusively to the active site of LAL but also to other serine hydrolases, including several neutral lipid hydrolases.

## 4 Discussion

The properties of L1 and L2 (originally reported as compounds 13 and 12, respectively) and their ability to pharmacologically inhibit LAL were described more than 10 years ago during screening for compounds as therapeutic agents for Niemann-Pick type C disease [30,31]. Surprisingly, the specificity of L1 and L2 has been analyzed only for secretory lipases [31], and their effects on neutral cytosolic lipases have never been studied. We observed decreased expression of genes involved in lipid synthesis and reduced isoproterenol-stimulated lipolysis upon L2 treatment of SVC, which resulted in unaltered cellular lipid levels. Inhibition of isoproterenol-stimulated lipolysis in L2-treated adipocytes differentiated from atLal−/− SVCs prompted us to thoroughly investigate the effects of L1 and L2 on the enzymatic activities of lipases responsible for neutral cellular lipolysis. A significant decrease in neutral TGH and CEH activity after in vitro or in lysate addition of 30 μM L1 or L2 to WT and Lal−/− BMDM suggested that both inhibitors acted on neutral lipases in addition to LAL.

We further tested whether lower concentrations of inhibitors were sufficient to inhibit LAL activity without affecting neutral lipid hydrolysis, as the IC_50_ values for L1 and L2 against purified human LAL are 68 nM and 152 nM, respectively [31]. To analyze the consequences of LAL-D on cellular metabolism, LAL must be substantially inhibited. Our dose-dependent inhibition experiments in BMDM showed that inhibitor concentrations of >1 μM are needed for sufficient LAL inhibition to a level comparable to that of Lal−/− BMDM, whereas the most commonly used L1 and L2 concentrations range between 10 and 100 μM [14,15,[18], [19], [20], [21],^24^,^26^,^51^]. However, these concentrations also impair neutral lipase activities. Of note, substantial LAL inhibition is required for studying LAL-dependent effects on cellular homeostasis as humans and mice with one functional allele of *Lipa*, the gene encoding for LAL, stay healthy without any symptoms [12,52]. In fact, individuals with LAL activity of >12% and symptoms of LAL-D have not been described.

Intriguingly, 30 μM L1 and L2 markedly inhibited the activity of the major neutral lipases ATGL and HSL [38] in COS-7 cells with overexpression of either human or mouse ATGL, ATGL/CGI-58, or HSL. By activity-based proteomic profiling, we confirmed the inhibitory action of L2 on mouse ATGL and HSL by 51% and 86%, respectively, and found that it binds to the active site of both enzymes. As expected, treatment with 30 μM L2 reduced ABP bound to LAL by >90%. This approach also revealed that L2 also binds to the active site of MGL and PNPLA6. Several reports have established MGL as a MG hydrolase [53,54] and one study has shown that PNPLA6 is a functional MG hydrolase [55]. We confirmed these activities and demonstrated reduced MGH activity in cells overexpressing MGL and PNPLA6 upon L1 and L2 treatment. Despite its predominant role in DG catabolism within the lipolytic cascade, HSL also exhibits MGH activity [56], which was inhibited by L1 and L2. Despite a 46% sequence homology of PNPLA3/adiponutrin to ATGL (also called PNPLA2) and its ability to hydrolyze TG, DG, and MG, neither PNPLA3 [57] nor other members of the PNPLA family were bound by the ABP probe, which might be due to their low expression in BMDM. Treatment with both inhibitors also lead to highly reduced ABP-bound NCEH1, which has been reported to cleave CE in macrophages and foam cells [50,58]. L1 showed the same inhibitory effect as L2 on ABP binding to LAL and PNPLA6 and markedly reduced binding to MGL and NCEH1, whereas the effects of L1 on HSL and ATGL showed only a tendency toward reduced probe binding. This may be due to the different structures and binding properties of L1 and L2 to the different enzymes.

In summary, our study provides conclusive evidence that L2 (and to a lesser extent L1) also inhibit the lipases ATGL, HSL, and MGL involved in neutral lipolysis in the cytosol. The applied inhibitor concentrations in cell culture studies should not exceed 1 μM.

Several studies have demonstrated reduced circulating LAL activity in patients with nonalcoholic fatty liver disease (NAFLD) [59,60]. Since we have observed inhibition of L2 on neutral lipases as low as 1 μM, this concentration of the inhibitor may also not be useful to study the role of LAL in diseases, including NAFLD. In various cellular processes, our findings raise questions regarding the data interpretation of studies in which higher concentrations of L2 or L1 were used to distinguish between the roles of LAL and neutral lipid hydrolases. In addition, results addressing specific signaling pathways dependent on lysosomal-derived FFA and cholesterol should be re-evaluated. Hence, this study critically questions the use of these inhibitors to investigate lysosomal lipid hydrolysis because they act on both the neutral and the acid lipid degradation pathways.

## Author contributions

**Ivan Bradić**: Conceptualization, Formal analysis, Investigation, Methodology, Writing – Original Draft, Writing – Review & Editing; **Katharina B. Kuentzel**: Conceptualization, Formal analysis, Investigation, Methodology, Writing – Original Draft, Writing – Review & Editing; **Sophie Honeder**: Formal analysis, Investigation, Methodology, Writing – Review & Editing; **Gernot F. Grabner**: Formal analysis, Investigation; **Nemanja Vujić**: Formal analysis, Investigation, Writing – Review & Editing; **Robert Zimmermann**: Funding acquisition, Resources, Writing – Review & Editing; **Ruth Birner-Gruenberger**: Formal analysis, Methodology, Funding acquisition, Project administration, Resources, Writing – Review & Editing; **Dagmar Kratky**: Conceptualization, Methodology, Funding acquisition, Resources, Supervision, Writing – Review & Editing.
