# Supplementary material for: Off-target effects of the lysosomal acid lipase inhibitors Lalistat-1 and Lalistat-2 on neutral lipid hydrolases
Source: Mol Metab. 2022 Apr 30;61:101510. doi: 10.1016/j.molmet.2022.101510 (PMC9118473; doi:10.1016/j.molmet.2022.101510)
Supplement: Multimedia component 1 [file mmc1.pdf]

Supplemental information

Off-target effects of the lysosomal acid lipase inhibitors Lalistat-1 and Lalistat-2 on neutral lipid hydrolases

Ivan Bradić, Katharina B. Kuentzel, Sophie Honeder, Gernot F. Grabner, Nemanja Vujić, Robert Zimmermann, Ruth Birner-Gruenberger, and Dagmar Kratky

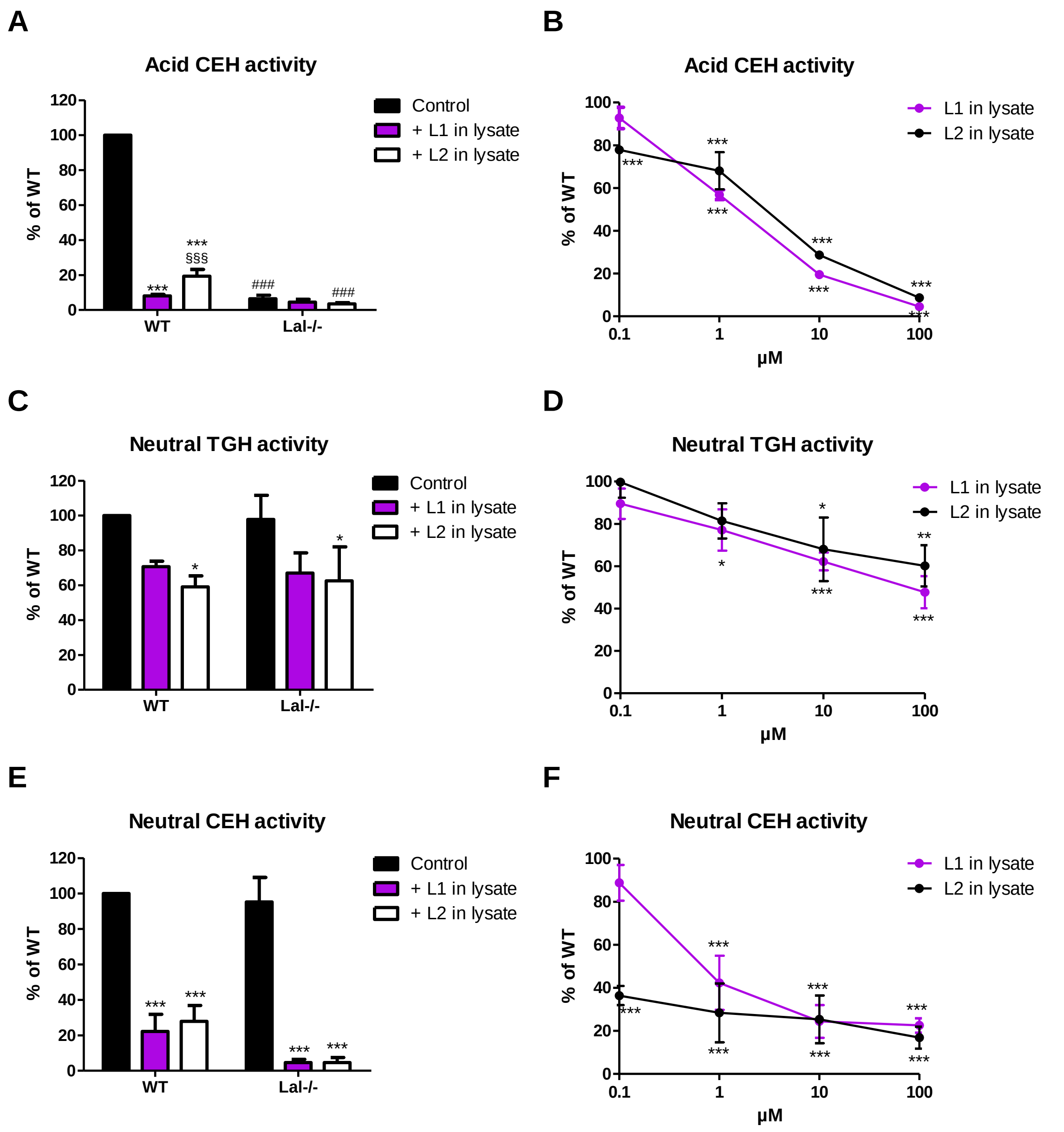

Figure S1: Lalistat-1 (L1) and L2 inhibit neutral lipid hydrolases in lysates of bone-marrow derived macrophages (BMDM). Bone marrow from WT and Lal-/- mice was differentiated to macrophages (BMDM) for 6 days. (A) Acid (pH 4.5) cholesteryl ester hydrolase (CEH), (C) neutral (pH 7) triglyceride hydrolase (TGH), and (E) neutral CEH activities in lysates of BMDM treated with 30 µM L1, L2, or 0.02% DMSO (control). Dose-dependent inhibition of L1 and L2 on (B) acid CEH, (D) neutral TGH, and (F) neutral CEH activity. Data (n = 3) represent mean ± SD. Lipid hydrolase activities were compared to untreated WT BMDM (arbitrarily set to 100%) and graphs represent the remaining hydrolase activity upon inhibitor treatment. Statistically significant differences were calculated by 1-way ANOVA followed by Bonferroni post-hoc test. * $p < 0.05$, ** $p \leq 0.01$, *** $p \leq 0.001$ for control versus L1 or L2 treatment within the same genotype; $^{###}$ $p \leq 0.001$ for comparing the same treatment between different genotypes; §§§ $p \leq 0.001$ for L1 versus L2 treatment within the same genotype.

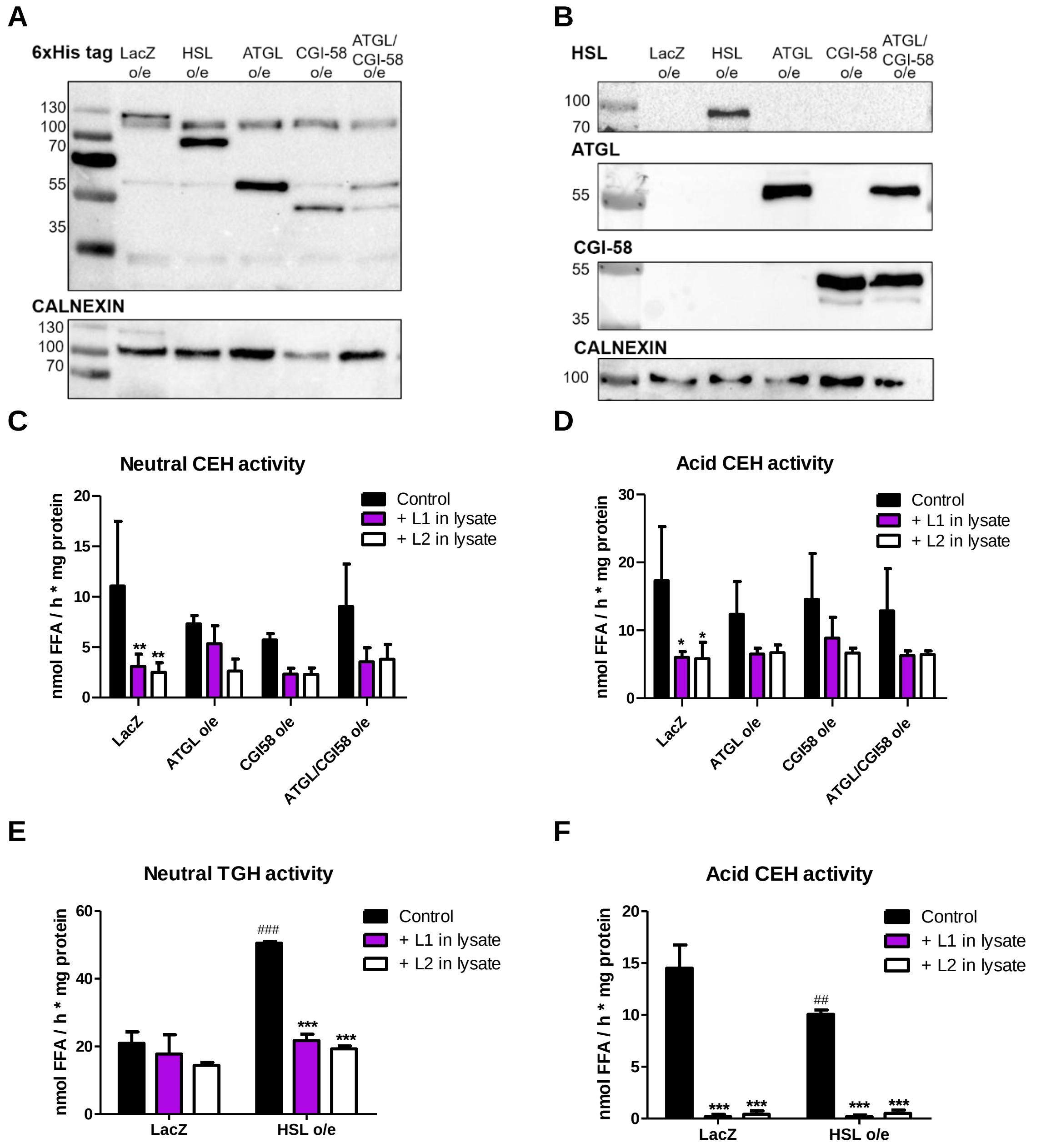

Figure S2: Lalistat-1 (L1) and L2 inhibit HSL. Mouse ATGL, HSL, and CGI-58 were cloned into a His-tagged pcDNA4/HisMax expression vector, and recombinant proteins were expressed in COS-7 cells. β-galactosidase (LacZ) was used as a control. His-tagged proteins were detected with (A) a monoclonal anti-His antibody or (B) HSL, ATGL, and CGI-58 antibodies in Western blotting experiments of cell extracts (25 µg protein). (C) Neutral cholesteryl ester hydrolase (CEH) and (D) acid CEH activity in lysates of COS7 cells overexpressing (o/e) ATGL, CGI-58, and ATGL/CGI-58 assayed in the presence of 30 µM L1 or L2. (E) Neutral (pH 7) triglyceride hydrolase (TGH) and (F) acid (pH 4.5) CEH activity of HSL o/e cell lysates. Data (n = 3) represent mean + SD. Statistically significant differences were calculated by 1-way ANOVA followed by Bonferroni post-hoc test; * $p < 0.05$, ** $p \leq 0.01$ for control versus L1 or L2 treatment within the same genotype; $^{\#\#}$ $p \leq 0.01$ for comparing the same treatment between different genotypes.

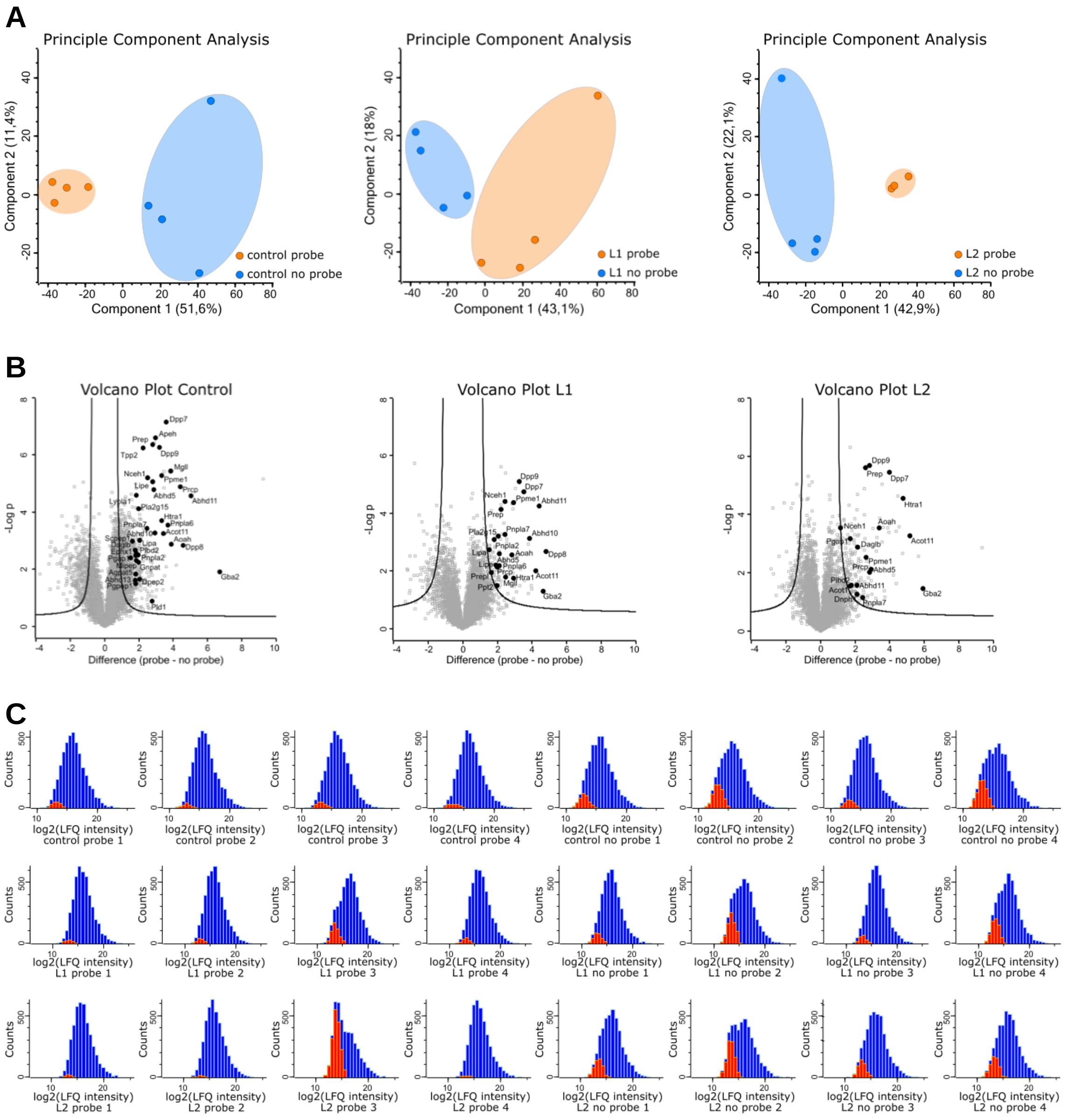

Figure S3: Activity-based proteomic profiling identified and quantitated serine hydrolases targeted by Lalistat-1 (L1) and L2. Bone marrow-derived macrophages were incubated with (probe) or without (no probe) ABP C6 and with 30 µM L1, L2 or 0.02% DMSO (control) (n = 4). Subsequent enrichment and label-free quantitation by mass spectrometry-based proteomics revealed (A) clustering of samples based on ABP C6 treatment, seen in principle component analysis based on protein abundance and (B) a number of significantly enriched serine hydrolases in the volcano plots. Statistical significance was determined by t-tests with permutation-based multiple testing correction; proteins that passed the significance threshold of the statistical tests are located outside the black lines on the volcano plot and serine hydrolases with a fold change > 3 are marked in black together with the corresponding gene names. (C) Histograms of log2 (LFQ intensities) visualize normal distribution of proteomics data (bars in red color represent imputed data).

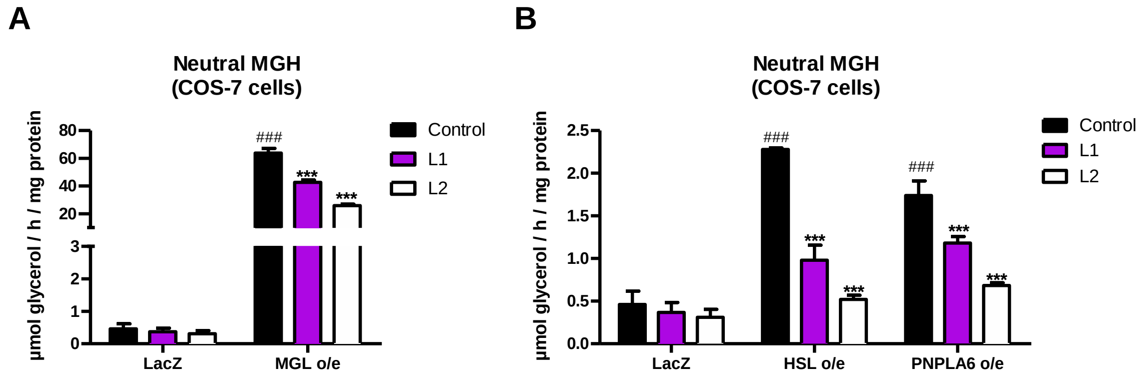

Figure S4: Lalistat-1 (L1) and L2 inhibit MGL and PNPLA6. Mouse MGL, HSL, and PNPLA6 were cloned into a His-tagged pcDNA4/HisMax expression vector, and recombinant proteins were expressed in COS-7 cells. β-galactosidase (LacZ) was used as a control. Neutral (pH 7) monoglyceride hydrolase (MGH) activity of COS7 cells overexpressing (o/e) (A) MGL, (B) HSL, and PNPLA6 assayed in the presence of 30 µM L1 or L2. Data (n = 3) represent mean + SD. Statistically significant differences were calculated by 1-way ANOVA followed by Bonferroni post-hoc test; *** $p \leq 0.001$ for control versus L1 or L2 treatment within the same genotype; ### $p \leq 0.001$ for comparing the same treatment between different genotypes.

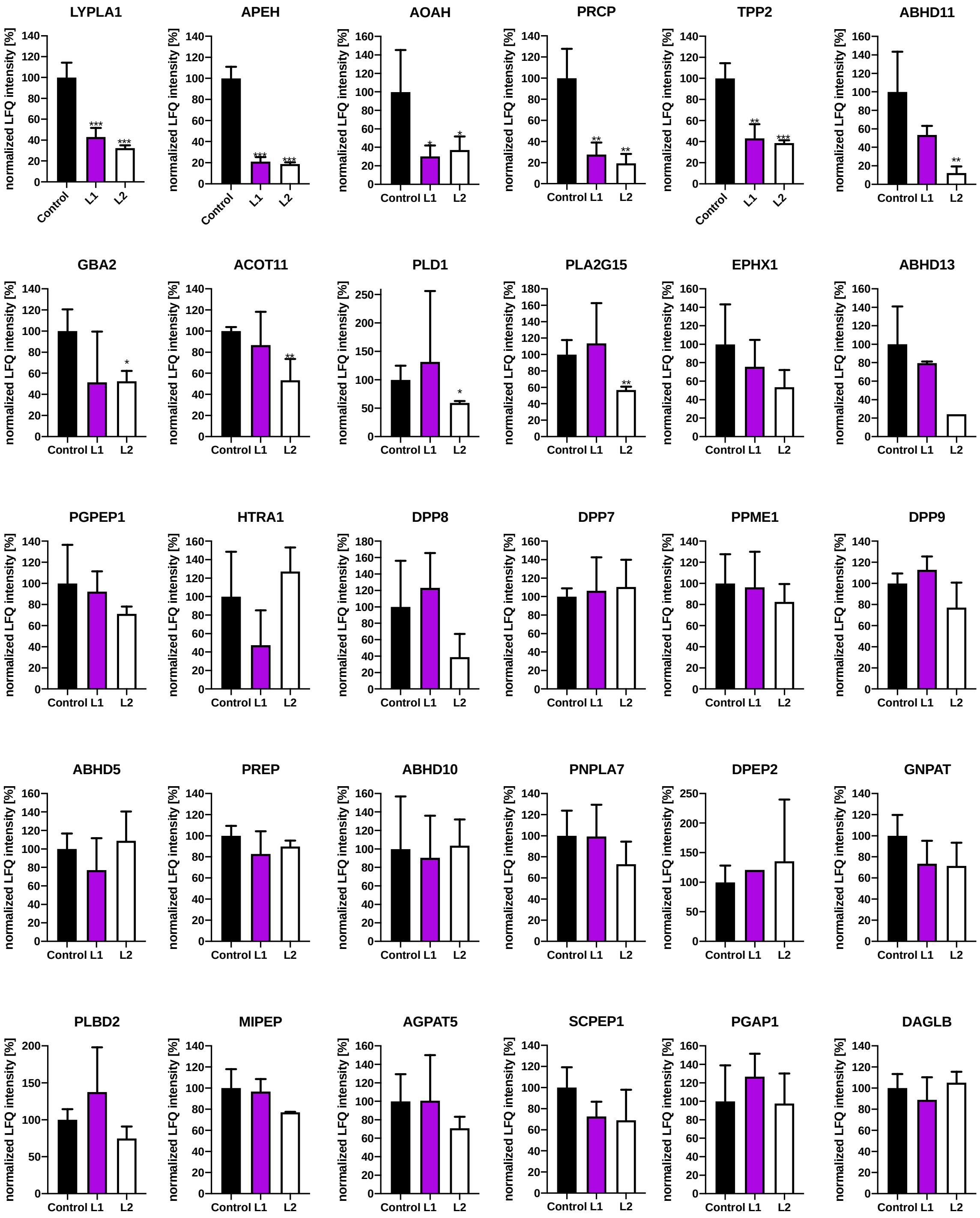

Figure S5: Lalistat-1 (L1) and L2 reduce activity-based probe binding of several serine hydrolases. Bone marrow-derived macrophages were treated with 30 µM L1, L2 or 0.02% DMSO (Control) for 20 h and labeled with the ABP C6 probe to enrich active serine hydrolases for activity-based proteomic profiling (ABPP). LFQ intensity values (representing probe binding in ABPP experiments) derived from label-free quantitation of MS data were normalized to control (arbitrarily set to 100%). Data (n = 4) represent mean + SD. Statistically significant differences were calculated by two-tailed Student's t-test; * $p < 0.05$, ** $p \leq 0.01$, *** ≤ 0.001 for control versus L1 or L2 treatment.

Table S1: Primers used for RT-qPCR

Gene	Forward Sequence 5’ – 3’	Reverse Sequence 5’ – 3’
Lal	GGATGAGTTCTGGGCCTTCAG	AAACCTATGGTGCAGCCTTGAG
Atgl	GCCACTCACATCTACGGAGC	GACAGCCACGGATGGTGTTC
Hsl	GATTTACGCACGATGACACAGT	ACCTGCAAAGACATTAGACAGC
Fabp4	AAGGTGAAGAGCATCATAACCCT	TCACGCCTTTCATAACACATTCC
Cd36	GCAGGTCTATCTACGCTGTG	GGTTGTCTGGATTCTGGAGG
Fasn	GAAGCCGAACACCTCTGTGCAGT	GCTCCTTGCTGCCATCTGTATTG
Scd1	CCGGAGACCCCTTAGATCGA	TAGCCTGTAAAAGATTTCTGCAAACC
Hmgcr	TGTTCACCGGCAACAACAAGA	CCGCGTTATCGTCAGGATGA
Srebf1	CACTCAGCAGCCACCATCTAGCCT	GCTGATGCCTGCAGTCTTCACG
Srebf2	TGAAGGACTTAGTCATGGGCAC	CGCAGCTTGTGATTGACCT
Dgat1	TCCGCCTCTGGGCATTC	GAATCGGCCCACAATCCA
Dgat2	AGTGGCAATGCTATCATCATCGT	TCTTCTGGACCCATCGGCCCCAGGA
Gpat1	ACAGTTGGCACAATAGAGGTTT	CCTTCCATTTCAGTGTTGCAGA
Agpat2	CAGCCAGGTTCTACGCCAAG	TGATGCTCATGTTATCCACGGT
Agpat3	CTGCTTGCCTACCTGAAGACC	GATACGGCGGTATAGGTGCTT
Pparg	GGAAGACCACTCGCATTCCTT	GTAATCAGCAACCATTGGGTCA

list of proteins in CONTROL samples:

log2 transformed LFQ intensity values (after removal of contaminants), missing values imputation from normal distribution (shift: 1.6; width: 0.4)

statistical test: two-sided t-test with a p-value of 0.05 and s0 of 1 and permutation-based FDR of 5 % (250 randomizations for multi-testing correction)

																			log2 transformed LFQ intensity values							
																			probe				no probe			
Significant	neg. log(p-value)	Difference (log2(p)-log2(np))	fold change (p/np)	Protein IDs	Protein names	Gene names	id	Peptides	Razor + unique peptides	Unique peptides	Sequence coverage [%]	Unique + razor sequence coverage [%]	Unique sequence coverage [%]	Mol. weight [kDa]	Q-value	Score	Intensity	MS/MS count	C+1	C+2	C+3	C+4	C-1	C-2	C-3	C-4
+	5.168	9.25	607.51	P35492	Histidine ammonia-lyase	Hal	1022	44	44	44	68	68	68	72.257	0.0000	323.31	146750000	393	20.17	19.75	19.75	19.94	12.32	10.00	10.88	9.42
+	1.898	6.72	105.44	Q69ZF3	Non-lysosomal glucosylceramidase	Gba2	2482	3	3	3	4.9	4.9	4.9	103.29	0.0002	5.3502	239110000	28	21.88	21.30	21.26	21.63	20.46	13.25	12.14	13.34
+	4.570	5.02	32.52	Q8K4F5	Alpha/beta hydrolase domain-containing protein 11	Abhd11	3715	12	12	12	60.9	60.9	60.9	33.56	0.0000	233.79	45328000	194	18.47	18.67	17.45	17.46	13.07	13.06	13.64	12.20
+	2.837	4.56	23.63	Q80YA7	Dipeptidyl peptidase 8	Dpp8	2900	22	21	21	25.4	24.6	24.6	102.18	0.0000	79.281	33327000	212	17.38	16.10	16.26	15.72	10.77	12.06	10.58	13.80
+	5.455	4.47	22.14	Q8BYH8	Chromodomain-helicase-DNA-binding protein 9	Chd9	3299	3	3	2	1.3	1.3	0.8	323.86	0.0073	1.9854	25571000	41	18.40	18.18	18.04	18.75	13.38	14.00	14.44	13.68
+	4.879	4.42	21.44	Q7TMR0	Lysosomal Pro-X carboxypeptidase	Prcp	2749	14	14	14	29.1	29.1	29.1	55.026	0.0000	119.84	114550000	296	18.91	19.03	18.18	18.37	14.84	13.52	14.33	14.12
+	3.646	4.31	19.82	Q5U4C3	Splicing factor, arginine/serine-rich 19	Scaf1	2165	5	5	5	5.4	5.4	5.4	133.84	0.0000	8.8592	40772000	9	17.06	17.62	17.84	17.65	14.55	13.17	13.22	11.99
+	2.149	4.17	18.03	Q80YR4	Zinc finger protein 598	Znf598	2905	5	5	5	7.7	7.7	7.7	99.191	0.0000	23.076	29529000	29	17.98	18.35	16.93	17.88	12.47	12.34	16.57	13.06
+	3.554	3.96	15.53	Q8BNI4	Derlin-2	Derl2	3154	2	2	2	16.3	16.3	16.3	27.639	0.0000	33.822	8609100	40	16.07	16.10	16.83	16.44	12.23	12.32	13.72	11.33
+	1.340	3.95	15.46	Q9WVM3	Anaphase-promoting complex subunit 7	Anapc7	5758	5	5	5	13.5	13.5	13.5	63.02	0.0000	7.876	31498000	38	18.74	18.67	19.17	19.34	11.32	16.47	18.50	13.82
+	2.881	3.89	14.78	O35298	Acyloxyacyl hydrolase;Acyloxyacyl hydrolase small subunit;Acylo	Aoah	354	14	14	14	25.1	25.1	25.1	65.154	0.0000	323.31	35804000	152	17.31	17.87	17.04	16.16	12.44	14.88	13.17	12.34
+	5.440	3.84	14.31	O35678	Monoglyceride lipase	Mgll	395	16	16	16	67.7	67.7	67.7	33.387	0.0000	140.93	41096000	144	17.15	16.84	16.69	16.15	13.15	12.95	12.76	12.61
+	3.535	3.70	12.98	Q3TRM4	Neuropathy target esterase	Pnpla6	1871	17	16	16	17.2	16.6	16.6	149.54	0.0000	126.55	12390000	130	17.03	15.54	16.96	16.74	13.37	11.91	13.41	12.79
+	2.087	3.60	12.15	P70158	Acid sphingomyelinase-like phosphodiesterase 3a	Smpdl3a	1528	15	15	15	42.2	42.2	42.2	49.857	0.0000	323.31	50300000	324	17.79	17.96	16.89	17.31	15.29	11.62	15.36	13.26
+	7.148	3.58	11.92	Q9ET22	Dipeptidyl peptidase 2	Dpp7	5354	20	20	20	48.6	48.6	48.6	56.253	0.0000	323.31	646300000	662	20.88	21.15	20.89	21.04	17.59	17.15	17.45	17.47
+	1.773	3.51	11.36	Q8BWW9	Serine/threonine-protein kinase N2	Pkn2	3269	7	6	6	9.4	8.5	8.5	111.61	0.0000	6.507	14609000	22	16.81	16.37	17.70	17.77	12.01	12.82	16.61	13.19
+	2.462	3.50	11.31	Q69ZF7	Metal transporter CNNM4	Cnnm4	2483	5	5	5	8	8	8	86.625	0.0000	16.584	11176000	39	18.02	16.96	17.79	17.26	13.45	15.28	15.04	12.26
+	5.422	3.47	11.07	A2AH22	Activating molecule in BECN1-regulated autophagy protein 1	Ambra1	22	3	3	3	3.9	3.9	3.9	142.88	0.0000	21.942	9893100	25	16.84	16.37	16.75	16.81	13.49	13.60	12.96	12.85
+	4.840	3.46	11.01	P16675	Lysosomal protective protein;Lysosomal protective protein 32 kD	Ctsa	813	21	21	21	43.2	43.2	43.2	53.844	0.0000	323.31	1641900000	1311	22.21	22.65	21.68	21.93	19.06	18.23	18.57	18.77
+	3.245	3.42	10.72	Q8VHQ9	Acyl-coenzyme A thioesterase 11	Acot11	4023	17	17	17	42.9	42.9	42.9	67.354	0.0000	323.31	67620000	316	17.16	17.27	17.22	17.28	14.23	13.38	15.01	12.63
+	1.281	3.39	10.51	P70245	3-beta-hydroxysteroid-Delta(8),Delta(7)-isomerase	Ebp	1538	2	2	2	10.9	10.9	10.9	26.215	0.0000	11.189	25463000	46	16.13	18.08	18.80	18.89	13.01	12.26	17.83	15.22
+	5.284	3.34	10.11	Q8BVQ5	Protein phosphatase methylesterase 1	Ppme1	3246	17	17	17	53.9	53.9	53.9	42.256	0.0000	200.22	64703000	347	17.87	17.04	17.43	17.11	14.33	13.90	14.05	13.81
+	3.701	3.32	9.97	Q9R118	Serine protease HTRA1	Htra1	5647	21	21	21	45.2	45.2	45.2	51.213	0.0000	202.98	42152000	249	16.10	16.22	17.46	17.45	13.23	13.58	13.94	13.21
+	6.273	3.21	9.24	Q8BVG4	Dipeptidyl peptidase 9	Dpp9	3239	41	41	40	57.4	57.4	57.2	98	0.0000	323.31	162960000	699	19.37	19.19	19.04	19.23	16.08	15.66	15.98	16.27
+	0.597	3.07	8.40	Q8CJ40	Rootletin	Crocc	3566	6	6	6	3	3	3	226.94	0.0044	2.4043	121400000	45	19.12	20.42	17.94	18.24	11.64	20.54	19.32	11.94
+	1.284	3.05	8.27	A2AJQ3	Probable C-mannosyltransferase DPY19L4	Dpy19l4	31	6	6	6	13.9	13.9	13.9	83.603	0.0000	10.727	24856000	32	18.13	16.96	18.09	17.09	17.67	13.90	14.73	11.77
+	2.658	2.99	7.92	Q6P4T0	Autophagy-related protein 2 homolog A	Atg2a	2565	8	8	8	9	9	9	210.94	0.0000	47.186	5079100	26	16.01	15.20	16.03	16.92	12.64	14.29	13.17	12.10
+	1.034	2.96	7.78	Q8BVW0	Neutral alpha-glucosidase C	Ganc	3249	5	5	5	8.6	8.6	8.6	102.01	0.0000	6.5977	21286000	8	17.93	12.28	16.18	17.11	11.22	12.57	12.85	15.04
+	6.606	2.95	7.75	Q8R146	Acylamino-acid-releasing enzyme	Apeh	3776	45	45	45	69	69	69	81.58	0.0000	323.31	388940000	1037	20.01	20.22	19.88	20.19	17.20	17.09	17.29	16.90
+	3.258	2.94	7.67	Q6PE15	Mycophenolic acid acyl-glucuronide esterase, mitochondrial	Abhd10	2623	17	17	17	63.3	63.3	63.3	33.04	0.0000	164.24	32945000	154	15.69	17.41	16.62	15.91	13.18	13.34	13.27	14.09
+	4.542	2.94	7.65	Q923D4	Splicing factor 3B subunit 5	Sf3b5	4283	6	6	6	68.6	68.6	68.6	10.119	0.0000	146.14	43936000	177	18.25	18.55	17.60	18.17	14.91	15.23	15.67	15.02
+	6.420	2.91	7.49	P41251	Natural resistance-associated macrophage protein 1	Slc11a1	1070	2	2	2	4.9	4.9	4.9	59.74	0.0000	6.6022	3576900	20	16.00	15.98	16.21	16.18	13.07	13.48	13.20	12.99
+	4.789	2.87	7.30	Q9DBL9	1-acylglycerol-3-phosphate O-acyltransferase ABHD5	Abhd5	5162	8	8	8	28.8	28.8	28.8	39.154	0.0000	59.913	21459000	104	16.58	16.55	16.04	16.58	13.49	13.08	13.75	13.95
+	1.530	2.85	7.23	Q91VU7		Pus7	4080	6	6	6	12.3	12.3	12.3	74.792	0.0000	11.175	13363000	20	17.57	16.78	16.77	16.05	12.78	12.71	16.75	13.52
+	6.358	2.80	6.96	Q9QUR6	Prolyl endopeptidase	Prep	5522	65	65	65	77.5	77.5	77.5	80.751	0.0000	323.31	840700000	1988	20.03	20.10	19.78	19.96	17.42	17.00	17.23	17.01
+	5.062	2.79	6.93	P54310	Hormone-sensitive lipase	Lipe	1251	34	34	34	64.8	64.8	64.8	83.347	0.0000	323.31	78384000	414	19.06	18.22	18.82	18.33	15.83	15.81	15.84	15.78
+	0.880	2.78	6.88	Q9Z280	Phospholipase D1	Pld1	5834	14	14	14	15.3	15.3	15.3	123.97	0.0000	54.275	70289000	118	20.24	19.35	20.15	20.18	18.35	18.84	19.12	12.47
+	1.215	2.78	6.85	Q91WF7	Polyphosphoinositide phosphatase	Fig4	4108	6	6	6	7.7	7.7	7.7	103.45	0.0000	24.906	29927000	36	18.47	16.53	17.93	17.77	17.46	12.44	16.04	13.67
+	1.792	2.78	6.85	Q91UZ5	Inositol monophosphatase 2	Impa2	4038	7	7	7	37.2	37.2	37.2	31.716	0.0000	119.24	9862900	90	16.50	16.06	16.40	15.88	13.49	12.08	15.74	12.43
+	2.224	2.77	6.83	Q6IRU2	Tropomyosin alpha-4 chain	Tpm4	2519	15	14	14	75.8	70.6	70.6	28.467	0.0000	89.828	18402000	144	16.75	16.90	15.77	16.42	15.18	12.23	14.01	13.34
+	1.056	2.74	6.68	P43024	Cytochrome c oxidase subunit 6A1, mitochondrial	Cox6a1	1085	4	4	4	59.5	59.5	59.5	12.352	0.0000	101.82	42226000	101	18.52	18.06	18.10	17.86	16.68	13.00	13.36	18.55
+	2.284	2.74	6.67	Q9CXW2	28S ribosomal protein S22, mitochondrial	Mrps22	4770	6	6	6	17.3	17.3	17.3	41.192	0.0000	7.7285	4951500	14	15.20	15.15	15.84	16.13	11.66	12.06	14.19	13.47
+	1.886	2.70	6.52	A2ABV5	Mediator of RNA polymerase II transcription subunit 14	Med14	16	2	2	2	2.1	2.1	2.1	160.96	0.0000	16.296	3790700	10	16.25	16.13	13.91	16.76	14.32	12.96	12.20	12.76
+	1.929	2.70	6.51	Q9JHJ3	Glycosylated lysosomal membrane protein	Glmp	5364	3	3	3	11.9	11.9	11.9	43.803	0.0000	42.36	8188400	43	17.08	16.14	15.21	14.91	11.49	13.24	13.70	14.11
+	3.238	2.70	6.49	Q99LJ1	Tissue alpha-L-fucosidase	Fuca1	4436	9	9	9	27.9	27.9	27.9	52.28	0.0000	113.14	19523000	117	17.76	17.35	17.17	17.13	15.26	13.82	15.34	14.19
+	2.276	2.68	6.40	Q61387	Cytochrome c oxidase subunit 7A-related protein, mitochondrial	Cox7a2l	2295	5	5	5	62.2	62.2	62.2	12.399	0.0000	58.859	4429300	39	14.95	16.68	16.85	14.71	13.78	13.34	12.91	12.45
+	1.429	2.65	6.30	P46735	Unconventional myosin-Ib	Myo1b	1111	4	4	4	6.9	6.9	6.9	128.56	0.0000	22.553	8479300	14	17.68	16.21	16.21	16.45	12.35	13.27	16.66	13.65
+	0.625	2.59	6.02	Q7TSS2	Ubiquitin-conjugating enzyme E2 Q1	Ube2q1	2788	6	6	6	16.8	16.8	16.8	46.172	0.0000	11.81	194050000	35	20.50	21.38	20.77	20.87	20.62	12.49	19.27	20.79
+	1.749	2.59	6.00	Q9JK38	Glucosamine 6-phosphate N-acetyltransferase	Gnpnat1	5443	3	3	3	23.4	23.4	23.4	20.791	0.0000	12.001	6672800	44	16.93	15.93	15.80	15.47	15.61	12.60	13.11	12.46
+	1.130	2.58	5.98	Q924N4	Solute carrier family 12 member 6	Slc12a6	4298	8	8	6	11.3	11.3	9.1	127.53	0.0000	59.721	9992000	94	16.19	15.92	16.55	16.37	10.95	15.51	15.82	12.43
+	0.658	2.55	5.86	Q5S006	Leucine-rich repeat serine/threonine-protein kinase 2	Lrrk2	2129	4	4	4	2.5	2.5	2.5	284.73	0.0020	3.0891	18011000	12	17.62	12.55	18.78	19.11	12.62	16.23	12.61	16.41
+	1.060	2.55	5.85	Q9CQK7	RWD domain-containing protein 1	Rwdd1	4608	2	2	2	11.1	11.1	11.1	27.785	0.0046	2.3688	11504000	14	16.29	16.86	15.68	11.93	10.96	13.26	12.97	13.38
+	0.771	2.52	5.72	P58801	Receptor-interacting serine/threonine-protein kinase 2	Ripk2	1323	9	9	9	25.2	25.2	25.2	60.399	0.0000	28.031	82728000	32	19.64	18.83	19.25	21.32	18.40	18.62	19.24	12.73
+	1.464	2.51	5.70	Q9D3E6	Cohesin subunit SA-1	Stag1	4965	8	5	4	9.8	7.2	5.4	144.44	0.0056	2.2214	9610600	4	16.58	16.27	12.74	16.01	12.44	13.08	12.63	13.42
+	5.191	2.51	5.68	Q8BLF1	Neutral cholesterol ester hydrolase 1	Nceh1	3117	31	31	31	67.2	67.2	67.2	45.739	0.0000	323.31	1348900000	1512	23.14	22.73	23.19	22.89	20.50	20.26	20.85	20.32
+	3.468	2.48	5.60	A6H5Z3	Exocyst complex component 6B	Exoc6b	60	30	30	29	44.1	44.1	43.1	94.128	0.0000	205.61	38086000	310	16.25	15.21	15.23	15.34	13.60	12.75	13.17	12.56
+	1.237	2.48	5.56	Q9CS00	Cactin	Cactin	4696	5	5	5	8.8	8.8	8.8	90.66	0.0000	26.286	8974700	16	16.63	15.99	15.35	16.02	11.60	15.33	15.26	11.89
+	3.431	2.47	5.55	A2AJ88	Patatin-like phospholipase domain-containing protein 7	Pnpla7	28	34	34	33	35.9	35.9	35.4	150.49	0.0000	250.69	58040000	439	17.19	16.36	17.16	16.98	14.49	13.72	15.11	14.50
+	0.934	2.46	5.51	O35309	N-myc-interactor	Nmi	355	4	4	4	18.5	18.5	18.5	35.235	0.0002	5.4976	76893000	32	20.20	20.40	19.29	20.05	18.78	18.15	19.52	13.66
+	1.896	2.46	5.49	Q3U9N9	Monocarboxylate transporter 10	Slc16a10	1937	1	1	1	3.5	3.5	3.5	55.32	0.0000	50.222	10650000	70	17.07	16.66	17.10	16.84	14.40	14.60	16.12	12.73
+	2.693	2.46	5.49	Q8BKC8	Phosphatidylinositol 4-kinase beta	Pi4kb	3099	4	4	4	6.9	6.9	6.9	91.514	0.0000	11.659	4100500	22	16.14	15.01	15.73	15.39	12.79	12.44	14.29	12.93
+	2.376	2.41	5.31	Q7TMY7	Importin-8	Ipo8	2753	6	5	5	8.6	7.6	7.6	117.08	0.0000	8.5303	3583000	16	16.92	14.69	15.97	15.77	13.35	13.31	12.85	14.20
+	2.564	2.41	5.31	Q64435	UDP-glucuronosyltransferase 1-6	Ugt1a6	2442	13	4	4	31.3	13.4	13.4	60.438	0.0000	13.465	5339700	53	15.54	15.51	15.00	15.45	11.80	12.62	13.50	13.94
+	2.704	2.40	5.29	Q8BMC0	Lysophosphatidic acid receptor 6	Lpar6	3132	3	3	3	11.3	11.3	11.3	39.438	0.0000	16.053	3537500	23	14.81	14.77	15.81	14.55	12.12	13.52	12.80	11.90
+	2.822	2.37	5.15	Q8VE09	Tetratricopeptide repeat protein 39C	Ttc39c	3979	7	7	7	17.4	17.4	17.4	65.444	0.0000	51.153	6001800	56	14.56	14.68	14.93	15.00	11.32	13.32	12.37	12.69
+	1.658	2.36	5.15	O55028	[3-methyl-2-oxobutanoate dehydrogenase [lipoamide]] kinase, m	Bckdk	444	9	9	9	36.7	36.7	36.7	46.587	0.0000	87.174	6868000	55	16.51	15.98	14.00	15.40	13.79	11.66	14.11	12.86
+	2.490	2.36	5.14	Q8VEK0	Cell cycle control protein 50A	Tmem30a	4010	4	4	4	15.9	15.9	15.9	41.06	0.0000	14.378	4216500	28	15.66	14.96	15.88	15.74	12.30	14.11	12.53	13.85
+	1.561	2.35	5.10	O35316	Sodium- and chloride-dependent taurine transporter	Slc6a6	356	4	4	4	7.9	7.9	7.9	69.855	0.0000	34.287	26750000	66	17.61	18.01	16.87	16.83	15.58	15.21	12.82	16.32
+	3.375	2.34	5.08	Q99LG4	Tetratricopeptide repeat protein 5	Ttc5	4428	5	5	5	15.9	15.9	15.9	48.795	0.0000	12.375	3524100	15	14.81	14.84	15.16	15.46	12.91	13.42	12.01	12.56
+	1.313	2.34	5.06	Q80Y56	Rabenosyn-5	Rbsn	2895	4	4	4	9.7	9.7	9.7	88.49	0.0000	76.184	3600200	58	16.29	15.41	15.02	16.11	14.84	11.44	14.97	12.23
+	1.537	2.34	5.05	E9Q309		Cep350	231	3	3	3	1.6	1.6	1.6	346.45	0.0044	2.4056	7828100	11	17.28	17.70	16.25	17.10	15.84	12.73	16.00	14.42
+	1.563	2.32	5.00	Q80U70	Polycomb protein Suz12	Suz12	2822	4	4	4	8.1	8.1	8.1	83.025	0.0000	16.602	9233700	24	15.83	16.01	16.60	16.62	14.14	13.64	15.88	12.12
+	2.421	2.29	4.88	Q8K409	DNA polymerase beta	Polb	3710	5	5	5	20	20	20	38.287	0.0000	10.433	5040400	20	15.60	15.98	16.35	16.63	14.42	12.63	14.62	13.76
+	2.447	2.27	4.83	O35379	Multidrug resistance-associated protein 1	Abcc1	364	49	49	47	40.8	40.8	39.5	171.18	0.0000	323.31	98211000	662	17.36	16.34	17.18	17.27	14.81	13.60	15.00	15.66
+	6.254	2.25	4.76	Q64514	Tripeptidyl-peptidase 2	Tpp2	2449	84	84	84	63.8	63.8	63.8	139.88	0.0000	323.31	581750000	2091	20.96	20.55	20.54	20.69	18.42	18.37	18.48	18.46
+	3.450	2.25	4.76	Q61239	Protein farnesyltransferase/geranylgeranyltransferase type-1 sub	Fnta	2284	19	19	19	53.1	53.1	53.1	44.013	0.0000	130.58	20787000	130	15.97	15.91	15.44	15.15	13.97	13.03	13.53	12.93
+	2.278	2.23	4.70	Q8BPN8	DmX-like protein 2	Dmxl2	3166	8	8	8	4.6	4.6	4.6	338.2	0.0000	27.593	5781700	34	16.32	15.50	17.65	16.89	14.66	14.44	14.74	13.59
+	0.604	2.23	4.69	Q9CPX8	Cytochrome b-c1 complex subunit 10	Uqcr11	4543	3	3	3	41.1	41.1	41.1	6.5386	0.0025	2.8287	23947000	20	16.97	12.49	17.08	17.43	17.61	12.92	12.33	12.18
+	4.088	2.21	4.63	Q02819	Nucleobindin-1	Nucb1	1720	15	15	15	34	34	34	53.408	0.0000	83.253	12525000	160	15.74	16.04	15.41	15.56	14.04	13.21	13.37	13.28
	0.418	2.20	4.60	Q6A009	E3 ubiquitin-protein ligase listerin	Ltn1	2494	3	3	3	4.5	4.5	4.5	198.92	0.0000	13.423	28419000	21	12.38	19.91	18.83	18.85	17.84	13.22	18.13	11.98
+	1.957	2.19	4.57	Q9DBM2	Peroxisomal bifunctional enzyme;Enoyl-CoA hydratase/3,2-trans	Ehhadh	5164	7	7	6	13	13	11	78.301	0.0000	6.6723	13039000	27	17.93	16.70	17.57	18.29	14.14	15.21	15.95	16.43
+	1.619	2.19	4.55	Q4VAC9	Pleckstrin homology domain-containing family G member 3	Plekhg3	2068	4	4	4	4.5	4.5	4.5	148.52	0.0000	7.4195	14687000	12	17.94	17.15	17.26	17.35	15.65	13.64	16.97	14.70
+	0.988	2.18	4.53	Q9WVD4	H(+)/Cl(-) exchange transporter 5	Clcn5	5745	5	5	5	8.7	8.7	8.7	90.705	0.0000	33.373	13932000	68	17.36	16.23	17.66	17.67	16.37	11.99	16.75	15.09
+	3.139	2.16	4.46	Q8K2J7	RELT-like protein 1	Rell1	3663	4	4	4	29.8	29.8	29.8	29.351	0.0000	18.216	2485700	32	14.59	14.04	13.90	14.57	11.62	11.65	12.79	12.41
+	1.903	2.15	4.44	Q5DTY9;Q	BTB/POZ domain-containing protein KCTD16;BTB/POZ domain-	Kctd16;Kctd8	2097	2	1	1	4.2	2.6	2.6	48.972	0.0004	4.3652	2707000	27	16.32	15.21	13.72	14.40	12.56	12.18	13.00	13.30
+	0.689	2.14	4.42	Q8CIV2	Membralin	Tmem259	3562	3	3	3	6.8	6.8	6.8	63.56	0.0000	6.5267	30502000	25	19.24	18.40	18.24	18.61	18.21	17.60	18.08	12.03
+	1.719	2.13	4.38	Q8R4U7	Leucine zipper protein 1	Luzp1	3873	13	13	13	14.7	14.7	14.7	119.31	0.0000	40.931	11861000	37	16.93	14.94	17.29	15.63	15.22	13.74	13.61	13.70
+	1.388	2.13	4.36	C0HKG6;C0HKG5			95	3	3	3	10	10	10	29.608	0.0054	2.2337	5268300	13	16.24	16.08	16.87	13.29	13.56	12.93	13.81	13.68
+	3.746	2.12	4.34	Q80XC2	tRNA (adenine(58)-N(1))-methyltransferase catalytic subunit TRM	Trmt61a	2879	6	6	6	40	40	40	31.639	0.0000	85.958	6244600	81	15.60	14.95	15.19	15.35	13.50	12.63	13.53	12.94
+	3.214	2.11	4.31	Q8BXA5	Cleft lip and palate transmembrane protein 1-like protein	Clptm1l	3284	2	2	2	5.4	5.4	5.4	62.183	0.0000	9.7294	4963500	60	15.59	15.23	15.21	14.81	13.74	12.84	13.37	12.47
+	1.265	2.10	4.29	Q9QYC7	Vitamin K-dependent gamma-carboxylase	Ggcx	5569	5	5	5	9.2	9.2	9.2	87.194	0.0000	14.203	6240000	58	16.11	13.55	15.78	15.48	11.95	12.14	13.65	14.78
+	4.117	2.10	4.29	P49710	Hematopoietic lineage cell-specific protein	Hcls1	1167	34	34	34	56.2	56.2	56.2	54.24	0.0000	323.31	148070000	749	19.48	19.68	19.19	19.47	17.83	17.20	17.48	16.91
+	1.504	2.10	4.28	Q9CPZ6	ORM1-like protein 3	Ormdl3	4547	1	1	1	9.8	9.8	9.8	17.476	0.0000	35.502	8986000	73	16.99	16.65	16.81	16.51	13.04	15.70	16.10	13.73
+	1.444	2.09	4.25	Q9CQB5	CDGSH iron-sulfur domain-containing protein 2	Cisd2	4581	3	3	3	36.3	36.3	36.3	15.242	0.0000	75.216	7765000	75	15.23	15.14	16.21	15.66	12.17	13.97	15.33	12.41
+	3.241	2.08	4.24	Q8BGD9	Eukaryotic translation initiation factor 4B	Eif4b	2978	27	27	26	51.4	51.4	49.9	68.839	0.0000	162.39	44645000	257	18.25	18.21	17.07	18.05	15.96	15.83	16.05	15.39
+	2.008	2.07	4.21	Q3UVG3	Protein FAM91A1	Fam91a1	2037	12	12	12	19.1	19.1	19.1	93.462	0.0000	47.67	7437400	83	15.52	14.82	14.39	15.19	14.00	11.91	12.23	13.49
+	0.666	2.07	4.19	O09005	Sphingolipid delta(4)-desaturase DES1	Degs1	316	5	5	5	16.1	16.1	16.1	38.241	0.0000	78.185	59348000	138	19.05	18.95	18.76	18.58	18.27	17.74	18.71	12.33
+	3.915	2.06	4.18	Q9DBB5	Eukaryotic translation initiation factor 4E type 3	Eif4e3	5139	6	6	6	30	30	30	22.836	0.0000	10.883	4904800	44	15.23	14.77	15.49	15.45	13.12	12.83	13.63	13.11
+	3.020	2.06	4.18	Q9Z0M5	Lysosomal acid lipase/cholesteryl ester hydrolase	Lipa	5776	16	16	16	41.6	41.6	41.6	45.325	0.0000	323.31	121540000	361	20.02	19.56	19.33	19.84	17.73	16.77	17.79	18.22

+	1.806	2.06	4.16	Q3UKC1	Tax1-binding protein 1 homolog	Tax1bp1	1987	6	6	6	8.5	8.5	8.5	93.629	0.0000	21.07	4571000	47	15.49	15.02	15.43	15.72	12.20	14.44	14.35	12.46
+	1.878	2.06	4.16	Q61133	Glutathione S-transferase theta-2	Gstt2	2256	4	4	4	19.7	19.7	19.7	27.634	0.0046	2.3758	8185600	15	16.25	15.87	17.25	15.72	15.39	13.22	14.57	13.69
	0.479	2.05	4.15	Q9DCZ4	Apolipoprotein O	Apoo	5238	6	6	6	38.9	38.9	38.9	22.604	0.0000	42.086	37077000	27	18.29	18.00	19.36	11.39	16.71	13.35	14.31	14.46
+	1.649	2.05	4.15	Q8C255	Dipeptidase 2	Dpep2	3369	3	3	3	10.3	10.3	10.3	52.664	0.0000	17.834	4692800	38	16.07	16.11	16.58	15.58	15.50	13.24	14.67	12.72
+	2.814	2.05	4.14	Q9JJA2	Conserved oligomeric Golgi complex subunit 8	Cog8	5421	10	10	10	29.2	29.2	29.2	71.601	0.0000	53.045	5428000	39	15.72	14.18	14.83	14.84	12.30	13.25	12.84	12.97
+	2.132	2.05	4.14	Q3UUG6	TBC1 domain family member 24	Tbc1d24	2031	4	4	4	9.8	9.8	9.8	63.235	0.0000	11.161	2967100	35	14.77	14.40	15.12	14.12	11.86	13.79	11.80	12.76
+	2.722	2.05	4.13	Q9R0C8	Guanine nucleotide exchange factor VAV3	Vav3	5622	11	10	10	16.6	15.5	15.5	97.967	0.0000	61.044	6007200	59	14.82	15.05	14.84	15.30	12.93	13.73	13.21	11.96
+	3.753	2.04	4.12	Q9WVG6	Histone-arginine methyltransferase CARM1	Carm1	5748	10	10	10	27.1	27.1	27.1	65.853	0.0000	69.757	13793000	163	15.85	15.27	15.97	15.51	13.61	13.47	14.13	13.23
+	1.921	2.04	4.11	Q8R366	Immunoglobulin superfamily member 8	Igsf8	3838	6	6	6	12.6	12.6	12.6	65.01	0.0000	45.641	4709600	39	15.55	15.38	16.03	15.50	13.16	12.80	15.23	13.12
+	1.847	2.03	4.08	Q9D338	39S ribosomal protein L19, mitochondrial	Mrpl19	4958	4	4	3	17.1	17.1	13.7	33.578	0.0000	6.2952	6352900	21	16.18	14.66	14.92	16.42	13.60	12.38	13.91	14.17
+	1.983	2.02	4.07	Q9D659	Platelet receptor Gi24		4989	2	2	2	14	14	14	33.559	0.0000	45.976	4618700	34	14.12	15.45	15.63	15.49	11.92	13.78	13.32	13.58
+	2.494	2.02	4.07	O35887	Calumenin	Calu	409	6	6	6	21	21	21	37.063	0.0000	13.659	4702600	42	15.22	15.86	15.32	15.55	13.22	13.84	14.33	12.46
+	2.922	2.02	4.07	Q8R3H7	Heparan sulfate 2-O-sulfotransferase 1	Hs2st1	3847	3	3	3	12.1	12.1	12.1	41.813	0.0000	16.406	5648700	57	15.32	14.83	15.37	15.41	12.93	14.12	13.15	12.62
+	1.756	2.02	4.07	Q8JZK9	Hydroxymethylglutaryl-CoA synthase, cytoplasmic	Hmgcs1	3573	6	6	6	21.2	21.2	21.2	57.568	0.0000	26.945	4533900	51	15.54	14.89	16.04	14.24	13.64	14.10	11.86	13.02
+	1.594	2.02	4.05	P50431	Serine hydroxymethyltransferase, cytosolic	Shmt1	1183	11	10	10	32	29.1	29.1	52.6	0.0000	30.309	10398000	82	16.19	15.57	16.19	16.24	14.30	14.63	15.09	12.10
+	2.184	2.01	4.03	Q8BGB2	Tetratricopeptide repeat protein 7A	Ttc7a	2972	4	4	4	7.1	7.1	7.1	96.155	0.0000	16.308	3907800	28	15.55	14.52	14.99	14.58	12.44	13.35	13.86	11.95
+	2.763	1.99	3.98	Q91YN5	UDP-N-acetylhexosamine pyrophosphorylase;UDP-N-acetylgala	Uap1	4168	5	4	4	15.9	13.4	13.4	58.608	0.0034	2.5012	1618900	4	14.82	15.21	15.00	13.96	12.21	12.47	13.31	13.02
+	1.408	1.99	3.98	Q80U95	Ubiquitin-protein ligase E3C	Ube3c	2827	5	5	5	7.7	7.7	7.7	123.97	0.0000	12.056	13068000	39	16.91	16.84	17.64	17.23	13.80	16.15	16.69	14.00
+	3.600	1.98	3.95	P34152	Focal adhesion kinase 1	Ptk2	1006	7	6	6	10.9	10.2	10.2	119.24	0.0000	40.006	2307000	35	14.08	13.77	14.48	13.85	12.26	11.97	12.47	11.54
+	2.046	1.97	3.93	Q9CTG6	Probable cation-transporting ATPase 13A2	Atp13a2	4703	7	7	7	11.1	11.1	11.1	126.44	0.0000	96.804	8874900	61	15.72	15.60	16.33	15.95	13.38	14.67	14.85	12.81
+	2.249	1.97	3.92	P98192	Dihydroxyacetone phosphate acyltransferase	Gnpat	1688	5	5	5	11.5	11.5	11.5	76.869	0.0000	16.907	4635100	39	15.37	14.79	15.19	15.48	13.93	12.70	14.05	12.27
+	4.118	1.97	3.90	Q8VEB4	Group XV phospholipase A2	Pla2g15	3998	12	12	12	29.9	29.9	29.9	47.307	0.0000	74.85	84748000	235	17.93	17.98	17.42	18.02	15.96	15.63	16.26	15.64
+	2.491	1.96	3.89	Q8BJ56	Patatin-like phospholipase domain-containing protein 2	Pnpla2	3072	12	12	12	42.2	42.2	42.2	53.656	0.0000	48.623	15457000	137	16.89	16.01	16.71	16.57	13.79	15.37	14.15	15.03
+	2.685	1.95	3.87	P70288	Histone deacetylase 2	Hdac2	1546	16	9	9	44.9	32.6	32.6	55.302	0.0000	48.131	12566000	131	15.95	15.56	15.87	15.45	14.38	12.73	14.05	13.86
+	1.513	1.95	3.86	Q8BMI0	F-box only protein 38	Fbxo38	3138	7	7	7	8.9	8.9	8.9	133.93	0.0000	22.409	5414100	42	15.41	14.65	15.72	15.14	14.37	12.79	14.31	11.65
+	1.923	1.94	3.84	Q8R313	Exocyst complex component 6	Exoc6	3829	9	8	8	15.2	14.1	14.1	93.076	0.0000	32.597	9201400	82	16.27	16.03	16.04	16.24	14.48	12.60	14.90	14.83
+	1.927	1.94	3.83	O35387	HCLS1-associated protein X-1	Hax1	367	3	3	3	18.2	18.2	18.2	31.654	0.0000	65.581	1691800	17	14.17	14.09	14.56	14.81	13.32	13.41	11.43	11.75
+	1.511	1.93	3.81	Q9JID9	SH2B adapter protein 2	Sh2b2	5394	3	3	3	8.1	8.1	8.1	66.557	0.0000	74.052	5513000	33	15.28	15.57	16.31	16.15	12.89	13.79	15.75	13.18
+	2.504	1.93	3.81	Q9D0K0	TBC1 domain family member 7	Tbc1d7	4871	3	3	3	16.4	16.4	16.4	33.826	0.0000	10.028	2754300	29	15.13	14.41	14.65	14.91	13.18	12.91	13.51	11.79
+	1.639	1.92	3.77	Q9DBU3	Serine/threonine-protein kinase RIO3	Riok3	5179	6	6	6	18.1	18.1	18.1	58.704	0.0000	15.82	5079100	39	14.97	15.12	15.91	15.75	14.50	12.18	14.51	12.89
+	1.578	1.91	3.76	Q8R123	FAD synthase;Molybdenum cofactor biosynthesis protein-like reg	Flad1	3773	6	6	6	20.5	20.5	20.5	54.766	0.0000	59.061	11234000	114	15.71	15.49	16.37	16.04	13.91	14.24	15.42	12.39
+	3.046	1.90	3.74	Q8BKX1	Brain-specific angiogenesis inhibitor 1-associated protein 2	Baiap2	3107	11	11	11	35	35	35	59.236	0.0000	89.507	7303400	70	15.49	14.92	16.09	15.04	13.82	13.09	13.70	13.32
+	1.959	1.90	3.74	Q8K358	Phosphatidylinositol glycan anchor biosynthesis class U protein	Pigu	3691	3	3	3	6.7	6.7	6.7	49.804	0.0000	44.154	5597300	53	16.25	15.52	16.14	16.12	15.12	14.67	12.88	13.77
+	1.289	1.90	3.73	P11531	Dystrophin	Dmd	746	6	6	6	2.5	2.5	2.5	425.83	0.0000	9.7677	7703900	31	16.54	14.89	16.55	16.00	15.11	12.42	15.28	13.57
+	2.418	1.90	3.72	Q3ULF4	Paraplegin	Spg7	1992	17	17	17	30.9	30.9	30.9	85.995	0.0000	72.375	15476000	155	15.84	15.51	16.41	16.14	13.73	13.36	15.07	14.16
+	2.350	1.89	3.70	O54885	TYRO protein tyrosine kinase-binding protein	Tyrobp	430	2	2	2	25.4	25.4	25.4	12.367	0.0000	56.103	4382600	26	15.39	15.07	14.38	15.02	12.22	12.72	13.88	13.47
+	2.103	1.87	3.67	Q69Z37	Sterile alpha motif domain-containing protein 9-like	Samd9l	2478	26	26	25	19.6	19.6	19	180.2	0.0000	85.874	14810000	105	16.59	15.73	15.47	15.60	14.84	13.06	14.45	13.55
+	2.558	1.87	3.65	Q3TCN2	Putative phospholipase B-like 2;Putative phospholipase B-like 2	Plbd2	1828	12	12	12	27.9	27.9	27.9	66.289	0.0000	166.15	27345000	212	17.39	17.01	16.95	17.10	15.46	14.24	16.01	15.28
+	0.721	1.86	3.63	Q8BLK9	Ribosomal protein S6 kinase delta-1	Rps6kc1	3119	6	6	6	8.6	8.6	8.6	115.71	0.0000	16.751	7009600	19	17.17	16.43	13.20	17.45	13.04	13.53	16.54	13.69
+	4.594	1.86	3.63	P97823	Acyl-protein thioesterase 1	Lypla1	1679	13	13	13	59.6	59.6	59.6	24.687	0.0000	214.27	83388000	232	17.49	17.66	17.46	17.15	15.43	15.43	15.51	15.94
+	1.804	1.86	3.62	Q5Y5T1	Probable palmitoyltransferase ZDHHC20	Zdhhc20	2176	4	4	4	16.8	16.8	16.8	43.975	0.0000	45.802	6717600	77	15.53	15.37	16.40	15.47	14.48	13.16	14.89	12.81
+	0.949	1.85	3.60	Q6ZPU9	KIF1-binding protein	Kbp	2684	7	7	7	16.5	16.5	16.5	71.051	0.0000	29.654	13165000	39	17.62	15.93	17.25	17.16	16.49	13.84	16.95	13.28
+	1.364	1.85	3.59	Q8VE10	N-alpha-acetyltransferase 40	Naa40	3980	3	3	3	14.8	14.8	14.8	27.229	0.0000	14.066	4633200	52	15.37	14.26	16.08	15.75	15.00	12.98	13.92	12.19
+	2.296	1.84	3.57	A6H611	Mitochondrial intermediate peptidase	Mipep	61	6	6	6	10.1	10.1	10.1	80.851	0.0000	19.92	6390800	53	15.75	15.24	15.88	15.77	14.50	13.31	14.52	12.96
+	4.831	1.83	3.57	Q6PFR5	Transformer-2 protein homolog alpha	Tra2a	2629	6	5	5	22.1	18.5	18.5	32.316	0.0000	15.823	12407000	59	16.97	17.35	17.10	17.31	15.31	15.67	15.27	15.14
+	4.159	1.83	3.55	Q8BUY9	Geranylgeranyl transferase type-1 subunit beta	Pggt1b	3233	6	6	5	21.8	21.8	16.2	42.354	0.0000	29.669	7461500	57	15.24	14.39	14.77	14.80	12.86	13.12	12.84	13.08
+	1.657	1.82	3.54	Q9CXI5	Mesencephalic astrocyte-derived neurotrophic factor	Manf	4757	7	7	7	26.8	26.8	26.8	20.373	0.0000	25.834	14629000	47	14.99	16.58	13.96	16.15	13.57	13.75	13.49	13.58
+	1.392	1.82	3.54	Q9D8N2	Protein FAM45A	Fam45a	5066	6	6	6	26.6	26.6	26.6	40.42	0.0000	87.408	5548000	56	15.88	14.55	15.01	14.09	11.91	12.20	14.16	13.97
+	2.470	1.82	3.53	Q9D379	Epoxide hydrolase 1	Ephx1	4961	18	18	18	33	33	33	52.576	0.0000	77.051	22651000	157	17.10	15.91	16.03	16.16	14.21	14.13	14.26	15.31
+	4.991	1.82	3.53	Q9QYF9	Protein NDRG3	Ndrg3	5572	6	6	5	23.5	23.5	22.4	41.555	0.0000	23.775	2625800	24	14.67	14.45	14.26	14.59	12.95	12.48	12.63	12.64
+	2.763	1.82	3.52	Q3UHQ6	Protein dopey-2	Dopey2	1975	13	13	12	8.7	8.7	8.1	257.48	0.0000	62.909	8616800	57	16.12	15.47	16.27	15.92	14.77	14.15	14.24	13.36
+	1.833	1.82	3.52	Q9D1E8	1-acyl-sn-glycerol-3-phosphate acyltransferase epsilon	Agpat5	4909	7	7	7	20.3	20.3	20.3	42.202	0.0000	23.491	11898000	82	16.26	15.63	15.49	15.41	14.46	13.73	14.79	12.54
+	1.498	1.81	3.51	P59108	Copine-2	Cpne2	1330	10	9	9	21.7	21.7	21.7	61.035	0.0000	48.17	14027000	86	16.66	16.96	17.67	17.15	16.01	15.10	16.43	13.65
+	3.114	1.81	3.51	Q9D023	Mitochondrial pyruvate carrier 2	Mpc2	4850	4	4	4	30.7	30.7	30.7	14.286	0.0000	14.184	7034600	83	15.51	14.64	15.38	14.81	13.71	13.00	12.89	13.49
+	2.046	1.81	3.50	P56542	Deoxyribonuclease-2-alpha	Dnase2	1289	4	4	4	17	17	17	38.81	0.0000	19.833	2391500	25	14.65	14.49	14.81	12.83	12.67	12.05	12.35	12.47
+	1.829	1.81	3.50	Q8C078	Calcium/calmodulin-dependent protein kinase kinase 2	Camkk2	3326	8	8	8	18.4	18.4	18.4	64.617	0.0000	26.601	6020700	63	14.99	14.86	15.46	15.38	14.55	12.05	13.50	13.36
+	1.724	1.81	3.50	Q8BJ64	Choline dehydrogenase, mitochondrial	Chdh	3074	2	2	2	6.2	6.2	6.2	66.414	0.0000	29.567	1848000	25	13.94	13.59	14.46	14.28	11.70	11.93	13.84	11.56
+	1.493	1.80	3.49	Q9ESW8	Pyroglutamyl-peptidase 1	Pgpep1	5349	5	5	5	41.6	41.6	41.6	22.934	0.0000	51.169	6572900	108	15.87	15.75	14.51	15.34	13.87	12.06	14.82	13.50
+	1.479	1.80	3.48	Q99L48	60S ribosomal export protein NMD3	Nmd3	4410	6	6	6	16.3	16.3	16.3	57.611	0.0000	27.11	5895000	67	15.84	15.35	15.81	15.49	12.77	12.65	14.84	15.03
+	1.619	1.80	3.47	Q80UX8	Alpha/beta hydrolase domain-containing protein 13	Abhd13	2841	2	2	2	11	11	11	38.525	0.0002	5.8081	1865200	13	14.20	14.19	15.30	14.50	14.34	12.05	12.51	12.11
+	2.880	1.79	3.46	Q8BH79	Anoctamin-10	Ano10	3021	5	5	5	8.3	8.3	8.3	76.187	0.0000	48.905	7623200	42	15.45	14.66	15.76	15.42	13.41	13.06	14.09	13.57
+	1.818	1.79	3.45	P06802	Ectonucleotide pyrophosphatase/phosphodiesterase family mem	Enpp1	666	4	4	4	7.1	7.1	7.1	103.17	0.0000	50.356	3461700	58	15.16	14.53	14.83	14.57	14.27	12.01	13.33	12.32
+	2.879	1.78	3.44	E9PZJ8	Activating signal cointegrator 1 complex subunit 3	Ascc3	225	23	23	23	12.5	12.5	12.5	250.55	0.0000	63.882	13589000	101	16.37	15.47	16.20	15.87	14.78	13.71	14.40	13.87
+	2.384	1.78	3.44	Q5HZI9	Solute carrier family 25 member 51	Slc25a51	2107	2	2	2	12.1	12.1	12.1	33.689	0.0000	82.491	5032100	50	15.68	14.42	15.15	15.35	13.17	12.61	13.86	13.82
+	1.333	1.76	3.40	Q8VC60	Beta-galactosidase-1-like protein	Glb1l	3910	3	2	2	6.7	4.8	4.8	73.278	0.0000	12.342	2709600	21	15.49	14.47	14.34	14.36	13.42	14.49	12.00	11.70
+	1.922	1.76	3.39	Q5SVR0	TBC1 domain family member 9B	Tbc1d9b	2152	13	13	12	12.7	12.7	12	141.78	0.0000	52.343	8591000	88	14.88	14.58	14.63	14.66	13.51	11.50	13.63	13.06
+	1.799	1.76	3.38	Q3TBW2	39S ribosomal protein L10, mitochondrial	Mrpl10	1821	6	6	6	34	34	34	29.395	0.0000	83.871	5035400	50	14.78	14.57	16.45	15.58	14.04	13.12	14.20	12.99
+	2.072	1.75	3.37	Q9Z2D0	Myotubularin-related protein 9	Mtmr9	5841	15	15	15	32.5	32.5	32.5	62.907	0.0000	123.9	9800800	116	15.22	14.83	15.60	15.61	13.30	13.70	14.62	12.63
+	3.683	1.74	3.35	Q6NWW9	Fibronectin type III domain-containing protein 3B	Fndc3b	2537	8	8	8	10.5	10.5	10.5	132.76	0.0000	38.087	4626500	40	15.27	15.03	15.74	15.11	13.20	13.45	13.92	13.63
+	1.654	1.74	3.34	Q9CY18	Sorting nexin-7	Snx7	4778	5	5	5	20.4	20.4	20.4	45	0.0000	12.623	5166000	42	15.43	15.27	15.47	15.56	13.51	12.54	15.25	13.47
+	2.704	1.74	3.34	Q91WC9	Sn1-specific diacylglycerol lipase beta	Daglb	4104	14	14	14	26.9	26.9	26.9	73.904	0.0000	143.86	43188000	230	17.32	16.85	17.11	17.05	15.62	15.36	15.94	14.45
+	1.865	1.74	3.33	P48774	Glutathione S-transferase Mu 5	Gstm5	1151	10	8	8	39.7	36.6	36.6	26.635	0.0000	21.619	7033300	86	15.50	15.57	14.20	14.61	13.92	12.86	13.79	12.36
+	2.926	1.74	3.33	Q5SRY7;Q	F-box/WD repeat-containing protein 11;F-box/WD repeat-contain	Fbxw11;Btrc	2134	4	4	4	11.6	11.6	11.6	62.062	0.0000	29.429	3058800	22	14.88	14.39	14.81	14.59	12.31	13.46	12.62	13.35
+	1.494	1.73	3.33	Q8BVA5	UPF0554 protein C2orf43 homolog		3236	8	8	8	35	35	35	37.374	0.0000	93.404	44246000	95	17.73	17.52	18.00	18.15	16.08	16.21	17.58	14.60
+	2.238	1.73	3.32	Q8CE96	tRNA (adenine(58)-N(1))-methyltransferase non-catalytic subunit	Trmt6	3469	7	7	7	19.5	19.5	19.5	55.518	0.0000	29.702	4236000	56	14.92	15.04	15.14	15.33	12.72	13.32	14.53	12.93
+	2.843	1.73	3.32	P54731	FAS-associated factor 1	Faf1	1255	4	4	4	8.3	8.3	8.3	73.862	0.0000	18.43	7765800	51	15.99	15.68	16.26	16.35	14.21	14.58	14.92	13.65
+	1.351	1.73	3.31	Q8CE90	Dual specificity mitogen-activated protein kinase kinase 7	Map2k7	3468	6	6	6	17.9	17.9	17.9	59.312	0.0000	21.084	8859500	31	16.74	16.17	16.92	16.59	15.49	13.82	16.46	13.74
+	1.738	1.71	3.26	Q6ZQH8	Nucleoporin NUP188 homolog	Nup188	2696	12	12	12	9.4	9.4	9.4	196.69	0.0000	25.574	6633300	68	15.56	14.78	15.65	14.71	13.31	12.26	13.81	14.49
+	0.759	1.70	3.26	P47941	Crk-like protein	Crkl	1132	7	7	7	36.3	36.3	36.3	33.83	0.0000	16.707	12447000	14	16.65	16.62	13.19	16.33	16.15	13.44	13.14	13.26
+	1.643	1.70	3.25	Q99J45	Nuclear receptor-binding protein	Nrbp1	4324	6	6	6	19.8	19.8	19.8	59.865	0.0000	55.866	12949000	151	16.32	15.74	16.12	15.85	14.42	14.69	15.34	12.79
+	1.379	1.69	3.23	Q8BLR5	PH and SEC7 domain-containing protein 4	Psd4	3122	5	5	5	7.2	7.2	7.2	112.73	0.0000	38.734	3860000	37	14.70	14.21	14.84	14.24	13.89	11.04	13.55	12.74
+	0.920	1.69	3.23	P68369;P0	Tubulin alpha-1A chain;Tubulin alpha-3 chain	Tuba1a;Tuba3a	1517	36	4	1	72.7	10.2	3.1	50.135	0.0000	79.259	72237000	138	18.80	18.97	18.75	18.33	18.00	14.25	18.07	17.76
+	1.215	1.68	3.20	Q8BWY7	Zinc transporter ZIP11	Slc39a11	3271	3	3	3	8.8	8.8	8.8	35.466	0.0000	7.1718	8798100	72	15.62	16.11	16.89	16.42	15.20	13.92	16.14	13.07
+	1.158	1.68	3.20	Q9CR70	EKC/KEOPS complex subunit Lage3	Lage3	4678	2	2	2	20.3	20.3	20.3	15.824	0.0000	165.67	5942800	53	15.73	15.51	16.36	16.02	13.13	15.19	15.77	12.82
+	2.895	1.67	3.19	Q9CXD6	Mitochondrial calcium uniporter regulator 1	Mcur1	4752	8	8	8	24.7	24.7	24.7	37.849	0.0000	16.142	8199000	60	15.29	14.67	15.67	15.66	13.68	13.37	14.14	13.40
+	2.151	1.67	3.19	Q8JZS9	39S ribosomal protein L48, mitochondrial	Mrpl48	3581	3	3	3	10.9	10.9	10.9	23.952	0.0009	3.7682	2548400	23	14.57	14.24	14.25	14.41	13.83	12.30	12.73	11.93
+	2.376	1.67	3.18	Q8R127	Saccharopine dehydrogenase-like oxidoreductase	Sccpdh	3774	5	5	5	19.1	19.1	19.1	47.129	0.0000	85.82	4243600	61	15.59	14.94	14.46	15.41	13.16	13.09	14.25	13.22
+	1.304	1.66	3.17	Q6PIP5	NudC domain-containing protein 1	Nudcd1	2646	3	3	3	10	10	10	66.704	0.0000	15.13	3141100	29	15.36	14.76	13.48	13.64	12.83	13.34	13.25	11.16
+	2.762	1.66	3.17	Q78IS1	Transmembrane emp24 domain-containing protein 3	Tmed3	2724	6	6	6	23.1	23.1	23.1	25.465	0.0000	48.412	11330000	96	16.74	16.12	17.25	17.13	15.35	14.76	15.53	14.93
+	3.734	1.66	3.16	P07742	Ribonucleoside-diphosphate reductase large subunit	Rrm1	672	19	19	19	36.4	36.4	36.4	90.209	0.0000	218.19	13964000	127	16.14	15.31	15.77	15.84	14.33	14.20	14.08	13.80
+	2.755	1.65	3.15	Q64729	TGF-beta receptor type-1	Tgfbr1	2462	1	1	1	3	3	3	56.178	0.0004	4.9048	1657000	18	14.16	13.93	14.40	14.54	12.84	11.94	12.41	13.23
+	2.766	1.65	3.14	Q3UV70	[Pyruvate dehydrogenase [acetyl-transferring]]-phosphatase 1, m	Pdp1	2036	11	11	11	33.8	33.8	33.8	61.18	0.0000	149.6	10495000	111	14.76	15.16	15.26	15.26	12.81	13.88	13.15	13.98
+	2.119	1.65	3.14	Q9R0A0	Peroxisomal membrane protein PEX14	Pex14	5619	8	8	7	35.4	35.4	26.6	41.207	0.0000	323.31	15284000	160	15.70	15.69	16.14	16.47	14.03	13.44	14.97	14.96
+	1.543	1.65	3.14	Q8CFE6	Sodium-coupled neutral amino acid transporter 2	Slc38a2	3485	4	4	4	12.5	12.5	12.5	55.503	0.0000	167.34	3159100	65	13.96	14.33	15.08	14.64	14.03	11.65	13.34	12.39
+	3.169	1.64	3.12	Q8K2C8;Q	Glycerol-3-phosphate acyltransferase 4	Agpat6	3655	7	7	7	16.4	16.4	16.4	52.181	0.0000	24.818	5592800	82	14.75	14.17	14.69	14.82	12.86	12.42	13.24	13.34
+	2.818	1.64	3.12	Q8CC88	von Willebrand factor A domain-containing protein 8	Vwa8	3444	35	35	35	27.6	27.6	27.6	213.42	0.0000	127.9	39382000	233	18.54	17.78	18.36	18.40	16.65	16.48	17.28	16.10
+	3.038	1.63	3.10	Q9R0P4	Small acidic protein	Smap	5632	2	2	2	25.4	25.4	25.4	20.046	0.0000	41.814	2216400	26	14.68	15.40	14.50	14.71	13.15	13.18	13.66	12.76
+	1.541	1.63	3.10	Q3UHB1	5-nucleotidase domain-containing protein 3	Nt5dc3	1969	7	7	7	19.8	19.8	19.8	63.17	0.0000	15.558	5328800	48	15.81	14.97	15.32	15.45	14.29	12.19	14.63	13.92
+	1.594	1.63	3.09	P82198	Transforming growth factor-beta-induced protein ig-h3	Tgfbi	1598	4	4	4	8.2	8.2	8.2	74.596	0.0000	7.3803	1841700	15	14.16	14.22	14.14	14.67	13.53	12.09	11.45	13.61
+	2.717	1.63	3.09	Q99J27	Acetyl-coenzyme A transporter 1	Slc33a1	4320	8	8	8	16.7	16.7	16.7	61.075	0.0000	17.2	5840100	42	14.71	14.03	15.25	14.49	12.70	12.69	13.40	13.18
+	2.993	1.62	3.08	Q920A5	Retinoid-inducible serine carboxypeptidase	Scpep1	4206	20	20	20	30.5	30.5	30.5	50.964	0.0000	217.27	177470000	509	18.41	19.02	18.77	19.04	16.70	16.90	17.47	17.67
+	0.895	1.62	3.08	Q9JLJ5	Elongation of very long chain fatty acids protein 1	Elovl1	5484	4	4	3	10.8	10.8	10.8	32.677	0.0000	22.876	21419000	82	17.13	16.24	18.31	18.33	16.77	16.56	16.60	13.59
+	1.198	1.62	3.07	Q8BGG7	Ubiquitin-associated and SH3 domain-containing protein B	Ubash3b	2982	8	8	8	16.6	16.6	16.6	71.443	0.0000	21.326	6982000	48	15.60	14.82	15.31	15.41	14.83	12.03	14.80	12.99
+	2.583	1.62	3.07	Q8BWM0	Prostaglandin E synthase 2;Prostaglandin E synthase 2 truncate	Ptges2	3262	8	8	8	34.6	34.6	34.6	43.323	0.0000	145.95	12733000	137	15.97	15.86	16.13	15.95	14.17	14.47	15.17	13.62
+	2.524	1.62	3.07	Q8C0L0	Thioredoxin-related transmembrane protein 4	Tmx4	3341	6	6	6	24.8	24.8	24.8	37.131	0.0000	67.716	5416800	64	15.55	14.68	15.22	15.22	14.19	13.77	13.39	12.85
	0.574	1.61	3.06	Q80VJ3	2-deoxynucleoside 5-phosphate N-hydrolase 1	Dnph1	2852	9	9	9	71.7	71.7	71.7	18.977	0.0000	124.93	20392000	146	16.08	17.51	16.79	17.01	12.16	14.07	17.27	17.43
+	1.634	1.61	3.06	Q8BG94	COMM domain-containing protein 7	Commd7	2968	2	2	2	16	16	16	22.652	0.0000	24.097	1918100	23	15.12	15.11	13.64	15.17	13.43	14.01	12.22	12.93
+	4.173	1.61	3.05	O35623	BET1 homolog	Bet1	388	3	3	3	28	28	28	13.274	0.0000	24.85	3590400	64	14.43	14.10	14.43	14.48	12.57	12.63	13.17	12.65

+	2.163	1.61	3.04	Q9CYZ6	Uncharacterized protein C19orf60 homolog		4811	3	3	3	28.4	28.4	28.4	18.706	0.0000	57.59	3387900	69	14.92	14.97	13.59	15.02	13.05	13.13	12.48	13.42
+	1.780	1.60	3.04	P54728	UV excision repair protein RAD23 homolog B	Rad23b	1253	7	7	6	26.2	26.2	26.2	43.512	0.0000	19.149	4802000	40	14.90	15.53	13.97	13.99	13.28	13.60	12.93	12.18
+	4.428	1.60	3.03	P55937	Golgin subfamily A member 3	Golga3	1274	81	81	81	57.5	57.5	57.5	167.22	0.0000	323.31	90766000	762	15.75	15.34	15.55	15.54	13.65	13.95	14.25	13.93
+	1.310	1.60	3.03	P54751	CMP-N-acetylneuraminate-beta-galactosamide-alpha-2,3-sialyltr	St3gal1	1256	3	3	3	14.2	14.2	14.2	39.072	0.0034	2.5371	1903700	15	15.29	14.49	14.48	15.45	13.23	13.00	12.11	14.97
+	2.334	1.60	3.03	Q3UUQ7	GPI inositol-deacylase	Pgap1	2033	18	18	18	26	26	26	104.58	0.0000	128.45	31020000	269	16.35	15.54	15.49	15.16	13.67	13.50	14.50	14.46
+	1.094	1.60	3.02	Q9D1C8	Vacuolar protein sorting-associated protein 28 homolog	Vps28	4905	10	10	10	62	62	62	25.452	0.0000	69.961	16125000	136	15.92	15.95	15.92	15.78	15.42	12.42	15.63	13.72
+	1.034	1.59	3.02	P03893	NADH-ubiquinone oxidoreductase chain 2	Mtnd2	629	1	1	1	7.5	7.5	7.5	38.752	0.0000	63.975	3230500	62	15.34	14.22	13.60	12.46	11.16	12.89	11.74	13.44
+	1.857	1.59	3.02	Q9EST4	Proteasome assembly chaperone 2	Psmg2	5344	5	5	5	18.9	18.9	18.9	29.524	0.0002	5.8302	4613100	44	14.70	14.79	16.03	15.38	13.84	14.48	12.87	13.33
+	0.827	1.59	3.01	Q8QZS3	Folliculin	Flcn	3734	7	7	7	17.4	17.4	17.4	64.327	0.0000	19.394	9943500	36	16.26	15.75	16.42	16.21	15.89	13.69	16.37	12.32
+	1.294	1.59	3.00	Q9CZL2	Uncharacterized protein C4orf32 homolog		4831	3	3	3	38.2	38.2	38.2	14.51	0.0000	44.166	2342700	25	15.28	13.64	13.05	14.19	11.13	12.74	13.07	12.87
+	1.035	1.59	3.00	Q3UUJ4	STE20-related kinase adapter protein alpha	Strada	2032	3	3	1	7	7	4.6	48.235	0.0000	7.1056	4849900	35	14.97	15.01	15.91	16.42	14.70	14.53	14.86	11.87
+	1.676	1.58	2.99	O88851	Putative hydrolase RBBP9	Rbbp9	577	7	7	6	63.4	63.4	60.8	20.911	0.0000	26.017	3492800	32	15.38	14.83	13.96	14.33	12.55	12.86	12.51	14.24
+	1.555	1.58	2.99	Q9CQ49	Nuclear cap-binding protein subunit 2	Ncbp2	4561	4	4	4	26.9	26.9	26.9	18.017	0.0000	8.4879	4440600	28	15.71	15.45	15.71	14.71	14.23	14.57	14.09	12.36
+	1.839	1.58	2.99	Q61083	Mitogen-activated protein kinase kinase kinase 2	Map3k2	2247	6	3	3	12.8	6.5	6.5	69.573	0.0000	53.481	3256800	51	14.65	14.76	14.98	14.74	12.10	14.11	13.77	12.82
+	2.263	1.58	2.99	Q8R5L3	Vam6/Vps39-like protein	Vps39	3892	25	25	25	31	31	31	101.69	0.0000	90.719	26539000	196	16.51	15.86	16.00	16.36	14.95	14.56	15.24	13.67
+	1.942	1.57	2.98	Q9D7V9	N-acylethanolamine-hydrolyzing acid amidase;N-acylethanolamine-hydr	Naaa	5041	4	4	4	13.8	13.8	13.8	40.074	0.0000	28.534	9614400	88	16.02	15.96	15.85	15.92	15.29	13.86	14.88	13.42
+	2.352	1.57	2.98	Q7TPM9	Myotubularin-related protein 10	Mtmr10	2769	11	11	11	17.5	17.5	17.5	88.196	0.0000	53.302	6564000	49	15.96	15.31	16.12	15.59	14.39	14.81	14.13	13.36
+	1.362	1.57	2.98	Q8BGW1	Alpha-ketoglutarate-dependent dioxygenase FTO	Fto	2998	6	6	6	15.7	15.7	15.7	58.006	0.0000	14.802	9123300	49	16.14	16.36	17.27	17.03	15.35	15.22	16.30	13.62
+	1.403	1.57	2.97	Q99KW3	TRIO and F-actin-binding protein	Triobp	4397	6	5	5	4.4	3.9	3.9	223.37	0.0000	35.215	2543000	32	13.81	13.57	13.37	14.39	13.48	10.90	12.69	11.78
+	3.494	1.57	2.97	P54987	Cis-aconitate decarboxylase	Irg1	1263	26	26	26	55.7	55.7	55.7	53.758	0.0000	323.31	148970000	728	18.31	17.80	17.86	17.71	16.82	16.10	16.35	16.13
	0.653	1.57	2.97	Q5QR91	Uncharacterized protein C17orf78 homolog	Gm11437	2125	2	2	2	8.3	8.3	8.3	32.583	0.0024	2.9771	75602000	33	20.01	18.16	18.41	18.93	17.97	14.13	18.27	18.87
+	1.812	1.57	2.97	Q62077	1-phosphatidylinositol 4,5-bisphosphate phosphodiesterase gamma-1	Plcg1	2351	4	4	4	2.6	2.6	2.6	149.67	0.0004	4.7877	2390100	8	14.38	14.15	14.92	14.44	13.25	12.82	11.74	13.81
+	0.960	1.57	2.97	P60762	Mortality factor 4-like protein 1	Morf4l1	1359	6	6	6	24.9	24.9	24.9	41.492	0.0000	10.871	4844900	27	15.64	14.84	16.23	14.76	12.57	13.32	16.01	13.30
+	1.047	1.57	2.96	Q9QXM0	Abhydrolase domain-containing protein 2	Abhd2	5546	4	4	4	14.8	14.8	14.8	48.377	0.0000	49.863	4484500	52	14.90	14.26	15.41	14.78	14.15	14.83	12.50	11.61
+	2.489	1.56	2.95	Q60823	RAC-beta serine/threonine-protein kinase	Akt2	2211	10	8	8	31	24.5	24.5	55.741	0.0000	46.55	6091600	65	15.35	14.85	15.36	15.25	13.11	14.26	14.08	13.12
+	1.692	1.56	2.94	Q8BH24	Transmembrane 9 superfamily member 4	Tm9sf4	3008	13	13	13	17.4	17.4	17.4	74.692	0.0000	79.273	45290000	297	17.80	17.00	17.67	17.94	16.54	16.17	16.74	14.74
+	1.733	1.55	2.93	Q91ZR2	Sorting nexin-18	Snx18	4199	8	8	8	16.6	16.6	16.6	67.903	0.0000	47.139	7333100	47	15.82	14.64	16.13	16.00	14.19	13.11	14.64	14.45
+	1.895	1.55	2.93	Q8R1U1	Conserved oligomeric Golgi complex subunit 4	Cog4	3801	11	11	11	18.1	18.1	18.1	88.66	0.0000	34.575	5795700	104	15.07	14.78	14.89	14.97	13.90	12.54	14.34	12.74
+	2.768	1.55	2.92	Q9CWP6	Motile sperm domain-containing protein 2	Mospd2	4723	16	16	15	28	28	26.4	59.854	0.0000	76.776	16060000	134	16.51	16.06	15.69	16.25	14.95	14.29	15.00	14.09
+	2.916	1.54	2.91	P50096	Inosine-5-monophosphate dehydrogenase 1	Impdh1	1174	15	13	13	36.6	34.8	34.8	55.278	0.0000	84.897	11945000	130	15.62	15.13	15.69	15.41	13.48	13.55	14.42	14.24
+	2.037	1.54	2.91	Q8BFY6	Peflin	Pef1	2952	8	8	8	38.2	38.2	38.2	29.227	0.0000	194.48	19386000	198	16.52	16.29	16.15	16.39	15.43	14.59	15.42	13.75
+	4.621	1.54	2.91	Q9R1J0	Sterol-4-alpha-carboxylate 3-dehydrogenase, decarboxylating	Nsdhl	5651	18	18	18	57.5	57.5	57.5	40.685	0.0000	204.4	54278000	391	17.04	16.58	16.67	16.68	15.42	15.25	15.06	15.07
+	1.899	1.54	2.91	Q3UHH1	Zinc finger SWIM domain-containing protein 8	Zswim8	1972	7	7	7	5.5	5.5	5.5	197.06	0.0000	14.042	3617700	29	14.75	14.27	14.01	13.84	13.58	11.74	12.95	12.44
+	1.294	1.54	2.91	B2RX12	Canalicular multispecific organic anion transporter 2	Abcc3	82	17	15	15	18.1	16.7	16.7	169.22	0.0000	141.8	21954000	174	16.91	16.32	17.22	17.02	16.29	13.57	15.79	15.67
+	2.073	1.54	2.91	E2JF22	Piezo-type mechanosensitive ion channel component 1	Piezo1	218	6	6	6	3.8	3.8	3.8	292	0.0000	23.026	4982100	52	15.72	14.98	15.71	15.62	13.82	14.65	14.38	13.02
+	3.469	1.54	2.91	Q9EPW0	Type I inositol 3,4-bisphosphate 4-phosphatase	Inpp4a	5267	8	8	8	12.5	12.5	12.5	105.54	0.0000	32.707	8004700	73	15.59	15.00	15.81	15.28	14.20	13.76	13.88	13.69
+	2.407	1.53	2.89	P52479	Ubiquitin carboxyl-terminal hydrolase 10	Usp10	1218	6	6	6	12.4	12.4	12.4	87.021	0.0000	37.525	2813000	36	14.63	13.94	14.80	14.61	13.09	12.17	13.49	13.11
	0.533	1.53	2.89	Q91WD1	DNA-directed RNA polymerase III subunit RPC4	Polr3d	4105	2	2	2	6	6	6	44.356	0.0002	5.6087	8804900	24	16.45	16.96	13.38	17.01	12.86	14.71	17.16	12.95
+	0.929	1.53	2.89	Q69ZR2	E3 ubiquitin-protein ligase HECTD1	Hectd1	2490	18	18	18	9.9	9.9	9.9	290.08	0.0000	41.257	25661000	78	18.32	17.25	18.39	17.88	16.02	14.48	16.99	18.24
+	1.612	1.53	2.88	Q91YX5	Acyl-CoA:lysophosphatidylglycerol acyltransferase 1	Lpgat1	4184	5	5	5	19.5	19.5	19.5	43.088	0.0000	26.035	4017700	53	15.14	14.80	14.32	14.61	13.92	11.78	13.43	13.63
+	1.397	1.52	2.88	P81117	Nucleobindin-2;Nesfatin-1	Nucb2	1597	11	11	11	32.1	32.1	32.1	50.304	0.0000	68.232	4826800	85	14.21	14.40	13.88	14.09	12.33	12.05	11.79	14.31
+	1.728	1.52	2.87	Q80YQ8;Q9	Protein RMD5 homolog A	Rmnd5a	2904	6	6	6	21.7	21.7	21.7	43.992	0.0000	15.66	5253100	89	14.71	14.48	14.24	14.54	14.02	12.06	13.48	12.33
+	2.098	1.52	2.86	Q8BGR2	Volume-regulated anion channel subunit LRRC8D	Lrrc8d	2989	9	9	9	17.7	17.7	17.7	98.112	0.0000	113.19	5464500	89	14.94	14.25	15.00	15.43	13.43	13.33	14.13	12.65
+	3.121	1.51	2.86	Q9CR39	WD repeat domain phosphoinositide-interacting protein 3	Wdr45b	4664	4	4	4	10.8	10.8	10.8	38.02	0.0000	11.466	6573500	70	15.59	15.12	15.54	15.56	13.59	13.57	14.43	14.16
+	1.271	1.51	2.85	Q99PN3	Tripartite motif-containing protein 26	Trim26	4510	8	8	8	22.9	22.9	22.9	62.808	0.0000	36.109	2525100	21	15.50	14.62	13.45	14.86	13.26	13.26	14.04	11.82
+	1.961	1.51	2.85	P58854	Gamma-tubulin complex component 3	Tubgcp3	1325	9	9	9	14.4	14.4	14.4	103.47	0.0000	23.877	7261300	77	16.08	15.38	16.01	15.74	14.59	14.74	14.70	13.14
	0.344	1.51	2.85	O88665	Bromodomain-containing protein 7	Brd7	555	3	3	3	7.4	7.4	7.4	73.999	0.0000	9.1874	23905000	13	16.95	18.97	12.91	17.13	16.94	13.29	17.68	12.01
+	2.202	1.51	2.84	Q91VX2	Ubiquitin-associated protein 2	Ubap2	4083	10	10	10	14.6	14.6	14.6	117.96	0.0000	42.435	10849000	77	15.27	15.29	15.81	15.60	14.49	14.31	14.16	12.98
+	2.652	1.50	2.84	Q9CXI0	2-methoxy-6-polyprenyl-1,4-benzoquinol methylase, mitochondrial	Coq5	4756	7	7	7	28.7	28.7	28.7	37.335	0.0000	28.001	6556200	46	15.11	14.64	15.30	15.31	13.64	13.40	13.05	14.25
	0.546	1.50	2.83	P39688	Tyrosine-protein kinase Fyn	Fyn	1054	7	6	3	15.3	15.3	8.4	60.674	0.0000	35.352	18855000	96	17.47	16.99	17.54	17.20	11.98	17.11	16.92	17.18
+	0.823	1.50	2.83	Q60974	Nuclear receptor corepressor 1	Ncor1	2233	7	7	6	5.6	5.6	4.6	270.64	0.0000	17.184	21390000	29	16.91	17.41	17.97	17.77	17.67	17.39	14.60	14.39
+	1.517	1.50	2.83	E9Q236		Abcc4	229	11	10	10	12	11.5	11.5	148.89	0.0000	22.11	10859000	57	15.46	15.07	16.70	16.16	13.96	15.16	14.82	13.46
+	1.731	1.50	2.82	P06801	NADP-dependent malic enzyme	Me1	665	11	11	11	36.4	36.4	36.4	63.953	0.0000	138.55	8946300	72	15.97	15.50	16.02	16.23	15.40	14.85	14.10	13.38
+	2.876	1.50	2.82	Q921Q3	Chitobiosyldiphosphodolichol beta-mannosyltransferase	Alg1	4237	5	5	5	12	12	12	54.417	0.0000	28.675	5160500	52	15.04	14.19	15.13	15.10	13.45	12.97	13.60	13.46
+	2.056	1.50	2.82	P68181	cAMP-dependent protein kinase catalytic subunit beta	Prkacb	1514	23	11	11	61	33.9	33.9	40.707	0.0000	95.51	20987000	141	17.13	16.55	16.81	16.74	15.60	15.16	16.13	14.36
+	2.778	1.49	2.82	Q3UDE2	Tubulin--tyrosine ligase-like protein 12	Ttll12	1946	18	18	18	41	41	41	74.042	0.0000	185.36	36517000	291	17.45	16.82	17.38	17.27	15.95	15.91	16.06	15.03
+	3.791	1.49	2.82	Q3UR70	Transforming growth factor-beta receptor-associated protein 1	Tgfbrap1	2020	6	6	6	10.7	10.7	10.7	97.302	0.0000	21.769	2668800	53	14.50	14.20	14.45	14.68	12.70	13.09	12.74	13.32
	0.564	1.49	2.81	E9Q8I9	Protein furry homolog	Fry	249	4	4	4	2.3	2.3	2.3	339.09	0.0002	5.5566	23790000	42	17.55	16.85	17.56	18.06	17.18	17.15	12.39	17.34
+	1.195	1.49	2.81	Q8VHI3	GDP-fucose protein O-fucosyltransferase 2	Pofut2	4020	4	4	4	10.3	10.3	10.3	49.429	0.0000	13.402	2074100	15	14.90	13.84	12.63	14.72	13.24	11.40	12.54	12.94
+	0.960	1.49	2.81	Q6NT99	Dual specificity protein phosphatase 23	Dusp23	2528	5	5	5	44.7	44.7	44.7	16.637	0.0000	6.8456	3626900	27	13.88	14.81	15.16	15.20	14.33	12.57	14.64	11.55
+	1.956	1.49	2.81	Q8C5H8	NAD kinase 2, mitochondrial	Nadk2	3395	6	6	6	18.4	18.4	18.4	50.858	0.0000	34.163	3999200	38	14.08	13.49	15.19	14.94	12.83	12.66	13.21	13.05
+	1.049	1.49	2.80	O88653	Ragulator complex protein LAMTOR3	Lamtor3	554	5	5	5	58.1	58.1	58.1	13.553	0.0000	30.438	7984100	66	15.70	15.71	14.07	15.51	12.85	14.32	15.24	12.64
+	2.131	1.49	2.80	P58681	Toll-like receptor 7	Tlr7	1319	26	26	26	28.6	28.6	28.6	121.84	0.0000	251.13	73394000	524	17.56	17.12	17.51	17.38	16.54	15.82	16.34	14.92
+	2.520	1.48	2.80	Q9WVS7	Dual specificity mitogen-activated protein kinase kinase 5	Map2k5	5761	3	3	3	9.4	9.4	9.4	50.104	0.0000	11.037	3704400	47	14.17	14.49	14.76	15.08	13.28	12.46	13.61	13.22
+	0.972	1.48	2.79	Q9R0Q3	Transmembrane emp24 domain-containing protein 2	Tmed2	5636	6	6	6	27.4	27.4	27.4	22.705	0.0000	15.976	9532400	58	16.50	16.13	15.13	15.93	15.64	12.33	14.91	14.90
+	1.643	1.48	2.79	Q91VU0	Protein FAM3C	Fam3c	4078	5	5	5	33.5	33.5	33.5	24.752	0.0000	10.659	4930500	21	15.20	15.33	15.05	15.48	13.25	13.23	15.21	13.46
+	2.691	1.48	2.79	Q00651	Integrin alpha-4	Itga4	1700	10	10	10	13.7	13.7	13.7	115.69	0.0000	82.171	55660000	145	19.21	19.15	18.94	18.82	18.26	17.16	17.68	17.10
+	0.864	1.47	2.78	Q9DAU1	Protein canopy homolog 3	Cnpy3	5112	5	5	4	23.6	23.6	14.9	30.537	0.0000	35.578	10172000	23	14.00	17.08	15.36	15.27	15.37	13.18	14.40	12.85
+	1.539	1.47	2.77	Q62245	Son of sevenless homolog 1	Sos1	2372	7	7	5	8.4	8.4	6.1	150.88	0.0000	15.858	5518500	19	16.42	14.89	16.03	15.02	13.20	14.10	14.25	14.92
+	1.054	1.47	2.77	P35737	Class II histocompatibility antigen, M beta 1 chain	H2-DMb1	1030	2	2	2	9.2	9.2	9.2	28.899	0.0000	6.9792	4474900	30	15.12	14.93	15.42	15.26	14.80	12.68	15.09	12.30
+	1.349	1.47	2.76	Q6Y5D8	Rho GTPase-activating protein 10	Arhgap10	2670	17	17	17	19.8	19.8	19.8	89.365	0.0000	33.322	8323000	83	16.07	15.55	15.69	14.96	14.73	13.34	15.26	13.07
+	0.986	1.46	2.76	Q8BGS7	Choline/ethanolaminephosphotransferase 1	Cept1	2992	5	5	5	13.9	13.9	13.9	46.434	0.0000	22.71	15786000	64	17.91	16.88	18.13	17.70	17.01	16.81	16.87	14.06
+	1.560	1.46	2.76	O55091	Protein IMPACT	Impact	449	5	5	5	19.8	19.8	19.8	36.276	0.0000	22.39	3519600	48	14.21	14.26	14.82	14.44	11.64	12.85	13.71	13.68
+	1.500	1.46	2.75	Q6PD19	UPF0668 protein C10orf76 homolog		2608	4	4	4	9.9	9.9	9.9	78.631	0.0002	6.0571	3676800	20	15.35	14.62	14.89	15.12	12.29	13.32	14.68	13.84
+	1.168	1.46	2.75	Q9CPS7	RNA-binding protein PNO1	Pno1	4531	2	2	2	12.9	12.9	12.9	27.453	0.0000	13.962	5849000	57	15.47	15.21	16.04	15.76	13.36	13.66	16.05	13.57
+	0.845	1.46	2.75	Q3U1T9	DENN domain-containing protein 1B	Dennd1b	1904	4	4	4	7.7	7.7	7.7	85.525	0.0000	6.8175	8163400	26	16.46	15.22	16.70	17.08	16.17	12.86	15.99	14.60
+	0.868	1.45	2.73	Q8BHK6	SLAM family member 7	Slamf7	3042	2	2	2	9	9	9	37.187	0.0011	3.6106	3595100	13	14.97	12.05	15.21	15.05	12.74	13.58	13.25	11.91
+	1.904	1.45	2.73	Q9DC63	F-box only protein 3	Fbxo3	5198	5	5	5	14.2	14.2	14.2	55.227	0.0000	10.198	4303400	25	14.37	14.36	15.07	14.31	12.76	12.20	13.53	13.82
+	3.845	1.44	2.72	Q8CFE3	REST corepressor 1	Rcor1	3482	6	6	6	16.9	16.9	16.9	52.714	0.0000	41.438	5661200	59	15.56	14.89	15.03	15.44	13.92	13.84	13.69	13.71
+	1.324	1.44	2.72	P24638	Lysosomal acid phosphatase	Acp2	901	5	5	5	15.4	15.4	15.4	48.508	0.0000	35.071	10716000	85	15.84	15.95	16.43	16.10	15.60	14.68	15.25	13.04
+	2.336	1.44	2.71	P42337	Phosphatidylinositol 4,5-bisphosphate 3-kinase catalytic subunit alpha is	Pik3ca	1079	5	5	5	6.9	6.9	6.9	124.41	0.0000	8.9222	4675800	23	14.70	14.27	15.02	15.04	13.17	13.53	13.93	12.64
+	1.474	1.44	2.71	Q9R0E2	Procollagen-lysine,2-oxoglutarate 5-dioxygenase 1	Plod1	5624	12	12	12	23.2	23.2	23.2	83.594	0.0000	57.426	13484000	102	15.83	15.39	15.85	15.90	15.02	12.81	14.49	14.90
+	2.593	1.44	2.71	P59326	YTH domain-containing family protein 1	Ythdf1	1335	10	6	4	21.3	15.2	12.7	60.878	0.0000	47.643	3988700	65	14.88	14.63	14.07	14.54	13.68	13.27	12.76	12.66
+	2.154	1.44	2.71	Q9CXU1	Mediator of RNA polymerase II transcription subunit 31	Med31	4767	2	2	2	25.2	25.2	25.2	15.771	0.0000	33.912	1834000	54	14.54	14.15	14.60	14.81	13.09	12.30	13.04	13.92
+	2.824	1.44	2.71	Q8K0C4	Lanosterol 14-alpha demethylase	Cyp51a1	3602	6	6	6	16.7	16.7	16.7	56.775	0.0000	19.219	5943200	59	14.44	14.48	15.23	15.11	13.46	12.97	13.34	13.74
+	1.565	1.44	2.70	Q9CPT4	Myeloid-derived growth factor	Mydgf	4533	3	3	3	17.5	17.5	17.5	17.982	0.0000	12.312	4551200	18	15.64	13.70	14.89	14.56	12.60	13.06	13.95	13.44
+	1.010	1.43	2.70	Q3TFD2	Lysophosphatidylcholine acyltransferase 1	Lpcat1	1837	5	5	5	19.1	19.1	19.1	59.743	0.0000	44.356	6072800	62	15.55	14.86	15.58	15.67	14.73	14.51	14.83	11.88
+	1.857	1.43	2.70	Q80XC6	Protein NRDE2 homolog	Nrde2	2880	3	3	3	4.6	4.6	4.6	133.47	0.0000	17.435	2633800	37	14.80	14.69	15.05	14.97	13.05	13.08	14.67	12.98
+	1.203	1.43	2.69	Q9DB43	Zinc finger protein-like 1	Zfpl1	5127	5	5	5	31.6	31.6	31.6	34.155	0.0000	46.507	4122100	40	15.09	13.89	15.02	15.05	14.42	13.15	13.90	11.87
+	1.703	1.43	2.69	Q8K339	DNA/RNA-binding protein KIN17	Kin	3688	2	2	2	6.6	6.6	6.6	44.721	0.0015	3.3936	3814100	11	14.54	14.57	14.36	13.57	13.78	13.15	12.15	12.25
+	1.512	1.42	2.68	Q9JL15	Galectin-8	Lgals8	5466	7	7	7	38	38	38	36.161	0.0000	70.616	4914400	44	14.93	14.39	14.71	14.74	13.68	12.04	14.36	12.99
+	3.981	1.42	2.68	Q921W4	Quinone oxidoreductase-like protein 1	Cryzl1	4243	13	13	13	46.8	46.8	46.8	38.725	0.0000	69.12	16365000	126	16.15	16.17	16.31	16.04	14.93	14.73	14.98	14.33
+	2.120	1.42	2.68	Q66GT5	Phosphatidylglycerophosphatase and protein-tyrosine phosphatase 1	Ptpmt1	2467	5	5	5	29	29	29	21.942	0.0000	12.405	3790300	38	15.11	14.60	14.25	14.42	13.84	13.52	12.80	12.52
+	2.219	1.42	2.68	Q8R5K4	Nucleolar protein 6	Nol6	3891	7	7	7	8.2	8.2	8.2	129.23	0.0000	29.002	3484000	41	14.69	13.55	14.72	14.16	12.65	12.63	12.69	13.47
+	2.481	1.42	2.68	Q99JV5	StAR-related lipid transfer protein 4	Stard4	4350	4	4	4	26.8	26.8	26.8	25.579	0.0000	42.217	1570300	21	14.18	13.36	14.02	14.63	12.78	12.94	12.51	12.28
+	2.114	1.42	2.67	Q5SQX6	Cytoplasmic FMR1-interacting protein 2	Cyfip2	2132	41	18	18	35.9	17.4	17.4	145.66	0.0000	75.842	19104000	114	16.90	16.22	17.14	17.01	15.62	15.07	16.12	14.79
+	3.380	1.41	2.66	Q9D4H8	Cullin-2	Cul2	4978	28	28	28	44	44	44	86.876	0.0000	181.77	42919000	456	16.58	16.07	16.05	16.23	14.78	14.62	15.28	14.60
+	1.211	1.41	2.66	Q8C4J7	Transducin beta-like protein 3	Tbl3	3386	8	8	7	16.2	16.2	15.1	88.265	0.0000	58.013	7397000	70	15.51	15.03	15.78	15.56	14.11	14.95	14.82	12.36
+	3.897	1.41	2.66	Q8R0A7	Uncharacterized protein KIAA0513	Kiaa0513	3754	6	6	6	22.9	22.9	22.9	46.318	0.0000	16.379	3746200	61	14.65	14.33	14.53	14.88	13.19	13.28	13.41	12.87
	0.504	1.41	2.66	Q8VE95	UPF0598 protein C8orf82 homolog		3993	2	2	2	13.8	13.8	13.8	24.33	0.0000	13.696	4500900	13	16.54	15.87	16.25	12.36	15.39	15.09	11.94	12.97
+	1.683	1.41	2.66	G3X9K3	Brefeldin A-inhibited guanine nucleotide-exchange protein 1	Arfgef1	261	21	15	15	14.1	10.4	10.4	208.5	0.0000	55.948	14502000	96	16.36	15.62	16.54	16.77	15.39	14.59	15.67	14.01
+	1.734	1.41	2.66	Q3UHC7	Disabled homolog 2-interacting protein	Dab2ip	1970	3	3	3	3.6	3.6	3.6	131.72	0.0066	2.0751	3872600	27	14.46	13.89	15.43	15.31	12.86	13.29	14.03	13.28
+	2.159	1.40	2.64	P15626;Q8F	Glutathione S-transferase Mu 2	Gstm2	800	17	10	7	69.3	51.8	38.5	25.716	0.0000	22.47	10188000	111	15.62	15.10	15.95	15.87	13.90	13.67	14.99	14.38
+	1.314	1.40	2.64	Q8R1K1	Ubiquitin-associated domain-containing protein 2	Ubac2	3791	5	5	5	18.6	18.6	18.6	39.056	0.0000	26.99	7535200	39	16.17	14.40	16.19	15.54	14.34	13.22	15.07	14.06
+	2.383	1.40	2.64	Q0VGB7	Serine/threonine-protein phosphatase 4 regulatory subunit 2	Ppp4r2	1780	6	6	6	22.3	22.3	22.3	46.478	0.0000	32.65	7211500	65	15.13	14.79	15.89	15.43	13.92	14.02	14.35	13.35
+	1.441	1.40	2.64	Q8BHK9	DNA excision repair protein ERCC-6-like	Ercc6l	3043	6	6	6	7.2	7.2	7.2	138.85	0.0000	58.601	4973200	42	15.02	14.97	16.01	15.25	13.02	14.81	14.60	13.23
+	2.534	1.40	2.63	Q8CEC6	Peptidylprolyl isomerase domain and WD repeat-containing protein 1	Ppwd1	3471	13	13	13	25.1	25.1	25.1	73.384	0.0000	29.539	7105000	70	15.40	14.46	14.33	14.38	13.35	12.95	13.59	13.09
+	3.590	1.39	2.63	Q99LI7	Cleavage stimulation factor subunit 3	Cstf3	4433	19	19	19	35.6	35.6	35.6	82.876	0.0000	125.21	68020000	236	19.64	19.14	19.43	19.42	18.03	17.70	18.41	17.90
+	1.535	1.39	2.62	Q80U63	Mitofusin-2	Mfn2	2821	8	8	8	13.3	13.3	13.3	86.187	0.0000	9.8838	3449200	32	13.55	13.20	14.49	14.31	13.30	11.82	12.98	11.89
+	2.837	1.39	2.62	Q91ZW2	GDP-fucose protein O-fucosyltransferase 1	Pofut1	4203	8	8	8	30	30	30	44.688	0.0000	110.69	11175000	85	15.56	15.60	15.85	15.46	14.66	14.00	14.58	13.67
	0.716	1.39	2.62	Q8K0B2	Probable lysosomal cobalamin transporter	Lmbrd1	3600	2	2	2	4.5	4.5	4.5	61.062	0.0000	104.27	11268000	84	16.08	15.94	16.35	16.26	15.66	15.25	16.16	12.00
+	2.674	1.39	2.62	Q9Z210	Peroxisomal membrane protein 11B	Pex11b	5829	5	5	5	23.2	23.2	23.2	28.709	0.0000	23.684	9128800	108	15.08	14.68	15.36	15.34	13.62	13.55	14.37	13.39
+	3.163	1.39	2.61	O88428	Bifunctional 3-phosphoadenosine 5-phosphosulfate synthase 2;Sulfate a	Papss2	528	20	20	19	44.1	44.1	41.9	70.35	0.0000	96.804	21169000	217	16.42	16.04	16.25	16.14	14.78	14.46	15.40	14.67
+	2.301	1.38	2.61	Q99MD9	Nuclear autoantigenic sperm protein	Nasp	4462	7	7	7	12.5	12.5	12.5	83.953	0.0000	28.999	5288600	79	15.22	15.18	14.95	15.40	14.29	13.53	13.06	14.33

+	1.367	1.38	2.60	P59438	Hermansky-Pudlak syndrome 5 protein homolog	Hps5	1337	7	7	7	10.4	10.4	10.4	126.34	0.0000	17.397	3812000	35	14.57	13.72	15.20	14.61	13.26	13.85	13.62	11.87
+	2.805	1.38	2.59	Q8K078	Solute carrier organic anion transporter family member 4A1	Slco4a1	3599	4	4	4	9	9	9	77.668	0.0000	47.978	7646000	74	15.79	15.53	15.19	15.92	14.36	14.41	14.51	13.66
	0.540	1.37	2.59	A8C756	Thyroid adenoma-associated protein homolog	Thada	66	3	3	3	2.3	2.3	2.3	217.29	0.0096	1.8097	16430000	16	17.27	17.62	17.84	17.74	17.46	17.22	17.56	12.73
+	1.051	1.37	2.59	Q8BHA3	Probable D-tyrosyl-tRNA(Tyr) deacylase 2	Dtd2	3026	5	5	5	54.8	54.8	54.8	18.236	0.0000	66.409	7863700	94	16.01	15.62	15.75	15.92	14.69	12.50	15.51	15.11
+	1.665	1.37	2.59	Q9WTU6	Mitogen-activated protein kinase 9	Mapk9	5689	4	4	2	9.9	9.9	5.7	48.189	0.0004	4.926	6219200	55	15.24	15.19	15.28	15.49	14.13	14.69	12.66	14.23
+	2.272	1.37	2.59	Q08642	Protein-arginine deiminase type-2	Padi2	1765	7	7	7	14.3	14.3	14.3	76.249	0.0000	74.121	62483000	77	19.52	19.83	19.62	19.65	18.84	18.20	17.43	18.67
+	2.040	1.37	2.59	Q9JM90	Signal-transducing adaptor protein 1	Stap1	5500	8	8	8	38.7	38.7	38.7	34.627	0.0000	129.59	7745400	76	15.77	15.28	14.99	14.99	14.64	13.12	13.82	13.98
+	2.231	1.37	2.59	Q8CH72	E3 ubiquitin-protein ligase TRIM32	Trim32	3519	5	5	5	10.1	10.1	10.1	72.057	0.0000	18.669	4381200	49	14.64	13.95	15.36	14.95	13.36	13.33	13.71	13.03
+	2.612	1.37	2.58	Q9DCM2	Glutathione S-transferase kappa 1	Gstk1	5223	8	8	8	37.2	37.2	37.2	25.704	0.0000	95.692	9778300	105	15.83	15.39	16.31	16.06	14.23	15.09	14.38	14.42
+	1.319	1.36	2.57	Q9DB50	AP-1 complex subunit sigma-2	Ap1s2	5128	2	2	2	11.9	11.9	11.9	18.929	0.0013	3.5297	5687500	59	14.89	15.34	16.14	16.00	13.74	13.26	15.39	14.52
+	2.925	1.36	2.57	Q8C4Y3	Negative elongation factor B	Nelfb	3390	10	10	10	25.3	25.3	25.3	65.636	0.0000	66.752	10473000	124	15.48	14.83	15.57	15.66	14.06	13.61	14.19	14.23
+	2.163	1.36	2.57	P70658	C-X-C chemokine receptor type 4	Cxcr4	1579	3	3	3	8.6	8.6	8.6	40.426	0.0000	165.6	9197500	106	16.17	15.50	16.03	16.26	14.47	13.90	15.26	14.89
+	1.427	1.36	2.57	Q8R2T8	General transcription factor 3C polypeptide 5	Gtf3c5	3820	6	6	6	14.4	14.4	14.4	60.501	0.0000	9.0236	2456600	33	14.70	14.08	14.58	13.91	13.76	11.59	13.26	13.22
+	1.459	1.36	2.56	Q91WL8	WW domain-containing oxidoreductase	Wwox	4119	3	3	3	9.7	9.7	9.7	46.512	0.0000	7.5278	2812300	34	14.46	13.73	14.99	14.68	13.49	13.04	13.94	11.97
+	1.308	1.35	2.56	Q9ERG2	Striatin-3	Strn3	5310	11	10	8	16.2	14.9	13.6	87.149	0.0000	30.312	8013100	62	15.18	15.28	15.37	15.39	13.92	14.63	14.83	12.41
+	1.231	1.35	2.55	Q6ZWR6	Nesprin-1	Syne1	2705	14	14	14	2.5	2.5	2.5	1009.9	0.0000	41.886	6835000	42	14.67	14.80	15.44	15.80	13.03	13.05	15.21	14.02
	0.505	1.35	2.55	P53808	Phosphatidylcholine transfer protein	Pctp	1239	6	6	6	25.7	25.7	25.7	24.785	0.0000	19.516	14410000	32	15.60	17.40	17.11	16.43	17.56	12.75	16.92	13.91
+	1.334	1.35	2.55	Q8BXQ2	GPI transamidase component PIG-T	Pigt	3289	10	10	10	18	18	18	65.704	0.0000	39.669	5936200	22	14.76	15.41	15.06	14.82	14.15	13.81	14.52	12.17
+	1.316	1.35	2.55	O08848	60 kDa SS-A/Ro ribonucleoprotein	Trove2	307	7	7	7	15.2	15.2	15.2	60.123	0.0000	22.965	5924000	72	15.02	14.57	15.51	15.24	14.13	14.22	14.38	12.21
+	1.208	1.35	2.55	Q91ZT5	FYVE, RhoGEF and PH domain-containing protein 4	Fgd4	4200	13	13	13	24.2	24.2	24.2	86.54	0.0000	69.503	6606400	93	15.34	14.07	14.93	14.96	14.67	13.84	13.20	12.19
+	1.065	1.35	2.55	Q9CWU2	Palmitoyltransferase ZDHHC13	Zdhhc13	4731	6	6	6	14.6	14.6	14.6	70.889	0.0000	10.063	6727000	41	14.85	13.50	15.87	15.57	13.68	13.29	14.64	12.78
+	0.833	1.35	2.55	P51125	Calpastatin	Cast	1190	25	25	25	41.5	41.5	41.5	84.921	0.0000	323.31	25823000	227	16.08	15.26	15.89	15.99	15.30	15.01	15.41	12.11
+	2.356	1.35	2.54	Q8K2V6	Importin-11	Ipo11	3675	4	4	4	7.8	7.8	7.8	112.41	0.0000	83.189	3770100	75	14.82	14.06	13.79	14.12	12.75	13.34	12.97	12.33
+	2.325	1.35	2.54	P31266;O08	Recombining binding protein suppressor of hairless	Rbpj	986	15	15	15	32.1	32.1	32.1	58.537	0.0000	65.088	26345000	207	16.78	16.43	16.38	16.51	15.87	14.64	15.47	14.74
+	2.832	1.34	2.54	Q8BFP9	[Pyruvate dehydrogenase (acetyl-transferring)] kinase isozyme 1, mitocl	Pdk1	2941	7	7	7	23.5	23.5	23.5	48.994	0.0000	31.795	10527000	106	16.00	15.42	15.54	15.40	14.10	13.76	14.47	14.65
+	1.036	1.34	2.54	Q9CPU4	Microsomal glutathione S-transferase 3	Mgst3	4536	4	4	4	34	34	34	16.958	0.0000	60.109	22417000	150	16.99	15.89	16.81	15.95	16.02	14.67	16.05	13.52
+	3.590	1.34	2.54	P19973	Lymphocyte-specific protein 1	Lsp1	850	17	17	17	78.2	78.2	78.2	36.713	0.0000	323.31	56913000	349	18.16	18.52	17.83	18.12	17.11	16.75	16.73	16.66
	0.820	1.34	2.54	E9PYH6	Histone-lysine N-methyltransferase	Setd1a	223	8	8	8	7	7	7	186.06	0.0000	13.41	3970900	40	14.94	15.10	16.64	16.17	13.60	15.77	15.32	12.79
+	1.454	1.34	2.53	Q6P9N1	Hyccin	Fam126a	2588	7	7	7	25.5	25.5	25.5	57.321	0.0000	38.245	7042300	94	15.26	15.23	15.71	15.45	13.95	14.37	15.14	12.83
+	2.944	1.34	2.53	O70503	Very-long-chain 3-oxoacyl-CoA reductase	Hsd17b12	510	22	22	22	63.5	63.5	63.5	34.741	0.0000	323.31	230240000	674	19.29	18.42	18.76	18.75	17.68	17.36	17.72	17.10
+	2.167	1.34	2.52	P21107;P58	Tropomyosin alpha-3 chain	Tpm3	859	17	17	8	47.4	47.4	27.4	32.994	0.0000	304.24	110780000	380	18.83	19.10	18.44	18.77	18.11	16.88	17.81	17.00
+	2.578	1.34	2.52	Q8VCH6	Delta(24)-sterol reductase	Dhcr24	3921	14	14	14	24.4	24.4	24.4	60.112	0.0000	42.376	10752000	75	15.98	15.02	15.51	15.26	14.06	14.45	13.64	14.28
+	1.665	1.34	2.52	Q8BU88	39S ribosomal protein L22, mitochondrial	Mrpl22	3221	6	6	6	34.5	34.5	34.5	23.805	0.0000	19.286	13226000	64	17.39	16.11	17.36	17.34	14.90	15.94	15.70	16.31
+	2.694	1.33	2.52	Q921M4	Golgin subfamily A member 2	Golga2	4233	12	12	12	16.8	16.8	16.8	113.28	0.0000	29.604	6380600	64	15.40	15.03	14.84	15.20	14.18	13.67	14.10	13.20
+	0.878	1.33	2.52	Q9D8Z1	Activating signal cointegrator 1 complex subunit 1	Ascc1	5086	5	5	5	17.4	17.4	17.4	41.279	0.0009	3.9257	4674100	17	16.02	15.55	14.73	14.58	14.93	15.20	12.77	12.64
+	1.988	1.33	2.51	Q9D2V8	Major facilitator superfamily domain-containing protein 10	Mfsd10	4954	4	4	4	11.4	11.4	11.4	49.369	0.0000	15.459	21375000	125	17.17	16.66	16.85	17.07	16.17	14.77	16.17	15.35
+	2.707	1.33	2.51	O88441	Metaxin-2	Mtx2	529	12	12	12	63.5	63.5	63.5	29.758	0.0000	323.31	25485000	226	17.23	16.79	17.22	17.33	16.19	15.17	16.00	15.90
+	1.795	1.33	2.51	Q6R5N8	Toll-like receptor 13	Tlr13	2653	19	19	19	21.5	21.5	21.5	114.44	0.0000	160.93	47569000	300	18.32	17.78	17.71	17.68	17.14	15.65	17.16	16.23
+	3.356	1.32	2.50	Q9CWU9	Nucleoporin Nup37	Nup37	4733	5	5	5	23	23	23	36.731	0.0000	17.641	3937000	35	14.69	15.09	14.84	14.91	13.75	13.53	13.87	13.09
+	3.452	1.32	2.50	P97484	Leukocyte immunoglobulin-like receptor subfamily B member 3	Lilrb3	1661	17	17	17	27.2	27.2	27.2	93.053	0.0000	235.99	29773000	318	16.67	16.17	16.15	16.15	15.26	14.86	15.06	14.65
+	2.139	1.32	2.49	Q499X9	Methionine--tRNA ligase, mitochondrial	Mars2	2056	6	6	6	15	15	15	65.804	0.0000	28.539	2882600	52	14.25	14.00	14.64	14.30	12.10	13.12	13.20	13.50
+	1.250	1.32	2.49	Q9DCD6	Gamma-aminobutyric acid receptor-associated protein	Gabarap	5209	4	4	3	26.5	26.5	12	13.918	0.0000	8.5303	8154200	96	15.83	15.78	15.72	16.04	12.90	14.76	15.32	15.12
	0.531	1.32	2.49	Q9CQZ0;Q9	ORM1-like protein 2;ORM1-like protein 1	Ormdl2;Ormdl1	4650	2	2	2	14.4	14.4	14.4	17.389	0.0000	42.929	20576000	100	17.31	16.95	17.36	17.43	16.78	12.54	17.16	17.32
+	1.378	1.32	2.49	Q7TPD0	Integrator complex subunit 3	Ints3	2766	7	7	7	12	12	12	117.94	0.0000	166.63	5117900	75	15.56	15.05	15.29	14.44	13.73	12.55	14.67	14.13
+	2.008	1.31	2.49	P55096	ATP-binding cassette sub-family D member 3	Abcd3	1266	11	11	11	19.9	19.9	19.9	75.474	0.0000	126.63	22965000	223	16.62	15.90	16.40	16.47	15.32	14.16	15.63	15.01
+	0.967	1.31	2.48	Q9D938	Transmembrane protein 160	Tmem160	5093	3	3	3	16.5	16.5	16.5	19.587	0.0011	3.6272	5912200	41	15.59	15.41	15.64	15.06	14.72	12.47	15.63	13.65
+	1.992	1.31	2.48	Q8BZ98	Dynamin-3	Dnm3	3311	18	4	4	17.8	5.2	5.2	97.189	0.0000	14.961	14447000	92	16.45	16.19	16.52	16.30	15.43	15.02	15.67	14.10
+	1.007	1.31	2.48	Q921C5	Protein bicaudal D homolog 2	Bicd2	4217	8	8	8	12.8	12.8	12.8	93.39	0.0000	42.957	7765000	89	15.45	14.87	15.56	15.35	15.28	13.07	14.95	12.69
+	2.769	1.31	2.48	Q9ESE1;Q9	Lipopolysaccharide-responsive and beige-like anchor protein	Lrba	5336	9	9	8	4.3	4.3	3.7	317.06	0.0000	14.005	3305400	52	14.84	14.13	14.76	14.69	13.03	13.00	13.78	13.37
+	1.177	1.31	2.48	P52875	Transmembrane protein 165	Tmem165	1226	2	2	2	11.5	11.5	11.5	34.79	0.0000	15.74	6312100	72	15.13	14.71	15.90	15.46	14.28	12.46	14.90	14.31
	0.816	1.31	2.48	Q3TX08	tRNA (guanine(26)-N(2))-dimethyltransferase	Trmt1	1885	4	4	4	11.3	11.3	11.3	72.349	0.0000	30.064	4750100	47	15.22	15.31	15.92	15.21	14.46	14.79	15.34	11.83
+	2.042	1.31	2.47	Q62018	RNA polymerase-associated protein CTR9 homolog	Ctr9	2346	9	9	9	12.8	12.8	12.8	133.41	0.0000	48.939	5371200	87	14.75	14.30	14.59	14.66	13.44	13.67	13.68	12.30
+	1.746	1.30	2.47	Q8BXZ1	Protein disulfide-isomerase TMX3	Tmx3	3291	11	11	11	23.5	23.5	23.5	51.847	0.0000	42.849	46545000	153	17.31	17.24	18.31	17.67	16.48	16.58	16.86	15.40
+	1.351	1.30	2.47	Q9D6I7	Protein FAM69A	Fam69a	4994	3	3	3	9.6	9.6	9.6	48.936	0.0000	18.718	4125100	38	15.12	15.01	13.99	13.57	12.34	13.70	13.70	12.74
+	1.215	1.30	2.47	Q61830	Macrophage mannose receptor 1	Mrc1	2338	29	29	29	25.3	25.3	25.3	164.98	0.0000	151.27	29369000	245	16.32	15.40	15.13	14.58	15.09	13.43	14.44	13.25
+	2.118	1.30	2.46	P29387	Guanine nucleotide-binding protein subunit beta-4	Gnb4	967	8	4	4	27.6	13.5	13.5	37.379	0.0013	3.503	1885900	6	15.48	14.39	14.65	14.82	13.40	13.06	14.18	13.50
+	3.427	1.30	2.46	Q9CYR6	Phosphoacetylglucosamine mutase	Pgm3	4806	14	14	14	31.5	31.5	31.5	59.452	0.0000	26.581	11181000	116	15.41	15.05	15.61	15.61	14.19	13.91	14.45	13.94
+	2.021	1.30	2.46	Q8VEL2	Myotubularin-related protein 14	Mtmr14	4013	6	6	6	15.1	15.1	15.1	72.442	0.0000	50.148	6768700	37	15.65	15.19	15.20	15.39	14.15	14.21	13.16	14.73
+	1.431	1.30	2.45	P08101	Low affinity immunoglobulin gamma Fc region receptor II	Fcgr2	678	3	1	1	10.3	4.8	4.8	36.695	0.0004	4.6315	3694000	32	14.26	13.92	14.75	15.59	13.68	12.41	13.85	13.39
	0.505	1.29	2.45	P21300	Aldose reductase-related protein 1	Akr1b7	862	8	4	4	21.8	13.9	13.9	35.988	0.0002	6.1491	30290000	80	18.38	17.58	18.09	17.98	17.73	18.06	17.83	13.24
+	2.239	1.29	2.45	P27600	Guanine nucleotide-binding protein subunit alpha-12	Gna12	935	9	8	8	29.8	27.7	27.7	44.095	0.0000	19.197	4855800	40	15.39	15.07	14.66	14.33	13.63	14.06	13.07	13.50
+	2.204	1.29	2.45	Q8VDS8	Syntaxin-18	Stx18	3971	10	10	10	32.9	32.9	32.9	38.381	0.0000	38.889	8733600	121	16.19	15.40	15.65	15.95	14.89	14.25	14.99	13.89
+	1.476	1.29	2.45	P98197	Probable phospholipid-transporting ATPase IH	Atp11a	1690	2	2	2	3.3	3.3	3.3	135.5	0.0000	23.176	1205000	23	14.15	13.96	14.36	13.96	11.69	13.87	12.57	13.14
+	1.645	1.29	2.44	Q64133	Amine oxidase [flavin-containing] A	Maoa	2420	20	20	20	40.9	40.9	40.9	59.601	0.0000	176.5	42252000	270	17.19	16.50	17.94	17.95	15.83	15.59	16.68	16.32
+	3.069	1.29	2.44	Q8R323	Replication factor C subunit 3	Rfc3	3832	9	9	9	30.1	30.1	30.1	40.526	0.0000	67.985	8432500	110	15.45	14.84	15.30	15.56	14.37	13.81	13.77	14.05
+	0.972	1.28	2.44	Q6QLQ4	C-type lectin domain family 7 member A	Clec7a	2652	3	3	3	17.6	17.6	17.6	27.42	0.0000	190.25	10707000	82	16.43	15.85	16.17	16.25	15.34	12.92	15.86	15.45
	0.549	1.28	2.43	P09041	Phosphoglycerate kinase 2	Pgk2	691	12	4	3	25.2	10.8	5.8	44.852	0.0009	3.8079	7468200	33	17.50	15.00	12.42	15.60	13.22	14.58	13.85	13.74
	0.364	1.28	2.43	Q6X893	Choline transporter-like protein 1	Slc44a1	2668	4	4	3	8.4	8.4	6	73.066	0.0013	3.421	6504000	42	16.53	11.29	15.22	16.55	12.25	16.22	12.90	13.08
+	1.963	1.28	2.43	Q8K4R4	Cytoplasmic phosphatidylinositol transfer protein 1	Pitpnc1	3728	4	4	4	13.9	13.9	13.9	38.383	0.0000	6.227	4478200	25	13.14	13.83	13.86	13.46	12.98	12.49	12.19	11.51
+	1.832	1.28	2.43	P70404	Isocitrate dehydrogenase [NAD] subunit gamma 1, mitochondrial	Idh3g	1567	15	15	15	48.1	48.1	48.1	42.785	0.0000	323.31	47393000	325	17.27	16.87	17.00	16.98	16.17	15.74	16.37	14.72
+	3.208	1.28	2.42	P28659	CUGBP Elav-like family member 1	Celf1	956	11	9	9	27.8	23.5	23.5	52.107	0.0000	65.313	10463000	122	15.62	15.28	15.92	15.84	14.52	14.17	14.70	14.15
+	1.057	1.28	2.42	B9EJR8	Dynein assembly factor 5, axonemal	Dnaaf5	94	3	3	3	6.7	6.7	6.7	93.85	0.0000	11.006	1864300	23	14.52	14.44	14.29	14.69	11.35	13.93	13.64	13.90
+	0.988	1.28	2.42	Q9D0M5	Dynein light chain 2, cytoplasmic	Dynll2	4880	9	6	6	74.2	43.8	43.8	10.35	0.0000	137.53	18315000	146	16.37	16.60	16.44	16.98	16.19	15.13	16.39	13.56
+	1.980	1.28	2.42	Q8BMQ2	General transcription factor 3C polypeptide 4	Gtf3c4	3146	10	10	10	19.2	19.2	19.2	91.612	0.0000	47	5832600	52	14.96	14.36	15.42	14.90	13.91	13.67	14.09	12.85
+	1.163	1.28	2.42	Q9JJA4	Ribosome biogenesis protein WDR12	Wdr12	5422	7	7	7	30	30	30	47.346	0.0000	45.657	8948300	116	14.58	15.18	15.59	15.24	14.69	12.49	14.76	13.56
+	1.236	1.28	2.42	P10833	Ras-related protein R-Ras	Rras	730	11	11	9	56	56	56	23.764	0.0000	258.97	13122000	153	15.86	15.48	15.35	15.12	14.62	15.14	14.24	12.71
+	3.588	1.28	2.42	P14234	Tyrosine-protein kinase Fgr	Fgr	779	13	13	13	35.6	35.6	35.6	58.866	0.0000	29.851	23017000	104	17.30	16.95	16.89	16.68	15.86	15.37	15.76	15.73
	0.794	1.28	2.42	Q9CZP5	Mitochondrial chaperone BCS1	Bcs1l	4836	6	6	6	23.7	23.7	23.7	47.406	0.0000	25.817	3184900	24	15.01	14.27	14.68	15.62	14.19	11.74	15.25	13.30
+	1.070	1.27	2.42	Q6DFW0	Protein C9orf72 homolog		2505	5	5	5	16.2	16.2	16.2	54.277	0.0000	11.801	4870400	37	15.83	14.37	15.96	13.62	13.23	13.90	14.23	13.31
	0.664	1.27	2.42	Q9QZN4	F-box only protein 6	Fbxo6	5602	5	5	5	19	19	19	34.492	0.0000	53.084	8495300	66	16.62	15.47	15.78	15.25	15.21	15.27	15.65	11.90
+	0.931	1.27	2.42	Q9QXG2	Rab proteins geranylgeranyltransferase component A 1	Chm	5540	5	5	5	15.2	15.2	15.2	73.976	0.0000	78.631	5839500	82	16.20	15.25	15.46	14.54	15.11	12.96	15.16	13.12
+	3.515	1.27	2.41	P26323;P81	Friend leukemia integration 1 transcription factor	Fli1	921	7	7	7	23.9	23.9	23.9	51.001	0.0000	42.381	5707900	86	15.12	15.01	14.72	15.14	13.51	14.03	13.47	13.90
+	1.274	1.27	2.41	Q99N16	Leukotriene-B(4) omega-hydroxylase 2	Cyp4f3	4482	2	2	2	4.8	4.8	4.8	59.842	0.0015	3.3629	1977100	18	14.42	12.82	14.42	14.50	12.18	13.71	12.76	12.43
+	1.779	1.27	2.41	Q99J83	Autophagy protein 5	Atg5	4331	11	11	11	30.9	30.9	30.9	32.402	0.0000	110.29	12451000	134	15.58	15.12	15.95	15.92	14.82	13.40	14.50	14.78
+	2.553	1.27	2.41	Q61211	Eukaryotic translation initiation factor 2D	Eif2d	2279	10	10	10	30.7	30.7	30.7	62.829	0.0000	40.428	9717600	90	16.39	15.93	16.18	16.28	14.97	15.00	15.46	14.29
+	2.639	1.27	2.41	O35218	Cleavage and polyadenylation specificity factor subunit 2	Cpsf2	347	13	13	13	18.7	18.7	18.7	88.382	0.0000	54.068	9534000	115	14.98	14.97	15.13	15.02	14.25	13.38	14.11	13.29
+	1.661	1.26	2.40	O70251	Elongation factor 1-beta	Eef1b	479	7	7	7	33.3	33.3	33.3	24.693	0.0000	60.173	26781000	126	17.21	17.69	17.20	17.27	16.52	16.60	14.92	16.28
+	0.951	1.26	2.40	Q925N0	Sideroflexin-5	Sfxn5	4309	4	4	4	18.7	18.7	18.7	37.328	0.0000	15.383	11960000	46	17.94	15.98	16.51	15.88	16.15	13.92	15.55	15.63
+	1.411	1.26	2.40	Q9QYB5	Gamma-adducin	Add3	5567	9	9	9	19.3	19.3	19.3	78.776	0.0000	67.75	5857500	66	15.01	14.95	15.05	15.21	14.64	14.15	13.95	12.44
	0.608	1.26	2.39	Q61285	ATP-binding cassette sub-family D member 2	Abcd2	2288	3	3	3	6.5	6.5	6.5	83.482	0.0000	12.249	2862200	14	14.82	12.73	12.45	16.43	12.43	12.26	13.35	13.36
+	2.232	1.26	2.39	P31786	Acyl-CoA-binding protein	Dbi	990	9	9	8	85.1	85.1	83.9	10	0.0000	72.266	29149000	159	17.99	18.47	17.53	17.87	16.90	17.26	16.29	16.36
+	2.934	1.26	2.39	Q8VHE0	Translocation protein SEC63 homolog	Sec63	4018	11	11	11	15.4	15.4	15.4	87.869	0.0000	82.615	31661000	252	16.40	15.65	16.52	16.28	14.79	14.80	15.06	15.18
+	1.150	1.25	2.39	P61211	ADP-ribosylation factor-like protein 1	Arl1	1378	5	5	5	22.1	22.1	22.1	20.411	0.0000	170.4	30006000	232	17.70	17.36	16.80	16.76	16.79	14.80	16.80	15.20
+	1.752	1.25	2.38	Q8C8U0	Liprin-beta-1	Ppfibp1	3423	11	10	10	17	15.8	15.8	108.54	0.0000	85.138	5842400	74	15.14	14.66	15.12	15.14	13.87	14.03	14.44	12.72
+	2.373	1.25	2.38	O55137;Q9(	Acyl-coenzyme A thioesterase 1	Acot1	460	14	4	3	44.9	15.8	12.6	46.135	0.0000	15.05	6507900	54	15.69	15.44	15.46	15.17	14.14	14.07	14.89	13.64
+	0.937	1.25	2.38	Q9WTZ1	RING-box protein 2	Rnf7	5694	3	3	3	37.2	37.2	37.2	12.707	0.0000	9.6279	1629300	19	14.29	13.17	13.90	13.45	12.50	11.43	14.23	11.66
+	2.724	1.25	2.38	Q9R1S8	Calpain-7	Capn7	5662	7	7	7	13.9	13.9	13.9	92.563	0.0000	50.577	6511300	93	15.74	15.09	15.37	15.53	13.89	13.96	14.74	14.13
+	2.221	1.25	2.38	Q9D4H1	Exocyst complex component 2	Exoc2	4976	20	20	20	25.8	25.8	25.8	103.96	0.0000	123.93	28619000	335	16.26	15.66	16.28	16.02	15.16	14.75	15.23	14.08
+	1.311	1.25	2.37	Q8BGY7	Protein FAM210A	Fam210a	3001	6	6	6	30	30	30	31.599	0.0000	22.493	4030500	43	14.35	14.21	13.74	14.29	11.63	13.59	13.73	12.66
	0.795	1.25	2.37	Q8BX02	KN motif and ankyrin repeat domain-containing protein 2	Kank2	3274	6	6	6	9.6	9.6	9.6	90.244	0.0000	39.37	3829500	37	15.03	14.56	14.59	14.05	14.37	13.55	14.21	11.12
+	1.812	1.24	2.37	O88643	Serine/threonine-protein kinase PAK 1	Pak1	553	15	9	8	31.9	22.2	20.6	60.736	0.0000	67.659	7336600	63	15.23	15.59	15.55	15.84	14.52	13.27	14.77	14.68
+	3.334	1.24	2.36	P62482	Voltage-gated potassium channel subunit beta-2	Kcnab2	1430	24	24	21	71.4	71.4	66.2	41.021	0.0000	323.31	131360000	621	18.36	17.87	18.27	18.23	17.01	16.54	17.23	16.98
+	1.201	1.24	2.36	Q3UDF0		Slc2a6	1947	11	11	10	17.9	17.9	17.1	54.433	0.0000	151.23	19161000	129	16.31	15.77	15.41	15.29	15.41	14.85	14.47	13.09
+	3.126	1.24	2.36	P29758	Ornithine aminotransferase, mitochondrial	Oat	972	34	34	34	78.6	78.6	78.6	48.354	0.0000	323.31	155750000	771	18.76	18.28	18.06	18.20	17.38	16.92	17.22	16.83
+	2.251	1.24	2.36	Q9D8V0	Minor histocompatibility antigen H13	Hm13	5077	4	4	4	17.2	17.2	17.2	41.747	0.0000	58.924	38310000	176	17.77	16.88	17.56	17.16	16.45	15.47	16.29	16.20
+	2.582	1.24	2.36	Q3KNM2	E3 ubiquitin-protein ligase MARCH5	March5	1809	4	4	4	16.5	16.5	16.5	31.231	0.0000	8.0107	3859900	44	14.53	13.70	14.61	13.86	12.70	12.90	13.19	12.96
+	2.158	1.24	2.36	Q8VDC0	Probable leucine--tRNA ligase, mitochondrial	Lars2	3946	13	13	13	22.1	22.1	22.1	101.48	0.0000	83.804	10386000	117	16.01	15.35	15.89	15.55	13.83	14.66	14.27	15.08
+	3.844	1.24	2.36	Q9DBR7	Protein phosphatase 1 regulatory subunit 12A	Ppp1r12a	5172	30	30	28	34.2	34.2	33.2	114.99	0.0000	136.05	20943000	159	17.11	16.52	16.67	16.85	15.74	15.42	15.48	15.58
	0.457	1.24	2.35	E9Q7D5		Arhgef5	246	4	4	4	2.8	2.8	2.8	176.66	0.0025	2.8085	29350000	46	17.82	18.33	18.16	17.92	17.95	17.84	13.20	18.30
+	1.412	1.23	2.35	Q3UM29	Conserved oligomeric Golgi complex subunit 7	Cog7	1996	15	15	15	29.6	29.6	29.6	86.072	0.0000	147.43	13972000	189	15.64	15.19	14.58	14.94	14.38	12.88	14.67	13.47
+	4.525	1.23	2.35	Q9JI08	Bridging integrator 3	Bin3	5380	16	16	16	59.7	59.7	59.7	29.65	0.0000	103.83	24141000	183	15.77	15.96	15.50	15.93	14.62	14.48	14.56	14.56
+	2.495	1.23	2.35	O35250	Exocyst complex component 7	Exoc7	351	35	35	35	61.1	61.1	61.1	79.959	0.0000	204.76	41200000	466	16.15	15.87	16.15	15.92	15.13	15.04	14.93	14.05
+	2.315	1.23	2.35	Q9JKF7	39S ribosomal protein L39, mitochondrial	Mrpl39	5451	13	13	13	43.2	43.2	43.2	38.549	0.0000	90.861	17385000	210	15.99	15.41	16.18	15.76	14.96	14.32	15.02	14.11
+	1.590	1.23	2.35	Q80TV8	CLIP-associating protein 1	Clasp1	2814	15	13	13	15.1	13.2	13.2	169.23	0.0000	36.998	6532700	100	14.90	14.46	15.18	14.84	13.24	14.27	14.25	12.69

+	1.285	1.23	2.35	Q91W89	Alpha-mannosidase 2C1	Man2c1	4097	15	15	15	18.6	18.6	18.6	115.69	0.0000	151.58	15687000	162	15.92	15.33	15.55	15.67	15.29	14.31	14.91	13.03
+	1.878	1.23	2.35	Q924L1	LETM1 domain-containing protein 1	Letmd1	4296	14	14	14	38.9	38.9	38.9	41.7	0.0000	57.028	17924000	140	15.84	14.76	15.39	15.16	14.24	13.56	14.74	13.69
	0.957	1.22	2.34	Q9ESJ4	NCK-interacting protein with SH3 domain	Nckipsd	5338	10	10	10	16.9	16.9	16.9	78.572	0.0000	18.622	5610400	59	15.13	15.07	15.33	14.97	14.21	14.32	15.04	12.03
+	2.832	1.22	2.34	Q8CB96	Ras association domain-containing protein 4	Rassf4	3434	13	11	11	49.4	41.3	41.3	36.934	0.0000	28.702	10030000	51	16.01	15.92	15.83	15.73	14.98	14.80	14.79	14.02
+	3.182	1.22	2.33	Q8CHH9	Septin-8	Sep.08	3523	21	14	13	56.2	44.3	44.3	49.812	0.0000	135.3	28268000	204	17.03	16.70	16.93	16.94	15.72	15.41	16.16	15.43
+	1.486	1.22	2.33	Q9CZB0	Succinate dehydrogenase cytochrome b560 subunit, mitochondrial	Sdhc	4824	1	1	1	7.1	7.1	7.1	18.382	0.0018	3.1453	1858500	36	14.60	14.06	13.64	13.35	13.06	12.43	11.84	13.42
+	3.101	1.22	2.33	Q80TM9	Nischarin	Nisch	2808	27	27	27	23.1	23.1	23.1	175.01	0.0000	198.93	30621000	352	16.57	15.85	16.73	16.41	15.18	15.12	15.28	15.10
+	3.738	1.22	2.33	P51569	Alpha-galactosidase A	Gla	1197	7	7	7	22	22	22	47.642	0.0000	69.169	82181000	370	18.28	17.95	18.40	18.21	16.96	16.71	17.27	17.03
+	2.490	1.22	2.33	Q8BIP0	Aspartate--tRNA ligase, mitochondrial	Dars2	3063	5	5	5	10.6	10.6	10.6	74.101	0.0000	9.2232	3860700	47	14.52	14.37	15.18	14.89	13.11	13.54	13.99	13.46
+	2.099	1.22	2.33	Q8BZQ7	Anaphase-promoting complex subunit 2	Anapc2	3317	2	2	2	3.6	3.6	3.6	95.306	0.0000	10.186	2686500	56	14.40	13.79	14.53	13.35	12.99	12.40	13.04	12.76
+	1.667	1.22	2.33	P28293	Cathepsin G	Ctsg	948	10	10	10	33	33	33	29.095	0.0000	35.973	30968000	171	15.98	16.33	17.50	16.88	15.34	16.04	15.02	15.43
+	1.289	1.21	2.32	Q2YDW2	Protein misato homolog 1	Msto1	1804	9	9	9	25.9	25.9	25.9	61.23	0.0000	33.596	5353400	37	14.63	13.46	15.18	15.08	14.22	12.92	13.44	12.91
+	1.867	1.21	2.32	Q8VDG5	Phosphopantothenate--cysteine ligase	Ppcs	3952	3	3	3	14.8	14.8	14.8	33.794	0.0000	7.8979	2526900	19	15.08	14.81	15.25	14.20	13.05	13.59	14.32	13.52
+	1.063	1.21	2.32	Q3UJP5	Protein C8orf37 homolog		1985	2	2	2	13.9	13.9	13.9	23.848	0.0000	15.335	4760100	66	15.08	14.51	15.52	15.07	14.56	13.87	14.64	12.25
+	3.039	1.21	2.32	Q60870	Receptor expression-enhancing protein 5	Reep5	2217	11	11	11	27	27	27	21.05	0.0000	240	113120000	507	18.58	17.94	18.10	18.45	17.08	16.86	17.43	16.84
+	1.661	1.21	2.32	Q99KU0	Vacuole membrane protein 1	Vmp1	4395	3	3	3	12.6	12.6	12.6	45.96	0.0000	38.841	9302700	60	16.44	15.37	16.70	16.47	14.91	14.71	15.80	14.71
+	0.990	1.21	2.32	P24788	Cyclin-dependent kinase 11B	Cdk11b	903	12	12	12	14.5	14.5	14.5	91.512	0.0000	19.195	7655300	62	15.58	14.71	15.27	15.80	14.73	15.07	14.26	12.45
+	2.094	1.21	2.32	Q8C547	HEAT repeat-containing protein 5B	Heatr5b	3392	23	23	23	17.6	17.6	17.6	224.32	0.0000	81.041	11416000	159	15.26	14.20	15.32	14.99	14.03	13.22	13.88	13.78
	0.877	1.21	2.31	Q505B7	Protein archease	Zbtb8os	2078	2	2	2	17.9	17.9	17.9	19.727	0.0002	5.2571	1228500	16	13.78	12.91	14.59	15.60	12.16	14.00	12.68	13.20
+	1.173	1.21	2.31	Q8VCY6	U3 small nucleolar RNA-associated protein 6 homolog	Utp6	3937	3	3	3	8.4	8.4	8.4	70.429	0.0000	30.576	2662900	43	14.27	13.59	15.48	14.38	13.30	12.73	14.25	12.60
+	2.153	1.21	2.31	Q8C7V3	U3 small nucleolar RNA-associated protein 15 homolog	Utp15	3416	7	7	7	18.8	18.8	18.8	59.374	0.0000	32.057	10047000	98	16.10	15.83	15.95	16.02	14.47	14.72	15.61	14.28
+	1.645	1.21	2.31	Q8K4X7	1-acyl-sn-glycerol-3-phosphate acyltransferase delta	Agpat4	3729	11	11	11	24.9	24.9	24.9	43.81	0.0000	26.729	11661000	97	15.78	14.55	16.13	15.87	14.37	14.25	14.87	14.00
+	1.125	1.21	2.31	Q9D8V7	Signal peptidase complex catalytic subunit SEC11C	Sec11c	5078	6	6	6	33.3	33.3	33.3	21.66	0.0000	50.162	4910500	47	13.99	13.87	15.07	15.04	14.18	12.35	13.95	12.66
+	1.272	1.20	2.30	P97872	Dimethylaniline monooxygenase [N-oxide-forming] 5	Fmo5	1683	4	4	4	11.3	11.3	11.3	60	0.0004	5.191	7241600	40	15.55	15.21	16.55	15.69	15.33	13.39	14.85	14.61
+	1.347	1.20	2.30	Q810S1	Calcium uniporter regulatory subunit MCUb, mitochondrial	Ccdc109b	2931	9	9	9	38.6	38.6	38.6	39.799	0.0000	108.09	14159000	104	16.61	14.94	16.28	16.62	15.17	15.48	14.31	14.68
+	2.565	1.20	2.30	Q9DCJ1	Target of rapamycin complex subunit LST8	Mlst8	5216	8	8	7	39	39	31.3	35.851	0.0000	20.891	6235100	71	15.54	15.31	15.66	15.28	14.52	13.87	14.75	13.85
+	1.182	1.20	2.30	Q571G4	Protein lin-54 homolog	Lin54	2091	4	4	4	11.2	11.2	11.2	79.565	0.0002	6.1886	3035700	22	14.43	15.28	15.29	13.79	12.36	14.07	13.97	13.59
+	2.296	1.20	2.30	O35134	DNA-directed RNA polymerase I subunit RPA1	Polr1a	341	8	8	8	7.3	7.3	7.3	194.11	0.0000	26.563	5280800	59	16.08	15.06	16.05	15.81	14.35	14.25	14.82	14.79
+	1.753	1.20	2.30	Q5U3K5	Rab-like protein 6	Rabl6	2162	7	7	7	13.9	13.9	13.9	79.83	0.0000	11.85	4612500	45	14.88	14.11	15.10	14.78	13.73	12.61	13.84	13.88
+	3.026	1.20	2.29	P58137	Acyl-coenzyme A thioesterase 8	Acot8	1309	10	10	10	42.2	42.2	42.2	35.827	0.0000	84.919	10995000	87	15.53	15.20	15.82	15.46	14.13	14.09	14.75	14.25
+	1.993	1.20	2.29	Q9EP82	tRNA (guanine-N(7)-)-methyltransferase non-catalytic subunit WDR4	Wdr4	5246	4	4	4	12.1	12.1	12.1	45.755	0.0002	5.5103	5416100	48	14.75	14.46	15.49	15.62	13.84	14.06	14.18	13.45
	0.751	1.20	2.29	Q8VCL2	Protein SCO2 homolog, mitochondrial	Sco2	3924	5	5	5	29.4	29.4	29.4	28.944	0.0000	16.626	7261300	66	15.25	14.90	15.60	15.27	14.56	14.70	15.19	11.79
+	2.060	1.19	2.29	Q9WVL0	Maleylacetoacetate isomerase	Gstz1	5753	3	3	3	19.4	19.4	19.4	24.275	0.0000	157.71	6361600	50	15.43	15.14	16.29	15.90	14.37	14.20	15.03	14.40
+	1.529	1.19	2.28	Q8C561	LMBR1 domain-containing protein 2	Lmbrd2	3393	8	8	8	14.3	14.3	14.3	81.1	0.0000	159.72	4103600	53	13.99	12.98	14.54	14.56	12.58	13.14	12.39	13.19
+	1.292	1.19	2.28	Q8C167	Prolyl endopeptidase-like	Prepl	3356	11	11	11	21.4	21.4	21.4	83.193	0.0000	83.775	7243300	106	14.70	14.58	15.12	14.63	13.86	13.21	12.48	14.71
+	2.819	1.19	2.28	P70699	Lysosomal alpha-glucosidase	Gaa	1587	19	19	19	26.8	26.8	26.8	106.25	0.0000	194.04	39876000	367	16.37	16.39	16.75	16.66	15.76	14.83	15.48	15.33
+	2.853	1.19	2.28	P97814	Proline-serine-threonine phosphatase-interacting protein 1	Pstpip1	1675	17	17	17	32.5	32.5	32.5	47.59	0.0000	29.465	34811000	246	17.00	16.64	16.91	16.77	15.59	15.10	15.98	15.89
+	1.950	1.19	2.28	Q9CQ26	STAM-binding protein	Stambp	4556	8	8	8	26.7	26.7	26.7	48.513	0.0000	23.25	7620400	92	15.75	15.16	15.91	15.60	14.58	14.62	14.88	13.58
+	3.292	1.19	2.27	O70551;Q9Z	SRSF protein kinase 1	Srpk1	512	7	5	5	12.8	10.2	10.2	73.088	0.0008	4.1129	1921400	24	13.90	13.89	13.97	14.40	12.92	12.98	12.49	13.05
	0.985	1.19	2.27	Q8R5F7	Interferon-induced helicase C domain-containing protein 1	Ifih1	3887	30	30	29	36.2	36.2	34.8	115.97	0.0000	84.3	31479000	182	18.03	17.12	17.25	16.53	16.87	16.04	16.74	14.54
+	1.204	1.18	2.27	Q8BYB9	Protein O-glucosyltransferase 1	Poglut1	3296	7	7	7	19.6	19.6	19.6	46.379	0.0000	14.095	10203000	91	16.26	15.71	15.87	15.59	14.99	15.25	15.26	13.19
+	1.624	1.18	2.27	Q9R0I7	YLP motif-containing protein 1	Ylpm1	5627	11	11	11	12.2	12.2	12.2	155.13	0.0000	16.553	9909700	34	16.96	16.97	15.76	16.89	15.86	14.71	15.54	15.73
+	1.354	1.18	2.27	Q3URF8	BTB/POZ domain-containing protein KCTD21	Kctd21	2023	4	4	4	30.4	30.4	30.4	29.623	0.0000	22.407	4350700	57	15.05	14.57	15.50	15.20	13.12	15.10	13.71	13.67
+	2.200	1.18	2.27	Q8VDQ1	Prostaglandin reductase 2	Ptgr2	3967	22	22	22	68.9	68.9	68.9	38.015	0.0000	323.31	84315000	357	16.88	17.52	17.85	17.24	16.42	15.65	16.56	16.13
+	2.989	1.18	2.27	Q8CI94	Glycogen phosphorylase, brain form	Pygb	3545	59	49	43	68.3	60	53.1	96.729	0.0000	323.31	154950000	737	18.62	18.03	18.41	18.55	17.27	16.99	17.63	17.00
	0.651	1.18	2.26	Q3UPH1	Protein PRRC1	Prrc1	2014	10	10	10	33.9	33.9	33.9	46.297	0.0000	149.69	25625000	166	16.69	16.43	16.40	16.45	16.19	16.36	15.98	12.73
	0.832	1.18	2.26	O88907	E3 SUMO-protein ligase PIAS1	Pias1	584	4	4	3	8.9	8.9	6.6	71.617	0.0000	18.798	4264800	21	14.67	14.16	15.65	15.38	11.92	14.39	14.44	14.39
+	2.316	1.18	2.26	Q9D7J6	Deoxyribonuclease-1-like 1	Dnase1l1	5033	5	5	5	19.4	19.4	19.4	35.604	0.0000	56.793	3741400	62	14.20	14.54	14.74	14.55	13.82	13.16	13.63	12.72
+	2.277	1.18	2.26	Q9R0H0	Peroxisomal acyl-coenzyme A oxidase 1	Acox1	5626	20	20	20	42.8	42.8	42.8	74.648	0.0000	90.093	15483000	120	15.57	15.61	15.97	16.19	14.55	14.75	15.23	14.11
+	1.154	1.18	2.26	Q61470	Leukocyte antigen CD37	Cd37	2304	3	3	2	14.2	14.2	10	31.852	0.0000	25.986	3606700	38	14.63	14.93	15.05	15.17	14.79	12.83	14.55	12.91
+	4.461	1.18	2.26	Q9WTL7	Acyl-protein thioesterase 2	Lypla2	5678	18	18	18	69.3	69.3	69.3	24.794	0.0000	323.31	215970000	638	18.17	18.38	18.28	18.41	16.88	17.10	17.28	17.28
+	1.256	1.18	2.26	Q8BTI9	Phosphatidylinositol 4,5-bisphosphate 3-kinase catalytic subunit beta is	Pik3cb	3202	9	9	9	12.3	12.3	12.3	121.71	0.0000	23.569	5994300	76	14.91	14.93	14.39	15.17	13.99	13.55	14.70	12.46
+	3.273	1.18	2.26	Q9QXE7	F-box-like/WD repeat-containing protein TBL1X	Tbl1x	5539	7	5	5	24.3	16.5	16.5	56.802	0.0000	37.124	4639400	65	14.79	14.49	14.76	14.69	13.29	13.29	13.98	13.46
	1.001	1.17	2.26	Q3TAP4	AP-5 complex subunit beta-1	Ap5b1	1814	6	6	6	13.6	13.6	13.6	94.678	0.0000	21.931	7355000	60	15.92	16.07	14.63	15.83	15.22	14.73	14.85	12.95
+	2.707	1.17	2.26	P70426	GTP-binding protein Rit1	Rit1	1571	5	5	5	32.4	32.4	32.4	25.169	0.0000	20.434	2214600	41	14.44	13.51	13.82	14.25	12.64	13.02	12.79	12.88
+	1.872	1.17	2.26	Q61214;Q9Z	Dual specificity tyrosine-phosphorylation-regulated kinase 1A	Dyrk1a	2280	8	8	7	11.3	11.3	9.2	85.493	0.0000	20.427	3701700	38	14.41	13.74	14.29	13.91	12.89	13.75	12.66	12.35
	0.978	1.17	2.25	A2ALW5	DnaJ homolog subfamily C member 25	Dnajc25	33	5	5	5	13.7	13.7	13.7	41.937	0.0000	30.855	2829100	39	13.34	12.72	14.29	14.07	11.57	13.86	12.01	12.30
+	1.293	1.17	2.25	Q9CRA5	Golgi phosphoprotein 3	Golph3	4685	11	11	10	53.4	53.4	53.4	33.752	0.0000	83.948	13305000	193	15.57	14.96	15.35	15.43	14.11	12.87	14.74	14.91
	0.441	1.17	2.25	P51437	Cathelin-related antimicrobial peptide	Camp	1196	3	3	3	21.5	21.5	21.5	19.453	0.0000	104.66	9438700	66	14.45	14.93	16.94	15.83	15.03	11.27	15.95	15.22
+	1.796	1.17	2.25	Q9CQT1	Methylthioribose-1-phosphate isomerase	Mri1	4632	11	11	11	50.7	50.7	50.7	39.41	0.0000	131.05	23505000	139	16.71	16.41	16.60	16.50	15.84	14.36	15.60	15.74
+	1.459	1.17	2.24	P97470	Serine/threonine-protein phosphatase 4 catalytic subunit	Ppp4c	1656	9	7	7	37.5	32.6	32.6	35.08	0.0000	36.433	16823000	60	16.74	16.44	17.44	16.86	16.32	14.96	16.38	15.16
+	1.784	1.16	2.24	Q3V0C5	Ubiquitin carboxyl-terminal hydrolase 48	Usp48	2048	11	11	11	17.3	17.3	17.3	120.63	0.0000	45.106	5987000	68	14.80	14.07	14.80	14.81	13.22	13.40	14.30	12.89
+	2.338	1.16	2.24	Q9D0Q7	39S ribosomal protein L45, mitochondrial	Mrpl45	4882	12	12	12	47.7	47.7	47.7	35.41	0.0000	49.605	16056000	124	16.12	15.40	15.26	15.53	14.61	14.13	14.84	14.07
+	1.072	1.16	2.24	Q8N7N5	DDB1- and CUL4-associated factor 8	Dcaf8	3732	5	5	5	9.8	9.8	9.8	66.031	0.0000	39.144	8163400	40	16.95	15.93	15.94	16.41	15.58	15.67	15.70	13.61
+	2.693	1.16	2.24	P40237	CD82 antigen	Cd82	1059	2	2	2	4.5	4.5	4.5	29.628	0.0002	5.3776	4971700	48	15.61	15.02	15.61	15.84	14.25	14.57	14.02	14.59
+	4.246	1.16	2.24	Q99K23	Ufm1-specific protease 2	Ufsp2	4360	13	13	13	47.9	47.9	47.9	52.515	0.0000	59.414	14738000	151	16.11	15.77	15.85	15.92	14.62	14.77	15.00	14.62
+	1.976	1.16	2.24	P61965	WD repeat-containing protein 5	Wdr5	1395	13	13	13	54.8	54.8	54.8	36.588	0.0000	49.696	24148000	161	16.54	15.99	16.00	16.25	15.49	14.96	15.45	14.25
+	2.049	1.16	2.23	Q8CGZ0	Calcium homeostasis endoplasmic reticulum protein	Cherp	3515	11	11	11	16.3	16.3	16.3	106.17	0.0000	105.76	11277000	172	15.29	15.18	15.17	15.41	14.19	14.03	14.83	13.37
+	2.730	1.16	2.23	P35821;REV	Tyrosine-protein phosphatase non-receptor type 1	Ptpn1	1033	24	24	24	63	63	63	49.593	0.0000	230.65	77658000	405	17.80	17.08	17.69	17.59	16.43	16.03	16.75	16.31
+	3.259	1.16	2.23	P61202	COP9 signalosome complex subunit 2	Cops2	1375	21	21	21	63	63	63	51.596	0.0000	323.31	55993000	479	17.19	16.74	16.92	16.93	15.90	15.58	16.15	15.53
+	1.395	1.16	2.23	Q9QUK4;Q9	Baculoviral IAP repeat-containing protein 1b	Naip2	5517	12	12	12	13.5	13.5	13.5	164.08	0.0000	53.067	9705900	97	15.04	14.11	15.32	15.21	13.64	13.32	14.78	13.32
+	2.551	1.16	2.23	Q920E5	Farnesyl pyrophosphate synthase	Fdps	4210	28	28	28	64	64	64	40.581	0.0000	323.31	211400000	715	19.26	18.94	18.87	18.94	18.31	18.09	17.67	17.32
+	1.571	1.16	2.23	Q9JJQ0	GPI mannosyltransferase 3	Pigb	5432	5	5	5	13.8	13.8	13.8	63.119	0.0000	26.339	3786700	25	14.80	13.96	14.64	14.41	12.88	13.08	14.35	12.89
+	1.503	1.15	2.23	Q7TSQ8	Pyruvate dehydrogenase phosphatase regulatory subunit, mitochondria	Pdpr	2787	10	10	10	16.1	16.1	16.1	99.229	0.0000	57.062	5787900	51	14.75	14.28	15.66	15.42	13.61	13.27	14.46	14.17
+	2.305	1.15	2.22	Q8BP40	Lysophosphatidic acid phosphatase type 6	Acp6	3157	14	14	14	34.4	34.4	34.4	47.624	0.0000	116.12	36438000	232	16.53	16.58	16.87	16.95	15.25	15.43	15.32	16.31
+	2.257	1.15	2.22	Q9D0T2	Dual specificity protein phosphatase 12	Dusp12	4888	3	3	3	17.4	17.4	17.4	37.158	0.0013	3.4419	21018000	34	18.18	17.40	18.48	18.47	17.17	16.85	17.14	16.77
	0.928	1.15	2.22	P48453	Serine/threonine-protein phosphatase 2B catalytic subunit beta isoform	Ppp3cb	1146	14	6	6	35.2	16.6	16.6	59.173	0.0000	22.835	5266400	48	15.07	13.29	15.49	15.10	14.63	13.57	13.45	12.70
+	2.492	1.15	2.22	Q3B7Z2;Q5	Oxysterol-binding protein 1	Osbp	1808	21	21	21	31.8	31.8	31.8	88.796	0.0000	222.77	36195000	327	16.61	15.93	16.65	16.38	15.23	15.35	15.62	14.77
+	3.244	1.15	2.22	Q6A4J8	Ubiquitin carboxyl-terminal hydrolase 7	Usp7	2503	32	32	32	40.4	40.4	40.4	128.47	0.0000	191.46	44414000	432	16.63	16.08	16.49	16.60	15.44	15.37	15.44	14.95
+	2.262	1.15	2.22	O35864	COP9 signalosome complex subunit 5	Cops5	408	15	15	15	49.4	49.4	49.4	37.548	0.0000	155.69	18361000	243	16.01	15.75	15.53	15.89	15.13	14.27	15.02	14.16
+	3.293	1.14	2.21	Q6ZQM8;Q6	UDP-glucuronosyltransferase 1-7C	Ugt1a7c	2702	26	26	17	56.1	56.1	38.2	59.757	0.0000	323.31	369020000	1036	20.23	19.67	20.04	19.98	18.89	18.49	19.08	18.89
	0.989	1.14	2.21	Q91WK5	Glycine cleavage system H protein, mitochondrial	Gcsh	4117	2	2	2	13.5	13.5	13.5	18.637	0.0000	20.437	2109200	28	13.89	14.40	15.25	14.23	13.16	14.48	11.99	13.58
	0.978	1.14	2.20	O35448	Lysosomal thioesterase PPT2	Ppt2	370	4	4	4	21.9	21.9	21.9	34.366	0.0000	74.372	9897900	42	14.35	14.75	14.02	13.96	13.73	13.60	13.78	11.43
	1.014	1.14	2.20	Q9QUK3	Protein CLN8	Cln8	5516	4	4	4	19.1	19.1	19.1	33.108	0.0000	53.759	3136800	35	14.55	14.33	14.73	15.25	14.50	13.03	14.47	12.31
+	3.391	1.13	2.19	Q8K3G9	DCC-interacting protein 13-beta	Appl2	3701	20	20	19	58.5	58.5	57.4	73.853	0.0000	323.31	20269000	194	16.88	16.58	17.18	16.48	15.71	15.60	15.67	15.59
+	2.049	1.13	2.19	A2AIL4	NADH dehydrogenase (ubiquinone) complex I, assembly factor 6	Ndufaf6	24	3	3	3	11.4	11.4	11.4	38.362	0.0000	8.9271	1714600	22	13.36	14.13	14.33	13.44	12.38	12.75	13.13	12.46
	0.850	1.13	2.19	Q5SVQ0	Histone acetyltransferase KAT7	Kat7	2151	4	4	4	7.2	7.2	7.2	70.641	0.0056	2.1789	5388400	15	15.80	15.31	15.44	15.30	15.43	13.87	15.34	12.67
+	2.772	1.13	2.19	Q99LG2	Transportin-2	Tnpo2	4427	11	4	4	17	8.1	8.1	100.46	0.0000	62.723	4910000	80	15.75	15.36	15.03	15.27	14.11	14.33	14.57	13.88
+	1.975	1.13	2.19	E9Q137		Tex264	227	13	13	13	47.2	47.2	47.2	33.589	0.0000	95.981	25909000	157	17.07	16.29	16.89	17.03	15.56	15.89	16.25	15.07
+	2.938	1.13	2.19	P09581;P05	Macrophage colony-stimulating factor 1 receptor	Csf1r	699	26	26	26	25.1	25.1	25.1	109.18	0.0000	266.35	137830000	532	19.04	18.66	19.19	18.75	18.08	17.59	17.98	17.47
+	1.322	1.13	2.19	Q8BHB4	WD repeat-containing protein 3	Wdr3	3027	10	10	10	16.6	16.6	16.6	105.77	0.0000	21.597	8994000	79	15.60	14.66	15.66	15.04	14.55	13.20	14.93	13.77
+	1.612	1.13	2.19	Q6ZPY7	Lysine-specific demethylase 3B	Kdm3b	2686	9	9	8	10.7	10.7	10	170.87	0.0000	47.317	7042300	53	15.58	15.49	14.96	15.35	15.08	14.26	14.18	13.35
+	2.969	1.13	2.18	Q8R001	Microtubule-associated protein RP/EB family member 2	Mapre2	3740	18	18	17	56.7	56.7	53.7	36.946	0.0000	175.28	37072000	304	16.94	16.57	17.26	16.90	15.71	15.84	16.12	15.50
+	2.877	1.13	2.18	Q99KR8	Plasma alpha-L-fucosidase	Fuca2	4393	5	5	5	14.8	14.8	14.8	53.645	0.0000	15.159	5111500	50	15.74	15.37	15.01	15.50	14.12	14.00	14.50	14.49
+	2.151	1.12	2.18	Q3URQ0	Testis-expressed sequence 10 protein	Tex10	2024	5	5	5	9.7	9.7	9.7	105.21	0.0000	27.785	2813500	35	14.38	14.08	14.04	13.56	12.99	12.43	13.47	12.68
+	1.200	1.12	2.18	Q61542	StAR-related lipid transfer protein 3	Stard3	2308	7	7	7	27.6	27.6	27.6	50.469	0.0000	88.531	13160000	142	15.50	15.20	15.27	15.22	14.91	12.82	14.87	14.11
+	1.455	1.12	2.18	P97300	Neuroplastin	Nptn	1618	5	5	5	16.1	16.1	16.1	44.373	0.0000	7.5884	4532000	32	15.63	15.37	15.53	14.23	14.74	14.11	13.89	13.52
+	3.127	1.12	2.18	Q8QZS1	3-hydroxyisobutyryl-CoA hydrolase, mitochondrial	Hibch	3733	14	14	14	46	46	46	43.037	0.0000	168.09	32394000	300	17.13	16.60	17.12	17.05	15.89	15.74	16.19	15.61
	0.786	1.12	2.18	Q6P2B1	Transportin-3	Tnpo3	2556	11	11	11	19.9	19.9	19.9	104.17	0.0000	78.495	7308400	58	15.88	15.69	15.59	14.57	15.11	14.69	15.04	12.40
+	2.037	1.12	2.17	O08800	Serpin B8	Serpinb8	301	14	14	14	53.7	53.7	53.7	42.15	0.0000	248.82	35383000	238	18.17	17.62	18.08	17.88	17.23	16.79	17.19	16.06
	0.886	1.12	2.17	Q6KAU4	Multivesicular body subunit 12B	Mvb12b	2521	5	5	5	31.5	31.5	31.5	35.411	0.0000	12.086	2920300	10	14.54	14.07	14.95	15.58	14.63	12.36	14.54	13.13
+	4.308	1.12	2.17	Q9DAW6	U4/U6 small nuclear ribonucleoprotein Prp4	Prpf4	5114	20	20	20	44.7	44.7	44.7	58.369	0.0000	63.004	29572000	285	16.13	15.77	16.01	15.74	14.79	14.91	14.82	14.65
	0.506	1.12	2.17	Q8K0G5	Protein TSSC1	Tssc1	3606	4	4	4	16.3	16.3	16.3	43.126	0.0000	23.597	8520000	124	15.17	15.30	15.42	15.34	15.10	15.35	15.16	11.15
	0.921	1.12	2.17	G5E897		Kdelc2	264	2	2	2	5.8	5.8	5.8	57.685	0.0000	21.409	3620000	31	15.16	15.25	15.15	15.21	14.97	12.89	15.29	13.13
	0.825	1.12	2.17	Q00422	GA-binding protein alpha chain	Gabpa	1696	2	2	2	7.5	7.5	7.5	51.344	0.0000	45.005	3786400	50	14.40	15.23	15.62	15.06	13.08	13.24	15.79	13.72
+	1.463	1.12	2.17	B8ZXI1	Queuine tRNA-ribosyltransferase subunit QTRTD1	Qtrtd1	91	5	5	5	17.3	17.3	17.3	46.288	0.0000	31.77	2213900	40	12.95	13.50	14.60	13.58	12.35	13.11	12.64	12.07
+	2.166	1.12	2.17	Q8BGZ4	Cell division cycle protein 23 homolog	Cdc23	3003	6	6	6	14.6	14.6	14.6	68.561	0.0000	58.936	4799400	52	15.56	14.67	15.82	15.15	14.48	14.02	13.99	14.25
+	1.661	1.12	2.17	Q8CFD4	Sorting nexin-8	Snx8	3480	13	13	13	36.6	36.6	36.6	52.058	0.0000	109.67	17582000	133	15.81	15.51	15.93	15.47	15.01	14.53	15.11	13.60
	0.773	1.11	2.17	Q99LS3	Phosphoserine phosphatase	Psph	4445	11	11	11	54.7	54.7	54.7	25.096	0.0000	19.508	12852000	113	15.33	15.44	15.78	15.57	14.47	15.42	15.37	12.40
+	3.739	1.11	2.17	Q9JI10	Serine/threonine-protein kinase 3;Serine/threonine-protein kinase 3 36k	Stk3	5381	18	13	13	37.6	31.4	31.4	56.855	0.0000	55.507	11051000	138	15.54	15.00	15.39	15.48	14.26	14.07	14.36	14.26
+	3.859	1.11	2.16	Q9CQQ7	ATP synthase F(0) complex subunit B1, mitochondrial	Atp5f1	4622	25	25	25	68.8	68.8	68.8	28.948	0.0000	320.8	470170000	884	20.45	19.96	20.29	20.38	19.09	19.17	19.36	19.02
+	3.018	1.11	2.16	Q61024	Asparagine synthetase [glutamine-hydrolyzing]	Asns	2238	25	25	25	52.4	52.4	52.4	64.282	0.0000	180.43	50549000	317	17.23	16.59	17.22	17.05	16.18	15.97	15.68	15.82
+	1.680	1.11	2.16	Q9ER69	Pre-mRNA-splicing regulator WTAP	Wtap	5295	4	4	4	15.2	15.2	15.2	44.176	0.0000	23.972	1873200	41	13.89	14.22	13.31	14.09	12.96	13.20	13.01	11.90
+	1.622	1.11	2.15	Q60759	Glutaryl-CoA dehydrogenase, mitochondrial	Gcdh	2202	9	9	9	37	37	37	48.605	0.0000	79.848	7499200	70	15.52	14.38	14.94	15.38	14.22	14.22	14.21	13.15

+	2.788	1.11	2.15	Q3TYX3	SET and MYND domain-containing protein 5	Smyd5	1890	5	5	5	13.2	13.2	13.2	47.095	0.0000	8.4097	5278000	68	15.73	14.76	15.31	15.11	14.15	14.16	14.03	14.13
	1.154	1.11	2.15	Q64669;P15	NAD(P)H dehydrogenase [quinone] 1	Nqo1	2456	13	13	13	46.7	46.7	46.7	30.959	0.0000	36.503	28066000	218	17.21	16.85	16.74	16.29	16.17	14.30	15.84	16.34
	0.623	1.11	2.15	A6H5Y3	Methionine synthase	Mtr	59	6	6	5	7.7	7.7	5.8	139.07	0.0000	24.006	5987600	24	15.55	15.45	16.15	14.97	15.34	12.53	16.13	13.69
	0.778	1.11	2.15	Q8R3I9	UDP-GlcNAc:betaGal beta-1,3-N-acetylglucosaminyltransferase 8	B3gnt8	3849	8	8	8	33.9	33.9	33.9	43.37	0.0000	28.86	7593000	63	15.52	15.46	16.47	15.67	15.41	16.03	14.22	13.03
	1.069	1.11	2.15	Q8CDJ3	Beclin 1-associated autophagy-related key regulator	Atg14	3461	3	3	3	8.1	8.1	8.1	55.388	0.0013	3.4959	3751300	20	15.79	15.18	14.07	15.67	14.28	13.71	13.30	14.99
+	2.157	1.10	2.15	Q80XR2	Calcium-transporting ATPase type 2C member 1	Atp2c1	2891	15	15	15	24.6	24.6	24.6	100.3	0.0000	46.407	14449000	137	15.38	15.04	15.81	15.67	14.42	13.89	14.93	14.23
	0.633	1.10	2.15	Q6PFY1	F-BAR and double SH3 domains protein 1	Fchsd1	2630	2	2	2	3.1	3.1	3.1	76.255	0.0032	2.5864	2241000	10	14.55	14.94	12.80	14.60	11.33	14.61	13.13	13.40
+	2.074	1.10	2.15	P43883	Perilipin-2	Plin2	1093	22	22	22	69.2	69.2	69.2	46.646	0.0000	283.67	20081000	224	16.21	15.83	15.95	15.64	15.08	14.92	15.17	14.04
	0.620	1.10	2.15	Q8CGA0	Protein phosphatase 1F	Ppm1f	3502	8	8	8	28.8	28.8	28.8	49.61	0.0000	48.23	7276000	60	15.03	14.34	15.08	14.45	15.33	12.16	14.73	12.25
+	2.495	1.10	2.15	Q3TLI0	Trafficking protein particle complex subunit 10	Trappc10	1860	11	11	11	13.9	13.9	13.9	141.49	0.0000	43.953	5511300	62	14.85	14.41	15.10	14.96	13.84	14.15	13.30	13.62
	1.132	1.10	2.14	Q80TI0	GRAM domain-containing protein 1B	Gramd1b	2805	2	2	2	4.7	4.7	4.7	85.354	0.0004	4.3144	1436800	3	14.04	13.89	14.17	13.64	11.79	12.30	13.23	14.02
+	1.911	1.10	2.14	B1AY13	Ubiquitin carboxyl-terminal hydrolase 24	Usp24	73	24	24	24	13.9	13.9	13.9	294	0.0000	110.64	18775000	193	15.33	14.65	15.46	15.13	14.58	13.49	14.35	13.74
+	3.741	1.10	2.14	A2BH40	AT-rich interactive domain-containing protein 1A	Arid1a	46	27	26	22	17.8	17.5	14.8	242.09	0.0000	118.54	22859000	242	15.98	15.63	16.16	15.89	14.71	14.85	15.02	14.67
+	1.711	1.10	2.14	Q6KAR6	Exocyst complex component 3	Exoc3	2520	18	18	18	24.2	24.2	24.2	86.454	0.0000	91.653	15359000	157	15.56	15.34	15.35	15.83	14.56	14.65	14.99	13.48
+	2.465	1.10	2.14	Q91X20	Set1/Ash2 histone methyltransferase complex subunit ASH2	Ash2l	4132	10	10	10	20.1	20.1	20.1	68.249	0.0000	24.399	11386000	71	16.96	16.48	17.01	16.76	15.18	15.97	15.60	16.07
+	1.525	1.10	2.14	Q78XF5	Oligosaccharyltransferase complex subunit OSTC	Ostc	2730	3	3	3	18.1	18.1	18.1	16.815	0.0000	17.611	23618000	127	17.31	16.79	16.19	16.74	16.14	15.16	16.25	15.07
	0.947	1.09	2.14	Q9Z120	tRNA (guanine-N(7)-)-methyltransferase	Mettl1	5796	2	2	2	10.8	10.8	10.8	30.603	0.0000	6.5996	890420	19	13.76	12.87	12.25	14.19	12.78	11.01	12.51	12.39
+	3.073	1.09	2.14	Q99KN2	Probable cytosolic iron-sulfur protein assembly protein CIAO1	Ciao1	4385	9	9	9	32.2	32.2	32.2	37.632	0.0000	36.998	21508000	145	16.35	15.97	16.51	16.27	15.52	14.98	15.29	14.94
	0.947	1.09	2.13	Q922M3	BTB/POZ domain-containing adapter for CUL3-mediated RhoA degrada	Kctd10	4264	2	2	2	9.8	9.8	9.8	35.701	0.0000	22.504	2060600	39	14.66	13.71	14.26	14.21	13.03	14.18	13.66	11.61
	1.038	1.09	2.13	Q99NB1	Acetyl-coenzyme A synthetase 2-like, mitochondrial	Acss1	4495	8	8	8	17	17	17	74.622	0.0000	56.464	6917400	112	14.79	14.51	15.09	14.94	13.10	12.59	14.79	14.48
+	2.518	1.09	2.13	Q8VI75	Importin-4	Ipo4	4030	16	16	15	19.3	19.3	19.3	119.27	0.0000	125.99	25319000	232	17.33	16.58	17.10	17.18	15.94	15.54	16.32	16.01
+	2.467	1.09	2.13	Q8C5N3	Pre-mRNA-splicing factor CWC22 homolog	Cwc22	3399	6	6	6	7.5	7.5	7.5	104.77	0.0000	14.662	7189000	57	15.96	15.32	16.19	15.60	14.66	14.65	15.01	14.37
+	2.468	1.09	2.13	Q8BLN5	Lanosterol synthase	Lss	3120	22	22	21	36.4	36.4	35.1	83.14	0.0000	75.892	36188000	286	16.76	16.39	16.50	16.37	15.94	15.17	15.57	14.98
	0.891	1.09	2.12	P11609;P11	Antigen-presenting glycoprotein CD1d1;Antigen-presenting glycoprotein	Cd1d1;Cd1d2	747	2	2	2	6.8	6.8	6.8	38.554	0.0000	8.8268	2101100	39	14.20	13.90	13.69	13.84	12.12	12.21	14.64	12.32
+	2.657	1.09	2.12	Q9QY36;Q3	N-alpha-acetyltransferase 10;N-alpha-acetyltransferase 11	Naa10;Naa11	5561	8	8	8	39.1	39.1	39.1	26.519	0.0000	60.462	20001000	170	16.99	16.35	16.82	16.78	15.85	15.44	16.00	15.31
+	2.146	1.08	2.12	B2RY04	Dedicator of cytokinesis protein 5	Dock5	88	19	18	17	15.7	15.1	14.2	214.43	0.0000	80.52	10946000	109	15.28	14.89	15.71	15.65	13.94	14.28	14.84	14.14
+	1.890	1.08	2.12	Q6PDG8	Vacuolar fusion protein MON1 homolog A	Mon1a	2612	4	4	4	11.7	11.7	11.7	62.131	0.0000	33.898	2517100	33	14.69	14.27	13.57	14.23	13.41	12.81	13.51	12.72
+	2.643	1.08	2.11	Q9R0E1	Procollagen-lysine,2-oxoglutarate 5-dioxygenase 3	Plod3	5623	22	22	22	38.7	38.7	38.7	84.921	0.0000	149.01	27642000	261	16.36	16.49	16.18	16.10	15.57	15.06	15.46	14.72
+	2.209	1.08	2.11	Q8K1A5	Transmembrane protein 41B	Tmem41b	3620	2	2	2	9.6	9.6	9.6	32.429	0.0000	30.706	9615100	59	16.33	16.06	16.50	16.74	15.18	15.22	15.96	14.96
+	1.768	1.08	2.11	Q8CIF6	SID1 transmembrane family member 2	Sidt2	3552	3	3	3	4.1	4.1	4.1	94.5	0.0000	7.7806	1885400	37	13.98	13.66	14.38	14.41	13.59	13.12	13.14	12.25
+	3.526	1.08	2.11	Q9ER39	Torsin-1A	Tor1a	5293	9	9	9	33	33	33	37.829	0.0000	38.163	6817400	72	14.97	14.92	14.72	14.87	14.13	13.60	13.55	13.89
+	1.370	1.08	2.11	P28843	Dipeptidyl peptidase 4;Dipeptidyl peptidase 4 membrane form;Dipeptidy	Dpp4	963	10	10	10	13.7	13.7	13.7	87.436	0.0000	28.209	7193100	63	14.23	14.70	15.76	15.03	13.11	13.82	14.08	14.39
+	1.529	1.08	2.11	Q5F2E8	Serine/threonine-protein kinase TAO1	Taok1	2104	21	14	14	20.2	15.8	15.8	116.05	0.0000	36.162	7653200	90	14.72	14.62	14.69	15.45	13.85	12.89	14.00	14.43
+	1.522	1.08	2.11	Q9DCV4	Regulator of microtubule dynamics protein 1	Rmdn1	5234	5	5	5	20.7	20.7	20.7	35	0.0008	4.0844	3914300	38	15.37	15.28	13.73	14.83	13.54	13.81	13.83	13.71
	1.138	1.08	2.11	Q8CIM5	G-protein coupled receptor 84	Gpr84	3559	3	3	3	11.6	11.6	11.6	43.717	0.0026	2.9467	2096500	17	14.79	14.72	14.93	15.24	14.74	12.56	14.41	13.66
+	2.689	1.08	2.11	Q8K274	Ketosamine-3-kinase	Fn3krp	3647	6	6	6	26.9	26.9	26.9	34.468	0.0000	29.815	7143500	88	15.73	15.37	15.62	15.51	14.32	14.84	14.75	14.02
+	1.565	1.07	2.10	Q9D0B0	Serine/arginine-rich splicing factor 9	Srsf9	4855	8	7	6	38.7	35.6	28.8	25.661	0.0000	6.5606	4394100	16	13.42	14.78	13.80	14.26	12.87	12.57	13.62	12.91
+	2.769	1.07	2.10	Q80XN0	D-beta-hydroxybutyrate dehydrogenase, mitochondrial	Bdh1	2887	11	11	11	40.5	40.5	40.5	38.299	0.0000	61.064	16512000	101	16.60	16.41	16.43	16.27	15.70	15.33	14.84	15.55
+	2.967	1.07	2.10	P97390	Vacuolar protein sorting-associated protein 45	Vps45	1643	16	16	16	33	33	33	65.052	0.0000	125.22	24545000	279	16.21	15.82	15.84	15.82	15.24	14.71	14.93	14.52
	0.546	1.07	2.10	O70456	14-3-3 protein sigma	Sfn	501	16	11	11	52	45.2	45.2	27.706	0.0000	22.246	89331000	82	17.70	17.85	19.02	18.56	17.77	15.33	19.29	16.46
	0.779	1.07	2.10	P70280	Vesicle-associated membrane protein 7	Vamp7	1545	7	7	7	41.4	41.4	41.4	24.967	0.0000	174.8	15858000	150	16.12	15.66	15.98	16.09	15.72	15.03	15.88	12.96
	1.065	1.07	2.10	Q9D5V6	Synapse-associated protein 1	Syap1	4987	4	4	4	13.7	13.7	13.7	41.349	0.0000	80.093	3073100	56	14.12	14.48	13.99	14.03	12.27	14.58	12.78	12.72
	0.715	1.07	2.10	Q9DCJ5	NADH dehydrogenase [ubiquinone] 1 alpha subcomplex subunit 8	Ndufa8	5217	5	5	5	45.3	45.3	45.3	19.992	0.0000	14.21	7469100	31	15.40	15.67	15.28	15.69	15.12	12.84	16.08	13.73
+	3.313	1.07	2.09	Q8R5H1;Q9	Ubiquitin carboxyl-terminal hydrolase 15	Usp15	3889	35	35	34	44.1	44.1	43.2	112.32	0.0000	283.76	45331000	424	16.89	16.79	16.65	16.70	16.07	15.51	15.78	15.42
+	2.891	1.07	2.09	P51829;P84	Adenylate cyclase type 7	Adcy7	1203	8	8	8	11.6	11.6	11.6	122.71	0.0000	65.04	9053400	134	15.36	14.61	15.06	14.73	14.06	13.87	13.90	13.66
+	1.636	1.07	2.09	Q9ERE3	Serine/threonine-protein kinase Sgk3	Sgk3	5305	7	7	7	20.6	20.6	20.6	57.145	0.0000	36.062	3237100	46	14.38	14.29	14.72	13.31	12.95	13.55	12.72	13.22
	0.959	1.07	2.09	Q8C407	Protein YIPF4	Yipf4	3384	4	4	4	28.9	28.9	28.9	27.283	0.0000	17.617	8641300	67	15.36	14.96	15.75	15.61	14.59	12.77	15.17	14.89
+	2.911	1.06	2.09	Q2NL51	Glycogen synthase kinase-3 alpha	Gsk3a	1799	12	12	9	48.6	48.6	40.6	51.66	0.0000	115.26	11215000	105	15.33	15.33	15.04	15.13	14.30	13.90	14.55	13.83
	0.814	1.06	2.09	Q60949	TBC1 domain family member 1	Tbc1d1	2226	8	8	8	6.5	6.5	6.5	142.02	0.0000	14.327	4390700	33	13.92	14.15	15.06	15.27	13.76	14.79	12.08	13.50
+	2.168	1.06	2.09	Q3TIR3	Synembryn-A	Ric8a	1849	12	12	11	29.6	29.6	27	59.846	0.0000	69.271	12337000	74	15.90	15.61	16.49	16.51	14.85	15.16	15.41	14.83
+	1.645	1.06	2.09	Q9D3S3	Sorting nexin-29	Snx29	4969	8	8	8	14.5	14.5	14.5	91.386	0.0000	7.2395	2373800	17	13.94	13.48	14.12	13.45	13.49	12.40	12.07	12.77
	1.167	1.06	2.09	Q99J93	Interferon-induced transmembrane protein 2	Ifitm2	4333	3	2	2	17.4	11.8	11.8	15.743	0.0000	16.2	5092600	70	13.34	14.58	15.31	14.57	12.91	13.01	13.87	13.77
+	1.443	1.06	2.09	E9Q6P5		Ttc7b	243	3	3	3	6.6	6.6	6.6	94.202	0.0000	13.102	1752100	19	14.90	14.64	14.68	13.63	13.07	14.17	13.41	12.95
	1.097	1.06	2.09	O88668	Protein CREG1	Creg1	557	7	7	7	33.6	33.6	33.6	24.452	0.0000	185.75	128620000	348	18.96	18.89	17.80	17.96	18.06	16.30	17.90	17.11
	1.223	1.06	2.09	Q7TSI1	Pleckstrin homology domain-containing family M member 1	Plekhm1	2785	5	5	5	6.8	6.8	6.8	118.53	0.0000	20.721	4484200	66	14.47	13.50	14.75	14.40	13.35	12.91	14.18	12.43
+	2.933	1.06	2.09	O08599	Syntaxin-binding protein 1	Stxbp1	279	15	15	15	33.2	33.2	33.2	67.568	0.0000	71.485	16453000	151	16.36	16.00	15.81	16.36	15.35	15.18	15.00	14.78
	1.006	1.06	2.08	P70704;P98	Phospholipid-transporting ATPase IA	Atp8a1	1589	11	11	11	12.8	12.8	12.8	131.41	0.0000	17.201	8451900	58	14.76	14.31	15.58	15.14	14.11	14.67	14.24	12.52
+	3.205	1.06	2.08	Q9DCD2	Pre-mRNA-splicing factor SYF1	Xab2	5208	21	21	21	29.5	29.5	29.5	99.986	0.0000	84.03	19227000	178	15.55	15.21	15.84	15.53	14.32	14.53	14.74	14.31
+	1.996	1.06	2.08	P14069	Protein S100-A6	S100a6	771	11	11	11	97.8	97.8	97.8	10.051	0.0000	82.254	36216000	173	19.05	18.83	18.03	18.17	17.71	17.71	17.24	17.18
+	2.059	1.06	2.08	Q61165	Sodium/hydrogen exchanger 1	Slc9a1	2265	8	8	8	15.7	15.7	15.7	91.467	0.0000	95.407	6639600	100	15.52	15.38	15.85	15.21	14.10	14.06	15.10	14.49
+	1.780	1.05	2.08	O35904	Phosphatidylinositol 4,5-bisphosphate 3-kinase catalytic subunit delta is	Pik3cd	412	12	12	12	16.8	16.8	16.8	119.71	0.0000	64.367	5738600	63	14.97	14.56	14.34	14.24	13.44	14.26	13.06	13.13
+	2.368	1.05	2.08	P97742	Carnitine O-palmitoyltransferase 1, liver isoform	Cpt1a	1666	29	29	28	42.6	42.6	41.1	88.25	0.0000	309.87	77048000	482	17.72	17.16	17.76	17.74	16.41	16.09	16.93	16.73
+	3.964	1.05	2.07	Q61792;Q9I	LIM and SH3 domain protein 1	Lasp1	2335	25	25	25	62	62	62	29.994	0.0000	315.31	456020000	1129	20.13	19.84	19.77	19.99	19.04	18.84	18.98	18.66
+	2.554	1.05	2.07	Q8VBT0	Thioredoxin-related transmembrane protein 1	Tmx1	3894	9	9	9	24.8	24.8	24.8	31.395	0.0000	35.203	39566000	251	17.18	16.77	17.30	17.56	16.19	15.93	16.53	15.96
	0.770	1.05	2.07	Q9CQ02	COMM domain-containing protein 4	Commd4	4550	6	6	6	39.2	39.2	39.2	21.86	0.0000	57.309	10833000	61	16.03	15.37	15.88	16.02	15.88	12.90	14.87	15.44
+	2.966	1.05	2.07	P11152	Lipoprotein lipase	Lpl	739	7	7	7	19	19	19	53.109	0.0000	211.62	22688000	204	16.22	16.38	16.21	16.27	15.36	15.36	15.45	14.70
+	3.447	1.05	2.07	P30993	C5a anaphylatoxin chemotactic receptor 1	C5ar1	981	4	4	4	7.4	7.4	7.4	39.023	0.0000	83.799	33417000	133	17.85	17.30	17.53	17.37	16.57	16.51	16.54	16.23
	1.200	1.04	2.06	Q99J31	Oligophrenin-1	Ophn1	4321	4	4	4	6.6	6.6	6.6	91.984	0.0002	5.8736	1901600	20	14.12	12.92	14.00	14.52	11.95	13.11	13.33	13.00
+	1.982	1.04	2.06	Q9DCZ1	GMP reductase 1	Gmpr	5237	8	6	6	27.8	21.7	21.7	37.482	0.0000	34.573	7635600	48	15.67	15.74	15.68	14.97	15.11	14.21	14.17	14.39
+	1.597	1.04	2.06	A2ADA5	tRNA pseudouridine synthase-like 1	Pusl1	17	4	4	4	14.8	14.8	14.8	31.992	0.0000	32.243	3414600	39	14.40	13.42	14.62	14.31	13.47	13.01	13.55	12.55
+	1.876	1.04	2.06	Q64518	Sarcoplasmic/endoplasmic reticulum calcium ATPase 3	Atp2a3	2451	22	16	16	26.7	21.1	21.1	109.53	0.0000	99.021	27788000	254	15.95	15.43	16.35	16.27	14.97	14.75	15.56	14.56
+	2.826	1.04	2.06	Q9CYN2	Signal peptidase complex subunit 2	Spcs2	4803	16	16	16	53.5	53.5	53.5	24.977	0.0000	222.82	77201000	424	17.49	17.31	17.56	17.58	16.50	16.41	16.88	16.01
+	2.401	1.04	2.06	Q8C1E7	Transmembrane protein 120A	Tmem120a	3365	7	7	6	24.2	24.2	20.1	40.751	0.0000	51.563	16569000	163	15.81	15.57	15.81	16.04	14.72	14.34	15.34	14.68
+	3.023	1.04	2.05	Q99KK9	Probable histidine--tRNA ligase, mitochondrial	Hars2	4383	15	11	11	33.3	27.7	27.7	56.985	0.0000	27.873	12767000	138	15.70	15.41	15.55	15.52	14.96	14.30	14.50	14.26
	0.770	1.04	2.05	Q6P8M1	Putative deoxyribonuclease TATDN1	Tatdn1	2584	5	5	5	19.7	19.7	19.7	33.365	0.0000	7.5268	4054400	35	14.68	14.36	14.06	14.43	14.20	12.05	14.71	12.42
	0.946	1.04	2.05	Q91WK1	SPRY domain-containing protein 4	Spryd4	4115	5	5	5	27.1	27.1	27.1	23.275	0.0004	5.1022	1957300	18	14.53	13.44	13.26	14.24	13.57	11.46	13.11	13.19
+	2.078	1.04	2.05	Q5KU39	Vacuolar protein sorting-associated protein 41 homolog	Vps41	2112	19	19	19	25.2	25.2	25.2	98.601	0.0000	61.053	29243000	254	17.61	17.02	17.67	17.49	16.52	16.64	16.75	15.76
+	1.955	1.04	2.05	A3KGB4	TBC1 domain family member 8B	Tbc1d8b	51	10	10	9	14.7	14.7	13.7	127.89	0.0000	44.41	6949100	90	15.02	14.37	15.16	15.13	13.43	13.61	14.35	14.14
+	1.640	1.03	2.05	Q61122	NGFI-A-binding protein 1	Nab1	2254	4	3	3	10.1	8.2	8.2	54.009	0.0000	7.6503	2303000	37	14.54	14.43	14.57	14.25	12.44	13.88	13.82	13.51
+	2.323	1.03	2.04	Q8BMG7	Rab3 GTPase-activating protein non-catalytic subunit	Rab3gap2	3136	26	26	26	29.6	29.6	29.6	152.53	0.0000	136.12	26868000	343	16.00	15.26	16.07	15.78	14.88	14.31	14.97	14.82
+	3.116	1.03	2.04	Q3UMR5	Calcium uniporter protein, mitochondrial	Mcu	2001	14	14	14	40	40	40	39.681	0.0000	109.93	24927000	189	16.52	15.81	16.19	16.28	15.32	14.97	15.19	15.20
	0.926	1.03	2.04	Q9CWI3	BRCA2 and CDKN1A-interacting protein	Bccip	4716	6	6	6	33.5	33.5	33.5	35.942	0.0000	33.651	5238700	54	14.90	15.02	15.32	15.32	14.36	14.71	14.89	12.48
+	2.004	1.03	2.04	Q8C7K6	Prenylcysteine oxidase-like	Pcyox1l	3412	6	6	6	16	16	16	54.874	0.0000	40.081	9575600	111	15.39	14.84	15.63	15.21	14.29	14.27	13.65	14.74
	0.594	1.03	2.04	Q9QZ15	C-type lectin domain family 4 member A	Clec4a	5585	3	3	3	15.5	15.5	15.5	27.323	0.0000	56.048	6187300	46	15.20	15.68	14.57	14.97	15.03	15.78	12.85	12.65
	1.330	1.03	2.04	Q6PCP5	Mitochondrial fission factor	Mff	2606	10	10	10	56	56	56	32.931	0.0000	133.21	14398000	104	16.32	16.11	16.35	15.63	15.77	14.37	15.68	14.49
	1.025	1.02	2.03	Q8BTY2	Sodium bicarbonate cotransporter 3	Slc4a7	3212	5	5	5	9.4	9.4	9.4	116.51	0.0000	45.826	5712300	60	15.08	15.03	15.39	15.05	15.13	12.80	14.66	13.87
+	2.402	1.02	2.03	Q922R0	cAMP-dependent protein kinase catalytic subunit PRKX	Prkx	4273	5	5	5	18.6	18.6	18.6	40.466	0.0000	13.585	3372300	42	14.36	14.49	14.70	14.58	13.76	13.99	13.09	13.21
	0.627	1.02	2.03	Q9CWN7	CCR4-NOT transcription complex subunit 11	Cnot11	4722	3	3	3	12.1	12.1	12.1	54.959	0.0025	2.9065	3035200	35	13.66	14.13	15.71	15.27	14.37	14.83	12.07	13.42
	0.982	1.02	2.03	E9Q6J5		Bod1l	242	8	8	8	3.3	3.3	3.3	327.45	0.0000	23.852	15061000	42	15.87	15.95	17.91	16.55	15.96	14.87	15.89	15.46
+	2.024	1.02	2.03	Q8BTX9	Inactive hydroxysteroid dehydrogenase-like protein 1	Hsdl1	3211	11	11	11	43	43	43	36.867	0.0000	118.28	28130000	170	16.16	16.24	16.06	16.44	15.76	14.52	15.37	15.17
	0.604	1.02	2.03	Q99P65	Equilibrative nucleoside transporter 3	Slc29a3	4501	4	4	4	11.8	11.8	11.8	51.719	0.0000	42.057	14142000	116	16.47	15.79	16.87	16.85	15.53	13.32	16.39	16.66
	0.818	1.02	2.03	B9EJ80		Pdzd8	92	3	3	3	4.2	4.2	4.2	127.74	0.0000	22.431	1827600	19	12.87	13.95	14.26	14.57	11.92	12.33	14.19	13.12
	1.413	1.02	2.02	O70161	Phosphatidylinositol 4-phosphate 5-kinase type-1 gamma	Pip5k1c	474	8	8	7	18.9	18.9	16.8	72.407	0.0000	51.756	8007100	88	15.85	15.60	15.87	15.83	15.01	13.66	15.38	15.03
	0.622	1.02	2.02	Q9ERS5	Pleckstrin homology domain-containing family A member 2	Plekha2	5318	8	8	8	24.7	24.7	24.7	47.379	0.0000	108.13	6224200	78	15.28	14.95	15.23	14.49	14.92	14.90	14.33	11.75
+	1.582	1.01	2.02	P70196	TNF receptor-associated factor 6	Traf6	1534	3	3	3	7	7	7	60.069	0.0000	8.3976	2129200	22	14.30	13.00	13.92	13.61	12.96	12.19	13.11	12.52
	1.020	1.01	2.02	Q62048	Astrocytic phosphoprotein PEA-15	Pea15	2349	5	5	5	48.5	48.5	48.5	15.054	0.0000	48.818	4701700	52	14.96	15.46	13.96	14.96	13.52	14.78	12.89	14.09
	0.956	1.01	2.02	O88597	Beclin-1	Becn1	549	6	6	6	20.3	20.3	20.3	51.588	0.0000	17.275	6261800	54	15.12	14.47	14.92	14.82	14.52	13.60	14.74	12.44
	1.268	1.01	2.02	B7ZMP1	Probable Xaa-Pro aminopeptidase 3	Xpnpep3	90	5	5	5	20	20	20	56.677	0.0000	35.153	2263700	12	13.88	13.80	15.30	14.98	13.53	12.94	13.78	13.65
	0.609	1.01	2.02	P33609	DNA polymerase alpha catalytic subunit	Pola1	1002	10	10	10	14.2	14.2	14.2	167.34	0.0000	26.25	6739500	51	14.63	14.27	15.91	15.86	14.67	15.02	14.77	12.17
	1.289	1.01	2.01	P36993	Protein phosphatase 1B	Ppm1b	1045	12	12	9	33.8	33.8	25.9	42.795	0.0000	20.801	8725800	95	15.58	15.76	15.20	15.60	14.33	13.56	15.47	14.75
	0.770	1.01	2.01	Q99ME2	WD repeat-containing protein 6	Wdr6	4463	10	10	10	14	14	14	121.9	0.0000	13.346	4091900	31	14.07	13.93	14.86	14.07	14.08	11.55	14.18	13.10
+	2.771	1.01	2.01	P09671	Superoxide dismutase [Mn], mitochondrial	Sod2	701	12	12	12	46.4	46.4	46.4	24.603	0.0000	141.07	84057000	238	18.50	18.84	18.50	19.02	17.86	17.36	17.97	17.65
+	2.776	1.01	2.01	Q9CRD0	OCIA domain-containing protein 1	Ociad1	4692	17	17	17	75.3	75.3	75.3	27.61	0.0000	323.31	76216000	289	17.84	17.89	17.77	17.97	16.66	17.02	17.29	16.48
+	3.246	1.01	2.01	Q8BH58	TIP41-like protein	Tiprl	3014	13	13	13	60.1	60.1	60.1	31.253	0.0000	203.02	28982000	209	17.75	17.42	17.92	17.68	16.71	16.38	16.90	16.74
+	2.185	1.01	2.01	P11688	Integrin alpha-5;Integrin alpha-5 heavy chain;Integrin alpha-5 light chain	Itga5	749	16	16	16	18	18	18	115.04	0.0000	217.49	28433000	218	16.42	16.20	17.05	16.57	15.54	15.46	16.01	15.20
+	1.725	1.01	2.01	Q00558	Factor VIII intron 22 protein	F8a1	1698	5	5	5	21.1	21.1	21.1	40.489	0.0000	44.363	16347000	112	16.68	16.33	16.62	16.59	15.77	16.04	15.72	14.66
+	2.076	1.01	2.01	Q8R349	Cell division cycle protein 16 homolog	Cdc16	3836	5	5	5	9.4	9.4	9.4	71.459	0.0000	10.569	2712000	30	14.04	13.68	14.53	14.83	13.23	13.39	13.27	13.17
+	2.919	1.00	2.01	Q9DBL1	Short/branched chain specific acyl-CoA dehydrogenase, mitochondrial	Acadsb	5159	20	20	20	54.4	54.4	54.4	47.874	0.0000	323.31	48374000	374	17.44	16.92	17.44	17.36	16.45	16.08	16.54	16.08
+	2.216	1.00	2.00	Q80SY5	Pre-mRNA-splicing factor 38B	Prpf38b	2797	7	7	7	14.2	14.2	14.2	63.752	0.0000	91.736	10474000	82	16.95	15.90	16.41	16.65	15.64	15.62	15.42	15.23
+	2.583	1.00	2.00	Q8BG67;Q6	Protein EFR3 homolog A	Efr3a	2965	11	11	11	19.5	19.5	19.5	92.612	0.0000	80.265	11222000	154	15.13	14.82	15.58	15.43	14.02	14.11	14.54	14.29
+	2.938	1.00	2.00	Q3V1L4	Cytosolic purine 5-nucleotidase	Nt5c2	2049	24	24	24	52.5	52.5	52.5	64.808	0.0000	199.56	41265000	344	16.97	16.49	16.46	16.72	15.80	15.38	15.93	15.52
	0.742	1.00	2.00	Q8BUV8	Protein GPR107	Gpr107	3231	3	3	3	5.3	5.3	5.3	62.055	0.0000	9.6631	1892600	28	13.87	13.52	14.27	13.95	13.18	13.33	11.05	14.04
	1.166	1.00	2.00	O70309	Integrin beta-5	Itgb5	485	10	10	10	15.5	15.5	15.5	87.908	0.0000	72.823	25078000	118	16.55	15.87	17.45	16.58	16.00	15.25	14.93	16.26
+	1.863	1.00	2.00	Q8BKT3	GC-rich sequence DNA-binding factor 2	Gcfc2	3105	5	5	5	9.8	9.8	9.8	87.441	0.0000	13.178	2762000	19	14.41	14.55	14.85	14.85	13.68	13.47	14.39	13.12

	1.375	1.00	2.00	Q64430	Copper-transporting ATPase 1	Atp7a	2440	10	10	10	10.9	10.9	10.9	161.96	0.0000	104.92	8133400	147	15.18	14.80	15.76	15.57	14.60	13.49	15.02	14.21
	0.660	1.00	2.00	O88942	Nuclear factor of activated T-cells, cytoplasmic 1	Nfatc1	586	4	4	4	14.2	14.2	14.2	77.832	0.0000	70.872	2228500	48	13.95	13.77	13.69	14.20	11.97	11.97	12.68	15.00
+	2.802	0.99	1.99	Q9WUN2	Serine/threonine-protein kinase TBK1	Tbk1	5718	26	26	26	44	44	44	83.424	0.0000	291.95	50592000	365	17.27	16.93	17.57	17.47	16.28	16.21	16.65	16.13
+	2.307	0.99	1.99	Q61263	Sterol O-acyltransferase 1	Soat1	2287	11	11	11	25.2	25.2	25.2	63.798	0.0000	323.31	213280000	490	19.08	18.50	19.31	19.08	17.93	17.81	18.44	17.80
	1.261	0.99	1.99	Q9CXY1	Transmembrane protein 175	Tmem175	4773	3	3	3	7.8	7.8	7.8	55.577	0.0000	44.838	2222500	39	13.03	12.93	14.02	13.99	12.73	12.67	12.96	11.64
+	2.643	0.99	1.99	P31750	RAC-alpha serine/threonine-protein kinase	Akt1	989	19	19	16	47.7	47.7	37.9	55.707	0.0000	151.04	39811000	288	16.87	16.54	16.63	16.96	15.91	15.81	16.05	15.27
+	1.893	0.99	1.99	P98195	Probable phospholipid-transporting ATPase IIB	Atp9b	1689	10	10	10	11.8	11.8	11.8	129.02	0.0000	24.473	8462600	67	15.61	14.87	16.12	15.83	14.55	14.38	14.84	14.69
	1.057	0.99	1.99	Q8BX94	Oxysterol-binding protein-related protein 2	Osbpl2	3282	9	9	9	31.6	31.6	31.6	55.384	0.0000	58.765	5104800	72	14.03	13.30	13.69	14.42	12.29	12.66	12.41	14.12
+	1.916	0.99	1.99	Q5SW19	Clustered mitochondria protein homolog	Cluh	2153	17	17	17	17.3	17.3	17.3	148.07	0.0000	43.816	12518000	129	15.35	14.70	15.23	15.22	14.75	14.08	14.11	13.58
+	2.337	0.99	1.99	P70202	Latexin	Lxn	1535	4	4	4	25.7	25.7	25.7	25.492	0.0000	42.938	8686400	86	15.66	15.63	15.86	15.62	14.19	14.49	15.14	14.98
+	2.233	0.99	1.99	Q91VB4	Hermansky-Pudlak syndrome 3 protein homolog	Hps3	4054	5	5	5	6.1	6.1	6.1	113.18	0.0000	10.764	3606300	70	14.22	13.46	14.21	14.24	13.22	12.66	13.30	12.98
	1.003	0.99	1.99	Q99L20		Gstt3	4404	9	9	9	45.2	45.2	45.2	27.403	0.0000	67.903	17996000	68	17.47	16.58	17.51	16.13	16.14	16.19	16.56	14.83
+	3.123	0.99	1.99	Q9R0P6	Signal peptidase complex catalytic subunit SEC11A	Sec11a	5634	11	11	11	35.2	35.2	35.2	20.626	0.0000	76.865	64196000	401	18.35	17.81	18.18	18.40	17.27	16.95	17.35	17.22
+	1.778	0.99	1.99	Q60967	Bifunctional 3-phosphoadenosine 5-phosphosulfate synthase 1;Sulfate a	Papss1	2230	14	13	13	32.4	30.1	30.1	70.793	0.0000	118.53	19520000	232	16.66	15.89	15.56	15.82	15.44	15.08	14.91	14.54
+	1.784	0.99	1.99	Q8BY87	Ubiquitin carboxyl-terminal hydrolase 47	Usp47	3294	20	20	20	22.6	22.6	22.6	157.45	0.0000	51.601	18988000	181	16.04	15.49	16.38	15.98	15.15	15.19	15.31	14.28
+	3.736	0.99	1.98	Q11011	Puromycin-sensitive aminopeptidase	Npepps	1782	50	50	50	56.7	56.7	56.7	103.32	0.0000	323.31	144400000	1046	17.46	17.32	17.67	17.60	16.48	16.41	16.80	16.41
	1.095	0.99	1.98	Q8CIM3	D-2-hydroxyglutarate dehydrogenase, mitochondrial	D2hgdh	3558	5	5	5	15.7	15.7	15.7	58.575	0.0000	19.516	2763600	19	14.79	15.59	14.84	15.31	13.45	15.39	13.93	13.82
	0.951	0.99	1.98	Q9QZB9	Dynactin subunit 5	Dctn5	5590	5	5	5	18.7	18.7	18.7	20.14	0.0000	55.262	5526100	47	15.93	15.32	15.44	15.95	14.60	15.39	15.43	13.28
	0.928	0.99	1.98	B1AVH7	TBC1 domain family member 2A	Tbc1d2	69	19	19	19	24.9	24.9	24.9	104.26	0.0000	41.354	16057000	115	15.89	15.35	16.07	16.44	13.79	14.51	15.91	15.61
	0.279	0.98	1.98	Q8C142	Low density lipoprotein receptor adapter protein 1	Ldlrap1	3351	4	4	4	21.1	21.1	21.1	33.975	0.0000	10.806	9718100	39	16.74	16.57	13.04	16.61	16.39	13.01	17.08	12.55
	1.068	0.98	1.98	Q9DBH0	NEDD4-like E3 ubiquitin-protein ligase WWP2	Wwp2	5156	4	3	3	6.3	5.4	5.4	98.76	0.0000	29.72	1723200	35	14.10	13.74	13.68	12.90	12.75	11.45	12.97	13.31
+	3.296	0.98	1.98	P12815	Programmed cell death protein 6	Pdcd6	760	10	10	10	72.3	72.3	72.3	21.867	0.0000	232.34	55287000	297	17.43	16.93	17.37	17.39	16.38	16.05	16.42	16.35
+	1.936	0.98	1.98	Q8C0P5	Coronin-2A	Coro2a	3346	14	14	13	28.1	28.1	26.7	59.573	0.0000	37.799	43670000	124	19.08	17.92	18.40	18.22	17.25	17.62	17.17	17.64
	1.474	0.98	1.98	Q80WC7	Arf-GAP domain and FG repeat-containing protein 2	Agfg2	2860	7	5	5	20	17.7	17.7	48.967	0.0000	34.835	10943000	109	16.19	15.91	15.09	15.23	15.08	14.30	14.99	14.12
+	3.080	0.98	1.97	P18181	CD48 antigen	Cd48	835	8	8	8	33.8	33.8	33.8	27.383	0.0000	35.306	25680000	198	16.22	16.50	16.29	16.45	15.54	15.16	15.71	15.12
+	4.067	0.98	1.97	Q91VJ4	Serine/threonine-protein kinase 38	Stk38	4065	23	23	20	46	46	41.9	54.174	0.0000	134.86	44681000	468	16.26	16.00	16.18	16.12	15.11	15.26	15.34	14.93
	1.408	0.98	1.97	Q9R1T4	Septin-6	Sep.06	5664	18	9	8	38.2	21.9	18.7	49.619	0.0000	15.043	4177100	59	14.80	14.34	13.66	13.71	13.57	13.51	12.47	13.05
	0.704	0.98	1.97	Q9CQS4	Solute carrier family 25 member 46	Slc25a46	4628	5	5	5	23.7	23.7	23.7	46.224	0.0000	32.431	4435400	40	14.71	14.47	14.55	14.52	14.29	12.48	15.14	12.44
	0.624	0.98	1.97	P97304	DNA-directed RNA polymerases I and III subunit RPAC2	Polr1d	1619	2	2	2	17.3	17.3	17.3	15.072	0.0008	4.2065	2367100	20	13.60	14.13	14.36	14.24	13.78	12.09	11.73	14.82
+	1.937	0.98	1.97	Q9JLV5	Cullin-3	Cul3	5491	21	21	21	32.2	32.2	32.2	88.947	0.0000	109.48	24518000	235	16.48	16.05	16.44	16.53	15.60	15.51	15.81	14.68
	1.437	0.98	1.97	Q9WV76	AP-4 complex subunit beta-1	Ap4b1	5735	4	4	4	8.1	8.1	8.1	82.975	0.0051	2.313	2462400	14	14.78	14.22	14.42	14.76	13.46	13.80	14.31	12.70
	0.439	0.97	1.97	Q8C0D4	Rho GTPase-activating protein 12	Arhgap12	3331	8	8	8	14.2	14.2	14.2	95.351	0.0000	16.856	9925300	31	16.36	15.64	15.51	15.57	13.36	16.79	16.11	12.91
	1.273	0.97	1.97	Q6NSU3	Glycosyltransferase 8 domain-containing protein 1	Glt8d1	2527	3	3	3	8.1	8.1	8.1	41.99	0.0000	6.589	1891000	26	14.04	13.73	15.20	14.01	13.99	13.14	13.03	12.93
+	3.674	0.97	1.96	Q9CQN1	Heat shock protein 75 kDa, mitochondrial	Trap1	4614	34	34	34	56.7	56.7	56.7	80.208	0.0000	323.31	142070000	930	18.52	18.27	18.71	18.54	17.60	17.45	17.74	17.37
+	3.000	0.97	1.96	Q9CXR1	Dehydrogenase/reductase SDR family member 7	Dhrs7	4763	12	12	12	37.9	37.9	37.9	38.167	0.0000	62.047	21702000	160	16.55	16.31	16.82	16.92	15.48	15.69	15.90	15.62
+	2.488	0.97	1.96	Q6PD26	GPI transamidase component PIG-S	Pigs	2609	11	11	11	34.2	34.2	34.2	61.71	0.0000	115.2	14674000	108	15.79	15.18	16.03	15.95	14.90	14.57	14.85	14.73
	0.724	0.97	1.96	P49070	Calcium signal-modulating cyclophilin ligand	Camlg	1154	4	4	4	23.8	23.8	23.8	32.542	0.0000	80.231	7273400	48	15.99	15.73	16.06	15.74	15.17	15.31	16.10	13.06
+	1.918	0.97	1.96	Q8VCI0	Phospholipase B-like 1;Phospholipase B-like 1 chain A;Phospholipase E	Plbd1	3923	18	18	18	36.4	36.4	36.4	62.998	0.0000	251.87	70800000	405	17.85	17.66	17.27	17.64	16.96	16.04	17.10	16.44
	1.650	0.97	1.96	Q3UHA3	Spatacsin	Spg11	1968	12	12	12	7.4	7.4	7.4	273.93	0.0000	89.172	26211000	85	17.08	16.84	17.61	17.20	16.14	16.01	16.99	15.72
+	2.077	0.97	1.96	P54818	Galactocerebrosidase	Galc	1259	7	7	7	15.2	15.2	15.2	77.256	0.0000	57.317	18472000	155	16.85	16.77	16.57	17.10	16.27	15.22	15.87	16.04
	1.247	0.97	1.96	Q8R3F5	Malonyl-CoA-acyl carrier protein transacylase, mitochondrial	Mcat	3844	6	6	6	27	27	27	41.928	0.0000	47.02	5236200	65	14.74	14.71	15.71	15.30	14.11	14.93	14.24	13.30
	0.764	0.97	1.96	Q8BQZ4	Ral GTPase-activating protein subunit beta	Ralgapb	3174	9	9	9	9.1	9.1	9.1	165.2	0.0000	24.901	3693900	39	14.70	13.67	13.88	14.15	12.15	12.09	14.03	14.25
+	2.874	0.97	1.96	Q8K1R7	Serine/threonine-protein kinase Nek9	Nek9	3633	19	19	19	22.3	22.3	22.3	107.14	0.0000	137.17	33430000	287	16.87	16.44	16.98	16.65	15.85	15.68	16.05	15.48
	1.085	0.97	1.96	Q8CHT3	Integrator complex subunit 5	Ints5	3532	2	2	2	2.5	2.5	2.5	108.35	0.0000	9.2417	1660400	17	13.57	13.42	14.12	14.26	13.05	13.02	13.71	11.72
+	2.369	0.97	1.95	Q91XU3	Phosphatidylinositol 5-phosphate 4-kinase type-2 gamma	Pip4k2c	4149	23	23	23	67.7	67.7	67.7	47.335	0.0000	133.29	32750000	302	16.57	16.14	16.64	16.95	15.82	15.70	15.71	15.20
	0.766	0.97	1.95	Q8BFW4	Tripartite motif-containing protein 65	Trim65	2950	8	8	8	23	23	23	58.458	0.0000	140.82	8834000	98	15.58	15.34	15.91	15.51	15.23	15.13	15.32	12.79
+	1.840	0.97	1.95	Q9EP53	Hamartin	Tsc1	5242	10	10	10	13.3	13.3	13.3	128.74	0.0000	17.613	4917500	51	15.20	14.74	15.50	15.29	13.80	14.36	14.82	13.88
+	2.701	0.97	1.95	O55234	Proteasome subunit beta type-5	Psmb5	466	12	12	12	47	47	47	28.532	0.0000	187.96	44091000	259	17.69	17.35	17.42	17.85	16.57	16.28	16.98	16.61
+	2.689	0.97	1.95	Q9CZD3	Glycine--tRNA ligase	Gars	4825	39	39	39	57.3	57.3	57.3	81.877	0.0000	323.31	138380000	924	17.53	17.30	17.92	17.69	16.82	16.73	16.78	16.25
	1.333	0.96	1.95	Q8BPM0	Disheveled-associated activator of morphogenesis 1	Daam1	3165	13	13	12	16.9	16.9	15.9	123.37	0.0000	28.818	8284300	77	15.16	14.69	15.65	14.94	14.24	13.95	14.98	13.41
	1.373	0.96	1.95	P58682	Toll-like receptor 8	Tlr8	1320	4	4	4	5	5	5	119.36	0.0000	16.039	3927800	42	14.10	15.16	14.24	14.39	13.64	13.70	14.03	12.67
+	3.375	0.96	1.95	Q80UM3	N-alpha-acetyltransferase 15, NatA auxiliary subunit	Naa15	2832	42	42	38	51.2	51.2	46.6	100.96	0.0000	293.45	101920000	600	17.44	16.96	17.17	17.39	16.21	16.12	16.51	16.27
	0.741	0.96	1.95	Q9ESJ0	Exportin-4	Xpo4	5337	4	4	4	5.9	5.9	5.9	129.98	0.0000	16.175	4120400	48	14.38	13.71	15.99	15.17	12.71	14.01	14.61	14.06
+	1.870	0.96	1.95	Q3UYV9	Nuclear cap-binding protein subunit 1	Ncbp1	2045	7	7	7	11.1	11.1	11.1	91.926	0.0000	82.179	10920000	127	15.71	15.33	15.73	15.61	14.46	14.78	15.27	14.02
	1.017	0.96	1.95	P30285	Cyclin-dependent kinase 4	Cdk4	976	3	2	2	11.6	8.9	8.9	33.75	0.0000	14.521	1412500	25	13.59	13.07	14.60	14.54	13.08	13.84	12.64	12.40
	0.914	0.96	1.95	Q3UMT1	Protein phosphatase 1 regulatory subunit 12C	Ppp1r12c	2002	14	14	14	28	28	28	84.684	0.0000	27.179	5494500	28	14.52	13.31	14.69	15.12	12.43	13.76	14.14	13.47
+	2.534	0.96	1.95	Q9JI11	Serine/threonine-protein kinase 4;Serine/threonine-protein kinase 4 37k	Stk4	5382	22	22	17	47.8	47.8	39.6	55.541	0.0000	178.14	67326000	559	17.41	17.06	17.38	17.43	16.42	16.51	16.67	15.84
	1.742	0.96	1.94	Q9QZE7	Translin-associated protein X	Tsnax	5596	22	22	22	79	79	79	32.926	0.0000	227.54	52044000	432	17.16	16.70	16.77	16.82	16.14	16.04	16.34	15.09
+	3.324	0.96	1.94	Q9Z2X2	26S proteasome non-ATPase regulatory subunit 10	Psmd10	5871	3	3	3	16.9	16.9	16.9	25.083	0.0000	7.7056	5332900	60	15.80	15.78	16.02	15.67	15.09	14.53	14.92	14.91
+	2.073	0.96	1.94	Q9Z0F8	Disintegrin and metalloproteinase domain-containing protein 17	Adam17	5767	23	23	23	34.1	34.1	34.1	93.055	0.0000	115.64	32172000	278	16.28	15.97	16.18	16.12	15.48	15.01	15.65	14.59
	1.136	0.96	1.94	Q91X21	Uncharacterized protein KIAA2013	Kiaa2013	4133	5	5	5	12.6	12.6	12.6	69.424	0.0000	23.809	6143700	55	14.80	14.75	13.63	14.99	13.69	12.69	13.81	14.16
	0.644	0.96	1.94	Q5NCF2	Trafficking protein particle complex subunit 1	Trappc1	2117	5	5	5	29.7	29.7	29.7	16.881	0.0002	5.3944	4424300	30	15.88	14.75	14.77	14.62	14.82	13.83	12.31	15.23
+	2.152	0.96	1.94	Q8BM72	Heat shock 70 kDa protein 13	Hspa13	3128	7	7	7	20.2	20.2	20.2	51.709	0.0000	25.17	8245100	62	15.44	15.17	15.53	15.31	14.57	14.79	14.51	13.76
+	2.965	0.96	1.94	Q99L27	GMP reductase 2	Gmpr2	4405	16	16	14	50.3	50.3	44.3	38.018	0.0000	99.388	38036000	249	17.04	16.50	16.91	17.13	16.08	15.98	16.00	15.69
+	2.384	0.96	1.94	Q8K2I4	Beta-mannosidase	Manba	3662	16	16	16	21.4	21.4	21.4	100.83	0.0000	185.71	32073000	254	17.48	17.25	16.80	17.08	16.09	15.83	16.56	16.32
+	2.514	0.96	1.94	Q7TN98;Q7	Cytoplasmic polyadenylation element-binding protein 4	Cpeb4	2757	13	13	9	25.1	25.1	17.7	80.121	0.0000	93.945	10911000	144	15.27	15.03	15.00	15.38	14.14	13.77	14.60	14.36
	1.469	0.96	1.94	Q8VDK1	Nitrilase homolog 1	Nit1	3957	8	8	8	40.2	40.2	40.2	35.705	0.0000	96.486	22808000	214	16.76	16.03	16.04	16.49	15.96	15.13	15.76	14.64
+	2.357	0.95	1.94	Q99JX4	Eukaryotic translation initiation factor 3 subunit M	Eif3m	4354	16	16	16	55.3	55.3	55.3	42.516	0.0000	291.95	63672000	243	19.28	19.00	18.95	19.19	18.19	17.57	18.45	18.39
+	3.507	0.95	1.94	P35123	Ubiquitin carboxyl-terminal hydrolase 4	Usp4	1010	27	26	26	36.5	35.6	35.6	108.34	0.0000	166.72	73239000	440	19.31	18.87	19.41	19.12	18.34	18.25	18.20	18.10
+	1.919	0.95	1.94	Q5SSW2	Proteasome activator complex subunit 4	Psme4	2140	13	13	13	10.8	10.8	10.8	211.19	0.0000	61.035	11301000	81	16.51	15.48	15.92	16.09	14.86	14.68	15.39	15.23
+	3.937	0.95	1.94	P55264	Adenosine kinase	Adk	1271	18	18	18	53.5	53.5	53.5	40.148	0.0000	115.62	50008000	339	17.15	16.81	16.74	16.72	15.95	15.97	15.89	15.80
	1.485	0.95	1.94	O09012	Peroxisomal targeting signal 1 receptor	Pex5	317	8	8	8	20.5	20.5	20.5	70.755	0.0000	24.031	2172200	39	13.98	13.49	14.73	13.45	12.89	12.51	13.34	13.09
	1.502	0.95	1.94	Q64331	Unconventional myosin-VI	Myo6	2431	5	5	5	6.2	6.2	6.2	146.41	0.0000	13.071	3310800	23	14.09	13.55	14.91	14.44	13.72	13.41	12.83	13.22
+	2.679	0.95	1.93	Q8BHG1	Nardilysin	Nrd1	3036	26	26	26	31.5	31.5	31.5	132.89	0.0000	156.75	29181000	295	16.72	16.32	16.46	16.58	15.72	15.44	15.94	15.18
	1.358	0.95	1.93	Q8R4C2	RUN and FYVE domain-containing protein 2	Rufy2	3868	8	6	6	14.9	12.4	12.4	70.092	0.0000	19.505	3836100	62	15.05	14.07	15.50	15.28	14.35	14.37	13.78	13.59
+	1.964	0.95	1.93	Q62192	CD180 antigen	Cd180	2367	17	17	17	30.1	30.1	30.1	74.302	0.0000	219.1	82930000	585	17.67	17.16	17.19	17.36	16.98	16.13	16.55	15.92
+	2.117	0.95	1.93	Q920I9	WD repeat-containing protein 7	Wdr7	4211	15	15	15	16.7	16.7	16.7	163.45	0.0000	139.44	13246000	182	15.80	15.16	15.56	15.76	14.80	14.14	15.03	14.51
	1.500	0.95	1.93	Q8VCX5	Calcium uptake protein 1, mitochondrial	Micu1	3936	11	11	11	27.5	27.5	27.5	54.352	0.0000	35.6	12843000	113	16.37	16.17	16.44	16.12	15.48	15.64	15.81	14.35
+	1.904	0.95	1.93	Q9EQ80	NIF3-like protein 1	Nif3l1	5273	6	6	6	23.9	23.9	23.9	41.745	0.0000	33.682	10578000	127	16.00	15.67	15.74	15.81	15.38	14.57	15.20	14.28
+	2.555	0.95	1.93	O08739	AMP deaminase 3	Ampd3	292	26	26	23	41.3	41.3	37.3	88.651	0.0000	243.31	50484000	444	17.59	16.89	17.38	17.32	16.20	16.09	16.65	16.45
+	2.212	0.95	1.93	P27046	Alpha-mannosidase 2	Man2a1	932	36	36	36	37.5	37.5	37.5	131.63	0.0000	314.53	57148000	557	16.89	16.56	16.55	16.44	15.94	15.10	16.01	15.60
	1.047	0.95	1.93	Q923B1	Lariat debranching enzyme	Dbr1	4281	5	5	5	15.6	15.6	15.6	62.289	0.0000	57.271	4693800	27	14.50	14.15	15.73	14.88	14.56	13.22	14.26	13.42
	0.419	0.95	1.93	Q9EPK2	Protein XRP2	Rp2	5254	7	7	7	21.9	21.9	21.9	39.376	0.0000	61.944	7658100	71	15.43	15.79	15.79	15.39	15.93	11.73	15.89	15.08
	1.871	0.95	1.93	P27808	Alpha-1,3-mannosyl-glycoprotein 2-beta-N-acetylglucosaminyltransfera:	Mgat1	941	12	12	12	26.2	26.2	26.2	51.69	0.0000	32.399	9462900	82	15.35	15.20	15.76	15.67	14.97	13.96	14.91	14.36
	0.871	0.94	1.92	Q9ES56	Trafficking protein particle complex subunit 4	Trappc4	5329	5	5	4	22.4	22.4	21.9	24.385	0.0004	4.6693	6757600	40	14.78	15.42	14.75	15.29	13.81	12.83	14.59	15.24
	0.612	0.94	1.92	P23611	Interferon regulatory factor 8	Irf8	887	4	4	4	14.9	14.9	14.9	48.237	0.0000	9.81	2569700	15	13.66	14.11	14.99	14.16	11.73	14.73	14.05	12.64
	1.768	0.94	1.92	Q9QX60	Deoxyguanosine kinase, mitochondrial	Dguok	5533	4	4	4	22	22	22	32.281	0.0000	140.24	5615300	47	15.58	15.17	15.25	15.29	14.63	13.92	15.03	13.93
	0.995	0.94	1.92	Q6PHQ8	N-alpha-acetyltransferase 35, NatC auxiliary subunit	Naa35	2641	11	11	11	20.8	20.8	20.8	83.305	0.0000	37.759	6055500	76	14.42	13.63	14.42	14.96	13.07	12.50	14.37	13.72
+	2.392	0.94	1.92	Q8BUR4	Dedicator of cytokinesis protein 1	Dock1	3229	22	21	20	15.1	14.6	14	215.08	0.0000	42.03	12822000	128	15.69	14.99	15.69	15.37	14.55	14.81	14.36	14.24
	1.669	0.94	1.92	P17665	Cytochrome c oxidase subunit 7C, mitochondrial	Cox7c	827	4	4	4	31.7	31.7	31.7	7.3325	0.0000	39.373	3648000	46	14.68	14.73	14.92	15.01	13.62	13.74	14.76	13.45
	1.640	0.94	1.92	Q9DC48	Pre-mRNA-processing factor 17	Cdc40	5192	7	7	6	20.2	20.2	16.8	65.46	0.0000	34.003	3314200	33	13.85	14.06	14.70	14.08	12.71	12.89	13.70	13.62
	1.018	0.94	1.92	P62715	Serine/threonine-protein phosphatase 2A catalytic subunit beta isoform	Ppp2cb	1437	24	3	3	85.4	9.4	9.4	35.575	0.0000	12.968	2571700	27	14.79	13.96	14.18	14.30	12.04	13.87	13.72	13.84
	1.803	0.94	1.92	O55125	Protein NipSnap homolog 1	Nipsnap1	455	6	6	6	19.7	19.7	19.7	33.363	0.0000	8.4139	4995800	78	15.46	15.15	15.62	14.88	14.52	13.66	14.63	14.56
+	2.356	0.94	1.92	Q9R1Q9	V-type proton ATPase subunit S1	Atp6ap1	5659	8	8	8	24.2	24.2	24.2	51.007	0.0000	158.06	10674000	118	15.68	15.59	15.78	15.89	14.71	14.27	14.99	15.20
+	3.615	0.94	1.92	Q8R0X7	Sphingosine-1-phosphate lyase 1	Sgpl1	3768	48	48	48	62.1	62.1	62.1	63.676	0.0000	323.31	954320000	1981	20.45	20.13	20.44	20.31	19.33	19.21	19.66	19.38
+	4.070	0.94	1.92	Q8K2K6	Arf-GAP domain and FG repeat-containing protein 1	Agfg1	3664	9	9	7	23.5	23.5	21.6	58.042	0.0000	69.107	13236000	150	16.07	15.68	16.05	15.79	14.93	14.90	15.01	14.98
+	2.459	0.94	1.92	Q9WU56	tRNA pseudouridine synthase A, mitochondrial	Pus1	5697	6	6	6	24.1	24.1	24.1	47.502	0.0000	9.2007	1665900	20	13.79	13.92	14.59	13.92	12.97	13.24	13.31	12.96
+	2.787	0.94	1.92	Q7TQ95	Protein lunapark	Lnp	2773	7	7	7	20.2	20.2	20.2	47.499	0.0000	25.415	13429000	131	15.54	15.25	15.68	15.42	14.25	14.38	14.93	14.59
	1.799	0.94	1.91	Q8K2Q7	BRO1 domain-containing protein BROX	Brox	3668	19	19	19	60.3	60.3	60.3	46.201	0.0000	230.61	69681000	437	18.28	17.84	17.44	17.70	17.25	16.37	17.24	16.64
+	2.666	0.93	1.91	Q9JHR7	Insulin-degrading enzyme	Ide	5370	36	36	36	37.7	37.7	37.7	117.77	0.0000	219.47	53079000	469	16.78	16.33	16.27	16.08	15.51	15.37	15.68	15.17
+	2.493	0.93	1.91	O08688	Calpain-5	Capn5	285	14	14	14	25.6	25.6	25.6	72.954	0.0000	32.828	12926000	150	15.55	15.21	15.29	15.38	14.69	13.88	14.55	14.57
+	2.059	0.93	1.91	P83887;Q8V	Tubulin gamma-1 chain;Tubulin gamma-2 chain	Tubg1;Tubg2	1604	10	10	10	41	41	41	51.1	0.0000	108.45	8161500	99	15.32	14.83	15.42	14.84	14.68	13.99	13.81	14.19
+	3.325	0.93	1.91	O55229	Choline/ethanolamine kinase	Chkb	465	12	12	12	44.4	44.4	44.4	45.126	0.0000	96.94	14775000	157	15.46	15.45	15.74	15.76	14.72	14.37	14.88	14.70
+	2.505	0.93	1.91	P97377	Cyclin-dependent kinase 2	Cdk2	1639	12	11	9	48.6	46.2	39	38.978	0.0000	54.743	23298000	145	16.51	16.30	16.91	16.74	15.97	15.70	15.76	15.29
	1.658	0.93	1.91	B1AVY7	Kinesin-like protein KIF16B	Kif16b	70	9	9	9	8.8	8.8	8.8	150.06	0.0000	129.33	8889100	139	15.17	14.74	14.90	14.99	14.22	13.16	14.29	14.40
	1.760	0.93	1.91	Q9D6N1	Carbonic anhydrase 13	Ca13	5004	4	4	4	24.4	24.4	24.4	29.522	0.0000	38.532	6242200	86	15.03	14.86	15.10	15.11	13.42	14.75	14.28	13.92
+	2.608	0.93	1.90	P70698	CTP synthase 1	Ctps1	1586	19	17	17	39.9	39.9	39.9	66.682	0.0000	92.879	26101000	201	16.90	16.85	17.37	17.07	15.81	16.41	16.32	15.94
+	3.472	0.93	1.90	Q8BMD8	Calcium-binding mitochondrial carrier protein SCaMC-1	Slc25a24	3134	27	27	27	61.3	61.3	61.3	52.901	0.0000	323.31	175660000	773	18.92	18.75	18.95	18.96	18.14	17.70	18.19	17.84
	1.182	0.93	1.90	Q14CH7	Alanine--tRNA ligase, mitochondrial	Aars2	1790	11	11	10	20.4	20.4	19.3	106.78	0.0000	36.172	17650000	80	17.06	17.00	17.52	17.19	16.53	16.50	16.92	15.11
	1.863	0.93	1.90	Q3UMB5	Smith-Magenis syndrome chromosomal region candidate gene 8 proteir	Smcr8	1998	13	13	13	17.6	17.6	17.6	104.96	0.0000	44.687	8543300	126	14.91	14.30	14.92	15.01	13.58	13.66	14.49	13.69
	0.883	0.93	1.90	Q6PDI6	Protein FAM63B	Fam63b	2614	3	2	2	6.2	4.7	4.7	65.636	0.0000	9.2199	3626500	16	14.69	15.41	14.87	14.29	14.16	14.62	12.49	14.28
	0.930	0.93	1.90	Q9JHS3	Ragulator complex protein LAMTOR2	Lamtor2	5371	6	6	6	70.4	70.4	70.4	13.48	0.0000	211.36	19603000	139	17.66	17.15	16.39	16.27	16.75	14.91	16.21	15.88
+	3.062	0.93	1.90	Q62393	Tumor protein D52	Tpd52	2385	10	10	10	58.9	58.9	58.9	24.313	0.0000	193.86	23286000	216	15.87	16.11	15.78	16.06	15.28	15.23	14.77	14.84
+	2.302	0.93	1.90	Q9WVQ5	Methylthioribulose-1-phosphate dehydratase	Apip	5759	15	15	15	68.5	68.5	68.5	26.949	0.0000	57.305	38741000	327	16.66	16.22	16.88	16.82	16.02	15.50	15.94	15.41

	1.865	0.93	1.90	Q9ESL4	Mitogen-activated protein kinase kinase kinase MLT	Zak	5340	8	8	8	9.6	9.6	9.6	91.719	0.0000	16.861	5511400	55	15.00	14.27	14.88	14.99	13.45	14.41	13.91	13.66
	1.092	0.93	1.90	A2AT37		Upf2	39	6	6	6	7.6	7.6	7.6	147.55	0.0000	14.763	1417900	18	13.17	12.89	14.14	13.22	11.66	12.42	12.28	13.35
	0.506	0.92	1.90	Q8VE19	WD repeat-containing protein mio	Mios	3983	7	7	7	13	13	13	98.334	0.0000	29.589	7877700	25	16.08	16.05	16.45	15.71	15.91	12.68	16.04	15.95
+	3.079	0.92	1.90	Q61081	Hsp90 co-chaperone Cdc37;Hsp90 co-chaperone Cdc37, N-terminally p	Cdc37	2246	28	28	28	53.8	53.8	53.8	44.593	0.0000	323.31	139030000	751	18.51	18.20	18.71	18.58	17.50	17.61	17.84	17.35
	1.904	0.92	1.89	Q61127	NGFI-A-binding protein 2	Nab2	2255	6	6	5	13.3	13.3	11.6	56.576	0.0000	37.454	3720200	30	14.67	14.59	14.59	15.13	13.56	13.41	13.88	14.44
	1.970	0.92	1.89	Q640Q5	PAB-dependent poly(A)-specific ribonuclease subunit PAN3	Pan3	2416	4	4	4	7	7	7	89.744	0.0000	12.212	3277200	45	14.45	14.40	14.76	14.38	13.28	14.05	13.08	13.90
+	2.435	0.92	1.89	Q5SSH7	Zinc finger ZZ-type and EF-hand domain-containing protein 1	Zzef1	2136	21	21	21	10.8	10.8	10.8	328.31	0.0000	65.385	19442000	183	16.44	15.74	16.47	16.03	15.31	15.02	15.49	15.19
	0.841	0.92	1.89	Q06180	Tyrosine-protein phosphatase non-receptor type 2	Ptpn2	1749	7	7	7	20.2	20.2	20.2	47.36	0.0000	44.3	4056100	44	14.61	13.95	14.12	13.71	14.31	12.17	12.46	13.77
	1.130	0.92	1.89	Q8BR65	Sin3 histone deacetylase corepressor complex component SDS3	Suds3	3176	4	4	4	12.2	12.2	12.2	38.107	0.0000	20.67	1448200	40	14.07	13.20	13.58	13.56	11.65	12.85	12.71	13.52
+	2.725	0.92	1.89	Q6P9J9	Anoctamin-6	Ano6	2586	17	17	17	21.3	21.3	21.3	106.25	0.0000	172.2	47598000	356	16.75	16.32	16.90	16.80	15.64	15.57	15.79	16.10
+	2.674	0.92	1.89	Q6ZQK5	Arf-GAP with coiled-coil, ANK repeat and PH domain-containing protein	Acap2	2700	25	25	24	42.6	42.6	41.6	87.21	0.0000	146.38	43330000	450	16.67	16.13	16.80	16.56	15.75	15.48	15.84	15.41
	2.080	0.92	1.89	Q61151	Serine/threonine-protein phosphatase 2A 56 kDa regulatory subunit eps	Ppp2r5e	2260	10	10	8	27	27	20.8	54.713	0.0000	23.352	19560000	82	16.89	16.97	16.99	16.91	16.20	16.30	16.27	15.32
+	3.556	0.92	1.89	Q8R307	Vacuolar protein sorting-associated protein 18 homolog	Vps18	3827	33	33	33	41	41	41	110.22	0.0000	122.29	40952000	400	16.67	16.37	16.25	16.48	15.57	15.30	15.67	15.56
	0.942	0.92	1.89	Q8BWR2	PITH domain-containing protein 1	Pithd1	3264	3	3	3	24.6	24.6	24.6	24.192	0.0000	9.6934	738040	10	14.14	13.02	13.58	13.73	12.88	13.87	11.96	12.10
	2.120	0.91	1.88	P55302	Alpha-2-macroglobulin receptor-associated protein	Lrpap1	1272	23	23	23	61.4	61.4	61.4	42.215	0.0000	79.455	37290000	279	16.69	17.14	16.13	16.85	15.79	16.02	15.77	15.57
+	2.249	0.91	1.88	Q9D2Y5	Sorting nexin-20	Snx20	4956	2	2	2	8.3	8.3	8.3	36.028	0.0002	5.9563	658290	7	13.36	13.36	13.77	13.78	12.44	12.83	12.27	13.07
	1.827	0.91	1.88	Q62230	Sialoadhesin	Siglec1	2370	38	38	38	28.6	28.6	28.6	182.98	0.0000	228.34	75068000	458	17.62	16.83	16.91	16.66	16.41	16.19	16.15	15.62
+	2.962	0.91	1.88	P35486;P35	Pyruvate dehydrogenase E1 component subunit alpha, somatic form, m	Pdha1	1021	32	32	32	66.7	66.7	66.7	43.231	0.0000	129.66	105090000	638	17.53	17.07	17.48	17.52	16.55	16.28	16.76	16.36
+	2.188	0.91	1.88	Q922J9	Fatty acyl-CoA reductase 1	Far1	4261	7	7	7	18.3	18.3	18.3	59.434	0.0000	16.113	9329100	58	15.84	15.62	14.86	15.30	14.57	14.30	14.59	14.52
+	2.886	0.91	1.88	P63328;P48	Serine/threonine-protein phosphatase 2B catalytic subunit alpha isoform	Ppp3ca	1506	25	25	16	48.9	48.9	29.9	58.643	0.0000	184.84	62502000	478	17.12	16.70	17.22	17.16	16.25	15.90	16.39	16.02
	1.818	0.91	1.88	P59114	Phosphorylated CTD-interacting factor 1	Pcif1	1332	3	3	3	6.9	6.9	6.9	80.504	0.0000	9.1816	1765500	33	13.73	13.66	14.54	14.01	13.07	12.82	12.81	13.59
+	3.890	0.91	1.88	P97310	DNA replication licensing factor MCM2	Mcm2	1620	36	36	36	47.1	47.1	47.1	102.08	0.0000	240	49955000	567	16.66	16.31	16.69	16.46	15.77	15.52	15.58	15.61
	1.579	0.91	1.88	Q924C1	Exportin-5	Xpo5	4291	13	13	13	17.1	17.1	17.1	136.97	0.0000	57.282	6515000	99	14.87	14.38	14.76	14.55	13.64	12.97	14.34	13.98
+	2.239	0.91	1.88	Q62383	Transcription elongation factor SPT6	Supt6h	2383	27	27	27	22.2	22.2	22.2	199.08	0.0000	42.089	255010000	110	21.97	21.33	21.97	21.78	20.91	21.03	21.07	20.39
	1.221	0.91	1.88	Q9QZ05	Eukaryotic translation initiation factor 2-alpha kinase 4	Eif2ak4	5582	3	3	3	2.3	2.3	2.3	186.48	0.0000	8.3241	1672300	17	13.53	12.93	14.59	13.49	12.22	12.69	12.95	13.04
+	2.242	0.91	1.88	P35991;Q60	Tyrosine-protein kinase BTK	Btk	1039	27	27	26	46.7	46.7	45.5	76.437	0.0000	323.31	81213000	546	17.91	17.24	17.42	17.63	16.94	16.25	16.87	16.51
+	2.498	0.91	1.88	Q9Z1K6	E3 ubiquitin-protein ligase ARIH2	Arih2	5811	10	10	10	30.7	30.7	30.7	57.696	0.0000	87.365	12501000	151	15.45	14.83	15.44	15.35	14.32	14.13	14.70	14.28
	0.949	0.91	1.88	Q80US4	Actin-related protein 5	Actr5	2836	7	7	7	17.4	17.4	17.4	67.844	0.0000	30.049	3016000	24	14.68	14.20	14.16	15.39	12.68	14.57	13.60	13.95
+	2.255	0.91	1.88	Q99LD9	Translation initiation factor eIF-2B subunit beta	Eif2b2	4422	12	12	12	42.7	42.7	42.7	38.897	0.0000	66.297	15680000	172	15.63	15.25	16.03	15.94	14.80	14.61	15.16	14.66
+	2.544	0.91	1.88	Q91YP2	Neurolysin, mitochondrial	Nln	4170	24	24	24	42.2	42.2	42.2	80.428	0.0000	134	44896000	306	17.05	16.60	17.17	16.95	15.95	16.05	16.41	15.73
	1.393	0.91	1.87	Q9CQ54	NADH dehydrogenase [ubiquinone] 1 subunit C2	Ndufc2	4562	9	9	9	60	60	60	14.164	0.0000	36.309	24107000	147	17.26	16.71	17.39	17.06	16.37	16.33	16.79	15.31
+	2.482	0.91	1.87	Q4PJX1	Protein odr-4 homolog	Odr4	2064	11	11	11	31.8	31.8	31.8	49.997	0.0000	129.04	18371000	149	16.69	16.19	16.52	16.60	15.81	15.34	15.92	15.30
	1.590	0.90	1.87	Q03347	Runt-related transcription factor 1	Runx1	1726	7	7	7	23.5	23.5	23.5	48.609	0.0000	33.2	7395600	56	15.69	15.09	16.14	15.71	14.59	15.40	14.41	14.62
+	2.588	0.90	1.87	Q91VU6	DDB1- and CUL4-associated factor 11	Dcaf11	4079	7	7	7	18	18	18	61.991	0.0000	64.588	5212800	100	14.77	14.28	15.01	14.75	13.54	13.74	14.02	13.89
	1.650	0.90	1.87	Q91VW3	SH3 domain-binding glutamic acid-rich-like protein 3	Sh3bgrl3	4081	7	7	7	79.6	79.6	79.6	10.477	0.0000	90.79	45794000	125	17.98	18.31	17.72	17.99	17.49	17.59	16.83	16.47
	1.821	0.90	1.87	Q91ZV0	Melanoma inhibitory activity protein 2	Mia2	4201	14	14	14	12	12	12	156.46	0.0000	69.504	16240000	90	16.37	15.96	16.29	16.44	15.48	14.95	16.01	15.01
+	3.499	0.90	1.87	P24527	Leukotriene A-4 hydrolase	Lta4h	898	42	42	42	74.1	74.1	74.1	69.05	0.0000	323.31	204930000	926	18.45	18.34	18.59	18.65	17.53	17.46	17.90	17.53
	0.933	0.90	1.87	Q5U4D9	THO complex subunit 6 homolog	Thoc6	2166	7	7	7	26.1	26.1	26.1	37.314	0.0000	52.323	6648900	58	14.94	14.28	15.73	15.12	13.94	15.18	14.03	13.31
	1.290	0.90	1.86	Q6PE01	U5 small nuclear ribonucleoprotein 40 kDa protein	Snrnp40	2622	13	13	13	57.3	57.3	57.3	39.275	0.0000	318.87	26890000	236	16.21	15.92	16.53	16.44	15.50	15.73	15.91	14.38
	1.729	0.90	1.86	Q3TIR1	Trafficking protein particle complex subunit 13	Trappc13	1848	6	6	6	22.3	22.3	22.3	46.476	0.0000	57.481	10301000	82	15.61	15.95	16.21	15.93	14.31	15.06	15.32	15.41
+	2.996	0.90	1.86	P47934	Carnitine O-acetyltransferase	Crat	1131	21	21	20	37.5	37.5	35.8	70.839	0.0000	108.8	35158000	306	16.45	16.21	16.45	16.26	15.45	15.47	15.77	15.10
	1.477	0.90	1.86	Q566J8	AarF domain-containing protein kinase 4	Adck4	2085	18	18	18	58	58	58	59.231	0.0000	279.28	27053000	248	16.52	15.91	16.01	15.99	15.45	15.58	15.48	14.33
	1.506	0.90	1.86	O70469	Docking protein 2	Dok2	502	11	11	11	32	32	32	45.522	0.0000	14.118	15459000	83	16.78	16.21	15.50	16.24	15.43	15.47	15.51	14.74
+	2.653	0.89	1.86	Q8CFQ3	Intron-binding protein aquarius	Aqr	3491	15	15	15	16.1	16.1	16.1	170.29	0.0000	192.83	12187000	147	15.31	14.83	15.51	15.22	14.31	14.04	14.45	14.49
	2.220	0.89	1.86	Q9D906	Ubiquitin-like modifier-activating enzyme ATG7	Atg7	5089	33	33	33	54	54	54	77.519	0.0000	302.48	77517000	514	16.53	16.39	16.80	16.97	15.93	15.51	16.19	15.49
+	2.424	0.89	1.86	Q99PV0	Pre-mRNA-processing-splicing factor 8	Prpf8	4518	83	83	82	41.6	41.6	40.3	273.61	0.0000	323.31	395960000	1135	21.60	20.91	21.50	21.46	20.63	20.26	20.73	20.29
	0.961	0.89	1.86	Q8BH15	CCR4-NOT transcription complex subunit 10	Cnot10	3007	3	3	3	6.6	6.6	6.6	81.817	0.0000	32.47	1221800	44	13.44	12.44	13.73	13.22	12.14	11.59	12.12	13.41
+	2.382	0.89	1.86	Q8R059	UDP-glucose 4-epimerase	Gale	3748	13	13	13	43.2	43.2	43.2	38.224	0.0000	137.25	14950000	164	15.92	15.64	15.86	15.57	14.93	15.27	14.83	14.40
	2.114	0.89	1.86	Q61037	Tuberin	Tsc2	2242	6	6	6	5.8	5.8	5.8	202.07	0.0000	53.43	5011500	67	14.69	14.80	14.92	14.87	13.89	13.63	14.57	13.63
	1.844	0.89	1.85	Q61235;Q9	Beta-2-syntrophin	Sntb2	2283	10	10	10	21.5	21.5	21.5	56.381	0.0000	28.839	14583000	132	16.09	16.05	16.29	16.45	15.59	15.54	15.59	14.60
+	2.963	0.89	1.85	P42227	Signal transducer and activator of transcription 3	Stat3	1076	33	33	33	56	56	56	88.053	0.0000	323.31	86484000	657	17.12	16.68	17.07	16.86	16.25	15.91	16.22	15.80
	1.541	0.89	1.85	Q5F2E7	Nuclear fragile X mental retardation-interacting protein 2	Nufip2	2103	12	12	12	23.6	23.6	23.6	75.656	0.0000	33.707	2826200	18	12.82	13.12	13.36	13.20	12.29	13.03	11.86	11.75
+	3.372	0.89	1.85	O35286	Pre-mRNA-splicing factor ATP-dependent RNA helicase DHX15	Dhx15	352	43	43	42	53.5	53.5	53.5	91.006	0.0000	308.44	186960000	1076	18.10	17.58	17.99	17.88	17.09	17.00	17.08	16.83
+	3.582	0.89	1.85	Q62186	Translocon-associated protein subunit delta	Ssr4	2364	9	9	9	45.3	45.3	45.3	18.936	0.0000	120.4	92116000	341	17.84	17.92	17.88	18.04	17.20	16.75	17.20	16.96
	1.185	0.89	1.85	Q6DFX2	Anthrax toxin receptor 2	Antxr2	2507	3	3	3	11.3	11.3	11.3	53.184	0.0000	9.7942	1926100	24	14.48	13.36	14.15	13.47	13.73	12.90	12.93	12.34
	0.739	0.89	1.85	Q8CFI0	E3 ubiquitin-protein ligase NEDD4-like	Nedd4l	3487	10	10	10	15.3	15.3	15.3	115.42	0.0000	24.342	7237500	60	15.14	14.70	15.19	15.79	15.33	13.55	15.17	13.22
	2.309	0.89	1.85	Q9D7B6	Isobutyryl-CoA dehydrogenase, mitochondrial	Acad8	5027	14	14	14	47.7	47.7	47.7	45.019	0.0000	105.8	31368000	256	16.52	16.16	16.90	16.85	15.58	15.55	16.05	15.70
+	2.569	0.89	1.85	Q6PGB6	N-alpha-acetyltransferase 50	Naa50	2631	9	9	9	46.2	46.2	46.2	19.414	0.0000	61.47	33131000	276	17.02	16.52	16.88	17.10	16.18	15.95	16.19	15.65
	1.489	0.89	1.85	Q6ZQ88	Lysine-specific histone demethylase 1A	Kdm1a	2692	14	14	14	26.4	26.4	26.4	92.849	0.0000	75.652	14421000	138	15.27	15.51	16.19	15.94	14.20	14.74	15.32	15.10
	1.830	0.89	1.85	Q60931	Voltage-dependent anion-selective channel protein 3	Vdac3	2223	22	22	22	73.1	73.1	73.1	30.752	0.0000	323.31	180230000	703	19.08	18.75	19.09	19.31	18.24	17.60	18.75	18.09
	2.016	0.89	1.85	O54692	Centromere/kinetochore protein zw10 homolog	Zw10	417	17	17	16	28.8	28.8	28.6	88.062	0.0000	156.02	14136000	172	15.94	15.46	16.36	16.10	15.13	14.91	15.46	14.82
+	2.738	0.89	1.85	Q9Z2N8;Q9	Actin-like protein 6A	Actl6a	5860	18	18	17	56.2	56.2	55.2	47.447	0.0000	252.47	55776000	344	17.11	16.71	17.42	17.15	16.26	16.16	16.41	16.03
+	2.688	0.88	1.84	Q8R0G9	Nuclear pore complex protein Nup133	Nup133	3759	29	29	29	37.9	37.9	37.9	128.62	0.0000	276.28	33160000	298	17.17	17.17	17.39	17.25	16.51	16.64	16.39	15.90
+	3.191	0.88	1.84	Q9D6Y9	1,4-alpha-glucan-branching enzyme	Gbe1	5013	21	21	21	35.8	35.8	35.8	80.363	0.0000	250.17	49354000	346	16.81	16.70	16.81	16.95	15.71	15.72	16.13	16.18
+	2.575	0.88	1.84	Q8CBE3	WD repeat-containing protein 37	Wdr37	3437	14	14	14	31.7	31.7	31.7	55.045	0.0000	103.88	17229000	158	15.88	15.62	16.20	15.91	14.90	14.93	15.42	14.83
	2.065	0.88	1.84	Q9CR89	Endoplasmic reticulum-Golgi intermediate compartment protein 2	Ergic2	4682	3	3	3	13.3	13.3	13.3	42.481	0.0000	16.663	2954000	57	14.28	13.76	14.50	14.19	13.18	12.88	13.52	13.62
	2.310	0.88	1.84	Q9ES74	Serine/threonine-protein kinase Nek7	Nek7	5332	15	15	13	58.9	58.9	53.3	34.537	0.0000	90.715	34016000	340	16.41	16.13	16.98	16.89	15.72	15.64	15.78	15.74
	1.651	0.88	1.84	O35744;Q91	Chitinase-like protein 3	Chil3	404	22	22	22	57.3	57.3	57.3	44.458	0.0000	134.22	109870000	425	17.99	18.27	17.92	18.91	17.87	17.45	17.04	17.21
	0.678	0.88	1.84	Q62293;Q3T9E4		Tgtp1	1811	9	9	9	28	28	28	47.12	0.0000	36.638	11499000	34	16.30	15.86	15.14	15.13	15.31	15.64	13.13	14.82
+	3.104	0.88	1.84	P10630	Eukaryotic initiation factor 4A-II;Eukaryotic initiation factor 4A-II, N-termi	Eif4a2	725	23	7	7	52.6	23.8	23.8	46.402	0.0000	62.978	3608600	54	14.73	14.79	15.08	15.10	13.96	14.28	13.81	14.15
	0.900	0.88	1.84	Q61084	Mitogen-activated protein kinase kinase kinase 3	Map3k3	2248	9	9	6	19.8	19.8	13.6	70.775	0.0000	50.576	6811800	52	14.89	14.29	16.06	15.03	14.29	14.51	13.23	14.72
+	3.684	0.88	1.84	P07901	Heat shock protein HSP 90-alpha	Hsp90aa1	673	71	50	50	73.5	61	61	84.787	0.0000	323.31	453040000	1411	19.34	18.88	19.08	19.13	18.31	18.18	18.34	18.09
+	3.199	0.88	1.84	Q91YH5	Atlastin-3	Atl3	4157	21	21	20	52.3	52.3	51	60.574	0.0000	323.31	64441000	411	17.87	17.67	17.77	18.00	17.01	16.85	17.25	16.71
+	3.615	0.88	1.84	P48025;P43	Tyrosine-protein kinase SYK	Syk	1139	46	46	45	73.6	73.6	73.6	71.376	0.0000	323.31	353590000	1200	19.27	19.00	19.27	19.22	18.47	18.31	18.43	18.06
	1.766	0.88	1.84	Q9D666	SUN domain-containing protein 1	Sun1	4991	9	8	8	14.7	13.8	13.8	101.98	0.0000	55.847	6404700	115	14.76	14.44	15.06	14.96	13.45	14.17	14.45	13.65
	1.444	0.88	1.83	Q01320	DNA topoisomerase 2-alpha	Top2a	1707	18	8	7	12.6	6.5	6.2	172.79	0.0000	15.058	2687500	25	13.46	13.98	14.08	13.77	12.96	13.33	13.39	12.11
	0.795	0.87	1.83	D3Z7Q2		Smim20	216	3	3	3	50.7	50.7	50.7	7.818	0.0002	5.4805	2307100	55	14.32	12.89	14.86	14.80	13.72	12.94	13.96	12.75
	0.750	0.87	1.83	Q9ER88	28S ribosomal protein S29, mitochondrial	Dap3	5300	11	11	11	33.5	33.5	33.5	44.699	0.0000	80.966	12295000	118	15.48	15.02	15.80	15.65	14.97	15.34	15.16	12.99
	2.550	0.87	1.83	Q9D6Y7	Mitochondrial peptide methionine sulfoxide reductase	Msra	5012	10	10	10	55.4	55.4	55.4	25.988	0.0000	28.872	32122000	177	16.71	16.69	16.55	16.22	15.93	15.46	15.89	15.40
	0.799	0.87	1.83	Q8JZN7	Mitochondrial Rho GTPase 2	Rhot2	3576	6	5	5	17.6	15.8	15.8	69.07	0.0000	15.468	2509300	23	13.46	13.17	14.67	14.55	13.46	13.72	13.21	11.96
+	3.754	0.87	1.83	Q8VCM8	Nicalin	Ncln	3927	17	17	17	33.9	33.9	33.9	62.907	0.0000	181.82	34954000	300	17.10	16.82	16.80	16.85	15.97	15.82	16.16	16.14
	2.374	0.87	1.83	Q9Z1T6	1-phosphatidylinositol 3-phosphate 5-kinase	Pikfyve	5823	22	22	22	15	15	15	236.87	0.0000	82.114	19894000	262	15.74	15.17	15.97	15.57	14.70	14.60	15.03	14.63
	0.733	0.87	1.83	Q6ZPR5	Sphingomyelin phosphodiesterase 4	Smpd4	2680	6	6	6	10.4	10.4	10.4	93.275	0.0000	27.803	8776000	49	15.60	15.51	16.56	15.36	15.17	13.37	15.64	15.36
	0.767	0.87	1.83	Q8CFA1	Interleukin-1 receptor-associated kinase-like 2	Irak2	3477	3	3	3	6.3	6.3	6.3	69.046	0.0010	4.013	1974300	11	15.12	14.54	14.55	14.43	14.54	12.54	13.26	14.82
	0.939	0.87	1.83	Q60591	Nuclear factor of activated T-cells, cytoplasmic 2	Nfatc2	2180	9	9	9	18.2	18.2	18.2	100.02	0.0000	60.001	11253000	96	15.74	15.57	16.39	15.64	15.14	15.58	15.44	13.69
	2.510	0.87	1.83	Q60715	Prolyl 4-hydroxylase subunit alpha-1	P4ha1	2198	10	10	10	24.2	24.2	24.2	60.909	0.0000	49.83	7714000	76	15.39	15.26	15.33	14.94	14.74	14.46	14.06	14.18
	2.548	0.87	1.82	Q91YJ2	Sorting nexin-4	Snx4	4161	21	21	21	45.3	45.3	45.3	51.777	0.0000	149.75	42073000	401	16.58	16.09	16.68	16.67	15.61	15.67	15.91	15.37
	2.600	0.87	1.82	Q8CFI7	DNA-directed RNA polymerase II subunit RPB2	Polr2b	3489	32	32	32	35.2	35.2	35.2	133.91	0.0000	291.89	51574000	558	17.06	16.43	16.72	16.80	15.81	15.79	16.23	15.70
	1.091	0.87	1.82	Q9CWS1	E3 ubiquitin-protein ligase RNF135	Rnf135	4727	2	2	2	7	7	7	46.461	0.0000	21.585	2226300	27	14.64	14.23	13.86	14.89	12.95	13.79	14.41	13.01
+	2.830	0.86	1.82	Q8BK72	28S ribosomal protein S27, mitochondrial	Mrps27	3097	13	13	13	41.7	41.7	41.7	47.778	0.0000	78.977	16542000	161	15.68	15.33	15.88	15.90	14.75	14.64	14.98	14.97
	0.947	0.86	1.82	O09126	Semaphorin-4D	Sema4d	325	8	8	8	13	13	13	95.639	0.0000	72.081	3998800	70	13.70	13.66	14.49	14.20	11.97	13.45	13.23	13.95
	0.617	0.86	1.82	Q810U5	Coiled-coil domain-containing protein 50	Ccdc50	2932	13	13	13	42.6	42.6	42.6	35.321	0.0000	30.798	6662600	43	12.50	15.32	14.02	13.59	13.44	13.54	12.17	12.83
	1.835	0.86	1.82	Q922L6	Negative elongation factor D	Nelfcd	4263	5	5	5	11.7	11.7	11.7	66.275	0.0000	11.242	4438900	38	14.76	14.29	15.14	14.92	13.53	13.72	14.32	14.09
	0.832	0.86	1.82	Q64010	Adapter molecule crk	Crk	2411	5	5	5	22.7	22.7	22.7	33.814	0.0000	18.451	1927800	17	13.66	14.90	14.22	13.52	12.09	13.20	14.02	13.55
	2.696	0.86	1.82	Q6PF93	Phosphatidylinositol 3-kinase catalytic subunit type 3	Pik3c3	2626	18	18	18	30.4	30.4	30.4	101.49	0.0000	78.232	14882000	184	15.70	15.12	15.08	15.23	14.54	14.28	14.59	14.28
	1.135	0.86	1.81	P70255	Nuclear factor 1 C-type	Nfic	1540	3	3	3	14.1	14.1	14.1	48.768	0.0002	6.1073	1774500	11	14.35	12.86	14.03	14.36	12.79	12.77	13.54	13.06
	1.864	0.86	1.81	Q8QZV7	Protein asunder homolog	Asun	3736	5	5	5	7.4	7.4	7.4	82.779	0.0002	5.6302	2542800	37	14.00	14.15	14.70	14.66	13.35	13.11	13.75	13.85
	2.759	0.86	1.81	Q6PHN9;Q9	Ras-related protein Rab-35	Rab35	2640	12	12	11	64.7	64.7	59.2	23.025	0.0000	151.9	47404000	340	17.54	17.00	16.91	17.11	16.30	16.20	16.50	16.13
	1.006	0.86	1.81	Q3TUH1	Phosphatidate cytidylyltransferase, mitochondrial	Tamm41	1876	8	8	8	36.8	36.8	36.8	37.827	0.0000	46.946	5348100	48	14.82	13.92	14.70	14.27	13.70	13.08	14.61	12.89
+	3.335	0.86	1.81	P58281	Dynamin-like 120 kDa protein, mitochondrial;Dynamin-like 120 kDa prot	Opa1	1313	54	54	54	61.6	61.6	61.6	111.34	0.0000	323.31	117220000	903	17.00	16.70	17.01	16.95	16.03	15.89	16.35	15.95
+	3.078	0.86	1.81	Q9R1P3	Proteasome subunit beta type-2	Psmb2	5655	17	17	17	52.7	52.7	52.7	22.906	0.0000	98.178	124730000	546	18.38	18.25	18.30	18.45	17.66	17.25	17.77	17.27
	1.111	0.86	1.81	Q99LT0	Protein dpy-30 homolog	Dpy30	4446	3	3	3	47.5	47.5	47.5	11.213	0.0000	10.824	3151700	65	14.36	15.34	13.76	14.48	14.27	13.47	13.13	13.63
	1.713	0.86	1.81	Q9ESZ8	General transcription factor II-I	Gtf2i	5352	13	13	12	15.8	15.8	13.9	112.26	0.0000	17.648	7832500	84	14.89	14.38	14.86	14.98	13.68	13.71	14.62	13.68
	1.980	0.86	1.81	Q9EQQ9	Protein O-GlcNAcase	Mgea5	5287	9	9	9	13.4	13.4	13.4	103.16	0.0000	40.826	10713000	104	16.53	15.58	16.28	16.14	15.35	15.18	15.56	15.01
	0.848	0.86	1.81	Q9CXE7	Transmembrane emp24 domain-containing protein 5	Tmed5	4753	4	4	4	17	17	17	26.172	0.0000	223.7	17076000	84	17.02	16.72	16.43	16.21	16.09	16.36	16.18	14.32
	1.078	0.86	1.81	Q91VC9	Growth hormone-inducible transmembrane protein	Ghitm	4056	7	7	7	14.5	14.5	14.5	37.275	0.0000	38.004	5948000	51	15.19	14.92	15.77	14.00	13.87	13.73	14.30	14.54
	1.071	0.86	1.81	Q8BYU6	Torsin-1A-interacting protein 2	Tor1aip2	3306	7	7	7	21.5	21.5	21.5	54.495	0.0000	50.776	5946800	56	13.81	14.34	13.45	13.46	13.39	13.09	13.31	11.85
	1.883	0.85	1.81	P54823	Probable ATP-dependent RNA helicase DDX6	Ddx6	1261	22	22	22	65	65	65	54.191	0.0000	297	56170000	295	17.33	16.98	17.30	17.36	16.40	15.75	16.62	16.79
	1.289	0.85	1.81	Q3U2P1	Protein transport protein Sec24A	Sec24a	1912	8	8	8	11.2	11.2	11.2	118.78	0.0000	12.422	5831100	44	14.52	14.65	15.68	15.52	13.69	14.45	14.53	14.28
	1.027	0.85	1.81	Q6PER3	Microtubule-associated protein RP/EB family member 3	Mapre3	2625	4	3	3	21.7	18.1	18.1	31.966	0.0000	9.5366	2265500	18	13.78	13.78	14.66	15.09	14.11	13.73	13.22	12.85
	1.639	0.85	1.81	Q78PG9	Coiled-coil domain-containing protein 25	Ccdc25	2727	6	6	6	23.6	23.6	23.6	24.48	0.0000	27.939	6484200	88	15.31	14.58	15.68	15.40	14.67	14.17	14.65	14.08
	1.553	0.85	1.80	P98078	Disabled homolog 2	Dab2	1685	19	19	19	33.4	33.4	33.4	82.311	0.0000	293.8	29339000	316	15.82	15.84	15.67	16.05	15.44	15.38	14.96	14.20
	2.826	0.85	1.80	Q3TFQ1	SPRY domain-containing protein 7	Spryd7	1839	6	6	6	35.7	35.7	35.7	21.681	0.0000	24.985	4796800	75	14.75	14.40	14.71	14.81	13.90	14.12	13.74	13.53
	0.766	0.85	1.80	Q9WTX8	Mitotic spindle assembly checkpoint protein MAD1	Mad1l1	5693	9	9	9	17.3	17.3	17.3	83.54	0.0000	60.005	6467900	112	14.38	14.25	15.40	14.81	14.43	13.97	14.58	12.46

	2.024	0.85	1.80	Q91VJ1	Interferon-inducible protein AIM2	Aim2	4064	9	9	9	41	41	41	40.155	0.0000	62.365	5788100	60	14.85	14.75	15.15	15.09	13.54	14.48	14.10	14.33
	2.196	0.85	1.80	Q8R3S6	Exocyst complex component 1	Exoc1	3857	26	26	26	41.9	41.9	41.9	101.89	0.0000	246.09	27758000	249	16.94	16.17	16.46	16.15	15.42	15.41	15.76	15.72
	0.900	0.85	1.80	Q3UH93	Plexin-D1	Plxnd1	1967	14	14	14	9.2	9.2	9.2	211.61	0.0000	43.683	18333000	96	16.58	15.73	17.70	17.64	15.86	15.98	16.17	16.24
	0.925	0.85	1.80	Q8R1J9;P0(	Torsin-2A	Tor2a	3790	3	3	3	14	14	14	35.897	0.0000	42.155	1562600	12	13.37	13.87	14.69	13.46	12.89	13.35	12.05	13.71
	1.567	0.85	1.80	Q8K2C9	Very-long-chain (3R)-3-hydroxyacyl-CoA dehydratase 3	Hacd3	3656	5	5	5	17.7	17.7	17.7	43.131	0.0000	46.94	28758000	83	17.52	16.85	17.86	17.79	16.21	16.69	17.08	16.65
	0.277	0.85	1.80	P06339	H-2 class I histocompatibility antigen, D-37 alpha chain	H2-T23	657	7	6	6	26.9	24.6	24.6	40.875	0.0000	11.34	14735000	37	16.48	15.64	17.10	15.99	17.69	17.37	12.73	14.03
	1.471	0.85	1.80	Q5PRF0	HEAT repeat-containing protein 5A	Heatr5a	2123	16	16	16	12.4	12.4	12.4	219.89	0.0000	182.45	9875800	139	15.53	14.88	15.02	14.83	14.44	13.80	14.86	13.77
	1.694	0.85	1.80	O35604	Niemann-Pick C1 protein	Npc1	385	11	11	11	8.2	8.2	8.2	142.88	0.0000	39.411	57186000	197	17.52	17.55	18.56	18.20	17.13	16.85	17.29	17.18
	1.496	0.85	1.80	Q9JJK2	LanC-like protein 2	Lancl2	5430	8	8	8	26.7	26.7	26.7	50.777	0.0000	89.079	19173000	144	16.16	15.95	16.50	16.41	15.53	15.43	16.01	14.67
	1.014	0.85	1.80	Q9Z2R6	Protein unc-119 homolog A	Unc119	5863	5	4	4	29.6	25.8	25.8	27.01	0.0000	22.925	3379000	29	14.49	14.44	15.31	14.74	14.04	14.06	14.65	12.84
	2.168	0.85	1.80	Q9WVL3	Solute carrier family 12 member 7	Slc12a7	5756	10	10	10	12.4	12.4	12.4	119.48	0.0000	91.93	7727000	108	15.24	14.73	14.87	15.06	14.59	14.22	13.90	13.80
	2.227	0.85	1.80	Q5SSZ5	Tensin-3	Tns3	2141	40	40	37	48	48	46.2	155.59	0.0000	323.31	109350000	758	17.81	17.18	18.00	17.76	16.73	16.83	17.12	16.68
+	3.478	0.84	1.80	Q8BMP6	Golgi resident protein GCP60	Acbd3	3145	11	11	11	30.7	30.7	30.7	60.18	0.0000	186.49	29304000	213	16.94	16.65	16.86	16.94	16.10	15.81	16.22	15.89
+	3.289	0.84	1.79	Q9CY27	Very-long-chain enoyl-CoA reductase	Tecr	4780	20	20	20	38.3	38.3	38.3	36.09	0.0000	89.824	119020000	517	18.78	18.33	18.74	18.65	17.85	17.73	17.93	17.61
	2.754	0.84	1.79	Q8C129	Leucyl-cystinyl aminopeptidase	Lnpep	3350	23	23	22	28.2	28.2	25.5	117.3	0.0000	157.96	41905000	326	16.84	16.65	16.80	16.45	16.15	15.72	15.95	15.56
	1.340	0.84	1.79	Q8K2L8	Trafficking protein particle complex subunit 12	Trappc12	3665	8	8	8	16.2	16.2	16.2	87.693	0.0000	49.236	11535000	79	16.38	16.17	16.95	16.92	15.43	16.25	16.20	15.17
	1.150	0.84	1.79	Q9DBC3	Cap-specific mRNA (nucleoside-2-O-)-methyltransferase 1	Cmtr1	5142	15	15	15	21.1	21.1	21.1	95.675	0.0000	42.837	14891000	152	15.76	14.91	15.56	15.25	14.79	14.85	14.96	13.53
	2.709	0.84	1.79	Q8BYL4	Tyrosine--tRNA ligase, mitochondrial	Yars2	3303	9	9	9	26.9	26.9	26.9	52.597	0.0000	27.726	5561000	85	14.69	14.54	15.09	14.97	13.71	14.15	14.12	13.94
	0.433	0.84	1.79	Q9R1Z7	6-pyruvoyl tetrahydrobiopterin synthase	Pts	5666	6	6	6	41	41	41	16.188	0.0002	5.8078	9704200	29	15.24	16.12	14.83	16.04	15.87	12.91	13.81	16.26
	0.440	0.84	1.79	E9Q735	Ubiquitin conjugation factor E4 A	Ube4a	244	8	8	8	14.2	14.2	14.2	118.2	0.0000	81.573	3920600	36	14.31	12.98	16.46	14.94	12.50	14.11	14.20	14.52
	1.114	0.84	1.79	Q9CXL3	Uncharacterized protein C7orf50 homolog		4761	8	8	8	37.4	37.4	37.4	22.168	0.0000	19.47	1693100	16	13.36	13.45	14.26	13.26	12.68	12.48	12.16	13.65
	0.345	0.84	1.79	P41233	ATP-binding cassette sub-family A member 1	Abca1	1067	7	7	7	5.4	5.4	5.4	253.91	0.0000	9.4724	15293000	22	17.82	16.94	16.74	16.55	16.63	13.22	17.20	17.63
	1.302	0.84	1.79	Q9DC50	Peroxisomal carnitine O-octanoyltransferase	Crot	5193	12	12	11	28.1	28.1	24.7	70.264	0.0000	53.199	12076000	111	14.70	15.28	15.84	15.41	14.59	14.04	15.12	14.13
	1.254	0.84	1.79	Q91VM9	Inorganic pyrophosphatase 2, mitochondrial	Ppa2	4069	6	6	6	27	27	27	38.114	0.0000	27.222	6767900	68	15.68	15.55	15.13	15.21	14.58	13.87	15.43	14.32
	2.160	0.84	1.79	Q3UQN2	F-BAR domain only protein 2	Fcho2	2019	24	24	24	43.5	43.5	43.5	88.733	0.0000	323.31	34313000	318	16.78	16.36	17.18	16.88	15.98	16.22	16.02	15.63
	0.876	0.84	1.79	Q6P3E7	Histone deacetylase 10	Hdac10	2562	3	3	3	6.3	6.3	6.3	72.108	0.0002	5.9251	2565900	33	14.68	14.51	14.85	14.97	12.54	14.56	14.51	14.05
	0.972	0.84	1.79	P06240	Proto-oncogene tyrosine-protein kinase LCK	Lck	656	2	1	1	2.8	2.8	2.8	57.942	0.0008	4.0969	1462500	27	13.42	13.08	14.92	13.23	12.58	13.11	12.90	12.69
	2.397	0.84	1.79	Q8BWZ3	N-alpha-acetyltransferase 25, NatB auxiliary subunit	Naa25	3273	21	21	21	30.7	30.7	30.7	111.71	0.0000	69.589	18022000	215	15.91	15.81	15.36	15.34	14.65	14.63	15.09	14.69
	1.253	0.84	1.79	P01027	Complement C3;Complement C3 beta chain;C3-beta-c;Complement C3	C3	617	23	22	22	20.4	20	20	186.48	0.0000	76.043	17835000	93	17.13	16.52	16.43	16.04	16.49	15.47	15.28	15.52
	1.858	0.84	1.79	Q99LC2	Cleavage stimulation factor subunit 1	Cstf1	4415	10	10	10	34.6	34.6	34.6	48.381	0.0000	102.7	18786000	174	15.75	15.21	16.21	16.08	15.11	14.87	15.16	14.76
	1.979	0.84	1.79	O35075	Down syndrome critical region protein 3 homolog	Dscr3	333	6	6	6	30.3	30.3	30.3	32.97	0.0000	104.97	23637000	183	17.37	16.77	17.37	17.06	16.45	16.66	16.28	15.82
	2.337	0.84	1.78	Q60737	Casein kinase II subunit alpha	Csnk2a1	2199	18	18	18	53.7	53.7	53.7	45.133	0.0000	323.31	81820000	501	17.73	17.25	17.04	17.26	16.53	16.69	16.58	16.14
	1.999	0.84	1.78	P0C6B7	Immunoglobulin superfamily member 6	Igsf6	706	7	7	7	24.1	24.1	24.1	26.354	0.0000	80.882	21114000	154	17.27	16.40	17.08	17.09	15.95	15.93	16.16	16.45
	2.228	0.83	1.78	Q07417	Short-chain specific acyl-CoA dehydrogenase, mitochondrial	Acads	1756	18	18	18	58.7	58.7	58.7	44.889	0.0000	323.31	131260000	608	18.73	18.34	18.88	18.91	18.08	17.56	18.19	17.69
	3.255	0.83	1.78	Q01405	Protein transport protein Sec23A	Sec23a	1708	29	26	26	56.6	52.9	52.9	86.161	0.0000	323.31	49539000	395	17.38	17.05	16.90	17.24	16.25	16.19	16.51	16.28
+	3.502	0.83	1.78	P11499	Heat shock protein HSP 90-beta	Hsp90ab1	745	83	83	60	78.2	78.2	65.2	83.28	0.0000	323.31	1714500000	3337	20.87	20.51	20.67	20.81	19.99	19.85	20.01	19.66
+	3.718	0.83	1.78	Q9JL62	Glycolipid transfer protein	Gltp	5473	10	10	10	49.8	49.8	49.8	23.689	0.0000	61.053	79385000	350	19.07	18.75	19.15	19.05	18.09	18.15	18.32	18.13
	0.578	0.83	1.78	Q78WZ7	DNA-directed RNA polymerase I subunit RPA43	Twistnb	2729	2	2	2	8.2	8.2	8.2	36.721	0.0004	4.6358	2775000	30	14.39	14.48	14.69	14.73	11.74	14.31	14.66	14.24
	2.505	0.83	1.78	Q9JHS4	ATP-dependent Clp protease ATP-binding subunit clpX-like, mitochondr	Clpx	5372	18	18	18	42.7	42.7	42.7	69.228	0.0000	120.34	16260000	262	15.25	14.70	15.32	15.25	14.20	14.11	14.58	14.29
	2.674	0.83	1.78	P16546	Spectrin alpha chain, non-erythrocytic 1	Sptan1	811	42	42	42	25.2	25.2	25.2	284.59	0.0000	299.32	31711000	407	16.00	15.59	15.64	16.03	14.70	14.93	15.08	15.22
	0.237	0.83	1.78	Q9JKJ9	24-hydroxycholesterol 7-alpha-hydroxylase	Cyp39a1	5452	1	1	1	2.8	2.8	2.8	53.574	0.0046	2.3954	10979000	34	13.65	17.11	16.08	16.14	16.78	13.08	17.22	12.56
	2.982	0.83	1.78	Q01965	T-lymphocyte surface antigen Ly-9	Ly9	1713	18	18	18	31.2	31.2	31.2	73.142	0.0000	323.31	61907000	394	17.74	17.29	17.64	17.73	16.78	16.65	17.03	16.62
	2.329	0.83	1.78	Q791V5	Mitochondrial carrier homolog 2	Mtch2	2734	14	14	14	50.8	50.8	50.8	33.499	0.0000	203.1	157010000	471	20.08	19.39	19.88	20.09	19.13	18.74	19.17	19.08
	1.465	0.83	1.78	Q6NS46	Protein RRP5 homolog	Pdcd11	2523	14	14	13	12.8	12.8	12.4	207.78	0.0000	81.04	10727000	139	15.08	14.49	15.68	15.48	14.44	14.48	14.59	13.90
	1.565	0.83	1.78	Q924T7	E3 ubiquitin-protein ligase RNF31	Rnf31	4301	14	14	14	20.2	20.2	20.2	119.31	0.0000	95.755	17989000	181	16.53	15.72	16.22	16.16	15.56	14.99	15.87	14.90
	2.062	0.83	1.78	Q80UP5	Ankyrin repeat domain-containing protein 13A	Ankrd13a	2835	9	9	9	19.2	19.2	19.2	67.177	0.0000	29.678	8624200	143	15.21	14.85	15.75	15.27	14.28	14.30	14.77	14.40
	2.425	0.83	1.78	Q8K2V1	Serine/threonine-protein phosphatase 4 regulatory subunit 1	Ppp4r1	3674	13	13	13	17.8	17.8	17.8	106.3	0.0000	31.539	21106000	195	17.11	16.56	17.25	17.08	16.12	16.27	16.37	15.92
	2.931	0.83	1.77	Q6PB66	Leucine-rich PPR motif-containing protein, mitochondrial	Lrpprc	2602	65	65	65	53.7	53.7	53.7	156.61	0.0000	323.31	166370000	1158	17.46	17.32	17.56	17.55	16.73	16.37	16.97	16.52
	2.920	0.83	1.77	Q9ERE7	LDLR chaperone MESD	Mesdc2	5306	6	6	6	27.7	27.7	27.7	25.206	0.0000	18.488	9529200	90	15.78	15.45	15.73	15.86	14.76	14.89	15.19	14.67
	1.255	0.83	1.77	Q9QYI4	DnaJ homolog subfamily B member 12	Dnajb12	5574	10	10	10	34.3	34.3	34.3	41.987	0.0000	53.79	7056100	74	14.91	13.96	14.28	14.75	13.04	13.64	13.56	14.36
	3.087	0.83	1.77	Q9D5V5	Cullin-5	Cul5	4986	27	27	27	40.3	40.3	40.3	90.973	0.0000	92.756	24174000	199	15.85	15.55	15.88	15.98	14.87	14.82	15.25	15.01
	1.819	0.82	1.77	Q61334	B-cell receptor-associated protein 29	Bcap29	2292	16	16	16	36.7	36.7	36.7	27.964	0.0000	71.382	37425000	318	16.52	15.86	16.37	16.57	15.05	15.38	15.84	15.77
	2.978	0.82	1.77	Q91WT8;P8	RNA-binding protein 47	Rbm47	4128	14	14	14	45.4	45.4	45.4	64.061	0.0000	153.25	35592000	264	17.10	17.10	17.15	17.26	16.40	16.51	16.48	15.93
	1.661	0.82	1.77	Q9ES97	Reticulon-3	Rtn3	5333	5	5	5	7.7	7.7	7.7	103.88	0.0000	198.57	65293000	239	18.61	17.71	18.35	18.67	17.18	17.36	17.87	17.65
	2.451	0.82	1.77	Q3TZZ7	Extended synaptotagmin-2	Esyt2	1893	24	24	24	38.1	38.1	38.1	94.138	0.0000	146.15	34612000	261	15.59	15.55	15.94	15.87	14.97	14.72	15.31	14.66
	0.670	0.82	1.77	Q69ZS7	HBS1-like protein	Hbs1l	2492	7	7	7	14.2	14.2	14.2	75.1	0.0000	22.163	4945400	59	15.05	14.78	13.60	14.55	14.09	14.36	14.05	12.19
	1.093	0.82	1.77	Q8BZ03	Serine/threonine-protein kinase D2	Prkd2	3309	3	3	3	4.9	4.9	4.9	96.541	0.0015	3.3737	1090500	16	14.12	12.74	13.48	13.49	12.24	12.98	13.21	12.12
	2.086	0.82	1.77	A2A432	Cullin-4B	Cul4b	5	13	9	9	20.2	15.2	15.2	110.7	0.0000	106.53	5350500	89	14.83	14.44	14.70	14.42	13.35	13.72	14.26	13.77
	2.730	0.82	1.77	P09528	Ferritin heavy chain;Ferritin heavy chain, N-terminally processed	Fth1	698	15	15	15	78	78	78	21.066	0.0000	323.31	228540000	655	18.81	18.59	18.97	19.25	17.90	18.22	18.08	18.13
	0.983	0.82	1.77	Q3U0B3	Dehydrogenase/reductase SDR family member 11	Dhrs11	1894	3	3	3	21.5	21.5	21.5	28.274	0.0000	18.875	1517500	12	13.82	13.87	13.57	13.69	11.86	12.75	13.18	13.88
	3.225	0.82	1.76	Q6P1F6;Q8I	Serine/threonine-protein phosphatase 2A 55 kDa regulatory subunit B a	Ppp2r2a	2554	18	18	13	50.1	50.1	38	51.691	0.0000	225.67	69068000	398	17.88	17.48	17.46	17.46	16.90	16.73	16.80	16.57
	1.877	0.82	1.76	P31324	cAMP-dependent protein kinase type II-beta regulatory subunit	Prkar2b	987	14	11	11	46.4	36.5	36.5	46.167	0.0000	60.451	9547900	128	15.69	15.22	14.99	14.95	14.61	13.93	14.41	14.62
	2.931	0.82	1.76	Q9Z2G9	Oxidoreductase HTATIP2	Htatip2	5852	20	20	20	67.4	67.4	67.4	26.87	0.0000	64.559	43431000	258	17.28	16.91	17.17	17.31	16.44	16.07	16.30	16.58
	1.638	0.82	1.76	Q80WG5	Volume-regulated anion channel subunit LRRC8A	Lrrc8a	2861	7	7	7	11.2	11.2	11.2	94.118	0.0000	19.704	6520300	58	14.94	14.77	15.01	15.42	14.53	14.04	14.65	13.65
	2.999	0.82	1.76	O88879	Apoptotic protease-activating factor 1	Apaf1	582	28	28	28	27.5	27.5	27.5	141	0.0000	115.68	30372000	291	16.51	16.28	16.39	16.30	15.63	15.27	15.87	15.46
	2.822	0.82	1.76	Q8BWQ6	UPF0505 protein C16orf62 homolog		3263	18	18	18	24.2	24.2	24.2	109.08	0.0000	102.91	20827000	154	16.99	16.57	16.74	16.77	15.75	15.77	16.26	16.02
	1.867	0.81	1.76	Q99K51	Plastin-3	Pls3	4363	26	21	19	50	43.8	39.7	70.741	0.0000	323.31	58686000	641	16.52	16.45	17.01	17.21	16.39	16.00	15.85	15.71
	1.678	0.81	1.76	Q31125	Zinc transporter SLC39A7	Slc39a7	1806	5	5	5	12.8	12.8	12.8	50.656	0.0000	47.57	17731000	92	16.44	16.30	15.70	16.44	15.99	15.22	15.25	15.17
	2.276	0.81	1.75	Q8BNV1	tRNA (uracil-5-)-methyltransferase homolog A	Trmt2a	3156	14	14	14	34.5	34.5	34.5	63.301	0.0000	57.679	12703000	161	15.66	15.22	15.28	15.43	14.82	14.21	14.89	14.42
	2.629	0.81	1.75	Q6P5E4	UDP-glucose:glycoprotein glucosyltransferase 1	Uggt1	2574	76	76	76	61.1	61.1	61.1	176.43	0.0000	323.31	278110000	1526	18.92	18.36	18.77	18.79	17.99	17.64	18.14	17.83
	1.057	0.81	1.75	P47857	ATP-dependent 6-phosphofructokinase, muscle type	Pfkm	1128	16	10	10	26.8	20.1	20.1	85.268	0.0000	76.001	7793800	98	15.03	14.88	14.45	14.75	14.24	13.91	14.76	12.97
	1.188	0.81	1.75	Q8R480	Nuclear pore complex protein Nup85	Nup85	3866	11	11	11	20.6	20.6	20.6	74.775	0.0000	86.218	12429000	132	15.74	14.88	15.24	15.70	14.67	15.28	13.85	14.54
	1.914	0.81	1.75	Q8K245		Uvrag	3644	9	9	9	14.6	14.6	14.6	77.524	0.0000	15.236	4732400	52	14.58	14.22	15.11	14.96	14.14	13.99	13.89	13.63
	1.490	0.80	1.75	Q793I8	TRAF-interacting protein with FHA domain-containing protein A	Tifa	2735	5	5	5	27.7	27.7	27.7	21.56	0.0000	22.12	8119000	43	15.42	15.69	16.45	16.05	15.43	14.82	15.40	14.74
	1.242	0.80	1.75	Q9Z2H5	Band 4.1-like protein 1	Epb41l1	5853	9	9	9	14.6	14.6	14.6	98.314	0.0000	42.498	5428900	82	15.43	15.00	15.05	14.91	14.47	13.34	14.60	14.75
	0.781	0.80	1.75	Q9JJT0	RNA 3-terminal phosphate cyclase-like protein	Rcl1	5434	3	3	3	10.7	10.7	10.7	40.84	0.0000	7.6851	4876200	42	15.04	14.84	15.28	15.32	15.53	14.01	14.57	13.16
	0.447	0.80	1.74	Q6PGH1	Protein BUD31 homolog	Bud31	2638	10	10	10	60.4	60.4	60.4	17	0.0000	19.105	13891000	73	16.35	16.58	16.18	15.95	16.19	16.04	16.52	13.11
	1.054	0.80	1.74	Q62095;Q61	ATP-dependent RNA helicase DDX3Y	Ddx3y	2356	35	3	3	47.3	6.1	6.1	73.427	0.0000	63.04	46787000	155	18.73	18.41	17.80	17.72	17.95	16.50	17.60	17.41
	1.902	0.80	1.74	Q9D5T0	ATPase family AAA domain-containing protein 1	Atad1	4985	9	9	9	31.9	31.9	31.9	40.744	0.0000	97.208	18570000	211	15.64	15.42	15.40	15.54	14.85	14.87	15.03	14.05
	1.663	0.80	1.74	P18052	Receptor-type tyrosine-protein phosphatase alpha	Ptpra	833	15	14	14	28.8	26.7	26.7	93.696	0.0000	105.77	15395000	148	16.17	15.95	16.09	16.24	14.68	15.28	15.91	15.39
	2.738	0.80	1.74	Q9D8W5	26S proteasome non-ATPase regulatory subunit 12	Psmd12	5079	35	35	34	76.1	76.1	73.7	52.895	0.0000	323.31	118390000	839	17.93	17.51	17.63	17.75	17.09	16.59	17.11	16.83
	2.409	0.80	1.74	Q8BK67	Protein RCC2	Rcc2	3096	29	29	29	62.7	62.7	62.7	55.983	0.0000	178.71	87742000	617	17.83	17.22	17.58	17.63	17.03	16.86	16.71	16.46
	2.988	0.80	1.74	Q8CFX1	GDH/6PGL endoplasmic bifunctional protein;Glucose 1-dehydrogenase	H6pd	3493	15	15	15	28.1	28.1	28.1	88.927	0.0000	147.25	40679000	240	18.39	18.22	18.44	18.13	17.67	17.31	17.30	17.72
	1.967	0.80	1.74	Q8C0Z1	Protein ITFG3	Itfg3	3349	6	6	6	17.7	17.7	17.7	60.575	0.0000	16.927	7349700	46	14.74	14.97	15.36	14.98	14.56	14.07	14.44	13.79
	1.342	0.80	1.74	Q9DCU6	39S ribosomal protein L4, mitochondrial	Mrpl4	5233	7	7	7	29.9	29.9	29.9	33.073	0.0000	18.634	8591500	126	15.48	14.68	15.49	15.81	14.63	14.70	14.96	13.99
	0.920	0.80	1.74	P97412	Lysosomal-trafficking regulator	Lyst	1644	12	12	12	4.9	4.9	4.9	425.28	0.0000	19.243	11570000	33	16.79	15.67	16.99	16.94	15.41	15.37	16.70	15.73
	2.969	0.80	1.74	P24547	Inosine-5-monophosphate dehydrogenase 2	Impdh2	899	31	31	29	57.8	57.8	56	55.814	0.0000	314.21	88459000	626	17.29	16.91	17.39	17.21	16.40	16.21	16.63	16.38
	2.671	0.80	1.74	Q8BIJ6	Isoleucine--tRNA ligase, mitochondrial	Iars2	3061	29	29	29	34.5	34.5	34.5	112.8	0.0000	239.49	70373000	627	17.15	16.79	17.39	17.21	16.30	16.28	16.60	16.19
	1.708	0.80	1.74	P13808	Anion exchange protein 2	Slc4a2	769	8	8	8	10.3	10.3	10.3	136.81	0.0000	36.311	8780200	72	16.13	15.32	16.27	16.20	15.26	15.39	14.84	15.24
	0.569	0.80	1.74	Q8VCD5	Mediator of RNA polymerase II transcription subunit 17	Med17	3916	4	4	4	12.6	12.6	12.6	72.461	0.0000	29.518	1054800	30	13.50	12.79	12.97	13.89	13.89	11.56	13.10	11.41
	2.031	0.80	1.74	Q91YR9;RE	Prostaglandin reductase 1	Ptgr1	4176	22	22	22	72.3	72.3	72.3	35.56	0.0000	291.04	301900000	984	19.17	19.01	19.11	19.18	18.07	17.88	18.76	18.57
	1.099	0.80	1.74	Q80UK0	SEC14 domain and spectrin repeat-containing protein 1	Sestd1	2830	7	7	7	14.2	14.2	14.2	79.375	0.0000	22.659	6206900	61	15.61	15.03	16.05	14.74	14.47	13.92	14.94	14.91
	2.997	0.80	1.74	Q60605	Myosin light polypeptide 6	Myl6	2185	10	10	8	67.5	67.5	58.9	16.93	0.0000	44.149	95055000	270	18.70	19.13	18.59	18.85	18.20	17.98	17.89	18.01
	2.131	0.79	1.73	Q8BVE3	V-type proton ATPase subunit H	Atp6v1h	3237	32	32	32	64.2	64.2	64.2	55.854	0.0000	323.31	279300000	1052	19.73	19.25	19.27	19.08	18.65	18.26	18.89	18.35
	3.175	0.79	1.73	Q91VH6	Protein MEMO1	Memo1	4062	12	12	12	63.6	63.6	63.6	33.692	0.0000	117.58	30102000	282	16.85	16.45	16.79	16.58	15.96	16.00	15.90	15.64
	2.247	0.79	1.73	Q8BMS9	Ras association domain-containing protein 2	Rassf2	3150	16	16	14	56.7	56.7	48.8	37.973	0.0000	62.017	35078000	234	17.32	16.76	17.05	16.99	16.42	15.81	16.47	16.23
	0.365	0.79	1.73	Q8R2Q8	Bone marrow stromal antigen 2	Bst2	3814	5	5	5	17.4	17.4	17.4	19.152	0.0000	27.353	6384300	50	15.22	15.02	14.54	14.22	15.83	11.45	14.23	14.32
	1.716	0.79	1.73	Q9CQC8	Maspardin	Spg21	4584	13	13	13	47.4	47.4	47.4	34.952	0.0000	97.67	26240000	173	16.78	16.80	17.51	17.17	16.31	16.62	16.40	15.77
	0.811	0.79	1.73	Q3TCH7	Cullin-4A	Cul4a	1826	15	15	10	25.3	25.3	18.8	87.752	0.0000	54.122	11488000	95	15.51	15.31	14.45	15.01	14.74	13.00	14.55	14.81
	1.788	0.79	1.73	Q8BFV2	PCI domain-containing protein 2	Pcid2	2949	9	9	9	28.8	28.8	28.8	46.132	0.0000	43.356	9425900	71	15.74	15.28	15.09	15.41	14.93	14.28	14.93	14.21
	4.206	0.79	1.73	Q9EP69	Phosphatidylinositide phosphatase SAC1	Sacm1l	5243	30	30	30	54.9	54.9	54.9	66.943	0.0000	323.31	106720000	721	17.83	17.57	17.76	17.70	16.93	16.77	16.99	17.02
	2.228	0.79	1.73	Q91VN6	Probable ATP-dependent RNA helicase DDX41	Ddx41	4071	14	14	13	30.9	30.9	29.6	69.819	0.0000	68.532	10557000	141	15.27	15.12	15.27	15.54	14.74	14.53	14.74	14.03
	1.010	0.79	1.73	P15105	Glutamine synthetase	Glul	790	8	8	8	23.1	23.1	23.1	42.119	0.0000	73.774	6942200	61	15.50	15.04	15.57	15.36	14.64	14.99	15.21	13.48
	1.175	0.79	1.73	P97770	THUMP domain-containing protein 3	Thumpd3	1669	9	9	9	21.4	21.4	21.4	56.431	0.0000	34.872	9861200	99	15.06	14.79	15.08	15.12	14.70	13.38	14.87	13.95
	2.689	0.79	1.73	P61164	Alpha-centractin	Actr1a	1373	29	29	16	70.2	70.2	42.3	42.613	0.0000	323.31	193400000	769	19.14	18.66	19.08	18.70	18.30	18.19	18.04	17.90
	1.207	0.79	1.73	Q61144	Presenilin-2;Presenilin-2 NTF subunit;Presenilin-2 CTF subunit	Psen2	2259	3	2	2	10.5	8.5	8.5	49.983	0.0000	35.075	1701500	15	14.12	13.88	14.85	13.84	12.89	13.96	13.03	13.64
	1.647	0.79	1.72	Q9D9V3	Ethylmalonyl-CoA decarboxylase	Echdc1	5103	8	8	8	39.4	39.4	39.4	35.467	0.0000	41.455	5954200	47	14.94	14.82	15.08	15.06	14.28	14.53	13.45	14.49
	1.158	0.79	1.72	P97499	Telomerase protein component 1	Tep1	1665	16	16	16	10.1	10.1	10.1	291.46	0.0000	95.473	10900000	120	15.54	14.61	15.45	14.92	14.51	14.38	14.91	13.57
	1.360	0.78	1.72	Q8K0C1	Importin-13	Ipo13	3601	5	5	5	7.8	7.8	7.8	108.23	0.0000	24.911	3630100	55	14.09	14.40	14.19	14.62	13.87	12.85	13.31	14.12
	2.311	0.78	1.72	Q8R3D1	TBC1 domain family member 13	Tbc1d13	3842	11	11	11	39.8	39.8	39.8	46.452	0.0000	47.531	40551000	296	16.73	16.53	17.29	17.02	15.99	16.10	16.30	16.04
	1.644	0.78	1.72	Q8K4Q0	Regulatory-associated protein of mTOR	Rptor	3725	6	6	6	7.7	7.7	7.7	149.47	0.0000	110.44	5964500	83	14.89	14.20	15.09	15.37	14.27	14.13	13.95	14.06
	0.826	0.78	1.72	Q8CAS9	Poly [ADP-ribose] polymerase 9	Parp9	3431	25	25	25	43.9	43.9	43.9	96.658	0.0000	235.63	86341000	280	18.69	17.93	18.06	17.82	18.08	17.61	17.59	16.09
	1.605	0.78	1.72	Q9DC37	Major facilitator superfamily domain-containing protein 1	Mfsd1	5190	7	7	7	9.9	9.9	9.9	51.396	0.0000	34.816	23438000	90	17.82	16.96	16.75	16.93	16.04	16.55	16.34	16.39
	1.141	0.78	1.72	Q64310	Surfeit locus protein 4	Surf4	2428	7	7	7	28.6	28.6	28.6	30.381	0.0000	172.66	25731000	197	16.54	16.16	17.54	17.48	16.13	16.39	16.15	15.91

1.807	0.78	1.72	Q9ER41	Torsin-1B	Tor1b	5294	5	5	5	18.5	18.5	18.5	37.817	0.0000	52.215	3889800	46	13.94	14.12	14.19	14.51	12.87	13.53	13.83	13.41
1.919	0.78	1.72	B2RXR6	Serine/threonine-protein phosphatase 6 regulatory ankyrin repeat subur	Ankrd44	85	32	32	31	35.8	35.8	34.7	107.38	0.0000	156.1	49594000	551	16.63	16.09	16.62	16.79	15.89	15.66	16.09	15.36
0.545	0.78	1.72	Q9CQH3	NADH dehydrogenase [ubiquinone] 1 beta subcomplex subunit 5, mitoc	Ndufb5	4599	6	6	6	30.7	30.7	30.7	21.71	0.0000	39.829	14856000	88	16.27	15.88	15.88	15.16	16.04	13.54	16.06	14.44
2.184	0.78	1.72	Q8VBZ3	Cleft lip and palate transmembrane protein 1 homolog	Clptm1	3902	14	14	14	27.7	27.7	27.7	75.29	0.0000	198.52	51895000	277	17.05	17.12	17.79	17.41	16.54	16.31	16.70	16.70
2.919	0.78	1.71	P50295;P50	Arylamine N-acetyltransferase 2	Nat2	1178	9	9	9	46.2	46.2	46.2	33.701	0.0000	65.58	18901000	127	16.94	16.75	16.82	17.04	15.84	16.32	16.31	15.97
1.600	0.78	1.71	O88738	Baculoviral IAP repeat-containing protein 6	Birc6	564	26	26	26	9.5	9.5	9.5	532.16	0.0000	151.08	31525000	271	17.25	16.76	17.47	17.16	16.34	16.24	16.99	15.96
3.028	0.78	1.71	Q8BHN3	Neutral alpha-glucosidase AB	Ganab	3049	41	41	41	47.1	47.1	47.1	106.91	0.0000	323.31	311200000	1230	18.80	18.60	18.77	19.02	18.23	17.84	18.14	17.88
3.076	0.78	1.71	O55128	Histone deacetylase complex subunit SAP18	Sap18	457	7	7	7	52.9	52.9	52.9	17.595	0.0000	77.541	17669000	139	16.79	16.46	16.45	16.68	15.62	15.70	15.98	15.98
2.641	0.78	1.71	Q80X90	Filamin-B	Flnb	2876	54	47	46	31.1	29.9	29.4	277.82	0.0000	192.54	46813000	424	16.41	16.25	16.48	16.15	15.41	15.69	15.83	15.24
1.041	0.78	1.71	Q8CI33	CWF19-like protein 1	Cwf19l1	3539	9	9	9	21.8	21.8	21.8	60.193	0.0000	47.316	4772900	59	14.78	14.58	14.17	13.77	13.15	12.89	14.22	13.93
0.914	0.78	1.71	Q8BZM1	Glomulin	Glmn	3315	3	3	2	3.9	3.9	3.9	67.755	0.0000	25.165	3366700	51	15.30	14.38	14.63	15.49	14.62	13.17	14.59	14.32
0.742	0.78	1.71	Q9CY28	GTP-binding protein 8	Gtpbp8	4781	3	3	3	17.5	17.5	17.5	31.872	0.0000	11.778	2525700	45	13.22	14.52	15.08	14.38	12.64	13.59	14.26	13.60
0.341	0.77	1.71	Q9EQW7	Kinesin-like protein KIF13A	Kif13a	5290	5	5	5	3.3	3.3	3.3	195.81	0.0000	23.227	5179000	65	14.96	13.30	15.96	15.65	15.26	15.43	13.97	12.10
1.625	0.77	1.71	Q80VI1	E3 ubiquitin-protein ligase TRIM56	Trim56	2850	9	9	9	19.8	19.8	19.8	79.512	0.0000	34.873	7428300	95	15.19	14.69	15.01	15.13	13.57	14.32	14.65	14.38
2.942	0.77	1.71	P62889	60S ribosomal protein L30	Rpl30	1463	13	13	13	82.6	82.6	82.6	12.784	0.0000	84.226	227260000	516	19.87	19.39	19.64	19.49	18.95	18.79	18.95	18.61
2.837	0.77	1.71	Q9QUM9	Proteasome subunit alpha type-6	Psma6	5519	23	23	23	69.5	69.5	69.5	27.372	0.0000	168.53	283110000	1083	19.27	18.82	19.47	19.27	18.43	18.45	18.50	18.37
1.388	0.77	1.71	P53995	Anaphase-promoting complex subunit 1	Anapc1	1244	9	9	9	8.2	8.2	8.2	215.99	0.0000	38.677	7654200	60	15.65	15.12	15.15	15.34	14.59	14.78	15.02	13.77
1.415	0.77	1.71	Q99KW9	T-cell immunomodulatory protein	Itfg1	4398	2	2	2	6.1	6.1	6.1	67.464	0.0000	12.65	1858000	19	14.81	14.58	14.06	13.64	13.56	13.17	13.48	13.79
2.191	0.77	1.71	Q9D0D5	General transcription factor IIE subunit 1	Gtf2e1	4860	5	5	5	17.3	17.3	17.3	49.593	0.0000	77.531	2494700	39	14.71	14.11	14.88	14.62	13.66	14.00	13.91	13.65
1.363	0.77	1.71	P62838	Ubiquitin-conjugating enzyme E2 D2	Ube2d2	1451	5	2	2	48.3	12.2	12.2	16.735	0.0034	2.494	1847400	12	13.05	13.03	13.28	13.52	12.93	12.26	12.86	11.74
0.915	0.77	1.71	Q9CZH3	Proteasome assembly chaperone 3	Psmg3	4829	4	4	4	50.8	50.8	50.8	13.331	0.0000	34.994	12456000	85	16.42	15.52	16.29	16.13	15.59	15.74	15.77	14.19
1.542	0.77	1.71	Q3UKJ7	WD40 repeat-containing protein SMU1;WD40 repeat-containing protein	Smu1	1988	18	18	18	41.9	41.9	41.9	57.543	0.0000	131.58	40524000	332	16.17	16.21	16.81	16.89	15.98	15.70	16.08	15.24
2.279	0.77	1.71	D3Z7P3	Glutaminase kidney isoform, mitochondrial	Gls	215	30	30	30	53.3	53.3	53.3	73.963	0.0000	309.86	78178000	550	17.59	17.32	17.45	17.74	16.63	16.38	17.08	16.93
1.353	0.77	1.71	O08795	Glucosidase 2 subunit beta	Prkcsh	300	9	9	9	15.4	15.4	15.4	58.792	0.0000	22.439	24852000	167	16.64	16.81	16.34	16.93	16.72	15.70	15.75	15.48
0.999	0.77	1.71	Q80WQ6	Inactive rhomboid protein 2	Rhbdf2	2865	7	7	7	10.5	10.5	10.5	93.433	0.0000	16.725	2269100	18	13.11	13.54	14.64	13.45	13.51	12.79	12.90	12.46
2.707	0.77	1.71	Q9D0R2;Q8	Threonine--tRNA ligase, cytoplasmic	Tars	4883	45	45	45	56.4	56.4	56.4	83.355	0.0000	257.64	130280000	862	17.33	17.03	17.42	17.47	16.58	16.58	16.77	16.24
1.203	0.77	1.70	P53612	Geranylgeranyl transferase type-2 subunit beta	Rabggtb	1234	3	3	3	10.6	10.6	10.6	37.803	0.0000	8.1985	6979300	35	15.73	15.02	16.12	16.29	15.39	15.24	14.88	14.57
1.959	0.77	1.70	P97333	Neuropilin-1	Nrp1	1624	16	16	16	25.6	25.6	25.6	103	0.0000	209.55	23227000	189	17.06	16.48	16.93	16.73	16.07	15.65	16.46	15.94
1.772	0.77	1.70	Q9EQU5	Protein SET	Set	5289	8	8	8	29.1	29.1	29.1	33.377	0.0000	91.015	12395000	102	16.10	16.21	15.82	15.96	15.15	15.79	14.73	15.33
1.886	0.77	1.70	Q9D3U0	Putative tRNA pseudouridine synthase Pus10	Pus10	4970	8	8	8	20.7	20.7	20.7	59.71	0.0000	33.882	3845700	42	14.55	13.91	13.87	14.15	13.13	13.52	13.71	13.06
0.831	0.77	1.70	Q9JKY5	Huntingtin-interacting protein 1-related protein	Hip1r	5465	5	4	4	5.5	4.6	4.6	119.43	0.0064	2.0909	65444000	29	18.36	18.34	19.88	18.80	18.63	18.15	18.26	17.28
0.553	0.77	1.70	Q8K221	Arfaptin-2	Arfip2	3642	4	4	4	15.8	15.8	15.8	37.772	0.0000	36.354	2576400	39	14.07	13.37	14.12	14.11	13.14	14.84	12.71	11.91
0.711	0.77	1.70	P70414	Sodium/calcium exchanger 1	Slc8a1	1569	3	3	3	4.9	4.9	4.9	108.03	0.0000	95.951	1351600	30	14.52	13.38	13.08	13.60	12.82	13.95	12.85	11.89
2.423	0.77	1.70	Q8K1Z0	Ubiquinone biosynthesis protein COQ9, mitochondrial	Coq9	3637	8	8	8	34.5	34.5	34.5	35.082	0.0000	100.35	11241000	146	15.76	15.59	15.71	15.90	14.59	14.88	15.31	15.11
1.842	0.77	1.70	P36552	Oxygen-dependent coproporphyrinogen-III oxidase, mitochondrial	Cpox	1043	13	13	13	35.4	35.4	35.4	49.714	0.0000	43.683	13868000	140	15.18	14.74	15.36	15.42	14.20	14.27	14.28	14.90
2.489	0.76	1.70	Q99LR1	Monoacylglycerol lipase ABHD12	Abhd12	4444	30	30	30	73.6	73.6	73.6	45.269	0.0000	323.31	168530000	668	18.66	18.18	18.44	18.31	17.47	17.50	18.01	17.56
0.911	0.76	1.70	P53690	Matrix metalloproteinase-14	Mmp14	1235	8	8	8	14.1	14.1	14.1	65.918	0.0000	15.721	6170600	71	15.08	15.06	15.61	15.85	14.36	15.72	14.48	13.98
0.757	0.76	1.70	Q8BSS9	Liprin-alpha-2	Ppfia2	3192	4	4	4	4.4	4.4	4.4	143.23	0.0002	5.6333	3119600	31	14.42	14.47	14.98	13.76	13.11	13.10	14.91	13.46
0.979	0.76	1.70	Q8R2U0	Nucleoporin SEH1	Seh1l	3821	11	11	11	44.4	44.4	44.4	39.774	0.0000	69.365	13673000	129	15.02	15.38	15.85	15.68	15.22	14.68	15.25	13.72
1.716	0.76	1.70	Q80TL7	Protein MON2 homolog	Mon2	2807	9	9	9	9	9	9	189.08	0.0000	58.786	9242400	83	15.53	15.08	15.67	15.56	15.13	14.17	14.85	14.63
1.531	0.76	1.70	Q9D3G5	Syntaxin-11	Stx11	4966	8	8	8	36.2	36.2	36.2	33.369	0.0000	27.106	18701000	110	16.59	16.55	16.38	16.67	15.88	15.25	16.46	15.54
3.580	0.76	1.70	P61028;REV	Ras-related protein Rab-8B	Rab8b	1367	19	16	14	59.9	54.6	50.7	23.603	0.0000	188.29	120520000	420	19.06	18.75	18.79	18.83	18.03	17.92	18.18	18.25
1.331	0.76	1.70	P70451	Tyrosine-protein kinase Fer	Fer	1576	4	3	3	5.8	4.5	4.5	94.578	0.0000	63.348	1301200	10	13.77	13.87	13.48	13.99	12.45	13.51	12.60	13.50
1.907	0.76	1.70	Q8K2D3	Enhancer of mRNA-decapping protein 3	Edc3	3657	11	11	11	32.1	32.1	32.1	55.957	0.0000	30.791	7669900	113	15.05	14.35	14.62	14.87	13.64	14.02	14.36	13.83
1.574	0.76	1.70	Q8K012	Formin-binding protein 1-like	Fnbp1l	3593	11	11	11	23.8	23.8	23.8	69.885	0.0000	47.143	7067500	87	14.84	14.62	14.46	14.56	14.41	13.96	13.21	13.85
1.553	0.76	1.70	Q8VEE4	Replication protein A 70 kDa DNA-binding subunit;Replication protein A	Rpa1	4002	16	16	16	28.9	28.9	28.9	69.036	0.0000	111.83	23353000	240	15.91	15.36	15.96	16.18	15.23	15.28	15.36	14.50
0.735	0.76	1.70	Q8BMG8	Mitochondrial folate transporter/carrier	Slc25a32	3137	2	2	2	13.3	13.3	13.3	35.048	0.0000	23.891	1380200	29	13.86	13.40	13.37	13.78	12.64	12.73	14.18	11.81
1.739	0.76	1.69	Q9DBB8	Trans-1,2-dihydrobenzene-1,2-diol dehydrogenase	Dhdh	5140	16	16	16	53.8	53.8	53.8	36.3	0.0000	224.29	29937000	282	16.06	15.60	16.12	16.09	15.30	14.85	14.94	15.74
2.653	0.76	1.69	A2A8Z1	Oxysterol-binding protein-related protein 9	Osbpl9	13	17	17	17	26.8	26.8	26.8	83.108	0.0000	56.744	25486000	198	16.63	15.95	16.33	16.40	15.51	15.55	15.71	15.49
2.274	0.76	1.69	Q60790	Ras GTPase-activating protein 3	Rasa3	2208	29	29	29	37.4	37.4	37.4	95.986	0.0000	117.72	32224000	290	15.90	15.54	15.99	16.01	15.04	14.93	15.52	14.92
0.515	0.76	1.69	Q8BW96	Calcium/calmodulin-dependent protein kinase type 1D	Camk1d	3256	9	7	7	24.2	19	19	42.918	0.0000	29.237	4642200	63	15.10	14.92	12.81	13.11	13.30	14.08	12.99	12.53
1.785	0.76	1.69	O54833	Casein kinase II subunit alpha	Csnk2a2	428	23	23	23	64.3	64.3	64.3	41.215	0.0000	225.34	44896000	393	16.86	16.75	16.85	16.70	16.02	16.65	15.89	15.57
1.818	0.76	1.69	Q9CYN9	Renin receptor	Atp6ap2	4804	9	9	9	31.4	31.4	31.4	39.091	0.0000	123.69	24846000	163	16.36	16.12	16.79	16.69	15.62	15.99	16.00	15.31
3.003	0.76	1.69	Q3U1J4	DNA damage-binding protein 1	Ddb1	1903	38	38	38	42.1	42.1	42.1	126.85	0.0000	323.31	71964000	726	17.07	16.66	16.72	16.98	16.19	16.03	16.27	15.91
3.083	0.76	1.69	P45376	Aldose reductase	Akr1b1	1094	27	27	27	69.9	69.9	69.9	35.732	0.0000	323.31	689630000	1095	20.54	20.23	20.58	20.63	19.79	19.88	19.77	19.49
1.338	0.76	1.69	Q99J99	3-mercaptopyruvate sulfurtransferase	Mpst	4335	14	14	14	49.2	49.2	49.2	33.097	0.0000	65.726	18430000	132	16.02	16.07	16.53	16.63	15.69	14.80	15.96	15.78
1.509	0.76	1.69	Q5XG73	Acyl-CoA-binding domain-containing protein 5	Acbd5	2171	11	11	11	26.6	26.6	26.6	56.613	0.0000	41.914	15879000	106	15.58	15.34	16.51	16.07	15.11	15.06	15.32	14.98
3.181	0.76	1.69	B9EJ86	Oxysterol-binding protein	Osbpl8	93	44	44	42	50.4	50.4	49.5	101.27	0.0000	323.31	192960000	1091	18.38	17.93	18.19	18.38	17.41	17.40	17.61	17.43
3.350	0.76	1.69	Q8R010	Aminoacyl tRNA synthase complex-interacting multifunctional protein 2	Aimp2	3742	11	11	11	54.1	54.1	54.1	35.377	0.0000	323.31	43628000	272	17.87	17.45	17.90	17.79	17.05	17.01	17.02	16.90
0.600	0.76	1.69	Q8R2U4	N-terminal Xaa-Pro-Lys N-methyltransferase 1;N-terminal Xaa-Pro-Lys	Ntmt1	3822	3	3	3	15.7	15.7	15.7	25.42	0.0000	8.1252	6145400	85	14.96	15.38	16.31	15.90	15.06	15.31	15.76	13.39
2.635	0.76	1.69	P53811	Phosphatidylinositol transfer protein beta isoform	Pitpnb	1241	15	13	13	53.1	46.9	46.9	31.487	0.0000	40.068	21397000	208	16.19	15.90	16.45	16.06	15.50	15.23	15.61	15.24
1.664	0.76	1.69	P62915	Transcription initiation factor IIB	Gtf2b	1469	8	8	8	39.2	39.2	39.2	34.819	0.0000	54.781	15509000	111	16.34	16.05	16.98	16.88	15.93	15.56	16.04	15.68
2.874	0.76	1.69	P45952	Medium-chain specific acyl-CoA dehydrogenase, mitochondrial	Acadm	1100	26	26	26	61.5	61.5	61.5	46.481	0.0000	323.31	136340000	780	18.71	18.16	18.38	18.45	17.79	17.50	17.78	17.61
2.041	0.76	1.69	Q8C172	Ceramide synthase 6	Cers6	3357	6	6	5	14.6	14.6	11.7	44.812	0.0000	46.264	19390000	149	16.66	16.33	15.92	16.15	15.87	15.28	15.44	15.45
3.704	0.75	1.69	O88958	Glucosamine-6-phosphate isomerase 1	Gnpda1	588	22	22	20	88.6	88.6	84.4	32.549	0.0000	323.31	150310000	592	18.90	18.59	18.79	18.56	17.83	17.99	18.05	17.95
2.013	0.75	1.69	O35969	Guanidinoacetate N-methyltransferase	Gamt	415	9	9	9	52.5	52.5	52.5	26.336	0.0000	60.122	31695000	191	16.23	16.76	16.82	16.79	15.90	16.18	16.00	15.49
2.732	0.75	1.69	Q61102	ATP-binding cassette sub-family B member 7, mitochondrial	Abcb7	2251	31	31	31	46.5	46.5	46.5	82.58	0.0000	249.75	50152000	496	16.18	15.89	15.80	16.11	15.40	14.94	15.41	15.22
3.329	0.75	1.69	P56395;P00	Cytochrome b5	Cyb5a	1285	12	12	12	67.2	67.2	67.2	15.241	0.0000	323.31	133730000	477	18.48	18.30	18.04	18.08	17.51	17.45	17.37	17.55
0.879	0.75	1.69	Q7TMC8		Fuk	2741	5	5	5	6.6	6.6	6.6	119.27	0.0000	9.3532	2017700	20	13.73	13.02	14.11	13.22	11.87	12.75	12.85	13.60
2.298	0.75	1.69	Q7TT50;Q3l	Serine/threonine-protein kinase MRCK beta	Cdc42bpb	2793	39	38	38	27.4	27.4	27.4	194.75	0.0000	133.72	28611000	345	16.02	15.58	16.23	16.06	14.94	15.18	15.43	15.34
2.172	0.75	1.68	Q9JIF0	Protein arginine N-methyltransferase 1	Prmt1	5395	12	12	12	38.8	38.8	38.8	42.435	0.0000	132.69	52902000	319	17.54	17.26	17.46	17.76	17.06	16.39	16.95	16.61
1.009	0.75	1.68	Q4VAA2	Protein CDV3	Cdv3	2067	11	11	10	48.4	48.4	46.3	29.729	0.0000	263.32	7775500	59	12.83	13.79	12.13	13.22	12.27	11.78	12.50	12.42
3.056	0.75	1.68	P06745	Glucose-6-phosphate isomerase	Gpi	661	47	47	37	71.1	71.1	58.8	62.766	0.0000	323.31	1188200000	2285	20.43	20.29	20.38	20.41	19.90	19.42	19.75	19.44
2.759	0.75	1.68	P61027	Ras-related protein Rab-10	Rab10	1366	15	14	13	60.5	55	55	22.541	0.0000	116.66	92571000	288	18.93	18.45	18.73	18.77	18.15	17.69	17.99	18.04
0.600	0.75	1.68	Q69ZN6	N-acetylglucosamine-1-phosphotransferase subunits alpha/beta;N-acety	Gnptab	2487	2	2	2	2.5	2.5	2.5	140.98	0.0000	26.002	2962000	10	15.16	12.67	14.12	13.33	13.73	13.11	12.89	12.56
1.823	0.75	1.68	P17047	Lysosome-associated membrane glycoprotein 2	Lamp2	817	10	10	10	14.2	14.2	14.2	45.681	0.0000	80.008	39421000	227	18.10	17.89	17.27	17.33	17.04	16.87	16.67	17.00
1.807	0.75	1.68	Q8BK63	Casein kinase I isoform alpha	Csnk1a1	3094	8	8	8	23.1	23.1	23.1	38.914	0.0000	142.85	11750000	137	16.39	15.75	15.62	15.60	14.93	15.18	15.40	14.84
2.514	0.75	1.68	Q9CQR6	Serine/threonine-protein phosphatase 6 catalytic subunit;Serine/threonii	Ppp6c	4626	15	15	15	60.3	60.3	60.3	35.159	0.0000	104.12	48077000	363	16.94	16.64	16.97	17.06	16.37	16.12	16.32	15.81
2.099	0.75	1.68	Q91ZE0	Trimethyllysine dioxygenase, mitochondrial	Tmlhe	4194	12	12	12	31.4	31.4	31.4	49.609	0.0000	88.719	19451000	210	15.97	15.89	15.65	15.42	15.10	14.68	15.34	14.82
2.421	0.75	1.68	O88544	COP9 signalosome complex subunit 4	Cops4	544	28	28	28	80.5	80.5	80.5	46.284	0.0000	323.31	98937000	601	17.76	17.51	17.51	17.36	17.10	16.45	16.91	16.70
2.398	0.75	1.68	Q8BHF7	CDP-diacylglycerol--glycerol-3-phosphate 3-phosphatidyltransferase, m	Pgs1	3035	10	10	10	22.4	22.4	22.4	62.488	0.0000	64.122	13837000	138	15.58	15.25	15.74	15.76	14.64	14.65	15.09	14.97
1.932	0.74	1.68	Q9JHI5	Isovaleryl-CoA dehydrogenase, mitochondrial	Ivd	5361	20	20	20	60.1	60.1	60.1	46.325	0.0000	165.62	87453000	525	17.52	17.35	17.98	17.94	16.83	16.67	17.32	16.99
1.756	0.74	1.68	Q8CJG1;Q8	Protein argonaute-1	Ago1	3572	14	9	7	22.4	15.3	12	97.213	0.0000	24.977	4970100	67	14.86	14.29	14.53	14.72	13.38	14.17	13.70	14.16
2.059	0.74	1.67	Q9QUN7	Toll-like receptor 2	Tlr2	5521	26	26	26	32.3	32.3	32.3	89.448	0.0000	165.23	78889000	508	17.68	17.51	17.80	17.72	17.28	16.97	17.07	16.42
4.409	0.74	1.67	P24668	Cation-dependent mannose-6-phosphate receptor	M6pr	902	7	7	7	28.1	28.1	28.1	31.172	0.0000	316.36	96721000	211	19.13	18.92	19.19	19.10	18.38	18.28	18.43	18.27
0.935	0.74	1.67	Q8BY35	FYVE, RhoGEF and PH domain-containing protein 2	Fgd2	3292	9	9	9	20	20	20	74.633	0.0000	14.021	3170000	14	13.50	14.34	14.40	14.27	13.75	12.47	13.28	14.05
1.255	0.74	1.67	Q925N2	Sideroflexin-2	Sfxn2	4310	7	7	7	29.2	29.2	29.2	36.141	0.0000	27.522	8868200	94	14.92	15.36	15.11	15.21	14.83	13.53	14.74	14.52
0.833	0.74	1.67	Q9D920	Loss of heterozygosity 12 chromosomal region 1 protein homolog	Loh12cr1	5090	5	5	5	40.5	40.5	40.5	22.12	0.0000	18.885	2776200	32	13.49	13.90	14.38	14.29	13.68	12.19	14.01	13.20
0.687	0.74	1.67	Q8BMB3	Eukaryotic translation initiation factor 4E type 2	Eif4e2	3131	3	3	3	13.9	13.9	13.9	28.263	0.0000	8.3792	1732100	25	14.57	14.15	13.74	13.86	14.37	12.99	13.85	12.14
2.896	0.74	1.67	Q91VH2	Sorting nexin-9	Snx9	4061	18	18	18	46.1	46.1	46.1	66.545	0.0000	78.504	42059000	228	17.30	17.01	17.46	17.19	16.55	16.57	16.64	16.23
1.233	0.74	1.67	P20934	Protein EVI2A	Evi2a	858	4	4	4	23.3	23.3	23.3	24.087	0.0000	31.459	4488100	44	14.49	14.64	14.40	15.03	14.01	14.38	14.12	13.07
3.129	0.74	1.67	Q8BVI4	Dihydropteridine reductase	Qdpr	3241	11	11	11	48.1	48.1	48.1	25.57	0.0000	241.64	117420000	449	18.32	18.18	18.43	18.47	17.72	17.74	17.66	17.32
1.868	0.74	1.67	Q9CZW4	Long-chain-fatty-acid--CoA ligase 3	Acsl3	4842	19	17	17	39	36.7	36.7	80.491	0.0000	149.27	20968000	183	16.45	16.19	16.27	15.90	15.81	15.43	15.63	14.97
3.059	0.74	1.67	Q9EP89	Serine beta-lactamase-like protein LACTB, mitochondrial	Lactb	5247	20	20	20	40.1	40.1	40.1	60.705	0.0000	154.38	34815000	348	16.13	16.07	16.47	16.17	15.46	15.51	15.65	15.26
1.636	0.74	1.67	Q99M15	Proline-serine-threonine phosphatase-interacting protein 2	Pstpip2	4453	15	15	14	55.4	55.4	53.3	38.948	0.0000	141.47	43607000	340	17.63	17.21	17.16	17.62	16.95	17.04	16.56	16.13
1.602	0.74	1.67	Q8CDM8	Protein FAM160B1	Fam160b1	3463	5	5	5	10.1	10.1	10.1	86.016	0.0000	22.691	4933900	63	14.80	14.52	14.51	14.63	14.56	13.44	13.84	13.68
2.341	0.74	1.67	Q61335	B-cell receptor-associated protein 31	Bcap31	2293	22	22	22	52.7	52.7	52.7	27.956	0.0000	323.31	204690000	791	18.63	18.24	18.35	18.55	17.54	17.40	17.90	17.99
3.065	0.74	1.67	Q06138;Q9D	Calcium-binding protein 39	Cab39	1748	18	18	18	60.7	60.7	60.7	39.842	0.0000	67.409	20244000	204	16.06	15.82	15.54	15.90	15.12	14.96	15.10	15.19
1.828	0.74	1.67	Q6P6M7	O-phosphoseryl-tRNA(Sec) selenium transferase	Sepsecs	2581	5	5	5	19.8	19.8	19.8	55.325	0.0000	31.756	4372300	52	14.67	14.26	14.25	14.80	13.63	13.35	14.08	13.97
1.440	0.74	1.67	E9Q2M9		Wdfy4	230	39	39	39	17.4	17.4	17.4	354.33	0.0000	138.5	35948000	300	16.52	15.79	16.51	16.50	15.84	15.02	15.94	15.59
1.248	0.74	1.67	Q99M31	Heat shock 70 kDa protein 14	Hspa14	4455	8	8	8	28.3	28.3	28.3	54.65	0.0000	92.764	7721700	68	15.49	15.08	15.53	15.22	14.88	15.15	14.54	13.80
1.796	0.74	1.66	Q60634	Flotillin-2	Flot2	2187	21	21	21	52.6	52.6	52.6	47.037	0.0000	132.34	24672000	236	16.11	15.57	16.24	16.02	15.18	14.88	15.69	15.24
0.329	0.74	1.66	Q01237	3-hydroxy-3-methylglutaryl-coenzyme A reductase	Hmgcr	1706	2	2	2	3.2	3.2	3.2	97.039	0.0000	6.6926	1019400	13	12.15	14.31	16.02	13.90	14.16	13.51	11.84	13.92
2.566	0.73	1.66	Q920B9	FACT complex subunit SPT16	Supt16h	4209	39	39	39	40.8	40.8	40.8	119.82	0.0000	314.76	70212000	642	16.74	16.32	16.81	16.99	15.83	16.03	16.05	16.02
2.625	0.73	1.66	Q810A3	Tetratricopeptide repeat protein 9C	Ttc9c	2924	4	4	4	22.8	22.8	22.8	19.997	0.0000	25.069	5873600	97	15.57	14.92	15.23	15.19	14.47	14.67	14.40	14.45
2.129	0.73	1.66	O35598	Disintegrin and metalloproteinase domain-containing protein 10	Adam10	383	9	9	9	14.6	14.6	14.6	83.967	0.0000	17.092	11702000	103	15.62	15.22	15.77	15.66	14.89	14.52	15.19	14.73
1.011	0.73	1.66	Q8C3X8	Lipase maturation factor 2	Lmf2	3382	8	8	8	13	13	13	79.996	0.0000	34.626	9307800	43	16.47	14.91	15.10	15.10	14.71	14.67	14.41	14.85
0.673	0.73	1.66	Q8C4X2	Casein kinase I isoform gamma-3	Csnk1g3	3389	7	7	4	17.9	17.9	11.1	48.937	0.0000	9.0168	3958700	20	14.32	13.27	14.62	14.92	13.89	13.84	14.06	12.42
0.600	0.73	1.66	Q8BMS4	Ubiquinone biosynthesis O-methyltransferase, mitochondrial	Coq3	3149	4	4	4	15.4	15.4	15.4	40.956	0.0000	8.999	3356800	27	14.38	14.34	15.65	15.01	14.39	14.74	14.66	12.67
2.846	0.73	1.66	Q9D787	Peptidyl-prolyl cis-trans isomerase-like 2	Ppil2	5023	12	12	12	35.1	35.1	35.1	59.064	0.0000	72.429	16643000	175	15.89	15.55	16.11	15.94	15.06	15.22	15.26	15.03
0.796	0.73	1.66	Q5SRX1	TOM1-like protein 2	Tom1l2	2133	4	4	4	17.6	17.6	17.6	55.662	0.0000	11.799	1342500	17	12.82	13.20	14.57	13.67	12.63	13.21	13.29	12.21
2.147	0.73	1.66	Q8JZQ2	AFG3-like protein 2	Afg3l2	3578	24	24	22	30.3	30.3	29.3	89.518	0.0000	94.562	55987000	328	16.87	16.41	16.76	16.71	15.75	15.67	16.31	16.11
2.253	0.73	1.65	Q924Z4	Ceramide synthase 2	Cers2	4303	7	7	7	12.4	12.4	12.4	45.024	0.0000	76.887	14378000	168	16.02	15.81	16.26	15.96	15.11	15.09	15.70	15.24
0.602	0.73	1.65	O35551	Rab GTPase-binding effector protein 1	Rabep1	380	5	5	4	8.6	8.6	6.7	99.523	0.0004	4.7729	1598900	13	14.58	13.16	14.45	13.47	12.39	13.92	12.44	14.01

2.890	0.72	1.65	O70435	Proteasome subunit alpha type-3	Psma3	498	21	21	21	69.4	69.4	69.4	28.405	0.0000	271.11	308030000	525	20.63	20.25	20.55	20.60	19.87	19.71	19.99	19.57
2.291	0.72	1.65	Q8K4Q7	Ceramide kinase	Cerk	3726	5	5	5	14.3	14.3	14.3	59.842	0.0000	23.475	5035300	71	15.41	14.96	15.28	14.93	14.07	14.57	14.49	14.55
3.160	0.72	1.65	P14685	26S proteasome non-ATPase regulatory subunit 3	Psmd3	784	39	39	39	60.9	60.9	60.9	60.718	0.0000	323.31	154390000	978	18.11	17.65	17.76	17.85	17.23	16.96	17.17	17.10
2.395	0.72	1.65	E9Q3L2		Pi4ka	233	29	29	29	18.7	18.7	18.7	237.04	0.0000	125.64	27785000	296	15.77	15.32	15.93	15.66	14.93	14.72	15.17	14.97
2.215	0.72	1.65	Q9CQ62	2,4-dienoyl-CoA reductase, mitochondrial	Decr1	4566	13	13	13	47.2	47.2	47.2	36.213	0.0000	113.14	48916000	307	17.38	16.75	17.20	17.14	16.35	16.33	16.72	16.19
2.328	0.72	1.65	O35704	Serine palmitoyltransferase 1	Sptlc1	400	17	17	17	46.3	46.3	46.3	52.534	0.0000	192.41	34782000	261	16.71	16.58	16.98	16.84	16.04	16.04	16.41	15.72
2.396	0.72	1.65	Q8VC28	Aldo-keto reductase family 1 member C13	Akr1c13	3906	21	21	21	77.4	77.4	77.4	37.057	0.0000	186.86	56711000	444	17.58	17.15	17.76	17.80	16.82	16.72	16.96	16.91
3.081	0.72	1.65	Q9JIF7	Coatomer subunit beta	Copb1	5397	43	43	42	52.7	52.7	51.7	107.06	0.0000	323.31	143740000	870	18.81	18.62	18.70	18.81	18.11	17.77	18.26	17.92
0.603	0.72	1.65	Q8VDG7	Platelet-activating factor acetylhydrolase 2, cytoplasmic	Pafah2	3953	10	10	10	35.1	35.1	35.1	43.561	0.0000	22.493	3315800	26	14.17	14.04	14.98	13.09	13.53	14.44	12.74	12.69
1.227	0.72	1.65	O89017	Legumain	Lgmn	595	9	9	9	25.7	25.7	25.7	49.372	0.0000	62.071	10324000	120	15.59	15.57	14.94	15.49	14.31	15.45	14.66	14.29
1.489	0.72	1.65	P59764	Dedicator of cytokinesis protein 4	Dock4	1344	17	17	16	12.1	12.1	11.4	226.55	0.0000	44.024	11875000	127	15.59	14.84	15.21	15.25	14.60	14.12	15.05	14.23
0.739	0.72	1.65	Q8BVU5	ADP-ribose pyrophosphatase, mitochondrial	Nudt9	3248	6	6	6	18.9	18.9	18.9	38.604	0.0000	6.2519	1533500	20	12.14	13.88	12.86	13.44	12.73	12.98	11.95	11.78
0.978	0.72	1.65	Q3UBX0	Transmembrane protein 109	Tmem109	1942	3	3	3	11.1	11.1	11.1	26.306	0.0002	5.6541	22993000	58	17.67	17.05	17.77	17.98	16.27	17.40	17.50	16.43
2.393	0.72	1.65	Q9WVE8	Protein kinase C and casein kinase substrate in neurons protein 2	Pacsin2	5746	27	27	27	56.6	56.6	56.6	55.832	0.0000	165.61	81990000	517	17.56	17.37	18.01	17.83	17.01	17.06	17.08	16.75
2.067	0.72	1.64	P20491	High affinity immunoglobulin epsilon receptor subunit gamma	Fcer1g	856	4	4	4	32.6	32.6	32.6	9.6523	0.0000	77.006	240180000	223	20.56	19.91	20.63	20.57	19.73	19.78	19.82	19.47
0.705	0.72	1.64	P52019	Squalene monooxygenase	Sqle	1212	3	3	3	8.9	8.9	8.9	63.769	0.0000	25.084	2157800	27	12.96	13.30	14.22	14.65	12.46	13.61	12.63	13.56
3.920	0.72	1.64	Q9R0N0	Galactokinase	Galk1	5630	20	20	20	57.9	57.9	57.9	42.295	0.0000	216.76	53732000	393	17.54	17.34	17.54	17.46	16.64	16.92	16.65	16.80
1.100	0.72	1.64	Q791T5	Mitochondrial carrier homolog 1	Mtch1	2733	12	12	12	36.8	36.8	36.8	41.565	0.0000	79.35	16249000	109	17.24	16.08	16.29	16.50	15.80	15.18	16.16	16.11
1.366	0.72	1.64	Q921E6	Polycomb protein EED	Eed	4219	5	5	5	12.9	12.9	12.9	50.197	0.0002	6.0124	2544600	25	13.49	13.41	14.49	13.86	12.92	13.37	13.27	12.83
1.369	0.72	1.64	Q9CZX7	Type 2 phosphatidylinositol 4,5-bisphosphate 4-phosphatase	Tmem55a	4845	8	8	7	54.9	54.9	51	28.038	0.0000	117.5	33045000	117	17.06	16.29	17.10	17.52	16.15	16.30	16.57	16.07
0.529	0.71	1.64	Q8K3K7	1-acyl-sn-glycerol-3-phosphate acyltransferase beta	Agpat2	3704	3	3	3	22.7	22.7	22.7	31.01	0.0000	8.0181	2318400	27	14.24	13.42	13.91	13.70	13.92	11.43	13.03	14.03
2.014	0.71	1.64	Q69ZK0	Phosphatidylinositol 3,4,5-trisphosphate-dependent Rac exchanger 1 pr	Prex1	2485	49	49	48	36	36	35.5	184.93	0.0000	323.31	66630000	745	16.14	15.69	16.18	16.13	15.63	15.20	15.51	14.95
1.448	0.71	1.64	P16125	L-lactate dehydrogenase B chain	Ldhb	806	16	14	14	54.2	46.7	46.7	36.572	0.0000	134.54	34373000	276	16.68	16.31	16.93	16.87	15.46	15.83	16.53	16.12
1.894	0.71	1.64	Q8CFE2	UPF0609 protein C4orf27 homolog		3481	10	10	10	35	35	35	39.291	0.0000	65.666	11221000	174	15.13	14.61	15.48	15.37	14.29	14.46	14.57	14.42
1.486	0.71	1.64	Q9CV28	Protein FAM188A	Fam188a	4705	2	2	2	6.8	6.8	6.8	49.612	0.0000	47.706	3977500	85	15.36	15.13	14.79	14.66	14.69	13.75	14.45	14.20
1.909	0.71	1.64	Q9JJZ6	Krueppel-like factor 13	Klf13	5441	5	5	4	23.5	23.5	16.6	31.136	0.0000	15.06	6480700	42	15.94	15.34	15.63	15.20	14.57	14.70	14.90	15.10
1.620	0.71	1.63	O88351	Inhibitor of nuclear factor kappa-B kinase subunit beta	Ikbkb	524	25	25	23	38.2	38.2	38.2	86.689	0.0000	104.58	27071000	250	15.66	15.34	16.03	15.96	15.36	14.92	15.28	14.60
2.380	0.71	1.63	Q8CGY8	UDP-N-acetylglucosamine--peptide N-acetylglucosaminyltransferase 11	Ogt	3514	36	36	36	42.9	42.9	42.9	116.95	0.0000	249.94	50863000	569	16.34	15.79	16.26	16.32	15.54	15.47	15.64	15.23
3.051	0.71	1.63	Q61510	E3 ubiquitin/ISG15 ligase TRIM25	Trim25	2307	23	23	23	42	42	42	71.726	0.0000	239.26	64923000	572	16.98	16.60	16.78	16.86	16.21	15.91	16.26	16.00
1.941	0.71	1.63	Q9DCS3	Trans-2-enoyl-CoA reductase, mitochondrial	Mecr	5227	17	17	17	68.9	68.9	68.9	40.342	0.0000	323.31	95646000	585	17.43	17.62	18.22	18.05	17.02	17.00	17.29	17.18
1.090	0.71	1.63	P36423	Thromboxane-A synthase	Tbxas1	1041	10	10	10	26.1	26.1	26.1	60.39	0.0000	49.063	13954000	93	15.46	15.42	15.55	15.63	15.58	14.04	15.09	14.52
2.066	0.71	1.63	Q8BH47	Vesicle-trafficking protein SEC22a	Sec22a	3010	3	3	3	13	13	13	35.024	0.0000	6.3211	1910000	38	13.83	13.74	13.61	13.74	13.17	12.79	13.46	12.68
1.361	0.71	1.63	O08738	Caspase-6;Caspase-6 subunit p18;Caspase-6 subunit p11	Casp6	291	9	9	9	35.5	35.5	35.5	31.595	0.0000	35.788	9659300	110	15.95	15.56	14.90	14.90	14.48	14.47	14.89	14.64
1.012	0.71	1.63	O89103	Complement component C1q receptor	Cd93	603	6	6	6	10.7	10.7	10.7	69.354	0.0000	23.12	6009400	77	15.08	14.36	15.29	15.35	14.28	13.60	14.95	14.43
0.524	0.71	1.63	P03911	NADH-ubiquinone oxidoreductase chain 4	Mtnd4	630	3	3	3	11.3	11.3	11.3	51.881	0.0000	39.508	4585600	58	15.50	14.65	14.48	13.49	15.08	13.62	12.84	13.76
1.308	0.71	1.63	Q8BJM7	S-adenosyl-L-methionine-dependent tRNA 4-demethylwyosine synthase	Tyw1	3082	5	5	5	8.2	8.2	8.2	81.598	0.0000	6.7919	2172600	23	13.95	13.10	14.04	14.12	12.81	13.32	12.82	13.43
3.198	0.71	1.63	Q91YQ5	Dolichyl-diphosphooligosaccharide--protein glycosyltransferase subunit	Rpn1	4172	53	53	53	74.2	74.2	74.2	68.527	0.0000	323.31	580910000	1598	19.98	19.59	19.88	19.87	19.09	18.99	19.32	19.09
0.961	0.71	1.63	Q9D6L8	Peptidyl-prolyl cis-trans isomerase-like 3	Ppil3	5002	2	2	2	20.5	20.5	20.5	18.127	0.0000	12.62	3244200	29	14.51	14.53	14.77	14.84	14.59	14.52	13.69	13.05
2.780	0.70	1.63	Q922V4	Pleiotropic regulator 1	Plrg1	4279	18	18	18	48.7	48.7	48.7	56.937	0.0000	229.29	40336000	306	17.31	16.86	17.01	17.13	16.33	16.21	16.63	16.32
3.662	0.70	1.63	Q8R1F1	Niban-like protein 1	Fam129b	3786	54	54	54	71.8	71.8	71.8	84.818	0.0000	323.31	382760000	1424	19.08	19.03	19.22	19.07	18.45	18.18	18.55	18.40
0.844	0.70	1.63	Q8CHS8	Vacuolar protein sorting-associated protein 37A	Vps37a	3530	2	2	2	9.6	9.6	9.6	44.418	0.0000	59.871	2690800	47	14.04	14.62	14.70	14.78	13.85	12.85	14.72	13.91
1.773	0.70	1.63	P58021	Transmembrane 9 superfamily member 2	Tm9sf2	1303	10	10	10	17.8	17.8	17.8	75.329	0.0000	38.027	13742000	124	15.86	15.18	16.04	15.63	15.16	14.96	15.10	14.68
3.777	0.70	1.63	Q8QZY1	Eukaryotic translation initiation factor 3 subunit L	Eif3l	3738	37	37	36	63.5	63.5	63.5	66.612	0.0000	323.31	165050000	847	17.66	17.59	17.64	17.78	17.12	16.79	17.05	16.90
3.084	0.70	1.63	Q99KP6	Pre-mRNA-processing factor 19	Prpf19	4388	24	24	24	55.8	55.8	55.8	55.238	0.0000	323.31	128250000	587	17.95	17.59	18.06	17.92	17.18	17.27	17.24	17.03
2.077	0.70	1.63	Q923T9	Calcium/calmodulin-dependent protein kinase type II subunit gamma	Camk2g	4287	11	7	4	27.2	18	13	59.606	0.0000	40.348	9165400	110	16.04	15.18	15.66	15.66	14.78	14.98	14.98	14.98
1.633	0.70	1.63	Q8K190	SAYSvFN domain-containing protein 1	Saysd1	3618	2	2	2	15.4	15.4	15.4	20.703	0.0000	48.707	2890900	47	14.79	14.53	14.49	14.51	13.32	13.90	13.88	14.41
2.816	0.70	1.63	Q3TPX4	Exocyst complex component 5	Exoc5	1867	19	19	19	38	38	38	81.737	0.0000	130.82	18172000	228	16.03	15.56	15.65	15.78	14.87	15.24	15.05	15.05
1.117	0.70	1.63	Q9DBR3	Armadillo repeat-containing protein 8	Armc8	5171	16	16	16	30	30	30	75.363	0.0000	36.697	9309700	114	15.19	14.76	15.42	15.42	14.19	13.91	14.66	15.23
2.741	0.70	1.63	P06800	Receptor-type tyrosine-protein phosphatase C	Ptprc	664	49	49	49	36	36	36	144.84	0.0000	323.31	180420000	985	18.63	18.14	18.21	18.27	17.81	17.49	17.64	17.50
1.283	0.70	1.63	A2A5R2	Brefeldin A-inhibited guanine nucleotide-exchange protein 2	Arfgef2	7	29	29	23	21.7	21.7	17	202.24	0.0000	276.91	21777000	249	15.51	14.99	16.02	15.60	14.99	14.38	15.30	14.66
2.713	0.70	1.63	Q9CR26	Vacuolar protein sorting-associated protein VTA1 homolog	Vta1	4661	11	11	11	39.5	39.5	39.5	33.913	0.0000	125.6	44259000	226	18.12	17.74	18.05	18.22	17.41	17.46	17.38	17.08
2.456	0.70	1.63	Q61249;Q9(	Immunoglobulin-binding protein 1	Igbp1	2286	18	18	18	66.2	66.2	66.2	38.97	0.0000	279.97	21083000	164	15.92	16.37	15.98	15.73	15.29	15.11	15.39	15.39
1.954	0.70	1.63	Q9DB27;Q9	Malignant T-cell-amplified sequence 1	Mcts1	5121	12	12	12	71.3	71.3	71.3	20.555	0.0000	108.46	51914000	362	17.75	17.23	17.31	17.69	16.92	16.73	17.10	16.43
0.989	0.70	1.63	Q3UNZ8	Quinone oxidoreductase-like protein 2		2011	6	6	6	36.6	36.6	36.6	37.808	0.0000	48.516	3315800	48	14.59	13.17	14.49	14.28	13.45	13.87	13.26	13.14
1.541	0.70	1.63	Q9JLT4	Thioredoxin reductase 2, mitochondrial	Txnrd2	5490	12	10	10	36.1	33	33	56.602	0.0000	135.04	24578000	125	16.70	16.27	17.04	16.99	16.08	15.67	16.49	15.96
1.639	0.70	1.62	Q9ER73	Elongator complex protein 4	Elp4	5297	3	3	3	10.2	10.2	10.2	46.325	0.0000	57.774	4394400	83	15.26	15.19	15.07	15.29	14.27	14.82	14.94	14.00
0.585	0.70	1.62	Q99K95	Protein RTF2 homolog	Rtfdc1	4368	4	4	4	15.3	15.3	15.3	33.934	0.0000	9.0683	1193500	8	14.18	13.92	13.56	12.53	11.98	12.67	12.72	14.03
1.120	0.70	1.62	Q9QY81	Nuclear pore membrane glycoprotein 210	Nup210	5563	22	22	22	17.1	17.1	17.1	204.1	0.0000	114.22	21851000	299	15.78	15.34	16.04	15.91	15.42	14.75	15.68	14.44
2.584	0.70	1.62	Q6ZQ08	CCR4-NOT transcription complex subunit 1	Cnot1	2688	31	31	31	17.7	17.7	17.7	266.8	0.0000	139.67	25157000	301	16.03	15.61	16.02	15.78	15.11	15.18	15.42	14.94
3.017	0.70	1.62	Q9ET01	Glycogen phosphorylase, liver form	Pygl	5353	66	66	54	74.5	74.5	64.4	97.462	0.0000	323.31	240710000	1362	18.20	17.87	17.97	18.03	17.38	17.06	17.50	17.35
2.894	0.70	1.62	P07356;RE\	Annexin A2	Anxa2	670	63	63	63	94.1	94.1	94.1	38.676	0.0000	323.31	3252200000	3618	21.84	21.56	21.58	21.58	21.04	20.85	21.19	20.72
2.112	0.69	1.62	B2RXC1	Trafficking protein particle complex subunit 11	Trappc11	84	24	24	24	32.7	32.7	32.7	128.39	0.0000	120.22	32634000	331	16.62	16.19	16.75	16.26	15.83	15.59	16.04	15.58
1.477	0.69	1.62	P42859	Huntingtin	Htt	1082	26	26	26	13.5	13.5	13.5	344.69	0.0000	201.48	32102000	245	17.34	16.69	17.46	17.12	16.32	16.36	17.00	16.15
3.050	0.69	1.62	P62827;Q61	GTP-binding nuclear protein Ran	Ran	1448	24	24	23	63.9	63.9	63.9	24.423	0.0000	186.34	504100000	751	20.67	20.32	20.36	20.48	19.95	19.79	19.77	19.55
0.443	0.69	1.62	Q76N33	AMSH-like protease	Stambpl1	2720	8	8	8	22.5	22.5	22.5	49.639	0.0000	17.824	5677100	45	12.63	14.47	15.00	15.19	12.68	14.49	13.42	13.94
1.999	0.69	1.62	Q8BVW3	Tripartite motif-containing protein 14	Trim14	3250	21	21	21	51.8	51.8	51.8	49.64	0.0000	66.502	30606000	331	16.35	16.03	16.19	15.89	15.58	15.56	15.60	14.95
1.345	0.69	1.62	Q9JJF3	Bifunctional lysine-specific demethylase and histidyl-hydroxylase NO66	No66	5425	8	8	8	18.7	18.7	18.7	67.556	0.0000	16.468	3712200	39	14.36	13.55	14.50	14.20	13.33	13.68	13.03	13.80
2.374	0.69	1.62	Q91W86	Vacuolar protein sorting-associated protein 11 homolog	Vps11	4096	36	36	36	44	44	44	107.72	0.0000	230.25	73749000	725	17.06	16.48	17.01	16.78	16.17	16.21	16.27	15.90
1.682	0.69	1.62	Q3UH60	Disco-interacting protein 2 homolog B	Dip2b	1966	24	24	23	21.8	21.8	21.2	171.12	0.0000	146.38	23786000	302	16.28	15.52	16.36	16.13	15.18	15.44	15.68	15.22
1.826	0.69	1.61	P54071	Isocitrate dehydrogenase [NADP], mitochondrial	Idh2	1246	38	36	36	71.5	71.5	71.5	50.906	0.0000	323.31	323530000	1118	18.96	18.52	19.05	19.02	18.21	18.53	18.30	17.75
1.141	0.69	1.61	Q9Z1J3	Cysteine desulfurase, mitochondrial	Nfs1	5809	16	16	15	48.8	48.8	46.2	50.569	0.0000	111.75	30472000	230	16.14	15.81	16.36	16.07	16.02	15.10	15.76	14.74
0.412	0.69	1.61	Q91YT7	YTH domain-containing family protein 2	Ythdf2	4179	10	5	4	16.2	9.3	7.4	62.279	0.0004	4.3449	5067500	32	14.87	14.99	12.28	14.94	12.83	13.79	13.36	14.35
3.301	0.69	1.61	P68254	14-3-3 protein theta	Ywhaq	1515	31	24	24	85.3	78.8	78.8	27.778	0.0000	323.31	234060000	783	19.72	19.35	19.40	19.66	18.81	18.75	18.93	18.90
1.136	0.69	1.61	Q8BH57	WD repeat-containing protein 48	Wdr48	3013	11	11	11	24.7	24.7	24.7	76.006	0.0000	51.712	8446800	67	15.32	14.70	15.44	15.31	14.08	15.09	14.02	14.83
2.079	0.69	1.61	Q921J2	GTP-binding protein Rheb	Rheb	4229	7	7	7	38.6	38.6	38.6	20.451	0.0000	60.555	25755000	153	17.16	17.03	17.67	17.62	16.47	16.68	16.76	16.82
2.312	0.69	1.61	P97372	Proteasome activator complex subunit 2	Psme2	1637	25	25	24	82.8	82.8	78.2	27.057	0.0000	323.31	243210000	663	19.05	18.77	18.41	18.54	18.22	17.92	17.98	17.91
1.379	0.69	1.61	Q91XB7	Protein YIF1A	Yif1a	4140	2	2	2	7.2	7.2	7.2	32.134	0.0000	10.094	6046600	41	15.16	14.98	15.30	14.91	13.65	14.59	14.66	14.71
1.519	0.69	1.61	Q9DB25	Dolichyl-phosphate beta-glucosyltransferase	Alg5	5120	9	9	9	32.1	32.1	32.1	36.79	0.0000	58.919	29877000	195	17.18	16.83	17.77	17.50	16.22	16.81	16.77	16.72
1.706	0.69	1.61	Q9EPU4	Cleavage and polyadenylation specificity factor subunit 1	Cpsf1	5265	23	23	23	21.5	21.5	21.5	160.82	0.0000	100.41	15260000	194	15.70	15.49	15.73	15.70	15.34	14.89	15.23	14.41
1.238	0.68	1.61	Q99LL5	Periodic tryptophan protein 1 homolog	Pwp1	4439	5	5	5	15.4	15.4	15.4	55.586	0.0000	19.592	4401800	64	14.95	14.38	15.01	15.12	13.90	13.95	13.98	14.91
1.565	0.68	1.61	O88512	AP-1 complex subunit gamma-like 2	Ap1g2	537	9	9	9	15.4	15.4	15.4	87.862	0.0000	35.044	11144000	157	15.17	14.99	15.56	15.49	14.50	14.75	15.06	14.15
2.439	0.68	1.61	Q9DBE8	Alpha-1,3/1,6-mannosyltransferase ALG2	Alg2	5146	15	15	15	45.5	45.5	45.5	47.404	0.0000	77.366	19872000	169	15.75	15.31	15.87	15.65	14.79	14.85	15.18	15.03
1.279	0.68	1.61	Q9Z1E4;Q8\	Glycogen [starch] synthase, muscle	Gys1	5805	15	15	15	28.9	28.9	28.9	83.926	0.0000	48.787	9537300	90	14.62	14.37	15.38	15.05	14.32	14.56	14.02	13.77
1.218	0.68	1.61	Q8R2Q4	Ribosome-releasing factor 2, mitochondrial	Gfm2	3813	15	15	15	25.2	25.2	25.2	86.108	0.0000	49.549	10580000	110	15.02	15.04	15.61	15.33	15.02	14.06	15.02	14.17
2.472	0.68	1.61	P25206	DNA replication licensing factor MCM3	Mcm3	905	36	36	34	48.8	48.8	48.8	91.545	0.0000	323.31	55428000	589	16.65	16.01	16.35	16.41	15.78	15.68	15.49	15.73
0.480	0.68	1.61	P07607	Thymidylate synthase	Tyms	671	3	3	3	14.3	14.3	14.3	34.958	0.0000	52.314	11448000	39	15.19	15.67	15.44	15.31	15.40	15.36	15.31	12.81
2.440	0.68	1.60	P24063	Integrin alpha-L	Itgal	894	34	34	34	30.3	30.3	30.3	128.33	0.0000	250.14	80717000	520	17.07	16.81	17.29	17.17	16.65	16.20	16.52	16.25
1.744	0.68	1.60	Q9DBS9	Oxysterol-binding protein-related protein 3	Osbpl3	5176	14	14	13	21.1	21.1	20	96.965	0.0000	50.922	10508000	111	15.51	14.70	15.55	15.26	14.37	14.52	14.73	14.66
2.275	0.68	1.60	Q8R016	Bleomycin hydrolase	Blmh	3744	30	30	30	63.7	63.7	63.7	52.511	0.0000	323.31	110870000	611	17.58	17.35	17.64	17.39	16.93	16.61	17.16	16.54
0.530	0.68	1.60	P53702	Cytochrome c-type heme lyase	Hccs	1236	5	5	5	26.8	26.8	26.8	30.977	0.0000	20.695	3948300	47	13.75	14.49	14.73	14.31	14.62	12.44	14.54	12.96
2.906	0.68	1.60	Q9DCH4	Eukaryotic translation initiation factor 3 subunit F	Eif3f	5214	15	15	15	48.2	48.2	48.2	37.984	0.0000	322.7	87371000	445	17.75	17.56	17.66	17.55	17.12	16.79	17.16	16.73
1.750	0.68	1.60	Q91VE0	Long-chain fatty acid transport protein 4	Slc27a4	4058	18	18	17	37	37	35.8	72.318	0.0000	162.76	26924000	260	15.82	15.48	16.36	15.72	14.95	15.06	15.41	15.24
1.905	0.68	1.60	Q9CZU3	Superkiller viralicidic activity 2-like 2	Skiv2l2	4839	35	35	35	39.8	39.8	39.8	117.64	0.0000	272.84	56433000	435	17.05	16.46	17.20	16.97	16.31	16.15	16.50	16.00
3.435	0.68	1.60	Q61881	DNA replication licensing factor MCM7	Mcm7	2342	35	35	35	55.2	55.2	55.2	81.21	0.0000	207.62	81082000	754	17.15	16.79	16.95	17.09	16.42	16.24	16.38	16.21
1.332	0.68	1.60	Q07797	Galectin-3-binding protein	Lgals3bp	1757	13	13	13	27.6	27.6	27.6	64.49	0.0000	323.31	78736000	453	17.46	17.79	16.83	17.10	17.09	16.56	16.55	16.26
2.738	0.68	1.60	Q9JJZ4	Ubiquitin-conjugating enzyme E2 J1	Ube2j1	5440	1	1	1	5.3	5.3	5.3	34.989	0.0000	15.298	2273600	67	14.46	14.01	14.44	14.36	13.57	13.52	13.86	13.60
1.195	0.68	1.60	Q4VBE8	WD repeat-containing protein 18	Wdr18	2071	10	10	10	28.3	28.3	28.3	47.211	0.0000	40.513	10879000	113	15.72	15.50	15.29	15.96	15.19	14.95	15.41	14.21
1.602	0.68	1.60	Q8BL99	Protein dopey-1	Dopey1	3115	2	2	2	1.4	1.4	1.4	269.25	0.0000	6.4674	1058900	12	13.96	13.00	13.75	13.51	13.16	12.76	12.75	12.82
0.599	0.68	1.60	Q9R0P9	Ubiquitin carboxyl-terminal hydrolase isozyme L1	Uchl1	5635	6	6	6	36.8	36.8	36.8	24.838	0.0000	29.336	7707500	59	14.56	15.36	15.31	14.73	15.38	14.91	13.35	13.60
0.324	0.68	1.60	Q8BVF2	Phosducin-like protein 3	Pdcl3	3238	3	3	3	15	15	15	27.581	0.0000	12.659	5172000	33	15.22	14.71	14.40	14.67	12.49	12.64	15.38	15.76
0.834	0.68	1.60	Q8BIW1	Protein prune homolog	Prune	3065	6	6	6	17.2	17.2	17.2	50.239	0.0000	38.39	4202700	62	13.86	13.91	14.19	13.82	13.34	12.19	14.10	13.45
0.752	0.68	1.60	Q8BJT9	alpha-1,2-Mannosidase	Edem2	3085	2	2	2	7.1	7.1	7.1	64.609	0.0004	4.3746	1909500	13	13.20	14.28	14.84	14.29	14.30	13.33	13.03	13.25
1.701	0.68	1.60	Q6PEB6	MOB-like protein phocein	Mob4	2624	9	9	9	59.6	59.6	59.6	26.032	0.0000	146.86	12588000	125	16.33	15.67	15.83	15.34	15.11	15.27	15.05	15.02
2.417	0.68	1.60	Q91V41	Ras-related protein Rab-14	Rab14	4046	25	25	25	87.4	87.4	87.4	23.897	0.0000	323.31	288280000	900	19.29	19.25	19.35	19.42	18.79	18.53	18.96	18.31
2.707	0.68	1.60	Q60597	2-oxoglutarate dehydrogenase, mitochondrial	Ogdh	2182	54	54	54	52.3	52.3	52.3	116.45	0.0000	323.31	290220000	1361	18.59	18.26	18.60	18.72	17.81	17.80	18.11	17.74
1.290	0.68	1.60	P11928;Q8\	2-5-oligoadenylate synthase 1A	Oas1a	752	21	21	21	56.4	56.4	56.4	42.428	0.0000	236.37	66143000	562	17.72	17.25	16.63	16.80	16.80	16.37	16.38	16.15
1.120	0.68	1.60	Q9D8Y0;Q9	EF-hand domain-containing protein D2	Efhd2	5082	20	20	20	61.7	61.7	61.7	26.791	0.0000	123.55	49296000	430	17.08	17.46	17.35	17.45	16.92	17.35	16.41	15.96
3.262	0.68	1.60	Q11136	Xaa-Pro dipeptidase	Pepd	1783	30	30	30	57.4	57.4	57.4	55.028	0.0000	323.31	136510000	804	18.40	18.08	18.42	18.43	17.63	17.64	17.82	17.55
0.561	0.68	1.60	Q2TBA3	Mucosa-associated lymphoid tissue lymphoma translocation protein 1 h	Malt1	1800	7	7	7	13.5	13.5	13.5	93.215	0.0000	60.389	4609800	57	14.34	14.93	15.32	14.20	14.73	13.20	15.02	13.13
2.898	0.67	1.60	Q91YI4	Beta-arrestin-2	Arrb2	4159	19	19	18	63.7	63.7	61.7	46.313	0.0000	226.28	40578000	276	17.53	17.10	17.15	17.22	16.58	16.56	16.75	16.41
0.305	0.67	1.60	Q9D142	Uridine diphosphate glucose pyrophosphatase	Nudt14	4895	3	3	3	15.8	15.8	15.8	24.443	0.0000	7.2144	5325900	50	13.67	15.25	14.96	14.33	15.36	12.20	15.37	12.59
1.004	0.67	1.60	Q5SW28	Phosphoinositide 3-kinase regulatory subunit 5	Pik3r5	2154	7	7	7	16.3	16.3	16.3	96.953	0.0000	49.215	6109300	80	16.27	15.16	15.63	15.54	14.39	15.57	15.20	14.75
0.592	0.67	1.59	Q9CQJ4	E3 ubiquitin-protein ligase RING2	Rnf2	4605	4	4	3	17	17	12.8	37.623	0.0000	13.064	4194400	41	14.40	14.36	14.54	14.58	13.11	14.40	14.97	12.70
1.456	0.67	1.59	Q9WU40	Inner nuclear membrane protein Man1	Lemd3	5696	17	17	17	27.5	27.5	27.5	100.31	0.0000	125.09	19121000	140	15.53	15.73	16.32	16.00	15.27	14.73	15.60	15.30

2.857	0.67	1.59	P84091	AP-2 complex subunit mu	Ap2m1	1608	30	30	30	62.5	62.5	62.5	49.654	0.0000	133.45	158780000	635	18.60	18.16	18.18	18.34	17.63	17.56	17.83	17.57
2.507	0.67	1.59	P35831	Tyrosine-protein phosphatase non-receptor type 12	Ptpn12	1034	20	20	20	29.7	29.7	29.7	86.525	0.0000	90.103	29391000	259	16.25	15.80	16.34	16.19	15.61	15.26	15.55	15.48
1.662	0.67	1.59	O88569	Heterogeneous nuclear ribonucleoproteins A2/B1	Hnrnpa2b1	547	36	36	36	78.8	78.8	78.8	37.402	0.0000	323.31	448760000	1091	19.86	20.18	19.25	19.88	18.88	19.10	19.13	19.37
2.959	0.67	1.59	Q8VD65	Phosphoinositide 3-kinase regulatory subunit 4	Pik3r4	3942	26	26	26	27.5	27.5	27.5	152.6	0.0000	164.35	21307000	209	15.93	15.66	15.99	15.63	15.02	15.15	15.32	15.04
1.596	0.67	1.59	Q922H2	[Pyruvate dehydrogenase (acetyl-transferring)] kinase isozyme 3, mitocl	Pdk3	4257	10	10	10	34.2	34.2	34.2	47.922	0.0000	42.272	12342000	146	15.82	15.26	16.08	15.80	14.71	15.36	14.94	15.25
1.535	0.67	1.59	Q5SUQ9	CST complex subunit CTC1	Ctc1	2147	12	12	12	17.4	17.4	17.4	134.03	0.0000	142.6	11796000	150	15.46	15.04	15.15	15.30	14.71	13.93	14.68	14.93
1.999	0.67	1.59	Q99KD5	Protein unc-45 homolog A	Unc45a	4371	17	17	17	21.3	21.3	21.3	103.45	0.0000	73.599	19020000	144	16.09	15.79	16.25	16.28	15.41	15.22	15.84	15.25
2.619	0.67	1.59	Q8K2B3	Succinate dehydrogenase [ubiquinone] flavoprotein subunit, mitochondr	Sdha	3653	39	39	39	61.7	61.7	61.7	72.585	0.0000	323.31	182790000	962	18.25	18.05	18.14	18.16	17.77	17.50	17.51	17.15
1.891	0.67	1.59	Q6VNB8	WD repeat and FYVE domain-containing protein 3	Wdfy3	2664	27	27	27	11.1	11.1	11.1	392.33	0.0000	93.661	16395000	161	15.22	14.70	15.51	15.03	14.59	14.20	14.57	14.41
0.522	0.67	1.59	Q0KL02	Triple functional domain protein	Trio	1775	7	7	7	3.8	3.8	3.8	347.86	0.0000	58.286	3848900	34	14.47	14.57	15.21	13.30	13.59	12.59	14.67	14.03
1.768	0.67	1.59	P16460	Argininosuccinate synthase	Ass1	810	29	29	29	67	67	67	46.584	0.0000	291.54	91003000	408	17.40	17.26	18.15	17.78	16.98	16.96	17.08	16.89
2.250	0.67	1.59	Q8BZW8	NHL repeat-containing protein 2	Nhlrc2	3320	9	9	9	19.7	19.7	19.7	78.429	0.0000	27.413	9063300	89	15.58	15.48	15.12	15.46	14.45	14.86	15.02	14.63
0.720	0.67	1.59	B2RR83	Probable ATP-dependent RNA helicase YTHDC2	Ythdc2	78	6	6	6	7	7	7	161.09	0.0000	9.7909	2252300	23	13.57	13.28	14.52	13.55	12.23	14.00	13.01	13.00
1.280	0.67	1.59	Q52KI8	Serine/arginine repetitive matrix protein 1	Srrm1	2084	8	8	8	12.5	12.5	12.5	106.86	0.0000	90.967	14379000	129	17.17	16.60	16.15	16.75	15.54	16.42	16.00	16.04
2.688	0.67	1.59	Q922J3	CAP-Gly domain-containing linker protein 1	Clip1	4260	12	12	10	10.4	10.4	9.3	155.81	0.0000	54.62	5278500	70	14.76	14.55	14.71	15.05	13.99	14.33	14.05	14.03
2.119	0.67	1.59	P06795	Multidrug resistance protein 1B	Abcb1b	662	33	33	13	33.2	33.2	13.3	140.99	0.0000	323.31	55450000	573	16.32	15.97	16.69	16.57	15.56	15.82	15.70	15.79
3.219	0.67	1.59	Q8BH59	Calcium-binding mitochondrial carrier protein Aralar1	Slc25a12	3015	41	41	35	71.9	71.9	65.6	74.569	0.0000	323.31	232860000	1068	18.58	18.45	18.82	18.62	17.97	17.84	18.13	17.86
2.147	0.67	1.59	Q8K1H1	Tudor domain-containing protein 7	Tdrd7	3627	7	7	7	11.3	11.3	11.3	122.17	0.0000	43.644	6371500	62	15.19	14.64	14.83	14.59	14.29	13.87	14.25	14.18
2.000	0.67	1.59	O35375	Neuropilin-2	Nrp2	363	10	10	10	13.3	13.3	13.3	104.63	0.0000	56.296	16496000	132	16.22	16.08	16.77	16.64	15.89	15.78	15.82	15.55
3.439	0.67	1.59	Q62348	Translin	Tsn	2380	17	17	17	64.9	64.9	64.9	26.201	0.0000	235.19	66526000	390	17.28	17.00	16.93	17.05	16.35	16.39	16.56	16.32
2.182	0.67	1.59	Q8VD04	GRIP1-associated protein 1	Gripap1	3938	17	17	17	28.8	28.8	28.8	92.714	0.0000	132.87	7342000	91	14.56	14.43	14.56	14.95	14.17	13.71	14.15	13.80
2.942	0.67	1.59	Q5DTM8	E3 ubiquitin-protein ligase BRE1A	Rnf20	2096	16	16	16	24.9	24.9	24.9	113.52	0.0000	80.923	15888000	99	16.67	16.42	16.70	16.71	16.18	16.05	15.78	15.84
1.437	0.66	1.59	P48410	ATP-binding cassette sub-family D member 1	Abcd1	1143	22	22	22	32.5	32.5	32.5	81.858	0.0000	143.79	41061000	346	17.13	16.43	16.79	17.23	16.12	15.80	16.46	16.54
3.416	0.66	1.58	Q9QXX4	Calcium-binding mitochondrial carrier protein Aralar2	Slc25a13	5554	40	34	34	72.6	66.7	66.7	74.466	0.0000	323.31	198520000	822	18.90	18.59	18.87	18.88	18.29	18.01	18.19	18.09
1.587	0.66	1.58	O35954	Membrane-associated phosphatidylinositol transfer protein 1	Pitpnm1	413	8	8	8	10.5	10.5	10.5	134.94	0.0000	60.242	5312200	77	14.26	14.38	14.65	14.46	14.03	13.16	14.05	13.85
1.711	0.66	1.58	P97449	Aminopeptidase N	Anpep	1650	57	57	57	51.2	51.2	51.2	109.65	0.0000	323.31	683560000	2077	19.81	19.50	20.06	20.24	19.09	18.93	19.52	19.41
0.888	0.66	1.58	Q8CIB6	Transmembrane protein 230	Tmem230	3547	3	3	3	21.7	21.7	21.7	13.188	0.0000	8.3668	2666700	25	13.36	13.28	13.69	13.46	13.72	12.47	12.93	12.00
3.639	0.66	1.58	P45377	Aldose reductase-related protein 2	Akr1b8	1095	21	21	17	64.9	64.9	56.3	36.12	0.0000	227.3	166980000	621	18.85	18.68	18.96	18.76	18.31	18.14	18.14	18.02
1.678	0.66	1.58	Q8CI71	Coiled-coil domain-containing protein 132	Ccdc132	3543	10	10	10	15	15	15	111.17	0.0000	97.473	17384000	176	15.94	15.64	15.86	16.11	14.67	15.39	15.33	15.51
2.118	0.66	1.58	Q60864	Stress-induced-phosphoprotein 1	Stip1	2214	51	51	51	74.6	74.6	74.6	62.581	0.0000	323.31	244710000	1268	18.42	18.86	18.09	18.36	17.78	17.64	17.77	17.88
0.726	0.66	1.58	O70481	E3 ubiquitin-protein ligase UBR1	Ubr1	505	2	2	2	2.2	2.2	2.2	200.24	0.0013	3.555	1776200	14	14.08	14.51	15.69	14.56	14.47	14.61	13.60	13.52
2.455	0.66	1.58	Q91V64	Isochorismatase domain-containing protein 1	Isoc1	4048	12	12	12	45.5	45.5	45.5	32.032	0.0000	57.412	34732000	239	16.89	16.73	17.31	17.11	16.51	16.19	16.37	16.34
0.815	0.66	1.58	Q9R020	Zinc finger Ran-binding domain-containing protein 2	Zranb2	5611	3	3	3	10.3	10.3	10.3	37.35	0.0002	5.881	2273000	41	13.93	13.96	14.31	14.36	13.62	12.49	14.38	13.42
1.159	0.66	1.58	Q8BS95	Golgi pH regulator	Gpr89a	3185	3	3	3	12.1	12.1	12.1	52.734	0.0000	102.02	5605100	51	14.12	14.10	14.71	15.24	13.60	14.03	14.14	13.75
1.403	0.66	1.58	Q8BX90	Fibronectin type-III domain-containing protein 3A	Fndc3a	3281	10	10	10	11.6	11.6	11.6	131.96	0.0000	28.593	12556000	64	16.07	15.68	16.02	15.74	15.15	15.60	14.58	15.53
1.194	0.66	1.58	O35405	Phospholipase D3	Pld3	368	10	10	10	25.6	25.6	25.6	54.388	0.0000	61.453	25145000	114	16.68	17.36	17.36	16.71	15.77	16.35	16.56	16.79
2.274	0.66	1.58	P37040	NADPH--cytochrome P450 reductase	Por	1046	55	55	55	73.3	73.3	73.3	77.043	0.0000	323.31	517180000	1608	19.16	18.98	19.20	19.32	18.37	18.18	18.77	18.71
2.463	0.66	1.58	P08030	Adenine phosphoribosyltransferase	Aprt	675	18	18	17	96.7	96.7	96.1	19.724	0.0000	265.05	285310000	644	20.42	20.20	20.37	19.84	19.64	19.46	19.45	19.63
0.685	0.66	1.58	Q3UZ39	Leucine-rich repeat flightless-interacting protein 1	Lrrfip1	2046	9	8	8	18.9	17.3	17.3	79.248	0.0000	41.339	4674400	72	14.43	14.04	13.83	14.55	14.47	13.93	13.39	12.44
1.291	0.66	1.58	Q8CEE7	Retinol dehydrogenase 13	Rdh13	3473	9	9	9	38.9	38.9	38.9	36.464	0.0000	83.626	19061000	171	16.77	15.95	16.65	16.51	16.07	15.21	16.03	15.93
2.591	0.66	1.58	Q3U6Q4	Phosphoinositide 3-kinase regulatory subunit 6	Pik3r6	1930	9	9	9	18.9	18.9	18.9	84.662	0.0000	31.625	12490000	64	16.62	16.44	16.66	16.12	15.66	15.87	15.84	15.85
2.492	0.66	1.58	Q8C650	Septin-10	Sep.10	3403	7	5	5	14.6	13.1	13.1	52.422	0.0000	16.329	6000300	72	15.29	15.00	15.22	14.72	14.46	14.26	14.50	14.38
1.565	0.66	1.58	P10605	Cathepsin B;Cathepsin B light chain;Cathepsin B heavy chain	Ctsb	724	23	23	23	62.8	62.8	62.8	37.279	0.0000	323.31	771270000	1535	20.97	21.17	20.73	21.02	20.52	19.77	20.24	20.73
2.544	0.66	1.58	Q9WTX6	Cullin-1	Cul1	5692	31	31	31	41.8	41.8	41.8	89.69	0.0000	323.31	78550000	621	17.91	17.40	17.73	17.73	16.91	17.05	17.27	16.92
1.060	0.66	1.58	Q9D0G0	28S ribosomal protein S30, mitochondrial	Mrps30	4866	12	12	12	35.7	35.7	35.7	49.939	0.0000	97.177	15593000	116	16.34	15.58	16.89	16.55	15.70	15.65	15.30	16.09
1.965	0.66	1.58	Q3UCV8	Ubiquitin thioesterase otulin	Otulin	1945	9	9	9	34.7	34.7	34.7	40.32	0.0000	24.28	21603000	184	16.01	15.88	16.22	16.40	15.57	15.36	15.80	15.16
1.906	0.66	1.58	Q8BHL5	Engulfment and cell motility protein 2	Elmo2	3045	23	17	17	42.2	38.1	38.1	83.886	0.0000	138.62	24623000	218	16.37	16.34	16.88	16.71	15.64	16.06	16.21	15.77
2.716	0.66	1.57	Q99LD4	COP9 signalosome complex subunit 1	Gps1	4420	27	27	27	58.4	58.4	58.4	53.442	0.0000	120.31	41239000	415	17.06	16.74	16.92	17.28	16.25	16.25	16.47	16.40
1.691	0.65	1.57	Q8BGB7	Enolase-phosphatase E1	Enoph1	2974	5	5	5	32.3	32.3	32.3	28.6	0.0000	37.914	4784600	93	15.20	14.85	14.42	14.55	14.43	14.08	14.02	13.87
2.248	0.65	1.57	Q2TBE6	Phosphatidylinositol 4-kinase type 2-alpha	Pi4k2a	1801	17	17	17	46.3	46.3	46.3	54.257	0.0000	136.13	32753000	265	16.93	16.45	16.70	16.27	16.09	15.81	15.90	15.94
0.534	0.65	1.57	Q811J3	Iron-responsive element-binding protein 2	Ireb2	2937	8	7	7	14	13.2	13.2	104.92	0.0000	57.051	6051200	75	14.90	14.68	15.74	15.75	15.02	13.37	14.38	15.68
0.369	0.65	1.57	Q8CA72	Gigaxonin	Gan	3426	6	6	5	10.2	10.2	8.2	67.67	0.0000	18.849	7424500	62	13.54	16.15	16.42	16.71	14.93	14.45	15.58	15.26
3.781	0.65	1.57	P12367	cAMP-dependent protein kinase type II-alpha regulatory subunit	Prkar2a	756	15	15	12	45.4	45.4	35.2	45.389	0.0000	175.76	28790000	361	16.25	15.99	16.10	16.05	15.56	15.44	15.48	15.29
2.019	0.65	1.57	Q91VA6	Polymerase delta-interacting protein 2	Poldip2	4053	6	6	6	28.8	28.8	28.8	41.87	0.0000	39.505	7040200	85	15.65	15.49	15.74	15.10	15.13	14.69	14.69	14.86
3.066	0.65	1.57	P52825	Carnitine O-palmitoyltransferase 2, mitochondrial	Cpt2	1224	26	26	26	50.8	50.8	50.8	73.98	0.0000	220.3	42375000	396	16.57	16.32	16.61	16.54	15.73	15.93	16.06	15.71
2.107	0.65	1.57	Q8BU30	Isoleucine--tRNA ligase, cytoplasmic	Iars	3218	48	48	48	42.9	42.9	42.9	144.27	0.0000	323.31	106720000	752	17.67	16.94	17.23	17.25	16.69	16.42	16.75	16.61
1.928	0.65	1.57	Q9EPL9	Peroxisomal acyl-coenzyme A oxidase 3	Acox3	5260	31	31	31	54.7	54.7	54.7	78.403	0.0000	224.46	103450000	611	17.49	17.28	17.61	17.53	16.94	16.37	17.17	16.81
1.754	0.65	1.57	Q3URE1	Acyl-CoA synthetase family member 3, mitochondrial	Acsf3	2022	12	12	12	30.7	30.7	30.7	65.076	0.0000	27.975	11130000	90	16.07	15.63	15.99	16.23	15.36	15.71	15.30	14.95
1.219	0.65	1.57	O35516	Neurogenic locus notch homolog protein 2;Notch 2 extracellular truncati	Notch2	378	6	6	5	4.2	4.2	4.2	265.63	0.0000	19.238	6994900	59	16.09	15.17	15.50	16.38	15.20	15.29	15.08	14.96
1.996	0.65	1.57	Q9Z1T1;Q9.	AP-3 complex subunit beta-1	Ap3b1	5822	51	51	51	46.3	46.3	46.3	122.74	0.0000	323.31	175240000	918	18.53	17.88	18.11	18.24	17.55	17.43	17.85	17.32
1.519	0.65	1.57	Q91WU5	Arsenite methyltransferase	As3mt	4129	5	5	5	19.7	19.7	19.7	41.793	0.0000	33.161	9653800	108	15.61	15.80	16.23	16.12	15.25	14.87	15.76	15.28
1.137	0.65	1.57	P00405	Cytochrome c oxidase subunit 2	Mtco2	610	8	8	8	30.8	30.8	30.8	25.976	0.0000	182.2	369640000	531	20.23	20.25	20.33	20.68	20.04	19.75	20.18	18.92
1.610	0.65	1.57	Q99KI3	ER membrane protein complex subunit 3	Emc3	4378	7	7	7	28.4	28.4	28.4	29.98	0.0000	63.944	20136000	243	16.31	16.07	16.73	16.67	15.89	15.34	15.98	15.97
0.469	0.65	1.57	Q9WVL2	Signal transducer and activator of transcription 2	Stat2	5755	17	17	17	19.2	19.2	19.2	105.42	0.0000	56.446	11195000	86	15.77	14.25	14.38	14.26	14.87	13.93	14.64	12.61
2.832	0.65	1.57	Q8CBY8	Dynactin subunit 4	Dctn4	3441	14	14	14	35.8	35.8	35.8	53.056	0.0000	109.19	39263000	285	17.49	17.32	17.67	17.48	16.78	16.80	17.11	16.67
1.496	0.65	1.57	Q9JHK4	Geranylgeranyl transferase type-2 subunit alpha	Rabggta	5365	18	18	18	45.1	45.1	45.1	64.989	0.0000	231.53	34411000	387	16.35	16.10	16.69	16.56	15.91	15.84	16.14	15.22
2.703	0.65	1.57	P41216	Long-chain-fatty-acid--CoA ligase 1	Acsl1	1066	37	37	35	50.8	50.8	49.5	77.951	0.0000	157.18	82367000	726	17.28	16.88	17.09	16.98	16.26	16.26	16.63	16.48
1.522	0.65	1.57	P59016	Vacuolar protein sorting-associated protein 33B	Vps33b	1328	15	15	15	30	30	30	70.525	0.0000	87.843	20164000	184	16.62	15.94	16.27	16.67	15.41	15.78	16.12	15.58
2.183	0.65	1.57	E9Q4N7		Arid1b	234	12	9	9	8.4	6.6	6.6	237.79	0.0000	136.6	10492000	137	15.51	15.25	15.87	15.45	14.64	14.83	15.08	14.94
2.616	0.65	1.57	P05132	cAMP-dependent protein kinase catalytic subunit alpha	Prkaca	648	25	25	13	54.7	54.7	30.2	40.57	0.0000	191.84	61700000	416	17.68	17.43	17.87	17.90	16.92	17.05	17.25	17.07
2.113	0.65	1.57	Q8VI36	Paxillin	Pxn	4027	18	18	18	50.1	50.1	50.1	64.476	0.0000	323.31	46747000	412	17.34	16.99	17.50	17.26	16.87	16.72	16.63	16.28
2.426	0.65	1.57	Q6PB44	Tyrosine-protein phosphatase non-receptor type 23	Ptpn23	2601	33	33	33	25.8	25.8	25.8	185.21	0.0000	146.14	54720000	384	17.54	17.03	17.50	17.54	16.58	16.88	16.73	16.84
1.855	0.65	1.57	Q6P8X1	Sorting nexin-6;Sorting nexin-6, N-terminally processed	Snx6	2585	33	31	30	69.7	67.7	64	46.648	0.0000	323.31	128150000	845	17.51	17.05	17.54	17.41	16.89	16.93	16.81	16.28
0.623	0.65	1.57	Q9ERV1	Probable E3 ubiquitin-protein ligase makorin-2	Mkrn2	5321	5	5	5	18	18	18	46.596	0.0034	2.5442	2286000	9	14.01	13.84	13.86	13.89	12.62	12.30	13.65	14.45
1.994	0.65	1.56	Q3TDQ1	Dolichyl-diphosphooligosaccharide--protein glycosyltransferase subunit	Stt3b	1834	18	18	17	22.2	22.2	22	93.245	0.0000	69.715	66506000	262	18.46	18.23	18.64	18.50	17.42	17.99	18.11	17.72
0.890	0.65	1.56	Q6PCN7	Helicase-like transcription factor	Hltf	2605	9	9	9	13.3	13.3	13.3	113.32	0.0000	28.773	3643500	29	14.00	13.19	14.95	13.89	13.34	13.49	13.22	13.41
2.283	0.65	1.56	P04117	Fatty acid-binding protein, adipocyte	Fabp4	636	10	10	10	68.9	68.9	68.9	14.65	0.0000	83.553	50391000	326	17.57	17.79	17.44	17.75	16.65	17.16	17.20	16.95
3.767	0.65	1.56	P61982	14-3-3 protein gamma;14-3-3 protein gamma, N-terminally processed	Ywhag	1399	36	31	28	96	93.1	93.1	28.302	0.0000	323.31	741920000	1250	20.80	20.57	20.69	20.80	20.09	19.98	20.22	19.99
1.863	0.64	1.56	P26450;Q64	Phosphatidylinositol 3-kinase regulatory subunit alpha	Pik3r1	925	27	26	26	39.1	37.2	37.2	83.516	0.0000	137.4	47714000	324	18.31	17.97	17.76	17.97	17.41	17.62	17.47	16.93
2.643	0.64	1.56	Q8BWU5	Probable tRNA N6-adenosine threonylcarbamoyltransferase	Osgep	3267	10	10	10	46.9	46.9	46.9	36.3	0.0000	67.894	18494000	184	16.34	16.11	16.65	16.26	15.59	15.78	15.81	15.61
1.684	0.64	1.56	Q00993	Tyrosine-protein kinase receptor UFO	Axl	1702	9	9	8	15.2	15.2	13.5	98.19	0.0000	26.476	3799700	31	13.85	13.33	13.75	13.85	13.43	12.77	13.23	12.78
1.035	0.64	1.56	Q80YE7	Death-associated protein kinase 1	Dapk1	2902	19	19	19	19.2	19.2	19.2	161.44	0.0000	55.716	10651000	99	14.66	14.15	15.01	15.05	13.86	14.57	14.39	13.50
1.324	0.64	1.56	Q8R1F9	Ribonuclease P protein subunit p40	Rpp40	3787	3	3	3	8	8	8	41.546	0.0017	3.2662	1177200	17	13.36	13.44	13.28	13.39	12.34	12.23	13.11	13.22
1.290	0.64	1.56	Q5SWU9	Acetyl-CoA carboxylase 1;Biotin carboxylase	Acaca	2157	48	48	41	28.9	28.9	25	265.25	0.0000	192.56	42133000	350	16.29	15.83	16.30	15.80	15.66	15.59	15.66	14.74
0.481	0.64	1.56	Q924W5	Structural maintenance of chromosomes protein 6	Smc6	4302	3	3	3	3.3	3.3	3.3	127.2	0.0025	2.8252	1197700	13	13.55	13.28	13.66	12.52	11.21	12.49	12.88	13.86
1.650	0.64	1.56	Q60766	Immunity-related GTPase family M protein 1	Irgm1	2204	22	22	22	56.5	56.5	56.5	46.551	0.0000	323.31	63359000	402	17.23	16.91	16.76	17.07	16.82	16.06	16.47	16.05
0.926	0.64	1.56	O54965	E3 ubiquitin-protein ligase RNF13	Rnf13	438	4	4	4	11.5	11.5	11.5	42.732	0.0000	18.104	3883300	37	14.54	14.14	15.14	14.37	14.41	13.88	14.21	13.14
0.293	0.64	1.56	Q9JKX4	Protein AATF	Aatf	5462	3	3	3	7.8	7.8	7.8	59.481	0.0000	9.9055	1279200	11	13.82	14.18	13.81	14.09	11.87	13.94	15.64	11.89
3.052	0.64	1.56	Q62351	Transferrin receptor protein 1	Tfrc	2381	26	26	26	45.6	45.6	45.6	85.73	0.0000	176.95	54471000	297	17.31	16.92	17.10	17.18	16.45	16.65	16.52	16.34
1.575	0.64	1.56	P47226	Testin	Tes	1115	15	15	15	32.2	32.2	32.2	47.983	0.0000	197.61	48438000	376	17.48	17.20	17.29	17.41	17.21	16.45	16.87	16.28
2.394	0.64	1.56	Q9ESX5	H/ACA ribonucleoprotein complex subunit 4	Dkc1	5350	20	20	20	47.3	47.3	47.3	57.401	0.0000	187.03	39135000	354	16.88	16.29	16.80	16.77	15.98	16.15	16.10	15.95
0.678	0.64	1.56	Q99L43	Phosphatidate cytidylyltransferase 2	Cds2	4407	3	3	3	11.7	11.7	11.7	51.313	0.0000	30.457	9911400	91	15.68	14.65	16.50	16.00	14.41	15.09	15.44	15.32
1.342	0.64	1.56	Q6P5F9	Exportin-1	Xpo1	2578	27	27	27	31.2	31.2	31.2	123.09	0.0000	306.22	96605000	571	17.92	17.49	17.72	17.89	17.42	16.42	17.37	17.25
2.504	0.64	1.56	Q91WM3	U3 small nucleolar RNA-interacting protein 2	Rrp9	4121	8	8	8	19.8	19.8	19.8	52.106	0.0000	29.921	7643300	98	15.59	15.22	15.60	15.45	14.69	14.63	14.90	15.08
1.152	0.64	1.56	Q6Y685	Transforming acidic coiled-coil-containing protein 1	Tacc1	2671	4	4	4	7	7	7	83.951	0.0002	5.4664	1589200	36	13.53	13.69	13.44	13.30	12.74	13.29	13.27	12.10
2.110	0.64	1.56	P70398	Probable ubiquitin carboxyl-terminal hydrolase FAF-X	Usp9x	1564	49	49	49	24.8	24.8	24.8	290.71	0.0000	248.34	65875000	618	16.57	16.13	16.53	16.51	15.77	15.49	16.11	15.80
2.820	0.64	1.56	Q8CI59	Metalloreductase STEAP3	Steap3	3541	17	17	17	37.5	37.5	37.5	54.748	0.0000	104.34	54839000	347	17.34	17.04	17.45	17.19	16.66	16.55	16.79	16.45
2.002	0.64	1.56	Q61595	Kinectin	Ktn1	2316	18	18	18	19.9	19.9	19.9	152.59	0.0000	57.494	10503000	114	15.16	14.90	15.45	15.51	14.69	14.86	14.49	14.42
0.629	0.64	1.56	Q8VCC1	15-hydroxyprostaglandin dehydrogenase [NAD(+)]	Hpgd	3915	7	7	7	43.5	43.5	43.5	29.18	0.0000	39.868	5691100	47	14.09	13.60	15.17	15.13	13.71	14.20	14.41	13.13
1.162	0.64	1.55	Q5DU37	Zinc finger FYVE domain-containing protein 26	Zfyve26	2099	12	12	12	7.3	7.3	7.3	282.96	0.0000	46.254	5982800	65	14.53	14.02	14.15	14.47	14.04	13.06	14.14	13.39
0.424	0.64	1.55	Q9ESW4	Acylglycerol kinase, mitochondrial	Agk	5348	9	9	9	30.2	30.2	30.2	46.975	0.0000	12.987	6138100	60	15.48	14.80	16.05	15.48	15.07	14.71	16.23	13.24
0.977	0.64	1.55	P63087	Serine/threonine-protein phosphatase PP1-gamma catalytic subunit	Ppp1cc	1489	21	4	3	63.2	12.7	9.3	36.983	0.0000	27.669	40884000	133	18.16	17.88	18.49	18.38	17.55	18.26	17.76	16.79
0.621	0.64	1.55	Q9CQM5	Thioredoxin domain-containing protein 17	Txndc17	4612	4	4	4	28.5	28.5	28.5	14.015	0.0000	46.096	4606500	62	15.41	15.07	14.31	15.20	14.94	13.12	14.86	14.55
1.139	0.64	1.55	Q8VBZ0	Dehydrogenase/reductase SDR family member on chromosome X hom	Dhrsx	3901	3	3	3	13.1	13.1	13.1	35.957	0.0000	8.2372	1577400	28	13.23	13.75	14.25	14.14	12.96	13.71	12.93	13.23
2.535	0.63	1.55	Q9QYB1	Chloride intracellular channel protein 4	Clic4	5566	20	20	20	88.9	88.9	88.9	28.729	0.0000	323.31	169020000	557	18.69	18.49	18.94	18.77	18.22	18.01	18.26	17.87
2.432	0.63	1.55	Q9ERG0	LIM domain and actin-binding protein 1	Lima1	5309	19	19	17	31.1	31.1	28.6	84.059	0.0000	44.918	22487000	206	16.85	16.45	16.94	16.74	15.96	16.16	16.34	15.98
0.567	0.63	1.55	Q8R2V5	Arf-GAP with dual PH domain-containing protein 2	Adap2	3823	5	5	5	18.6	18.6	18.6	43.989	0.0000	13.747	3499700	21	14.20	14.29	14.23	14.19	14.41	12.23	13.32	14.41
0.782	0.63	1.55	Q8BJW5	Nucleolar protein 11	Nol11	3088	5	5	5	7.2	7.2	7.2	80.817	0.0000	7.3087	2051300	32	14.62	14.20	14.34	14.15	13.04	14.74	13.18	13.81
1.546	0.63	1.55	Q9ES00	Ubiquitin conjugation factor E4 B	Ube4b	5322	19	19	19	27.2	27.2	27.2	133.32	0.0000	169.64	16384000	178	15.47	14.86	15.52	15.74	14.72	15.09	14.68	14.56
0.971	0.63	1.55	Q811U4	Mitofusin-1	Mfn1	2940	5	5	5	9.6	9.6	9.6	83.725	0.0000	29.84	2208900	19	14.12	13.12	14.67	13.99	13.47	13.19	13.18	13.53
0.837	0.63	1.55	P97765	WW domain-binding protein 2	Wbp2	1668	3	3	3	13.8	13.8	13.8	28.032	0.0000	6.2419	3403400	18	13.55	14.45	14.81	15.08	14.29	13.56	13.55	13.96
1.302	0.63	1.55	Q64455	Receptor-type tyrosine-protein phosphatase eta	Ptprj	2445	15	15	15	13.1	13.1	13.1	136.77	0.0000	73.657	14105000	130	16.60	15.81	16.40	16.40	16.18	15.26	15.68	15.55
0.324	0.63	1.55	Q9D173	Mitochondrial import receptor subunit TOM7 homolog	Tomm7	4900	1	1	1	30.9	30.9	30.9	6.1773	0.0002	5.8757	7429500	48	14.74	15.35	16.10	15.97	13.12	16.50	15.80	14.22
0.355	0.63	1.55	Q9D753	Exosome complex component RRP43	Exosc8	5020	6	6	6	29.7	29.7	29.7	29.949	0.0000	25.614	6160900	79	15.07	15.35	15.27	15.11	15.14	15.13	15.69	12.32

3.951	0.63	1.55	E9Q912		Rap1gds1	251	23	23	22	47.3	47.3	46.3	66.076	0.0000	226.14	58364000	370	17.56	17.58	17.82	17.59	16.97	17.11	16.97	16.99
0.750	0.63	1.55	P35285	Ras-related protein Rab-22A	Rab22a	1017	7	5	5	55.2	43.8	43.8	21.802	0.0000	110.07	11240000	90	16.34	16.05	15.61	15.88	15.59	14.25	16.05	15.48
2.946	0.63	1.55	P62137	Serine/threonine-protein phosphatase PP1-alpha catalytic subunit	Ppp1ca	1404	22	22	5	63.3	63.3	13.9	37.54	0.0000	323.31	192260000	667	18.88	18.51	18.67	18.85	18.17	18.25	17.97	18.00
2.162	0.63	1.55	Q9D7X3	Dual specificity protein phosphatase 3	Dusp3	5043	9	9	9	69.2	69.2	69.2	20.472	0.0000	323.31	120590000	426	19.44	19.19	19.57	19.53	18.74	18.55	19.17	18.74
0.656	0.63	1.55	Q9Z0W3	Nuclear pore complex protein Nup160	Nup160	5790	18	18	18	21.7	21.7	21.7	158.23	0.0000	142.29	15316000	152	16.72	14.97	15.72	15.14	15.04	14.75	15.66	14.60
2.060	0.63	1.55	Q8BVL3	Sorting nexin-17	Snx17	3244	16	16	16	43.2	43.2	43.2	52.797	0.0000	96.733	31058000	230	16.56	16.18	16.73	16.54	15.95	16.15	15.81	15.59
2.805	0.63	1.55	P29416	Beta-hexosaminidase subunit alpha	Hexa	969	26	26	26	43.8	43.8	43.8	60.612	0.0000	323.31	173130000	671	18.99	18.92	19.29	19.33	18.48	18.52	18.63	18.39
3.347	0.63	1.55	Q05512;Q8\	Serine/threonine-protein kinase MARK2	Mark2	1740	15	15	11	25.8	25.8	20	86.305	0.0000	86.57	21568000	215	15.96	15.66	15.88	15.89	15.18	15.16	15.41	15.12
2.856	0.63	1.55	P47757	F-actin-capping protein subunit beta	Capzb	1121	30	30	30	81.6	81.6	81.6	31.345	0.0000	323.31	643890000	1148	20.35	19.92	20.16	20.29	19.66	19.48	19.65	19.42
3.191	0.63	1.55	P53810	Phosphatidylinositol transfer protein alpha isoform	Pitpna	1240	29	29	27	86.3	86.3	83	31.893	0.0000	278.85	272300000	878	19.41	19.21	19.23	19.43	18.73	18.72	18.84	18.47
2.180	0.63	1.55	Q6P549	Phosphatidylinositol 3,4,5-trisphosphate 5-phosphatase 2	Inppl1	2568	41	39	37	40.1	38.7	36.9	138.97	0.0000	323.31	72598000	414	18.17	17.66	17.81	17.63	17.24	17.35	17.24	16.92
1.846	0.63	1.55	Q8BJU0	Small glutamine-rich tetratricopeptide repeat-containing protein alpha	Sgta	3086	6	6	6	24.4	24.4	24.4	34.322	0.0000	9.968	8073600	73	14.77	15.55	14.94	15.11	14.47	14.35	14.67	14.35
0.732	0.63	1.55	Q9D8X1	Copper homeostasis protein cutC homolog	Cutc	5080	7	7	7	46.7	46.7	46.7	29.003	0.0000	65.843	3694800	55	14.97	13.91	14.39	14.45	13.53	12.94	14.59	14.14
0.816	0.63	1.55	Q8R0S2;Q3	IQ motif and SEC7 domain-containing protein 1	Iqsec1	3765	14	14	10	17.9	17.9	13.7	108.01	0.0000	29.592	12264000	107	16.10	14.97	15.30	14.76	14.68	14.24	15.34	14.37
1.730	0.63	1.54	P97363	Serine palmitoyltransferase 2	Sptlc2	1631	19	19	19	51.1	51.1	51.1	62.981	0.0000	220.82	44297000	314	17.19	16.93	17.35	17.17	16.82	16.02	16.65	16.63
1.963	0.63	1.54	Q8BNU0	Armadillo repeat-containing protein 6	Armc6	3155	8	8	8	29.7	29.7	29.7	50.683	0.0000	67.483	12470000	132	15.73	15.50	15.97	15.85	15.03	14.83	15.18	15.50
2.661	0.63	1.54	A3KMP2	Tetratricopeptide repeat protein 38	Ttc38	55	8	8	8	24.7	24.7	24.7	52.223	0.0000	43.197	9941400	78	15.34	15.54	15.59	15.44	15.12	14.89	14.81	14.59
1.282	0.63	1.54	Q2EMV9	Poly [ADP-ribose] polymerase 14	Parp14	1796	50	50	50	34.6	34.6	34.6	203.8	0.0000	286.34	48370000	407	16.79	16.16	16.36	15.96	15.92	15.96	15.74	15.14
2.704	0.62	1.54	Q9CQC6	Basic leucine zipper and W2 domain-containing protein 1	Bzw1	4582	22	22	21	58.7	58.7	57	48.043	0.0000	277.82	73775000	324	17.93	17.88	18.02	18.31	17.28	17.29	17.51	17.54
2.169	0.62	1.54	Q6PDI5	Proteasome-associated protein ECM29 homolog	Ecm29	2613	51	51	51	44.1	44.1	44.1	203.7	0.0000	323.31	91405000	799	17.77	17.18	17.37	17.47	16.76	16.61	17.05	16.87
1.774	0.62	1.54	Q9Z1S8	GRB2-associated-binding protein 2	Gab2	5821	7	7	6	17.3	17.3	15	73.207	0.0000	40.424	7242200	97	15.36	14.74	15.50	15.46	14.55	14.52	14.83	14.66
2.097	0.62	1.54	Q8CI95;S4R	Oxysterol-binding protein-related protein 11	Osbpl11	3546	25	25	25	40.2	40.2	40.2	83.628	0.0000	123.93	37356000	315	17.06	16.55	16.75	16.50	16.03	15.85	16.18	16.31
2.118	0.62	1.54	Q924T2	28S ribosomal protein S2, mitochondrial	Mrps2	4300	6	6	6	29.2	29.2	29.2	32.313	0.0000	70.838	12846000	112	16.50	15.90	16.47	16.42	15.58	15.77	15.86	15.58
0.745	0.62	1.54	Q8VC42	Uncharacterized protein C18orf8 homolog	Mic1	3909	8	8	8	18.6	18.6	18.6	74.922	0.0000	27.71	3994200	40	14.89	14.01	14.83	14.21	14.79	13.88	13.64	13.13
1.097	0.62	1.54	Q60996	Serine/threonine-protein phosphatase 2A 56 kDa regulatory subunit gar	Ppp2r5c	2236	10	8	8	23.5	17.9	17.9	60.824	0.0000	68.733	9164500	109	15.46	15.56	15.79	15.73	15.32	14.28	14.87	15.58
2.477	0.62	1.54	P31938	Dual specificity mitogen-activated protein kinase kinase 1	Map2k1	991	25	25	17	62.8	62.8	48.6	43.474	0.0000	211.21	78235000	454	17.82	17.49	17.93	17.94	17.34	17.27	17.01	17.07
2.507	0.62	1.54	P51174	Long-chain specific acyl-CoA dehydrogenase, mitochondrial	Acadl	1192	32	32	32	55.8	55.8	55.8	47.907	0.0000	317.86	255740000	1010	19.10	18.82	19.17	19.19	18.36	18.23	18.67	18.54
3.253	0.62	1.54	Q64521	Glycerol-3-phosphate dehydrogenase, mitochondrial	Gpd2	2453	45	45	45	65.6	65.6	65.6	80.953	0.0000	323.31	148980000	855	17.70	17.56	17.90	17.85	17.21	17.03	17.23	17.06
1.118	0.62	1.54	O70480	Vesicle-associated membrane protein 4	Vamp4	504	4	4	4	39	39	39	16.353	0.0000	54.043	7611100	87	15.76	14.88	15.98	16.08	15.31	15.03	15.00	14.88
2.389	0.62	1.54	Q3UPF5	Zinc finger CCCH-type antiviral protein 1	Zc3hav1	2013	30	30	30	41.2	41.2	41.2	106.69	0.0000	181.58	32599000	360	16.77	16.27	16.40	16.42	15.91	15.85	16.01	15.60
1.228	0.62	1.54	Q99K70;Q7	Ras-related GTP-binding protein C;Ras-related GTP-binding protein D	Rragc;Rragd	4364	14	14	14	34.7	34.7	34.7	44.12	0.0000	53.67	17672000	239	16.25	16.03	16.04	15.64	15.39	15.73	14.70	15.65
2.387	0.62	1.54	Q8BJY1	26S proteasome non-ATPase regulatory subunit 5	Psmd5	3090	29	29	29	70.2	70.2	70.2	55.971	0.0000	323.31	113900000	733	18.00	17.81	18.28	18.33	17.46	17.43	17.68	17.39
0.797	0.62	1.54	P70182;P70	Phosphatidylinositol 4-phosphate 5-kinase type-1 alpha	Pip5k1a	1530	6	5	5	17.2	14.7	14.7	60.484	0.0000	18.905	5004500	68	15.03	14.30	14.46	14.86	13.52	14.83	14.42	13.41
3.360	0.62	1.54	Q9QZD9	Eukaryotic translation initiation factor 3 subunit I	Eif3i	5594	21	21	21	70.5	70.5	70.5	36.46	0.0000	322.13	124840000	578	17.76	17.66	17.88	17.92	17.25	17.11	17.34	17.04
2.318	0.62	1.53	Q3UMB9	WASH complex subunit 7	Kiaa1033	1999	35	35	35	39	39	39	136.37	0.0000	305.48	74192000	501	18.29	18.10	18.07	17.85	17.63	17.33	17.66	17.21
0.790	0.62	1.53	O35130	Ribosomal RNA small subunit methyltransferase NEP1	Emg1	340	4	4	4	22.5	22.5	22.5	26.974	0.0000	9.8384	2589500	27	14.85	13.37	14.74	14.39	13.71	13.22	14.15	13.80
1.844	0.62	1.53	P32921	Tryptophan--tRNA ligase, cytoplasmic;T1-TrpRS;T2-TrpRS	Wars	998	24	24	24	62	62	62	54.357	0.0000	323.31	144330000	587	18.07	17.90	18.59	18.46	17.59	17.72	17.80	17.44
2.453	0.62	1.53	Q6VN19	Ran-binding protein 10	Ranbp10	2663	12	12	12	30.2	30.2	30.2	67.188	0.0000	113.44	21912000	205	16.49	16.24	16.50	16.62	15.89	16.07	15.87	15.56
2.637	0.62	1.53	Q9QUJ7	Long-chain-fatty-acid--CoA ligase 4	Acsl4	5515	35	35	33	59.2	59.2	56.8	79.076	0.0000	253.05	95855000	645	18.35	18.02	17.94	17.84	17.40	17.49	17.50	17.28
2.483	0.62	1.53	Q9DC61	Mitochondrial-processing peptidase subunit alpha	Pmpca	5197	22	22	22	51.3	51.3	51.3	58.278	0.0000	235.9	61220000	467	17.19	16.77	17.13	16.98	16.50	16.13	16.47	16.52
1.447	0.62	1.53	Q6ZQA0	Neurobeachin-like protein 2	Nbeal2	2693	23	23	23	14.2	14.2	14.2	301.92	0.0000	101.09	13006000	181	15.01	14.47	15.52	15.11	14.45	14.32	14.60	14.27
3.093	0.62	1.53	Q9DBG3	AP-2 complex subunit beta	Ap2b1	5151	51	51	28	55.2	55.2	36.3	104.58	0.0000	323.31	201480000	1114	17.91	17.68	18.04	18.01	17.18	17.30	17.44	17.27
1.088	0.62	1.53	Q8K1C9	Leucine-rich repeat-containing protein 41	Lrrc41	3624	5	5	5	9	9	9	88.179	0.0000	15.575	1993000	36	14.36	13.88	13.87	13.65	13.64	13.39	13.69	12.59
2.905	0.62	1.53	Q60854	Serpin B6	Serpinb6	2212	44	44	44	94.4	94.4	94.4	42.598	0.0000	323.31	563390000	2098	19.93	19.78	19.71	19.62	19.14	19.03	19.39	19.02
2.540	0.61	1.53	Q8K183	Pyridoxal kinase	Pdxk	3617	20	20	20	62.2	62.2	62.2	35.015	0.0000	323.31	327760000	839	19.65	19.52	19.92	19.94	19.04	19.17	19.35	19.02
1.153	0.61	1.53	P11440	Cyclin-dependent kinase 1	Cdk1	744	12	10	10	54.2	47.8	47.8	34.106	0.0000	40.695	11099000	99	14.77	14.79	15.85	15.39	14.65	14.32	14.79	14.60
2.089	0.61	1.53	Q80VP0	Tectonin beta-propeller repeat-containing protein 1	Tecpr1	2853	20	20	20	23.2	23.2	23.2	130.26	0.0000	57.631	19645000	189	15.72	15.46	15.86	15.86	15.11	14.77	15.21	15.37
0.896	0.61	1.53	Q8R2Y0	Monoacylglycerol lipase ABHD6	Abhd6	3824	8	8	8	32.1	32.1	32.1	38.204	0.0000	14.253	3481800	29	14.24	13.33	13.83	13.75	13.53	13.61	13.22	12.34
0.962	0.61	1.53	Q8C4B4	Protein unc-119 homolog B	Unc119b	3385	5	5	4	27.1	27.1	23.5	28.302	0.0000	34.59	5281200	78	15.27	15.10	14.79	15.33	14.69	13.61	14.84	14.89
1.834	0.61	1.53	Q9D0L8	mRNA cap guanine-N7 methyltransferase	Rnmt	4876	21	21	21	48.6	48.6	48.6	53.291	0.0000	215.79	36940000	347	17.10	16.75	17.31	17.10	16.45	16.35	16.83	16.18
2.653	0.61	1.53	P08113	Endoplasmin	Hsp90b1	680	73	71	71	66.3	64.6	64.6	92.475	0.0000	323.31	1570500000	3003	20.40	20.07	20.55	20.41	19.71	19.78	19.90	19.59
0.616	0.61	1.53	Q9CR56	NF-kappa-B inhibitor-interacting Ras-like protein 2	Nkiras2	4668	2	2	2	12.6	12.6	12.6	21.494	0.0004	5.0157	6784300	58	15.41	15.41	15.34	15.85	15.32	15.48	15.23	13.53
0.799	0.61	1.53	P70261	Paladin	Pald1	1541	10	10	10	16.8	16.8	16.8	96.739	0.0000	48.271	9570400	122	14.95	14.59	15.53	15.59	15.01	14.77	14.75	13.69
2.239	0.61	1.53	Q68FD5	Clathrin heavy chain 1	Cltc	2473	133	133	133	68.2	68.2	68.2	191.55	0.0000	323.31	2046700000	5207	20.54	20.22	20.20	20.37	19.87	19.41	19.95	19.65
1.543	0.61	1.53	O55135	Eukaryotic translation initiation factor 6	Eif6	459	7	7	7	46.1	46.1	46.1	26.511	0.0000	243.28	45737000	173	18.01	18.23	17.86	17.84	17.84	17.23	17.50	16.93
1.082	0.61	1.53	Q9R117	Non-receptor tyrosine-protein kinase TYK2	Tyk2	5646	9	8	8	10.5	9.5	9.5	133.31	0.0000	11.785	4801600	41	14.30	14.14	15.29	14.80	14.12	13.70	14.34	13.94
2.137	0.61	1.53	P42669	Transcriptional activator protein Pur-alpha	Pura	1081	13	11	11	47.7	41.7	41.7	34.883	0.0000	228.92	30980000	193	17.35	16.94	17.59	17.12	16.60	16.80	16.65	16.50
3.699	0.61	1.53	P83940	Transcription elongation factor B polypeptide 1	Tceb1	1605	9	9	9	98.2	98.2	98.2	12.473	0.0000	104.49	37516000	230	17.55	17.43	17.52	17.53	16.97	17.04	16.71	16.87
2.169	0.61	1.53	Q8VBW6	NEDD8-activating enzyme E1 regulatory subunit	Nae1	3900	16	16	16	42.5	42.5	42.5	60.273	0.0000	123.39	22963000	220	15.91	15.77	16.08	15.94	15.22	14.97	15.57	15.50
1.058	0.61	1.52	P11881	Inositol 1,4,5-trisphosphate receptor type 1	Itpr1	751	5	5	5	2.7	2.7	2.7	313.16	0.0000	9.2333	2290500	25	13.60	12.54	13.82	13.27	12.41	12.87	12.71	12.81
0.924	0.61	1.52	Q8CIV8	Tubulin-specific chaperone E	Tbce	3563	10	10	10	31.3	31.3	31.3	59.085	0.0000	45.079	11860000	136	15.36	14.94	15.99	15.58	14.98	15.28	15.05	14.12
3.015	0.61	1.52	Q8C1B7	Septin-11	Sep.11	3363	26	26	15	52.7	52.7	35.5	49.694	0.0000	323.31	93367000	491	18.15	17.78	17.86	18.08	17.33	17.48	17.38	17.26
0.958	0.61	1.52	Q99M08	Uncharacterized protein C4orf3 homolog		4452	2	2	2	24.6	24.6	24.6	7.4043	0.0000	48.406	28367000	91	17.71	17.85	18.06	17.86	16.32	17.46	17.60	17.67
2.740	0.61	1.52	P13439	Uridine 5-monophosphate synthase;Orotate phosphoribosyltransferase;	Umps	765	26	26	26	62.8	62.8	62.8	52.292	0.0000	250.66	51136000	409	16.99	16.85	17.09	17.09	16.48	16.18	16.63	16.29
1.842	0.61	1.52	Q6PCM1	Lysine-specific demethylase 3A	Kdm3a	2604	4	4	4	4.7	4.7	4.7	147.85	0.0000	8.9769	1729200	15	13.77	13.55	13.78	13.84	13.39	12.88	13.44	12.80
0.876	0.61	1.52	Q8CIM8	Integrator complex subunit 4	Ints4	3560	11	11	11	18.2	18.2	18.2	108.19	0.0000	46.493	4701400	87	13.81	14.00	14.65	14.65	13.74	13.84	14.19	12.91
2.397	0.60	1.52	P56135	ATP synthase subunit f, mitochondrial	Atp5j2	1275	7	7	7	53.4	53.4	53.4	10.344	0.0000	20.357	105580000	203	19.22	18.80	19.04	19.16	18.47	18.24	18.71	18.38
1.403	0.60	1.52	Q61696;P17	Heat shock 70 kDa protein 1A;Heat shock 70 kDa protein 1B	Hspa1a;Hspa1b	2324	33	25	19	52.7	41.7	32.1	70.078	0.0000	66.607	31779000	240	16.41	16.20	15.74	15.93	15.33	15.98	15.37	15.18
1.563	0.60	1.52	Q3UW53	Protein Niban	Fam129a	2040	49	49	49	56.4	56.4	56.4	102.65	0.0000	323.31	183880000	824	19.83	19.16	19.84	19.46	19.10	19.16	19.03	18.59
3.713	0.60	1.52	P14824	Annexin A6	Anxa6	786	78	78	77	86.2	86.2	85.9	75.884	0.0000	323.31	821550000	2620	19.43	19.40	19.43	19.54	18.86	18.68	19.01	18.83
3.949	0.60	1.52	P30999	Catenin delta-1	Ctnnd1	982	44	44	44	58.5	58.5	58.5	104.92	0.0000	323.31	97535000	764	17.11	16.93	17.14	16.93	16.38	16.53	16.43	16.37
1.790	0.60	1.52	Q8C166	Copine-1	Cpne1	3355	20	19	19	47.2	45.5	45.5	58.886	0.0000	139.43	82054000	342	18.46	18.24	18.79	18.43	18.02	18.16	17.82	17.51
2.744	0.60	1.52	Q99JW4;Q9	LIM and senescent cell antigen-like-containing domain protein 1	Lims1	4352	17	17	17	51.1	51.1	51.1	37.24	0.0000	77.39	58165000	584	16.78	16.32	16.71	16.67	16.06	15.96	16.13	15.92
1.518	0.60	1.52	Q8C2E7	WASH complex subunit strumpellin	Kiaa0196	3370	38	38	37	35	35	33.6	134.11	0.0000	165.31	67823000	620	16.85	16.12	16.54	16.35	16.06	15.86	16.08	15.45
1.829	0.60	1.52	Q78IK4	MICOS complex subunit Mic27	Apool	2723	12	12	12	56.2	56.2	56.2	29.26	0.0000	158.48	38082000	310	16.84	16.16	16.92	16.77	15.97	16.05	16.16	16.09
3.724	0.60	1.52	Q07076	Annexin A7	Anxa7	1753	33	32	32	52.7	52.7	52.7	49.925	0.0000	323.31	313780000	1119	18.84	18.97	18.99	18.92	18.33	18.14	18.40	18.44
1.465	0.60	1.52	Q6NZC7	SEC23-interacting protein	Sec23ip	2544	19	19	19	24.8	24.8	24.8	110.78	0.0000	66.056	27280000	215	16.69	16.10	16.56	15.96	15.60	15.98	15.91	15.42
0.824	0.60	1.52	Q8CE50	Sorting nexin-30	Snx30	3467	16	16	16	51.5	51.5	51.5	49.52	0.0000	127.34	14962000	163	15.91	15.56	15.06	15.04	15.13	14.78	15.30	13.96
0.526	0.60	1.51	Q6ZWY8	Thymosin beta-10	Tmsb10	2711	2	2	2	36.4	36.4	36.4	5.0256	0.0004	4.5672	3260200	29	14.55	15.04	14.35	13.87	12.96	13.47	15.14	13.85
0.789	0.60	1.51	Q9D0B6	Protein PBDC1	Pbdc1	4856	6	6	6	36.9	36.9	36.9	22.223	0.0000	10.611	3851200	23	13.85	14.93	14.32	14.14	14.52	13.32	13.80	13.20
1.097	0.60	1.51	Q9QWV9	Cyclin-T1	Ccnt1	5529	6	6	6	14.8	14.8	14.8	80.597	0.0000	110.82	4771500	62	14.73	14.41	14.77	14.79	14.27	14.27	14.48	13.28
3.517	0.60	1.51	Q9CQI6	Coactosin-like protein	Cotl1	4602	28	28	28	99.3	99.3	99.3	15.944	0.0000	323.31	607760000	1065	20.18	20.12	20.13	20.08	19.52	19.36	19.74	19.50
1.095	0.60	1.51	Q6Q899	Probable ATP-dependent RNA helicase DDX58	Ddx58	2650	47	47	47	57.1	57.1	57.1	105.97	0.0000	287.09	82742000	581	17.49	16.92	16.80	16.54	16.71	16.63	16.13	15.88
1.713	0.60	1.51	Q6ZPE2	Myotubularin-related protein 5	Sbf1	2674	19	19	19	17.1	17.1	17.1	208.69	0.0000	60.766	24494000	189	16.51	16.49	17.07	16.92	16.08	16.05	16.50	15.97
0.649	0.60	1.51	Q80UK8	Integrator complex subunit 2	Ints2	2831	6	6	6	9.1	9.1	9.1	133.51	0.0000	28.215	1279700	18	13.64	12.97	14.49	13.19	13.33	13.38	13.06	12.15
0.929	0.60	1.51	Q9CQU0	Thioredoxin domain-containing protein 12	Txndc12	4634	3	3	3	29.4	29.4	29.4	19.048	0.0009	3.9211	1370100	19	13.97	14.18	12.82	13.25	12.83	12.96	12.89	13.17
2.753	0.59	1.51	Q99L04	Dehydrogenase/reductase SDR family member 1	Dhrs1	4402	31	31	31	85	85	85	34.005	0.0000	323.31	271520000	796	19.35	19.23	19.38	18.97	18.53	18.76	18.53	18.71
0.995	0.59	1.51	Q8BRH0	Transmembrane and TPR repeat-containing protein 3	Tmtc3	3181	9	9	9	13.7	13.7	13.7	104.2	0.0000	54.736	3250500	33	14.15	13.95	14.34	13.33	13.37	13.51	13.75	12.74
1.421	0.59	1.51	P18242	Cathepsin D	Ctsd	836	27	27	25	56.6	56.6	55.1	44.953	0.0000	323.31	1295100000	1402	21.13	21.23	21.36	21.72	20.58	20.35	21.13	21.01
1.343	0.59	1.51	Q99JF5	Diphosphomevalonate decarboxylase	Mvd	4337	12	12	12	47.4	47.4	47.4	44.072	0.0000	158.14	23278000	221	16.02	15.97	15.96	15.94	15.57	15.89	15.26	14.79
1.424	0.59	1.51	Q9D1G5	Leucine-rich repeat-containing protein 57	Lrrc57	4913	10	10	10	55.2	55.2	55.2	26.76	0.0000	61.871	10374000	123	15.74	15.12	15.56	15.31	14.51	15.28	14.96	14.60
2.679	0.59	1.51	Q9QYI3	DnaJ homolog subfamily C member 7	Dnajc7	5573	22	22	22	50.2	50.2	50.2	56.475	0.0000	105.47	30132000	343	15.61	15.45	15.80	15.67	15.21	15.07	15.09	14.79
0.540	0.59	1.51	Q05920	Pyruvate carboxylase, mitochondrial	Pc	1744	30	30	30	35.8	35.8	35.8	129.68	0.0000	105.32	37456000	357	16.10	15.52	16.36	16.38	16.02	15.04	16.50	14.44
1.798	0.59	1.51	Q8BGQ1	Spermatogenesis-defective protein 39 homolog	Vipas39	2987	10	10	10	27.1	27.1	27.1	56.624	0.0000	87.858	10403000	104	15.39	15.08	15.17	15.50	14.74	14.31	15.03	14.69
1.726	0.59	1.51	Q8BZN6	Dedicator of cytokinesis protein 10	Dock10	3316	56	56	56	31.6	31.6	31.6	245.76	0.0000	323.31	89185000	908	16.71	16.48	17.22	17.04	16.16	16.29	16.50	16.14
2.550	0.59	1.51	Q924K8	Metastasis-associated protein MTA3	Mta3	4295	14	11	9	29.6	25.5	20.8	67.076	0.0000	80.674	20205000	190	16.94	16.55	16.96	16.74	16.16	16.14	16.42	16.09
2.284	0.59	1.51	Q99KF1	Transmembrane emp24 domain-containing protein 9	Tmed9	4373	14	14	12	49.8	49.8	39.6	27.127	0.0000	229.95	75405000	235	18.38	18.00	18.38	17.93	17.67	17.44	17.73	17.49
2.110	0.59	1.51	Q7TMB8	Cytoplasmic FMR1-interacting protein 1	Cyfip1	2740	63	63	40	52.4	52.4	35.8	145.24	0.0000	323.31	241380000	1232	18.92	18.32	18.32	18.42	18.03	17.84	17.89	17.86
0.887	0.59	1.51	Q8VDI9	Alpha-1,2-mannosyltransferase ALG9	Alg9	3955	5	5	5	11.8	11.8	11.8	69.56	0.0004	4.7415	2048100	18	14.49	13.05	13.41	13.67	12.90	13.20	12.77	13.39
2.154	0.59	1.51	Q9DB05;P2	Alpha-soluble NSF attachment protein	Napa	5116	21	21	21	76.3	76.3	76.3	33.189	0.0000	302.15	198380000	760	18.49	18.29	18.77	18.73	18.12	17.98	18.11	17.71
3.017	0.59	1.51	Q99JI4	26S proteasome non-ATPase regulatory subunit 6	Psmd6	4340	22	22	22	57.1	57.1	57.1	45.536	0.0000	137.64	281210000	592	20.82	20.47	20.60	20.80	20.03	20.06	20.23	20.02
0.776	0.59	1.51	Q3UN02	Lysocardiolipin acyltransferase 1	Lclat1	2007	4	4	4	11.2	11.2	11.2	44.399	0.0000	8.477	1668800	12	14.60	13.46	13.75	13.41	13.23	13.00	13.91	12.72
2.004	0.59	1.51	Q8BP48	Methionine aminopeptidase 1	Metap1	3159	16	16	16	59.3	59.3	59.3	43.221	0.0000	93.344	58056000	273	17.93	17.57	17.51	17.33	16.90	17.08	17.22	16.77
1.005	0.59	1.50	E9Q9A9	2-5-oligoadenylate synthase 2	Oas2	252	5	5	5	9.4	9.4	9.4	85.05	0.0000	9.9362	2371600	14	13.94	13.48	13.68	12.80	13.39	12.83	12.54	12.79
2.335	0.59	1.50	Q6ZQ38	Cullin-associated NEDD8-dissociated protein 1	Cand1	2689	51	51	50	47.1	47.1	46.3	136.33	0.0000	323.31	160400000	1105	18.02	17.67	17.93	18.03	17.33	17.05	17.56	17.34
2.360	0.59	1.50	P50136	2-oxoisovalerate dehydrogenase subunit alpha, mitochondrial	Bckdha	1175	11	11	11	30.3	30.3	30.3	50.37	0.0000	61.532	14169000	164	15.87	15.53	16.15	15.92	15.31	15.26	15.19	15.36
1.260	0.59	1.50	Q9JLI6	Selenocysteine lyase	Scly	5480	19	19	18	48.1	48.1	47.2	47.173	0.0000	168.39	31824000	378	15.63	15.41	16.29	16.28	15.44	15.35	15.46	15.01
0.396	0.59	1.50	Q3UGY8	Brefeldin A-inhibited guanine nucleotide-exchange protein 3	Arfgef3	1964	6	6	6	4.6	4.6	4.6	240.09	0.0000	28.411	2112500	30	13.32	12.79	15.18	14.86	13.32	14.31	13.24	12.93
0.946	0.59	1.50	Q7TQI3	Ubiquitin thioesterase OTUB1	Otub1	2775	14	14	14	67.2	67.2	67.2	31.27	0.0000	242.89	44134000	231	18.06	17.55	16.82	16.86	16.84	16.91	16.77	16.42
1.579	0.59	1.50	Q8VD75	Huntingtin-interacting protein 1	Hip1	3944	40	40	39	51	51	50	115.2	0.0000	323.31	73852000	810	16.66	16.26	17.00	16.76	15.93	15.94	16.47	16.00
0.602	0.59	1.50	Q9DD03	Ras-related protein Rab-13	Rab13	5240	9	6	5	44.6	35.1	30.2	22.77	0.0000	6.4338	3378400	22	14.60	13.52	13.97	14.42	13.52	12.53	13.66	14.44
0.969	0.59	1.50	Q8JZU0	Nucleoside diphosphate-linked moiety X motif 13	Nudt13	3582	3	3	3	14.8	14.8	14.8	39.136	0.0000	13.881	1484600	21	14.40	13.98	13.50	13.98	13.37	13.47	13.94	12.73
0.946	0.59	1.50	O55022	Membrane-associated progesterone receptor component 1	Pgrmc1	442	10	10	10	32.8	32.8	32.8	21.694	0.0000	56.186	18398000	76	16.57	16.19	16.66	16.05	15.11	16.17	15.54	16.31
2.410	0.58	1.50	Q8CCJ3	E3 UFM1-protein ligase 1	Ufl1	3449	23	23	23	41.1	41.1	41.1	89.519	0.0000	202.6	32466000	352	16.42	16.00	16.35	16.35	15.67	15.77	15.88	15.47

1.220	0.58	1.50	Q9CYZ2	Tumor protein D54	Tpd52l2	4810	10	10	10	62.7	62.7	62.7	24.043	0.0000	65.213	19633000	128	16.46	16.69	16.32	16.46	15.47	15.81	15.73	16.59
1.341	0.58	1.50	Q9DC28	Casein kinase I isoform delta	Csnk1d	5188	9	9	5	25.1	25.1	13	47.316	0.0000	18.438	6817400	69	14.69	14.52	14.81	14.81	13.71	14.72	14.19	13.87
2.296	0.58	1.50	Q8VE11	Myotubularin-related protein 6	Mtmr6	3981	20	20	20	35.8	35.8	35.8	70.932	0.0000	74.136	24833000	303	15.87	15.62	16.09	15.90	15.49	15.22	15.39	15.05
0.950	0.58	1.50	Q9JJU8	SH3 domain-binding glutamic acid-rich-like protein	Sh3bgrl	5436	6	6	6	59.6	59.6	59.6	12.811	0.0000	137.49	17259000	183	16.30	16.87	15.97	16.19	15.71	16.33	15.12	15.84
0.537	0.58	1.50	P30115;P08	Glutathione S-transferase A3	Gsta3	973	15	15	15	60.6	60.6	60.6	25.36	0.0000	98.472	40981000	206	16.74	16.71	17.93	17.84	15.90	16.29	17.45	17.26
0.785	0.58	1.50	Q9D154;Q5	Leukocyte elastase inhibitor A	Serpinb1a	4896	16	16	16	44.9	44.9	44.9	42.574	0.0000	25.593	17027000	118	15.23	15.25	15.81	15.81	14.82	14.08	15.30	15.58
2.016	0.58	1.50	Q8C0C7	Phenylalanine--tRNA ligase alpha subunit	Farsa	3330	36	36	36	63.2	63.2	63.2	57.598	0.0000	323.31	160340000	721	19.05	18.51	19.08	18.86	18.26	18.09	18.49	18.33
0.789	0.58	1.50	Q9WUD1	STIP1 homology and U box-containing protein 1	Stub1	5708	9	9	9	35.5	35.5	35.5	34.909	0.0000	12.108	8694900	53	14.97	15.47	15.38	15.84	15.21	15.17	15.08	13.89
2.734	0.58	1.50	Q91X78	Erlin-1	Erlin1	4137	19	19	13	43.7	43.7	31.3	39.182	0.0000	85.506	105220000	340	19.98	19.54	19.80	19.68	19.22	19.03	19.30	19.13
1.178	0.58	1.50	Q9JJY4	Probable ATP-dependent RNA helicase DDX20	Ddx20	5439	11	11	11	21.9	21.9	21.9	91.709	0.0000	55	10344000	125	15.09	15.30	16.22	15.69	14.95	15.16	14.82	15.04
2.099	0.58	1.49	Q9JHU9	Inositol-3-phosphate synthase 1	Isyna1	5375	18	18	17	38.8	38.8	38.1	60.931	0.0000	295.85	87690000	387	18.43	18.08	18.68	18.38	17.80	17.67	18.05	17.74
2.504	0.58	1.49	Q9EPE9	Manganese-transporting ATPase 13A1	Atp13a1	5252	31	31	31	31.3	31.3	31.3	132.39	0.0000	151.4	64149000	390	16.90	16.55	16.90	16.94	16.17	16.20	16.48	16.12
1.758	0.58	1.49	Q9DBT5	AMP deaminase 2	Ampd2	5177	21	21	21	31.5	31.5	31.5	92.023	0.0000	51.9	32608000	300	16.48	16.07	16.81	16.65	15.92	16.09	15.97	15.72
1.076	0.58	1.49	Q91W90	Thioredoxin domain-containing protein 5	Txndc5	4098	9	9	9	32.9	32.9	32.9	46.415	0.0000	25.202	15867000	143	15.39	15.74	14.99	15.27	15.39	14.76	14.67	14.27
0.163	0.58	1.49	Q6ZPV2	DNA helicase INO80	Ino80	2685	4	4	4	3	3	3	176.52	0.0000	6.4063	5166200	8	12.21	16.64	16.89	15.95	16.34	13.63	16.17	13.23
2.717	0.58	1.49	Q64324	Syntaxin-binding protein 2	Stxbp2	2430	45	45	45	74.5	74.5	74.5	66.357	0.0000	323.31	216800000	1226	18.28	18.06	18.00	18.08	17.51	17.40	17.79	17.41
1.261	0.58	1.49	Q3U2S8	Voltage-gated hydrogen channel 1	Hvcn1	1913	8	8	8	34.6	34.6	34.6	31.242	0.0000	100.33	9528400	74	15.87	15.72	15.30	15.67	14.50	15.08	15.18	15.50
3.380	0.58	1.49	P70318	Nucleolysin TIAR	Tial1	1553	15	15	12	46.7	46.7	38	43.388	0.0000	218.76	49091000	309	18.37	18.21	18.14	18.06	17.61	17.75	17.57	17.54
1.595	0.58	1.49	Q8CI04	Conserved oligomeric Golgi complex subunit 3	Cog3	3535	5	5	5	7.8	7.8	7.8	93.282	0.0000	8.3602	3691200	55	14.63	14.16	14.36	14.72	13.67	13.71	14.31	13.90
0.293	0.57	1.49	Q99NH8	Triggering receptor expressed on myeloid cells 2	Trem2	4499	3	3	3	32.2	32.2	32.2	24.527	0.0000	29.906	9404100	84	14.10	15.88	16.19	16.34	15.86	15.53	15.65	13.16
2.934	0.57	1.49	P62259	14-3-3 protein epsilon	Ywhae	1410	32	29	29	85.5	85.5	85.5	29.174	0.0000	323.31	564100000	1397	20.60	20.53	20.36	20.83	20.06	20.00	20.01	19.96
0.279	0.57	1.49	D3Z4S3	Putative peptidyl-tRNA hydrolase PTRHD1	Ptrhd1	213	4	4	4	29.3	29.3	29.3	16.037	0.0000	12.369	10124000	55	15.05	15.86	16.02	15.77	15.51	15.79	16.41	12.69
2.763	0.57	1.49	Q61194	Phosphatidylinositol 4-phosphate 3-kinase C2 domain-containing subun	Pik3c2a	2274	11	11	11	10.6	10.6	10.6	190.76	0.0000	32.808	7415900	105	14.88	14.62	14.52	14.61	14.18	14.05	14.21	13.89
0.738	0.57	1.49	Q3TIU4	2,5-phosphodiesterase 12	Pde12	1850	11	11	11	28.1	28.1	28.1	67.513	0.0000	53.863	14863000	149	16.16	15.99	15.10	16.15	15.61	15.72	14.46	15.31
0.565	0.57	1.49	Q6PNC0	DmX-like protein 1	Dmxl1	2648	16	16	16	8.8	8.8	8.8	336	0.0000	40.599	7006600	81	15.54	14.66	15.44	14.11	14.57	14.44	13.44	15.01
1.895	0.57	1.49	Q9QXB9	Developmentally-regulated GTP-binding protein 2	Drg2	5535	22	22	22	54.1	54.1	54.1	40.718	0.0000	180.68	51589000	383	17.18	16.59	17.14	17.07	16.47	16.52	16.54	16.16
2.067	0.57	1.49	Q921G8	Gamma-tubulin complex component 2	Tubgcp2	4224	13	13	13	20	20	20	103.22	0.0000	36.167	8169400	133	14.55	14.27	14.66	14.64	13.78	13.94	13.81	14.30
0.963	0.57	1.49	Q921E2	Ras-related protein Rab-31	Rab31	4218	12	12	10	73.7	73.7	62.4	21.331	0.0000	168.58	38251000	255	17.35	17.21	17.81	17.22	17.18	16.55	17.37	16.21
1.217	0.57	1.49	Q9CZS1	Aldehyde dehydrogenase X, mitochondrial	Aldh1b1	4838	19	17	16	41.8	40.1	38.3	57.552	0.0000	100.26	31280000	270	15.91	16.09	16.65	15.87	15.58	15.55	15.97	15.12
0.803	0.57	1.49	P58044	Isopentenyl-diphosphate Delta-isomerase 1	Idi1	1304	6	6	6	34.4	34.4	34.4	26.289	0.0000	131.06	13356000	115	16.09	16.10	16.45	15.90	15.90	16.12	14.61	15.62
2.061	0.57	1.49	Q810B6	Rabankyrin-5	Ankfy1	2926	40	40	40	46.8	46.8	46.8	128.65	0.0000	323.31	113880000	849	17.38	17.01	17.34	17.28	16.82	16.58	16.95	16.39
2.203	0.57	1.49	Q91YI0	Argininosuccinate lyase	Asl	4158	32	32	32	64	64	64	51.739	0.0000	323.31	121490000	654	18.09	17.47	17.92	17.93	17.32	17.36	17.22	17.23
0.489	0.57	1.49	Q9D2E2	Target of EGR1 protein 1	Toe1	4941	3	3	3	11.7	11.7	11.7	56.87	0.0000	21.574	1965600	36	14.33	12.81	13.95	14.32	13.85	13.23	13.86	12.18
2.111	0.57	1.49	P28867;REV	Protein kinase C delta type;Protein kinase C delta type regulatory subur	Prkcd	964	51	51	49	67.8	67.8	66.5	77.546	0.0000	222.41	227380000	1018	18.40	18.03	18.40	18.41	17.76	17.69	18.02	17.48
0.841	0.57	1.49	P28574	Protein max	Max	952	3	3	3	26.9	26.9	26.9	18.245	0.0000	21.96	911030	26	14.55	13.02	14.26	13.68	13.26	13.40	13.28	13.29
1.520	0.57	1.49	A2AGH6;Q8	Mediator of RNA polymerase II transcription subunit 12	Med12	20	8	8	8	7.4	7.4	7.4	244.56	0.0000	16.744	3448300	56	14.15	14.03	14.25	14.00	13.09	14.04	13.49	13.53
2.006	0.57	1.49	Q60963	Platelet-activating factor acetylhydrolase	Pla2g7	2229	22	22	22	50.7	50.7	50.7	49.258	0.0000	203.42	109020000	570	18.16	18.21	18.30	18.29	17.76	17.98	17.68	17.26
0.748	0.57	1.49	Q3UGP9	Leucine-rich repeat-containing protein 58	Lrrc58	1962	6	6	6	24.6	24.6	24.6	40.139	0.0000	38.298	3417800	30	14.15	14.60	15.24	13.87	13.26	14.11	13.91	14.30
1.092	0.57	1.48	Q9BCZ4	Selenoprotein S	Vimp	4521	8	8	7	49.5	49.5	38.9	21.509	0.0000	83.492	12443000	108	15.89	15.23	15.54	15.39	15.28	14.56	15.41	14.53
2.728	0.57	1.48	Q8BML9		Qars	3144	46	46	45	57.3	57.3	57.2	87.676	0.0000	323.31	188870000	954	18.51	18.03	18.16	18.25	17.71	17.67	17.72	17.56
1.452	0.57	1.48	Q9DB73	NADH-cytochrome b5 reductase 1	Cyb5r1	5130	14	14	14	46.9	46.9	46.9	34.134	0.0000	104.03	33915000	256	16.98	16.51	16.86	16.86	16.18	15.74	16.41	16.60
1.020	0.57	1.48	Q8CCH2	NHL repeat-containing protein 3	Nhlrc3	3447	5	5	5	17.9	17.9	17.9	38.195	0.0000	20.841	4974100	51	14.55	15.61	14.58	14.69	13.87	14.37	14.46	14.44
2.144	0.57	1.48	O08756	3-hydroxyacyl-CoA dehydrogenase type-2	Hsd17b10	295	13	13	13	64.4	64.4	64.4	27.418	0.0000	323.31	124960000	574	18.64	18.28	18.51	18.77	17.78	18.23	18.04	17.88
1.724	0.57	1.48	Q99JR1	Sideroflexin-1	Sfxn1	4344	18	17	17	64.3	58.4	58.4	35.649	0.0000	323.31	91882000	550	17.76	17.35	18.07	17.94	17.13	17.02	17.32	17.37
0.673	0.57	1.48	P0DP28;P0DP27;P0DP26			716	7	7	5	80.5	80.5	70.5	16.837	0.0000	264.86	13203000	80	16.05	17.58	15.97	16.46	15.88	15.81	15.66	16.44
0.290	0.57	1.48	Q9DB90	Protein SMG9	Smg9	5133	5	5	5	14.4	14.4	14.4	57.62	0.0000	31.101	6426200	63	15.05	15.10	15.49	14.94	15.52	15.20	12.16	15.43
0.950	0.57	1.48	Q9QXT0	Protein canopy homolog 2	Cnpy2	5549	2	2	2	15.4	15.4	15.4	20.767	0.0000	10.156	1375000	15	14.23	14.51	13.85	14.25	13.51	12.92	14.01	14.12
0.797	0.57	1.48	O35459	Delta(3,5)-Delta(2,4)-dienoyl-CoA isomerase, mitochondrial	Ech1	372	11	11	11	45.9	45.9	45.9	36.118	0.0000	112.9	17467000	159	16.09	16.07	17.25	17.12	15.64	16.26	16.29	16.08
0.742	0.57	1.48	Q9WTK3	Glycosylphosphatidylinositol anchor attachment 1 protein	Gpaa1	5675	5	5	5	11	11	11	67.948	0.0000	27.356	12557000	85	15.80	15.83	16.16	15.81	15.47	14.27	15.92	15.66
1.335	0.57	1.48	O55201	Transcription elongation factor SPT5	Supt5h	463	39	39	39	42.6	42.6	42.6	120.66	0.0000	247.47	50889000	501	16.30	15.80	16.82	16.36	15.50	15.93	15.84	15.73
2.468	0.57	1.48	Q9WV32	Actin-related protein 2/3 complex subunit 1B	Arpc1b	5730	36	36	33	68.3	68.3	59.7	41.063	0.0000	323.31	771640000	1813	20.73	20.45	20.39	20.50	20.15	19.96	20.01	19.69
0.430	0.57	1.48	Q8C3X2	Coiled-coil domain-containing protein 90B, mitochondrial	Ccdc90b	3380	11	11	11	55.9	55.9	55.9	29.596	0.0000	53.888	10354000	86	15.11	15.48	14.19	14.71	15.18	12.81	14.58	14.65
1.975	0.57	1.48	Q9EQH2	Endoplasmic reticulum aminopeptidase 1	Erap1	5277	27	27	27	31.4	31.4	31.4	106.6	0.0000	323.31	89830000	690	17.59	17.32	17.39	17.57	17.17	16.68	17.12	16.64
0.985	0.57	1.48	Q3U1Y4	DENN domain-containing protein 4B	Dennd4b	1906	9	9	9	9.3	9.3	9.3	164.74	0.0000	25.709	7232800	86	14.98	14.09	14.85	14.21	13.83	13.58	14.50	13.96
1.272	0.57	1.48	P24604	Tyrosine-protein kinase Tec	Tec	900	7	7	7	13.2	13.2	13.2	73.425	0.0000	35.05	3048800	62	14.33	14.09	13.63	13.57	13.75	13.03	13.31	13.27
0.942	0.56	1.48	Q91XE8	Transmembrane protein 205	Tmem205	4145	4	4	4	21.2	21.2	21.2	21.18	0.0000	49.417	42392000	107	17.06	17.37	18.10	17.70	16.88	16.89	17.59	16.60
0.897	0.56	1.48	Q9CRC9	Glucosamine-6-phosphate isomerase 2	Gnpda2	4691	7	5	5	31.2	22.8	22.8	31.083	0.0000	13.501	3580000	39	14.84	13.72	14.86	14.38	13.59	13.61	14.06	14.28
0.487	0.56	1.48	Q9CR60	Vesicle transport protein GOT1B	Golt1b	4672	3	3	3	26.1	26.1	26.1	15.421	0.0000	18.1	7591600	25	15.29	16.13	16.55	15.44	13.98	15.79	15.74	15.64
0.467	0.56	1.48	Q8K2Y7	39S ribosomal protein L47, mitochondrial	Mrpl47	3678	8	8	8	32.5	32.5	32.5	29.725	0.0000	48.688	10017000	144	15.09	14.73	15.45	14.86	14.64	14.91	15.36	12.98
1.598	0.56	1.48	Q571E4	N-acetylgalactosamine-6-sulfatase	Galns	2090	17	17	17	38.3	38.3	38.3	57.673	0.0000	246.09	65092000	396	17.48	17.28	17.85	17.90	16.86	16.92	17.39	17.10
0.423	0.56	1.48	Q9Z0U1	Tight junction protein ZO-2	Tjp2	5787	8	8	8	11	11	11	131.28	0.0000	25.459	8379000	53	15.92	16.01	14.40	15.39	15.55	13.86	15.73	14.32
1.598	0.56	1.48	P47811	Mitogen-activated protein kinase 14	Mapk14	1126	21	21	21	80.6	80.6	80.6	41.287	0.0000	228.85	101210000	615	18.33	17.90	17.51	18.07	17.50	17.55	17.34	17.20
0.677	0.56	1.48	Q7TQK1	Integrator complex subunit 7	Ints7	2776	5	5	5	8.7	8.7	8.7	106.86	0.0000	15.233	3048800	21	13.78	14.10	14.19	12.59	13.09	13.43	12.69	13.21
0.690	0.56	1.47	Q91XL9	Oxysterol-binding protein-related protein 1	Osbpl1a	4148	9	9	9	11.2	11.2	11.2	107.79	0.0000	16.324	8626200	47	15.90	15.28	15.38	15.86	15.46	13.97	15.46	15.30
1.742	0.56	1.47	Q9WUP7	Ubiquitin carboxyl-terminal hydrolase isozyme L5	Uchl5	5719	17	17	17	63.8	63.8	63.8	37.616	0.0000	129.87	46220000	331	16.43	16.64	17.18	16.94	16.19	16.26	16.39	16.13
1.761	0.56	1.47	Q9JHG7	Phosphatidylinositol 4,5-bisphosphate 3-kinase catalytic subunit gamma	Pik3cg	5359	17	17	16	20.3	20.3	18.6	126.4	0.0000	109.84	20546000	194	16.08	15.71	16.43	16.23	15.44	15.64	15.73	15.40
1.397	0.56	1.47	Q9EPK7	Exportin-7	Xpo7	5256	17	17	17	20.3	20.3	20.3	123.81	0.0000	62.467	24672000	216	16.58	16.30	16.50	16.47	15.98	15.32	16.28	16.03
1.988	0.56	1.47	Q8BKC5;Q8	Importin-5	Ipo5	3098	49	49	49	60.2	60.2	60.2	123.59	0.0000	323.31	275480000	1470	19.13	18.74	19.10	19.04	18.35	18.17	18.75	18.52
1.528	0.56	1.47	Q8BFQ4	WD repeat-containing protein 82	Wdr82	2942	11	11	11	26.5	26.5	26.5	35.079	0.0000	146.93	29508000	247	17.26	17.21	17.75	17.43	16.92	17.26	16.69	16.56
1.581	0.56	1.47	Q3TXS7	26S proteasome non-ATPase regulatory subunit 1	Psmd1	1886	36	36	36	46.9	46.9	46.9	105.73	0.0000	323.31	95159000	834	17.02	16.65	16.44	17.14	16.45	15.99	16.36	16.22
0.545	0.56	1.47	Q9JLC8	Sacsin	Sacs	5477	7	7	7	1.4	1.4	1.4	520.68	0.0011	3.6222	9963500	30	15.42	15.53	16.71	15.62	15.09	14.32	15.59	16.04
1.723	0.56	1.47	Q8BFR4	N-acetylglucosamine-6-sulfatase	Gns	2944	23	23	23	45.6	45.6	45.6	61.174	0.0000	194.94	101670000	386	18.10	18.25	18.81	18.67	17.81	17.92	17.99	17.88
0.262	0.56	1.47	Q8K284	General transcription factor 3C polypeptide 1	Gtf3c1	3648	6	6	6	5.4	5.4	5.4	237.47	0.0000	50.483	2544200	29	12.51	13.46	15.74	15.26	12.97	13.11	13.81	14.85
2.493	0.56	1.47	Q4FZC9	Nesprin-3	Syne3	2058	18	18	18	28	28	28	112.03	0.0000	116.63	23153000	281	15.85	15.49	15.98	15.72	15.23	15.14	15.34	15.10
2.076	0.56	1.47	Q18PI6	SLAM family member 5	Cd84	1793	16	16	16	36.2	36.2	36.2	37.378	0.0000	159.71	31674000	236	17.41	17.04	17.25	16.89	16.61	16.44	16.83	16.48
1.415	0.55	1.47	Q8C7X2	ER membrane protein complex subunit 1	Emc1	3418	27	27	27	36.7	36.7	36.7	111.6	0.0000	191.45	45701000	411	16.75	16.04	16.33	16.25	15.88	15.47	15.66	16.16
2.761	0.55	1.47	O09131;Q8K	Glutathione S-transferase omega-1	Gsto1	327	29	29	29	74.2	74.2	74.2	27.497	0.0000	271	219320000	871	19.24	19.15	19.46	19.51	18.76	18.67	18.76	18.95
0.879	0.55	1.47	Q3U186	Probable arginine--tRNA ligase, mitochondrial	Rars2	1900	8	8	8	14.5	14.5	14.5	65.335	0.0000	20.358	6215300	86	14.51	14.16	14.98	14.66	13.59	13.55	14.36	14.61
2.923	0.55	1.47	Q9JLJ2	4-trimethylaminobutyraldehyde dehydrogenase	Aldh9a1	5483	40	40	40	76.7	76.7	76.7	53.514	0.0000	323.31	322850000	1317	18.88	18.69	19.10	18.75	18.33	18.28	18.37	18.22
0.714	0.55	1.47	P42232	Signal transducer and activator of transcription 5B	Stat5b	1078	16	5	5	24.3	9.3	9.3	90.001	0.0000	11.998	6144900	72	15.68	14.97	15.13	15.16	13.91	15.34	14.29	15.17
1.372	0.55	1.47	Q9CQ65	S-methyl-5-thioadenosine phosphorylase	Mtap	4567	16	16	16	58.7	58.7	58.7	31.062	0.0000	252.43	101420000	431	18.42	18.54	18.95	18.70	18.39	18.13	18.29	17.58
0.205	0.55	1.47	Q8BW94	Dynein heavy chain 3, axonemal	Dnah3	3255	5	5	5	1.4	1.4	1.4	467.77	0.0095	1.8173	8593200	26	15.35	12.17	15.69	15.59	15.96	14.27	13.23	13.16
1.675	0.55	1.46	Q9CQ22	Ragulator complex protein LAMTOR1	Lamtor1	4554	11	11	11	76.4	76.4	76.4	17.749	0.0000	277.03	77061000	372	17.87	17.56	18.31	18.01	17.42	17.39	17.58	17.17
2.125	0.55	1.46	Q3UBG2	PTB-containing, cubilin and LRP1-interacting protein	Pid1	1941	11	11	10	55.8	55.8	52.1	24.784	0.0000	55.522	15003000	112	16.12	15.97	15.58	15.64	15.22	15.41	15.31	15.18
0.394	0.55	1.46	Q9D6N5	Dr1-associated corepressor	Drap1	5005	5	5	5	20	20	20	22.278	0.0000	11.511	3835600	39	15.50	15.50	13.26	13.98	13.31	14.15	14.13	14.44
1.664	0.55	1.46	Q9JJF9	Signal peptide peptidase-like 2A	Sppl2a	5426	5	5	5	13.4	13.4	13.4	58.128	0.0000	68.384	9578100	73	16.18	15.78	15.80	16.15	15.81	15.48	15.18	15.24
1.631	0.55	1.46	Q99N95	39S ribosomal protein L3, mitochondrial	Mrpl3	4493	8	8	8	43.1	43.1	43.1	39.109	0.0000	39.939	10014000	129	15.31	15.01	15.58	15.09	14.48	14.58	15.07	14.67
1.036	0.55	1.46	Q5DU25	IQ motif and SEC7 domain-containing protein 2	Iqsec2	2098	9	5	4	7.2	4.5	3.7	161.78	0.0000	10.585	3583900	47	14.89	13.92	14.29	14.55	14.01	13.32	14.06	14.06
0.823	0.55	1.46	Q9WUK4	Replication factor C subunit 2	Rfc2	5713	11	11	11	54.7	54.7	54.7	38.724	0.0000	51.62	6468300	70	14.88	15.07	15.17	14.99	15.03	13.83	14.00	15.05
0.680	0.55	1.46	Q9CQ79	Thioredoxin domain-containing protein 9	Txndc9	4571	5	5	5	31.4	31.4	31.4	26.259	0.0000	58.879	2219800	47	14.35	14.08	13.85	14.50	12.76	13.75	13.56	14.52
1.553	0.55	1.46	Q8R5A3	Amyloid beta A4 precursor protein-binding family B member 1-interactin	Apbb1ip	3882	18	18	18	28.2	28.2	28.2	74.318	0.0000	261.52	97435000	538	18.44	18.21	18.48	18.32	17.95	17.73	18.22	17.36
2.233	0.55	1.46	Q9QZB7	Actin-related protein 10	Actr10	5589	17	17	17	52.5	52.5	52.5	46.207	0.0000	33.536	24839000	141	16.29	16.02	16.55	16.27	15.71	15.63	15.95	15.65
1.932	0.55	1.46	Q9CQF3	Cleavage and polyadenylation specificity factor subunit 5	Nudt21	4593	16	16	16	62.1	62.1	62.1	26.24	0.0000	173.97	49202000	294	17.41	17.17	16.87	17.36	16.71	16.68	16.40	16.83
1.841	0.55	1.46	Q8BYI6	Lysophosphatidylcholine acyltransferase 2	Lpcat2	3300	15	15	15	36.9	36.9	36.9	60.253	0.0000	100.88	45007000	265	17.12	16.78	17.06	17.21	16.85	16.21	16.46	16.46
1.762	0.55	1.46	P21958	Antigen peptide transporter 1	Tap1	870	17	17	17	34.5	34.5	34.5	78.863	0.0000	323.31	70294000	326	17.15	17.01	17.28	16.79	16.82	16.35	16.63	16.24
2.472	0.55	1.46	P40124;Q9C	Adenylyl cyclase-associated protein 1	Cap1	1056	63	63	63	89.9	89.9	89.9	51.564	0.0000	323.31	2910800000	4020	22.33	21.98	22.21	22.22	21.74	21.65	21.78	21.38
1.303	0.55	1.46	Q9D1D4	Transmembrane emp24 domain-containing protein 10	Tmed10	4907	19	19	19	46.6	46.6	46.6	24.911	0.0000	172.99	318740000	742	19.10	18.85	19.64	19.56	18.72	18.52	19.08	18.65
0.984	0.55	1.46	Q7TMK6	Protein Hook homolog 2	Hook2	2745	5	5	5	12.7	12.7	12.7	83.365	0.0000	32.74	3260400	26	13.83	13.66	14.57	13.94	13.84	13.00	13.75	13.22
1.325	0.55	1.46	P70388	DNA repair protein RAD50	Rad50	1563	27	27	27	25.8	25.8	25.8	153.49	0.0000	184.84	17765000	238	16.12	15.51	16.48	15.96	15.53	15.62	15.53	15.22
2.134	0.55	1.46	P26516	26S proteasome non-ATPase regulatory subunit 7	Psmd7	926	16	16	16	50.5	50.5	50.5	36.539	0.0000	285.93	63018000	353	18.36	18.09	17.90	18.31	17.57	17.42	17.64	17.85
1.914	0.55	1.46	P80318	T-complex protein 1 subunit gamma	Cct3	1595	46	46	46	74.3	74.3	74.3	60.629	0.0000	323.31	704220000	1952	20.15	19.87	20.25	20.23	19.65	19.74	19.73	19.21
1.225	0.54	1.46	P62996	Transformer-2 protein homolog beta	Tra2b	1476	11	11	10	38.5	38.5	35.1	33.665	0.0000	70.253	61353000	237	17.80	18.18	17.27	17.66	16.80	17.48	17.17	17.28
0.573	0.54	1.46	Q8C079	Striatin-interacting protein 1	Strip1	3327	6	6	6	12.7	12.7	12.7	95.584	0.0000	88.513	7492200	91	14.38	14.19	15.49	15.27	13.42	14.33	14.82	14.59
2.237	0.54	1.46	O09159	Lysosomal alpha-mannosidase	Man2b1	328	35	35	35	44.6	44.6	44.6	114.65	0.0000	323.31	264190000	982	18.81	18.65	19.02	18.90	18.35	18.19	18.58	18.10
0.540	0.54	1.46	Q6PAM1	Alpha-taxilin	Txlna	2597	18	18	17	41.3	41.3	39.9	62.368	0.0000	69.317	15806000	144	15.43	15.34	14.83	15.59	15.28	13.46	15.24	15.04
1.420	0.54	1.46	Q3UFY8	Mitochondrial ribonuclease P protein 1	Trmt10c	1959	2	2	2	9.7	9.7	9.7	48.385	0.0000	47.374	2941200	50	14.93	14.57	14.96	15.18	14.00	14.79	14.39	14.29
0.727	0.54	1.46	Q8BY71	Histone acetyltransferase type B catalytic subunit	Hat1	3293	7	7	7	23.8	23.8	23.8	49.278	0.0000	28.011	8465800	68	15.88	14.28	15.61	15.47	14.71	14.62	15.02	14.73
0.508	0.54	1.46	Q9Z172	Small ubiquitin-related modifier 3	Sumo3	5801	2	1	1	22.7	11.8	11.8	12.43	0.0027	2.6943	1690100	11	13.58	13.75	13.16	14.10	12.04	13.65	12.71	14.02
0.709	0.54	1.46	Q9D483	DNA-directed RNA polymerase III subunit RPC3	Polr3c	4972	4	4	4	13.5	13.5	13.5	60.705	0.0000	35.131	3153500	49	14.81	13.89	13.77	13.83	13.68	12.84	14.19	13.43
0.334	0.54	1.46	Q8BFT2	HAUS augmin-like complex subunit 4	Haus4	2947	4	4	3	20.4	20.4	14.6	42.196	0.0000	36.432	4237700	24	13.67	14.59	15.68	15.62	14.89	15.01	14.63	12.87
0.321	0.54	1.46	Q8BL86	Metallo-beta-lactamase domain-containing protein 2	Mblac2	3113	3	3	3	15.1	15.1	15.1	31.205	0.0000	12.083	7135700	26	15.71	15.98	17.02	15.72	15.69	13.71	16.45	16.42
0.442	0.54	1.46	Q8CI32	BAG family molecular chaperone regulator 5	Bag5	3538	4	4	4	13.9	13.9	13.9	50.942	0.0000	19.078	4581100	76	14.29	14.90	15.05	14.80	14.18	12.92	15.47	14.30
1.845	0.54	1.45	P63154	Crooked neck-like protein 1	Crnkl1	1492	16	16	16	31.2	31.2	31.2	83.415	0.0000	91.859	18545000	221	16.00	15.61	16.31	16.11	15.43	15.34	15.62	15.48
1.120	0.54	1.45	Q8BKT7	THO complex subunit 5 homolog	Thoc5	3106	10	10	10	23.6	23.6	23.6	78.685	0.0000	80.253	6657700	74	14.92	14.95	15.31	15.10	14.09	14.87	14.16	15.00

2.182	0.54	1.45	Q91V12	Cytosolic acyl coenzyme A thioester hydrolase	Acot7		4043	14	14	14	39.4	39.4	39.4	42.536	0.0000	83.054	73204000	310	17.19	17.16	17.48	17.35	16.86	16.80	16.92	16.43
1.587	0.54	1.45	Q61735	Leukocyte surface antigen CD47	Cd47		2329	6	6	6	13.9	13.9	13.9	33.097	0.0000	133.3	39526000	131	18.09	17.80	18.39	17.83	17.34	17.74	17.65	17.23
1.691	0.54	1.45	Q6GQT9	Nodal modulator 1	Nomo1		2514	32	32	32	35.9	35.9	35.9	133.42	0.0000	247.28	94310000	564	17.70	17.39	17.60	17.82	17.40	16.74	17.24	16.97
1.885	0.54	1.45	O08917	Flotillin-1	Flot1		312	19	19	19	52.8	52.8	52.8	47.513	0.0000	61.964	24446000	155	15.90	15.43	15.68	15.55	14.84	15.26	15.34	14.96
1.826	0.54	1.45	P43247	DNA mismatch repair protein Msh2	Msh2		1086	14	14	13	22.1	22.1	21.7	104.15	0.0000	87.573	11141000	110	15.79	15.43	15.40	15.74	15.21	15.20	15.10	14.69
1.425	0.54	1.45	P47758	Signal recognition particle receptor subunit beta	Srprb		1122	16	16	16	62.8	62.8	62.8	29.579	0.0000	218.49	55836000	391	17.06	16.68	17.50	17.28	16.29	16.75	16.63	16.70
2.060	0.54	1.45	Q8K1X1	WD repeat-containing protein 11	Wdr11		3635	29	29	29	33.8	33.8	33.8	135.94	0.0000	256.77	26999000	322	16.15	15.58	16.02	15.79	15.41	15.15	15.40	15.42
1.122	0.54	1.45	Q8VEM1	E3 ubiquitin-protein ligase RNF130	Rnf130		4014	3	3	3	12.9	12.9	12.9	46.375	0.0000	8.6206	1922700	20	13.12	14.11	13.94	13.54	13.44	13.23	12.93	12.96
0.983	0.54	1.45	Q9DB85	Ribosomal RNA-processing protein 8	Rrp8		5132	5	5	5	19.5	19.5	19.5	51.066	0.0000	19.385	3176400	39	14.29	14.16	14.57	14.49	13.99	13.12	14.38	13.87
1.252	0.54	1.45	O88796	Ribonuclease P protein subunit p30	Rpp30		566	8	8	8	36.2	36.2	36.2	29.473	0.0000	33.431	6045300	83	15.20	14.99	15.21	14.91	15.06	14.24	14.72	14.14
2.573	0.54	1.45	Q80UG5	Septin-9		Sep.09	2828	28	28	28	39.1	39.1	39.1	65.574	0.0000	323.31	108900000	709	17.88	17.66	18.08	17.77	17.26	17.31	17.48	17.19
2.363	0.54	1.45	P47753	F-actin-capping protein subunit alpha-1	Capza1		1119	24	19	19	83.9	73.8	73.8	32.939	0.0000	323.31	240370000	641	19.72	19.45	19.46	19.68	19.17	19.02	19.20	18.78
0.554	0.54	1.45	Q9WUZ9	Ectonucleoside triphosphate diphosphohydrolase 5	Entpd5		5726	4	4	4	14.8	14.8	14.8	47.101	0.0004	4.8901	2070600	15	14.37	13.51	14.36	14.56	12.58	13.72	14.36	13.99
0.436	0.54	1.45	P30677	Guanine nucleotide-binding protein subunit alpha-14	Gna14		979	8	4	3	25.1	14.1	8.2	41.527	0.0000	27.343	1636000	50	14.36	13.56	13.36	13.59	12.54	13.65	12.18	14.36
0.928	0.54	1.45	Q3URD3	Sarcolemmal membrane-associated protein	Slmap		2021	12	12	12	16.9	16.9	16.9	96.932	0.0000	39.099	3897000	72	14.42	13.54	14.57	14.38	13.19	13.75	14.03	13.79
0.483	0.54	1.45	Q7TMI3	E3 ubiquitin-protein ligase UHRF2	Uhrf2		2744	6	6	6	12.8	12.8	12.8	90.105	0.0000	29.682	8760700	55	15.27	15.59	15.83	15.84	15.67	15.35	15.71	13.66
0.282	0.54	1.45	Q8R502	Volume-regulated anion channel subunit LRRC8C	Lrrc8c		3878	4	4	4	8	8	8	92.371	0.0000	28.352	9688800	36	15.81	15.66	15.28	16.02	16.32	15.45	15.95	12.90
0.621	0.54	1.45	Q921V5	Alpha-1,6-mannosyl-glycoprotein 2-beta-N-acetylglucosaminyltransfera	Mgat2		4241	3	3	3	7.2	7.2	7.2	51.029	0.0000	9.7895	2400600	33	13.11	14.33	13.07	14.05	12.60	13.39	13.65	12.77
1.761	0.54	1.45	Q9CZ42	ATP-dependent (S)-NAD(P)H-hydrate dehydratase	Carkd		4817	12	12	12	54.2	54.2	54.2	36.717	0.0000	165.84	39397000	320	17.28	16.96	17.47	17.08	16.55	16.60	17.02	16.48
2.840	0.54	1.45	Q60973	Histone-binding protein RBBP7	Rbbp7		2232	11	11	4	29.4	29.4	19.1	47.789	0.0000	102.01	15592000	141	16.00	15.93	15.77	16.08	15.37	15.61	15.38	15.28
2.454	0.53	1.45	Q920Q4	Vacuolar protein sorting-associated protein 16 homolog	Vps16		4213	27	27	27	41	41	41	94.927	0.0000	212.92	59140000	446	17.17	16.79	17.15	17.09	16.49	16.57	16.68	16.33
1.493	0.53	1.45	Q9D1Q6	Endoplasmic reticulum resident protein 44	Erp44		4933	13	13	13	36.2	36.2	36.2	46.852	0.0000	150.11	24347000	254	16.18	16.19	16.00	16.10	15.75	16.03	15.26	15.29
1.138	0.53	1.45	Q9D1K7	UPF0687 protein C20orf27 homolog			4923	5	5	5	44.8	44.8	44.8	19.476	0.0000	12.407	6084200	52	14.62	15.41	15.51	15.37	14.49	14.99	14.43	14.85
1.119	0.53	1.45	Q9D6K5	Synaptojanin-2-binding protein	Synj2bp		4998	4	4	4	37.2	37.2	37.2	15.815	0.0000	36.644	7815900	106	14.99	14.71	14.81	15.38	14.77	13.85	14.52	14.60
0.735	0.53	1.45	Q9JIZ9	Phospholipid scramblase 3	Plscr3		5413	3	3	3	14.9	14.9	14.9	31.802	0.0000	130.7	3450100	39	14.62	14.85	14.62	14.86	14.66	14.94	13.53	13.70
3.698	0.53	1.45	Q9WU78	Programmed cell death 6-interacting protein	Pdcd6ip		5698	76	76	76	74.2	74.2	74.2	96.023	0.0000	323.31	534680000	2218	19.20	18.97	19.20	19.22	18.54	18.61	18.69	18.62
0.475	0.53	1.45	Q8K114	Integrator complex subunit 9	Ints9		3612	6	6	6	12.8	12.8	12.8	74.077	0.0000	13.897	2387100	21	14.89	13.21	13.34	12.87	12.89	13.22	12.48	13.59
1.538	0.53	1.45	Q4VC33	Macrophage erythroblast attacher	Maea		2073	15	15	15	45.5	45.5	45.5	45.336	0.0000	88.476	22347000	211	15.66	15.30	16.06	15.85	15.14	15.25	15.40	14.96
1.105	0.53	1.45	P59017	Bcl-2-like protein 13	Bcl2l13		1329	5	5	5	18.7	18.7	18.7	46.719	0.0000	20.637	12931000	93	16.02	15.58	16.55	16.60	15.66	15.58	15.84	15.54
1.532	0.53	1.45	Q80U28	MAP kinase-activating death domain protein	Madd		2816	14	14	14	13.8	13.8	13.8	175.18	0.0000	87.9	13352000	133	15.32	15.03	15.53	15.02	14.62	14.69	15.07	14.40
0.568	0.53	1.45	Q9CZN8	Glutamyl-tRNA(Gln) amidotransferase subunit A, mitochondrial	Qrsl1		4835	8	8	8	28.4	28.4	28.4	56.782	0.0000	130.89	7560500	123	14.26	14.50	15.37	14.85	14.68	14.31	14.71	13.15
2.723	0.53	1.44	Q61035	Histidine--tRNA ligase, cytoplasmic	Hars		2241	35	35	18	62.5	62.5	28.7	57.432	0.0000	323.31	139270000	784	17.88	17.53	17.71	17.97	17.20	17.25	17.31	17.21
2.276	0.53	1.44	Q9CSH3	Exosome complex exonuclease RRP44	Dis3		4698	22	22	22	35.3	35.3	35.3	108.84	0.0000	112.13	21888000	316	15.98	15.51	15.85	15.92	15.22	15.34	15.45	15.15
2.324	0.53	1.44	Q9WV54	Acid ceramidase;Acid ceramidase subunit alpha;Acid ceramidase subur	Asah1		5731	29	29	29	54.8	54.8	54.8	44.669	0.0000	200.45	265670000	803	18.59	18.32	18.26	18.34	18.08	17.63	17.90	17.78
0.666	0.53	1.44	Q9DBU0	Transmembrane 9 superfamily member 1	Tm9sf1		5178	2	2	2	5	5	5	68.927	0.0000	21.693	2095900	20	13.48	14.22	14.03	13.80	12.99	12.61	14.19	13.61
1.348	0.53	1.44	O08759	Ubiquitin-protein ligase E3A	Ube3a		296	15	15	15	25.7	25.7	25.7	99.818	0.0000	112.46	14874000	142	15.80	15.12	15.55	15.71	15.13	14.78	15.38	14.77
1.055	0.53	1.44	Q9CR09	Ubiquitin-fold modifier-conjugating enzyme 1	Ufc1		4656	10	10	10	56.9	56.9	56.9	19.481	0.0000	60.684	29344000	189	16.50	16.10	16.87	17.05	16.19	16.47	16.01	15.74
0.963	0.53	1.44	Q9JHQ5	Leucine zipper transcription factor-like protein 1	Lztfl1		5369	2	2	2	16.7	16.7	16.7	34.773	0.0000	39.453	1085200	15	13.40	14.09	13.45	13.45	12.52	12.97	13.16	13.63
1.417	0.53	1.44	Q9CS42	Ribose-phosphate pyrophosphokinase 2	Prps2		4697	15	7	7	52.8	29.2	29.2	34.786	0.0000	72.864	13058000	157	15.29	15.20	15.33	15.58	14.71	15.27	14.92	14.40
0.573	0.53	1.44	Q3UMW8	Ceroid-lipofuscinosis neuronal protein 5 homolog	Cln5		2005	4	4	4	7.9	7.9	7.9	39.329	0.0018	3.1927	2972900	24	14.24	14.15	15.38	14.83	13.77	13.85	13.79	15.10
0.549	0.53	1.44	Q80Y14	Glutaredoxin-related protein 5, mitochondrial	Glrx5		2893	3	3	3	29.6	29.6	29.6	16.292	0.0000	9.865	4464700	52	14.92	15.94	14.73	14.97	13.68	14.60	14.77	15.40
0.359	0.53	1.44	Q925Q3	Sodium/potassium/calcium exchanger 6, mitochondrial	Slc8b1		4311	2	2	2	3.1	3.1	3.1	64.364	0.0000	38.453	1391900	30	14.40	13.59	12.14	12.13	12.24	12.90	11.87	13.14
1.523	0.53	1.44	O88520	Leucine-rich repeat protein SHOC-2	Shoc2		538	7	7	7	18.9	18.9	18.9	64.892	0.0000	68.84	9360500	124	15.51	15.55	15.57	15.77	14.92	15.57	15.05	14.76
3.426	0.53	1.44	Q922B2	Aspartate--tRNA ligase, cytoplasmic	Dars		4247	41	41	41	78.6	78.6	78.6	57.147	0.0000	323.31	189520000	1104	18.36	18.25	18.51	18.30	17.93	17.71	17.88	17.80
2.455	0.53	1.44	Q91YW3	DnaJ homolog subfamily C member 3	Dnajc3		4182	21	21	21	47	47	47	57.463	0.0000	267.65	49862000	309	16.74	16.64	16.32	16.61	16.12	16.18	16.04	15.87
2.364	0.53	1.44	Q3THS6;Q9	S-adenosylmethionine synthase isoform type-2	Mat2a		1847	19	19	19	43.3	43.3	43.3	43.688	0.0000	107.64	69173000	374	17.25	17.14	17.61	17.32	16.68	16.97	16.81	16.76
0.903	0.53	1.44	Q9D110	5-formyltetrahydrofolate cyclo-ligase	Mthfs		4892	8	8	8	38.9	38.9	38.9	23.201	0.0000	21.148	7921500	67	14.60	14.12	14.67	14.90	14.24	14.62	13.84	13.49
3.135	0.53	1.44	Q9DCL9	Multifunctional protein ADE2;Phosphoribosylaminoimidazole-succinocar	Paics		5221	32	32	32	70.8	70.8	70.8	47.006	0.0000	208.46	153500000	782	18.47	18.21	18.15	18.41	17.82	17.85	17.73	17.75
1.744	0.53	1.44	O70325	Phospholipid hydroperoxide glutathione peroxidase, mitochondrial	Gpx4		489	14	14	14	62.4	62.4	62.4	22.228	0.0000	112.55	98872000	515	18.17	17.67	18.02	17.97	17.59	17.26	17.70	17.18
1.596	0.53	1.44	Q9WTR1	Transient receptor potential cation channel subfamily V member 2	Trpv2		5684	22	22	22	32.7	32.7	32.7	85.964	0.0000	252.58	82567000	525	17.21	16.94	17.61	17.31	16.84	16.41	16.86	16.86
1.890	0.53	1.44	Q9Z2C5	Myotubularin	Mtm1		5840	24	24	24	47.1	47.1	47.1	69.558	0.0000	211.67	27418000	277	16.49	15.97	16.25	16.19	15.55	15.71	15.99	15.55
3.604	0.52	1.44	Q9Z0N1	Eukaryotic translation initiation factor 2 subunit 3, X-linked	Eif2s3x		5778	34	34	11	64.4	64.4	23.5	51.065	0.0000	323.31	223310000	1055	18.94	18.73	18.79	18.82	18.32	18.15	18.38	18.34
0.858	0.52	1.44	P63082	V-type proton ATPase 16 kDa proteolipid subunit	Atp6v0c		1487	3	3	3	34.2	34.2	34.2	15.808	0.0000	215.98	223320000	437	19.87	19.28	20.22	19.41	19.56	18.54	19.34	19.24
1.366	0.52	1.44	Q80SW1	Putative adenosylhomocysteinase 2	Ahcyl1		2795	23	14	13	43.8	27.9	27.9	58.951	0.0000	101.45	19847000	107	17.06	16.87	16.75	16.87	16.29	16.60	16.72	15.85
1.089	0.52	1.44	P60605	Ubiquitin-conjugating enzyme E2 G2	Ube2g2		1356	6	6	6	53.3	53.3	53.3	18.566	0.0000	70.447	14662000	106	16.28	17.26	16.41	16.63	16.22	16.20	16.31	15.75
2.094	0.52	1.44	O70310	Glycylpeptide N-tetradecanoyltransferase 1	Nmt1		486	27	27	23	46.6	46.6	42.7	56.888	0.0000	323.31	90814000	558	17.80	17.30	17.67	17.84	17.12	17.14	17.26	17.00
2.308	0.52	1.44	P11983	T-complex protein 1 subunit alpha	Tcp1		753	50	50	50	88.8	88.8	88.8	60.448	0.0000	323.31	734380000	2228	20.53	20.42	20.76	20.82	20.12	19.98	20.31	20.02
0.839	0.52	1.44	Q923W1	Trimethylguanosine synthase	Tgs1		4288	2	2	2	3.5	3.5	3.5	96.789	0.0004	4.435	767590	8	13.36	13.43	13.57	13.36	12.91	12.62	12.33	13.76
2.271	0.52	1.44	Q9Z1K5	E3 ubiquitin-protein ligase ARIH1	Arih1		5810	17	17	17	36.8	36.8	36.8	64.016	0.0000	61.274	26100000	202	16.23	16.01	16.24	15.76	15.65	15.56	15.43	15.50
1.885	0.52	1.44	Q921S7	39S ribosomal protein L37, mitochondrial	Mrpl37		4239	16	16	16	45.9	45.9	45.9	48.34	0.0000	63.049	16108000	134	15.39	14.81	15.32	15.30	14.71	14.54	14.86	14.61
1.684	0.52	1.44	Q8R4N0	Citrate lyase subunit beta-like protein, mitochondrial	Clybl		3871	12	12	12	43.5	43.5	43.5	37.548	0.0000	52.248	7068300	101	15.23	14.76	14.78	15.21	14.63	14.62	14.17	14.48
0.468	0.52	1.44	Q9D0L4	Uncharacterized aarF domain-containing protein kinase 1	Adck1		4874	5	5	5	15.4	15.4	15.4	59.735	0.0000	20.503	4915400	74	14.95	14.34	13.84	14.61	12.66	13.87	14.57	14.55
0.617	0.52	1.44	Q99PP7	E3 ubiquitin-protein ligase TRIM33	Trim33		4512	3	3	3	4	4	4	123.84	0.0000	9.0553	1855900	29	14.39	13.45	15.07	14.22	13.65	14.34	13.24	13.81
1.698	0.52	1.44	Q99N69	Leupaxin	Lpxn		4484	22	22	22	55.4	55.4	55.4	43.478	0.0000	323.31	139590000	674	18.38	18.00	18.11	18.08	17.95	17.25	17.69	17.59
2.311	0.52	1.44	O09061	Proteasome subunit beta type-1	Psmb1		321	16	16	15	73.8	73.8	62.1	26.372	0.0000	323.31	222310000	578	19.68	19.42	19.72	19.47	19.24	18.94	19.18	18.85
2.024	0.52	1.44	P12382	ATP-dependent 6-phosphofructokinase, liver type	Pfkl		757	38	35	32	58.8	56.5	54.4	85.359	0.0000	323.31	146090000	887	18.04	17.59	17.46	17.74	17.23	17.01	17.29	17.21
1.691	0.52	1.44	Q9CZW5	Mitochondrial import receptor subunit TOM70	Tomm70a		4843	19	19	19	36.2	36.2	36.2	67.589	0.0000	212.27	29897000	330	16.01	15.86	16.33	16.23	15.45	15.39	15.96	15.55
1.390	0.52	1.44	Q8VE97	Serine/arginine-rich splicing factor 4	Srsf4		3995	10	7	7	18.8	15.5	15.5	55.979	0.0000	18.646	7867700	68	15.55	15.47	15.40	14.97	14.52	15.18	14.98	14.62
0.957	0.52	1.44	Q8VEM8	Phosphate carrier protein, mitochondrial	Slc25a3		4015	22	22	22	54.1	54.1	54.1	39.632	0.0000	261.91	490090000	736	20.70	20.28	19.97	19.58	19.79	19.22	19.59	19.86
0.926	0.52	1.43	Q6P9R1	ATP-dependent RNA helicase DDX51	Ddx51		2591	5	5	5	12.8	12.8	12.8	70.367	0.0000	19.432	2904900	47	13.92	13.33	14.24	13.92	12.87	13.57	13.81	13.09
1.060	0.52	1.43	A2AWA9	Rab GTPase-activating protein 1	Rabgap1		42	16	16	15	18.4	18.4	17.6	120.8	0.0000	98.549	15638000	158	14.96	14.82	15.50	15.58	14.56	14.28	14.99	14.94
1.717	0.52	1.43	Q8CIH5;Q9(	1-phosphatidylinositol 4,5-bisphosphate phosphodiesterase gamma-2	Plcg2		3555	81	81	80	61.7	61.7	61.7	147.59	0.0000	323.31	365540000	1784	18.42	17.95	18.45	18.39	17.95	17.84	17.89	17.45
1.990	0.52	1.43	Q8C6G8	WD repeat-containing protein 26	Wdr26		3405	15	15	15	37.3	37.3	37.3	70.543	0.0000	85.981	26713000	188	16.63	16.34	16.36	15.99	15.79	15.68	15.92	15.85
1.711	0.52	1.43	Q8C878	NEDD8-activating enzyme E1 catalytic subunit	Uba3		3422	16	16	16	51.3	51.3	51.3	51.737	0.0000	268.14	48214000	355	17.57	17.45	17.93	18.00	17.18	17.06	17.50	17.14
0.959	0.52	1.43	Q9JK23	Proteasome assembly chaperone 1	Psmg1		5442	12	12	12	43.6	43.6	43.6	33.104	0.0000	51.135	19620000	166	15.39	15.57	15.75	15.73	15.24	15.27	15.52	14.32
0.177	0.52	1.43	A2BDX3	Adenylyltransferase and sulfurtransferase MOCS3;Molybdopterin-synth	Mocs3		44	6	6	5	22.4	22.4	17.2	49.374	0.0000	14.235	6951800	48	16.30	15.78	14.35	15.35	16.55	16.96	12.99	13.19
1.308	0.52	1.43	Q9DCT2	NADH dehydrogenase [ubiquinone] iron-sulfur protein 3, mitochondrial	Ndufs3		5230	12	12	12	38.8	38.8	38.8	30.149	0.0000	40.965	30188000	147	16.99	17.15	16.82	16.92	16.66	16.66	16.64	15.86
1.650	0.52	1.43	Q9CXT8	Mitochondrial-processing peptidase subunit beta	Pmpcb		4766	19	18	18	50.1	48.7	48.7	54.614	0.0000	195.45	52843000	331	16.86	16.39	17.16	16.74	16.19	16.41	16.31	16.17
3.272	0.52	1.43	Q8K1T1	Leucine-rich repeat-containing protein 25	Lrrc25		3634	7	7	7	32.7	32.7	32.7	32.673	0.0000	151.29	28228000	249	16.22	16.07	16.03	16.04	15.60	15.74	15.50	15.44
1.807	0.52	1.43	Q922E4	Ethanolamine-phosphate cytidylyltransferase	Pcyt2		4252	8	8	8	23.8	23.8	23.8	45.234	0.0000	27.084	8394800	65	15.57	15.43	15.71	15.15	15.07	15.14	14.87	14.71
2.599	0.52	1.43	Q8CJG0	Protein argonaute-2	Ago2		3571	20	20	14	29.4	29.4	20.3	97.303	0.0000	54.857	30161000	301	16.25	15.83	16.10	16.12	15.52	15.46	15.72	15.54
3.193	0.52	1.43	P57780	Alpha-actinin-4	Actn4		1300	94	94	67	86.2	86.2	67.9	104.98	0.0000	323.31	715250000	2744	19.50	19.35	19.63	19.59	18.91	18.97	19.15	19.00
2.014	0.52	1.43	Q91V61	Sideroflexin-3	Sfxn3		4047	24	24	23	71.7	71.7	65.7	35.406	0.0000	323.31	151060000	794	18.20	17.85	18.44	18.40	17.65	17.68	17.78	17.71
1.166	0.52	1.43	Q9D824	Pre-mRNA 3-end-processing factor FIP1	Fip1l1		5048	13	13	13	29.6	29.6	29.6	64.958	0.0000	86.179	9022700	69	15.41	15.69	15.05	15.32	14.81	14.64	14.56	15.40
1.446	0.51	1.43	Q6P5E6	ADP-ribosylation factor-binding protein GGA2	Gga2		2575	11	10	10	33.8	32.7	32.7	66.048	0.0000	187.55	20227000	183	16.24	16.04	16.61	16.89	15.87	15.97	15.95	15.92
0.806	0.51	1.43	Q8BGH7	CDC42 small effector protein 2	Cdc42se2		2984	3	3	3	69	69	69	9.2234	0.0000	232.65	32790000	152	17.71	17.50	18.18	17.94	16.73	17.72	17.87	16.95
0.876	0.51	1.43	Q8BZA9	Fructose-2,6-bisphosphatase TIGAR	Tigar		3312	7	7	7	45.4	45.4	45.4	29.19	0.0000	124.85	21813000	117	16.28	16.36	17.10	16.62	16.07	15.43	16.48	16.32
2.664	0.51	1.43	Q9D662	Protein transport protein Sec23B	Sec23b		4990	38	38	35	64.8	64.8	61.1	86.436	0.0000	323.31	162520000	948	18.06	17.85	17.98	18.09	17.39	17.30	17.67	17.58
1.146	0.51	1.43	P10810	Monocyte differentiation antigen CD14	Cd14		729	14	14	14	54.1	54.1	54.1	39.203	0.0000	230.25	80178000	381	18.60	18.40	18.26	18.35	18.13	18.28	17.26	17.89
2.083	0.51	1.43	Q3V3R1	Monofunctional C1-tetrahydrofolate synthase, mitochondrial	Mthfd1l		2054	36	35	35	47.2	46.2	46.2	105.73	0.0000	323.31	66153000	600	16.67	16.32	16.84	16.75	16.09	16.29	16.16	15.98
1.175	0.51	1.43	Q9ET30	Transmembrane 9 superfamily member 3	Tm9sf3		5356	9	9	9	18.6	18.6	18.6	67.544	0.0000	73.059	29737000	177	16.60	15.77	16.73	16.68	15.84	15.98	15.91	16.00
1.424	0.51	1.43	Q3UVK0	Endoplasmic reticulum metallopeptidase 1	Ermp1		2038	11	11	11	14.6	14.6	14.6	100.15	0.0000	41.968	16528000	136	15.86	15.16	16.02	15.55	15.18	15.03	15.13	15.20
1.422	0.51	1.43	O35638	Cohesin subunit SA-2	Stag2		389	20	20	17	22.2	22.2	19.5	141.28	0.0000	90.742	16069000	174	15.77	15.08	15.68	15.83	15.15	15.28	14.97	14.91
1.777	0.51	1.43	P70303	CTP synthase 2	Ctps2		1551	24	24	22	50.3	50.3	46.8	65.514	0.0000	79.629	55084000	436	16.77	16.60	17.10	16.99	16.24	16.18	16.67	16.32
1.122	0.51	1.43	Q8VDV3	Guanine nucleotide exchange factor for Rab-3A	Rab3il1		3973	20	20	20	59.5	59.5	59.5	42.712	0.0000	258.44	27467000	317	16.05	15.69	16.62	16.34	15.87	15.59	15.88	15.33
1.570	0.51	1.43	Q9D287	Pre-mRNA-splicing factor SPF27	Bcas2		4938	4	4	4	30.7	30.7	30.7	26.131	0.0000	98.838	13459000	146	16.22	16.33	15.66	16.42	15.69	15.64	15.74	15.53
2.677	0.51	1.42	P53395	Lipoamide acyltransferase component of branched-chain alpha-keto aci	Dbt		1231	16	16	16	33.4	33.4	33.4	53.246	0.0000	64.447	24800000	223	16.13	15.67	15.96	15.89	15.42	15.34	15.49	15.37
0.392	0.51	1.42	Q9D8B4	NADH dehydrogenase [ubiquinone] 1 alpha subcomplex subunit 11	Ndufa11		5058	3	3	3	27	27	27	14.982	0.0000	12.862	2122600	11	14.21	13.76	14.15	13.23	12.52	12.42	14.60	13.77
2.063	0.51	1.42	P62334	26S protease regulatory subunit 10B	Psmc6		1427	37	37	36	83.8	83.8	83.8	44.172	0.0000	323.31	242910000	1172	18.09	18.00	18.40	18.27	17.80	17.77	17.77	17.39
2.101	0.51	1.42	Q80U58	Pumilio homolog 2	Pum2		2820	10	6	6	9.8	5.7	5.7	114.31	0.0000	31.795	7364100	87	14.96	14.82	14.75	15.21	14.38	14.23	14.47	14.62
1.941	0.51	1.42	F6ZDS4	Nucleoprotein TPR	Tpr		257	50	50	50	27.8	27.8	27.8	273.99	0.0000	310.82	40299000	557	16.18	15.73	16.31	16.18	15.44	15.74	15.61	15.58
0.296	0.51	1.42	Q9DBS2	Tumor protein p63-regulated gene 1-like protein	Tprg1l		5174	4	4	4	36.1	36.1	36.1	29.814	0.0025	2.8776	455270	4	12.31	12.51	14.98	12.93	12.13	13.28	13.38	11.91
1.849	0.51	1.42	Q8BFZ3	Beta-actin-like protein 2	Actbl2		2954	21	5	5	46.8	18.1	18.1	42.004	0.0000	52.736	51789000	290	17.52	17.20	17.64	17.50	16.83	17.31	16.87	16.81
1.540	0.51	1.42	Q80UJ7	Rab3 GTPase-activating protein catalytic subunit	Rab3gap1		2829	28	28	28	35.7	35.7	35.7	110.2	0.0000	190.73	51588000	436	17.44	16.87	17.64	17.53	16.90	16.87	16.94	16.73
2.240	0.51	1.42	Q9Z0S1	3(2),5-bisphosphate nucleotidase 1	Bpnt1		5784	19	19	19	64.6	64.6	64.6	33.196	0.0000	177.76	76999000	597	17.54	17.12	17.41	17.56	16.89	16.82	17.09	16.80
2.367	0.51	1.42	Q3U7R1	Extended synaptotagmin-1	Esyt1		1932	79	79	79	69.3	69.3	69.3	121.55	0.0000	323.31	751930000	2376	19.19	19.00	19.38	19.21	18.70	18.48	18.89	18.67
1.568	0.51	1.42	Q8BG32	26S proteasome non-ATPase regulatory subunit 11	Psmd11		2962	34	34	34	78.7	78.7	78.7	47.436	0.0000	309.94	190220000	690	18.95	18.64	19.27	19.10	18.37	18.82	18.39	18.36
1.147	0.51	1.42	Q8VDP3	Protein-methionine sulfoxide oxidase MICAL1	Mical1		3964	30	30	29	36.3	36.3	33	116.78	0.0000	145.02	47583000	489	16.45	15.89	16.75	16.34	15.72	15.58	16.26	15.85
0.604	0.51	1.42	Q8CBA2	Schlafen family member 5	Slfn5		3435	30	30	30	47.1	47.1	47.1	100.82	0.0000	323.31	66655000	425	17.95	17.40	16.77	16.52	17.24	16.70	16.56	16.10
0.196	0.51	1.42	Q924D0	Reticulon-4-interacting protein 1, mitochondrial	Rtn4ip1		4292	6	6	6	23.5	23.5	23.5	43.371	0.0000	19.67	4650100	35	15.35	12.59	15.83	14.16	14.35	14.82	14.88	11.85
2.367	0.51	1.42	O70145	Neutrophil cytosol factor 2	Ncf2		471	36	36	36	62.1	62.1	62.1	59.485	0.0000	323.31	250590000	1133	18.84	18.59	18.45	18.56	18.21	18.13	18.19	17.88
1.908	0.51	1.42	Q8BJS4	SUN domain-containing protein 2	Sun2		3084	40	40	39	63.9	63.9	62.8	81.604	0.0000	323.31	85062000	740	17.02	16.63	17.09	17.16	16.31	16.56	16.65	16.35
1.518	0.51	1.42	O35345	Importin subunit alpha-7	Kpna6		360	15	15	11	40.5	40.5	33.2	59.964	0.0000	149.04	29823000	314	16.62	16.31	16.18	16.40	15.96	15.43	15.93	16.16
1.798	0.51	1.42	P35585;Q9V	AP-1 complex subunit mu-1	Ap1m1		1027	21	21	21	55.1	55.1	55.1	48.542	0.0000	162.01	71593000	410	16.87	16.84	17.32	17.20	16.64	16.36	16.77	16.44

0.567	0.51	1.42	Q3U821		Wdr75	1934	4	4	4	7.2	7.2	7.2	94.036	0.0000	53.898	3726400	42	13.81	14.11	15.57	14.75	14.01	14.27	14.30	13.64
1.409	0.51	1.42	Q6PA06	Atlastin-2	Atl2	2593	12	12	10	24.5	24.5	21.1	66.223	0.0000	63.162	14806000	155	15.92	15.40	16.11	15.52	15.00	15.18	15.44	15.31
0.902	0.51	1.42	O88986	2-amino-3-ketobutyrate coenzyme A ligase, mitochondrial	Gcat	592	15	15	15	42.3	42.3	42.3	44.93	0.0000	196.99	38441000	292	16.68	16.21	16.56	16.69	16.32	16.50	16.00	15.31
2.541	0.51	1.42	O54941	SWI/SNF-related matrix-associated actin-dependent regulator of chroma	Smarce1	435	9	9	9	29	29	29	46.638	0.0000	70.772	14119000	150	16.43	16.44	16.31	16.59	15.87	15.92	15.78	16.19
1.511	0.51	1.42	O88842	FYVE, RhoGEF and PH domain-containing protein 3	Fgd3	572	29	29	28	52.4	52.4	50.9	80.623	0.0000	323.31	76599000	584	17.44	17.02	17.59	17.47	17.02	17.09	16.87	16.52
0.677	0.50	1.42	Q8R2R9	AP-3 complex subunit mu-2	Ap3m2	3817	7	4	4	24.2	14.6	14.6	46.916	0.0000	12.978	2942500	39	14.09	13.93	15.06	15.26	14.01	13.89	14.46	13.97
2.034	0.50	1.42	Q64704	Syntaxin-3	Stx3	2459	14	14	14	41.5	41.5	41.5	33.243	0.0000	236.06	26282000	191	16.58	16.38	16.88	16.86	16.10	16.14	16.34	16.11
1.075	0.50	1.42	P55772	Ectonucleoside triphosphate diphosphohydrolase 1	Entpd1	1273	10	10	10	21.8	21.8	21.8	57.205	0.0000	119.19	24796000	138	16.70	16.35	17.22	17.12	16.08	16.20	16.70	16.40
2.512	0.50	1.42	Q8K3H0	DCC-interacting protein 13-alpha	Appl1	3702	20	19	19	41.6	40.6	40.6	79.327	0.0000	104.42	21893000	205	16.20	15.90	16.28	16.09	15.42	15.69	15.64	15.70
1.725	0.50	1.42	Q9CZY3	Ubiquitin-conjugating enzyme E2 variant 1	Ube2v1	4848	12	12	6	82.3	82.3	49	16.355	0.0000	201.03	131530000	351	19.12	18.84	19.31	19.29	18.53	18.36	18.85	18.80
1.678	0.50	1.42	Q8VDL4	ADP-dependent glucokinase	Adpgk	3958	11	11	11	32.1	32.1	32.1	53.902	0.0000	75.699	22349000	163	16.30	15.85	16.39	16.21	15.79	15.72	15.87	15.36
1.106	0.50	1.42	Q8BH65	Protein DENND6A	Dennd6a	3018	8	8	8	20.2	20.2	20.2	68.966	0.0000	10.98	8155000	43	16.00	15.66	16.31	15.99	15.40	15.74	15.85	14.97
0.730	0.50	1.42	Q9CQE7	Endoplasmic reticulum-Golgi intermediate compartment protein 3	Ergic3	4590	6	6	6	17	17	17	43.208	0.0000	21.273	4819900	56	14.79	14.83	14.34	14.35	14.35	13.96	14.71	13.27
2.192	0.50	1.42	Q9CY64	Biliverdin reductase A	Blvra	4788	30	30	30	80.7	80.7	80.7	33.524	0.0000	323.31	270970000	913	19.47	19.24	19.75	19.30	18.92	18.98	19.02	18.82
1.805	0.50	1.42	Q3U308	Cytoplasmic tRNA 2-thiolation protein 2	Ctu2	1915	5	5	5	16.5	16.5	16.5	56.104	0.0000	36.117	14172000	73	16.76	16.46	16.96	16.80	16.33	15.92	16.43	16.29
1.967	0.50	1.42	P26443;P00	Glutamate dehydrogenase 1, mitochondrial	Glud1	924	56	56	56	73.5	73.5	73.5	61.336	0.0000	323.31	1083900000	2443	20.28	20.03	20.46	20.30	19.76	19.89	19.93	19.47
3.018	0.50	1.42	Q63932	Dual specificity mitogen-activated protein kinase kinase 2	Map2k2	2408	17	9	9	30.9	16	16	44.402	0.0000	45.56	19320000	206	16.10	16.04	16.15	16.37	15.63	15.70	15.75	15.57
1.740	0.50	1.42	Q59J78	Mimitin, mitochondrial	Ndufaf2	2094	7	7	7	51.8	51.8	51.8	19.628	0.0000	94.014	9179900	118	15.47	15.56	15.43	15.51	15.03	14.55	15.22	15.16
1.549	0.50	1.42	Q9JJL8	Serine--tRNA ligase, mitochondrial	Sars2	5431	10	10	10	25.7	25.7	25.7	58.316	0.0000	133.11	7633200	98	14.80	14.45	15.22	15.09	14.41	14.50	14.35	14.30
0.432	0.50	1.42	Q8CFI5	Probable proline--tRNA ligase, mitochondrial	Pars2	3488	3	3	3	9.3	9.3	9.3	53.517	0.0004	4.5254	2063000	17	13.99	13.47	14.91	13.08	13.76	13.34	13.92	12.43
1.958	0.50	1.42	Q9EPJ9	ADP-ribosylation factor GTPase-activating protein 1	Arfgap1	5253	13	13	13	51	51	51	45.288	0.0000	131.12	25086000	214	16.31	16.23	16.75	16.61	15.92	15.81	16.07	16.09
3.160	0.50	1.42	P68510	14-3-3 protein eta	Ywhah	1522	31	25	25	84.1	81.3	81.3	28.211	0.0000	323.31	560210000	938	20.53	20.32	20.33	20.46	20.01	19.91	19.97	19.74
2.262	0.50	1.41	Q91YP3	Deoxyribose-phosphate aldolase	Dera	4171	12	12	12	52.8	52.8	52.8	34.975	0.0000	155.04	29859000	241	17.01	16.70	17.15	16.98	16.60	16.35	16.56	16.33
3.277	0.50	1.41	Q8BJ71	Nuclear pore complex protein Nup93	Nup93	3075	32	32	32	52.7	52.7	52.7	93.28	0.0000	323.31	67897000	528	17.62	17.34	17.55	17.67	17.05	17.01	17.07	17.04
2.518	0.50	1.41	Q9QVP9	Protein-tyrosine kinase 2-beta	Ptk2b	5524	53	53	52	61.3	61.3	60.6	115.79	0.0000	323.31	162910000	1227	17.80	17.53	17.93	17.80	17.30	17.19	17.42	17.15
1.948	0.50	1.41	Q9Z1F9	SUMO-activating enzyme subunit 2	Uba2	5806	29	29	29	59.9	59.9	59.9	70.568	0.0000	208.38	55257000	526	16.55	16.27	16.47	16.19	15.93	16.15	15.74	15.66
2.293	0.50	1.41	Q9QZE5	Coatomer subunit gamma-1	Copg1	5595	38	38	35	61.3	61.3	57.2	97.512	0.0000	323.31	198690000	1116	18.58	18.29	18.55	18.67	18.15	17.82	18.17	17.96
1.610	0.50	1.41	P16330	2,3-cyclic-nucleotide 3-phosphodiesterase	Cnp	808	13	13	13	37.6	37.6	37.6	47.123	0.0000	94.216	17881000	198	15.73	15.31	15.69	15.51	15.21	15.26	15.10	14.66
1.431	0.50	1.41	O88543	COP9 signalosome complex subunit 3	Cops3	543	16	16	16	51.1	51.1	51.1	47.832	0.0000	323.31	36557000	303	17.05	16.57	17.29	16.91	16.26	16.75	16.51	16.32
1.466	0.50	1.41	Q8C0J2	Autophagy-related protein 16-1	Atg16l1	3338	15	15	15	32.8	32.8	32.8	68.171	0.0000	119.44	10250000	135	15.41	14.80	15.42	15.25	14.59	14.48	14.89	14.92
2.204	0.50	1.41	Q9Z1Z2	Serine-threonine kinase receptor-associated protein	Strap	5827	21	21	21	66.6	66.6	66.6	38.442	0.0000	323.31	119450000	574	17.85	17.72	18.07	18.18	17.46	17.51	17.57	17.28
0.475	0.50	1.41	Q9CQQ8	U6 snRNA-associated Sm-like protein LSm7	Lsm7	4623	1	1	1	25.2	25.2	25.2	11.636	0.0002	5.882	1881000	39	13.96	13.40	13.34	14.43	13.30	14.41	12.70	12.73
2.102	0.50	1.41	P62869	Transcription elongation factor B polypeptide 2	Tceb2	1458	11	11	11	68.6	68.6	68.6	13.17	0.0000	67.803	35513000	192	17.72	17.49	17.17	17.66	17.03	16.95	16.96	17.10
0.634	0.50	1.41	Q8CDA1	Phosphatidylinositide phosphatase SAC2	Inpp5f	3458	15	15	15	19.1	19.1	19.1	127.61	0.0000	48.151	6188100	67	14.59	14.18	15.10	14.74	13.34	13.93	14.65	14.70
0.869	0.50	1.41	Q5XG71	Small subunit processome component 20 homolog	Utp20	2170	12	12	12	6.3	6.3	6.3	317.74	0.0000	17.792	14107000	72	16.56	16.47	17.18	16.60	15.89	15.82	16.23	16.86
0.791	0.50	1.41	Q80YV4	Pantothenate kinase 4	Pank4	2909	11	11	11	13.9	13.9	13.9	91.521	0.0000	17.752	7061700	51	15.62	14.15	15.10	15.16	14.55	14.42	14.51	14.57
1.314	0.50	1.41	Q9Z1G4	V-type proton ATPase 116 kDa subunit a isoform 1	Atp6v0a1	5808	26	26	25	27.8	27.8	26.9	96.466	0.0000	180.32	101430000	530	17.60	16.90	17.70	17.30	16.62	16.96	17.05	16.90
0.775	0.50	1.41	Q8VE62	Polyadenylate-binding protein-interacting protein 1	Paip1	3987	4	4	3	14	14	10.2	45.701	0.0000	18.054	3515800	32	14.03	14.12	14.20	14.20	12.71	13.81	14.00	14.04
0.548	0.50	1.41	O70131	Ninjurin-1	Ninj1	469	2	2	1	31.6	31.6	13.2	16.555	0.0000	98.18	8916400	84	16.77	16.15	15.84	16.25	16.18	14.97	15.31	16.57
2.299	0.50	1.41	P28271	Cytoplasmic aconitate hydratase	Aco1	947	58	58	57	71.9	71.9	71	98.124	0.0000	323.31	178200000	1129	18.03	17.54	18.01	17.87	17.41	17.38	17.39	17.29
1.506	0.49	1.41	Q80W54	CAAX prenyl protease 1 homolog	Zmpste24	2859	11	11	11	27.4	27.4	27.4	54.734	0.0000	158.58	50531000	288	17.09	16.83	16.55	16.82	16.58	16.00	16.54	16.20
3.254	0.49	1.41	Q9CQV8	14-3-3 protein beta/alpha;14-3-3 protein beta/alpha, N-terminally proces	Ywhab	4641	34	25	24	85.4	81.7	78.9	28.086	0.0000	323.31	366190000	897	20.02	19.82	20.04	19.95	19.43	19.55	19.55	19.32
0.833	0.49	1.41	Q3UE37	Ubiquitin-conjugating enzyme E2 Z	Ube2z	1953	6	6	6	24.7	24.7	24.7	38.368	0.0000	38.193	25067000	93	16.41	16.84	17.34	16.68	16.46	16.09	16.88	15.87
1.168	0.49	1.41	Q8BRG8	Transmembrane protein 209	Tmem209	3180	13	13	12	31.6	31.6	31.6	62.981	0.0000	55.374	11090000	111	15.60	15.50	16.27	15.31	15.15	15.15	15.39	15.01
0.364	0.49	1.41	O70589	Peripheral plasma membrane protein CASK	Cask	516	5	5	5	10.7	10.7	10.7	105.11	0.0000	14.373	3805200	39	15.16	15.21	14.11	14.72	14.41	13.28	15.73	13.81
1.308	0.49	1.41	Q9QXK3	Coatomer subunit gamma-2	Copg2	5543	34	31	31	48.7	46.5	46.5	97.679	0.0000	323.31	84500000	522	17.99	17.50	17.72	17.80	17.43	16.78	17.58	17.24
0.577	0.49	1.41	Q9DBA9	General transcription factor IIH subunit 1	Gtf2h1	5137	2	2	2	5.5	5.5	5.5	61.851	0.0000	10.979	1235100	35	14.02	13.36	13.32	13.20	13.05	13.82	12.08	12.98
0.968	0.49	1.41	Q920A7	AFG3-like protein 1	Afg3l1	4207	12	10	10	20.2	18.1	18.1	87.046	0.0000	117.82	4896800	52	13.37	14.00	13.98	13.21	13.30	13.42	13.17	12.70
1.550	0.49	1.41	P97452	Ribosome biogenesis protein BOP1	Bop1	1652	12	12	12	23.1	23.1	23.1	82.545	0.0000	100.51	14485000	198	15.48	14.92	15.68	15.51	14.81	15.00	14.96	14.85
2.423	0.49	1.41	Q8JZQ9	Eukaryotic translation initiation factor 3 subunit B	Eif3b	3579	37	37	37	56.2	56.2	56.2	91.369	0.0000	323.31	147200000	838	17.78	17.33	17.51	17.67	17.15	16.96	17.15	17.07
0.407	0.49	1.41	Q62193	Replication protein A 32 kDa subunit	Rpa2	2368	6	6	6	40.7	40.7	40.7	29.718	0.0000	45.664	7932000	54	15.34	15.84	15.74	15.38	16.18	15.26	15.21	13.68
2.324	0.49	1.41	Q80UY2	E3 ubiquitin-protein ligase KCMF1	Kcmf1	2842	5	5	5	18.6	18.6	18.6	41.791	0.0000	172.81	9020500	97	16.16	15.76	16.07	15.95	15.55	15.38	15.37	15.67
1.263	0.49	1.41	Q99NB9	Splicing factor 3B subunit 1	Sf3b1	4497	49	49	49	42.1	42.1	42.1	145.81	0.0000	323.31	62711000	588	16.85	16.15	16.76	16.57	15.87	15.85	16.32	16.32
1.517	0.49	1.40	A2AN08	E3 ubiquitin-protein ligase UBR4	Ubr4	34	86	86	86	23.3	23.3	23.3	572.28	0.0000	323.31	115760000	1125	17.47	16.86	17.44	17.47	16.83	16.65	17.06	16.75
0.878	0.49	1.40	Q3UBZ5	MIF4G domain-containing protein	Mif4gd	1943	2	2	2	17.6	17.6	17.6	25.493	0.0000	10.505	1027000	6	13.23	13.36	14.38	13.33	12.98	13.16	13.29	12.91
1.761	0.49	1.40	Q91W98	Solute carrier family 15 member 4	Slc15a4	4100	5	5	5	8.7	8.7	8.7	62.255	0.0000	32.557	20455000	123	16.60	16.11	16.80	16.68	16.08	16.04	16.06	16.06
0.237	0.49	1.40	P70403	Protein CASP	Cux1	1566	16	4	4	33	7.2	7.2	77.268	0.0000	16.889	5447300	15	15.08	15.45	13.28	15.60	12.95	13.61	15.51	15.39
1.290	0.49	1.40	Q8K268	ATP-binding cassette sub-family F member 3	Abcf3	3645	14	14	14	30.7	30.7	30.7	79.864	0.0000	83.039	16371000	166	16.02	15.82	16.22	16.00	14.98	15.68	15.80	15.63
1.293	0.49	1.40	Q60710	Deoxynucleoside triphosphate triphosphohydrolase SAMHD1	Samhd1	2196	51	51	51	68.7	68.7	68.7	75.892	0.0000	323.31	259260000	1353	18.59	18.15	17.97	17.91	17.93	17.83	17.53	17.37
0.903	0.49	1.40	P21855	B-cell differentiation antigen CD72	Cd72	868	15	15	15	44.6	44.6	44.6	40.347	0.0000	112.61	18691000	141	16.05	15.73	16.31	15.42	15.64	15.77	15.24	14.92
2.090	0.49	1.40	P05202	Aspartate aminotransferase, mitochondrial	Got2	650	36	36	36	73	73	73	47.411	0.0000	323.31	431080000	1054	19.99	19.63	19.83	19.82	19.36	19.03	19.50	19.42
2.182	0.49	1.40	P17710	Hexokinase-1	Hk1	828	57	57	50	53.8	53.8	48.4	108.3	0.0000	323.31	271750000	1314	18.17	17.78	18.05	17.97	17.56	17.29	17.71	17.46
0.891	0.49	1.40	Q91YT8	CSC1-like protein 1	Tmem63a	4180	7	7	7	11.9	11.9	11.9	91.859	0.0000	59.194	10005000	114	15.86	15.41	16.58	15.90	15.52	15.37	15.78	15.13
2.018	0.49	1.40	Q3ULD5	Methylcrotonoyl-CoA carboxylase beta chain, mitochondrial	Mccc2	1991	18	18	18	43.9	43.9	43.9	61.378	0.0000	129.04	29215000	222	16.82	16.66	16.87	16.86	16.26	16.12	16.66	16.20
1.343	0.49	1.40	Q8BMK4	Cytoskeleton-associated protein 4	Ckap4	3142	48	48	48	74.6	74.6	74.6	63.691	0.0000	323.31	162360000	1168	17.21	16.72	17.28	17.35	16.36	16.57	16.97	16.71
1.882	0.49	1.40	P58749	Transmembrane 6 superfamily member 1	Tm6sf1	1322	4	4	4	14.6	14.6	14.6	41.662	0.0000	6.9371	3126100	25	14.35	14.38	14.33	14.49	14.23	13.58	13.83	13.97
1.580	0.49	1.40	Q3UHD6;Q9	Sorting nexin-27	Snx27	1971	22	22	22	50.5	50.5	50.5	60.988	0.0000	106.99	31659000	281	16.50	15.89	15.95	16.10	15.55	15.82	15.40	15.73
0.839	0.48	1.40	Q9ET26	E3 ubiquitin-protein ligase RNF114	Rnf114	5355	6	6	6	28.4	28.4	28.4	25.745	0.0000	40.509	8372900	83	15.44	14.51	15.26	15.11	15.10	14.39	14.75	14.15
0.881	0.48	1.40	E9PYK3		Parp4	224	19	19	19	15.2	15.2	15.2	216.13	0.0000	69.218	16761000	199	15.42	15.17	15.76	15.54	15.18	15.16	15.36	14.26
0.398	0.48	1.40	A0A0B4J1G0		Fcgr4	0	3	3	3	13.7	13.7	13.7	28.398	0.0000	11.71	4586100	45	14.43	15.06	13.57	13.65	12.56	14.05	14.42	13.73
2.354	0.48	1.40	Q64337	Sequestosome-1	Sqstm1	2432	11	11	11	36.9	36.9	36.9	48.162	0.0000	130.89	36478000	256	17.45	17.35	17.25	17.38	17.15	16.68	16.89	16.78
1.536	0.48	1.40	Q91VZ6	Stromal membrane-associated protein 1	Smap1	4086	9	8	8	18.4	16.6	16.6	47.66	0.0000	48.188	11150000	181	15.25	14.90	15.24	15.03	14.62	14.23	14.70	14.94
0.248	0.48	1.40	Q71RI9	Kynurenine--oxoglutarate transaminase 3	Ccbl2	2716	5	5	5	16.5	16.5	16.5	51.126	0.0000	6.7273	3425000	39	12.09	14.25	14.70	14.30	13.15	12.73	14.92	12.61
0.351	0.48	1.40	Q9WV98	Mitochondrial import inner membrane translocase subunit Tim9	Timm9	5742	2	2	2	32.6	32.6	32.6	10.344	0.0000	10.078	2983500	47	13.78	15.03	13.88	12.58	12.54	13.78	13.91	13.10
1.088	0.48	1.40	Q99JX3	Golgi reassembly-stacking protein 2	Gorasp2	4353	4	4	4	16	16	16	47.038	0.0000	31.033	7590300	79	15.41	15.78	15.05	15.18	15.14	14.82	15.10	14.42
2.209	0.48	1.40	Q9QWR8	Alpha-N-acetylgalactosaminidase	Naga	5527	17	17	17	44.8	44.8	44.8	47.234	0.0000	70.782	80968000	289	18.83	18.59	18.68	18.46	18.29	18.02	18.00	18.33
2.412	0.48	1.40	Q8VC03	Echinoderm microtubule-associated protein-like 3	Eml3	3903	17	17	17	29.9	29.9	29.9	95.694	0.0000	111.31	18365000	222	15.54	15.13	15.44	15.44	14.83	14.91	14.82	15.07
2.363	0.48	1.40	P52332	Tyrosine-protein kinase JAK1	Jak1	1215	26	26	24	29.2	29.2	27.6	133.37	0.0000	110.76	31170000	307	16.34	16.07	16.43	16.32	15.94	15.65	15.94	15.71
2.945	0.48	1.40	O70172	Phosphatidylinositol 5-phosphate 4-kinase type-2 alpha	Pip4k2a	475	23	23	16	52.3	52.3	42.7	46.151	0.0000	146.37	76937000	456	17.68	17.40	17.58	17.74	17.05	17.06	17.18	17.18
2.794	0.48	1.40	Q8BK64	Activator of 90 kDa heat shock protein ATPase homolog 1	Ahsa1	3095	20	20	20	55.3	55.3	55.3	38.117	0.0000	323.31	104240000	514	18.25	18.19	18.23	18.19	17.51	17.69	17.90	17.83
0.409	0.48	1.40	Q9EPR4	Solute carrier family 23 member 2	Slc23a2	5262	3	3	3	8.2	8.2	8.2	70.048	0.0000	18.335	2442100	31	13.93	13.91	14.76	14.14	14.87	13.56	12.54	13.85
2.009	0.48	1.40	Q01730	Ras suppressor protein 1	Rsu1	1710	19	19	19	74.4	74.4	74.4	31.55	0.0000	223.25	73699000	308	17.79	17.85	17.95	18.16	17.47	17.18	17.55	17.63
0.322	0.48	1.40	Q6ZPF4	Formin-like protein 3	Fmnl3	2675	5	3	2	5.4	4.4	3.1	117.17	0.0022	3.0725	1797100	19	14.61	12.55	14.92	14.87	13.58	14.59	13.52	13.32
2.155	0.48	1.40	Q5U458	DnaJ homolog subfamily C member 11	Dnajc11	2164	18	18	18	34.3	34.3	34.3	63.232	0.0000	129.23	18097000	178	15.54	15.34	15.54	15.56	14.89	14.81	15.07	15.30
2.372	0.48	1.40	P16110	Galectin-3	Lgals3	805	16	16	16	45.1	45.1	45.1	27.515	0.0000	323.31	4239300000	4217	22.53	22.64	22.49	22.48	21.98	21.80	22.24	22.19
0.288	0.48	1.39	Q8CHP5	Partner of Y14 and mago	Wibg	3527	14	14	13	70.4	70.4	70.4	22.689	0.0000	141.08	11253000	95	12.99	12.96	12.49	14.02	11.30	14.00	13.29	11.95
0.441	0.48	1.39	Q60649	Caseinolytic peptidase B protein homolog	Clpb	2189	7	7	7	14.6	14.6	14.6	76.003	0.0006	4.2979	2395500	18	13.95	14.10	14.25	13.87	13.68	13.16	14.83	12.58
1.574	0.48	1.39	Q3U487	E3 ubiquitin-protein ligase HECTD3	Hectd3	1923	20	20	20	35.5	35.5	35.5	97.346	0.0000	61.948	25810000	216	16.00	15.66	16.30	16.06	15.43	15.49	15.81	15.38
1.022	0.48	1.39	O88587	Catechol O-methyltransferase	Comt	548	13	13	13	52.1	52.1	52.1	29.486	0.0000	216.16	55193000	347	17.42	17.52	16.96	17.66	17.12	16.35	17.03	17.15
1.722	0.48	1.39	Q61549	EGF-like module-containing mucin-like hormone receptor-like 1	Emr1	2311	10	10	10	10	10	10	102.13	0.0000	197.52	62871000	322	17.84	17.26	17.33	17.39	17.01	17.10	17.02	16.78
0.402	0.48	1.39	Q5SWT3	Solute carrier family 25 member 35	Slc25a35	2156	4	4	4	20.3	20.3	20.3	32.63	0.0000	37.571	1486400	24	13.62	13.59	13.32	13.37	12.75	14.39	11.91	12.94
0.183	0.48	1.39	P48441	Alpha-L-iduronidase	Idua	1145	2	2	2	4.6	4.6	4.6	71.253	0.0002	5.7507	8905300	41	15.82	16.15	16.50	16.01	16.91	12.74	15.83	17.09
0.610	0.48	1.39	Q7M753		Pank2	2738	5	5	5	14.7	14.7	14.7	50.212	0.0000	28.071	3720900	51	14.03	13.83	15.08	15.03	13.57	14.28	13.92	14.30
2.980	0.47	1.39	Q60676	Serine/threonine-protein phosphatase 5	Ppp5c	2193	19	19	19	43.1	43.1	43.1	56.876	0.0000	146.07	52535000	329	17.70	17.37	17.40	17.48	16.98	16.99	16.99	17.09
0.945	0.47	1.39	Q8BHZ0	Protein FAM49A	Fam49a	3053	14	12	12	45.5	38.7	38.7	37.342	0.0000	185.84	44913000	204	16.81	16.75	16.84	16.88	16.55	16.57	16.67	15.58
0.635	0.47	1.39	Q9QXD8	LIM domain-containing protein 1	Limd1	5537	8	8	8	25.4	25.4	25.4	71.421	0.0000	119.22	9952900	104	14.66	14.77	15.24	14.78	14.59	14.10	15.21	13.66
0.846	0.47	1.39	P03930	ATP synthase protein 8	Mtatp8	632	4	4	4	31.3	31.3	31.3	7.7662	0.0000	6.5461	30127000	84	18.37	18.04	18.04	17.41	17.69	17.62	17.75	16.91
2.231	0.47	1.39	Q91V92	ATP-citrate synthase	Acly	4052	55	55	55	55.1	55.1	55.1	119.73	0.0000	323.31	269590000	1401	18.74	18.58	18.56	18.56	18.27	18.33	18.07	17.87
1.252	0.47	1.39	Q60714	Long-chain fatty acid transport protein 1	Slc27a1	2197	13	12	12	27.2	26	26	71.275	0.0000	76.723	17295000	148	16.56	16.03	16.44	16.70	16.01	15.89	16.31	15.64
2.316	0.47	1.39	Q9CWJ9	Bifunctional purine biosynthesis protein PURH;Phosphoribosylaminoimi	Atic	4718	42	42	42	87.8	87.8	87.8	64.217	0.0000	323.31	395010000	1574	18.87	18.49	18.85	18.83	18.44	18.24	18.30	18.17
1.681	0.47	1.39	Q69ZN7	Myoferlin	Myof	2488	133	133	132	66.4	66.4	66.4	233.32	0.0000	323.31	516740000	2971	18.37	17.95	18.32	18.34	17.70	17.50	18.04	17.85
0.690	0.47	1.39	O35083	1-acyl-sn-glycerol-3-phosphate acyltransferase alpha	Agpat1	334	4	4	4	21.4	21.4	21.4	31.709	0.0000	81.267	24370000	142	17.16	16.81	17.73	17.72	17.04	16.24	17.41	16.84
0.895	0.47	1.39	Q9CQG2	Methyltransferase-like protein 16	Mettl16	4598	6	6	6	10.3	10.3	10.3	62.34	0.0004	4.8804	2422800	22	14.11	13.62	13.90	14.10	13.13	13.82	13.92	12.97
1.019	0.47	1.39	Q9D0F3	Protein ERGIC-53	Lman1	4862	14	14	14	30.9	30.9	30.9	57.788	0.0000	149.13	47630000	298	16.42	16.48	17.28	16.90	16.29	16.33	16.59	15.98
1.860	0.47	1.39	Q5SSL4	Active breakpoint cluster region-related protein	Abr	2139	42	42	40	59.1	59.1	57	97.666	0.0000	307.91	114430000	714	17.63	17.46	17.84	17.79	17.31	17.08	17.45	16.99
1.856	0.47	1.39	Q9EPL8	Importin-7	Ipo7	5259	21	21	20	21.5	21.5	20.5	119.49	0.0000	160.2	51005000	373	17.22	16.69	16.89	17.10	16.56	16.63	16.30	16.52
1.258	0.47	1.39	Q80Y98	Phospholipase DDHD2	Ddhd2	2898	7	7	7	13.9	13.9	13.9	79.576	0.0000	18.194	4740000	32	14.74	14.66	14.64	14.60	14.23	14.04	13.78	14.71
1.059	0.47	1.39	Q80U78	Pumilio homolog 1	Pum1	2824	15	15	11	16.8	16.8	13.2	126.62	0.0000	47.309	13490000	147	15.66	15.18	15.24	15.74	14.59	15.12	14.82	15.42
0.840	0.47	1.38	Q80TP3	E3 ubiquitin-protein ligase UBR5	Ubr5	2811	12	12	12	8.5	8.5	8.5	308.35	0.0000	80.171	6326300	85	14.61	14.43	15.13	15.14	13.96	14.21	14.96	14.30
0.707	0.47	1.38	Q8VI84	Nucleolar complex protein 3 homolog	Noc3l	4031	7	7	7	11.2	11.2	11.2	93.21	0.0000	8.5874	2278900	19	13.58	13.70	13.72	13.74	12.33	13.47	13.21	13.84
1.924	0.47	1.38	P97465	Docking protein 1	Dok1	1654	13	13	13	34.9	34.9	34.9	52.451	0.0000	93.984	35039000	298	16.33	16.14	16.74	16.42	15.90	15.87	16.06	15.92
0.863	0.47	1.38	Q8CFJ7	Solute carrier family 25 member 45	Slc25a45	3490	3	3	3	11.8	11.8	11.8	31.689	0.0000	29.325	1693200	20	13.63	14.00	13.96	14.07	13.31	14.05	12.83	13.60
0.947	0.47	1.38	Q91WK2	Eukaryotic translation initiation factor 3 subunit H	Eif3h	4116	23	23	23	68.5	68.5	68.5	39.832	0.0000	323.31	68116000	409	17.95	17.63	16.96	17.48	17.25	16.63	17.24	17.02
0.333	0.47	1.38	Q921I9	Exosome complex component RRP41	Exosc4	4228	7	7	7	39.2	39.2	39.2	26.249	0.0000	182.34	11028000	130	15.85	15.68	15.49	15.37	15.70	13.37	15.84	15.62

2.464	0.47	1.38	D3Z6Q9;Q7	Bridging integrator 2	Bin2	214	26	26	24	56.9	56.9	55	52.553	0.0000	291.98	76745000	600	17.38	17.19	17.41	17.66	16.87	16.96	16.98	16.97
2.019	0.47	1.38	P61620;Q9J	Protein transport protein Sec61 subunit alpha isoform 1	Sec61a1	1387	11	11	11	29.2	29.2	29.2	52.264	0.0000	128.35	61830000	322	17.96	17.61	17.79	17.95	17.59	17.32	17.14	17.39
0.320	0.47	1.38	O08908	Phosphatidylinositol 3-kinase regulatory subunit beta	Pik3r2	309	4	4	3	5	5	3.7	81.265	0.0022	3.0546	3066900	39	14.49	14.62	15.25	13.47	14.53	14.78	14.08	12.57
0.999	0.47	1.38	Q3UA06	Pachytene checkpoint protein 2 homolog	Trip13	1938	4	4	4	18.1	18.1	18.1	48.376	0.0000	17.86	2474200	32	14.00	14.01	14.96	14.21	13.87	13.62	13.88	13.95
0.352	0.47	1.38	Q8BG95	Protein phosphatase 1 regulatory subunit 12B	Ppp1r12b	2969	8	6	6	9.6	7.1	7.1	109.05	0.0000	8.209	3747700	16	14.89	12.94	14.00	14.19	13.27	14.42	12.56	13.91
0.452	0.47	1.38	Q8R2N2	Cirhin	Cirh1a	3811	4	4	4	10.2	10.2	10.2	76.908	0.0000	22.249	2025200	36	14.06	13.64	13.26	13.78	13.47	12.31	14.30	12.80
0.195	0.47	1.38	Q9Z2F2	2-5-oligoadenylate synthase-like protein 2	Oasl2	5849	8	8	7	23.2	23.2	17.9	58.767	0.0000	26.838	8915500	65	15.18	15.16	12.65	13.04	15.49	13.04	12.80	12.83
0.390	0.47	1.38	Q99LC8	Translation initiation factor eIF-2B subunit alpha	Eif2b1	4418	7	7	7	31.5	31.5	31.5	33.816	0.0000	20.801	8740100	70	15.58	15.16	15.22	13.44	14.99	14.08	14.23	14.24
0.767	0.46	1.38	Q6SJQ0	CMRF35-like molecule 8	Cd300a	2657	6	6	6	18.6	18.6	18.6	35.629	0.0000	16.262	5320100	42	15.12	14.97	15.58	14.82	15.06	14.52	14.01	15.03
0.219	0.46	1.38	Q8BKS9	Pumilio domain-containing protein KIAA0020	Kiaa0020	3104	5	5	5	8.5	8.5	8.5	72.799	0.0000	14.108	4537700	36	15.14	14.78	14.59	14.65	14.84	15.62	14.99	11.86
0.102	0.46	1.38	P47955	60S acidic ribosomal protein P1	Rplp1	1133	3	3	3	79.8	79.8	79.8	11.475	0.0000	87.004	25982000	67	18.39	17.18	17.70	17.25	18.72	18.92	18.81	12.21
1.199	0.46	1.38	Q62418	Drebrin-like protein	Dbnl	2387	19	19	19	48.9	48.9	48.9	48.699	0.0000	162.64	52686000	314	18.13	18.59	17.68	17.86	17.71	17.55	17.49	17.65
1.757	0.46	1.38	Q99K85	Phosphoserine aminotransferase	Psat1	4366	26	26	26	65.7	65.7	65.7	40.472	0.0000	223.76	87357000	612	17.41	17.27	16.86	16.99	16.82	16.75	16.56	16.55
1.507	0.46	1.38	P47809	Dual specificity mitogen-activated protein kinase kinase 4	Map2k4	1125	7	7	7	23.9	23.9	23.9	44.113	0.0000	57.418	20620000	189	16.26	16.04	16.63	16.52	15.92	15.99	16.07	15.61
0.589	0.46	1.38	Q3U2C5	E3 ubiquitin-protein ligase RNF149	Rnf149	1910	5	5	5	17.8	17.8	17.8	42.552	0.0000	6.7526	4513200	57	14.89	13.87	14.80	14.06	13.66	14.74	13.65	13.70
1.616	0.46	1.38	E9Q5K9	YTH domain-containing protein 1	Ythdc1	239	9	9	9	12.4	12.4	12.4	85.648	0.0000	22.561	10081000	91	15.34	15.04	15.54	15.21	14.56	15.08	14.71	14.92
2.210	0.46	1.38	P97311	DNA replication licensing factor MCM6	Mcm6	1621	28	28	27	35.3	35.3	35.3	92.866	0.0000	119.26	42384000	312	16.75	16.30	16.74	16.60	16.24	16.09	16.14	16.07
0.914	0.46	1.38	Q8BTY8	Sec1 family domain-containing protein 2	Scfd2	3213	9	9	9	23.2	23.2	23.2	74.75	0.0000	84.081	10207000	111	15.73	15.33	15.18	16.01	15.26	14.61	15.40	15.13
0.669	0.46	1.38	P43406	Integrin alpha-V;Integrin alpha-V heavy chain;Integrin alpha-V light chai	Itgav	1092	16	16	16	19.6	19.6	19.6	115.36	0.0000	56.932	20604000	154	16.40	16.00	17.24	16.82	16.44	15.63	16.47	16.07
0.670	0.46	1.38	O88627	Sodium/nucleoside cotransporter 2	Slc28a2	551	4	4	4	10.6	10.6	10.6	72.916	0.0000	31.66	3770000	30	15.21	14.39	15.44	14.81	13.83	14.87	14.81	14.48
0.520	0.46	1.38	Q9D0M0	Exosome complex exonuclease RRP42	Exosc7	4877	6	6	6	36.8	36.8	36.8	31.825	0.0000	53.98	5286700	47	14.53	14.03	14.59	14.35	13.92	13.69	14.96	13.09
2.207	0.46	1.38	Q8BU31	Ras-related protein Rap-2c	Rap2c	3219	9	4	4	58.5	29.5	29.5	20.745	0.0000	21.197	18982000	115	16.45	16.72	16.54	16.58	16.18	16.11	16.32	15.85
2.331	0.46	1.38	Q61425	Hydroxyacyl-coenzyme A dehydrogenase, mitochondrial	Hadh	2299	21	21	21	80.9	80.9	80.9	34.463	0.0000	323.31	113590000	539	18.31	17.94	18.03	18.29	17.80	17.65	17.57	17.71
1.895	0.46	1.38	P51807	Dynein light chain Tctex-type 1	Dynlt1	1202	4	4	4	49.6	49.6	49.6	12.483	0.0000	281.93	20244000	150	16.90	16.64	16.72	17.05	16.45	16.11	16.37	16.55
1.914	0.46	1.38	Q9DBS1	Transmembrane protein 43	Tmem43	5173	24	24	24	63.5	63.5	63.5	44.783	0.0000	323.31	124620000	675	17.99	17.44	17.90	17.81	17.23	17.45	17.34	17.27
2.062	0.46	1.38	P63085	Mitogen-activated protein kinase 1	Mapk1	1488	24	18	18	70.1	60.3	60.3	41.275	0.0000	221.28	132410000	534	18.94	18.57	18.71	18.57	18.39	18.24	18.31	18.01
1.508	0.46	1.38	O35711	Liprin-beta-2	Ppfibp2	401	18	18	17	27.9	27.9	26.5	98.752	0.0000	90.546	20461000	197	16.15	15.90	16.54	16.39	15.54	15.84	15.94	15.82
0.329	0.46	1.38	Q9D125	28S ribosomal protein S25, mitochondrial	Mrps25	4893	4	4	4	21.6	21.6	21.6	19.92	0.0000	32.195	4116600	45	15.72	14.39	14.76	13.94	14.91	14.52	14.67	12.88
0.935	0.46	1.38	Q08024	Core-binding factor subunit beta	Cbfb	1759	12	12	12	63.1	63.1	63.1	22.03	0.0000	35.838	11158000	109	16.04	15.50	15.74	15.82	15.48	15.22	14.74	15.81
1.749	0.46	1.38	Q6PGF7	Exocyst complex component 8	Exoc8	2635	23	23	23	33.1	33.1	33.1	81.034	0.0000	159.77	42112000	367	16.94	16.38	16.48	16.74	16.24	16.07	16.32	16.06
0.930	0.46	1.38	Q3UGR5	Haloacid dehalogenase-like hydrolase domain-containing protein 2	Hdhd2	1963	6	6	6	44.4	44.4	44.4	28.73	0.0000	56.04	21942000	168	16.88	16.95	17.29	17.42	16.80	17.10	16.72	16.08
1.032	0.46	1.37	Q78IK2	Up-regulated during skeletal muscle growth protein 5	Usmg5	2722	4	4	4	44.8	44.8	44.8	6.3814	0.0000	21.442	12122000	112	15.47	15.51	15.97	15.74	15.30	14.63	15.49	15.43
1.789	0.46	1.37	P10852	4F2 cell-surface antigen heavy chain	Slc3a2	731	31	31	30	58.7	58.7	54.4	58.336	0.0000	323.31	171900000	695	18.91	18.59	18.61	19.03	18.47	18.09	18.43	18.31
1.580	0.46	1.37	Q99KK7	Dipeptidyl peptidase 3	Dpp3	4382	35	35	35	58.1	58.1	58.1	82.897	0.0000	323.31	132800000	610	18.68	18.53	18.24	18.35	18.19	17.84	18.22	17.73
1.046	0.46	1.37	P09925	Surfeit locus protein 1	Surf1	702	6	6	6	29.7	29.7	29.7	34.798	0.0000	34.801	9396200	72	14.86	14.98	15.82	15.36	14.85	14.73	14.96	14.63
1.696	0.46	1.37	Q9JIW9	Ras-related protein Ral-B	Ralb	5408	12	12	7	51.5	51.5	27.2	23.349	0.0000	98.563	76931000	307	18.23	17.71	18.02	18.22	17.69	17.71	17.60	17.36
1.420	0.46	1.37	Q99P88	Nuclear pore complex protein Nup155	Nup155	4503	19	19	19	18.2	18.2	18.2	155.12	0.0000	323.31	31919000	370	16.40	15.94	16.16	16.39	15.98	15.38	15.88	15.82
1.080	0.46	1.37	Q8BX10	Serine/threonine-protein phosphatase PGAM5, mitochondrial	Pgam5	3276	7	7	7	25	25	25	31.994	0.0000	21.2	12161000	121	16.37	15.89	15.47	16.32	15.71	15.63	15.44	15.45
2.624	0.46	1.37	Q80TY0;P1	Formin-binding protein 1	Fnbp1	2815	28	28	27	47.1	47.1	47.1	71.343	0.0000	173.6	49960000	407	16.76	16.50	16.61	16.82	16.34	16.22	16.07	16.23
2.485	0.46	1.37	Q68FL4	Putative adenosylhomocysteinase 3	Ahcyl2	2476	23	23	14	40.5	40.5	26.8	66.899	0.0000	177.2	108130000	580	17.55	17.52	17.91	17.70	17.13	17.28	17.26	17.18
1.953	0.46	1.37	Q3TDN2	FAS-associated factor 2	Faf2	1833	17	17	17	43.6	43.6	43.6	52.471	0.0000	290.46	33970000	301	16.50	16.15	16.57	16.58	15.99	15.83	15.95	16.20
2.262	0.46	1.37	Q80X73	Protein pelota homolog	Pelo	2872	11	11	11	33.5	33.5	33.5	43.349	0.0000	35.896	9697500	118	15.65	15.29	15.30	15.42	14.93	14.89	14.87	15.16
0.539	0.46	1.37	Q9QZH6	Evolutionarily conserved signaling intermediate in Toll pathway, mitochc	Ecsit	5598	4	4	4	12	12	12	49.798	0.0000	30.079	1858000	16	14.07	13.47	13.63	13.16	13.11	13.94	13.20	12.26
1.092	0.46	1.37	P62046	Leucine-rich repeat and calponin homology domain-containing protein 1	Lrch1	1400	7	7	7	13.5	13.5	13.5	79.075	0.0000	21.555	7874900	98	15.22	14.96	15.82	15.75	14.80	14.96	15.04	15.13
1.185	0.46	1.37	E9PVA8		Gcn1l1	221	79	79	79	39.5	39.5	39.5	293.02	0.0000	323.31	124280000	1358	16.77	16.27	17.15	16.78	16.24	16.15	16.55	16.20
1.931	0.45	1.37	P68404;P20	Protein kinase C beta type	Prkcb	1520	24	24	24	42.6	42.6	42.6	76.75	0.0000	66.789	56240000	390	17.02	16.84	17.23	17.15	16.63	16.46	16.86	16.46
1.120	0.45	1.37	Q3U0V2	Tumor necrosis factor receptor type 1-associated DEATH domain protei	Tradd	1898	6	6	6	37.1	37.1	37.1	34.577	0.0000	250.41	23856000	185	16.49	16.62	16.62	17.05	16.21	16.38	16.60	15.78
1.035	0.45	1.37	Q99J56	Derlin-1	Derl1	4326	4	4	4	16.3	16.3	16.3	28.834	0.0000	9.4962	25817000	74	16.83	17.28	17.38	17.04	17.21	16.37	16.69	16.44
0.657	0.45	1.37	Q08122	Transducin-like enhancer protein 3	Tle3	1761	8	8	4	15.7	15.7	9.8	83.446	0.0000	21.187	20540000	58	17.72	16.85	17.87	17.83	17.48	16.90	17.51	16.58
1.602	0.45	1.37	Q5FWK3	Rho GTPase-activating protein 1	Arhgap1	2105	22	22	22	61.3	61.3	61.3	50.41	0.0000	306.58	107950000	659	18.15	17.94	18.64	18.36	17.89	17.87	17.74	17.77
1.013	0.45	1.37	O70496	H(+)/Cl(-) exchange transporter 7	Clcn7	509	15	15	15	24.4	24.4	24.4	88.712	0.0000	111.05	24490000	172	16.74	16.05	16.48	16.10	15.44	16.22	15.95	15.95
0.244	0.45	1.37	O35239	Tyrosine-protein phosphatase non-receptor type 9	Ptpn9	350	6	6	6	13.3	13.3	13.3	67.969	0.0000	15.443	6124000	57	15.18	15.03	15.39	12.36	14.62	14.00	14.08	13.45
0.429	0.45	1.37	Q80Y17	Lethal(2) giant larvae protein homolog 1	Llgl1	2894	7	7	7	12	12	12	112.62	0.0000	14.495	3015800	30	13.96	14.13	14.55	13.88	12.85	14.78	13.06	14.02
1.835	0.45	1.37	P30416	Peptidyl-prolyl cis-trans isomerase FKBP4;Peptidyl-prolyl cis-trans isom	Fkbp4	978	30	30	30	69.9	69.9	69.9	51.572	0.0000	267.15	68119000	507	17.25	16.65	16.98	17.05	16.65	16.52	16.51	16.43
1.485	0.45	1.37	Q8C754	Vacuolar protein sorting-associated protein 52 homolog	Vps52	3409	17	17	17	32.4	32.4	32.4	82.043	0.0000	141.61	31805000	335	16.19	15.91	16.33	16.28	15.35	15.76	16.00	15.78
0.675	0.45	1.37	Q5RKZ7	Molybdenum cofactor biosynthesis protein 1;Cyclic pyranopterin monop	Mocs1	2127	10	10	10	22.3	22.3	22.3	69.858	0.0000	57.286	8627300	116	14.91	14.99	14.87	14.56	15.09	13.57	14.44	14.42
1.144	0.45	1.37	Q8BN21	Serine/threonine-protein kinase VRK2	Vrk2	3152	10	10	10	23.9	23.9	23.9	58.118	0.0000	29.703	14006000	87	16.37	15.77	16.39	15.86	15.36	15.96	15.54	15.72
1.546	0.45	1.37	E9QAT4		Sec16a	254	23	23	23	17.4	17.4	17.4	254.2	0.0000	106.43	21990000	220	16.23	15.83	16.44	16.42	15.69	15.64	15.84	15.94
1.138	0.45	1.37	Q9EPV8	Ubiquitin-like protein 5	Ubl5	5266	6	6	6	49.3	49.3	49.3	8.5468	0.0000	12.085	10098000	69	15.27	15.42	15.07	15.64	14.82	14.46	15.10	15.22
2.444	0.45	1.37	Q8C147	Dedicator of cytokinesis protein 8	Dock8	3352	69	69	67	38.1	38.1	37.8	238.98	0.0000	323.31	130060000	1155	17.13	16.76	17.02	17.00	16.63	16.43	16.63	16.41
1.068	0.45	1.37	Q8R3C0	Mini-chromosome maintenance complex-binding protein	Mcmbp	3840	9	9	9	22.1	22.1	22.1	72.89	0.0000	28.103	3606100	45	14.54	14.19	14.02	14.39	14.18	13.93	13.30	13.93
1.715	0.45	1.37	O55029	Coatomer subunit beta	Copb2	445	50	50	50	61.2	61.2	61.2	102.45	0.0000	323.31	240470000	963	18.97	18.86	18.83	18.76	18.64	18.11	18.63	18.24
0.431	0.45	1.37	Q99LH2	Phosphatidylserine synthase 1	Ptdss1	4430	3	3	3	9.3	9.3	9.3	55.603	0.0002	5.7954	9239000	43	16.12	15.32	16.39	16.44	15.66	16.30	15.98	14.52
0.802	0.45	1.37	Q9D4F8	Gamma-tubulin complex component 4	Tubgcp4	4975	5	5	5	12.3	12.3	12.3	76.125	0.0000	10.517	2802200	21	13.84	13.64	14.80	14.17	13.58	13.89	13.38	13.79
0.912	0.45	1.37	Q91VK1;Q2	Basic leucine zipper and W2 domain-containing protein 2	Bzw2	1797	7	6	6	21	19.3	19.3	48.063	0.0000	55.35	7467200	109	14.94	14.98	14.78	14.72	15.11	14.02	14.27	14.21
1.574	0.45	1.37	O55106	Striatin	Strn	454	20	20	19	47.8	47.8	46.5	85.965	0.0000	228.61	35636000	285	16.48	16.26	16.78	16.76	15.86	16.29	16.15	16.18
1.889	0.45	1.37	Q9Z1Q5	Chloride intracellular channel protein 1	Clic1	5818	24	24	24	79.7	79.7	79.7	27.013	0.0000	323.31	830680000	1199	21.26	21.30	21.71	21.36	20.98	21.05	21.06	20.73
3.688	0.45	1.36	Q61598	Rab GDP dissociation inhibitor beta	Gdi2	2317	58	58	51	95.3	95.3	83.4	50.537	0.0000	323.31	1315500000	2755	19.97	20.16	20.01	20.00	19.65	19.58	19.61	19.49
1.474	0.45	1.36	Q3TWW8	Serine/arginine-rich splicing factor 6	Srsf6	1884	14	14	11	37.2	37.2	32.4	39.025	0.0000	147.71	39258000	361	17.28	17.46	17.30	17.25	16.82	17.16	16.45	17.06
1.047	0.45	1.36	Q9CRT8	Exportin-T	Xpot	4694	3	3	3	4.5	4.5	4.5	109.73	0.0000	29.154	4950000	58	14.98	14.92	15.35	15.68	14.46	14.96	15.04	14.68
2.232	0.45	1.36	P19783	Cytochrome c oxidase subunit 4 isoform 1, mitochondrial	Cox4i1	849	18	18	18	67.5	67.5	67.5	19.53	0.0000	181.22	382210000	658	20.04	19.75	20.12	20.20	19.56	19.51	19.70	19.55
1.674	0.45	1.36	Q7M6Y3	Phosphatidylinositol-binding clathrin assembly protein	Picalm	2737	24	24	22	42.7	42.7	39.7	71.543	0.0000	323.31	101580000	542	19.24	18.67	19.17	19.22	18.58	18.76	18.63	18.54
1.833	0.45	1.36	O09106	Histone deacetylase 1	Hdac1	322	17	17	10	47.1	47.1	34.6	55.074	0.0000	265.86	116660000	403	18.70	18.48	18.80	18.96	18.54	18.23	18.13	18.25
2.113	0.45	1.36	P25911	Tyrosine-protein kinase Lyn	Lyn	912	36	36	34	72.3	72.3	69.9	58.812	0.0000	323.31	159530000	941	17.75	17.54	17.60	17.29	17.21	17.12	17.14	16.92
0.462	0.45	1.36	P48999	Arachidonate 5-lipoxygenase	Alox5	1153	13	13	13	21.5	21.5	21.5	77.966	0.0000	33.542	11349000	121	15.12	15.16	14.59	15.47	15.16	15.35	14.45	13.61
0.706	0.44	1.36	P08226	Apolipoprotein E	Apoe	682	32	32	32	78.5	78.5	78.5	35.866	0.0000	323.31	328940000	943	19.14	18.81	19.64	19.71	18.71	18.36	19.10	19.35
1.098	0.44	1.36	Q9QY76	Vesicle-associated membrane protein-associated protein B	Vapb	5562	18	17	17	60.9	60.9	60.9	26.946	0.0000	118.42	143800000	492	18.24	18.11	18.79	18.70	17.68	18.02	18.31	18.05
0.819	0.44	1.36	Q7TPH6	E3 ubiquitin-protein ligase MYCBP2	Mycbp2	2768	18	18	18	5.8	5.8	5.8	521.23	0.0000	47.665	16704000	133	16.46	15.91	16.46	16.50	15.69	15.44	16.51	15.93
0.198	0.44	1.36	O35492	Dual specificity protein kinase CLK3	Clk3	376	2	2	2	6	6	6	73.797	0.0000	40.813	864860	20	14.74	14.36	12.25	11.67	11.97	12.84	14.04	12.42
0.175	0.44	1.36	Q9D3B1	Very-long-chain (3R)-3-hydroxyacyl-CoA dehydratase 2	Hacd2	4963	3	3	3	11.8	11.8	11.8	28.402	0.0000	19.016	8811600	71	16.26	15.64	12.24	16.26	14.61	14.40	15.14	14.51
0.516	0.44	1.36	Q9D0C4	tRNA (guanine(37)-N1)-methyltransferase	Trmt5	4858	4	4	4	15.4	15.4	15.4	56.794	0.0000	50.181	4778000	52	14.75	15.02	13.83	15.06	14.63	13.62	13.92	14.73
1.715	0.44	1.36	Q8K0Z7	Translational activator of cytochrome c oxidase 1	Taco1	3611	8	8	8	45.9	45.9	45.9	32.314	0.0000	47.827	11367000	94	15.68	15.50	15.79	16.00	15.17	15.14	15.36	15.54
0.703	0.44	1.36	Q80VC6	tRNA selenocysteine 1-associated protein 1	Trnau1ap	2848	2	2	2	10.1	10.1	10.1	32.423	0.0000	7.772	1047600	24	13.13	13.80	13.77	14.30	13.55	13.71	13.00	12.98
2.143	0.44	1.36	Q9CVD2	Ataxin-3	Atxn3	4707	6	6	6	22.5	22.5	22.5	40.533	0.0000	37.68	7811500	67	15.58	15.56	15.34	15.62	15.06	14.84	15.23	15.21
1.965	0.44	1.36	Q9JHU4	Cytoplasmic dynein 1 heavy chain 1	Dync1h1	5374	297	297	297	63.4	63.4	63.4	532.04	0.0000	323.31	1162200000	6469	19.36	18.94	19.43	19.34	18.83	18.81	18.96	18.71
1.663	0.44	1.36	P46638;Q9V	Ras-related protein Rab-11B	Rab11b	1107	19	19	4	70.6	70.6	17.9	24.489	0.0000	206.12	207410000	471	19.38	19.07	18.92	19.39	18.68	18.55	18.84	18.93
1.041	0.44	1.36	Q9CX30	Protein YIF1B	Yif1b	4745	5	5	5	35.4	35.4	35.4	33.982	0.0000	60.855	9865700	86	16.06	15.67	15.97	16.21	15.06	15.80	15.42	15.86
0.921	0.44	1.36	P04627	Serine/threonine-protein kinase A-Raf	Araf	643	11	7	6	25.3	18.2	15.6	67.581	0.0000	23.665	10799000	64	15.18	15.24	15.79	15.48	14.56	15.29	15.35	14.74
1.275	0.44	1.36	Q61712	DnaJ homolog subfamily C member 1	Dnajc1	2327	11	11	11	23.7	23.7	23.7	63.869	0.0000	32.258	8122400	75	14.29	14.92	14.31	14.38	13.82	13.90	14.25	14.18
0.884	0.44	1.36	P15535	Beta-1,4-galactosyltransferase 1;Lactose synthase A protein;N-acetylla	B4galt1	799	5	5	5	13.5	13.5	13.5	44.411	0.0000	11.029	5956600	34	14.50	14.54	14.93	15.45	14.54	14.17	14.68	14.27
0.805	0.44	1.36	P33215	Protein NEDD1	Nedd1	1001	5	5	5	14.7	14.7	14.7	71.292	0.0000	32.41	6452100	79	15.02	14.40	15.65	15.09	14.48	14.51	14.88	14.54
1.560	0.44	1.36	P16332	Methylmalonyl-CoA mutase, mitochondrial	Mut	809	20	20	20	47.2	47.2	47.2	82.843	0.0000	323.31	28162000	320	15.95	15.49	15.71	15.73	15.46	15.37	15.36	14.93
4.678	0.44	1.35	P17427	AP-2 complex subunit alpha-2	Ap2a2	823	45	37	37	54.5	45.1	45.1	104.02	0.0000	323.31	105300000	766	17.32	17.32	17.40	17.39	16.93	16.84	16.98	16.93
0.484	0.44	1.35	Q9EPS3	D-glucuronyl C5-epimerase	Glce	5263	4	4	4	8.1	8.1	8.1	70.088	0.0004	4.9685	2471200	15	14.19	13.89	14.27	13.83	14.76	13.12	13.51	13.04
4.198	0.44	1.35	P63101	14-3-3 protein zeta/delta	Ywhaz	1491	37	37	29	84.1	84.1	77.6	27.771	0.0000	323.31	1581100000	2128	21.60	21.49	21.52	21.57	21.03	21.13	21.20	21.06
0.675	0.44	1.35	Q9R0L7	A-kinase anchor protein 8-like	Akap8l	5628	8	8	8	17	17	17	71.453	0.0000	20.045	5365000	50	14.72	14.09	15.11	14.75	14.12	14.35	14.78	13.67
1.624	0.44	1.35	A2AF47	Dedicator of cytokinesis protein 11	Dock11	19	43	43	42	25.5	25.5	25	237.77	0.0000	183.54	51875000	528	16.46	15.91	16.46	16.29	15.74	15.83	16.02	15.78
1.709	0.44	1.35	Q9WTI7	Unconventional myosin-Ic	Myo1c	5673	65	65	64	55.6	55.6	54.8	121.94	0.0000	323.31	233080000	1328	18.53	17.99	18.50	18.28	17.85	17.78	18.06	17.85
0.877	0.44	1.35	Q9DCT1	1,5-anhydro-D-fructose reductase	Akr1e2	5229	12	12	12	48.8	48.8	48.8	34.46	0.0000	75.477	21224000	190	15.92	15.81	16.76	16.12	15.55	15.93	15.95	15.42
1.654	0.44	1.35	P05201	Aspartate aminotransferase, cytoplasmic	Got1	649	34	34	34	80.6	80.6	80.6	46.247	0.0000	323.31	264300000	805	18.61	18.60	19.04	18.87	18.29	18.47	18.52	18.10
0.424	0.44	1.35	Q9EPL0	Xylosyltransferase 2	Xylt2	5258	5	5	5	6.4	6.4	6.4	96.81	0.0000	8.9232	3086800	37	12.23	13.54	14.12	13.97	13.43	12.67	12.97	13.05
0.774	0.44	1.35	Q8CAA7	Glucose 1,6-bisphosphate synthase	Pgm2l1	3427	12	12	12	27.2	27.2	27.2	70.279	0.0000	284.1	20543000	170	15.90	15.77	16.78	16.40	15.78	15.36	16.08	15.89
2.567	0.44	1.35	Q91V76	Ester hydrolase C11orf54 homolog		4049	10	10	10	45.1	45.1	45.1	34.995	0.0000	294.59	38181000	268	16.77	16.70	16.96	16.89	16.39	16.31	16.58	16.29
0.879	0.43	1.35	Q7TNG5	Echinoderm microtubule-associated protein-like 2	Eml2	2762	16	16	16	36.1	36.1	36.1	70.733	0.0000	68.971	19991000	152	15.86	15.93	15.75	16.24	16.02	15.60	15.50	14.92
1.478	0.43	1.35	O70252	Heme oxygenase 2	Hmox2	480	26	26	26	72.4	72.4	72.4	35.738	0.0000	323.31	141270000	702	18.11	17.68	18.33	18.10	17.73	17.51	17.79	17.45
0.990	0.43	1.35	Q3U0M1	Trafficking protein particle complex subunit 9	Trappc9	1896	7	7	7	7.2	7.2	7.2	128.23	0.0000	16.347	3629500	38	13.60	13.59	14.35	13.91	13.07	13.38	13.70	13.56
0.887	0.43	1.35	Q91VF2	Histamine N-methyltransferase	Hnmt	4060	6	6	6	32.9	32.9	32.9	33.664	0.0000	23.129	7429500	62	15.16	14.90	15.27	15.39	15.19	14.71	14.94	14.14
1.276	0.43	1.35	Q9JKW0	ADP-ribosylation factor-like protein 6-interacting protein 1	Arl6ip1	5461	6	6	6	30.5	30.5	30.5	23.437	0.0000	84.658	28190000	212	16.37	16.62	16.22	16.32	16.08	15.56	16.31	15.85
1.350	0.43	1.35	P50429	Arylsulfatase B	Arsb	1182	9	9	9	17	17	17	59.646	0.0000	18.37	18253000	117	16.73	17.05	16.46	16.77	16.03	16.25	16.61	16.39
1.918	0.43	1.35	Q3UM45	Protein phosphatase 1 regulatory subunit 7	Ppp1r7	1997	20	20	20	47.6	47.6	47.6	41.291	0.0000	178.66	36308000	335	16.94	16.71	16.96	17.04	16.61	16.18	16.54	16.58
1.948	0.43	1.35	P27601	Guanine nucleotide-binding protein subunit alpha-13	Gna13	936	21	21	20	55.2	55.2	53.1	44.054	0.0000	218.56	179250000	517	20.06	19.78	20.07	20.22	19.56	19.41	19.77	19.67
1.169	0.43	1.35	Q8BHC4	Dephospho-CoA kinase domain-containing protein	Dcakd	3029	8	8	8	40.7	40.7	40.7	26.476	0.0000	27.211	14093000	93	15.79	15.77	15.40	15.59	15.37	14.76	15.56	15.12
0.695	0.43	1.35	Q5SUC9	Protein SCO1 homolog, mitochondrial	Sco1	2144	3	3	3	14.8	14.8	14.8	31.617	0.0000	25.243	8072900	37	15.91	15.91	16.11	15.75	14.61	15.83	15.78	15.73
0.583	0.43	1.35	Q8BGU5	Cyclin-Y	Ccny	2995	3	3	3	12	12	12	39.394	0.0000	14.24	5850200	80	15.31	14.96	14.80	14.98	14.90	14.84	15.00	13.59

2.566	0.43	1.35	Q99PT1	Rho GDP-dissociation inhibitor 1	Arhgdia	4516	24	24	24	94.6	94.6	94.6	23.407	0.0000	323.31	1174400000	1400	21.41	21.25	21.44	21.47	21.02	21.02	21.06	20.74
0.893	0.43	1.35	Q3UIU2	NADH dehydrogenase [ubiquinone] 1 beta subcomplex subunit 6	Ndufb6	1981	4	4	4	35.2	35.2	35.2	15.515	0.0000	26.109	22089000	105	16.49	16.48	17.24	16.98	16.49	16.10	16.76	16.12
0.863	0.43	1.35	Q9EQQ2	Protein YIPF5	Yipf5	5286	3	3	3	22.2	22.2	22.2	27.873	0.0000	96.637	9222900	81	16.45	15.97	15.37	16.39	15.57	15.65	15.67	15.55
2.382	0.43	1.35	Q5EG47;Q8	5-AMP-activated protein kinase catalytic subunit alpha-1	Prkaa1	2101	19	19	19	39.2	39.2	39.2	63.928	0.0000	86.296	39699000	263	16.54	16.48	16.53	16.69	16.21	16.18	16.24	15.88
0.977	0.43	1.35	Q9D0M1	Phosphoribosyl pyrophosphate synthase-associated protein 1	Prpsap1	4878	16	14	14	58.1	54.2	54.2	39.431	0.0000	95.453	64687000	209	19.08	18.36	18.84	18.58	18.10	18.33	18.73	17.99
2.671	0.43	1.35	P27612	Phospholipase A-2-activating protein	Plaa	937	35	35	35	50	50	50	87.22	0.0000	323.31	74411000	558	17.62	17.39	17.61	17.75	17.21	17.08	17.13	17.23
2.187	0.43	1.35	Q9DBG6	Dolichyl-diphosphooligosaccharide--protein glycosyltransferase subunit	Rpn2	5153	29	29	29	50.7	50.7	50.7	69.062	0.0000	323.31	254170000	912	18.94	18.59	18.69	18.86	18.31	18.16	18.48	18.41
0.701	0.43	1.35	Q9QYC0	Alpha-adducin	Add1	5568	17	17	16	30.6	30.6	29.5	80.646	0.0000	55.103	25075000	184	15.07	15.75	16.34	16.26	15.37	15.42	15.33	15.57
0.525	0.43	1.35	Q9CQW2	ADP-ribosylation factor-like protein 8B	Arl8b	4643	10	10	5	55.9	55.9	23.7	21.539	0.0000	122.51	91601000	270	19.31	18.56	17.70	18.25	18.33	17.67	18.31	17.79
0.400	0.43	1.35	Q8BMQ8	Vacuolar fusion protein MON1 homolog B	Mon1b	3147	5	5	5	21.5	21.5	21.5	60.015	0.0000	8.6104	1269700	9	13.87	13.29	14.86	13.61	14.25	12.78	13.76	13.12
0.374	0.43	1.35	Q8R570	Synaptosomal-associated protein 47	Snap47	3880	5	5	5	18.2	18.2	18.2	46.524	0.0000	23.361	3628700	54	14.26	14.28	15.05	15.03	14.67	14.79	14.54	12.90
0.885	0.43	1.35	P08508	Low affinity immunoglobulin gamma Fc region receptor III	Fcgr3	685	4	4	2	19.2	19.2	12.3	30.036	0.0000	26.34	11187000	71	16.56	16.25	15.95	16.07	15.97	15.24	16.20	15.70
1.863	0.43	1.35	Q00262	Syntaxin-2	Stx2	1695	6	6	6	35.3	35.3	35.3	33.177	0.0000	285.76	14422000	184	16.36	15.95	16.00	16.36	15.72	15.67	15.89	15.67
0.370	0.43	1.35	Q8BVI5	Syntaxin-16	Stx16	3242	5	5	5	19.9	19.9	19.9	37.08	0.0000	135.99	5617000	73	15.38	14.81	14.51	14.65	14.16	13.22	14.94	15.32
0.539	0.43	1.35	Q9JHL0	Linker for activation of T-cells family member 2	Lat2	5367	4	4	4	30	30	30	22.876	0.0000	52.453	3040300	66	13.84	13.73	14.75	14.00	13.25	13.07	14.10	14.19
1.113	0.43	1.34	Q00519;Q31	Xanthine dehydrogenase/oxidase;Xanthine dehydrogenase;Xanthine ox	Xdh	1697	57	57	55	47.3	47.3	46.6	146.56	0.0000	323.31	289430000	1532	18.91	18.31	18.65	18.49	18.44	17.87	18.42	17.92
2.981	0.43	1.34	P97384	Annexin A11	Anxa11	1641	35	35	34	48.9	48.9	48.9	54.079	0.0000	323.31	178500000	966	18.06	17.83	18.13	18.02	17.56	17.68	17.55	17.56
0.383	0.43	1.34	Q8BFQ8	Parkinson disease 7 domain-containing protein 1	Pddc1	2943	5	5	5	37.3	37.3	37.3	23.277	0.0000	168.96	14655000	114	14.46	16.20	16.35	16.18	15.57	15.46	15.63	14.82
0.483	0.43	1.34	Q8K370	Acyl-CoA dehydrogenase family member 10	Acad10	3693	19	19	19	21.6	21.6	21.6	118.98	0.0000	52.965	38262000	143	17.74	17.81	18.07	18.19	16.89	16.90	17.84	18.47
2.194	0.43	1.34	Q80VQ0;E9	Aldehyde dehydrogenase family 3 member B1	Aldh3b1	2855	22	22	16	59.6	59.6	42.3	52.292	0.0000	323.31	89522000	448	17.85	17.43	17.63	17.75	17.25	17.16	17.39	17.17
0.755	0.42	1.34	A3KGF7	1-phosphatidylinositol 4,5-bisphosphate phosphodiesterase beta-2	Plcb2	52	8	8	8	10.8	10.8	10.8	134.54	0.0000	57.611	4046400	65	13.86	13.25	14.44	14.24	13.61	13.33	13.71	13.44
1.776	0.42	1.34	Q3UEB3	Poly(U)-binding-splicing factor PUF60	Puf60	1954	15	15	15	46.3	46.3	46.3	60.248	0.0000	182	67210000	337	17.77	17.57	17.69	17.87	17.31	17.40	17.51	16.99
0.560	0.42	1.34	Q3TAA7	Serine/threonine-protein kinase 11-interacting protein	Stk11ip	1813	6	6	6	8.1	8.1	8.1	118	0.0000	27.489	3748400	89	13.62	13.58	14.20	13.77	13.55	13.69	13.82	12.41
2.343	0.42	1.34	Q9CRD2	ER membrane protein complex subunit 2	Emc2	4693	16	16	15	48.8	48.8	47.8	34.934	0.0000	112.05	41044000	251	16.94	16.63	16.88	16.89	16.24	16.56	16.43	16.41
2.179	0.42	1.34	Q921F4	Heterogeneous nuclear ribonucleoprotein L-like	Hnrnpll	4221	31	31	31	64.6	64.6	64.6	64.124	0.0000	289.8	94525000	709	18.19	17.82	18.19	18.26	17.67	17.75	17.72	17.62
1.713	0.42	1.34	P68368	Tubulin alpha-4A chain	Tuba4a	1516	33	9	9	58.7	16.5	16.5	49.924	0.0000	97.877	83518000	272	18.60	18.30	18.81	18.75	17.99	18.23	18.25	18.31
1.385	0.42	1.34	Q61543	Golgi apparatus protein 1	Glg1	2309	28	28	28	28.8	28.8	28.8	133.73	0.0000	65.819	38166000	350	16.57	16.06	16.26	16.10	16.11	15.74	15.89	15.56
1.645	0.42	1.34	Q8BYA0	Tubulin-specific chaperone D	Tbcd	3295	21	21	21	24.8	24.8	24.8	133.32	0.0000	223	38690000	382	16.75	16.21	16.66	16.69	16.20	16.27	16.18	15.97
1.839	0.42	1.34	Q8VDM4	26S proteasome non-ATPase regulatory subunit 2	Psmd2	3960	51	51	51	64.2	64.2	64.2	100.2	0.0000	323.31	193040000	995	19.07	18.82	18.91	18.99	18.50	18.25	18.55	18.79
2.406	0.42	1.34	P54729	NEDD8 ultimate buster 1	Nub1	1254	27	27	27	47.7	47.7	47.7	70.306	0.0000	270.59	58885000	389	17.32	17.10	17.28	17.23	16.57	16.86	16.93	16.87
0.947	0.42	1.34	Q9Z2A5	Arginyl-tRNA--protein transferase 1	Ate1	5836	12	12	12	27.9	27.9	27.9	59.146	0.0000	101.09	42147000	217	18.45	17.87	18.31	18.42	17.42	18.15	17.64	18.14
0.707	0.42	1.34	Q4JIM5	Abelson tyrosine-protein kinase 2	Abl2	2060	3	3	2	5.5	5.5	4.3	128.19	0.0000	38.145	2563000	59	13.77	14.18	14.14	13.79	13.13	13.31	14.33	13.43
0.834	0.42	1.34	Q8C7R4	Ubiquitin-like modifier-activating enzyme 6	Uba6	3414	25	25	24	29.4	29.4	27.6	117.96	0.0000	99.171	39601000	282	16.43	15.77	16.65	16.47	16.10	16.02	16.10	15.42
2.265	0.42	1.34	Q80WQ2	Protein VAC14 homolog	Vac14	2864	20	20	20	30.3	30.3	30.3	88.047	0.0000	148.04	51152000	386	17.25	17.05	17.32	17.21	16.74	16.78	16.62	17.01
1.302	0.42	1.34	Q8BX17	Gem-associated protein 5	Gemin5	3277	8	8	8	7.9	7.9	7.9	166.59	0.0000	34.591	11065000	69	15.84	15.65	15.70	15.99	15.34	15.17	15.82	15.17
0.774	0.42	1.34	Q60960	Importin subunit alpha-5;Importin subunit alpha-5, N-terminally processe	Kpna1	2228	15	11	11	39.2	32	32	60.182	0.0000	104	18461000	151	16.80	16.43	16.24	15.81	15.50	16.31	15.77	16.01
2.500	0.42	1.34	Q91XD6	Vacuolar protein-sorting-associated protein 36	Vps36	4142	19	19	19	58	58	58	43.735	0.0000	128.41	31495000	248	16.24	16.16	16.54	16.33	15.85	15.85	15.88	16.01
0.102	0.42	1.34	Q6ZQK0	Condensin-2 complex subunit D3	Ncapd3	2699	4	4	4	5.4	5.4	5.4	169.43	0.0002	5.5244	26334000	45	17.47	16.99	17.28	17.55	17.60	12.51	19.30	18.20
1.711	0.42	1.34	Q8BGB5	LIM domain-containing protein 2	Limd2	2973	10	9	9	59.4	53.9	53.9	14.237	0.0000	217.92	53571000	280	18.91	18.33	18.77	18.67	18.30	18.35	18.14	18.21
0.691	0.42	1.34	Q9Z0L8	Gamma-glutamyl hydrolase	Ggh	5775	5	5	5	19.9	19.9	19.9	35.469	0.0000	15.865	43028000	97	17.63	17.92	18.57	18.03	18.07	17.36	17.89	17.14
0.747	0.42	1.34	O09047	C3a anaphylatoxin chemotactic receptor	C3ar1	320	5	5	5	10.3	10.3	10.3	53.575	0.0000	13.357	17388000	65	16.60	16.41	15.99	16.52	15.83	15.34	16.37	16.31
0.490	0.42	1.34	Q811S7	Upstream-binding protein 1	Ubp1	2939	2	2	2	5.2	5.2	5.2	60.211	0.0000	7.6688	1628100	24	13.45	13.96	14.27	14.24	13.57	12.60	14.01	14.06
1.032	0.42	1.34	Q61093;Q8(	Cytochrome b-245 heavy chain	Cybb	2250	27	27	27	42.5	42.5	42.5	65.304	0.0000	323.31	334160000	716	19.81	19.62	19.27	19.34	19.55	18.75	18.99	19.07
1.511	0.42	1.34	Q9DC51	Guanine nucleotide-binding protein G(k) subunit alpha	Gnai3	5194	25	15	14	61.6	40.4	39.3	40.538	0.0000	147.46	32499000	331	16.12	15.79	15.98	16.21	15.50	15.63	15.93	15.37
0.701	0.42	1.34	Q9QZC2	Plexin-C1	Plxnc1	5591	7	7	7	5.1	5.1	5.1	176.47	0.0000	15.225	7000700	77	15.03	14.29	15.27	15.00	14.51	14.13	14.25	15.03
0.484	0.42	1.33	Q8R5J9	PRA1 family protein 3	Arl6ip5	3890	15	15	15	39.9	39.9	39.9	21.557	0.0000	126.72	36792000	180	18.11	16.95	17.48	16.90	16.76	16.86	16.44	17.71
0.267	0.42	1.33	Q8VDG3	Poly(A)-specific ribonuclease PARN	Parn	3951	6	6	6	16	16	16	71.558	0.0000	28.355	3986000	24	13.26	13.46	14.84	14.53	14.28	12.11	14.25	13.77
1.675	0.42	1.33	P50396	Rab GDP dissociation inhibitor alpha	Gdi1	1179	22	15	14	67.6	51.5	51.5	50.521	0.0000	142.36	21677000	170	16.21	16.26	16.41	16.01	15.72	16.12	15.75	15.65
0.549	0.42	1.33	Q04207	Transcription factor p65	Rela	1730	12	12	10	29.7	29.7	27.7	60.211	0.0000	117.18	24004000	144	16.87	16.46	16.53	16.65	16.42	16.57	16.65	15.20
0.238	0.42	1.33	O54781	SRSF protein kinase 2;SRSF protein kinase 2 N-terminal;SRSF protein	Srpk2	420	7	7	5	10.7	10.7	8.2	76.756	0.0000	10.886	3506200	25	14.94	14.33	15.37	12.51	13.19	14.11	14.65	13.54
1.321	0.42	1.33	Q9Z0G0	PDZ domain-containing protein GIPC1	Gipc1	5768	8	8	8	30.9	30.9	30.9	36.129	0.0000	25.665	12469000	158	15.31	15.36	15.33	15.66	15.00	15.28	14.60	15.12
0.945	0.41	1.33	Q99N57	RAF proto-oncogene serine/threonine-protein kinase	Raf1	4483	14	14	9	33.8	33.8	25.5	72.917	0.0000	154.12	25857000	168	15.94	16.29	16.92	16.50	15.87	15.91	16.26	15.94
1.872	0.41	1.33	Q6PDQ2	Chromodomain-helicase-DNA-binding protein 4	Chd4	2620	50	50	39	32.1	32.1	25.8	217.75	0.0000	323.31	72070000	674	16.71	16.17	16.42	16.44	16.01	16.03	16.12	15.91
0.911	0.41	1.33	P61290	Proteasome activator complex subunit 3	Psme3	1382	18	18	18	65	65	65	29.506	0.0000	82.102	59044000	237	16.91	17.22	17.91	17.56	16.85	17.19	17.06	16.85
1.860	0.41	1.33	P26043	Radixin	Rdx	917	57	40	37	75.1	57.3	54.9	68.542	0.0000	323.31	164250000	1047	17.41	17.03	17.18	17.56	16.90	16.85	16.93	16.84
1.287	0.41	1.33	P22315	Ferrochelatase, mitochondrial	Fech	873	9	9	9	36.9	36.9	36.9	47.13	0.0000	81.086	28311000	300	16.30	16.16	16.09	16.05	16.13	15.86	15.53	15.42
1.124	0.41	1.33	Q8QZZ7	EKC/KEOPS complex subunit Tprkb	Tprkb	3739	5	5	5	45.7	45.7	45.7	19.556	0.0000	80.674	6296200	81	14.63	15.00	15.39	15.00	14.47	14.53	14.93	14.43
0.447	0.41	1.33	Q09200	Beta-1,4 N-acetylgalactosaminyltransferase 1	B4galnt1	1772	11	11	11	31.3	31.3	31.3	59.211	0.0000	51.919	8674100	133	13.12	13.69	14.80	14.66	13.64	13.78	13.81	13.40
1.688	0.41	1.33	Q5SFM8	RNA-binding protein 27	Rbm27	2130	10	10	9	10.6	10.6	10.6	118.55	0.0000	36.18	4127700	48	14.60	14.76	14.50	14.43	13.86	14.28	14.15	14.37
0.389	0.41	1.33	Q91XD7	Cysteine-rich with EGF-like domain protein 1	Creld1	4143	3	3	3	7.6	7.6	7.6	45.717	0.0000	29.405	3595200	41	13.53	14.34	15.45	13.74	13.34	14.14	13.99	13.94
1.796	0.41	1.33	Q8R2Y8	Peptidyl-tRNA hydrolase 2, mitochondrial	Ptrh2	3826	8	8	8	65.7	65.7	65.7	19.526	0.0000	107.07	55619000	291	17.53	17.26	17.54	17.49	17.12	16.91	17.31	16.84
0.271	0.41	1.33	D3YZP9	Coiled-coil domain-containing protein 6	Ccdc6	211	5	5	5	15.6	15.6	15.6	52.938	0.0000	21.813	2858100	25	12.82	14.69	13.87	12.56	12.96	13.65	13.65	12.03
2.783	0.41	1.33	Q91VC3	Eukaryotic initiation factor 4A-III;Eukaryotic initiation factor 4A-III, N-tern	Eif4a3	4055	37	33	33	64.2	58.2	58.2	46.839	0.0000	323.31	136890000	776	18.41	18.11	18.22	18.40	17.91	17.81	17.88	17.90
1.443	0.41	1.33	Q3TC93	HCLS1-binding protein 3	Hs1bp3	1825	12	12	12	48.4	48.4	48.4	43.691	0.0000	95.237	16284000	206	15.69	15.33	15.62	15.59	15.15	15.32	15.34	14.78
2.213	0.41	1.33	O09110	Dual specificity mitogen-activated protein kinase kinase 3	Map2k3	323	19	19	16	63.1	63.1	56.2	39.296	0.0000	127.14	31001000	245	16.96	16.58	16.79	16.75	16.24	16.53	16.36	16.31
0.460	0.41	1.33	Q3TCX3	UPF0469 protein KIAA0907	Kiaa0907	1830	3	3	3	7	7	7	64.562	0.0000	7.2271	1697200	24	13.44	13.87	13.70	14.14	13.54	12.28	13.81	13.89
1.864	0.41	1.33	P08775	DNA-directed RNA polymerase II subunit RPB1	Polr2a	689	46	46	46	35.7	35.7	35.7	217.17	0.0000	323.31	49219000	523	16.33	16.09	16.56	16.29	15.92	16.00	16.01	15.71
1.519	0.41	1.33	P97494	Glutamate--cysteine ligase catalytic subunit	Gclc	1663	23	23	23	44.4	44.4	44.4	72.571	0.0000	116.9	39585000	333	16.15	15.90	16.52	16.10	15.64	15.64	15.91	15.82
1.430	0.41	1.33	Q9CZ13	Cytochrome b-c1 complex subunit 1, mitochondrial	Uqcrc1	4813	30	30	29	59.6	59.6	59.6	52.851	0.0000	323.31	179090000	846	18.07	18.17	18.63	18.24	17.88	17.87	18.09	17.64
1.217	0.41	1.33	P68033;P68	Actin, alpha cardiac muscle 1;Actin, alpha skeletal muscle;Actin, gamma	Actc1;Acta1;Actg2	1511	39	11	10	66.6	35.8	31.6	42.019	0.0000	323.31	785630000	489	21.64	21.84	21.67	22.16	21.41	21.09	21.73	21.44
0.252	0.41	1.33	Q9D958	Signal peptidase complex subunit 1	Spcs1	5096	3	3	3	13	13	13	18.186	0.0000	18.236	6345200	52	15.45	15.81	15.34	14.17	14.91	15.35	13.18	15.70
0.391	0.41	1.33	P59279	Ras-related protein Rab-2B	Rab2b	1333	8	2	2	42.1	13.9	13.9	24.198	0.0000	9.4268	1056600	28	12.79	13.60	13.61	13.02	12.58	14.04	12.22	12.55
2.145	0.41	1.33	Q8CAQ8	MICOS complex subunit Mic60	Immt	3430	58	58	58	69.6	69.6	69.6	83.899	0.0000	323.31	268290000	1595	18.38	17.96	18.38	18.28	17.83	17.83	17.92	17.80
1.222	0.41	1.33	P14901	Heme oxygenase 1	Hmox1	788	22	22	22	70.6	70.6	70.6	32.928	0.0000	192.86	128920000	472	17.70	18.19	18.38	18.29	17.62	17.78	17.96	17.57
1.258	0.41	1.33	P19258	Protein Mpv17	Mpv17	845	6	6	6	34.7	34.7	34.7	19.686	0.0000	153.21	14984000	131	15.68	15.48	15.47	15.78	15.16	15.28	14.81	15.54
1.331	0.41	1.32	P18654	Ribosomal protein S6 kinase alpha-3	Rps6ka3	840	32	19	18	50.7	35	33.2	83.693	0.0000	164.27	64341000	363	17.46	17.02	16.72	17.02	16.79	16.53	16.66	16.63
1.858	0.40	1.32	Q9D0I9	Arginine--tRNA ligase, cytoplasmic	Rars	4869	51	51	51	68.6	68.6	68.6	75.673	0.0000	323.31	316650000	1459	19.12	18.87	19.21	19.04	18.41	18.77	18.83	18.62
0.814	0.40	1.32	Q8VE47	Ubiquitin-like modifier-activating enzyme 5	Uba5	3986	12	12	12	42.9	42.9	42.9	44.789	0.0000	231.59	41472000	262	16.92	16.96	17.85	17.58	16.67	17.08	17.01	16.94
2.307	0.40	1.32	Q8R1B4	Eukaryotic translation initiation factor 3 subunit C	Eif3c	3783	54	54	54	52.9	52.9	52.9	105.53	0.0000	323.31	197520000	1189	18.02	17.61	17.91	17.93	17.42	17.54	17.43	17.46
1.296	0.40	1.32	Q922F4	Tubulin beta-6 chain	Tubb6	4254	30	17	15	72.5	55.3	50.1	50.09	0.0000	220.1	47549000	338	17.40	17.11	17.76	17.39	16.81	16.90	17.24	17.09
1.063	0.40	1.32	Q61699	Heat shock protein 105 kDa	Hsph1	2325	45	41	41	51.2	47.3	47.3	96.406	0.0000	323.31	63609000	606	16.11	15.91	16.66	16.56	15.83	15.75	15.95	16.11
0.202	0.40	1.32	Q9CX11	rRNA-processing protein UTP23 homolog	Utp23	4743	4	4	4	22.5	22.5	22.5	28.38	0.0000	13.071	4128600	26	13.97	15.39	14.19	14.10	15.14	13.05	15.33	12.52
2.094	0.40	1.32	P51660	Peroxisomal multifunctional enzyme type 2;(3R)-hydroxyacyl-CoA dehy	Hsd17b4	1199	39	39	39	58.8	58.8	58.8	79.481	0.0000	323.31	242740000	1093	18.45	18.21	18.46	18.60	18.10	17.87	18.16	17.97
0.246	0.40	1.32	Q9JKN1	Zinc transporter 7	Slc30a7	5455	3	3	3	10.8	10.8	10.8	41.789	0.0000	12.115	10562000	97	16.18	16.08	13.77	14.38	15.14	15.21	14.26	14.19
1.171	0.40	1.32	P52633	Signal transducer and transcription activator 6	Stat6	1222	24	24	24	49.6	49.6	49.6	93.496	0.0000	318.97	36951000	367	16.63	16.21	16.48	16.55	16.20	15.64	16.37	16.06
1.041	0.40	1.32	O35114	Lysosome membrane protein 2	Scarb2	337	9	9	9	25.9	25.9	25.9	54.043	0.0000	51.923	23010000	141	16.94	16.73	17.40	17.29	16.54	16.46	16.73	17.03
2.161	0.40	1.32	P54822	Adenylosuccinate lyase	Adsl	1260	29	29	28	67.4	67.4	66.1	54.866	0.0000	323.31	104140000	585	17.50	17.31	17.47	17.57	17.18	16.91	17.23	16.92
0.742	0.40	1.32	F7BJB9		Morc3	258	10	10	10	18.2	18.2	18.2	106.52	0.0000	62.062	6017600	77	15.29	14.89	14.78	14.36	14.78	14.69	14.22	14.02
1.675	0.40	1.32	P54775	26S protease regulatory subunit 6B	Psmc4	1257	26	26	26	81.1	81.1	81.1	47.408	0.0000	323.31	134060000	858	18.04	17.99	17.85	18.20	17.78	17.50	17.83	17.38
2.682	0.40	1.32	P21279;O70	Guanine nucleotide-binding protein G(q) subunit alpha	Gnaq	861	20	19	15	58.5	56.3	40.1	42.158	0.0000	239.18	64576000	479	17.01	16.84	17.00	16.72	16.56	16.41	16.49	16.50
1.871	0.40	1.32	Q8K411	Presequence protease, mitochondrial	Pitrm1	3711	42	42	42	56.3	56.3	56.3	117.37	0.0000	323.31	125970000	921	17.08	17.04	17.40	17.44	16.72	16.80	16.92	16.92
0.899	0.40	1.32	Q8K3W0	BRCA1-A complex subunit BRE	Bre	3705	11	11	11	33.7	33.7	33.7	43.544	0.0000	82.551	22683000	136	16.42	16.28	17.06	16.59	15.87	16.48	16.39	16.02
1.333	0.40	1.32	P50544	Very long-chain specific acyl-CoA dehydrogenase, mitochondrial	Acadvl	1187	30	30	30	61.4	61.4	61.4	70.875	0.0000	323.31	105580000	792	17.35	17.34	17.70	17.88	17.06	16.99	17.27	17.35
0.400	0.40	1.32	Q9QZM0	Ubiquilin-2	Ubqln2	5601	5	3	3	9.6	5.6	5.6	67.35	0.0000	12.174	2074700	23	12.77	14.53	14.01	14.00	13.11	13.15	14.10	13.37
1.353	0.40	1.32	Q6PAR5	GTPase-activating protein and VPS9 domain-containing protein 1	Gapvd1	2599	28	28	28	23.3	23.3	23.3	162.4	0.0000	91.637	53583000	326	18.05	17.67	18.29	18.26	17.50	17.76	17.77	17.65
0.836	0.40	1.32	Q9DBF1	Alpha-aminoadipic semialdehyde dehydrogenase	Aldh7a1	5148	12	12	12	39.9	39.9	39.9	58.861	0.0000	50.117	10305000	108	15.27	14.70	15.66	15.21	14.60	14.57	15.04	15.05
1.858	0.40	1.32	P63005	Platelet-activating factor acetylhydrolase IB subunit alpha	Pafah1b1	1478	27	27	27	65.4	65.4	65.4	46.67	0.0000	169.1	82874000	549	17.94	17.58	17.79	18.08	17.51	17.52	17.33	17.43
0.361	0.40	1.32	Q8VBW1	Sodium- and chloride-dependent creatine transporter 1	Slc6a8	3899	3	3	3	5.9	5.9	5.9	70.999	0.0000	11.376	1433500	22	13.70	13.19	14.19	14.70	14.07	13.34	14.13	12.65
0.833	0.40	1.32	Q5XKN4	Protein jagunal homolog 1	Jagn1	2174	4	4	4	14.8	14.8	14.8	21.102	0.0000	14.132	14069000	126	16.00	15.22	16.22	15.79	15.40	15.13	15.48	15.63
2.152	0.40	1.32	P42208	Septin-2	Sep.02	1074	24	24	24	71.2	71.2	71.2	41.525	0.0000	323.31	195720000	751	19.40	19.03	19.45	19.31	18.98	18.82	18.88	18.93
1.244	0.40	1.32	Q8C2K1	Differentially expressed in FDCP 6	Def6	3371	22	22	22	33.7	33.7	33.7	73.453	0.0000	48.214	18645000	204	15.35	14.69	15.13	15.40	14.80	14.85	14.68	14.66
0.569	0.40	1.32	Q8R3L2	Transcription factor 25	Tcf25	3850	3	3	3	7.2	7.2	7.2	76.684	0.0000	7.3635	1862600	19	13.86	13.87	13.46	14.00	13.81	13.02	12.76	14.02
0.763	0.39	1.31	Q9D7H3	RNA 3-terminal phosphate cyclase	RtcA	5030	8	8	8	32.2	32.2	32.2	39.254	0.0000	51.267	6760700	81	14.63	14.56	14.92	15.17	14.52	14.15	14.04	14.98
0.321	0.39	1.31	Q99JP6	Homer protein homolog 3	Homer3	4343	7	7	7	26.4	26.4	26.4	39.694	0.0000	13.478	3124000	29	14.82	14.24	14.43	13.19	14.18	13.76	14.46	12.71
1.476	0.39	1.31	Q6PDG5	SWI/SNF complex subunit SMARCC2	Smarcc2	2611	27	27	18	26.5	26.5	20.4	132.6	0.0000	248.55	53120000	488	17.17	16.74	17.19	17.22	16.76	16.88	16.65	16.46
2.794	0.39	1.31	P60843	Eukaryotic initiation factor 4A-I	Eif4a1	1362	38	38	22	80.3	80.3	51.5	46.153	0.0000	323.31	883380000	2132	20.24	20.22	20.21	20.42	19.82	19.76	19.98	19.95
0.536	0.39	1.31	Q9JI90	E3 ubiquitin-protein ligase RNF14	Rnf14	5391	6	6	6	18.6	18.6	18.6	54.926	0.0000	26.98	3132900	33	14.41	13.87	14.90	13.99	13.51	13.67	13.80	14.62
0.158	0.39	1.31	Q9JI39	ATP-binding cassette sub-family B member 10, mitochondrial	Abcb10	5385	5	5	5	11.7	11.7	11.7	77.187	0.0000	20.417	4017700	33	14.22	12.21	15.34	15.86	14.71	13.27	15.06	13.03
2.462	0.39	1.31	Q3UPL0	Protein transport protein Sec31A	Sec31a	2015	49	49	49	39.2	39.2	39.2	133.57	0.0000	323.31	143540000	1058	17.72	17.47	17.41	17.72	17.19	17.12	17.22	17.22
1.095	0.39	1.31	P56546	C-terminal-binding protein 2	Ctbp2	1290	15	9	8	29.9	22.5	20.9	48.956	0.0000	60.155	18091000	123	16.65	16.35	15.91	16.27	16.16	15.86	15.95	15.63
1.292	0.39	1.31	Q8C1A5	Thimet oligopeptidase	Thop1	3362	27	27	27	47	47	47	78.025	0.0000	130.69	44336000	377	16.02	15.87	16.49	16.21	15.81	15.90	15.82	15.49
1.228	0.39	1.31	Q8BU33	Acetolactate synthase-like protein	Ilvbl	3220	18	18	18	43.2	43.2	43.2	68.155	0.0000	316.56	51536000	402	16.73	16.49	17.04	17.11	16.39	16.46	16.69	16.26
1.050	0.39	1.31	Q80Y81	Zinc phosphodiesterase ELAC protein 2	Elac2	2896	15	15	15	22.1	22.1	22.1	92.718	0.0000	70.363	13801000	118	15.29	15.02	15.56	15.52	15.07	14.96	15.24	14.54
1.069	0.39	1.31	P49443	Protein phosphatase 1A	Ppm1a	1163	7	4	4	22	13.9	13.9	42.432	0.0000	21.232	6657300	88	15.05	15.29	15.47	15.58	15.05	14.74	15.34	14.68

1.332	0.39	1.31	O08528	Hexokinase-2	Hk2	267	53	48	48	55.7	53.7	53.7	102.53	0.0000	323.31	153570000	1105	17.61	17.33	17.74	17.85	17.23	17.05	17.55	17.13
0.542	0.39	1.31	P43346	Deoxycytidine kinase	Dck	1091	8	8	8	45.8	45.8	45.8	30.367	0.0000	33.115	17768000	127	16.24	16.10	15.91	15.86	16.00	16.24	15.53	14.77
1.321	0.39	1.31	P46467;Q8E	Vacuolar protein sorting-associated protein 4B	Vps4b	1105	27	27	21	60.8	60.8	50.2	49.419	0.0000	323.31	106920000	576	17.46	17.33	17.81	17.94	17.13	17.17	17.42	17.26
0.612	0.39	1.31	Q8C5W3	Tubulin-specific chaperone cofactor E-like protein	Tbcel	3402	8	8	8	30.9	30.9	30.9	48.031	0.0000	28.095	7441000	91	14.56	15.52	15.55	15.84	15.32	14.97	14.81	14.81
4.197	0.39	1.31	P50247	Adenosylhomocysteinase	Ahcy	1177	29	29	29	65.3	65.3	65.3	47.688	0.0000	323.31	156610000	832	18.38	18.48	18.54	18.42	18.02	18.05	18.09	18.11
0.525	0.39	1.31	Q9Z1Q2;Q8	Abhydrolase domain-containing protein 16A	Abhd16a	5817	16	16	16	36	36	36	63.085	0.0000	83.382	19348000	178	14.83	14.26	15.56	14.48	14.38	14.08	14.94	14.18
0.889	0.39	1.31	O88545	COP9 signalosome complex subunit 6	Cops6	545	12	12	12	49.4	49.4	49.4	35.88	0.0000	193.01	33157000	271	16.82	16.67	16.05	16.45	16.34	15.85	16.37	15.87
1.645	0.39	1.31	Q9DCN2	NADH-cytochrome b5 reductase 3;NADH-cytochrome b5 reductase 3 m	Cyb5r3	5224	16	16	16	57.5	57.5	57.5	34.127	0.0000	323.31	252800000	597	20.08	19.70	20.04	19.91	19.48	19.32	19.63	19.76
0.804	0.39	1.31	Q9DBE0	Cysteine sulfinic acid decarboxylase	Csad	5145	11	11	11	42	42	42	55.144	0.0000	94.076	8738700	76	15.59	14.91	14.85	15.00	15.06	14.29	14.87	14.58
2.137	0.39	1.31	Q9R062	Glycogenin-1	Gyg1	5617	12	12	12	42	42	42	37.402	0.0000	264.98	60327000	313	17.87	17.78	17.88	17.68	17.24	17.32	17.62	17.48
1.924	0.39	1.31	P53026	60S ribosomal protein L10a	Rpl10a	1229	16	16	16	49.3	49.3	49.3	24.916	0.0000	133.04	280960000	650	19.45	19.02	19.40	19.44	18.89	18.97	19.04	18.86
0.803	0.39	1.31	Q80TA6	Myotubularin-related protein 12	Mtmr12	2800	13	13	13	24.5	24.5	24.5	85.515	0.0000	37.82	6398200	58	14.86	14.20	14.26	14.44	14.12	13.53	14.43	14.12
1.751	0.39	1.31	Q91YR1	Twinfilin-1	Twf1	4173	21	18	18	50.3	46.3	46.3	40.079	0.0000	323.31	124220000	527	17.72	17.79	17.78	17.92	17.08	17.53	17.54	17.51
0.383	0.39	1.31	Q76LS9	Protein FAM63A	Fam63a	2718	7	7	5	17.5	17.5	12.8	51.225	0.0000	9.8926	12034000	89	15.52	14.68	16.51	16.16	15.26	14.91	15.77	15.39
1.552	0.38	1.31	Q8BH74	Nuclear pore complex protein Nup107	Nup107	3020	12	12	12	20.7	20.7	20.7	106.72	0.0000	219.32	15221000	148	16.44	16.04	16.46	16.31	15.84	16.18	15.77	15.93
1.531	0.38	1.31	Q921L5	Conserved oligomeric Golgi complex subunit 2	Cog2	4231	19	19	19	37.6	37.6	37.6	82.039	0.0000	171.24	19232000	126	15.88	16.03	16.01	16.00	15.73	15.28	15.88	15.49
0.411	0.38	1.30	Q9D786	HAUS augmin-like complex subunit 5	Haus5	5022	8	8	7	19.4	19.4	18.1	69.571	0.0000	16.738	7015600	40	15.45	15.28	15.73	14.57	14.94	15.54	15.04	13.97
1.521	0.38	1.30	Q9R1C7	Pre-mRNA-processing factor 40 homolog A	Prpf40a	5650	24	24	24	30.7	30.7	30.7	108.48	0.0000	149.37	33266000	378	15.92	15.86	15.60	16.13	15.25	15.61	15.57	15.54
0.875	0.38	1.30	Q99LC9	Peroxisome assembly factor 2	Pex6	4419	3	3	3	7	7	7	104.55	0.0000	47.747	2056300	45	13.76	13.74	13.30	14.29	13.57	13.28	13.20	13.51
1.026	0.38	1.30	Q9WVA3	Mitotic checkpoint protein BUB3	Bub3	5743	17	17	16	42.9	42.9	42.6	36.954	0.0000	173.92	51604000	310	17.21	16.96	17.76	17.39	17.10	17.01	17.02	16.67
1.554	0.38	1.30	Q6GV12	3-ketodihydrosphingosine reductase	Kdsr	2516	4	4	4	14.8	14.8	14.8	35.955	0.0000	9.8791	12791000	69	15.67	15.40	15.55	15.91	15.15	15.16	15.48	15.20
1.159	0.38	1.30	Q9R1T2	SUMO-activating enzyme subunit 1;SUMO-activating enzyme subunit 1	Sae1	5663	23	23	22	66.6	66.6	66.3	38.62	0.0000	176.25	55197000	389	16.63	16.36	16.13	15.96	16.05	16.00	15.86	15.63
0.400	0.38	1.30	Q3U1V6	Ubiquitin-conjugating enzyme E2 variant 3	Uevld	1905	9	9	9	31.8	31.8	31.8	51.681	0.0000	26.248	4092200	42	15.12	14.12	15.18	15.04	15.42	14.32	14.39	13.81
1.226	0.38	1.30	P98083;Q61	SHC-transforming protein 1	Shc1	1686	6	6	5	18	18	15.9	62.607	0.0000	60.045	10775000	98	16.21	15.85	16.51	16.37	15.66	15.97	16.01	15.77
1.360	0.38	1.30	Q9CWK8	Sorting nexin-2	Snx2	4719	39	39	37	58.2	58.2	54.1	58.47	0.0000	323.31	362350000	1251	19.15	18.79	18.99	18.86	18.78	18.74	18.53	18.23
1.294	0.38	1.30	Q9Z1G3;Q9	V-type proton ATPase subunit C 1	Atp6v1c1	5807	39	39	39	87.4	87.4	87.4	43.887	0.0000	323.31	230220000	954	18.23	18.29	18.17	18.38	17.62	17.64	18.11	18.18
0.236	0.38	1.30	Q64281;Q61	Leukocyte immunoglobulin-like receptor subfamily B member 4	Lilrb4	2426	5	5	5	26	26	26	37.544	0.0000	26.155	11635000	74	15.86	16.60	16.11	16.14	16.56	16.30	16.41	13.91
1.133	0.38	1.30	Q9D024	Coiled-coil domain-containing protein 47	Ccdc47	4851	19	19	19	40.8	40.8	40.8	55.843	0.0000	122.81	66408000	353	17.64	17.46	17.68	17.83	16.86	17.25	17.38	17.61
1.483	0.38	1.30	Q8K1J6	CCA tRNA nucleotidyltransferase 1, mitochondrial	Trnt1	3629	18	18	18	50.9	50.9	50.9	49.895	0.0000	111.47	33020000	311	16.19	15.75	16.33	16.31	15.74	15.84	15.78	15.72
1.088	0.38	1.30	Q9Z0Y1	Dynactin subunit 3	Dctn3	5792	13	13	12	61.8	61.8	61.8	20.978	0.0000	68.58	11096000	100	15.32	14.95	15.00	14.98	14.67	14.41	14.53	15.13
0.796	0.38	1.30	Q8CG50	Ras-related protein Rab-43	Rab43	3498	7	6	6	43.8	37.6	37.6	23.263	0.0000	31.654	6989300	60	15.63	14.99	15.20	15.25	15.25	15.02	14.96	14.33
1.470	0.38	1.30	Q9CX34	Suppressor of G2 allele of SKP1 homolog	Sugt1	4746	15	15	15	55.7	55.7	55.7	38.158	0.0000	220.1	40160000	323	17.00	17.16	17.08	17.07	16.52	17.09	16.52	16.67
0.648	0.38	1.30	Q8R0F6	Integrin-linked kinase-associated serine/threonine phosphatase 2C	Ilkap	3756	9	9	9	32.9	32.9	32.9	42.774	0.0000	19.189	6223000	63	14.64	14.42	14.97	14.99	15.05	14.38	13.91	14.17
1.806	0.38	1.30	B2RXS4	Plexin-B2	Plxnb2	86	48	48	47	30.8	30.8	30.2	206.23	0.0000	323.31	141470000	833	18.03	17.71	17.69	17.80	17.58	17.27	17.56	17.32
0.554	0.38	1.30	Q9JLN9	Serine/threonine-protein kinase mTOR	Mtor	5486	26	26	26	15.6	15.6	15.6	288.79	0.0000	137.44	21247000	250	15.30	14.54	16.02	15.48	15.02	14.99	15.10	14.72
1.037	0.38	1.30	P14576	Signal recognition particle 54 kDa protein	Srp54	782	26	26	26	62.5	62.5	62.5	55.72	0.0000	260.59	66627000	462	17.51	17.06	17.49	17.46	16.99	17.25	17.20	16.57
1.053	0.38	1.30	Q8CG47	Structural maintenance of chromosomes protein 4	Smc4	3496	43	43	43	39.6	39.6	39.6	146.89	0.0000	213.12	43006000	518	16.64	15.94	16.52	16.63	15.96	16.30	15.96	16.01
0.407	0.38	1.30	Q8R317	Ubiquilin-1	Ubqln1	3830	7	7	5	25.9	25.9	21.6	61.976	0.0000	207.25	11255000	123	15.14	15.75	15.77	15.11	14.07	15.62	15.58	15.00
1.093	0.37	1.30	P28740	Kinesin-like protein KIF2A	Kif2a	960	19	19	18	30.6	30.6	29.4	79.755	0.0000	77.417	16098000	231	15.16	14.64	15.38	15.26	14.55	14.80	14.68	14.90
1.488	0.37	1.30	Q8BYK6	YTH domain-containing family protein 3	Ythdf3	3302	15	15	10	32	32	25	63.961	0.0000	48.973	24954000	185	16.18	16.22	16.22	16.17	15.77	15.65	15.66	16.22
1.721	0.37	1.30	Q8JZN5	Acyl-CoA dehydrogenase family member 9, mitochondrial	Acad9	3575	18	18	18	41.8	41.8	41.8	68.721	0.0000	224.74	34406000	352	16.72	16.34	16.56	16.86	16.32	16.12	16.26	16.28
1.590	0.37	1.30	Q9CW03	Structural maintenance of chromosomes protein 3	Smc3	4708	69	69	69	57.4	57.4	57.4	141.55	0.0000	323.31	107730000	975	17.03	16.71	16.94	17.09	16.59	16.78	16.59	16.31
0.869	0.37	1.30	Q9R059	Four and a half LIM domains protein 3	Fhl3	5614	7	7	7	31.8	31.8	31.8	31.794	0.0000	11.865	10992000	65	15.90	16.02	15.19	15.76	15.53	15.36	15.47	15.02
1.740	0.37	1.30	O89023	Tripeptidyl-peptidase 1	Tpp1	596	8	8	8	23.1	23.1	23.1	61.341	0.0000	94.805	73061000	235	18.90	18.56	19.05	19.02	18.53	18.52	18.43	18.57
1.720	0.37	1.30	Q99JY0	Trifunctional enzyme subunit beta, mitochondrial;3-ketoacyl-CoA thiolas	Hadhb	4356	35	35	35	66.7	66.7	66.7	51.386	0.0000	305.99	193020000	768	18.53	18.01	18.27	18.15	17.86	17.97	17.88	17.77
1.134	0.37	1.30	Q8K0C9	GDP-mannose 4,6 dehydratase	Gmds	3603	16	16	16	54.3	54.3	54.3	41.984	0.0000	144.16	27421000	240	16.31	16.19	16.11	15.72	16.00	15.68	15.72	15.44
0.876	0.37	1.30	Q80ZS3	28S ribosomal protein S26, mitochondrial	Mrps26	2920	6	6	6	37	37	37	23.443	0.0000	25.695	6895800	71	15.08	14.67	15.47	15.41	15.11	14.73	14.64	14.66
0.718	0.37	1.30	Q9Z2G6	Protein sel-1 homolog 1	Sel1l	5851	13	13	13	23.2	23.2	23.2	88.339	0.0000	127.89	30930000	219	16.47	16.95	16.20	17.14	16.66	16.10	16.39	16.11
0.223	0.37	1.29	Q80W47	WD repeat domain phosphoinositide-interacting protein 2	Wipi2	2857	4	4	4	19.8	19.8	19.8	48.477	0.0000	129.38	4378100	30	13.50	14.89	15.91	15.12	14.66	13.69	13.92	15.67
1.871	0.37	1.29	P80314	T-complex protein 1 subunit beta	Cct2	1591	62	62	62	91	91	91	57.477	0.0000	323.31	655340000	2289	19.71	19.50	19.86	19.88	19.38	19.47	19.42	19.19
1.301	0.37	1.29	Q8C863	E3 ubiquitin-protein ligase Itchy	Itch	3421	8	8	6	9.6	9.6	6.8	98.992	0.0000	13.775	14759000	108	16.55	16.20	16.58	16.04	16.05	15.77	16.10	15.96
1.251	0.37	1.29	O70475	UDP-glucose 6-dehydrogenase	Ugdh	503	22	22	22	58.6	58.6	58.6	54.831	0.0000	224	49581000	433	16.82	16.27	16.23	16.61	16.10	15.94	16.29	16.10
0.294	0.37	1.29	O35900	U6 snRNA-associated Sm-like protein LSm2	Lsm2	411	4	4	4	49.5	49.5	49.5	10.834	0.0000	33.215	18895000	121	17.42	15.95	16.74	17.09	17.13	17.08	16.15	15.35
3.846	0.37	1.29	Q80X50	Ubiquitin-associated protein 2-like	Ubap2l	2870	23	23	23	29.9	29.9	29.9	116.8	0.0000	301.15	37911000	412	16.44	16.49	16.34	16.52	16.06	16.07	16.06	16.12
0.119	0.37	1.29	P14106	Complement C1q subcomponent subunit B	C1qb	772	6	6	6	31.6	31.6	31.6	26.717	0.0000	43.662	10015000	51	17.54	16.23	12.98	16.62	16.50	16.02	13.73	15.63
1.685	0.37	1.29	Q921F2	TAR DNA-binding protein 43	Tardbp	4220	18	18	18	54.1	54.1	54.1	44.547	0.0000	323.31	119650000	495	18.46	18.13	18.04	18.17	18.04	17.67	17.83	17.79
1.144	0.37	1.29	Q58A65	C-Jun-amino-terminal kinase-interacting protein 4	Spag9	2093	38	38	30	33	33	28.4	146.22	0.0000	290	48435000	472	16.36	15.83	16.49	16.36	15.72	15.82	15.91	16.12
1.302	0.37	1.29	P97807	Fumarate hydratase, mitochondrial	Fh	1673	27	27	27	70.8	70.8	70.8	54.356	0.0000	323.31	188160000	762	19.04	18.73	18.72	18.78	18.43	18.09	18.66	18.62
0.811	0.37	1.29	P97371	Proteasome activator complex subunit 1	Psme1	1636	21	20	20	72.3	67.9	67.9	28.673	0.0000	193.22	53621000	446	16.74	16.90	16.00	16.93	16.43	16.14	16.30	16.21
0.884	0.37	1.29	Q80SY3	V-type proton ATPase subunit d 2	Atp6v0d2	2796	10	10	10	42.3	42.3	42.3	40.493	0.0000	89.893	18667000	115	16.87	16.73	16.98	16.80	15.99	16.29	16.85	16.78
0.246	0.37	1.29	Q9CX13	Protein cornichon homolog 4	Cnih4	4744	1	1	1	14.4	14.4	14.4	16.089	0.0000	9.7567	2783400	27	14.20	12.70	13.88	14.43	13.29	14.15	12.15	14.14
0.959	0.37	1.29	Q9EQG9	Collagen type IV alpha-3-binding protein	Col4a3bp	5276	20	20	20	40.7	40.7	40.7	71.11	0.0000	121.86	25003000	287	16.11	15.21	15.60	15.73	15.41	15.11	15.32	15.35
1.422	0.37	1.29	Q8BFS6	Serine/threonine-protein phosphatase CPPED1	Cpped1	2946	7	7	6	31.7	31.7	31.1	35.247	0.0000	47.133	10432000	120	15.11	15.05	15.52	15.48	14.83	15.10	14.91	14.84
2.055	0.37	1.29	Q9ES28	Rho guanine nucleotide exchange factor 7	Arhgef7	5324	27	27	24	40.7	40.7	38.1	97.055	0.0000	113.82	54932000	416	16.67	16.44	16.57	16.80	16.22	16.28	16.12	16.40
0.115	0.37	1.29	Q64339	Ubiquitin-like protein ISG15	Isg15	2433	4	4	4	26.1	26.1	26.1	17.897	0.0000	73.059	6973500	71	15.69	15.68	11.63	12.36	13.42	13.01	14.91	12.55
0.537	0.37	1.29	Q922Q9	Chitinase domain-containing protein 1	Chid1	4272	10	10	10	30	30	30	44.905	0.0000	30.398	13902000	133	16.04	15.60	15.08	15.77	15.12	14.94	14.98	15.98
1.060	0.37	1.29	Q8R4K2	Interleukin-1 receptor-associated kinase 4	Irak4	3870	13	13	13	29.6	29.6	29.6	50.871	0.0000	48.823	22798000	226	15.67	15.53	16.25	16.09	15.47	15.38	15.66	15.56
1.511	0.37	1.29	Q8K2T1	NmrA-like family domain-containing protein 1	Nmral1	3671	12	12	12	56.3	56.3	56.3	34.376	0.0000	137.12	25442000	218	16.19	16.25	16.53	16.34	15.83	16.28	15.92	15.81
0.159	0.37	1.29	Q3TVP5	Inactive ubiquitin thioesterase FAM105A	Fam105a	1879	5	5	5	11	11	11	41.576	0.0000	8.311	5101200	23	15.02	15.18	12.92	14.95	15.57	13.57	15.03	12.44
0.563	0.37	1.29	Q8JZM7	Parafibromin	Cdc73	3574	16	16	16	32.8	32.8	32.8	60.576	0.0000	61.345	19060000	181	15.67	15.53	16.00	16.32	15.17	16.10	15.04	15.74
1.274	0.37	1.29	Q9JII6	Alcohol dehydrogenase [NADP(+)]	Akr1a1	5402	35	35	35	94.5	94.5	94.5	36.586	0.0000	323.31	1318500000	2017	20.83	20.86	21.22	21.14	20.54	20.41	20.94	20.71
0.271	0.37	1.29	Q14DL3	Leucine-rich repeat and IQ domain-containing protein 3	Lrriq3	1791	3	3	3	6.6	6.6	6.6	75.363	0.0041	2.4409	11538000	58	16.50	16.23	15.46	16.02	16.11	15.92	16.52	14.20
0.698	0.36	1.29	Q9CZX0	Elongator complex protein 3	Elp3	4844	9	9	9	22.5	22.5	22.5	62.384	0.0000	25.11	10013000	106	15.79	15.43	15.69	15.62	15.71	14.93	15.66	14.77
1.097	0.36	1.29	P49138	MAP kinase-activated protein kinase 2	Mapkapk2	1156	26	26	25	61.4	61.4	58.3	44.049	0.0000	155.09	73545000	420	17.62	17.34	17.36	17.40	17.18	17.28	16.59	17.22
0.576	0.36	1.29	Q8BGH2	Sorting and assembly machinery component 50 homolog	Samm50	2983	19	19	19	50.1	50.1	50.1	51.863	0.0000	69.963	39169000	298	16.12	16.00	16.14	15.97	15.75	14.85	16.18	15.98
1.380	0.36	1.29	Q91V09	WD repeat-containing protein 13	Wdr13	4042	6	6	6	15.3	15.3	15.3	53.663	0.0000	17.733	5363200	98	14.93	14.50	14.87	14.55	14.53	14.40	14.35	14.10
1.111	0.36	1.29	O54782	Epididymis-specific alpha-mannosidase	Man2b2	421	27	27	27	29.7	29.7	29.7	115.61	0.0000	280.68	68944000	533	16.92	16.64	17.17	17.00	16.48	16.28	16.90	16.62
1.955	0.36	1.29	Q8R2K1	Fucose mutarotase	Fuom	3807	3	3	3	33.3	33.3	33.3	16.805	0.0000	22.478	5386500	86	15.01	15.14	15.27	14.92	14.69	14.74	14.88	14.56
1.756	0.36	1.29	Q9DC23	DnaJ homolog subfamily C member 10	Dnajc10	5187	18	18	18	26.7	26.7	26.7	90.582	0.0000	157.13	28081000	301	16.01	15.81	16.16	16.04	15.88	15.52	15.67	15.52
1.381	0.36	1.29	Q8JZU2	Tricarboxylate transport protein, mitochondrial	Slc25a1	3583	11	11	11	28.3	28.3	28.3	33.931	0.0000	27.184	33388000	244	16.70	16.41	16.32	16.58	16.35	15.85	16.10	16.27
0.527	0.36	1.29	Q6NVF4	DNA helicase B	Helb	2534	6	6	6	10.8	10.8	10.8	121.47	0.0000	13.057	6188800	58	15.75	15.27	16.13	15.39	15.05	15.57	15.79	14.68
0.267	0.36	1.28	Q8VDM1	Zinc finger CCCH-type with G patch domain-containing protein	Zgpat	3959	2	2	2	7.4	7.4	7.4	56.41	0.0004	4.6588	994620	6	13.42	13.49	14.11	11.67	12.59	13.07	13.19	12.41
0.620	0.36	1.28	P34960	Macrophage metalloelastase	Mmp12	1009	6	6	6	15.6	15.6	15.6	54.97	0.0000	11.661	4924500	46	14.49	13.97	13.74	13.64	13.15	13.47	13.67	14.11
0.771	0.36	1.28	Q64373	Bcl-2-like protein 1	Bcl2l1	2437	3	3	3	13.7	13.7	13.7	26.132	0.0004	4.8422	4766600	46	14.79	14.41	15.17	14.16	14.40	14.26	14.10	14.33
0.586	0.36	1.28	Q9CQW9	Interferon-induced transmembrane protein 3	Ifitm3	4644	6	6	5	48.9	48.9	43.1	14.954	0.0000	205.56	151520000	393	18.98	18.48	18.55	18.44	18.64	18.75	17.91	17.70
1.003	0.36	1.28	Q61136	Serine/threonine-protein kinase PRP4 homolog	Prpf4b	2257	15	15	15	18	18	18	116.97	0.0000	80.534	20018000	218	16.74	16.26	16.47	16.64	15.92	16.61	16.04	16.11
1.067	0.36	1.28	Q9DC69	NADH dehydrogenase [ubiquinone] 1 alpha subcomplex subunit 9, mito	Ndufa9	5199	23	23	23	66	66	66	42.525	0.0000	244.89	51771000	420	17.46	16.80	16.91	16.73	16.76	16.64	16.49	16.58
1.374	0.36	1.28	P62880	Guanine nucleotide-binding protein G(I)/G(S)/G(T) subunit beta-2	Gnb2	1462	20	12	10	65.3	44.1	40.3	37.331	0.0000	323.31	307080000	533	20.82	20.57	20.91	20.60	20.40	20.63	20.33	20.09
1.648	0.36	1.28	Q4LDD4	Arf-GAP with Rho-GAP domain, ANK repeat and PH domain-containing	Arap1	2063	53	53	53	43.6	43.6	43.6	162.27	0.0000	323.31	126300000	871	17.17	16.98	17.41	17.35	16.69	16.99	16.93	16.85
1.073	0.36	1.28	P22892	AP-1 complex subunit gamma-1	Ap1g1	877	16	16	16	24.6	24.6	24.6	91.349	0.0000	117.82	41284000	340	16.97	16.31	16.34	16.71	16.19	16.11	16.43	16.15
1.196	0.36	1.28	P55258;P62	Ras-related protein Rab-8A	Rab8a	1270	19	14	14	60.9	51.7	51.7	23.668	0.0000	79.579	61220000	304	17.99	17.36	17.54	17.89	17.26	17.26	17.49	17.33
1.222	0.36	1.28	Q99KE1;Q8	NAD-dependent malic enzyme, mitochondrial	Me2	4372	34	34	34	77.1	77.1	77.1	65.798	0.0000	323.31	126280000	725	17.56	17.40	18.02	17.94	17.35	17.50	17.32	17.31
1.899	0.36	1.28	P05213	Tubulin alpha-1B chain	Tuba1b	651	36	36	0	72.7	72.7	0	50.151	0.0000	323.31	2761500000	3433	22.41	22.36	22.40	22.38	22.17	21.89	22.22	21.82
0.424	0.36	1.28	Q9Z0H7	B-cell lymphoma/leukemia 10	Bcl10	5772	4	4	3	35.2	35.2	24.5	25.948	0.0000	6.5683	2346100	28	13.57	13.22	13.15	14.54	13.41	13.39	13.57	12.70
0.148	0.36	1.28	Q9ER81	Torsin-1A-interacting protein 2, isoform IFRG15	Tor1aip2	5299	2	2	2	25.2	25.2	25.2	15.278	0.0000	10.757	4492700	66	15.03	15.29	14.86	12.21	14.99	12.34	14.39	14.25
2.225	0.36	1.28	O08529	Calpain-2 catalytic subunit	Capn2	268	53	53	52	72.6	72.6	71.7	79.871	0.0000	323.31	244720000	1209	18.47	18.23	18.09	18.35	17.91	17.98	17.97	17.85
1.359	0.36	1.28	P99026	Proteasome subunit beta type-4	Psmb4	1692	13	13	13	56.8	56.8	56.8	29.116	0.0000	323.31	207710000	520	18.96	18.75	19.21	19.23	18.92	18.61	18.57	18.63
1.148	0.36	1.28	P49718	DNA replication licensing factor MCM5	Mcm5	1169	37	37	36	56.7	56.7	56.5	82.406	0.0000	275.41	82540000	561	16.57	16.22	16.76	16.87	16.19	16.41	16.34	16.06
0.323	0.36	1.28	Q9CWS4	Integrator complex subunit 11	Cpsf3l	4728	5	5	5	13.2	13.2	13.2	67.843	0.0000	21.505	3949500	29	14.22	13.63	14.00	14.68	14.70	13.64	14.05	12.73
0.858	0.35	1.28	Q9D0L7	Armadillo repeat-containing protein 10	Armc10	4875	10	10	10	40.2	40.2	40.2	33.31	0.0000	212.97	46769000	276	17.34	16.97	17.85	17.28	16.98	16.74	17.13	17.19
1.455	0.35	1.28	Q7TSE6	Serine/threonine-protein kinase 38-like	Stk38l	2781	7	4	4	13.6	9.5	9.5	53.771	0.0000	13.945	2615700	32	14.41	14.21	14.67	14.48	14.21	13.87	14.25	14.02
1.767	0.35	1.28	Q9CXW4	60S ribosomal protein L11	Rpl11	4772	17	17	17	61.2	61.2	61.2	20.252	0.0000	171.44	430130000	530	20.69	20.27	20.69	20.46	20.09	20.27	20.20	20.13
1.264	0.35	1.28	Q8BTZ7	Mannose-1-phosphate guanyltransferase beta	Gmppb	3215	10	10	10	39.4	39.4	39.4	39.916	0.0000	75.679	28164000	169	16.68	16.32	16.38	16.70	16.23	16.33	16.26	15.84
1.712	0.35	1.28	O35166	Golgi SNAP receptor complex member 2	Gosr2	345	14	14	14	50	50	50	24.725	0.0000	119.73	24249000	268	15.94	15.69	16.07	16.13	15.54	15.49	15.72	15.67
1.264	0.35	1.28	Q9WUQ2	Prolactin regulatory element-binding protein	Preb	5720	13	13	13	38.4	38.4	38.4	45.437	0.0000	190.63	30619000	269	16.21	16.38	16.74	16.67	15.95	16.18	16.34	16.11
0.415	0.35	1.28	Q9EQ20	Methylmalonate-semialdehyde dehydrogenase [acylating], mitochondria	Aldh6a1	5269	15	15	15	37.6	37.6	37.6	57.915	0.0000	122.85	17464000	140	15.50	15.81	16.87	16.44	15.54	15.55	15.66	16.44
2.439	0.35	1.28	Q8C3J5	Dedicator of cytokinesis protein 2	Dock2	3376	91	91	90	55.6	55.6	55.1	211.7	0.0000	323.31	305970000	2056	17.83	17.50	17.64	17.71	17.21	17.34	17.36	17.36
1.267	0.35	1.28	Q62465	Synaptic vesicle membrane protein VAT-1 homolog	Vat1	2397	26	26	26	60.3	60.3	60.3	43.096	0.0000	323.31	655080000	1402	20.17	19.98	20.44	20.24	19.65	19.73	20.16	19.88
0.443	0.35	1.28	Q8K337	Type II inositol 1,4,5-trisphosphate 5-phosphatase	Inpp5b	3687	5	5	5	6.7	6.7	6.7	112.76	0.0000	19.876	6953900	58	15.52	14.46	14.39	15.19	13.88	14.62	14.83	14.82
0.445	0.35	1.28	Q8BMZ5	tRNA-splicing endonuclease subunit Sen34	Tsen34	3151	2	2	2	10.1	10.1	10.1	34.196	0.0004	4.4217	2169200	28	14.48	14.10	13.89	14.06	13.59	12.92	14.30	14.31
2.246	0.35	1.28	Q93092	Transaldolase	Taldo1	4313	44	44	44	70.3	70.3	70.3	37.387	0.0000	323.31	1825900000	2338	21.39	21.15	21.51	21.48	21.04	21.00	21.07	21.01
0.548	0.35	1.28	O70305	Ataxin-2	Atxn2	484	5	5	5	6.5	6.5	6.5	136.48	0.0000	25.907	3957500	62	14.35	14.19	14.55	14.76	13.70	14.78	14.33	13.63
2.366	0.35	1.28	Q3UMY5	Echinoderm microtubule-associated protein-like 4	Eml4	2006	13	13	13	18.4	18.4	18.4	110.03	0.0000	27.558	11247000	97	15.56	15.25	15.44	15.35	15.09	15.14	15.01	14.95
1.321	0.35	1.28	P62196	26S protease regulatory subunit 8	Psmc5	1407	37	37	37	83.3	83.3	83.3	45.626	0.0000	323.31	205520000	1036	18.66	18.54	18.76	18.96	18.44	18.50	18.52	18.05

0.470	0.35	1.28	P59672	Ankyrin repeat and SAM domain-containing protein 1A	Anks1a	1340	13	13	13	18.6	18.6	18.6	125.24	0.0000	17.437	7911400	39	14.61	13.74	14.20	14.25	13.74	13.11	14.47	14.07
1.101	0.35	1.28	Q99LE6	ATP-binding cassette sub-family F member 2	Abcf2	4424	23	23	23	35.7	35.7	35.7	71.781	0.0000	64.074	49674000	405	16.51	15.99	16.46	16.43	15.66	16.11	16.02	16.18
0.814	0.35	1.28	P47713	Cytosolic phospholipase A2;Phospholipase A2;Lysophospholipase	Pla2g4a	1116	16	16	15	28.9	28.9	28.7	85.221	0.0000	80.446	20513000	173	15.77	15.93	15.81	16.28	16.12	15.49	15.29	15.49
0.445	0.35	1.28	Q922Q8	Leucine-rich repeat-containing protein 59	Lrrc59	4271	17	17	17	48.9	48.9	48.9	34.877	0.0000	194.61	294440000	621	19.01	18.71	19.63	19.38	18.67	19.44	19.11	18.10
0.685	0.35	1.27	Q9WUL7	ADP-ribosylation factor-like protein 3	Arl3	5714	7	7	7	53.3	53.3	53.3	20.486	0.0000	38.699	10340000	119	15.60	15.65	15.80	15.61	15.05	14.81	15.91	15.49
0.714	0.35	1.27	Q9JKV1	Proteasomal ubiquitin receptor ADRM1	Adrm1	5459	7	7	7	19.9	19.9	19.9	42.06	0.0000	65.122	31485000	118	17.76	16.89	17.60	17.77	17.11	17.28	17.38	16.85
1.379	0.35	1.27	P42230	Signal transducer and activator of transcription 5A	Stat5a	1077	17	17	6	23	23	8.1	90.83	0.0000	90.869	24632000	251	15.79	15.46	16.03	15.92	15.38	15.55	15.54	15.33
1.503	0.35	1.27	Q9D8N0	Elongation factor 1-gamma	Eef1g	5065	34	34	34	62.9	62.9	62.9	50.06	0.0000	323.31	586990000	1119	20.52	20.29	20.28	20.42	20.16	19.74	20.24	19.96
1.026	0.35	1.27	E9QAM5	Helicase with zinc finger domain 2	Helz2	253	34	34	34	15.1	15.1	15.1	331.56	0.0000	158.93	27853000	207	16.56	15.86	16.35	16.10	16.10	15.82	15.87	15.68
0.489	0.35	1.27	Q62086	Serum paraoxonase/arylesterase 2	Pon2	2353	10	10	10	42.7	42.7	42.7	39.617	0.0000	101.37	14071000	111	16.48	15.27	15.66	15.89	15.74	15.58	14.87	15.71
0.530	0.35	1.27	Q9D2R0	Acetoacetyl-CoA synthetase	Aacs	4949	18	18	18	28.7	28.7	28.7	75.199	0.0000	125.19	50704000	334	17.17	17.08	17.38	16.64	17.03	17.27	16.40	16.16
0.671	0.35	1.27	P48962;Q3V	ADP/ATP translocase 1	Slc25a4	1152	36	21	21	70.5	46.3	46.3	32.904	0.0000	181.39	141290000	410	19.45	18.99	18.79	19.04	19.02	18.11	18.85	18.91
0.398	0.35	1.27	Q9CWZ3	RNA-binding protein 8A	Rbm8a	4740	4	4	4	32.2	32.2	32.2	19.889	0.0000	112.82	3093000	26	13.00	13.12	11.80	12.65	11.82	12.26	12.14	12.97
0.943	0.35	1.27	Q8BZ20	Poly [ADP-ribose] polymerase 12	Parp12	3310	13	13	13	28.7	28.7	28.7	79.916	0.0000	61.12	11658000	146	15.39	14.92	15.05	14.87	15.01	14.33	14.85	14.64
0.379	0.35	1.27	Q99JR6	Nicotinamide/nicotinic acid mononucleotide adenylyltransferase 3	Nmnat3	4345	3	3	3	24.9	24.9	24.9	27.702	0.0056	2.2224	1209400	10	13.54	13.54	14.02	13.77	14.21	13.38	12.36	13.52
0.308	0.35	1.27	Q99LU0	Charged multivesicular body protein 1b-1	Chmp1b1	4447	6	6	2	20.6	20.6	5	22.124	0.0000	15.094	22317000	100	16.89	16.86	16.58	16.78	16.97	15.03	16.91	16.82
2.228	0.35	1.27	P51881	ADP/ATP translocase 2;ADP/ATP translocase 2, N-terminally processe	Slc25a5	1208	42	42	27	74.2	74.2	50.7	32.931	0.0000	323.31	1150500000	1648	21.20	20.96	20.95	21.00	20.69	20.61	20.58	20.84
0.533	0.35	1.27	Q8CIG8	Protein arginine N-methyltransferase 5	Prmt5	3553	13	13	13	25.7	25.7	25.7	72.679	0.0000	64.574	14976000	169	15.95	15.51	15.98	15.29	15.60	15.79	14.65	15.31
0.442	0.35	1.27	Q8K385	Ferric-chelate reductase 1	FRRS1	3695	3	3	3	6.4	6.4	6.4	66.047	0.0000	7.675	4982800	56	14.61	15.69	14.94	14.99	15.15	14.21	15.20	14.30
1.040	0.34	1.27	P46471	26S protease regulatory subunit 7	Psmc2	1106	34	34	34	78.8	78.8	78.8	48.647	0.0000	323.31	193710000	898	18.26	17.94	18.68	18.34	17.94	18.06	18.10	17.74
0.412	0.34	1.27	Q8CD92	Tetratricopeptide repeat protein 27	Ttc27	3457	6	6	6	9.9	9.9	9.9	96.43	0.0000	21.171	1991200	15	14.72	14.07	13.33	13.52	13.62	13.88	13.77	12.99
0.273	0.34	1.27	Q8K273	Membrane magnesium transporter 1	Mmgt1	3646	3	3	3	42.7	42.7	42.7	14.677	0.0000	74.281	11132000	66	16.34	16.62	15.15	15.22	16.01	16.06	15.36	14.53
0.513	0.34	1.27	Q8R3Y8	Interferon regulatory factor 2-binding protein 1	Irf2bp1	3860	5	4	4	12.5	11.3	11.3	61.75	0.0000	23.07	5377400	85	15.65	15.23	15.91	14.52	14.89	15.14	14.90	15.01
1.801	0.34	1.27	P41241	Tyrosine-protein kinase CSK	Csk	1068	31	31	30	74.2	74.2	72	50.716	0.0000	203.77	97358000	613	17.63	17.49	17.64	17.73	17.36	17.49	17.15	17.10
0.750	0.34	1.27	Q8BKE6	Cytochrome P450 20A1	Cyp20a1	3100	10	10	10	25.3	25.3	25.3	52.149	0.0000	59.661	11741000	99	16.42	15.69	15.59	16.05	15.77	15.30	15.82	15.48
0.554	0.34	1.27	Q91Z96	BMP-2-inducible protein kinase	Bmp2k	4191	11	10	10	16.8	16	16	126.18	0.0000	21.209	13615000	97	15.93	15.80	16.21	15.99	15.62	15.86	14.89	16.20
0.741	0.34	1.27	Q8BYH7	TBC1 domain family member 17	Tbc1d17	3298	9	9	9	21.9	21.9	21.9	72.859	0.0000	19.553	9428900	81	15.51	15.05	15.49	15.17	15.28	15.27	14.86	14.46
1.533	0.34	1.27	Q80UP3	Diacylglycerol kinase zeta	Dgkz	2834	13	13	11	17.2	17.2	14.5	104.03	0.0000	40.911	14482000	123	16.03	15.70	15.90	15.65	15.39	15.51	15.69	15.32
1.964	0.34	1.27	Q8BH04	Phosphoenolpyruvate carboxykinase [GTP], mitochondrial	Pck2	3005	36	36	35	68.4	68.4	67.2	70.527	0.0000	323.31	137210000	873	17.78	17.65	17.98	17.94	17.58	17.39	17.60	17.40
1.582	0.34	1.27	Q3THK7	GMP synthase [glutamine-hydrolyzing]	Gmps	1846	30	30	30	58.6	58.6	58.6	76.723	0.0000	317.57	65815000	528	17.03	16.95	17.26	17.24	16.82	16.60	17.00	16.68
0.643	0.34	1.27	Q8CJ53	Cdc42-interacting protein 4	Trip10	3567	15	15	15	30.2	30.2	30.2	68.489	0.0000	148.24	11641000	122	15.26	15.63	15.42	15.85	15.07	15.48	14.63	15.60
1.332	0.34	1.27	Q9D1E6	Tubulin-folding cofactor B	Tbcb	4908	18	18	18	80.7	80.7	80.7	27.385	0.0000	183.8	55696000	416	17.78	17.46	17.35	17.73	17.03	17.26	17.19	17.47
1.113	0.34	1.27	P28474;Q64	Alcohol dehydrogenase class-3	Adh5	950	19	19	19	44.4	44.4	44.4	39.547	0.0000	199.56	123500000	555	17.43	17.23	17.87	17.45	16.97	17.30	17.31	17.04
0.845	0.34	1.27	Q5NC05	Transcription termination factor 2	Ttf2	2116	10	10	10	12.8	12.8	12.8	125.53	0.0000	36.599	3795500	50	13.60	13.54	14.22	14.25	13.46	13.50	13.56	13.73
2.249	0.34	1.27	P06151	L-lactate dehydrogenase A chain	Ldha	655	40	40	37	91	91	84.3	36.498	0.0000	323.31	1410900000	2137	21.22	21.10	20.88	20.99	20.81	20.66	20.67	20.68
1.160	0.34	1.27	Q9CXF4	TBC1 domain family member 15	Tbc1d15	4754	25	25	25	42.2	42.2	42.2	76.526	0.0000	107.65	38944000	258	16.34	16.37	16.83	16.62	16.34	15.90	16.26	16.29
0.669	0.34	1.27	Q8BLY7	Hermansky-Pudlak syndrome 6 protein homolog	Hps6	3125	9	9	9	19.8	19.8	19.8	87.34	0.0000	12.824	4480600	36	14.15	14.55	15.00	13.90	14.00	14.21	14.00	14.03
0.601	0.34	1.27	Q14CH1	Molybdenum cofactor sulfurase	Mocos	1789	32	32	32	54.9	54.9	54.9	95.012	0.0000	323.31	61334000	690	16.52	16.35	16.37	16.33	16.57	15.42	16.40	15.82
0.169	0.34	1.27	Q14C51	Pentatricopeptide repeat domain-containing protein 3, mitochondrial	Ptcd3	1788	10	10	10	21.5	21.5	21.5	77.795	0.0000	28.699	8018300	61	13.60	15.21	15.81	15.50	15.33	13.04	15.80	14.59
0.805	0.34	1.27	Q91YE6	Importin-9	Ipo9	4155	19	19	19	28.8	28.8	28.8	116.05	0.0000	323.31	59831000	469	17.70	16.96	17.31	17.47	17.11	16.60	17.21	17.16
1.334	0.34	1.27	Q99P72	Reticulon-4	Rtn4	4502	25	25	25	32.4	32.4	32.4	126.61	0.0000	323.31	339280000	558	20.05	19.46	19.53	19.72	19.40	19.37	19.38	19.26
0.383	0.34	1.27	Q80XL6	Acyl-CoA dehydrogenase family member 11	Acad11	2885	10	10	10	18.4	18.4	18.4	87.365	0.0000	75.791	7086200	77	13.63	14.19	15.25	13.98	14.07	13.70	13.60	14.33
1.209	0.34	1.27	Q8CHC4	Synaptojanin-1	Synj1	3521	32	32	32	30.2	30.2	30.2	172.62	0.0000	322.53	68612000	521	16.59	16.38	17.07	16.62	16.34	16.28	16.40	16.27
0.584	0.34	1.26	Q99104	Unconventional myosin-Va	Myo5a	4315	70	70	67	44.3	44.3	43.5	215.54	0.0000	323.31	128900000	1263	17.09	16.60	17.72	17.36	16.49	16.85	17.12	16.96
0.817	0.34	1.26	Q9Z2X8	Kelch-like ECH-associated protein 1	Keap1	5872	8	8	8	14.7	14.7	14.7	69.552	0.0000	12.119	3875400	40	14.52	14.29	14.36	13.67	13.99	13.84	14.02	13.64
1.421	0.34	1.26	P19157;P46	Glutathione S-transferase P 1	Gstp1	843	8	8	8	51.9	51.9	51.9	23.609	0.0000	224.21	87875000	291	17.82	18.09	18.14	17.79	17.79	17.64	17.38	17.68
1.261	0.34	1.26	Q8K0V4	CCR4-NOT transcription complex subunit 3	Cnot3	3610	8	8	7	11.3	11.3	11.3	81.945	0.0000	13.394	7474900	72	14.77	14.60	15.10	14.82	14.55	14.39	14.73	14.29
1.058	0.34	1.26	Q9ER38	Torsin-3A	Tor3a	5292	11	11	11	33.8	33.8	33.8	43.813	0.0000	74.736	27922000	161	17.17	16.84	17.40	17.05	16.99	16.86	16.82	16.45
0.804	0.34	1.26	Q99LI8	Hepatocyte growth factor-regulated tyrosine kinase substrate	Hgs	4434	21	21	20	32.4	32.4	31.4	86.014	0.0000	139.57	24375000	185	16.61	16.14	16.74	15.90	16.03	16.06	16.12	15.83
0.988	0.33	1.26	Q9Z2V5	Histone deacetylase 6	Hdac6	5866	8	8	8	9.7	9.7	9.7	125.79	0.0000	19.54	8813700	79	16.21	15.45	15.92	15.85	15.40	15.46	15.75	15.49
1.452	0.33	1.26	Q9Z2W0	Aspartyl aminopeptidase	Dnpep	5868	20	20	20	52.2	52.2	52.2	52.206	0.0000	323.31	90121000	494	17.42	17.27	17.61	17.25	17.07	17.20	17.15	16.79
0.640	0.33	1.26	Q80XI4	Phosphatidylinositol 5-phosphate 4-kinase type-2 beta	Pip4k2b	2882	20	14	14	49.3	38.2	38.2	47.318	0.0000	99.178	24752000	171	16.22	16.22	17.07	16.84	16.37	16.19	16.50	15.94
1.189	0.33	1.26	Q5SUA5	Unconventional myosin-Ig	Myo1g	2143	62	62	61	62.1	62.1	60.8	117.23	0.0000	323.31	349690000	1615	19.39	18.97	19.15	18.89	18.68	18.54	18.99	18.86
0.289	0.33	1.26	Q8VEH8	Endoplasmic reticulum lectin 1	Erlec1	4006	3	3	3	6.4	6.4	6.4	54.906	0.0011	3.634	3276200	31	13.80	13.95	14.27	12.18	12.88	13.31	13.34	13.34
1.204	0.33	1.26	Q6PD03	Serine/threonine-protein phosphatase 2A 56 kDa regulatory subunit alpl	Ppp2r5a	2607	15	15	15	33.1	33.1	33.1	56.346	0.0000	41.9	15261000	221	15.08	14.84	15.43	15.32	14.68	14.95	14.94	14.77
0.929	0.33	1.26	Q6A0A9;Q8(	Constitutive coactivator of PPAR-gamma-like protein 1	FAM120A	2501	31	31	31	36.2	36.2	36.2	121.64	0.0000	323.31	97432000	761	17.20	16.51	16.94	16.99	16.37	16.39	16.74	16.80
0.892	0.33	1.26	Q80TH2	Protein LAP2	Erbb2ip	2804	21	21	19	23.3	23.3	21.4	157.25	0.0000	94.214	33838000	276	17.00	16.58	17.35	16.95	16.39	16.65	16.89	16.62
0.993	0.33	1.26	Q6PIC6	Sodium/potassium-transporting ATPase subunit alpha-3	Atp1a3	2643	56	36	31	52.2	37.1	32.8	111.69	0.0000	323.31	100880000	654	17.68	17.28	17.98	17.95	17.38	17.24	17.44	17.51
1.324	0.33	1.26	P14211	Calreticulin	Calr	778	31	31	31	59.1	59.1	59.1	47.994	0.0000	323.31	315410000	913	18.99	19.23	18.73	19.03	18.50	18.66	18.60	18.89
1.424	0.33	1.26	Q6DFW4	Nucleolar protein 58	Nop58	2506	26	26	26	49.6	49.6	49.6	60.342	0.0000	301.66	86125000	627	17.02	16.57	16.95	16.89	16.47	16.72	16.56	16.36
0.633	0.33	1.26	Q9CYH2	Redox-regulatory protein FAM213A	Fam213a	4797	5	5	5	17.4	17.4	17.4	24.394	0.0000	19.949	5429300	68	14.92	14.25	14.62	15.12	14.24	14.06	14.48	14.80
0.372	0.33	1.26	P46737;Q7N	Lys-63-specific deubiquitinase BRCC36	Brcc3	1112	9	9	9	36.1	36.1	36.1	33.34	0.0000	30.118	16743000	130	15.81	14.77	15.64	15.00	15.20	14.10	15.38	15.23
1.058	0.33	1.26	Q9D071	MMS19 nucleotide excision repair protein homolog	Mms19	4853	12	12	12	20.9	20.9	20.9	113.09	0.0000	150.33	23222000	214	16.32	16.05	16.16	16.19	16.12	15.41	15.96	15.91
1.105	0.33	1.26	Q91ZR1	Ras-related protein Rab-4B	Rab4b	4198	12	12	11	70	70	63.4	23.629	0.0000	135.42	23147000	264	16.01	15.77	16.17	16.39	15.90	15.52	15.88	15.72
1.553	0.33	1.26	Q9CXW3	Calcyclin-binding protein	Cacybp	4771	23	23	23	90.8	90.8	90.8	26.51	0.0000	323.31	74229000	598	16.92	16.64	16.68	16.76	16.40	16.61	16.51	16.16
1.760	0.33	1.26	Q9ER72	Cysteine--tRNA ligase, cytoplasmic	Cars	5296	46	46	46	68.1	68.1	68.1	94.859	0.0000	323.31	101720000	740	17.02	16.87	17.25	17.12	16.62	16.86	16.82	16.63
0.233	0.33	1.26	Q9CQE3	28S ribosomal protein S17, mitochondrial	Mrps17	4588	2	2	2	15	15	15	13.382	0.0025	2.8818	2051900	37	14.67	13.55	13.62	13.27	12.80	14.65	13.79	12.56
2.074	0.33	1.26	Q9WUA3	ATP-dependent 6-phosphofructokinase, platelet type	Pfkp	5703	45	45	41	58.3	58.3	55.2	85.454	0.0000	323.31	273890000	1425	19.09	18.83	18.86	18.79	18.67	18.56	18.61	18.42
1.047	0.33	1.26	Q3URS9	Coiled-coil domain-containing protein 51	Ccdc51	2025	8	8	8	24.6	24.6	24.6	45.132	0.0000	39.564	14587000	226	15.19	14.60	15.12	15.33	14.64	14.80	14.76	14.73
2.141	0.33	1.26	P16879	Tyrosine-protein kinase Fes/Fps	Fes	816	25	25	24	36.9	36.9	35.5	93.778	0.0000	238.47	48422000	378	16.51	16.29	16.59	16.65	16.26	16.17	16.17	16.13
0.374	0.33	1.26	Q8R5G7	Arf-GAP with Rho-GAP domain, ANK repeat and PH domain-containing	Arap3	3888	14	14	14	14.8	14.8	14.8	169.74	0.0000	56.643	9226600	69	15.53	15.69	15.95	15.50	15.63	15.47	15.98	14.29
0.226	0.33	1.26	Q3TC72	Fumarylacetoacetate hydrolase domain-containing protein 2A	Fahd2	1824	4	4	4	26.8	26.8	26.8	34.69	0.0000	19.617	2099100	14	14.33	14.13	13.23	14.63	14.53	12.54	14.60	13.33
1.164	0.33	1.26	P47199	Quinone oxidoreductase	Cryz	1114	6	6	6	29.3	29.3	29.3	35.268	0.0000	24.786	8607100	158	14.78	14.63	14.86	14.97	14.48	14.15	14.50	14.79
0.657	0.33	1.25	Q9DAA6	Exosome complex component CSL4	Exosc1	5106	4	4	4	28.2	28.2	28.2	21.423	0.0000	10.249	6443200	52	14.77	15.03	15.65	14.98	14.87	14.69	15.14	14.43
0.882	0.33	1.25	B2RQC6	CAD protein;Glutamine-dependent carbamoyl-phosphate synthase;Asp	Cad	77	43	43	43	27	27	27	243.24	0.0000	323.31	75280000	637	16.93	16.32	16.91	16.78	16.71	16.38	16.44	16.11
1.159	0.33	1.25	Q7TQK5	Coiled-coil domain-containing protein 93	Ccdc93	2778	34	34	34	53.6	53.6	53.6	72.602	0.0000	187.31	61938000	604	16.77	16.21	16.64	16.64	16.09	16.47	16.16	16.22
2.060	0.33	1.25	Q7TSV4	Phosphoglucomutase-2	Pgm2	2789	22	22	22	49.2	49.2	49.2	68.747	0.0000	221.78	67493000	561	17.16	17.49	17.38	17.29	16.87	16.99	17.09	17.07
0.546	0.33	1.25	Q8BG05	Heterogeneous nuclear ribonucleoprotein A3	Hnrnpa3	2956	28	28	28	51.7	51.7	51.7	39.652	0.0000	323.31	265160000	957	19.50	19.85	18.57	19.32	19.03	18.82	19.01	19.08
2.438	0.33	1.25	Q02053	Ubiquitin-like modifier-activating enzyme 1	Uba1	1715	64	64	55	72.1	72.1	65.8	117.81	0.0000	323.31	944130000	2496	20.72	20.49	20.77	20.63	20.40	20.28	20.37	20.25
1.027	0.33	1.25	G5E829;Q9I	Plasma membrane calcium-transporting ATPase 1	Atp2b1	262	43	43	30	38.2	38.2	26.2	134.75	0.0000	323.31	174300000	976	18.17	17.76	18.49	18.27	17.72	17.79	18.00	17.86
0.853	0.32	1.25	Q80YV3	Transformation/transcription domain-associated protein	Trrap	2908	5	5	5	3	3	3	291.55	0.0000	14.262	3990700	55	14.50	14.17	15.07	14.59	14.35	14.23	14.32	14.15
0.793	0.32	1.25	Q91WJ8	Far upstream element-binding protein 1	Fubp1	4113	18	15	15	37.2	33	33	68.539	0.0000	47.725	23106000	202	15.60	16.09	16.01	15.97	15.31	15.64	15.36	16.06
0.437	0.32	1.25	Q8VE18	Protein SMG8	Smg8	3982	6	6	6	11.5	11.5	11.5	109.67	0.0000	68.774	4682400	84	13.88	13.87	14.72	14.78	14.56	13.94	13.92	13.52
0.961	0.32	1.25	Q9ERU9	E3 SUMO-protein ligase RanBP2	Ranbp2	5320	71	71	70	29.8	29.8	29.5	341.12	0.0000	323.31	99914000	714	18.72	18.22	19.01	18.48	18.27	18.27	18.39	18.20
0.574	0.32	1.25	P40240	CD9 antigen	Cd9	1060	4	4	4	10.2	10.2	10.2	25.258	0.0000	17.691	40963000	112	17.91	17.51	17.45	18.46	17.45	17.57	17.81	17.20
0.678	0.32	1.25	Q91WS0	CDGSH iron-sulfur domain-containing protein 1	Cisd1	4125	6	6	6	42.6	42.6	42.6	12.097	0.0000	67.501	20745000	122	16.32	15.91	16.48	16.56	15.76	15.72	15.99	16.50
0.561	0.32	1.25	Q8R0X2	CDK2-associated and cullin domain-containing protein 1	Cacul1	3767	5	5	5	23.9	23.9	23.9	42.106	0.0000	29.657	4656000	53	15.22	14.75	15.70	15.90	15.21	15.10	14.83	15.12
1.207	0.32	1.25	Q571I9	Aldehyde dehydrogenase family 16 member A1	Aldh16a1	2092	19	19	18	31.9	31.9	30.7	84.755	0.0000	299.88	61750000	361	17.34	16.85	16.98	17.42	16.81	16.75	16.86	16.87
2.024	0.32	1.25	Q9D7G0	Ribose-phosphate pyrophosphokinase 1	Prps1	5029	17	17	9	64.8	64.8	42.5	34.834	0.0000	128.09	79030000	413	17.74	17.76	17.76	17.71	17.62	17.50	17.26	17.29
0.180	0.32	1.25	Q8C196	Carbamoyl-phosphate synthase [ammonia], mitochondrial	Cps1	3361	28	28	27	26.3	26.3	25.2	164.62	0.0000	82.208	8783400	34	12.02	13.46	12.87	12.92	11.79	14.37	12.16	11.65
1.391	0.32	1.25	Q9JJ28	Protein flightless-1 homolog	Flii	5417	53	53	53	54.3	54.3	54.3	144.8	0.0000	323.31	175230000	1029	18.11	17.59	18.07	17.98	17.60	17.53	17.66	17.68
0.908	0.32	1.25	Q3ULJ0	Glycerol-3-phosphate dehydrogenase 1-like protein	Gpd1l	1993	18	18	16	56.7	56.7	51.6	38.225	0.0000	120.9	45532000	410	16.85	16.61	17.04	17.29	16.36	16.54	16.78	16.82
1.269	0.32	1.25	O55222	Integrin-linked protein kinase	Ilk	464	31	31	31	62.4	62.4	62.4	51.373	0.0000	238.21	165230000	761	18.24	18.07	18.49	18.29	17.91	18.24	17.88	17.76
0.719	0.32	1.25	Q8BFZ9	Erlin-2	Erlin2	2955	18	12	12	48.8	39.7	39.7	37.872	0.0000	200.39	40516000	270	16.57	16.87	17.04	16.81	16.61	16.41	16.96	16.02
1.762	0.32	1.25	Q9DC16	Endoplasmic reticulum-Golgi intermediate compartment protein 1	Ergic1	5186	10	10	10	30	30	30	32.562	0.0000	78.641	14195000	179	15.16	15.31	15.23	15.30	15.03	14.65	15.03	15.00
2.270	0.32	1.25	Q9DCD0	6-phosphogluconate dehydrogenase, decarboxylating	Pgd	5207	50	50	50	89.9	89.9	89.9	53.247	0.0000	323.31	1955300000	2821	21.11	20.88	21.11	20.89	20.75	20.62	20.73	20.60
0.614	0.32	1.25	Q9D0F6	Replication factor C subunit 5	Rfc5	4864	8	8	8	34.8	34.8	34.8	38.096	0.0000	27.074	4688300	44	13.62	13.28	13.64	13.75	13.35	12.63	13.31	13.72
0.152	0.32	1.25	Q9ERD8	Gamma-parvin	Parvg	5304	4	4	4	19.9	19.9	19.9	37.603	0.0000	21.543	8186500	72	16.18	16.19	14.17	15.82	16.03	13.51	15.10	16.44
0.463	0.32	1.25	Q05860	Formin-1	Fmn1	1743	3	3	3	2.6	2.6	2.6	163.58	0.0000	8.1526	1393100	33	13.72	13.38	13.46	14.24	13.49	13.91	13.39	12.73
0.440	0.32	1.25	Q9CPP6	NADH dehydrogenase [ubiquinone] 1 alpha subcomplex subunit 5	Ndufa5	4524	11	11	11	86.2	86.2	86.2	13.36	0.0000	125.51	16160000	148	15.96	16.28	15.07	16.08	15.41	15.06	15.88	15.75
0.711	0.32	1.25	Q9Z2X1	Heterogeneous nuclear ribonucleoprotein F;Heterogeneous nuclear ribc	Hnrnpf	5870	26	26	24	68	68	65.5	45.729	0.0000	323.31	163360000	689	18.63	19.14	18.13	18.68	18.37	18.49	18.15	18.28
0.528	0.32	1.25	O88708	Origin recognition complex subunit 4	Orc4	561	2	2	1	6.9	6.9	3.7	49.981	0.0002	5.4064	1097100	16	13.21	13.43	13.14	13.59	12.69	13.80	12.78	12.81
1.600	0.32	1.25	Q99LC3	NADH dehydrogenase [ubiquinone] 1 alpha subcomplex subunit 10, mit	Ndufa10	4416	17	17	17	61.1	61.1	61.1	40.603	0.0000	133.08	44003000	388	16.07	16.16	16.05	15.97	16.00	15.80	15.65	15.53
1.589	0.32	1.25	P28352	DNA-(apurinic or apyrimidinic site) lyase;DNA-(apurinic or apyrimidinic s	Apex1	949	19	19	19	71.6	71.6	71.6	35.49	0.0000	196.43	80228000	507	17.30	17.02	17.28	17.12	17.06	16.68	16.94	16.77
0.597	0.32	1.25	P97370	Sodium/potassium-transporting ATPase subunit beta-3	Atp1b3	1635	12	12	12	38.5	38.5	38.5	31.775	0.0000	41.707	63159000	216	17.92	17.78	17.42	17.54	17.73	16.70	17.40	17.55
2.340	0.32	1.25	Q76MZ3	Serine/threonine-protein phosphatase 2A 65 kDa regulatory subunit A a	Ppp2r1a	2719	41	41	31	70.6	70.6	57	65.322	0.0000	323.31	421040000	1558	18.82	18.74	18.78	18.80	18.47	18.30	18.64	18.44
0.301	0.32	1.25	Q9R0Q6	Actin-related protein 2/3 complex subunit 1A	Arpc1a	5637	7	4	4	25.1	16.5	16.5	41.626	0.0000	20.053	2765700	40	14.06	13.54	13.55	14.40	14.52	12.89	12.99	13.89
1.357	0.32	1.25	Q8CG48	Structural maintenance of chromosomes protein 2	Smc2	3497	64	64	64	55.2	55.2	55.2	134.24	0.0000	294.97	76308000	671	16.43	16.14	16.61	16.46	16.18	16.26	16.01	15.92
1.467	0.32	1.25	Q99KX1	Myeloid leukemia factor 2	Mlf2	4399	4	4	4	23.5	23.5	23.5	28.055	0.0000	7.1161	2014300	18	13.64	13.45	13.66	13.27	13.18	13.03	13.37	13.17
2.219	0.32	1.25	Q99JX7	Nuclear RNA export factor 1	Nxf1	4355	19	19	19	39.8	39.8	39.8	70.299	0.0000	84.122	29203000	276	16.13	16.06	16.25	16.18	15.69	15.94	15.78	15.95
0.175	0.32	1.25	Q8BYM8	Probable cysteine--tRNA ligase, mitochondrial	Cars2	3304	2	2	2	5.8	5.8	5.8	61.271	0.0002	5.794	809090	20	13.22	13.27	13.77	12.89	14.99	12.30	12.09	12.50
0.257	0.32	1.24	Q8BGK6	Y+L amino acid transporter 2	Slc7a6	2986	3	3	2	9.7	9.7	8.3	56.77	0.0000	31.551	11291000	60	15.85	16.06	17.03	16.03	14.73	16.75	16.04	16.19
1.410	0.32	1.24	Q61599	Rho GDP-dissociation inhibitor 2	Arhgdib	2318	22	22	22	93.5	93.5	93.5	22.851	0.0000	323.31	589710000	837	21.62	21.63	21.78	21.89	21.48	21.62	21.44	21.14
0.224	0.31	1.24	O70572	Sphingomyelin phosphodiesterase 2	Smpd2	514	7	7	7	22	22	22	47.466	0.0000	16.442	4560400	34	15.06	14.27	13.89	13.21	12.89	14.85	13.92	13.52

0.694	0.31	1.24	Q9CZ44	NSFL1 cofactor p47	Nsfl1c	4818	9	9	9	38.1	38.1	38.1	40.709	0.0000	67.787	14392000	120	16.09	16.51	15.98	15.86	15.56	16.28	15.76	15.59
0.401	0.31	1.24	P42567	Epidermal growth factor receptor substrate 15	Eps15	1080	13	13	13	24	24	24	98.47	0.0000	259.59	23019000	182	17.12	17.11	17.70	17.50	17.16	17.51	17.37	16.13
0.766	0.31	1.24	Q91YD3	mRNA-decapping enzyme 1A	Dcp1a	4153	3	3	3	8.8	8.8	8.8	65.218	0.0000	10.689	6885800	64	14.77	15.13	15.43	15.02	14.97	14.52	15.08	14.51
0.854	0.31	1.24	O54984	ATPase Asna1	Asna1	439	19	19	19	68.1	68.1	68.1	38.822	0.0000	288.27	72323000	368	18.02	17.71	17.64	17.98	17.57	17.44	17.92	17.16
1.249	0.31	1.24	Q9EQH3	Vacuolar protein sorting-associated protein 35	Vps35	5278	50	50	50	68.3	68.3	68.3	91.712	0.0000	323.31	237250000	1034	19.32	18.82	18.78	18.97	18.75	18.53	18.63	18.73
1.844	0.31	1.24	P62821;Q8K	Ras-related protein Rab-1A	Rab1A	1447	27	27	16	86.3	86.3	58	22.677	0.0000	323.31	666880000	1365	20.29	19.95	20.32	20.18	19.80	19.94	19.93	19.82
0.358	0.31	1.24	Q8R043	Uncharacterized protein C6orf1 homolog		3746	2	2	2	19.6	19.6	19.6	11.553	0.0022	3.0483	1150600	23	13.03	13.35	13.95	13.39	12.28	13.02	13.32	13.84
0.789	0.31	1.24	P27870	Proto-oncogene vav	Vav1	942	60	60	59	65.2	65.2	64	98.136	0.0000	323.31	154000000	975	18.12	17.57	17.29	17.80	17.56	17.17	17.33	17.47
0.715	0.31	1.24	P05555	Integrin alpha-M	Itgam	652	45	45	45	40	40	40	127.48	0.0000	323.31	243250000	1075	18.14	18.13	18.13	18.03	18.33	17.46	17.94	17.45
1.117	0.31	1.24	Q9JIG7	Coiled-coil domain-containing protein 22	Ccdc22	5398	36	36	36	67.5	67.5	67.5	70.843	0.0000	312.28	79701000	727	17.00	16.88	17.34	17.31	16.73	17.09	16.70	16.77
0.367	0.31	1.24	Q9WUU8	TNFAIP3-interacting protein 1	Tnip1	5724	5	5	5	9.4	9.4	9.4	73.049	0.0000	20.585	1716400	45	13.75	14.22	12.83	12.92	12.81	12.99	13.18	13.50
0.339	0.31	1.24	Q9Z0V8	Mitochondrial import inner membrane translocase subunit Tim17-A	Timm17a	5789	3	3	3	37.4	37.4	37.4	18.111	0.0000	38.573	1700100	13	13.79	13.64	13.73	14.24	13.24	14.23	14.04	12.64
1.249	0.31	1.24	Q8CG72	Poly(ADP-ribose) glycohydrolase ARH3	Adprhl2	3499	9	9	9	37	37	37	39.414	0.0000	221.7	27986000	169	16.43	16.31	16.57	16.73	15.98	16.10	16.37	16.35
1.584	0.31	1.24	O88487;O88	Cytoplasmic dynein 1 intermediate chain 2	Dync1i2	534	18	18	18	42.5	42.5	42.5	68.393	0.0000	323.31	138280000	626	18.95	18.52	18.82	18.94	18.45	18.56	18.56	18.43
0.402	0.31	1.24	Q8BUB4	WD repeat and FYVE domain-containing protein 2	Wdfy2	3222	11	11	11	43.5	43.5	43.5	45.094	0.0000	54.28	13738000	114	15.77	15.49	14.98	15.72	15.77	14.59	15.57	14.81
2.290	0.31	1.24	Q6ZWX6	Eukaryotic translation initiation factor 2 subunit 1	Eif2s1	2709	30	30	30	93.3	93.3	93.3	36.108	0.0000	323.31	146810000	610	18.88	18.69	18.86	19.03	18.53	18.57	18.59	18.55
0.426	0.31	1.24	Q6ZWQ7		Spcs3	2704	7	7	7	27.8	27.8	27.8	20.313	0.0000	14.097	18940000	81	16.27	16.21	16.20	16.84	16.59	15.30	16.36	16.03
0.126	0.31	1.24	Q9CY66	H/ACA ribonucleoprotein complex subunit 1	Gar1	4789	2	2	2	9.5	9.5	9.5	23.474	0.0004	4.9343	5165100	45	14.26	15.18	14.62	15.45	15.27	15.93	15.08	12.00
1.313	0.31	1.24	O08810	116 kDa U5 small nuclear ribonucleoprotein component	Eftud2	304	41	39	39	56.6	55.5	55.5	109.36	0.0000	244.55	81746000	748	16.99	16.62	17.04	17.06	16.74	16.62	16.70	16.42
0.946	0.31	1.24	Q61655	ATP-dependent RNA helicase DDX19A	Ddx19a	2320	21	21	20	57.9	57.9	55.6	53.932	0.0000	274.42	70916000	448	17.39	17.04	17.73	17.48	17.17	17.27	17.12	16.86
1.983	0.31	1.24	P35235	Tyrosine-protein phosphatase non-receptor type 11	Ptpn11	1011	25	25	25	46.4	46.4	46.4	68.034	0.0000	101.37	43099000	351	16.64	16.40	16.78	16.60	16.24	16.25	16.36	16.35
0.364	0.31	1.24	P10518	Delta-aminolevulinic acid dehydratase	Alad	722	9	9	9	49.7	49.7	49.7	36.023	0.0000	110.04	246280000	110	22.33	21.85	21.78	20.94	21.83	21.64	20.80	21.39
0.595	0.31	1.24	P13597	Intercellular adhesion molecule 1	Icam1	767	8	8	8	12.8	12.8	12.8	58.844	0.0000	226.49	26084000	139	16.64	16.51	17.39	17.01	16.62	16.72	16.81	16.18
0.123	0.31	1.24	Q9DC29	ATP-binding cassette sub-family B member 6, mitochondrial	Abcb6	5189	10	9	9	21	20.1	20.1	93.769	0.0000	175.96	14086000	102	13.74	15.22	17.15	16.50	15.60	15.76	16.28	13.74
0.402	0.31	1.24	P51175	Protoporphyrinogen oxidase	Ppox	1193	15	15	15	46.8	46.8	46.8	50.87	0.0000	142.53	11764000	138	14.99	14.25	14.53	14.21	14.22	14.23	14.84	13.45
0.363	0.31	1.24	P23949	Zinc finger protein 36, C3H1 type-like 2	Zfp36l2	892	6	6	5	15.7	15.7	15.1	50.069	0.0000	21.836	6233400	87	15.42	15.89	15.40	15.82	15.31	14.41	15.56	16.03
0.692	0.31	1.24	Q9D710	Thioredoxin-related transmembrane protein 2	Tmx2	5017	5	5	5	17.3	17.3	17.3	33.942	0.0000	27.886	11068000	121	15.30	14.98	15.44	14.92	14.91	14.96	14.36	15.18
1.687	0.31	1.24	O88712	C-terminal-binding protein 1	Ctbp1	562	17	17	11	42	42	34.5	47.744	0.0000	119.89	42133000	312	16.95	16.73	16.93	16.90	16.62	16.74	16.57	16.35
0.248	0.30	1.24	Q80SU7	Interferon-induced very large GTPase 1	Gvin1	2794	86	86	86	41.3	41.3	41.3	280.81	0.0000	323.31	94693000	875	16.99	16.39	15.70	15.25	16.31	16.28	15.58	14.95
0.547	0.30	1.24	Q6Q477	Calcium-transporting ATPase	Atp2b4	2649	19	6	6	21.2	9.1	9.1	133.07	0.0000	15.28	3989500	31	13.83	14.23	14.96	14.74	14.11	14.28	14.08	14.08
0.169	0.30	1.23	Q148V7	LisH domain and HEAT repeat-containing protein KIAA1468	Kiaa1468	1785	9	9	9	11.3	11.3	11.3	134.59	0.0000	10.641	18938000	30	16.52	16.89	16.80	17.05	16.97	17.33	17.28	14.46
0.652	0.30	1.23	O88448	Kinesin light chain 2	Klc2	531	13	6	6	27	16.5	16.5	66.662	0.0000	26.582	6356300	60	15.21	15.37	15.95	15.61	15.31	14.97	15.00	15.64
1.405	0.30	1.23	Q64442	Sorbitol dehydrogenase	Sord	2444	19	19	19	57.4	57.4	57.4	38.249	0.0000	155.49	54886000	317	17.47	17.02	17.26	17.31	16.80	16.89	17.10	17.05
1.010	0.30	1.23	O35609	Secretory carrier-associated membrane protein 3	Scamp3	386	11	11	11	26.4	26.4	26.4	38.458	0.0000	293.2	77611000	368	17.73	17.63	18.23	18.06	17.53	17.57	17.79	17.53
1.578	0.30	1.23	Q99KB8	Hydroxyacylglutathione hydrolase, mitochondrial	Hagh	4369	8	8	8	34	34	34	34.084	0.0000	84.058	23330000	189	16.47	16.46	16.65	16.39	15.94	16.30	16.32	16.18
0.691	0.30	1.23	Q9CQ56	Vesicle transport protein USE1	Use1	4563	7	7	7	28.1	28.1	28.1	30.593	0.0000	9.7614	6437600	52	15.03	14.52	15.31	15.16	14.45	14.65	14.66	15.04
0.760	0.30	1.23	O08663	Methionine aminopeptidase 2	Metap2	284	20	20	19	53.3	53.3	46	52.921	0.0000	200.84	43423000	325	17.07	16.58	16.68	17.15	16.83	16.23	16.74	16.47
0.550	0.30	1.23	Q91YS8	Calcium/calmodulin-dependent protein kinase type 1	Camk1	4177	11	11	9	44.4	44.4	39	41.624	0.0000	57.48	7852900	106	14.73	15.44	14.60	15.38	14.92	14.88	14.34	14.79
1.546	0.30	1.23	Q80YW0	Cytohesin-4	Cyth4	2910	14	14	14	41	41	41	45.284	0.0000	120.14	25624000	161	16.51	16.38	16.60	16.75	16.08	16.40	16.32	16.23
0.913	0.30	1.23	Q8R1V4	Transmembrane emp24 domain-containing protein 4	Tmed4	3802	10	8	8	48.5	37.9	37.9	26.022	0.0000	129.43	30032000	126	16.82	16.49	17.02	16.97	16.21	16.73	16.67	16.48
0.244	0.30	1.23	Q99J39	Malonyl-CoA decarboxylase, mitochondrial	Mlycd	4323	3	3	3	10.8	10.8	10.8	54.735	0.0000	22.894	2274300	35	13.08	13.27	14.33	14.20	13.75	13.31	14.22	12.40
0.816	0.30	1.23	Q91WC0	Histone-lysine N-methyltransferase setd3	Setd3	4103	13	13	13	31.8	31.8	31.8	67.175	0.0000	111.72	17667000	214	15.87	15.74	16.11	16.02	15.75	16.00	15.57	15.22
0.987	0.30	1.23	P61924	Coatomer subunit zeta-1	Copz1	1391	10	10	10	65.5	65.5	65.5	20.198	0.0000	255.35	76806000	360	18.23	18.13	18.51	18.03	18.00	17.92	18.17	17.60
0.540	0.30	1.23	P82343	N-acylglucosamine 2-epimerase	Renbp	1599	20	20	20	38.8	38.8	38.8	49.771	0.0000	323.31	90722000	579	17.74	17.56	16.80	17.66	17.28	16.73	17.19	17.36
0.990	0.30	1.23	Q6NSR8	Probable aminopeptidase NPEPL1	Npepl1	2526	16	16	16	49.6	49.6	49.6	55.939	0.0000	96.435	27602000	206	16.92	16.77	16.97	17.14	16.28	16.61	16.87	16.83
1.252	0.30	1.23	Q9WV55	Vesicle-associated membrane protein-associated protein A	Vapa	5732	26	26	25	81.5	81.5	81.5	27.855	0.0000	253.71	183440000	823	18.21	18.08	18.32	18.40	17.89	17.68	18.15	18.09
0.186	0.30	1.23	Q810V0	U3 small nucleolar ribonucleoprotein protein MPP10	Mphosph10	2933	5	5	5	11.5	11.5	11.5	78.734	0.0000	18.622	11763000	22	15.92	15.83	16.05	16.27	13.98	15.72	16.34	16.84
1.149	0.30	1.23	Q8R050;Q14	Eukaryotic peptide chain release factor GTP-binding subunit ERF3A;Eu	Gspt1;Gspt2	3747	18	18	18	37.1	37.1	37.1	68.625	0.0000	111.27	48390000	374	16.66	16.69	16.50	16.61	16.51	16.13	16.56	16.06
0.856	0.30	1.23	Q3TGW2	Endonuclease/exonuclease/phosphatase family domain-containing prot	Eepd1	1841	11	11	11	26.9	26.9	26.9	62.953	0.0000	93.367	26538000	169	17.53	17.34	17.91	17.43	16.89	17.32	17.41	17.39
0.406	0.30	1.23	Q8VC30	Bifunctional ATP-dependent dihydroxyacetone kinase/FAD-AMP lyase (	Dak	3907	10	10	10	31.1	31.1	31.1	59.69	0.0000	136.53	15875000	170	15.49	15.26	16.32	16.13	15.11	15.71	15.97	15.22
0.863	0.30	1.23	Q9EPK6	Nucleotide exchange factor SIL1	Sil1	5255	4	4	4	9.9	9.9	9.9	52.429	0.0004	4.4515	4727300	22	15.67	15.05	15.38	15.06	14.92	15.25	14.98	14.83
0.565	0.30	1.23	P05063	Fructose-bisphosphate aldolase C	Aldoc	646	38	28	28	78.2	62.5	62.5	39.394	0.0000	323.31	122300000	574	17.60	17.90	18.32	18.11	18.01	18.02	17.45	17.27
0.225	0.30	1.23	Q9D1G2	Phosphomevalonate kinase	Pmvk	4912	6	6	6	40.6	40.6	40.6	21.916	0.0000	14.843	3066400	21	12.88	13.34	13.18	13.00	13.61	12.01	13.78	11.80
1.577	0.30	1.23	Q9D6K9	Ceramide synthase 5	Cers5	5001	7	7	7	16.2	16.2	16.2	48.166	0.0000	58.647	13843000	78	16.04	15.62	15.81	15.81	15.68	15.51	15.50	15.42
1.091	0.30	1.23	O08582	GTP-binding protein 1	Gtpbp1	275	22	22	22	40.3	40.3	40.3	72.3	0.0000	150.25	42214000	355	17.07	16.65	17.01	17.03	16.55	16.82	16.40	16.81
1.142	0.30	1.23	O35382	Exocyst complex component 4	Exoc4	366	23	23	23	32.5	32.5	32.5	110.54	0.0000	189.12	31477000	303	15.93	15.55	16.13	15.99	15.62	15.55	15.75	15.50
0.383	0.30	1.23	Q80UW8	DNA-directed RNA polymerases I, II, and III subunit RPABC1	Polr2e	2840	4	4	4	16.2	16.2	16.2	24.57	0.0000	8.4697	5422700	55	14.50	15.26	15.91	15.32	15.04	15.32	14.97	14.48
0.588	0.29	1.23	P69566	Ran-binding protein 9	Ranbp9	1524	11	11	11	21.9	21.9	21.9	71.011	0.0000	75.449	19294000	164	15.61	15.56	15.99	15.73	15.37	15.63	15.86	14.86
0.755	0.29	1.23	Q9QZ88	Vacuolar protein sorting-associated protein 29	Vps29	5587	9	9	9	48.4	48.4	48.4	20.495	0.0000	116.87	86322000	258	18.43	18.83	18.11	18.53	17.89	18.39	18.07	18.37
1.086	0.29	1.23	P46978	Dolichyl-diphosphooligosaccharide--protein glycosyltransferase subunit	Stt3a	1113	25	25	25	29.8	29.8	29.8	80.597	0.0000	170.61	85737000	381	17.89	17.47	17.66	18.08	17.47	17.37	17.50	17.59
1.361	0.29	1.23	Q61074	Protein phosphatase 1G	Ppm1g	2245	9	9	9	20.1	20.1	20.1	58.727	0.0000	33.064	19318000	188	15.94	15.70	15.94	15.96	15.37	15.79	15.72	15.48
2.821	0.29	1.23	P50516	V-type proton ATPase catalytic subunit A	Atp6v1a	1184	56	56	56	96.1	96.1	96.1	68.325	0.0000	323.31	1364500000	2659	20.65	20.68	20.80	20.69	20.42	20.36	20.53	20.34
1.426	0.29	1.23	O35465	Peptidyl-prolyl cis-trans isomerase FKBP8	Fkbp8	373	16	16	16	54.5	54.5	54.5	43.528	0.0000	323.31	60114000	354	17.55	17.28	17.49	17.53	17.02	17.01	17.36	17.28
0.325	0.29	1.23	Q99JP0	Mitogen-activated protein kinase kinase kinase kinase 3	Map4k3	4342	5	5	4	9.1	9.1	7	101.12	0.0000	31.134	2743500	35	14.99	13.94	15.26	13.89	14.29	13.81	14.50	14.31
0.153	0.29	1.22	Q91WR3	Activating signal cointegrator 1 complex subunit 2	Ascc2	4124	6	6	6	13	13	13	85.652	0.0000	37.948	3752100	35	14.91	14.22	13.74	14.78	12.30	14.01	14.65	15.52
0.735	0.29	1.22	P39429	TNF receptor-associated factor 2	Traf2	1053	3	3	3	8.4	8.4	8.4	56.026	0.0000	33.883	9626600	53	16.04	15.69	16.47	16.50	15.82	15.87	15.96	15.89
0.202	0.29	1.22	Q99LI2	Chloride channel CLIC-like protein 1	Clcc1	4431	8	8	8	23.9	23.9	23.9	60.621	0.0000	41.397	3542300	41	12.98	12.77	12.97	12.92	12.10	14.25	11.63	12.48
0.907	0.29	1.22	Q80UM7	Mannosyl-oligosaccharide glucosidase	Mogs	2833	22	22	22	34.1	34.1	34.1	91.83	0.0000	158.53	31568000	300	16.27	15.68	16.05	16.19	15.51	15.70	15.98	15.83
0.808	0.29	1.22	Q9WV60	Glycogen synthase kinase-3 beta	Gsk3b	5733	11	9	9	41.4	35.7	35.7	46.71	0.0000	86.021	14553000	153	15.86	15.21	15.84	15.54	15.48	15.47	15.13	15.19
1.157	0.29	1.22	Q9R111	Guanine deaminase	Gda	5644	17	17	17	61.2	61.2	61.2	51.012	0.0000	169.5	54545000	321	17.43	17.10	17.03	17.40	16.88	16.78	17.18	16.96
1.162	0.29	1.22	P38060;Q8J	Hydroxymethylglutaryl-CoA lyase, mitochondrial	Hmgcl	1048	21	21	21	57.8	57.8	57.8	34.238	0.0000	323.31	119390000	617	19.17	19.09	19.59	19.28	18.96	19.18	19.01	18.82
1.178	0.29	1.22	P70290	55 kDa erythrocyte membrane protein	Mpp1	1547	38	38	38	87.3	87.3	87.3	52.227	0.0000	323.31	225220000	1136	18.78	18.45	18.55	18.52	18.49	18.03	18.43	18.18
1.735	0.29	1.22	Q8CI75	DIS3-like exonuclease 2	Dis3l2	3544	16	16	16	28.2	28.2	28.2	97.774	0.0000	68.658	16997000	181	15.98	15.91	16.25	16.08	15.69	15.71	15.74	15.92
2.292	0.29	1.22	O55131;Q9D	Septin-7	Sep.07	458	30	30	29	56.9	56.9	56.9	50.549	0.0000	323.31	244110000	1255	18.00	17.79	17.83	17.98	17.52	17.68	17.56	17.68
0.872	0.29	1.22	Q61333	Tumor necrosis factor alpha-induced protein 2	Tnfaip2	2291	15	15	15	25.9	25.9	25.9	78.101	0.0000	100.43	33158000	276	16.47	16.29	16.39	16.58	16.08	15.78	16.53	16.18
1.137	0.29	1.22	Q9R1P0	Proteasome subunit alpha type-4	Psma4	5653	18	18	18	68.6	68.6	68.6	29.47	0.0000	258.47	69020000	407	17.17	17.58	17.47	17.65	17.34	17.28	16.99	17.10
1.697	0.29	1.22	Q8CGC7	Bifunctional glutamate/proline--tRNA ligase;Glutamate--tRNA ligase;Pro	Eprs	3507	111	111	111	74.7	74.7	74.7	170.08	0.0000	323.31	401510000	2651	18.04	17.66	17.90	17.95	17.48	17.68	17.59	17.65
1.298	0.29	1.22	Q3U829	AP-5 complex subunit zeta-1	Ap5z1	1935	10	10	10	19.8	19.8	19.8	89.406	0.0000	81.697	15428000	74	16.27	16.02	16.52	16.32	16.16	15.96	15.94	15.93
0.321	0.29	1.22	Q61462	Cytochrome b-245 light chain	Cyba	2301	2	2	2	19.8	19.8	19.8	20.748	0.0000	75.219	107530000	114	19.79	19.87	19.41	19.58	19.55	20.24	19.23	18.48
1.276	0.29	1.22	Q8R3N6	THO complex subunit 1	Thoc1	3852	9	9	9	17.4	17.4	17.4	75.435	0.0000	45.897	12324000	98	15.36	15.29	15.75	15.47	15.05	15.35	15.19	15.13
1.110	0.29	1.22	Q9D0I8	mRNA turnover protein 4 homolog	Mrto4	4868	7	7	7	32.6	32.6	32.6	27.545	0.0000	24.793	8440800	89	14.78	14.43	14.74	15.06	14.46	14.41	14.56	14.44
1.341	0.29	1.22	P12265	Beta-glucuronidase	Gusb	755	37	37	37	49.7	49.7	49.7	74.194	0.0000	323.31	477460000	1070	19.78	19.93	19.75	19.74	19.42	19.28	19.60	19.76
1.505	0.29	1.22	Q9EQK5	Major vault protein	Mvp	5282	78	78	78	82.6	82.6	82.6	95.923	0.0000	323.31	457440000	1954	19.34	19.14	19.35	19.51	18.85	19.04	19.15	19.15
2.435	0.29	1.22	Q9CZU6	Citrate synthase, mitochondrial	Cs	4840	27	27	27	59.9	59.9	59.9	51.736	0.0000	323.31	461630000	814	19.68	19.69	19.87	19.64	19.48	19.38	19.50	19.37
0.716	0.29	1.22	Q9Z1N5	Spliceosome RNA helicase Ddx39b	Ddx39b	5815	26	11	11	65.7	38.1	38.1	49.035	0.0000	323.31	80388000	419	18.08	17.75	17.80	17.69	17.86	17.42	17.78	17.11
0.827	0.28	1.22	Q3UMW7	MAP kinase-activated protein kinase 3	Mapkapk3	2004	17	16	16	64.8	64.8	64.8	43.293	0.0000	105	25825000	228	16.46	16.07	16.76	16.80	16.20	16.22	16.34	16.20
0.888	0.28	1.22	A2AQ19	RNA polymerase-associated protein RTF1 homolog	Rtf1	35	10	10	10	13.1	13.1	13.1	80.796	0.0000	18.289	5692900	106	14.53	14.18	14.49	14.86	14.10	14.12	14.26	14.45
0.274	0.28	1.22	P97480;P97	Eyes absent homolog 3	Eya3	1660	3	3	3	9.2	9.2	9.2	55.973	0.0000	23.959	1061500	20	13.46	13.31	14.09	13.92	12.60	13.00	13.69	14.35
0.378	0.28	1.22	Q6P3D0	U8 snoRNA-decapping enzyme	Nudt16	2561	4	4	3	25.6	25.6	20	21.825	0.0000	17.761	2998500	39	13.64	14.03	14.99	14.75	13.81	14.08	14.27	14.12
1.003	0.28	1.22	Q64261	Cyclin-dependent kinase 6	Cdk6	2424	15	15	14	57.7	57.7	55.2	37.028	0.0000	144.39	35732000	311	17.07	16.76	17.21	17.17	16.70	16.76	16.56	17.05
0.745	0.28	1.22	Q3U0J8	TBC1 domain family member 2B	Tbc1d2b	1895	9	9	9	9.8	9.8	9.8	109.95	0.0000	19.851	8944500	74	15.33	15.16	15.76	14.98	14.79	15.06	15.15	15.11
0.562	0.28	1.22	Q8BU14	Translocation protein SEC62	Sec62	3217	7	7	7	15.6	15.6	15.6	45.58	0.0000	39.044	25954000	163	16.92	16.06	16.83	16.85	16.05	16.38	16.58	16.52
0.864	0.28	1.22	Q99LB2	Dehydrogenase/reductase SDR family member 4	Dhrs4	4413	8	8	8	25.8	25.8	25.8	29.884	0.0000	17.097	12772000	147	14.90	15.14	15.51	14.93	14.70	14.77	15.09	14.78
0.523	0.28	1.22	Q6QD59	Vesicle transport protein SEC20	Bnip1	2651	7	7	7	49.6	49.6	49.6	26.175	0.0000	103.54	16151000	139	15.84	15.56	16.38	15.48	15.40	15.40	15.96	15.37
0.870	0.28	1.22	Q60855	Receptor-interacting serine/threonine-protein kinase 1	Ripk1	2213	23	23	23	38.7	38.7	38.7	74.854	0.0000	225.3	48928000	389	16.59	16.77	16.84	16.77	16.14	16.87	16.33	16.49
1.600	0.28	1.22	Q9EPU0	Regulator of nonsense transcripts 1	Upf1	5264	44	44	44	48.6	48.6	48.6	123.97	0.0000	323.31	108270000	835	17.05	16.84	17.24	17.22	16.81	16.84	16.81	16.76
1.225	0.28	1.21	Q6A028	Switch-associated protein 70	Swap70	2496	41	41	41	62.9	62.9	62.9	68.995	0.0000	323.31	115450000	801	17.74	17.38	17.34	17.40	17.33	17.31	17.05	17.04
0.810	0.28	1.21	P58466;P58	Carboxy-terminal domain RNA polymerase II polypeptide A small phosp	Ctdsp1	1316	4	4	4	21.8	21.8	21.8	29.266	0.0000	70.75	13277000	119	16.54	16.35	16.90	16.75	16.19	16.51	16.61	16.11
0.813	0.28	1.21	Q9CWY8	Ribonuclease H2 subunit A	Rnaseh2a	4739	6	6	6	32.9	32.9	32.9	33.512	0.0000	20.558	8002800	93	15.15	15.41	15.24	15.32	14.89	15.06	15.42	14.64
0.258	0.28	1.21	Q8C5P5	5-nucleotidase domain-containing protein 1	Nt5dc1	3400	9	9	9	26.8	26.8	26.8	53.094	0.0000	34.761	3280300	39	13.31	13.44	13.72	13.11	14.19	12.32	13.39	12.55
0.512	0.28	1.21	Q6DTY7;P70	6-phosphofructo-2-kinase/fructose-2,6-bisphosphatase 4;6-phosphofruc	Pfkfb4	2511	13	13	13	38.4	38.4	38.4	54.066	0.0000	87.984	11045000	115	14.66	14.64	14.55	14.37	14.66	14.61	14.21	13.61
1.894	0.28	1.21	P42932	T-complex protein 1 subunit theta	Cct8	1084	58	58	58	83.6	83.6	83.6	59.555	0.0000	323.31	598490000	1813	19.23	19.01	19.27	19.20	18.92	18.96	18.99	18.74
0.260	0.28	1.21	P01942	Hemoglobin subunit alpha	Hba	624	6	4	4	42.3	35.9	35.9	15.085	0.0000	14.277	11636000	71	14.67	15.05	15.01	15.46	14.88	15.25	15.36	13.57
0.722	0.28	1.21	Q9DCA2	28S ribosomal protein S11, mitochondrial	Mrps11	5202	4	4	4	35.1	35.1	35.1	20.208	0.0000	79.294	3413800	51	14.90	14.81	14.28	14.60	14.11	14.68	14.47	14.21
0.848	0.28	1.21	Q6NVE8	WD repeat-containing protein 44	Wdr44	2531	5	5	5	6.2	6.2	6.2	101.55	0.0000	6.8243	2953500	30	14.44	14.17	14.42	13.93	13.96	14.11	13.64	14.14
1.162	0.28	1.21	Q1HFZ0	tRNA (cytosine(34)-C(5))-methyltransferase	Nsun2	1795	31	31	31	47.7	47.7	47.7	85.451	0.0000	176.73	79640000	642	16.89	16.55	17.12	16.91	16.66	16.64	16.48	16.58
0.332	0.28	1.21	Q6P4S6	Serine/threonine-protein kinase SIK3	Sik3	2563	4	4	4	4.7	4.7	4.7	145.78	0.0000	13.482	1734000	24	13.29	12.74	13.55	13.75	13.16	12.46	12.80	13.78
0.680	0.28	1.21	Q9D2N9	Vacuolar protein sorting-associated protein 33A	Vps33a	4946	26	26	26	52	52	52	67.554	0.0000	232.63	45380000	397	17.19	16.96	16.54	16.64	16.59	16.19	16.65	16.79
0.540	0.28	1.21	E9Q555	E3 ubiquitin-protein ligase RNF213	Rnf213	236	149	149	149	35.8	35.8	35.8	584.78	0.0000	323.31	236780000	1777	17.57	16.98	16.87	16.72	17.01	16.95	16.70	16.36
0.503	0.28	1.21	Q9QYF1	Retinol dehydrogenase 11	Rdh11	5571	14	14	14	38.9	38.9	38.9	35.147	0.0000	100.02	37154000	238	16.68	17.17	17.56	17.13	17.06	16.86	17.16	16.36
1.436	0.28	1.21	Q99MN1	Lysine--tRNA ligase	Kars	4468	37	37	37	47.6	47.6	47.6	67.839	0.0000	299.57	96053000	754	18.15	17.77	18.08	18.12	17.75	17.74	17.63	17.89
0.453	0.28	1.21	Q8C708	Transmembrane protein C16orf54 homolog		3407	3	3	3	11.1	11.1	11.1	24.532	0.0000	8.2037	2484400	46	14.62	14.17	14.10	13.75	14.40	13.73	13.42	13.99
1.090	0.28	1.21	Q99PG4	Regulator of G-protein signaling 18	Rgs18	4506	3	3	3	18.3	18.3	18.3	27.61	0.0000	11.738	3708300	69	14.27	14.06	14.09	14.09	13.87	14.08	13.51	13.95
0.483	0.28	1.21	Q7TMY8	E3 ubiquitin-protein ligase HUWE1	Huwe1	2754	37	37	37	15.2	15.2	15.2	482.63	0.0000	323.31	34529000	438	15.77	15.37	16.49	16.16	15.45	15.78	15.85	15.60

1.922	0.28	1.21	Q60770	Syntaxin-binding protein 3	Stxbp3	2205	30	30	29	54.2	54.2	53	67.942	0.0000	145.37	65371000	595	16.89	16.72	16.60	16.81	16.48	16.44	16.39	16.60
1.817	0.27	1.21	P62960;Q92	Nuclease-sensitive element-binding protein 1	Ybx1	1472	11	11	5	52.2	52.2	29.2	35.73	0.0000	323.31	35578000	272	17.55	17.52	17.25	17.53	17.08	17.28	17.19	17.20
0.967	0.27	1.21	Q60575	Kinesin-like protein KIF1B	Kif1b	2179	20	20	15	15.1	15.1	11.7	204.08	0.0000	95.719	25050000	240	16.04	15.90	16.42	16.01	15.58	15.77	15.99	15.93
1.637	0.27	1.21	Q68FL6	Methionine--tRNA ligase, cytoplasmic	Mars	2477	41	41	41	51.9	51.9	51.9	101.43	0.0000	323.31	115680000	803	17.47	17.21	17.22	17.41	16.97	16.95	17.10	17.21
0.461	0.27	1.21	A2RSJ4	UHRF1-binding protein 1-like	Uhrf1bp1l	47	6	6	6	6.3	6.3	6.3	161.94	0.0000	13.996	7116000	49	15.36	15.20	15.91	15.59	14.86	14.92	15.79	15.39
0.639	0.27	1.21	P84089	Enhancer of rudimentary homolog	Erh	1607	8	8	8	85.6	85.6	85.6	12.259	0.0000	113.15	20642000	256	16.01	16.05	15.44	15.79	15.68	15.48	15.17	15.86
0.781	0.27	1.21	Q8R3V5	Endophilin-B2	Sh3glb2	3858	19	18	18	57.8	57.8	57.8	44.503	0.0000	78.976	27387000	230	15.99	15.28	15.66	15.31	15.36	15.22	15.24	15.33
0.751	0.27	1.21	B1AXP6	Mitochondrial import receptor subunit TOM5 homolog	Tomm5	72	6	6	6	37.3	37.3	37.3	6.0372	0.0000	17.487	13751000	118	16.19	15.54	16.11	16.03	15.42	15.87	15.80	15.69
0.162	0.27	1.21	Q3TNH5	Protein FAM172A	Fam172a	1865	4	4	4	15.6	15.6	15.6	48.072	0.0000	13.602	1706900	14	13.92	13.92	11.53	13.48	13.13	12.40	13.70	12.52
0.833	0.27	1.21	Q9R1P1	Proteasome subunit beta type-3	Psmb3	5654	11	11	11	53.7	53.7	53.7	22.965	0.0000	169.58	125470000	413	19.66	19.08	19.28	19.08	19.15	19.17	18.81	18.90
0.383	0.27	1.21	P57716	Nicastrin	Ncstn	1296	9	9	9	15.7	15.7	15.7	78.491	0.0000	55.77	14817000	124	15.95	15.39	15.36	15.76	15.98	15.49	15.23	14.66
0.544	0.27	1.21	Q03963	Interferon-induced, double-stranded RNA-activated protein kinase	Eif2ak2	1729	17	17	17	36.7	36.7	36.7	58.279	0.0000	209.53	48464000	450	17.07	16.89	16.91	16.61	16.87	17.02	16.39	16.12
0.887	0.27	1.21	Q9DBW0	Cytochrome P450 4V2	Cyp4v2	5181	15	15	15	35.2	35.2	35.2	60.938	0.0000	71.145	18736000	171	15.73	15.46	15.85	15.63	15.71	15.13	15.49	15.25
0.904	0.27	1.21	O55126	Protein NipSnap homolog 2	Gbas	456	11	11	11	39.1	39.1	39.1	32.932	0.0000	33.339	32284000	267	16.42	15.94	16.47	16.16	15.80	16.17	15.86	16.07
0.327	0.27	1.21	Q810J8	Zinc finger FYVE domain-containing protein 1	Zfyve1	2928	11	11	11	21.5	21.5	21.5	86.939	0.0000	68.267	7636600	108	14.00	14.58	15.43	14.29	14.31	14.34	14.69	13.89
0.292	0.27	1.21	Q8C050	Ribosomal protein S6 kinase alpha-5	Rps6ka5	3324	19	19	19	30.9	30.9	30.9	96.582	0.0000	175.12	22607000	155	16.54	16.91	16.69	16.82	16.90	16.86	16.78	15.35
1.208	0.27	1.21	Q9R099	Transducin beta-like protein 2	Tbl2	5618	12	12	12	29.4	29.4	29.4	49.583	0.0000	72.98	19090000	240	15.94	15.65	16.17	15.95	15.55	15.59	15.76	15.74
0.456	0.27	1.21	A2AB59	Rho GTPase-activating protein 27	Arhgap27	15	12	12	12	21.2	21.2	21.2	97.047	0.0000	70.654	9977300	77	15.57	14.67	15.44	15.81	15.00	15.39	15.02	14.99
1.499	0.27	1.20	P01899;P01	H-2 class I histocompatibility antigen, D-B alpha chain;H-2 class I histoc	H2-D1;H2-L	621	26	26	18	53.9	53.9	48.3	40.836	0.0000	222.05	309290000	670	19.48	19.51	19.18	19.30	19.24	19.12	19.03	19.00
1.516	0.27	1.20	P60229	Eukaryotic translation initiation factor 3 subunit E	Eif3e	1352	25	25	25	55.7	55.7	55.7	52.22	0.0000	187.06	106230000	541	17.61	17.91	17.79	17.83	17.59	17.39	17.68	17.40
0.447	0.27	1.20	Q8K3G5	Inactive serine/threonine-protein kinase VRK3	Vrk3	3700	12	12	12	37.3	37.3	37.3	50.829	0.0000	24.916	7999300	89	14.16	14.02	14.63	14.25	13.96	14.18	14.48	13.37
1.716	0.27	1.20	Q99MK8;Q3	Beta-adrenergic receptor kinase 1	Adrbk1	4467	36	36	36	49.3	49.3	49.3	79.638	0.0000	279.03	85519000	828	17.34	17.08	17.36	17.37	17.04	17.10	16.88	17.06
0.822	0.27	1.20	Q8BSF4	Phosphatidylserine decarboxylase proenzyme;Phosphatidylserine deca	Pisd	3187	7	7	7	20.4	20.4	20.4	45.926	0.0000	28.85	4466600	46	13.84	14.10	14.13	14.38	13.70	14.16	13.88	13.64
0.332	0.27	1.20	Q99LP6	GrpE protein homolog 1, mitochondrial	Grpel1	4441	10	10	10	50.2	50.2	50.2	24.307	0.0000	129.88	9478100	120	15.38	15.78	15.01	15.69	15.48	15.89	14.75	14.66
1.302	0.27	1.20	Q99K48	Non-POU domain-containing octamer-binding protein	Nono	4362	46	44	44	75.9	75.9	75.9	54.54	0.0000	323.31	252450000	1208	18.82	18.52	18.49	18.69	18.53	18.41	18.35	18.16
1.564	0.27	1.20	Q9QXS1	Plectin	Plec	5548	472	472	466	78.1	78.1	77.2	534.18	0.0000	323.31	2618600000	12403	19.29	18.96	19.27	19.29	18.89	19.06	18.88	18.91
0.631	0.26	1.20	Q80YV2	Nuclear-interacting partner of ALK	Zc3hc1	2907	6	6	6	17.6	17.6	17.6	55.196	0.0000	14.903	6480000	74	14.88	14.66	15.51	15.19	14.81	14.87	14.92	14.58
1.089	0.26	1.20	Q9CQF9	Prenylcysteine oxidase	Pcyox1	4597	20	20	20	44.6	44.6	44.6	56.494	0.0000	242.02	64896000	406	17.15	17.14	17.11	16.78	16.71	16.59	16.83	17.00
0.948	0.26	1.20	P14869	60S acidic ribosomal protein P0	Rplp0	787	22	22	22	64.4	64.4	64.4	34.216	0.0000	323.31	375820000	1036	19.43	19.45	19.30	19.80	19.24	18.97	19.38	19.35
0.663	0.26	1.20	P97864	Caspase-7;Caspase-7 subunit p20;Caspase-7 subunit p11	Casp7	1681	5	5	5	18.8	18.8	18.8	34.06	0.0000	23.143	5812800	71	15.34	14.92	15.51	15.74	15.11	14.92	15.29	15.15
1.375	0.26	1.20	Q9QZL0	Receptor-interacting serine/threonine-protein kinase 3	Ripk3	5600	15	15	15	39.3	39.3	39.3	53.322	0.0000	151.8	37977000	245	16.60	16.51	16.66	16.65	16.19	16.45	16.17	16.56
1.791	0.26	1.20	Q9Z1Q9	Valine--tRNA ligase	Vars	5819	61	61	61	59.3	59.3	59.3	140.21	0.0000	323.31	249030000	1361	18.91	18.58	18.71	18.82	18.47	18.59	18.49	18.44
0.477	0.26	1.20	Q6ZQ73	Cullin-associated NEDD8-dissociated protein 2	Cand2	2691	2	1	1	1.9	1.2	1.2	135.63	0.0004	4.9232	5886700	81	14.94	14.67	15.51	14.92	15.11	14.55	14.38	14.96
0.224	0.26	1.20	Q4FK66	Pre-mRNA-splicing factor 38A	Prpf38a	2057	5	5	5	16	16	16	37.436	0.0000	28.254	7912200	68	15.97	15.49	15.53	15.80	14.55	16.32	14.77	16.11
0.513	0.26	1.20	P04202	Transforming growth factor beta-1;Latency-associated peptide	Tgfb1	637	11	11	11	30	30	30	44.309	0.0000	41.037	13864000	130	15.23	15.06	15.64	15.83	14.95	15.32	14.92	15.54
1.326	0.26	1.20	Q80VA0	N-acetylgalactosaminyltransferase 7	Galnt7	2847	15	15	15	27.1	27.1	27.1	75.418	0.0000	99.928	33298000	258	16.99	16.77	17.12	16.97	16.73	16.82	16.49	16.78
1.120	0.26	1.20	Q8R0H9	ADP-ribosylation factor-binding protein GGA1	Gga1	3760	16	16	15	33.2	33.2	32.1	69.971	0.0000	163.33	39672000	340	16.91	16.55	16.81	17.12	16.56	16.63	16.60	16.56
1.711	0.26	1.20	Q9Z0H4	CUGBP Elav-like family member 2	Celf2	5771	17	17	15	32.3	32.3	28.1	54.27	0.0000	162.27	79207000	573	17.25	17.08	17.22	17.35	17.08	16.85	17.06	16.88
0.422	0.26	1.20	Q5M8N0	CB1 cannabinoid receptor-interacting protein 1	Cnrip1	2113	5	5	5	53.7	53.7	53.7	18.612	0.0000	49.026	2617400	29	14.10	14.06	14.31	14.31	14.06	13.16	14.28	14.25
0.383	0.26	1.20	Q9JHP7	KDEL motif-containing protein 1	Kdelc1	5368	5	5	5	13.7	13.7	13.7	57.985	0.0000	52.766	3424500	54	14.60	13.85	14.74	14.64	13.91	14.37	14.71	13.80
0.424	0.26	1.20	Q8BL97	Serine/arginine-rich splicing factor 7	Srsf7	3114	7	6	6	27	27	27	30.817	0.0000	70.904	48394000	178	17.49	18.00	16.77	17.41	16.92	17.38	17.17	17.16
0.404	0.26	1.20	Q9DC71	28S ribosomal protein S15, mitochondrial	Mrps15	5201	10	10	10	37.2	37.2	37.2	29.463	0.0000	16.428	5272700	74	14.21	14.18	14.38	14.54	14.28	13.34	14.61	14.05
1.318	0.26	1.20	O35350	Calpain-1 catalytic subunit	Capn1	361	32	32	32	50.1	50.1	50.1	82.105	0.0000	298.56	90110000	575	17.33	17.27	17.63	17.61	17.12	17.12	17.28	17.29
0.921	0.26	1.20	Q8K1M6	Dynamin-1-like protein	Dnm1l	3630	38	38	38	60.5	60.5	60.5	82.657	0.0000	323.31	119400000	853	17.56	17.07	17.56	17.51	16.84	17.17	17.19	17.22
0.974	0.26	1.20	Q3UQ44	Ras GTPase-activating-like protein IQGAP2	Iqgap2	2016	67	64	64	46.1	44.4	44.4	180.53	0.0000	323.31	135090000	1261	17.57	17.03	17.48	17.44	16.95	17.25	17.19	17.12
0.598	0.26	1.20	P17439	Glucosylceramidase	Gba	825	24	24	24	51.1	51.1	51.1	57.621	0.0000	323.31	123530000	554	17.76	17.51	17.72	17.87	17.39	16.97	17.60	17.86
1.225	0.26	1.20	O08573	Galectin-9	Lgals9	273	12	12	12	48.7	48.7	48.7	40.035	0.0000	223.23	57999000	214	17.77	17.33	17.65	17.50	17.26	17.38	17.17	17.41
0.685	0.26	1.20	Q8R242	Di-N-acetylchitobiase	Ctbs	3805	5	5	5	20.2	20.2	20.2	41.295	0.0000	90.108	7625300	115	15.03	15.42	14.61	14.96	14.54	14.88	14.74	14.83
0.627	0.26	1.19	O54734	Dolichyl-diphosphooligosaccharide--protein glycosyltransferase 48 kDa	Ddost	418	18	18	18	48.1	48.1	48.1	49.027	0.0000	323.31	150040000	490	18.35	18.61	18.20	18.62	18.49	17.71	18.28	18.27
0.163	0.26	1.19	P49135	TFIIH basal transcription factor complex helicase XPB subunit	Ercc3	1155	3	3	3	7.2	7.2	7.2	89.125	0.0000	10.916	1729500	15	13.42	13.35	14.12	14.05	13.73	14.36	14.01	11.81
0.793	0.26	1.19	Q8R5C5	Beta-centractin	Actr1b	3884	24	11	11	78.7	46.8	46.8	42.281	0.0000	194.28	35737000	236	17.10	17.16	17.43	17.05	16.90	16.94	17.27	16.60
1.069	0.26	1.19	Q8VDW0	ATP-dependent RNA helicase DDX39A	Ddx39a	3975	27	27	11	65.6	65.6	39.6	49.067	0.0000	269.37	158020000	577	18.39	18.32	18.49	18.30	18.40	17.99	18.21	17.88
0.138	0.26	1.19	P0C7N9	Proteasome assembly chaperone 4	Psmg4	708	4	4	4	44.7	44.7	44.7	13.974	0.0000	12.627	3430500	40	15.16	14.83	12.53	15.34	14.39	13.50	14.65	14.30
0.468	0.26	1.19	Q9DB60	Prostamide/prostaglandin F synthase	Fam213b	5129	5	5	5	26.9	26.9	26.9	21.67	0.0000	14.688	13875000	111	16.29	16.39	16.57	16.66	15.56	16.63	16.42	16.28
1.294	0.26	1.19	Q8K021	Secretory carrier-associated membrane protein 1	Scamp1	3596	10	10	10	36.4	36.4	36.4	38.028	0.0000	323.31	28814000	319	16.30	16.25	16.69	16.37	16.19	16.09	16.23	16.08
0.791	0.26	1.19	Q68FH4	N-acetylgalactosamine kinase	Galk2	2475	23	23	23	56.3	56.3	56.3	50.503	0.0000	323.31	67146000	390	17.21	17.18	17.80	17.47	17.20	17.20	17.28	16.96
0.601	0.26	1.19	Q8VHH5	Arf-GAP with GTPase, ANK repeat and PH domain-containing protein 3	Agap3	4019	8	8	6	12.9	12.9	10.2	97.964	0.0000	64.7	9859000	181	14.63	14.68	14.91	15.26	14.48	15.00	14.35	14.62
0.488	0.26	1.19	Q8C570	mRNA export factor	Rae1	3394	11	11	11	35.1	35.1	35.1	40.965	0.0000	66.464	31999000	235	16.48	15.75	16.80	16.68	16.26	16.14	16.09	16.21
0.466	0.26	1.19	Q3UA37	Glutamine-rich protein 1	Qrich1	1939	6	6	6	11.8	11.8	11.8	86.555	0.0000	30.165	6605100	74	14.34	14.87	14.68	14.53	14.06	14.13	15.00	14.20
0.813	0.25	1.19	O88447	Kinesin light chain 1	Klc1	530	30	30	24	46.2	46.2	39.6	61.449	0.0000	135.45	36882000	405	15.65	16.20	16.24	16.23	15.95	15.89	15.66	15.79
1.293	0.25	1.19	O88342	WD repeat-containing protein 1	Wdr1	523	52	52	52	79	79	79	66.406	0.0000	323.31	1206500000	2665	20.23	20.42	20.04	20.03	19.93	20.02	19.96	19.80
0.817	0.25	1.19	Q64378	Peptidyl-prolyl cis-trans isomerase FKBP5;Peptidyl-prolyl cis-trans isom	Fkbp5	2439	18	18	18	42.1	42.1	42.1	50.966	0.0000	23.362	16238000	141	15.47	15.50	15.97	15.97	15.66	15.48	15.38	15.37
0.887	0.25	1.19	Q9CX00	IST1 homolog	Ist1	4742	16	16	16	46.4	46.4	46.4	39.468	0.0000	278.44	28260000	286	16.08	15.91	15.90	16.50	15.84	15.76	15.93	15.83
0.175	0.25	1.19	Q8VI63	MOB kinase activator 2	Mob2	4029	5	5	4	20	20	20	26.851	0.0000	9.5597	4890500	48	15.20	14.74	14.66	13.01	14.71	14.13	14.43	13.32
0.528	0.25	1.19	P46061	Ran GTPase-activating protein 1	Rangap1	1101	19	19	19	37	37	37	63.53	0.0000	169.66	37328000	324	16.36	16.28	16.95	16.76	16.09	16.68	16.50	16.05
0.206	0.25	1.19	P03958	Adenosine deaminase	Ada	633	6	6	5	28.4	28.4	25.6	39.991	0.0000	21.323	3813400	27	13.98	14.03	14.11	14.39	13.65	14.96	14.21	12.68
0.912	0.25	1.19	P63168	Dynein light chain 1, cytoplasmic	Dynll1	1495	10	10	7	74.2	74.2	43.8	10.366	0.0000	191.42	51690000	321	17.27	17.36	17.39	17.60	17.07	16.94	17.09	17.51
0.932	0.25	1.19	A2ADY9;Q9	Protein DDI1 homolog 2	Ddi2	18	19	19	19	58.4	58.4	58.4	44.59	0.0000	316.11	65085000	469	17.29	17.21	17.68	17.48	17.23	17.17	17.34	16.92
0.230	0.25	1.19	Q8R1S0	Ubiquinone biosynthesis monooxygenase COQ6, mitochondrial	Coq6	3797	11	11	11	27.5	27.5	27.5	51.392	0.0000	53.218	17030000	125	17.54	17.07	17.81	16.94	16.76	17.77	16.14	17.69
1.905	0.25	1.19	O35639	Annexin A3	Anxa3	390	38	38	38	82.7	82.7	82.7	36.384	0.0000	323.31	416320000	1370	19.51	19.43	19.52	19.73	19.23	19.38	19.30	19.27
0.174	0.25	1.19	O70293;Q8\	G protein-coupled receptor kinase 6	Grk6	483	5	5	4	10.9	10.9	8.2	65.978	0.0000	47.872	6583500	77	15.38	15.70	16.08	15.96	14.64	16.88	15.90	14.70
0.507	0.25	1.19	Q8R164	Valacyclovir hydrolase	Bphl	3779	15	15	15	60.8	60.8	60.8	32.851	0.0000	70.509	23840000	203	16.24	15.46	16.09	15.37	15.50	15.62	15.40	15.64
0.495	0.25	1.19	Q9D3P8	Plasminogen receptor (KT)	Plgrkt	4968	9	9	9	41.5	41.5	41.5	17.261	0.0000	20.322	28815000	136	17.06	16.59	17.26	17.04	16.66	16.26	17.10	16.92
1.295	0.25	1.19	Q61187	Tumor susceptibility gene 101 protein	Tsg101	2270	18	18	18	53.2	53.2	53.2	44.123	0.0000	162	34855000	302	16.25	16.16	16.25	16.57	16.07	16.12	16.11	15.93
0.291	0.25	1.19	P33610	DNA primase large subunit	Prim2	1003	11	11	11	25.7	25.7	25.7	58.408	0.0000	19.816	4565700	39	14.78	13.69	13.57	13.91	13.39	13.70	13.48	14.39
0.861	0.25	1.19	Q9R0P3	S-formylglutathione hydrolase	Esd	5631	29	29	29	96.1	96.1	96.1	31.319	0.0000	323.31	151500000	1812	20.81	20.89	21.41	21.05	20.65	20.91	20.84	20.77
1.700	0.25	1.19	Q7TPR4	Alpha-actinin-1	Actn1	2771	72	46	45	77.7	58.6	57	103.07	0.0000	323.31	225670000	1167	18.50	18.35	18.36	18.37	18.05	18.02	18.32	18.21
2.312	0.25	1.19	Q8C0E2	Vacuolar protein sorting-associated protein 26B	Vps26b	3334	12	11	11	38.7	36.6	36.6	39.124	0.0000	33.515	20563000	124	16.00	15.87	15.78	15.96	15.66	15.58	15.66	15.73
0.696	0.25	1.19	E9Q5C9		Nolc1	238	9	9	8	13	13	12	73.697	0.0000	17.598	13545000	122	15.98	15.79	16.05	16.32	15.46	16.10	15.78	15.83
1.213	0.25	1.19	Q8BTV2	Cleavage and polyadenylation specificity factor subunit 7	Cpsf7	3208	13	13	13	35.7	35.7	35.7	52.01	0.0000	219.22	20126000	238	16.18	15.94	15.80	16.05	15.84	15.56	15.73	15.85
0.716	0.25	1.19	Q99KY4;Q8	Cyclin-G-associated kinase	Gak	4400	29	29	28	30.3	30.3	29.1	143.64	0.0000	84.106	55757000	423	16.95	16.27	16.54	16.70	16.26	16.27	16.32	16.63
0.174	0.25	1.19	P35505	Fumarylacetoacetase	Fah	1023	5	5	5	20	20	20	46.175	0.0000	10.364	2547100	18	12.88	13.49	13.38	12.23	11.36	13.07	13.21	13.36
0.387	0.25	1.19	Q99M71	Mammalian ependymin-related protein 1	Epdr1	4457	3	3	2	16.1	16.1	10.7	25.485	0.0002	5.8667	1910600	33	13.75	13.76	14.53	14.28	13.83	14.38	13.44	13.69
0.508	0.25	1.19	O35643	AP-1 complex subunit beta-1	Ap1b1	392	45	22	22	49	30.2	30.2	103.93	0.0000	323.31	81607000	403	17.86	17.61	18.41	18.23	17.60	18.07	17.53	17.93
1.682	0.25	1.19	P47754	F-actin-capping protein subunit alpha-2	Capza2	1120	27	27	22	89.2	89.2	79	32.967	0.0000	323.31	445770000	1001	20.02	20.10	20.20	20.25	19.78	19.82	19.94	20.05
0.947	0.25	1.19	Q9D0W5	Peptidyl-prolyl cis-trans isomerase-like 1	Ppil1	4889	7	7	7	44.6	44.6	44.6	18.237	0.0000	39.31	31733000	203	16.72	16.72	17.24	16.98	16.60	16.59	16.76	16.73
0.696	0.24	1.18	Q9JKR6	Hypoxia up-regulated protein 1	Hyou1	5458	50	50	50	51.4	51.4	51.4	111.18	0.0000	323.31	315180000	1485	19.26	18.97	19.62	19.57	18.96	18.99	19.22	19.26
1.244	0.24	1.18	Q8CIE6	Coatomer subunit alpha;Xenin;Proxenin	Copa	3550	73	73	73	61.8	61.8	61.8	138.43	0.0000	323.31	412070000	1819	19.28	18.82	19.15	19.25	18.87	18.86	18.94	18.82
0.964	0.24	1.18	P17426	AP-2 complex subunit alpha-1	Ap2a1	822	46	46	38	58.4	58.4	48.5	107.66	0.0000	323.31	169650000	1144	18.00	18.16	18.33	18.44	18.05	17.73	18.11	18.07
0.757	0.24	1.18	Q8BSE0	Regulator of microtubule dynamics protein 2	Rmdn2	3186	2	2	2	6.8	6.8	6.8	47.016	0.0002	5.6962	4256500	56	14.78	14.66	15.12	14.51	14.58	14.36	14.76	14.40
0.516	0.24	1.18	P70122	Ribosome maturation protein SBDS	Sbds	1526	18	18	18	68.8	68.8	68.8	28.78	0.0000	205.26	46726000	330	16.60	16.12	16.20	16.75	16.30	16.16	16.48	15.75
1.768	0.24	1.18	P10107	Annexin A1	Anxa1	718	62	62	61	93.9	93.9	93.9	38.734	0.0000	323.31	3145400000	3743	22.83	22.56	22.70	22.67	22.45	22.57	22.46	22.32
0.938	0.24	1.18	P26040	Ezrin	Ezr	915	56	39	39	76.6	59	59	69.406	0.0000	323.31	236140000	1373	18.19	17.91	18.26	18.46	17.82	17.91	18.00	18.13
0.481	0.24	1.18	P35276	Ras-related protein Rab-3D	Rab3d	1012	6	5	4	33.8	30.1	21.5	24.416	0.0000	21.681	9082200	74	15.66	15.70	15.13	15.64	15.74	14.90	15.43	15.09
1.143	0.24	1.18	P47802	Metaxin-1	Mtx1	1124	11	11	11	39.7	39.7	39.7	35.623	0.0000	40.676	26014000	241	16.65	16.47	16.68	16.65	16.19	16.27	16.65	16.38
0.546	0.24	1.18	Q99LM2	CDK5 regulatory subunit-associated protein 3	Cdk5rap3	4440	11	11	11	26.2	26.2	26.2	56.99	0.0000	24.073	13779000	149	15.82	15.33	15.57	15.96	15.02	15.75	15.51	15.45
0.213	0.24	1.18	P15920	V-type proton ATPase 116 kDa subunit a isoform 2	Atp6v0a2	803	5	5	5	6.7	6.7	6.7	98.144	0.0017	3.2905	1508100	11	13.26	12.94	13.28	13.33	12.08	12.39	14.02	13.34
1.022	0.24	1.18	O55143	Sarcoplasmic/endoplasmic reticulum calcium ATPase 2	Atp2a2	462	45	45	32	42.6	42.6	29.3	114.86	0.0000	323.31	271560000	1265	18.04	17.73	18.18	18.15	17.78	17.62	17.95	17.79
0.983	0.24	1.18	Q9D8U8	Sorting nexin-5	Snx5	5076	49	49	47	85.1	85.1	83.7	46.797	0.0000	323.31	578660000	1635	20.97	20.77	21.00	20.90	20.73	20.97	20.47	20.51
0.876	0.24	1.18	P08103	Tyrosine-protein kinase HCK	Hck	679	29	28	28	58.8	58.8	58.8	59.129	0.0000	224.05	116870000	612	17.97	17.82	17.42	17.66	17.67	17.51	17.35	17.38
1.881	0.24	1.18	Q03265	ATP synthase subunit alpha, mitochondrial	Atp5a1	1724	69	69	69	79.7	79.7	79.7	59.752	0.0000	323.31	2838600000	4330	21.17	21.02	21.10	21.28	20.80	21.00	20.90	20.92
0.889	0.24	1.18	Q3U9G9	Lamin-B receptor	Lbr	1936	22	22	22	31.8	31.8	31.8	71.439	0.0000	149	40757000	316	16.71	16.46	16.28	16.86	16.38	16.45	16.26	16.27
0.603	0.24	1.18	P48193	Protein 4.1	Epb41	1141	26	26	23	30.1	30.1	27.7	95.91	0.0000	66.011	27489000	223	15.94	15.56	16.27	15.98	16.01	15.74	15.44	15.59
0.807	0.24	1.18	Q9DAR7	m7GpppX diphosphatase	Dcps	5109	21	21	21	59.8	59.8	59.8	38.988	0.0000	113.83	54933000	293	17.06	17.05	17.31	17.22	17.10	17.12	16.54	16.92
0.492	0.24	1.18	Q8BH61	Coagulation factor XIII A chain	F13a1	3017	29	29	29	43.2	43.2	43.2	83.206	0.0000	122.93	86112000	369	17.32	17.16	16.59	16.93	16.68	16.38	16.91	17.08
0.241	0.24	1.18	Q9WV03;Q5	Protein FAM50A	Fam50a	5728	14	14	13	37.2	37.2	33.6	40.251	0.0000	28.288	10661000	93	14.44	14.97	13.64	14.76	14.18	13.48	14.79	14.41
0.412	0.24	1.18	Q80XP8	Protein FAM76B	Fam76b	2888	5	5	5	14.5	14.5	14.5	38.555	0.0000	8.8249	3569000	59	14.17	13.66	14.59	14.54	13.92	14.38	13.72	13.98
0.611	0.24	1.18	Q8BH86	UPF0317 protein C14orf159 homolog, mitochondrial		3022	20	20	20	39.7	39.7	39.7	66.365	0.0000	162.61	34185000	320	16.06	15.98	16.71	16.57	16.08	16.01	16.13	16.14
0.347	0.24	1.18	Q08857	Platelet glycoprotein 4	Cd36	1767	10	10	10	17.8	17.8	17.8	52.697	0.0000	69.66	28927000	153	16.94	16.77	17.26	17.25	16.34	16.37	17.33	17.22
2.369	0.24	1.18	Q6P4T2	U5 small nuclear ribonucleoprotein 200 kDa helicase	Snrnp200	2566	70	70	70	43.5	43.5	43.5	244.54	0.0000	323.31	146540000	1067	17.50	17.37	17.52	17.55	17.23	17.28	17.32	17.16
0.623	0.24	1.18	Q9DBL7	Bifunctional coenzyme A synthase;Phosphopantetheine adenylyltransfe	Coasy	5161	13	13	13	35.7	35.7	35.7	62.022	0.0000	107.86	21084000	178	16.04	15.64	15.44	15.69	15.34	15.68	15.16	15.69
0.754	0.24	1.18	O88508	DNA (cytosine-5)-methyltransferase 3A	Dnmt3a	536	10	10	10	14.9	14.9	14.9	101.67	0.0000	48.94	13596000	169	15.26	15.07	15.60	15.05	14.91	14.98	15.26	14.89
0.928	0.24	1.18	P56382	ATP synthase subunit epsilon, mitochondrial	Atp5e	1283	8	8	8	78.8	78.8	78.8	5.8378	0.0000	12.971	41423000	183	17.86	17.66	17.98	18.05	17.42	17.71	17.88	17.59
0.529	0.24	1.18	Q9CR62	Mitochondrial 2-oxoglutarate/malate carrier protein	Slc25a11	4674	22	22	22	71	71	71	34.155	0.0000	238.4	138160000	584	18.80	18.05	18.13	18.47	18.30	17.95	17.91	18.35

1.738	0.23	1.18	P25799	Nuclear factor NF-kappa-B p105 subunit;Nuclear factor NF-kappa-B p5(	Nfkb1	911	19	19	19	29.9	29.9	29.9	105.61	0.0000	208.29	28864000	252	16.58	16.68	16.42	16.65	16.46	16.35	16.34	16.23
0.849	0.23	1.18	O35593	26S proteasome non-ATPase regulatory subunit 14	Psmd14	381	13	13	13	55.8	55.8	55.8	34.577	0.0000	322.53	144240000	531	18.54	18.46	18.48	18.53	18.46	18.21	18.50	17.91
0.212	0.23	1.18	Q8R420;E9F	ATP-binding cassette sub-family A member 3	Abca3	3864	13	13	13	10.9	10.9	10.9	191.97	0.0000	33.534	95833000	74	19.48	18.80	20.45	20.22	19.36	20.03	19.69	18.94
0.788	0.23	1.18	Q8CHP8	Phosphoglycolate phosphatase	Pgp	3528	15	15	14	65.4	65.4	64.5	34.54	0.0000	191.06	64310000	337	17.70	17.80	18.24	18.13	17.92	17.60	17.65	17.77
0.843	0.23	1.18	Q9Z1Z0;RE\	General vesicular transport factor p115	Uso1	5826	29	29	29	40.6	40.6	40.6	106.98	0.0000	243.83	150200000	781	17.74	17.48	18.00	17.96	17.40	17.51	17.73	17.60
0.495	0.23	1.18	Q925E7;Q6Z	Serine/threonine-protein phosphatase 2A 55 kDa regulatory subunit B d	Ppp2r2d	4305	11	6	6	31.1	16.1	16.1	51.957	0.0000	7.9746	2320700	15	13.33	14.02	13.79	13.68	13.91	13.43	13.40	13.15
0.348	0.23	1.18	Q6DFV7	Nuclear receptor coactivator 7	Ncoa7	2504	4	4	4	6.6	6.6	6.6	106.36	0.0000	6.3131	2807400	22	13.91	14.26	14.10	14.24	13.79	14.34	14.28	13.15
0.586	0.23	1.18	P97315	Cysteine and glycine-rich protein 1	Csrp1	1623	8	8	8	59.1	59.1	59.1	20.583	0.0000	138.27	73794000	300	17.97	17.49	18.21	17.83	17.42	17.93	17.69	17.53
1.025	0.23	1.17	Q60932	Voltage-dependent anion-selective channel protein 1	Vdac1	2224	30	30	30	86.1	86.1	86.1	32.351	0.0000	323.31	452670000	1408	20.12	20.04	20.38	20.39	19.80	19.97	20.13	20.10
0.108	0.23	1.17	P08071	Lactotransferrin	Ltf	676	12	12	12	20.8	20.8	20.8	77.837	0.0000	58.549	19551000	85	14.80	14.75	15.12	15.38	15.81	15.65	15.19	12.47
0.940	0.23	1.17	Q9CZN7	Serine hydroxymethyltransferase	Shmt2	4834	35	35	34	75	75	75	55.758	0.0000	323.31	169470000	893	17.51	17.61	17.83	17.73	17.55	17.17	17.39	17.66
0.589	0.23	1.17	Q9ER00	Syntaxin-12	Stx12	5291	14	14	13	45.6	45.6	45.6	31.195	0.0000	283.75	69997000	407	17.59	17.20	17.96	17.66	17.10	17.57	17.42	17.39
0.422	0.23	1.17	P49615	Cyclin-dependent-like kinase 5	Cdk5	1166	13	12	12	52.7	50	50	33.288	0.0000	42.999	19319000	161	16.07	16.10	17.01	16.45	16.05	16.51	16.09	16.07
0.585	0.23	1.17	Q9QZ73	DCN1-like protein 1	Dcun1d1	5586	4	4	4	17.4	17.4	17.4	30.097	0.0000	33.289	11377000	73	15.82	15.93	16.45	16.55	15.98	15.99	16.01	15.86
0.425	0.23	1.17	Q9WTK5	Nuclear factor NF-kappa-B p100 subunit;Nuclear factor NF-kappa-B p5:	Nfkb2	5676	22	22	22	35.8	35.8	35.8	96.831	0.0000	173.84	25286000	253	16.10	15.66	15.62	15.48	15.30	16.00	15.58	15.06
0.531	0.23	1.17	P19096	Fatty acid synthase;[Acyl-carrier-protein] S-acetyltransferase;[Acyl-carri	Fasn	842	134	134	134	63	63	63	272.43	0.0000	323.31	639710000	3101	19.05	18.58	18.50	18.49	18.75	18.59	18.26	18.10
0.271	0.23	1.17	O35495;Q3\	Cyclin-dependent kinase 14	Cdk14	377	6	5	5	13.6	11.9	11.9	52.995	0.0000	8.2641	2737900	30	13.67	13.35	14.26	13.61	13.95	13.20	12.81	14.01
0.653	0.23	1.17	Q99P91	Transmembrane glycoprotein NMB	Gpnmb	4504	23	23	23	36.8	36.8	36.8	63.675	0.0000	272.84	1296100000	1154	22.49	22.26	22.44	22.63	22.29	21.80	22.29	22.52
0.446	0.23	1.17	Q9D6W8	Uncharacterized protein C17orf59 homolog		5011	4	4	4	18.9	18.9	18.9	38.011	0.0000	35.209	3521800	37	14.37	14.28	15.01	14.10	14.25	13.86	14.31	14.42
0.877	0.23	1.17	Q91VR5	ATP-dependent RNA helicase DDX1	Ddx1	4073	39	39	39	61.5	61.5	61.5	82.499	0.0000	323.31	115830000	811	17.43	17.11	17.27	17.23	17.29	17.13	16.94	16.77
0.187	0.23	1.17	Q3UDW8	Heparan-alpha-glucosaminide N-acetyltransferase	Hgsnat	1951	5	5	5	9.3	9.3	9.3	72.503	0.0000	71.434	6327600	75	15.86	14.83	14.20	14.80	14.96	15.30	13.76	14.76
0.113	0.23	1.17	Q7TMV3	FAST kinase domain-containing protein 5	Fastkd5	2750	3	3	3	5.6	5.6	5.6	86.599	0.0000	11.123	1709400	12	13.79	12.86	13.57	14.24	12.41	13.60	12.28	15.26
1.006	0.23	1.17	Q9Z2U0;Q9	Proteasome subunit alpha type-7	Psma7	5864	23	23	23	70.6	70.6	70.6	27.855	0.0000	323.31	226740000	663	19.09	18.99	18.77	19.01	18.83	18.49	18.93	18.71
0.741	0.23	1.17	Q6ZWY3	40S ribosomal protein S27-like	Rps27l	2710	6	3	3	41.7	15.5	15.5	9.4771	0.0000	15.159	52000000	122	18.56	18.41	18.98	18.53	18.36	18.57	18.46	18.17
0.340	0.23	1.17	Q9D8S4	Oligoribonuclease, mitochondrial	Rexo2	5070	9	9	9	34.2	34.2	34.2	26.738	0.0000	35.032	14372000	83	16.79	16.39	15.99	16.60	16.22	15.61	16.72	16.29
0.203	0.23	1.17	Q05117	Tartrate-resistant acid phosphatase type 5	Acp5	1738	3	3	3	15.6	15.6	15.6	36.807	0.0000	41.956	6101700	36	15.32	15.06	15.19	15.27	15.51	15.20	15.54	13.69
1.186	0.23	1.17	P80313	T-complex protein 1 subunit eta	Cct7	1590	47	47	47	77.2	77.2	77.2	59.652	0.0000	323.31	466630000	1631	18.35	18.32	18.59	18.65	18.32	18.09	18.24	18.35
0.417	0.22	1.17	Q05921	2-5A-dependent ribonuclease	Rnasel	1745	6	6	6	11.6	11.6	11.6	83.274	0.0000	17.385	5245300	51	14.29	13.94	14.54	14.20	13.63	14.43	13.71	14.31
0.130	0.22	1.17	Q9QZV9	NTF2-related export protein 1	Nxt1	5608	2	2	2	25.7	25.7	25.7	15.847	0.0000	9.5023	2088000	25	14.17	14.63	13.16	14.02	13.54	15.27	13.76	12.51
0.210	0.22	1.17	Q8VDS4	Regulation of nuclear pre-mRNA domain-containing protein 1A	Rprd1a	3970	2	2	2	10.9	10.9	10.9	35.7	0.0000	9.5828	1584400	35	12.92	13.10	13.61	13.41	13.36	13.44	13.51	11.85
1.448	0.22	1.17	Q3UJD6	Ubiquitin carboxyl-terminal hydrolase 19	Usp19	1984	21	21	21	29.6	29.6	29.6	150.55	0.0000	135.28	24951000	234	15.66	15.72	15.93	15.95	15.57	15.64	15.66	15.49
0.136	0.22	1.17	P22437	Prostaglandin G/H synthase 1	Ptgs1	875	2	2	2	5.6	5.6	5.6	69.042	0.0000	11.966	1823400	18	14.34	13.99	14.54	12.23	14.21	13.44	13.86	12.70
0.420	0.22	1.17	A1L314	Macrophage-expressed gene 1 protein	Mpeg1	4	24	24	24	33.9	33.9	33.9	78.39	0.0000	253.16	336230000	813	19.35	19.38	20.23	19.74	19.68	19.56	19.43	19.14
0.395	0.22	1.17	P21956	Lactadherin	Mfge8	869	16	16	16	42.1	42.1	42.1	51.24	0.0000	112.09	58828000	393	16.55	16.26	16.56	16.99	16.12	15.95	16.77	16.63
0.244	0.22	1.17	Q8R418	Endoribonuclease Dicer	Dicer1	3863	5	5	5	4	4	4	216.82	0.0000	18.004	2567200	41	14.76	14.37	14.93	14.57	13.70	14.15	14.56	15.34
0.772	0.22	1.17	Q9EP72	ER membrane protein complex subunit 7	Emc7	5245	7	7	7	42.7	42.7	42.7	26.31	0.0000	100.73	17624000	132	16.10	16.06	16.64	16.31	15.95	16.15	16.14	15.98
0.164	0.22	1.16	Q6ZPJ3	E2/E3 hybrid ubiquitin-protein ligase UBE2O	Ube2o	2677	5	5	5	7.1	7.1	7.1	140.83	0.0000	12.461	3158300	20	13.45	14.24	14.98	13.76	12.79	14.21	14.66	13.89
0.319	0.22	1.16	O54916	RalBP1-associated Eps domain-containing protein 1	Reps1	432	11	11	11	18.9	18.9	18.9	86.518	0.0000	31.755	8496100	103	14.89	14.81	15.72	15.46	14.54	15.20	14.86	15.41
0.608	0.22	1.16	Q8VDQ8	NAD-dependent protein deacetylase sirtuin-2	Sirt2	3968	18	18	17	52.2	52.2	48.3	43.256	0.0000	133.78	39199000	260	16.26	16.22	16.95	16.57	16.30	16.23	16.32	16.28
0.790	0.22	1.16	P68372	Tubulin beta-4B chain	Tubb4b	1518	39	39	2	77.3	77.3	3.6	49.83	0.0000	323.31	2639800000	3938	21.11	21.09	21.46	21.49	21.17	20.87	21.23	21.01
0.246	0.22	1.16	P85094	Isochorismatase domain-containing protein 2A, mitochondrial	Isoc2a	1615	10	10	10	76.2	76.2	76.2	22.417	0.0000	189.76	55052000	224	17.76	16.93	18.60	17.98	17.52	17.80	17.74	17.34
0.270	0.22	1.16	Q9JKK1	Syntaxin-6	Stx6	5453	5	5	5	27.5	27.5	27.5	28.996	0.0000	26.699	4424800	42	14.48	14.30	15.08	15.00	14.41	14.02	14.29	15.27
0.745	0.22	1.16	P24270	Catalase	Cat	895	49	49	37	80.3	80.3	64.1	59.795	0.0000	323.31	878160000	2253	19.68	19.83	19.92	20.04	19.52	19.38	19.82	19.89
0.795	0.22	1.16	P59999	Actin-related protein 2/3 complex subunit 4	Arpc4	1348	22	22	22	89.9	89.9	89.9	19.667	0.0000	220.75	366760000	886	19.82	19.36	19.66	19.38	19.44	19.50	19.25	19.17
0.275	0.22	1.16	Q9CR68	Cytochrome b-c1 complex subunit Rieske, mitochondrial;Cytochrome b-	Uqcrfs1	4677	11	11	11	53.6	53.6	53.6	29.367	0.0000	158.44	68774000	308	18.13	18.25	17.77	18.49	18.48	18.35	17.69	17.25
1.361	0.22	1.16	Q99LF4	tRNA-splicing ligase RtcB homolog	Rtcb	4425	28	28	28	65.1	65.1	65.1	55.249	0.0000	323.31	159700000	887	17.90	17.70	18.03	17.96	17.56	17.73	17.77	17.66
0.350	0.21	1.16	P56371	Ras-related protein Rab-4A	Rab4a	1279	4	3	3	25.2	18.8	18.8	24.408	0.0000	25.87	2061100	22	13.80	13.52	14.06	14.19	14.16	13.12	13.63	13.81
2.009	0.21	1.16	Q8BPU7	Engulfment and cell motility protein 1	Elmo1	3168	45	45	39	59.4	59.4	55.6	83.935	0.0000	323.31	208650000	1032	18.27	18.10	18.27	18.25	18.02	18.11	17.98	17.92
0.798	0.21	1.16	P09411	Phosphoglycerate kinase 1	Pgk1	696	62	62	54	99.5	99.5	97.4	44.55	0.0000	323.31	3891600000	4039	21.90	21.82	22.21	22.08	21.73	22.02	21.84	21.54
0.273	0.21	1.16	Q91YR5	Methyltransferase-like protein 13	Mettl13	4174	7	7	7	15.8	15.8	15.8	78.756	0.0000	54.309	3767700	61	14.11	14.47	15.37	14.62	14.67	14.29	14.79	13.96
0.055	0.21	1.16	Q6NXJ0	Protein WWC2	Wwc2	2539	4	4	3	5.1	5.1	3.5	132.62	0.0025	2.8228	6361200	15	13.17	15.33	16.18	16.24	16.66	17.11	14.15	12.14
0.735	0.21	1.16	P20060	Beta-hexosaminidase subunit beta	Hexb	852	28	28	27	48.5	48.5	47.2	61.115	0.0000	190.04	244440000	694	19.10	18.72	18.90	19.37	18.88	18.74	18.81	18.80
0.710	0.21	1.16	Q04899;Q04	Cyclin-dependent kinase 18	Cdk18	1737	15	14	11	35.3	33.5	26.8	51.847	0.0000	139.42	17989000	196	16.25	16.11	15.98	15.78	15.84	15.53	15.87	16.03
0.800	0.21	1.16	Q91VI7	Ribonuclease inhibitor	Rnh1	4063	43	43	43	92.1	92.1	92.1	49.816	0.0000	323.31	1307700000	2379	20.47	20.35	20.86	20.77	20.40	20.26	20.43	20.51
0.461	0.21	1.16	P51855	Glutathione synthetase	Gss	1204	15	15	15	45.1	45.1	45.1	52.246	0.0000	226.48	23121000	130	16.33	16.43	17.05	17.10	16.42	16.63	16.52	16.49
0.543	0.21	1.16	Q91W59	RNA-binding motif, single-stranded-interacting protein 1	Rbms1	4094	9	9	6	35.7	35.7	34	43.99	0.0000	93.467	14106000	156	15.32	15.65	15.07	15.21	14.98	14.90	15.04	15.49
0.810	0.21	1.16	Q9CPX6	Ubiquitin-like-conjugating enzyme ATG3	Atg3	4541	11	11	11	37.6	37.6	37.6	35.796	0.0000	36.42	36175000	289	16.27	16.51	16.77	16.34	16.15	16.45	16.29	16.17
0.111	0.21	1.16	Q8BPX9	Solute carrier family 15 member 3	Slc15a3	3169	4	4	4	8	8	8	64.05	0.0000	12.704	6386700	52	15.65	15.29	15.28	13.59	15.60	13.25	15.41	14.69
0.163	0.21	1.16	Q9D7A8	Armadillo repeat-containing protein 1	Armc1	5025	6	6	6	45	45	45	31.246	0.0000	130.33	10534000	92	15.60	14.90	16.28	16.54	15.93	15.46	16.33	14.77
0.480	0.21	1.16	Q9QYE6	Golgin subfamily A member 5	Golga5	5570	34	34	34	56.5	56.5	56.5	82.367	0.0000	258.6	42744000	488	15.90	15.76	16.32	16.33	15.47	15.93	16.00	16.06
0.792	0.21	1.16	Q63844;Q61	Mitogen-activated protein kinase 3	Mapk3	2405	25	25	19	70	70	60.8	43.066	0.0000	323.31	272570000	764	19.87	19.70	19.45	19.41	19.41	19.55	19.45	19.19
0.201	0.21	1.16	Q8K157	Aldose 1-epimerase	Galm	3616	9	9	9	36.3	36.3	36.3	37.798	0.0000	62.501	14780000	116	15.35	16.53	16.84	16.84	16.50	15.66	16.03	16.54
0.826	0.21	1.16	P30204	Macrophage scavenger receptor types I and II	Msr1	974	11	11	11	31.4	31.4	31.4	50.169	0.0000	268.48	41070000	236	17.08	16.88	17.28	17.34	16.88	16.86	16.86	17.15
0.478	0.21	1.16	Q8BFY9	Transportin-1	Tnpo1	2953	22	22	15	38.2	38.2	29.4	102.36	0.0000	206.46	57330000	291	16.97	16.71	17.55	17.07	16.67	16.81	16.87	17.11
0.606	0.21	1.15	Q8VBV7	COP9 signalosome complex subunit 8	Cops8	3898	8	8	8	70.3	70.3	70.3	23.255	0.0000	35.57	56617000	128	18.81	18.66	18.53	19.07	18.39	18.85	18.62	18.38
0.254	0.21	1.15	Q9D964	Glycine amidinotransferase, mitochondrial	Gatm	5097	17	17	17	55.8	55.8	55.8	48.297	0.0000	83.455	24088000	204	16.31	15.08	16.15	15.84	15.70	16.06	15.14	15.65
0.372	0.21	1.15	Q91WG4	Elongator complex protein 2	Elp2	4110	4	4	4	8.2	8.2	8.2	93.092	0.0000	17.333	2348300	57	13.97	13.67	13.47	13.61	13.39	14.06	13.42	13.01
0.090	0.21	1.15	Q9D6K8	FUN14 domain-containing protein 2	Fundc2	5000	3	3	3	22.5	22.5	22.5	16.564	0.0000	22.646	6289600	42	13.30	13.92	16.22	15.46	13.37	15.01	15.58	14.11
0.247	0.21	1.15	Q9CXY9	GPI-anchor transamidase	Pigk	4775	11	11	11	45.3	45.3	45.3	44.895	0.0000	58.238	14446000	112	15.64	15.36	15.08	15.51	15.57	14.35	15.77	15.07
0.328	0.21	1.15	Q99KK2	N-acylneuraminate cytidylyltransferase	Cmas	4381	14	14	14	39.4	39.4	39.4	48.057	0.0000	74.031	33483000	199	16.82	16.35	17.23	17.37	16.52	16.73	17.12	16.57
0.067	0.21	1.15	Q7TPN9	Proline-rich protein 14	Prr14	2770	5	5	5	11.4	11.4	11.4	67.811	0.0000	7.6794	5007900	30	14.45	14.59	14.02	14.70	11.75	16.74	15.07	13.37
0.186	0.21	1.15	Q91VN4	MICOS complex subunit Mic25	Chchd6	4070	5	5	5	22	22	22	29.852	0.0000	13.553	3681000	14	13.09	13.69	15.10	13.80	13.67	13.50	13.87	13.83
0.158	0.20	1.15	Q3UQ84	Threonine--tRNA ligase, mitochondrial	Tars2	2017	10	10	10	16.3	16.3	16.3	81.699	0.0000	14.635	7084000	60	13.56	14.27	14.94	14.33	14.41	14.01	14.89	12.97
0.140	0.20	1.15	A6H5X4	PHD finger protein 11	Phf11	57	6	6	6	27.3	27.3	27.3	37.728	0.0000	64.233	1726500	18	12.86	12.17	12.44	12.79	12.57	11.49	11.61	13.78
0.168	0.20	1.15	Q8CD10	Calcium uptake protein 2, mitochondrial	Micu2	3454	7	7	7	22.2	22.2	22.2	49.475	0.0000	153.4	5137000	67	15.21	14.57	13.45	15.03	14.99	14.38	14.34	13.73
0.111	0.20	1.15	Q8C7U7	Polypeptide N-acetylgalactosaminyltransferase 6	Galnt6	3415	3	3	3	8.8	8.8	8.8	71.536	0.0000	10.836	1840900	27	12.31	13.75	15.18	13.85	13.46	12.68	13.82	14.33
0.538	0.20	1.15	Q80X82	Symplekin	Sympk	2874	14	14	14	17.7	17.7	17.7	142.62	0.0000	88.8	10836000	152	15.25	14.85	15.34	15.45	14.74	14.92	15.28	15.13
0.686	0.20	1.15	Q6PFQ7	Ras GTPase-activating protein 4	Rasa4	2628	28	28	28	46.3	46.3	46.3	90.059	0.0000	313.91	61063000	480	16.83	16.61	17.15	16.61	16.63	16.62	16.72	16.41
0.197	0.20	1.15	Q9CPQ8	ATP synthase subunit g, mitochondrial	Atp5l	4527	6	6	6	50.5	50.5	50.5	11.424	0.0000	70.611	95726000	225	19.19	18.51	19.58	18.19	18.86	18.25	18.24	19.30
0.918	0.20	1.15	Q9DB77	Cytochrome b-c1 complex subunit 2, mitochondrial	Uqcrc2	5131	27	27	27	52.8	52.8	52.8	48.234	0.0000	323.31	228330000	865	19.42	19.17	19.42	19.38	18.99	19.40	19.01	19.18
0.795	0.20	1.15	Q8BWG8	Beta-arrestin-1	Arrb1	3258	18	17	17	49.5	47.6	47.6	46.972	0.0000	138.38	39566000	347	16.20	16.38	16.45	16.50	16.48	16.05	16.18	16.01
0.406	0.20	1.15	Q8BGA9	Mitochondrial inner membrane protein OXA1L	Oxa1l	2971	7	7	7	13.9	13.9	13.9	48.219	0.0000	8.7987	7128200	105	14.54	14.13	15.15	14.58	14.52	14.33	14.50	14.25
0.250	0.20	1.15	Q60664	Lymphoid-restricted membrane protein;Processed lymphoid-restricted n	Lrmp	2190	9	9	9	27.1	27.1	27.1	59.587	0.0000	79.398	147010000	86	20.66	20.31	21.22	20.74	20.77	19.75	20.96	20.65
1.355	0.20	1.15	Q9WVJ2	26S proteasome non-ATPase regulatory subunit 13	Psmd13	5750	25	25	25	60.9	60.9	60.9	42.809	0.0000	323.31	99701000	480	17.46	17.61	17.58	17.74	17.36	17.25	17.48	17.49
0.180	0.20	1.15	Q8C0M0	WD repeat-containing protein 59	Wdr59	3344	3	3	3	4.6	4.6	4.6	111.72	0.0017	3.2988	873630	15	13.64	12.79	13.52	12.62	13.90	12.26	12.60	13.02
1.233	0.20	1.15	Q921T2	Torsin-1A-interacting protein 1	Tor1aip1	4240	19	19	19	41.3	41.3	41.3	66.78	0.0000	304.81	78991000	420	17.53	17.38	17.70	17.66	17.25	17.46	17.41	17.34
0.986	0.20	1.15	Q5SUR0	Phosphoribosylformylglycinamidine synthase	Pfas	2148	41	41	41	46.1	46.1	46.1	144.63	0.0000	323.31	98707000	913	17.49	17.11	17.57	17.38	17.15	17.21	17.27	17.13
0.189	0.20	1.15	Q9JJI8	60S ribosomal protein L38	Rpl38	5429	6	6	6	57.1	57.1	57.1	8.2038	0.0000	31.079	127650000	305	19.39	18.90	17.95	18.42	18.93	18.75	18.49	17.68
0.088	0.20	1.15	Q8BXV2	BRI3-binding protein	Bri3bp	3290	4	4	4	20.6	20.6	20.6	28.263	0.0000	20.729	3999500	28	13.84	12.84	14.87	13.85	14.72	13.62	14.61	11.65
0.392	0.20	1.15	Q6PDM2	Serine/arginine-rich splicing factor 1	Srsf1	2618	19	19	18	58.1	58.1	56	27.744	0.0000	102.96	59799000	220	17.06	17.73	16.74	17.14	16.94	17.21	16.91	16.83
0.163	0.20	1.15	Q9WUA6	RAC-gamma serine/threonine-protein kinase	Akt3	5704	9	6	5	26.1	16.3	15.9	55.714	0.0000	19.579	1326000	24	13.43	12.86	13.44	13.30	13.87	13.20	11.79	13.37
0.524	0.20	1.15	Q9QZ06	Toll-interacting protein	Tollip	5583	9	9	9	52.2	52.2	52.2	30.345	0.0000	123.39	20794000	184	16.74	16.96	16.39	17.05	16.34	16.62	16.66	16.74
0.961	0.19	1.14	Q9ERK4	Exportin-2	Cse1l	5313	26	26	26	31.8	31.8	31.8	110.45	0.0000	323.31	200390000	714	20.90	20.62	20.88	20.96	20.56	20.78	20.75	20.50
0.509	0.19	1.14	Q80ZJ1	Ras-related protein Rap-2a	Rap2a	2914	9	4	4	58.5	29.5	29.5	20.642	0.0000	21.515	18821000	112	16.81	16.61	16.65	16.88	16.64	16.08	16.84	16.61
0.244	0.19	1.14	Q9DAZ9	Abscission/NoCut checkpoint regulator	Zfyve19	5115	6	6	6	21.3	21.3	21.3	43.272	0.0000	8.5019	3261300	42	14.70	14.21	14.90	14.78	14.01	15.17	13.97	14.66
0.981	0.19	1.14	P80317	T-complex protein 1 subunit zeta	Cct6a	1594	37	37	32	61.4	61.4	58.2	58.004	0.0000	323.31	598070000	1903	19.38	19.13	19.45	19.48	19.06	19.34	19.14	19.12
0.841	0.19	1.14	Q9EQJ0	Two pore calcium channel protein 1	Tpcn1	5281	4	4	4	6.1	6.1	6.1	94.495	0.0000	7.9797	3736600	27	14.43	14.16	14.57	14.52	14.15	14.09	14.41	14.26
0.414	0.19	1.14	Q8BRN9	Coiled-coil and C2 domain-containing protein 1B	Cc2d1b	3183	22	22	22	41.3	41.3	41.3	93.09	0.0000	193.67	28434000	288	16.16	15.48	16.33	16.25	15.74	16.03	15.77	15.90
0.077	0.19	1.14	P30355	Arachidonate 5-lipoxygenase-activating protein	Alox5ap	977	4	4	4	19.9	19.9	19.9	18.136	0.0000	11.942	63424000	88	18.28	16.88	20.03	19.47	19.13	19.22	16.80	18.74
0.406	0.19	1.14	O89112	LanC-like protein 1	Lancl1	606	10	10	10	27.3	27.3	27.3	45.341	0.0000	16.399	7331700	68	15.03	14.96	14.58	14.52	14.18	14.47	14.74	14.93
0.666	0.19	1.14	P46062	Signal-induced proliferation-associated protein 1	Sipa1	1102	27	27	27	32.1	32.1	32.1	112.06	0.0000	155.61	54544000	404	17.24	17.02	17.50	17.48	17.04	17.30	17.19	16.94
0.758	0.19	1.14	Q99L45	Eukaryotic translation initiation factor 2 subunit 2	Eif2s2	4408	22	22	21	50.2	50.2	46.2	38.092	0.0000	123.73	75039000	517	17.14	16.70	16.73	16.96	16.75	16.84	16.65	16.51
0.331	0.19	1.14	Q3U319	E3 ubiquitin-protein ligase BRE1B	Rnf40	1916	16	16	16	22.9	22.9	22.9	113.97	0.0000	91.03	9028000	110	14.10	13.92	14.21	14.42	14.00	14.31	14.24	13.33
1.096	0.19	1.14	Q8CG76	Aflatoxin B1 aldehyde reductase member 2	Akr7a2	3500	13	13	13	47.4	47.4	47.4	40.612	0.0000	114.83	50486000	273	16.86	16.76	17.09	16.76	16.67	16.56	16.67	16.79
0.292	0.19	1.14	Q5H8C4	Vacuolar protein sorting-associated protein 13A	Vps13a	2106	11	11	11	5.9	5.9	5.9	359.4	0.0000	33.801	4382400	65	14.26	14.03	14.75	13.99	13.68	14.66	13.91	14.02
0.780	0.19	1.14	Q6WVG3	BTB/POZ domain-containing protein KCTD12	Kctd12	2667	17	17	16	55.4	55.4	55.4	35.892	0.0000	322.4	156480000	624	18.65	18.54	19.01	18.78	18.55	18.67	18.62	18.36
0.210	0.19	1.14	Q99KR6;Q6	E3 ubiquitin-protein ligase RNF34	Rnf34	4391	6	6	6	23.7	23.7	23.7	42.03	0.0000	19.865	2581900	22	14.22	13.29	14.55	14.19	13.67	14.57	13.50	13.75
0.628	0.19	1.14	P63330	Serine/threonine-protein phosphatase 2A catalytic subunit alpha isoforr	Ppp2ca	1507	28	28	7	85.4	85.4	9.4	35.608	0.0000	323.31	113990000	636	18.00	17.98	17.51	17.75	17.72	17.35	17.67	17.73
0.831	0.19	1.14	Q9D1M4	Eukaryotic translation elongation factor 1 epsilon-1	Eef1e1	4926	8	8	8	54.6	54.6	54.6	19.859	0.0000	125.17	23533000	152	16.38	16.42	16.54	16.28	16.05	16.35	16.03	16.43
1.504	0.19	1.14	Q9D554	Splicing factor 3A subunit 3	Sf3a3	4982	26	26	26	51.7	51.7	51.7	58.841	0.0000	115.61	69793000	431	17.22	17.26	17.22	17.28	16.99	17.00	16.98	17.25
0.681	0.19	1.14	Q9EQI8	39S ribosomal protein L46, mitochondrial	Mrpl46	5280	8	8	8	39.9	39.9	39.9	32.131	0.0000	70.24	19479000	224	15.79	15.72	16.17	16.18	15.81	15.63	15.75	15.92
0.155	0.19	1.14	P97760	DNA-directed RNA polymerase II subunit RPB3	Polr2c	1667	3	3	3	17.8	17.8	17.8	31.443	0.0000	14.896	6315700	59	14.60	15.26	15.04	15.19	15.21	15.65	14.91	13.58
0.049	0.19	1.14	Q04863	Transcription factor RelB	Relb	1736	4	4	4	12.2	12.2	12.2	60.305	0.0000	36.475	9073500	53	15.90	15.81	16.53	13.00	16.06	16.45	16.10	11.87
0.158	0.19	1.14	Q8K2M0	39S ribosomal protein L38, mitochondrial	Mrpl38	3666	7	7	6	17.9	17.9	16.1	45.029	0.0004	4.747	5963100	48	15.10	15.10	14.24	13.85	14.85	13.41	14.74	14.54

0.148	0.19	1.14	Q60680	Inhibitor of nuclear factor kappa-B kinase subunit alpha	Chuk	2194	4	4	4	7.2	7.2	7.2	84.728	0.0002	5.6884	1474000	10	13.70	13.42	14.20	13.90	14.47	13.72	12.34	13.95
0.260	0.19	1.14	Q8VD76	General transcription factor IIH subunit 3	Gtf2h3	3945	3	3	3	16.2	16.2	16.2	34.244	0.0000	9.4318	1156100	21	13.45	12.69	12.52	13.57	12.51	13.11	12.99	12.87
0.292	0.19	1.14	Q9CQX2	Cytochrome b5 type B	Cyb5b	4645	9	9	8	70.5	70.5	70.5	16.318	0.0000	239.66	77632000	267	17.80	18.21	17.74	17.47	17.60	17.05	18.10	17.73
0.128	0.19	1.14	P08074	Carbonyl reductase [NADPH] 2	Cbr2	677	11	11	11	62.7	62.7	62.7	25.958	0.0000	164.27	6432400	68	13.74	14.05	12.69	14.16	13.70	14.28	12.24	13.67
0.550	0.19	1.14	Q6P9Q6	FK506-binding protein 15	Fkbp15	2590	37	37	37	38.2	38.2	38.2	132.96	0.0000	323.31	84254000	725	17.10	16.87	17.38	17.31	16.76	17.25	17.03	16.87
1.178	0.19	1.14	Q8BVU0	Leucine-rich repeat and calponin homology domain-containing protein 3	Lrch3	3247	13	13	13	23.8	23.8	23.8	86.34	0.0000	80.364	15653000	154	16.18	16.06	16.12	16.13	15.75	16.09	15.87	16.04
0.700	0.19	1.14	A6H8H2	DENN domain-containing protein 4C	Dennd4c	63	40	40	40	25.8	25.8	25.8	211.46	0.0000	235.04	56514000	494	16.34	16.01	16.63	16.27	16.08	16.11	16.15	16.17
1.078	0.19	1.14	Q9D0K2	Succinyl-CoA:3-ketoacid coenzyme A transferase 1, mitochondrial	Oxct1	4873	30	30	30	68.1	68.1	68.1	55.988	0.0000	323.31	420750000	1276	18.90	18.73	18.93	18.83	18.75	18.64	18.80	18.45
1.228	0.19	1.14	Q9CPY7	Cytosol aminopeptidase	Lap3	4546	30	30	30	77.5	77.5	77.5	56.141	0.0000	323.31	159850000	927	17.76	17.57	17.72	17.81	17.45	17.57	17.42	17.69
0.159	0.19	1.14	Q80X80	C2 domain-containing protein 2-like	C2cd2l	2873	5	5	5	10.9	10.9	10.9	76.328	0.0000	15.02	1705400	38	13.92	12.51	13.50	13.13	13.49	12.27	12.83	13.74
0.110	0.19	1.14	Q9R1S3	GPI ethanolamine phosphate transferase 1	Pign	5661	3	3	3	3.1	3.1	3.1	105.04	0.0000	9.7234	883740	15	13.42	12.28	13.17	12.44	12.51	14.14	11.43	12.50
1.100	0.18	1.14	Q61166	Microtubule-associated protein RP/EB family member 1	Mapre1	2266	22	22	22	70.5	70.5	70.5	30.016	0.0000	323.31	171230000	704	18.49	18.50	18.76	18.63	18.31	18.49	18.30	18.54
0.705	0.18	1.14	Q9JHK5	Pleckstrin	Plek	5366	25	25	25	56.6	56.6	56.6	39.9	0.0000	305.44	96551000	489	17.53	17.71	17.79	18.08	17.49	17.58	17.74	17.56
0.454	0.18	1.14	O70404	Vesicle-associated membrane protein 8	Vamp8	494	9	9	9	59.4	59.4	59.4	11.451	0.0000	120.87	114930000	272	18.26	17.63	18.19	18.47	17.87	18.04	17.96	17.94
0.462	0.18	1.14	P09103	Protein disulfide-isomerase	P4hb	693	54	54	54	81.1	81.1	81.1	57.058	0.0000	323.31	431830000	1855	18.73	19.05	18.24	18.79	18.66	18.38	18.49	18.54
0.505	0.18	1.14	P11835	Integrin beta-2	Itgb2	750	37	37	37	45.1	45.1	45.1	85.025	0.0000	323.31	691180000	1282	20.42	20.14	20.83	20.33	20.30	20.23	20.43	20.03
0.608	0.18	1.13	Q8R180	ERO1-like protein alpha	Ero1l	3781	11	11	11	31.7	31.7	31.7	54.084	0.0000	105.75	19300000	141	16.38	16.55	16.86	16.94	16.50	16.36	16.63	16.51
0.152	0.18	1.13	Q80VP1;Q9	Epsin-1	Epn1	2854	7	7	7	16.5	16.5	16.5	60.211	0.0000	24.024	7229600	82	15.56	14.90	15.22	15.35	15.15	15.62	13.81	15.71
0.416	0.18	1.13	Q921G6	Leucine-rich repeat and calponin homology domain-containing protein 4	Lrch4	4222	23	23	23	53.4	53.4	53.4	73.162	0.0000	241.83	76335000	404	18.43	17.84	18.49	18.32	18.12	18.33	18.16	17.75
0.400	0.18	1.13	B2RWS6	Histone acetyltransferase p300	Ep300	81	9	9	8	4.4	4.4	3.7	263.3	0.0000	16.679	5008600	68	14.32	14.09	14.40	13.90	13.99	14.34	14.10	13.56
0.296	0.18	1.13	Q9EPB4	Apoptosis-associated speck-like protein containing a CARD	Pycard	5250	7	7	7	48.7	48.7	48.7	21.458	0.0000	205.52	18467000	173	16.03	16.27	16.11	16.20	16.09	16.61	15.76	15.44
0.097	0.18	1.13	Q3TN34	MICAL-like protein 2	Micall2	1864	10	10	10	15.9	15.9	15.9	108.29	0.0000	23.121	3203100	46	14.25	14.48	12.91	14.13	12.41	14.24	13.30	15.11
0.715	0.18	1.13	Q3UVL4	Vacuolar protein sorting-associated protein 51 homolog	Vps51	2039	17	17	16	25.4	25.4	25.3	86.186	0.0000	145.47	20113000	218	15.78	15.32	15.86	15.76	15.48	15.49	15.52	15.52
0.653	0.18	1.13	Q8VDM6	Heterogeneous nuclear ribonucleoprotein U-like protein 1	Hnrnpul1	3961	26	26	26	34.3	34.3	34.3	96.001	0.0000	204.83	56222000	485	16.37	16.05	16.47	16.60	16.06	16.33	16.15	16.22
0.426	0.18	1.13	Q3UJU9	Regulator of microtubule dynamics protein 3	Rmdn3	1986	7	7	7	29.4	29.4	29.4	52.028	0.0000	78.21	12698000	184	15.20	15.19	15.32	15.35	15.04	14.66	15.55	15.11
0.163	0.18	1.13	P11031	Activated RNA polymerase II transcriptional coactivator p15	Sub1	735	6	6	6	42.5	42.5	42.5	14.427	0.0000	60.314	7044600	58	15.44	15.50	14.79	14.76	14.84	15.95	14.82	14.17
0.448	0.18	1.13	Q8BGE6	Cysteine protease ATG4B	Atg4b	2979	9	9	9	38.7	38.7	38.7	44.375	0.0000	51.121	10116000	102	15.07	15.26	15.71	14.99	14.99	15.28	14.96	15.07
0.591	0.18	1.13	P52623	Uridine-cytidine kinase 1	Uck1	1221	8	8	7	43	43	40.1	31.068	0.0000	33.196	7621500	86	14.80	14.77	15.17	15.18	14.64	15.02	14.85	14.70
0.075	0.18	1.13	Q9Z127	Large neutral amino acids transporter small subunit 1	Slc7a5	5797	3	2	2	4.7	3.3	3.3	55.872	0.0000	9.179	3022000	35	11.50	14.47	14.64	13.98	14.43	12.42	13.27	13.76
0.298	0.18	1.13	Q8R395	COMM domain-containing protein 5	Commd5	3839	5	5	5	27.7	27.7	27.7	24.493	0.0000	41.596	6422800	37	14.71	15.46	15.62	15.12	14.98	15.48	14.92	14.82
0.307	0.18	1.13	Q91YL3	Uridine-cytidine kinase-like 1	Uckl1	4165	12	12	12	34.7	34.7	34.7	60.841	0.0000	78.967	12053000	147	15.17	15.34	15.72	15.43	14.62	15.56	15.32	15.45
0.464	0.18	1.13	Q8BYC6;Q6	Serine/threonine-protein kinase TAO3	Taok3	3297	60	60	53	49	49	45.1	105.33	0.0000	323.31	105930000	1078	16.66	16.38	16.69	16.99	16.34	16.77	16.28	16.62
0.912	0.18	1.13	Q8VCW8	Acyl-CoA synthetase family member 2, mitochondrial	Acsf2	3934	13	13	13	43.9	43.9	43.9	67.95	0.0000	139.26	16415000	89	16.05	16.33	16.29	16.05	15.88	15.94	16.04	16.17
0.609	0.17	1.13	P36371	Antigen peptide transporter 2	Tap2	1040	15	15	15	29.2	29.2	29.2	77.444	0.0000	303.14	39051000	297	15.92	15.70	16.16	16.16	15.88	16.00	15.73	15.64
0.173	0.17	1.13	Q61206	Platelet-activating factor acetylhydrolase IB subunit beta	Pafah1b2	2276	4	4	4	30.1	30.1	30.1	25.581	0.0000	48.951	10535000	83	15.22	16.11	14.99	15.12	15.89	15.44	14.59	14.83
1.000	0.17	1.13	Q99KQ4	Nicotinamide phosphoribosyltransferase	Nampt	4389	39	39	39	68.6	68.6	68.6	55.446	0.0000	323.31	305610000	1175	18.78	18.50	18.71	18.66	18.55	18.65	18.38	18.38
0.443	0.17	1.13	P49722	Proteasome subunit alpha type-2	Psma2	1170	17	17	17	68.4	68.4	68.4	25.926	0.0000	263.36	227680000	654	19.18	18.55	18.42	18.85	18.63	18.45	18.62	18.60
0.468	0.17	1.13	O55023	Inositol monophosphatase 1	Impa1	443	16	16	16	48.4	48.4	48.4	30.436	0.0000	123.7	116070000	412	18.15	18.36	18.76	18.73	18.22	18.45	18.47	18.16
0.475	0.17	1.13	Q9R233	Tapasin	Tapbp	5670	19	19	19	45.4	45.4	45.4	49.736	0.0000	232.77	124680000	538	17.85	17.94	18.17	17.77	17.94	18.05	17.45	17.60
0.103	0.17	1.13	Q2YFS3	Paired immunoglobulin-like type 2 receptor alpha	Pilra	1805	2	2	1	7.6	7.6	3.6	33.063	0.0022	3.0835	2563600	13	14.22	12.83	14.28	13.85	13.21	15.13	12.81	13.35
0.328	0.17	1.13	Q504P2	C-type lectin domain family 12 member A	Clec12a	2077	10	10	10	34.5	34.5	34.5	30.757	0.0000	34.719	21510000	148	16.70	16.11	15.86	16.02	16.30	15.94	16.06	15.70
0.873	0.17	1.13	Q91Z67	SLIT-ROBO Rho GTPase-activating protein 2	Srgap2	4188	39	39	35	42.4	42.4	40.3	120.8	0.0000	263.71	61761000	688	16.35	16.05	16.23	16.11	16.06	16.19	15.84	15.96
0.234	0.17	1.13	Q8VEJ9	Vacuolar protein sorting-associated protein 4A	Vps4a	4009	15	9	9	40	28.4	28.4	48.906	0.0000	54.192	9063200	99	15.22	14.60	15.42	15.55	14.76	15.35	14.59	15.41
0.487	0.17	1.13	Q9ERL7	Glia maturation factor gamma	Gmfg	5314	10	8	8	58.5	48.6	48.6	16.748	0.0000	118.55	35550000	214	16.41	16.86	16.71	16.78	16.32	16.88	16.49	16.37
0.370	0.17	1.13	Q99KP3	Lambda-crystallin homolog	Cryl1	4387	21	21	21	90	90	90	35.208	0.0000	203.12	51094000	364	16.94	16.69	17.57	17.25	17.04	17.00	16.95	16.78
0.283	0.17	1.13	O35071	Kinesin-like protein KIF1C	Kif1c	332	10	6	6	10.6	6.9	6.9	122.43	0.0000	18.074	6041100	57	15.38	15.11	15.61	16.07	15.18	15.61	15.63	15.07
0.226	0.17	1.12	Q9DBH5	Vesicular integral-membrane protein VIP36	Lman2	5157	13	13	13	48.6	48.6	48.6	40.429	0.0000	114.74	21002000	180	16.24	15.94	15.53	16.29	15.96	15.16	15.86	16.34
0.848	0.17	1.12	Q99J36	THUMP domain-containing protein 1	Thumpd1	4322	10	10	10	32.9	32.9	32.9	38.884	0.0000	103.6	17528000	160	16.17	16.06	16.11	16.01	16.03	16.13	15.79	15.74
0.094	0.17	1.12	P51908	C->U-editing enzyme APOBEC-1	Apobec1	1209	7	7	7	29.7	29.7	29.7	27.521	0.0000	36.896	28469000	109	17.22	16.55	18.73	18.62	17.48	18.69	17.36	16.91
0.068	0.17	1.12	Q3UV16	Inositol 1,4,5-trisphosphate receptor-interacting protein-like 2	Itpripl2	2034	4	4	4	9.2	9.2	9.2	58.664	0.0002	5.9434	810750	17	12.66	11.21	12.07	15.07	12.93	12.59	11.67	13.15
0.627	0.17	1.12	Q9D7N3	28S ribosomal protein S9, mitochondrial	Mrps9	5036	10	10	10	31.8	31.8	31.8	44.929	0.0000	72.551	46277000	118	18.52	18.62	18.69	18.78	18.17	18.72	18.54	18.53
0.345	0.17	1.12	Q9Z2Z6	Mitochondrial carnitine/acylcarnitine carrier protein	Slc25a20	5874	9	9	9	42.5	42.5	42.5	33.026	0.0000	34.55	19327000	81	15.60	15.91	16.06	16.22	15.32	15.95	15.80	16.05
0.171	0.17	1.12	Q9Z329	Inositol 1,4,5-trisphosphate receptor type 2	Itpr2	5879	10	10	10	5.8	5.8	5.8	307.47	0.0000	47.355	2477700	47	13.98	12.96	14.22	13.57	13.02	13.60	14.21	13.22
0.665	0.17	1.12	Q9DBG7	Signal recognition particle receptor subunit alpha	Srpr	5154	22	22	22	45	45	45	69.622	0.0000	245.63	50959000	505	16.84	16.48	16.33	16.59	16.54	16.28	16.35	16.41
1.550	0.17	1.12	Q99JY9;Q6	Actin-related protein 3	Actr3	4358	44	44	44	85.2	85.2	85.2	47.357	0.0000	323.31	1385700000	2163	21.46	21.26	21.35	21.32	21.16	21.29	21.14	21.13
0.713	0.17	1.12	O35435	Dihydroorotate dehydrogenase (quinone), mitochondrial	Dhodh	369	16	16	16	55.7	55.7	55.7	42.699	0.0000	115.15	31153000	305	15.90	16.06	16.43	16.09	15.91	15.94	15.95	16.02
0.343	0.17	1.12	Q9D6U8	Protein FAM162A	Fam162a	5010	7	7	7	40	40	40	17.725	0.0000	32.45	14397000	64	16.16	15.37	15.74	15.72	15.41	15.32	15.69	15.90
0.188	0.17	1.12	O08912	Polypeptide N-acetylgalactosaminyltransferase 1;Polypeptide N-acetylg	Galnt1	310	4	4	3	12.2	12.2	10.7	64.254	0.0000	16.108	1487000	12	14.18	13.53	13.64	13.47	12.83	13.43	14.32	13.58
1.508	0.17	1.12	P56399	Ubiquitin carboxyl-terminal hydrolase 5	Usp5	1286	48	48	48	66.1	66.1	66.1	95.832	0.0000	323.31	300370000	1217	19.37	19.29	19.50	19.36	19.18	19.15	19.33	19.20
0.351	0.17	1.12	Q80U87	Ubiquitin carboxyl-terminal hydrolase 8	Usp8	2825	25	25	25	33.7	33.7	33.7	122.61	0.0000	292.63	36406000	306	16.40	15.82	16.70	16.00	15.95	16.08	16.15	16.07
0.114	0.17	1.12	Q501J7;Q2N	Phosphatase and actin regulator 4	Phactr4	2076	3	3	3	5.2	5.2	5.2	76.631	0.0025	2.806	1149300	5	12.46	12.89	12.37	14.58	12.90	12.64	12.72	13.37
0.522	0.17	1.12	O88844	Isocitrate dehydrogenase [NADP] cytoplasmic	Idh1	574	52	52	50	89.6	89.6	87.4	46.674	0.0000	323.31	1272500000	2627	20.43	20.13	20.05	20.18	20.19	20.29	19.84	19.82
0.112	0.17	1.12	O09111	NADH dehydrogenase [ubiquinone] 1 beta subcomplex subunit 11, mito	Ndufb11	324	5	5	5	28.5	28.5	28.5	17.444	0.0000	47.051	13132000	58	16.30	15.39	16.32	17.33	15.22	16.62	15.96	16.89
1.087	0.17	1.12	P05064	Fructose-bisphosphate aldolase A	Aldoa	647	52	52	12	98.9	98.9	30.5	39.355	0.0000	323.31	6032700000	5509	22.68	22.49	22.77	22.72	22.62	22.48	22.53	22.37
0.296	0.16	1.12	Q9CXG3	Peptidyl-prolyl cis-trans isomerase-like 4	Ppil4	4755	10	10	9	29.7	29.7	25.2	57.23	0.0000	24.561	9837000	98	14.83	14.95	15.20	15.44	15.12	15.38	14.72	14.54
0.176	0.16	1.12	Q8BMJ3	Eukaryotic translation initiation factor 1A, X-chromosomal	Eif1ax	3141	11	2	2	61.8	7.6	7.6	16.46	0.0008	4.1678	2012400	15	11.61	13.08	12.27	12.94	11.98	12.60	12.40	12.26
0.592	0.16	1.12	Q8BT60;Q1	Copine-3	Cpne3	3198	23	23	21	44.8	44.8	43.2	59.584	0.0000	178.5	104210000	578	17.46	17.63	17.24	17.53	17.40	17.00	17.36	17.45
0.142	0.16	1.12	Q8BP92	Reticulocalbin-2	Rcn2	3161	2	2	2	6.2	6.2	6.2	37.27	0.0008	4.1802	834590	8	13.06	13.65	13.63	13.50	12.78	13.51	14.37	12.53
0.313	0.16	1.12	Q91XI1	tRNA-dihydrouridine(47) synthase [NAD(P)(+)]-like	Dus3l	4147	11	11	11	25.1	25.1	25.1	71.078	0.0000	134.61	24574000	175	16.61	16.54	16.94	16.71	16.38	16.92	16.80	16.04
0.226	0.16	1.12	Q99J72	DNA dC->dU-editing enzyme APOBEC-3	Apobec3	4329	8	8	8	25.2	25.2	25.2	52.213	0.0000	52.384	6407600	88	14.66	14.94	13.90	14.22	14.75	14.01	14.02	14.28
0.627	0.16	1.12	P35564	Calnexin	Canx	1026	29	29	29	44.8	44.8	44.8	67.277	0.0000	284.93	228090000	533	19.23	18.97	19.37	19.33	18.94	18.90	19.14	19.27
0.360	0.16	1.12	P62245	40S ribosomal protein S15a	Rps15a	1409	15	15	15	82.3	82.3	82.3	14.839	0.0000	111.44	177940000	509	19.44	18.69	18.62	18.98	18.68	18.89	18.68	18.84
0.448	0.16	1.12	Q99KH8	Serine/threonine-protein kinase 24;Serine/threonine-protein kinase 24 3	Stk24	4376	18	18	9	53.6	53.6	36.7	47.953	0.0000	112.65	39379000	305	16.37	16.82	16.92	16.99	16.43	16.76	16.73	16.53
1.020	0.16	1.12	P97429	Annexin A4	Anxa4	1648	39	39	38	87.5	87.5	87.5	35.915	0.0000	323.31	1054500000	1736	20.69	20.54	20.45	20.68	20.34	20.32	20.52	20.53
0.660	0.16	1.12	P22366	Myeloid differentiation primary response protein MyD88	Myd88	874	12	12	12	46.3	46.3	46.3	33.753	0.0000	40.034	13271000	143	15.20	15.34	15.05	15.55	15.06	15.25	15.06	15.14
0.338	0.16	1.12	P97287	Induced myeloid leukemia cell differentiation protein Mcl-1 homolog	Mcl1	1617	11	11	11	51.1	51.1	51.1	35.217	0.0000	57.391	29590000	201	16.86	16.60	17.29	16.69	16.95	16.89	16.43	16.53
0.368	0.16	1.12	Q91ZJ5	UTP--glucose-1-phosphate uridylyltransferase	Ugp2	4196	42	42	42	67.1	67.1	67.1	56.979	0.0000	323.31	209780000	1166	18.08	18.06	18.32	18.22	17.69	17.73	18.37	18.26
0.564	0.16	1.12	P99024	Tubulin beta-5 chain	Tubb5	1691	37	7	6	73.2	20.5	17.1	49.67	0.0000	323.31	1078300000	824	22.31	21.73	22.22	22.14	21.97	21.99	21.93	21.88
0.557	0.16	1.12	Q8CGK3	Lon protease homolog, mitochondrial	Lonp1	3509	46	46	46	63.1	63.1	63.1	105.84	0.0000	323.31	86030000	712	16.75	16.72	17.26	16.90	16.62	16.73	16.83	16.83
0.262	0.16	1.12	Q8BKI2	Trinucleotide repeat-containing gene 6B protein	Tnrc6b	3102	6	6	6	5.2	5.2	5.2	191.96	0.0000	45.592	2124400	44	13.74	12.82	13.62	13.44	13.18	13.36	13.55	12.89
0.354	0.16	1.12	Q8CHQ0	F-box only protein 4	Fbxo4	3529	4	4	4	11.7	11.7	11.7	43.776	0.0000	9.4259	5760900	36	15.00	15.11	15.48	15.32	14.61	15.20	15.15	15.33
0.110	0.16	1.12	P84244;P02	Histone H3.3;Histone H3.3C	H3f3a;H3f3c	1614	19	1	1	77.9	23.5	23.5	15.328	0.0000	301.05	44899000	102	19.65	19.70	17.90	17.95	18.27	18.56	19.02	18.71
0.299	0.16	1.11	Q921L3	Transmembrane and coiled-coil domain-containing protein 1	Tmco1	4230	8	8	8	31.9	31.9	31.9	21.175	0.0000	25.357	24678000	104	16.48	16.38	16.39	16.49	16.80	15.90	15.94	16.47
0.094	0.16	1.11	Q80ZW2	Protein THEM6	Them6	2923	2	2	2	12.6	12.6	12.6	23.802	0.0000	6.6899	2294600	27	14.32	13.44	15.11	14.17	13.06	14.39	13.61	15.35
0.411	0.16	1.11	Q9JLQ2	ARF GTPase-activating protein GIT2	Git2	5488	36	36	32	67.2	67.2	63.8	78.765	0.0000	323.31	85527000	694	17.12	16.98	17.42	17.38	16.94	17.46	16.89	16.99
0.632	0.15	1.11	Q8CDG3	Deubiquitinating protein VCIP135	Vcpip1	3460	31	31	31	31.7	31.7	31.7	134.5	0.0000	180.18	47850000	438	16.21	15.95	16.29	16.18	15.94	16.27	15.86	15.94
0.115	0.15	1.11	P62342	Selenoprotein T	Selt	1429	6	6	6	22.1	22.1	22.1	22.292	0.0000	9.4545	4085800	24	14.52	13.19	13.89	14.11	12.93	13.87	13.42	14.87
0.154	0.15	1.11	Q9CR11	YEATS domain-containing protein 4	Yeats4	4657	5	5	4	34.4	34.4	28.2	26.529	0.0000	27.281	2694800	33	13.96	12.94	14.02	14.53	14.19	13.29	13.79	13.57
0.684	0.15	1.11	Q9JKC8	AP-3 complex subunit mu-1	Ap3m1	5449	17	17	14	56.9	56.9	47.4	46.936	0.0000	270.7	79185000	461	17.48	17.66	17.71	17.48	17.28	17.26	17.59	17.58
0.702	0.15	1.11	P08752	Guanine nucleotide-binding protein G(i) subunit alpha-2	Gnai2	687	36	36	20	78.3	78.3	49	40.489	0.0000	323.31	910920000	1830	20.83	20.80	20.51	20.83	20.76	20.45	20.66	20.48
0.410	0.15	1.11	P26638;RE\	Serine--tRNA ligase, cytoplasmic	Sars	927	46	46	46	69.5	69.5	69.5	58.388	0.0000	323.31	303710000	1413	18.42	18.25	18.82	18.70	18.22	18.68	18.37	18.31
0.142	0.15	1.11	P42128;Q3L	Forkhead box protein K1	Foxk1	1073	5	5	5	8.8	8.8	8.8	74.919	0.0000	16.314	4196500	41	14.27	13.82	14.36	14.05	14.03	14.75	14.21	12.90
0.374	0.15	1.11	P70452	Syntaxin-4	Stx4	1577	14	14	14	55	55	55	34.165	0.0000	285.02	62632000	406	17.22	16.92	17.63	17.03	16.86	17.17	17.20	16.95
0.344	0.15	1.11	P47791	Glutathione reductase, mitochondrial	Gsr	1123	23	23	23	62.8	62.8	62.8	53.662	0.0000	307.74	180950000	488	20.02	19.70	20.39	19.71	19.61	19.68	20.05	19.88
0.103	0.15	1.11	A2RSY6	TRMT1-like protein	Trmt1l	49	12	12	12	22.7	22.7	22.7	80.86	0.0000	108.17	8189500	100	15.61	14.92	15.31	15.51	14.55	14.47	15.01	16.71
0.090	0.15	1.11	Q64282	Interferon-induced protein with tetratricopeptide repeats 1	Ifit1	2427	29	29	28	66.1	66.1	65.9	53.737	0.0000	323.31	52123000	310	16.79	16.49	15.52	15.37	16.61	16.79	15.52	14.64
0.333	0.15	1.11	Q9CZ04	COP9 signalosome complex subunit 7a	Cops7a	4812	12	12	12	59.3	59.3	59.3	30.224	0.0000	160.28	29595000	313	16.80	16.64	16.00	16.71	16.54	16.21	16.42	16.36
0.399	0.15	1.11	Q9JMA1	Ubiquitin carboxyl-terminal hydrolase 14	Usp14	5503	25	25	25	50.3	50.3	50.3	56.001	0.0000	323.31	113090000	589	17.51	17.93	17.48	17.31	17.51	17.09	17.50	17.51
0.598	0.15	1.11	Q3UFY7	7-methylguanosine phosphate-specific 5-nucleotidase	Nt5c3b	1958	6	6	6	30.7	30.7	30.7	34.425	0.0000	105.94	7266300	131	14.72	14.53	14.54	14.89	14.63	14.41	14.68	14.34
0.421	0.15	1.11	Q99MR3	Solute carrier family 12 member 9	Slc12a9	4473	7	7	7	12.1	12.1	12.1	96.329	0.0000	143.22	17329000	137	16.26	15.99	16.41	16.29	15.88	16.45	16.09	15.93
0.368	0.15	1.11	P97820	Mitogen-activated protein kinase kinase kinase kinase 4	Map4k4	1676	16	7	6	15.7	8.9	8.4	140.6	0.0000	43.828	14089000	77	16.85	16.98	17.59	17.26	16.86	17.01	17.07	17.15
0.694	0.15	1.11	Q8VDN2	Sodium/potassium-transporting ATPase subunit alpha-1	Atp1a1	3962	70	70	48	52.9	52.9	41.1	112.98	0.0000	323.31	860670000	2486	19.86	19.54	19.81	19.83	19.71	19.43	19.74	19.56
0.772	0.15	1.11	Q8BHD7	Polypyrimidine tract-binding protein 3	Ptbp3	3033	23	18	18	53.3	48.6	48.6	56.7	0.0000	323.31	140440000	472	18.46	18.37	18.62	18.60	18.25	18.45	18.23	18.53
0.675	0.15	1.11	O70133	ATP-dependent RNA helicase A	Dhx9	470	56	56	56	40.9	40.9	40.9	149.47	0.0000	323.31	204580000	1291	18.21	17.97	18.00	17.93	17.85	18.09	17.68	17.91
0.322	0.15	1.11	Q61990;P57	Poly(rC)-binding protein 2	Pcbp2	2345	21	15	15	78.2	66.3	66.3	38.221	0.0000	264.14	112430000	395	17.77	18.08	18.24	18.64	17.91	18.20	18.10	17.94
0.087	0.15	1.11	Q6P2L6	Histone-lysine N-methyltransferase NSD3	Whsc1l1	2558	2	2	2	3.3	3.3	3.3	161	0.0000	18.28	570410	10	13.48	11.82	13.79	13.29	12.60	12.98	12.11	14.10
0.338	0.15	1.11	Q9QYR9	Acyl-coenzyme A thioesterase 2, mitochondrial	Acot2	5579	16	16	6	48.8	48.8	21.9	49.656	0.0000	156.24	42129000	381	16.40	16.51	16.77	16.46	15.91	16.44	16.65	16.56
0.279	0.14	1.11	Q921Z5	Tumor necrosis factor alpha-induced protein 8	Tnfaip8	4245	10	10	10	51.5	51.5	51.5	22.96	0.0000	245.5	42390000	197	17.33	17.21	17.92	17.75	17.38	17.55	17.04	17.65
0.943	0.14	1.10	P14206	40S ribosomal protein SA	Rpsa	777	20	20	18	64.4	64.4	64.1	32.838	0.0000	323.31	469100000	741	20.40	20.52	20.49	20.52	20.43	20.39	20.12	20.41
0.056	0.14	1.10	A2RTL5	Arginine/serine-rich coiled-coil protein 2	Rsrc2	50	5	5	5	28.2	28.2	28.2	43.876	0.0000	14.147	4303900	27	15.06	15.48	14.68	13.09	14.57	16.43	13.77	12.97
0.323	0.14	1.10	Q64511	DNA topoisomerase 2-beta	Top2b	2448	45	45	35	32.7	32.7	26.9	181.91	0.0000	145	56095000	440	16.53	16.05	16.73	16.48	16.11	16.59	16.13	16.41
0.091	0.14	1.10	Q9DBR0	A-kinase anchor protein 8	Akap8	5169	9	9	9	21.3	21.3	21.3	76.293	0.0000	41.582	31572000	66	15.96	17.69	18.27	18.22	17.55	17.06	17.20	17.75
1.374	0.14	1.10	P58252	Elongation factor 2	Eef2	1312	91	91	89	82.6	82.6	81.4	95.313	0.0000	323.31	3129900000	5539	21.00	21.02	20.91	21.04	20.82	20.77	20.98	20.83
0.091	0.14	1.10	Q3TKY6	Peptidyl-prolyl cis-trans isomerase CWC27 homolog	Cwc27	1857	7	7	7	24.3	24.3	24.3	53.542	0.0000	36.211	5516000	54	13.33	14.38	14.31	14.12	13.72	14.98	14.29	12.58

0.323	0.14	1.10	Q8C0T5	Signal-induced proliferation-associated 1-like protein 1	Sipa1l1	3348	4	4	3	3.8	3.8	3.1	197.03	0.0000	9.8859	1259500	34	12.93	12.64	13.05	12.99	12.37	13.16	12.73	12.80
0.082	0.14	1.10	Q9R210	Transcription factor EB	Tfeb	5669	5	3	3	11.2	7.8	7.8	52.614	0.0000	48.651	4596900	50	14.33	15.23	14.72	15.28	15.21	13.05	15.55	15.20
0.573	0.14	1.10	P18653;Q9V	Ribosomal protein S6 kinase alpha-1	Rps6ka1	839	37	37	24	52.9	52.9	36.9	81.594	0.0000	234.71	91418000	731	17.07	16.70	16.59	16.98	16.68	16.69	16.69	16.73
0.591	0.14	1.10	Q8BMS1	Trifunctional enzyme subunit alpha, mitochondrial;Long-chain enoyl-Co/	Hadha	3148	74	74	74	77.2	77.2	77.2	82.669	0.0000	323.31	522860000	2488	19.18	18.78	18.97	18.89	18.65	18.99	18.83	18.79
0.744	0.14	1.10	O88845	A-kinase anchor protein 10, mitochondrial	Akap10	575	17	17	17	38.4	38.4	38.4	73.631	0.0000	99.141	41275000	313	16.99	16.63	16.81	16.81	16.51	16.76	16.75	16.67
0.341	0.14	1.10	Q61687	Transcriptional regulator ATRX	Atrx	2323	10	10	10	5.5	5.5	5.5	278.58	0.0000	41.829	6752600	107	14.35	14.05	14.77	14.52	14.29	14.49	14.07	14.28
0.303	0.14	1.10	Q9CQV4	Protein FAM134C	Fam134c	4637	4	4	4	17.4	17.4	17.4	51.637	0.0000	65.233	19836000	115	16.75	16.57	16.94	16.30	16.41	16.56	16.84	16.20
0.556	0.14	1.10	Q99MN9	Propionyl-CoA carboxylase beta chain, mitochondrial	Pccb	4469	25	25	25	71.9	71.9	71.9	58.408	0.0000	308.83	91355000	476	18.05	18.15	18.25	18.54	18.10	18.08	18.23	18.02
0.183	0.14	1.10	P97434	Myosin phosphatase Rho-interacting protein	Mprip	1649	8	8	7	10.2	10.2	9.3	116.41	0.0000	12.553	4320500	46	14.25	14.13	14.88	14.91	14.64	13.79	14.70	14.48
0.194	0.14	1.10	Q9D1H6	NADH dehydrogenase [ubiquinone] 1 alpha subcomplex assembly factc	Ndufaf4	4914	3	3	3	22	22	22	20.082	0.0004	4.839	2226100	15	13.57	13.44	13.15	13.31	13.16	13.35	13.84	12.57
0.510	0.14	1.10	O89079	Coatomer subunit epsilon	Cope	600	19	19	19	60.7	60.7	60.7	34.567	0.0000	207.38	76489000	460	17.18	17.53	17.14	17.13	17.04	16.92	17.20	17.27
0.426	0.14	1.10	Q9D1G1	Ras-related protein Rab-1B	Rab1b	4911	25	14	13	83.1	59.7	54.2	22.187	0.0000	323.31	243490000	698	18.77	18.99	19.31	19.26	18.77	19.05	19.05	18.90
0.381	0.13	1.10	Q99JB2	Stomatin-like protein 2, mitochondrial	Stoml2	4336	20	20	20	66.9	66.9	66.9	38.384	0.0000	323.31	62926000	475	16.77	16.82	17.17	17.31	16.76	16.95	17.07	16.75
0.492	0.13	1.10	P32233	Developmentally-regulated GTP-binding protein 1	Drg1	996	16	16	16	44.4	44.4	44.4	40.512	0.0000	308.73	31232000	295	16.16	15.96	16.34	16.13	15.95	16.25	16.07	15.79
0.221	0.13	1.10	Q9JJX6	P2X purinoceptor 4	P2rx4	5438	8	8	8	24.5	24.5	24.5	43.437	0.0000	123	21349000	171	16.53	15.92	16.79	16.80	16.71	16.26	16.12	16.41
0.375	0.13	1.10	O88630	Golgi SNAP receptor complex member 1	Gosr1	552	12	12	12	60.4	60.4	60.4	28.489	0.0000	109.93	25089000	175	16.94	16.64	16.91	17.01	16.64	16.61	16.59	17.13
0.306	0.13	1.09	O88696	ATP-dependent Clp protease proteolytic subunit, mitochondrial	Clpp	559	10	10	10	38.2	38.2	38.2	29.8	0.0000	108.04	29706000	175	16.07	16.03	16.51	16.76	16.19	16.14	16.21	16.31
0.247	0.13	1.09	O35955	Proteasome subunit beta type-10	Psmb10	414	11	11	11	50.9	50.9	50.9	29.063	0.0000	196.08	104050000	364	18.06	18.02	17.74	17.78	18.17	18.03	17.54	17.34
0.070	0.13	1.09	O88622	Poly(ADP-ribose) glycohydrolase	Parg	550	5	5	5	6.1	6.1	6.1	109.32	0.0000	10.679	2371600	30	14.78	14.13	14.98	12.36	13.40	13.59	14.13	14.62
0.321	0.13	1.09	O09172	Glutamate--cysteine ligase regulatory subunit	Gclm	330	14	14	14	45.3	45.3	45.3	30.534	0.0000	323.31	157180000	533	18.35	18.17	18.66	18.80	18.25	18.19	18.52	18.52
0.273	0.13	1.09	Q9JKB1;P5ɛ	Ubiquitin carboxyl-terminal hydrolase isozyme L3;Ubiquitin carboxyl-terr	Uchl3;Uchl4	5446	8	8	8	42.6	42.6	42.6	26.151	0.0000	33.781	13544000	153	15.61	15.84	15.40	15.63	15.69	15.86	15.12	15.30
0.422	0.13	1.09	Q64727	Vinculin	Vcl	2461	69	69	69	69.6	69.6	69.6	116.72	0.0000	323.31	274040000	1600	17.85	17.80	18.25	18.01	17.60	17.98	17.90	17.92
0.348	0.13	1.09	Q9QXZ0	Microtubule-actin cross-linking factor 1	Macf1	5557	153	152	151	28.5	28.4	28.1	831.87	0.0000	323.31	171040000	1972	16.37	16.20	16.84	16.68	16.35	16.32	16.56	16.35
0.074	0.13	1.09	Q99KN1	Arrestin domain-containing protein 1	Arrdc1	4384	2	2	2	9	9	9	46.329	0.0000	7.9014	2525500	14	12.76	14.05	15.08	13.43	13.82	12.65	14.10	14.24
0.126	0.13	1.09	Q922H9	Zinc finger protein 330	Znf330	4259	3	3	3	14.9	14.9	14.9	35.607	0.0000	33.445	2891700	64	14.57	13.70	15.13	13.74	14.47	14.30	13.85	14.03
0.389	0.12	1.09	Q9CQD1	Ras-related protein Rab-5A	Rab5a	4586	12	7	6	67	47.4	44.2	23.598	0.0000	83.788	35613000	191	17.22	17.33	16.91	17.20	16.93	16.79	17.23	17.21
0.220	0.12	1.09	Q7TMW6;Q	Cytosolic Fe-S cluster assembly factor NARFL	Narfl	2751	12	12	12	45	45	45	53.107	0.0000	115.78	6996500	119	13.89	14.42	14.43	14.29	14.52	14.11	14.27	13.63
0.409	0.12	1.09	Q8C0I1	Alkyldihydroxyacetonephosphate synthase, peroxisomal	Agps	3337	31	31	31	56.3	56.3	56.3	71.683	0.0000	281.96	101300000	636	17.11	17.03	17.45	17.40	16.89	17.21	17.25	17.14
0.315	0.12	1.09	Q8BI84	Melanoma inhibitory activity protein 3	Mia3	3055	22	22	22	13.9	13.9	13.9	213.67	0.0000	45.563	24141000	170	16.47	16.34	16.71	16.66	16.02	16.43	16.57	16.66
0.153	0.12	1.09	Q8BGS0	Protein MAK16 homolog	Mak16	2990	4	4	4	22.6	22.6	22.6	35.185	0.0000	30.384	9331000	107	15.51	15.29	15.80	15.11	15.45	15.10	15.97	14.71
0.108	0.12	1.09	P51880	Fatty acid-binding protein, brain	Fabp7	1207	5	5	5	45.5	45.5	45.5	14.893	0.0000	13.053	9114800	110	15.80	15.98	14.77	15.23	14.72	14.84	15.87	15.87
0.055	0.12	1.09	Q99KK1	Receptor expression-enhancing protein 3	Reep3	4380	5	5	5	24	24	24	29.213	0.0000	11.917	2799100	26	13.40	14.12	14.54	12.50	14.15	14.30	14.02	11.60
0.229	0.12	1.09	Q925I1	ATPase family AAA domain-containing protein 3	Atad3	4307	32	32	32	51.4	51.4	51.4	66.741	0.0000	192.53	43267000	423	16.80	16.08	16.57	16.46	15.93	16.45	16.42	16.63
0.159	0.12	1.09	Q8K212	Phosphofurin acidic cluster sorting protein 1	Pacs1	3640	10	10	10	18	18	18	104.83	0.0000	48.196	8398700	81	15.22	14.62	15.51	14.59	14.98	15.30	14.43	14.75
0.271	0.12	1.09	Q9CRB9	MICOS complex subunit Mic19	Chchd3	4689	14	14	14	47.6	47.6	47.6	26.334	0.0000	128.23	44327000	360	16.63	17.03	16.77	17.17	16.57	16.55	16.87	17.13
0.541	0.12	1.09	Q8CHT0	Delta-1-pyrroline-5-carboxylate dehydrogenase, mitochondrial	Aldh4a1	3531	13	13	13	26	26	26	61.84	0.0000	134.98	38914000	317	16.33	16.22	16.29	16.34	15.91	16.13	16.32	16.33
0.147	0.12	1.09	Q9R0T8	Inhibitor of nuclear factor kappa-B kinase subunit epsilon	Ikbke	5641	7	7	7	14.1	14.1	14.1	80.952	0.0000	39.318	3219100	29	13.71	13.68	14.21	13.51	13.13	14.40	13.68	13.43
0.300	0.12	1.09	Q9WVK4	EH domain-containing protein 1	Ehd1	5752	46	46	33	83.9	83.9	62.5	60.602	0.0000	323.31	346960000	1232	18.85	18.80	19.20	19.04	18.81	19.26	18.64	18.71
0.158	0.12	1.09	Q9JHI7	Exosome complex component RRP45	Exosc9	5362	3	3	3	7.8	7.8	7.8	48.936	0.0002	5.6968	2907700	20	13.86	14.18	14.78	14.10	14.50	13.99	14.39	13.55
0.177	0.12	1.09	Q9DCT5	Stromal cell-derived factor 2	Sdf2	5231	2	2	2	18	18	18	23.159	0.0000	7.4174	1934500	41	13.76	14.47	13.96	13.91	13.41	13.80	13.99	14.42
0.364	0.12	1.09	Q9CYG7	Mitochondrial import receptor subunit TOM34	Tomm34	4796	27	27	27	78	78	78	34.278	0.0000	262.76	143170000	696	18.24	18.00	18.59	18.34	18.04	18.35	18.08	18.22
0.181	0.12	1.08	Q60780	Growth arrest-specific protein 7	Gas7	2206	14	14	14	33	33	33	48.173	0.0000	135.53	20877000	259	16.13	16.20	15.38	15.75	15.98	16.04	15.32	15.65
0.108	0.12	1.08	Q91WM2	Cat eye syndrome critical region protein 5 homolog	Cecr5	4120	5	5	5	16.2	16.2	16.2	46.306	0.0000	22.316	5659800	81	14.40	14.68	15.41	14.88	14.84	15.25	15.07	13.74
0.172	0.12	1.08	Q8BQM4	HEAT repeat-containing protein 3	Heatr3	3171	6	6	6	11.8	11.8	11.8	74.305	0.0000	15.506	6911300	65	15.15	14.99	14.73	15.17	14.43	15.21	15.39	14.52
0.553	0.12	1.08	Q8VCT3	Aminopeptidase B	Rnpep	3932	36	36	36	57.8	57.8	57.8	72.415	0.0000	323.31	209300000	906	18.18	18.29	18.02	18.00	18.17	17.86	17.93	18.06
0.387	0.11	1.08	P54116	Erythrocyte band 7 integral membrane protein	Stom	1248	19	19	19	65.1	65.1	65.1	31.375	0.0000	323.31	469160000	1016	19.72	19.70	20.03	19.71	19.66	19.94	19.66	19.45
0.429	0.11	1.08	O35129	Prohibitin-2	Phb2	339	28	28	28	82.3	82.3	82.3	33.296	0.0000	323.31	305020000	851	19.33	18.95	18.88	19.11	18.96	18.86	19.13	18.86
0.391	0.11	1.08	O08547	Vesicle-trafficking protein SEC22b	Sec22b	270	20	20	20	79.1	79.1	79.1	24.74	0.0000	294.03	121490000	407	17.96	18.07	18.39	18.10	17.87	17.87	18.21	18.11
0.209	0.11	1.08	P63325	40S ribosomal protein S10	Rps10	1505	17	16	16	67.3	67.3	67.3	18.916	0.0000	107.02	168720000	491	19.33	19.00	18.63	19.12	18.96	18.46	19.11	19.10
0.343	0.11	1.08	Q922R8	Protein disulfide-isomerase A6	Pdia6	4276	27	27	27	53.9	53.9	53.9	48.1	0.0000	323.31	453530000	956	19.27	19.31	19.80	19.58	19.39	19.22	19.52	19.38
0.229	0.11	1.08	Q9R112	Sulfide:quinone oxidoreductase, mitochondrial	Sqrdl	5645	33	33	33	62.9	62.9	62.9	50.282	0.0000	323.31	170830000	778	18.05	17.80	17.70	17.52	18.03	17.32	17.81	17.46
0.212	0.11	1.08	Q8K0D5	Elongation factor G, mitochondrial	Gfm1	3604	19	19	19	36.2	36.2	36.2	83.549	0.0000	87.59	28322000	203	16.23	15.68	16.38	16.48	16.18	15.77	16.12	16.26
0.245	0.11	1.08	Q9D0F9	Phosphoglucomutase-1	Pgm1	4865	37	37	37	66.4	66.4	66.4	61.417	0.0000	301.81	87643000	767	17.18	16.82	17.09	17.27	16.57	17.00	17.02	17.33
0.073	0.11	1.08	P59997	Lysine-specific demethylase 2A	Kdm2a	1347	2	2	2	2.8	2.8	2.8	132.68	0.0000	7.0915	1995600	43	13.26	13.78	15.02	13.43	13.69	12.76	14.20	14.41
0.244	0.11	1.08	Q07813	Apoptosis regulator BAX	Bax	1758	9	9	9	47.4	47.4	47.4	21.394	0.0000	323.31	199600000	554	19.08	18.87	19.60	19.19	18.81	19.21	19.26	19.01
0.300	0.11	1.08	Q8BX70	Vacuolar protein sorting-associated protein 13C	Vps13c	3279	116	116	116	41.1	41.1	41.1	420.08	0.0000	323.31	199780000	1792	17.34	16.92	17.57	17.51	17.20	17.22	17.35	17.13
0.133	0.11	1.08	Q9DCS9	NADH dehydrogenase [ubiquinone] 1 beta subcomplex subunit 10	Ndufb10	5228	7	7	7	38.1	38.1	38.1	21.024	0.0000	21.571	25402000	126	16.19	17.28	16.39	16.69	16.64	16.28	17.04	16.15
0.291	0.11	1.08	P04230;P01	H-2 class II histocompatibility antigen, E-B beta chain	H2-Eb1	639	5	5	2	20.1	20.1	8	30.166	0.0000	13.12	6768500	31	14.81	14.46	14.79	15.05	14.71	14.77	14.38	14.81
0.601	0.11	1.08	Q00612	Glucose-6-phosphate 1-dehydrogenase X	G6pdx	1699	56	56	44	79	79	68.7	59.262	0.0000	323.31	888940000	2038	20.15	20.23	20.00	19.96	19.96	20.13	19.86	19.95
0.280	0.11	1.08	P17225	Polypyrimidine tract-binding protein 1	Ptbp1	821	26	26	21	62.2	62.2	57.1	56.477	0.0000	323.31	345040000	1109	19.10	18.92	19.55	19.37	19.04	19.01	19.08	19.37
0.089	0.11	1.08	P17809	Solute carrier family 2, facilitated glucose transporter member 1	Slc2a1	831	5	5	5	11.2	11.2	11.2	53.984	0.0000	68.322	8924900	80	15.26	14.57	16.48	15.91	15.31	15.32	15.94	15.22
0.117	0.11	1.08	O54950	5-AMP-activated protein kinase subunit gamma-1	Prkag1	436	12	12	11	49.1	49.1	46.1	37.52	0.0000	104.52	8867900	61	15.54	14.63	16.04	15.75	15.77	15.41	15.33	15.01
0.199	0.11	1.08	P42225	Signal transducer and activator of transcription 1	Stat1	1075	49	49	49	57.3	57.3	57.3	87.196	0.0000	323.31	208230000	1213	17.67	17.47	17.31	17.17	17.65	17.57	17.07	16.90
0.123	0.11	1.08	P63028	Translationally-controlled tumor protein	Tpt1	1481	10	10	10	37.2	37.2	37.2	19.462	0.0000	70.794	41537000	224	16.75	17.43	16.36	17.14	16.94	17.35	16.71	16.26
0.189	0.11	1.08	Q8VCF0	Mitochondrial antiviral-signaling protein	Mavs	3918	10	10	10	32.8	32.8	32.8	53.398	0.0000	112.89	32455000	175	16.65	16.75	16.78	16.65	16.02	16.50	16.97	16.91
0.916	0.11	1.08	Q6PHZ2;P1	Calcium/calmodulin-dependent protein kinase type II subunit delta	Camk2d	2642	22	22	17	48.5	48.5	40.3	56.369	0.0000	296.59	104430000	529	17.77	17.91	18.04	17.93	17.82	17.86	17.76	17.78
0.227	0.11	1.08	O54774	AP-3 complex subunit delta-1	Ap3d1	419	34	34	34	37.8	37.8	37.8	135.08	0.0000	323.31	111700000	763	17.95	17.50	18.08	18.04	17.39	17.94	17.89	17.93
0.232	0.11	1.08	Q9JM52	Misshapen-like kinase 1	Mink1	5498	20	20	10	19.4	19.4	12.3	147.29	0.0000	173.44	19606000	194	15.68	15.21	15.49	15.50	14.94	15.35	15.50	15.67
0.048	0.11	1.08	Q80U56	Late secretory pathway protein AVL9 homolog	Avl9	2819	5	5	5	13.6	13.6	13.6	72.185	0.0000	17.335	2994200	36	15.12	14.42	12.11	14.59	14.49	12.86	14.48	13.99
0.181	0.10	1.08	P62878	E3 ubiquitin-protein ligase RBX1;E3 ubiquitin-protein ligase RBX1, N-tei	Rbx1	1461	4	4	4	33.3	33.3	33.3	12.274	0.0000	48.521	23257000	165	16.47	16.83	17.08	16.86	16.87	16.15	16.81	16.99
0.327	0.10	1.08	Q61210	Rho guanine nucleotide exchange factor 1	Arhgef1	2278	45	45	45	47.4	47.4	47.4	102.8	0.0000	323.31	108880000	819	17.81	17.55	17.32	17.36	17.30	17.63	17.28	17.40
0.040	0.10	1.07	Q8BJ34	Meiosis arrest female protein 1	Marf1	3070	7	7	7	4.9	4.9	4.9	192.06	0.0000	8.1017	2827200	26	12.14	12.80	15.19	12.88	13.09	12.16	14.85	12.49
0.103	0.10	1.07	Q7TNP2	Serine/threonine-protein phosphatase 2A 65 kDa regulatory subunit A b	Ppp2r1b	2763	22	12	11	49.1	34.6	28.1	65.934	0.0000	56.292	12099000	125	14.99	14.77	15.68	15.19	15.80	15.29	14.78	14.34
0.150	0.10	1.07	P21460	Cystatin-C	Cst3	865	3	3	3	26.4	26.4	26.4	15.531	0.0000	32.809	6307500	73	14.74	15.66	15.41	15.35	15.20	15.64	15.11	14.79
0.095	0.10	1.07	Q9JLC3	Methionine-R-sulfoxide reductase B1	Msrb1	5476	3	3	2	36.2	36.2	15.5	12.788	0.0025	2.7961	7617300	44	15.70	15.28	15.84	17.07	15.82	15.83	16.15	15.68
0.074	0.10	1.07	Q8CF89	TGF-beta-activated kinase 1 and MAP3K7-binding protein 1	Tab1	3476	7	7	7	21.1	21.1	21.1	54.616	0.0000	7.6827	3301400	23	14.51	14.80	14.50	13.24	13.98	14.77	14.65	13.24
0.730	0.10	1.07	P29452	Caspase-1;Caspase-1 subunit p20;Caspase-1 subunit p10	Casp1	970	29	29	29	60.7	60.7	60.7	45.64	0.0000	186.35	115900000	691	17.53	17.54	17.59	17.74	17.37	17.47	17.54	17.60
0.204	0.10	1.07	Q8K224	N-acetyltransferase 10	Nat10	3643	27	27	27	36.6	36.6	36.6	115.42	0.0000	323.31	28055000	409	15.50	15.03	15.50	15.56	14.90	15.64	15.28	15.36
0.128	0.10	1.07	Q62504	Msx2-interacting protein	Spen	2399	5	5	5	1.9	1.9	1.9	398.75	0.0000	12.694	1857500	16	12.61	13.45	13.34	13.59	12.95	12.92	13.75	12.98
0.051	0.10	1.07	Q9JL56	Glycerophosphodiester phosphodiesterase 1	Gde1	5472	5	5	5	17.5	17.5	17.5	37.629	0.0000	9.6362	4802000	38	14.73	12.41	15.30	14.96	13.90	13.84	14.60	14.66
0.386	0.10	1.07	Q60902	Epidermal growth factor receptor substrate 15-like 1	Eps15l1	2220	16	16	16	27.7	27.7	27.7	99.307	0.0000	82.486	14381000	169	15.63	15.48	15.55	15.73	15.32	15.79	15.43	15.45
0.282	0.10	1.07	Q8R1A4;Q8	Dedicator of cytokinesis protein 7	Dock7	3782	36	34	34	22.4	21.2	21.2	241.44	0.0000	146.95	46772000	463	16.14	15.74	15.96	16.01	15.60	15.72	16.06	16.08
0.551	0.10	1.07	Q78PY7	Staphylococcal nuclease domain-containing protein 1	Snd1	2728	70	70	70	78.9	78.9	78.9	102.09	0.0000	323.31	484700000	1686	19.77	19.66	20.04	19.76	19.68	19.66	19.75	19.74
0.428	0.10	1.07	Q9R0X4;Q3	Acyl-coenzyme A thioesterase 9, mitochondrial;Acyl-coenzyme A thioes	Acot9;Acot10	5643	25	25	24	50.6	50.6	50.6	50.56	0.0000	129.22	84996000	550	17.27	17.17	17.19	16.89	17.03	16.88	17.04	17.17
0.452	0.10	1.07	Q8BPB0;Q9	MOB kinase activator 1B;MOB kinase activator 1A	Mob1b;Mob1a	3163	8	8	8	37.5	37.5	37.5	25.091	0.0000	60.161	41792000	176	17.36	17.35	17.44	17.69	17.25	17.46	17.28	17.46
0.312	0.10	1.07	Q64311	Protein N-terminal asparagine amidohydrolase	Ntan1	2429	3	3	3	14.5	14.5	14.5	34.595	0.0000	15.372	4707400	58	14.29	14.70	14.56	14.38	14.38	14.12	14.56	14.49
0.080	0.10	1.07	Q9ERF3	WD repeat-containing protein 61;WD repeat-containing protein 61, N-tei	Wdr61	5308	6	6	6	38.7	38.7	38.7	33.772	0.0000	45.677	4149400	43	13.79	14.90	14.44	14.59	14.39	15.22	14.33	13.40
0.207	0.10	1.07	Q8K019	Bcl-2-associated transcription factor 1	Bclaf1	3595	24	24	24	26.6	26.6	26.6	106	0.0000	48.928	43874000	405	16.52	16.51	16.14	16.46	16.21	16.49	15.90	16.64
0.067	0.10	1.07	Q8VDV8	MIT domain-containing protein 1	Mitd1	3974	3	3	3	17.3	17.3	17.3	28.847	0.0000	9.5053	1761100	24	13.50	13.46	13.04	13.35	13.71	12.12	12.73	14.39
0.199	0.10	1.07	O55013	Trafficking protein particle complex subunit 3	Trappc3	441	7	7	7	33.3	33.3	33.3	20.302	0.0000	16.325	15080000	124	16.20	15.69	16.23	16.31	16.18	15.65	16.01	16.20
0.545	0.10	1.07	P70168	Importin subunit beta-1	Kpnb1	1529	41	41	40	51.1	51.1	51.1	97.183	0.0000	323.31	302790000	1009	19.21	19.02	19.29	19.19	18.93	19.22	19.10	19.08
0.221	0.10	1.07	Q61550	Double-strand-break repair protein rad21 homolog	Rad21	2312	12	12	12	21.9	21.9	21.9	72.082	0.0000	59.918	23581000	245	15.96	15.71	16.36	16.11	15.68	16.09	16.15	15.84
0.078	0.10	1.07	Q6P1E1	Zinc finger MIZ domain-containing protein 1	Zmiz1	2553	3	3	2	4.7	4.7	3.7	115.85	0.0000	15.067	941270	22	13.32	13.21	13.37	12.83	12.92	13.37	12.02	14.04
0.070	0.10	1.07	A2AJL3	FGGY carbohydrate kinase domain-containing protein	Fggy	30	2	2	2	5.8	5.8	5.8	60.336	0.0000	17.266	767480	20	13.04	13.13	12.79	13.89	14.39	12.72	12.71	12.65
0.184	0.09	1.07	P07091	Protein S100-A4	S100a4	667	13	13	13	64.4	64.4	64.4	11.721	0.0000	87.084	496300000	529	21.27	21.80	20.98	21.32	21.29	21.47	20.97	21.27
0.129	0.09	1.07	Q8BHL8	Proteasome inhibitor PI31 subunit	Psmf1	3047	4	4	4	20.7	20.7	20.7	29.664	0.0000	7.0481	3920200	40	14.93	15.11	14.14	14.67	14.12	14.81	14.64	14.91
0.136	0.09	1.07	Q8CAY6	Acetyl-CoA acetyltransferase, cytosolic	Acat2	3432	24	24	24	75.1	75.1	75.1	41.297	0.0000	323.31	103490000	498	18.03	17.99	17.73	17.89	18.16	18.33	17.40	17.37
0.252	0.09	1.07	P70336	Rho-associated protein kinase 2	Rock2	1556	33	30	29	27.5	26.3	24.4	160.58	0.0000	102.15	46987000	346	16.96	16.56	17.17	17.12	16.71	16.84	16.97	16.92
0.429	0.09	1.07	P61161	Actin-related protein 2	Actr2	1372	45	45	44	80.2	80.2	80.2	44.76	0.0000	323.31	727520000	1648	20.56	20.38	20.36	20.10	20.30	20.26	20.25	20.22
0.089	0.09	1.07	Q91WG8	Bifunctional UDP-N-acetylglucosamine 2-epimerase/N-acetylmannosam	Gne	4112	6	6	6	13.7	13.7	13.7	79.198	0.0000	12.28	1984400	13	14.17	14.16	13.51	14.08	13.02	14.56	14.26	13.72
0.160	0.09	1.07	Q9DBD5	Proline-, glutamic acid- and leucine-rich protein 1	Pelp1	5144	5	5	5	8.8	8.8	8.8	118.07	0.0000	102.19	6158800	100	14.90	14.58	15.18	14.98	14.86	14.55	15.31	14.55
0.118	0.09	1.07	Q64520	Guanylate kinase	Guk1	2452	8	8	7	48.5	48.5	40.4	21.918	0.0000	42.335	6052000	61	14.64	14.65	14.06	14.71	14.83	13.72	14.51	14.64
0.091	0.09	1.07	O54784	Death-associated protein kinase 3	Dapk3	422	3	3	3	8.3	8.3	8.3	51.421	0.0000	7.7025	1958400	29	14.34	13.97	13.69	13.27	13.76	12.91	14.07	14.17
0.075	0.09	1.06	Q3TSG4	RNA demethylase ALKBH5	Alkbh5	1873	5	5	5	17.2	17.2	17.2	44.41	0.0000	132.75	9034400	103	14.73	14.50	15.50	15.68	14.34	15.78	15.26	14.68
0.656	0.09	1.06	Q99KC8	von Willebrand factor A domain-containing protein 5A	Vwa5a	4370	49	49	49	55.6	55.6	55.6	87.142	0.0000	323.31	499770000	1873	19.64	19.44	19.70	19.60	19.48	19.45	19.61	19.48
0.370	0.09	1.06	Q7TMQ7	WD repeat-containing protein 91	Wdr91	2748	28	28	28	41.2	41.2	41.2	83.42	0.0000	212.02	54782000	500	16.88	16.45	16.75	16.72	16.50	16.61	16.58	16.75
0.228	0.09	1.06	Q9D1M0	Protein SEC13 homolog	Sec13	4925	9	9	9	47.8	47.8	47.8	35.565	0.0000	250.91	51084000	186	17.95	17.86	17.89	18.17	17.92	17.70	17.64	18.26
0.133	0.09	1.06	Q9JLQ0	CD2-associated protein	Cd2ap	5487	14	14	13	34.5	34.5	31.4	70.449	0.0000	56.774	3779500	25	11.19	11.54	11.76	11.92	11.53	12.05	11.32	11.16
0.218	0.09	1.06	Q9EQ61	Pescadillo homolog	Pes1	5272	10	10	10	17	17	17	67.795	0.0000	56.412	16081000	181	15.61	15.32	15.93	15.71	15.40	15.65	15.38	15.79
0.404	0.09	1.06	Q9JHF7	Hematopoietic prostaglandin D synthase	Hpgds	5358	9	9	9	56.8	56.8	56.8	23.227	0.0000	105.35	39718000	178	17.81	17.71	17.54	17.84	17.82	17.66	17.57	17.51
0.292	0.09	1.06	P57080	Ubiquitin carboxyl-terminal hydrolase 25	Usp25	1295	13	13	13	14.3	14.3	14.3	121.42	0.0000	64.067	12193000	188	15.36	15.24	15.07	15.27	14.94	15.42	15.01	15.23
0.473	0.09	1.06	Q8K1X4		Nckap1l	3636	41	41	39	42.6	42.6	41.8	128.9	0.0000	323.31	135770000	953	17.44	17.21	17.25	17.29	17.35	17.05	17.16	17.28
0.173	0.09	1.06	Q6NVF0	Inositol polyphosphate 5-phosphatase OCRL-1	Ocrl	2533	7	7	7	11.3	11.3	11.3	104.28	0.0000	29.857	7392300	89	14.62	14.62	15.25	15.22	14.74	14.71	15.05	14.86
0.140	0.09	1.06	P70206;P70	Plexin-A1	Plxna1	1536	33	32	29	23	22.4	20.5	211.1	0.0000	223.13	36141000	327	15.73	15.74	16.52	16.12	16.09	15.79	16.24	15.65

0.312	0.09	1.06	Q80U93	Nuclear pore complex protein Nup214	Nup214	2826	24	24	24	18.4	18.4	18.4	212.98	0.0000	144.68	31229000	413	16.05	15.80	16.30	16.06	15.91	16.02	15.86	16.08
0.132	0.08	1.06	Q80X95	Ras-related GTP-binding protein A	Rraga	2877	15	15	6	52.4	52.4	23	36.566	0.0000	117.78	26790000	250	16.13	16.42	15.55	15.51	16.09	15.75	15.74	15.68
0.076	0.08	1.06	Q3UHH8	Glucoside xylosyltransferase 1	Gxylt1	1973	6	6	6	14.6	14.6	14.6	46.49	0.0000	11.19	1893600	44	13.58	13.02	13.85	13.85	12.85	13.06	13.65	14.39
0.246	0.08	1.06	Q9WV80;RE	Sorting nexin-1	Snx1	5736	34	32	32	57.7	53.6	53.6	58.951	0.0000	323.31	199820000	823	18.17	18.19	18.62	18.60	18.17	18.29	18.32	18.46
0.756	0.08	1.06	Q99020	Heterogeneous nuclear ribonucleoprotein A/B	Hnrnpab	4314	22	22	21	54.4	54.4	54.4	30.831	0.0000	207.81	60887000	411	17.35	17.38	17.33	17.33	17.19	17.20	17.26	17.41
0.262	0.08	1.06	Q99JI6	Ras-related protein Rap-1b	Rap1b	4341	20	20	8	85.9	85.9	38.6	20.825	0.0000	323.31	304390000	855	19.97	19.63	19.40	19.83	19.60	19.55	19.66	19.69
0.923	0.08	1.06	Q8BTM8	Filamin-A	Flna	3204	184	184	175	70	70	68.4	281.22	0.0000	323.31	2804600000	6735	20.12	20.03	20.23	20.13	20.02	20.03	20.05	20.09
0.261	0.08	1.06	Q61316	Heat shock 70 kDa protein 4	Hspa4	2290	69	69	64	71.6	71.6	67.7	94.132	0.0000	323.31	373430000	1895	17.94	17.89	18.37	18.25	17.89	18.10	18.03	18.10
0.250	0.08	1.06	Q91VR2	ATP synthase subunit gamma, mitochondrial	Atp5c1	4072	22	22	22	58.7	58.7	58.7	32.886	0.0000	323.31	175060000	728	18.57	18.09	18.02	18.25	18.26	18.20	18.10	18.06
0.162	0.08	1.06	P70429	Ena/VASP-like protein	Evl	1572	23	23	23	63.8	63.8	63.8	44.337	0.0000	224.64	93860000	586	16.97	16.89	16.77	16.71	16.37	16.52	17.04	17.09
0.296	0.08	1.06	O09044	Synaptosomal-associated protein 23	Snap23	319	18	18	18	85.2	85.2	85.2	23.261	0.0000	323.31	129520000	527	18.56	18.27	18.49	18.72	18.26	18.52	18.39	18.55
0.279	0.08	1.06	Q8CI11	Guanine nucleotide-binding protein-like 3	Gnl3	3537	6	6	6	17.5	17.5	17.5	60.786	0.0000	73.755	3353500	30	13.89	13.71	13.97	13.71	13.51	13.81	13.97	13.67
0.161	0.08	1.06	O09000	Nuclear receptor coactivator 3	Ncoa3	315	6	6	6	6.8	6.8	6.8	151.57	0.0000	35.541	4911100	76	14.46	14.60	15.22	15.01	14.80	14.60	14.90	14.68
0.147	0.08	1.05	Q91X52	L-xylulose reductase	Dcxr	4135	14	14	14	69.7	69.7	69.7	25.746	0.0000	111.45	57029000	424	17.17	16.70	17.23	17.15	16.88	17.45	16.83	16.78
0.086	0.08	1.05	P84084	ADP-ribosylation factor 5	Arf5	1606	12	5	5	67.8	27.8	27.8	20.529	0.0000	42.167	30805000	175	16.60	17.50	17.83	17.82	17.27	17.46	17.72	17.01
0.178	0.08	1.05	Q62426	Cystatin-B	Cstb	2391	12	12	12	93.9	93.9	93.9	11.045	0.0000	252.45	523680000	892	20.52	20.82	20.36	20.71	20.15	20.60	20.56	20.79
0.069	0.08	1.05	Q9EPB5	Serine hydrolase-like protein	Serhl	5251	3	3	3	18	18	18	35.31	0.0000	10.493	2197000	27	14.90	13.71	13.72	14.11	13.82	14.84	13.66	13.83
0.060	0.08	1.05	Q8BJM3	Coiled-coil domain-containing protein R3HCC1L	R3hcc1l	3081	4	4	4	7.6	7.6	7.6	84.48	0.0000	13.116	976500	11	12.94	13.26	14.18	12.09	13.02	12.81	13.02	13.32
0.066	0.07	1.05	Q8R5M8	Cell adhesion molecule 1	Cadm1	3893	3	3	3	13.6	13.6	13.6	49.788	0.0000	59.541	2725300	38	14.56	14.30	14.80	14.71	15.21	15.03	14.34	13.49
0.070	0.07	1.05	Q05793	Basement membrane-specific heparan sulfate proteoglycan core protein	Hspg2	1741	7	7	7	2.9	2.9	2.9	398.29	0.0000	32.668	1963000	40	12.49	13.05	13.91	12.78	13.21	12.39	12.91	13.42
0.226	0.07	1.05	Q9D880	Mitochondrial import inner membrane translocase subunit TIM50	Timm50	5053	18	18	18	38	38	38	39.776	0.0000	170.07	46372000	331	16.70	16.72	17.14	16.97	16.60	16.82	16.84	16.98
0.163	0.07	1.05	Q4VA53	Sister chromatid cohesion protein PDS5 homolog B	Pds5b	2066	25	25	25	23	23	23	164.42	0.0000	123.48	37546000	248	17.16	16.69	17.29	17.12	16.81	17.30	16.85	17.00
0.103	0.07	1.05	Q99MR8	Methylcrotonoyl-CoA carboxylase subunit alpha, mitochondrial	Mccc1	4475	23	23	23	44.6	44.6	44.6	79.343	0.0000	91.578	34850000	270	15.93	15.62	16.34	16.23	16.05	15.71	16.49	15.59
0.129	0.07	1.05	Q78ZA7	Nucleosome assembly protein 1-like 4	Nap1l4	2732	16	16	14	56.3	56.3	53.6	42.679	0.0000	186.36	64991000	325	17.02	17.32	16.97	17.12	16.78	17.59	16.76	17.01
0.215	0.07	1.05	P24369	Peptidyl-prolyl cis-trans isomerase B	Ppib	896	24	24	24	75.9	75.9	75.9	23.713	0.0000	254.98	304520000	793	19.03	18.95	19.18	19.38	18.83	19.08	19.29	19.05
0.112	0.07	1.05	Q9ES46;Q9	Beta-parvin	Parvb	5327	14	14	13	41.9	41.9	38.6	41.669	0.0000	82.632	35878000	187	17.13	17.28	17.59	17.76	17.27	16.93	17.47	17.82
0.175	0.07	1.05	Q99J47	Dehydrogenase/reductase SDR family member 7B	Dhrs7b	4325	10	10	10	36.5	36.5	36.5	34.986	0.0000	67.839	25974000	276	16.04	15.47	16.11	16.00	15.85	15.70	15.80	15.97
0.269	0.07	1.05	Q9ERS2	NADH dehydrogenase [ubiquinone] 1 alpha subcomplex subunit 13	Ndufa13	5317	12	12	12	60.4	60.4	60.4	16.859	0.0000	84.173	38221000	237	16.74	16.71	17.02	16.97	16.75	16.88	16.93	16.60
0.343	0.07	1.05	P08003	Protein disulfide-isomerase A4	Pdia4	674	52	52	52	59.4	59.4	59.4	71.982	0.0000	323.31	312360000	1390	18.24	18.31	18.39	18.52	18.15	18.26	18.31	18.46
0.052	0.07	1.05	Q61207	Prosaposin	Psap	2277	9	9	9	19.9	19.9	19.9	61.422	0.0000	38.247	51786000	205	16.21	18.05	18.28	17.87	17.46	17.70	17.33	17.64
0.434	0.07	1.05	Q9Z0P5	Twinfilin-2	Twf2	5780	25	25	22	77.4	77.4	71.3	39.47	0.0000	323.31	147000000	642	18.22	18.21	17.98	18.21	17.98	18.14	18.17	18.04
0.179	0.07	1.05	Q9QXK7	Cleavage and polyadenylation specificity factor subunit 3	Cpsf3	5544	12	12	12	24.3	24.3	24.3	77.504	0.0000	90.465	19409000	85	15.95	16.56	16.24	16.06	16.25	15.94	16.09	16.25
0.110	0.07	1.05	P49935	Pro-cathepsin H;Cathepsin H mini chain;Cathepsin H;Cathepsin H heav	Ctsh	1172	10	10	10	38.4	38.4	38.4	37.17	0.0000	29.898	24968000	149	17.34	16.89	17.05	16.62	16.95	16.40	17.00	17.26
0.220	0.07	1.05	Q60865	Caprin-1	Caprin1	2215	14	14	14	26	26	26	78.168	0.0000	121.42	19608000	217	15.38	15.67	15.86	15.46	15.55	15.49	15.37	15.69
0.049	0.07	1.05	Q3TYS2	Uncharacterized protein C17orf62 homolog		1889	6	6	6	34.2	34.2	34.2	20.921	0.0000	35.977	26278000	132	17.62	17.08	15.35	16.31	16.43	16.54	16.63	16.49
0.041	0.07	1.05	Q8R3F9	Speckle targeted PIP5K1A-regulated poly(A) polymerase	Tut1	3845	5	5	5	11.6	11.6	11.6	94.602	0.0000	32.71	2772000	46	14.91	13.42	13.88	13.73	12.51	14.53	14.27	14.36
0.158	0.07	1.05	Q62167;P16	ATP-dependent RNA helicase DDX3X;Putative ATP-dependent RNA he	Ddx3x;D1Pas1	2360	52	51	20	78.9	78.9	37.9	73.101	0.0000	323.31	383660000	1355	19.51	19.22	19.07	19.34	19.21	19.53	18.87	19.25
0.301	0.07	1.05	O35654	DNA polymerase delta subunit 2	Pold2	393	10	10	10	30.9	30.9	30.9	51.354	0.0000	44.819	7882500	95	15.62	15.53	15.60	15.27	15.42	15.58	15.37	15.38
0.057	0.07	1.05	Q6ZPZ3	Zinc finger CCCH domain-containing protein 4	Zc3h4	2687	5	5	5	7.4	7.4	7.4	140.97	0.0000	17.335	3098800	49	13.03	13.79	14.60	13.93	14.11	14.17	13.75	13.04
0.042	0.07	1.05	Q8CD15	Bifunctional lysine-specific demethylase and histidyl-hydroxylase MINA	Mina	3455	3	3	3	8.4	8.4	8.4	53.516	0.0000	21.866	2411200	26	14.47	14.05	12.29	14.21	13.60	14.35	13.23	13.59
0.243	0.07	1.05	P47738	Aldehyde dehydrogenase, mitochondrial	Aldh2	1117	53	53	50	80.7	80.7	79.2	56.537	0.0000	323.31	1524400000	2402	20.62	20.74	21.10	20.84	20.74	20.65	20.85	20.78
0.258	0.06	1.05	Q7TQH0	Ataxin-2-like protein	Atxn2l	2774	12	12	12	17.6	17.6	17.6	110.65	0.0000	48.943	32251000	225	16.92	16.79	16.90	16.96	16.66	17.09	16.72	16.86
0.107	0.06	1.05	P47856;Q92	Glutamine--fructose-6-phosphate aminotransferase [isomerizing] 1	Gfpt1	1127	11	11	11	24	24	24	78.538	0.0000	143.11	19633000	200	15.63	16.53	16.37	16.15	16.39	15.97	15.98	16.09
0.247	0.06	1.05	P53994	Ras-related protein Rab-2A	Rab2a	1243	18	18	12	75.5	75.5	47.2	23.547	0.0000	272.83	139920000	455	18.46	18.42	18.35	18.36	18.59	18.12	18.40	18.23
0.081	0.06	1.04	Q99ME9	Nucleolar GTP-binding protein 1	Gtpbp4	4464	15	15	15	24.8	24.8	24.8	74.112	0.0000	21.193	14642000	111	15.63	15.29	15.12	15.49	14.81	16.04	15.18	15.25
0.086	0.06	1.04	Q9CZX9	ER membrane protein complex subunit 4	Emc4	4847	7	7	7	32.2	32.2	32.2	20.116	0.0000	30.724	11052000	96	14.97	15.49	16.10	15.85	15.29	15.59	15.53	15.75
0.012	0.06	1.04	Q924K1	Aryl-hydrocarbon-interacting protein-like 1	Aipl1	4294	3	3	3	14.3	14.3	14.3	38.212	0.0056	2.1784	21238000	30	16.65	17.01	12.79	17.31	17.92	14.26	18.32	13.01
0.162	0.06	1.04	O70318	Band 4.1-like protein 2	Epb41l2	488	39	39	37	44.4	44.4	43.7	109.94	0.0000	278.5	94522000	626	17.29	16.92	17.48	17.50	17.08	17.25	17.35	17.28
0.092	0.06	1.04	P15379	CD44 antigen	Cd44	796	5	5	5	8.7	8.7	8.7	85.616	0.0000	40.254	57891000	174	18.89	18.15	18.43	19.07	18.28	18.57	18.56	18.88
0.091	0.06	1.04	Q5ND34	WD repeat-containing protein 81	Wdr81	2121	31	31	31	27.5	27.5	27.5	211.94	0.0000	323.31	44050000	378	15.83	16.07	16.77	16.37	16.05	16.23	16.56	15.94
0.093	0.06	1.04	Q02105	Complement C1q subcomponent subunit C	C1qc	1716	6	6	6	29.7	29.7	29.7	25.991	0.0000	28.581	34675000	128	17.93	17.62	17.57	17.53	17.93	17.96	17.48	17.05
0.137	0.06	1.04	P40336	Vacuolar protein sorting-associated protein 26A	Vps26a	1061	22	22	21	70.9	70.9	68.8	38.113	0.0000	323.31	93534000	549	17.81	17.78	17.21	17.44	17.65	17.58	17.30	17.48
0.042	0.06	1.04	Q8K0P3	TLD domain-containing protein 1	Tldc1	3608	2	2	2	7	7	7	50.844	0.0000	29.804	861570	25	14.41	13.50	13.43	12.90	14.47	13.53	13.32	12.67
0.395	0.06	1.04	B2RY56	RNA-binding protein 25	Rbm25	89	26	26	26	32.6	32.6	32.6	99.551	0.0000	180.75	33380000	353	16.19	16.03	16.19	15.99	15.93	16.08	16.03	16.12
0.222	0.06	1.04	Q9JKY0	Cell differentiation protein RCD1 homolog	Rqcd1	5464	6	6	6	26.8	26.8	26.8	33.601	0.0000	72.456	11415000	141	15.71	15.48	15.75	15.43	15.40	15.65	15.42	15.65
0.107	0.06	1.04	Q8BRF7	Sec1 family domain-containing protein 1	Scfd1	3179	25	25	25	50.2	50.2	50.2	72.322	0.0000	202.58	78367000	571	16.53	16.68	17.21	16.97	16.41	16.82	16.90	17.03
0.068	0.06	1.04	Q8VI93	2-5-oligoadenylate synthase 3	Oas3	4032	30	30	30	42.3	42.3	42.3	126.33	0.0000	323.31	121980000	607	18.58	18.47	17.80	17.46	18.23	18.28	17.91	17.66
0.182	0.06	1.04	P70697	Uroporphyrinogen decarboxylase	Urod	1585	15	15	15	61	61	61	40.691	0.0000	170.06	27493000	191	16.59	16.47	16.49	16.33	16.66	16.36	16.49	16.14
0.096	0.06	1.04	Q3UJB9	Enhancer of mRNA-decapping protein 4	Edc4	1983	23	23	22	26.9	26.9	26.6	152.48	0.0000	286.32	43908000	417	16.35	15.91	16.83	16.37	16.18	16.57	16.38	16.10
0.336	0.06	1.04	P40142	Transketolase	Tkt	1057	67	67	66	90.5	90.5	89.9	67.63	0.0000	323.31	4068900000	5169	22.25	22.12	22.40	22.35	22.28	22.28	22.21	22.12
0.135	0.06	1.04	Q8C854	Myelin expression factor 2	Myef2	3420	18	18	18	34.9	34.9	34.9	63.294	0.0000	89.675	39833000	270	16.65	16.90	17.29	16.86	16.83	17.05	16.94	16.66
0.093	0.06	1.04	Q9CVB6	Actin-related protein 2/3 complex subunit 2	Arpc2	4706	35	35	35	90.3	90.3	90.3	34.357	0.0000	323.31	464170000	1489	19.38	19.21	18.43	19.18	19.00	18.90	18.91	19.16
0.191	0.06	1.04	Q9D1A2	Cytosolic non-specific dipeptidase	Cndp2	4903	57	57	57	86.7	86.7	86.7	52.767	0.0000	323.31	2197000000	3173	20.65	20.89	21.11	21.01	20.80	20.85	21.03	20.76
0.149	0.06	1.04	Q9Z110	Delta-1-pyrroline-5-carboxylate synthase;Glutamate 5-kinase;Gamma-g	Aldh18a1	5795	28	28	28	45.8	45.8	45.8	87.265	0.0000	303.36	72541000	463	16.76	16.76	17.21	16.64	16.97	16.79	16.76	16.64
0.089	0.05	1.04	P46664	Adenylosuccinate synthetase isozyme 2	Adss	1109	29	29	26	55.7	55.7	49.3	50.02	0.0000	245.34	182350000	726	18.37	18.69	19.33	18.85	18.57	19.02	18.72	18.72
0.110	0.05	1.04	O70494	Transcription factor Sp3	Sp3	508	3	2	2	3.3	2.2	2.2	82.361	0.0000	17.99	909160	38	13.00	13.27	13.10	13.50	13.18	12.76	13.35	13.38
0.057	0.05	1.04	Q9CZR8	Elongation factor Ts, mitochondrial	Tsfm	4837	10	10	10	60.5	60.5	60.5	35.334	0.0000	166.76	16524000	135	16.10	16.55	16.96	16.76	17.33	16.35	16.10	16.37
0.050	0.05	1.04	Q99M04	Lipoyl synthase, mitochondrial	Lias	4451	4	4	4	19	19	19	41.879	0.0000	11.531	2920000	25	13.99	14.18	13.94	13.70	14.23	14.19	14.37	12.82
0.179	0.05	1.04	Q7TMM9	Tubulin beta-2A chain	Tubb2a	2747	37	9	2	73	28.8	3.6	49.906	0.0000	210.43	95885000	296	18.20	18.28	18.26	18.45	17.97	18.35	18.21	18.44
0.071	0.05	1.04	Q5SNZ0	Girdin	Ccdc88a	2131	13	13	13	10.2	10.2	10.2	215.92	0.0000	48.173	17747000	91	16.52	16.93	17.55	17.45	16.74	17.12	17.30	17.08
0.070	0.05	1.04	Q69Z38	Pseudopodium-enriched atypical kinase 1	Peak1	2479	18	18	18	14.8	14.8	14.8	191.09	0.0000	36.037	10039000	99	13.92	14.05	14.75	14.71	14.28	14.76	14.10	14.07
0.022	0.05	1.04	Q6URW6	Myosin-14	Myh14	2662	22	10	9	10.4	5.8	5.2	228.58	0.0000	8.8054	10632000	45	14.28	15.96	16.65	16.08	16.08	16.42	16.50	13.76
0.035	0.05	1.04	Q9JKP8	Chromatin accessibility complex protein 1	Chrac1	5457	4	4	4	47.3	47.3	47.3	14.127	0.0000	18.12	4152300	59	13.38	15.16	14.57	14.91	13.56	14.42	15.18	14.64
0.240	0.05	1.04	Q9CZD5	Translation initiation factor IF-3, mitochondrial	Mtif3	4826	4	4	4	18.8	18.8	18.8	31.739	0.0000	16.264	2248600	43	13.35	13.32	13.31	13.46	13.21	13.13	13.44	13.44
0.133	0.05	1.04	O08915	AH receptor-interacting protein	Aip	311	15	15	15	51.8	51.8	51.8	37.605	0.0000	61.257	32254000	273	16.33	16.52	16.34	16.61	16.19	16.60	16.17	16.64
0.028	0.05	1.04	Q9DBS5	Kinesin light chain 4	Klc4	5175	22	18	17	43.3	38.4	37	68.612	0.0000	87.15	16591000	156	13.83	16.25	16.11	16.25	15.69	15.21	15.41	15.93
0.039	0.05	1.04	P52431	DNA polymerase delta catalytic subunit	Pold1	1216	28	28	28	37.4	37.4	37.4	123.79	0.0000	71.693	25988000	134	16.65	15.92	16.30	15.78	16.98	15.88	16.48	15.12
0.030	0.05	1.04	P70671	Interferon regulatory factor 3	Irf3	1582	10	10	10	42.2	42.2	42.2	46.852	0.0000	88.753	8719100	92	13.50	14.91	15.89	15.77	14.48	15.32	15.03	15.04
0.115	0.05	1.03	Q8R3C6	Probable RNA-binding protein 19	Rbm19	3841	7	7	7	11.4	11.4	11.4	106.08	0.0000	18.794	5185500	74	14.49	14.06	14.37	14.29	14.48	14.24	13.88	14.40
0.162	0.05	1.03	P60670	Nuclear protein localization protein 4 homolog	Nploc4	1357	25	25	25	49.8	49.8	49.8	68.016	0.0000	115.63	58369000	373	16.76	16.79	16.64	17.03	16.84	16.66	16.95	16.58
0.084	0.05	1.03	Q6P5D8	Structural maintenance of chromosomes flexible hinge domain-containir	Smchd1	2573	38	38	38	26.7	26.7	26.7	225.65	0.0000	201.55	33288000	323	15.86	15.36	16.08	15.41	15.64	15.95	15.45	15.48
0.071	0.05	1.03	Q9ERA6	Tuftelin-interacting protein 11	Tfip11	5301	17	17	17	22.4	22.4	22.4	96.304	0.0000	124.82	15212000	181	15.21	15.07	15.65	15.68	14.90	15.62	15.18	15.71
0.048	0.05	1.03	P28656	Nucleosome assembly protein 1-like 1	Nap1l1	954	12	10	10	38.1	35.5	35.5	45.345	0.0000	120.88	62303000	311	16.83	17.84	17.21	17.45	17.14	17.99	17.35	16.64
0.070	0.05	1.03	Q3TL44	NLR family member X1	Nlrx1	1858	14	14	14	18.3	18.3	18.3	107.83	0.0000	41.586	8997200	81	14.77	14.48	15.29	14.54	14.65	15.08	14.82	14.35
0.054	0.05	1.03	Q8R4B8	NACHT, LRR and PYD domains-containing protein 3	Nlrp3	3867	6	6	6	6.7	6.7	6.7	118.27	0.0000	29.207	3292200	35	13.76	13.72	14.09	13.72	14.24	14.08	12.97	13.80
0.066	0.05	1.03	Q8K3X4	Interferon regulatory factor 2-binding protein-like	Irf2bpl	3707	6	5	5	11.4	10.5	10.5	80.564	0.0000	20.402	7334000	77	14.89	14.98	15.80	15.62	15.18	15.40	15.44	15.08
0.061	0.04	1.03	P21995	Embigin	Emb	872	4	4	4	16.4	16.4	16.4	37.064	0.0000	7.2849	25578000	115	15.92	16.72	16.91	16.66	16.76	16.24	16.68	16.36
0.087	0.04	1.03	Q3UIR3	E3 ubiquitin-protein ligase DTX3L	Dtx3l	1980	29	29	29	46.9	46.9	46.9	83.043	0.0000	269.61	61296000	559	16.82	16.56	16.58	16.44	16.60	17.00	16.30	16.32
0.063	0.04	1.03	Q9Z0R4;CO	Intersectin-1	Itsn1	5781	12	12	12	11.8	11.8	11.8	194.29	0.0000	31.723	11929000	72	16.31	15.72	15.31	15.86	15.86	16.04	15.43	15.72
0.052	0.04	1.03	O08734	Bcl-2 homologous antagonist/killer	Bak1	290	6	6	6	40.7	40.7	40.7	23.294	0.0000	85.702	11028000	105	15.15	14.96	15.88	15.78	14.95	15.30	15.69	15.67
0.082	0.04	1.03	Q9QWY8	Arf-GAP with SH3 domain, ANK repeat and PH domain-containing prote	Asap1	5530	18	18	18	21.8	21.8	21.8	127.42	0.0000	133.11	32824000	270	16.62	16.55	16.91	16.85	16.30	16.87	16.57	17.03
0.055	0.04	1.03	Q5RJG1	Nucleolar protein 10	Nol10	2126	6	6	6	13.1	13.1	13.1	80.076	0.0000	12.482	5331500	69	14.77	14.30	14.59	14.78	14.09	14.95	14.25	14.99
0.275	0.04	1.03	P51863	V-type proton ATPase subunit d 1	Atp6v0d1	1206	21	21	21	54.7	54.7	54.7	40.301	0.0000	323.31	237140000	690	19.31	19.47	19.27	19.27	19.35	19.19	19.33	19.30
0.029	0.04	1.03	P70188	Kinesin-associated protein 3	Kifap3	1531	6	6	6	9.3	9.3	9.3	91.29	0.0000	45.752	3556200	78	13.48	13.39	15.32	13.50	13.71	13.93	13.89	13.99
0.026	0.04	1.03	P55065	Phospholipid transfer protein	Pltp	1265	9	9	9	26.8	26.8	26.8	54.452	0.0000	86.953	12151000	108	15.95	15.67	15.19	15.25	16.03	15.94	15.90	14.03
0.272	0.04	1.03	P39054;P39	Dynamin-2	Dnm2	1051	52	52	38	55.1	55.1	44	98.144	0.0000	323.31	306160000	1468	18.90	18.86	18.90	18.97	18.72	18.88	18.91	18.97
0.077	0.04	1.03	P25425;P31	POU domain, class 2, transcription factor 1;POU domain, class 2, trans	Pou2f1;Pou2f3;Po	906	3	3	3	6.1	6.1	6.1	79.546	0.0000	38.233	9401900	49	16.47	16.33	16.53	16.03	16.71	16.14	16.13	16.22
0.110	0.04	1.03	Q9ES52	Phosphatidylinositol 3,4,5-trisphosphate 5-phosphatase 1	Inpp5d	5328	69	69	67	56.5	56.5	55.7	133.54	0.0000	323.31	218900000	1316	18.00	17.68	17.87	18.14	17.69	17.87	17.92	18.07
0.139	0.04	1.03	Q9QZS3	Protein numb homolog	Numb	5605	13	13	13	22.5	22.5	22.5	70.812	0.0000	26.875	28461000	213	16.32	16.29	16.33	16.37	16.01	16.47	16.33	16.36
0.038	0.04	1.03	Q6P069	Sorcin	Sri	2551	14	14	14	63.1	63.1	63.1	21.627	0.0000	104.54	58905000	224	17.37	18.53	17.33	17.63	17.36	17.48	17.79	18.10
0.050	0.04	1.03	Q9JM13	Rab5 GDP/GTP exchange factor	Rabgef1	5496	16	16	16	37.7	37.7	37.7	56.868	0.0000	41.393	10707000	105	14.71	14.65	15.24	14.45	14.91	14.99	14.82	14.17
0.107	0.04	1.02	P49717;Q9C	DNA replication licensing factor MCM4	Mcm4	1168	30	30	30	36.2	36.2	36.2	96.735	0.0000	313.38	70365000	566	17.49	17.46	17.08	17.58	17.40	17.39	17.45	17.22
0.024	0.03	1.02	Q9DAV9	Trimeric intracellular cation channel type B	Tmem38b	5113	4	4	4	13.7	13.7	13.7	32.64	0.0000	11.588	7771900	81	14.42	15.74	14.88	16.36	15.95	15.36	15.20	14.75
0.029	0.03	1.02	Q3U214	Microtubule-associated serine/threonine-protein kinase 3	Mast3	1908	4	3	3	4.7	4.1	4.1	144.17	0.0000	9.7976	1159300	29	14.13	12.92	12.32	13.07	12.89	13.41	12.69	13.32
0.021	0.03	1.02	Q8BK08	Transmembrane protein 11, mitochondrial	Tmem11	3092	4	4	4	26.3	26.3	26.3	21.311	0.0000	11.545	3318300	46	13.31	14.09	15.40	12.83	14.11	13.66	13.67	14.05
0.024	0.03	1.02	Q8VCB1	Nucleoporin NDC1	Ndc1	3913	8	8	8	21.2	21.2	21.2	75.409	0.0000	66.266	20963000	120	15.57	15.65	17.35	16.21	15.69	16.62	16.57	15.77
0.099	0.03	1.02	Q8BL66	Early endosome antigen 1	Eea1	3110	65	65	65	51	51	51	160.91	0.0000	323.31	70811000	828	15.85	15.45	15.92	15.94	15.87	15.73	15.76	15.67
0.091	0.03	1.02	Q63829	COMM domain-containing protein 3	Commd3	2404	9	9	9	36.4	36.4	36.4	22.037	0.0000	66.616	16019000	166	16.50	16.39	15.91	16.37	16.31	16.25	16.21	16.27
0.080	0.03	1.02	Q922H4	Mannose-1-phosphate guanyltransferase alpha	Gmppa	4258	17	17	17	41.4	41.4	41.4	46.244	0.0000	53.738	28330000	261	16.51	16.20	16.60	16.62	16.27	16.40	16.36	16.76
0.037	0.03	1.02	Q91ZX7;O8	Prolow-density lipoprotein receptor-related protein 1;Low-density lipopro	Lrp1	4205	111	111	109	25.1	25.1	24.8	504.74	0.0000	323.31	577650000	2984	18.55	18.20	18.43	18.32	19.01	17.83	18.65	17.88
0.084	0.03	1.02	P70295	Ancient ubiquitous protein 1	Aup1	1548	15	15	15	38.5	38.5	38.5	46.121	0.0000	26.29	25524000	187	16.38	16.31	16.33	16.49	15.97	16.46	16.37	16.58
0.036	0.03	1.02	Q8K4B2	Interleukin-1 receptor-associated kinase 3	Irak3	3713	5	5	5	8	8	8	68.455	0.0000	22.225	2150600	34	14.05	13.68	13.08	12.82	13.22	13.70	13.24	13.34
0.113	0.03	1.02	P68040	Guanine nucleotide-binding protein subunit beta-2-like 1;Guanine nucle	Gnb2l1	1513	29	29	29	87.7	87.7	87.7	35.076	0.0000	323.31	602030000	1108	20.16	20.20	19.96	19.90	20.16	19.90	20.13	19.91
0.133	0.03	1.02	O88531	Palmitoyl-protein thioesterase 1	Ppt1	540	12	12	12	37.6	37.6	37.6	34.49	0.0000	285.44	77989000	331	17.96	17.85	18.03	18.09	17.74	18.04	18.01	18.01

0.066	0.03	1.02	Q9JK81	UPF0160 protein MYG1, mitochondrial	Myg1	5445	14	14	14	46.1	46.1	46.1	42.722	0.0000	80.81	66371000	286	17.25	17.27	17.79	17.40	17.23	17.69	17.43	17.25
0.044	0.03	1.02	Q3TJD7	PDZ and LIM domain protein 7	Pdlim7	1853	7	7	7	23.4	23.4	23.4	50.118	0.0000	22.286	6386500	70	14.98	14.26	14.71	14.03	14.23	14.73	14.49	14.41
0.068	0.03	1.02	Q8C1Q6	Small integral membrane protein 4	Smim4	3366	1	1	1	15	15	15	9.7463	0.0018	3.1433	1259900	27	13.29	13.04	13.45	13.40	13.10	13.34	13.05	13.57
0.051	0.03	1.02	Q91VR8	Protein BRICK1	Brk1	4074	10	10	10	96	96	96	8.7608	0.0000	37.598	34739000	127	16.62	17.50	17.17	17.31	16.96	17.17	17.25	17.12
0.070	0.03	1.02	Q9QZD8	Mitochondrial dicarboxylate carrier	Slc25a10	5593	17	17	17	58.9	58.9	58.9	31.715	0.0000	116.3	37578000	179	16.88	16.72	16.89	16.66	17.16	16.67	16.57	16.65
0.040	0.03	1.02	Q3UDP0	WD repeat-containing protein 41	Wdr41	1949	12	12	12	38.3	38.3	38.3	51.51	0.0000	38.701	12123000	112	14.61	15.45	15.64	15.58	15.23	15.28	15.47	15.19
0.091	0.03	1.02	Q9DBJ1	Phosphoglycerate mutase 1	Pgam1	5158	36	36	29	95.7	95.7	89.8	28.832	0.0000	323.31	2507300000	2081	22.54	22.18	22.60	22.40	22.26	22.55	22.34	22.46
0.051	0.03	1.02	O54825	Bystin	Bysl	426	6	6	6	18.8	18.8	18.8	49.783	0.0000	25.026	4590900	93	14.51	14.34	14.88	14.79	14.24	14.61	14.96	14.60
0.016	0.03	1.02	Q5F285	Transmembrane protein 256	Tmem256	2102	3	3	3	37.2	37.2	37.2	11.655	0.0000	6.4037	1375500	11	13.07	14.03	14.30	12.49	12.65	12.81	14.09	14.24
0.041	0.03	1.02	P57784	U2 small nuclear ribonucleoprotein A	Snrpa1	1301	18	18	18	75.7	75.7	75.7	28.357	0.0000	323.31	38891000	280	16.63	16.80	16.37	16.89	16.67	17.15	16.61	16.15
0.062	0.03	1.02	Q3UFK8	FERM domain-containing protein 8	Frmd8	1955	17	17	17	49.4	49.4	49.4	51.827	0.0000	220.42	52414000	383	17.59	17.90	18.08	17.92	17.56	17.79	18.10	17.92
0.015	0.03	1.02	Q8R0F3	Sulfatase-modifying factor 1	Sumf1	3755	3	3	3	12.6	12.6	12.6	40.659	0.0000	8.15	4762200	36	13.48	15.91	15.44	15.19	14.99	14.39	15.13	15.40
0.099	0.03	1.02	P28650	Adenylosuccinate synthetase isozyme 1	Adssl1	953	29	26	26	69.1	62.8	62.8	50.254	0.0000	237.82	125790000	537	17.91	17.82	18.02	18.20	17.83	17.98	18.00	18.04
0.094	0.02	1.02	Q3UE31	Uncharacterized protein KIAA0930 homolog		1952	12	12	12	39.4	39.4	39.4	45.96	0.0000	49.351	16963000	149	15.45	15.50	15.70	15.28	15.41	15.42	15.59	15.40
0.024	0.02	1.02	Q8BMC4	Nucleolar protein 9	Nop9	3133	12	12	12	23.3	23.3	23.3	70.046	0.0000	49.28	12870000	105	15.40	15.36	15.29	15.33	14.70	14.79	15.66	16.13
0.024	0.02	1.02	Q78JE5	F-box only protein 22	Fbxo22	2725	6	6	6	18.9	18.9	18.9	44.202	0.0000	95.559	8552700	118	14.86	14.80	15.41	15.17	14.78	14.34	15.33	15.70
0.011	0.02	1.02	Q9WUB0	RanBP-type and C3HC4-type zinc finger-containing protein 1	Rbck1	5705	2	2	2	5.3	5.3	5.3	57.534	0.0000	6.6537	1964800	36	13.40	12.23	14.79	14.79	13.92	13.78	14.49	12.92
0.076	0.02	1.02	P14483;P01	H-2 class II histocompatibility antigen, A beta chain;H-2 class II histocor	H2-Ab1	781	12	12	11	42.3	42.3	42.3	30.128	0.0000	270.72	113220000	310	18.69	18.61	18.73	18.64	18.94	18.54	18.65	18.46
0.038	0.02	1.01	P62774	Myotrophin	Mtpn	1444	6	6	6	68.6	68.6	68.6	12.861	0.0000	198.68	24789000	233	16.82	17.21	16.75	17.03	16.80	17.34	16.55	17.03
0.019	0.02	1.01	Q9EQ28	DNA polymerase delta subunit 3	Pold3	5270	5	5	4	13.9	13.9	12.8	50.835	0.0000	10.335	1581200	39	13.51	13.13	13.10	13.39	14.06	13.70	12.58	12.71
0.041	0.02	1.01	Q9CQI3	Glia maturation factor beta	Gmfb	4601	12	12	9	64.1	64.1	59.9	16.723	0.0000	84.669	26666000	206	16.18	16.61	15.98	16.32	16.03	16.51	16.07	16.40
0.013	0.02	1.01	Q99L85	Elongator complex protein 5	Elp5	4411	3	3	3	21.7	21.7	21.7	33.497	0.0000	6.725	2567600	32	13.69	13.98	13.60	14.51	14.13	14.09	14.99	12.48
0.059	0.02	1.01	Q9ESU6	Bromodomain-containing protein 4	Brd4	5346	10	9	5	9.9	9	6.2	155.89	0.0000	19.869	5120500	53	13.97	14.18	14.55	14.38	14.25	14.25	14.22	14.26
0.038	0.02	1.01	P21981	Protein-glutamine gamma-glutamyltransferase 2	Tgm2	871	37	37	37	64.1	64.1	64.1	77.06	0.0000	323.31	330560000	1090	19.19	18.93	19.26	19.54	19.10	18.93	19.56	19.25
0.018	0.02	1.01	Q8WTY4	Anamorsin	Ciapin1	4036	5	5	5	27.2	27.2	27.2	33.429	0.0000	12.297	2570200	21	13.34	14.12	13.96	13.59	14.46	13.15	14.19	13.14
0.020	0.02	1.01	A2AWP8	Rho guanine nucleotide exchange factor 10-like protein	Arhgef10l	43	17	17	17	24	24	24	139.99	0.0000	107.79	23184000	192	15.93	15.85	16.03	16.03	16.04	16.38	14.98	16.35
0.048	0.02	1.01	P54276	DNA mismatch repair protein Msh6	Msh6	1250	25	25	24	26.3	26.3	25.4	151.08	0.0000	62.213	61904000	166	18.97	18.53	19.08	18.76	18.64	18.99	18.85	18.79
0.014	0.02	1.01	Q9DBN5	Lon protease homolog 2, peroxisomal	Lonp2	5165	2	2	2	3.6	3.6	3.6	94.525	0.0002	6.1015	1850200	25	13.59	13.28	14.22	13.59	12.65	14.67	13.45	13.82
0.030	0.02	1.01	P70333	Heterogeneous nuclear ribonucleoprotein H2	Hnrnph2	1554	26	25	15	63.9	61.7	44.8	49.279	0.0000	323.31	173240000	604	19.48	19.95	19.35	19.71	19.53	19.38	19.45	20.06
0.024	0.02	1.01	Q99P31	Hsp70-binding protein 1	Hspbp1	4500	8	8	8	38.7	38.7	38.7	39.166	0.0000	65.138	22850000	175	15.50	15.66	16.37	16.26	15.90	15.60	16.13	16.09
0.052	0.02	1.01	P97492	Regulator of G-protein signaling 14	Rgs14	1662	11	11	11	32.2	32.2	32.2	59.846	0.0000	65.746	10090000	131	14.82	15.26	14.83	14.84	14.81	14.91	14.94	15.02
0.034	0.02	1.01	Q9D2Y4	Mixed lineage kinase domain-like protein	Mlkl	4955	11	11	11	25	25	25	54.317	0.0000	38.651	12247000	120	15.37	14.76	15.47	15.20	15.00	15.20	15.25	15.29
0.048	0.02	1.01	Q07113	Cation-independent mannose-6-phosphate receptor	Igf2r	1755	17	17	17	8.3	8.3	8.3	273.81	0.0000	70.869	19213000	202	15.72	15.35	15.80	15.67	15.62	15.62	15.80	15.44
0.032	0.02	1.01	Q9ERN0	Secretory carrier-associated membrane protein 2	Scamp2	5315	6	6	6	18.8	18.8	18.8	36.464	0.0000	196.41	33388000	140	17.31	17.28	17.90	17.80	17.69	17.69	17.37	17.48
0.075	0.02	1.01	Q5XJY5	Coatomer subunit delta	Arcn1	2173	44	44	44	71.2	71.2	71.2	57.229	0.0000	323.31	220050000	1199	17.85	17.66	17.86	17.93	17.79	17.90	17.66	17.89
0.020	0.02	1.01	Q9D4H2	GRIP and coiled-coil domain-containing protein 1	Gcc1	4977	6	6	6	11.1	11.1	11.1	87.676	0.0000	14.75	3238800	15	13.28	13.78	13.19	13.90	13.15	13.89	13.92	13.12
0.033	0.02	1.01	Q9CPX7	28S ribosomal protein S16, mitochondrial	Mrps16	4542	4	4	4	43.7	43.7	43.7	15.192	0.0000	30.657	6551600	73	14.45	14.34	15.07	14.65	14.70	14.51	14.71	14.52
0.022	0.02	1.01	Q9D020	Cytosolic 5-nucleotidase 3A	Nt5c3a	4849	9	9	9	45	45	45	37.252	0.0000	110.7	21935000	121	16.87	17.06	17.54	16.78	17.20	17.30	17.17	16.52
0.027	0.01	1.01	Q62191	E3 ubiquitin-protein ligase TRIM21	Trim21	2366	10	10	10	23	23	23	54.174	0.0000	55.282	8545700	108	14.77	15.18	15.55	15.04	15.12	15.32	15.15	14.89
0.027	0.01	1.01	P35278	Ras-related protein Rab-5C	Rab5c	1013	17	17	12	75	75	55.6	23.412	0.0000	259.78	245490000	726	19.24	18.84	18.60	19.12	18.96	18.61	19.13	19.03
0.010	0.01	1.01	Q91VS7	Microsomal glutathione S-transferase 1	Mgst1	4075	10	10	10	45.2	45.2	45.2	17.551	0.0000	117.97	51849000	384	16.71	16.48	16.97	16.48	16.00	15.63	17.64	17.30
0.029	0.01	1.01	Q99JY4	TraB domain-containing protein	Trabd	4357	6	6	6	28.2	28.2	28.2	42.189	0.0000	72.763	15420000	100	16.05	15.75	16.44	16.17	15.92	16.11	16.30	16.02
0.039	0.01	1.01	O89116	Vesicle transport through interaction with t-SNAREs homolog 1A	Vti1a	607	4	4	4	39.2	39.2	39.2	24.986	0.0000	99.06	10923000	124	15.53	15.26	15.68	15.45	15.47	15.59	15.54	15.27
0.087	0.01	1.01	Q921M3	Splicing factor 3B subunit 3	Sf3b3	4232	40	40	40	42.3	42.3	42.3	135.55	0.0000	271.78	95076000	523	17.19	17.14	17.05	17.09	17.21	17.06	17.02	17.14
0.014	0.01	1.01	Q5MJS3;Q8	Extracellular serine/threonine protein kinase FAM20C;Protein FAM20A	Fam20c;Fam20a	2114	2	2	2	6.9	6.9	6.9	65.765	0.0000	6.8122	920770	8	13.13	13.02	13.20	13.67	13.26	12.89	12.90	13.92
0.005	0.01	1.01	Q9WUB4	Dynactin subunit 6	Dctn6	5707	2	2	2	14.7	14.7	14.7	20.67	0.0000	24.661	5600600	67	14.62	15.11	13.85	14.88	15.06	15.25	15.50	12.61
0.013	0.01	1.01	E1U8D0	Protein SOGA1;N-terminal form;C-terminal 80 kDa form	Soga1	217	13	13	13	13.8	13.8	13.8	159.18	0.0000	85.544	5020500	63	13.88	13.68	13.49	14.25	13.81	13.33	14.42	13.69
0.016	0.01	1.01	Q6P1H6	Ankyrin repeat and LEM domain-containing protein 2	Ankle2	2555	13	13	13	22.2	22.2	22.2	106.2	0.0000	97.223	12144000	152	14.60	14.33	14.51	14.78	14.31	15.07	14.63	14.17
0.031	0.01	1.01	O08605	MAP kinase-interacting serine/threonine-protein kinase 1	Mknk1	281	8	8	8	26.2	26.2	26.2	47.914	0.0000	204.46	11815000	132	14.92	15.08	15.23	15.20	14.86	15.13	15.11	15.30
0.026	0.01	1.01	Q7JJ13	Bromodomain-containing protein 2	Brd2	2736	10	10	9	19	19	17.4	88.066	0.0000	31.916	11733000	91	15.84	15.87	15.90	16.18	15.60	16.04	16.07	16.02
0.007	0.01	1.01	Q9EQ06;Q8	Estradiol 17-beta-dehydrogenase 11	Hsd17b11	5268	14	14	14	55	55	55	32.88	0.0000	239.59	72657000	372	18.19	17.70	17.35	16.14	17.79	16.75	17.64	17.16
0.010	0.01	1.01	Q9WV02	RNA-binding motif protein, X chromosome;RNA-binding motif protein, X	Rbmx	5727	20	3	3	39.1	7.2	7.2	42.3	0.0024	3.0055	771250	7	12.78	13.27	12.09	12.46	12.83	13.14	12.32	12.26
0.016	0.01	1.01	Q80UU9	Membrane-associated progesterone receptor component 2	Pgrmc2	2839	7	7	7	38.7	38.7	38.7	23.334	0.0000	68.638	13147000	114	15.32	15.43	15.97	15.89	15.66	15.39	15.59	15.92
0.040	0.01	1.01	P80316	T-complex protein 1 subunit epsilon	Cct5	1593	59	59	59	85.8	85.8	85.8	59.623	0.0000	323.31	559260000	2012	19.44	19.34	19.37	19.56	19.42	19.24	19.48	19.53
0.004	0.01	1.01	Q9WVS8	Mitogen-activated protein kinase 7	Mapk7	5762	5	5	4	9.7	9.7	7.3	87.732	0.0000	6.5383	4219200	12	14.20	12.44	14.46	15.49	14.54	13.51	14.58	13.91
0.038	0.01	1.01	Q6PGG2	GEM-interacting protein	Gmip	2636	34	34	34	40.2	40.2	40.2	107.54	0.0000	266.87	82442000	797	16.65	16.59	16.89	16.74	16.62	16.74	16.73	16.75
0.010	0.01	1.01	P70302	Stromal interaction molecule 1	Stim1	1550	22	22	22	31.8	31.8	31.8	77.566	0.0000	130.97	24575000	269	15.43	15.46	15.59	15.75	15.03	15.25	15.84	16.07
0.016	0.01	1.00	Q8R151	NFX1-type zinc finger-containing protein 1	Znfx1	3778	43	43	43	25.8	25.8	25.8	218.83	0.0000	305.67	37743000	424	15.48	15.11	15.25	15.10	15.45	15.06	15.38	15.03
0.015	0.01	1.00	O88736	3-keto-steroid reductase	Hsd17b7	563	4	4	4	21.3	21.3	21.3	37.316	0.0000	64.009	6168500	65	15.22	15.15	15.69	15.67	15.38	15.35	15.38	15.58
0.021	0.01	1.00	Q3TKT4	Transcription activator BRG1	Smarca4	1856	37	37	27	24.4	24.4	18.5	181.43	0.0000	213.53	82767000	711	18.07	17.66	17.76	17.67	17.67	17.83	17.75	17.89
0.007	0.01	1.00	Q65Z40	Wings apart-like protein homolog	Wapal	2466	15	15	15	19.1	19.1	19.1	134.07	0.0000	35.873	15218000	100	15.53	15.91	16.15	16.05	15.07	16.28	16.07	16.18
0.019	0.01	1.00	Q6P1B1	Xaa-Pro aminopeptidase 1	Xpnpep1	2552	30	30	30	64.5	64.5	64.5	69.59	0.0000	242.41	102480000	517	17.81	17.80	18.12	18.09	17.80	18.12	17.94	17.94
0.015	0.01	1.00	Q8BJ48	N-acetylglucosamine-1-phosphodiester alpha-N-acetylglucosaminidase	Nagpa	3071	3	3	3	9.3	9.3	9.3	56.043	0.0000	19.103	3401100	18	14.33	14.31	14.57	13.92	14.26	14.33	14.36	14.16
0.009	0.01	1.00	Q61107	Guanylate-binding protein 4	Gbp4	2253	16	16	16	32.6	32.6	32.6	70.801	0.0000	74.575	13231000	127	15.93	14.97	15.29	15.12	15.59	15.40	15.20	15.10
0.020	0.01	1.00	Q9JKF1	Ras GTPase-activating-like protein IQGAP1	Iqgap1	5450	139	139	135	74.6	74.6	73	188.74	0.0000	323.31	2371900000	5676	20.45	20.24	20.56	20.59	20.27	20.48	20.55	20.52
0.007	0.01	1.00	Q8BMF4	Dihydrolipoyllysine-residue acetyltransferase component of pyruvate de	Dlat	3135	18	18	18	31.6	31.6	31.6	67.941	0.0000	142.01	87816000	401	17.95	17.76	18.86	18.38	17.92	18.24	18.43	18.34
0.017	0.01	1.00	Q8K0Q5	Rho GTPase-activating protein 18	Arhgap18	3609	10	10	10	17.5	17.5	17.5	74.929	0.0000	33.145	13694000	140	15.29	15.36	15.64	15.63	15.29	15.56	15.43	15.62
0.007	0.01	1.00	Q9CT10	Ran-binding protein 3	Ranbp3	4702	8	8	8	22.6	22.6	22.6	52.572	0.0000	42.038	8292700	103	14.84	14.64	15.31	15.09	14.44	15.38	15.18	14.86
0.022	0.01	1.00	Q6PB93	Polypeptide N-acetylgalactosaminyltransferase 2;Polypeptide N-acetylg	Galnt2	2603	15	15	15	33.2	33.2	33.2	64.514	0.0000	53.682	20843000	162	15.41	15.58	15.50	15.49	15.52	15.51	15.63	15.29
0.009	0.01	1.00	Q62087	Serum paraoxonase/lactonase 3	Pon3	2354	13	13	13	52	52	52	39.351	0.0000	224.9	110830000	406	18.44	18.50	19.12	18.76	18.51	18.66	18.61	19.02
0.010	0.00	1.00	Q9D1I2	Bcl10-interacting CARD protein		4917	11	11	11	63.9	63.9	63.9	20.936	0.0000	21.15	11018000	92	16.49	15.93	16.36	16.05	16.03	16.46	16.13	16.18
0.004	0.00	1.00	P09055	Integrin beta-1	Itgb1	692	16	16	16	20.2	20.2	20.2	88.231	0.0000	295.31	75682000	352	16.97	17.36	18.30	17.85	17.55	17.59	17.80	17.53
0.003	0.00	1.00	Q8VDC1	FYVE and coiled-coil domain-containing protein 1	Fyco1	3947	5	5	4	5.2	5.2	4.5	162.33	0.0000	38.132	2045400	37	13.41	13.62	14.69	13.69	13.41	14.05	13.98	13.96
0.005	0.00	1.00	P48722	Heat shock 70 kDa protein 4L	Hspa4l	1148	32	28	28	45.9	43	43	94.381	0.0000	109.26	29178000	257	15.52	15.34	15.98	16.09	15.58	15.48	15.85	16.01
0.007	0.00	1.00	Q91ZW3	SWI/SNF-related matrix-associated actin-dependent regulator of chroma	Smarca5	4204	41	41	30	39	39	29.1	121.63	0.0000	162.98	77487000	623	16.59	16.39	16.45	16.69	16.40	16.72	16.38	16.61
0.002	0.00	1.00	Q9CQE1	Protein NipSnap homolog 3B	Nipsnap3b	4587	13	13	13	55.9	55.9	55.9	28.308	0.0000	60.537	51558000	186	16.52	17.32	17.78	18.12	17.33	17.22	17.00	18.18
0.001	0.00	1.00	O08585	Clathrin light chain A	Clta	277	5	5	5	20.4	20.4	20.4	25.604	0.0000	18.104	3588300	43	14.17	14.82	13.54	14.30	13.94	14.24	13.72	14.91
0.001	0.00	1.00	Q60866	Phosphotriesterase-related protein	Pter	2216	6	6	6	25.5	25.5	25.5	39.218	0.0000	63.439	7926500	87	15.27	15.25	15.63	15.35	15.36	15.65	15.22	15.26
0.000	0.00	1.00	Q99KR3	Beta-lactamase-like protein 2	Lactb2	4390	13	13	13	48.3	48.3	48.3	32.754	0.0000	45.509	41060000	311	16.90	17.05	16.88	17.00	16.90	17.12	16.68	17.13
0.001	0.00	1.00	P62874;Q61	Guanine nucleotide-binding protein G(I)/G(S)/G(T) subunit beta-1	Gnb1	1459	20	20	12	65.3	65.3	44.1	37.377	0.0000	323.31	339700000	911	19.52	19.29	19.37	19.51	19.57	19.42	19.34	19.37
0.001	0.00	1.00	Q9D819	Inorganic pyrophosphatase	Ppa1	5046	11	11	11	58.5	58.5	58.5	32.667	0.0000	35.713	17532000	95	16.07	15.90	15.71	16.12	16.24	15.85	15.77	15.95
0.003	0.00	1.00	Q9JL26	Formin-like protein 1	Fmnl1	5470	59	59	54	45.6	45.6	44.6	122.06	0.0000	323.31	187780000	1151	18.30	18.04	18.26	18.33	18.14	18.61	18.08	18.11
0.004	0.00	1.00	P58389	Serine/threonine-protein phosphatase 2A activator	Ppp2r4	1314	16	16	16	68.4	68.4	68.4	36.71	0.0000	323.31	63609000	388	16.72	17.24	17.28	17.31	17.25	17.03	17.12	17.16
0.004	0.00	1.00	P61222	ATP-binding cassette sub-family E member 1	Abce1	1379	37	37	37	64.8	64.8	64.8	67.314	0.0000	323.31	177700000	834	17.72	17.58	17.32	17.58	18.00	17.42	17.68	17.11
0.004	0.00	1.00	Q9CPQ3	Mitochondrial import receptor subunit TOM22 homolog	Tomm22	4526	6	6	6	50	50	50	15.537	0.0000	104.83	75904000	201	18.65	18.17	18.99	18.71	18.21	18.67	18.90	18.76
0.003	0.00	1.00	O89086	RNA-binding protein 3	Rbm3	601	7	7	7	51	51	51	16.604	0.0000	31.93	15096000	102	15.81	16.76	15.03	14.62	15.51	15.86	15.12	15.75
0.013	0.00	1.00	Q99JT1	Glutamyl-tRNA(Gln) amidotransferase subunit B, mitochondrial	Gatb	4347	5	5	5	15.1	15.1	15.1	62.118	0.0000	17.455	10727000	64	16.19	16.25	16.62	16.43	16.30	16.40	16.40	16.41
0.010	0.00	1.00	Q8VCH8	UBX domain-containing protein 4	Ubxn4	3922	9	9	9	23.3	23.3	23.3	56.471	0.0000	24.615	18473000	100	16.68	16.48	16.86	16.67	16.40	16.65	16.69	16.96
0.009	0.00	1.00	A2AIV8	Caspase recruitment domain-containing protein 9	Card9	26	12	12	12	32.6	32.6	32.6	62.461	0.0000	77.716	19510000	144	16.05	15.99	16.61	16.47	16.20	16.52	16.19	16.23
0.004	0.00	1.00	Q8R5F3	O-acetyl-ADP-ribose deacetylase 1	Oard1	3886	3	3	3	22.4	22.4	22.4	17.107	0.0000	16.903	3007700	29	14.41	14.10	14.71	14.42	14.57	13.44	14.54	15.11
0.009	-0.01	1.00	Q3TBD2	Minor histocompatibility protein HA-1	Hmha1	1819	57	57	57	47.2	47.2	47.2	122.9	0.0000	323.31	227150000	1475	17.77	17.41	17.87	17.67	17.78	18.14	17.37	17.45
0.011	-0.01	1.00	Q9CQ80	Vacuolar protein-sorting-associated protein 25	Vps25	4572	13	13	13	77.3	77.3	77.3	20.747	0.0000	226.5	19544000	199	15.76	15.72	15.45	16.12	16.02	15.46	15.79	15.79
0.009	-0.01	1.00	Q9JLZ3	Methylglutaconyl-CoA hydratase, mitochondrial	Auh	5494	6	6	6	20.4	20.4	20.4	33.395	0.0000	44.154	3485300	41	14.12	13.93	14.47	14.08	13.87	13.97	14.14	14.64
0.014	-0.01	1.00	P13020	Gelsolin	Gsn	763	62	62	24	75.3	75.3	34.5	85.941	0.0000	323.31	1259400000	2044	21.15	21.47	20.83	21.33	21.19	21.16	21.17	21.28
0.015	-0.01	1.00	Q569Z6	Thyroid hormone receptor-associated protein 3	Thrap3	2087	21	21	21	22.8	22.8	22.8	108.18	0.0000	53.307	44661000	309	16.96	16.55	16.75	16.79	16.75	17.01	16.46	16.86
0.008	-0.01	1.00	O88379	Bromodomain adjacent to zinc finger domain protein 1A	Baz1a	525	6	6	6	6.1	6.1	6.1	178.46	0.0000	18.022	3455600	43	14.78	14.42	14.18	14.21	15.12	14.25	14.09	14.15
0.005	-0.01	1.00	Q8BKX6	Serine/threonine-protein kinase SMG1	Smg1	3108	15	15	15	6.9	6.9	6.9	409.76	0.0000	40.918	6297900	43	13.99	13.54	14.85	14.43	13.77	13.99	13.95	15.14
0.013	-0.01	0.99	Q8BRT1	CLIP-associating protein 2	Clasp2	3184	30	30	28	29	29	26.7	140.74	0.0000	246.69	42683000	464	15.78	15.32	16.09	15.74	15.44	15.72	16.03	15.77
0.009	-0.01	0.99	Q8K2X3	CST complex subunit STN1	Obfc1	3677	5	5	5	21.4	21.4	21.4	43.485	0.0000	9.0947	2596500	31	13.63	13.72	14.50	13.17	13.76	13.67	13.94	13.68
0.018	-0.01	0.99	Q7TT37	Elongator complex protein 1	Ikbkap	2792	15	15	15	17.3	17.3	17.3	149.58	0.0000	67.699	14021000	145	14.94	14.79	14.37	15.06	14.80	14.70	15.03	14.67
0.007	-0.01	0.99	Q3TMX7	Sulfhydryl oxidase 2	Qsox2	1863	7	7	7	15.3	15.3	15.3	77.774	0.0000	40.503	2543000	22	13.15	12.70	12.72	13.01	13.21	11.76	13.07	13.57
0.011	-0.01	0.99	Q8BJL0	SWI/SNF-related matrix-associated actin-dependent regulator of chroma	Smarcal1	3079	4	4	4	6.7	6.7	6.7	100.84	0.0002	5.7276	2768000	46	14.64	14.78	13.93	13.70	14.36	14.09	14.44	14.20
0.018	-0.01	0.99	Q9D2G2	Dihydrolipoyllysine-residue succinyltransferase component of 2-oxoglut	Dlst	4942	17	17	17	37.4	37.4	37.4	48.994	0.0000	173.5	74936000	326	17.60	17.81	18.37	18.09	17.92	17.90	18.17	17.92
0.011	-0.01	0.99	Q8C156	Condensin complex subunit 2	Ncaph	3353	7	7	7	13.3	13.3	13.3	82.302	0.0000	24.701	3802200	75	13.74	13.79	14.06	14.11	14.53	13.84	13.21	14.17
0.073	-0.01	0.99	Q91W50	Cold shock domain-containing protein E1	Csde1	4091	27	27	27	32.6	32.6	32.6	88.79	0.0000	154.15	59729000	504	16.50	16.38	16.51	16.58	16.56	16.46	16.47	16.51
0.020	-0.01	0.99	Q9CPQ1	Cytochrome c oxidase subunit 6C	Cox6c	4525	10	10	10	64.5	64.5	64.5	8.4689	0.0000	89.107	250040000	468	19.96	19.43	20.05	20.10	19.62	20.10	19.99	19.86
0.015	-0.01	0.99	E9Q634	Unconventional myosin-Ie	Myo1e	241	53	44	44	47.2	41.6	41.6	126.82	0.0000	313.77	78842000	573	16.95	16.57	16.47	16.56	16.47	17.35	16.34	16.44
0.027	-0.01	0.99	Q9EP97	Sentrin-specific protease 3	Senp3	5248	5	5	5	10.4	10.4	10.4	64.402	0.0000	15.882	4810800	73	14.91	14.45	14.74	14.82	14.55	14.70	14.62	15.10
0.030	-0.01	0.99	Q8C0J6	Ankyrin repeat domain-containing protein SOWAHC	Sowahc	3339	8	8	8	21.9	21.9	21.9	54.937	0.0000	39.159	10195000	160	14.72	14.35	14.72	14.73	14.40	14.54	14.75	14.89
0.016	-0.01	0.99	O88384	Vesicle transport through interaction with t-SNAREs homolog 1B	Vti1b	526	14	14	14	56	56	56	26.713	0.0000	166.75	46969000	272	16.66	16.23	17.28	17.19	16.51	17.08	17.01	16.81
0.025	-0.01	0.99	O88811	Signal transducing adapter molecule 2	Stam2	567	6	6	4	16.8	16.8	13.2	57.454	0.0000	43.857	13055000	72	16.53	16.18	15.92	16.60	16.17	16.41	16.21	16.49
0.051	-0.01	0.99	Q9JM76	Actin-related protein 2/3 complex subunit 3	Arpc3	5499	19	19	19	74.7	74.7	74.7	20.524	0.0000	245.03	253680000	649	19.93	19.91	19.69	20.10	20.04	19.95	19.79	19.91
0.024	-0.01	0.99	P63024;Q62	Vesicle-associated membrane protein 3	Vamp3	1480	8	8	3	67	67	32	11.48	0.0000	197.3	64606000	349	17.96	17.91	18.52	18.54	18.11	18.56	18.30	18.01
0.041	-0.02	0.99	Q8BP47	Asparagine--tRNA ligase, cytoplasmic	Nars	3158	33	33	33	54.9	54.9	54.9	64.279	0.0000	323.31	238080000	814	18.57	18.41	18.84	18.81	18.54	18.87	18.55	18.73

0.012	-0.02	0.99	P58802	TBC1 domain family member 10A	Tbc1d10a	1324	5	5	5	13.4	13.4	13.4	56.202	0.0000	13.766	3416100	46	14.18	13.90	13.25	14.09	12.92	14.78	14.17	13.61
0.045	-0.02	0.99	Q8R5A6	TBC1 domain family member 22A	Tbc1d22a	3883	15	15	15	37.6	37.6	37.6	59.362	0.0000	98.294	20132000	168	16.00	15.91	16.17	15.97	15.72	16.14	16.03	16.21
0.030	-0.02	0.99	Q9DCM0	Persulfide dioxygenase ETHE1, mitochondrial	Ethe1	5222	13	13	13	65.7	65.7	65.7	27.738	0.0000	84.124	31866000	280	15.96	16.07	15.69	16.27	15.91	15.80	15.95	16.39
0.013	-0.02	0.99	Q8VBV3	Exosome complex component RRP4	Exosc2	3897	6	6	6	24.6	24.6	24.6	32.631	0.0000	25.784	5589900	59	13.71	14.52	15.07	14.10	14.86	14.34	14.74	13.52
0.039	-0.02	0.99	P57746	V-type proton ATPase subunit D	Atp6v1d	1297	17	17	17	68	68	68	28.369	0.0000	323.31	88498000	404	18.21	17.95	18.44	18.11	18.33	18.07	18.41	17.98
0.023	-0.02	0.99	P17563;Q63	Selenium-binding protein 1;Selenium-binding protein 2	Selenbp1;Selenbp	826	18	18	18	54.9	54.9	54.9	52.513	0.0000	128.85	25034000	225	16.18	16.21	16.26	15.30	16.02	16.26	15.96	15.76
0.044	-0.02	0.99	Q60930	Voltage-dependent anion-selective channel protein 2	Vdac2	2222	24	24	24	78.3	78.3	78.3	31.732	0.0000	323.31	581200000	1185	20.27	20.03	20.52	20.34	20.18	20.17	20.31	20.56
0.048	-0.02	0.99	Q9CZE3	Ras-related protein Rab-32	Rab32	4827	13	13	11	61	61	52.5	25.068	0.0000	323.31	113440000	488	18.15	18.07	18.14	18.59	18.36	18.27	18.16	18.22
0.034	-0.02	0.99	P01901;P14	H-2 class I histocompatibility antigen, K-B alpha chain	H2-K1	622	17	15	5	42.5	39.8	15.2	41.301	0.0000	88.172	472210000	349	22.26	22.44	22.12	22.03	22.61	22.33	22.00	21.98
0.031	-0.02	0.99	Q8CII9	Cytokine receptor-like factor 2	Crlf2	3556	2	2	2	7.5	7.5	7.5	37.762	0.0004	5.0794	1223400	13	13.42	13.34	13.98	13.39	13.51	13.35	13.93	13.42
0.034	-0.02	0.99	Q9D7N9	Adipocyte plasma membrane-associated protein	Apmap	5037	13	13	13	42.2	42.2	42.2	46.434	0.0000	191.83	37324000	325	16.01	16.41	16.73	16.75	16.51	16.50	16.61	16.35
0.049	-0.02	0.99	P47968	Ribose-5-phosphate isomerase	Rpia	1137	10	10	10	45.2	45.2	45.2	32.45	0.0000	74.886	14813000	144	15.16	15.21	15.51	15.39	15.33	15.12	15.60	15.29
0.044	-0.02	0.98	P97821	Dipeptidyl peptidase 1;Dipeptidyl peptidase 1 exclusion domain chain;D	Ctsc	1677	8	8	8	16.7	16.7	16.7	52.376	0.0000	155.59	47981000	235	16.99	17.11	17.44	17.55	17.30	17.48	16.96	17.44
0.029	-0.02	0.98	O88967	ATP-dependent zinc metalloprotease YME1L1	Yme1l1	589	20	20	20	33.6	33.6	33.6	80.027	0.0000	229.91	39202000	168	17.39	16.85	17.83	17.06	16.82	17.45	17.55	17.40
0.057	-0.02	0.98	Q8BI72	CDKN2A-interacting protein	Cdkn2aip	3054	14	14	14	33.6	33.6	33.6	59.744	0.0000	35.813	17811000	162	15.70	15.40	15.78	15.63	15.33	15.86	15.65	15.77
0.090	-0.02	0.98	Q8BMJ2	Leucine--tRNA ligase, cytoplasmic	Lars	3140	66	66	66	57.7	57.7	57.7	134.19	0.0000	323.31	175430000	1161	17.34	17.32	17.62	17.49	17.42	17.65	17.35	17.44
0.041	-0.02	0.98	Q9QZ08	N-acetyl-D-glucosamine kinase	Nagk	5584	16	16	16	48.4	48.4	48.4	37.268	0.0000	63.926	37123000	324	17.17	16.50	16.37	16.63	16.48	16.76	16.57	16.96
0.060	-0.02	0.98	P47740	Fatty aldehyde dehydrogenase	Aldh3a2	1118	19	19	18	46.7	46.7	44.2	53.97	0.0000	323.31	62865000	488	16.92	16.66	17.15	17.18	16.88	16.91	16.98	17.25
0.026	-0.03	0.98	Q8VEJ4	Notchless protein homolog 1	Nle1	4008	6	6	6	16.9	16.9	16.9	53.133	0.0000	36.132	4005900	67	13.89	14.69	14.67	14.66	14.64	15.16	13.76	14.46
0.044	-0.03	0.98	Q91ZN5	Adenosine 3-phospho 5-phosphosulfate transporter 1	Slc35b2	4197	6	6	6	16.9	16.9	16.9	47.369	0.0000	21.026	5004000	36	14.20	14.43	14.26	14.10	13.97	14.01	14.28	14.85
0.065	-0.03	0.98	P28063	Proteasome subunit beta type-8	Psmb8	945	20	20	20	67.8	67.8	67.8	30.26	0.0000	236.09	122190000	504	18.39	18.26	18.01	18.53	18.49	18.44	18.05	18.32
0.046	-0.03	0.98	Q8BFW7	Lipoma-preferred partner homolog	Lpp	2951	9	9	9	24.3	24.3	24.3	65.89	0.0000	106.76	15941000	172	15.33	15.97	15.71	15.15	15.39	15.49	15.79	15.61
0.022	-0.03	0.98	P58501	PAX3- and PAX7-binding protein 1	Paxbp1	1318	5	5	5	8.8	8.8	8.8	104.83	0.0000	30.922	2410600	22	13.79	14.02	13.33	13.50	12.96	14.42	14.32	13.05
0.049	-0.03	0.98	Q99LB6	Methionine adenosyltransferase 2 subunit beta	Mat2b	4414	12	12	12	44	44	44	37.392	0.0000	79.441	23292000	162	16.42	15.73	16.40	16.24	16.30	16.48	15.93	16.20
0.073	-0.03	0.98	Q9R0G7;Q6	Zinc finger E-box-binding homeobox 2	Zeb2	5625	14	14	14	16.9	16.9	16.9	136.61	0.0000	89.856	13854000	200	15.31	15.00	15.13	15.30	14.96	15.43	15.07	15.40
0.042	-0.03	0.98	Q9EQC5	N-terminal kinase-like protein	Scyl1	5275	19	19	19	34.9	34.9	34.9	89.159	0.0000	88.18	45884000	213	17.17	17.00	17.19	17.28	16.59	17.69	17.11	17.37
0.108	-0.03	0.98	P00493	Hypoxanthine-guanine phosphoribosyltransferase	Hprt1	612	20	20	20	71.1	71.1	71.1	24.57	0.0000	246.82	97595000	719	17.92	17.97	17.71	17.89	18.05	17.71	17.82	18.03
0.047	-0.03	0.98	Q8CCB4	Vacuolar protein sorting-associated protein 53 homolog	Vps53	3445	16	16	16	24.8	24.8	24.8	94.422	0.0000	65.235	18697000	96	16.26	15.81	16.57	16.13	15.83	16.15	16.51	16.41
0.033	-0.03	0.98	Q91YN0	Protein C12orf4 homolog	D6Wsu163e	4167	8	8	8	23.7	23.7	23.7	63.643	0.0000	25.24	2463800	26	13.74	13.45	13.53	14.37	13.76	13.55	13.43	14.47
0.041	-0.03	0.98	O88522	NF-kappa-B essential modulator	Ikbkg	539	4	4	4	13.8	13.8	13.8	47.972	0.0000	9.6973	7457500	18	15.07	15.64	15.39	15.47	15.24	15.53	14.91	16.01
0.039	-0.03	0.98	Q9D0J4	ADP-ribosylation factor-like protein 2	Arl2	4870	11	11	11	71.7	71.7	71.7	20.864	0.0000	63.544	20119000	191	15.97	16.20	15.79	15.53	16.38	15.87	16.09	15.28
0.059	-0.03	0.98	Q9DBP5	UMP-CMP kinase	Cmpk1	5167	20	20	20	76.5	76.5	76.5	22.165	0.0000	323.31	178060000	715	18.01	18.28	17.69	17.90	17.84	17.72	18.38	18.07
0.061	-0.03	0.98	P35282	Ras-related protein Rab-21	Rab21	1015	14	14	14	56.3	56.3	56.3	24.106	0.0000	107.42	74175000	361	17.80	18.10	18.13	17.54	17.93	17.62	17.93	18.21
0.019	-0.03	0.98	Q91VX9	Transmembrane protein 168	Tmem168	4084	3	3	3	4.7	4.7	4.7	79.673	0.0000	9.5737	4807300	37	15.04	14.21	14.85	12.72	14.65	14.29	13.54	14.48
0.065	-0.03	0.98	Q6NV83	U2 snRNP-associated SURP motif-containing protein	U2surp	2530	20	20	19	26.6	26.6	25.7	118.26	0.0000	50.1	21338000	226	15.58	15.42	15.15	15.23	15.19	15.06	15.52	15.73
0.010	-0.03	0.98	G5E8P1		Brd1	265	5	5	5	5.8	5.8	5.8	119.71	0.0002	6.1244	5029700	7	15.54	11.59	15.36	15.51	15.32	12.88	15.49	14.45
0.078	-0.03	0.98	Q9QY06	Unconventional myosin-IXb	Myo9b	5558	56	56	54	33.5	33.5	33.1	238.83	0.0000	261.52	74847000	651	16.35	16.00	16.51	16.46	16.11	16.38	16.34	16.63
0.084	-0.04	0.98	P62301	40S ribosomal protein S13	Rps13	1416	21	21	21	72.2	72.2	72.2	17.222	0.0000	128.97	479710000	875	19.94	19.47	19.95	19.99	19.72	19.74	20.13	19.91
0.090	-0.04	0.97	O35295	Transcriptional activator protein Pur-beta	Purb	353	17	17	13	70.7	70.7	61.4	33.901	0.0000	301.33	62329000	446	17.78	17.55	17.51	17.86	17.45	17.92	17.55	17.91
0.040	-0.04	0.97	Q9D855	Cytochrome b-c1 complex subunit 7	Uqcrb	5050	13	13	13	76.6	76.6	76.6	13.527	0.0000	118.26	30042000	182	15.48	16.54	15.64	16.43	15.69	16.12	15.94	16.49
0.060	-0.04	0.97	Q80XI3	Eukaryotic translation initiation factor 4 gamma 3	Eif4g3	2881	18	14	14	12.5	9.6	9.6	174.89	0.0000	29.293	16765000	181	15.67	15.48	15.61	15.80	15.06	15.93	15.79	15.92
0.041	-0.04	0.97	Q99PG2	Opioid growth factor receptor	Ogfr	4505	8	8	8	15	15	15	70.678	0.0000	24.598	10245000	75	15.90	15.55	16.08	15.59	15.22	16.30	15.44	16.30
0.072	-0.04	0.97	Q8BZT9	Laccase domain-containing protein 1	Lacc1	3319	6	6	6	23.5	23.5	23.5	47.514	0.0000	155.97	9112100	72	15.65	15.56	15.92	16.00	15.77	16.28	15.63	15.61
0.022	-0.04	0.97	Q8K2H2	OTU domain-containing protein 6B	Otud6b	3659	8	8	8	32	32	32	33.758	0.0000	45.768	3704400	25	12.77	12.90	12.62	12.64	12.12	14.16	11.58	13.21
0.050	-0.04	0.97	P22907	Porphobilinogen deaminase	Hmbs	878	13	13	13	45.2	45.2	45.2	39.344	0.0000	185.73	28841000	253	16.77	16.57	17.24	16.56	17.16	17.21	16.41	16.52
0.037	-0.04	0.97	Q7TND5	Ribosome production factor 1	Rpf1	2760	2	2	2	11.5	11.5	11.5	40.036	0.0000	11.602	860570	26	13.24	13.26	12.73	12.73	13.99	12.84	12.60	12.68
0.029	-0.04	0.97	Q6PGG6	Guanine nucleotide-binding protein-like 3-like protein	Gnl3l	2637	3	3	3	9	9	9	65.194	0.0000	10.726	3282800	15	14.27	14.17	13.83	14.50	14.75	14.67	14.63	12.89
0.057	-0.04	0.97	Q9QXL1;Q9	Kinesin-like protein KIF21B	Kif21b	5545	11	11	11	10.3	10.3	10.3	186.18	0.0000	13.521	8813200	44	15.22	15.49	16.20	15.65	15.52	15.90	15.38	15.93
0.060	-0.04	0.97	Q9QZW0	Phospholipid-transporting ATPase 11C	Atp11c	5609	9	9	9	12.7	12.7	12.7	129.24	0.0000	25.015	4090600	45	14.06	13.74	14.23	14.22	14.16	13.61	14.65	13.99
0.042	-0.04	0.97	Q8VHK9	ATP-dependent RNA helicase DHX36	Dhx36	4021	11	11	11	20.5	20.5	20.5	113.88	0.0000	38.594	6679800	103	15.03	14.65	13.73	14.71	14.81	13.95	14.65	14.88
0.083	-0.04	0.97	Q9WTP6	Adenylate kinase 2, mitochondrial;Adenylate kinase 2, mitochondrial, N-	Ak2	5681	17	17	17	72	72	72	26.468	0.0000	101.09	44029000	314	16.45	16.51	16.23	16.36	15.93	16.62	16.47	16.70
0.156	-0.04	0.97	P62962	Profilin-1	Pfn1	1473	21	21	11	97.1	97.1	66.4	14.957	0.0000	323.31	3134700000	3656	22.44	22.28	22.51	22.74	22.52	22.54	22.43	22.66
0.165	-0.04	0.97	O35685	Nuclear migration protein nudC	Nudc	398	12	12	12	34.9	34.9	34.9	38.358	0.0000	40.751	24228000	203	16.24	16.36	16.24	16.40	16.31	16.59	16.13	16.38
0.129	-0.05	0.97	Q9D394	Protein RUFY3	Rufy3	4962	25	24	24	45.8	44.1	44.1	53.006	0.0000	66.804	38784000	213	16.52	16.45	16.83	16.94	16.59	16.69	16.75	16.87
0.068	-0.05	0.97	Q922B1	O-acetyl-ADP-ribose deacetylase MACROD1	Macrod1	4246	4	4	4	17.6	17.6	17.6	35.294	0.0000	21.263	4804300	37	14.77	14.36	14.64	14.11	14.33	14.12	14.98	14.63
0.149	-0.05	0.97	E9Q612	Receptor-type tyrosine-protein phosphatase O	Ptpro	240	7	7	7	9.3	9.3	9.3	138.59	0.0000	71.17	10602000	92	15.60	15.44	15.52	15.45	15.72	15.46	15.74	15.27
0.036	-0.05	0.97	Q3TWF6	WD repeat-containing protein 70	Wdr70	1881	8	8	8	15.7	15.7	15.7	73.118	0.0000	12.822	3230900	48	13.38	12.42	14.08	13.82	12.85	14.02	13.58	13.43
0.040	-0.05	0.97	Q8C3R1	BRCA1-associated ATM activator 1	Brat1	3379	4	4	4	8.8	8.8	8.8	89.085	0.0000	36.496	2504000	34	13.12	13.76	14.82	14.25	14.30	14.28	13.48	14.07
0.034	-0.05	0.97	P53566	CCAAT/enhancer-binding protein alpha	Cebpa	1233	2	2	2	6.1	6.1	6.1	37.43	0.0027	2.7272	1653300	19	13.40	13.68	13.78	14.16	14.61	14.61	12.91	13.07
0.049	-0.05	0.97	Q9JHW4	Selenocysteine-specific elongation factor	Eefsec	5377	11	11	11	31.4	31.4	31.4	63.538	0.0000	95.786	17864000	154	15.98	16.12	15.60	15.71	16.15	14.93	16.20	16.33
0.082	-0.05	0.97	Q60520	Paired amphipathic helix protein Sin3a	Sin3a	2178	24	24	24	20.2	20.2	20.2	145.09	0.0000	95.532	18822000	149	15.89	15.18	15.75	15.27	15.50	15.87	15.26	15.66
0.112	-0.05	0.97	Q8JZX4	Splicing factor 45	Rbm17	3586	8	8	8	24	24	24	45.303	0.0000	31.787	15830000	139	15.94	16.26	16.07	16.47	16.16	16.01	16.20	16.56
0.027	-0.05	0.97	A2BE28	Ribosomal biogenesis protein LAS1L	Las1l	45	6	6	6	10.4	10.4	10.4	89.413	0.0000	12.26	4183300	21	13.07	14.57	14.76	15.36	13.53	15.51	14.56	14.36
0.014	-0.05	0.97	Q8R105	Vacuolar protein sorting-associated protein 37C	Vps37c	3772	7	7	7	22.4	22.4	22.4	38.452	0.0000	40.204	22621000	71	17.58	17.66	12.77	17.00	16.80	15.24	15.95	17.24
0.160	-0.05	0.97	Q9JK48	Endophilin-B1	Sh3glb1	5444	24	24	22	55.9	55.9	55.9	40.855	0.0000	323.31	96090000	484	16.99	17.14	17.28	17.44	17.39	17.30	17.04	17.33
0.035	-0.05	0.96	Q9JHZ2	Progressive ankylosis protein	Ankh	5379	1	1	1	4.3	4.3	4.3	54.297	0.0000	6.6917	2344900	32	14.34	14.14	12.91	13.12	12.84	14.35	14.19	13.34
0.202	-0.05	0.96	Q8BUK6	Protein Hook homolog 3	Hook3	3226	21	21	21	39	39	39	83.217	0.0000	125.38	132230000	253	20.26	20.23	20.31	20.60	20.45	20.52	20.25	20.40
0.064	-0.05	0.96	Q920L1	Fatty acid desaturase 1	Fads1	4212	4	4	4	13.6	13.6	13.6	52.322	0.0000	8.0352	2700700	14	13.00	13.12	13.35	14.23	13.21	13.61	13.47	13.63
0.171	-0.05	0.96	O70439	Syntaxin-7	Stx7	499	11	11	11	51.7	51.7	51.7	29.82	0.0000	247.03	124720000	406	18.57	18.36	18.88	18.55	18.56	18.80	18.56	18.65
0.105	-0.05	0.96	O70493	Sorting nexin-12	Snx12	507	15	13	13	76.4	72.1	72.1	19.116	0.0000	112.77	83004000	375	17.83	17.82	17.48	17.93	17.63	17.58	17.77	18.29
0.044	-0.06	0.96	Q9D1P0	39S ribosomal protein L13, mitochondrial	Mrpl13	4929	7	7	7	51.7	51.7	51.7	20.677	0.0000	17.373	7727900	41	15.24	14.76	14.46	15.68	15.63	15.61	14.21	14.92
0.109	-0.06	0.96	P49312	Heterogeneous nuclear ribonucleoprotein A1;Heterogeneous nuclear rit	Hnrnpa1	1160	23	23	23	79.7	79.7	79.7	34.196	0.0000	283.15	65335000	458	17.34	17.55	16.71	17.32	17.35	17.29	17.14	17.36
0.085	-0.06	0.96	Q99K01	Pyridoxal-dependent decarboxylase domain-containing protein 1	Pdxdc1	4359	27	27	27	38.5	38.5	38.5	87.334	0.0000	249.93	50631000	411	16.13	16.20	17.09	16.66	16.40	16.66	16.50	16.72
0.029	-0.06	0.96	P04370	Myelin basic protein	Mbp	641	4	4	4	14.8	14.8	14.8	27.167	0.0000	11.048	3443900	45	13.58	14.78	12.97	14.67	13.17	13.51	14.24	15.31
0.155	-0.06	0.96	Q9Z0R6	Intersectin-2	Itsn2	5782	33	33	33	23.4	23.4	23.4	188.91	0.0000	232.1	37682000	371	15.71	15.64	15.40	15.73	15.33	15.80	15.73	15.84
0.133	-0.06	0.96	Q62261	Spectrin beta chain, non-erythrocytic 1	Sptbn1	2373	83	83	81	43	43	42.2	274.22	0.0000	323.31	131300000	1218	16.95	16.67	16.97	17.09	16.59	17.06	17.07	17.18
0.064	-0.06	0.96	Q80U38	Protein KHNYN	Khnyn	2817	5	5	5	12.2	12.2	12.2	74.564	0.0000	27.192	3575300	89	14.79	13.82	13.62	14.35	14.25	14.66	13.98	13.91
0.252	-0.06	0.96	P70335	Rho-associated protein kinase 1	Rock1	1555	35	35	31	32.1	32.1	29.6	158.17	0.0000	207.31	35153000	533	15.58	15.37	15.40	15.75	15.53	15.52	15.63	15.66
0.032	-0.06	0.96	P70444	BH3-interacting domain death agonist;BH3-interacting domain death ag	Bid	1575	5	5	5	34.4	34.4	34.4	21.951	0.0000	46.989	6643400	94	14.62	14.88	14.31	14.38	14.55	16.04	14.82	13.01
0.059	-0.06	0.96	Q922D4	Serine/threonine-protein phosphatase 6 regulatory subunit 3	Ppp6r3	4250	4	4	4	6.8	6.8	6.8	94.652	0.0000	16.464	15790000	41	16.69	16.64	18.00	17.27	16.76	17.43	17.25	17.39
0.028	-0.06	0.96	Q8VI94	2-5-oligoadenylate synthase-like protein 1	Oasl1	4033	7	7	7	19	19	19	59.088	0.0000	22.603	2897100	24	13.18	11.46	12.72	12.42	11.78	12.10	11.73	14.41
0.139	-0.06	0.96	Q3SXD3	HD domain-containing protein 2	Hddc2	1810	3	3	3	20.1	20.1	20.1	22.753	0.0000	6.8691	1333100	15	12.93	12.61	13.11	13.21	13.09	12.83	12.93	13.26
0.069	-0.06	0.96	Q8C1Y8	Vacuolar fusion protein CCZ1 homolog	Ccz1	3368	9	9	9	25.8	25.8	25.8	55.504	0.0000	36.494	12892000	170	14.68	15.12	16.09	15.52	15.43	15.47	15.20	15.56
0.144	-0.06	0.96	Q9QZQ1	Afadin	Mllt4	5603	39	39	39	30.4	30.4	30.4	206.5	0.0000	167.49	49910000	515	16.18	16.11	16.66	16.59	16.23	16.62	16.43	16.51
0.092	-0.06	0.96	B2RUP2	Protein unc-13 homolog D	Unc13d	80	16	16	16	21	21	21	123.12	0.0000	73.081	25492000	191	17.30	16.81	16.77	16.76	16.81	16.49	17.13	17.46
0.216	-0.06	0.96	Q9CQC7	NADH dehydrogenase [ubiquinone] 1 beta subcomplex subunit 4	Ndufb4	4583	8	8	8	54.3	54.3	54.3	15.081	0.0000	77.319	31219000	199	16.39	16.46	16.73	16.34	16.50	16.77	16.44	16.48
0.025	-0.06	0.96	Q9D289	Trafficking protein particle complex subunit 6B	Trappc6b	4939	5	5	5	34.8	34.8	34.8	17.936	0.0000	43.742	7520600	73	16.82	16.07	13.40	15.30	16.49	14.86	14.58	15.91
0.089	-0.06	0.96	P11247	Myeloperoxidase;Myeloperoxidase light chain;Myeloperoxidase heavy c	Mpo	740	36	36	33	48.6	48.6	45.7	81.181	0.0000	320.36	166900000	698	17.31	17.14	17.85	17.49	17.99	17.67	17.36	17.01
0.056	-0.06	0.96	Q3V1V3	ESF1 homolog	Esf1	2050	3	3	3	3.7	3.7	3.7	98.047	0.0000	6.5781	986480	4	13.86	12.73	12.34	12.62	13.60	12.74	12.56	12.91
0.166	-0.06	0.96	Q8R4V1	NFAT activation molecule 1	Nfam1	3874	6	6	6	25.4	25.4	25.4	29.987	0.0000	39.166	14343000	106	15.98	16.07	15.78	15.65	16.27	15.90	15.81	15.75
0.094	-0.07	0.96	O88939	Zinc finger and BTB domain-containing protein 7A	Zbtb7a	585	4	4	4	10.4	10.4	10.4	60.28	0.0000	87.153	9307600	90	15.61	15.67	15.96	15.96	15.30	16.04	15.70	16.42
0.151	-0.07	0.96	Q9Z2I0	LETM1 and EF-hand domain-containing protein 1, mitochondrial	Letm1	5855	25	25	25	37.9	37.9	37.9	82.988	0.0000	317.46	70968000	470	16.87	16.76	17.45	17.15	17.04	17.01	17.29	17.16
0.153	-0.07	0.96	Q8CCP0	Nuclear export mediator factor Nemf	Nemf	3452	24	24	24	26.8	26.8	26.8	121.19	0.0000	65.838	26964000	280	16.06	15.85	15.86	16.14	15.69	16.36	15.94	16.19
0.064	-0.07	0.96	P70227	Inositol 1,4,5-trisphosphate receptor type 3	Itpr3	1537	8	8	8	4.5	4.5	4.5	304.27	0.0000	62.845	5736000	67	14.94	14.02	15.31	14.18	15.08	14.82	14.70	14.12
0.323	-0.07	0.95	Q8CIN4	Serine/threonine-protein kinase PAK 2;PAK-2p27;PAK-2p34	Pak2	3561	40	40	29	76.1	76.1	57.3	57.93	0.0000	323.31	274290000	1154	19.14	19.16	18.99	19.23	19.04	19.33	19.10	19.32
0.126	-0.07	0.95	Q8BSQ9	Protein polybromo-1	Pbrm1	3191	25	25	25	20.9	20.9	20.9	187.19	0.0000	79.074	16997000	133	15.79	15.19	15.75	16.02	15.73	15.65	15.59	16.07
0.259	-0.07	0.95	Q9CZ30	Obg-like ATPase 1	Ola1	4816	21	21	21	59.6	59.6	59.6	44.729	0.0000	215.42	76849000	459	17.58	17.56	17.46	17.86	17.48	17.79	17.69	17.77
0.071	-0.07	0.95	Q6A065	Centrosomal protein of 170 kDa	Cep170	2497	19	19	19	18.7	18.7	18.7	175.05	0.0000	78.582	12656000	143	14.09	14.93	14.14	14.97	14.31	15.23	14.84	14.03
0.197	-0.07	0.95	O70370	Cathepsin S	Ctss	491	21	21	21	59.4	59.4	59.4	38.474	0.0000	323.31	584330000	865	20.33	20.43	20.09	20.57	20.28	20.33	20.38	20.74
0.115	-0.07	0.95	Q8CCN5	Breast carcinoma-amplified sequence 3 homolog	Bcas3	3451	11	11	11	18.1	18.1	18.1	101.02	0.0000	75.917	10227000	128	14.64	14.90	15.68	15.23	15.20	15.20	15.02	15.31
0.059	-0.07	0.95	Q9ERI6	Retinol dehydrogenase 14	Rdh14	5312	7	7	7	33.5	33.5	33.5	36.365	0.0000	13.467	4010100	20	13.62	13.27	14.09	13.03	12.58	13.56	13.84	14.34
0.397	-0.07	0.95	P23492	Purine nucleoside phosphorylase	Pnp	884	25	25	25	79.2	79.2	79.2	32.277	0.0000	323.31	608620000	1283	20.23	20.26	20.13	20.19	20.47	20.32	20.11	20.21
0.164	-0.07	0.95	Q8BWY3	Eukaryotic peptide chain release factor subunit 1	Etf1	3270	13	13	13	38.4	38.4	38.4	49.03	0.0000	267.46	72592000	351	17.72	17.88	17.93	18.26	17.77	17.92	18.02	18.37
0.296	-0.07	0.95	P22682	E3 ubiquitin-protein ligase CBL	Cbl	876	15	15	13	22.3	22.3	20.5	100.56	0.0000	153.87	40444000	261	16.45	16.48	16.79	16.75	16.56	16.84	16.69	16.68
0.456	-0.07	0.95	P26041	Moesin	Msn	916	98	98	81	92.9	92.9	74.9	67.766	0.0000	323.31	5052600000	6590	21.41	21.31	21.40	21.63	21.45	21.54	21.47	21.59
0.152	-0.08	0.95	Q8K297	Procollagen galactosyltransferase 1	Colgalt1	3651	17	17	17	32.1	32.1	32.1	71.06	0.0000	63.054	56244000	251	17.41	17.63	18.05	17.32	17.66	17.93	17.46	17.66
0.231	-0.08	0.95	O08992	Syntenin-1	Sdcbp	313	19	19	19	71.6	71.6	71.6	32.379	0.0000	227.1	65124000	408	16.88	16.97	17.11	17.35	17.01	17.01	17.26	17.33
0.273	-0.08	0.95	P60122	RuvB-like 1	Ruvbl1	1351	30	30	29	75.2	75.2	75.2	50.213	0.0000	256.59	103170000	673	16.96	17.11	17.43	17.36	17.21	17.30	17.38	17.28
0.102	-0.08	0.95	Q9R049	E3 ubiquitin-protein ligase AMFR	Amfr	5613	9	9	9	21.3	21.3	21.3	73.104	0.0000	62.321	9293700	108	15.52	14.85	15.54	14.65	14.91	15.18	15.15	15.64
0.294	-0.08	0.95	O08788	Dynactin subunit 1	Dctn1	299	56	56	56	49.2	49.2	49.2	141.67	0.0000	323.31	143300000	1079	17.39	17.22	17.50	17.50	17.42	17.72	17.48	17.30
0.139	-0.08	0.95	Q3THG9	Alanyl-tRNA editing protein Aarsd1	Aarsd1	1844	11	11	10	40.8	40.8	39.1	44.97	0.0000	56.784	14381000	147	15.11	15.17	16.00	15.52	15.61	15.42	15.60	15.47
0.261	-0.08	0.95	P62305	Small nuclear ribonucleoprotein E	Snrpe	1417	5	5	5	59.8	59.8	59.8	10.803	0.0000	107.04	111310000	263	18.96	19.18	19.28	19.54	19.27	19.38	19.30	19.33
0.058	-0.08	0.95	Q9DAI2	Intraflagellar transport protein 22 homolog	Ift22	5107	2	2	2	22.7	22.7	22.7	20.833	0.0000	12.262	2274400	30	14.04	14.40	13.52	14.31	14.63	12.92	14.84	14.19
0.237	-0.08	0.95	Q61584	Fragile X mental retardation syndrome-related protein 1	Fxr1	2315	20	20	19	35.9	35.9	34.7	76.221	0.0000	102.49	34005000	412	16.03	15.87	15.91	16.21	15.82	16.14	16.02	16.35

0.126	-0.08	0.95	Q3TC46	Protein PAT1 homolog 1	Patl1	1823	10	10	10	16.6	16.6	16.6	86.769	0.0000	71.248	17111000	165	15.79	15.48	15.88	16.12	15.72	16.46	15.58	15.83
0.197	-0.08	0.95	Q99LJ0	CTTNBP2 N-terminal-like protein	Cttnbp2nl	4435	19	19	19	37.9	37.9	37.9	69.84	0.0000	54.139	14515000	165	14.94	15.18	14.74	15.00	14.78	15.02	14.98	15.41
0.066	-0.08	0.95	Q9D404	3-oxoacyl-[acyl-carrier-protein] synthase, mitochondrial	Oxsm	4971	5	5	5	21.4	21.4	21.4	48.627	0.0000	52.531	3925400	68	13.96	14.71	15.11	14.41	13.93	14.10	15.29	15.18
0.165	-0.08	0.95	P13864	DNA (cytosine-5)-methyltransferase 1	Dnmt1	770	36	36	36	30.2	30.2	30.2	183.19	0.0000	126.28	37291000	395	16.12	15.99	16.33	16.40	16.12	16.78	16.14	16.11
0.456	-0.08	0.95	P57787	Monocarboxylate transporter 4	Slc16a3	1302	3	3	3	7.9	7.9	7.9	50.373	0.0000	14.643	3455500	29	13.76	13.94	13.89	13.90	14.01	13.82	13.86	14.12
0.063	-0.08	0.94	Q8K4J6	MKL/myocardin-like protein 1	Mkl1	3718	6	5	5	12.8	11.4	11.4	102.54	0.0000	39.183	2627400	50	14.07	14.62	13.49	13.97	13.34	13.87	14.04	15.23
0.062	-0.08	0.94	Q80ZQ9	Protein Simiate	Fam206a	2919	5	5	5	46.6	46.6	46.6	21.597	0.0000	20.544	1162900	15	13.43	13.43	12.81	12.29	12.42	13.90	12.39	13.58
0.178	-0.08	0.94	Q5SWD9	Pre-rRNA-processing protein TSR1 homolog	Tsr1	2155	10	10	10	17.3	17.3	17.3	92.104	0.0000	40.131	7235100	105	14.91	14.24	14.48	14.71	14.38	14.76	14.64	14.90
0.211	-0.08	0.94	O55142	60S ribosomal protein L35a	Rpl35a	461	14	14	14	67.3	67.3	67.3	12.554	0.0000	49.023	84327000	275	18.94	18.31	18.32	18.61	18.59	18.72	18.48	18.73
0.231	-0.09	0.94	Q9R1P4	Proteasome subunit alpha type-1	Psma1	5656	22	22	22	76.8	76.8	76.8	29.546	0.0000	323.31	304380000	809	19.33	19.23	19.78	19.33	19.47	19.75	19.38	19.42
0.088	-0.09	0.94	Q9CPY1	39S ribosomal protein L51, mitochondrial	Mrpl51	4545	3	3	3	32.8	32.8	32.8	15.103	0.0000	25.465	12245000	113	16.11	15.86	15.46	15.80	15.87	16.35	16.38	14.97
0.097	-0.09	0.94	Q9WUU7	Cathepsin Z	Ctsz	5723	6	6	6	17.6	17.6	17.6	33.996	0.0000	28.671	76798000	198	18.21	19.20	18.79	18.12	18.42	19.04	18.21	18.99
0.082	-0.09	0.94	Q99PU8	Putative ATP-dependent RNA helicase DHX30	Dhx30	4517	18	18	18	19.1	19.1	19.1	136.67	0.0000	51.56	14488000	150	15.23	13.85	15.42	15.22	14.77	15.16	15.27	14.87
0.058	-0.09	0.94	Q9JI78	Peptide-N(4)-(N-acetyl-beta-glucosaminyl)asparagine amidase	Ngly1	5390	5	5	4	14.9	14.9	10	74.274	0.0004	5.112	1617000	11	13.14	12.92	13.30	13.30	13.42	12.08	12.92	14.59
0.232	-0.09	0.94	Q61183;Q9\	Poly(A) polymerase alpha;Poly(A) polymerase beta	Papola;Papolb	2269	6	6	6	12.2	12.2	12.2	82.308	0.0000	18.805	12463000	81	15.54	15.59	16.13	16.04	15.97	15.86	15.90	15.92
0.104	-0.09	0.94	Q8VEA8	Ras-related protein Rab-7b	Rab7b	3997	6	6	6	37.7	37.7	37.7	22.502	0.0000	36.459	8470500	91	14.67	13.86	14.83	14.99	14.30	14.43	14.90	15.09
0.179	-0.09	0.94	P62281	40S ribosomal protein S11	Rps11	1415	26	26	26	78.5	78.5	78.5	18.431	0.0000	139.02	575860000	1091	20.31	20.01	20.01	20.15	19.90	20.65	19.94	20.34
0.204	-0.09	0.94	Q8C0L8	Conserved oligomeric Golgi complex subunit 5	Cog5	3343	15	15	15	26.8	26.8	26.8	91.39	0.0000	61.782	21589000	116	16.72	16.92	17.34	17.28	16.97	17.16	17.38	17.10
0.092	-0.09	0.94	Q9R257	Heme-binding protein 1	Hebp1	5671	8	8	8	71.6	71.6	71.6	21.067	0.0000	153.69	36223000	209	16.52	17.21	16.32	17.40	16.66	16.54	17.00	17.61
0.180	-0.09	0.94	Q8BX57	PX domain-containing protein kinase-like protein	Pxk	3278	11	11	11	26.8	26.8	26.8	65.23	0.0000	31.468	7964000	101	14.60	14.76	15.06	14.70	14.85	15.26	14.44	14.94
0.133	-0.09	0.94	Q9CY94	DNA replication complex GINS protein PSF3	Gins3	4791	4	4	4	26.9	26.9	26.9	24.576	0.0000	17.542	2487100	39	13.77	13.97	12.91	13.85	13.85	13.63	13.50	13.88
0.336	-0.09	0.94	Q6ZQI3	Malectin	Mlec	2697	17	17	17	51.2	51.2	51.2	32.342	0.0000	126.01	50723000	261	17.34	17.18	17.68	17.53	17.42	17.58	17.58	17.52
0.062	-0.09	0.94	Q8K2Y9	Cerebral cavernous malformations protein 2 homolog	Ccm2	3679	3	3	3	12.4	12.4	12.4	49.917	0.0000	6.6034	1900000	29	13.92	15.05	13.06	13.35	13.33	13.67	14.63	14.12
0.145	-0.09	0.94	Q80X41	Serine/threonine-protein kinase VRK1	Vrk1	2869	18	18	18	45	45	45	49.74	0.0000	175.9	38151000	360	17.21	16.55	16.94	17.18	16.97	17.61	16.73	16.94
0.152	-0.09	0.94	Q8K301	Probable ATP-dependent RNA helicase DDX52	Ddx52	3682	6	6	6	15.6	15.6	15.6	67.473	0.0000	83.499	3402800	34	14.12	13.86	14.25	14.30	13.84	14.17	14.05	14.83
0.308	-0.09	0.94	Q3TCJ1	BRISC complex subunit Abro1	Fam175b	1827	17	17	17	48.9	48.9	48.9	46.942	0.0000	41.662	48612000	274	16.80	16.33	16.68	16.58	16.51	16.60	16.80	16.86
0.274	-0.09	0.94	Q64737	Trifunctional purine biosynthetic protein adenosine-3;Phosphoribosylam	Gart	2464	48	48	48	58.3	58.3	58.3	107.5	0.0000	323.31	183360000	1349	18.07	18.22	18.40	18.52	18.14	18.34	18.55	18.57
0.529	-0.09	0.94	P35279;P61	Ras-related protein Rab-6A;Ras-related protein Rab-6B	Rab6a;Rab6b	1014	16	16	14	64.9	64.9	58.7	23.59	0.0000	139.85	185030000	452	19.89	19.74	19.82	19.88	20.04	19.72	19.91	20.04
0.247	-0.10	0.94	O70566	Protein diaphanous homolog 2	Diaph2	513	34	34	34	39	39	39	124.87	0.0000	308.47	80109000	527	17.39	17.03	17.11	17.55	17.10	17.58	17.41	17.37
0.137	-0.10	0.94	Q9CRA8	Exosome complex component RRP46	Exosc5	4686	8	8	8	39.6	39.6	39.6	25.194	0.0000	147.69	8244600	97	14.87	14.99	14.56	14.40	14.19	15.22	14.75	15.04
0.216	-0.10	0.94	P48678	Prelamin-A/C;Lamin-A/C	Lmna	1147	94	94	92	90.1	90.1	90.1	74.237	0.0000	323.31	862330000	2869	19.44	19.66	19.23	19.66	19.30	19.94	19.41	19.73
0.174	-0.10	0.93	Q9D868	Peptidyl-prolyl cis-trans isomerase H	Ppih	5052	8	8	8	60.6	60.6	60.6	20.463	0.0000	60.608	16044000	160	15.37	15.91	16.01	16.23	15.79	15.75	16.19	16.17
0.336	-0.10	0.93	Q9CXY6	Interleukin enhancer-binding factor 2	Ilf2	4774	16	16	16	49.5	49.5	49.5	43.062	0.0000	107.94	43560000	283	17.51	17.35	17.59	17.56	17.55	17.93	17.49	17.43
0.334	-0.10	0.93	Q8R574	Phosphoribosyl pyrophosphate synthase-associated protein 2	Prpsap2	3881	18	18	16	60.2	60.2	56.4	40.88	0.0000	138.74	41018000	328	16.11	16.02	16.40	16.16	16.21	16.24	16.09	16.54
0.055	-0.10	0.93	Q9CRB2	H/ACA ribonucleoprotein complex subunit 2	Nhp2	4687	3	3	3	23.5	23.5	23.5	17.247	0.0000	24.463	10764000	119	14.56	15.36	14.53	14.67	15.41	15.62	15.40	13.09
0.157	-0.10	0.93	Q8C4Q6	Axin interactor, dorsalization-associated protein	Aida	3387	6	6	6	28.5	28.5	28.5	34.888	0.0000	18.504	15125000	43	17.48	17.35	16.65	17.22	17.51	16.83	17.44	17.32
0.094	-0.10	0.93	Q9JJR8;Q9(	Transmembrane protein 9B;Transmembrane protein 9	Tmem9b;Tmem9	5433	4	4	4	25.6	25.6	25.6	22.607	0.0000	32.687	4583200	58	13.42	14.62	15.04	14.83	14.75	14.40	14.86	14.29
0.159	-0.10	0.93	Q8K296	Myotubularin-related protein 3	Mtmr3	3650	9	9	9	13.7	13.7	13.7	133.84	0.0000	43.594	15033000	46	16.06	15.81	15.98	16.28	15.54	16.64	16.23	16.13
0.415	-0.10	0.93	Q9JLI8	Squamous cell carcinoma antigen recognized by T-cells 3	Sart3	5481	24	24	23	32.1	32.1	32	109.62	0.0000	186.31	30081000	323	15.71	15.70	15.71	16.08	15.84	16.05	15.81	15.91
0.320	-0.10	0.93	O35601	FYN-binding protein	Fyb	384	28	28	28	38.1	38.1	38.1	90.054	0.0000	323.31	51254000	447	16.25	16.40	16.27	16.62	16.31	16.67	16.30	16.67
0.164	-0.10	0.93	P36916	Guanine nucleotide-binding protein-like 1	Gnl1	1044	10	10	10	22.4	22.4	22.4	68.77	0.0000	20.445	6260800	45	14.66	14.62	14.55	15.04	15.07	14.84	14.21	15.16
0.200	-0.10	0.93	Q8VE88	Protein FAM114A2	Fam114a2	3990	6	6	6	19.7	19.7	19.7	54.044	0.0000	104.97	10599000	162	14.71	14.95	15.08	15.50	14.84	15.31	15.13	15.38
0.207	-0.10	0.93	Q9D8S3	ADP-ribosylation factor GTPase-activating protein 3	Arfgap3	5069	20	19	19	41.7	39.8	39.8	57.455	0.0000	114.39	38987000	291	15.67	15.96	16.26	15.91	15.59	16.12	16.23	16.28
0.101	-0.11	0.93	A2A4P0	ATP-dependent RNA helicase DHX8	Dhx8	6	7	6	6	9	8.3	8.3	142.57	0.0000	10.048	14697000	33	16.67	16.67	17.79	17.27	17.63	17.69	16.87	16.62
0.209	-0.11	0.93	Q9Z1R2	Large proline-rich protein BAG6	Bag6	5820	23	23	23	31.9	31.9	31.9	121.04	0.0000	216.32	41630000	326	16.66	16.54	17.06	16.83	16.44	16.83	16.99	17.26
0.644	-0.11	0.93	Q9CRY7	Glycerophosphodiester phosphodiesterase domain-containing protein 1	Gdpd1	4695	9	9	9	27.1	27.1	27.1	35.866	0.0000	19.514	16941000	172	15.98	15.91	16.24	15.94	16.05	16.17	16.16	16.12
0.251	-0.11	0.93	P51432	1-phosphatidylinositol 4,5-bisphosphate phosphodiesterase beta-3	Plcb3	1195	23	23	23	26.7	26.7	26.7	139.49	0.0000	81.994	21527000	197	15.58	15.32	15.92	15.29	15.55	15.89	15.42	15.68
0.201	-0.11	0.93	P00375	Dihydrofolate reductase	Dhfr	609	15	15	15	75.9	75.9	75.9	21.606	0.0000	130.13	41741000	218	16.87	17.27	17.74	17.54	17.58	17.42	17.19	17.66
0.276	-0.11	0.93	P48428	Tubulin-specific chaperone A	Tbca	1144	7	7	7	70.4	70.4	70.4	12.758	0.0000	57.961	18704000	146	16.17	16.39	15.77	16.37	16.37	16.45	16.11	16.21
0.056	-0.11	0.93	Q62093	Serine/arginine-rich splicing factor 2	Srsf2	2355	10	10	9	34.8	34.8	34.8	25.476	0.0000	57.496	19025000	89	17.58	15.56	15.51	16.79	15.86	16.86	15.49	17.66
0.121	-0.11	0.93	Q9WU28	Prefoldin subunit 5	Pfdn5	5695	8	8	8	57.1	57.1	57.1	17.356	0.0000	64.102	6014800	102	14.49	14.89	13.53	14.80	14.23	14.95	14.50	14.47
0.325	-0.11	0.93	Q91YT0	NADH dehydrogenase [ubiquinone] flavoprotein 1, mitochondrial	Ndufv1	4178	21	21	21	61.4	61.4	61.4	50.834	0.0000	81.645	42011000	282	16.11	16.06	15.94	15.89	16.48	16.02	15.82	16.12
0.599	-0.11	0.92	O35857	Mitochondrial import inner membrane translocase subunit TIM44	Timm44	407	26	26	26	52.7	52.7	52.7	51.091	0.0000	137.99	67268000	601	16.51	16.30	16.57	16.44	16.39	16.69	16.55	16.65
0.058	-0.11	0.92	Q91YJ3	Thymocyte nuclear protein 1	Thyn1	4162	6	6	6	36.3	36.3	36.3	26.178	0.0000	28.43	6026800	62	14.16	15.10	12.19	14.36	13.28	14.04	14.13	14.82
0.547	-0.12	0.92	Q8BUM3	Tyrosine-protein phosphatase non-receptor type 7	Ptpn7	3228	11	11	11	39.6	39.6	39.6	40.351	0.0000	86.873	22270000	159	16.36	16.40	16.76	16.66	16.64	16.69	16.71	16.62
0.320	-0.12	0.92	Q9WTX2	Interferon-inducible double-stranded RNA-dependent protein kinase act	Prkra	5691	15	15	15	46	46	46	34.371	0.0000	46.063	28377000	202	16.21	16.17	15.83	16.36	15.98	16.48	16.36	16.23
0.358	-0.12	0.92	Q8CH18	Cell division cycle and apoptosis regulator protein 1	Ccar1	3517	20	20	20	21.9	21.9	21.9	132.06	0.0000	188.54	33251000	302	16.43	16.47	16.70	16.79	16.63	16.92	16.87	16.44
1.054	-0.12	0.92	P52480	Pyruvate kinase PKM	Pkm	1219	79	79	76	94.2	94.2	90	57.844	0.0000	323.31	8844600000	9714	23.27	23.15	23.19	23.08	23.36	23.32	23.33	23.16
0.561	-0.12	0.92	Q9Z0X1	Apoptosis-inducing factor 1, mitochondrial	Aifm1	5791	31	31	31	53.4	53.4	53.4	66.765	0.0000	295.92	101030000	560	17.49	17.24	17.61	17.55	17.46	17.61	17.57	17.73
0.112	-0.12	0.92	Q8R088	Golgi phosphoprotein 3-like	Golph3l	3752	8	7	7	33	28.4	28.4	32.905	0.0000	22.755	4417500	29	13.92	13.91	14.03	14.03	12.91	14.37	14.55	14.54
0.175	-0.12	0.92	Q149L6	DnaJ homolog subfamily B member 14	Dnajb14	1786	8	8	8	23	23	23	42.325	0.0000	18.291	6509400	60	14.10	13.77	14.72	14.24	13.88	14.69	14.19	14.56
0.058	-0.12	0.92	Q5XPI3	E3 ubiquitin-protein ligase RNF123	Rnf123	2175	3	3	3	5.2	5.2	5.2	148.65	0.0000	58.87	2481800	46	14.34	11.95	15.22	14.58	14.63	13.78	14.28	13.88
0.218	-0.12	0.92	Q9CR51	V-type proton ATPase subunit G 1	Atp6v1g1	4667	10	10	10	55.1	55.1	55.1	13.724	0.0000	323.31	35276000	204	16.29	16.79	15.98	16.33	16.16	16.86	16.36	16.50
0.217	-0.12	0.92	Q9QZK7	Docking protein 3	Dok3	5599	15	15	15	55.2	55.2	55.2	48.027	0.0000	73.4	37997000	300	15.39	15.99	16.37	16.30	16.18	16.14	16.14	16.07
0.302	-0.12	0.92	Q8BWW4	La-related protein 4	Larp4	3268	6	6	6	11.8	11.8	11.8	79.763	0.0000	29.883	4898100	54	14.04	14.06	14.00	14.14	14.04	14.41	13.78	14.50
0.116	-0.12	0.92	Q6ZQL4	WD repeat-containing protein 43	Wdr43	2701	7	7	7	14.5	14.5	14.5	75.38	0.0000	14.987	4962600	59	14.86	13.43	14.57	13.81	14.65	13.80	14.66	14.05
0.406	-0.12	0.92	O89110	Caspase-8;Caspase-8 subunit p18;Caspase-8 subunit p10	Casp8	605	32	32	32	71.7	71.7	71.7	55.356	0.0000	181.6	88587000	749	16.80	17.05	16.45	16.81	16.77	16.85	16.97	17.02
0.585	-0.12	0.92	P60710	Actin, cytoplasmic 1;Actin, cytoplasmic 1, N-terminally processed	Actb	1358	59	59	3	99.5	99.5	7.5	41.736	0.0000	323.31	27455000000	13874	24.23	24.17	24.34	24.13	24.40	24.57	24.21	24.19
0.252	-0.13	0.92	Q62384	Zinc finger protein ZPR1	Zpr1	2384	6	6	6	20	20	20	50.714	0.0000	59.74	5833500	77	14.00	14.79	14.34	14.47	14.44	14.86	14.52	14.28
0.269	-0.13	0.92	Q99J95	Cyclin-dependent kinase 9	Cdk9	4334	6	5	5	16.7	14.5	14.5	42.761	0.0000	20.327	4411200	72	14.22	14.11	13.84	14.56	14.10	14.16	14.31	14.65
0.983	-0.13	0.92	O88685	26S protease regulatory subunit 6A	Psmc3	558	36	36	36	86.7	86.7	86.7	49.548	0.0000	323.31	179930000	886	17.91	18.00	18.12	18.17	18.11	18.20	18.16	18.25
0.168	-0.13	0.92	Q9D6J5	NADH dehydrogenase [ubiquinone] 1 beta subcomplex subunit 8, mitoc	Ndufb8	4996	6	6	6	43	43	43	21.876	0.0000	52.811	21004000	150	15.50	15.78	16.62	16.22	16.03	16.57	16.20	15.82
0.635	-0.13	0.92	Q8BHG2	UPF0587 protein C1orf123 homolog		3037	4	4	4	31.9	31.9	31.9	18.02	0.0000	15.059	11778000	138	15.33	15.41	15.44	15.45	15.32	15.48	15.59	15.76
0.573	-0.13	0.92	Q3TRM8	Hexokinase-3	Hk3	1872	55	54	52	69.6	68.3	66.8	100.1	0.0000	323.31	504320000	1766	19.18	19.22	19.08	19.25	19.33	19.03	19.46	19.41
0.157	-0.13	0.92	Q8BG51	Mitochondrial Rho GTPase 1	Rhot1	2964	13	13	12	25.8	25.8	24.1	72.241	0.0000	73.893	11161000	118	14.96	14.14	15.57	15.26	15.12	15.04	15.26	15.02
0.728	-0.13	0.92	Q8BSY0	Aspartyl/asparaginyl beta-hydroxylase	Asph	3193	36	36	36	52.9	52.9	52.9	83.041	0.0000	314.68	88165000	761	17.19	17.01	17.31	17.01	17.18	17.39	17.21	17.26
0.068	-0.13	0.91	Q9CR29	Coiled-coil domain-containing protein 43	Ccdc43	4663	7	7	7	40.1	40.1	40.1	25.049	0.0000	84.577	3060500	27	13.71	11.29	11.07	11.78	12.72	12.41	11.36	11.87
0.831	-0.13	0.91	Q8K1B8	Fermitin family homolog 3	Fermt3	3622	47	47	47	73.7	73.7	73.7	75.634	0.0000	323.31	321670000	1289	18.65	18.58	18.83	18.60	18.82	18.66	18.79	18.91
0.133	-0.13	0.91	Q8VDD9	PH-interacting protein	Phip	3950	7	7	7	4.6	4.6	4.6	206.72	0.0000	14.054	11630000	52	16.42	15.78	17.11	16.25	16.13	17.02	16.11	16.82
0.222	-0.13	0.91	Q61072	Disintegrin and metalloproteinase domain-containing protein 9	Adam9	2244	2	2	2	4.5	4.5	4.5	92.079	0.0000	34.098	4226800	48	14.74	15.15	15.22	14.69	14.52	15.24	15.32	15.23
0.307	-0.13	0.91	Q62318	Transcription intermediary factor 1-beta	Trim28	2379	36	36	36	46.6	46.6	46.6	88.846	0.0000	323.31	148910000	774	18.11	17.94	18.45	18.22	18.64	18.19	18.42	17.99
0.907	-0.13	0.91	P48036	Annexin A5	Anxa5	1140	51	51	29	86.2	86.2	52.4	35.752	0.0000	323.31	2107300000	2678	21.45	21.36	21.35	21.37	21.39	21.51	21.44	21.70
0.183	-0.13	0.91	Q8VCR7	Alpha/beta hydrolase domain-containing protein 14B	Abhd14b	3930	9	9	9	67.1	67.1	67.1	22.45	0.0000	63.852	14416000	149	15.09	15.11	15.76	15.77	15.55	15.11	16.08	15.52
0.173	-0.13	0.91	Q6PGL7	WASH complex subunit FAM21	Fam21	2639	23	23	23	36.1	36.1	36.1	145.31	0.0000	158.92	14115000	139	15.19	15.29	14.84	15.66	15.22	16.00	15.40	14.86
0.200	-0.13	0.91	Q9CZ28	Vacuolar-sorting protein SNF8	Snf8	4815	10	10	10	51.2	51.2	51.2	28.886	0.0000	81.385	20674000	111	15.87	16.30	16.67	16.87	16.23	16.69	16.84	16.47
0.099	-0.13	0.91	Q9CQU3	Protein RER1	Rer1	4635	6	6	6	34.7	34.7	34.7	22.988	0.0000	19.899	4460800	34	14.66	13.67	15.09	14.41	14.86	13.44	14.99	15.05
0.053	-0.13	0.91	Q9JKL4	NADH dehydrogenase [ubiquinone] 1 alpha subcomplex assembly factc	Ndufaf3	5454	3	3	3	30.8	30.8	30.8	20.733	0.0000	34.891	4181300	34	12.85	15.18	15.35	15.81	15.80	14.96	15.58	13.37
0.418	-0.13	0.91	Q3UH06	Ras-responsive element-binding protein 1	Rreb1	1965	7	7	7	8.3	8.3	8.3	184.15	0.0000	14.317	1751200	31	13.27	13.74	13.42	13.79	13.74	13.53	13.81	13.67
0.201	-0.13	0.91	Q08093;Q9[	Calponin-2	Cnn2	1760	12	12	12	49.5	49.5	49.5	33.155	0.0000	51.134	24382000	133	16.44	16.09	16.63	16.99	16.25	16.58	16.75	17.10
0.971	-0.13	0.91	Q8K124	Pleckstrin homology domain-containing family O member 2	Plekho2	3613	33	33	33	63.4	63.4	63.4	53.872	0.0000	323.31	153680000	904	17.62	17.47	17.51	17.62	17.59	17.81	17.58	17.75
0.301	-0.13	0.91	P49442	Inositol polyphosphate 1-phosphatase	Inpp1	1162	12	12	12	37.4	37.4	37.4	43.346	0.0000	64.396	9912100	130	15.60	15.44	14.86	15.05	15.50	15.19	15.41	15.38
0.378	-0.13	0.91	Q68FF6	ARF GTPase-activating protein GIT1	Git1	2474	18	14	14	35.3	30.3	30.3	85.299	0.0000	96.015	11872000	138	15.40	15.11	15.59	15.46	15.28	15.43	15.82	15.57
0.361	-0.13	0.91	O08807	Peroxiredoxin-4	Prdx4	302	15	13	13	67.9	65.3	65.3	31.052	0.0000	198.9	393340000	476	21.63	21.71	21.21	21.61	21.98	21.57	21.69	21.44
0.265	-0.13	0.91	Q8BTI8	Serine/arginine repetitive matrix protein 2	Srrm2	3201	29	29	29	14.4	14.4	14.4	294.84	0.0000	188.2	136730000	620	18.30	18.11	18.25	18.11	17.81	18.76	18.23	18.51
0.414	-0.13	0.91	Q5SUF2	Luc7-like protein 3	Luc7l3	2146	16	16	16	32.4	32.4	32.4	51.45	0.0000	123.99	29109000	376	16.32	16.11	16.09	16.38	16.35	16.58	16.02	16.50
0.176	-0.13	0.91	O35855	Branched-chain-amino-acid aminotransferase, mitochondrial	Bcat2	406	17	17	17	49.9	49.9	49.9	44.127	0.0000	153.79	115220000	480	18.52	18.45	18.31	17.53	18.35	18.86	18.18	17.96
0.289	-0.14	0.91	Q60817;P70	Nascent polypeptide-associated complex subunit alpha;Nascent polype	Naca	1581	6	6	6	28.8	28.8	28.8	23.384	0.0000	160.29	20276000	133	16.01	16.42	16.61	16.77	16.40	16.87	16.43	16.66
0.146	-0.14	0.91	Q60953	Protein PML	Pml	2227	10	10	10	16.5	16.5	16.5	98.241	0.0000	57.576	3143600	35	13.59	13.71	12.82	12.73	14.01	13.45	12.87	13.07
0.221	-0.14	0.91	Q61578	NADPH:adrenodoxin oxidoreductase, mitochondrial	Fdxr	2314	11	11	11	35	35	35	54.201	0.0000	66.771	14940000	129	15.54	15.57	16.29	15.85	15.94	16.45	15.83	15.58
0.551	-0.14	0.91	Q9Z160	Conserved oligomeric Golgi complex subunit 1	Cog1	5800	16	16	16	25.2	25.2	25.2	109.05	0.0000	136.35	20233000	263	15.55	15.42	15.96	15.67	15.81	15.72	15.83	15.79
0.227	-0.14	0.91	P56960	Exosome component 10	Exosc10	1294	16	16	16	24.6	24.6	24.6	100.94	0.0000	53.802	14827000	158	15.33	15.31	15.20	15.40	14.84	15.74	15.29	15.93
0.249	-0.14	0.91	P62835	Ras-related protein Rap-1A	Rap1a	1450	17	5	5	83.2	35.3	35.3	20.987	0.0000	33.606	19004000	164	16.08	15.99	16.11	16.60	16.28	16.37	15.91	16.79
0.328	-0.14	0.91	Q9JMH6	Thioredoxin reductase 1, cytoplasmic	Txnrd1	5511	49	49	47	65.9	65.9	63.5	67.083	0.0000	323.31	469310000	1451	19.77	19.41	19.35	19.68	19.91	19.25	19.80	19.80
0.267	-0.14	0.91	Q3TZX8	Polynucleotide 5-hydroxyl-kinase NOL9	Nol9	1892	9	9	8	13.4	13.4	13.3	80.839	0.0000	15.783	12659000	127	15.90	15.42	15.70	15.49	15.68	16.22	15.33	15.83
0.169	-0.14	0.91	Q99N93	39S ribosomal protein L16, mitochondrial	Mrpl16	4491	7	7	7	34.7	34.7	34.7	28.803	0.0000	43.038	6332000	39	15.47	14.63	15.28	15.38	14.59	15.55	15.43	15.75
0.541	-0.14	0.91	P51410	60S ribosomal protein L9	Rpl9	1194	18	18	18	75.5	75.5	75.5	21.881	0.0000	323.31	241160000	716	20.19	19.78	19.94	19.87	20.15	19.88	20.26	20.05
0.214	-0.14	0.91	Q6A0A2	La-related protein 4B	Larp4b	2500	7	7	7	13.9	13.9	13.9	81.626	0.0000	68.929	8605600	70	15.05	15.17	15.58	15.51	14.96	15.84	15.19	15.87
0.117	-0.14	0.91	Q62311	Transcription initiation factor TFIID subunit 6	Taf6	2376	2	2	2	5.6	5.6	5.6	72.673	0.0000	14.221	1090500	18	12.95	13.86	13.75	13.17	13.29	14.55	13.75	12.70
0.197	-0.14	0.91	Q61490	CD166 antigen	Alcam	2305	13	13	13	23.8	23.8	23.8	65.091	0.0000	82.758	33139000	197	16.84	16.91	16.65	17.36	16.52	16.89	17.28	17.63
0.657	-0.14	0.91	P15307	Proto-oncogene c-Rel	Rel	794	11	10	10	22.8	21.3	21.3	64.959	0.0000	31.176	22202000	176	16.28	16.02	16.31	15.98	16.24	16.45	16.30	16.17
0.131	-0.14	0.91	Q14AX6	Cyclin-dependent kinase 12	Cdk12	1787	5	4	4	5.1	4.6	4.6	163.68	0.0000	7.8156	2727200	48	13.37	14.24	13.52	13.93	13.49	14.96	13.39	13.79
0.637	-0.14	0.91	O08808	Protein diaphanous homolog 1	Diaph1	303	63	63	63	51.7	51.7	51.7	139.34	0.0000	323.31	155670000	1155	17.07	16.98	17.10	17.37	17.13	17.20	17.32	17.44
0.368	-0.14	0.91	Q9D6Z1	Nucleolar protein 56	Nop56	5014	31	31	31	66.9	66.9	66.9	64.464	0.0000	323.31	98856000	852	16.16	16.02	16.63	16.49	16.25	16.67	16.57	16.39
0.247	-0.14	0.90	Q91Z53	Glyoxylate reductase/hydroxypyruvate reductase	Grhpr	4187	8	8	8	40.5	40.5	40.5	35.328	0.0000	53.691	9217700	97	15.54	15.25	14.79	14.57	15.27	15.40	15.01	15.06

0.330	-0.15	0.90	P63276	40S ribosomal protein S17	Rps17	1501	17	17	17	83.7	83.7	83.7	15.524	0.0000	323.31	532160000	813	20.39	20.12	20.85	20.66	20.45	20.85	20.79	20.51
0.521	-0.15	0.90	O70405	Serine/threonine-protein kinase ULK1	Ulk1	495	3	3	3	3	3	3	112.46	0.0000	9.3402	8950400	31	16.40	16.49	16.45	16.52	16.30	16.70	16.55	16.90
0.612	-0.15	0.90	P23591	GDP-L-fucose synthase	Tsta3	886	17	17	17	46.1	46.1	46.1	35.877	0.0000	193.76	42643000	305	16.71	16.74	16.83	16.78	16.75	17.12	16.69	17.08
0.280	-0.15	0.90	Q920B0	FERM domain-containing protein 4B	Frmd4b	4208	7	7	7	10.8	10.8	10.8	118.01	0.0002	5.5054	1297900	15	13.37	13.31	13.37	13.24	13.84	13.28	12.95	13.81
0.083	-0.15	0.90	Q99MB1	Toll-like receptor 3	Tlr3	4460	3	3	3	4.5	4.5	4.5	103.67	0.0000	82.729	773810	9	12.61	12.18	13.98	12.30	13.37	14.05	11.83	12.40
0.374	-0.15	0.90	P62307	Small nuclear ribonucleoprotein F	Snrpf	1418	4	4	4	58.1	58.1	58.1	9.7251	0.0000	61.814	23341000	163	16.48	16.71	16.59	16.91	16.51	16.90	16.69	17.18
0.094	-0.15	0.90	Q9CQ06	39S ribosomal protein L24, mitochondrial	Mrpl24	4551	6	6	6	43.5	43.5	43.5	24.944	0.0000	18.105	2782800	47	13.18	13.98	13.75	13.42	14.87	14.24	13.51	12.31
0.270	-0.15	0.90	O88532	Zinc finger RNA-binding protein	Zfr	541	16	16	16	21.9	21.9	21.9	116.86	0.0000	225.83	17268000	299	14.60	14.41	14.88	15.06	14.49	15.27	14.70	15.08
0.281	-0.15	0.90	P08207	Protein S100-A10	S100a10	681	8	8	8	67	67	67	11.186	0.0000	68.213	127530000	357	18.94	18.81	18.05	18.79	18.56	18.98	18.75	18.89
0.200	-0.15	0.90	Q8R1T1	Charged multivesicular body protein 7	Chmp7	3799	8	8	8	25.7	25.7	25.7	50.632	0.0000	67.12	7570600	93	14.97	14.05	14.96	14.60	14.36	14.58	14.98	15.26
0.221	-0.15	0.90	Q923G2	DNA-directed RNA polymerases I, II, and III subunit RPABC3	Polr2h	4286	5	5	5	34	34	34	17.143	0.0000	30.977	10403000	78	15.41	16.67	16.01	15.73	16.13	15.97	16.18	16.14
0.117	-0.15	0.90	Q9Z2A0	3-phosphoinositide-dependent protein kinase 1	Pdpk1	5835	7	7	7	17.7	17.7	17.7	63.758	0.0000	39.56	4825600	71	15.26	15.19	14.09	13.53	14.26	14.53	15.31	14.59
0.709	-0.15	0.90	Q8BV13	COP9 signalosome complex subunit 7b	Cops7b	3234	5	5	4	28	28	20.8	29.689	0.0000	83.065	8058200	124	14.94	14.90	15.02	15.20	15.04	15.40	15.14	15.10
0.247	-0.15	0.90	A2AGT5	Cytoskeleton-associated protein 5	Ckap5	21	60	60	60	40.3	40.3	40.3	225.63	0.0000	323.31	74522000	711	16.03	15.73	16.12	16.33	15.74	15.96	16.42	16.69
0.149	-0.15	0.90	O55236	mRNA-capping enzyme;Polynucleotide 5-triphosphatase;mRNA guanyl	Rngtt	467	10	10	10	21.3	21.3	21.3	68.683	0.0000	10.129	5585500	40	14.52	14.17	12.83	14.05	14.37	13.88	13.83	14.10
1.938	-0.15	0.90	P60766;Q8F	Cell division control protein 42 homolog	Cdc42	1360	19	19	18	53.9	53.9	53.9	21.258	0.0000	255.26	1072900000	1512	20.82	20.73	20.75	20.70	20.85	20.88	21.01	20.87
0.393	-0.15	0.90	Q05D44	Eukaryotic translation initiation factor 5B	Eif5b	1747	43	43	43	48.8	48.8	48.8	137.61	0.0000	216.45	76055000	498	16.99	16.90	17.11	17.31	16.83	17.52	17.33	17.23
0.118	-0.15	0.90	Q99J87	Probable ATP-dependent RNA helicase DHX58	Dhx58	4332	30	30	30	45.7	45.7	45.7	76.708	0.0000	199.07	40493000	345	16.41	16.36	15.22	14.69	16.45	15.90	15.50	15.45
0.391	-0.15	0.90	Q3TIX9	U4/U6.U5 tri-snRNP-associated protein 2	Usp39	1852	11	11	11	28.9	28.9	28.9	65.146	0.0000	59.009	12036000	138	15.12	15.42	15.55	15.50	15.35	15.65	15.30	15.92
0.233	-0.16	0.90	O88983	Syntaxin-8	Stx8	591	9	9	9	37.3	37.3	37.3	26.925	0.0000	103.38	35869000	187	16.74	16.83	17.76	17.48	17.07	17.43	17.37	17.56
0.344	-0.16	0.90	P97351	40S ribosomal protein S3a	Rps3a	1627	39	39	39	75.8	75.8	75.8	29.885	0.0000	323.31	705210000	1848	19.70	19.39	19.56	19.68	19.41	20.20	19.50	19.84
0.236	-0.16	0.90	Q9R1Q6	Transmembrane protein 176B	Tmem176b	5657	3	3	3	10.6	10.6	10.6	28.367	0.0000	20.101	1294200	23	12.86	12.97	12.47	12.28	12.94	12.27	13.28	12.72
0.308	-0.16	0.90	Q9CWL8	Beta-catenin-like protein 1	Ctnnbl1	4720	14	14	13	26.5	26.5	26.5	64.979	0.0000	35.224	24350000	188	16.32	16.47	16.94	16.69	16.92	17.15	16.48	16.49
0.106	-0.16	0.90	P11276	Fibronectin;Anastellin	Fn1	741	12	12	12	7.1	7.1	7.1	272.53	0.0000	73.367	10080000	101	13.95	14.42	16.05	15.77	14.80	15.12	15.71	15.18
0.730	-0.16	0.90	Q9JHW2	Omega-amidase NIT2	Nit2	5376	19	19	19	87.7	87.7	87.7	30.501	0.0000	156.72	40913000	293	16.29	16.34	16.41	16.69	16.47	16.69	16.51	16.69
1.331	-0.16	0.90	P84096	Rho-related GTP-binding protein RhoG	Rhog	1609	14	13	13	63.4	57.6	57.6	21.308	0.0000	256.67	335320000	573	19.33	19.39	19.43	19.54	19.51	19.69	19.51	19.61
0.666	-0.16	0.90	Q9R0P5	Destrin	Dstn	5633	23	18	18	94.5	87.3	87.3	18.521	0.0000	91.881	69173000	388	17.99	17.85	17.63	17.49	17.84	17.91	17.92	17.92
0.496	-0.16	0.90	P29391;P49	Ferritin light chain 1;Ferritin light chain 2	Ftl1;Ftl2	968	19	19	18	69.4	69.4	69.4	20.802	0.0000	323.31	557210000	1034	19.61	19.70	19.97	20.07	19.79	19.88	20.10	20.21
0.081	-0.16	0.89	O35454	Chloride transport protein 6	Clcn6	371	3	3	3	4.7	4.7	4.7	96.979	0.0000	6.7139	906670	9	12.72	12.08	13.97	12.30	13.87	11.51	12.50	13.84
0.553	-0.16	0.89	P97386	DNA ligase 3	Lig3	1642	8	8	8	9.7	9.7	9.7	113.07	0.0000	16.727	7552000	51	15.15	15.11	15.34	15.43	15.13	15.67	15.44	15.43
1.143	-0.16	0.89	P70460	Vasodilator-stimulated phosphoprotein	Vasp	1578	17	17	17	44.3	44.3	44.3	39.666	0.0000	192.42	114710000	529	17.73	17.94	17.75	17.83	18.13	17.96	17.94	17.86
0.825	-0.16	0.89	P32883	GTPase KRas;GTPase KRas, N-terminally processed	Kras	997	13	6	5	77.2	32.8	24.9	21.656	0.0000	34.411	11192000	132	15.07	15.09	15.32	15.27	15.19	15.47	15.48	15.26
0.892	-0.16	0.89	Q99MS7	EH domain-binding protein 1-like protein 1	Ehbp1l1	4476	21	21	21	18.6	18.6	18.6	184.83	0.0000	169.44	31198000	279	16.18	15.88	16.16	16.02	16.10	16.17	16.23	16.38
0.754	-0.16	0.89	Q6ZWU9	40S ribosomal protein S27	Rps27	2706	6	6	3	41.7	41.7	15.5	9.461	0.0000	51.717	142890000	275	19.21	19.24	19.44	19.33	19.27	19.71	19.40	19.48
0.269	-0.16	0.89	Q91VT4	Carbonyl reductase family member 4	Cbr4	4077	8	8	7	44.9	44.9	39.8	25.414	0.0000	27.776	6164500	68	14.60	14.25	15.20	14.88	14.72	15.03	14.60	15.22
0.124	-0.16	0.89	Q8R0Y6	Cytosolic 10-formyltetrahydrofolate dehydrogenase	Aldh1l1	3769	21	21	19	35.4	35.4	33	98.708	0.0000	178.3	22743000	178	14.81	15.17	15.40	15.15	16.12	16.11	14.62	14.34
0.323	-0.16	0.89	Q8VE80	THO complex subunit 3	Thoc3	3989	6	6	6	16.5	16.5	16.5	38.737	0.0000	7.4402	7229500	69	15.34	15.44	14.92	15.41	15.54	15.22	15.89	15.12
0.517	-0.16	0.89	Q3TW96	UDP-N-acetylhexosamine pyrophosphorylase-like protein 1	Uap1l1	1880	38	37	36	69.6	69.6	67.1	56.613	0.0000	323.31	481710000	1297	19.37	19.45	19.30	18.89	19.63	19.41	19.28	19.34
0.304	-0.16	0.89	P14733	Lamin-B1	Lmnb1	785	79	77	70	83.2	83.2	75.9	66.785	0.0000	323.31	404700000	2053	18.31	18.27	18.03	18.22	18.39	18.91	17.85	18.32
0.724	-0.16	0.89	Q8R1Q8	Cytoplasmic dynein 1 light intermediate chain 1	Dync1li1	3794	29	29	28	52.4	52.4	49.9	56.614	0.0000	323.31	109310000	596	18.08	17.83	18.06	18.05	18.01	18.11	18.10	18.44
0.390	-0.16	0.89	Q9JMH9	Unconventional myosin-XVIIIa	Myo18a	5512	51	51	51	30.8	30.8	30.8	232.75	0.0000	323.31	53586000	630	15.58	15.12	15.85	15.87	15.60	15.84	15.86	15.76
0.168	-0.16	0.89	Q8BH60	Golgi-associated PDZ and coiled-coil motif-containing protein	Gopc	3016	7	7	7	15.3	15.3	15.3	50.662	0.0000	8.7531	4394800	43	14.57	13.44	14.55	14.81	15.15	14.43	14.35	14.11
0.513	-0.17	0.89	Q91WD5	NADH dehydrogenase [ubiquinone] iron-sulfur protein 2, mitochondrial	Ndufs2	4106	15	15	15	44.3	44.3	44.3	52.625	0.0000	156.29	39572000	306	16.06	16.36	16.62	16.36	16.46	16.78	16.34	16.48
0.178	-0.17	0.89	O35737	Heterogeneous nuclear ribonucleoprotein H;Heterogeneous nuclear ribo	Hnrnph1	403	23	11	11	61	35.6	35.6	49.199	0.0000	86.829	26312000	178	15.62	17.09	15.61	16.44	16.21	16.37	16.49	16.34
0.185	-0.17	0.89	Q9Z1M4	Ribosomal protein S6 kinase beta-2	Rps6kb2	5813	4	4	4	8.7	8.7	8.7	53.538	0.0000	8.4627	1722200	28	13.73	13.29	13.02	12.36	12.81	13.75	13.10	13.39
0.345	-0.17	0.89	Q6PGC1	ATP-dependent RNA helicase Dhx29	Dhx29	2632	28	28	28	24.6	24.6	24.6	153.97	0.0000	144.06	28833000	305	15.69	15.50	16.08	15.89	15.61	15.94	15.88	16.40
0.331	-0.17	0.89	Q8K4Z3	NAD(P)H-hydrate epimerase	Apoa1bp	3730	9	9	9	33	33	33	30.972	0.0000	51.655	39102000	185	16.11	16.77	17.01	17.00	16.86	16.97	16.88	16.85
0.657	-0.17	0.89	Q9Z2M7;O3	Phosphomannomutase 2	Pmm2	5859	15	15	15	50	50	50	27.656	0.0000	166.2	83662000	351	18.03	18.04	18.28	18.27	18.16	18.61	18.23	18.28
0.403	-0.17	0.89	Q9DBC0	Selenoprotein O	Selo	5141	14	14	14	31.8	31.8	31.8	74.22	0.0000	70.554	17457000	135	15.06	14.71	14.67	14.86	15.34	14.57	15.07	15.00
0.109	-0.17	0.89	Q6NZQ2		Ddx31	2549	3	3	3	6.1	6.1	6.1	76.912	0.0004	4.7277	1027600	8	12.82	12.76	13.21	13.30	12.49	13.53	12.13	14.62
0.695	-0.17	0.89	Q80ZV0	Ribonuclease H2 subunit B	Rnaseh2b	2922	11	11	11	38	38	38	34.729	0.0000	53.459	11144000	174	15.33	15.33	15.22	15.37	15.33	15.73	15.25	15.62
0.118	-0.17	0.89	Q8CHK3	Lysophospholipid acyltransferase 7	Mboat7	3525	4	4	4	13.1	13.1	13.1	53.435	0.0000	26.5	2515800	20	13.76	12.88	11.98	13.64	12.81	12.91	14.28	12.94
0.480	-0.17	0.89	Q6A0D4	Raftlin	Rftn1	2502	25	25	25	51.3	51.3	51.3	61.536	0.0000	223.15	47251000	379	16.63	16.44	16.61	16.81	16.72	17.21	16.69	16.56
0.366	-0.17	0.89	Q61753	D-3-phosphoglycerate dehydrogenase	Phgdh	2332	30	30	30	47.5	47.5	47.5	56.585	0.0000	323.31	199310000	947	18.06	17.77	18.31	18.16	18.38	18.66	18.02	17.93
0.308	-0.17	0.89	Q9CWZ7	Gamma-soluble NSF attachment protein	Napg	4741	26	26	26	68.3	68.3	68.3	34.732	0.0000	204.46	53227000	470	16.10	16.68	16.10	16.60	16.25	16.46	16.41	17.05
0.285	-0.17	0.89	Q80WJ7	Protein LYRIC	Mtdh	2863	20	20	20	40.4	40.4	40.4	63.845	0.0000	323.31	95079000	620	17.12	17.11	17.50	17.60	17.06	18.09	17.33	17.54
0.324	-0.17	0.89	Q6KCD5	Nipped-B-like protein	Nipbl	2522	16	16	15	8	8	6.6	315.45	0.0000	32.677	10698000	74	15.27	15.13	15.14	15.67	14.99	15.40	15.87	15.66
0.377	-0.17	0.89	Q8BHS6	Armadillo repeat-containing X-linked protein 3	Armcx3	3051	10	10	10	39.1	39.1	39.1	42.619	0.0000	131.73	15000000	146	16.24	16.10	16.54	16.21	16.10	16.28	16.49	16.92
0.118	-0.17	0.89	P23298	Protein kinase C eta type	Prkch	882	4	4	4	7.5	7.5	7.5	77.918	0.0000	8.3028	2952600	44	12.19	13.85	14.12	14.19	14.57	13.37	13.78	13.32
1.365	-0.17	0.89	Q9WUA2	Phenylalanine--tRNA ligase beta subunit	Farsb	5702	32	32	32	60.1	60.1	60.1	65.696	0.0000	188.38	112990000	669	17.21	17.16	17.45	17.28	17.43	17.53	17.43	17.41
0.275	-0.18	0.89	P10649	Glutathione S-transferase Mu 1	Gstm1	727	39	39	25	91.3	91.3	52.8	25.97	0.0000	323.31	2009400000	2223	20.98	21.00	21.08	21.32	20.74	21.02	21.44	21.89
0.549	-0.18	0.89	Q8CI51	PDZ and LIM domain protein 5	Pdlim5	3540	26	26	26	49.7	49.7	49.7	63.299	0.0000	280.4	150810000	718	18.58	18.40	18.36	18.55	18.45	19.00	18.41	18.74
0.051	-0.18	0.88	P49769	Presenilin-1;Presenilin-1 NTF subunit;Presenilin-1 CTF subunit;Presenil	Psen1	1171	5	5	3	19.1	19.1	11.6	52.639	0.0000	21.104	8806200	72	13.94	12.55	16.46	16.45	15.61	12.81	16.13	15.56
0.358	-0.18	0.88	Q9CQA3	Succinate dehydrogenase [ubiquinone] iron-sulfur subunit, mitochondria	Sdhb	4578	19	19	19	46.8	46.8	46.8	31.814	0.0000	97.322	131240000	571	17.28	17.38	17.61	17.39	18.15	17.45	17.60	17.18
0.490	-0.18	0.88	Q99K90	TGF-beta-activated kinase 1 and MAP3K7-binding protein 2	Tab2	4367	9	9	9	21.9	21.9	21.9	76.441	0.0000	39.613	8694100	97	14.91	14.79	14.58	14.59	14.61	14.91	14.78	15.29
0.785	-0.18	0.88	O08749	Dihydrolipoyl dehydrogenase, mitochondrial	Dld	294	25	25	25	56.4	56.4	56.4	54.272	0.0000	322.61	147840000	701	17.99	17.75	17.87	18.01	17.98	17.97	18.01	18.36
0.303	-0.18	0.88	Q9CYA6	Zinc finger CCHC domain-containing protein 8	Zcchc8	4793	5	5	5	11.3	11.3	11.3	78.025	0.0000	19.989	4999900	51	13.91	14.35	14.51	14.32	15.03	14.21	14.50	14.06
0.527	-0.18	0.88	Q3UHJ0	AP2-associated protein kinase 1	Aak1	1974	16	16	15	30.9	30.9	30.2	103.35	0.0000	82.475	30755000	256	16.54	16.43	16.65	16.78	16.41	16.81	16.84	17.08
0.123	-0.18	0.88	Q9D4J7	PHD finger protein 6	Phf6	4980	4	4	4	8.8	8.8	8.8	41.139	0.0000	12.552	1292700	12	13.31	12.98	13.27	13.21	14.08	12.14	12.79	14.48
0.128	-0.18	0.88	Q8K215	LYR motif-containing protein 4	Lyrm4	3641	2	2	2	23.1	23.1	23.1	10.854	0.0009	3.689	4134200	26	15.06	14.59	12.94	15.23	14.39	14.64	14.90	14.61
0.757	-0.18	0.88	Q8BMA6	Signal recognition particle subunit SRP68	Srp68	3130	35	35	35	60.3	60.3	60.3	70.573	0.0000	323.31	88518000	650	16.89	16.76	16.89	17.00	17.09	16.76	17.24	17.18
0.162	-0.18	0.88	Q4VBD2	Transmembrane anterior posterior transformation protein 1	Tapt1	2070	4	4	4	10.8	10.8	10.8	63.893	0.0002	6.1564	1539400	6	13.58	12.95	13.54	13.41	13.46	13.38	12.71	14.66
0.283	-0.18	0.88	P15533	Tripartite motif-containing protein 30A	Trim30a	798	7	7	7	19.4	19.4	19.4	57.329	0.0000	21.242	2840000	41	14.14	13.59	13.35	13.01	13.76	14.03	13.43	13.60
0.191	-0.18	0.88	Q6PAV2	Probable E3 ubiquitin-protein ligase HERC4	Herc4	2600	19	19	19	23.7	23.7	23.7	118.41	0.0000	92.589	11425000	125	15.04	15.10	15.34	13.80	15.15	14.81	15.37	14.69
0.289	-0.18	0.88	Q9CQS2	H/ACA ribonucleoprotein complex subunit 3	Nop10	4627	5	5	5	87.5	87.5	87.5	7.7059	0.0000	37.971	8858700	72	15.89	15.12	14.76	14.87	15.42	15.51	15.16	15.30
0.994	-0.19	0.88	Q921M7	Protein FAM49B	Fam49b	4234	26	26	24	69.8	69.8	63	36.776	0.0000	323.31	292870000	845	19.32	19.24	19.45	19.37	19.61	19.67	19.29	19.56
0.877	-0.19	0.88	Q8K310	Matrin-3	Matr3	3685	44	44	44	54.6	54.6	54.6	94.629	0.0000	323.31	210680000	1065	18.45	18.28	18.44	18.67	18.54	18.67	18.53	18.84
0.566	-0.19	0.88	Q9D0M3	Cytochrome c1, heme protein, mitochondrial	Cyc1	4879	9	9	9	39.1	39.1	39.1	35.327	0.0000	62.855	45544000	201	17.33	17.06	17.28	17.32	17.71	17.05	17.57	17.40
0.198	-0.19	0.88	Q99LG0	Ubiquitin carboxyl-terminal hydrolase 16	Usp16	4426	5	5	5	8.6	8.6	8.6	93.433	0.0000	9.0463	3548200	21	14.00	13.23	14.93	13.69	14.11	14.34	13.90	14.25
0.303	-0.19	0.88	O89051	Integral membrane protein 2B;BRI2, membrane form;BRI2 intracellular (	Itm2b	598	5	5	5	18.8	18.8	18.8	30.26	0.0000	15.702	7780200	69	15.63	15.47	15.21	14.55	15.46	15.36	15.15	15.65
0.544	-0.19	0.88	Q3UND0	Src kinase-associated phosphoprotein 2	Skap2	2010	18	18	18	52	52	52	40.712	0.0000	174.66	59641000	325	16.81	17.04	16.89	16.67	17.28	17.29	16.79	16.80
0.599	-0.19	0.88	P97808	FXYD domain-containing ion transport regulator 5	Fxyd5	1674	5	5	5	38.2	38.2	38.2	19.454	0.0000	120.22	7360900	111	14.42	14.42	14.52	15.00	14.82	14.77	14.65	14.87
0.405	-0.19	0.88	Q8VDZ4;Q5	Palmitoyltransferase ZDHHC5	Zdhhc5	3977	4	4	4	7	7	7	77.5	0.0000	8.2244	1480600	21	13.17	13.27	13.89	13.07	13.78	13.38	13.42	13.56
0.413	-0.19	0.88	Q9DCX2	ATP synthase subunit d, mitochondrial	Atp5h	5236	19	19	19	91.3	91.3	91.3	18.749	0.0000	260.91	197080000	742	18.67	19.15	19.02	19.33	19.08	19.11	19.06	19.67
0.643	-0.19	0.88	Q8BX80	Cytosolic endo-beta-N-acetylglucosaminidase	Engase	3280	5	5	5	10.1	10.1	10.1	82.944	0.0000	17.144	8958000	97	14.73	14.95	15.28	15.08	14.96	15.21	15.30	15.32
0.245	-0.19	0.88	Q8CAK1	Putative transferase CAF17 homolog, mitochondrial	Iba57	3429	6	6	6	19.8	19.8	19.8	38.399	0.0000	22.326	4293800	46	14.47	14.03	14.66	14.85	14.11	14.60	15.38	14.68
0.280	-0.19	0.88	Q91ZA3	Propionyl-CoA carboxylase alpha chain, mitochondrial	Pcca	4192	48	48	48	69.5	69.5	69.5	79.921	0.0000	323.31	176390000	1070	17.41	16.89	17.56	17.29	17.76	17.06	18.02	17.08
0.112	-0.19	0.88	Q3UMU9	Hepatoma-derived growth factor-related protein 2	Hdgfrp2	2003	5	4	4	10.3	9.1	9.1	74.29	0.0000	29.467	2050200	40	12.43	12.62	11.85	14.40	12.14	13.35	13.09	13.49
0.527	-0.19	0.88	Q6P542	ATP-binding cassette sub-family F member 1	Abcf1	2567	32	32	31	44.4	44.4	44.4	94.944	0.0000	323.31	80225000	527	16.85	16.58	16.63	16.76	16.58	17.26	16.70	17.03
0.876	-0.19	0.88	P35293	Ras-related protein Rab-18	Rab18	1020	19	19	19	85	85	85	23.035	0.0000	173.38	72698000	418	16.66	16.91	17.13	16.89	16.97	17.03	17.20	17.15
0.156	-0.19	0.88	P56379	6.8 kDa mitochondrial proteolipid	Mp68	1281	2	2	2	34.5	34.5	34.5	6.6979	0.0009	3.7181	18471000	71	16.82	17.79	16.52	16.64	17.55	16.15	16.99	17.85
0.322	-0.19	0.87	Q6P9R2	Serine/threonine-protein kinase OSR1	Oxsr1	2592	9	9	9	28.1	28.1	28.1	58.213	0.0000	134.92	21560000	180	15.23	16.04	16.37	16.01	16.20	15.86	16.18	16.17
0.076	-0.19	0.87	Q8BXA1	Golgi integral membrane protein 4	Golim4	3283	5	5	5	8.1	8.1	8.1	76.784	0.0000	13.24	3736000	41	11.83	14.68	16.12	14.24	13.94	14.69	14.88	14.13
0.633	-0.19	0.87	Q3TDD9	Protein phosphatase 1 regulatory subunit 21	Ppp1r21	1831	41	41	41	67.1	67.1	67.1	88.337	0.0000	323.31	115580000	743	17.20	17.12	17.52	17.49	17.37	17.83	17.39	17.52
0.481	-0.19	0.87	G5E870	E3 ubiquitin-protein ligase TRIP12	Trip12	263	26	26	26	20.9	20.9	20.9	224.13	0.0000	323.31	39501000	462	16.17	15.87	16.01	16.15	15.75	16.49	16.30	16.44
0.457	-0.19	0.87	P57776	Elongation factor 1-delta	Eef1d	1299	16	16	15	56.2	56.2	56.2	31.293	0.0000	323.31	95706000	428	17.21	17.66	17.53	17.52	17.31	18.03	17.48	17.87
0.819	-0.19	0.87	P97868	E3 ubiquitin-protein ligase RBBP6	Rbbp6	1682	7	7	7	6.8	6.8	6.8	199.59	0.0000	24.638	4644400	79	14.11	14.10	14.12	14.25	14.24	14.22	14.22	14.68
1.507	-0.20	0.87	Q9WTM5	RuvB-like 2	Ruvbl2	5679	33	33	33	72.6	72.6	72.6	51.112	0.0000	323.31	113950000	676	16.95	17.13	17.19	17.24	17.30	17.40	17.29	17.30
0.149	-0.20	0.87	P20664	DNA primase small subunit	Prim1	857	6	6	6	16.5	16.5	16.5	49.295	0.0000	7.0226	1913800	15	13.26	13.08	14.47	13.42	13.95	13.79	12.69	14.58
1.143	-0.20	0.87	Q61233	Plastin-2	Lcp1	2282	73	73	67	92	92	90.7	70.148	0.0000	323.31	3779900000	5128	21.42	21.65	21.83	21.69	21.91	21.88	21.83	21.76
0.501	-0.20	0.87	Q8BIJ7	RUN and FYVE domain-containing protein 1	Rufy1	3062	34	34	31	47.2	47.2	46.2	80.376	0.0000	178.89	71223000	655	16.39	16.38	17.09	16.59	16.61	16.92	16.87	16.82
0.471	-0.20	0.87	Q8C2Q3	RNA-binding protein 14	Rbm14	3373	26	26	26	44.8	44.8	44.8	69.448	0.0000	202.91	120240000	536	17.66	17.61	17.16	17.61	17.56	18.07	17.40	17.80
0.131	-0.20	0.87	P35290	Ras-related protein Rab-24	Rab24	1019	7	7	7	43.8	43.8	43.8	23.144	0.0000	33.124	6871500	40	14.59	14.68	12.53	14.44	14.66	13.64	14.60	14.13
0.228	-0.20	0.87	Q8BM39	Pre-mRNA-splicing factor 18	Prpf18	3126	3	3	3	9.9	9.9	9.9	39.889	0.0002	5.9316	1410500	16	14.50	12.99	14.16	14.03	13.79	14.35	14.25	14.08
0.623	-0.20	0.87	Q9WTP7	GTP:AMP phosphotransferase AK3, mitochondrial	Ak3	5682	17	17	17	74	74	74	25.426	0.0000	153.61	63149000	424	16.82	16.79	17.10	16.81	16.74	17.28	17.00	17.30
0.428	-0.20	0.87	Q9D6M3	Mitochondrial glutamate carrier 1	Slc25a22	5003	11	11	9	38.1	38.1	31.6	34.67	0.0000	31.752	16268000	98	15.14	15.54	15.47	14.76	15.32	15.70	15.45	15.23
0.326	-0.20	0.87	Q7TSG2	RNA polymerase II subunit A C-terminal domain phosphatase	Ctdp1	2782	9	9	9	17.3	17.3	17.3	104.55	0.0000	72.164	6429000	92	14.86	13.96	13.86	14.06	14.59	14.53	14.05	14.36
0.247	-0.20	0.87	Q80WW9	DDRGK domain-containing protein 1	Ddrgk1	2867	9	9	9	44.8	44.8	44.8	35.977	0.0000	64.104	6706700	119	14.47	14.15	15.23	15.27	14.79	14.61	15.09	15.43
0.067	-0.20	0.87	P40201	Chromodomain-helicase-DNA-binding protein 1	Chd1	1058	7	7	6	5.8	5.8	5.3	196.38	0.0000	8.4545	7186400	33	16.36	13.07	16.25	16.82	15.79	13.97	16.76	16.78
0.181	-0.20	0.87	Q6PIJ4	Nuclear factor related to kappa-B-binding protein	Nfrkb	2645	12	12	12	14.5	14.5	14.5	138.76	0.0000	50.446	7526700	85	14.23	15.03	14.44	15.11	13.86	15.51	14.82	15.43
0.758	-0.20	0.87	Q9DAS9	Guanine nucleotide-binding protein G(I)/G(S)/G(O) subunit gamma-12	Gng12	5110	6	6	6	70.8	70.8	70.8	7.9972	0.0000	61.019	100570000	318	18.31	18.58	18.53	18.70	18.45	18.90	18.88	18.71
0.155	-0.20	0.87	Q99JW2	Aminoacylase-1	Acy1	4351	5	5	5	22.3	22.3	22.3	45.78	0.0000	38.191	1994000	23	12.48	14.23	14.51	13.47	13.95	13.23	14.15	14.18
0.555	-0.20	0.87	Q60875	Rho guanine nucleotide exchange factor 2	Arhgef2	2219	37	37	37	45.3	45.3	45.3	111.97	0.0000	310.11	113950000	661	17.80	18.01	18.54	18.16	18.20	18.39	18.50	18.23
0.473	-0.21	0.87	Q9WUR9	Adenylate kinase 4, mitochondrial	Ak4	5722	8	8	8	56.5	56.5	56.5	25.061	0.0000	17.166	6011500	64	14.37	14.64	14.87	15.17	14.90	15.26	14.85	14.85

0.128	-0.21	0.87	P62748;P84	Hippocalcin-like protein 1;Neuron-specific calcium-binding protein hippo	Hpcal1;Hpca	1441	8	8	8	42.5	42.5	42.5	42.5	22.338	0.0000	18.691	6926200	68	14.74	15.87	15.23	15.22	15.82	13.91	15.60	16.57
0.625	-0.21	0.87	P61327;Q9C	Protein mago nashi homolog;Protein mago nashi homolog 2	Magoh;Magohb	1383	10	10	10	66.4	66.4	66.4	66.4	17.163	0.0000	69.555	52310000	203	17.50	17.34	17.66	17.55	17.47	17.98	17.47	17.95
0.594	-0.21	0.87	P29351	Tyrosine-protein phosphatase non-receptor type 6	Ptpn6	966	61	61	61	85.2	85.2	85.2	85.2	67.558	0.0000	323.31	831080000	1950	19.96	19.99	19.74	19.90	20.07	20.51	19.76	20.08
0.291	-0.21	0.87	P58058	NAD kinase	Nadk	1305	16	16	16	28	28	28	28	48.597	0.0000	74.454	43506000	279	17.23	17.25	17.24	16.16	17.02	17.48	16.91	17.30
0.301	-0.21	0.86	Q62419;Q62	Endophilin-A2	Sh3gl1	2388	25	25	25	58.2	58.2	58.2	58.2	41.518	0.0000	198.71	74183000	411	16.73	17.28	16.31	17.25	17.04	17.16	16.67	17.55
0.618	-0.21	0.86	Q61033	Lamina-associated polypeptide 2, isoforms alpha/zeta	Tmpo	2240	31	31	18	65.5	65.5	65.5	44.6	75.167	0.0000	323.31	134020000	714	18.09	18.14	18.10	18.37	18.20	18.48	18.10	18.75
0.409	-0.21	0.86	Q5NCR9	Nuclear speckle splicing regulatory protein 1	Nsrp1	2119	6	6	6	13.1	13.1	13.1	13.1	63.798	0.0000	20.8	3044100	19	13.04	13.34	12.68	13.52	13.15	13.58	13.59	13.10
0.123	-0.21	0.86	Q6ZWY9;Q6	Histone H2B type 1-C/E/G;Histone H2B type 1-H	Hist1h2bc;Hist1h2	2712	27	2	0	96	96	17.5	0	13.906	0.0000	142.82	102030000	57	20.29	20.27	18.63	18.21	20.45	18.85	19.72	19.24
0.623	-0.21	0.86	Q9WUM5	Succinyl-CoA ligase [ADP/GDP-forming] subunit alpha, mitochondrial	Suclg1	5717	15	15	15	38.2	38.2	38.2	38.2	36.154	0.0000	233.69	140850000	632	18.12	17.74	17.48	17.81	17.81	18.23	17.89	18.06
0.222	-0.21	0.86	Q9QXH4	Integrin alpha-X	Itgax	5542	12	12	11	12.1	12.1	12.1	11.2	129.15	0.0000	30.485	5563800	57	14.09	13.42	14.38	14.02	13.86	13.43	14.78	14.69
0.444	-0.21	0.86	P97789	5-3 exoribonuclease 1	Xrn1	1670	16	16	16	12.7	12.7	12.7	12.7	194.31	0.0000	42.743	12956000	114	15.61	15.34	15.14	15.10	15.34	15.45	15.21	16.04
0.992	-0.22	0.86	Q9CU62	Structural maintenance of chromosomes protein 1A	Smc1a	4704	78	78	78	58.9	58.9	58.9	58.9	143.23	0.0000	323.31	226530000	1423	18.27	18.04	18.27	18.39	18.36	18.69	18.31	18.47
0.463	-0.22	0.86	Q99J62	Replication factor C subunit 4	Rfc4	4327	11	11	11	29.7	29.7	29.7	29.7	39.866	0.0000	42.8	13326000	117	15.29	15.18	15.94	15.86	15.62	16.01	15.76	15.74
1.166	-0.22	0.86	Q8BYW1	Rho GTPase-activating protein 25	Arhgap25	3307	40	40	39	78.1	78.1	78.1	78.1	73.382	0.0000	323.31	87090000	671	16.91	17.02	16.92	17.22	17.12	17.24	17.16	17.42
0.879	-0.22	0.86	Q8BJW6	Eukaryotic translation initiation factor 2A;Eukaryotic translation initiation	Eif2a	3089	30	30	30	64	64	64	64	64.403	0.0000	323.31	108980000	728	17.34	17.28	17.34	17.16	17.31	17.76	17.29	17.62
0.289	-0.22	0.86	O08539	Myc box-dependent-interacting protein 1	Bin1	269	21	19	19	40	40	40	40	64.469	0.0000	206.57	51028000	447	16.32	16.81	15.90	16.79	16.53	16.46	16.35	17.34
0.166	-0.22	0.86	Q922R1	UPF0183 protein C16orf70 homolog		4274	4	4	4	16.4	16.4	16.4	16.4	47.416	0.0000	14.457	2724500	35	14.55	14.00	12.52	13.78	13.16	14.00	14.13	14.43
0.423	-0.22	0.86	Q7TSI3	Serine/threonine-protein phosphatase 6 regulatory subunit 1	Ppp6r1	2786	12	12	12	27.7	27.7	27.7	27.7	94.526	0.0000	88.073	8551900	97	14.20	14.00	14.94	14.56	14.40	14.56	14.82	14.79
0.733	-0.22	0.86	Q99KN9	Clathrin interactor 1	Clint1	4386	21	21	21	34.5	34.5	34.5	34.5	68.512	0.0000	135.26	50202000	354	16.84	16.80	16.48	17.04	16.76	17.04	17.04	17.19
0.353	-0.22	0.86	Q9D0R4	Probable ATP-dependent RNA helicase DDX56	Ddx56	4884	8	8	8	24.5	24.5	24.5	24.5	61.211	0.0000	36.58	8928200	80	14.60	14.41	15.16	15.00	14.44	15.03	15.17	15.42
0.393	-0.22	0.86	Q9D0S9	Histidine triad nucleotide-binding protein 2, mitochondrial	Hint2	4886	6	6	6	47.9	47.9	47.9	47.9	17.32	0.0000	56.729	18991000	185	15.94	15.98	15.78	15.93	15.84	15.65	16.30	16.74
1.388	-0.22	0.86	Q99JR8;Q6I	SWI/SNF-related matrix-associated actin-dependent regulator of chrom	Smarcd2	4346	18	18	16	42.4	42.4	42.4	38.8	59.084	0.0000	99.723	34673000	252	16.10	16.11	15.98	16.08	16.13	16.50	16.20	16.32
0.482	-0.22	0.86	O70378	ER membrane protein complex subunit 8	Emc8	492	8	8	8	52.2	52.2	52.2	52.2	23.348	0.0000	106.12	33064000	284	16.14	16.48	16.30	16.80	16.26	16.56	16.83	16.98
0.561	-0.23	0.86	P27659	60S ribosomal protein L3	Rpl3	938	45	45	45	76.2	76.2	76.2	76.2	46.109	0.0000	323.31	545920000	1621	19.62	19.25	19.11	19.36	19.26	19.91	19.35	19.73
0.734	-0.23	0.86	Q05144	Ras-related C3 botulinum toxin substrate 2	Rac2	1739	19	18	9	63.5	63.5	63.5	32.3	21.441	0.0000	87.717	539890000	761	19.49	19.45	19.99	19.73	19.65	20.03	19.97	19.92
0.383	-0.23	0.86	A2AUY4		Baz2b	41	4	4	4	3.3	3.3	3.3	3.3	234	0.0000	21.261	2469400	41	13.87	13.52	14.53	14.04	13.78	14.39	14.41	14.29
0.609	-0.23	0.85	Q60787	Lymphocyte cytosolic protein 2	Lcp2	2207	12	12	12	23.5	23.5	23.5	23.5	60.238	0.0000	32.264	24418000	189	16.27	16.08	15.76	16.51	16.43	16.35	16.19	16.56
0.122	-0.23	0.85	Q8C5L7	RNA-binding protein 34	Rbm34	3398	4	4	4	13.1	13.1	13.1	13.1	49.116	0.0000	11.03	2300500	17	12.98	14.66	13.23	14.43	13.93	15.68	13.36	13.25
0.216	-0.23	0.85	Q921N6	Probable ATP-dependent RNA helicase DDX27	Ddx27	4235	8	8	8	14.2	14.2	14.2	14.2	85.938	0.0000	9.8902	7326800	80	15.65	15.34	14.59	16.32	15.25	16.30	15.48	15.79
0.767	-0.23	0.85	Q61937	Nucleophosmin	Npm1	2343	17	17	16	59.9	59.9	59.9	51.7	32.56	0.0000	323.31	88123000	399	18.15	18.54	17.99	18.12	18.36	18.60	18.21	18.55
0.329	-0.23	0.85	Q9R1R2	Tripartite motif-containing protein 3	Trim3	5660	7	7	7	16	16	16	16	80.774	0.0000	29.52	5068100	44	13.70	13.94	14.46	14.23	15.05	14.18	14.03	14.00
0.694	-0.23	0.85	O70194	Eukaryotic translation initiation factor 3 subunit D	Eif3d	476	27	27	27	56.6	56.6	56.6	56.6	63.988	0.0000	323.31	251540000	1203	18.17	18.07	18.07	18.23	18.27	18.77	18.02	18.41
0.331	-0.23	0.85	Q8CI08	SLAIN motif-containing protein 2	Slain2	3536	10	10	9	20.3	20.3	20.3	20.3	62.377	0.0000	19.523	4670700	23	13.11	13.97	14.16	13.60	14.48	13.87	13.59	13.81
0.517	-0.23	0.85	P61087	Ubiquitin-conjugating enzyme E2 K	Ube2k	1370	17	17	17	79.5	79.5	79.5	79.5	22.406	0.0000	93.675	39018000	283	16.65	16.16	16.22	16.54	16.50	16.72	16.24	17.04
0.559	-0.23	0.85	Q91WQ3	Tyrosine--tRNA ligase, cytoplasmic;Tyrosine--tRNA ligase, cytoplasmic,	Yars	4123	35	35	35	67.8	67.8	67.8	67.8	59.105	0.0000	239.69	122400000	767	17.90	17.92	17.39	18.15	17.86	18.06	18.00	18.39
0.917	-0.23	0.85	P23116	Eukaryotic translation initiation factor 3 subunit A	Eif3a	879	99	99	99	58.6	58.6	58.6	58.6	161.93	0.0000	323.31	376990000	2049	17.53	17.44	17.67	17.67	17.46	17.88	17.94	17.95
0.438	-0.23	0.85	Q9D2M8	Ubiquitin-conjugating enzyme E2 variant 2	Ube2v2	4945	11	5	5	78.6	78.6	44.8	44.8	16.367	0.0000	85.942	39002000	191	17.23	17.34	18.23	17.65	17.61	17.92	17.95	17.92
0.305	-0.23	0.85	Q8BH02	Torsin-4A	Tor4a	3004	12	12	12	39	39	39	39	47.585	0.0000	146.44	22049000	206	17.02	16.43	15.71	16.02	16.92	16.63	16.37	16.20
0.630	-0.24	0.85	Q8VCN9	Tubulin-specific chaperone C	Tbcc	3929	8	8	8	38.7	38.7	38.7	38.7	38.124	0.0000	57.772	20628000	200	15.87	15.99	16.49	16.50	16.56	16.54	16.42	16.27
0.774	-0.24	0.85	Q8BL80	Rho GTPase-activating protein 22	Arhgap22	3112	23	23	23	40.6	40.6	40.6	40.6	77.785	0.0000	113.74	44078000	382	16.23	15.70	16.27	16.23	16.34	16.47	16.17	16.41
0.202	-0.24	0.85	Q8CGA3	Large neutral amino acids transporter small subunit 4	Slc43a2	3503	8	8	8	10.7	10.7	10.7	10.7	62.43	0.0000	38.472	13643000	149	15.65	15.21	15.51	13.79	15.44	14.98	15.73	14.96
0.732	-0.24	0.85	Q62203	Splicing factor 3A subunit 2	Sf3a2	2369	11	11	11	27.8	27.8	27.8	27.8	49.911	0.0000	104.2	24505000	204	15.91	15.53	15.65	16.13	15.88	16.27	15.95	16.09
0.878	-0.24	0.85	P28741	Kinesin-like protein KIF3A	Kif3a	961	17	16	14	33.1	33.1	31.2	28.1	80.169	0.0000	169.48	24991000	237	15.91	15.89	16.11	16.06	15.94	16.28	16.17	16.55
0.413	-0.24	0.85	P62313	U6 snRNA-associated Sm-like protein LSm6	Lsm6	1421	7	7	7	50	50	50	50	9.1275	0.0000	10.338	10196000	118	15.36	15.02	15.06	14.73	14.97	15.05	15.17	15.94
0.358	-0.24	0.85	Q8BXC6	COMM domain-containing protein 2	Commd2	3285	5	5	5	30.7	30.7	30.7	30.7	22.848	0.0000	21.074	6298800	100	14.72	14.50	14.29	15.36	14.68	15.34	14.64	15.19
0.798	-0.24	0.85	Q9CR16	Peptidyl-prolyl cis-trans isomerase D	Ppid	4658	23	22	22	61.1	61.1	59.2	59.2	40.742	0.0000	149.69	82121000	622	17.50	17.19	17.80	17.70	17.65	17.77	17.76	17.98
0.393	-0.24	0.85	Q8K2Q9	Shootin-1	Kiaa1598	3669	30	30	30	44.7	44.7	44.7	44.7	71.342	0.0000	115.59	37527000	308	15.53	16.09	15.30	16.43	16.23	15.84	16.08	16.18
0.783	-0.24	0.85	Q8R4R6	Nucleoporin NUP53	Nup35	3872	13	13	13	67.4	67.4	67.4	67.4	34.785	0.0000	87.458	30110000	224	16.17	16.20	16.48	16.79	16.51	16.70	16.69	16.72
0.336	-0.24	0.84	Q8CJF7	Protein ELYS	Ahctf1	3569	10	10	10	6.2	6.2	6.2	6.2	247.64	0.0000	12.647	2849500	25	12.72	13.39	13.67	12.45	13.00	13.58	13.40	13.22
0.390	-0.24	0.84	Q9JI75;P16(	Ribosyldihydronicotinamide dehydrogenase [quinone]	Nqo2	5389	12	12	12	41.1	41.1	41.1	41.1	26.248	0.0000	77.31	27001000	228	16.69	16.12	16.13	15.70	16.21	16.51	16.04	16.86
0.349	-0.24	0.84	Q5U5Q9	BRCA1-A complex subunit RAP80	Uimc1	2168	5	5	5	8.1	8.1	8.1	8.1	81.477	0.0000	12.979	1269700	13	11.83	12.14	13.00	11.84	12.48	12.74	12.19	12.37
0.450	-0.24	0.84	P18572	Basigin	Bsg	837	4	4	4	12.9	12.9	12.9	12.9	42.444	0.0000	8.3718	3665000	42	14.01	14.60	14.76	13.92	14.47	14.90	14.58	14.32
0.165	-0.25	0.84	Q9R269	Periplakin	Ppl	5672	12	12	12	7.3	7.3	7.3	7.3	204	0.0000	17.136	4428400	29	14.24	11.99	13.88	13.69	14.35	13.43	13.96	13.04
0.672	-0.25	0.84	Q8C2K5	RAS protein activator like-3	Rasal3	3372	13	13	13	18.9	18.9	18.9	18.9	114.78	0.0000	45.547	15579000	143	15.57	15.25	15.60	15.32	15.57	16.04	15.80	15.32
0.207	-0.25	0.84	Q8CFV9	Riboflavin kinase	Rfk	3492	4	4	4	42.6	42.6	42.6	42.6	17.437	0.0044	2.4028	2324900	11	14.25	15.10	13.03	13.64	14.17	14.26	13.85	14.73
0.838	-0.25	0.84	O08579	Emerin	Emd	274	8	8	8	45.9	45.9	45.9	45.9	29.435	0.0000	147.67	48716000	319	16.82	17.12	17.23	16.92	17.10	17.60	17.10	17.28
0.602	-0.25	0.84	Q61771	Kinesin-like protein KIF3B;Kinesin-like protein KIF3B, N-terminally proce	Kif3b	2334	29	29	24	42.8	42.8	42.8	36.5	85.287	0.0000	203.21	48075000	443	16.72	16.56	16.34	16.52	16.51	17.05	16.44	17.14
0.597	-0.25	0.84	Q6Y7W8	PERQ amino acid-rich with GYF domain-containing protein 2	Gigyf2	2672	16	16	16	17.7	17.7	17.7	17.7	149.19	0.0000	193.38	28645000	255	17.09	17.10	17.11	17.55	17.21	17.30	17.40	17.94
0.269	-0.25	0.84	Q91XB0	Three-prime repair exonuclease 1	Trex1	4139	13	13	13	52.2	52.2	52.2	52.2	33.675	0.0000	120.89	31379000	194	16.67	16.16	15.85	15.53	16.56	17.01	15.97	15.67
0.845	-0.25	0.84	Q9D6R2	Isocitrate dehydrogenase [NAD] subunit alpha, mitochondrial	Idh3a	5007	21	21	21	51.6	51.6	51.6	51.6	39.638	0.0000	182.15	90883000	611	16.83	17.31	17.11	17.48	17.43	17.41	17.33	17.57
0.902	-0.25	0.84	Q9QUI0	Transforming protein RhoA	Rhoa	5514	18	18	10	67.9	67.9	67.9	39.9	21.782	0.0000	239.34	492120000	1176	19.34	19.21	18.92	19.29	19.27	19.35	19.39	19.75
0.262	-0.25	0.84	Q8BH69	Selenide, water dikinase 1	Sephs1	3019	11	11	11	46.2	46.2	46.2	46.2	42.906	0.0000	217.69	41540000	220	17.38	17.62	17.95	17.57	18.25	18.29	18.24	16.75
0.355	-0.25	0.84	Q61739	Integrin alpha-6;Integrin alpha-6 heavy chain;Integrin alpha-6 light chain	Itga6	2330	9	9	9	12	12	12	12	122.16	0.0000	20.508	13071000	100	15.10	15.60	15.94	16.40	16.25	16.23	15.88	15.70
0.706	-0.25	0.84	Q99J77		Nans	4330	25	25	25	73	73	73	73	40.024	0.0000	272.46	127790000	640	17.83	17.83	17.67	17.51	18.05	18.24	18.05	17.51
0.908	-0.25	0.84	P56477	Interferon regulatory factor 5	Irf5	1287	16	16	16	43.5	43.5	43.5	43.5	56.004	0.0000	80.705	33576000	293	15.84	16.18	15.97	16.09	16.06	16.20	16.23	16.62
0.545	-0.25	0.84	Q9CPR5	39S ribosomal protein L15, mitochondrial	Mrpl15	4530	16	16	16	66.1	66.1	66.1	66.1	33.541	0.0000	125.63	35081000	329	16.07	15.89	16.18	16.01	15.81	16.55	16.09	16.72
0.115	-0.26	0.84	Q64112	Interferon-induced protein with tetratricopeptide repeats 2	Ifit2	2418	15	15	15	36.7	36.7	36.7	36.7	55.02	0.0000	208.33	22477000	141	15.08	15.47	13.51	13.03	16.13	14.56	13.77	13.66
0.867	-0.26	0.84	Q8K400	Syntaxin-binding protein 5	Stxbp5	3709	15	15	15	22	22	22	22	127.65	0.0000	96.131	35062000	264	16.41	16.48	16.64	16.14	16.55	16.52	16.64	16.98
0.510	-0.26	0.84	Q9EP71	Ankycorbin	Rai14	5244	13	13	13	20.1	20.1	20.1	20.1	108.85	0.0000	40.103	8994700	121	14.43	14.31	14.26	14.31	14.60	13.94	14.81	14.98
1.500	-0.26	0.84	Q9WUM4	Coronin-1C	Coro1c	5716	46	46	41	67.5	67.5	67.5	61.8	53.12	0.0000	323.31	547230000	1473	19.18	19.09	19.15	19.42	19.49	19.36	19.40	19.62
0.470	-0.26	0.84	Q99N84	28S ribosomal protein S18b, mitochondrial	Mrps18b	4485	3	3	3	24.8	24.8	24.8	24.8	28.702	0.0000	26.142	4572200	48	14.24	14.75	14.18	14.94	14.79	14.36	14.84	15.15
0.357	-0.26	0.84	P28658	Ataxin-10	Atxn10	955	9	9	9	17.1	17.1	17.1	17.1	53.706	0.0000	111.76	15974000	83	15.65	15.83	17.03	15.98	16.39	16.42	16.43	16.27
0.430	-0.26	0.84	P62141	Serine/threonine-protein phosphatase PP1-beta catalytic subunit	Ppp1cb	1405	19	5	5	53.5	53.5	17.1	17.1	37.186	0.0000	36.361	25667000	155	17.11	16.16	16.65	16.19	16.50	17.19	16.81	16.65
0.745	-0.26	0.83	P70362	Ubiquitin fusion degradation protein 1 homolog	Ufd1l	1560	13	13	13	45.9	45.9	45.9	45.9	34.481	0.0000	48.029	38201000	208	16.45	16.62	16.92	16.58	16.71	17.31	16.89	16.70
0.590	-0.26	0.83	P49446	Receptor-type tyrosine-protein phosphatase epsilon	Ptpre	1164	18	18	17	36.1	36.1	36.1	33.5	80.687	0.0000	130.12	22479000	258	14.98	15.92	15.55	15.65	15.61	15.77	15.83	15.94
1.726	-0.26	0.83	Q8BJZ4	28S ribosomal protein S35, mitochondrial	Mrps35	3091	9	9	9	36.2	36.2	36.2	36.2	35.975	0.0000	84.356	15751000	119	15.97	15.87	15.97	15.84	16.00	16.36	16.20	16.13
0.378	-0.26	0.83	P0DOV2			715	42	42	25	70.4	70.4	70.4	44.6	69.437	0.0000	323.31	196010000	939	19.12	18.82	18.14	18.10	18.96	19.21	18.54	18.52
0.375	-0.26	0.83	Q9CQV6	Microtubule-associated proteins 1A/1B light chain 3B	Map1lc3b	4639	3	3	3	29.6	29.6	29.6	29.6	14.617	0.0002	5.3047	5187800	56	14.24	14.71	15.03	14.48	14.99	15.08	15.30	14.14
0.827	-0.26	0.83	Q9D1J1	Adaptin ear-binding coat-associated protein 2	Necap2	4920	9	9	9	47	47	47	47	28.598	0.0000	209.18	34332000	279	16.01	16.48	16.65	16.29	16.70	16.78	16.60	16.41
0.944	-0.26	0.83	Q6NZJ6	Eukaryotic translation initiation factor 4 gamma 1	Eif4g1	2546	47	47	43	33.4	33.4	33.4	30.6	176.07	0.0000	295.24	152010000	1012	18.01	17.95	18.19	18.31	18.09	18.59	18.29	18.55
0.495	-0.27	0.83	Q9Z315	U4/U6.U5 tri-snRNP-associated protein 1	Sart1	5876	35	35	35	44.3	44.3	44.3	44.3	90.884	0.0000	235.16	28588000	243	15.24	15.42	15.38	15.90	15.18	16.07	15.76	15.99
1.009	-0.27	0.83	P27773	Protein disulfide-isomerase A3	Pdia3	940	59	59	59	73.3	73.3	73.3	73.3	56.678	0.0000	323.31	1713800000	2793	20.36	20.86	20.64	20.82	20.98	20.91	20.75	21.10
0.915	-0.27	0.83	Q9D358	Low molecular weight phosphotyrosine protein phosphatase	Acp1	4959	8	8	8	42.4	42.4	42.4	42.4	18.192	0.0000	82.917	22304000	172	16.34	16.30	16.40	16.34	16.50	16.58	16.35	17.03
0.177	-0.27	0.83	Q9JL16	Interferon-stimulated gene 20 kDa protein	Isg20	5467	3	3	3	13.7	13.7	13.7	13.7	32.503	0.0000	26.807	5198800	48	15.27	14.18	13.38	13.76	15.60	14.39	14.08	13.60
1.158	-0.27	0.83	P60867	40S ribosomal protein S20	Rps20	1363	13	13	13	79.8	79.8	79.8	79.8	13.373	0.0000	235.24	422050000	909	19.56	19.48	19.73	19.88	19.73	19.86	20.08	20.06
0.290	-0.27	0.83	Q9JIA7	Sphingosine kinase 2	Sphk2	5393	14	14	14	38.2	38.2	38.2	38.2	65.617	0.0000	172.7	23017000	201	16.85	16.04	15.98	14.97	16.21	16.09	16.35	16.26
0.502	-0.27	0.83	Q8K1E0	Syntaxin-5	Stx5	3625	11	11	11	44.2	44.2	44.2	44.2	39.713	0.0000	93.409	30866000	204	16.25	16.11	17.11	16.67	16.54	17.02	16.78	16.87
0.126	-0.27	0.83	Q5NBU8	XIAP-associated factor 1	Xaf1	2115	8	8	8	43.2	43.2	43.2	43.2	31.117	0.0000	27.147	10111000	91	14.51	14.81	13.74	14.66	15.23	16.64	13.60	13.34
0.242	-0.27	0.83	Q9Z0R9	Fatty acid desaturase 2	Fads2	5783	4	4	4	11.9	11.9	11.9	11.9	52.387	0.0000	6.9898	1395200	15	13.33	11.77	12.50	12.77	13.48	13.13	12.84	11.99
0.425	-0.27	0.83	Q03958	Prefoldin subunit 6	Pfdn6	1728	6	6	6	50.4	50.4	50.4	50.4	14.454	0.0000	15.749	4731600	56	13.07	14.12	13.36	13.57	13.88	13.84	13.32	14.17
0.175	-0.27	0.83	Q4KML4	Costars family protein ABRACL	Abracl	2061	3	3	3	46.9	46.9	46.9	46.9	9.0303	0.0000	29.25	6622700	76	15.53	16.25	14.84	15.81	15.75	14.44	16.70	16.63
0.672	-0.27	0.83	Q922P9	Putative oxidoreductase GLYR1	Glyr1	4266	13	13	13	31.5	31.5	31.5	31.5	59.715	0.0000	121.08	32777000	416	15.98	15.81	16.02	16.02	16.14	16.78	15.92	16.07
0.727	-0.27	0.83	O35226	26S proteasome non-ATPase regulatory subunit 4	Psmd4	348	10	10	10	40.7	40.7	40.7	40.7	40.703	0.0000	135.92	49883000	391	16.41	16.46	16.82	17.06	16.70	16.94	17.17	17.02
0.585	-0.28	0.83	Q9WTK7	Serine/threonine-protein kinase STK11	Stk11	5677	3	3	3	11.5	11.5	11.5	11.5	49.266	0.0000	14.194	1785100	31	12.97	13.42	13.41	13.46	13.57	13.07	13.90	13.83
1.595	-0.28	0.83	P59729	Ras and Rab interactor 3	Rin3	1342	7	7	6	11.9	11.9	11.9	10.5	107.22	0.0000	46.128	7546600	124	14.74	14.56	14.86	14.88	14.86	15.08	15.08	15.11
0.274	-0.28	0.82	B2RRE7	OTU domain-containing protein 4	Otud4	79	7	7	6	9.4	9.4	9.4	6.7	123.05	0.0000	16.01	3997700	71	13.62	14.03	12.96	14.75	13.85	14.54	13.74	14.34
0.647	-0.28	0.82	P62331	ADP-ribosylation factor 6	Arf6	1426	10	10	10	66.9	66.9	66.9	66.9	20.082	0.0000	156.51	40720000	299	16.74	16.85	16.44	16.70	17.10	16.72	16.60	17.42
0.871	-0.28	0.82	Q3UYC0;Q1	Protein phosphatase 1H	Ppm1h	2043	18	18	17	42.3	42.3	42.3	40.4	56.379	0.0000	56.925	22753000	187	15.68	15.23	15.61	15.81	15.76	15.62	16.03	16.04
1.052	-0.28	0.82	O88746	Target of Myb protein 1	Tom1	565	18	18	18	60	60	60	60	54.325	0.0000	323.31	39894000	383	16.46	16.29	16.85	16.79	16.79	16.92	16.92	16.88
0.534	-0.28	0.82	Q8VC70	RNA-binding motif, single-stranded-interacting protein 2	Rbms2	3911	3	2	2	11	11	9.1	9.1	40.724	0.0008	4.1985	1013100	15	12.96	13.45	13.51	13.33	13.09	13.84	13.43	14.02
0.979	-0.28	0.82	Q6PDL0	Cytoplasmic dynein 1 light intermediate chain 2	Dync1li2	2616	16	15	15	50.8	50.8	48.2	48.2	54.218	0.0000	190.58	37032000	289	16.49	16.35	16.63	16.32	16.46	16.65	16.73	17.08
0.625	-0.28	0.82	P68037	Ubiquitin-conjugating enzyme E2 L3	Ube2l3	1512	20	20	20	89.6	89.6	89.6	89.6	17.861	0.0000	239.7	94728000	369	17.83	18.56	17.66	18.15	18.08	18.46	18.39	18.39
0.443	-0.28	0.82	Q9D187	Mitotic spindle-associated MMXD complex subunit MIP18	Fam96b	4901	6	6	6	57.1	57.1	57.1	57.1	17.667	0.0000	92.65	8879700	133	14.75	14.79	15.52	14.76	15.12	15.71	15.41	14.70
0.613	-0.28	0.82	P47962	60S ribosomal protein L5	Rpl5	1134	35	35	35	67.7	67.7	67.7	67.7	34.4	0.0000	323.31	459640000	1555	19.05	18.82	18.67	19.07	18.80	19.55	18.89	19.50
1.004	-0.28	0.82	Q6A026	Sister chromatid cohesion protein PDS5 homolog A	Pds5a	2495	33	33	33	37.5	37.5	37.5	37.5	150.33	0.0000	323.01	68400000	663	16.36	16.33	16.72	16.67	16.50	16.93	16.94	16.84
0.278	-0.28	0.82	Q8R322	Nucleoporin GLE1	Gle1	3831	6	6	6	12	12	12	12	79.573	0.0000	13.241	5486100	30	14.58	15.23	15.08	14.46	15.47	15.59	15.43	13.98
0.302	-0.28	0.82	Q99J23	GH3 domain-containing protein	Ghdc	4318	2	2	2	4.3	4.3	4.3	4.3	58.507	0.0004	4.5681	539010	7	13.52	12.85	12.50	12.30	12.76	12.47	13.31	13.77
0.199	-0.28	0.82	Q9CR67	Transmembrane protein 33	Tmem33	4676	3	3	3	15.4	15.4	15.4	15.4	28.031	0.0000	9.9279	17466000	30	17.80	17.04	15.87	15.37	17.14	16.61	16.60	16.88
0.425	-0.28	0.82	P97379	Ras GTPase-activating protein-binding protein 2	G3bp2	1640	15	12	12	33	33	29.5	29.5	54.087	0.0000	60.342	20168000	221	15.36	16.30	15.42	15.91	15.77	15.70	16.06	16.58
0.376	-0.29	0.82	Q15843;P29	NEDD8	NEDD8;Nedd8	971	5	5	5	71.6	71.6	71.6	71.6	9.0714	0.0000	79.674	55180000	197	17.99	18.41	17.00	18.21	17.93	18.09	18.33	18.40
0.263	-0.29	0.82	Q03267	DNA-binding protein Ikaros	Ikzf1	1725	5	5	5	12.4	12.4	12.4	12.4	57.336	0.0000	11.083	10943000	36	17.05	15.76	16.57	15.40	16.74	17.03	16.27	15.90
1.205	-0.29	0.82	Q99MR6	Serrate RNA effector molecule homolog	Srrt	4474	46	46	46	44.2	44.2	44.2	44.2	100.45	0.0000	258.49	79269000	631	17.11	17.39	17.41	17.44	17.52	17.65	17.43	17.91
0.700	-0.29	0.82	Q6NXI6	Regulation of nuclear pre-mRNA domain-containing protein 2	Rprd2	2538	14	14	14	15	15	15	15	156.58	0.0000	103.42	12626000	204	14.35	14.61	14.64	14.84	14.69	15.12	14.52	15.26
0.359	-0.29	0.82	Q8VCA8	Secernin-2	Scrn2	3912	3	3	3	14.6	14.6	14.6	14.6	46.6	0.0000	9.6669	954290	11	13.14	12.62	13.54	13.96	13.11	13.73	14.08	13.52

0.154	-0.29	0.82	Q8R4X3	RNA-binding protein 12	Rbm12	3875	5	5	5	6.8	6.8	6.8	102.8	0.0002	6.01	1907100	30	13.80	14.68	11.54	14.12	13.99	13.75	13.23	14.35
0.196	-0.29	0.82	O35381;Q64	Acidic leucine-rich nuclear phosphoprotein 32 family member A	Anp32a	365	14	10	10	40.5	36.4	36.4	28.537	0.0000	42.021	14264000	127	15.28	15.97	14.18	15.33	15.43	16.65	15.44	14.41
1.437	-0.29	0.82	Q06185	ATP synthase subunit e, mitochondrial	Atp5i	1750	7	7	7	67.6	67.6	67.6	8.2355	0.0000	28.136	71789000	159	18.45	18.26	18.04	17.99	18.43	18.47	18.46	18.55
1.011	-0.29	0.82	Q99K28	ADP-ribosylation factor GTPase-activating protein 2	Arfgap2	4361	26	26	25	58.7	58.7	56.7	56.598	0.0000	254.77	65777000	490	16.53	16.55	17.10	16.71	16.80	17.09	17.05	17.13
1.740	-0.30	0.81	P70372	ELAV-like protein 1	Elavl1	1561	16	16	16	54.9	54.9	54.9	36.169	0.0000	220.73	71470000	395	17.38	17.56	17.59	17.34	17.68	17.84	17.63	17.90
1.127	-0.30	0.81	P97461	40S ribosomal protein S5;40S ribosomal protein S5, N-terminally proces	Rps5	1653	19	19	19	54.4	54.4	54.4	22.889	0.0000	323.31	577400000	762	20.25	20.29	20.62	20.69	20.59	20.94	20.67	20.83
0.676	-0.30	0.81	P14131	40S ribosomal protein S16	Rps16	774	22	22	22	78.8	78.8	78.8	16.445	0.0000	48.605	247350000	655	19.49	19.04	18.87	18.81	19.36	19.70	19.00	19.34
0.818	-0.30	0.81	Q569Z5	Probable ATP-dependent RNA helicase DDX46	Ddx46	2086	36	36	35	37.6	37.6	36.1	117.45	0.0000	236.23	76761000	642	16.79	16.44	16.74	16.85	16.65	17.13	16.86	17.37
0.223	-0.30	0.81	Q64345	Interferon-induced protein with tetratricopeptide repeats 3	Ifit3	2435	32	32	32	70.7	70.7	70.7	47.222	0.0000	254.53	85299000	412	17.19	17.07	16.11	15.81	17.59	17.49	16.37	15.92
0.312	-0.30	0.81	Q80TE0	RNA polymerase II-associated protein 1	Rpap1	2802	7	7	7	6.7	6.7	6.7	155.27	0.0000	47.053	4565600	58	14.78	14.31	13.96	14.03	14.00	14.66	15.55	14.07
0.517	-0.30	0.81	Q9CYR0	Single-stranded DNA-binding protein, mitochondrial	Ssbp1	4805	7	7	7	43.4	43.4	43.4	17.319	0.0000	106.78	24510000	171	16.44	17.04	16.06	16.25	16.55	16.47	16.80	17.18
0.827	-0.30	0.81	Q8K1R3	Polyribonucleotide nucleotidyltransferase 1, mitochondrial	Pnpt1	3632	31	31	31	45.1	45.1	45.1	85.682	0.0000	102.41	46593000	273	15.90	16.33	16.47	16.13	16.44	16.80	16.17	16.62
1.009	-0.30	0.81	P18760	Cofilin-1	Cfl1	841	31	31	22	94.6	94.6	86.1	18.559	0.0000	323.31	1570800000	1815	21.68	21.86	21.32	21.67	21.99	22.14	21.64	21.97
1.109	-0.30	0.81	P46460	Vesicle-fusing ATPase	Nsf	1104	49	49	49	69.4	69.4	69.4	82.613	0.0000	323.31	191710000	984	17.67	17.53	17.96	17.86	17.88	17.88	18.24	18.24
0.614	-0.30	0.81	O35343	Importin subunit alpha-3	Kpna4	358	17	17	12	37.2	37.2	28.4	57.922	0.0000	249.57	39838000	324	16.95	16.92	16.89	17.35	16.93	17.03	17.58	17.79
0.865	-0.31	0.81	Q8VD66	Abhydrolase domain-containing protein 4	Abhd4	3943	4	4	4	16.1	16.1	16.1	38.86	0.0000	11.942	4463900	46	13.89	14.58	13.91	14.09	14.47	14.38	14.25	14.59
0.232	-0.31	0.81	P28076;O35	Proteasome subunit beta type-9	Psmb9	379	3	3	3	20.1	20.1	20.1	23.396	0.0000	43.504	13137000	63	16.61	17.02	15.30	15.27	16.75	16.14	15.64	16.88
0.926	-0.31	0.81	Q8JZR0	Long-chain-fatty-acid--CoA ligase 5	Acsl5	3580	28	26	26	51.1	48.6	48.6	76.205	0.0000	193.32	48689000	381	15.63	15.90	16.28	16.16	16.13	16.50	16.18	16.38
0.849	-0.31	0.81	Q5SSI6	U3 small nucleolar RNA-associated protein 18 homolog	Utp18	2137	13	13	13	33.9	33.9	33.9	61.217	0.0000	102.82	14407000	156	15.31	15.22	14.76	15.43	15.26	15.75	15.36	15.58
0.392	-0.31	0.81	Q9Z2L7	Cytokine receptor-like factor 3	Crlf3	5858	9	9	9	31	31	31	49.558	0.0000	82.035	9415900	119	14.31	15.40	15.14	15.29	15.43	15.86	15.37	14.70
0.551	-0.31	0.81	Q8VDP2	UPF0428 protein CXorf56 homolog		3963	5	5	5	26.1	26.1	26.1	25.594	0.0000	10.416	4260200	56	13.75	14.37	14.83	14.47	14.35	14.92	14.85	14.53
0.716	-0.31	0.81	P54103	DnaJ homolog subfamily C member 2;DnaJ homolog subfamily C meml	Dnajc2	1247	21	21	21	36.7	36.7	36.7	71.721	0.0000	44.107	14306000	132	14.81	14.62	14.59	15.01	14.92	15.51	14.64	15.21
0.240	-0.31	0.81	Q8BUH8	Sentrin-specific protease 7	Senp7	3224	4	4	4	5.3	5.3	5.3	116.34	0.0000	8.7879	3090400	20	13.31	13.49	14.30	13.57	14.06	12.61	14.74	14.50
1.008	-0.31	0.81	Q8BGQ7	Alanine--tRNA ligase, cytoplasmic	Aars	2988	57	57	57	63.8	63.8	63.8	106.91	0.0000	323.31	298210000	1294	18.59	18.47	19.00	19.00	18.89	19.04	19.11	19.27
0.521	-0.31	0.81	Q04750;Q8F	DNA topoisomerase 1	Top1	1734	33	33	33	41.6	41.6	41.6	90.875	0.0000	130.77	57833000	548	15.95	15.64	15.21	15.67	15.67	16.56	15.52	15.97
1.282	-0.31	0.81	Q922Q4;Q9:	Pyrroline-5-carboxylate reductase 2	Pycr2	4269	14	14	14	45.3	45.3	45.3	33.659	0.0000	60.447	52523000	351	16.68	16.24	16.35	16.36	16.75	16.93	16.51	16.70
1.646	-0.31	0.81	P11352	Glutathione peroxidase 1	Gpx1	742	21	21	21	84.6	84.6	84.6	22.329	0.0000	323.31	503900000	783	20.44	20.61	20.65	20.64	21.13	20.69	20.85	20.91
0.533	-0.31	0.81	Q9D7I5	Phospholysine phosphohistidine inorganic pyrophosphate phosphatase	Lhpp	5031	4	4	4	26.7	26.7	26.7	29.144	0.0000	52.17	3529100	46	13.77	14.90	14.44	13.95	14.57	14.32	14.78	14.64
1.301	-0.31	0.80	Q9CW46	Ribonucleoprotein PTB-binding 1	Raver1	4709	28	28	28	71.9	71.9	71.9	79.381	0.0000	323.31	40217000	320	16.02	16.19	15.73	16.18	16.26	16.47	16.18	16.46
0.632	-0.32	0.80	Q9Z2I9	Succinyl-CoA ligase [ADP-forming] subunit beta, mitochondrial	Sucla2	5857	25	25	25	57.5	57.5	57.5	50.113	0.0000	219.39	85942000	461	16.84	17.41	17.87	17.48	17.42	17.72	17.91	17.81
0.503	-0.32	0.80	Q9WUK2	Eukaryotic translation initiation factor 4H	Eif4h	5712	13	13	13	53.2	53.2	53.2	27.341	0.0000	93.209	19224000	85	15.68	16.36	14.96	15.75	15.92	15.99	16.09	16.02
2.520	-0.32	0.80	P62814;Q91	V-type proton ATPase subunit B, brain isoform	Atp6v1b2	1446	50	50	50	92.4	92.4	92.4	56.55	0.0000	323.31	1598500000	2916	20.68	20.79	20.56	20.67	20.97	21.09	20.87	21.04
0.278	-0.32	0.80	P56183	Ribosomal RNA processing protein 1 homolog A	Rrp1	1276	4	4	4	16.6	16.6	16.6	54.776	0.0000	21.957	1020500	20	11.69	13.72	13.06	12.33	12.58	13.29	12.89	13.32
0.763	-0.32	0.80	Q91VM5	RNA binding motif protein, X-linked-like-1	Rbmxl1	4068	23	23	6	46.9	46.9	14.7	42.161	0.0000	100.79	55475000	417	16.66	16.68	16.32	16.58	16.88	16.55	16.67	17.40
0.304	-0.32	0.80	Q7TN29	Stromal membrane-associated protein 2	Smap2	2756	11	11	10	27.6	27.6	25.7	46.577	0.0000	57.201	23718000	155	14.99	15.83	16.92	16.60	16.43	16.67	16.21	16.32
0.344	-0.32	0.80	Q3U6N9	UPF0488 protein C8orf33 homolog		1929	3	3	3	22.1	22.1	22.1	24.464	0.0000	9.6909	1706600	21	13.13	13.34	12.15	12.43	12.47	13.82	12.87	13.18
0.626	-0.32	0.80	Q6R891;Q9I	Neurabin-2	Ppp1r9b	2654	15	15	15	30	30	30	89.519	0.0000	170.53	17114000	230	15.00	15.46	14.62	15.33	15.13	15.51	15.22	15.83
0.447	-0.32	0.80	P63321	Ras-related protein Ral-A	Rala	1503	10	5	5	51	26.7	26.7	23.553	0.0000	26.016	25829000	172	15.30	16.14	16.52	16.61	16.15	16.39	16.61	16.69
0.339	-0.32	0.80	Q91VW5	Golgin subfamily A member 4	Golga4	4082	10	10	9	6	6	5.4	257.56	0.0000	17.554	3346400	27	14.15	13.15	13.51	14.51	13.45	14.09	14.38	14.70
0.186	-0.32	0.80	P58742	Aladin	Aaas	1321	4	4	4	10.1	10.1	10.1	59.43	0.0000	39.849	4531200	66	12.24	14.11	14.74	13.69	12.75	14.38	14.42	14.53
0.970	-0.32	0.80	P70195	Proteasome subunit beta type-7	Psmb7	1533	8	8	8	23.8	23.8	23.8	29.891	0.0000	226.12	117190000	412	19.13	19.19	19.18	19.42	19.28	19.95	19.32	19.66
1.429	-0.32	0.80	Q921G7	Electron transfer flavoprotein-ubiquinone oxidoreductase, mitochondrial	Etfdh	4223	24	24	24	48.1	48.1	48.1	68.09	0.0000	61.45	36032000	258	15.96	16.32	16.45	15.99	16.52	16.51	16.50	16.49
3.933	-0.32	0.80	Q99KI0	Aconitate hydratase, mitochondrial	Aco2	4377	52	52	52	62.8	62.8	62.8	85.462	0.0000	323.31	631830000	2050	19.19	19.17	19.31	19.27	19.53	19.57	19.61	19.53
0.300	-0.32	0.80	D0QMC3	Myeloid cell nuclear differentiation antigen-like protein	Mndal	207	21	10	5	39.8	18.6	12.6	60.526	0.0000	74.87	9416800	97	15.59	15.13	15.38	14.98	15.00	16.77	15.68	14.91
0.237	-0.32	0.80	Q5SX39	Myosin-4	Myh4	2158	16	15	1	11.2	10.7	0.7	222.86	0.0000	31.098	6378100	39	14.19	13.47	12.75	12.04	12.77	13.90	14.00	13.08
0.447	-0.32	0.80	Q08943	FACT complex subunit SSRP1	Ssrp1	1769	17	17	17	28.7	28.7	28.7	80.859	0.0000	122.33	26127000	240	15.51	15.54	16.54	16.51	16.04	16.76	16.36	16.23
0.536	-0.33	0.80	Q9EQ32	Phosphoinositide 3-kinase adapter protein 1	Pik3ap1	5271	16	16	16	24.4	24.4	24.4	90.927	0.0000	97.436	47811000	320	17.31	17.42	16.31	16.97	17.20	17.50	17.02	17.58
0.571	-0.33	0.80	Q8BIH0	Histone deacetylase complex subunit SAP130	Sap130	3060	4	4	4	5	5	5	111.23	0.0029	2.6585	1526100	16	13.62	13.32	13.15	13.62	13.37	13.91	13.36	14.37
0.585	-0.33	0.80	O88856	Protein-tyrosine sulfotransferase 2	Tpst2	578	3	3	3	12	12	12	42.067	0.0000	9.3738	1467300	16	13.71	13.50	13.33	13.86	13.84	14.53	13.39	13.95
0.945	-0.33	0.80	P49962	Signal recognition particle 9 kDa protein	Srp9	1173	6	6	6	55.8	55.8	55.8	10.194	0.0000	10.554	7260000	33	15.49	15.39	15.42	16.00	15.65	16.14	15.97	15.85
0.251	-0.33	0.80	P97355	Spermine synthase	Sms	1629	10	10	10	54.6	54.6	54.6	41.313	0.0000	171.8	36608000	219	17.29	18.00	15.54	17.15	17.39	17.46	17.47	16.97
0.462	-0.33	0.80	O70152	Dolichol-phosphate mannosyltransferase subunit 1	Dpm1	473	12	12	12	47.7	47.7	47.7	29.174	0.0000	78.347	12633000	108	15.63	15.16	15.44	14.60	15.83	15.19	15.11	16.02
0.487	-0.33	0.80	Q60631	Growth factor receptor-bound protein 2	Grb2	2186	22	22	22	94.5	94.5	94.5	25.238	0.0000	120.19	28312000	277	15.45	16.02	15.36	15.88	15.74	16.36	15.41	16.52
2.706	-0.33	0.80	Q8C0E3	Tripartite motif-containing protein 47	Trim47	3335	12	12	12	31	31	31	69.912	0.0000	109.14	18401000	211	16.24	16.17	16.09	16.25	16.42	16.66	16.49	16.49
0.357	-0.33	0.80	P11672	Neutrophil gelatinase-associated lipocalin	Lcn2	748	5	5	5	28.5	28.5	28.5	22.875	0.0000	10.456	3434200	30	13.31	13.15	12.40	12.86	13.07	14.27	12.86	12.82
0.944	-0.33	0.80	Q9CQM9	Glutaredoxin-3	Glrx3	4613	18	18	17	60.2	60.2	59.6	37.778	0.0000	232.42	62574000	479	17.07	17.18	16.91	17.41	17.48	17.38	17.17	17.86
0.834	-0.33	0.80	P53564	Homeobox protein cut-like 1	Cux1	1232	28	28	16	28.4	28.4	16.9	165.59	0.0000	169.48	32110000	354	15.55	15.85	16.02	16.36	16.11	16.44	16.09	16.46
0.926	-0.33	0.80	Q9D8Y7	Tumor necrosis factor alpha-induced protein 8-like protein 2	Tnfaip8l2	5084	15	15	15	67.4	67.4	67.4	20.615	0.0000	129.14	93946000	345	18.53	18.42	18.18	18.00	18.29	18.89	18.49	18.78
0.160	-0.33	0.79	Q9CQN6	Transmembrane protein 14C	Tmem14c	4616	3	3	3	50	50	50	11.642	0.0000	16.032	4899400	62	15.39	13.94	13.47	12.78	14.76	13.93	12.77	15.44
0.922	-0.33	0.79	P61079;Q6Z	Ubiquitin-conjugating enzyme E2 D3	Ube2d3	1368	6	6	3	48.3	48.3	12.2	16.687	0.0000	229.02	83862000	238	17.83	18.39	18.27	18.10	18.60	18.08	18.51	18.72
1.925	-0.33	0.79	P56480	ATP synthase subunit beta, mitochondrial	Atp5b	1288	41	41	41	86.6	86.6	86.6	56.3	0.0000	323.31	1849000000	2950	21.03	21.37	21.33	21.27	21.47	21.68	21.68	21.51
2.945	-0.33	0.79	Q8K4I3	Rho guanine nucleotide exchange factor 6	Arhgef6	3717	23	20	20	32.4	29.4	29.4	87.051	0.0000	219.3	34973000	322	16.10	16.10	16.14	16.10	16.43	16.55	16.29	16.50
0.386	-0.33	0.79	Q8K382	DENN domain-containing protein 1A	Dennd1a	3694	10	10	10	14.4	14.4	14.4	111.54	0.0000	31.938	7313100	45	14.58	13.94	14.48	14.38	13.65	14.81	15.17	15.08
1.024	-0.33	0.79	Q9DB29	Isoamyl acetate-hydrolyzing esterase 1 homolog	Iah1	5122	9	9	9	43	43	43	27.974	0.0000	164.94	33939000	246	16.13	16.12	16.44	16.74	16.55	16.61	16.69	16.92
0.546	-0.33	0.79	Q8R1S4	Metastasis suppressor protein 1	Mtss1	3798	14	14	14	28.5	28.5	28.5	82.407	0.0000	59.936	11814000	106	15.79	15.03	15.41	15.35	15.55	16.30	15.19	15.89
1.391	-0.33	0.79	P61750	ADP-ribosylation factor 4	Arf4	1388	13	9	7	67.8	43.9	27.8	20.396	0.0000	109.05	39940000	173	17.86	17.84	18.32	18.02	18.49	18.23	18.40	18.25
0.250	-0.33	0.79	Q8BGF6	ELMO domain-containing protein 2	Elmod2	2980	4	4	4	15.7	15.7	15.7	34.747	0.0002	6.055	2572000	22	15.20	13.75	14.19	13.56	13.51	14.85	15.40	14.28
1.199	-0.33	0.79	Q8K4Z5	Splicing factor 3A subunit 1	Sf3a1	3731	32	32	31	44.6	44.6	43.4	88.544	0.0000	202.27	223090000	518	19.77	19.83	20.12	20.17	20.07	20.46	20.19	20.52
0.618	-0.33	0.79	Q9CX86	Heterogeneous nuclear ribonucleoprotein A0	Hnrnpa0	4750	18	18	18	56.7	56.7	56.7	30.53	0.0000	90.438	56471000	349	17.15	17.13	16.27	17.10	17.43	17.19	16.88	17.51
0.419	-0.34	0.79	Q69ZA1	Cyclin-dependent kinase 13	Cdk13	2480	3	2	2	2.8	2.3	2.3	164.55	0.0000	46.912	1390700	58	13.60	12.74	13.07	12.81	13.61	13.95	12.56	13.46
0.982	-0.34	0.79	Q9DB20	ATP synthase subunit O, mitochondrial	Atp5o	5119	24	24	24	79.8	79.8	79.8	23.363	0.0000	323.31	443680000	982	19.59	19.30	20.00	19.50	19.65	20.01	20.00	20.07
0.735	-0.34	0.79	Q8R326	Paraspeckle component 1	Pspc1	3833	12	12	12	34.2	34.2	34.2	58.758	0.0000	88.849	21582000	215	14.70	15.19	15.28	15.50	15.07	15.65	15.57	15.74
0.171	-0.34	0.79	Q99MP8	BRCA1-associated protein	Brap	4470	8	8	8	18.4	18.4	18.4	66.99	0.0000	9.255	6860700	24	13.33	16.00	16.63	14.29	15.56	15.40	15.17	15.47
0.425	-0.34	0.79	Q3UKU4	Protein FAM83F	Fam83f	1989	3	3	3	10.7	10.7	10.7	55.012	0.0000	63.221	1932000	40	14.10	13.99	14.51	12.93	14.03	14.42	14.38	14.05
0.756	-0.34	0.79	Q9JIH2	Nuclear pore complex protein Nup50	Nup50	5400	11	11	11	30.3	30.3	30.3	49.485	0.0000	59.335	14205000	218	14.90	15.28	14.88	15.28	15.03	15.37	15.35	15.94
0.758	-0.34	0.79	P61971	Nuclear transport factor 2	Nutf2	1397	5	5	5	40.2	40.2	40.2	14.478	0.0000	21.212	20809000	119	16.33	16.14	16.51	16.73	16.48	17.02	16.44	17.13
0.241	-0.34	0.79	Q4VBF2	R3H domain-containing protein 4	R3hdm4	2072	3	3	3	17.9	17.9	17.9	29.659	0.0000	24.826	2140100	53	14.24	14.24	13.00	12.88	14.70	13.44	12.96	14.63
0.476	-0.34	0.79	Q9QYA2	Mitochondrial import receptor subunit TOM40 homolog	Tomm40	5565	9	9	9	36.3	36.3	36.3	37.895	0.0000	29.367	23668000	182	16.78	16.44	16.76	15.43	16.77	16.56	16.86	16.60
1.170	-0.34	0.79	Q8VDD5	Myosin-9	Myh9	3948	254	254	216	80.8	80.8	73.5	226.37	0.0000	323.31	3082800000	9892	20.28	20.30	20.47	20.68	20.46	20.88	20.74	21.03
0.239	-0.34	0.79	Q99MR1	PERQ amino acid-rich with GYF domain-containing protein 1	Gigyf1	4472	4	4	4	6.3	6.3	6.3	116.24	0.0000	17.48	3223800	33	13.05	14.11	13.84	14.00	12.52	14.57	14.44	14.84
0.603	-0.35	0.79	Q9JI99	Sphingosine-1-phosphate phosphatase 1	Sgpp1	5392	6	6	6	17.4	17.4	17.4	47.744	0.0000	18.214	13197000	100	15.00	15.53	15.79	14.66	15.35	15.74	15.53	15.75
0.253	-0.35	0.79	Q9DC70	NADH dehydrogenase [ubiquinone] iron-sulfur protein 7, mitochondrial	Ndufs7	5200	8	8	8	34.8	34.8	34.8	24.683	0.0000	92.219	18104000	127	16.69	16.56	14.58	15.51	16.86	16.27	15.99	15.61
0.785	-0.35	0.79	Q9CQ69	Cytochrome b-c1 complex subunit 8	Uqcrq	4568	9	9	9	80.5	80.5	80.5	9.7681	0.0000	19.738	23014000	128	16.10	16.48	16.69	16.72	16.79	16.64	16.63	17.33
0.890	-0.35	0.79	Q9Z199;P63	Transcription elongation factor SPT4-B;Transcription elongation factor S	Supt4h1b;Supt4h1	1500	3	3	3	22.2	22.2	22.2	13.194	0.0009	3.7542	6913100	46	15.86	15.70	15.16	15.85	15.77	16.11	15.86	16.23
0.675	-0.35	0.79	Q60668	Heterogeneous nuclear ribonucleoprotein D0	Hnrnpd	2191	23	23	22	47.9	47.9	45.6	38.354	0.0000	323.31	158740000	708	18.93	19.25	18.49	19.14	19.07	19.13	19.15	19.85
0.802	-0.35	0.79	Q6ZQB6	Inositol hexakisphosphate and diphosphoinositol-pentakisphosphate kin	Ppip5k2	2694	16	16	14	19.8	19.8	16.9	128.43	0.0000	123.18	16384000	231	15.26	14.94	14.99	14.99	15.39	15.78	14.83	15.58
0.389	-0.35	0.79	P52432	DNA-directed RNA polymerases I and III subunit RPAC1	Polr1c	1217	7	7	7	28.9	28.9	28.9	39.106	0.0000	42.195	6818200	63	13.57	14.47	14.87	15.23	14.69	14.62	14.88	15.35
0.796	-0.35	0.78	Q8K4L3	Supervillin	Svil	3721	37	37	37	23.2	23.2	23.2	243.16	0.0000	216.17	29968000	339	15.24	15.35	15.13	15.39	15.22	16.14	15.35	15.80
1.003	-0.35	0.78	P11103	Poly [ADP-ribose] polymerase 1	Parp1	738	44	44	44	46.7	46.7	46.7	113.1	0.0000	224.92	123720000	925	18.09	17.75	17.80	18.03	17.98	18.62	18.02	18.45
0.421	-0.35	0.78	Q9DBF7	Pre-mRNA-splicing factor CWC25 homolog	Cwc25	5149	5	5	5	12.3	12.3	12.3	48.811	0.0002	5.691	3139900	25	13.60	13.23	13.41	14.18	13.96	13.79	13.29	14.77
1.278	-0.35	0.78	Q8K2Q0	COMM domain-containing protein 9	Commd9	3667	6	6	6	51	51	51	21.85	0.0000	54.234	11382000	117	15.70	15.41	15.93	15.75	15.89	16.33	16.04	15.94
0.552	-0.35	0.78	P83093	Stromal interaction molecule 2	Stim2	1600	5	5	5	8.6	8.6	8.6	83.924	0.0000	8.4176	1568100	29	12.20	13.54	13.06	12.97	13.04	13.22	13.50	13.43
0.135	-0.35	0.78	Q9CPN8	Insulin-like growth factor 2 mRNA-binding protein 3	Igf2bp3	4522	12	12	11	30.7	30.7	28.5	63.574	0.0000	35.384	7971800	81	14.99	15.63	13.76	12.49	13.25	13.49	15.56	15.98
0.925	-0.35	0.78	Q8BG07	Phospholipase D4	Pld4	2957	20	20	20	48.7	48.7	48.7	56.153	0.0000	194.84	188180000	581	18.78	19.37	19.15	18.52	19.42	19.24	19.26	19.32
0.465	-0.35	0.78	Q9CWD8	Iron-sulfur protein NUBPL	Nubpl	4710	4	4	4	23.5	23.5	23.5	34.139	0.0000	31.185	2826200	43	14.36	14.09	14.01	13.18	14.16	13.65	14.68	14.56
1.117	-0.35	0.78	B1AZI6	THO complex subunit 2	Thoc2	75	17	17	17	14.3	14.3	14.3	182.77	0.0000	92.387	14577000	173	15.66	15.52	15.74	15.72	15.74	16.46	16.01	15.85
0.751	-0.35	0.78	O35841	Apoptosis inhibitor 5	Api5	405	27	27	27	47.2	47.2	47.2	56.784	0.0000	323.31	93651000	640	16.90	16.88	16.84	17.15	16.72	17.43	17.25	17.78
0.599	-0.35	0.78	Q9DBG5	Perilipin-3	Plin3	5152	33	33	33	90.4	90.4	90.4	47.262	0.0000	323.31	193150000	1036	17.93	18.30	18.32	18.69	18.21	18.71	18.45	19.30
0.953	-0.35	0.78	Q3U0V1	Far upstream element-binding protein 2	Khsrp	1897	37	37	34	51.2	51.2	47.6	76.775	0.0000	323.31	223730000	1145	17.61	18.00	17.39	17.84	17.99	18.08	17.78	18.43
0.487	-0.36	0.78	Q9Z0E6;Q0	Interferon-induced guanylate-binding protein 2	Gbp2	5765	24	24	23	59.4	59.4	56.2	66.739	0.0000	323.31	24421000	246	16.06	15.48	15.21	15.14	16.10	16.41	15.36	15.43
1.000	-0.36	0.78	Q6P5B0	RRP12-like protein	Rrp12	2569	17	17	17	17.9	17.9	17.9	143.13	0.0000	91.951	13531000	145	14.96	14.63	14.60	15.09	14.90	15.02	15.28	15.52
1.054	-0.36	0.78	P97369	Neutrophil cytosol factor 4	Ncf4	1634	29	29	29	76.1	76.1	76.1	38.707	0.0000	323.31	264540000	935	17.90	18.12	18.61	18.45	18.58	18.78	18.44	18.72
0.515	-0.36	0.78	Q64705	Upstream stimulatory factor 2	Usf2	2460	3	3	2	12.4	12.4	9	36.953	0.0002	5.8719	1434900	14	12.75	13.42	13.36	13.16	13.26	13.23	13.26	14.38
0.532	-0.36	0.78	Q6YGZ1	Heparanase;Heparanase 8 kDa subunit;Heparanase 50 kDa subunit	Hpse	2673	14	14	14	37.9	37.9	37.9	60.065	0.0000	146.29	36182000	249	16.47	17.04	16.09	16.89	16.57	17.15	16.67	17.55
0.293	-0.36	0.78	Q9D975	Sulfiredoxin-1	Srxn1	5099	5	5	5	50.7	50.7	50.7	14.149	0.0000	43.041	20051000	181	15.82	16.44	14.89	16.22	15.81	15.43	16.39	17.19
0.915	-0.36	0.78	Q7TPV4	Myb-binding protein 1A	Mybbp1a	2772	55	55	55	46.4	46.4	46.4	152.04	0.0000	323.31	142510000	1255	17.21	16.87	16.74	16.98	16.87	17.59	17.20	17.59
0.702	-0.36	0.78	Q8CFE4	SCY1-like protein 2	Scyl2	3483	16	16	16	29	29	29	103.32	0.0000	139.38	19157000	214	15.48	15.72	16.30	15.99	15.77	16.53	16.14	16.48
1.199	-0.36	0.78	Q61545	RNA-binding protein EWS	Ewsr1	2310	5	5	5	11.3	11.3	11.3	68.461	0.0000	95.176	18932000	148	15.84	16.09	16.32	16.07	16.19	16.61	16.27	16.70
1.460	-0.37	0.78	Q9CPW4	Actin-related protein 2/3 complex subunit 5	Arpc5	4539	12	12	11	84.8	84.8	83.4	16.288	0.0000	323.31	192510000	460	18.96	18.85	19.04	19.01	19.22	19.14	19.25	19.71
0.475	-0.37	0.78	Q91XV3	Brain acid soluble protein 1	Basp1	4150	10	10	10	51.8	51.8	51.8	22.086	0.0000	261.36	23943000	228	15.49	15.95	16.73	16.93	16.55	16.68	16.45	16.89
1.106	-0.37	0.78	Q9D7S7	60S ribosomal protein L22-like 1	Rpl22l1	5039	7	6	6	54.9	48.4	48.4	14.467	0.0000	100.15	32313000	163	16.50	16.76	16.54	16.80	16.84	17.36	16.68	17.18
0.935	-0.37	0.78	Q61749	Translation initiation factor eIF-2B subunit delta	Eif2b4	2331	17	17	17	42.6	42.6	42.6	57.624	0.0000	97.038	23930000	241	15.16	15.28	15.77	15.59	15.67	16.20	15.54	15.84
0.690	-0.37	0.77	Q9D1H8	39S ribosomal protein L53, mitochondrial	Mrpl53	4916	6	6	6	55.1	55.1	55.1	12.737	0.0000	41.869	9647600	103	15.14	15.76	15.25	15.70	15.55	15.46	15.95	16.35
0.768	-0.37	0.77	Q9CQJ8	NADH dehydrogenase [ubiquinone] 1 beta subcomplex subunit 9	Ndufb9	4607	10	10	10	58.7	58.7	58.7	21.984	0.0000	52.294	18489000	123	15.59	15.55	15.33	15.38	16.03	16.36	15.60	15.33

1.247	-0.37	0.77	P63094;Q6F	Guanine nucleotide-binding protein G(s) subunit alpha isoforms short;G	Gnas	1490	22	20	14	53.6	49.5	39.3	45.663	0.0000	122.95	58442000	351	16.65	16.56	16.94	16.28	17.00	16.84	16.89	17.20
0.371	-0.37	0.77	Q8CHW4	Translation initiation factor eIF-2B subunit epsilon	Eif2b5	3533	13	13	13	23	23	23	80.085	0.0000	37.785	15445000	72	15.84	15.74	17.13	15.46	16.75	16.70	16.42	15.79
1.199	-0.37	0.77	Q09014	Neutrophil cytosol factor 1	Ncf1	1770	37	37	37	75.6	75.6	75.6	44.666	0.0000	323.31	278040000	1177	18.44	18.32	18.10	18.39	18.69	19.05	18.35	18.65
0.713	-0.37	0.77	Q8CDM1	ATPase family AAA domain-containing protein 2	Atad2	3462	9	9	9	14.1	14.1	14.1	117.94	0.0000	61.261	2953700	24	13.07	12.36	12.76	13.12	13.00	13.73	13.14	12.93
0.328	-0.37	0.77	Q91WK0	Leucine-rich repeat flightless-interacting protein 2	Lrrfip2	4114	13	13	12	32.8	32.8	29.9	47.147	0.0000	31.349	4491400	47	13.28	13.17	12.74	13.26	14.60	13.82	12.45	13.07
1.958	-0.37	0.77	P14152	Malate dehydrogenase, cytoplasmic	Mdh1	776	27	27	27	75.1	75.1	75.1	36.511	0.0000	323.31	506610000	815	19.90	19.94	19.88	19.91	20.15	20.11	20.56	20.30
0.758	-0.37	0.77	Q0VBL3		Rbm15	1777	12	12	12	18.8	18.8	18.8	105.72	0.0000	51.813	12191000	86	15.31	15.33	15.36	15.64	15.27	16.10	15.53	16.23
0.819	-0.37	0.77	Q61205	Platelet-activating factor acetylhydrolase IB subunit gamma	Pafah1b3	2275	11	11	11	64.2	64.2	64.2	25.853	0.0000	58.817	16208000	124	15.30	16.05	15.91	15.62	16.54	15.84	15.92	16.07
0.742	-0.38	0.77	Q9CQC9	GTP-binding protein SAR1b	Sar1b	4585	13	9	9	57.6	46	46	22.382	0.0000	78.309	45409000	124	17.98	17.79	17.13	17.30	17.68	18.05	17.69	18.28
2.300	-0.38	0.77	Q8C3Q9	Caspase-9;Caspase-9 subunit p35;Caspase-9 subunit p10	Casp9	3378	6	6	6	22.9	22.9	22.9	49.979	0.0000	104.2	10712000	134	15.20	15.04	15.34	15.07	15.41	15.59	15.65	15.51
1.297	-0.38	0.77	Q61768	Kinesin-1 heavy chain	Kif5b	2333	90	90	73	72.8	72.8	64.3	109.55	0.0000	323.31	337950000	2028	18.07	18.06	18.16	18.41	18.29	18.54	18.46	18.91
1.535	-0.38	0.77	P17918	Proliferating cell nuclear antigen	Pcna	832	20	20	20	80.5	80.5	80.5	28.785	0.0000	254.73	193580000	798	17.67	18.02	18.05	18.19	18.40	18.52	18.37	18.16
0.636	-0.38	0.77	Q9DCJ9	N-acetylneuraminate lyase	Npl	5219	11	11	11	64.7	64.7	64.7	35.13	0.0000	139.45	13171000	146	14.84	14.66	15.18	14.99	15.53	14.54	15.39	15.72
0.420	-0.38	0.77	Q8K4M5	COMM domain-containing protein 1	Commd1	3723	6	6	6	25	25	25	20.996	0.0000	61.523	5961600	60	14.42	14.66	14.58	15.29	14.13	15.11	15.55	15.66
0.416	-0.38	0.77	Q8BGC4	Zinc-binding alcohol dehydrogenase domain-containing protein 2	Zadh2	2976	11	11	11	47.7	47.7	47.7	40.528	0.0000	22.906	12263000	97	14.11	15.58	15.74	15.52	15.45	15.81	15.87	15.32
2.456	-0.38	0.77	P70268;Q8K	Serine/threonine-protein kinase N1	Pkn1	1542	32	32	31	42.6	42.6	41.8	104.41	0.0000	323.31	71043000	480	17.02	16.85	17.21	17.04	17.44	17.45	17.31	17.43
1.556	-0.38	0.77	Q8VE70	Programmed cell death protein 10	Pdcd10	3988	10	10	10	45.3	45.3	45.3	24.715	0.0000	262.7	33379000	214	16.82	16.51	16.76	16.81	16.81	17.08	17.30	17.23
0.375	-0.38	0.77	Q91XF0	Pyridoxine-5-phosphate oxidase	Pnpo	4146	4	4	4	20.7	20.7	20.7	30.114	0.0000	17.018	6120000	26	14.97	16.10	14.74	14.35	14.80	15.34	15.80	15.74
1.313	-0.38	0.77	Q60692	Proteasome subunit beta type-6	Psmb6	2195	9	9	9	52.9	52.9	52.9	25.378	0.0000	240.82	99778000	226	18.17	18.78	18.49	18.17	18.74	18.92	18.70	18.77
1.243	-0.39	0.77	Q91YX0	Protein THEMIS2	Themis2	4183	19	19	19	45.2	45.2	45.2	74.377	0.0000	188.87	37600000	259	16.44	16.24	16.71	16.41	16.74	17.16	16.54	16.90
0.894	-0.39	0.76	Q9Z2B9	Ribosomal protein S6 kinase alpha-4	Rps6ka4	5838	11	11	11	18.6	18.6	18.6	85.651	0.0000	24.323	13469000	103	15.94	15.77	16.03	16.10	16.61	16.75	15.85	16.17
0.359	-0.39	0.76	P40338	Von Hippel-Lindau disease tumor suppressor	Vhl	1062	4	4	4	21	21	21	20.769	0.0000	6.5018	3926800	32	12.62	13.86	14.17	14.63	14.50	14.37	13.70	14.25
0.586	-0.39	0.76	Q9CZV8;Q8	F-box/LRR-repeat protein 20	Fbxl20	4841	6	6	6	13.8	13.8	13.8	48.396	0.0000	9.2105	2458600	22	12.80	13.45	13.70	13.66	13.64	13.44	14.47	13.60
0.559	-0.39	0.76	Q9R0U0	Serine/arginine-rich splicing factor 10	Srsf10	5642	8	8	8	24.4	24.4	24.4	31.3	0.0000	39.163	26846000	134	17.00	17.55	16.23	16.67	17.38	17.65	16.92	17.04
2.310	-0.39	0.76	Q8BUV3	Gephyrin;Molybdopterin adenylyltransferase;Molybdopterin molybdenur	Gphn	3230	12	12	12	25.1	25.1	25.1	83.281	0.0000	62.68	15461000	146	15.26	15.27	15.33	15.22	15.41	15.69	15.75	15.78
0.890	-0.39	0.76	Q8R550	SH3 domain-containing kinase-binding protein 1	Sh3kbp1	3879	24	24	24	37.1	37.1	37.1	78.169	0.0000	122.6	21342000	215	14.49	14.84	14.98	15.28	14.86	15.51	15.43	15.36
1.155	-0.39	0.76	Q9Z1D1	Eukaryotic translation initiation factor 3 subunit G	Eif3g	5804	11	11	11	40.6	40.6	40.6	35.638	0.0000	83.783	28575000	228	15.80	16.59	16.11	16.11	16.54	16.37	16.56	16.70
1.787	-0.39	0.76	Q9WUM3	Coronin-1B	Coro1b	5715	34	31	31	52.9	49.6	49.6	53.912	0.0000	323.31	213640000	752	19.09	18.94	19.02	18.66	19.48	19.38	19.14	19.26
0.862	-0.39	0.76	Q9Z1M0	P2X purinoceptor 7	P2rx7	5812	13	13	13	34.3	34.3	34.3	68.388	0.0000	116.47	19565000	211	15.91	15.65	15.44	14.85	15.90	15.86	15.86	15.80
0.805	-0.39	0.76	P42125	Enoyl-CoA delta isomerase 1, mitochondrial	Eci1	1072	12	12	12	35.3	35.3	35.3	32.25	0.0000	144.15	61385000	372	17.20	17.10	17.61	16.53	17.37	17.77	17.47	17.38
1.923	-0.39	0.76	Q6ZWV3	60S ribosomal protein L10	Rpl10	2707	26	26	6	75.2	75.2	14.5	24.604	0.0000	323.31	374820000	1013	18.88	19.02	19.17	19.14	19.21	19.59	19.41	19.56
2.811	-0.39	0.76	P16858	Glyceraldehyde-3-phosphate dehydrogenase	Gapdh	814	49	49	46	97.6	97.6	97.6	35.81	0.0000	323.31	6991100000	6082	22.61	22.60	22.66	22.87	23.03	23.17	23.04	23.07
0.513	-0.39	0.76	Q922K7	Probable 28S rRNA (cytosine-C(5))-methyltransferase	Nop2	4262	12	12	12	18	18	18	86.751	0.0000	59.288	11866000	138	15.10	15.09	15.20	15.43	14.93	16.43	15.16	15.89
0.721	-0.39	0.76	P21550	Beta-enolase	Eno3	866	13	7	7	42.2	23.7	23.7	47.024	0.0000	12.572	6195000	71	14.58	14.57	15.16	15.21	14.69	15.46	15.37	15.56
1.046	-0.39	0.76	P67778	Prohibitin	Phb	1508	32	32	32	94.5	94.5	94.5	29.82	0.0000	323.31	419520000	1111	18.77	18.49	19.30	18.98	19.02	19.44	19.39	19.26
0.727	-0.40	0.76	Q61029	Lamina-associated polypeptide 2, isoforms beta/delta/epsilon/gamma	Tmpo	2239	29	16	16	68.1	36.1	36.1	50.372	0.0000	165.53	131830000	446	17.47	17.57	18.37	17.71	17.85	18.65	18.02	18.18
1.052	-0.40	0.76	Q8BWT1	3-ketoacyl-CoA thiolase, mitochondrial	Acaa2	3265	33	33	33	84.9	84.9	84.9	41.829	0.0000	323.31	363610000	1412	18.58	18.70	19.18	19.15	18.98	19.24	19.51	19.46
0.302	-0.40	0.76	P17156	Heat shock-related 70 kDa protein 2	Hspa2	819	26	8	8	42	17.5	17.5	69.641	0.0000	17.593	6599600	68	13.12	14.96	15.64	14.81	14.70	15.05	15.30	15.06
1.029	-0.40	0.76	P61979	Heterogeneous nuclear ribonucleoprotein K	Hnrnpk	1398	37	37	35	72.8	72.8	67.2	50.976	0.0000	323.31	534240000	1400	19.75	20.37	19.57	20.05	20.24	20.32	20.16	20.59
2.654	-0.40	0.76	O89053	Coronin-1A	Coro1a	599	37	36	35	67.7	66.2	64.6	50.989	0.0000	323.31	873280000	1381	19.77	20.04	20.01	19.98	20.46	20.35	20.23	20.34
1.584	-0.40	0.76	P59325	Eukaryotic translation initiation factor 5	Eif5	1334	21	21	21	45.7	45.7	45.7	48.968	0.0000	38.453	64459000	420	16.91	16.97	16.44	16.91	17.20	17.26	17.05	17.31
0.656	-0.40	0.76	Q8R332	Nucleoporin p58/p45	Nupl1	3834	6	6	5	12.8	12.8	10.7	59.445	0.0000	45.467	10453000	117	14.85	14.66	14.75	15.03	15.03	16.02	15.17	14.68
0.549	-0.40	0.76	Q60872	Eukaryotic translation initiation factor 1A	Eif1a	2218	11	11	2	61.8	61.8	7.6	16.502	0.0000	176.94	57202000	280	16.68	17.26	16.12	17.21	16.79	17.24	17.06	17.80
2.119	-0.40	0.76	Q9D051	Pyruvate dehydrogenase E1 component subunit beta, mitochondrial	Pdhb	4852	19	19	19	51.8	51.8	51.8	38.937	0.0000	219.54	130680000	508	18.08	18.15	18.35	18.39	18.66	18.79	18.68	18.46
0.564	-0.40	0.76	P33174	Chromosome-associated kinesin KIF4	Kif4	999	3	2	2	3	2.1	2.1	139.52	0.0002	5.7268	648960	16	12.20	12.73	12.45	13.34	13.50	12.45	13.27	13.10
0.352	-0.41	0.75	D3YXJ0	Diacylglycerol kinase	Dgkh	208	3	3	3	4	4	4	134.13	0.0000	7.2117	2181900	23	13.30	14.01	14.69	12.54	13.82	13.67	14.23	14.44
0.353	-0.41	0.75	Q61827	Transcription factor MafK	Mafk	2337	8	8	6	47.4	47.4	38.5	17.537	0.0000	10.717	15099000	42	16.91	15.67	16.06	15.75	16.23	17.02	15.49	17.28
1.274	-0.41	0.75	Q8CCF0	U4/U6 small nuclear ribonucleoprotein Prp31	Prpf31	3446	19	19	19	41.7	41.7	41.7	55.429	0.0000	137.87	35066000	336	15.81	15.97	16.03	15.83	16.02	16.71	16.10	16.44
1.954	-0.41	0.75	Q0GNC1	Inverted formin-2	Inf2	1774	25	25	25	30.5	30.5	30.5	138.56	0.0000	230.98	53267000	403	16.71	17.12	16.90	16.72	17.36	17.35	17.10	17.27
0.621	-0.41	0.75	Q9CQR4	Acyl-coenzyme A thioesterase 13;Acyl-coenzyme A thioesterase 13, N-t	Acot13	4625	11	11	11	82.1	82.1	82.1	15.183	0.0000	61.82	42407000	209	17.23	17.19	16.98	17.90	17.14	17.55	18.22	18.04
0.689	-0.41	0.75	Q8BT07	Centrosomal protein of 55 kDa	Cep55	3195	5	5	5	13.6	13.6	13.6	53.93	0.0000	6.822	1981900	10	13.23	13.23	13.02	13.34	14.37	13.46	13.02	13.62
0.375	-0.41	0.75	Q6WKZ8	E3 ubiquitin-protein ligase UBR2	Ubr2	2666	8	8	8	5.9	5.9	5.9	199.15	0.0000	94.846	7449300	74	14.65	13.15	15.28	14.92	15.04	15.02	14.98	14.60
1.736	-0.41	0.75	P62908	40S ribosomal protein S3	Rps3	1467	36	36	36	93.8	93.8	93.8	26.674	0.0000	278.55	445340000	1176	19.38	19.62	19.84	19.53	20.15	19.91	19.82	20.14
0.531	-0.42	0.75	P59808	SAM and SH3 domain-containing protein 1	Sash1	1345	21	21	20	26.4	26.4	26.4	135.59	0.0000	105.65	16689000	69	16.28	15.59	15.66	15.18	15.93	15.93	15.61	16.91
1.386	-0.42	0.75	Q9JLV6	Bifunctional polynucleotide phosphatase/kinase;Polynucleotide 3-phosp	Pnkp	5492	28	28	28	63.6	63.6	63.6	57.223	0.0000	289.18	59987000	454	16.20	16.26	16.71	16.22	16.74	17.07	16.60	16.64
0.938	-0.42	0.75	Q9Z321	DNA topoisomerase 3-beta-1	Top3b	5878	7	7	6	12.9	12.9	11.6	96.948	0.0000	20.786	4533300	47	13.69	13.58	14.22	13.61	14.53	14.42	13.80	14.02
0.675	-0.42	0.75	Q9DCR2	AP-3 complex subunit sigma-1	Ap3s1	5225	7	7	7	37.8	37.8	37.8	21.732	0.0000	43.173	72934000	177	17.95	18.58	18.56	17.60	18.18	18.43	18.76	18.98
1.164	-0.42	0.75	Q8BHJ5	F-box-like/WD repeat-containing protein TBL1XR1	Tbl1xr1	3040	9	9	7	33.3	33.3	25.3	55.66	0.0000	181.5	10277000	93	15.14	15.21	15.78	15.33	15.72	15.79	15.53	16.11
0.294	-0.42	0.75	Q9EQU3	Toll-like receptor 9	Tlr9	5288	11	11	11	15.3	15.3	15.3	116.41	0.0000	49.093	7251400	49	13.62	13.85	14.98	13.73	14.85	15.51	14.44	13.08
0.515	-0.42	0.75	O88876	Short-chain dehydrogenase/reductase 3	Dhrs3	580	6	6	6	21.5	21.5	21.5	33.652	0.0000	23.558	3265300	30	14.81	14.23	13.46	13.55	14.23	15.02	14.08	14.42
1.135	-0.42	0.75	Q8BM55	Transmembrane protein 214	Tmem214	3127	26	26	26	36.5	36.5	36.5	76.429	0.0000	233.52	35401000	340	15.96	15.77	15.73	15.77	15.71	16.20	16.48	16.53
0.864	-0.42	0.75	Q9WVR4	Fragile X mental retardation syndrome-related protein 2	Fxr2	5760	11	9	9	22.4	18.4	18.4	73.742	0.0000	62.064	5863800	106	14.55	14.76	14.26	14.79	14.70	15.33	14.60	15.44
1.675	-0.42	0.75	P08249	Malate dehydrogenase, mitochondrial	Mdh2	684	34	34	34	75.4	75.4	75.4	35.611	0.0000	323.31	1314700000	1758	20.40	20.36	20.76	20.48	20.91	21.22	20.83	20.75
1.561	-0.43	0.74	P84078;P61	ADP-ribosylation factor 1;ADP-ribosylation factor 3	Arf1;Arf3	1376	15	15	5	65.2	65.2	28.7	20.697	0.0000	323.31	213410000	574	19.48	19.59	19.24	19.26	19.76	19.63	19.70	20.17
0.390	-0.43	0.74	P70677	Caspase-3;Caspase-3 subunit p17;Caspase-3 subunit p12	Casp3	1583	9	9	9	40.1	40.1	40.1	31.474	0.0000	31.356	17693000	101	16.18	15.33	17.33	15.32	16.34	16.59	16.42	16.52
0.351	-0.43	0.74	P52196	Thiosulfate sulfurtransferase	Tst	1213	6	6	6	25.3	25.3	25.3	33.466	0.0000	19.65	12334000	93	15.11	14.50	16.26	15.49	15.05	15.21	16.34	16.46
0.415	-0.43	0.74	Q99LE1	RILP-like protein 2	Rilpl2	4423	14	14	13	78.2	78.2	78.2	22.393	0.0000	118.36	17370000	251	14.95	15.51	13.94	15.57	15.19	15.16	15.15	16.18
1.746	-0.43	0.74	D3YXK2	Scaffold attachment factor B1	Safb	210	22	22	18	28.4	28.4	21.1	105.1	0.0000	262.51	21204000	249	14.62	15.01	15.13	15.18	15.34	15.35	15.46	15.50
1.514	-0.43	0.74	P06537	Glucocorticoid receptor	Nr3c1	659	11	11	11	22.3	22.3	22.3	86.052	0.0000	56.241	11883000	145	15.09	15.41	15.49	15.22	15.54	15.80	15.53	16.04
2.316	-0.43	0.74	Q8VDJ3	Vigilin	Hdlbp	3956	80	80	80	56.1	56.1	56.1	141.74	0.0000	323.31	327640000	1681	18.29	18.11	18.25	18.45	18.51	18.79	18.83	18.69
1.757	-0.43	0.74	Q9R190	Metastasis-associated protein MTA2	Mta2	5648	28	28	23	43.6	43.6	37.6	75.029	0.0000	231	72366000	580	17.27	17.09	16.91	17.25	17.35	17.83	17.47	17.58
1.175	-0.43	0.74	Q8VIJ6	Splicing factor, proline- and glutamine-rich	Sfpq	4034	47	47	45	51.5	51.5	50.1	75.441	0.0000	323.31	242630000	992	17.88	18.25	17.88	18.41	18.30	18.56	18.36	18.92
1.270	-0.43	0.74	O08600	Endonuclease G, mitochondrial	Endog	280	4	4	4	19	19	19	32.19	0.0000	11.942	3697100	30	14.21	14.50	14.26	13.94	14.62	15.00	14.68	14.33
0.968	-0.43	0.74	Q9JLB0	MAGUK p55 subfamily member 6	Mpp6	5475	10	10	10	28.4	28.4	28.4	62.63	0.0000	38.706	7896200	98	15.28	14.48	14.87	14.42	15.45	15.27	15.13	14.92
0.677	-0.43	0.74	Q6P5D3	Putative ATP-dependent RNA helicase DHX57	Dhx57	2571	5	5	5	6.4	6.4	6.4	155.76	0.0000	8.7751	1554000	27	11.94	12.88	12.92	12.93	12.98	12.83	13.65	12.94
0.487	-0.43	0.74	Q64516;Q9V	Glycerol kinase	Gk	2450	16	16	16	34.3	34.3	34.3	61.227	0.0000	66.337	17719000	142	15.40	15.20	15.47	13.80	15.44	15.28	15.24	15.62
1.985	-0.43	0.74	P48758	Carbonyl reductase [NADPH] 1	Cbr1	1149	25	24	24	87.4	84.5	84.5	30.641	0.0000	188.04	70303000	459	16.93	16.87	16.88	17.19	17.27	17.56	17.22	17.55
0.807	-0.43	0.74	Q8CBW3	Abl interactor 1	Abi1	3439	25	25	18	50.3	50.3	43.5	52.287	0.0000	323.31	168570000	811	17.96	18.44	17.94	18.37	18.27	18.69	18.24	19.23
0.637	-0.43	0.74	P40630	Transcription factor A, mitochondrial	Tfam	1063	7	7	7	36.6	36.6	36.6	27.987	0.0000	50.005	4924200	85	13.78	14.44	13.42	14.30	14.09	15.05	14.12	14.40
0.736	-0.43	0.74	Q8C0D5	Elongation factor Tu GTP-binding domain-containing protein 1	Eftud1	3332	15	15	15	17.7	17.7	17.7	125.78	0.0000	59.071	15644000	169	14.08	14.79	15.43	14.84	15.05	15.45	15.15	15.23
1.507	-0.43	0.74	P49586	Choline-phosphate cytidylyltransferase A	Pcyt1a	1165	23	23	15	65.1	65.1	45.8	41.666	0.0000	156.04	57034000	529	16.20	16.45	16.43	16.65	16.59	16.94	16.76	17.17
0.230	-0.44	0.74	Q9JIG8	PRA1 family protein 2	Praf2	5399	3	3	3	21.3	21.3	21.3	19.478	0.0000	26.795	7546900	52	14.00	14.36	15.69	15.64	15.75	13.48	16.08	16.13
2.361	-0.44	0.74	P62270	40S ribosomal protein S18	Rps18	1413	25	25	25	70.4	70.4	70.4	17.718	0.0000	323.31	526050000	1005	19.89	19.48	19.79	19.76	20.12	20.30	20.10	20.15
1.485	-0.44	0.74	Q9CPV4	Glyoxalase domain-containing protein 4	Glod4	4538	20	20	20	67.4	67.4	67.4	33.316	0.0000	269.38	92346000	586	16.73	17.01	16.95	17.35	17.17	17.53	17.57	17.53
1.451	-0.44	0.74	Q61171	Peroxiredoxin-2	Prdx2	2267	12	11	11	57.6	52	52	21.778	0.0000	323.31	524580000	738	19.84	20.41	20.16	20.06	20.88	20.43	20.51	20.40
0.742	-0.44	0.74	Q8BGX2	Uncharacterized protein C19orf52 homolog		3000	5	5	5	30.5	30.5	30.5	29.415	0.0000	95.396	4579800	66	13.95	13.86	14.31	14.08	14.73	14.75	14.82	13.67
0.418	-0.44	0.74	Q811M1	Rho GTPase-activating protein 15	Arhgap15	2938	5	5	5	12.7	12.7	12.7	55.339	0.0000	9.4624	3374300	39	14.25	13.67	14.69	13.97	15.33	14.13	13.66	15.24
0.366	-0.44	0.74	Q8R4Y4	Stabilin-1	Stab1	3876	21	21	21	10.6	10.6	10.6	276.25	0.0000	79.591	19707000	221	14.75	14.56	14.99	14.63	16.03	15.53	15.46	13.68
0.379	-0.44	0.74	Q80XL7	Metallophosphoesterase 1	Mppe1	2886	1	1	1	4	4	4	46.012	0.0002	5.3276	1058000	13	13.19	12.62	12.39	12.77	13.58	13.51	11.76	13.88
1.247	-0.44	0.74	Q6ZWN5	40S ribosomal protein S9	Rps9	2703	25	25	25	66.5	66.5	66.5	22.591	0.0000	110.19	384080000	854	19.59	19.09	19.37	19.49	19.86	19.78	19.46	20.21
0.857	-0.44	0.73	Q99KG3	RNA-binding protein 10	Rbm10	4374	11	10	10	19.9	18.7	18.7	103.49	0.0000	24.586	3990300	41	13.23	13.59	13.10	13.58	13.16	13.90	14.19	14.03
0.936	-0.45	0.73	Q7TSC1	Protein PRRC2A	Prrc2a	2780	5	4	4	3.4	3.1	3.1	229.2	0.0000	23.349	2409900	38	13.41	13.44	13.20	13.87	13.70	14.35	14.15	13.50
2.461	-0.45	0.73	Q9CSU0	Regulation of nuclear pre-mRNA domain-containing protein 1B	Rprd1b	4700	12	12	12	46.3	46.3	46.3	36.883	0.0000	147.04	19612000	202	15.67	15.48	15.79	15.80	16.30	16.14	16.05	16.04
0.513	-0.45	0.73	P62743	AP-2 complex subunit sigma	Ap2s1	1439	5	5	5	27.5	27.5	27.5	17.018	0.0000	8.9936	10934000	106	14.40	15.65	15.90	15.38	16.19	16.11	15.21	15.60
1.227	-0.45	0.73	Q9JIY0	Pleckstrin homology domain-containing family O member 1	Plekho1	5411	12	12	12	30.4	30.4	30.4	45.996	0.0000	41.883	16827000	118	15.11	15.60	15.38	15.74	15.94	15.89	16.23	15.57
1.820	-0.45	0.73	P51859;Q9J	Hepatoma-derived growth factor	Hdgf	1205	6	5	5	22.8	19.4	19.4	26.268	0.0000	18.572	8521100	80	14.62	15.04	14.56	14.99	15.32	15.21	15.13	15.34
1.410	-0.45	0.73	O08553;Q62	Dihydropyrimidinase-related protein 2	Dpysl2	272	47	47	42	80.6	80.6	78.5	62.277	0.0000	323.31	903250000	1997	19.44	19.77	19.66	19.67	20.31	20.40	19.84	19.80
2.145	-0.45	0.73	P50580	Proliferation-associated protein 2G4	Pa2g4	1188	37	37	37	67	67	67	43.698	0.0000	323.31	211400000	936	18.31	18.19	18.32	18.28	18.67	18.86	18.45	18.94
1.053	-0.45	0.73	P70248	Unconventional myosin-If	Myo1f	1539	59	59	50	46.2	46.2	41.7	125.95	0.0000	323.31	314820000	1454	18.24	17.97	17.90	18.16	18.09	19.00	18.27	18.73
1.925	-0.46	0.73	Q9DBZ5	Eukaryotic translation initiation factor 3 subunit K	Eif3k	5185	10	10	10	50.5	50.5	50.5	25.086	0.0000	63.039	49424000	228	16.58	16.66	16.91	16.74	17.14	17.12	17.48	16.98
0.868	-0.46	0.73	Q8K2Z4	Condensin complex subunit 1	Ncapd2	3681	20	20	20	22.2	22.2	22.2	155.66	0.0000	123.43	16826000	158	14.82	14.76	15.63	15.16	15.52	16.03	15.40	15.23
0.652	-0.46	0.73	Q9CPT3	N-acylneuraminate-9-phosphatase	Nanp	4532	6	6	6	31.9	31.9	31.9	27.808	0.0000	22.441	6825900	62	15.43	15.35	15.11	15.33	16.03	16.26	14.79	15.97
0.661	-0.46	0.73	Q9JJ89	Coiled-coil domain-containing protein 86	Ccdc86	5420	4	4	4	9.4	9.4	9.4	46.486	0.0002	5.6567	1593700	6	12.15	12.74	13.37	12.83	13.66	13.37	12.63	13.27
1.478	-0.46	0.73	Q99LC5	Electron transfer flavoprotein subunit alpha, mitochondrial	Etfa	4417	31	31	31	79.6	79.6	79.6	35.009	0.0000	323.31	300480000	942	18.75	18.99	18.96	19.28	19.10	19.47	19.56	19.68
0.805	-0.46	0.73	Q9D8T7	SRA stem-loop-interacting RNA-binding protein, mitochondrial	Slirp	5074	8	8	8	68.8	68.8	68.8	12.605	0.0000	108.84	51728000	299	15.90	16.75	16.06	16.89	16.51	16.79	16.94	17.18
0.997	-0.46	0.73	Q91VD9	NADH-ubiquinone oxidoreductase 75 kDa subunit, mitochondrial	Ndufs1	4057	43	43	43	61.9	61.9	61.9	79.776	0.0000	323.31	166230000	939	17.85	17.73	17.98	17.69	18.83	18.15	18.36	17.74
0.585	-0.46	0.73	P62855	40S ribosomal protein S26	Rps26	1455	4	4	4	27	27	27	13.015	0.0000	141.76	62230000	153	18.77	18.18	18.13	18.31	18.50	19.72	18.87	18.14
0.316	-0.46	0.73	Q9JIX0	Transcription and mRNA export factor ENY2	Eny2	5409	2	2	2	25.7	25.7	25.7	11.528	0.0009	3.7395	2680600	28	12.98	12.22	13.40	11.78	14.17	13.28	11.77	13.02
1.312	-0.46	0.73	Q8BHL3	TBC1 domain family member 10B	Tbc1d10b	3044	13	13	13	24.6	24.6	24.6	87.274	0.0000	25.141	13038000	96	15.24	15.23	14.82	15.23	15.42	16.06	15.47	15.41
1.254	-0.46	0.73	P26231	Catenin alpha-1	Ctnna1	920	24	24	22	43	43	39.2	100.11	0.0000	260.51	20505000	219	15.11	14.94	15.24	15.25	15.15	15.92	15.45	15.86
1.205	-0.46	0.73	Q9CQF0	39S ribosomal protein L11, mitochondrial	Mrpl11	4592	10	10	10	59.4	59.4	59.4	20.68	0.0000	47.207	28152000	252	15.86	15.98	16.02	16.11	16.00	16.27	16.84	16.71
1.956	-0.46	0.73	Q6NVF9	Cleavage and polyadenylation specificity factor subunit 6	Cpsf6	2535	12	12	12	26.5	26.5	26.5	59.152	0.0000	323.31	52342000	378	17.29	17.45	16.98	17.51	17.77	17.88	17.66	17.77
0.721	-0.47	0.72	P63260	Actin, cytoplasmic 2;Actin, cytoplasmic 2, N-terminally processed	Actg1	1499	59	4	3	99.5	7.5	7.5	41.792	0.0000	312.15	467310000	571	21.39	21.23	21.72	21.96	21.94	22.77	21.47	21.98
1.246	-0.47	0.72	Q99L13	3-hydroxyisobutyrate dehydrogenase, mitochondrial	Hibadh	4403	18	18	18	63	63	63	35.44	0.0000	323.31	75549000	539	16.98	17.34	17.20	17.45	17.58	17.36	17.73	18.17

0.548	-0.47	0.72	Q9D2C2	Protein SAAL1	Saal1	4940	6	6	6	17.7	17.7	17.7	52.768	0.0000	8.3609	2012600	22	12.60	13.73	13.25	13.99	13.93	14.38	13.97	13.16
1.403	-0.47	0.72	Q99PL6	UBX domain-containing protein 6	Ubxn6	4508	21	21	21	55.7	55.7	55.7	49.795	0.0000	79.579	68308000	304	17.31	17.42	17.83	17.88	17.87	18.00	18.07	18.38
0.862	-0.47	0.72	Q99L47	Hsc70-interacting protein	St13	4409	16	16	16	35.6	35.6	35.6	41.655	0.0000	69.11	66067000	323	16.88	17.43	16.63	17.51	17.37	17.47	17.40	18.10
1.803	-0.47	0.72	P26039;Q71	Talin-1	Tln1	914	230	230	230	83.2	83.2	83.2	269.82	0.0000	323.31	4504900000	10924	20.37	20.40	20.81	20.79	20.87	21.05	21.11	21.22
0.677	-0.47	0.72	Q3ULW8		Parp3	1994	16	16	16	37.7	37.7	37.7	59.948	0.0000	98.377	14129000	121	14.57	14.24	15.56	14.77	15.28	15.75	15.14	14.85
1.120	-0.47	0.72	P97496	SWI/SNF complex subunit SMARCC1	Smarcc1	1664	13	4	4	13.4	6.7	6.7	122.89	0.0000	44.207	5854800	63	14.31	14.88	15.29	14.84	15.32	15.57	15.15	15.19
2.976	-0.47	0.72	Q8R361	Rab11 family-interacting protein 5	Rab11fip5	3837	23	23	23	49.1	49.1	49.1	69.552	0.0000	169.28	46578000	380	16.33	16.57	16.39	16.48	16.84	17.03	16.79	17.01
0.422	-0.47	0.72	Q9DBN9	Probable ATP-dependent RNA helicase DDX59	Ddx59	5166	3	3	3	6.6	6.6	6.6	68.234	0.0000	9.4085	1254700	11	12.36	14.48	13.14	13.24	13.09	13.85	14.05	14.13
1.437	-0.48	0.72	P10126	Elongation factor 1-alpha 1	Eef1a1	719	53	53	33	74.5	74.5	45.9	50.113	0.0000	323.31	6814600000	5720	23.50	23.45	23.13	23.52	23.54	23.79	23.91	24.27
0.435	-0.48	0.72	Q32NY4	Metal transporter CNNM3	Cnnm3	1807	5	5	5	12.8	12.8	12.8	76.278	0.0000	18.856	3789700	57	14.37	13.46	13.57	13.41	14.86	12.91	14.54	14.40
0.162	-0.48	0.72	Q6P2K6	Serine/threonine-protein phosphatase 4 regulatory subunit 3A	Smek1	2557	6	6	4	10.6	10.6	8	93.841	0.0000	21.423	11905000	66	12.33	16.47	16.56	17.10	16.72	15.51	16.08	16.06
1.111	-0.48	0.72	Q922Q1;Q9	Mitochondrial amidoxime reducing component 2	Marc2	4267	16	16	16	54.4	54.4	54.4	38.194	0.0000	102.51	42624000	269	16.39	16.45	17.14	16.26	17.01	17.10	16.77	17.27
0.647	-0.48	0.72	Q99M51;O5	Cytoplasmic protein NCK1	Nck1	4456	14	14	13	43.2	43.2	40.8	42.89	0.0000	34.524	11731000	99	13.89	15.21	14.19	14.68	14.85	15.01	14.53	15.50
0.697	-0.48	0.72	Q8VDQ9	Protein KRI1 homolog	Kri1	3969	11	11	11	21.7	21.7	21.7	82.056	0.0000	14.553	5698500	28	14.06	14.25	13.37	13.89	14.35	14.69	13.60	14.83
0.672	-0.48	0.72	Q91YU8	Suppressor of SWI4 1 homolog	Ppan	4181	7	7	7	16.8	16.8	16.8	52.755	0.0000	23.813	5831100	71	14.43	14.38	14.06	15.15	14.31	15.53	14.94	15.15
1.951	-0.48	0.72	P34022	Ran-specific GTPase-activating protein	Ranbp1	1005	11	11	11	52.2	52.2	52.2	23.596	0.0000	71.201	35928000	289	16.64	16.63	16.51	16.48	17.13	17.35	16.76	16.95
0.621	-0.48	0.72	Q80TQ5	Pleckstrin homology domain-containing family M member 2	Plekhm2	2813	5	5	5	7.6	7.6	7.6	112.73	0.0000	20.768	1408800	11	14.23	12.76	13.24	13.27	13.83	14.14	14.17	13.29
1.816	-0.48	0.72	Q9R0M6	Ras-related protein Rab-9A	Rab9a	5629	7	7	7	46.8	46.8	46.8	22.909	0.0000	101.85	19436000	137	15.92	16.05	16.47	16.43	16.57	16.71	16.76	16.74
1.350	-0.48	0.72	P26369	Splicing factor U2AF 65 kDa subunit	U2af2	923	16	16	16	47.6	47.6	47.6	53.516	0.0000	260.47	78503000	398	17.75	18.19	17.54	18.20	18.18	18.64	18.40	18.38
1.436	-0.48	0.72	Q62422	Osteoclast-stimulating factor 1	Ostf1	2389	17	17	17	72.6	72.6	72.6	23.782	0.0000	257.33	104710000	487	16.94	17.32	17.49	17.77	17.93	17.96	17.82	17.73
0.313	-0.48	0.72	Q9D8M4	60S ribosomal protein L7-like 1	Rpl7l1	5064	4	4	4	21.1	21.1	21.1	28.543	0.0000	12.566	4609900	68	15.19	13.24	15.48	13.50	15.46	14.52	14.12	15.23
0.852	-0.48	0.72	P0DOV1			714	27	16	5	64.5	39.3	14.4	46.977	0.0000	180.86	53775000	382	17.34	17.45	16.91	16.78	17.77	18.18	17.35	17.12
1.265	-0.48	0.71	Q9Z0H3	SWI/SNF-related matrix-associated actin-dependent regulator of chroma	Smarcb1	5770	12	12	12	43.4	43.4	43.4	44.141	0.0000	210.75	18032000	200	15.21	15.67	15.77	15.87	15.76	16.01	16.35	16.33
0.553	-0.49	0.71	Q09XV5	Chromodomain-helicase-DNA-binding protein 8	Chd8	1773	8	8	7	6	6	5.6	290.84	0.0000	24.798	3860000	56	13.45	13.56	14.23	14.78	13.73	14.95	14.73	14.55
1.708	-0.49	0.71	P08556	GTPase NRas	Nras	686	14	14	4	82	82	30.2	21.199	0.0000	140.53	33021000	246	16.25	16.53	16.64	16.88	16.84	17.14	17.03	17.22
1.936	-0.49	0.71	P83741;Q80	Serine/threonine-protein kinase WNK1	Wnk1	1601	17	17	12	10.6	10.6	8	250.93	0.0000	27.525	9532700	94	14.49	14.33	14.69	14.76	14.77	15.17	15.13	15.16
1.394	-0.49	0.71	P38647	Stress-70 protein, mitochondrial	Hspa9	1050	50	50	50	67.2	67.2	67.2	73.46	0.0000	323.31	526750000	2045	18.32	18.84	18.24	18.90	18.89	19.05	19.08	19.26
1.470	-0.49	0.71	Q9CQ10	Charged multivesicular body protein 3	Chmp3	4552	8	8	8	34.8	34.8	34.8	25.219	0.0000	20.34	21687000	137	15.88	15.96	15.87	16.17	16.30	16.09	16.82	16.65
0.877	-0.49	0.71	Q9CQP0	39S ribosomal protein L33, mitochondrial	Mrpl33	4618	4	4	4	53.8	53.8	53.8	7.4158	0.0000	7.3798	6960300	80	15.00	14.80	15.03	15.17	15.02	16.24	15.57	15.15
1.400	-0.50	0.71	P50518;Q9D	V-type proton ATPase subunit E 1	Atp6v1e1	1185	34	34	34	84.5	84.5	84.5	26.157	0.0000	323.31	526470000	1135	19.76	19.67	20.12	20.03	19.93	20.48	20.55	20.61
2.553	-0.50	0.71	Q8VD58	Protein EVI2B	Evi2b	3940	5	5	5	11	11	11	47.993	0.0000	19.254	10913000	134	14.93	14.87	15.27	15.01	15.49	15.57	15.39	15.62
1.561	-0.50	0.71	P61957	Small ubiquitin-related modifier 2	Sumo2	1392	4	4	3	33.7	33.7	21.1	10.871	0.0000	14.2	44197000	92	18.48	18.27	18.07	18.35	18.52	18.88	18.59	19.16
0.234	-0.50	0.71	Q3U5F4	YrdC domain-containing protein, mitochondrial	Yrdc	1926	3	3	3	15.4	15.4	15.4	29.454	0.0004	4.4616	2269500	22	12.38	12.49	15.28	13.65	15.52	13.33	13.58	13.35
1.015	-0.50	0.71	Q9Z130	Heterogeneous nuclear ribonucleoprotein D-like	Hnrnpdl	5798	12	10	10	52.5	48.2	48.2	33.558	0.0000	74.51	15793000	150	15.06	15.76	15.07	14.97	15.90	15.41	15.42	16.12
0.694	-0.50	0.71	Q9DBM1	G patch domain-containing protein 1	Gpatch1	5163	10	10	10	15.7	15.7	15.7	103.01	0.0000	72.75	4248600	37	13.13	13.60	14.13	14.00	14.03	14.96	13.71	14.16
1.453	-0.50	0.71	P35700	Peroxiredoxin-1	Prdx1	1029	33	33	21	99.5	99.5	80.9	22.176	0.0000	323.31	3129500000	2868	21.54	22.31	22.05	22.05	22.37	22.30	22.70	22.58
1.322	-0.50	0.71	Q9CQW1	Synaptobrevin homolog YKT6	Ykt6	4642	19	19	19	85.4	85.4	85.4	22.314	0.0000	262.84	32124000	231	16.27	16.00	16.32	15.96	16.33	16.70	16.39	17.11
1.038	-0.50	0.71	Q8VE37	Regulator of chromosome condensation	Rcc1	3985	14	14	14	53	53	53	44.93	0.0000	323.31	42334000	279	16.58	16.96	16.85	17.38	17.25	17.72	17.02	17.78
1.596	-0.50	0.71	Q9EQP2	EH domain-containing protein 4	Ehd4	5285	43	39	35	81.1	77.6	73.6	61.48	0.0000	323.31	558670000	1342	19.16	19.44	19.56	19.02	19.60	20.11	19.66	19.81
0.641	-0.50	0.71	Q9WV27	Sodium/potassium-transporting ATPase subunit alpha-4	Atp1a4	5729	14	1	1	11.9	1.5	1.5	114.89	0.0004	4.8234	6899200	51	14.97	15.67	14.55	14.46	15.88	14.92	15.82	15.05
0.456	-0.50	0.71	Q8BGS2	BolA-like protein 2	Bola2	2991	7	7	7	77.9	77.9	77.9	10.214	0.0000	24.338	8532200	95	15.88	14.14	15.54	15.33	15.22	16.37	15.13	16.17
0.382	-0.50	0.71	Q60972	Histone-binding protein RBBP4	Rbbp4	2231	11	4	4	29.6	19.3	19.3	47.655	0.0000	72.561	8681700	57	15.64	17.01	14.76	15.98	16.75	17.05	15.53	16.07
1.293	-0.50	0.70	Q62433	Protein NDRG1	Ndrg1	2393	10	10	10	40.1	40.1	40.1	43.008	0.0000	93.979	31286000	154	16.67	17.19	16.46	16.70	17.01	17.27	17.11	17.64
1.180	-0.51	0.70	Q04857	Collagen alpha-1(VI) chain	Col6a1	1735	5	5	5	7.4	7.4	7.4	108.49	0.0000	37.919	4404800	69	14.06	14.05	14.86	14.58	15.04	14.95	15.00	14.58
1.296	-0.51	0.70	O88291	DBIRD complex subunit ZNF326	Znf326	521	9	9	9	22.4	22.4	22.4	65.225	0.0000	190.91	15667000	140	15.60	15.57	15.92	16.01	16.02	16.63	15.94	16.53
1.127	-0.51	0.70	Q8CIE4		Parp10	3549	6	6	6	10.9	10.9	10.9	103.68	0.0000	18.857	3247500	22	13.99	13.37	13.78	13.74	14.45	14.67	13.99	13.81
1.507	-0.51	0.70	P97376	Protein FRG1	Frg1	1638	12	12	12	37.2	37.2	37.2	29.127	0.0000	31.107	19157000	159	16.07	15.99	15.73	15.78	15.98	16.60	16.33	16.70
0.359	-0.51	0.70	Q9JI13	Something about silencing protein 10	Utp3	5383	3	3	3	9.6	9.6	9.6	53.398	0.0000	8.6125	2569600	21	13.30	13.74	13.32	14.89	12.96	15.28	14.48	14.57
2.286	-0.51	0.70	P62702	40S ribosomal protein S4, X isoform	Rps4x	1436	34	34	34	79.5	79.5	79.5	29.597	0.0000	323.31	663610000	1226	19.80	19.52	19.40	19.64	19.91	20.02	20.24	20.23
1.978	-0.51	0.70	Q9CQA9	Cancer-related nucleoside-triphosphatase homolog	Ntpcr	4580	11	11	11	79.5	79.5	79.5	20.667	0.0000	104.02	46362000	264	16.89	16.83	17.23	17.28	17.34	17.66	17.68	17.59
1.239	-0.51	0.70	P58404	Striatin-4	Strn4	1315	8	5	5	12.9	10.1	10.1	81.644	0.0000	12.975	2885900	28	13.55	14.50	13.92	14.26	14.57	14.50	14.77	14.43
0.332	-0.51	0.70	Q69ZQ2	Pre-mRNA-splicing factor ISY1 homolog	Isy1	2489	3	3	3	9.8	9.8	9.8	32.989	0.0000	32.098	5195500	104	13.83	14.95	12.84	14.80	13.85	14.42	14.32	15.88
0.115	-0.51	0.70	Q06830	Peroxiredoxin-1	PRDX1	1752	18	6	4	69.3	32.2	23.1	22.11	0.0000	23.876	77606000	26	17.55	19.75	17.33	14.88	19.35	13.93	19.01	19.27
1.158	-0.52	0.70	O08997	Copper transport protein ATOX1	Atox1	314	5	5	5	69.1	69.1	69.1	7.3384	0.0000	102.72	33510000	251	16.35	16.95	16.30	16.83	16.91	17.56	17.20	16.83
0.908	-0.52	0.70	Q68ED3	Non-canonical poly(A) RNA polymerase PAPD5	Papd5	2471	2	2	2	5.1	5.1	5.1	69.703	0.0000	13.453	579770	35	12.46	12.19	12.44	13.00	13.18	13.61	12.51	12.86
0.800	-0.52	0.70	Q9CQY5	Magnesium transporter protein 1	Magt1	4648	10	10	9	22.7	22.7	22.7	37.969	0.0000	27.617	23829000	120	15.78	16.90	17.14	16.48	17.09	16.79	17.37	17.11
1.326	-0.52	0.70	Q64343	ATP-binding cassette sub-family G member 1	Abcg1	2434	11	11	11	20.9	20.9	20.9	74.032	0.0000	75.184	7237100	65	13.95	14.47	14.34	14.19	14.93	14.23	14.96	14.90
0.445	-0.52	0.70	P56812	Programmed cell death protein 5	Pdcd5	1292	3	3	3	28.6	28.6	28.6	14.275	0.0000	6.3996	1543800	11	13.54	12.37	12.41	13.74	13.94	13.41	14.25	12.54
0.461	-0.52	0.70	Q6DVA0	LEM domain-containing protein 2	Lemd2	2512	7	7	7	20.5	20.5	20.5	57.506	0.0000	37.6	17640000	119	14.54	15.27	16.65	16.36	15.86	16.21	16.49	16.34
1.583	-0.52	0.70	P62075	Mitochondrial import inner membrane translocase subunit Tim13	Timm13	1402	3	3	3	53.7	53.7	53.7	10.458	0.0000	44.621	2328900	20	13.51	13.63	13.63	13.47	13.70	14.36	13.89	14.39
0.572	-0.52	0.70	Q9D2R6	Cytochrome c oxidase assembly factor 3 homolog, mitochondrial	Coa3	4950	5	5	5	40.7	40.7	40.7	11.987	0.0000	21.38	13056000	68	15.62	14.41	16.18	14.57	15.80	15.68	15.74	15.63
1.636	-0.52	0.70	Q62448	Eukaryotic translation initiation factor 4 gamma 2	Eif4g2	2396	32	32	32	42.6	42.6	42.6	102.1	0.0000	323.31	115070000	780	17.09	17.07	17.06	17.32	17.23	17.76	17.64	17.99
1.174	-0.52	0.70	Q3UDR8	Protein YIPF3;Protein YIPF3, N-terminally processed	Yipf3	1950	7	7	7	23.3	23.3	23.3	37.998	0.0000	28.135	7553900	43	14.24	14.77	15.26	14.72	15.26	15.19	15.55	15.06
0.944	-0.52	0.70	Q9CR47	Ribosome biogenesis protein NSA2 homolog	Nsa2	4665	6	6	6	26.9	26.9	26.9	30.036	0.0027	2.6928	2129900	13	13.07	13.16	12.89	13.83	13.32	14.04	13.54	14.13
1.621	-0.52	0.70	P62082	40S ribosomal protein S7	Rps7	1403	28	28	28	84	84	84	22.127	0.0000	323.31	873660000	1180	21.09	21.05	21.10	21.21	21.22	21.49	21.89	21.94
0.516	-0.52	0.70	Q8K3J1	NADH dehydrogenase [ubiquinone] iron-sulfur protein 8, mitochondrial	Ndufs8	3703	12	12	12	45.8	45.8	45.8	24.038	0.0000	45.874	32180000	311	16.32	16.41	15.19	15.85	17.48	15.87	16.56	15.95
0.393	-0.52	0.70	Q60648	Ganglioside GM2 activator	Gm2a	2188	3	3	3	24.4	24.4	24.4	20.824	0.0000	28.955	28250000	54	17.98	19.19	16.92	17.03	18.51	17.80	18.92	17.99
1.268	-0.52	0.70	P25444	40S ribosomal protein S2	Rps2	907	25	25	25	63.5	63.5	63.5	31.231	0.0000	242.4	503740000	937	20.12	19.80	19.80	19.79	20.11	20.66	20.01	20.84
1.764	-0.52	0.70	Q9CQV7	Mitochondrial import inner membrane translocase subunit TIM14	Dnajc19	4640	10	10	10	78.4	78.4	78.4	12.436	0.0000	100.84	32884000	308	16.45	16.13	16.40	16.34	16.69	17.24	16.58	16.91
1.245	-0.52	0.69	P45481	CREB-binding protein	Crebbp	1096	5	4	4	3.5	2.8	2.8	265.49	0.0000	57.92	3150500	41	13.37	14.13	14.26	14.12	14.23	14.66	14.50	14.58
1.682	-0.53	0.69	Q61191;Q9D	Host cell factor 1;HCF N-terminal chain 1;HCF N-terminal chain 2;HCF I	Hcfc1	2272	38	38	38	27.3	27.3	27.3	210.43	0.0000	286.01	103050000	694	17.16	17.13	17.11	17.54	17.49	18.07	17.59	17.90
0.832	-0.53	0.69	Q921I2	Kelch domain-containing protein 4	Klhdc4	4227	4	4	4	13.7	13.7	13.7	64.86	0.0000	37.104	2097500	35	13.53	12.20	12.70	12.24	13.19	13.18	13.05	13.35
1.104	-0.53	0.69	Q9R207	Nibrin	Nbn	5668	4	4	4	6.7	6.7	6.7	83.794	0.0000	22.445	4898200	67	15.13	14.33	14.32	15.14	15.39	15.24	15.01	15.40
2.785	-0.53	0.69	Q9D1P4	Cysteine and histidine-rich domain-containing protein 1	Chordc1	4930	11	11	11	41.4	41.4	41.4	37.35	0.0000	81.619	25968000	212	15.66	15.86	16.02	15.85	16.37	16.55	16.35	16.23
1.722	-0.53	0.69	P70315	Wiskott-Aldrich syndrome protein homolog	Was	1552	18	18	17	40	40	37.1	54.191	0.0000	323.31	42928000	242	16.70	17.00	17.04	17.24	17.49	17.51	17.25	17.85
1.034	-0.53	0.69	E9Q1P8	Interferon regulatory factor 2-binding protein 2	Irf2bp2	228	12	12	11	28.9	28.9	27.7	59.291	0.0000	74.301	11372000	134	14.74	15.16	14.53	15.28	15.38	15.11	15.30	16.03
0.944	-0.53	0.69	Q9JIK9	28S ribosomal protein S34, mitochondrial	Mrps34	5404	10	10	10	52.8	52.8	52.8	25.826	0.0000	45.739	29164000	92	17.41	17.37	17.76	17.65	18.21	17.35	18.10	18.66
1.103	-0.53	0.69	P23506	Protein-L-isoaspartate(D-aspartate) O-methyltransferase	Pcmt1	885	16	16	16	73.1	73.1	73.1	24.634	0.0000	309.04	187150000	676	18.66	19.42	18.76	19.49	19.38	19.51	19.60	19.97
1.446	-0.53	0.69	P62717	60S ribosomal protein L18a	Rpl18a	1438	22	22	22	75	75	75	20.732	0.0000	132.18	204140000	770	19.30	19.04	18.79	18.45	19.30	19.64	19.31	19.47
1.155	-0.53	0.69	Q9D0F4;Q5	NF-kappa-B-activating protein	Nkap	4863	5	5	5	18.8	18.8	18.8	47.227	0.0000	6.687	4171700	30	13.84	14.35	13.96	13.35	14.13	14.57	14.27	14.68
1.297	-0.54	0.69	Q9EST5	Acidic leucine-rich nuclear phosphoprotein 32 family member B	Anp32b	5345	15	15	11	32	32	25.4	31.078	0.0000	171.58	155240000	472	18.27	18.65	18.64	18.99	18.69	19.32	19.29	19.39
1.829	-0.54	0.69	Q9CZX8	40S ribosomal protein S19	Rps19	4846	23	23	23	73.1	73.1	73.1	16.085	0.0000	214.68	389850000	688	19.54	19.23	19.43	19.76	19.72	19.99	20.17	20.23
1.505	-0.54	0.69	Q9Z1X4;Q9	Interleukin enhancer-binding factor 3	Ilf3	5825	22	22	22	27.6	27.6	27.6	96.02	0.0000	168.33	55039000	413	16.21	16.15	16.38	16.44	16.52	17.25	16.54	17.03
1.575	-0.54	0.69	Q3TIV5	Zinc finger CCCH domain-containing protein 15	Zc3h15	1851	10	10	10	21.1	21.1	21.1	48.327	0.0000	20.268	8888400	120	14.17	14.35	13.90	14.46	14.65	14.84	14.44	15.09
2.247	-0.54	0.69	P80315	T-complex protein 1 subunit delta	Cct4	1592	44	44	44	87.4	87.4	87.4	58.066	0.0000	323.31	761510000	2046	19.64	19.66	19.82	19.75	19.98	20.48	20.12	20.44
0.710	-0.54	0.69	Q8BRK9	Alpha-mannosidase 2x	Man2a2	3182	4	4	4	5.9	5.9	5.9	130.65	0.0018	3.1326	754640	4	12.60	12.29	12.71	12.11	11.97	13.24	13.11	13.54
0.895	-0.54	0.69	P26883	Peptidyl-prolyl cis-trans isomerase FKBP1A	Fkbp1a	929	6	6	6	59.3	59.3	59.3	11.922	0.0000	33.774	31960000	137	17.38	17.93	16.92	17.79	17.77	18.36	17.62	18.42
1.814	-0.54	0.69	Q99M87	DnaJ homolog subfamily A member 3, mitochondrial	Dnaja3	4459	12	12	11	31.9	31.9	30.2	52.443	0.0000	62.281	19243000	149	16.20	15.92	15.64	15.65	16.14	16.39	16.49	16.55
1.108	-0.54	0.69	Q9Z2U1	Proteasome subunit alpha type-5	Psma5	5865	10	10	10	56.8	56.8	56.8	26.411	0.0000	207.01	49333000	218	16.66	17.74	17.43	17.49	17.59	17.91	18.09	17.90
0.927	-0.54	0.69	P14434;P14	H-2 class II histocompatibility antigen, A-B alpha chain;H-2 class II histo	H2-Aa	780	6	6	6	27	27	27	28.093	0.0000	116.82	21872000	125	15.34	16.38	16.14	16.61	16.94	16.67	16.38	16.66
1.480	-0.54	0.69	Q8CGF7;Q3	Transcription elongation regulator 1	Tcerg1	3508	41	41	40	38.6	38.6	38.4	123.79	0.0000	160.41	59994000	434	15.67	15.91	15.52	16.14	16.24	16.36	16.07	16.73
1.274	-0.54	0.69	Q4KMM3	Oxidation resistance protein 1	Oxr1	2062	16	16	16	26.6	26.6	26.6	95.911	0.0000	160.86	10938000	151	14.23	15.05	14.68	14.62	14.74	15.29	15.28	15.45
1.480	-0.55	0.69	Q9CRC8	Leucine-rich repeat-containing protein 40	Lrrc40	4690	22	22	22	50.8	50.8	50.8	68.075	0.0000	86.102	29685000	340	15.24	15.45	15.81	15.62	15.77	16.28	15.85	16.40
0.259	-0.55	0.68	P56391	Cytochrome c oxidase subunit 6B1	Cox6b1	1284	3	3	3	53.5	53.5	53.5	10.071	0.0004	4.8241	8940800	32	15.91	16.13	13.40	15.18	14.88	14.73	15.84	17.37
1.208	-0.55	0.68	P39749	Flap endonuclease 1	Fen1	1055	18	18	18	48.4	48.4	48.4	42.314	0.0000	149.3	82708000	419	16.93	17.15	17.18	17.36	17.38	18.35	17.44	17.64
1.933	-0.55	0.68	Q8K354	Carbonyl reductase [NADPH] 3	Cbr3	3690	27	27	26	84.5	84.5	81.6	30.953	0.0000	260.56	141670000	755	17.80	17.89	18.02	18.24	18.32	18.75	18.33	18.74
0.916	-0.55	0.68	O35344	Importin subunit alpha-4	Kpna3	359	16	11	11	41.8	33	33	57.772	0.0000	201.6	20523000	119	17.01	16.58	16.52	17.38	16.78	17.41	17.80	17.69
0.667	-0.55	0.68	Q99PP6	Tripartite motif-containing protein 34A	Trim34a	4511	11	11	11	29.3	29.3	29.3	55.907	0.0000	71.578	4325300	35	12.74	13.00	12.50	12.42	14.09	13.13	12.27	13.38
1.329	-0.55	0.68	Q05816;RE\	Fatty acid-binding protein, epidermal	Fabp5	1742	23	23	23	91.1	91.1	91.1	15.137	0.0000	323.31	734750000	1776	19.74	20.16	19.63	20.20	20.01	20.56	20.55	20.81
1.662	-0.55	0.68	P19536	Cytochrome c oxidase subunit 5B, mitochondrial	Cox5b	848	7	7	7	43	43	43	13.813	0.0004	5.1791	8074300	26	15.66	15.55	15.21	15.87	16.47	15.94	16.01	16.08
0.641	-0.55	0.68	Q3THW5;P0	Histone H2A.V;Histone H2A.Z	H2afv;H2afz	705	9	7	7	64.8	57.8	57.8	13.509	0.0000	323.31	472960000	1246	20.68	20.57	19.58	20.47	20.23	20.74	20.76	21.79
1.169	-0.56	0.68	Q9CZM2	60S ribosomal protein L15	Rpl15	4833	17	17	17	59.8	59.8	59.8	24.146	0.0000	93.054	126080000	358	18.77	18.54	18.53	18.51	18.86	19.65	18.62	19.45
2.692	-0.56	0.68	P59759	MKL/myocardin-like protein 2	Mkl2	1343	10	10	9	13.1	13.1	11.9	117.55	0.0000	25.122	4337100	33	13.67	13.75	13.88	13.96	14.14	14.52	14.47	14.36
1.007	-0.56	0.68	O54988	STE20-like serine/threonine-protein kinase	Slk	440	53	53	50	44.6	44.6	43	141.46	0.0000	323.31	110100000	1208	15.71	16.03	16.26	16.67	16.28	16.73	16.63	17.25
0.472	-0.56	0.68	Q9QZH3	Peptidyl-prolyl cis-trans isomerase E	Ppie	5597	4	4	4	15	15	15	33.448	0.0004	4.5204	2060900	39	12.74	12.96	12.93	14.62	13.91	14.29	12.99	14.29
1.545	-0.56	0.68	P08905	Lysozyme C-2	Lyz2	690	9	9	4	54.7	54.7	41.2	16.689	0.0000	210.75	496610000	487	20.48	21.09	20.50	20.95	21.07	21.42	21.18	21.58
1.384	-0.56	0.68	Q8C181	Muscleblind-like protein 2	Mbnl2	3359	15	5	5	38.3	16.9	16.9	40.156	0.0000	70.847	15544000	108	16.03	16.23	16.57	16.67	16.52	16.96	16.98	17.27
2.232	-0.56	0.68	Q9JIX8	Apoptotic chromatin condensation inducer in the nucleus	Acin1	5410	29	29	29	24.7	24.7	24.7	150.72	0.0000	203.16	29029000	266	15.30	15.05	14.90	15.20	15.43	15.87	15.57	15.80
1.259	-0.56	0.68	Q9WV85	Nucleoside diphosphate kinase 3	Nme3	5738	7	7	7	63.3	63.3	63.3	19.099	0.0000	59.355	18159000	100	16.53	16.23	17.20	16.87	17.31	17.18	17.04	17.53
1.220	-0.56	0.68	P04441	H-2 class II histocompatibility antigen gamma chain	Cd74	642	13	13	13	42.7	42.7	42.7	31.557	0.0000	173.89	78060000	229	17.97	18.26	18.10	17.36	18.75	18.68	18.35	18.14
1.973	-0.56	0.68	Q3UDK1	TRAF-type zinc finger domain-containing protein 1	Trafd1	1948	7	7	7	15.7	15.7	15.7	64.282	0.0000	22.342	6208700	78	14.66	14.67	14.62	14.44	15.59	15.03	14.99	15.02
1.049	-0.56	0.68	Q6P9Q4	FH1/FH2 domain-containing protein 1	Fhod1	2589	22	22	22	28.2	28.2	28.2	129.6	0.0000	323.31	41198000	409	15.50	15.70	16.59	16.09	16.15	16.67	16.75	16.54
0.791	-0.56	0.68	Q8BGT6	MICAL-like protein 1	Micall1	2993	2	2	2	3.9	3.9	3.9	94.078	0.0000	7.4607	905860	14	13.25	12.47	13.39	13.21	14.10	13.10	13.19	14.16
1.143	-0.56	0.68	P70349	Histidine triad nucleotide-binding protein 1	Hint1	1558	12	12	12	93.7	93.7	93.7	13.777	0.0000	220.69	106640000	236	18.52	19.52	18.73	18.73	19.05	19.59	19.46	19.64

0.394	-0.56	0.68	Q7M759	Alpha/beta hydrolase domain-containing protein 17B	Abhd17b	2739	3	3	3	19.4	19.4	19.4	32.2	0.0000	14.96	2942600	26	14.02	14.39	14.61	12.15	14.41	13.58	14.90	14.52
0.971	-0.56	0.68	P61089;Q9C	Ubiquitin-conjugating enzyme E2 N	Ube2n	1371	14	14	14	79.6	79.6	79.6	17.138	0.0000	160.63	120760000	445	18.01	18.41	18.25	18.70	18.27	19.19	18.73	19.43
0.351	-0.56	0.68	Q925U4	ER degradation-enhancing alpha-mannosidase-like protein 1	Edem1	4312	3	3	3	8.9	8.9	8.9	73.7	0.0000	52.964	347100	6	12.07	12.05	14.05	11.88	12.70	12.56	12.59	14.43
1.678	-0.56	0.68	Q9Z204;Q8E	Heterogeneous nuclear ribonucleoproteins C1/C2	Hnrnpc	5828	25	25	25	58.5	58.5	58.5	34.384	0.0000	131.49	164770000	512	18.34	18.50	18.37	18.55	18.85	19.37	18.60	19.20
1.116	-0.56	0.68	Q9ESV0	ATP-dependent RNA helicase DDX24	Ddx24	5347	8	8	8	13.3	13.3	13.3	96.428	0.0000	14.806	4939700	76	14.25	13.97	13.50	13.91	13.94	14.66	14.34	14.93
3.089	-0.56	0.68	Q01853	Transitional endoplasmic reticulum ATPase	Vcp	1712	88	88	87	78.9	78.9	78.9	89.321	0.0000	323.31	1160100000	3210	20.15	20.40	20.22	20.46	20.83	20.75	20.88	21.01
1.476	-0.56	0.68	Q9Z1M8	Protein Red	Ik	5814	19	19	19	42.4	42.4	42.4	65.615	0.0000	106.81	45252000	348	16.40	16.50	16.16	16.72	16.73	17.23	16.71	17.37
1.875	-0.56	0.68	Q64105	Sepiapterin reductase	Spr	2417	12	12	12	54	54	54	27.883	0.0000	132.98	100610000	523	17.53	18.03	18.25	18.10	18.45	18.53	18.55	18.65
2.520	-0.57	0.68	P35979	60S ribosomal protein L12	Rpl12	1037	17	17	17	83	83	83	17.804	0.0000	323.31	304760000	838	18.99	19.31	19.50	19.30	19.77	19.73	19.91	19.95
1.148	-0.57	0.68	Q8BFU3	RING finger protein 214	Rnf214	2948	6	6	6	13	13	13	73.624	0.0000	13.299	3157700	28	13.87	13.71	13.59	12.94	13.76	14.27	13.90	14.44
0.728	-0.57	0.68	Q9D9M5	Pyridoxal phosphate phosphatase PHOSPHO2	Phospho2	5102	2	2	2	13.7	13.7	13.7	27.562	0.0018	3.1189	538580	6	12.91	12.73	13.03	12.43	13.81	12.49	14.03	13.03
1.607	-0.57	0.68	P63037	DnaJ homolog subfamily A member 1	Dnaja1	1482	24	24	24	65	65	65	44.868	0.0000	260.39	74628000	485	17.00	17.47	17.13	17.30	17.53	17.59	17.81	18.23
0.949	-0.57	0.68	P63158	High mobility group protein B1	Hmgb1	1493	29	22	22	74	57.2	57.2	24.893	0.0000	207.94	371440000	935	18.70	19.38	18.19	19.20	19.29	19.33	19.25	19.87
1.117	-0.57	0.68	Q8K3A9	7SK snRNA methylphosphate capping enzyme	Mepce	3698	6	6	6	18.3	18.3	18.3	72.05	0.0000	40.228	4370900	55	14.77	14.43	14.50	13.62	14.84	15.07	15.02	14.67
1.751	-0.57	0.67	P20029	78 kDa glucose-regulated protein	Hspa5	851	68	66	66	69.5	69.5	69.5	72.421	0.0000	323.31	1749500000	2902	20.46	21.09	20.51	20.92	21.18	21.56	21.24	21.28
1.601	-0.57	0.67	P37913	DNA ligase 1	Lig1	1047	33	33	33	44	44	44	102.29	0.0000	152.32	63234000	649	15.48	15.62	15.79	15.91	15.79	16.50	16.29	16.49
2.333	-0.57	0.67	Q64674	Spermidine synthase	Srm	2457	14	14	14	62.3	62.3	62.3	33.995	0.0000	190.92	31995000	275	16.18	16.04	16.39	16.20	17.03	16.84	16.51	16.71
1.583	-0.57	0.67	P29341	Polyadenylate-binding protein 1	Pabpc1	965	43	43	43	67.1	67.1	67.1	70.67	0.0000	305.31	243420000	1003	17.55	17.97	17.56	18.13	18.13	18.54	18.18	18.65
0.931	-0.57	0.67	Q9WUR2;Q7	Enoyl-CoA delta isomerase 2, mitochondrial	Eci2	5721	13	13	13	38.4	38.4	38.4	43.267	0.0000	58.743	14999000	144	15.98	14.62	15.62	15.53	16.04	15.82	15.84	16.32
0.762	-0.57	0.67	P19324	Serpin H1	Serpinh1	846	3	3	3	11.8	11.8	11.8	46.533	0.0000	50.303	1190400	35	11.72	11.88	11.72	12.49	13.12	12.95	11.72	12.30
0.553	-0.57	0.67	P61021	Ras-related protein Rab-5B	Rab5b	1364	11	7	7	66	47.9	47.9	23.707	0.0000	74.936	65302000	204	18.26	18.44	16.45	17.98	18.05	18.13	18.53	18.70
0.614	-0.57	0.67	Q8BIG7	Catechol O-methyltransferase domain-containing protein 1	Comtd1	3059	13	13	13	67.2	67.2	67.2	28.96	0.0000	178.93	35351000	254	16.92	16.26	16.92	16.05	17.36	16.31	16.73	18.05
0.448	-0.58	0.67	Q9CR95	Adaptin ear-binding coat-associated protein 1	Necap1	4683	5	5	5	24.4	24.4	24.4	29.639	0.0004	4.5864	5277600	27	14.92	15.02	15.33	12.82	15.01	15.20	15.07	15.11
0.555	-0.58	0.67	O35215	D-dopachrome decarboxylase	Ddt	346	9	9	9	86.4	86.4	86.4	13.077	0.0000	27.682	13702000	124	14.27	16.04	15.58	16.12	15.41	16.45	16.17	16.31
1.778	-0.58	0.67	Q99KJ8	Dynactin subunit 2	Dctn2	4379	21	21	21	60.2	60.2	60.2	44.116	0.0000	181.17	55145000	444	15.68	16.33	16.01	16.19	16.59	16.87	16.36	16.71
1.267	-0.58	0.67	Q8BH43;Q8	Wiskott-Aldrich syndrome protein family member 2	Wasf2	3009	25	25	25	37.8	37.8	37.8	54.073	0.0000	190.35	163500000	691	17.41	17.73	17.21	17.63	17.68	18.41	17.74	18.47
2.590	-0.58	0.67	P60335;P57	Poly(rC)-binding protein 1	Pcbp1	1354	23	23	17	94.1	94.1	82	37.497	0.0000	323.31	664580000	1379	19.81	20.27	20.30	20.15	20.70	20.76	20.62	20.77
1.512	-0.58	0.67	Q5BLK4	Terminal uridylyltransferase 7	Zcchc6	2095	19	19	19	17	17	17	169.1	0.0000	54.583	13271000	151	14.20	14.38	14.30	14.25	14.46	14.95	14.65	15.39
1.274	-0.59	0.67	Q60749;Q9V	KH domain-containing, RNA-binding, signal transduction-associated prc	Khdrbs1	2201	17	17	17	36.6	36.6	36.6	48.37	0.0000	104.41	57278000	346	16.94	17.29	16.95	17.70	17.37	18.07	17.73	18.05
1.877	-0.59	0.66	Q9CQ60	6-phosphogluconolactonase	Pgls	4564	16	16	16	95.7	95.7	95.7	27.254	0.0000	323.31	279570000	768	18.76	19.23	18.77	19.26	19.46	19.46	19.58	19.88
1.355	-0.59	0.66	P48024	Eukaryotic translation initiation factor 1	Eif1	1138	5	5	1	65.5	65.5	29.2	12.746	0.0000	134.84	19118000	122	15.87	16.63	16.81	16.07	16.86	16.90	17.13	16.86
1.412	-0.59	0.66	P23780	Beta-galactosidase	Glb1	889	23	23	22	35.2	35.2	33.4	73.12	0.0000	204.24	63321000	399	16.21	15.88	16.06	16.48	16.90	16.26	17.13	16.71
0.140	-0.59	0.66	Q8K072	Receptor expression-enhancing protein 4	Reep4	3598	5	5	4	26.1	26.1	21	29.69	0.0000	7.3357	12427000	37	12.47	13.35	17.24	17.24	16.44	12.71	16.84	16.68
0.564	-0.59	0.66	Q91V01	Lysophospholipid acyltransferase 5	Lpcat3	4039	3	3	3	7.8	7.8	7.8	56.146	0.0000	74.934	12372000	84	14.70	16.37	17.00	15.92	16.53	16.75	16.36	16.74
0.390	-0.60	0.66	O54962	Barrier-to-autointegration factor;Barrier-to-autointegration factor, N-term	Banf1	437	5	5	5	46.1	46.1	46.1	10.102	0.0000	28.593	12686000	78	16.04	16.54	16.12	14.17	16.74	17.04	16.30	15.18
1.228	-0.60	0.66	Q501J6	Probable ATP-dependent RNA helicase DDX17	Ddx17	2075	48	36	36	54.8	44.3	44.3	72.399	0.0000	323.31	175920000	970	17.74	17.77	17.48	17.53	18.19	18.88	17.69	18.14
1.501	-0.60	0.66	Q6P4S8	Integrator complex subunit 1	Ints1	2564	10	10	10	8	8	8	245.16	0.0000	73.524	5933600	104	14.15	13.93	13.70	14.16	14.75	14.04	14.66	14.86
0.456	-0.60	0.66	O54879	High mobility group protein B3	Hmgb3	429	17	15	15	58	53	53	23.01	0.0000	91.998	13268000	121	14.25	15.99	14.21	15.88	15.30	15.82	15.07	16.54
0.493	-0.60	0.66	Q9D7J4	Cytochrome c oxidase protein 20 homolog	Cox20	5032	4	4	4	43.6	43.6	43.6	13.163	0.0000	40.928	3665400	46	15.02	13.82	13.15	14.63	15.22	13.67	15.05	15.07
1.440	-0.60	0.66	Q923D2	Flavin reductase (NADPH)	Blvrb	4282	19	19	19	85.9	85.9	85.9	22.197	0.0000	323.31	546370000	1335	19.30	19.52	19.23	19.67	19.61	19.78	20.24	20.47
0.365	-0.60	0.66	Q9D898	Actin-related protein 2/3 complex subunit 5-like protein	Arpc5l	5056	4	4	4	26.1	26.1	26.1	16.98	0.0000	22.113	9363400	51	14.39	14.30	14.28	16.86	15.05	15.33	15.35	16.49
1.536	-0.60	0.66	Q9R0Q7	Prostaglandin E synthase 3	Ptges3	5638	6	6	5	39.4	39.4	32.5	18.721	0.0000	13.489	9969600	54	15.53	15.96	15.22	15.48	16.22	16.50	15.82	16.03
1.207	-0.60	0.66	Q9JIK5	Nucleolar RNA helicase 2	Ddx21	5403	36	36	36	53.1	53.1	53.1	93.55	0.0000	189.68	70790000	635	16.20	16.15	16.08	16.37	16.46	17.38	16.29	17.07
0.582	-0.60	0.66	Q9D6S7	Ribosome-recycling factor, mitochondrial	Mrrf	5008	8	8	8	46.2	46.2	46.2	29.05	0.0000	183.98	12519000	103	13.90	15.27	13.91	15.13	15.54	15.36	15.49	14.24
2.084	-0.60	0.66	Q8C052	Microtubule-associated protein 1S;MAP1S heavy chain;MAP1S light ch	Map1s	3325	26	26	26	31.8	31.8	31.8	102.94	0.0000	122.29	127530000	500	19.01	19.20	19.38	19.27	19.47	19.88	19.79	20.12
0.203	-0.60	0.66	Q9JL95	Proteoglycan 3	Prg3	5474	4	4	4	18.9	18.9	18.9	25.204	0.0000	6.9152	7877100	36	13.02	13.58	16.37	17.24	14.33	17.03	15.90	15.36
0.532	-0.60	0.66	Q9CQE8	UPF0568 protein C14orf166 homolog		4591	12	12	12	54.1	54.1	54.1	28.152	0.0000	63.859	22395000	228	15.65	16.20	14.23	16.40	15.77	16.22	16.23	16.68
1.228	-0.60	0.66	P23198	Chromobox protein homolog 3	Cbx3	880	5	5	4	36.6	36.6	27.9	20.855	0.0000	19.461	12422000	118	15.18	15.70	14.66	15.63	16.12	15.99	15.67	15.80
1.061	-0.61	0.66	Q9CR59	Growth arrest and DNA damage-inducible proteins-interacting protein 1	Gadd45gip1	4671	8	8	8	39.6	39.6	39.6	25.82	0.0000	47.463	24406000	145	16.22	16.23	15.56	16.38	16.06	17.13	16.69	16.94
0.645	-0.61	0.66	P56671	Myc-associated zinc finger protein	Maz	1291	2	2	2	4.6	4.6	4.6	48.769	0.0000	6.2623	1507100	34	12.30	13.53	13.18	13.65	13.35	13.71	13.31	14.73
2.435	-0.61	0.65	Q3UN04	Ubiquitin carboxyl-terminal hydrolase 30	Usp30	2008	3	3	3	10.1	10.1	10.1	58.221	0.0010	3.9643	1072500	12	13.09	12.76	13.05	13.08	13.50	13.83	13.73	13.37
0.344	-0.61	0.65	Q8BIG4	F-box only protein 28	Fbxo28	3058	2	2	2	7.6	7.6	7.6	40.977	0.0000	20.384	1052300	19	12.85	13.58	12.24	13.67	12.99	14.13	12.28	15.40
2.341	-0.61	0.65	Q8R1D1	Zinc finger protein 426	Znf426	3784	2	2	1	5.3	5.3	1.3	63.02	0.0056	2.1964	1865200	23	13.31	13.57	13.35	13.32	13.82	13.77	14.31	14.10
0.290	-0.61	0.65	Q9D7B1	tRNA-dihydrouridine(20) synthase [NAD(P)+]-like	Dus2	5026	5	5	5	13.2	13.2	13.2	55.324	0.0004	5.0167	37404000	89	16.51	17.89	16.35	18.65	17.85	17.78	16.44	19.77
2.882	-0.61	0.65	P50171	Estradiol 17-beta-dehydrogenase 8	Hsd17b8	1176	9	9	9	53.3	53.3	53.3	26.588	0.0000	46.502	25226000	238	15.82	15.62	15.91	15.68	16.18	16.59	16.32	16.41
0.425	-0.61	0.65	Q9CQI7	U2 small nuclear ribonucleoprotein B	Snrpb2	4603	7	6	6	35.1	32	32	25.323	0.0000	40.296	7341500	84	13.22	15.37	15.66	15.47	15.63	14.73	15.75	16.07
0.872	-0.61	0.65	Q9DCF9	Translocon-associated protein subunit gamma	Ssr3	5211	6	6	6	20	20	20	21.064	0.0000	215.16	37449000	108	17.25	17.47	18.40	16.92	17.85	18.47	18.32	17.86
1.695	-0.61	0.65	P70441	Na(+)/H(+) exchange regulatory cofactor NHE-RF1	Slc9a3r1	1574	18	18	18	61.7	61.7	61.7	38.6	0.0000	113.01	22676000	277	14.97	15.59	14.96	15.44	15.86	15.74	15.66	16.16
0.656	-0.62	0.65	Q91WZ8	Dysbindin	Dtnbp1	4131	4	4	4	11.9	11.9	11.9	39.651	0.0000	9.052	3384900	55	14.12	13.93	14.70	12.88	14.28	14.67	14.02	15.13
0.461	-0.62	0.65	O35613	Death domain-associated protein 6	Daxx	387	4	4	4	7.6	7.6	7.6	81.488	0.0000	9.2296	1325000	13	13.74	12.00	12.56	12.12	13.50	13.98	13.48	11.91
0.326	-0.62	0.65	Q9JII5	DAZ-associated protein 1	Dazap1	5401	9	9	9	35	35	35	43.214	0.0000	175.79	21286000	130	16.60	16.48	13.21	16.04	16.10	16.10	16.24	16.34
0.352	-0.62	0.65	P23950	Zinc finger protein 36, C3H1 type-like 1	Zfp36l1	893	2	2	2	6.2	6.2	6.2	36.384	0.0000	18.732	728380	42	12.93	12.93	13.73	10.65	13.48	12.46	12.74	14.03
2.063	-0.62	0.65	Q8BFR5	Elongation factor Tu, mitochondrial	Tufm	2945	35	35	35	77.2	77.2	77.2	49.508	0.0000	323.31	195990000	1130	17.27	17.65	17.53	17.97	18.11	18.19	18.17	18.41
0.869	-0.62	0.65	Q80VD1	Protein FAM98B	Fam98b	2849	10	10	9	36.1	36.1	33.1	45.349	0.0000	107.17	20163000	191	15.99	15.65	14.77	14.91	16.08	16.44	15.46	15.82
0.395	-0.62	0.65	Q8BUE4	Apoptosis-inducing factor 2	Aifm2	3223	5	5	5	14.7	14.7	14.7	40.634	0.0015	3.4053	6621600	26	14.38	15.56	12.86	15.89	15.48	15.30	15.06	15.34
0.723	-0.62	0.65	Q78HU3	Multivesicular body subunit 12A	Mvb12a	2721	4	4	4	19.2	19.2	19.2	28.705	0.0000	40.514	12318000	111	15.68	15.89	15.51	15.70	15.84	15.73	16.18	17.52
1.603	-0.62	0.65	Q99PW4	TP53-regulating kinase	Tp53rk	4520	3	3	3	17.2	17.2	17.2	27.393	0.0000	49.229	7606700	70	15.85	15.61	14.94	15.38	16.18	16.00	15.88	16.22
1.055	-0.62	0.65	Q9JIY5	Serine protease HTRA2, mitochondrial	Htra2	5412	14	14	13	37.1	37.1	37.1	49.348	0.0000	120.87	36951000	213	15.64	16.58	16.52	15.89	16.24	16.72	17.04	17.12
1.602	-0.62	0.65	Q8K1E6	Alpha-ketoglutarate-dependent dioxygenase alkB homolog 3	Alkbh3	3626	7	7	7	32.9	32.9	32.9	33.061	0.0000	16.713	6228900	69	14.72	14.56	13.93	14.29	15.03	15.15	14.65	15.17
1.182	-0.62	0.65	Q80X85	28S ribosomal protein S7, mitochondrial	Mrps7	2875	11	11	10	50	50	47.1	28.062	0.0000	46.484	15422000	150	15.42	15.51	16.35	15.84	15.90	16.59	16.40	16.72
1.245	-0.63	0.65	Q9CQZ6	NADH dehydrogenase [ubiquinone] 1 beta subcomplex subunit 3	Ndufb3	4652	5	5	5	42.3	42.3	42.3	11.692	0.0000	10.34	13581000	50	16.12	15.89	15.78	14.93	16.15	16.33	16.31	16.43
1.579	-0.63	0.65	Q80XQ2	TBC1 domain family member 5	Tbc1d5	2889	16	16	16	32.1	32.1	32.1	91.836	0.0000	138.16	21324000	175	15.62	15.24	15.88	15.45	15.78	16.43	16.01	16.48
1.980	-0.63	0.65	P63323	40S ribosomal protein S12	Rps12	1504	12	12	12	72.7	72.7	72.7	14.525	0.0000	323.31	207020000	627	19.20	19.78	19.54	19.85	20.11	20.31	20.04	20.43
1.311	-0.63	0.65	Q8CIL4	Uncharacterized protein C1orf131 homolog		3557	5	5	5	22.4	22.4	22.4	31.344	0.0000	45.248	2630300	43	12.62	12.97	12.78	13.05	13.14	13.68	13.06	14.06
3.817	-0.63	0.65	P63017	Heat shock cognate 71 kDa protein	Hspa8	1479	69	69	50	91	91	79.7	70.87	0.0000	323.31	3808000000	4527	21.44	21.71	21.54	21.64	22.17	22.32	22.11	22.25
1.880	-0.63	0.64	Q8BTS4	Nuclear pore complex protein Nup54	Nup54	3205	9	9	9	25.9	25.9	25.9	55.731	0.0000	47.606	9413400	143	14.15	14.87	14.58	14.77	14.99	15.26	15.22	15.44
1.266	-0.63	0.64	P59708	Splicing factor 3B subunit 6	Sf3b6	1341	5	5	5	37.6	37.6	37.6	14.585	0.0000	7.4121	10204000	71	15.72	15.53	14.64	15.20	16.18	15.71	16.04	15.71
2.052	-0.64	0.64	P10639	Thioredoxin	Txn	726	10	10	8	59	59	59	11.675	0.0000	55.891	122610000	429	17.53	17.84	17.42	17.78	18.09	18.61	18.04	18.36
0.379	-0.64	0.64	Q02248	Catenin beta-1	Ctnnb1	1717	8	5	5	13.2	10	10	85.47	0.0000	8.6562	938420	10	12.42	12.35	12.46	14.48	12.03	14.02	14.24	13.96
0.602	-0.64	0.64	D3YXK1	Atherin	Samd1	209	4	4	4	13.5	13.5	13.5	54.532	0.0002	6.0703	1963700	23	14.27	13.05	13.26	14.72	14.39	14.81	15.00	13.65
2.043	-0.64	0.64	P61226	Ras-related protein Rap-2b	Rap2b	1380	11	11	6	66.7	66.7	43.7	20.504	0.0000	323.31	64622000	288	17.26	17.23	17.85	17.74	18.03	18.22	18.15	18.24
0.780	-0.64	0.64	Q8VE22	28S ribosomal protein S23, mitochondrial	Mrps23	3984	10	10	10	62.1	62.1	62.1	20.348	0.0000	65.516	10628000	100	13.68	15.12	15.00	15.35	15.79	15.13	15.25	15.53
1.561	-0.64	0.64	Q3TDX8	Cytochrome b5 reductase 4	Cyb5r4	1835	3	3	2	7.8	7.8	4.5	59.716	0.0002	5.9227	7027900	28	14.80	15.44	15.72	14.97	15.75	16.02	15.89	15.83
1.499	-0.64	0.64	P15532	Nucleoside diphosphate kinase A	Nme1	797	13	7	7	82.2	48.7	48.7	17.208	0.0000	33.751	136960000	276	18.99	19.05	18.66	19.26	19.35	19.38	19.61	20.18
0.973	-0.64	0.64	Q8BMI3	ADP-ribosylation factor-binding protein GGA3	Gga3	3139	12	11	11	22.1	21.2	21.2	77.972	0.0000	85.72	14473000	144	14.20	15.07	15.80	15.23	15.56	15.73	15.87	15.70
0.393	-0.64	0.64	Q91WG2	Rab GTPase-binding effector protein 2	Rabep2	4109	3	3	3	7.6	7.6	7.6	62.132	0.0002	5.4051	6369300	15	13.25	15.89	16.19	15.29	15.85	16.14	16.16	15.03
1.612	-0.64	0.64	A2AR02	Peptidyl-prolyl cis-trans isomerase G	Ppig	36	11	11	11	14.2	14.2	14.2	88.324	0.0000	32.054	14864000	169	14.95	15.55	14.88	15.21	15.60	16.06	15.46	16.03
0.501	-0.64	0.64	Q924S8	Sprouty-related, EVH1 domain-containing protein 1	Spred1	4299	2	2	2	6.8	6.8	6.8	50.664	0.0000	7.4748	426390	5	12.36	12.11	12.88	12.11	13.31	14.45	12.23	12.03
2.675	-0.65	0.64	P17182	Alpha-enolase	Eno1	820	66	66	58	97.9	97.9	84.1	47.14	0.0000	323.31	8708300000	9696	21.94	22.10	22.05	22.17	22.44	22.85	22.59	22.95
1.119	-0.65	0.64	P67871	Casein kinase II subunit beta	Csnk2b	1509	10	10	10	69.3	69.3	69.3	24.942	0.0000	82.345	40320000	209	16.19	16.89	17.45	17.33	17.51	17.89	17.45	17.60
3.403	-0.65	0.64	P83870	PHD finger-like domain-containing protein 5A	Phf5a	1602	6	6	6	49.1	49.1	49.1	12.405	0.0000	23.853	15601000	166	14.84	15.04	14.70	14.90	15.34	15.62	15.54	15.56
1.132	-0.65	0.64	Q8R2L5	28S ribosomal protein S18c, mitochondrial	Mrps18c	3809	3	3	3	35	35	35	16.275	0.0000	16.485	3900300	39	13.43	13.72	14.18	14.57	14.38	14.36	15.06	14.70
2.132	-0.65	0.64	Q62523	Zyxin	Zyx	2401	9	9	9	27	27	27	60.545	0.0000	35.76	23305000	164	15.90	16.56	16.15	16.02	16.64	16.78	16.78	17.02
1.801	-0.65	0.64	Q91YR7	Pre-mRNA-processing factor 6	Prpf6	4175	35	35	35	41.7	41.7	41.7	106.72	0.0000	216.48	59510000	532	16.07	16.49	16.50	16.49	16.60	17.15	17.01	17.38
1.548	-0.65	0.64	Q8VBT9	Tether containing UBX domain for GLUT4	Aspscr1	3896	20	20	20	41.6	41.6	41.6	59.795	0.0000	151.92	37279000	251	16.19	16.61	16.75	16.76	16.77	17.24	17.23	17.67
1.343	-0.65	0.64	P35980	60S ribosomal protein L18	Rpl18	1038	15	15	15	52.7	52.7	52.7	21.644	0.0000	111.05	182830000	420	19.65	19.34	19.38	19.13	19.77	20.70	19.64	19.98
0.917	-0.65	0.64	O70311	Glycylpeptide N-tetradecanoyltransferase 2	Nmt2	487	10	6	6	18.1	14.6	14.6	60.484	0.0000	47.951	4182100	59	14.18	13.85	14.59	13.79	15.23	15.31	14.44	14.04
2.014	-0.65	0.64	Q921H8;Q8	3-ketoacyl-CoA thiolase A, peroxisomal	Acaa1a	4225	28	28	28	75.7	75.7	75.7	43.953	0.0000	323.31	628120000	1537	18.92	19.36	19.58	19.56	19.77	20.15	19.99	20.12
0.359	-0.65	0.64	O35657	Sialidase-1	Neu1	394	5	5	5	21.8	21.8	21.8	44.591	0.0000	32.185	4509200	62	13.64	14.95	12.61	13.61	14.86	12.50	14.97	15.10
0.962	-0.65	0.64	P63073	Eukaryotic translation initiation factor 4E	Eif4e	1486	7	7	7	27.6	27.6	27.6	25.053	0.0000	24.312	20154000	117	15.94	16.20	15.22	15.33	16.63	15.60	16.72	16.35
0.673	-0.65	0.64	Q923D5	WW domain-binding protein 11	Wbp11	4284	22	22	22	29.5	29.5	29.5	69.874	0.0000	157.11	46020000	392	15.34	16.21	14.70	15.95	15.75	16.32	15.66	17.08
1.838	-0.65	0.64	Q3TBT3	Stimulator of interferon genes protein	Tmem173	1820	11	11	11	31.7	31.7	31.7	42.829	0.0000	162.86	28186000	175	16.31	16.33	16.54	16.25	16.96	17.48	16.59	17.03
0.310	-0.66	0.63	Q9D3D9	ATP synthase subunit delta, mitochondrial	Atp5d	4964	2	2	2	13.7	13.7	13.7	17.6	0.0004	4.7723	4285100	42	13.84	14.88	12.67	13.97	15.20	12.21	15.05	15.53
0.234	-0.66	0.63	Q9WTQ8	Mitochondrial import inner membrane translocase subunit Tim23	Timm23	5683	4	4	4	37.8	37.8	37.8	21.978	0.0000	105.28	10148000	64	16.94	16.16	16.86	12.22	16.27	15.98	15.86	16.70
1.712	-0.66	0.63	Q8R3N1	Nucleolar protein 14	Nop14	3851	14	14	14	20.3	20.3	20.3	98.768	0.0000	50.521	15303000	178	15.44	15.34	15.10	15.59	15.75	16.42	15.69	16.26
1.529	-0.66	0.63	Q689Z5	Protein strawberry notch homolog 1	Sbno1	2470	8	8	6	9.9	9.9	8.1	153.74	0.0000	22.285	4222200	54	14.40	13.46	14.03	13.60	14.60	14.25	14.66	14.61
0.959	-0.66	0.63	Q5SV85	Synergin gamma	Synrg	2150	4	4	4	4.1	4.1	4.1	139.61	0.0000	6.8095	1579300	15	12.90	12.93	13.73	13.39	14.05	14.21	13.04	14.28
1.773	-0.66	0.63	Q8R081	Heterogeneous nuclear ribonucleoprotein L	Hnrnpl	3750	31	31	31	61.6	61.6	61.6	63.963	0.0000	323.31	324740000	1095	18.25	19.05	18.53	18.33	19.04	19.28	19.08	19.42
0.352	-0.66	0.63	Q8K2T4	Ubiquinol-cytochrome-c reductase complex assembly factor 3	Uqcc3	3672	3	3	3	36	36	36	9.5839	0.0009	3.7502	4715700	14	14.28	14.34	13.75	13.55	12.38	14.89	15.92	15.38
1.433	-0.66	0.63	Q3UM18	Large subunit GTPase 1 homolog	Lsg1	1995	17	17	17	30.6	30.6	30.6	73.156	0.0000	91.325	14075000	178	15.18	15.46	14.90	15.22	15.37	15.76	15.83	16.44
1.159	-0.66	0.63	Q99NH0	Ankyrin repeat domain-containing protein 17	Ankrd17	4498	10	10	10	6.4	6.4	6.4	274.21	0.0000	42.194	8526900	132	14.71	15.10	14.21	15.44	15.23	15.71	15.34	15.83
1.692	-0.66	0.63	Q9ERR7	15 kDa selenoprotein	Sep.15	5316	6	6	6	30.2	30.2	30.2	17.806	0.0000	71.215	13516000	106	15.94	16.37	15.72	16.15	16.53	16.84	16.37	17.09
1.758	-0.67	0.63	Q9D8T2	Gasdermin-D	Gsdmdc1	5072	22	22	22	53.8	53.8	53.8	53.237	0.0000	321.69	113080000	548	17.93	17.81	17.68	17.45	18.53	18.82	18.09	18.09
2.219	-0.67	0.63	O70492	Sorting nexin-3	Snx3	506	21	21	19	87.7	87.7	87.7	18.757	0.0000	250.88	260090000	549	19.20	19.82	19.60	19.80	20.13	20.44	20.18	20.34

1.123	-0.67	0.63	Q3U6B2	G-protein coupled receptor 183	Gpr183	1928	2	2	2	5.6	5.6	5.6	40.184	0.0000	9.0131	1120800	18	13.18	12.51	13.38	12.91	13.19	14.36	13.59	13.50
0.632	-0.67	0.63	Q8K136	Sodium channel modifier 1	Scnm1	3615	5	5	5	28.8	28.8	28.8	25.823	0.0000	12.788	3446600	34	13.94	13.11	13.99	12.81	12.93	14.37	14.45	14.77
2.262	-0.67	0.63	Q9R061	Cytosolic Fe-S cluster assembly factor NUBP2	Nubp2	5616	6	6	5	32.7	32.7	26.2	29.518	0.0000	34.892	63192000	156	17.71	17.92	18.15	17.69	18.24	18.80	18.61	18.50
0.798	-0.67	0.63	Q9WUE3	Cytochrome b561 domain-containing protein 2	Cyb561d2	5710	2	2	2	9.5	9.5	9.5	24.187	0.0018	3.1786	1995200	19	13.45	13.28	12.93	14.24	13.98	15.07	13.73	13.81
0.764	-0.67	0.63	Q924M7	Mannose-6-phosphate isomerase	Mpi	4297	8	8	8	33.1	33.1	33.1	46.575	0.0000	51.349	11058000	80	15.27	15.38	16.38	15.48	16.16	16.80	15.37	16.87
0.730	-0.67	0.63	O08691	Arginase-2, mitochondrial	Arg2	286	3	3	3	12.4	12.4	12.4	38.878	0.0000	7.1375	4324300	15	13.30	14.92	13.74	15.09	14.73	15.01	15.20	14.81
0.685	-0.67	0.63	B1AZP2	Disks large-associated protein 4	Dlgap4	76	7	7	7	9	9	9	108.04	0.0000	63.642	10870000	96	14.64	16.44	15.66	15.75	16.21	16.56	15.51	16.91
0.772	-0.67	0.63	P56959	RNA-binding protein FUS	Fus	1293	9	9	9	26.1	26.1	26.1	52.673	0.0000	65.936	17256000	101	15.93	14.97	16.10	16.37	16.43	17.40	16.17	16.06
1.251	-0.68	0.63	P17751	Triosephosphate isomerase	Tpi1	830	34	34	34	93.2	93.2	93.2	26.712	0.0000	323.31	1546800000	2082	20.46	21.26	20.24	20.69	21.17	21.29	21.02	21.87
2.057	-0.68	0.63	Q91YE7	RNA-binding protein 5	Rbm5	4156	11	11	10	20.6	20.6	19.3	92.31	0.0000	67.676	6482500	102	13.83	14.38	13.88	14.17	14.60	14.79	14.51	15.06
2.771	-0.68	0.63	Q640N3	Rho GTPase-activating protein 30	Arhgap30	2415	20	20	19	27.5	27.5	26.9	120.11	0.0000	85.156	25242000	230	15.59	15.41	15.74	15.43	16.04	16.49	16.09	16.26
0.911	-0.68	0.63	P52293	Importin subunit alpha-1	Kpna2	1214	12	12	12	29.5	29.5	29.5	57.927	0.0000	129.25	19843000	218	14.93	15.39	15.69	15.27	15.23	16.71	15.63	16.43
2.441	-0.68	0.62	Q7TMK9	Heterogeneous nuclear ribonucleoprotein Q	Syncrip	2746	34	34	34	52.8	52.8	52.8	69.632	0.0000	323.31	151680000	827	17.60	17.84	17.42	17.70	18.04	18.54	18.22	18.48
0.589	-0.68	0.62	Q62189	U1 small nuclear ribonucleoprotein A	Snrpa	2365	10	10	9	35.9	35.9	35.9	31.835	0.0000	31.416	27630000	184	16.57	16.04	18.00	15.69	16.81	17.29	17.18	17.74
0.839	-0.68	0.62	Q91WN1	DnaJ homolog subfamily C member 9	Dnajc9	4122	16	16	16	62.9	62.9	62.9	30.059	0.0000	109.91	21993000	261	14.59	15.53	14.34	15.39	15.16	16.03	15.17	16.22
1.018	-0.68	0.62	Q8R310	Transmembrane and coiled-coil domains protein 3	Tmcc3	3828	5	5	5	14	14	14	53.721	0.0000	9.7722	2756200	30	12.86	13.61	13.71	13.67	13.75	14.54	13.57	14.70
0.516	-0.68	0.62	P07339	Cathepsin D;Cathepsin D light chain;Cathepsin D heavy chain	CTSD	669	9	7	7	25.7	18.7	18.7	44.552	0.0000	13.487	5924600	56	14.38	14.14	12.39	13.89	13.60	14.52	15.47	13.95
1.266	-0.68	0.62	P51949	CDK-activating kinase assembly factor MAT1	Mnat1	1211	6	6	6	26.9	26.9	26.9	35.848	0.0000	17.264	2742600	42	12.96	13.96	13.48	13.25	13.78	13.75	14.43	14.42
1.718	-0.68	0.62	P51150	Ras-related protein Rab-7a	Rab7a	1191	25	25	25	87.9	87.9	87.9	23.489	0.0000	323.31	445930000	942	18.76	19.58	19.64	19.59	20.00	20.12	20.01	20.18
0.687	-0.69	0.62	Q6PDN3	Myosin light chain kinase, smooth muscle;Myosin light chain kinase, sm	Mylk	2619	6	6	6	3.7	3.7	3.7	212.92	0.0027	2.6964	43992000	48	16.86	17.71	18.65	18.63	18.51	19.31	18.42	18.35
1.268	-0.69	0.62	Q8BVY0	Ribosomal L1 domain-containing protein 1	Rsl1d1	3251	16	16	16	37.6	37.6	37.6	50.421	0.0000	226.52	35053000	173	16.51	16.19	16.44	16.43	16.55	17.71	16.67	17.39
1.509	-0.69	0.62	Q99N96	39S ribosomal protein L1, mitochondrial	Mrpl1	4494	16	16	16	53	53	53	37.596	0.0000	275.6	30870000	392	15.19	15.34	15.80	15.92	15.77	16.27	16.41	16.55
1.283	-0.69	0.62	Q60675	Laminin subunit alpha-2	Lama2	2192	5	5	5	2.5	2.5	2.5	343.81	0.0059	2.097	11051000	21	15.52	16.12	16.57	16.74	16.72	17.06	16.85	17.06
1.158	-0.69	0.62	Q3U7U3	F-box only protein 7	Fbxo7	1933	3	3	3	10.9	10.9	10.9	57.648	0.0000	23.273	2393400	46	12.75	13.70	13.70	13.95	13.84	14.11	14.62	14.30
1.970	-0.69	0.62	Q9QX47	Protein SON	Son	5532	14	14	14	8	8	8	265.65	0.0000	43.305	16316000	134	15.17	14.88	15.18	15.24	15.52	16.30	15.70	15.71
2.017	-0.69	0.62	P62192	26S protease regulatory subunit 4	Psmc1	1406	35	35	35	71.6	71.6	71.6	49.184	0.0000	323.31	234470000	1095	17.31	17.96	17.66	17.89	18.14	18.52	18.29	18.64
3.153	-0.69	0.62	Q9D2V7	Coronin-7	Coro7	4953	42	42	42	71.4	71.4	71.4	100.81	0.0000	323.31	240490000	1014	18.06	18.12	18.03	17.76	18.77	18.84	18.54	18.58
0.917	-0.69	0.62	P63166;P63	Small ubiquitin-related modifier 1	Sumo1;SUMO1	1494	6	6	5	53.5	53.5	37.6	11.557	0.0002	5.676	11621000	56	14.51	14.97	16.26	15.55	16.04	16.15	15.99	15.87
1.679	-0.69	0.62	B2RX14	Terminal uridylyltransferase 4	Zcchc11	83	6	6	6	4.7	4.7	4.7	184.65	0.0000	25.522	3840200	61	14.04	13.81	13.93	13.45	13.99	14.85	14.50	14.66
1.627	-0.69	0.62	P31230	Aminoacyl tRNA synthase complex-interacting multifunctional protein 1;	Aimp1	984	17	17	17	70.3	70.3	70.3	33.997	0.0000	282.25	59019000	483	15.98	16.47	15.59	16.36	16.73	16.69	16.62	17.13
2.124	-0.70	0.62	P62274	40S ribosomal protein S29	Rps29	1414	6	6	6	67.9	67.9	67.9	6.6767	0.0000	47.096	221640000	335	19.22	19.53	19.29	19.42	19.92	20.47	19.71	20.14
0.755	-0.70	0.62	Q61733	28S ribosomal protein S31, mitochondrial	Mrps31	2328	15	15	15	37.5	37.5	37.5	43.88	0.0000	177.21	26270000	313	14.65	15.92	14.87	15.96	15.48	15.99	15.84	16.87
0.984	-0.70	0.62	P70271	PDZ and LIM domain protein 4	Pdlim4	1543	11	11	11	45.2	45.2	45.2	35.556	0.0000	37.599	14361000	103	14.52	15.25	13.93	14.22	14.90	14.87	15.09	15.84
1.109	-0.70	0.62	P32020	Non-specific lipid-transfer protein	Scp2	993	23	23	22	32.5	32.5	32	59.125	0.0000	139.11	127940000	622	17.21	17.93	17.42	18.17	17.95	18.05	18.52	19.01
1.378	-0.70	0.62	Q8BVK9	Sp110 nuclear body protein	Sp110	3243	15	15	15	35.1	35.1	35.1	50.14	0.0000	141.87	25379000	218	16.80	16.62	16.43	16.44	17.12	17.98	16.76	17.24
2.767	-0.70	0.62	Q9QYJ0	DnaJ homolog subfamily A member 2	Dnaja2	5575	17	17	17	50.2	50.2	50.2	45.745	0.0000	96.681	42979000	357	15.58	16.12	15.76	15.68	16.38	16.41	16.54	16.62
1.278	-0.70	0.61	P97797	Tyrosine-protein phosphatase non-receptor type substrate 1	Sirpa	1671	11	11	11	34.9	34.9	34.9	56.413	0.0000	127.03	50425000	224	17.66	16.73	16.39	16.71	17.86	17.54	17.39	17.51
1.290	-0.70	0.61	Q80ZK9	WD and tetratricopeptide repeats protein 1	Wdtc1	2916	2	2	2	5.2	5.2	5.2	75.732	0.0000	6.3524	1251700	21	13.15	13.16	14.17	12.98	14.19	14.09	14.22	13.77
1.845	-0.71	0.61	Q8BHS3	Pre-mRNA-splicing factor RBM22	Rbm22	3050	8	8	8	28.3	28.3	28.3	46.895	0.0000	24.332	8436200	82	14.88	15.19	14.46	14.44	15.30	15.52	15.27	15.70
1.389	-0.71	0.61	Q01768	Nucleoside diphosphate kinase B	Nme2	1711	16	16	10	86.8	86.8	53.3	17.363	0.0000	232.08	733750000	1016	20.52	20.72	20.73	21.07	21.06	21.07	21.65	22.08
1.748	-0.71	0.61	Q9Z2I8	Succinyl-CoA ligase [GDP-forming] subunit beta, mitochondrial	Suclg2	5856	20	20	20	51	51	51	46.839	0.0000	256.78	80761000	550	16.91	17.21	17.42	17.62	17.53	18.16	18.07	18.22
2.395	-0.71	0.61	A2AAY5;O8	SH3 and PX domain-containing protein 2B	Sh3pxd2b	14	23	23	23	35.4	35.4	35.4	101.52	0.0000	121.06	65141000	562	16.01	15.91	15.83	16.13	16.34	16.91	16.54	16.92
1.331	-0.71	0.61	Q811D0;Q9	Disks large homolog 1	Dlg1	2936	14	14	14	20.1	20.1	20.1	100.12	0.0000	74.109	32042000	152	15.87	16.64	15.67	16.50	16.75	17.11	16.49	17.17
2.108	-0.71	0.61	Q04447	Creatine kinase B-type	Ckb	1731	29	29	28	79.5	79.5	79.5	42.713	0.0000	323.31	605340000	1473	19.59	20.16	19.32	19.67	20.45	20.33	20.51	20.31
0.350	-0.71	0.61	Q3V4B5	COMM domain-containing protein 6	Commd6	2055	6	6	6	70.1	70.1	70.1	9.7951	0.0000	11.749	8567300	41	15.11	12.50	15.09	15.00	13.36	15.72	15.60	15.87
1.042	-0.71	0.61	Q9JMF7	Dolichyldiphosphatase 1	Dolpp1	5508	2	2	2	12.6	12.6	12.6	27.098	0.0002	5.5276	796640	20	11.93	12.91	12.10	12.45	12.48	13.26	13.75	12.74
0.657	-0.72	0.61	Q61301	Catenin alpha-2	Ctnna2	2289	4	2	2	7	3.4	3.4	105.28	0.0000	9.626	2942300	33	12.77	13.91	14.19	14.46	14.12	15.41	14.89	13.78
2.368	-0.72	0.61	P18155	Bifunctional methylenetetrahydrofolate dehydrogenase/cyclohydrolase,	Mthfd2	834	17	17	17	53.1	53.1	53.1	37.863	0.0000	129.78	40830000	259	16.39	16.75	16.32	16.44	16.87	17.50	17.25	17.15
0.475	-0.72	0.61	Q9DB10	Essential MCU regulator, mitochondrial	Smdt1	5117	3	3	3	35.5	35.5	35.5	11.542	0.0004	5.0136	3114200	17	12.63	13.70	12.97	14.75	14.63	15.25	14.15	12.89
0.658	-0.72	0.61	G3X9C2	F-box only protein 50	Nccrp1	259	2	2	2	6.8	6.8	6.8	30.407	0.0025	2.7587	3858100	45	13.77	14.60	13.90	13.49	14.75	15.24	13.31	15.33
1.498	-0.72	0.61	Q61160	FAS-associated death domain protein	Fadd	2262	9	9	9	50.2	50.2	50.2	22.96	0.0000	171.26	17985000	230	14.84	15.55	15.38	15.85	15.71	16.15	16.21	16.42
2.672	-0.72	0.61	Q8K1A6	Coiled-coil and C2 domain-containing protein 1A	Cc2d1a	3621	12	12	12	17	17	17	103.7	0.0000	26.242	11687000	108	14.95	14.72	14.72	14.84	15.52	15.44	15.27	15.88
0.834	-0.72	0.61	Q80XI6	Mitogen-activated protein kinase kinase kinase 11	Map3k11	2883	4	4	4	8.4	8.4	8.4	93.225	0.0000	8.3241	1459000	22	12.92	12.66	13.83	12.14	13.55	14.15	12.96	13.78
2.101	-0.73	0.60	Q8K363	ATP-dependent RNA helicase DDX18	Ddx18	3692	13	13	13	27.1	27.1	27.1	74.18	0.0000	34.467	12521000	115	14.34	14.65	14.66	14.46	14.83	15.36	15.18	15.64
0.796	-0.73	0.60	Q8VDP6	CDP-diacylglycerol--inositol 3-phosphatidyltransferase	Cdipt	3966	3	3	3	10.8	10.8	10.8	23.599	0.0000	6.7674	9519800	69	14.22	16.22	15.28	14.50	15.97	15.64	15.86	15.67
1.611	-0.73	0.60	Q99L00	HAUS augmin-like complex subunit 8	Haus8	4401	5	5	5	17.7	17.7	17.7	41.653	0.0000	18.949	2707300	18	13.82	13.87	13.61	13.82	14.06	15.08	14.18	14.73
2.022	-0.73	0.60	Q6A068	Cell division cycle 5-like protein	Cdc5l	2498	36	36	36	53.6	53.6	53.6	92.188	0.0000	255.25	58895000	519	16.15	16.33	16.10	16.62	16.71	17.29	16.82	17.30
1.519	-0.73	0.60	P62852	40S ribosomal protein S25	Rps25	1454	16	16	16	68.8	68.8	68.8	13.742	0.0000	160.92	215100000	449	18.88	18.75	19.02	19.50	19.20	19.82	19.93	20.13
1.358	-0.73	0.60	Q9D620;G3	Rab11 family-interacting protein 1	Rab11fip1	4988	9	9	9	16.4	16.4	16.4	70.683	0.0000	120.2	7790200	106	14.25	14.59	14.61	14.97	15.35	15.76	14.64	15.60
0.250	-0.73	0.60	Q8BHD0	Ras-related protein Rab-39A	Rab39a	3032	3	1	1	6.9	6	6	24.977	0.0017	3.2824	15053000	44	14.35	15.83	15.54	13.13	17.38	13.14	16.95	14.32
1.157	-0.73	0.60	Q9CX56	26S proteasome non-ATPase regulatory subunit 8	Psmd8	4748	8	8	8	24.9	24.9	24.9	39.93	0.0000	15.389	8077000	65	16.05	15.03	14.99	15.00	16.34	16.31	15.46	15.90
2.033	-0.73	0.60	P11438	Lysosome-associated membrane glycoprotein 1	Lamp1	743	7	7	7	12.3	12.3	12.3	43.865	0.0000	90.118	86716000	175	17.88	18.27	18.48	17.85	18.67	19.05	18.63	19.08
2.113	-0.73	0.60	Q14DQ1	Protein FAM219B	Fam219b	1792	3	3	3	19.3	19.3	19.3	21.006	0.0000	50.556	2240500	47	13.16	12.64	12.90	12.85	13.25	13.94	13.51	13.78
2.249	-0.74	0.60	P99029	Peroxiredoxin-5, mitochondrial	Prdx5	1694	19	19	19	59.5	59.5	59.5	21.897	0.0000	323.31	405710000	934	19.39	19.96	19.56	19.92	20.14	20.49	20.59	20.58
1.837	-0.74	0.60	P36536	GTP-binding protein SAR1a	Sar1a	1042	13	13	9	51.5	51.5	44.4	22.371	0.0000	124.75	89746000	256	18.25	18.57	17.86	18.57	18.85	19.18	18.80	19.39
1.444	-0.74	0.60	O08784	Treacle protein	Tcof1	298	30	30	30	29	29	29	135	0.0000	160.88	46711000	364	16.02	16.26	16.15	16.56	16.53	17.66	16.70	17.07
1.719	-0.74	0.60	Q9JKP5	Muscleblind-like protein 1	Mbnl1	5456	20	20	7	52.5	52.5	25.2	36.975	0.0000	277.21	250970000	876	18.90	19.16	18.52	19.18	19.49	19.77	19.33	20.14
1.106	-0.75	0.60	A2ASS6	Titin	Ttn	37	25	25	25	0.9	0.9	0.9	3906.4	0.0015	3.3944	109690000	136	17.85	17.77	17.34	18.63	18.20	19.11	18.94	18.31
1.151	-0.75	0.60	P61759	Prefoldin subunit 3	Vbp1	1389	8	8	8	48.5	48.5	48.5	22.435	0.0000	323.31	12554000	138	14.34	15.45	14.22	15.38	15.67	15.42	15.50	15.79
1.112	-0.75	0.60	P08228	Superoxide dismutase [Cu-Zn]	Sod1	683	11	11	11	54.5	54.5	54.5	15.942	0.0000	113.21	28287000	304	16.43	16.89	15.58	16.49	17.32	17.35	16.45	17.25
1.301	-0.75	0.60	O88286	Protein Wiz	Wiz	520	4	4	4	3.6	3.6	3.6	184.29	0.0000	29.358	5984000	55	15.08	15.02	15.20	14.77	15.31	16.21	15.22	16.32
2.850	-0.75	0.60	Q922D8	C-1-tetrahydrofolate synthase, cytoplasmic;Methylenetetrahydrofolate d	Mthfd1	4251	66	66	65	76.3	76.3	75.2	101.2	0.0000	323.31	286760000	1555	17.34	17.49	17.59	17.57	17.92	18.38	18.21	18.47
2.906	-0.75	0.60	Q9CY58	Plasminogen activator inhibitor 1 RNA-binding protein	Serbp1	4787	17	17	17	43.2	43.2	43.2	44.714	0.0000	267.23	67104000	297	16.93	17.01	16.88	17.31	17.88	17.69	17.58	17.97
0.918	-0.75	0.60	O88952;Q8	Protein lin-7 homolog C	Lin7c	587	7	7	7	39.6	39.6	39.6	21.834	0.0000	14.873	10012000	125	14.99	15.53	14.12	15.60	15.83	15.98	15.16	16.25
1.560	-0.75	0.60	Q8VE99	Coiled-coil domain-containing protein 115	Ccdc115	3996	14	14	14	81.1	81.1	81.1	19.742	0.0000	119.72	52912000	341	16.01	16.73	16.49	16.84	16.89	17.28	17.14	17.76
2.177	-0.75	0.59	Q9Z0H1	WD repeat-containing protein 46	Wdr46	5769	8	8	8	17.8	17.8	17.8	69.047	0.0000	58.189	5702600	106	14.33	14.03	14.08	14.33	14.78	15.41	14.64	14.94
1.309	-0.75	0.59	Q8CEC0	Nuclear pore complex protein Nup88	Nup88	3470	9	9	9	18.1	18.1	18.1	84.937	0.0000	75.411	11082000	125	15.66	15.03	14.20	14.97	15.83	15.60	15.82	15.62
0.960	-0.75	0.59	O35143	ATPase inhibitor, mitochondrial	Atpif1	342	8	8	8	43.4	43.4	43.4	12.159	0.0000	8.2938	6779000	32	13.25	13.34	13.62	14.34	15.33	14.09	14.16	13.98
0.495	-0.75	0.59	Q9CX84	Regulator of G-protein signaling 19	Rgs19	4749	5	5	5	34.7	34.7	34.7	24.677	0.0000	18.349	4195900	16	14.21	12.28	14.96	15.36	15.02	14.65	15.19	14.95
0.505	-0.75	0.59	Q9CQ48	NudC domain-containing protein 2	Nudcd2	4560	5	5	5	40.8	40.8	40.8	17.66	0.0000	19.958	3700400	26	12.88	14.00	12.57	14.27	12.74	15.34	14.46	14.19
2.564	-0.75	0.59	Q8BH95	Enoyl-CoA hydratase, mitochondrial	Echs1	3024	18	18	18	62.4	62.4	62.4	31.474	0.0000	323.31	209100000	806	18.01	18.13	17.76	18.07	18.45	18.80	18.65	19.07
1.608	-0.75	0.59	P14148	60S ribosomal protein L7	Rpl7	775	31	31	31	61.5	61.5	61.5	31.419	0.0000	141.86	424640000	881	19.42	19.24	19.54	19.01	19.75	20.52	19.60	20.35
0.605	-0.75	0.59	Q9Z2E2	Methyl-CpG-binding domain protein 1	Mbd1	5847	2	2	2	4.2	4.2	4.2	70.022	0.0000	7.1024	719460	6	11.64	12.53	13.53	13.20	13.75	14.38	13.41	12.38
1.105	-0.76	0.59	P21126	Ubiquitin-like protein 4A	Ubl4a	860	10	10	10	70.1	70.1	70.1	17.8	0.0000	30.414	34373000	281	15.17	16.41	15.50	16.21	16.30	16.68	16.22	17.14
1.094	-0.76	0.59	P06797	Cathepsin L1;Cathepsin L1 heavy chain;Cathepsin L1 light chain	Ctsl	663	8	8	8	24	24	24	37.547	0.0000	76.164	14757000	128	15.73	16.24	15.53	14.56	16.05	16.28	16.29	16.48
0.654	-0.76	0.59	Q8C0L6	Peroxisomal N(1)-acetyl-spermine/spermidine oxidase	Paox	3342	9	9	9	22.4	22.4	22.4	55.446	0.0000	56.023	10347000	110	14.50	15.07	14.83	14.90	14.21	15.21	16.45	16.49
1.093	-0.76	0.59	Q80U72;Q8	Protein scribble homolog	Scrib	2823	21	21	21	20.9	20.9	20.9	174.06	0.0000	89.354	18111000	222	14.38	14.48	15.22	15.31	14.84	15.86	15.67	16.09
1.072	-0.77	0.59	Q9D6T0	Nitric oxide synthase-interacting protein	Nosip	5009	7	7	7	33.2	33.2	33.2	33.209	0.0000	41.716	7024900	69	14.99	15.25	14.73	13.60	15.19	15.56	15.48	15.40
0.784	-0.77	0.59	P34884	Macrophage migration inhibitory factor	Mif	1007	7	7	7	60.9	60.9	60.9	12.504	0.0000	50.328	111070000	250	18.33	19.80	17.71	18.87	19.20	19.48	19.11	19.99
2.024	-0.77	0.59	P62849	40S ribosomal protein S24	Rps24	1453	13	13	13	52.6	52.6	52.6	15.423	0.0000	203.15	248060000	520	19.52	19.54	19.39	19.57	19.89	20.51	19.97	20.71
2.405	-0.77	0.59	Q9JMD0	BUB3-interacting and GLEBS motif-containing protein ZNF207	Znf207	5506	5	5	5	7.7	7.7	7.7	52.792	0.0000	11.623	14948000	98	15.11	15.35	14.96	15.38	15.76	16.14	15.72	16.26
1.436	-0.77	0.59	O35972	39S ribosomal protein L23, mitochondrial	Mrpl23	416	7	7	7	47.9	47.9	47.9	17.121	0.0000	70.336	23719000	136	15.79	16.16	16.90	16.56	16.66	17.27	17.20	17.37
1.544	-0.78	0.58	Q9CQ92	Mitochondrial fission 1 protein	Fis1	4576	11	11	11	53.3	53.3	53.3	17.008	0.0000	210.3	138050000	417	18.78	19.16	18.51	19.03	19.14	19.60	19.59	20.25
0.458	-0.78	0.58	Q9CR27	WASH complex subunit CCDC53	Ccdc53	4662	5	5	3	35.1	35.1	22.7	21.092	0.0000	6.5824	6286400	34	15.60	12.88	14.09	13.58	14.99	15.64	15.25	13.37
1.046	-0.78	0.58	P61514	60S ribosomal protein L37a	Rpl37a	1386	8	8	8	73.9	73.9	73.9	10.275	0.0000	133.91	74001000	258	17.75	17.99	16.45	17.31	18.07	18.51	17.67	18.37
1.605	-0.78	0.58	P63254	Cysteine-rich protein 1	Crip1	1498	7	7	6	84.4	84.4	84.4	8.5497	0.0000	19.011	82817000	259	18.09	18.65	18.07	18.41	18.77	19.06	18.79	19.72
0.890	-0.78	0.58	Q9JJ80	Ribosome production factor 2 homolog	Rpf2	5419	5	5	5	21.6	21.6	21.6	35.363	0.0000	15.845	6378700	81	13.84	13.92	14.49	14.07	13.84	15.88	14.75	14.98
0.480	-0.78	0.58	Q9CZ69	CKLF-like MARVEL transmembrane domain-containing protein 6	Cmtm6	4819	2	2	2	15.8	15.8	15.8	19.824	0.0004	5.1288	2450600	18	12.71	11.58	12.98	13.70	14.41	13.91	11.77	14.00
1.911	-0.78	0.58	Q8C5L3	CCR4-NOT transcription complex subunit 2	Cnot2	3397	5	5	5	17.4	17.4	17.4	59.71	0.0000	19.177	8214700	60	15.56	15.52	15.26	15.33	16.07	15.74	16.26	16.74
0.689	-0.78	0.58	Q7TQK4	Exosome complex component RRP40	Exosc3	2777	7	7	7	36.1	36.1	36.1	29.546	0.0000	77.35	8556600	77	15.54	15.09	15.19	14.37	16.09	16.77	16.04	14.44
0.641	-0.79	0.58	Q8R2M2	Deoxynucleotidyltransferase terminal-interacting protein 2	Dnttip2	3810	10	10	10	20.2	20.2	20.2	84.276	0.0000	23.759	8467700	52	12.96	14.27	14.65	14.15	13.87	15.66	14.15	15.49
1.265	-0.79	0.58	Q9EST3	Eukaryotic translation initiation factor 4E transporter	Eif4enif1	5343	4	4	4	6.2	6.2	6.2	107.98	0.0000	11.215	7222600	55	14.52	15.37	14.61	14.78	15.08	15.39	15.60	16.35
2.613	-0.79	0.58	Q505F5	Leucine-rich repeat-containing protein 47	Lrrc47	2082	27	27	27	48.5	48.5	48.5	63.589	0.0000	310.33	152370000	708	17.10	17.33	17.63	17.66	17.96	18.26	18.34	18.30
1.879	-0.79	0.58	O55098	Serine/threonine-protein kinase 10	Stk10	450	44	41	41	43.3	41	41	111.9	0.0000	198.76	60888000	550	15.82	16.24	15.99	16.57	16.55	17.12	16.84	17.26
1.227	-0.79	0.58	Q64191	N(4)-(beta-N-acetylglucosaminyl)-L-asparaginase;Glycosylasparaginase	Aga	2422	7	7	7	30.9	30.9	30.9	37.022	0.0000	109.75	21388000	172	14.94	15.93	16.01	16.49	16.42	16.56	16.67	16.89
2.216	-0.79	0.58	Q9DCC4	Pyrroline-5-carboxylate reductase 3	Pycrl	5204	11	11	11	48.9	48.9	48.9	28.721	0.0000	81.236	102370000	484	17.09	17.47	17.25	17.60	17.79	18.08	18.18	18.53
2.672	-0.79	0.58	Q3UIA2	Rho GTPase-activating protein 17	Arhgap17	1979	36	36	34	54.1	54.1	54.1	92.201	0.0000	323.31	115160000	679	17.20	17.37	17.16	17.20	17.99	18.40	17.69	18.02
0.897	-0.79	0.58	Q0P678	Zinc finger CCCH domain-containing protein 18	Zc3h18	1776	5	5	5	6.2	6.2	6.2	105.69	0.0000	15.371	2654300	31	13.62	13.22	12.54	13.85	13.25	14.87	13.90	14.38
0.746	-0.80	0.58	Q9DB15	39S ribosomal protein L12, mitochondrial	Mrpl12	5118	9	9	9	60.7	60.7	60.7	21.708	0.0000	109.92	11908000	140	14.40	16.13	13.82	15.01	15.30	15.71	15.45	16.09
1.908	-0.80	0.58	P63038	60 kDa heat shock protein, mitochondrial	Hspd1	1483	71	71	71	90.2	90.2	90.2	60.955	0.0000	323.31	1748600000	4305	20.44	20.92	20.52	21.14	21.28	21.37	21.59	21.96
1.404	-0.80	0.58	P62315	Small nuclear ribonucleoprotein Sm D1	Snrpd1	1422	4	4	4	54.6	54.6	54.6	13.281	0.0000	70.875	148440000	199	20.12	20.26	19.28	20.41	20.55	21.30	20.70	20.70
1.303	-0.80	0.58	Q9CWG8	NADH dehydrogenase [ubiquinone] complex I, assembly factor 7	Ndufaf7	4714	8	8	8	29.6	29.6	29.6	48.384	0.0000	35.752	3133400	39	13.51	12.29	13.56	13.35	13.77	13.81	14.00	14.33
1.548	-0.80	0.58	P63242;Q8E	Eukaryotic translation initiation factor 5A-1;Eukaryotic translation initiatio	Eif5a;Eif5a2	1497	13	13	13	77.3	77.3	77.3	16.832	0.0000	216.41	45973000	292	16.68	17.22	16.83	16.74	17.74	18.01	16.94	17.96
0.793	-0.80	0.57	Q3TLH4	Protein PRRC2C	Prrc2c	1859	24	24	23	12.1	12.1	11.8	310.89	0.0000	56.425	13756000	113	13.49	13.96	14.47	13.84	15.30	14.63	13.49	15.54

	1.525	-0.80	0.57	Q6PIU9	Uncharacterized protein FLJ45252 homolog		2647	12	12	12	60.5	60.5	60.5	37.704	0.0000	119.14	45210000	197	17.23	17.67	18.44	18.04	18.35	18.90	18.64	18.69
	1.599	-0.80	0.57	Q8BIQ5	Cleavage stimulation factor subunit 2	Cstf2	3064	6	6	3	23.1	23.1	12.4	61.341	0.0000	36.868	3296000	42	13.72	13.65	12.66	13.47	14.05	14.51	14.04	14.10
	2.533	-0.80	0.57	Q00PI9;REV	Heterogeneous nuclear ribonucleoprotein U-like protein 2	Hnrnpul2	1703	24	24	24	39.1	39.1	39.1	84.939	0.0000	230.24	84807000	485	17.09	17.00	16.99	16.93	17.63	18.17	17.45	17.97
	0.981	-0.80	0.57	Q9D735	Uncharacterized protein C19orf43 homolog		5019	3	3	3	13.9	13.9	13.9	18.376	0.0000	12.595	2411000	29	14.31	12.72	12.58	13.17	13.84	13.95	13.77	14.43
	0.587	-0.80	0.57	P60060	Protein transport protein Sec61 subunit gamma	Sec61g	1350	3	3	3	29.4	29.4	29.4	7.7412	0.0000	42.123	25663000	99	15.82	16.15	18.14	17.24	18.26	17.02	17.00	18.28
	1.356	-0.80	0.57	Q8VC04	Transmembrane protein 106A	Tmem106a	3904	4	4	4	17.6	17.6	17.6	29.109	0.0000	17.177	11122000	88	15.11	15.14	14.47	15.01	16.04	15.99	14.91	15.99
	1.513	-0.81	0.57	P30681	High mobility group protein B2	Hmgb2	980	29	29	21	69.5	69.5	48.6	24.162	0.0000	212.88	325910000	761	18.51	19.05	17.97	18.91	19.26	19.37	19.17	19.87
	0.999	-0.81	0.57	Q62446	Peptidyl-prolyl cis-trans isomerase FKBP3	Fkbp3	2395	23	23	23	72.3	72.3	72.3	25.147	0.0000	79.784	66560000	436	16.21	17.32	16.07	17.37	17.08	17.54	17.42	18.15
	2.991	-0.81	0.57	Q8JZV7	Putative N-acetylglucosamine-6-phosphate deacetylase	Amdhd2	3584	18	18	18	62.6	62.6	62.6	43.5	0.0000	188.49	138380000	685	17.34	17.72	17.72	17.48	18.28	18.64	18.18	18.38
	1.698	-0.81	0.57	Q9CSN1	SNW domain-containing protein 1	Snw1	4699	23	23	23	53.4	53.4	53.4	61.475	0.0000	254.13	53849000	494	15.56	15.83	15.37	15.99	16.16	16.67	16.12	17.03
	1.980	-0.81	0.57	E9Q7G0		Numa1	247	88	88	88	51.1	51.1	51.1	235.63	0.0000	323.31	139930000	1575	16.04	16.63	16.43	16.55	16.90	17.60	16.93	17.45
	1.737	-0.81	0.57	Q810A7	ATP-dependent RNA helicase DDX42	Ddx42	2925	19	19	19	26	26	26	101.96	0.0000	87.165	17246000	184	15.85	15.71	14.92	15.08	16.04	16.47	16.03	16.25
	1.921	-0.81	0.57	O35099	Mitogen-activated protein kinase kinase kinase 5	Map3k5	336	5	5	3	5.1	5.1	3.3	154.51	0.0000	10.878	1863100	16	13.30	13.94	13.31	13.51	14.08	14.83	14.16	14.22
	0.465	-0.81	0.57	Q6PAQ4	RNA exonuclease 4	Rexo4	2598	5	5	5	14.1	14.1	14.1	47.598	0.0000	9.6187	2474900	27	13.55	12.66	12.39	13.58	13.57	15.42	12.00	14.42
	1.847	-0.81	0.57	Q99M28	RNA-binding protein with serine-rich domain 1	Rnps1	4454	6	6	6	25.6	25.6	25.6	34.208	0.0000	37.781	21112000	120	16.47	16.42	16.05	16.03	16.96	17.55	16.56	17.13
	0.819	-0.81	0.57	Q9D706	RNA polymerase II-associated protein 3	Rpap3	5015	7	7	7	11.5	11.5	11.5	74.095	0.0000	31.696	4509800	47	15.06	14.04	12.72	13.89	14.42	14.98	14.87	14.68
	0.508	-0.81	0.57	Q9JKX6	ADP-sugar pyrophosphatase	Nudt5	5463	7	7	7	36.2	36.2	36.2	23.984	0.0000	19.57	6463000	83	14.84	14.24	12.06	15.34	14.74	14.83	14.89	15.27
	1.246	-0.81	0.57	Q3THK3	General transcription factor IIF subunit 1	Gtf2f1	1845	16	16	16	30.7	30.7	30.7	57.241	0.0000	187.98	43630000	223	16.15	17.09	15.94	16.60	16.85	17.53	16.88	17.77
	1.830	-0.81	0.57	Q61103	Zinc finger protein ubi-d4	Dpf2	2252	10	10	10	38.9	38.9	38.9	44.229	0.0000	62.882	11473000	140	14.38	14.84	15.04	14.60	15.26	15.50	15.27	16.08
	0.417	-0.81	0.57	Q9D7S9	Charged multivesicular body protein 5	Chmp5	5040	5	5	5	36.5	36.5	36.5	24.575	0.0000	198.72	4969900	69	13.00	15.31	12.21	13.09	15.30	15.02	13.22	13.32
+	4.498	-0.82	0.57	Q64012	RNA-binding protein Raly	Raly	2412	25	25	25	56.4	56.4	56.4	33.188	0.0000	227.4	89783000	494	17.13	17.06	17.03	17.12	17.82	18.07	17.76	17.95
	0.638	-0.82	0.57	Q61878	Bone marrow proteoglycan;Eosinophil granule major basic protein	Prg2	2340	6	6	6	30	30	30	24.255	0.0000	49.768	119080000	151	19.26	18.60	19.82	19.91	18.93	21.48	19.95	20.51
	0.955	-0.82	0.57	Q9Z2V6	Histone deacetylase 5	Hdac5	5867	6	5	5	12.4	11.8	11.8	120.94	0.0000	28.63	2926500	37	14.44	14.17	13.57	13.18	13.79	14.76	14.69	15.41
	1.330	-0.82	0.57	Q62036	Centrosomal protein of 131 kDa	Cep131	2348	5	5	5	6.9	6.9	6.9	120.31	0.0000	14.271	3076000	46	13.73	14.21	12.88	13.88	14.02	14.50	14.65	14.82
	1.442	-0.82	0.56	P09450	Transcription factor jun-B	Junb	697	7	7	7	37.8	37.8	37.8	35.765	0.0000	59.199	17926000	156	14.94	15.78	15.44	16.05	15.96	16.62	16.16	16.77
	0.451	-0.83	0.56	Q3UGC7;Q6	Eukaryotic translation initiation factor 3 subunit J-A;Eukaryotic translatio	Eif3j1;Eif3j2	1960	10	10	10	57.5	57.5	57.5	29.343	0.0000	144.42	6823600	80	12.32	15.19	14.13	13.16	15.05	15.57	14.46	13.04
	1.858	-0.83	0.56	P62897;P00	Cytochrome c, somatic	Cycs	1465	14	14	8	67.6	67.6	41	11.605	0.0000	172.43	59130000	289	16.31	16.88	16.14	16.66	17.61	17.15	16.92	17.63
	0.939	-0.83	0.56	Q8CC12	Codanin-1	Cdan1	3442	6	6	6	8.7	8.7	8.7	135.88	0.0000	68.247	10271000	105	14.36	14.67	15.83	15.70	15.43	16.62	15.69	16.13
	1.151	-0.83	0.56	Q9CYH6	Ribosome biogenesis regulatory protein homolog	Rrs1	4798	7	7	7	23.6	23.6	23.6	41.551	0.0000	19.494	3407500	30	14.06	14.10	13.18	13.63	13.73	14.78	14.58	15.21
	0.883	-0.83	0.56	Q9D9B4	Leucine-rich repeat-containing protein C10orf11 homolog		5100	9	9	9	38	38	38	25.988	0.0000	54.336	11195000	102	14.97	15.51	13.82	15.66	15.52	16.01	15.38	16.37
	2.023	-0.83	0.56	Q8VDP4	Cell cycle and apoptosis regulator protein 2	Ccar2	3965	31	31	31	41.1	41.1	41.1	103	0.0000	220.89	60400000	487	15.83	16.22	16.73	16.56	16.98	17.43	17.05	17.21
	0.621	-0.83	0.56	Q9Z2Q5	39S ribosomal protein L40, mitochondrial	Mrpl40	5862	7	7	7	40.3	40.3	40.3	24.301	0.0000	67.101	4921900	61	12.60	14.59	11.68	12.80	13.72	13.47	13.53	14.29
	1.686	-0.83	0.56	Q9JHJ0	Tropomodulin-3	Tmod3	5363	12	12	12	43.8	43.8	43.8	39.502	0.0000	56.689	30864000	223	15.62	16.59	15.63	16.01	16.74	17.09	16.43	16.93
	1.681	-0.83	0.56	P41105	60S ribosomal protein L28	Rpl28	1065	19	19	19	67.2	67.2	67.2	15.733	0.0000	179.58	410520000	646	19.23	18.79	18.43	19.02	19.56	20.28	19.30	19.67
	1.692	-0.84	0.56	Q6NSQ9	Glucose-6-phosphatase 3	G6pc3	2525	3	3	3	15.6	15.6	15.6	38.781	0.0000	15.915	1254000	16	12.94	12.21	12.56	13.27	13.63	13.37	13.39	13.95
	1.220	-0.84	0.56	Q9CQ25	Mitotic-spindle organizing protein 2	Mzt2	4555	4	4	4	54.1	54.1	54.1	16.524	0.0000	76.772	17536000	128	15.71	16.17	16.48	16.87	16.67	17.79	16.75	17.38
	3.211	-0.84	0.56	Q62425	Cytochrome c oxidase subunit NDUFA4	Ndufa4	2390	12	12	12	65.9	65.9	65.9	9.3267	0.0000	77.975	218640000	496	18.96	18.99	18.59	19.12	19.59	19.87	19.74	19.81
	1.026	-0.84	0.56	Q9R060	Cytosolic Fe-S cluster assembly factor NUBP1	Nubp1	5615	5	5	5	24.7	24.7	24.7	34.085	0.0000	33.373	7161600	74	13.88	14.78	13.74	13.81	14.54	15.70	14.15	15.19
	1.724	-0.84	0.56	O08709	Peroxiredoxin-6	Prdx6	288	22	22	22	82.6	82.6	82.6	24.87	0.0000	120.21	140420000	555	17.77	18.04	17.45	17.32	18.25	18.08	18.61	19.01
	1.579	-0.84	0.56	Q8CHI8	E1A-binding protein p400	Ep400	3524	10	10	10	5.1	5.1	5.1	337.18	0.0000	34.668	5846300	88	13.55	14.05	13.74	14.32	14.14	15.13	14.66	15.10
	1.179	-0.84	0.56	Q76KJ5	DNA-directed RNA polymerase I subunit RPA34	Cd3eap	2717	8	8	8	32.3	32.3	32.3	43.082	0.0000	32.675	6943900	62	12.85	13.39	13.56	13.77	13.61	14.17	15.12	14.04
	1.408	-0.84	0.56	Q9Z2C4	Myotubularin-related protein 1	Mtmr1	5839	4	4	4	8.8	8.8	8.8	75.313	0.0000	8.453	1097800	16	12.86	12.68	13.27	13.31	13.21	14.42	13.61	14.24
	0.805	-0.84	0.56	Q8VDI1	Epithelial-stromal interaction protein 1	Epsti1	3954	5	5	5	18.8	18.8	18.8	36.098	0.0000	11.815	3628100	30	12.89	13.51	13.50	12.84	13.14	15.29	13.40	14.28
	0.582	-0.84	0.56	Q8BZX4	Splicing regulatory glutamine/lysine-rich protein 1	Srek1	3321	4	4	4	11.3	11.3	11.3	56.763	0.0000	18.516	2024300	12	13.16	13.00	12.67	13.13	13.50	12.17	15.35	14.30
	2.598	-0.84	0.56	Q922B9	Sperm-specific antigen 2 homolog	Ssfa2	4249	14	14	14	15.5	15.5	15.5	136.95	0.0000	32.451	10306000	109	14.05	14.14	14.68	14.51	15.24	15.38	15.04	15.09
	0.998	-0.84	0.56	O70279	Protein DGCR14	Dgcr14	482	4	4	4	15	15	15	52.603	0.0000	56.808	4349200	85	13.09	14.37	13.70	14.48	14.39	14.53	14.48	15.63
	1.137	-0.85	0.56	P98086	Complement C1q subcomponent subunit A	C1qa	1687	3	3	3	18	18	18	25.974	0.0000	25.19	2984700	41	14.23	14.23	13.92	12.93	15.36	14.62	14.33	14.39
	2.773	-0.85	0.56	Q7TNV0	Protein DEK	Dek	2765	14	14	14	31.3	31.3	31.3	43.158	0.0000	52.303	43205000	276	16.73	17.16	16.80	17.18	17.75	18.12	17.66	17.73
	1.352	-0.85	0.55	Q9CYX7	RRP15-like protein	Rrp15	4809	4	4	4	14.6	14.6	14.6	31.058	0.0000	20.303	3720700	40	12.94	13.33	12.66	13.83	13.63	14.22	13.74	14.58
+	4.674	-0.85	0.55	Q91VY9	Zinc finger protein 622	Znf622	4085	13	13	13	37	37	37	53.451	0.0000	65.582	12109000	113	14.64	14.70	14.72	14.58	15.36	15.58	15.46	15.64
	1.635	-0.85	0.55	Q9DBC7	cAMP-dependent protein kinase type I-alpha regulatory subunit;cAMP-d	Prkar1a	5143	27	27	20	60.9	60.9	49.3	43.185	0.0000	218.86	174730000	827	17.42	17.97	17.01	17.79	18.03	18.39	18.26	18.91
	1.949	-0.86	0.55	Q8BPG6	Sulfatase-modifying factor 2	Sumf2	3164	3	3	3	13.6	13.6	13.6	34.737	0.0000	15.254	9776500	82	14.91	15.17	14.69	14.93	15.59	15.93	15.30	16.30
	0.684	-0.86	0.55	P69905	Hemoglobin subunit alpha	HBA1	1525	6	2	2	42.3	18.3	18.3	15.257	0.0002	5.3565	1957500	15	13.56	13.47	12.37	14.54	14.09	13.77	13.93	15.58
	0.205	-0.86	0.55	O55101	Synaptogyrin-2	Syngr2	452	2	2	2	12.9	12.9	12.9	24.778	0.0000	26.998	20013000	37	13.08	11.89	18.24	17.51	16.51	15.89	17.08	14.66
	1.278	-0.86	0.55	Q7TNS2	MICOS complex subunit Mic10	Minos1	2764	3	3	3	14.5	14.5	14.5	8.5669	0.0002	5.9123	70079000	24	18.97	19.19	18.74	18.75	18.92	19.97	19.65	20.56
	1.530	-0.86	0.55	Q99N87	28S ribosomal protein S5, mitochondrial	Mrps5	4487	10	10	10	20.1	20.1	20.1	48.206	0.0000	12.06	16008000	123	14.94	15.59	16.01	14.93	15.91	16.39	16.06	16.56
	0.845	-0.86	0.55	Q62432;Q8E	Mothers against decapentaplegic homolog 2	Smad2	2392	7	7	6	22.9	22.9	20.6	52.265	0.0000	204.61	18092000	88	15.31	16.16	17.18	16.76	17.27	17.96	17.20	16.43
	1.797	-0.86	0.55	P32067	Lupus La protein homolog	Ssb	995	28	28	28	62.7	62.7	62.7	47.756	0.0000	198.05	118300000	749	17.09	16.83	16.57	17.21	17.47	18.27	17.38	18.04
	1.630	-0.86	0.55	Q6PDY2	2-aminoethanethiol dioxygenase	Ado	2621	7	7	7	34.8	34.8	34.8	28.372	0.0000	13.023	13662000	76	15.69	15.54	15.12	14.66	15.84	15.83	16.52	16.27
	1.179	-0.87	0.55	Q62376	U1 small nuclear ribonucleoprotein 70 kDa	Snrnp70	2382	23	23	23	45.3	45.3	45.3	51.991	0.0000	150.02	52438000	393	15.77	16.80	15.46	16.33	16.75	17.20	16.37	17.50
	1.268	-0.87	0.55	P62984	Ubiquitin-60S ribosomal protein L40;Ubiquitin;60S ribosomal protein L4(	Uba52	1475	17	4	4	75	18.8	18.8	14.728	0.0000	46.761	67583000	342	16.51	17.52	16.08	16.84	17.55	17.75	17.09	18.03
	0.948	-0.87	0.55	Q9QXG4	Acetyl-coenzyme A synthetase, cytoplasmic	Acss2	5541	8	8	8	16.4	16.4	16.4	78.861	0.0000	41.689	4237900	18	13.65	13.67	13.27	12.67	14.07	15.28	14.07	13.32
	1.993	-0.87	0.55	P27546	Microtubule-associated protein 4	Map4	934	18	18	18	26	26	26	117.43	0.0000	119.31	27982000	300	15.31	15.17	15.72	16.01	16.29	16.63	16.10	16.66
	1.289	-0.87	0.55	Q8K330	Protein phosphatase Slingshot homolog 3	Ssh3	3686	13	13	13	32.2	32.2	32.2	72.227	0.0000	99.604	10938000	112	14.54	15.22	15.03	15.23	15.26	15.55	15.94	16.73
	0.521	-0.87	0.55	Q01149	Collagen alpha-2(I) chain	Col1a2	1705	5	5	5	5.2	5.2	5.2	129.56	0.0000	15.271	5083100	19	14.11	12.40	13.12	11.33	13.47	14.93	12.50	13.56
	2.520	-0.87	0.55	P35550	rRNA 2-O-methyltransferase fibrillarin	Fbl	1025	17	17	16	58.1	58.1	54.7	34.306	0.0000	134.48	59194000	397	16.70	16.66	16.29	16.40	17.01	17.71	17.28	17.53
	2.524	-0.87	0.55	P53996	Cellular nucleic acid-binding protein	Cnbp	1245	12	12	12	60.7	60.7	60.7	19.592	0.0000	78.186	80612000	269	16.58	17.36	17.00	17.18	17.86	17.95	17.73	18.07
	0.918	-0.87	0.55	Q9D162	Coiled-coil domain-containing protein 167	Ccdc167	4897	4	4	4	45.4	45.4	45.4	11.475	0.0000	7.7901	3241600	35	13.31	14.30	13.68	13.00	15.04	13.42	14.23	15.08
	1.126	-0.87	0.55	Q9QUH0	Glutaredoxin-1	Glrx	5513	12	12	12	95.3	95.3	95.3	11.871	0.0000	323.31	113980000	359	17.30	18.97	18.30	18.55	18.83	18.84	19.28	19.66
	1.246	-0.87	0.55	Q99LB0	Deoxynucleotidyltransferase terminal-interacting protein 1	Dnttip1	4412	7	7	7	30.5	30.5	30.5	36.852	0.0000	57.112	8053800	70	13.92	14.56	14.68	14.90	15.64	16.01	15.33	14.57
	0.889	-0.88	0.55	Q61193	Ral guanine nucleotide dissociation stimulator-like 2	Rgl2	2273	11	11	11	23.4	23.4	23.4	83.825	0.0000	60.953	9141300	76	14.75	15.16	14.59	14.23	14.96	16.90	15.38	14.99
	1.412	-0.88	0.55	Q61656	Probable ATP-dependent RNA helicase DDX5	Ddx5	2321	57	57	45	68.4	68.4	57.3	69.289	0.0000	323.31	452770000	1632	18.33	18.46	17.83	18.09	19.03	19.82	18.34	19.02
	1.164	-0.88	0.54	O89050	Muskelin	Mkln1	597	3	3	3	9.1	9.1	9.1	84.877	0.0000	9.9262	453370	5	11.84	12.87	11.60	11.90	13.09	12.29	12.72	13.61
	1.926	-0.88	0.54	P09405	Nucleolin	Ncl	695	36	36	36	42.1	42.1	42.1	76.722	0.0000	323.31	108680000	702	16.65	17.25	16.19	16.90	17.47	17.86	17.42	17.73
	1.782	-0.88	0.54	Q9QXN3	Activating signal cointegrator 1	Trip4	5547	6	6	6	16.7	16.7	16.7	66.197	0.0000	33.93	4656600	56	13.85	14.58	13.96	14.70	14.90	15.30	15.52	14.87
	1.062	-0.88	0.54	P23249	Putative helicase MOV-10	Mov10	881	13	13	13	17.3	17.3	17.3	113.58	0.0000	29.226	8321600	75	14.56	14.02	12.59	13.48	14.54	14.76	14.49	14.37
	1.587	-0.88	0.54	Q8BJ05	Zinc finger CCCH domain-containing protein 14	Zc3h14	3069	21	21	21	40.8	40.8	40.8	82.408	0.0000	145.18	35746000	340	14.96	15.61	15.10	15.83	15.98	16.23	15.94	16.88
	1.837	-0.89	0.54	P62627	Dynein light chain roadblock-type 1	Dynlrb1	1433	5	5	5	64.6	64.6	64.6	10.99	0.0000	17.687	18544000	91	15.98	16.52	16.96	16.15	17.11	17.72	17.16	17.16
	2.248	-0.89	0.54	Q00899;Q3T	Transcriptional repressor protein YY1	Yy1	1701	8	8	8	15.7	15.7	15.7	44.716	0.0000	46.454	5027100	108	13.87	14.22	13.73	14.09	14.61	15.16	14.51	15.19
	1.817	-0.89	0.54	Q91W39	Nuclear receptor coactivator 5	Ncoa5	4090	10	10	9	24	24	22.5	65.318	0.0000	21.478	9132200	99	14.52	14.53	13.61	13.95	14.81	15.35	14.82	15.17
	1.133	-0.89	0.54	G3X9J0		Sipa1l3	260	5	5	5	4.6	4.6	4.6	195.06	0.0000	14.975	1723100	35	12.77	13.19	12.76	13.29	12.90	14.48	13.65	14.54
	1.579	-0.89	0.54	Q9D967	Magnesium-dependent phosphatase 1	Mdp1	5098	7	7	7	61.6	61.6	61.6	18.582	0.0000	254.74	18876000	188	15.28	15.99	15.18	15.79	15.99	16.45	16.27	17.09
	2.285	-0.89	0.54	P35922	Fragile X mental retardation protein 1 homolog	Fmr1	1035	13	12	11	30.3	29	25.9	68.988	0.0000	103.18	13355000	185	14.97	15.27	14.76	15.53	15.72	16.09	15.99	16.30
	0.381	-0.89	0.54	Q8R3G1	Nuclear inhibitor of protein phosphatase 1	Ppp1r8	3846	5	5	5	21.4	21.4	21.4	38.527	0.0000	38.167	14487000	69	16.09	16.88	13.21	16.77	16.53	17.89	16.86	15.23
	2.082	-0.89	0.54	Q99KV1	DnaJ homolog subfamily B member 11	Dnajb11	4396	12	12	12	35.8	35.8	35.8	40.555	0.0000	107.63	47749000	229	16.45	17.15	16.98	17.45	17.96	18.14	17.73	17.76
+	2.378	-0.89	0.54	Q9CQT2	RNA-binding protein 7	Rbm7	4633	8	8	8	47.2	47.2	47.2	30.148	0.0000	116.61	29793000	199	16.22	16.88	16.12	16.24	17.18	17.48	17.03	17.36
	1.708	-0.90	0.54	Q99N94	39S ribosomal protein L9, mitochondrial	Mrpl9	4492	10	10	10	35.1	35.1	35.1	30.244	0.0000	23.118	13752000	118	15.77	15.35	14.68	15.38	15.72	16.31	16.22	16.54
	1.351	-0.90	0.53	Q9JI19	Acidic fibroblast growth factor intracellular-binding protein	Fibp	5384	2	2	2	10.1	10.1	10.1	41.204	0.0000	32.738	1161700	22	12.10	12.59	12.91	12.68	12.90	13.67	13.05	14.27
	1.660	-0.90	0.53	A2AI08	Taperin	Tprn	23	4	4	4	9.1	9.1	9.1	80.088	0.0000	12.324	2698800	25	13.60	13.46	13.07	13.60	13.96	14.39	13.92	15.07
	0.352	-0.90	0.53	Q9DA08	SAGA-associated factor 29 homolog	Ccdc101	5104	3	3	3	15	15	15	33.298	0.0018	3.1263	2655200	33	13.11	14.26	14.02	13.35	16.57	12.93	16.31	12.55
	1.550	-0.91	0.53	Q8CDN6	Thioredoxin-like protein 1	Txnl1	3464	11	11	11	63	63	63	32.237	0.0000	153.21	32609000	204	15.42	16.35	15.83	14.87	16.49	16.52	16.48	16.62
	1.759	-0.91	0.53	P54923	[Protein ADP-ribosylarginine] hydrolase	Adprh	1262	15	15	15	58.6	58.6	58.6	40.068	0.0000	167.14	40939000	205	16.22	16.65	16.79	15.89	17.36	17.80	16.96	17.07
	0.913	-0.91	0.53	Q9CQH7	Transcription factor BTF3 homolog 4	Btf3l4	4600	7	6	6	59.5	55.7	55.7	17.27	0.0000	99.129	23621000	213	15.09	16.50	14.58	16.17	16.06	16.69	16.18	17.03
	1.965	-0.91	0.53	Q8CGP5	Histone H2A type 1-F	Hist1h2af	3512	18	5	1	80	28.5	6.9	14.161	0.0000	154.48	2311700000	2259	22.37	22.36	22.35	22.71	23.14	23.90	22.83	23.55
	1.119	-0.91	0.53	Q9CPW7	Zinc finger matrin-type protein 2	Zmat2	4540	5	5	5	21.1	21.1	21.1	23.612	0.0000	7.4486	4330100	30	13.64	14.56	13.30	13.51	14.26	14.52	14.26	15.61
	1.945	-0.91	0.53	Q80SZ7	Guanine nucleotide-binding protein G(I)/G(S)/G(O) subunit gamma-5	Gng5	2798	5	5	5	51.5	51.5	51.5	7.3184	0.0000	9.9109	29548000	117	17.55	17.21	17.04	17.21	17.54	18.38	18.13	18.61
	1.508	-0.91	0.53	P10711;Q9C	Transcription elongation factor A protein 1	Tcea1	728	28	28	28	78.4	78.4	78.4	33.88	0.0000	323.31	100550000	677	15.79	16.72	15.78	16.54	16.74	17.58	16.78	17.39
	0.591	-0.91	0.53	Q673H1	Tumor suppressor candidate gene 1 protein homolog	Tusc1	2469	4	4	4	18	18	18	22.712	0.0000	22.836	4551100	61	13.43	15.28	13.34	14.79	14.46	15.34	14.14	16.57
+	3.145	-0.91	0.53	Q8CGP1	Histone H2B type 1-K	Hist1h2bk	3511	27	2	0	96	13.5	0	13.92	0.0026	2.944	14130000	35	16.68	16.59	16.75	16.49	17.61	17.85	17.21	17.49
	0.404	-0.91	0.53	Q8BM85	TBC domain-containing protein kinase-like protein	Tbck	3129	8	8	8	18.5	18.5	18.5	86.37	0.0000	25.333	28024000	54	17.73	17.02	16.97	16.09	15.33	19.17	17.56	19.40
+	3.186	-0.92	0.53	P61082	NEDD8-conjugating enzyme Ubc12	Ube2m	1369	12	12	11	55.7	55.7	54.1	20.9	0.0000	22.889	81727000	247	17.64	17.82	18.08	17.84	18.48	18.82	18.73	19.01
	1.416	-0.92	0.53	Q99MU3	Double-stranded RNA-specific adenosine deaminase	Adar	4478	30	30	30	34.4	34.4	34.4	130.45	0.0000	222.66	29708000	259	15.28	15.55	14.44	15.39	15.80	16.75	15.67	16.12
+	2.167	-0.92	0.53	P0C0A3	Charged multivesicular body protein 6	Chmp6	704	6	6	6	29.5	29.5	29.5	23.415	0.0000	43.543	21639000	161	15.39	15.54	16.14	16.00	16.38	16.93	16.51	16.92
	0.891	-0.92	0.53	Q71FD5	E3 ubiquitin-protein ligase ZNRF2	Znrf2	2714	4	4	4	42.9	42.9	42.9	23.705	0.0000	45.245	6478700	87	14.56	15.05	13.22	15.56	15.09	15.65	15.72	15.62
	1.423	-0.92	0.53	A6X919	Probable C-mannosyltransferase DPY19L1	Dpy19l1	65	5	5	5	10.9	10.9	10.9	84.186	0.0000	25.285	25299000	56	16.11	16.71	16.15	15.94	17.00	17.90	16.44	17.26
	1.953	-0.93	0.53	P57759	Endoplasmic reticulum resident protein 29	Erp29	1298	21	21	21	63.7	63.7	63.7	28.823	0.0000	300.32	223410000	756	18.18	19.09	18.12	18.55	19.22	19.64	19.18	19.62
	1.201	-0.93	0.53	P58242	Acid sphingomyelinase-like phosphodiesterase 3b	Smpdl3b	1311	10	10	10	32	32	32	51.599	0.0000	50.671	10404000	141	13.34	13.29	14.74	14.32	15.13	15.21	14.47	14.60
	1.286	-0.93	0.53	Q9CQS8	Protein transport protein Sec61 subunit beta	Sec61b	4630	3	3	3	37.5	37.5	37.5	9.9583	0.0000	11.734	28652000	125	15.83	16.75	17.28	17.45	17.60	17.82	17.54	18.07
+	2.970	-0.93	0.53	Q8BTW3	Exosome complex component MTR3	Exosc6	3209	10	10	10	44	44	44	28.37	0.0000	159.82	15029000	132	15.94	15.97	15.67	16.08	16.62	17.23	16.75	16.80
	1.946	-0.93	0.52	Q9Z2Y8	Proline synthase co-transcribed bacterial homolog protein	Prosc	5873	17	17	17	65	65	65	30.048	0.0000	126.8	135650000	463	17.73	18.07	18.19	18.48	18.48	19.06	19.21	19.45
	1.136	-0.93	0.52	Q3TEA8	Heterochromatin protein 1-binding protein 3	Hp1bp3	1836	28	28	28	47.8	47.8	47.8	60.866	0.0000	184.22	116900000	565	17.45	17.38	17.42	17.59	17.97	19.61	17.67	18.34
	1.319	-0.93	0.52	Q61464	Zinc finger protein 638	Znf638	2302	17	17	17	12.4	12.4	12.4	218.13	0.0000	100.28	18872000	131	14.63	15.69	14.85	15.55	15.50	16.55	15.81	16.60
+	2.038	-0.94	0.52	Q8QZT1	Acetyl-CoA acetyltransferase, mitochondrial	Acat1	3735	31	31	31	72.4	72.4	72.4	44.816	0.0000	323.31	264330000	981	18.07	18.72	18.80	18.99	19.24	19.75	19.45	19.88
	0.682	-0.94	0.52	E9Q784		Zc3h13	245	8	8	8	6.2	6.2	6.2	203.75	0.0000	16.216	3851700	48	12.36	13.77	12.67	14.42	13.34	14.52	13.70	15.40

	1.510	-0.94	0.52	Q9QY24	Z-DNA-binding protein 1	Zbp1	5560	8	8	8	17.3	17.3	17.3	44.331	0.0000	16.747	17439000	42	15.18	15.85	16.46	16.16	16.57	17.01	16.50	17.32
	1.365	-0.94	0.52	Q80UZ2	Protein SDA1 homolog	Sdad1	2843	4	4	4	7	7	7	79.558	0.0000	11.531	3866800	39	13.97	14.25	13.25	14.77	15.54	14.85	14.89	14.72
+	2.286	-0.94	0.52	P10853;Q8C	Histone H2B type 1-F/J/L;Histone H2B type 1-P	Hist1h2bf;Hist1h2	732	27	27	0	96	96	0	13.936	0.0000	323.31	7820800000	3130	23.38	23.35	23.30	23.38	24.20	24.88	23.83	24.27
	0.765	-0.94	0.52	Q6NZM9	Histone deacetylase 4	Hdac4	2547	10	10	9	13.8	13.8	13.2	118.56	0.0000	27.361	5763100	48	12.20	14.27	14.81	14.40	14.73	15.02	14.47	15.22
+	2.010	-0.95	0.52	Q8VEK3	Heterogeneous nuclear ribonucleoprotein U	Hnrnpu	4011	43	43	43	46.5	46.5	46.5	87.917	0.0000	323.31	550140000	1607	19.02	19.09	19.02	19.26	19.63	20.70	19.70	20.15
+	2.699	-0.95	0.52	Q8C163	Nuclease EXOG, mitochondrial	Exog	3354	14	14	13	50.8	50.8	48.6	41.383	0.0000	100.84	14872000	177	14.71	15.01	15.27	14.71	15.77	16.22	15.65	15.84
	0.541	-0.95	0.52	Q8VC31	Coiled-coil domain-containing protein 9	Ccdc9	3908	3	3	3	7.6	7.6	7.6	61.445	0.0000	6.3492	1179800	6	12.34	12.17	11.22	12.45	11.25	14.33	12.19	14.21
+	2.334	-0.95	0.52	P20152;P46	Vimentin	Vim	855	100	100	85	98.1	98.1	93.1	53.687	0.0000	323.31	13470000000	12596	22.12	22.70	22.19	22.66	23.05	23.47	23.21	23.75
	0.645	-0.95	0.52	P68373;Q9J	Tubulin alpha-1C chain	Tuba1c	1519	36	3	3	72.6	11.4	11.4	49.909	0.0000	137.79	27565000	160	14.55	16.94	16.54	16.38	17.69	17.15	17.65	15.72
	0.851	-0.95	0.52	A2RSY1	KAT8 regulatory NSL complex subunit 3	Kansl3	48	8	8	8	19	19	19	96.134	0.0000	78.995	4040500	59	12.75	13.89	14.44	13.59	13.56	14.28	15.12	15.50
	1.828	-0.95	0.52	Q9WVA4;Q9	Transgelin-2	Tagln2	5744	32	32	32	99.5	99.5	99.5	22.395	0.0000	323.31	1284400000	1747	19.99	21.00	20.22	20.24	21.21	21.32	20.94	21.78
+	2.287	-0.95	0.52	Q05CL8	La-related protein 7	Larp7	1746	22	22	22	44.9	44.9	44.9	64.802	0.0000	56.633	23866000	182	15.51	15.57	16.06	15.61	16.30	17.08	16.39	16.80
	0.861	-0.95	0.52	Q64152	Transcription factor BTF3	Btf3	2421	14	14	13	75.5	75.5	71.1	22.031	0.0000	323.31	119920000	520	16.16	18.26	16.37	17.86	17.74	18.17	17.96	18.60
+	2.044	-0.95	0.52	Q6PFD9	Nuclear pore complex protein Nup98-Nup96;Nuclear pore complex prot	Nup98	2627	44	44	44	32.8	32.8	32.8	197.24	0.0000	323.31	121540000	795	17.02	16.93	17.06	16.84	17.69	18.48	17.36	18.13
+	2.376	-0.95	0.52	P31725	Protein S100-A9	S100a9	988	4	4	4	32.7	32.7	32.7	13.049	0.0000	59.567	4990500	61	12.94	13.47	12.84	13.27	14.33	13.65	14.09	14.27
	1.007	-0.96	0.52	P49290;REV	Eosinophil peroxidase;Eosinophil peroxidase light chain;Eosinophil perc	Epx	1158	32	29	29	47.8	44.4	44.4	81.379	0.0000	126.21	111860000	534	16.65	16.43	17.89	17.97	17.42	18.60	18.17	18.57
+	2.315	-0.96	0.51	Q9QYY8	Spastin	Spast	5581	20	20	20	44.1	44.1	44.1	66.455	0.0000	152.98	33581000	296	15.53	15.63	15.65	16.04	16.33	16.98	16.37	17.04
	1.366	-0.97	0.51	Q9CQE5	Regulator of G-protein signaling 10	Rgs10	4589	15	15	15	77.9	77.9	77.9	21.151	0.0000	188.32	147400000	496	17.14	18.20	17.01	18.05	18.30	18.58	18.20	19.18
	1.216	-0.97	0.51	Q9Z2D6	Methyl-CpG-binding protein 2	Mecp2	5844	6	6	5	16.3	16.3	13.8	52.307	0.0000	10.869	2703800	13	13.38	12.75	13.47	12.37	13.37	14.82	13.53	14.10
	0.940	-0.97	0.51	P56376	Acylphosphatase-1	Acyp1	1280	4	4	4	52.5	52.5	52.5	11.241	0.0000	13.297	5617800	58	13.44	14.57	14.07	15.68	15.16	16.02	14.99	15.45
	1.185	-0.97	0.51	Q8VEH3	ADP-ribosylation factor-like protein 8A	Arl8a	4005	9	4	4	53.2	25.8	25.8	21.39	0.0000	23.116	49023000	122	17.64	16.69	18.18	16.37	18.42	18.28	18.08	17.97
+	2.846	-0.97	0.51	P63001	Ras-related C3 botulinum toxin substrate 1	Rac1	1477	18	8	5	64.1	33.9	24.5	21.45	0.0000	35.548	267020000	512	18.74	18.84	19.02	18.57	19.57	20.02	19.45	20.00
+	2.661	-0.97	0.51	Q8BW00	Probable peptidyl-tRNA hydrolase	Ptrh1	3252	5	5	5	40.2	40.2	40.2	22.16	0.0000	40.077	8688900	37	15.71	15.61	15.58	15.40	16.59	16.04	16.67	16.88
	0.724	-0.97	0.51	Q8BXL7	ADP-ribosylation factor-related protein 1	Arfrp1	3288	4	4	3	31.8	31.8	29.4	22.659	0.0004	5.0351	3073200	37	12.40	12.77	14.66	14.26	14.73	15.16	13.50	14.59
+	1.766	-0.97	0.51	Q9CWE0	Mitochondrial fission regulator 1-like	Mtfr1l	4711	7	7	7	39.1	39.1	39.1	31.726	0.0000	21.674	6348400	92	13.74	14.63	13.83	14.48	14.65	15.18	15.12	15.61
+	1.934	-0.97	0.51	Q6GSS7;Q6	Histone H2A type 2-A;Histone H2A type 2-C	Hist2h2aa1;Hist2h	2515	19	13	4	77.7	66.2	28.5	14.095	0.0000	323.31	5547600000	1865	24.34	24.90	24.39	24.94	25.16	25.75	25.39	26.16
+	2.358	-0.97	0.51	Q9DBR1	5-3 exoribonuclease 2	Xrn2	5170	34	34	34	37.2	37.2	37.2	108.69	0.0000	279.99	117430000	675	16.84	16.87	16.44	16.64	17.41	18.13	17.29	17.84
+	1.794	-0.97	0.51	Q9DB96	Neuroguidin	Ngdn	5135	6	6	6	31.1	31.1	31.1	35.658	0.0000	42.289	10529000	113	14.61	15.02	15.26	15.50	15.57	16.52	15.82	16.36
	1.513	-0.97	0.51	O70200	Allograft inflammatory factor 1	Aif1	477	5	5	5	37.4	37.4	37.4	16.91	0.0000	33.897	5939900	53	14.30	14.24	14.37	14.04	14.76	16.21	15.07	14.81
	1.541	-0.97	0.51	Q8R0A0	General transcription factor IIF subunit 2	Gtf2f2	3753	13	13	13	52.6	52.6	52.6	28.381	0.0000	215.02	89164000	383	16.41	17.59	16.78	17.39	17.65	18.07	17.78	18.57
+	1.992	-0.98	0.51	O09167	60S ribosomal protein L21	Rpl21	329	18	18	18	70	70	70	18.562	0.0000	142.75	410760000	382	19.89	19.64	19.43	19.68	20.04	21.08	20.41	21.00
	1.372	-0.98	0.51	Q9WU84	Copper chaperone for superoxide dismutase	Ccs	5701	7	7	7	38	38	38	28.911	0.0000	42.69	10476000	79	15.48	15.76	14.41	14.91	15.55	16.01	16.22	16.67
+	2.133	-0.98	0.51	P25976	Nucleolar transcription factor 1	Ubtf	913	29	29	29	35.4	35.4	35.4	89.508	0.0000	129.12	40897000	356	14.92	15.68	15.35	15.36	16.07	16.70	15.89	16.55
+	2.193	-0.98	0.51	Q8R404	Protein QIL1	Qil1	3861	6	6	6	86.6	86.6	86.6	13.373	0.0000	225.69	29793000	210	16.05	16.13	16.30	16.66	17.00	17.07	17.12	17.84
	1.283	-0.98	0.51	P61255	60S ribosomal protein L26	Rpl26	1381	22	22	21	77.2	77.2	72.4	17.258	0.0000	142.62	375250000	869	19.12	18.86	17.66	18.88	19.29	19.91	19.12	20.11
+	2.354	-0.98	0.51	P24452	Macrophage-capping protein	Capg	897	24	24	24	52.6	52.6	52.6	39.24	0.0000	323.31	2872900000	2006	21.21	21.83	22.06	21.83	22.37	22.85	22.68	22.95
	1.156	-0.98	0.51	Q9CY73	39S ribosomal protein L44, mitochondrial	Mrpl44	4790	10	10	10	37.2	37.2	37.2	37.527	0.0000	43.835	15612000	96	15.68	15.70	14.59	16.03	16.79	17.23	15.86	16.06
	0.904	-0.98	0.51	Q3U5Q7	UMP-CMP kinase 2, mitochondrial	Cmpk2	1927	22	22	22	62.2	62.2	62.2	50.036	0.0000	181.13	48650000	332	16.24	16.35	15.75	14.85	17.25	17.75	15.92	16.19
+	2.111	-0.98	0.51	P27048;P63	Small nuclear ribonucleoprotein-associated protein B;Small nuclear ribo	Snrpb;Snrpn	933	8	8	8	30.7	30.7	30.7	23.656	0.0000	18.195	92489000	255	17.20	18.09	17.62	18.09	18.93	18.96	18.40	18.65
	0.779	-0.98	0.51	Q9D168	Integrator complex subunit 12	Ints12	4898	4	4	4	11.9	11.9	11.9	48.568	0.0000	11.934	3405400	36	14.01	13.49	13.41	14.84	14.47	16.13	13.69	15.39
+	2.184	-0.98	0.51	Q99N92	39S ribosomal protein L27, mitochondrial	Mrpl27	4490	7	7	7	52.7	52.7	52.7	15.944	0.0000	35.442	25475000	165	15.65	16.54	15.67	16.38	17.01	17.09	16.90	17.17
+	1.738	-0.98	0.51	Q9D1J3	SAP domain-containing ribonucleoprotein	Sarnp	4921	13	13	13	51.4	51.4	51.4	23.532	0.0000	266.03	67058000	429	15.29	16.15	15.54	16.49	16.67	17.09	16.58	17.07
+	1.865	-0.99	0.51	Q8VEE0	Ribulose-phosphate 3-epimerase	Rpe	4001	4	4	4	29.4	29.4	29.4	24.945	0.0000	29.046	18452000	163	15.18	16.38	15.52	15.93	16.49	16.85	16.59	17.03
+	2.096	-0.99	0.51	P97855	Ras GTPase-activating protein-binding protein 1	G3bp1	1680	19	19	16	47.1	47.1	43.4	51.828	0.0000	196.79	102410000	630	16.30	17.09	16.92	17.07	17.46	17.95	17.67	18.25
+	2.157	-0.99	0.50	P55194	SH3 domain-binding protein 1	Sh3bp1	1268	28	28	28	48.2	48.2	48.2	74.172	0.0000	318.41	123540000	719	16.99	17.49	17.22	17.63	17.89	18.65	18.07	18.67
+	1.852	-0.99	0.50	O35691	Pinin	Pnn	399	24	24	24	26.5	26.5	26.5	82.435	0.0000	132.43	54371000	412	15.56	15.81	16.11	16.35	16.28	17.08	17.08	17.33
	1.431	-0.99	0.50	P21619	Lamin-B2	Lmnb2	867	59	50	49	80	72.8	70	67.317	0.0000	323.31	102620000	812	16.23	16.15	15.59	16.09	17.15	17.76	16.11	16.98
	0.548	-0.99	0.50	Q9CR02	Translation machinery-associated protein 16	Tma16	4654	7	7	7	28.1	28.1	28.1	25.782	0.0000	23.363	7080300	75	12.80	14.78	12.35	14.59	13.28	14.68	14.47	16.06
+	1.795	-0.99	0.50	Q9Z0V7	Mitochondrial import inner membrane translocase subunit Tim17-B	Timm17b	5788	3	3	3	30.2	30.2	30.2	18.352	0.0000	20.052	5128400	47	14.12	14.62	15.49	15.04	15.97	15.70	15.75	15.81
+	2.151	-1.00	0.50	Q9QZQ8;Q8	Core histone macro-H2A.1	H2afy	5604	23	23	23	61.3	61.3	61.3	39.735	0.0000	323.31	302290000	884	19.19	19.27	19.20	19.11	19.95	20.91	19.81	20.09
	1.236	-1.00	0.50	Q9QWT9	Kinesin-like protein KIFC1	Kifc1	5528	5	5	5	9.6	9.6	9.6	74.152	0.0000	6.791	2675000	17	13.19	12.94	13.94	14.02	14.79	14.34	13.70	15.25
+	2.110	-1.00	0.50	Q99J09	Methylosome protein 50	Wdr77	4316	5	5	5	28.4	28.4	28.4	36.942	0.0000	63.285	3297700	37	12.59	13.43	13.36	13.08	14.54	13.84	14.22	13.85
	1.399	-1.00	0.50	A2AJI0	MAP7 domain-containing protein 1	Map7d1	29	16	16	15	24	24	23.3	93.275	0.0000	30.391	14740000	171	13.80	14.67	14.84	14.81	15.19	16.39	15.34	15.18
+	1.722	-1.00	0.50	Q8BKZ9	Pyruvate dehydrogenase protein X component, mitochondrial	Pdhx	3109	16	16	16	33.7	33.7	33.7	53.998	0.0000	136.27	24795000	255	15.22	15.50	15.28	15.99	15.93	16.41	16.46	17.20
	0.949	-1.01	0.50	Q9D7Z3	Nucleolar protein 7	Nol7	5045	4	4	4	18.9	18.9	18.9	28.97	0.0000	11.54	3049200	25	12.52	13.24	13.90	13.47	13.42	15.54	13.85	14.34
	1.150	-1.02	0.49	Q8CHP0	Zinc finger CCCH domain-containing protein 3	Zc3h3	3526	5	5	5	6	6	6	103.22	0.0024	2.9979	2354800	19	13.11	13.15	14.17	13.69	14.24	15.45	13.64	14.85
	0.756	-1.02	0.49	Q9Z183;Q9Z	Protein-arginine deiminase type-4	Padi4	5802	6	6	5	14.4	14.4	10.1	74.414	0.0000	56.309	6587100	46	15.22	12.79	13.43	14.36	15.68	15.52	13.98	14.68
+	1.793	-1.02	0.49	P19253	60S ribosomal protein L13a	Rpl13a	844	22	22	22	70.4	70.4	70.4	23.464	0.0000	73.545	171710000	504	18.37	18.41	18.87	17.92	19.19	19.72	18.84	19.88
	0.874	-1.02	0.49	Q64092	Transcription factor E3	Tfe3	2413	8	8	4	13.5	13.5	7.5	61.535	0.0000	9.8491	11468000	60	15.91	15.82	14.21	14.11	15.92	16.29	15.20	16.72
+	1.645	-1.02	0.49	Q61048	WW domain-binding protein 4	Wbp4	2243	7	7	7	24.5	24.5	24.5	42.136	0.0000	50.369	84338000	52	17.76	19.19	18.39	18.60	19.15	19.90	19.38	19.58
+	1.897	-1.03	0.49	Q9QYJ3	DnaJ homolog subfamily B member 1	Dnajb1	5576	8	8	7	28.2	28.2	24.7	38.167	0.0000	36.573	15276000	118	15.13	14.74	15.83	15.87	16.23	16.42	16.36	16.67
+	1.590	-1.03	0.49	P83882	60S ribosomal protein L36a	Rpl36a	1603	15	15	15	63.2	63.2	63.2	12.441	0.0000	32.767	132830000	468	18.06	19.11	17.75	18.63	19.14	19.73	19.09	19.70
+	3.393	-1.03	0.49	O88456;Q9D	Calpain small subunit 1	Capns1	533	26	26	26	66.5	66.5	66.5	28.463	0.0000	323.31	647540000	1424	19.69	19.86	19.32	19.63	20.68	20.86	20.41	20.67
	1.112	-1.03	0.49	B1AVZ0	Uracil phosphoribosyltransferase homolog	Uprt	71	4	4	4	18.4	18.4	18.4	34.278	0.0000	13.225	6103300	48	14.73	15.11	14.29	12.92	15.16	15.16	15.41	15.45
+	2.061	-1.04	0.49	Q922U1	U4/U6 small nuclear ribonucleoprotein Prp3	Prpf3	4278	24	24	24	41.9	41.9	41.9	77.454	0.0000	158.4	61316000	457	15.53	15.70	15.46	16.15	16.16	17.01	16.66	17.16
	0.874	-1.04	0.49	P54227	Stathmin	Stmn1	1249	5	5	4	38.9	38.9	32.9	17.274	0.0000	8.5728	3097200	22	13.93	13.63	11.87	14.01	15.36	14.12	14.05	14.05
	0.630	-1.04	0.49	Q9CY50	Translocon-associated protein subunit alpha	Ssr1	4784	3	3	3	17.1	17.1	17.1	32.065	0.0000	6.5913	4402400	47	12.86	14.04	14.77	14.89	16.08	15.92	15.37	13.34
	1.001	-1.04	0.49	Q9D832	DnaJ homolog subfamily B member 4	Dnajb4	5049	6	5	5	28.8	25.2	25.2	37.781	0.0000	11.215	8238600	49	14.21	15.40	16.10	14.14	15.33	16.27	15.99	16.41
+	2.009	-1.04	0.49	Q8VHR5	Transcriptional repressor p66-beta	Gatad2b	4024	22	22	22	46.5	46.5	46.5	65.41	0.0000	140.82	58977000	407	15.91	16.50	15.81	16.24	16.80	17.38	16.73	17.69
+	1.890	-1.04	0.49	Q9CWW6	Peptidyl-prolyl cis-trans isomerase NIMA-interacting 4	Pin4	4735	11	11	11	74.8	74.8	74.8	13.815	0.0000	206.16	101700000	270	17.13	18.32	17.38	17.71	18.32	18.73	18.60	19.05
+	1.575	-1.04	0.49	P84228	Histone H3.2	Hist1h3b	1613	22	22	2	88.2	88.2	24.3	15.388	0.0000	323.31	2035400000	1151	22.48	22.28	21.94	22.09	22.90	24.16	22.60	23.31
	0.563	-1.04	0.48	Q3U1G5	Interferon-stimulated 20 kDa exonuclease-like 2	Isg20l2	1901	4	4	4	10.9	10.9	10.9	41.019	0.0000	9.8449	3040500	38	12.26	12.96	15.23	13.82	14.26	16.28	14.34	13.57
	0.873	-1.04	0.48	Q9CY21	Probable 18S rRNA (guanine-N(7))-methyltransferase	Wbscr22	4779	3	3	3	18.9	18.9	18.9	31.586	0.0000	44.267	4391900	57	14.89	13.46	12.28	14.62	14.73	14.92	14.71	15.07
	0.645	-1.04	0.48	Q5PSV9	Mediator of DNA damage checkpoint protein 1	Mdc1	2124	4	4	4	3.7	3.7	3.7	184.67	0.0004	4.3099	1009000	20	12.48	11.24	11.39	13.71	12.94	14.62	12.10	13.34
+	1.449	-1.05	0.48	P62320	Small nuclear ribonucleoprotein Sm D3	Snrpd3	1424	9	9	9	58.7	58.7	58.7	13.916	0.0000	143.76	117300000	339	18.73	19.13	17.75	18.62	19.39	20.33	19.14	19.55
+	1.607	-1.05	0.48	P12970	60S ribosomal protein L7a	Rpl7a	761	26	26	26	60.2	60.2	60.2	29.976	0.0000	323.31	465520000	828	19.26	19.21	18.80	19.18	19.70	21.08	19.62	20.25
+	2.274	-1.05	0.48	O35326	Serine/arginine-rich splicing factor 5	Srsf5	357	10	9	9	28.3	24.9	24.9	30.891	0.0000	77.042	29918000	258	15.42	16.25	15.13	15.58	16.72	16.78	16.63	16.46
+	3.306	-1.05	0.48	Q8VH51	RNA-binding protein 39	Rbm39	4016	20	20	20	37.9	37.9	37.9	59.406	0.0000	280.68	79249000	435	16.72	17.07	17.21	16.96	17.78	18.34	18.00	18.05
+	2.060	-1.06	0.48	Q9JM14	5(3)-deoxyribonucleotidase, cytosolic type	Nt5c	5497	8	8	8	54	54	54	23.076	0.0000	109.72	27120000	256	16.03	16.60	15.48	16.04	17.14	17.18	16.66	17.40
+	1.405	-1.06	0.48	Q8CFB4	Guanylate-binding protein 5	Gbp5	3478	10	9	9	30	26.8	26.8	66.929	0.0000	22.033	3520600	39	12.93	13.17	12.98	12.43	14.91	14.11	13.27	13.45
+	1.369	-1.06	0.48	P48771	Cytochrome c oxidase subunit 7A2, mitochondrial	Cox7a2	1150	5	5	5	59	59	59	9.2908	0.0000	117.3	22155000	131	16.15	16.24	16.07	14.63	16.78	16.63	16.62	17.30
	0.510	-1.06	0.48	P35762	CD81 antigen	Cd81	1032	1	1	1	8.5	8.5	8.5	25.814	0.0000	69.602	7115100	53	13.06	12.95	16.22	16.38	15.56	16.01	15.65	15.63
+	1.607	-1.07	0.48	Q8R1X6	Spartin	Spg20	3803	8	8	7	20.3	20.3	17.9	72.654	0.0000	31.108	4202500	66	13.28	14.35	13.25	12.90	14.38	14.94	14.13	14.60
	1.249	-1.07	0.48	Q8R0F8	Acylpyruvase FAHD1, mitochondrial	Fahd1	3757	3	3	3	28.2	28.2	28.2	25.172	0.0000	32.541	2157600	27	13.04	13.58	14.64	13.26	14.81	15.24	14.86	13.90
+	2.102	-1.07	0.48	Q7TNC4	Putative RNA-binding protein Luc7-like 2	Luc7l2	2758	28	28	22	49.5	49.5	41.1	46.582	0.0000	223.96	120610000	710	17.52	17.49	17.13	17.07	18.38	18.89	17.70	18.53
	0.745	-1.07	0.48	P05977;P09	Myosin light chain 1/3, skeletal muscle isoform	Myl1	654	3	3	3	19.1	19.1	19.1	20.594	0.0000	8.5512	3496900	45	13.50	14.58	12.02	12.84	15.52	14.36	14.02	13.34
+	1.708	-1.07	0.48	Q9QXA5	U6 snRNA-associated Sm-like protein LSm4	Lsm4	5534	11	11	11	58.4	58.4	58.4	15.076	0.0000	56.37	52565000	273	15.91	17.17	16.15	16.54	17.21	17.98	17.16	17.72
+	4.197	-1.08	0.47	P62806	Histone H4	Hist1h4a	1445	24	24	24	77.7	77.7	77.7	11.367	0.0000	323.31	11310000000	7987	23.29	23.33	23.23	23.38	24.43	24.60	24.10	24.41
+	2.276	-1.08	0.47	Q91WB4	Pleckstrin homology domain-containing family F member 2	Plekhf2	4102	7	7	7	52.6	52.6	52.6	27.754	0.0000	19.845	11794000	87	14.12	14.47	14.34	15.16	15.75	15.59	15.79	15.27
	0.799	-1.08	0.47	P97425	Eosinophil cationic protein 2	Ear2	1645	4	4	3	30.1	30.1	17.9	17.62	0.0000	67.054	69505000	106	17.57	18.47	18.61	18.62	17.91	18.92	20.02	20.75
+	2.747	-1.08	0.47	P84104	Serine/arginine-rich splicing factor 3	Srsf3	1612	11	11	10	45.1	45.1	45.1	19.329	0.0000	120.85	174890000	451	18.44	18.83	18.05	18.32	19.42	19.68	19.18	19.70
+	2.092	-1.09	0.47	P61358	60S ribosomal protein L27	Rpl27	1384	14	14	14	60.3	60.3	60.3	15.798	0.0000	225.62	374280000	648	19.30	19.31	19.08	19.67	19.89	20.79	20.11	20.92
+	1.615	-1.09	0.47	Q9D172	ES1 protein homolog, mitochondrial	D10Jhu81e	4899	17	17	17	69.2	69.2	69.2	28.09	0.0000	238.25	92740000	461	16.61	17.87	16.69	17.58	17.80	18.39	18.26	18.65
+	1.407	-1.09	0.47	Q64213	Splicing factor 1	Sf1	2423	24	24	24	31.2	31.2	31.2	70.402	0.0000	183.7	104770000	592	16.51	17.08	15.54	16.74	17.33	17.63	17.05	18.21
	0.987	-1.09	0.47	Q8K2T8	RNA polymerase II-associated factor 1 homolog	Paf1	3673	4	4	4	11.2	11.2	11.2	60.518	0.0000	14.616	2773500	37	13.08	14.00	13.48	12.01	13.23	15.01	14.55	14.15
+	1.284	-1.09	0.47	Q9CQZ5	NADH dehydrogenase [ubiquinone] 1 alpha subcomplex subunit 6	Ndufa6	4651	5	5	5	45.8	45.8	45.8	15.283	0.0000	37.458	8055700	84	13.07	15.09	14.64	14.44	15.62	15.15	15.61	15.23
	1.087	-1.09	0.47	P61963	DDB1- and CUL4-associated factor 7	Dcaf7	1394	4	4	4	12.6	12.6	12.6	38.926	0.0000	7.3118	4570900	54	12.98	13.97	14.99	12.95	14.23	14.91	15.07	15.05
+	3.000	-1.09	0.47	P17742	Peptidyl-prolyl cis-trans isomerase A;Peptidyl-prolyl cis-trans isomerase	Ppia	829	24	24	9	95.7	95.7	43.3	17.971	0.0000	323.31	4129500000	3245	21.40	21.93	21.52	21.96	22.76	22.95	22.49	23.00
+	2.084	-1.10	0.47	P63280;P63	SUMO-conjugating enzyme UBC9	Ube2i;UBE2I	1502	8	8	8	54.4	54.4	54.4	18.007	0.0000	213.83	46838000	170	16.44	17.25	17.01	16.94	17.45	18.42	17.83	18.34
+	2.449	-1.10	0.47	Q9CQA6	Coiled-coil-helix-coiled-coil-helix domain-containing protein 1	Chchd1	4579	2	2	2	16.1	16.1	16.1	13.608	0.0000	66.402	13143000	67	15.77	16.61	16.43	16.15	17.22	17.78	17.23	17.12
+	2.279	-1.10	0.47	Q9D0T1	NHP2-like protein 1;NHP2-like protein 1, N-terminally processed	Nhp2l1	4887	11	11	11	78.1	78.1	78.1	14.173	0.0000	118.46	130870000	312	18.13	19.16	18.70	18.58	19.48	19.85	19.53	20.11
+	2.251	-1.11	0.46	Q8BYZ1	ABI gene family member 3	Abi3	3308	15	15	15	47.7	47.7	47.7	39.106	0.0000	189.5	28300000	245	16.41	16.65	15.77	16.65	17.13	17.65	17.30	17.83
+	1.322	-1.11	0.46	Q9R1Q7	Proteolipid protein 2	Plp2	5658	2	2	2	21.1	21.1	21.1	16.607	0.0000	34.638	71457000	118	17.95	19.19	19.16	17.50	19.49	19.42	19.92	19.40
+	1.220	-1.12	0.46	O35892;Q99	Nuclear autoantigen Sp-100	Sp100	410	18	18	18	41.7	41.7	41.7	54.726	0.0000	125.74	33833000	183	16.13	15.91	14.78	15.54	16.19	17.64	15.99	17.04
+	1.926	-1.12	0.46	Q9D8P4	39S ribosomal protein L17, mitochondrial	Mrpl17	5068	8	8	8	45.5	45.5	45.5	20.249	0.0000	13.162	15896000	127	15.55	15.36	15.05	15.54	15.81	16.82	16.24	17.11
+	1.247	-1.12	0.46	Q8R1F0	Leydig cell tumor 10 kDa protein homolog	D8Ertd738e	3785	5	5	5	30.9	30.9	30.9	10.197	0.0004	4.7779	5324000	48	13.32	13.36	13.25	14.08	13.32	15.01	15.02	15.15
+	1.233	-1.13	0.46	Q99PL5	Ribosome-binding protein 1	Rrbp1	4507	95	95	95	64.6	64.6	64.6	172.88	0.0000	323.31	317360000	2201	16.93	17.10	16.91	17.30	17.67	19.57	17.51	18.00
	0.852	-1.13	0.46	Q9CPT5	Nucleolar protein 16	Nop16	4534	15	15	15	69.1	69.1	69.1	21.139	0.0000	94.625	13615000	161	12.46	14.57	12.59	14.49	13.93	14.79	14.44	15.46
	1.101	-1.13	0.46	Q9D0R8	Protein LSM12 homolog	Lsm12	4885	6	6	6	32.8	32.8	32.8	21.701	0.0000	16.33	15610000	123	15.64	16.01	13.69	15.70	16.45	16.23	16.40	16.47
+	2.791	-1.13	0.46	Q8VD12	Zinc finger protein 385A	Znf385a	3939	12	12	12	35.5	35.5	35.5	40.446	0.0000	107.88	14107000	181	14.72	14.76	15.39	15.13	15.82	16.13	16.08	16.47
	0.989	-1.13	0.46	Q9D6J6	NADH dehydrogenase [ubiquinone] flavoprotein 2, mitochondrial	Ndufv2	4997	4	4	4	25.4	25.4	25.4	27.285	0.0000	27.903	2858600	39	12.76	14.23	12.52	14.35	14.70	15.02	13.61	15.06
	1.100	-1.14	0.45	Q8BHG9	CGG triplet repeat-binding protein 1	Cggbp1	3038	7	7	7	37.1	37.1	37.1	18.761	0.0000	19.899	5715400	47	13.78	13.55	14.48	12.55	14.45	15.68	13.95	14.84
+	2.946	-1.14	0.45	P62317	Small nuclear ribonucleoprotein Sm D2	Snrpd2	1423	14	14	14	70.3	70.3	70.3	13.527	0.0000	186	185460000	438	18.09	18.82	18.47	18.65	19.47	19.68	19.47	19.98
+	1.535	-1.14	0.45	Q5SXY1	Cytospin-B	Specc1	2160	28	28	28	38.2	38.2	38.2	118.1	0.0000	183.85	32214000	262	13.93	15.36	15.19	15.44	15.82	16.33	15.79	16.55

+	1.608	-1.14	0.45	Q3UHX2	28 kDa heat- and acid-stable phosphoprotein	Pdap1	1977	11	11	11	41.4	41.4	41.4	20.605	0.0000	42.69	8709200	87	12.88	14.18	13.29	14.14	14.72	14.21	15.20	14.92
+	3.247	-1.15	0.45	P84099	60S ribosomal protein L19	Rpl19	1610	19	19	19	56.1	56.1	56.1	23.466	0.0000	155.11	318280000	752	20.13	19.80	20.03	20.04	20.95	21.60	20.90	21.13
	0.970	-1.15	0.45	Q9CXK9	RNA-binding protein 33	Rbm33	4760	5	5	5	6.6	6.6	6.6	137.32	0.0000	22.904	2412800	32	13.79	14.87	12.40	12.96	14.29	15.53	14.36	14.47
+	2.392	-1.15	0.45	Q3TWL2	Type 1 phosphatidylinositol 4,5-bisphosphate 4-phosphatase	Tmem55b	1882	5	4	4	21.5	18	18	30.047	0.0000	31.298	8913000	89	14.13	15.08	15.16	14.52	16.08	15.69	15.83	15.90
+	1.804	-1.16	0.45	Q91YK2	Ribosomal RNA processing protein 1 homolog B	Rrp1b	4164	9	9	9	18.4	18.4	18.4	80.581	0.0000	24.931	8058800	91	13.98	13.32	14.14	14.36	14.33	15.36	15.25	15.50
+	1.392	-1.16	0.45	Q9Z266	SNARE-associated protein Snapin	Snapin	5832	6	6	6	72.8	72.8	72.8	14.904	0.0000	146.41	20095000	96	15.28	17.09	16.76	15.79	17.03	17.37	17.40	17.75
+	1.226	-1.16	0.45	Q8CH02	SURP and G-patch domain-containing protein 1	Sugp1	3516	8	8	8	17.9	17.9	17.9	72.648	0.0000	26.703	2198500	28	13.80	12.81	11.87	11.73	13.37	13.95	13.61	13.93
+	2.724	-1.16	0.45	Q9ESP1	Stromal cell-derived factor 2-like protein 1	Sdf2l1	5341	4	4	4	23.5	23.5	23.5	23.648	0.0000	31.908	24058000	144	15.68	16.60	16.27	16.27	17.25	17.63	17.45	17.14
+	1.936	-1.16	0.45	Q9D1H7	Golgi to ER traffic protein 4 homolog	Get4	4915	5	5	5	16.8	16.8	16.8	36.525	0.0000	42.367	5339200	50	13.79	14.16	15.20	13.93	15.50	15.29	15.57	15.37
+	2.249	-1.16	0.45	Q3UMC0;Q9	Spermatogenesis-associated protein 5	Spata5	2000	13	13	13	21.1	21.1	21.1	97.255	0.0000	56.74	31962000	116	16.47	17.42	16.69	16.79	17.95	18.52	17.93	17.62
+	1.471	-1.17	0.45	Q9D902	General transcription factor IIE subunit 2	Gtf2e2	5087	11	11	11	47.3	47.3	47.3	33.046	0.0000	34.515	11253000	131	13.48	14.88	13.59	14.84	15.18	15.52	14.95	15.80
+	2.572	-1.17	0.45	P16254	Signal recognition particle 14 kDa protein;Signal recognition particle 14	Srp14	807	9	9	9	55.5	55.5	55.5	12.51	0.0000	72.679	42055000	158	16.95	16.87	16.19	17.08	17.68	18.11	17.75	18.21
+	1.898	-1.17	0.45	Q8K2R5	Zinc finger protein 668	Znf668	3670	4	4	4	9.5	9.5	9.5	68.346	0.0000	12.29	3896600	75	13.53	13.09	13.99	14.05	14.39	15.37	14.44	15.14
+	2.082	-1.17	0.44	Q61164	Transcriptional repressor CTCF	Ctcf	2264	6	6	5	6.5	6.5	6.2	83.745	0.0000	61.987	13511000	134	15.54	15.26	15.23	15.78	16.44	17.22	15.96	16.87
	0.538	-1.17	0.44	Q8VCW4	Protein unc-93 homolog B1	Unc93b1	3933	5	5	5	8.9	8.9	8.9	66.98	0.0000	23.179	18756000	128	16.10	16.37	16.17	12.25	16.60	16.08	16.15	16.73
+	1.795	-1.17	0.44	Q99LX0	Protein deglycase DJ-1	Park7	4448	19	19	19	95.2	95.2	95.2	20.021	0.0000	306.51	181220000	865	16.96	18.15	17.16	17.87	18.31	18.66	18.55	19.30
+	1.688	-1.17	0.44	Q3UL36	Arginine and glutamate-rich protein 1	Arglu1	1990	15	15	15	41.3	41.3	41.3	32.887	0.0000	35.603	30396000	243	15.98	15.65	15.13	15.40	16.57	17.58	15.98	16.72
+	2.504	-1.18	0.44	Q8VDD8	WAS protein family homolog 1	Wash1	3949	12	12	12	38.1	38.1	38.1	51.658	0.0000	98.808	19636000	186	15.17	15.06	15.73	15.84	16.25	16.93	16.53	16.81
+	1.036	-1.20	0.44	Q9Z2D8	Methyl-CpG-binding domain protein 3	Mbd3	5845	11	11	10	36.8	36.8	32.6	32.168	0.0000	45.753	18917000	220	13.72	15.08	13.05	14.98	14.96	15.46	14.88	16.33
+	1.408	-1.20	0.44	Q9QYS9	Protein quaking	Qki	5580	17	17	16	52.2	52.2	50.1	37.67	0.0000	148.95	142800000	473	16.77	18.49	17.19	18.04	18.64	18.91	18.31	19.42
+	1.106	-1.21	0.43	Q8BV49;Q5I	Pyrin and HIN domain-containing protein 1;Pyrin and HIN domain-conta	Pyhin1	3235	20	18	18	49	44	44	46.886	0.0000	172.47	51999000	321	16.58	16.30	15.26	14.78	17.05	17.92	16.15	16.62
	0.962	-1.21	0.43	P56213	FAD-linked sulfhydryl oxidase ALR	Gfer	1278	3	3	2	16.2	16.2	10.6	22.876	0.0000	8.1701	2900300	38	13.24	12.51	13.16	12.52	14.40	14.62	12.27	14.97
+	2.193	-1.21	0.43	Q9Z277	Tyrosine-protein kinase BAZ1B	Baz1b	5833	28	28	28	22	22	22	170.65	0.0000	95.075	34719000	273	15.81	15.88	15.18	15.65	16.50	17.45	16.37	17.03
+	3.102	-1.21	0.43	Q8R0G7	Protein spinster homolog 1	Spns1	3758	5	5	5	21.2	21.2	21.2	56.708	0.0000	135.47	9315000	91	13.91	14.58	14.56	14.24	15.85	15.39	15.36	15.52
	0.459	-1.21	0.43	Q7TSF1;Q6	Desmoglein-1-beta;Desmoglein-1-alpha	Dsg1b;Dsg1a	2306	5	5	5	3.4	3.4	3.4	114.45	0.0000	9.9617	14194000	32	14.03	14.46	14.08	14.50	15.68	16.39	17.68	12.16
+	2.619	-1.21	0.43	P62267	40S ribosomal protein S23	Rps23	1412	16	16	16	70.6	70.6	70.6	15.807	0.0000	121.65	201880000	440	19.13	19.33	18.73	18.91	20.10	20.46	19.73	20.64
+	1.548	-1.21	0.43	Q61216	Double-strand break repair protein MRE11A	Mre11a	2281	12	12	12	24.5	24.5	24.5	80.222	0.0000	46.333	10285000	93	13.27	14.05	14.30	14.69	14.44	15.57	15.35	15.79
+	2.207	-1.21	0.43	Q9CYI4	Putative RNA-binding protein Luc7-like 1	Luc7l	4800	19	13	12	43.1	36.4	34.2	43.934	0.0000	34.34	28420000	140	16.13	16.85	16.70	16.17	17.65	18.21	17.09	17.75
+	2.184	-1.22	0.43	Q80W00	Serine/threonine-protein phosphatase 1 regulatory subunit 10	Ppp1r10	2856	14	14	14	20.5	20.5	20.5	94.371	0.0000	54.971	20007000	228	14.40	14.95	14.95	15.23	15.63	16.53	15.73	16.50
+	1.066	-1.22	0.43	Q8BG15	CTD small phosphatase-like protein 2	Ctdspl2	2958	3	3	3	6.9	6.9	6.9	52.812	0.0017	3.2981	2167600	17	13.04	13.74	12.78	13.67	15.08	15.24	12.91	14.87
	0.559	-1.22	0.43	O35682	Myeloid-associated differentiation marker	Myadm	397	3	3	3	10.9	10.9	10.9	35.284	0.0000	82.263	52616000	141	18.11	18.26	18.76	14.36	18.32	18.67	18.78	18.59
+	5.035	-1.23	0.43	Q9D1B9	39S ribosomal protein L28, mitochondrial	Mrpl28	4904	13	13	13	51.4	51.4	51.4	30.169	0.0000	74.389	42045000	328	15.70	15.75	15.52	15.70	16.75	16.86	16.87	17.10
+	0.965	-1.23	0.43	P35601	Replication factor C subunit 1	Rfc1	1028	9	9	9	12.4	12.4	12.4	125.98	0.0000	27.05	4598800	55	12.85	13.50	13.36	13.36	13.74	16.39	13.76	14.10
+	2.347	-1.23	0.43	P16045	Galectin-1	Lgals1	804	11	11	11	76.3	76.3	76.3	14.866	0.0000	225.94	377560000	780	18.65	19.84	19.13	19.42	20.30	20.61	20.26	20.77
+	1.292	-1.23	0.43	O54918	Bcl-2-like protein 11	Bcl2l11	433	4	4	4	20.4	20.4	20.4	22.066	0.0000	7.0722	6471200	60	12.95	15.13	14.60	13.84	15.49	14.93	15.25	15.75
+	3.434	-1.23	0.43	P97364	Selenide, water dikinase 2	Sephs2	1632	8	8	8	37.6	37.6	37.6	47.833	0.0000	197.58	18888000	102	15.99	16.21	16.67	16.02	17.28	17.41	17.55	17.58
+	2.070	-1.23	0.43	Q8CHY6	Transcriptional repressor p66 alpha	Gatad2a	3534	18	18	18	42.6	42.6	42.6	67.333	0.0000	195.05	30735000	377	14.47	15.23	14.70	15.29	15.54	16.54	15.96	16.59
+	2.747	-1.23	0.43	Q9CPU0	Lactoylglutathione lyase	Glo1	4535	11	11	11	64.1	64.1	64.1	20.809	0.0000	76.317	15534000	147	14.85	15.47	14.67	14.86	16.52	16.36	15.84	16.07
+	1.674	-1.23	0.43	P12787	Cytochrome c oxidase subunit 5A, mitochondrial	Cox5a	758	7	7	7	59.6	59.6	59.6	16.101	0.0000	77.355	21985000	102	16.13	16.84	15.54	15.53	16.93	17.92	16.81	17.30
	0.915	-1.23	0.42	Q9CR80	Protein FAM32A	Fam32a	4680	8	8	8	57.1	57.1	57.1	13.215	0.0000	16.478	4103100	31	12.54	12.52	13.02	13.79	13.05	14.64	13.37	15.74
+	1.616	-1.24	0.42	Q56A08	G patch domain and KOW motifs-containing protein	Gpkow	2088	15	15	15	37.1	37.1	37.1	53.831	0.0000	35.408	17841000	163	14.29	14.90	14.26	15.41	15.22	16.29	15.70	16.63
+	1.145	-1.25	0.42	P70279	Surfeit locus protein 6	Surf6	1544	20	20	20	40.3	40.3	40.3	41.234	0.0000	46.758	21854000	151	14.55	15.28	13.51	15.52	15.57	16.71	15.20	16.38
+	1.364	-1.25	0.42	Q9D8E6	60S ribosomal protein L4	Rpl4	5060	44	44	44	64.4	64.4	64.4	47.153	0.0000	323.31	756480000	1728	18.99	19.29	18.07	18.61	19.41	20.99	19.23	20.34
	0.864	-1.25	0.42	P61961	Ubiquitin-fold modifier 1	Ufm1	1393	3	3	3	63.5	63.5	63.5	9.1175	0.0000	153.92	16596000	86	14.02	16.52	16.75	16.88	16.63	17.46	17.20	17.89
+	3.043	-1.25	0.42	O08583	THO complex subunit 4	Alyref	276	11	11	6	52.5	52.5	35.7	26.94	0.0000	323.31	64252000	294	16.72	17.18	16.94	16.95	18.00	18.59	17.79	18.42
	0.715	-1.25	0.42	P62309	Small nuclear ribonucleoprotein G	Snrpg	1419	5	5	5	77.6	77.6	77.6	8.496	0.0000	36.327	14939000	82	16.36	16.60	15.24	12.93	16.76	16.28	16.87	16.23
+	1.318	-1.25	0.42	Q9D1N9	39S ribosomal protein L21, mitochondrial	Mrpl21	4928	6	6	6	40.2	40.2	40.2	23.366	0.0000	19.982	7254300	74	12.84	14.52	15.25	14.23	15.44	15.42	15.42	15.59
	0.527	-1.25	0.42	Q62241	U1 small nuclear ribonucleoprotein C	Snrpc	2371	2	2	2	13.2	13.2	13.2	17.364	0.0000	41.687	7192500	44	13.00	14.27	11.68	14.54	15.51	12.23	16.29	14.46
+	3.240	-1.26	0.42	Q6P8I4	PEST proteolytic signal-containing nuclear protein	Pcnp	2583	7	7	7	45.5	45.5	45.5	18.963	0.0000	21.946	9546900	90	14.55	14.38	14.39	15.13	15.85	16.04	15.69	15.89
+	1.906	-1.26	0.42	P26187	Methylated-DNA--protein-cysteine methyltransferase	Mgmt	919	5	5	5	30.3	30.3	30.3	22.434	0.0000	10.916	3064900	53	13.12	12.39	14.00	13.30	14.09	14.66	14.46	14.63
	0.560	-1.26	0.42	Q8C3P7	N6-adenosine-methyltransferase subunit METTL3	Mettl3	3377	3	3	3	7.1	7.1	7.1	64.615	0.0000	8.0361	4409500	40	13.16	14.92	12.25	15.68	13.19	16.03	15.85	15.97
+	2.387	-1.26	0.42	Q9CQR2	40S ribosomal protein S21	Rps21	4624	8	8	8	77.1	77.1	77.1	9.1413	0.0000	115.07	51713000	255	17.04	17.62	17.28	16.90	18.30	19.02	17.93	18.63
+	2.239	-1.27	0.42	Q9D892	Inosine triphosphate pyrophosphatase	Itpa	5055	10	10	10	59.6	59.6	59.6	21.897	0.0000	95.851	99766000	110	18.07	19.16	18.86	19.43	19.97	20.15	20.28	20.19
+	1.359	-1.27	0.41	Q9CPR4	60S ribosomal protein L17	Rpl17	4529	20	20	20	66.8	66.8	66.8	21.423	0.0000	323.31	412650000	1012	18.51	19.46	17.46	18.98	19.43	19.98	19.52	20.55
+	2.683	-1.27	0.41	Q9DB34	Charged multivesicular body protein 2a	Chmp2a	5124	18	18	18	50.5	50.5	50.5	25.134	0.0000	138.54	93218000	472	16.46	16.93	16.50	17.01	17.80	18.17	17.56	18.46
+	2.733	-1.28	0.41	Q9D0E1;Q9	Heterogeneous nuclear ribonucleoprotein M	Hnrnpm	4861	69	69	69	78.9	78.9	78.9	77.648	0.0000	323.31	1004600000	2248	18.54	19.11	19.24	19.29	19.94	20.56	20.14	20.65
	0.847	-1.28	0.41	Q9WU81	Sugar phosphate exchanger 2	Slc37a2	5700	3	3	3	9.2	9.2	9.2	55.072	0.0000	13.323	10893000	78	13.07	16.17	16.01	15.83	16.87	16.82	16.19	16.30
+	2.450	-1.29	0.41	P62830	60S ribosomal protein L23	Rpl23	1449	16	16	16	77.1	77.1	77.1	14.865	0.0000	157.63	818850000	1007	20.50	20.47	21.10	21.03	21.40	22.34	22.20	22.30
+	1.092	-1.29	0.41	P47963	60S ribosomal protein L13	Rpl13	1135	31	31	31	68.7	68.7	68.7	24.305	0.0000	323.31	1839000000	1825	21.04	21.28	19.26	20.74	21.32	22.73	21.01	22.43
+	1.585	-1.30	0.41	Q5U5M8	Biogenesis of lysosome-related organelles complex 1 subunit 3	Bloc1s3	2167	2	2	2	15.9	15.9	15.9	20.398	0.0000	15.899	1222500	20	12.17	13.26	12.70	12.91	13.36	13.65	14.13	15.08
+	2.993	-1.30	0.41	P62983;P62	Ubiquitin-40S ribosomal protein S27a;Ubiquitin;40S ribosomal protein S	Rps27a;RPS27A;	1474	20	20	7	82.1	82.1	35.9	17.951	0.0000	323.31	1022100000	1528	20.68	21.33	20.55	20.91	21.93	22.53	21.99	22.22
+	1.119	-1.30	0.41	P09602	Non-histone chromosomal protein HMG-17	Hmgn2	700	5	5	5	60	60	60	9.4226	0.0000	25.991	1325500	20	11.23	12.28	12.69	10.89	11.89	13.78	12.95	13.66
+	2.547	-1.30	0.41	Q9D883	Splicing factor U2AF 35 kDa subunit	U2af1	5054	14	14	10	56.9	56.9	43.9	27.815	0.0000	223.73	96165000	492	17.02	17.53	16.81	17.25	18.11	18.79	18.04	18.88
+	3.334	-1.30	0.41	O70274	Protein tyrosine phosphatase type IVA 2	Ptp4a2	481	7	7	5	38.3	38.3	34.1	19.127	0.0000	37.035	31137000	209	16.30	16.48	16.24	16.44	17.55	17.95	17.21	17.96
+	1.053	-1.31	0.40	P62892	60S ribosomal protein L39	Rpl39	1464	3	3	3	33.3	33.3	33.3	6.4066	0.0013	3.5337	10607000	49	12.94	15.30	14.39	15.33	15.16	16.51	15.39	16.12
+	2.190	-1.31	0.40	Q8BJF9	Charged multivesicular body protein 2b	Chmp2b	3078	13	12	12	42.3	42.3	42.3	23.934	0.0000	40.87	38045000	185	15.61	16.57	15.66	15.88	16.87	17.10	17.07	17.91
+	1.848	-1.31	0.40	Q9Z103	Activity-dependent neuroprotector homeobox protein	Adnp	5793	8	8	8	9.3	9.3	9.3	124.31	0.0000	9.0247	6397200	49	13.35	13.50	14.05	14.18	14.92	15.91	14.34	15.15
+	0.896	-1.31	0.40	Q80ZE4	Membrane progestin receptor alpha	Paqr7	2912	1	1	1	6.1	6.1	6.1	39.305	0.0000	7.569	540080	6	12.13	11.42	12.82	11.34	12.50	14.59	11.80	14.07
+	1.943	-1.31	0.40	Q64433	10 kDa heat shock protein, mitochondrial	Hspe1	2441	15	15	15	99	99	99	10.963	0.0000	56.879	56801000	331	16.52	17.37	16.20	17.12	17.95	18.25	17.54	18.72
+	2.016	-1.32	0.40	O55102	Biogenesis of lysosome-related organelles complex 1 subunit 1	Bloc1s1	453	6	6	6	74.4	74.4	74.4	14.281	0.0000	54.508	5956500	75	14.96	14.68	13.73	13.64	15.57	15.70	15.24	15.80
+	3.552	-1.32	0.40	Q8C9B9	Death-inducer obliterator 1	Dido1	3424	10	10	9	6.9	6.9	6.2	247.17	0.0000	96.57	14676000	85	15.43	15.51	15.35	15.52	16.37	17.18	16.67	16.87
+	0.915	-1.33	0.40	Q8C0G2	TRAF3-interacting JNK-activating modulator	Traf3ip3	3336	8	8	8	20.3	20.3	20.3	58.563	0.0000	14.646	9567400	29	12.59	15.84	14.64	15.50	16.21	15.89	15.80	15.98
+	1.710	-1.33	0.40	Q99JF8	PC4 and SFRS1-interacting protein	Psip1	4338	15	15	14	30.1	30.1	28.6	59.696	0.0000	123.7	65474000	144	17.18	17.53	17.51	18.65	18.77	19.80	18.58	19.01
+	1.802	-1.33	0.40	Q9CQX8	28S ribosomal protein S36, mitochondrial	Mrps36	4646	5	5	5	70.6	70.6	70.6	11.101	0.0000	216.52	22715000	138	14.76	15.99	15.48	16.48	16.58	17.04	17.12	17.27
+	1.563	-1.33	0.40	Q922Y1	UBX domain-containing protein 1	Ubxn1	4280	12	12	11	42.8	42.8	42.1	33.572	0.0000	186.62	25527000	167	15.81	16.64	14.96	16.42	17.15	17.40	16.67	17.93
+	2.235	-1.33	0.40	P20108	Thioredoxin-dependent peroxide reductase, mitochondrial	Prdx3	854	14	14	14	47.1	47.1	47.1	28.127	0.0000	97.919	57972000	257	16.56	17.59	16.82	16.52	18.57	17.72	18.04	18.49
+	1.619	-1.33	0.40	P68871	Hemoglobin subunit beta;LVV-hemorphin-7;Spinorphin	HBB	1523	6	4	4	49.7	36.7	36.7	15.998	0.0000	15.978	3447700	35	12.72	12.53	13.46	14.41	14.53	14.64	14.34	14.95
+	1.556	-1.34	0.40	P97478	5-demethoxyubiquinone hydroxylase, mitochondrial	Coq7	1659	7	7	7	33.2	33.2	33.2	24.041	0.0000	36.956	17578000	97	14.47	15.30	15.85	16.39	16.48	17.18	16.45	17.23
+	2.539	-1.34	0.40	Q9QUR7	Peptidyl-prolyl cis-trans isomerase NIMA-interacting 1	Pin1	5523	8	8	8	55.8	55.8	55.8	18.37	0.0000	83.958	14048000	171	13.46	14.53	13.89	14.38	15.26	15.56	15.11	15.68
+	0.936	-1.34	0.40	Q921N8		Gps2	4236	4	4	4	12.8	12.8	12.8	36.737	0.0025	2.9007	8809200	53	12.69	13.26	13.34	15.48	15.39	13.85	15.43	15.43
+	1.546	-1.34	0.40	Q8R1I1	Cytochrome b-c1 complex subunit 9	Uqcr10	3789	3	3	3	42.2	42.2	42.2	7.4454	0.0000	140.15	5538300	88	14.31	13.55	14.53	14.51	16.04	15.56	14.42	16.23
+	0.858	-1.34	0.40	Q8C522	Endonuclease domain-containing 1 protein	Endod1	3391	6	6	6	18.8	18.8	18.8	55.262	0.0000	180.59	15917000	117	13.92	13.73	16.89	16.02	16.51	16.61	16.32	16.48
+	2.652	-1.34	0.39	P67984	60S ribosomal protein L22	Rpl22	1510	9	9	8	68.8	68.8	62.5	14.759	0.0000	323.31	356070000	506	18.89	19.64	18.98	19.54	20.27	20.81	20.33	21.02
+	1.612	-1.35	0.39	Q9CQ75	NADH dehydrogenase [ubiquinone] 1 alpha subcomplex subunit 2	Ndufa2	4570	5	5	5	39.4	39.4	39.4	10.916	0.0000	31.991	43827000	92	15.78	17.49	16.96	17.02	17.76	18.95	17.92	18.00
+	1.621	-1.35	0.39	P62751	60S ribosomal protein L23a	Rpl23a	1442	18	18	18	60.9	60.9	60.9	17.695	0.0000	131.61	600120000	749	19.59	20.87	19.73	20.86	20.96	21.98	21.34	22.18
+	1.791	-1.35	0.39	Q8VBT6	Apolipoprotein B receptor	Apobr	3895	39	39	39	51.9	51.9	51.9	102.7	0.0000	323.31	94119000	764	15.33	16.37	15.30	15.86	16.56	17.64	16.47	17.59
+	1.614	-1.36	0.39	Q6NZB0	DnaJ homolog subfamily C member 8	Dnajc8	2542	19	19	18	75.5	75.5	73.5	29.812	0.0000	175.9	23278000	177	14.49	15.38	13.65	14.95	15.53	16.15	15.59	16.64
+	2.139	-1.37	0.39	Q6NZN0	RNA-binding protein 26	Rbm26	2548	12	12	12	14.7	14.7	14.7	114.14	0.0000	37.989	6878900	94	13.19	14.11	13.20	14.33	14.72	15.34	14.85	15.40
+	1.497	-1.37	0.39	Q9ERU3	Zinc finger protein 22	Znf22	5319	5	5	5	20.3	20.3	20.3	27.294	0.0000	16.706	8865300	94	13.95	14.79	13.27	15.37	15.37	16.15	15.53	15.81
+	2.364	-1.37	0.39	P09926	Surfeit locus protein 2	Surf2	703	4	4	4	21	21	21	30.355	0.0000	22.456	6448800	43	14.66	13.85	13.65	14.20	14.89	15.87	15.68	15.40
+	1.088	-1.37	0.39	Q8BHE8	Uncharacterized protein C2orf47 homolog, mitochondrial		3034	4	4	4	16.5	16.5	16.5	32.985	0.0000	10.749	5493600	28	12.35	14.23	12.41	13.91	14.76	13.43	15.54	14.66
+	1.210	-1.38	0.38	P43275	Histone H1.1	Hist1h1a	1088	15	10	10	43.7	38.5	38.5	21.785	0.0000	323.31	430210000	493	19.73	20.16	19.22	19.77	20.70	22.71	20.04	20.96
+	1.005	-1.38	0.38	P61967	AP-1 complex subunit sigma-1A	Ap1s1	1396	4	4	4	27.8	27.8	27.8	18.733	0.0000	79.655	10473000	98	13.37	14.47	16.66	15.51	16.33	16.58	16.19	16.45
+	1.108	-1.39	0.38	Q8R1N0	Zinc finger protein 830	Znf830	3792	3	3	3	16.3	16.3	16.3	40.658	0.0000	41.426	5862700	82	12.40	14.79	14.51	15.08	15.24	15.48	15.33	16.29
+	2.044	-1.39	0.38	Q8BG81	Polymerase delta-interacting protein 3	Poldip3	2966	16	16	16	47.4	47.4	47.4	46.132	0.0000	70.436	36759000	310	15.48	16.62	15.59	16.40	16.94	17.89	17.12	17.72
+	2.556	-1.39	0.38	Q8R035	Peptidyl-tRNA hydrolase ICT1, mitochondrial	Ict1	3745	9	9	9	55.3	55.3	55.3	23.477	0.0000	46.432	18715000	157	14.37	15.22	15.38	15.28	16.07	16.59	16.33	16.84
+	1.832	-1.39	0.38	P62911;P17	60S ribosomal protein L32	Rpl32	1468	14	14	14	61.5	61.5	61.5	15.86	0.0000	83.477	192540000	542	18.48	18.38	17.39	17.80	19.15	20.13	18.65	19.70
+	2.150	-1.40	0.38	E9Q394		Akap13	232	13	13	13	9.5	9.5	9.5	303.97	0.0000	53.712	8261300	59	14.32	14.01	13.38	13.65	14.52	15.87	15.43	15.14
+	0.984	-1.40	0.38	Q8K285	F-BAR domain only protein 1	Fcho1	3649	2	2	2	3	3	3	95.121	0.0013	3.4143	193600	2	11.22	10.50	13.54	11.54	12.53	12.68	13.16	14.03
+	2.301	-1.40	0.38	P12804	Fibroleukin	Fgl2	759	2	2	2	5.6	5.6	5.6	48.95	0.0009	3.7139	891970	5	11.78	11.28	12.56	12.03	13.52	13.37	12.77	13.60
+	1.312	-1.41	0.38	Q9CZ83	39S ribosomal protein L55, mitochondrial	Mrpl55	4821	8	8	8	43.3	43.3	43.3	15.121	0.0000	23.497	12714000	78	13.65	15.76	14.96	15.37	16.19	15.98	15.85	17.36
+	0.942	-1.42	0.37	Q5ND52	rRNA methyltransferase 3, mitochondrial	Rnmtl1	2122	3	3	3	11.7	11.7	11.7	46.795	0.0002	5.4264	2894100	20	13.94	13.40	13.18	11.86	15.13	15.68	14.49	12.76
+	2.176	-1.42	0.37	P47911	60S ribosomal protein L6	Rpl6	1129	28	28	28	57.4	57.4	57.4	33.509	0.0000	323.31	354020000	898	18.60	18.49	18.14	18.65	19.12	20.52	19.56	20.37
+	1.285	-1.42	0.37	Q3UFM5	Nucleolar MIF4G domain-containing protein 1	Nom1	1956	8	8	8	10.9	10.9	10.9	95.959	0.0000	16.857	40228000	71	16.67	18.04	16.75	17.28	17.85	19.57	17.66	19.36
+	2.976	-1.42	0.37	Q9DCW4	Electron transfer flavoprotein subunit beta	Etfb	5235	27	27	27	84.3	84.3	84.3	27.623	0.0000	181.49	251270000	802	17.63	18.77	18.22	18.29	19.57	19.73	19.53	19.79
+	2.092	-1.42	0.37	Q6NZF1	Zinc finger CCCH domain-containing protein 11A	Zc3h11a	2545	13	13	13	22.3	22.3	22.3	86.491	0.0000	25.911	10802000	127	13.42	14.50	13.16	14.06	14.89	15.60	14.83	15.51
+	1.908	-1.43	0.37	Q9D8B3;Q9	Charged multivesicular body protein 4b	Chmp4b	5057	14	14	14	52.7	52.7	52.7	24.936	0.0000	236.84	32781000	155	15.07	16.25	14.72	14.93	16.81	16.96	16.04	16.86
+	1.703	-1.43	0.37	P62843	40S ribosomal protein S15	Rps15	1452	11	11	11	77.2	77.2	77.2	17.04	0.0000	323.31	669510000	1029	20.18	20.39	19.12	20.52	20.79	21.78	21.12	22.22
+	2.503	-1.43	0.37	Q8CH25	SAFB-like transcription modulator	Sltm	3518	15	15	15	15.3	15.3	15.3	116.92	0.0000	42.657	21399000	178	15.04	15.77	15.03	15.59	16.34	17.28	16.44	17.08
+	1.639	-1.44	0.37	Q9CQF8	Ribosomal protein 63, mitochondrial	Mrpl57	4596	5	5	5	31.4	31.4	31.4	11.952	0.0000	12.393	15959000	134	13.40	15.36	14.48	15.15	15.77	15.99	15.85	16.52
+	1.640	-1.44	0.37	P62918	60S ribosomal protein L8	Rpl8	1470	24	24	24	58.8	58.8	58.8	28.024	0.0000	323.31	879460000	1103	20.34	20.45	19.10	20.39	20.90	21.98	20.93	22.21
+	2.310	-1.44	0.37	Q8K4L0	ATP-dependent RNA helicase DDX54	Ddx54	3720	11	11	11	22.9	22.9	22.9	97.747	0.0000	52.926	10633000	141	13.61	14.58	14.23	14.32	15.26	16.24	15.13	15.88
+	1.233	-1.45	0.37	P03921	NADH-ubiquinone oxidoreductase chain 5	Mtnd5	631	2	2	2	5.4	5.4	5.4	68.474	0.0002	5.8973	773950	8	12.82	11.94	11.48	10.61	14.34	12.45	13.09	12.76
+	2.005	-1.45	0.37	Q9Z108	Double-stranded RNA-binding protein Staufen homolog 1	Stau1	5794	14	14	14	32	32	32	53.924	0.0000	68.86	22505000	172	14.41	15.84	14.97	15.58	16.16	16.85	16.41	17.16

+	1.962	-1.45	0.37	Q9CWT6	Probable ATP-dependent RNA helicase DDX28	Ddx28	4730	10	10	9	25	25	23.5	59.514	0.0000	36.596	12057000	103	15.06	15.42	15.40	14.70	16.50	17.54	15.79	16.54
+	0.846	-1.45	0.37	Q8BGA5	KRR1 small subunit processome component homolog	Krr1	2970	8	8	8	30.3	30.3	30.3	43.537	0.0000	12.058	7852700	37	12.75	13.61	14.37	14.47	14.00	16.74	13.88	16.40
+	3.145	-1.45	0.36	Q99N89	39S ribosomal protein L43, mitochondrial	Mrpl43	4488	7	7	7	42.6	42.6	42.6	20.203	0.0000	25.236	17770000	106	14.65	15.38	15.54	15.50	16.46	16.86	16.70	16.88
+	2.836	-1.46	0.36	Q9D1I6	39S ribosomal protein L14, mitochondrial	Mrpl14	4919	7	7	7	54.5	54.5	54.5	15.874	0.0000	41.935	26512000	187	15.22	16.31	15.83	16.23	17.20	17.41	17.25	17.56
+	2.065	-1.46	0.36	Q8R3Y5	Uncharacterized protein C19orf47 homolog		3859	10	10	10	28.8	28.8	28.8	44.41	0.0000	115.21	29519000	206	15.70	16.66	15.30	16.11	16.86	17.66	17.14	17.95
+	1.032	-1.47	0.36	Q9CWV6	PRKR-interacting protein 1	Prkrip1	4734	6	6	6	32.8	32.8	32.8	21.491	0.0000	10.537	13495000	18	13.87	16.30	13.34	13.77	15.24	15.78	15.50	16.66
+	1.659	-1.47	0.36	P19426	Negative elongation factor E	Nelfe	847	13	13	13	44.3	44.3	44.3	42.554	0.0000	99.677	10421000	71	12.59	13.34	14.03	14.48	14.61	15.66	15.30	14.76
+	1.227	-1.48	0.36	Q9EQN3	TSC22 domain family protein 4	Tsc22d4	5284	5	5	5	17.1	17.1	17.1	39.978	0.0000	14.038	5105200	58	12.30	15.02	13.71	14.02	14.38	15.33	15.53	15.73
+	1.200	-1.48	0.36	Q6ZWV7	60S ribosomal protein L35	Rpl35	2708	16	16	16	61.8	61.8	61.8	14.552	0.0000	323.31	835630000	946	20.23	20.60	18.57	19.77	20.94	22.54	20.26	21.34
+	1.222	-1.48	0.36	Q9D903	Probable rRNA-processing protein EBP2	Ebna1bp2	5088	11	11	11	42.2	42.2	42.2	34.702	0.0000	48.653	7782000	64	12.94	13.82	12.41	13.79	13.56	15.99	14.14	15.20
+	1.573	-1.48	0.36	Q9CR57	60S ribosomal protein L14	Rpl14	4669	20	20	20	64.5	64.5	64.5	23.564	0.0000	66.091	295890000	848	18.78	18.96	18.14	18.89	19.70	21.43	19.25	20.33
+	0.871	-1.49	0.36	Q8CF66	Ragulator complex protein LAMTOR4;Ragulator complex protein LAMT	Lamtor4	3475	2	2	2	17.2	17.2	17.2	10.678	0.0020	3.0928	3666000	34	13.96	14.35	12.81	14.30	16.24	16.03	13.00	16.09
+	1.157	-1.49	0.36	P04040	Catalase	CAT	635	18	6	6	36.6	15.7	15.7	59.755	0.0000	24.227	4222200	41	13.99	14.14	11.88	13.54	14.19	14.28	14.95	16.06
+	2.464	-1.49	0.36	Q8CGC6	RNA-binding protein 28	Rbm28	3506	12	12	12	18.8	18.8	18.8	84.213	0.0000	46.168	14557000	196	14.52	14.74	14.07	14.72	15.45	16.65	15.64	16.29
+	1.437	-1.50	0.35	A2A7S8	Uncharacterized protein KIAA1522	Kiaa1522	11	10	10	10	17.4	17.4	17.4	104.84	0.0000	53.23	6081700	53	12.96	12.07	13.79	13.33	14.02	14.52	15.73	13.86
+	1.350	-1.50	0.35	Q61823	Programmed cell death protein 4	Pdcd4	2336	8	8	8	28.6	28.6	28.6	51.702	0.0000	13.578	4237700	30	11.96	13.11	13.59	13.45	13.70	14.28	14.27	15.84
+	1.761	-1.50	0.35	Q8BP67	60S ribosomal protein L24	Rpl24	3160	16	16	16	65	65	65	17.779	0.0000	147.16	282230000	674	19.50	19.32	18.48	19.06	19.84	21.56	20.02	20.93
+	1.764	-1.50	0.35	P50543	Protein S100-A11	S100a11	1186	5	5	5	51	51	51	11.083	0.0000	72.168	35048000	216	15.81	16.71	14.78	16.20	17.47	17.06	17.05	17.94
+	2.140	-1.51	0.35	Q8VIM9	Immunity-related GTPase family Q protein	Irgq	4035	4	4	4	9.6	9.6	9.6	59.323	0.0015	3.396	1869600	27	13.46	14.24	13.56	12.47	14.72	15.20	14.86	14.98
+	3.029	-1.51	0.35	P63213	Guanine nucleotide-binding protein G(I)/G(S)/G(O) subunit gamma-2	Gng2	1496	11	11	11	84.5	84.5	84.5	7.8501	0.0000	63.74	142420000	385	18.56	19.11	18.47	18.46	19.60	20.35	20.21	20.48
+	2.130	-1.51	0.35	Q91YJ5	Translation initiation factor IF-2, mitochondrial	Mtif2	4163	5	5	5	8.8	8.8	8.8	81.288	0.0000	10.819	4707900	32	13.39	14.26	14.17	12.68	15.10	15.08	14.98	15.39
+	3.079	-1.52	0.35	P62264	40S ribosomal protein S14	Rps14	1411	11	11	11	43	43	43	16.273	0.0000	135.23	541830000	880	19.09	19.72	18.86	19.41	20.41	21.05	20.63	21.06
+	2.113	-1.52	0.35	Q9D8C4	Interferon-induced 35 kDa protein homolog	Ifi35	5059	13	13	13	60.5	60.5	60.5	31.875	0.0000	99.903	35767000	160	15.27	16.48	14.98	16.22	17.29	17.42	16.86	17.46
+	1.431	-1.52	0.35	O54824	Pro-interleukin-16;Interleukin-16	Il16	425	12	12	12	11	11	11	141.43	0.0000	59.488	14793000	149	13.14	14.49	14.02	15.19	15.32	15.27	15.50	16.85
+	1.394	-1.52	0.35	Q8K3W3	Protein CASC3	Casc3	3706	4	4	4	6.3	6.3	6.3	75.769	0.0027	2.7335	312090	4	10.97	10.94	11.06	12.84	12.58	13.68	13.44	12.18
+	3.814	-1.52	0.35	Q9CR88	28S ribosomal protein S14, mitochondrial	Mrps14	4681	7	7	7	35.9	35.9	35.9	14.92	0.0000	190.83	26785000	187	16.30	16.00	16.45	16.10	17.63	18.18	17.52	17.60
+	0.977	-1.53	0.35	Q99KR7	Peptidyl-prolyl cis-trans isomerase F, mitochondrial	Ppif	4392	6	5	5	36.9	33.5	33.5	21.737	0.0000	16.546	6546700	42	12.26	13.31	12.80	15.21	14.86	13.65	15.95	15.22
+	2.509	-1.53	0.35	P99027	60S acidic ribosomal protein P2	Rplp2	1693	7	7	7	79.1	79.1	79.1	11.651	0.0000	160.82	25281000	239	15.30	16.29	15.58	14.96	17.13	17.44	16.75	16.92
+	2.263	-1.53	0.35	O70591	Prefoldin subunit 2	Pfdn2	517	3	3	3	22.7	22.7	22.7	16.534	0.0000	11.828	2877600	38	12.75	14.01	13.06	13.82	14.97	15.35	14.40	15.01
+	1.271	-1.54	0.34	P43274	Histone H1.4	Hist1h1e	1087	27	13	7	57.5	38.4	20.1	21.977	0.0000	147.02	1359600000	852	20.97	21.69	20.31	21.00	22.03	24.12	21.43	22.57
+	2.186	-1.55	0.34	Q9D8X2	Coiled-coil domain-containing protein 124	Ccdc124	5081	13	13	13	60.4	60.4	60.4	25.345	0.0000	47.664	25487000	114	14.63	15.04	13.85	15.54	16.05	16.14	16.58	16.48
+	1.812	-1.55	0.34	E9Q557	Desmoplakin	Dsp	237	123	123	123	37.1	37.1	37.1	332.91	0.0000	323.31	185540000	1220	16.58	16.40	14.81	16.55	17.18	18.01	17.80	17.54
	0.415	-1.55	0.34	Q8CJF9	Protein argonaute-3	Ago3	3570	10	3	3	16.3	5.1	5.1	97.276	0.0000	30.282	6032600	24	12.29	15.82	17.17	10.30	16.02	14.24	16.43	15.08
+	0.943	-1.55	0.34	Q9WUH1	Transmembrane protein 115	Tmem115	5711	3	3	3	11.7	11.7	11.7	38.098	0.0000	15.339	3885000	41	12.50	12.87	16.10	14.49	15.10	15.67	15.73	15.66
+	3.703	-1.56	0.34	P14115	60S ribosomal protein L27a	Rpl27a	773	13	13	13	66.9	66.9	66.9	16.605	0.0000	238.26	587490000	679	19.85	20.10	19.75	19.58	21.09	21.68	21.10	21.62
+	1.639	-1.56	0.34	Q9DCL2	MIP18 family protein FAM96A	Fam96a	5220	4	4	4	30	30	30	18.418	0.0000	7.3362	6466500	64	15.17	15.55	13.92	13.42	16.07	16.11	15.85	16.26
+	1.921	-1.56	0.34	P62242	40S ribosomal protein S8	Rps8	1408	19	19	19	58.7	58.7	58.7	24.205	0.0000	275.68	296760000	814	18.88	19.17	18.09	18.94	19.99	21.33	19.61	20.39
+	2.090	-1.56	0.34	Q80TJ7	Histone lysine demethylase PHF8	Phf8	2806	6	6	5	8.7	8.7	7.3	113.55	0.0000	32.603	8244600	68	14.84	14.63	14.19	13.37	15.40	16.49	15.61	15.76
+	2.045	-1.56	0.34	Q9CX48	Zinc finger CCHC domain-containing protein 10	Zcchc10	4747	3	3	3	14.6	14.6	14.6	19.147	0.0000	26.975	8010000	70	14.55	14.70	14.00	14.13	15.30	16.91	15.36	16.06
+	2.385	-1.56	0.34	Q9CZA6;Q9	Nuclear distribution protein nudE homolog 1;Nuclear distribution protein	Nde1;Ndel1	4823	5	5	5	14.2	14.2	14.2	38.522	0.0000	7.6349	844510	12	11.89	11.68	11.87	11.88	12.64	13.00	14.15	13.77
+	1.468	-1.56	0.34	Q9D945	Protein LLP homolog	Llph	5094	2	2	2	29.2	29.2	29.2	15.391	0.0000	22.087	1526900	19	11.03	11.85	11.04	9.99	11.47	12.49	12.66	13.55
+	2.025	-1.57	0.34	Q9D7A6	Signal recognition particle 19 kDa protein	Srp19	5024	4	4	4	33.3	33.3	33.3	16.191	0.0000	66.932	18853000	103	14.32	14.90	15.28	15.66	16.20	17.11	15.98	17.15
+	3.016	-1.57	0.34	Q8K194	U4/U6.U5 small nuclear ribonucleoprotein 27 kDa protein	Snrnp27	3619	8	8	8	39.4	39.4	39.4	18.885	0.0000	35.748	19824000	114	15.50	15.15	14.93	15.38	16.66	17.35	16.26	16.96
+	2.017	-1.57	0.34	Q9CQJ6	Density-regulated protein	Denr	4606	5	5	5	42.4	42.4	42.4	22.166	0.0000	51.043	7997300	62	13.76	13.33	13.65	15.10	15.48	15.61	15.14	15.89
+	1.320	-1.57	0.34	Q91V81	RNA-binding protein 42	Rbm42	4050	11	11	11	30.5	30.5	30.5	50.235	0.0000	106.91	23497000	141	13.89	15.95	15.91	15.97	16.12	17.30	16.74	17.86
+	2.393	-1.58	0.34	P27661	Histone H2AX	H2afx	939	21	21	8	81.1	81.1	47.6	15.142	0.0000	323.31	4640700000	2863	22.55	23.07	22.72	23.12	24.04	24.80	23.78	25.14
+	1.178	-1.59	0.33	Q9Z2E1	Methyl-CpG-binding domain protein 2	Mbd2	5846	6	5	5	16.9	14	14	43.5	0.0000	15.505	8210700	67	14.31	15.24	14.78	12.16	15.33	16.22	15.47	15.82
+	3.033	-1.60	0.33	Q60596	DNA repair protein XRCC1	Xrcc1	2181	3	3	3	9.8	9.8	9.8	68.97	0.0000	19.489	4247600	25	13.68	14.23	14.43	14.62	15.47	16.18	15.63	16.06
+	1.524	-1.61	0.33	P70296	Phosphatidylethanolamine-binding protein 1;Hippocampal cholinergic ne	Pebp1	1549	9	9	9	85.6	85.6	85.6	20.83	0.0000	116.53	34555000	173	14.61	17.03	15.79	14.99	16.95	17.55	16.82	17.52
+	1.025	-1.61	0.33	Q99L28	Probable ribosome biogenesis protein RLP24	Rsl24d1	4406	6	6	6	33.1	33.1	33.1	19.611	0.0000	8.364	5812000	49	14.30	12.09	13.38	14.31	14.87	16.11	13.49	16.05
+	3.201	-1.62	0.33	Q9CQV1	Mitochondrial import inner membrane translocase subunit TIM16	Pam16	4636	10	10	10	60.8	60.8	60.8	13.784	0.0000	323.31	25825000	147	15.64	15.61	16.04	15.86	16.81	17.67	17.30	17.84
+	2.409	-1.62	0.33	Q8CB77	Transcription elongation factor B polypeptide 3	Tceb3	3433	28	28	28	45.5	45.5	45.5	87.16	0.0000	196.26	48137000	446	15.09	15.33	14.67	15.27	16.20	17.48	16.15	17.02
+	1.586	-1.62	0.32	Q99N91	39S ribosomal protein L34, mitochondrial	Mrpl34	4489	3	3	3	25	25	25	10.531	0.0004	4.6748	8182800	42	14.41	14.19	12.95	15.46	15.72	16.17	15.40	16.22
+	2.203	-1.62	0.32	Q9QUN3	B-cell linker protein	Blnk	5520	8	8	8	20.8	20.8	20.8	50.67	0.0000	49.453	7963400	81	14.89	15.19	13.64	13.84	15.75	16.00	16.07	16.22
+	1.527	-1.63	0.32	P43276	Histone H1.5	Hist1h1b	1089	25	15	14	49.8	31.8	31.4	22.576	0.0000	323.31	1166500000	886	20.28	20.86	20.01	20.47	21.58	23.51	20.94	22.10
+	1.047	-1.63	0.32	Q9CR00	26S proteasome non-ATPase regulatory subunit 9	Psmd9	4653	8	8	7	45.5	45.5	38.7	24.72	0.0000	14.044	5821600	64	11.29	14.70	14.71	13.56	15.17	15.04	15.20	15.36
+	2.621	-1.63	0.32	Q3TC33	Coiled-coil domain-containing protein 127	Ccdc127	1822	4	4	4	23.1	23.1	23.1	30.509	0.0000	7.4652	3051500	31	13.27	12.16	13.29	12.34	14.47	14.48	14.60	14.04
+	2.203	-1.63	0.32	Q02257	Junction plakoglobin	Jup	1718	32	32	29	40.8	40.8	37.4	81.8	0.0000	288.43	118170000	652	16.88	16.80	15.31	16.65	17.63	18.16	18.26	18.11
+	3.689	-1.63	0.32	Q9Z1P6	NADH dehydrogenase [ubiquinone] 1 alpha subcomplex subunit 7	Ndufa7	5816	9	9	9	78.8	78.8	78.8	12.575	0.0000	41.344	71028000	218	16.29	16.91	16.19	16.71	18.18	18.45	17.92	18.07
+	1.463	-1.64	0.32	P10922	Histone H1.0;Histone H1.0, N-terminally processed	H1f0	734	12	12	12	40.7	40.7	40.7	20.861	0.0000	65.023	44981000	323	15.56	16.13	14.97	15.97	16.40	18.82	16.66	17.31
+	1.405	-1.64	0.32	Q9D0I4	Syntaxin-17	Stx17	4867	10	10	9	42.9	42.9	42.9	33.221	0.0000	44.579	9571000	90	15.08	14.86	12.82	14.69	15.47	16.70	15.35	16.49
	0.443	-1.64	0.32	Q9CQJ2	PIH1 domain-containing protein 1	Pih1d1	4604	6	6	5	24.1	24.1	21.7	32.208	0.0000	9.5625	25438000	30	15.59	16.71	13.99	15.46	12.39	18.87	18.50	18.57
+	2.749	-1.65	0.32	Q3V2Q8	NEDD4-binding protein 2-like 1	N4bp2l1	2051	5	5	5	25.2	25.2	25.2	28.038	0.0000	11.997	34171000	54	17.33	17.43	16.67	17.26	18.82	19.47	18.79	18.21
+	1.976	-1.66	0.32	Q8JZY2	COMM domain-containing protein 10	Commd10	3587	6	6	6	35.1	35.1	35.1	22.812	0.0000	13.434	12217000	77	15.35	15.60	15.52	13.81	16.66	17.19	16.57	16.49
+	1.337	-1.66	0.32	Q9D823	60S ribosomal protein L37	Rpl37	5047	6	6	6	52.6	52.6	52.6	11.078	0.0004	4.338	44977000	99	16.55	17.55	14.80	16.74	17.58	18.38	17.50	18.81
+	1.674	-1.66	0.32	Q9CY16	28S ribosomal protein S28, mitochondrial	Mrps28	4777	5	5	5	32.8	32.8	32.8	20.52	0.0000	21.263	5654100	49	13.17	14.97	13.10	14.29	15.14	15.34	15.29	16.38
+	1.374	-1.66	0.32	Q9DCG9	Multifunctional methyltransferase subunit TRM112-like protein	Trmt112	5212	3	3	3	31.2	31.2	31.2	14.141	0.0000	9.6226	8907200	67	13.32	16.33	14.88	14.97	16.22	16.55	16.30	17.05
+	1.565	-1.66	0.32	P18608	Non-histone chromosomal protein HMG-14	Hmgn1	838	6	6	6	67.7	67.7	67.7	10.152	0.0000	10.639	9819800	83	13.94	14.51	12.84	15.39	15.49	16.39	15.57	15.88
+	3.839	-1.66	0.32	Q7TMF3	NADH dehydrogenase [ubiquinone] 1 alpha subcomplex subunit 12	Ndufa12	2743	13	13	13	88.3	88.3	88.3	17.086	0.0000	80.009	64198000	360	16.98	17.09	16.87	16.95	18.48	19.16	18.26	18.62
+	2.205	-1.67	0.31	Q9DBE9	pre-rRNA processing protein FTSJ3	Ftsj3	5147	7	7	7	13.4	13.4	13.4	95.531	0.0000	41.941	7830700	84	13.05	13.41	14.31	14.25	15.26	16.12	15.44	14.89
+	3.052	-1.67	0.31	O35680	28S ribosomal protein S12, mitochondrial	Mrps12	396	3	3	3	20.9	20.9	20.9	15.437	0.0013	3.512	8775500	38	14.32	14.60	13.56	13.95	15.59	15.63	15.65	16.24
+	1.385	-1.67	0.31	Q6ZQ58	La-related protein 1	Larp1	2690	13	13	13	20	20	20	121.12	0.0000	72.931	8170900	76	13.77	13.60	14.04	12.45	15.41	16.45	14.85	13.84
+	2.137	-1.68	0.31	Q3THE2;Q9	Myosin regulatory light chain 12B;Myosin regulatory light polypeptide 9	Myl12b;Myl9	1842	6	6	6	44.2	44.2	44.2	19.779	0.0000	96.621	13688000	75	14.71	15.75	14.54	14.81	16.90	17.35	15.83	16.46
+	1.799	-1.69	0.31	P47915	60S ribosomal protein L29	Rpl29	1130	17	17	17	56.9	56.9	56.9	17.587	0.0000	76.888	258830000	435	18.84	19.39	17.53	18.55	19.85	21.07	19.64	20.53
+	2.743	-1.69	0.31	O54890	Integrin beta-3	Itgb3	431	3	3	3	5.2	5.2	5.2	86.738	0.0000	38.83	2802200	45	12.45	12.07	12.81	13.17	13.98	13.92	14.72	14.65
+	1.451	-1.70	0.31	Q8R1G6	PDZ and LIM domain protein 2	Pdlim2	3788	7	7	7	27.8	27.8	27.8	37.703	0.0000	94.347	13806000	142	13.01	14.74	15.39	15.59	16.60	16.81	15.74	16.39
+	1.033	-1.70	0.31	Q9CY57	Chromatin target of PRMT1 protein	Chtop	4786	8	8	8	34.1	34.1	34.1	26.585	0.0000	323.31	232390000	507	18.67	19.33	16.51	18.14	19.51	21.20	18.39	20.36
+	2.160	-1.73	0.30	Q91VE6	MKI67 FHA domain-interacting nucleolar phosphoprotein	Nifk	4059	8	8	8	39.4	39.4	39.4	36.265	0.0000	50.679	21264000	141	15.03	15.30	14.69	14.13	16.12	17.26	15.76	16.91
+	0.858	-1.74	0.30	Q8K3Z9	Nuclear envelope pore membrane protein POM 121	Pom121	3708	7	7	7	12.3	12.3	12.3	121.02	0.0000	48.023	4702900	80	10.81	14.69	13.23	15.17	14.45	15.72	15.27	15.42
+	1.428	-1.74	0.30	Q8C6B9	Active regulator of SIRT1	Rps19bp1	3404	5	5	5	38.5	38.5	38.5	15.977	0.0000	17.564	4708000	43	13.37	13.99	12.65	13.50	14.02	16.00	14.18	16.28
+	1.436	-1.75	0.30	P15864	Histone H1.2	Hist1h1c	802	28	28	10	61.3	61.3	30.7	21.266	0.0000	214.81	2900800000	1845	21.07	21.74	20.50	21.24	22.42	24.50	21.68	22.94
+	1.923	-1.76	0.29	Q9D2H6;Q9	Transcription factor Sp2	Sp2	4944	4	4	3	14.1	14.1	12.6	64.907	0.0000	29.01	2809200	44	13.09	12.04	12.97	12.17	13.95	15.10	13.34	14.93
+	4.894	-1.76	0.29	P50427	Steryl-sulfatase	Sts	1180	18	18	18	31.1	31.1	31.1	66.59	0.0000	92.507	39256000	218	15.62	15.95	15.79	15.43	17.23	17.49	17.58	17.54
+	1.931	-1.77	0.29	P84102	Small EDRK-rich factor 2	Serf2	1611	3	3	1	33.9	33.9	16.9	6.8998	0.0000	6.3143	5275800	33	13.55	13.94	12.20	14.10	14.53	15.44	15.24	15.67
+	1.566	-1.78	0.29	Q80UU1	Ankyrin repeat and zinc finger domain-containing protein 1	Ankzf1	2837	6	6	6	12.4	12.4	12.4	82.975	0.0000	16.946	5013500	32	14.00	12.85	14.86	12.21	14.80	15.42	15.54	15.31
+	1.582	-1.79	0.29	Q8BHA0	INO80 complex subunit C	Ino80c	3025	5	5	5	40.3	40.3	40.3	20.405	0.0000	27.682	9126800	99	14.54	15.18	12.95	15.34	15.68	16.85	16.00	16.63
+	2.164	-1.79	0.29	O54790	Transcription factor MafG	Mafg	424	6	4	4	36.4	27.8	27.8	17.876	0.0000	8.817	3669000	42	13.12	13.92	11.94	13.41	14.70	15.10	14.58	15.15
+	2.065	-1.79	0.29	Q8BQ30	Phostensin	Ppp1r18	3170	17	17	17	29.1	29.1	29.1	65.629	0.0000	57.829	47880000	202	15.36	16.54	16.14	14.61	17.51	17.35	17.03	17.91
+	0.688	-1.80	0.29	Q9D2R8	28S ribosomal protein S33, mitochondrial	Mrps33	4951	2	2	2	17.9	17.9	17.9	12.459	0.0018	3.1413	5257900	36	14.87	12.46	13.30	14.10	12.33	17.43	15.21	16.94
+	1.420	-1.80	0.29	P43277	Histone H1.3	Hist1h1d	1090	27	3	3	57	9.5	9.5	22.099	0.0000	66.351	600190000	298	20.50	21.20	19.77	20.16	21.84	23.93	21.06	22.02
+	3.467	-1.81	0.28	P45878	Peptidyl-prolyl cis-trans isomerase FKBP2	Fkbp2	1099	3	3	3	14.3	14.3	14.3	15.344	0.0000	10.835	13885000	56	16.09	15.37	15.44	15.28	16.97	17.62	17.19	17.66
+	1.518	-1.81	0.28	P58059	28S ribosomal protein S21, mitochondrial	Mrps21	1306	4	4	4	49.4	49.4	49.4	10.561	0.0000	18.747	10140000	48	15.63	15.68	13.29	14.84	16.67	17.25	15.78	17.00
+	2.465	-1.82	0.28	P26151	High affinity immunoglobulin gamma Fc receptor I	Fcgr1	918	10	10	10	24	24	24	44.887	0.0000	81.935	13880000	81	15.04	13.82	14.62	14.64	16.93	16.74	15.68	16.03
+	1.868	-1.84	0.28	P62754	40S ribosomal protein S6	Rps6	1443	33	33	33	69.5	69.5	69.5	28.68	0.0000	323.31	1583500000	1973	20.94	21.19	19.59	20.56	21.81	23.33	21.67	22.83
+	1.754	-1.86	0.28	Q921W0	Charged multivesicular body protein 1a	Chmp1a	4242	11	11	11	36.2	36.2	36.2	21.607	0.0000	22.244	35753000	216	14.45	15.42	14.19	16.18	16.28	17.27	16.40	17.72
+	2.438	-1.87	0.27	O09130	NFATC2-interacting protein	Nfatc2ip	326	4	4	4	18.9	18.9	18.9	45.121	0.0000	34.13	6878200	80	13.00	14.41	12.79	13.84	15.19	15.41	15.12	15.79
+	1.420	-1.88	0.27	Q9CYL5	Golgi-associated plant pathogenesis-related protein 1	Glipr2	4802	5	5	5	50.6	50.6	50.6	17.09	0.0000	20.304	6666400	48	14.90	14.23	12.99	12.59	15.57	16.57	14.33	15.76
+	1.478	-1.88	0.27	Q8VI33;Q6N	Transcription initiation factor TFIID subunit 9	Taf9	4026	7	7	7	37.1	37.1	37.1	28.979	0.0000	34.103	12557000	81	12.55	15.63	15.02	14.61	16.58	16.57	16.10	16.08
+	1.451	-1.90	0.27	E9PVX6		Mki67	222	25	25	25	13.9	13.9	13.9	350.86	0.0000	96.347	14579000	96	13.16	14.66	14.72	14.69	16.06	17.72	14.83	16.24
+	1.653	-1.92	0.26	P97822	Acidic leucine-rich nuclear phosphoprotein 32 family member E	Anp32e	1678	2	2	2	10.4	10.4	10.4	29.622	0.0000	24.04	4384600	57	13.37	12.09	12.38	13.52	14.83	16.14	14.37	13.69
+	3.041	-1.93	0.26	P62900	60S ribosomal protein L31	Rpl31	1466	12	12	12	64	64	64	14.463	0.0000	196.51	454470000	548	18.85	19.71	19.33	19.28	20.82	21.43	20.77	21.87
+	2.943	-1.93	0.26	Q9EPQ7	StAR-related lipid transfer protein 5	Stard5	5261	6	6	6	30	30	30	23.922	0.0000	22.719	12724000	100	14.86	15.54	14.23	15.11	16.75	16.96	16.42	17.31
+	1.703	-1.93	0.26	Q08288	Cell growth-regulating nucleolar protein	Lyar	1763	12	12	12	31.7	31.7	31.7	43.735	0.0000	46.755	6515000	48	12.98	14.15	11.70	13.66	15.28	15.27	14.15	15.51
+	2.371	-1.95	0.26	Q4QRL3	Coiled-coil domain-containing protein 88B	Ccdc88b	2065	64	64	64	48.8	48.8	48.8	166.61	0.0000	246.32	84677000	654	15.91	16.80	15.75	16.20	17.90	18.98	17.23	18.37
+	1.891	-1.96	0.26	Q9D1C9	Ribosomal RNA-processing protein 7 homolog A	Rrp7a	4906	6	6	6	31.1	31.1	31.1	32.399	0.0000	13.369	6846300	45	12.54	14.21	13.60	13.44	14.45	16.13	14.88	16.20
+	2.533	-1.98	0.25	Q9CWX9	Probable ATP-dependent RNA helicase DDX47	Ddx47	4737	6	6	6	17.6	17.6	17.6	50.638	0.0000	30.264	6940800	95	12.98	13.29	14.04	12.69	14.86	15.83	14.62	15.60
+	2.056	-1.98	0.25	Q8BG30	Negative elongation factor A	Nelfa	2961	16	16	16	46.2	46.2	46.2	57.584	0.0000	103.62	89515000	325	15.84	17.84	16.69	17.76	18.72	19.47	18.60	19.27
+	2.034	-1.99	0.25	Q9DBY8	Nuclear valosin-containing protein-like	Nvl	5184	10	10	10	17.1	17.1	17.1	94.475	0.0000	22.266	5254000	53	13.58	12.73	13.00	11.85	14.22	15.91	14.48	14.50
+	2.340	-1.99	0.25	Q8CCS6	Polyadenylate-binding protein 2	Pabpn1	3453	4	4	4	16.2	16.2	16.2	32.296	0.0000	15.916	10728000	53	14.41	15.16	15.39	13.93	16.54	17.17	15.94	17.22
+	1.193	-2.00	0.25	P17095	High mobility group protein HMG-I/HMG-Y	Hmga1	818	6	6	6	61.7	61.7	61.7	11.614	0.0000	42.975	10929000	64	13.59	13.72	12.18	15.37	14.96	17.20	16.13	14.57
+	2.408	-2.03	0.24	P58064	28S ribosomal protein S6, mitochondrial	Mrps6	1307	8	8	8	69.6	69.6	69.6	14.308	0.0000	13.647	4405600	56	13.80	12.68	13.72	12.32	14.90	15.27	14.66	15.82
+	2.386	-2.04	0.24	P31996	Macrosialin	Cd68	992	5	5	5	13.5	13.5	13.5	34.817	0.0002	6.0657	52814000	60	16.03	16.85	17.30	16.13	19.19	18.81	17.64	18.82
+	2.144	-2.04	0.24	Q6SKR2		N6amt1	2660	8	8	8	47.2	47.2	47.2	22.983	0.0000	27.032	12489000	94	13.53	14.69	15.78	14.79	16.10	16.85	16.90	17.10
+	2.305	-2.04	0.24	Q9D937	Uncharacterized protein C11orf98 homolog		5092	6	6	6	41.5	41.5	41.5	14.098	0.0000	14.513	16939000	108	13.37	14.46	14.01	14.40	15.30	16.91	15.54	16.66

+	2.231	-2.06	0.24	Q9DB42	Zinc finger protein 593	Znf593	5126	5	5	5	41	41	41	15.147	0.0000	23.496	7338400	70	13.83	13.36	13.34	14.56	14.93	16.39	15.37	16.61
+	1.929	-2.10	0.23	Q8VDT9	39S ribosomal protein L50, mitochondrial	Mrpl50	3972	3	3	3	27	27	27	18.213	0.0000	12.972	6894500	80	13.49	14.33	12.84	12.38	14.50	15.38	15.12	16.46
+	2.897	-2.12	0.23	Q9CPU9	Probable low affinity copper uptake protein 2	Slc31a2	4537	1	1	1	17.5	17.5	17.5	16.069	0.0000	54.272	8638300	66	12.78	13.33	13.25	14.43	15.78	15.75	15.52	15.22
+	1.837	-2.13	0.23	Q9CQN7	39S ribosomal protein L41, mitochondrial	Mrpl41	4617	8	8	8	60.7	60.7	60.7	15.261	0.0000	11.867	13994000	102	12.81	15.26	13.82	13.89	15.62	16.04	15.50	17.14
+	0.760	-2.13	0.23	O82803			518	16	16	16	91.2	91.2	91.2	22.344	0.0000	238.41	41075000	123	11.60	14.20	10.90	11.82	12.61	12.30	14.71	17.43
+	3.528	-2.18	0.22	Q8C1D8	Protein IWS1 homolog	Iws1	3364	6	6	6	9.5	9.5	9.5	85.247	0.0000	37.891	4307300	78	12.75	13.03	13.12	12.44	14.54	15.68	14.77	15.07
+	1.511	-2.18	0.22	O08550	Histone-lysine N-methyltransferase 2B	Kmt2b	271	14	14	14	7.5	7.5	7.5	294.82	0.0000	71.898	13973000	74	13.60	14.57	14.96	15.36	16.83	18.06	14.91	17.40
+	2.518	-2.19	0.22	Q8BZR9	Uncharacterized protein C17orf85 homolog		3318	12	12	12	19.7	19.7	19.7	70.042	0.0000	37.218	9230600	92	13.18	14.33	13.43	13.04	14.97	15.98	15.26	16.53
+	1.570	-2.20	0.22	Q91Z49	UAP56-interacting factor	Fyttd1	4186	15	15	15	41	41	41	35.887	0.0000	119.67	28629000	182	14.85	14.92	13.14	14.86	15.16	17.60	16.08	17.71
+	0.927	-2.20	0.22	P01887	Beta-2-microglobulin	B2m	619	3	3	3	22.7	22.7	22.7	13.779	0.0004	4.5623	11159000	52	15.59	14.03	12.50	15.85	17.56	18.36	16.73	14.10
+	1.663	-2.20	0.22	Q9D5R3	Centrosomal protein of 83 kDa	Cep83	4984	1	1	1	1.4	1.4	1.4	81.997	0.0095	1.8176	22604000	78	13.13	16.05	15.72	15.73	16.96	16.99	17.63	17.85
+	2.792	-2.22	0.22	Q99JH1	Ribonuclease P protein subunit p25-like protein	Rpp25l	4339	6	6	6	46	46	46	17.675	0.0000	59.082	15898000	137	13.52	15.08	14.86	15.12	16.69	17.18	16.53	17.04
+	3.121	-2.23	0.21	P47964	60S ribosomal protein L36	Rpl36	1136	10	10	10	40	40	40	12.215	0.0000	37.347	520770000	803	18.39	19.13	18.05	18.55	20.30	21.44	20.33	20.97
+	2.047	-2.24	0.21	Q9CXZ1	NADH dehydrogenase [ubiquinone] iron-sulfur protein 4, mitochondrial	Ndufs4	4776	8	8	8	53.1	53.1	53.1	19.784	0.0000	20.09	4511800	35	11.26	13.43	12.60	13.27	15.02	15.18	13.94	15.39
+	1.440	-2.25	0.21	Q9WVF8	Tumor suppressor candidate 2	Tusc2	5747	2	2	2	33.6	33.6	33.6	12.136	0.0011	3.598	7490900	21	13.88	15.91	12.88	16.27	17.30	16.54	16.74	17.36
+	2.685	-2.25	0.21	Q8K003	Translation machinery-associated protein 7	Tma7	3590	7	6	6	53.1	40.6	40.6	7.0662	0.0000	11.939	25853000	94	14.26	14.88	14.11	15.09	16.39	17.14	16.10	17.71
+	1.434	-2.25	0.21	P58468	Protein FAM207A	Fam207a	1317	9	9	9	47	47	47	24.814	0.0000	20.39	12197000	76	13.92	12.29	11.76	15.29	15.56	15.12	15.30	16.29
+	2.069	-2.27	0.21	Q8CGP0;Q9	Histone H2B type 3-B;Histone H2B type 3-A	Hist3h2bb;Hist3h2	3510	18	2	0	79.4	7.9	0	13.908	0.0025	2.9282	3927700	23	13.93	12.79	12.22	12.19	14.99	15.00	14.06	16.15
+	2.471	-2.37	0.19	Q91X96	Guanine nucleotide exchange factor MSS4	Rabif	4138	3	3	3	28.5	28.5	28.5	13.915	0.0000	9.2748	4970000	46	13.22	13.07	14.84	13.64	15.75	16.57	15.34	16.58
+	1.142	-2.40	0.19	A0A5K7RLP0			1	2	2	2	3.9	3.9	3.9	65.089	0.0090	1.8647	14007000	14	13.91	13.31	13.25	14.74	17.90	13.15	16.74	17.03
+	3.851	-2.48	0.18	Q9JMG1	Endothelial differentiation-related factor 1	Edf1	5509	10	10	10	68.9	68.9	68.9	16.369	0.0000	71.628	13406000	109	13.94	13.46	14.13	13.79	15.74	16.36	16.17	16.97
+	2.102	-2.49	0.18	P97350	Plakophilin-1	Pkp1	1626	17	17	17	27.2	27.2	27.2	80.895	0.0000	52.979	24980000	73	14.37	15.69	13.17	14.53	15.87	16.94	17.43	17.48
+	2.361	-2.50	0.18	Q9D1R9	60S ribosomal protein L34	Rpl34	4936	11	11	11	52.1	52.1	52.1	13.293	0.0000	40.795	404730000	550	18.54	18.75	16.74	17.72	20.26	21.05	19.59	20.85
+	2.639	-2.50	0.18	Q9D773	39S ribosomal protein L2, mitochondrial	Mrpl2	5021	12	12	12	43.8	43.8	43.8	33.34	0.0000	176	30239000	186	14.06	15.15	14.27	14.82	15.98	17.62	16.85	17.87
+	2.210	-2.54	0.17	P61166	Transmembrane protein 258	Tmem258	1374	2	2	2	10.1	10.1	10.1	9.0788	0.0009	3.658	3167600	28	13.58	13.08	13.87	11.86	16.07	16.14	14.36	15.97
+	2.238	-2.56	0.17	P61804	Dolichyl-diphosphooligosaccharide--protein glycosyltransferase subunit	Dad1	1390	2	2	2	19.5	19.5	19.5	12.497	0.0002	6.0643	7126900	46	14.46	12.95	13.50	13.27	16.70	15.96	14.71	17.05
+	2.578	-2.60	0.16	P0CG14	Chromosome transmission fidelity protein 8 homolog isoform 2	Chtf8	709	12	12	12	36.2	36.2	36.2	52.244	0.0000	51.981	16896000	107	14.21	15.02	13.37	12.86	16.34	17.09	15.97	16.49
+	1.561	-2.67	0.16	Q8CI70	Leucine-rich repeat-containing protein 20	Lrrc20	3542	5	5	5	39.7	39.7	39.7	20.809	0.0000	219.94	16508000	76	13.90	16.17	16.76	12.99	17.15	18.03	17.45	17.88
+	1.356	-2.73	0.15	Q8K4K2	Tribbles homolog 3	Trib3	3719	1	1	1	4.2	4.2	4.2	39.022	0.0027	2.7002	5545500	36	13.88	13.36	13.99	11.80	14.23	18.18	14.58	16.96
+	2.189	-2.76	0.15	Q64475	Histone H2B type 1-B	Hist1h2bb	2447	25	1	1	92.1	8.7	8.7	13.952	0.0058	2.1731	6676700	14	12.01	13.95	13.45	15.26	16.62	16.40	16.43	16.27
+	2.627	-2.82	0.14	P62858	40S ribosomal protein S28	Rps28	1456	9	9	9	72.5	72.5	72.5	7.8409	0.0000	71.422	49709000	237	14.11	16.20	15.35	14.75	17.44	17.83	17.50	18.91
+	2.706	-2.88	0.14	Q8K1I7	WAS/WASL-interacting protein family member 1	Wipf1	3628	10	10	10	24.9	24.9	24.9	50.08	0.0000	21.263	31171000	123	14.50	15.88	15.24	13.83	17.24	18.05	17.17	18.52
+	2.613	-2.92	0.13	Q9CQY6	Ubiquinol-cytochrome-c reductase complex assembly factor 2	Uqcc2	4649	3	3	3	27.9	27.9	27.9	16.32	0.0000	15.97	7918300	63	13.85	12.56	12.56	14.84	16.12	16.42	16.09	16.88
+	2.944	-3.13	0.11	P62862	40S ribosomal protein S30	Fau	1457	4	4	4	33.9	33.9	33.9	6.6478	0.0002	5.6604	102650000	102	15.77	18.05	16.93	16.66	19.31	20.51	19.82	20.27
+	2.482	-3.15	0.11	Q9CR61	NADH dehydrogenase [ubiquinone] 1 beta subcomplex subunit 7	Ndufb7	4673	4	4	4	45.3	45.3	45.3	16.331	0.0000	36.778	10809000	56	15.54	13.14	12.98	13.56	16.41	17.25	16.46	17.69
+	2.775	-3.17	0.11	P0DN34			713	4	4	4	49.1	49.1	49.1	6.954	0.0000	7.0288	30373000	97	13.92	15.75	16.37	16.16	18.77	19.13	18.24	18.72
+	1.304	-3.18	0.11	P15252			792	13	13	13	87	87	87	14.722	0.0000	104.74	19718000	60	9.13	11.49	10.67	10.27	13.55	12.04	11.72	16.96
+	2.184	-3.25	0.11	P52760	Ribonuclease UK114	Hrsp12	1223	7	7	7	52.6	52.6	52.6	14.255	0.0000	23.684	16919000	86	12.30	15.34	14.77	13.45	16.32	17.59	16.77	18.18
+	1.831	-3.77	0.07	Q8BK35		Gltscr2	3093	18	18	18	43	43	43	55.792	0.0000	72.209	15067000	180	12.41	14.49	10.61	12.79	16.35	17.69	14.17	17.17

list of proteins in L1 samples:

log2 transformed LFQ intensity values (after removal of contaminants), missing values imputation from normal distribution (shift: 1.6; width: 0.4)
statistical test: two-sided t-test with a p-value of 0.05 and s0 of 1 and permutation-based FDR of 5 % (250 randomizations for multi-testing correction)

																			log2 transformed LFQ intensity values							
																			probe				no probe			
Significant	neg. log(p-value)	Difference (log2(p)-log2(np))	fold change (p/np)	Protein IDs	Protein names	Gene names	id	Peptides	Razor + unique peptides	Unique peptides	Sequence coverage [%]	Unique + razor sequence coverage [%]	Unique sequence coverage [%]	Mol. weight [kDa]	Q-value	Score	Intensity	MS/MS count	L1+1	L1+2	L1+3	L1+4	L1-1	L1-2	L1-3	L1-4
+	2.676	4.80	27.87	Q80YA7	Dipeptidyl peptidase 8	Dpp8	2900	22	21	21	25.4	24.6	24.6	102.18	0.0000	79.281	33327000	212	17.27	15.94	16.92	16.81	9.64	13.51	11.45	13.15
+	1.297	4.64	24.90	Q69ZF3	Non-lysosomal glucosylceramidase	Gba2	2482	3	3	3	4.9	4.9	4.9	103.29	0.0002	5.3502	239110000	28	21.61	20.16	13.15	18.38	12.84	14.92	13.72	13.27
+	4.260	4.42	21.35	Q8K4F5	Alpha/beta hydrolase domain-containing protein 11	Abhd11	3715	12	12	12	60.9	60.9	60.9	33.56	0.0000	233.79	45328000	194	17.44	16.80	17.29	17.29	12.91	13.69	11.68	12.86
+	2.012	4.22	18.57	Q8VHQ9	Acyl-coenzyme A thioesterase 11	Acot11	4023	17	17	17	42.9	42.9	42.9	67.354	0.0000	323.31	67620000	316	17.41	16.65	16.32	17.43	13.87	10.65	15.25	11.18
+	1.365	3.97	15.67	O35309	N-myc-interactor	Nmi	355	4	4	4	18.5	18.5	18.5	35.235	0.0002	5.4976	76893000	32	20.34	19.32	19.57	20.53	18.37	12.63	18.74	14.13
+	1.487	3.94	15.33	Q91WF7	Polyphosphoinositide phosphatase	Fig4	4108	6	6	6	7.7	7.7	7.7	103.45	0.0000	24.906	29927000	36	18.44	16.52	20.11	18.78	18.02	13.65	12.38	14.05
+	3.126	3.85	14.38	Q6PE15	Mycophenolic acid acyl-glucuronide esterase, mitochondrial	Abhd10	2623	17	17	17	63.3	63.3	63.3	33.04	0.0000	164.24	32945000	154	16.72	17.03	16.31	14.87	13.26	11.41	12.51	12.35
+	1.220	3.80	13.94	Q5QR91	Uncharacterized protein C17orf78 homolog	Gm11437	2125	2	2	2	8.3	8.3	8.3	32.583	0.0024	2.9771	75602000	33	20.25	18.08	18.85	20.16	12.11	18.70	17.58	13.74
+	2.929	3.79	13.81	P70158	Acid sphingomyelinase-like phosphodiesterase 3a	Smpdl3a	1528	15	15	15	42.2	42.2	42.2	49.857	0.0000	323.31	50300000	324	17.81	17.41	18.10	17.98	15.55	12.68	14.57	13.36
+	1.669	3.55	11.71	Q9WVM3	Anaphase-promoting complex subunit 7	Anapc7	5758	5	5	5	13.5	13.5	13.5	63.02	0.0000	7.876	31498000	38	18.48	17.70	19.22	19.28	13.88	14.37	18.36	13.87
+	4.752	3.52	11.49	Q9ET22	Dipeptidyl peptidase 2	Dpp7	5354	20	20	20	48.6	48.6	48.6	56.253	0.0000	323.31	646300000	662	21.44	21.42	20.25	20.93	17.64	17.39	17.57	17.35
+	2.063	3.33	10.08	Q9DBM2	Peroxisomal bifunctional enzyme;Enoyl-CoA hydratase/3,2-trans	Ehhadh	5164	7	7	6	13	13	11	78.301	0.0000	6.6723	13039000	27	17.73	15.90	17.05	17.90	12.97	13.63	15.96	12.69
+	1.483	3.30	9.88	Q9DCZ4	Apolipoprotein O	Apoo	5238	6	6	6	38.9	38.9	38.9	22.604	0.0000	42.086	37077000	27	18.25	18.63	13.51	17.50	13.27	13.37	13.84	14.19
+	2.572	3.27	9.64	A2AJQ3	Probable C-mannosyltransferase DPY19L4	Dpy19l4	31	6	6	6	13.9	13.9	13.9	83.603	0.0000	10.727	24856000	32	16.41	17.36	17.52	16.76	12.24	14.44	15.00	13.29
+	5.099	3.26	9.56	Q8BVG4	Dipeptidyl peptidase 9	Dpp9	3239	41	41	40	57.4	57.4	57.2	98	0.0000	323.31	162960000	699	19.35	19.16	19.47	19.54	16.42	15.49	16.37	16.21
+	2.475	3.14	8.80	P41233	ATP-binding cassette sub-family A member 1	Abca1	1067	7	7	7	5.4	5.4	5.4	253.91	0.0000	9.4724	15293000	22	16.86	16.33	14.29	16.65	12.20	13.39	12.54	13.45
+	1.267	3.09	8.52	P43024	Cytochrome c oxidase subunit 6A1, mitochondrial	Cox6a1	1085	4	4	4	59.5	59.5	59.5	12.352	0.0000	101.82	42226000	101	18.31	17.96	19.80	19.00	13.06	14.14	18.10	17.40
+	4.867	3.02	8.10	P16675	Lysosomal protective protein;Lysosomal protective protein 32 kD	Ctsa	813	21	21	21	43.2	43.2	43.2	53.844	0.0000	323.31	1641900000	1311	22.27	22.54	21.53	22.03	19.28	19.04	18.83	19.15
+	1.466	2.97	7.82	Q91W96	Anaphase-promoting complex subunit 4	Anapc4	4099	4	4	4	6.6	6.6	6.6	91.707	0.0002	5.4453	15282000	26	16.78	12.88	17.47	16.50	11.96	12.93	13.44	13.42
+	4.372	2.95	7.72	Q8BVQ5	Protein phosphatase methylesterase 1	Ppme1	3246	17	17	17	53.9	53.9	53.9	42.256	0.0000	200.22	64703000	347	16.96	16.85	17.90	17.42	14.07	14.73	14.23	14.32
+	1.746	2.94	7.65	Q9R118	Serine protease HTRA1	Htra1	5647	21	21	21	45.2	45.2	45.2	51.213	0.0000	202.98	42152000	249	16.39	16.78	12.97	15.06	11.67	12.11	12.64	13.04
+	1.049	2.93	7.61	Q6A009	E3 ubiquitin-protein ligase listerin	Ltn1	2494	3	3	3	4.5	4.5	4.5	198.92	0.0000	13.423	28419000	21	17.57	17.47	19.55	17.57	13.00	14.78	19.03	13.65
+	4.154	2.92	7.55	Q61239	Protein farnesyltransferase/geranylgeranyltransferase type-1 sub	Fnta	2284	19	19	19	53.1	53.1	53.1	44.013	0.0000	130.58	20787000	130	15.62	16.09	15.95	16.67	12.64	13.13	13.64	13.26
+	1.922	2.91	7.50	Q8BLK9	Ribosomal protein S6 kinase delta-1	Rps6kc1	3119	6	6	6	8.6	8.6	8.6	115.71	0.0000	16.751	7009600	19	16.94	16.32	16.94	16.56	15.85	12.02	14.02	13.24
+	2.567	2.84	7.18	O35298	Acyloxyacyl hydrolase;Acyloxyacyl hydrolase small subunit;Acylo	Aoah	354	14	14	14	25.1	25.1	25.1	65.154	0.0000	323.31	35804000	152	14.80	15.15	16.07	15.64	11.18	13.16	12.46	13.48
+	4.325	2.83	7.10	Q923D4	Splicing factor 3B subunit 5	Sf3b5	4283	6	6	6	68.6	68.6	68.6	10.119	0.0000	146.14	43936000	177	18.28	18.39	18.95	18.61	15.16	15.60	16.24	15.92
	0.922	2.79	6.94	Q80YR4	Zinc finger protein 598	Znf598	2905	5	5	5	7.7	7.7	7.7	99.191	0.0000	23.076	29529000	29	16.94	17.71	20.77	17.18	16.77	14.56	17.89	12.20
+	1.553	2.75	6.74	Q9CR67	Transmembrane protein 33	Tmem33	4676	3	3	3	15.4	15.4	15.4	28.031	0.0000	9.9279	17466000	30	18.06	17.92	18.28	17.78	16.95	14.16	16.78	13.15
+	2.094	2.69	6.47	O88653	Ragulator complex protein LAMTOR3	Lamtor3	554	5	5	5	58.1	58.1	58.1	13.553	0.0000	30.438	7984100	66	15.63	16.04	17.39	16.14	14.85	13.87	13.65	12.06
+	1.208	2.59	6.02	Q8BVA5	UPF0554 protein C2orf43 homolog		3236	8	8	8	35	35	35	37.374	0.0000	93.404	44246000	95	17.71	17.46	18.35	17.51	16.81	14.15	17.19	12.52
	0.754	2.54	5.83	Q8BYH8	Chromodomain-helicase-DNA-binding protein 9	Chd9	3299	3	3	2	1.3	1.3	0.8	323.86	0.0073	1.9854	25571000	41	13.07	17.59	17.78	18.66	11.93	13.51	17.10	14.39
+	2.967	2.54	5.81	P06801	NADP-dependent malic enzyme	Me1	665	11	11	11	36.4	36.4	36.4	63.953	0.0000	138.55	8946300	72	15.73	15.93	16.58	16.43	14.00	12.81	14.50	13.20
+	3.103	2.54	5.80	Q3U1T9	DENN domain-containing protein 1B	Dennd1b	1904	4	4	4	7.7	7.7	7.7	85.525	0.0000	6.8175	8163400	26	16.25	15.61	15.06	16.17	12.58	13.18	13.16	14.03
	0.797	2.53	5.76	P61211	ADP-ribosylation factor-like protein 1	Arl1	1378	5	5	5	22.1	22.1	22.1	20.411	0.0000	170.4	30006000	232	17.26	16.88	18.21	17.40	16.92	10.31	15.90	16.53
	0.833	2.52	5.74	Q8C196	Carbamoyl-phosphate synthase [ammonia], mitochondrial	Cps1	3361	28	28	27	26.3	26.3	25.2	164.62	0.0000	82.208	8783400	34	20.04	14.45	13.77	14.39	12.67	12.80	12.86	14.25
+	5.396	2.47	5.53	Q02819	Nucleobindin-1	Nucb1	1720	15	15	15	34	34	34	53.408	0.0000	83.253	12525000	160	16.23	15.93	16.16	16.07	14.03	13.62	13.47	13.39
+	2.338	2.46	5.50	A6H5Z3	Exocyst complex component 6B	Exoc6b	60	30	30	29	44.1	44.1	43.1	94.128	0.0000	205.61	38086000	310	15.53	14.47	16.57	15.72	12.87	14.03	13.25	12.32
+	1.782	2.46	5.50	O35678	Monoglyceride lipase	Mgll	395	16	16	16	67.7	67.7	67.7	33.387	0.0000	140.93	41096000	144	16.16	16.46	14.65	14.86	11.47	14.31	13.36	13.14
+	3.265	2.46	5.48	A2AJ88	Patatin-like phospholipase domain-containing protein 7	Pnpla7	28	34	34	33	35.9	35.9	35.4	150.49	0.0000	250.69	58040000	439	17.01	16.56	17.45	16.61	14.70	13.54	14.82	14.75
+	4.413	2.44	5.42	Q8BLF1	Neutral cholesterol ester hydrolase 1	Nceh1	3117	31	31	31	67.2	67.2	67.2	45.739	0.0000	323.31	1348900000	1512	21.96	21.50	22.42	21.86	19.54	19.47	19.79	19.19
+	2.174	2.44	5.41	Q7TPD0	Integrator complex subunit 3	Ints3	2766	7	7	7	12	12	12	117.94	0.0000	166.63	5117900	75	15.18	14.11	16.31	15.35	13.94	12.73	12.40	12.14
+	3.242	2.41	5.30	Q6IRU2	Tropomyosin alpha-4 chain	Tpm4	2519	15	14	14	75.8	70.6	70.6	28.467	0.0000	89.828	18402000	144	16.58	16.57	17.43	17.20	15.00	14.19	15.06	13.90
+	2.815	2.40	5.29	P70665	Sialate O-acetylesterase;Sialate O-acetylesterase small subunit;	Siae	1580	3	3	3	5.9	5.9	5.9	60.775	0.0004	4.4182	3293500	16	15.86	14.15	14.76	15.40	12.96	12.57	13.01	12.02
	0.979	2.39	5.26	Q62095	ATP-dependent RNA helicase DDX3Y	Ddx3y	2356	35	3	3	47.3	6.1	6.1	73.427	0.0000	63.04	46787000	155	18.18	18.13	18.65	18.54	17.91	12.40	16.20	17.42
+	3.656	2.39	5.24	P48774	Glutathione S-transferase Mu 5	Gstm5	1151	10	8	8	39.7	36.6	36.6	26.635	0.0000	21.619	7033300	86	15.49	15.26	15.45	15.59	13.51	12.21	13.42	13.08
	0.525	2.38	5.21	P58801	Receptor-interacting serine/threonine-protein kinase 2	Ripk2	1323	9	9	9	25.2	25.2	25.2	60.399	0.0000	28.031	82728000	32	19.59	19.37	18.65	18.67	20.79	12.37	19.66	13.93
+	2.423	2.38	5.21	E9Q309		Cep350	231	3	3	3	1.6	1.6	1.6	346.45	0.0044	2.4056	7828100	11	16.92	16.26	17.95	17.25	15.69	14.76	13.83	14.59
	0.732	2.33	5.03	Q5S006	Leucine-rich repeat serine/threonine-protein kinase 2	Lrrk2	2129	4	4	4	2.5	2.5	2.5	284.73	0.0020	3.0891	18011000	12	17.52	17.23	16.78	17.52	12.88	11.74	18.13	16.96
+	2.336	2.32	4.99	Q3UVG3	Protein FAM91A1	Fam91a1	2037	12	12	12	19.1	19.1	19.1	93.462	0.0000	47.67	7437400	83	14.94	14.53	16.00	15.35	13.91	11.94	13.17	12.52
+	1.999	2.30	4.91	Q9D3B1	Very-long-chain (3R)-3-hydroxyacyl-CoA dehydratase 2	Hacd2	4963	3	3	3	11.8	11.8	11.8	28.402	0.0000	19.016	8811600	71	16.19	15.49	17.81	15.90	14.28	13.96	14.83	13.14
+	1.145	2.29	4.88	Q9JII5	DAZ-associated protein 1	Dazap1	5401	9	9	9	35	35	35	43.214	0.0000	175.79	21286000	130	16.31	16.87	16.06	16.97	16.01	12.03	13.03	16.00
+	3.265	2.24	4.72	Q8BUY9	Geranylgeranyl transferase type-1 subunit beta	Pggt1b	3233	6	6	5	21.8	21.8	16.2	42.354	0.0000	29.669	7461500	57	14.83	14.67	14.16	14.83	11.76	12.42	13.17	12.19
+	5.611	2.22	4.65	P49710	Hematopoietic lineage cell-specific protein	Hcls1	1167	34	34	34	56.2	56.2	56.2	54.24	0.0000	323.31	148070000	749	19.79	19.62	19.91	20.08	17.81	17.59	17.72	17.42
+	1.651	2.21	4.63	Q9R0I7	YLP motif-containing protein 1	Ylpm1	5627	11	11	11	12.2	12.2	12.2	155.13	0.0000	16.553	9909700	34	16.49	16.88	16.78	16.97	15.65	13.51	15.96	13.17
+	4.131	2.21	4.61	Q9QUR6	Prolyl endopeptidase	Prep	5522	65	65	65	77.5	77.5	77.5	80.751	0.0000	323.31	840700000	1988	19.87	19.78	19.01	19.96	17.44	17.28	17.65	17.44
	0.734	2.20	4.59	A8C756	Thyroid adenoma-associated protein homolog	Thada	66	3	3	3	2.3	2.3	2.3	217.29	0.0096	1.8097	16430000	16	17.63	17.40	17.49	17.72	17.57	13.41	18.15	12.30
+	4.287	2.20	4.59	Q8BGD9	Eukaryotic translation initiation factor 4B	Eif4b	2978	27	27	26	51.4	51.4	49.9	68.839	0.0000	162.39	44645000	257	18.25	17.99	18.49	18.43	16.57	15.87	16.18	15.75
+	3.109	2.19	4.56	Q9D0B0	Serine/arginine-rich splicing factor 9	Srsf9	4855	8	7	6	38.7	35.6	28.8	25.661	0.0000	6.5606	4394100	16	15.23	14.73	14.84	15.35	12.08	13.21	13.50	12.61
+	1.520	2.19	4.56	Q8BZ98	Dynamin-3	Dnm3	3311	18	4	4	17.8	5.2	5.2	97.189	0.0000	14.961	14447000	92	16.04	16.10	17.22	16.36	15.49	13.53	15.41	12.54
	0.641	2.15	4.45	P09041	Phosphoglycerate kinase 2	Pgk2	691	12	4	3	25.2	10.8	5.8	44.852	0.0009	3.8079	7468200	33	15.01	12.62	19.53	17.17	12.51	14.36	13.41	15.44
+	2.154	2.14	4.42	P54728	UV excision repair protein RAD23 homolog B	Rad23b	1253	7	7	6	26.2	26.2	26.2	43.512	0.0000	19.149	4802000	40	14.91	15.35	14.78	15.52	13.24	13.77	13.45	11.52
+	2.600	2.12	4.34	Q9DBL9	1-acylglycerol-3-phosphate O-acyltransferase ABHD5	Abhd5	5162	8	8	8	28.8	28.8	28.8	39.154	0.0000	59.913	21459000	104	16.36	15.93	14.86	16.63	13.92	13.68	14.25	13.46
+	2.685	2.11	4.31	Q9CYZ6	Uncharacterized protein C19orf60 homolog		4811	3	3	3	28.4	28.4	28.4	18.706	0.0000	57.59	3387900	69	14.94	14.70	15.37	14.81	12.66	12.79	13.88	12.06
+	2.190	2.10	4.30	Q3TRM4	Neuropathy target esterase	Pnpla6	1871	17	16	16	17.2	16.6	16.6	149.54	0.0000	126.55	12390000	130	15.17	13.58	14.95	14.66	12.66	12.05	13.46	11.76
+	1.437	2.10	4.30	Q9D0M5	Dynein light chain 2, cytoplasmic	Dynll2	4880	9	6	6	74.2	43.8	43.8	10.35	0.0000	137.53	18315000	146	16.72	16.78	17.57	16.72	15.82	13.83	16.43	13.30
+	2.166	2.10	4.29	Q8K2V6	Importin-11	Ipo11	3675	4	4	4	7.8	7.8	7.8	112.41	0.0000	83.189	3770100	75	13.64	14.62	15.68	14.70	13.34	11.83	12.49	12.59
+	1.976	2.09	4.26	P84244;P0	Histone H3.3;Histone H3.3C	H3f3a;H3f3c	1614	19	1	1	77.9	23.5	23.5	15.328	0.0000	301.05	44899000	102	19.23	18.99	19.13	19.60	18.68	17.19	16.12	16.60
+	2.048	2.08	4.24	P08101	Low affinity immunoglobulin gamma Fc region receptor II	Fcgr2	678	3	1	1	10.3	4.8	4.8	36.695	0.0004	4.6315	3694000	32	14.82	14.46	15.72	16.05	12.40	12.57	13.85	13.89
+	1.686	2.08	4.23	O70251	Elongation factor 1-beta	Eef1b	479	7	7	7	33.3	33.3	33.3	24.693	0.0000	60.173	26781000	126	17.37	17.61	17.19	17.80	13.51	16.49	15.81	15.84
	1.074	2.08	4.22	Q8C142	Low density lipoprotein receptor adapter protein 1	Ldlrap1	3351	4	4	4	21.1	21.1	21.1	33.975	0.0000	10.806	9718100	39	16.47	16.77	16.07	16.67	17.33	14.05	13.06	13.22
+	1.780	2.08	4.22	P14069	Protein S100-A6	S100a6	771	11	11	11	97.8	97.8	97.8	10.051	0.0000	82.254	36216000	173	18.20	18.72	19.26	18.71	17.40	14.87	17.17	17.14
+	1.799	2.08	4.22	Q91WU5	Arsenite methyltransferase	As3mt	4129	5	5	5	19.7	19.7	19.7	41.793	0.0000	33.161	9653800	108	16.10	16.07	16.33	16.48	15.71	12.81	14.47	13.67
+	1.710	2.07	4.21	Q8BMC0	Lysophosphatidic acid receptor 6	Lpar6	3132	3	3	3	11.3	11.3	11.3	39.438	0.0000	16.053	3537500	23	14.25	13.32	15.68	15.25	13.68	12.47	12.07	11.99
	0.851	2.07	4.20	Q3UE37	Ubiquitin-conjugating enzyme E2 Z	Ube2z	1953	6	6	6	24.7	24.7	24.7	38.368	0.0000	38.193	25067000	93	16.92	17.03	18.41	16.56	16.70	14.66	17.17	12.11
+	3.213	2.05	4.14	Q8VEB4	Group XV phospholipase A2	Pla2g15	3998	12	12	12	29.9	29.9	29.9	47.307	0.0000	74.85	84748000	235	17.61	17.74	18.76	17.73	16.18	15.47	16.12	15.87
	0.677	2.04	4.13	Q6ZWY9;Q	Histone H2B type 1-C/E/G;Histone H2B type 1-H	Hist1h2bc;Hist1h	2712	27	2	0	96	17.5	0	13.906	0.0000	142.82	102030000	57	17.31	18.29	22.25	19.85	18.03	14.67	17.50	19.33
+	2.357	2.04	4.11	Q8CFE3	REST corepressor 1	Rcor1	3482	6	6	6	16.9	16.9	16.9	52.714	0.0000	41.438	5661200	59	15.25	14.95	16.40	15.03	13.15	14.22	13.36	12.74
	0.891	2.03	4.09	Q4VAC9	Pleckstrin homology domain-containing family G member 3	Plekhg3	2068	4	4	4	4.5	4.5	4.5	148.52	0.0000	7.4195	14687000	12	17.07	17.01	17.13	17.55	16.75	13.92	17.39	12.57
+	1.769	2.03	4.08	Q3UUQ7	GPI inositol-deacylase	Pgap1	2033	18	18	18	26	26	26	104.58	0.0000	128.45	31020000	269	16.31	15.61	16.04	16.14	14.99	14.21	14.53	12.25
+	1.692	2.03	4.08	Q9Z329	Inositol 1,4,5-trisphosphate receptor type 2	Itpr2	5879	10	10	10	5.8	5.8	5.8	307.47	0.0000	47.355	2477700	47	14.80	14.23	16.36	14.46	12.16	14.16	12.53	12.89
+	2.032	2.00	4.00	Q9R0Q3	Transmembrane emp24 domain-containing protein 2	Tmed2	5636	6	6	6	27.4	27.4	27.4	22.705	0.0000	15.976	9532400	58	16.45	16.16	15.73	16.40	14.91	14.20	14.88	12.76
+	2.145	1.98	3.93	Q7TMR0	Lysosomal Pro-X carboxypeptidase	Prcp	2749	14	14	14	29.1	29.1	29.1	55.026	0.0000	119.84	114550000	296	17.29	17.08	15.68	16.76	15.35	14.41	15.24	13.92
+	1.485	1.97	3.93	O35448	Lysosomal thioesterase PPT2	Ppt2	370	4	4	4	21.9	21.9	21.9	34.366	0.0000	74.372	9897900	42	15.16	16.95	13.89	14.10	13.20	13.39	12.85	12.76
	1.207	1.96	3.89	P14106	Complement C1q subcomponent subunit B	C1qb	772	6	6	6	31.6	31.6	31.6	26.717	0.0000	43.662	10015000	51	16.25	16.16	17.79	17.89	15.88	16.38	13.14	14.84
+	2.198	1.91	3.77	P54310	Hormone-sensitive lipase	Lipe	1251	34	34	34	64.8	64.8	64.8	83.347	0.0000	323.31	78384000	414	17.65	17.19	19.07	18.17	15.76	15.79	16.77	16.10
	0.941	1.91	3.75	Q80VJ3	2-deoxynucleoside 5-phosphate N-hydrolase 1	Dnph1	2852	9	9	9	71.7	71.7	71.7	18.977	0.0000	124.93	20392000	146	16.93	16.81	16.43	17.11	16.24	17.06	13.42	12.92
+	1.463	1.90	3.74	Q9D8N2	Protein FAM45A	Fam45a	5066	6	6	6	26.6	26.6	26.6	40.42	0.0000	87.408	5548000	56	15.64	14.25	16.27	14.67	12.19	14.63	13.59	12.81
+	2.863	1.89	3.71	P54987	Cis-aconitate decarboxylase	Irg1	1263	26	26	26	55.7	55.7	55.7	53.758	0.0000	323.31	148970000	728	17.81	17.26	18.88	17.98	16.04	16.09	16.19	16.05
+	2.819	1.85	3.61	Q8VE09	Tetratricopeptide repeat protein 39C	Ttc39c	3979	7	7	7	17.4	17.4	17.4	65.444	0.0000	51.153	6001800	56	15.07	14.31	15.13	13.91	12.34	12.95	12.69	13.03
	1.446	1.85	3.60	Q8VEK0	Cell cycle control protein 50A	Tmem30a	4010	4	4	4	15.9	15.9	15.9	41.06	0.0000	14.378	4216500	28	13.47	14.59	16.61	15.47	13.48	13.17	13.37	12.72
+	2.483	1.85	3.60	Q921M4	Golgin subfamily A member 2	Golga2	4233	12	12	12	16.8	16.8	16.8	113.28	0.0000	29.604	6380600	64	15.43	14.68	16.34	15.14	13.47	13.87	13.76	13.09
+	2.046	1.85	3.59	P51829	Adenylate cyclase type 7	Adcy7	1203	8	8	8	11.6	11.6	11.6	122.71	0.0000	65.04	9053400	134	15.06	14.18	15.63	15.13	13.82	12.71	13.77	12.31
+	1.567	1.84	3.58	Q9JK38	Glucosamine 6-phosphate N-acetyltransferase	Gnpnat1	5443	3	3	3	23.4	23.4	23.4	20.791	0.0000	12.001	6672800	44	16.71	16.10	15.24	17.04	15.85	13.90	13.63	14.34
+	1.606	1.84	3.57	Q9CWU2	Palmitoyltransferase ZDHHC13	Zdhhc13	4731	6	6	6	14.6	14.6	14.6	70.889	0.0000	10.063	6727000	41	14.95	14.51	15.79	14.93	12.00	13.06	14.70	13.07
+	2.422	1.83	3.56	Q91VW3	SH3 domain-binding glutamic acid-rich-like protein 3	Sh3bgrl3	4081	7	7	7	79.6	79.6	79.6	10.477	0.0000	90.79	45794000	125	18.50	18.90	19.31	18.40	17.18	17.81	16.48	16.32
+	3.099	1.83	3.55	Q8BJ56	Patatin-like phospholipase domain-containing protein 2	Pnpla2	3072	12	12	12	42.2	42.2	42.2	53.656	0.0000	48.623	15457000	137	16.34	15.48	16.79	16.49	14.21	14.55	14.61	14.42
	1.113	1.83	3.55	Q7TMY7	Importin-8	Ipo8	2753	6	5	5	8.6	7.6	7.6	117.08	0.0000	8.5303	3583000	16	15.07	13.41	16.43	16.42	12.41	13.16	14.61	13.84
	0.419	1.82	3.53	Q7TNS2	MICOS complex subunit Mic10	Minos1	2764	3	3	3	14.5	14.5	14.5	8.5669	0.0002	5.9123	70079000	24	19.19	18.98	17.22	18.92	13.34	19.87	20.12	13.71
+	1.819	1.82	3.52	Q8CI33	CWF19-like protein 1	Cwf19l1	3539	9	9	9	21.8	21.8	21.8	60.193	0.0000	47.316	4772900	59	14.40	14.49	16.31	15.08	12.62	13.07	14.13	13.20

	1.349	1.81	3.51	Q99J45	Nuclear receptor-binding protein	Nrbp1	4324	6	6	6	19.8	19.8	19.8	59.865	0.0000	55.866	12949000	151	15.94	15.32	16.71	16.05	14.95	13.07	15.66	13.10
+	1.946	1.80	3.48	A6H630	Protein-glutamate O-methyltransferase	Armt1	62	3	3	3	7.5	7.5	7.5	50.548	0.0000	12.771	4370800	25	16.51	14.83	15.31	16.28	13.43	13.73	14.81	13.75
+	1.656	1.80	3.48	Q8BGG7	Ubiquitin-associated and SH3 domain-containing protein B	Ubash3b	2982	8	8	8	16.6	16.6	16.6	71.443	0.0000	21.326	6982000	48	15.65	15.47	16.52	15.12	15.28	13.50	12.89	13.89
+	1.866	1.80	3.47	Q14DL3	Leucine-rich repeat and IQ domain-containing protein 3	Lrriq3	1791	3	3	3	6.6	6.6	6.6	75.363	0.0041	2.4409	11538000	58	16.31	16.72	18.09	17.50	15.44	16.05	15.51	14.44
	0.977	1.79	3.46	Q8CE90	Dual specificity mitogen-activated protein kinase kinase 7	Map2k7	3468	6	6	6	17.9	17.9	17.9	59.312	0.0000	21.084	8859500	31	16.60	15.28	17.82	16.49	15.57	13.09	16.52	13.85
+	2.215	1.79	3.46	O35379	Multidrug resistance-associated protein 1	Abcc1	364	49	49	47	40.8	40.8	39.5	171.18	0.0000	323.31	98211000	662	17.02	16.28	16.74	16.51	14.79	15.62	15.23	13.75
	0.938	1.78	3.42	Q6P4T0	Autophagy-related protein 2 homolog A	Atg2a	2565	8	8	8	9	9	9	210.94	0.0000	47.186	5079100	26	15.39	12.56	16.00	15.16	12.62	12.06	14.75	12.57
	0.976	1.76	3.39	P97470	Serine/threonine-protein phosphatase 4 catalytic subunit	Ppp4c	1656	9	7	7	37.5	32.6	32.6	35.08	0.0000	36.433	16823000	60	16.18	16.58	18.63	16.52	16.57	15.18	15.94	13.16
	1.029	1.76	3.39	Q3TAP4	AP-5 complex subunit beta-1	Ap5b1	1814	6	6	6	13.6	13.6	13.6	94.678	0.0000	21.931	7355000	60	16.30	15.69	16.92	15.78	15.72	12.80	15.99	13.14
+	2.654	1.75	3.37	Q99MD9	Nuclear autoantigenic sperm protein	Nasp	4462	7	7	7	12.5	12.5	12.5	83.953	0.0000	28.999	5288600	79	14.61	15.12	15.71	15.58	13.90	13.63	12.82	13.68
	0.988	1.75	3.36	O88441	Metaxin-2	Mtx2	529	12	12	12	63.5	63.5	63.5	29.758	0.0000	323.31	25485000	226	17.06	16.84	16.92	17.09	16.25	12.55	16.41	15.70
	1.415	1.75	3.35	Q99KW3	TRIO and F-actin-binding protein	Triobp	4397	6	5	5	4.4	3.9	3.9	223.37	0.0000	35.215	2543000	32	14.29	12.65	15.75	13.90	12.07	12.20	12.48	12.85
	1.178	1.74	3.34	P68181	cAMP-dependent protein kinase catalytic subunit beta	Prkacb	1514	23	11	11	61	33.9	33.9	40.707	0.0000	95.51	20987000	141	16.54	15.97	17.33	16.95	15.73	13.08	16.37	14.67
+	1.980	1.73	3.31	Q78XF5	Oligosaccharyltransferase complex subunit OSTC	Ostc	2730	3	3	3	18.1	18.1	18.1	16.815	0.0000	17.611	23618000	127	17.00	16.54	17.69	17.33	16.33	14.45	15.73	15.13
	1.421	1.72	3.30	Q8BXQ2	GPI transamidase component PIG-T	Pigt	3289	10	10	10	18	18	18	65.704	0.0000	39.669	5936200	22	15.21	15.32	17.11	14.96	14.82	13.19	14.49	13.23
	1.562	1.72	3.29	Q9CWP6	Motile sperm domain-containing protein 2	Mospd2	4723	16	16	15	28	28	26.4	59.854	0.0000	76.776	16060000	134	16.00	15.87	16.41	16.54	14.44	12.93	15.63	14.94
+	2.663	1.71	3.27	Q9D5V6	Synapse-associated protein 1	Syap1	4987	4	4	4	13.7	13.7	13.7	41.349	0.0000	80.093	3073100	56	14.15	14.07	15.18	14.11	12.75	12.14	13.10	12.68
	1.097	1.71	3.27	Q9CPU4	Microsomal glutathione S-transferase 3	Mgst3	4536	4	4	4	34	34	34	16.958	0.0000	60.109	22417000	150	16.60	16.78	15.69	16.53	15.65	13.30	16.36	13.44
+	2.280	1.71	3.27	Q8N7N5	DDB1- and CUL4-associated factor 8	Dcaf8	3732	5	5	5	9.8	9.8	9.8	66.031	0.0000	39.144	8163400	40	16.79	15.79	16.75	16.87	15.28	14.37	15.46	14.26
	0.873	1.70	3.25	Q9CPT4	Myeloid-derived growth factor	Mydgf	4533	3	3	3	17.5	17.5	17.5	17.982	0.0000	12.312	4551200	18	15.92	12.51	16.42	15.96	12.67	13.72	14.44	13.19
	1.211	1.69	3.23	Q99P65	Equilibrative nucleoside transporter 3	Slc29a3	4501	4	4	4	11.8	11.8	11.8	51.719	0.0000	42.057	14142000	116	16.45	16.41	16.93	16.54	16.14	15.31	15.33	12.78
	0.687	1.69	3.23	A2AH22	Activating molecule in BECN1-regulated autophagy protein 1	Ambra1	22	3	3	3	3.9	3.9	3.9	142.88	0.0000	21.942	9893100	25	16.47	15.69	14.32	16.75	13.44	13.46	17.20	12.35
	1.638	1.69	3.23	Q9D338	39S ribosomal protein L19, mitochondrial	Mrpl19	4958	4	4	3	17.1	17.1	13.7	33.578	0.0000	6.2952	6352900	21	15.88	14.14	15.89	14.97	13.84	13.05	14.42	12.82
	0.565	1.68	3.20	Q69ZF7	Metal transporter CNNM4	Cnnm4	2483	5	5	5	8	8	8	86.625	0.0000	16.584	11176000	39	13.50	15.48	16.71	17.33	15.01	12.70	16.72	11.88
	1.492	1.67	3.18	Q9CY18	Sorting nexin-7	Snx7	4778	5	5	5	20.4	20.4	20.4	45	0.0000	12.623	5166000	42	15.29	15.23	17.46	15.38	13.39	14.38	14.32	14.58
	1.225	1.66	3.17	Q924D0	Reticulon-4-interacting protein 1, mitochondrial	Rtn4ip1	4292	6	6	6	23.5	23.5	23.5	43.371	0.0000	19.67	4650100	35	15.06	14.00	15.83	15.32	13.00	12.26	15.10	13.19
+	1.942	1.65	3.15	Q8C167	Prolyl endopeptidase-like	Prepl	3356	11	11	11	21.4	21.4	21.4	83.193	0.0000	83.775	7243300	106	14.31	14.09	14.94	13.94	11.63	13.60	12.77	12.68
+	4.088	1.65	3.13	P19973	Lymphocyte-specific protein 1	Lsp1	850	17	17	17	78.2	78.2	78.2	36.713	0.0000	323.31	56913000	349	18.50	18.31	19.04	18.71	16.94	16.90	17.23	16.91
+	2.524	1.65	3.13	Q91XU3	Phosphatidylinositol 5-phosphate 4-kinase type-2 gamma	Pip4k2c	4149	23	23	23	67.7	67.7	67.7	47.335	0.0000	133.29	32750000	302	16.29	16.05	17.14	16.47	14.81	14.23	15.46	14.86
	1.542	1.64	3.12	Q8C0L0	Thioredoxin-related transmembrane protein 4	Tmx4	3341	6	6	6	24.8	24.8	24.8	37.131	0.0000	67.716	5416800	64	14.87	13.73	16.16	15.46	13.90	13.10	13.78	12.88
	0.987	1.64	3.12	Q9CPS7	RNA-binding protein PNO1	Pno1	4531	2	2	2	12.9	12.9	12.9	27.453	0.0000	13.962	5849000	57	15.53	15.63	16.27	15.16	12.17	13.36	16.03	14.48
+	2.052	1.64	3.12	Q6PIP5	NudC domain-containing protein 1	Nudcd1	2646	3	3	3	10	10	10	66.704	0.0000	15.13	3141100	29	15.41	14.04	15.67	15.59	14.04	13.62	13.39	13.10
	1.577	1.64	3.11	Q6ZPU9	KIF1-binding protein	Kbp	2684	7	7	7	16.5	16.5	16.5	71.051	0.0000	29.654	13165000	39	17.64	16.15	17.67	17.59	15.84	16.41	15.81	14.44
	1.209	1.63	3.10	Q61263	Sterol O-acyltransferase 1	Soat1	2287	11	11	11	25.2	25.2	25.2	63.798	0.0000	323.31	213280000	490	18.79	18.28	19.49	18.75	18.03	15.32	18.25	17.19
	1.766	1.63	3.09	Q9CS42	Ribose-phosphate pyrophosphokinase 2	Prps2	4697	15	7	7	52.8	29.2	29.2	34.786	0.0000	72.864	13058000	157	15.42	15.46	16.56	15.55	14.77	13.73	14.86	13.13
	0.623	1.63	3.09	Q5DTY9;Q	BTB/POZ domain-containing protein KCTD16;BTB/POZ domain-	Kctd16;Kctd8	2097	2	1	1	4.2	2.6	2.6	48.972	0.0004	4.3652	2707000	27	15.40	12.64	17.41	14.47	11.39	13.93	13.17	14.93
	1.138	1.62	3.08	Q3UDW8	Heparan-alpha-glucosaminide N-acetyltransferase	Hgsnat	1951	5	5	5	9.3	9.3	9.3	72.503	0.0000	71.434	6327600	75	14.99	15.11	16.72	15.50	13.86	14.77	14.98	12.19
	1.500	1.62	3.08	Q9R0E2	Procollagen-lysine,2-oxoglutarate 5-dioxygenase 1	Plod1	5624	12	12	12	23.2	23.2	23.2	83.594	0.0000	57.426	13484000	102	15.74	15.79	15.93	16.41	15.37	13.49	15.25	13.26
+	3.567	1.62	3.08	Q6PFR5	Transformer-2 protein homolog alpha	Tra2a	2629	6	5	5	22.1	18.5	18.5	32.316	0.0000	15.823	12407000	59	17.00	16.93	17.35	17.38	15.33	15.89	15.15	15.80
+	2.718	1.61	3.06	P07742	Ribonucleoside-diphosphate reductase large subunit	Rrm1	672	19	19	19	36.4	36.4	36.4	90.209	0.0000	218.19	13964000	127	15.45	15.47	16.45	16.12	14.06	13.93	14.77	14.27
	1.312	1.61	3.05	P59108	Copine-2	Cpne2	1330	10	9	9	21.7	21.7	21.7	61.035	0.0000	48.17	14027000	86	16.91	16.41	17.48	15.73	15.92	13.49	15.55	15.13
	0.429	1.60	3.02	O88665	Bromodomain-containing protein 7	Brd7	555	3	3	3	7.4	7.4	7.4	73.999	0.0000	9.1874	23905000	13	17.40	17.16	13.67	17.04	11.73	17.24	12.93	16.97
	1.675	1.59	3.02	Q8BKC8	Phosphatidylinositol 4-kinase beta	Pi4kb	3099	4	4	4	6.9	6.9	6.9	91.514	0.0000	11.659	4100500	22	15.22	14.13	15.97	15.64	13.87	12.70	14.11	13.92
+	2.779	1.59	3.02	Q8R5L3	Vam6/Vps39-like protein	Vps39	3892	25	25	25	31	31	31	101.69	0.0000	90.719	26539000	196	16.10	15.55	15.92	16.32	14.72	14.48	14.67	13.66
+	2.153	1.59	3.01	P59326	YTH domain-containing family protein 1	Ythdf1	1335	10	6	4	21.3	15.2	12.7	60.878	0.0000	47.643	3988700	65	14.67	14.01	15.14	14.97	13.10	13.70	13.37	12.27
	1.048	1.59	3.01	Q99PN3	Tripartite motif-containing protein 26	Trim26	4510	8	8	8	22.9	22.9	22.9	62.808	0.0000	36.109	2525100	21	14.30	13.75	16.29	15.40	12.10	13.62	14.69	12.98
	1.923	1.58	3.00	O88668	Protein CREG1	Creg1	557	7	7	7	33.6	33.6	33.6	24.452	0.0000	185.75	128620000	348	19.04	18.75	19.03	19.16	18.57	16.62	16.87	17.57
+	2.220	1.58	3.00	Q3TCN2	Putative phospholipase B-like 2;Putative phospholipase B-like 2	Plbd2	1828	12	12	12	27.9	27.9	27.9	66.289	0.0000	166.15	27345000	212	17.33	17.24	18.31	17.12	16.63	15.35	15.87	15.81
	0.668	1.58	2.99	Q8BWW9	Serine/threonine-protein kinase N2	Pkn2	3269	7	6	6	9.4	8.5	8.5	111.61	0.0000	6.507	14609000	22	16.44	15.76	13.40	16.73	13.50	13.41	16.49	12.60
	1.330	1.58	2.99	Q9CS00	Cactin	Cactin	4696	5	5	5	8.8	8.8	8.8	90.66	0.0000	26.286	8974700	16	15.59	15.66	15.75	16.73	13.40	13.95	16.02	14.04
	1.020	1.58	2.98	E9Q236		Abcc4	229	11	10	10	12	11.5	11.5	148.89	0.0000	22.11	10859000	57	15.65	14.75	16.12	15.80	15.44	12.51	15.11	12.97
	0.999	1.57	2.98	Q64339	Ubiquitin-like protein ISG15	Isg15	2433	4	4	4	26.1	26.1	26.1	17.897	0.0000	73.059	6973500	71	14.67	12.80	14.96	15.65	12.44	11.69	14.02	13.62
	1.667	1.57	2.97	Q9D379	Epoxide hydrolase 1	Ephx1	4961	18	18	18	33	33	33	52.576	0.0000	77.051	22651000	157	15.65	15.37	16.59	16.02	15.09	13.09	14.57	14.61
+	2.890	1.57	2.96	Q9JHZ2	Progressive ankylosis protein	Ankh	5379	1	1	1	4.3	4.3	4.3	54.297	0.0000	6.6917	2344900	32	14.65	14.36	14.98	14.47	13.18	13.00	12.43	13.59
	1.573	1.56	2.96	Q8BGB7	Enolase-phosphatase E1	Enoph1	2974	5	5	5	32.3	32.3	32.3	28.6	0.0000	37.914	4784600	93	14.53	15.40	14.91	14.31	13.53	13.78	13.79	11.79
+	2.466	1.56	2.96	O35887	Calumenin	Calu	409	6	6	6	21	21	21	37.063	0.0000	13.659	4702600	42	16.27	15.76	16.40	16.34	14.76	14.51	13.89	15.34
	1.374	1.56	2.96	C0HKG6;C0HKG5			95	3	3	3	10	10	10	29.608	0.0054	2.2337	5268300	13	16.25	15.04	13.94	16.19	13.11	14.20	13.65	14.21
	1.231	1.56	2.95	E9PZJ8	Activating signal cointegrator 1 complex subunit 3	Ascc3	225	23	23	23	12.5	12.5	12.5	250.55	0.0000	63.882	13589000	101	15.59	15.20	17.21	16.15	14.78	13.08	15.51	14.52
	0.505	1.56	2.95	Q8CIV2	Membralin	Tmem259	3562	3	3	3	6.8	6.8	6.8	63.56	0.0000	6.5267	30502000	25	18.83	18.23	17.56	18.60	18.07	18.13	12.57	18.20
+	2.477	1.56	2.95	Q9D023	Mitochondrial pyruvate carrier 2	Mpc2	4850	4	4	4	30.7	30.7	30.7	14.286	0.0000	14.184	7034600	83	14.86	14.35	15.43	14.99	13.73	13.60	13.43	12.63
	1.038	1.55	2.94	Q8BGB2	Tetratricopeptide repeat protein 7A	Ttc7a	2972	4	4	4	7.1	7.1	7.1	96.155	0.0000	16.308	3907800	28	14.88	12.95	16.36	15.20	12.81	14.19	13.24	12.93
	1.284	1.55	2.93	Q8BGU5	Cyclin-Y	Ccny	2995	3	3	3	12	12	12	39.394	0.0000	14.24	5850200	80	15.15	14.82	16.57	15.34	14.86	13.21	14.74	12.86
	0.971	1.55	2.92	P53808	Phosphatidylcholine transfer protein	Pctp	1239	6	6	6	25.7	25.7	25.7	24.785	0.0000	19.516	14410000	32	17.23	17.27	17.95	16.85	17.27	16.99	14.43	14.42
	1.684	1.54	2.91	Q9WVG6	Histone-arginine methyltransferase CARM1	Carm1	5748	10	10	10	27.1	27.1	27.1	65.853	0.0000	69.757	13793000	163	15.68	14.92	16.25	15.89	14.36	13.58	15.20	13.42
	1.440	1.54	2.91	Q9CXI0	2-methoxy-6-polyprenyl-1,4-benzoquinol methylase, mitochondrial	Coq5	4756	7	7	7	28.7	28.7	28.7	37.335	0.0000	28.001	6556200	46	14.95	13.69	15.85	14.98	12.27	13.78	13.46	13.80
	1.229	1.53	2.89	Q9CQE7	Endoplasmic reticulum-Golgi intermediate compartment protein 3	Ergic3	4590	6	6	6	17	17	17	43.208	0.0000	21.273	4819900	56	15.14	13.40	15.26	15.27	14.33	12.26	12.65	13.69
+	2.890	1.53	2.89	Q3TYS2	Uncharacterized protein C17orf62 homolog		1889	6	6	6	34.2	34.2	34.2	20.921	0.0000	35.977	26278000	132	17.36	17.42	17.14	17.60	16.15	16.12	16.03	15.10
	0.720	1.53	2.89	P70388	DNA repair protein RAD50	Rad50	1563	27	27	27	25.8	25.8	25.8	153.49	0.0000	184.84	17765000	238	15.81	15.41	17.47	15.64	15.06	15.34	15.96	11.83
+	2.742	1.53	2.88	Q9Z0M5	Lysosomal acid lipase/cholesteryl ester hydrolase	Lipa	5776	16	16	16	41.6	41.6	41.6	45.325	0.0000	323.31	121540000	361	16.05	16.32	15.98	15.38	13.97	14.94	14.51	14.20
	1.697	1.53	2.88	O55028	[3-methyl-2-oxobutanoate dehydrogenase [lipoamide]] kinase, mitochon	Bckdk	444	9	9	9	36.7	36.7	36.7	46.587	0.0000	87.174	6868000	55	15.39	14.87	16.31	15.83	14.85	14.59	13.35	13.50
+	2.558	1.52	2.88	Q3V1L4	Cytosolic purine 5-nucleotidase	Nt5c2	2049	24	24	24	52.5	52.5	52.5	64.808	0.0000	199.56	41265000	344	16.91	16.54	17.76	17.12	15.87	15.39	15.84	15.13
	1.422	1.52	2.88	Q9D7X8	Gamma-glutamylcyclotransferase	Ggct	5044	2	2	2	16.5	16.5	16.5	21.166	0.0000	8.1334	2226100	23	14.59	15.31	14.04	14.02	12.44	12.53	14.43	12.47
	1.661	1.52	2.87	P27046	Alpha-mannosidase 2	Man2a1	932	36	36	36	37.5	37.5	37.5	131.63	0.0000	314.53	57148000	557	16.58	16.30	17.73	16.86	15.92	14.36	16.01	15.10
	1.510	1.52	2.86	Q9DBU3	Serine/threonine-protein kinase RIO3	Riok3	5179	6	6	6	18.1	18.1	18.1	58.704	0.0000	15.82	5079100	39	15.54	14.95	15.07	15.53	14.11	12.54	14.98	13.41
+	2.300	1.51	2.85	Q31125	Zinc transporter SLC39A7	Slc39a7	1806	5	5	5	12.8	12.8	12.8	50.656	0.0000	47.57	17731000	92	16.56	16.15	17.37	16.37	15.76	14.95	14.93	14.75
	2.207	1.51	2.85	Q9D6N1	Carbonic anhydrase 13	Ca13	5004	4	4	4	24.4	24.4	24.4	29.522	0.0000	38.532	6242200	86	14.65	14.46	15.41	15.16	13.72	12.79	14.07	13.07
	2.056	1.50	2.83	Q8BTI9	Phosphatidylinositol 4,5-bisphosphate 3-kinase catalytic subunit beta is	Pik3cb	3202	9	9	9	12.3	12.3	12.3	121.71	0.0000	23.569	5994300	76	15.19	14.93	15.18	15.05	13.84	12.77	14.55	13.19
	1.997	1.50	2.83	Q8C3X2	Coiled-coil domain-containing protein 90B, mitochondrial	Ccdc90b	3380	11	11	11	55.9	55.9	55.9	29.596	0.0000	53.888	10354000	86	15.09	14.89	16.16	15.37	13.78	14.43	13.09	14.22
	1.166	1.50	2.83	Q9D938	Transmembrane protein 160	Tmem160	5093	3	3	3	16.5	16.5	16.5	19.587	0.0011	3.6272	5912200	41	15.48	15.89	13.26	15.67	14.02	13.79	13.76	12.73
	0.705	1.49	2.82	P68369;P05	Tubulin alpha-1A chain;Tubulin alpha-3 chain	Tuba1a;Tuba3a	1517	36	4	1	72.7	10.2	3.1	50.135	0.0000	79.259	72237000	138	18.98	18.62	17.27	18.98	18.22	14.15	17.78	17.73
	0.755	1.49	2.81	Q8VEL2	Myotubularin-related protein 14	Mtmr14	4013	6	6	6	15.1	15.1	15.1	72.442	0.0000	50.148	6768700	37	15.27	13.20	16.10	15.81	14.43	12.21	12.57	15.20
	0.485	1.49	2.81	Q99J56	Derlin-1	Derl1	4326	4	4	4	16.3	16.3	16.3	28.834	0.0000	9.4962	25817000	74	17.58	17.13	14.64	17.93	17.13	13.08	17.60	13.51
	1.001	1.49	2.81	Q3UDF0		Slc2a6	1947	11	11	10	17.9	17.9	17.1	54.433	0.0000	151.23	19161000	129	15.08	15.11	16.91	16.23	14.66	12.57	15.47	14.68
	1.897	1.48	2.79	Q99J27	Acetyl-coenzyme A transporter 1	Slc33a1	4320	8	8	8	16.7	16.7	16.7	61.075	0.0000	17.2	5840100	42	15.36	14.83	13.56	14.32	13.31	13.35	12.87	12.62
	0.946	1.48	2.78	Q80TA6	Myotubularin-related protein 12	Mtmr12	2800	13	13	13	24.5	24.5	24.5	85.515	0.0000	37.82	6398200	58	14.66	14.41	16.18	15.37	13.99	13.93	15.04	11.75
	1.566	1.48	2.78	Q08642	Protein-arginine deiminase type-2	Padi2	1765	7	7	7	14.3	14.3	14.3	76.249	0.0000	74.121	62483000	77	20.42	18.83	20.25	19.05	18.83	18.31	18.17	17.35
	0.420	1.47	2.78	P21300	Aldose reductase-related protein 1	Akr1b7	862	8	4	4	21.8	13.9	13.9	35.988	0.0002	6.1491	30290000	80	18.22	18.13	17.43	17.48	18.01	11.73	18.03	17.60
	0.357	1.47	2.78	Q7TSS2	Ubiquitin-conjugating enzyme E2 Q1	Ube2q1	2788	6	6	6	16.8	16.8	16.8	46.172	0.0000	11.81	194050000	35	20.78	19.95	19.71	21.29	21.09	20.46	20.54	13.76
	0.984	1.47	2.77	P70403	Protein CASP	Cux1	1566	16	4	4	33	7.2	7.2	77.268	0.0000	16.889	5447300	15	15.48	15.51	16.67	15.57	12.91	15.32	15.78	13.33
	1.121	1.47	2.77	P35290	Ras-related protein Rab-24	Rab24	1019	7	7	7	43.8	43.8	43.8	23.144	0.0000	33.124	6871500	40	15.01	14.88	15.92	15.28	14.63	11.88	14.24	14.46
	1.066	1.47	2.76	Q8CE96	tRNA (adenine(58)-N(1))-methyltransferase non-catalytic subunit TRM6	Trmt6	3469	7	7	7	19.5	19.5	19.5	55.518	0.0000	29.702	4236000	56	15.11	14.81	13.44	15.23	12.77	13.26	14.73	11.96
	0.908	1.46	2.75	Q8VDG3	Poly(A)-specific ribonuclease PARN	Parn	3951	6	6	6	16	16	16	71.558	0.0000	28.355	3986000	24	13.84	13.34	16.25	14.65	12.82	11.90	13.14	14.38
	1.429	1.46	2.75	P03893	NADH-ubiquinone oxidoreductase chain 2	Mtnd2	629	1	1	1	7.5	7.5	7.5	38.752	0.0000	63.975	3230500	62	15.05	14.94	15.76	15.52	15.25	13.01	13.17	14.00
	1.531	1.45	2.74	O89017	Legumain	Lgmn	595	9	9	9	25.7	25.7	25.7	49.372	0.0000	62.071	10324000	120	15.53	16.01	16.75	16.40	14.47	16.02	14.22	14.17
	1.026	1.45	2.74	Q91W89	Alpha-mannosidase 2C1	Man2c1	4097	15	15	15	18.6	18.6	18.6	115.69	0.0000	151.58	15687000	162	15.65	15.37	16.68	15.79	15.21	14.54	15.43	12.47
	1.457	1.45	2.74	Q8BU88	39S ribosomal protein L22, mitochondrial	Mrpl22	3221	6	6	6	34.5	34.5	34.5	23.805	0.0000	19.286	13226000	64	16.09	16.31	17.99	17.11	15.95	15.18	15.92	14.64
	1.580	1.45	2.72	Q9R1Q9	V-type proton ATPase subunit S1	Atp6ap1	5659	8	8	8	24.2	24.2	24.2	51.007	0.0000	158.06	10674000	118	15.45	15.35	17.01	15.85	14.82	13.87	15.18	14.02
	2.135	1.45	2.72	Q9CQ02	COMM domain-containing protein 4	Commd4	4550	6	6	6	39.2	39.2	39.2	21.86	0.0000	57.309	10833000	61	16.79	15.79	16.31	16.87	15.58	15.29	14.67	14.44
	1.585	1.44	2.72	Q60967	Bifunctional 3-phosphoadenosine 5-phosphosulfate synthase 1;Sulfate	Papss1	2230	14	13	13	32.4	30.1	30.1	70.793	0.0000	118.53	19520000	232	16.06	15.44	17.32	16.28	15.01	13.97	15.03	15.32
	2.194	1.44	2.71	Q91UZ5	Inositol monophosphatase 2	Impa2	4038	7	7	7	37.2	37.2	37.2	31.716	0.0000	119.24	9862900	90	15.69	16.04	17.22	16.24	14.59	14.70	15.12	15.02
	0.667	1.44	2.71	P97412	Lysosomal-trafficking regulator	Lyst	1644	12	12	12	4.9	4.9	4.9	425.28	0.0000	19.243	11570000	33	16.40	15.22	16.42	15.51	15.23	12.67	16.89	13.00
	1.697	1.44	2.71	O88428	Bifunctional 3-phosphoadenosine 5-phosphosulfate synthase 2;Sulfate	Papss2	528	20	20	19	44.1	44.1	41.9	70.35	0.0000	96.804	21169000	217	16.25	15.46	16.66	15.72	15.36	13.98	15.08	13.93
	0.955	1.43	2.70	P70699	Lysosomal alpha-glucosidase	Gaa	1587	19	19	19	26.8	26.8	26.8	106.25	0.0000	194.04	39876000	367	16.58	16.55	16.17	16.51	15.88	12.74	15.64	15.82
	2.419	1.43	2.70	Q6P2B1	Transportin-3	Tnpo3	2556	11	11	11	19.9	19.9	19.9	104.17	0.0000	78.495	7308400	58	16.00	15.90	16.29	16.02	14.65	14.30	15.45	14.08
	0.834	1.43	2.69	Q9JKL4	NADH dehydrogenase [ubiquinone] 1 alpha subcomplex assembly factc	Ndufaf3	5454	3	3	3	30.8	30.8	30.8	20.733	0.0000	34.891	4181300	34	15.56	15.52	13.40	15.96	12.90	13.19	15.57	13.07
	0.921	1.43	2.69	P62342	Selenoprotein T	Selt	1429	6	6	6	22.1	22.1	22.1	22.292	0.0000	9.4545	4085800	24	14.02	13.74	14.23	14.14	11.56	12.83	14.72	11.31
	1.417	1.42	2.68	Q9D6U8	Protein FAM162A	Fam162a	5010	7	7	7	40	40	40	17.725	0.0000	32.45	14397000	64	16.08	15.66	17.65	16.32	14.35	15.45	14.54	15.67
	1.549	1.42	2.68	O08738	Caspase-6;Caspase-6 subunit p18;Caspase-6 subunit p11	Casp6	291	9	9	9	35.5	35.5	35.5	31.595	0.0000	35.788	9659300	110	15.67	15.09	16.19	15.35	14.46	13.85	15.17	13.13

	1.828	1.42	2.68	Q6X893	Choline transporter-like protein 1	Slc44a1	2668	4	4	3	8.4	8.4	6	73.066	0.0013	3.421	6504000	42	15.05	14.67	15.90	15.57	14.33	13.64	14.45	13.08
	0.948	1.42	2.68	O70131	Ninjurin-1	Ninj1	469	2	2	1	31.6	31.6	13.2	16.555	0.0000	98.18	8916400	84	16.24	16.62	17.66	16.79	16.68	13.46	15.32	16.14
	1.223	1.41	2.67	Q8VE97	Serine/arginine-rich splicing factor 4	Srsf4	3995	10	7	7	18.8	15.5	15.5	55.979	0.0000	18.646	7867700	68	15.36	15.51	15.16	15.60	14.11	15.05	12.28	14.53
	2.124	1.41	2.66	Q8BW96	Calcium/calmodulin-dependent protein kinase type 1D	Camk1d	3256	9	7	7	24.2	19	19	42.918	0.0000	29.237	4642200	63	14.79	14.89	15.88	15.27	13.29	13.94	14.45	13.51
	2.435	1.41	2.66	Q9JIS8	Solute carrier family 12 member 4	Slc12a4	5407	7	5	5	9.8	7.5	7.5	120.62	0.0000	16.353	4015200	17	14.68	15.13	15.79	14.63	14.02	13.42	13.75	13.41
	1.973	1.41	2.66	Q80XC2	tRNA (adenine(58)-N(1))-methyltransferase catalytic subunit TRMT61A	Trmt61a	2879	6	6	6	40	40	40	31.639	0.0000	85.958	6244600	81	15.07	14.51	15.48	15.20	13.66	14.48	13.61	12.88
	1.244	1.41	2.66	Q9D8V7	Signal peptidase complex catalytic subunit SEC11C	Sec11c	5078	6	6	6	33.3	33.3	33.3	21.66	0.0000	50.162	4910500	47	14.80	14.34	16.00	14.14	14.18	12.24	13.90	13.33
	1.376	1.40	2.65	P70414	Sodium/calcium exchanger 1	Slc8a1	1569	3	3	3	4.9	4.9	4.9	108.03	0.0000	95.951	1351600	30	14.60	14.56	15.29	13.27	12.98	13.86	13.09	12.17
	1.755	1.40	2.65	Q80SW1	Putative adenosylhomocysteinase 2	Ahcyl1	2795	23	14	13	43.8	27.9	27.9	58.951	0.0000	101.45	19847000	107	17.36	16.15	17.88	17.16	15.70	15.15	16.30	15.78
+	2.837	1.40	2.65	P26323	Friend leukemia integration 1 transcription factor	Fli1	921	7	7	7	23.9	23.9	23.9	51.001	0.0000	42.381	5707900	86	15.00	14.84	15.86	15.08	13.72	14.09	13.80	13.56
	0.989	1.40	2.64	Q5DU25	IQ motif and SEC7 domain-containing protein 2	Iqsec2	2098	9	5	4	7.2	4.5	3.7	161.78	0.0000	10.585	3583900	47	13.97	13.60	16.41	14.95	13.77	14.07	13.07	12.42
	1.078	1.40	2.64	Q80U56	Late secretory pathway protein AVL9 homolog	Avl9	2819	5	5	5	13.6	13.6	13.6	72.185	0.0000	17.335	2994200	36	14.86	13.91	13.56	15.20	11.43	12.94	13.81	13.75
	1.015	1.40	2.64	A2BDX3	Adenylyltransferase and sulfurtransferase MOCS3;Molybdopterin-synth	Mocs3	44	6	6	5	22.4	22.4	17.2	49.374	0.0000	14.235	6951800	48	15.38	15.43	15.96	15.77	15.90	14.13	14.41	12.50
	0.898	1.40	2.64	Q3UJP5	Protein C8orf37 homolog		1985	2	2	2	13.9	13.9	13.9	23.848	0.0000	15.335	4760100	66	14.66	14.26	15.09	15.01	13.04	14.18	14.85	11.34
	0.517	1.40	2.64	Q8BWY9	Protein CIP2A	Kiaa1524	3272	3	3	3	4.4	4.4	4.4	102.04	0.0017	3.265	5708100	16	12.46	15.22	16.19	16.21	12.35	12.47	16.14	13.50
	1.171	1.40	2.64	Q5HZI9	Solute carrier family 25 member 51	Slc25a51	2107	2	2	2	12.1	12.1	12.1	33.689	0.0000	82.491	5032100	50	15.01	14.11	16.80	15.47	13.75	13.27	14.62	14.14
	1.192	1.40	2.63	Q3UHB1	5-nucleotidase domain-containing protein 3	Nt5dc3	1969	7	7	7	19.8	19.8	19.8	63.17	0.0000	15.558	5328800	48	15.15	14.16	14.95	14.77	13.49	12.97	14.87	12.10
	2.533	1.40	2.63	Q9CRD0	OCIA domain-containing protein 1	Ociad1	4692	17	17	17	75.3	75.3	75.3	27.61	0.0000	323.31	76216000	289	18.19	18.17	18.73	17.77	16.43	16.89	17.38	16.56
	1.631	1.39	2.63	P70404	Isocitrate dehydrogenase [NAD] subunit gamma 1, mitochondrial	Idh3g	1567	15	15	15	48.1	48.1	48.1	42.785	0.0000	323.31	47393000	325	16.98	16.70	17.35	17.19	16.21	14.39	16.28	15.77
	1.333	1.39	2.62	Q64010	Adapter molecule crk	Crk	2411	5	5	5	22.7	22.7	22.7	33.814	0.0000	18.451	1927800	17	13.79	15.03	14.09	14.03	11.42	13.56	13.15	13.25
	0.462	1.39	2.62	Q9CXI5	Mesencephalic astrocyte-derived neurotrophic factor	Manf	4757	7	7	7	26.8	26.8	26.8	20.373	0.0000	25.834	14629000	47	16.24	17.30	15.81	15.80	16.73	13.28	17.49	12.09
	2.744	1.39	2.62	P31786	Acyl-CoA-binding protein	Dbi	990	9	9	8	85.1	85.1	83.9	10	0.0000	72.266	29149000	159	17.83	18.12	18.16	18.86	16.52	17.14	16.73	17.03
	2.316	1.39	2.61	P18181	CD48 antigen	Cd48	835	8	8	8	33.8	33.8	33.8	27.383	0.0000	35.306	25680000	198	16.46	16.16	16.84	16.65	15.58	14.31	15.36	15.32
	1.581	1.38	2.60	Q8VCH6	Delta(24)-sterol reductase	Dhcr24	3921	14	14	14	24.4	24.4	24.4	60.112	0.0000	42.376	10752000	75	14.89	14.27	15.52	15.09	13.11	12.70	14.16	14.30
	1.746	1.38	2.60	Q80YV4	Pantothenate kinase 4	Pank4	2909	11	11	11	13.9	13.9	13.9	91.521	0.0000	17.752	7061700	51	15.59	14.42	15.97	15.53	14.51	13.94	14.27	13.28
	0.934	1.38	2.59	Q8VHK9	ATP-dependent RNA helicase DHX36	Dhx36	4021	11	11	11	20.5	20.5	20.5	113.88	0.0000	38.594	6679800	103	15.02	14.79	14.04	14.91	14.74	12.10	14.35	12.06
	2.122	1.37	2.59	Q8CGZ0	Calcium homeostasis endoplasmic reticulum protein	Cherp	3515	11	11	11	16.3	16.3	16.3	106.17	0.0000	105.76	11277000	172	15.23	14.95	16.40	15.45	14.33	13.69	14.24	14.28
	1.332	1.37	2.59	Q3UHH1	Zinc finger SWIM domain-containing protein 8	Zswim8	1972	7	7	7	5.5	5.5	5.5	197.06	0.0000	14.042	3617700	29	14.78	14.15	14.72	14.38	11.61	13.22	13.87	13.83
	1.154	1.37	2.59	Q91VX2	Ubiquitin-associated protein 2	Ubap2	4083	10	10	10	14.6	14.6	14.6	117.96	0.0000	42.435	10849000	77	15.51	15.18	16.62	14.82	14.59	13.99	15.17	12.89
	0.491	1.37	2.59	P28076;O35	Proteasome subunit beta type-9	Psmb9	379	3	3	3	20.1	20.1	20.1	23.396	0.0000	43.504	13137000	63	16.46	16.71	12.83	17.15	16.79	13.69	13.47	13.73
	1.414	1.37	2.58	Q9DCJ5	NADH dehydrogenase [ubiquinone] 1 alpha subcomplex subunit 8	Ndufa8	5217	5	5	5	45.3	45.3	45.3	19.992	0.0000	14.21	7469100	31	15.94	16.43	17.28	16.16	16.12	14.09	15.31	14.81
	1.579	1.36	2.58	P03911	NADH-ubiquinone oxidoreductase chain 4	Mtnd4	630	3	3	3	11.3	11.3	11.3	51.881	0.0000	39.508	4585600	58	15.05	14.52	16.42	15.21	14.45	13.84	13.33	14.13
	0.491	1.36	2.57	Q9CXW2	28S ribosomal protein S22, mitochondrial	Mrps22	4770	6	6	6	17.3	17.3	17.3	41.192	0.0000	7.7285	4951500	14	14.89	11.83	17.01	15.31	13.13	13.18	15.22	12.06
	1.752	1.36	2.57	Q8K2C8	Glycerol-3-phosphate acyltransferase 4	Agpat6	3655	7	7	7	16.4	16.4	16.4	52.181	0.0000	24.818	5592800	82	14.67	13.41	15.37	14.52	13.06	13.43	12.89	13.15
	1.104	1.36	2.57	Q923T9	Calcium/calmodulin-dependent protein kinase type II subunit gamma	Camk2g	4287	11	7	4	27.2	18	13	59.606	0.0000	40.348	9165400	110	15.36	14.99	16.78	15.90	14.76	14.95	15.01	12.87
	0.617	1.36	2.57	Q9JHJ3	Glycosylated lysosomal membrane protein	Glmp	5364	3	3	3	11.9	11.9	11.9	43.803	0.0000	42.36	8188400	43	16.44	13.00	14.82	16.06	15.34	14.42	12.30	12.82
	0.770	1.36	2.56	Q9QZN4	F-box only protein 6	Fbxo6	5602	5	5	5	19	19	19	34.492	0.0000	53.084	8495300	66	16.45	15.00	16.68	16.67	15.72	12.58	15.16	15.91
	1.036	1.35	2.56	Q9D1C8	Vacuolar protein sorting-associated protein 28 homolog	Vps28	4905	10	10	10	62	62	62	25.452	0.0000	69.961	16125000	136	15.86	15.86	16.83	15.66	15.75	12.99	15.49	14.56
	1.019	1.35	2.55	Q8R2K1	Fucose mutarotase	Fuom	3807	3	3	3	33.3	33.3	33.3	16.805	0.0000	22.478	5386500	86	14.86	14.92	16.38	15.27	14.39	12.27	14.71	14.67
	3.169	1.35	2.55	Q9D2V8	Major facilitator superfamily domain-containing protein 10	Mfsd10	4954	4	4	4	11.4	11.4	11.4	49.369	0.0000	15.459	21375000	125	17.11	16.86	17.36	17.14	15.44	15.48	16.18	15.96
	1.406	1.35	2.55	Q8BXA5	Cleft lip and palate transmembrane protein 1-like protein	Clptm1l	3284	2	2	2	5.4	5.4	5.4	62.183	0.0000	9.7294	4963500	60	15.56	14.46	13.89	15.45	14.23	12.75	13.23	13.74
	1.221	1.35	2.55	Q8CIG8	Protein arginine N-methyltransferase 5	Prmt5	3553	13	13	13	25.7	25.7	25.7	72.679	0.0000	64.574	14976000	169	15.68	15.81	16.89	16.02	14.71	13.33	15.24	15.73
	0.930	1.35	2.54	Q8CD26	Solute carrier family 35 member E1	Slc35e1	3456	4	4	4	9.5	9.5	9.5	44.325	0.0000	6.3997	3102600	31	15.52	14.56	14.24	13.29	12.12	13.79	12.03	14.29
	0.859	1.34	2.54	Q9CPY1	39S ribosomal protein L51, mitochondrial	Mrpl51	4545	3	3	3	32.8	32.8	32.8	15.103	0.0000	25.465	12245000	113	15.93	15.38	15.75	16.17	15.55	13.14	16.02	13.14
	1.131	1.34	2.53	Q9D289	Trafficking protein particle complex subunit 6B	Trappc6b	4939	5	5	5	34.8	34.8	34.8	17.936	0.0000	43.742	7520600	73	16.16	16.56	16.24	16.27	16.55	13.76	14.28	15.28
	0.947	1.34	2.53	Q9CZB0	Succinate dehydrogenase cytochrome b560 subunit, mitochondrial	Sdhc	4824	1	1	1	7.1	7.1	7.1	18.382	0.0018	3.1453	1858500	36	14.19	13.44	14.89	14.49	11.50	13.69	12.15	14.30
	1.074	1.34	2.53	Q8BPX9	Solute carrier family 15 member 3	Slc15a3	3169	4	4	4	8	8	8	64.05	0.0000	12.704	6386700	52	15.80	15.66	16.00	15.56	13.66	13.09	15.93	14.97
	1.987	1.33	2.52	Q922L6	Negative elongation factor D	Nelfcd	4263	5	5	5	11.7	11.7	11.7	66.275	0.0000	11.242	4438900	38	14.97	14.27	15.27	14.82	13.92	14.09	13.07	12.91
	1.342	1.33	2.52	Q3TBW2	39S ribosomal protein L10, mitochondrial	Mrpl10	1821	6	6	6	34	34	34	29.395	0.0000	83.871	5035400	50	15.46	14.91	17.02	14.85	14.46	14.07	14.51	13.86
	1.494	1.33	2.52	Q8BHG1	Nardilysin	Nrd1	3036	26	26	26	31.5	31.5	31.5	132.89	0.0000	156.75	29181000	295	16.57	16.21	17.34	16.72	15.70	14.16	16.06	15.59
	1.203	1.33	2.52	Q9ERD8	Gamma-parvin	Parvg	5304	4	4	4	19.9	19.9	19.9	37.603	0.0000	21.543	8186500	72	16.40	15.64	17.29	15.71	15.60	13.73	14.79	15.59
	1.296	1.33	2.52	P30285	Cyclin-dependent kinase 4	Cdk4	976	3	2	2	11.6	8.9	8.9	33.75	0.0000	14.521	1412500	25	13.84	13.33	15.63	14.19	12.59	13.59	12.66	12.83
	0.722	1.33	2.51	Q99MP8	BRCA1-associated protein	Brap	4470	8	8	8	18.4	18.4	18.4	66.99	0.0000	9.255	6860700	24	16.96	17.36	15.71	14.83	14.12	13.39	16.40	15.63
	1.050	1.33	2.51	P51682	C-C chemokine receptor type 5	Ccr5	1200	1	1	1	3.1	3.1	3.1	40.785	0.0070	2.0516	1867100	19	15.38	13.95	16.48	15.80	13.14	14.95	14.32	13.88
	1.581	1.33	2.51	P98192	Dihydroxyacetone phosphate acyltransferase	Gnpat	1688	5	5	5	11.5	11.5	11.5	76.869	0.0000	16.907	4635100	39	14.62	14.15	15.00	15.18	14.37	12.83	13.72	12.70
	0.825	1.32	2.50	Q3TCH7	Cullin-4A	Cul4a	1826	15	15	10	25.3	25.3	18.8	87.752	0.0000	54.122	11488000	95	15.28	15.17	16.28	15.22	14.57	11.93	15.23	14.92
	0.988	1.32	2.50	Q8K0B2	Probable lysosomal cobalamin transporter	Lmbrd1	3600	2	2	2	4.5	4.5	4.5	61.062	0.0000	104.27	11268000	84	15.84	16.03	16.53	15.88	15.61	13.51	16.17	13.70
	1.267	1.32	2.50	Q9Z2D3	Non-syndromic hearing impairment protein 5 homolog	Dfna5	5843	5	5	5	17.6	17.6	17.6	56.63	0.0000	20.258	33252000	58	17.73	18.06	18.47	19.83	17.00	17.05	18.08	16.68
	1.049	1.32	2.49	Q9QXW9	Large neutral amino acids transporter small subunit 2	Slc7a8	5553	3	3	3	8.5	8.5	8.5	57.872	0.0000	25.562	3623400	55	14.58	12.85	15.41	15.26	13.33	13.36	13.74	12.40
	1.329	1.32	2.49	Q9CXU1	Mediator of RNA polymerase II transcription subunit 31	Med31	4767	2	2	2	25.2	25.2	25.2	15.771	0.0000	33.912	1834000	54	13.97	13.15	15.66	14.36	12.86	13.10	13.05	12.86
	1.436	1.31	2.48	Q8BMQ2	General transcription factor 3C polypeptide 4	Gtf3c4	3146	10	10	10	19.2	19.2	19.2	91.612	0.0000	47	5832600	52	14.62	14.19	15.98	14.15	13.56	13.08	14.03	13.03
	0.708	1.31	2.48	Q9QXL1	Kinesin-like protein KIF21B	Kif21b	5545	11	11	11	10.3	10.3	10.3	186.18	0.0000	13.521	8813200	44	14.94	14.82	17.22	15.95	15.29	13.02	15.94	13.45
	1.604	1.31	2.47	Q99K23	Ufm1-specific protease 2	Ufsp2	4360	13	13	13	47.9	47.9	47.9	52.515	0.0000	59.414	14738000	151	15.67	15.13	16.86	16.10	14.73	13.92	15.02	14.86
	1.161	1.30	2.47	Q8BWQ6	UPF0505 protein C16orf62 homolog		3263	18	18	18	24.2	24.2	24.2	109.08	0.0000	102.91	20827000	154	16.64	16.00	17.11	16.93	15.69	13.91	16.48	15.38
	1.327	1.30	2.46	P97300	Neuroplastin	Nptn	1618	5	5	5	16.1	16.1	16.1	44.373	0.0000	7.5884	4532000	32	14.61	14.35	16.23	14.75	14.59	13.42	13.23	13.49
	2.323	1.30	2.46	P15626	Glutathione S-transferase Mu 2	Gstm2	800	17	10	7	69.3	51.8	38.5	25.716	0.0000	22.47	10188000	111	15.42	15.03	14.67	15.11	14.38	13.74	13.78	13.13
	1.352	1.29	2.45	Q8BM72	Heat shock 70 kDa protein 13	Hspa13	3128	7	7	7	20.2	20.2	20.2	51.709	0.0000	25.17	8245100	62	14.99	14.80	15.57	15.32	14.17	13.86	14.89	12.59
	1.084	1.29	2.45	Q99J39	Malonyl-CoA decarboxylase, mitochondrial	Mlycd	4323	3	3	3	10.8	10.8	10.8	54.735	0.0000	22.894	2274300	35	14.72	14.28	14.79	14.13	14.40	12.56	13.97	11.83
	0.388	1.29	2.45	P51908	C->U-editing enzyme APOBEC-1	Apobec1	1209	7	7	7	29.7	29.7	29.7	27.521	0.0000	36.896	28469000	109	16.72	16.83	18.85	17.23	16.96	17.31	18.12	12.07
	1.738	1.28	2.44	Q6A4J8	Ubiquitin carboxyl-terminal hydrolase 7	Usp7	2503	32	32	32	40.4	40.4	40.4	128.47	0.0000	191.46	44414000	432	16.31	15.67	17.23	16.65	15.49	14.62	15.62	15.00
	2.614	1.28	2.43	P21107	Tropomyosin alpha-3 chain	Tpm3	859	17	17	8	47.4	47.4	27.4	32.994	0.0000	304.24	110780000	380	18.87	18.58	19.67	19.16	18.01	17.63	17.93	17.59
	1.308	1.28	2.42	Q8C0P5	Coronin-2A	Coro2a	3346	14	14	13	28.1	28.1	26.7	59.573	0.0000	37.799	43670000	124	18.10	17.36	19.37	19.13	17.08	17.09	16.81	17.87
	1.259	1.28	2.42	P97484	Leukocyte immunoglobulin-like receptor subfamily B member 3	Lilrb3	1661	17	17	17	27.2	27.2	27.2	93.053	0.0000	235.99	29773000	318	16.20	15.64	17.73	16.43	15.47	14.59	15.96	14.88
	1.489	1.28	2.42	Q8C5N3	Pre-mRNA-splicing factor CWC22 homolog	Cwc22	3399	6	6	6	7.5	7.5	7.5	104.77	0.0000	14.662	7189000	57	15.50	14.87	15.91	16.09	14.17	13.80	15.41	13.89
	1.264	1.28	2.42	B2RX12	Canalicular multispecific organic anion transporter 2	Abcc3	82	17	15	15	18.1	16.7	16.7	169.22	0.0000	141.8	21954000	174	16.67	16.32	17.75	16.37	15.33	14.48	16.50	15.69
	2.508	1.28	2.42	O88569	Heterogeneous nuclear ribonucleoproteins A2/B1	Hnrnpa2b1	547	36	36	36	78.8	78.8	78.8	37.402	0.0000	323.31	448760000	1091	19.79	19.93	20.78	20.39	18.69	19.22	18.72	19.16
	1.390	1.28	2.42	Q9QZB9	Dynactin subunit 5	Dctn5	5590	5	5	5	18.7	18.7	18.7	20.14	0.0000	55.262	5526100	47	15.55	14.66	15.24	15.44	14.77	12.81	14.55	13.67
	1.813	1.27	2.42	Q9D0Q7	39S ribosomal protein L45, mitochondrial	Mrpl45	4882	12	12	12	47.7	47.7	47.7	35.41	0.0000	49.605	16056000	124	15.52	15.11	16.42	15.95	14.87	14.13	14.95	13.95
	0.830	1.27	2.42	G5E897		Kdelc2	264	2	2	2	5.8	5.8	5.8	57.685	0.0000	21.409	3620000	31	15.31	15.19	12.89	15.44	12.46	13.66	14.63	12.99
	0.549	1.27	2.42	Q3UBX0	Transmembrane protein 109	Tmem109	1942	3	3	3	11.1	11.1	11.1	26.306	0.0002	5.6541	22993000	58	15.94	16.29	17.76	17.80	16.30	15.70	17.64	13.05
	1.016	1.27	2.41	Q62093	Serine/arginine-rich splicing factor 2	Srsf2	2355	10	10	9	34.8	34.8	34.8	25.476	0.0000	57.496	19025000	89	17.33	17.16	18.59	16.91	16.15	17.04	14.77	16.95
	1.810	1.27	2.41	O89086	RNA-binding protein 3	Rbm3	601	7	7	7	51	51	51	16.604	0.0000	31.93	15096000	102	16.47	16.87	16.01	16.68	15.55	15.96	14.44	15.01
	1.000	1.27	2.41	Q8BPN8	DmX-like protein 2	Dmxl2	3166	8	8	8	4.6	4.6	4.6	338.2	0.0000	27.593	5781700	34	16.44	14.56	16.83	15.24	14.03	14.84	15.42	13.71
	0.516	1.27	2.41	P0DP28;P0DP27;P0DP26			716	7	7	5	80.5	80.5	70.5	16.837	0.0000	264.86	13203000	80	16.99	17.56	14.45	17.28	15.21	16.46	12.87	16.66
	1.710	1.27	2.40	Q8R3H7	Heparan sulfate 2-O-sulfotransferase 1	Hs2st1	3847	3	3	3	12.1	12.1	12.1	41.813	0.0000	16.406	5648700	57	15.19	15.32	15.07	14.99	12.89	13.84	14.82	13.97
	1.069	1.27	2.40	Q80X71	Transmembrane protein 106B	Tmem106b	2871	2	2	2	9.1	9.1	9.1	31.172	0.0002	6.115	5041000	49	15.89	14.32	16.12	16.01	13.80	13.67	15.61	14.21
	1.062	1.26	2.40	Q8VDG5	Phosphopantothenate--cysteine ligase	Ppcs	3952	3	3	3	14.8	14.8	14.8	33.794	0.0000	7.8979	2526900	19	15.16	14.71	13.77	15.19	12.08	14.22	14.30	13.18
	1.493	1.26	2.40	O08585	Clathrin light chain A	Clta	277	5	5	5	20.4	20.4	20.4	25.604	0.0000	18.104	3588300	43	14.74	15.15	15.00	14.49	14.84	12.89	13.36	13.25
	1.424	1.26	2.39	Q78PG9	Coiled-coil domain-containing protein 25	Ccdc25	2727	6	6	6	23.6	23.6	23.6	24.48	0.0000	27.939	6484200	88	15.16	14.42	15.87	14.82	14.31	12.75	13.91	14.25
	1.388	1.26	2.39	Q9DCV4	Regulator of microtubule dynamics protein 1	Rmdn1	5234	5	5	5	20.7	20.7	20.7	35	0.0008	4.0844	3914300	38	15.38	14.53	14.29	15.51	13.57	14.62	12.78	13.70
	0.884	1.26	2.39	Q9ESL4	Mitogen-activated protein kinase kinase kinase MLT	Zak	5340	8	8	8	9.6	9.6	9.6	91.719	0.0000	16.861	5511400	55	14.78	13.67	16.77	14.46	13.62	12.97	13.70	14.36
	1.812	1.26	2.39	Q9D4H8	Cullin-2	Cul2	4978	28	28	28	44	44	44	86.876	0.0000	181.77	42919000	456	15.98	15.46	17.22	16.27	15.10	14.80	15.03	14.99
	1.360	1.25	2.38	Q8BHC4	Dephospho-CoA kinase domain-containing protein	Dcakd	3029	8	8	8	40.7	40.7	40.7	26.476	0.0000	27.211	14093000	93	15.62	15.22	16.16	15.41	14.86	13.89	15.31	13.35
	0.616	1.25	2.38	Q80U70	Polycomb protein Suz12	Suz12	2822	4	4	4	8.1	8.1	8.1	83.025	0.0000	16.602	9233700	24	15.71	15.89	17.96	15.82	16.14	14.79	16.49	12.97
	1.133	1.25	2.37	P63082	V-type proton ATPase 16 kDa proteolipid subunit	Atp6v0c	1487	3	3	3	34.2	34.2	34.2	15.808	0.0000	215.98	223320000	437	19.70	19.88	21.52	20.35	19.78	19.33	19.42	17.93
	0.852	1.25	2.37	P36423	Thromboxane-A synthase	Tbxas1	1041	10	10	10	26.1	26.1	26.1	60.39	0.0000	49.063	13954000	93	15.91	15.69	16.84	15.79	15.34	12.76	15.50	15.64
	1.532	1.24	2.37	P58681	Toll-like receptor 7	Tlr7	1319	26	26	26	28.6	28.6	28.6	121.84	0.0000	251.13	73394000	524	17.21	16.54	18.15	17.38	16.30	15.45	16.76	15.80
	1.821	1.24	2.37	Q9R1J0	Sterol-4-alpha-carboxylate 3-dehydrogenase, decarboxylating	Nsdhl	5651	18	18	18	57.5	57.5	57.5	40.685	0.0000	204.4	54278000	391	16.46	15.95	17.51	16.89	15.09	15.29	15.71	15.75
	0.744	1.24	2.36	Q8BHA3	Probable D-tyrosyl-tRNA(Tyr) deacylase 2	Dtd2	3026	5	5	5	54.8	54.8	54.8	18.236	0.0000	66.409	7863700	94	15.79	15.02	15.37	15.46	14.73	14.88	15.28	11.78
	0.569	1.24	2.36	Q6NVF4	DNA helicase B	Helb	2534	6	6	6	10.8	10.8	10.8	121.47	0.0000	13.057	6188800	58	13.55	15.13	15.81	15.87	12.83	15.04	15.57	11.96
	1.301	1.24	2.36	Q9JKW0	ADP-ribosylation factor-like protein 6-interacting protein 1	Arl6ip1	5461	6	6	6	30.5	30.5	30.5	23.437	0.0000	84.658	28190000	212	16.78	16.49	16.12	16.54	16.26	13.94	15.56	15.23
	0.789	1.23	2.35	Q62086	Serum paraoxonase/arylesterase 2	Pon2	2353	10	10	10	42.7	42.7	42.7	39.617	0.0000	101.37	14071000	111	16.06	15.27	17.28	16.48	15.89	13.32	14.75	16.19
	0.648	1.23	2.35	Q925N0	Sideroflexin-5	Sfxn5	4309	4	4	4	18.7	18.7	18.7	37.328	0.0000	15.383	11960000	46	17.42	15.69	16.89	17.21	16.68	13.13	16.47	15.99
	2.534	1.23	2.35	Q91VM9	Inorganic pyrophosphatase 2, mitochondrial	Ppa2	4069	6	6	6	27	27	27	38.114	0.0000	27.222	6767900	68	15.43	15.17	16.31	15.68	14.62	14.36	14.42	14.26
	1.207	1.23	2.35	Q9CPX7	28S ribosomal protein S16, mitochondrial	Mrps16	4542	4	4	4	43.7	43.7	43.7	15.192	0.0000	30.657	6551600	73	14.65	14.27	16.04	14.89	14.00	12.74	14.54	13.64
	1.437	1.23	2.35	Q8BFV2	PCI domain-containing protein 2	Pcid2	2949	9	9	9	28.8	28.8	28.8	46.132	0.0000	43.356	9425900	71	15.44	14.79	16.85	15.78	14.95	14.36	14.34	14.29
	1.056	1.23	2.35	Q9Z1M0	P2X purinoceptor 7	P2rx7	5812	13	13	13	34.3	34.3	34.3	68.388	0.0000	116.47	19565000	211	15.99	15.85	17.09	16.05	15.60	13.42	15.61	15.42
	0.554	1.23	2.34	Q8R105	Vacuolar protein sorting-associated protein 37C	Vps37c	3772	7	7	7	22.4	22.4	22.4	38.452	0.0000	40.204	22621000	71	17.42	18.13	17.63	17.93	17.61	13.49	17.63	17.47
	0.822	1.23	2.34	Q9CQ49	Nuclear cap-binding protein subunit 2	Ncbp2	4561	4	4	4	26.9	26.9	26.9	18.017	0.0000	8.4879	4440600	28	15.02	14.62	16.47	13.93	14.57	13.41	14.66	12.48
	1.425	1.23	2.34	Q920A5	Retinoid-inducible serine carboxypeptidase	Scpep1	4206	20	20	20	30.5	30.5	30.5	50.964	0.0000	217.27	177470000	509	18.13	18.67	18.48	18.12	17.29	18.20	16.93	16.06
	1.120	1.23	2.34	Q91VK1;Q2I	Basic leucine zipper and W2 domain-containing protein 2	Bzw2	1797	7	6	6	21	19.3	19.3	48.063	0.0000	55.35	7467200	109	14.92	15.17	16.03	14.92	13.89	15.35	14.04	12.87

2.656	1.23	2.34	Q9CRA5	Golgi phosphoprotein 3	Golph3	4685	11	11	10	53.4	53.4	53.4	33.752	0.0000	83.948	13305000	193	15.26	14.93	15.55	15.30	14.40	13.80	14.36	13.57
0.984	1.22	2.34	O70309	Integrin beta-5	Itgb5	485	10	10	10	15.5	15.5	15.5	87.908	0.0000	72.823	25078000	118	16.88	15.95	17.68	16.44	16.23	13.96	15.84	16.01
1.670	1.22	2.34	Q8CC88	von Willebrand factor A domain-containing protein 8	Vwa8	3444	35	35	35	27.6	27.6	27.6	213.42	0.0000	127.9	39382000	233	18.09	17.24	18.56	18.03	17.15	16.56	17.27	16.04
1.865	1.22	2.33	P62482	Voltage-gated potassium channel subunit beta-2	Kcnab2	1430	24	24	21	71.4	71.4	66.2	41.021	0.0000	323.31	131360000	621	18.13	17.82	19.14	18.26	17.29	16.79	17.63	16.75
1.651	1.22	2.33	Q8R127	Saccharopine dehydrogenase-like oxidoreductase	Sccpdh	3774	5	5	5	19.1	19.1	19.1	47.129	0.0000	85.82	4243600	61	14.65	14.75	15.43	14.76	14.72	13.45	13.40	13.12
3.458	1.22	2.33	Q8BG05	Heterogeneous nuclear ribonucleoprotein A3	Hnrnpa3	2956	28	28	28	51.7	51.7	51.7	39.652	0.0000	323.31	265160000	957	19.59	19.66	20.04	20.16	18.81	18.41	18.59	18.76
1.267	1.22	2.33	Q8C4Y3	Negative elongation factor B	Nelfb	3390	10	10	10	25.3	25.3	25.3	65.636	0.0000	66.752	10473000	124	14.79	14.56	16.42	15.01	14.14	13.76	14.69	13.31
1.151	1.22	2.33	Q6P8M1	Putative deoxyribonuclease TATDN1	Tatdn1	2584	5	5	5	19.7	19.7	19.7	33.365	0.0000	7.5268	4054400	35	14.65	14.24	15.18	14.83	14.32	13.53	14.13	12.02
3.811	1.22	2.33	Q9JI08	Bridging integrator 3	Bin3	5380	16	16	16	59.7	59.7	59.7	29.65	0.0000	103.83	24141000	183	15.92	15.97	16.35	15.72	14.74	14.95	14.73	14.66
1.453	1.22	2.33	Q64435	UDP-glucuronosyltransferase 1-6	Ugt1a6	2442	13	4	4	31.3	13.4	13.4	60.438	0.0000	13.465	5339700	53	14.76	14.88	13.38	13.91	13.20	12.35	12.85	13.65
1.394	1.22	2.33	P97480	Eyes absent homolog 3	Eya3	1660	3	3	3	9.2	9.2	9.2	55.973	0.0000	23.959	1061500	20	14.16	12.93	14.73	13.14	12.88	12.45	12.76	12.00
1.367	1.22	2.32	Q99JX4	Eukaryotic translation initiation factor 3 subunit M	Eif3m	4354	16	16	16	55.3	55.3	55.3	42.516	0.0000	291.95	63672000	243	18.79	18.76	19.90	19.04	18.44	16.81	17.85	18.52
1.514	1.21	2.32	Q9JLV5	Cullin-3	Cul3	5491	21	21	21	32.2	32.2	32.2	88.947	0.0000	109.48	24518000	235	16.44	16.07	17.42	16.52	15.69	15.00	16.16	14.74
2.935	1.21	2.32	Q80US4	Actin-related protein 5	Actr5	2836	7	7	7	17.4	17.4	17.4	67.844	0.0000	30.049	3016000	24	14.38	14.45	14.77	14.31	12.74	13.42	13.58	13.33
0.834	1.21	2.31	P48999	Arachidonate 5-lipoxygenase	Alox5	1153	13	13	13	21.5	21.5	21.5	77.966	0.0000	33.542	11349000	121	15.48	15.55	15.32	14.82	14.26	15.01	15.02	12.03
0.809	1.21	2.31	Q80UY2	E3 ubiquitin-protein ligase KCMF1	Kcmf1	2842	5	5	5	18.6	18.6	18.6	41.791	0.0000	172.81	9020500	97	15.81	15.92	16.79	16.44	15.72	12.92	15.56	15.92
0.601	1.21	2.31	Q5SRY7;Q3	F-box/WD repeat-containing protein 11;F-box/WD repeat-containing pro	Fbxw11;Btrc	2134	4	4	4	11.6	11.6	11.6	62.062	0.0000	29.429	3058800	22	14.59	12.74	16.52	15.41	13.09	12.50	14.86	13.97
1.525	1.21	2.31	Q9CQU0	Thioredoxin domain-containing protein 12	Txndc12	4634	3	3	3	29.4	29.4	29.4	19.048	0.0009	3.9211	1370100	19	14.58	13.63	14.91	15.27	13.83	12.73	13.67	13.35
0.769	1.20	2.31	Q8R3I9	UDP-GlcNAc:betaGal beta-1,3-N-acetylglucosaminyltransferase 8	B3gnt8	3849	8	8	8	33.9	33.9	33.9	43.37	0.0000	28.86	7593000	63	15.52	15.41	15.13	15.69	15.62	12.66	15.47	13.18
1.250	1.20	2.30	Q91VA6	Polymerase delta-interacting protein 2	Poldip2	4053	6	6	6	28.8	28.8	28.8	41.87	0.0000	39.505	7040200	85	15.52	15.55	16.95	15.18	15.20	14.46	14.99	13.73
1.877	1.20	2.30	Q9D110	5-formyltetrahydrofolate cyclo-ligase	Mthfs	4892	8	8	8	38.9	38.9	38.9	23.201	0.0000	21.148	7921500	67	14.87	14.31	15.69	14.80	14.11	13.93	13.60	13.22
1.224	1.20	2.30	Q810U5	Coiled-coil domain-containing protein 50	Ccdc50	2932	13	13	13	42.6	42.6	42.6	35.321	0.0000	30.798	6662600	43	14.71	15.39	16.41	15.53	14.80	13.79	15.12	13.54
1.423	1.19	2.28	P10833	Ras-related protein R-Ras	Rras	730	11	11	9	56	56	56	23.764	0.0000	258.97	13122000	153	15.18	14.36	15.97	15.95	13.95	13.63	14.59	14.53
0.440	1.19	2.28	Q9DAU1	Protein canopy homolog 3	Cnpy3	5112	5	5	4	23.6	23.6	14.9	30.537	0.0000	35.578	10172000	23	15.33	17.65	12.69	15.49	15.23	12.53	15.14	13.51
2.436	1.19	2.27	P70658	C-X-C chemokine receptor type 4	Cxcr4	1579	3	3	3	8.6	8.6	8.6	40.426	0.0000	165.6	9197500	106	15.79	15.05	16.23	15.60	14.24	14.51	14.57	14.60
0.700	1.19	2.27	Q8R4U7	Leucine zipper protein 1	Luzp1	3873	13	13	13	14.7	14.7	14.7	119.31	0.0000	40.931	11861000	37	16.59	15.86	15.22	14.75	14.95	15.16	15.28	12.27
2.196	1.18	2.27	Q9ESW8	Pyroglutamyl-peptidase 1	Pgpep1	5349	5	5	5	41.6	41.6	41.6	22.934	0.0000	51.169	6572900	108	15.57	15.23	14.90	15.55	14.83	13.72	14.01	13.95
2.386	1.18	2.27	Q78IS1	Transmembrane emp24 domain-containing protein 3	Tmed3	2724	6	6	6	23.1	23.1	23.1	25.465	0.0000	48.412	11330000	96	16.71	15.96	16.62	16.40	15.33	14.88	15.78	14.97
0.721	1.18	2.27	Q91XL9	Oxysterol-binding protein-related protein 1	Osbpl1a	4148	9	9	9	11.2	11.2	11.2	107.79	0.0000	16.324	8626200	47	15.43	14.69	17.47	15.82	14.94	13.06	15.31	15.36
1.149	1.18	2.27	Q8VDS8	Syntaxin-18	Stx18	3971	10	10	10	32.9	32.9	32.9	38.381	0.0000	38.889	8733600	121	15.32	14.80	17.00	15.76	14.50	14.77	15.06	13.82
1.090	1.18	2.27	Q8BHL8	Proteasome inhibitor PI31 subunit	Psmf1	3047	4	4	4	20.7	20.7	20.7	29.664	0.0000	7.0481	3920200	40	14.89	14.56	14.79	15.71	14.47	13.30	14.81	12.63
1.321	1.18	2.27	Q9ERE3	Serine/threonine-protein kinase Sgk3	Sgk3	5305	7	7	7	20.6	20.6	20.6	57.145	0.0000	36.062	3237100	46	14.16	12.97	15.09	14.08	12.33	12.93	13.23	13.08
2.279	1.18	2.26	Q9CQQ7	ATP synthase F(0) complex subunit B1, mitochondrial	Atp5f1	4622	25	25	25	68.8	68.8	68.8	28.948	0.0000	320.8	470170000	884	20.28	19.86	21.11	20.21	19.26	19.27	19.25	18.95
0.606	1.18	2.26	Q9CZH3	Proteasome assembly chaperone 3	Psmg3	4829	4	4	4	50.8	50.8	50.8	13.331	0.0000	34.994	12456000	85	15.69	14.97	17.29	15.98	15.19	12.52	16.04	15.46
1.488	1.18	2.26	P24638	Lysosomal acid phosphatase	Acp2	901	5	5	5	15.4	15.4	15.4	48.508	0.0000	35.071	10716000	85	16.17	15.69	15.28	15.83	15.60	14.61	13.79	14.27
1.655	1.17	2.26	P55096	ATP-binding cassette sub-family D member 3	Abcd3	1266	11	11	11	19.9	19.9	19.9	75.474	0.0000	126.63	22965000	223	16.03	15.55	17.18	16.18	15.49	14.68	15.17	14.89
1.566	1.17	2.25	Q9D7J6	Deoxyribonuclease-1-like 1	Dnase1l1	5033	5	5	5	19.4	19.4	19.4	35.604	0.0000	56.793	3741400	62	14.58	13.83	15.53	14.42	13.13	13.34	14.00	13.19
0.456	1.17	2.25	Q9D6K8	FUN14 domain-containing protein 2	Fundc2	5000	3	3	3	22.5	22.5	22.5	16.564	0.0000	22.646	6289600	42	12.59	14.98	16.82	15.40	12.72	12.30	15.41	14.67
1.957	1.17	2.25	Q8BTX9	Inactive hydroxysteroid dehydrogenase-like protein 1	Hsdl1	3211	11	11	11	43	43	43	36.867	0.0000	118.28	28130000	170	16.36	16.03	17.02	16.12	15.74	14.64	15.08	15.40
2.211	1.17	2.25	Q91WK2	Eukaryotic translation initiation factor 3 subunit H	Eif3h	4116	23	23	23	68.5	68.5	68.5	39.832	0.0000	323.31	68116000	409	17.67	17.37	18.50	18.32	17.03	16.80	16.56	16.79
1.811	1.16	2.24	Q9D1E8	1-acyl-sn-glycerol-3-phosphate acyltransferase epsilon	Agpat5	4909	7	7	7	20.3	20.3	20.3	42.202	0.0000	23.491	11898000	82	15.52	15.17	16.54	15.32	14.79	14.52	14.02	14.56
2.205	1.16	2.24	E9Q6P5		Ttc7b	243	3	3	3	6.6	6.6	6.6	94.202	0.0000	13.102	1752100	19	14.46	14.04	13.50	13.82	12.24	13.18	12.88	12.87
0.466	1.16	2.24	O09111	NADH dehydrogenase [ubiquinone] 1 beta subcomplex subunit 11, mito	Ndufb11	324	5	5	5	28.5	28.5	28.5	17.444	0.0000	47.051	13132000	58	15.18	15.59	16.00	16.39	15.50	13.70	17.18	12.13
1.287	1.16	2.23	Q8QZV7	Protein asunder homolog	Asun	3736	5	5	5	7.4	7.4	7.4	82.779	0.0002	5.6302	2542800	37	14.42	13.74	14.35	15.82	13.84	13.16	13.65	13.04
0.727	1.16	2.23	P07607	Thymidylate synthase	Tyms	671	3	3	3	14.3	14.3	14.3	34.958	0.0000	52.314	11448000	39	15.06	15.58	15.89	15.29	13.23	12.75	15.72	15.47
1.485	1.16	2.23	P43247	DNA mismatch repair protein Msh2	Msh2	1086	14	14	13	22.1	22.1	21.7	104.15	0.0000	87.573	11141000	110	15.78	15.27	16.38	15.39	15.22	13.81	15.01	14.14
0.474	1.16	2.23	P84084	ADP-ribosylation factor 5	Arf5	1606	12	5	5	67.8	27.8	27.8	20.529	0.0000	42.167	30805000	175	17.64	17.49	18.18	17.26	17.25	13.23	17.80	17.65
0.783	1.16	2.23	Q923G2	DNA-directed RNA polymerases I, II, and III subunit RPABC3	Polr2h	4286	5	5	5	34	34	34	17.143	0.0000	30.977	10403000	78	16.32	16.80	17.00	16.40	16.26	13.33	16.22	16.07
1.121	1.16	2.23	Q8C5H8	NAD kinase 2, mitochondrial	Nadk2	3395	6	6	6	18.4	18.4	18.4	50.858	0.0000	34.163	3999200	38	14.51	13.01	15.58	14.52	13.26	12.94	13.32	13.47
1.521	1.16	2.23	Q9DCU6	39S ribosomal protein L4, mitochondrial	Mrpl4	5233	7	7	7	29.9	29.9	29.9	33.073	0.0000	18.634	8591500	126	15.21	14.74	15.87	15.57	14.76	13.60	14.76	13.63
1.606	1.15	2.23	O35218	Cleavage and polyadenylation specificity factor subunit 2	Cpsf2	347	13	13	13	18.7	18.7	18.7	88.382	0.0000	54.068	9534000	115	15.06	14.18	15.85	15.08	13.88	14.14	14.16	13.37
0.767	1.15	2.23	P50096	Inosine-5-monophosphate dehydrogenase 1	Impdh1	1174	15	13	13	36.6	34.8	34.8	55.278	0.0000	84.897	11945000	130	14.80	13.74	16.95	15.42	13.29	14.61	14.56	13.81
4.781	1.15	2.22	P08226	Apolipoprotein E	Apoe	682	32	32	32	78.5	78.5	78.5	35.866	0.0000	323.31	328940000	943	19.26	19.08	19.13	18.99	17.95	18.17	17.85	17.89
2.393	1.15	2.22	Q8BY71	Histone acetyltransferase type B catalytic subunit	Hat1	3293	7	7	7	23.8	23.8	23.8	49.278	0.0000	28.011	8465800	68	15.93	15.34	15.80	15.81	14.33	14.24	15.21	14.49
0.828	1.15	2.22	A3KGB4	TBC1 domain family member 8B	Tbc1d8b	51	10	10	9	14.7	14.7	13.7	127.89	0.0000	44.41	6949100	90	14.62	13.88	16.93	15.13	13.86	13.42	14.60	14.06
1.222	1.15	2.22	Q8R123	FAD synthase;Molybdenum cofactor biosynthesis protein-like region;FA	Flad1	3773	6	6	6	20.5	20.5	20.5	54.766	0.0000	59.061	11234000	114	15.62	15.21	15.61	15.60	14.72	14.14	15.43	13.13
1.052	1.15	2.22	Q91YN0	Protein C12orf4 homolog	D6Wsu163e	4167	8	8	8	23.7	23.7	23.7	63.643	0.0000	25.24	2463800	26	13.81	13.82	15.49	14.01	13.56	12.20	13.97	12.80
1.498	1.15	2.22	Q3V0C5	Ubiquitin carboxyl-terminal hydrolase 48	Usp48	2048	11	11	11	17.3	17.3	17.3	120.63	0.0000	45.106	5987000	68	14.39	14.32	16.02	14.75	13.58	13.59	13.65	14.07
1.034	1.15	2.22	Q5SUQ9	CST complex subunit CTC1	Ctc1	2147	12	12	12	17.4	17.4	17.4	134.03	0.0000	142.6	11796000	150	15.29	14.72	15.42	15.11	14.81	12.63	14.96	13.54
1.175	1.15	2.21	Q9DBB5	Eukaryotic translation initiation factor 4E type 3	Eif4e3	5139	6	6	6	30	30	30	22.836	0.0000	10.883	4904800	44	15.03	13.69	14.10	15.42	13.00	14.14	13.75	12.76
1.364	1.14	2.21	P24788	Cyclin-dependent kinase 11B	Cdk11b	903	12	12	12	14.5	14.5	14.5	91.512	0.0000	19.195	7655300	62	14.67	14.52	16.22	15.57	14.54	13.78	14.36	13.72
1.858	1.14	2.21	Q62186	Translocon-associated protein subunit delta	Ssr4	2364	9	9	9	45.3	45.3	45.3	18.936	0.0000	120.4	92116000	341	17.97	17.81	17.31	18.17	17.00	15.86	17.06	16.76
1.071	1.14	2.20	Q8CEC6	Peptidylprolyl isomerase domain and WD repeat-containing protein 1	Ppwd1	3471	13	13	13	25.1	25.1	25.1	73.384	0.0000	29.539	7105000	70	14.80	13.64	15.48	14.77	13.53	14.63	12.74	13.23
2.650	1.14	2.20	Q9R1P3	Proteasome subunit beta type-2	Psmb2	5655	17	17	17	52.7	52.7	52.7	22.906	0.0000	98.178	124730000	546	18.34	18.18	18.96	18.60	17.43	16.97	17.64	17.50
1.334	1.13	2.19	Q99J99	3-mercaptopyruvate sulfurtransferase	Mpst	4335	14	14	14	49.2	49.2	49.2	33.097	0.0000	65.726	18430000	132	15.84	15.27	17.30	16.17	15.24	14.77	15.29	14.74
0.842	1.13	2.19	O08663	Methionine aminopeptidase 2	Metap2	284	20	20	19	53.3	53.3	46	52.921	0.0000	200.84	43423000	325	16.84	16.90	17.60	16.78	16.82	14.09	16.83	15.84
0.976	1.13	2.19	O88942	Nuclear factor of activated T-cells, cytoplasmic 1	Nfatc1	586	4	4	4	14.2	14.2	14.2	77.832	0.0000	70.872	2228500	48	13.91	13.17	14.19	14.27	11.20	12.99	13.69	13.12
1.838	1.13	2.19	Q7TQI3	Ubiquitin thioesterase OTUB1	Otub1	2775	14	14	14	67.2	67.2	67.2	31.27	0.0000	242.89	44134000	231	17.27	16.76	18.08	18.05	16.37	16.49	16.19	16.59
0.975	1.13	2.19	Q05860	Formin-1	Fmn1	1743	3	3	3	2.6	2.6	2.6	163.58	0.0000	8.1526	1393100	33	13.09	12.87	15.17	13.83	12.02	13.39	12.47	12.57
1.171	1.13	2.19	Q9D0B6	Protein PBDC1	Pbdc1	4856	6	6	6	36.9	36.9	36.9	22.223	0.0000	10.611	3851200	23	14.99	15.08	16.23	13.83	13.79	13.85	13.70	14.27
1.093	1.13	2.18	Q62048	Astrocytic phosphoprotein PEA-15	Pea15	2349	5	5	5	48.5	48.5	48.5	15.054	0.0000	48.818	4701700	52	14.71	15.69	14.09	15.55	14.00	14.88	13.04	13.62
1.755	1.12	2.18	Q06138	Calcium-binding protein 39	Cab39	1748	18	18	18	60.7	60.7	60.7	39.842	0.0000	67.409	20244000	204	15.73	15.69	17.03	16.05	14.82	15.06	15.40	14.71
0.911	1.12	2.18	Q9D0F6	Replication factor C subunit 5	Rfc5	4864	8	8	8	34.8	34.8	34.8	38.096	0.0000	27.074	4688300	44	13.51	13.98	15.29	13.87	11.68	13.19	14.03	13.25
0.941	1.12	2.18	Q9QYI4	DnaJ homolog subfamily B member 12	Dnajb12	5574	10	10	10	34.3	34.3	34.3	41.987	0.0000	53.79	7056100	74	14.09	13.45	15.65	14.92	12.49	14.04	13.11	13.99
1.675	1.12	2.17	Q8BX94	Oxysterol-binding protein-related protein 2	Osbpl2	3282	9	9	9	31.6	31.6	31.6	55.384	0.0000	58.765	5104800	72	14.04	12.89	13.42	14.10	11.97	12.73	12.29	12.97
1.215	1.12	2.17	Q8BH79	Anoctamin-10	Ano10	3021	5	5	5	8.3	8.3	8.3	76.187	0.0000	48.905	7623200	42	14.57	14.30	16.45	14.98	13.92	13.85	14.22	13.83
0.897	1.12	2.17	Q9D8B4	NADH dehydrogenase [ubiquinone] 1 alpha subcomplex subunit 11	Ndufa11	5058	3	3	3	27	27	27	14.982	0.0000	12.862	2122600	11	14.35	12.48	14.03	15.44	12.90	12.61	12.93	13.38
1.146	1.12	2.17	Q61127	NGFI-A-binding protein 2	Nab2	2255	6	6	5	13.3	13.3	11.6	56.576	0.0000	37.454	3720200	30	14.07	13.64	15.65	15.17	13.48	12.99	14.00	13.58
2.057	1.11	2.16	Q8R4X3	RNA-binding protein 12	Rbm12	3875	5	5	5	6.8	6.8	6.8	102.8	0.0002	6.01	1907100	30	13.87	14.10	14.88	14.77	13.33	13.45	12.85	13.54
2.050	1.11	2.16	Q9R1S8	Calpain-7	Capn7	5662	7	7	7	13.9	13.9	13.9	92.563	0.0000	50.577	6511300	93	15.19	14.79	15.87	15.21	13.74	14.38	14.56	13.94
1.231	1.11	2.16	Q3TIR3	Synembryn-A	Ric8a	1849	12	12	11	29.6	29.6	27	59.846	0.0000	69.271	12337000	74	15.42	15.68	16.20	15.74	14.33	15.16	15.56	13.55
1.765	1.11	2.16	Q3U1J4	DNA damage-binding protein 1	Ddb1	1903	38	38	38	42.1	42.1	42.1	126.85	0.0000	323.31	71964000	726	16.96	16.61	17.92	17.07	16.16	15.50	16.44	16.03
1.302	1.11	2.16	O55137	Acyl-coenzyme A thioesterase 1	Acot1	460	14	4	3	44.9	15.8	12.6	46.135	0.0000	15.05	6507900	54	14.98	14.71	15.76	15.16	14.14	13.57	15.11	13.35
2.683	1.11	2.15	Q8BP40	Lysophosphatidic acid phosphatase type 6	Acp6	3157	14	14	14	34.4	34.4	34.4	47.624	0.0000	116.12	36438000	232	15.77	16.50	16.09	16.60	14.92	15.22	15.34	15.03
0.573	1.10	2.15	Q99KW9	T-cell immunomodulatory protein	Itfg1	4398	2	2	2	6.1	6.1	6.1	67.464	0.0000	12.65	1858000	19	14.75	12.18	16.04	15.21	13.75	14.20	12.57	13.24
0.512	1.10	2.15	Q9DC37	Major facilitator superfamily domain-containing protein 1	Mfsd1	5190	7	7	7	9.9	9.9	9.9	51.396	0.0000	34.816	23438000	90	16.08	16.35	20.32	16.99	16.37	16.11	16.71	16.13
0.789	1.10	2.14	Q61083	Mitogen-activated protein kinase kinase kinase 2	Map3k2	2247	6	3	3	12.8	6.5	6.5	69.573	0.0000	53.481	3256800	51	14.00	13.88	15.78	14.60	13.98	11.85	13.98	14.05
1.703	1.10	2.14	P55302	Alpha-2-macroglobulin receptor-associated protein	Lrpap1	1272	23	23	23	61.4	61.4	61.4	42.215	0.0000	79.455	37290000	279	17.20	17.07	16.43	17.07	15.72	16.42	15.04	16.19
0.593	1.10	2.14	Q80UK0	SEC14 domain and spectrin repeat-containing protein 1	Sestd1	2830	7	7	7	14.2	14.2	14.2	79.375	0.0000	22.659	6206900	61	15.30	14.10	16.68	14.50	14.70	12.16	15.19	14.13
1.266	1.10	2.14	O70503	Very-long-chain 3-oxoacyl-CoA reductase	Hsd17b12	510	22	22	22	63.5	63.5	63.5	34.741	0.0000	323.31	230240000	674	18.49	18.02	20.04	18.99	17.87	17.85	18.08	17.34
1.342	1.10	2.14	Q810S1	Calcium uniporter regulatory subunit MCUb, mitochondrial	Ccdc109b	2931	9	9	9	38.6	38.6	38.6	39.799	0.0000	108.09	14159000	104	15.75	15.44	17.26	16.51	15.05	15.21	15.53	14.77
1.236	1.09	2.14	Q8CI94	Glycogen phosphorylase, brain form	Pygb	3545	59	49	43	68.3	60	53.1	96.729	0.0000	323.31	154950000	737	18.16	17.70	19.60	18.74	17.46	17.43	18.01	16.91
0.734	1.09	2.13	P58044	Isopentenyl-diphosphate Delta-isomerase 1	Idi1	1304	6	6	6	34.4	34.4	34.4	26.289	0.0000	131.06	13356000	115	16.03	15.94	17.33	16.85	15.12	13.75	16.54	16.37
0.725	1.09	2.13	O88851	Putative hydrolase RBBP9	Rbbp9	577	7	7	6	63.4	63.4	60.8	20.911	0.0000	26.017	3492800	32	14.79	12.49	15.37	15.32	12.71	13.30	13.53	14.07
1.012	1.09	2.13	Q91ZR2	Sorting nexin-18	Snx18	4199	8	8	8	16.6	16.6	16.6	67.903	0.0000	47.139	7333100	47	15.64	14.44	15.34	15.92	14.24	13.02	14.52	15.19
1.187	1.09	2.13	Q3UVK0	Endoplasmic reticulum metallopeptidase 1	Ermp1	2038	11	11	11	14.6	14.6	14.6	100.15	0.0000	41.968	16528000	136	15.84	14.97	16.58	15.74	15.10	13.65	15.15	14.87
1.041	1.09	2.13	Q6QLQ4	C-type lectin domain family 7 member A	Clec7a	2652	3	3	3	17.6	17.6	17.6	27.42	0.0000	190.25	10707000	82	16.54	15.73	17.04	16.63	16.18	14.08	15.92	15.40
0.966	1.09	2.12	Q9CQW2	ADP-ribosylation factor-like protein 8B	Arl8b	4643	10	10	5	55.9	55.9	23.7	21.539	0.0000	122.51	91601000	270	18.78	18.26	20.54	18.89	18.45	18.18	17.15	18.34
1.897	1.09	2.12	Q9R0P6	Signal peptidase complex catalytic subunit SEC11A	Sec11a	5634	11	11	11	35.2	35.2	35.2	20.626	0.0000	76.865	64196000	401	18.06	17.51	18.91	18.43	17.23	17.11	17.32	16.91
1.617	1.09	2.12	Q9Z2D1	Myotubularin-related protein 2	Mtmr2	5842	3	3	3	6.5	6.5	6.5	73.231	0.0008	4.1089	1844600	18	13.49	14.25	14.94	13.85	12.79	12.68	13.42	13.31
0.867	1.08	2.12	G5E8P1		Brd1	265	5	5	5	5.8	5.8	5.8	119.71	0.0002	6.1244	5029700	7	15.41	15.15	15.86	15.63	15.18	13.26	15.74	13.53
1.202	1.08	2.12	Q66GT5	Phosphatidylglycerophosphatase and protein-tyrosine phosphatase 1	Ptpmt1	2467	5	5	5	29	29	29	21.942	0.0000	12.405	3790300	38	14.36	13.88	15.68	14.73	14.31	12.97	13.66	13.37
1.024	1.08	2.11	Q924Z4	Ceramide synthase 2	Cers2	4303	7	7	7	12.4	12.4	12.4	45.024	0.0000	76.887	14378000	168	15.47	15.61	16.46	16.20	15.18	13.42	15.66	15.15
2.024	1.08	2.11	Q99LT0	Protein dpy-30 homolog	Dpy30	4446	3	3	3	47.5	47.5	47.5	11.213	0.0000	10.824	3151700	65	14.70	15.09	15.36	15.10	14.18	13.54	13.60	14.61
1.473	1.08	2.11	Q8VDK1	Nitrilase homolog 1	Nit1	3957	8	8	8	40.2	40.2	40.2	35.705	0.0000	96.486	22808000	214	16.59	16.23	16.96	16.54	16.02	15.42	16.06	14.50
1.349	1.08	2.11	Q91YW3	DnaJ homolog subfamily C member 3	Dnajc3	4182	21	21	21	47	47	47	57.463	0.0000	267.65	49862000	309	16.76	16.41	17.50	16.79	16.26	16.05	16.11	14.72
1.230	1.07	2.10	Q91ZV0	Melanoma inhibitory activity protein 2	Mia2	4201	14	14	14	12	12	12	156.46	0.0000	69.504	16240000	90	15.89	15.80	17.18	16.52	15.16	14.47	15.41	16.06
1.313	1.07	2.10	Q8BX02	KN motif and ankyrin repeat domain-containing protein 2	Kank2	3274	6	6	6	9.6	9.6	9.6	90.244	0.0000	39.37	3829500	37	14.18	13.78	14.86	15.23	13.94	12.62	13.54	13.66

1.426	1.07	2.10	Q99J83	Autophagy protein 5	Atg5	4331	11	11	11	30.9	30.9	30.9	32.402	0.0000	110.29	12451000	134	15.53	14.76	16.57	15.77	14.62	14.16	14.68	14.89
0.715	1.07	2.10	P14234	Tyrosine-protein kinase Fgr	Fgr	779	13	13	13	35.6	35.6	35.6	58.866	0.0000	29.851	23017000	104	16.41	15.70	15.35	16.80	15.33	13.13	16.17	15.36
1.911	1.07	2.10	P55937	Golgin subfamily A member 3	Golga3	1274	81	81	81	57.5	57.5	57.5	167.22	0.0000	323.31	90766000	762	15.23	14.92	16.25	15.36	14.38	14.50	14.51	14.09
0.718	1.07	2.10	Q9CWQ0	Diphthine synthase	Dph5	4724	2	2	2	13.2	13.2	13.2	31.218	0.0000	28.25	990200	10	13.33	13.96	15.91	13.88	12.00	13.93	13.88	12.99
0.851	1.07	2.10	Q9DBC3	Cap-specific mRNA (nucleoside-2-O-)-methyltransferase 1	Cmtr1	5142	15	15	15	21.1	21.1	21.1	95.675	0.0000	42.837	14891000	152	14.75	14.71	16.86	15.49	14.39	13.45	15.30	14.41
2.259	1.07	2.09	P12815	Programmed cell death protein 6	Pdcd6	760	10	10	10	72.3	72.3	72.3	21.867	0.0000	232.34	55287000	297	17.36	17.17	18.21	17.33	16.40	16.53	16.65	16.22
1.108	1.07	2.09	Q5SUC9	Protein SCO1 homolog, mitochondrial	Sco1	2144	3	3	3	14.8	14.8	14.8	31.617	0.0000	25.243	8072900	37	15.76	15.23	16.68	15.71	15.17	13.63	15.45	14.86
1.268	1.07	2.09	Q80TV8	CLIP-associating protein 1	Clasp1	2814	15	13	13	15.1	13.2	13.2	169.23	0.0000	36.998	6532700	100	14.71	13.96	15.66	14.92	13.15	13.48	14.42	13.93
1.099	1.06	2.09	P27808	Alpha-1,3-mannosyl-glycoprotein 2-beta-N-acetylglucosaminyltransfera:	Mgat1	941	12	12	12	26.2	26.2	26.2	51.69	0.0000	32.399	9462900	82	15.49	15.27	16.64	15.41	15.00	14.64	15.37	13.54
1.230	1.06	2.09	Q9ER39	Torsin-1A	Tor1a	5293	9	9	9	33	33	33	37.829	0.0000	38.163	6817400	72	14.66	14.73	14.61	15.08	13.97	14.26	14.22	12.39
2.118	1.06	2.09	P51569	Alpha-galactosidase A	Gla	1197	7	7	7	22	22	22	47.642	0.0000	69.169	82181000	370	18.35	17.80	18.79	18.14	17.65	16.93	17.33	16.95
2.534	1.06	2.09	P35821	Tyrosine-protein phosphatase non-receptor type 1	Ptpn1	1033	24	24	24	63	63	63	49.593	0.0000	230.65	77658000	405	17.67	17.08	18.06	17.86	16.64	16.76	16.54	16.49
1.178	1.06	2.09	Q6P9N1	Hyccin	Fam126a	2588	7	7	7	25.5	25.5	25.5	57.321	0.0000	38.245	7042300	94	15.35	15.19	16.36	15.25	14.35	13.85	15.58	14.12
1.921	1.06	2.08	O35250	Exocyst complex component 7	Exoc7	351	35	35	35	61.1	61.1	61.1	79.959	0.0000	204.76	41200000	466	16.08	15.50	16.83	15.91	15.25	14.90	15.15	14.78
1.757	1.06	2.08	Q9Z0W3	Nuclear pore complex protein Nup160	Nup160	5790	18	18	18	21.7	21.7	21.7	158.23	0.0000	142.29	15316000	152	15.93	15.60	16.88	16.11	15.12	14.72	14.90	15.54
0.555	1.06	2.08	Q9CXV1	Succinate dehydrogenase [ubiquinone] cytochrome b small subunit, mit	Sdhd	4769	3	3	3	11.3	11.3	11.3	17.014	0.0000	10.838	9112200	20	16.34	15.83	14.96	13.01	14.43	14.85	12.55	14.08
2.303	1.06	2.08	P61202	COP9 signalosome complex subunit 2	Cops2	1375	21	21	21	63	63	63	51.596	0.0000	323.31	55993000	479	16.92	16.53	16.96	17.10	15.99	15.49	16.34	15.46
0.776	1.05	2.08	Q499X9	Methionine--tRNA ligase, mitochondrial	Mars2	2056	6	6	6	15	15	15	65.804	0.0000	28.539	2882600	52	13.93	12.72	14.66	13.80	11.84	11.79	13.91	13.35
1.054	1.05	2.08	Q5SRX1	TOM1-like protein 2	Tom1l2	2133	4	4	4	17.6	17.6	17.6	55.662	0.0000	11.799	1342500	17	12.94	13.23	14.81	13.19	13.28	11.87	12.32	12.48
3.295	1.05	2.08	Q8BL97	Serine/arginine-rich splicing factor 7	Srsf7	3114	7	6	6	27	27	27	30.817	0.0000	70.904	48394000	178	17.79	17.82	17.82	18.28	16.86	17.06	16.60	16.98
1.005	1.05	2.07	Q921I9	Exosome complex component RRP41	Exosc4	4228	7	7	7	39.2	39.2	39.2	26.249	0.0000	182.34	11028000	130	15.74	15.93	15.82	15.98	15.56	13.58	14.27	15.85
0.643	1.05	2.07	Q8C3X8	Lipase maturation factor 2	Lmf2	3382	8	8	8	13	13	13	79.996	0.0000	34.626	9307800	43	16.07	14.53	18.21	16.77	15.32	14.96	15.76	15.33
0.750	1.05	2.07	P42337	Phosphatidylinositol 4,5-bisphosphate 3-kinase catalytic subunit alpha is	Pik3ca	1079	5	5	5	6.9	6.9	6.9	124.41	0.0000	8.9222	4675800	23	15.21	13.51	16.53	14.78	13.54	13.94	14.81	13.53
0.577	1.05	2.07	Q9EQ80	NIF3-like protein 1	Nif3l1	5273	6	6	6	23.9	23.9	23.9	41.745	0.0000	33.682	10578000	127	15.42	15.61	16.39	15.73	15.24	16.15	15.22	12.34
1.376	1.05	2.07	Q9CV28	Protein FAM188A	Fam188a	4705	2	2	2	6.8	6.8	6.8	49.612	0.0000	47.706	3977500	85	14.90	14.59	14.34	15.04	14.52	13.66	13.80	12.70
1.579	1.05	2.07	Q99LP6	GrpE protein homolog 1, mitochondrial	Grpel1	4441	10	10	10	50.2	50.2	50.2	24.307	0.0000	129.88	9478100	120	15.54	15.73	16.39	15.99	15.43	14.46	14.22	15.35
1.087	1.05	2.06	Q3TYX3	SET and MYND domain-containing protein 5	Smyd5	1890	5	5	5	13.2	13.2	13.2	47.095	0.0000	8.4097	5278000	68	14.82	14.71	16.11	15.29	14.63	14.05	14.92	13.17
2.229	1.04	2.06	Q9ES97	Reticulon-3	Rtn3	5333	5	5	5	7.7	7.7	7.7	103.88	0.0000	198.57	65293000	239	17.86	18.00	18.67	18.57	17.14	17.27	17.62	16.90
0.987	1.04	2.06	Q8BJW5	Nucleolar protein 11	Nol11	3088	5	5	5	7.2	7.2	7.2	80.817	0.0000	7.3087	2051300	32	13.21	14.20	14.87	14.22	14.12	13.39	12.26	12.56
1.519	1.04	2.06	Q8K2M0	39S ribosomal protein L38, mitochondrial	Mrpl38	3666	7	7	6	17.9	17.9	16.1	45.029	0.0004	4.747	5963100	48	15.39	15.00	15.03	15.17	14.91	13.41	13.60	14.51
2.785	1.04	2.06	Q6PDM2	Serine/arginine-rich splicing factor 1	Srsf1	2618	19	19	18	58.1	58.1	56	27.744	0.0000	102.96	59799000	220	17.39	17.75	17.52	17.90	16.61	16.93	16.18	16.67
0.746	1.04	2.06	Q8BZM1	Glomulin	Glmn	3315	3	3	2	3.9	3.9	3.9	67.755	0.0000	25.165	3366700	51	14.80	15.17	16.28	14.62	15.28	12.87	14.99	13.55
1.727	1.04	2.06	P55264	Adenosine kinase	Adk	1271	18	18	18	53.5	53.5	53.5	40.148	0.0000	115.62	50008000	339	16.64	16.47	16.48	16.89	15.96	14.66	15.96	15.73
1.537	1.04	2.06	P97333	Neuropilin-1	Nrp1	1624	16	16	16	25.6	25.6	25.6	103	0.0000	209.55	23227000	189	17.05	16.39	17.58	17.34	16.17	15.78	16.72	15.53
0.904	1.04	2.05	Q61037	Tuberin	Tsc2	2242	6	6	6	5.8	5.8	5.8	202.07	0.0000	53.43	5011500	67	15.02	14.20	15.96	14.66	14.24	14.02	14.76	12.66
0.821	1.04	2.05	Q5SVR0	TBC1 domain family member 9B	Tbc1d9b	2152	13	13	12	12.7	12.7	12	141.78	0.0000	52.343	8591000	88	14.85	13.93	16.06	14.88	14.53	14.07	14.41	12.55
1.110	1.04	2.05	Q91WC9	Sn1-specific diacylglycerol lipase beta	Daglb	4104	14	14	14	26.9	26.9	26.9	73.904	0.0000	143.86	43188000	230	17.10	16.52	16.68	17.27	16.57	15.07	16.72	15.06
0.386	1.04	2.05	P54731	FAS-associated factor 1	Faf1	1255	4	4	4	8.3	8.3	8.3	73.862	0.0000	18.43	7765800	51	15.77	14.22	15.65	16.40	14.75	11.34	15.79	16.01
0.179	1.04	2.05	P35492	Histidine ammonia-lyase	Hal	1022	44	44	44	68	68	68	72.257	0.0000	323.31	146750000	393	10.56	15.59	7.74	5.89	9.23	11.02	7.85	7.54
0.622	1.04	2.05	P54818	Galactocerebrosidase	Galc	1259	7	7	7	15.2	15.2	15.2	77.256	0.0000	57.317	18472000	155	16.77	16.48	16.54	16.71	16.69	13.25	16.24	16.18
1.769	1.04	2.05	Q62393	Tumor protein D52	Tpd52	2385	10	10	10	58.9	58.9	58.9	24.313	0.0000	193.86	23286000	216	16.11	15.99	17.14	16.18	15.09	15.46	14.99	15.75
2.275	1.03	2.05	P29758	Ornithine aminotransferase, mitochondrial	Oat	972	34	34	34	78.6	78.6	78.6	48.354	0.0000	323.31	155750000	771	18.26	17.72	18.81	18.40	17.52	17.22	17.23	17.09
1.368	1.03	2.05	Q99KR8	Plasma alpha-L-fucosidase	Fuca2	4393	5	5	5	14.8	14.8	14.8	53.645	0.0000	15.159	5111500	50	14.51	15.37	15.85	15.81	14.98	13.80	14.52	14.11
0.457	1.03	2.05	Q5SS00	DBF4-type zinc finger-containing protein 2 homolog	Zdbf2	2135	2	2	2	0.7	0.7	0.7	273.74	0.0088	1.8804	16116000	37	16.32	15.94	16.64	13.40	16.64	13.85	13.72	13.96
0.875	1.03	2.05	Q3UNZ8	Quinone oxidoreductase-like protein 2		2011	6	6	6	36.6	36.6	36.6	37.808	0.0000	48.516	3315800	48	14.67	13.66	16.14	13.96	13.51	13.79	14.02	13.00
0.564	1.03	2.04	Q9QYC7	Vitamin K-dependent gamma-carboxylase	Ggcx	5569	5	5	5	9.2	9.2	9.2	87.194	0.0000	14.203	6240000	58	14.19	13.80	16.57	14.15	12.34	15.10	13.77	13.38
1.618	1.03	2.04	Q3UM29	Conserved oligomeric Golgi complex subunit 7	Cog7	1996	15	15	15	29.6	29.6	29.6	86.072	0.0000	147.43	13972000	189	15.25	14.99	15.56	15.20	14.77	14.76	13.86	13.50
1.864	1.03	2.04	Q8R3S6	Exocyst complex component 1	Exoc1	3857	26	26	26	41.9	41.9	41.9	101.89	0.0000	246.09	27758000	249	16.41	15.95	17.32	16.60	15.69	15.47	15.68	15.32
1.771	1.03	2.04	Q9JHR7	Insulin-degrading enzyme	Ide	5370	36	36	36	37.7	37.7	37.7	117.77	0.0000	219.47	53079000	469	16.38	16.27	17.62	16.77	15.54	15.85	15.73	15.80
0.725	1.03	2.04	O35904	Phosphatidylinositol 4,5-bisphosphate 3-kinase catalytic subunit delta is	Pik3cd	412	12	12	12	16.8	16.8	16.8	119.71	0.0000	64.367	5738600	63	14.41	13.44	15.04	15.30	12.83	12.33	14.60	14.31
0.703	1.02	2.03	O08599	Syntaxin-binding protein 1	Stxbp1	279	15	15	15	33.2	33.2	33.2	67.568	0.0000	71.485	16453000	151	15.59	14.25	17.12	16.03	14.47	13.76	15.16	15.50
0.850	1.02	2.03	Q05921	2-5A-dependent ribonuclease	Rnasel	1745	6	6	6	11.6	11.6	11.6	83.274	0.0000	17.385	5245300	51	14.56	13.23	15.43	14.83	14.34	12.59	13.86	13.19
0.617	1.02	2.03	P97760	DNA-directed RNA polymerase II subunit RPB3	Polr2c	1667	3	3	3	17.8	17.8	17.8	31.443	0.0000	14.896	6315700	59	14.89	15.41	14.18	15.46	14.83	12.53	15.55	12.94
1.200	1.02	2.02	Q8VC60	Beta-galactosidase-1-like protein	Glb1l	3910	3	2	2	6.7	4.8	4.8	73.278	0.0000	12.342	2709600	21	14.51	13.96	14.75	14.84	12.76	13.65	13.02	14.56
1.920	1.02	2.02	Q8R016	Bleomycin hydrolase	Blmh	3744	30	30	30	63.7	63.7	63.7	52.511	0.0000	323.31	110870000	611	17.42	17.53	18.45	17.59	16.66	16.38	17.16	16.73
1.350	1.02	2.02	Q8R001	Microtubule-associated protein RP/EB family member 2	Mapre2	3740	18	18	17	56.7	56.7	53.7	36.946	0.0000	175.28	37072000	304	16.65	16.06	17.68	16.97	15.80	15.70	16.43	15.38
2.350	1.02	2.02	Q9CXY9	GPI-anchor transamidase	Pigk	4775	11	11	11	45.3	45.3	45.3	44.895	0.0000	58.238	14446000	112	15.82	15.77	15.95	15.77	15.36	14.89	14.26	14.74
1.272	1.02	2.02	Q921Q3	Chitobiosyldiphosphodolichol beta-mannosyltransferase	Alg1	4237	5	5	5	12	12	12	54.417	0.0000	28.675	5160500	52	14.41	13.66	15.19	14.72	13.73	13.63	13.90	12.67
1.268	1.01	2.02	Q9CQT1	Methylthioribose-1-phosphate isomerase	Mri1	4632	11	11	11	50.7	50.7	50.7	39.41	0.0000	131.05	23505000	139	16.64	15.83	17.66	16.76	15.27	16.17	15.56	15.85
1.413	1.01	2.01	P70698	CTP synthase 1	Ctps1	1586	19	17	17	39.9	39.9	39.9	66.682	0.0000	92.879	26101000	201	16.68	16.35	17.93	16.96	15.80	15.75	16.49	15.83
1.652	1.01	2.01	P40240	CD9 antigen	Cd9	1060	4	4	4	10.2	10.2	10.2	25.258	0.0000	17.691	40963000	112	18.03	17.67	18.15	17.77	16.09	17.58	16.86	17.06
1.288	1.01	2.01	Q62192	CD180 antigen	Cd180	2367	17	17	17	30.1	30.1	30.1	74.302	0.0000	219.1	82930000	585	17.38	17.11	18.01	17.70	16.93	15.53	17.20	16.52
2.388	1.01	2.01	Q6PB66	Leucine-rich PPR motif-containing protein, mitochondrial	Lrpprc	2602	65	65	65	53.7	53.7	53.7	156.61	0.0000	323.31	166370000	1158	17.54	17.29	18.29	17.62	16.78	16.63	16.79	16.50
1.169	1.00	2.01	O89112	LanC-like protein 1	Lancl1	606	10	10	10	27.3	27.3	27.3	45.341	0.0000	16.399	7331700	68	15.24	15.10	16.97	15.35	14.64	14.96	14.62	14.41
1.427	1.00	2.01	Q9DAW6	U4/U6 small nuclear ribonucleoprotein Prp4	Prpf4	5114	20	20	20	44.7	44.7	44.7	58.369	0.0000	63.004	29572000	285	15.63	15.26	16.23	16.01	14.98	13.86	15.25	15.02
0.718	1.00	2.00	Q8K358	Phosphatidylinositol glycan anchor biosynthesis class U protein	Pigu	3691	3	3	3	6.7	6.7	6.7	49.804	0.0000	44.154	5597300	53	13.46	15.20	15.93	16.04	14.64	13.43	14.78	13.77
0.928	1.00	2.00	Q2NL51	Glycogen synthase kinase-3 alpha	Gsk3a	1799	12	12	9	48.6	48.6	40.6	51.66	0.0000	115.26	11215000	105	14.90	14.90	16.89	15.06	14.36	14.46	15.08	13.83
0.780	1.00	2.00	Q9CXJ4	ATP-binding cassette sub-family B member 8, mitochondrial	Abcb8	4758	6	6	6	13.7	13.7	13.7	77.999	0.0000	13.693	2482400	31	14.50	13.10	15.75	14.42	13.74	14.05	13.43	12.53
1.108	1.00	2.00	Q9DAI2	Intraflagellar transport protein 22 homolog	Ift22	5107	2	2	2	22.7	22.7	22.7	20.833	0.0000	12.262	2274400	30	14.30	14.54	14.79	14.35	13.47	13.08	14.78	12.66
3.298	1.00	2.00	P62996	Transformer-2 protein homolog beta	Tra2b	1476	11	11	10	38.5	38.5	35.1	33.665	0.0000	70.253	61353000	237	17.64	17.94	17.84	18.26	16.78	17.11	16.84	16.96
2.062	1.00	2.00	Q8R5H1	Ubiquitin carboxyl-terminal hydrolase 15	Usp15	3889	35	35	34	44.1	44.1	43.2	112.32	0.0000	283.76	45331000	424	16.74	16.28	17.29	16.95	15.88	15.37	16.07	15.95
0.799	1.00	2.00	Q3URQ0	Testis-expressed sequence 10 protein	Tex10	2024	5	5	5	9.7	9.7	9.7	105.21	0.0000	27.785	2813500	35	13.18	14.01	15.61	14.43	13.19	12.94	14.35	12.74
1.183	1.00	2.00	Q3UYV9	Nuclear cap-binding protein subunit 1	Ncbp1	2045	7	7	7	11.1	11.1	11.1	91.926	0.0000	82.179	10920000	127	15.20	14.94	16.50	15.82	14.69	14.06	15.34	14.38
0.994	0.99	1.99	Q8K0Z7	Translational activator of cytochrome c oxidase 1	Taco1	3611	8	8	8	45.9	45.9	45.9	32.314	0.0000	47.827	11367000	94	15.69	14.80	15.95	15.92	14.14	13.61	15.55	15.08
1.440	0.99	1.99	P35285	Ras-related protein Rab-22A	Rab22a	1017	7	5	5	55.2	43.8	43.8	21.802	0.0000	110.07	11240000	90	16.21	15.65	15.97	16.49	15.13	14.25	15.14	15.83
1.187	0.99	1.99	Q8BFY6	Peflin	Pef1	2952	8	8	8	38.2	38.2	38.2	29.227	0.0000	194.48	19386000	198	16.35	16.13	15.03	16.60	15.58	14.34	15.35	14.87
0.792	0.99	1.99	Q9Z280	Phospholipase D1	Pld1	5834	14	14	14	15.3	15.3	15.3	123.97	0.0000	54.275	70289000	118	19.55	19.23	21.69	19.68	19.07	19.35	19.48	18.27
0.306	0.99	1.99	P68373	Tubulin alpha-1C chain	Tuba1c	1519	36	3	3	72.6	11.4	11.4	49.909	0.0000	137.79	27565000	160	17.20	17.59	13.79	17.24	17.29	15.28	16.65	12.63
1.485	0.99	1.98	P28659	CUGBP Elav-like family member 1	Celf1	956	11	9	9	27.8	23.5	23.5	52.107	0.0000	65.313	10463000	122	15.44	14.76	16.46	15.53	14.53	14.39	14.76	14.55
0.648	0.99	1.98	O08586	Phosphatidylinositol 3,4,5-trisphosphate 3-phosphatase and dual-specif	Pten	278	2	2	2	5.5	5.5	5.5	47.152	0.0018	3.2251	1787600	19	12.96	13.69	14.72	13.91	13.68	12.22	14.07	11.35
0.561	0.98	1.98	Q8R2Q4	Ribosome-releasing factor 2, mitochondrial	Gfm2	3813	15	15	15	25.2	25.2	25.2	86.108	0.0000	49.549	10580000	110	15.01	14.97	16.27	15.11	15.21	15.04	15.09	12.08
0.782	0.98	1.98	Q9Z2D0	Myotubularin-related protein 9	Mtmr9	5841	15	15	15	32.5	32.5	32.5	62.907	0.0000	123.9	9800800	116	14.52	14.13	16.01	15.15	14.33	13.66	15.05	12.85
1.358	0.98	1.98	Q3UV70	[Pyruvate dehydrogenase [acetyl-transferring]]-phosphatase 1, mitochoı	Pdp1	2036	11	11	11	33.8	33.8	33.8	61.18	0.0000	149.6	10495000	111	14.53	14.49	13.83	15.26	13.07	13.51	14.25	13.35
1.772	0.98	1.98	Q9DCA2	28S ribosomal protein S11, mitochondrial	Mrps11	5202	4	4	4	35.1	35.1	35.1	20.208	0.0000	79.294	3413800	51	15.01	14.49	15.67	15.07	14.00	14.26	14.44	13.61
0.598	0.98	1.97	P01027	Complement C3;Complement C3 beta chain;C3-beta-c;Complement C3	C3	617	23	22	22	20.4	20	20	186.48	0.0000	76.043	17835000	93	16.16	14.91	17.84	17.42	16.28	14.53	16.15	15.43
0.371	0.98	1.97	Q60648	Ganglioside GM2 activator	Gm2a	2188	3	3	3	24.4	24.4	24.4	20.824	0.0000	28.955	28250000	54	18.57	17.51	18.33	18.68	18.54	13.94	18.42	18.26
1.104	0.98	1.97	Q8R0A7	Uncharacterized protein KIAA0513	Kiaa0513	3754	6	6	6	22.9	22.9	22.9	46.318	0.0000	16.379	3746200	61	14.50	13.86	15.84	14.32	13.71	13.42	14.14	13.34
1.108	0.98	1.97	Q9R0H0	Peroxisomal acyl-coenzyme A oxidase 1	Acox1	5626	20	20	20	42.8	42.8	42.8	74.648	0.0000	90.093	15483000	120	15.66	15.61	15.40	15.51	15.13	14.37	15.40	13.37
0.634	0.98	1.97	O54885	TYRO protein tyrosine kinase-binding protein	Tyrobp	430	2	2	2	25.4	25.4	25.4	12.367	0.0000	56.103	4382600	26	15.06	13.61	13.55	15.98	12.29	13.81	13.95	14.24
1.857	0.97	1.96	Q8VEE4	Replication protein A 70 kDa DNA-binding subunit;Replication protein A	Rpa1	4002	16	16	16	28.9	28.9	28.9	69.036	0.0000	111.83	23353000	240	15.82	15.61	16.31	16.43	14.77	14.85	15.67	14.98
1.320	0.97	1.96	Q8R323	Replication factor C subunit 3	Rfc3	3832	9	9	9	30.1	30.1	30.1	40.526	0.0000	67.985	8432500	110	15.10	14.36	16.03	15.54	14.01	14.22	14.77	14.12
0.728	0.97	1.96	Q8R5K4	Nucleolar protein 6	Nol6	3891	7	7	7	8.2	8.2	8.2	129.23	0.0000	29.002	3484000	41	14.17	12.82	15.44	14.20	12.64	14.09	12.49	13.53
0.376	0.97	1.96	P25425;P31	POU domain, class 2, transcription factor 1;POU domain, class 2, trans	Pou2f1;Pou2f3;Po	906	3	3	3	6.1	6.1	6.1	79.546	0.0000	38.233	9401900	49	15.85	15.91	14.67	16.68	12.41	16.11	16.95	13.75
2.001	0.97	1.96	Q3UMR5	Calcium uniporter protein, mitochondrial	Mcu	2001	14	14	14	40	40	40	39.681	0.0000	109.93	24927000	189	16.05	15.65	16.66	16.19	15.12	14.80	15.58	15.18
1.490	0.97	1.96	Q3UKC1	Tax1-binding protein 1 homolog	Tax1bp1	1987	6	6	6	8.5	8.5	8.5	93.629	0.0000	21.07	4571000	47	15.09	14.49	14.62	14.77	13.89	14.13	14.25	12.82
1.305	0.97	1.96	P40237	CD82 antigen	Cd82	1059	2	2	2	4.5	4.5	4.5	29.628	0.0002	5.3776	4971700	48	15.36	14.36	16.01	14.91	13.70	14.52	14.14	14.41
2.292	0.97	1.96	Q64514	Tripeptidyl-peptidase 2	Tpp2	2449	84	84	84	63.8	63.8	63.8	139.88	0.0000	323.31	581750000	2091	19.28	19.05	20.02	19.39	18.35	18.31	18.70	18.49
0.996	0.97	1.96	Q80TM9	Nischarin	Nisch	2808	27	27	27	23.1	23.1	23.1	175.01	0.0000	198.93	30621000	352	15.73	15.41	17.38	16.26	15.10	14.84	15.96	15.01
2.042	0.97	1.96	P58242	Acid sphingomyelinase-like phosphodiesterase 3b	Smpdl3b	1311	10	10	10	32	32	32	51.599	0.0000	50.671	10404000	141	15.41	15.09	16.23	15.69	14.56	14.80	14.74	14.44
0.780	0.97	1.95	Q99LG2	Transportin-2	Tnpo2	4427	11	4	4	17	8.1	8.1	100.46	0.0000	62.723	4910000	80	15.04	14.56	15.69	15.45	14.55	13.95	15.52	12.85
1.169	0.97	1.95	Q80UM3	N-alpha-acetyltransferase 15, NatA auxiliary subunit	Naa15	2832	42	42	38	51.2	51.2	46.6	100.96	0.0000	293.45	101920000	600	17.06	16.54	18.44	17.25	16.46	15.90	16.70	16.37
0.818	0.97	1.95	Q8R395	COMM domain-containing protein 5	Commd5	3839	5	5	5	27.7	27.7	27.7	24.493	0.0000	41.596	6422800	37	15.04	15.34	16.60	15.45	14.77	13.30	15.54	14.96
2.281	0.96	1.95	Q8BHN3	Neutral alpha-glucosidase AB	Ganab	3049	41	41	41	47.1	47.1	47.1	106.91	0.0000	323.31	311200000	1230	18.85	18.59	19.33	19.01	18.29	17.56	18.17	17.89
2.198	0.96	1.95	Q920A7	AFG3-like protein 1	Afg3l1	4207	12	10	10	20.2	18.1	18.1	87.046	0.0000	117.82	4896800	52	13.78	14.22	14.61	13.78	13.44	12.92	12.95	13.23
1.762	0.96	1.95	Q9Z2G9	Oxidoreductase HTATIP2	Htatip2	5852	20	20	20	67.4	67.4	67.4	26.87	0.0000	64.559	43431000	258	17.20	16.96	17.71	17.14	16.71	15.67	16.67	16.12
1.301	0.96	1.95	Q8BZW8	NHL repeat-containing protein 2	Nhlrc2	3320	9	9	9	19.7	19.7	19.7	78.429	0.0000	27.413	9063300	89	15.63	15.03	16.40	15.34	14.31	14.57	14.28	15.40
1.406	0.96	1.95	P70196	TNF receptor-associated factor 6	Traf6	1534	3	3	3	7	7	7	60.069	0.0000	8.3976	2129200	22	13.68	13.60	14.69	13.90	13.15	12.92	13.62	12.33
0.731	0.96	1.95	O35657	Sialidase-1	Neu1	394	5	5	5	21.8	21.8	21.8	44.591	0.0000	32.185	4509200	62	14.29	15.12	14.48	15.23	15.11	13.84	12.22	14.13

0.473	0.96	1.94	Q5SSW2	Proteasome activator complex subunit 4	Psme4		2140	13	13	13	10.8	10.8	10.8	211.19	0.0000	61.035	11301000	81	15.70	14.96	17.32	16.12	15.62	12.87	16.48	15.29
2.228	0.96	1.94	Q01405	Protein transport protein Sec23A	Sec23a		1708	29	26	26	56.6	52.9	52.9	86.161	0.0000	323.31	49539000	395	17.15	16.90	17.95	17.47	16.52	16.35	16.41	16.35
0.863	0.96	1.94	Q9D0C4	tRNA (guanine(37)-N1)-methyltransferase	Trmt5		4858	4	4	4	15.4	15.4	15.4	56.794	0.0000	50.181	4778000	52	15.74	14.19	15.95	15.06	14.33	14.77	14.86	13.16
1.082	0.96	1.94	Q791T5	Mitochondrial carrier homolog 1	Mtch1		2733	12	12	12	36.8	36.8	36.8	41.565	0.0000	79.35	16249000	109	16.56	16.34	16.93	16.08	15.96	14.42	16.36	15.36
0.655	0.95	1.94	Q99JV5	StAR-related lipid transfer protein 4	Stard4		4350	4	4	4	26.8	26.8	26.8	25.579	0.0000	42.217	1570300	21	13.29	12.84	15.94	13.77	13.20	12.80	13.25	12.76
1.278	0.95	1.93	Q8R1U1	Conserved oligomeric Golgi complex subunit 4	Cog4		3801	11	11	11	18.1	18.1	18.1	88.66	0.0000	34.575	5795700	104	14.79	14.20	15.62	14.87	14.02	13.40	13.63	14.63
0.419	0.95	1.93	Q9CZL2	Uncharacterized protein C4orf32 homolog			4831	3	3	3	38.2	38.2	38.2	14.51	0.0000	44.166	2342700	25	14.98	12.86	16.34	13.77	13.79	14.74	13.98	11.63
2.588	0.95	1.93	P07901	Heat shock protein HSP 90-alpha	Hsp90aa1		673	71	50	50	73.5	61	61	84.787	0.0000	323.31	453040000	1411	19.02	18.71	19.45	19.36	18.18	18.01	18.43	18.12
1.690	0.95	1.93	Q5KU39	Vacuolar protein sorting-associated protein 41 homolog	Vps41		2112	19	19	19	25.2	25.2	25.2	98.601	0.0000	61.053	29243000	254	17.49	16.75	17.19	17.39	16.55	15.78	16.82	15.87
0.989	0.95	1.93	Q9CWI3	BRCA2 and CDKN1A-interacting protein	Bccip		4716	6	6	6	33.5	33.5	33.5	35.942	0.0000	33.651	5238700	54	15.57	14.72	15.93	15.23	14.56	15.03	14.87	13.18
0.989	0.95	1.93	O55135	Eukaryotic translation initiation factor 6	Eif6		459	7	7	7	46.1	46.1	46.1	26.511	0.0000	243.28	45737000	173	18.15	17.40	18.58	18.71	17.49	16.10	17.77	17.69
1.941	0.95	1.93	Q91VS7	Microsomal glutathione S-transferase 1	Mgst1		4075	10	10	10	45.2	45.2	45.2	17.551	0.0000	117.97	51849000	384	16.89	16.81	17.24	16.87	16.51	16.05	16.11	15.34
1.647	0.95	1.93	Q61024	Asparagine synthetase [glutamine-hydrolyzing]	Asns		2238	25	25	25	52.4	52.4	52.4	64.282	0.0000	180.43	50549000	317	16.57	16.35	17.63	17.09	15.86	15.73	16.29	15.98
1.179	0.95	1.93	Q9CQV6	Microtubule-associated proteins 1A/1B light chain 3B	Map1lc3b		4639	3	3	3	29.6	29.6	29.6	14.617	0.0002	5.3047	5187800	56	14.75	15.07	16.04	15.52	14.61	13.48	14.93	14.57
1.052	0.95	1.93	Q9DC63	F-box only protein 3	Fbxo3		5198	5	5	5	14.2	14.2	14.2	55.227	0.0000	10.198	4303400	25	14.88	14.87	16.01	14.40	13.32	13.82	14.63	14.60
0.658	0.95	1.93	Q7TPM9	Myotubularin-related protein 10	Mtmr10		2769	11	11	11	17.5	17.5	17.5	88.196	0.0000	53.302	6564000	49	13.55	14.73	15.98	15.48	14.03	12.95	15.11	13.87
0.747	0.94	1.92	Q8K0G5	Protein TSSC1	Tssc1		3606	4	4	4	16.3	16.3	16.3	43.126	0.0000	23.597	8520000	124	15.29	15.47	15.95	15.48	14.63	15.56	15.33	12.89
0.859	0.94	1.92	Q8BJ48	N-acetylglucosamine-1-phosphodiester alpha-N-acetylglucosaminidase	Nagpa		3071	3	3	3	9.3	9.3	9.3	56.043	0.0000	19.103	3401100	18	14.60	13.98	15.10	14.41	14.34	12.80	14.55	12.63
1.581	0.94	1.92	Q61194	Phosphatidylinositol 4-phosphate 3-kinase C2 domain-containing subun	Pik3c2a		2274	11	11	11	10.6	10.6	10.6	190.76	0.0000	32.808	7415900	105	14.90	14.43	15.80	15.02	14.01	13.79	14.51	14.07
0.746	0.94	1.92	Q9EPW0	Type I inositol 3,4-bisphosphate 4-phosphatase	Inpp4a		5267	8	8	8	12.5	12.5	12.5	105.54	0.0000	32.707	8004700	73	14.97	13.87	16.26	15.66	14.48	14.45	14.83	13.24
0.515	0.94	1.92	O88811	Signal transducing adapter molecule 2	Stam2		567	6	6	4	16.8	16.8	13.2	57.454	0.0000	43.857	13055000	72	16.52	15.50	18.56	16.71	16.04	14.29	16.66	16.54
1.181	0.94	1.92	Q8BIW1	Protein prune homolog	Prune		3065	6	6	6	17.2	17.2	17.2	50.239	0.0000	38.39	4202700	62	14.40	13.85	15.32	14.27	13.78	14.02	13.56	12.72
0.473	0.94	1.92	G3X9K3	Brefeldin A-inhibited guanine nucleotide-exchange protein 1	Arfgef1		261	21	15	15	14.1	10.4	10.4	208.5	0.0000	55.948	14502000	96	15.73	15.57	17.12	15.76	12.71	15.35	16.02	16.33
0.517	0.94	1.92	P48453	Serine/threonine-protein phosphatase 2B catalytic subunit beta isoform	Ppp3cb		1146	14	6	6	35.2	16.6	16.6	59.173	0.0000	22.835	5266400	48	14.09	13.27	16.62	14.69	14.03	12.44	14.28	14.17
1.075	0.94	1.91	Q9Z1N5	Spliceosome RNA helicase Ddx39b	Ddx39b		5815	26	11	11	65.7	38.1	38.1	49.035	0.0000	323.31	80388000	419	18.02	18.03	19.33	18.01	17.78	16.49	17.80	17.58
1.965	0.94	1.91	Q9D1Q6	Endoplasmic reticulum resident protein 44	Erp44		4933	13	13	13	36.2	36.2	36.2	46.852	0.0000	150.11	24347000	254	16.40	16.65	17.41	17.04	15.86	16.31	15.71	15.87
1.270	0.94	1.91	P61027	Ras-related protein Rab-10	Rab10		1366	15	14	13	60.5	55	55	22.541	0.0000	116.66	92571000	288	18.62	18.36	19.50	18.82	18.26	16.99	18.02	18.29
1.530	0.93	1.91	Q8VBW6	NEDD8-activating enzyme E1 regulatory subunit	Nae1		3900	16	16	16	42.5	42.5	42.5	60.273	0.0000	123.39	22963000	220	15.89	15.87	16.32	16.02	14.91	14.29	15.48	15.68
1.817	0.93	1.91	Q9D7G0	Ribose-phosphate pyrophosphokinase 1	Prps1		5029	17	17	9	64.8	64.8	42.5	34.834	0.0000	128.09	79030000	413	17.80	17.69	18.44	17.58	17.29	16.39	17.18	16.93
1.533	0.93	1.91	Q8CHH9	Septin-8		39692	3523	21	14	13	56.2	44.3	44.3	49.812	0.0000	135.3	28268000	204	16.64	16.10	16.55	16.70	16.04	15.16	16.10	14.95
1.577	0.93	1.91	Q99K51	Plastin-3	Pls3		4363	26	21	19	50	43.8	39.7	70.741	0.0000	323.31	58686000	641	16.31	16.29	17.11	16.97	15.23	15.47	16.26	15.99
0.484	0.93	1.90	O09005	Sphingolipid delta(4)-desaturase DES1	Degs1		316	5	5	5	16.1	16.1	16.1	38.241	0.0000	78.185	59348000	138	18.89	18.43	16.17	19.23	18.54	16.39	17.72	16.35
1.935	0.93	1.90	Q9WTL7	Acyl-protein thioesterase 2	Lypla2		5678	18	18	18	69.3	69.3	69.3	24.794	0.0000	323.31	215970000	638	17.78	17.82	17.44	17.70	16.80	16.05	17.11	17.07
3.028	0.93	1.90	Q60605	Myosin light polypeptide 6	Myl6		2185	10	10	8	67.5	67.5	58.9	16.93	0.0000	44.149	95055000	270	18.97	19.08	19.57	19.10	18.30	18.13	18.12	18.46
1.118	0.93	1.90	Q8K0C4	Lanosterol 14-alpha demethylase	Cyp51a1		3602	6	6	6	16.7	16.7	16.7	56.775	0.0000	19.219	5943200	59	14.66	13.92	15.41	15.15	13.60	13.28	14.60	13.96
1.406	0.92	1.90	Q7TQK1	Integrator complex subunit 7	Ints7		2776	5	5	5	8.7	8.7	8.7	106.86	0.0000	15.233	3048800	21	14.83	14.30	14.99	14.71	13.11	13.36	14.38	14.27
2.113	0.92	1.90	P16460	Argininosuccinate synthase	Ass1		810	29	29	29	67	67	67	46.584	0.0000	291.54	91003000	408	18.13	17.71	17.61	17.47	16.88	17.30	16.49	16.55
0.972	0.92	1.90	Q3ULF4	Paraplegin	Spg7		1992	17	17	17	30.9	30.9	30.9	85.995	0.0000	72.375	15476000	155	15.57	14.06	15.81	15.55	14.70	13.63	14.83	14.14
1.068	0.92	1.90	Q9D710	Thioredoxin-related transmembrane protein 2	Tmx2		5017	5	5	5	17.3	17.3	17.3	33.942	0.0000	27.886	11068000	121	15.42	14.98	16.82	15.34	15.18	14.28	14.57	14.86
0.717	0.92	1.89	Q9D7J4	Cytochrome c oxidase protein 20 homolog	Cox20		5032	4	4	4	43.6	43.6	43.6	13.163	0.0000	40.928	3665400	46	14.76	14.21	16.20	14.67	15.31	13.15	13.95	13.73
1.350	0.92	1.89	Q4PJX1	Protein odr-4 homolog	Odr4		2064	11	11	11	31.8	31.8	31.8	49.997	0.0000	129.04	18371000	149	16.31	16.11	17.26	16.49	15.92	15.57	16.09	14.90
0.415	0.92	1.89	P57716	Nicastrin	Ncstn		1296	9	9	9	15.7	15.7	15.7	78.491	0.0000	55.77	14817000	124	14.75	15.42	18.49	15.52	16.10	15.51	15.22	13.67
0.945	0.92	1.89	Q9D0I8	mRNA turnover protein 4 homolog	Mrto4		4868	7	7	7	32.6	32.6	32.6	27.545	0.0000	24.793	8440800	89	14.89	14.28	15.72	14.83	14.23	12.83	14.58	14.39
1.781	0.92	1.89	Q8QZS1	3-hydroxyisobutyryl-CoA hydrolase, mitochondrial	Hibch		3733	14	14	14	46	46	46	43.037	0.0000	168.09	32394000	300	16.69	16.18	16.75	17.05	16.02	15.78	16.05	15.13
1.161	0.92	1.89	Q8K078	Solute carrier organic anion transporter family member 4A1	Slco4a1		3599	4	4	4	9	9	9	77.668	0.0000	47.978	7646000	74	15.07	14.50	16.27	15.41	13.84	14.55	14.71	14.48
1.646	0.92	1.89	P10649	Glutathione S-transferase Mu 1	Gstm1		727	39	39	25	91.3	91.3	52.8	25.97	0.0000	323.31	2009400000	2223	21.23	21.65	21.60	20.93	20.50	21.11	20.02	20.10
1.156	0.92	1.89	Q5U4D9	THO complex subunit 6 homolog	Thoc6		2166	7	7	7	26.1	26.1	26.1	37.314	0.0000	52.323	6648900	58	14.81	14.56	15.38	14.87	14.69	14.36	13.95	12.94
1.568	0.92	1.89	Q9DC61	Mitochondrial-processing peptidase subunit alpha	Pmpca		5197	22	22	22	51.3	51.3	51.3	58.278	0.0000	235.9	61220000	467	16.96	16.56	18.02	16.99	16.38	16.17	16.20	16.12
1.139	0.92	1.89	Q9ES56	Trafficking protein particle complex subunit 4	Trappc4		5329	5	5	4	22.4	22.4	21.9	24.385	0.0004	4.6693	6757600	40	15.68	15.29	14.47	14.92	14.27	13.29	14.22	14.91
2.361	0.92	1.89	Q9D7V9	N-acylethanolamine-hydrolyzing acid amidase;N-acylethanolamine-hydr	Naaa		5041	4	4	4	13.8	13.8	13.8	40.074	0.0000	28.534	9614400	88	15.52	15.55	16.08	15.44	15.13	14.50	14.53	14.76
2.055	0.92	1.89	P70288	Histone deacetylase 2	Hdac2		1546	16	9	9	44.9	32.6	32.6	55.302	0.0000	48.131	12566000	131	15.44	14.99	15.73	15.30	14.54	14.01	14.35	14.89
1.379	0.92	1.89	Q9D0T2	Dual specificity protein phosphatase 12	Dusp12		4888	3	3	3	17.4	17.4	17.4	37.158	0.0013	3.4419	21018000	34	17.94	17.01	18.62	18.27	16.98	17.18	17.18	16.85
0.554	0.92	1.89	Q99L20		Gstt3		4404	9	9	9	45.2	45.2	45.2	27.403	0.0000	67.903	17996000	68	16.59	15.73	17.97	16.43	16.30	16.28	16.54	13.94
0.918	0.91	1.88	Q8BRH0	Transmembrane and TPR repeat-containing protein 3	Tmtc3		3181	9	9	9	13.7	13.7	13.7	104.2	0.0000	54.736	3250500	33	13.50	13.56	14.84	14.33	12.79	12.65	12.84	14.30
1.497	0.91	1.88	O35405	Phospholipase D3	Pld3		368	10	10	10	25.6	25.6	25.6	54.388	0.0000	61.453	25145000	114	17.75	17.53	16.33	17.46	16.51	16.43	16.24	16.25
1.299	0.91	1.88	Q00651	Integrin alpha-4	Itga4		1700	10	10	10	13.7	13.7	13.7	115.69	0.0000	82.171	55660000	145	18.62	18.47	19.69	19.53	18.20	17.60	18.58	18.31
1.158	0.91	1.88	Q9QZV9	NTF2-related export protein 1	Nxt1		5608	2	2	2	25.7	25.7	25.7	15.847	0.0000	9.5023	2088000	25	14.46	14.29	13.54	14.59	12.49	14.15	13.32	13.28
2.298	0.91	1.88	Q91ZW2	GDP-fucose protein O-fucosyltransferase 1	Pofut1		4203	8	8	8	30	30	30	44.688	0.0000	110.69	11175000	85	15.60	14.92	15.77	15.68	14.34	14.68	14.66	14.66
1.761	0.91	1.88	Q9ESZ8	General transcription factor II-I	Gtf2i		5352	13	13	12	15.8	15.8	13.9	112.26	0.0000	17.648	7832500	84	14.71	14.08	14.91	14.50	13.61	13.80	14.09	13.07
1.140	0.91	1.88	Q9D1P0	39S ribosomal protein L13, mitochondrial	Mrpl13		4929	7	7	7	51.7	51.7	51.7	20.677	0.0000	17.373	7727900	41	15.36	14.84	16.59	15.03	14.79	14.43	14.78	14.21
0.597	0.91	1.87	O35473	Nuclear nucleic acid-binding protein C1D	C1d		374	2	2	2	29.8	29.8	29.8	15.945	0.0000	35.609	2188900	38	14.01	14.21	15.09	13.59	11.67	14.33	12.92	14.35
1.055	0.91	1.87	Q920E5	Farnesyl pyrophosphate synthase	Fdps		4210	28	28	28	64	64	64	40.581	0.0000	323.31	211400000	715	18.59	18.28	19.71	19.52	17.69	17.61	18.73	18.44
1.118	0.91	1.87	P58281	Dynamin-like 120 kDa protein, mitochondrial;Dynamin-like 120 kDa prot	Opa1		1313	54	54	54	61.6	61.6	61.6	111.34	0.0000	323.31	117220000	903	16.55	16.25	17.95	16.82	16.21	16.01	16.33	15.40
0.522	0.90	1.87	Q9JI39	ATP-binding cassette sub-family B member 10, mitochondrial	Abcb10		5385	5	5	5	11.7	11.7	11.7	77.187	0.0000	20.417	4017700	33	14.59	14.56	13.21	14.74	11.65	12.79	14.83	14.21
1.043	0.90	1.87	Q6P3E7	Histone deacetylase 10	Hdac10		2562	3	3	3	6.3	6.3	6.3	72.108	0.0002	5.9251	2565900	33	14.13	14.07	14.26	14.78	13.59	14.51	12.68	12.84
0.926	0.90	1.87	O70589	Peripheral plasma membrane protein CASK	Cask		516	5	5	5	10.7	10.7	10.7	105.11	0.0000	14.373	3805200	39	14.97	15.04	14.33	14.99	14.33	13.58	12.82	14.98
1.509	0.90	1.87	Q8BYL4	Tyrosine--tRNA ligase, mitochondrial	Yars2		3303	9	9	9	26.9	26.9	26.9	52.597	0.0000	27.726	5561000	85	14.94	13.89	15.28	14.65	13.72	13.48	14.10	13.84
1.007	0.90	1.87	Q64518	Sarcoplasmic/endoplasmic reticulum calcium ATPase 3	Atp2a3		2451	22	16	16	26.7	21.1	21.1	109.53	0.0000	99.021	27788000	254	15.77	15.40	17.38	16.13	15.06	15.22	15.75	15.04
1.480	0.90	1.87	P84089	Enhancer of rudimentary homolog	Erh		1607	8	8	8	85.6	85.6	85.6	12.259	0.0000	113.15	20642000	256	15.88	16.22	17.22	16.71	15.61	15.61	15.22	15.96
1.258	0.90	1.87	Q91V64	Isochorismatase domain-containing protein 1	Isoc1		4048	12	12	12	45.5	45.5	45.5	32.032	0.0000	57.412	34732000	239	16.71	16.65	17.86	16.97	16.31	15.39	16.56	16.33
0.978	0.90	1.87	Q6NT99	Dual specificity protein phosphatase 23	Dusp23		2528	5	5	5	44.7	44.7	44.7	16.637	0.0000	6.8456	3626900	27	14.77	14.67	15.63	15.23	14.82	12.95	14.53	14.39
1.104	0.90	1.87	P03958	Adenosine deaminase	Ada		633	6	6	5	28.4	28.4	25.6	39.991	0.0000	21.323	3813400	27	13.46	13.90	14.56	14.46	12.26	13.36	13.28	13.88
0.342	0.90	1.86	P48441	Alpha-L-iduronidase	Idua		1145	2	2	2	4.6	4.6	4.6	71.253	0.0002	5.7507	8905300	41	16.50	16.19	13.87	16.30	15.39	12.46	14.45	16.96
0.868	0.90	1.86	P24604	Tyrosine-protein kinase Tec	Tec		900	7	7	7	13.2	13.2	13.2	73.425	0.0000	35.05	3048800	62	14.51	14.10	14.92	14.41	12.96	14.13	12.57	14.68
0.640	0.90	1.86	P81117	Nucleobindin-2;Nesfatin-1	Nucb2		1597	11	11	11	32.1	32.1	32.1	50.304	0.0000	68.232	4826800	85	14.71	14.64	12.51	14.48	14.24	13.35	12.26	12.91
0.332	0.90	1.86	Q9JIK9	28S ribosomal protein S34, mitochondrial	Mrps34		5404	10	10	10	52.8	52.8	52.8	25.826	0.0000	45.739	29164000	92	17.72	17.98	18.03	17.91	18.14	13.57	18.22	18.13
0.882	0.90	1.86	Q8BHF7	CDP-diacylglycerol--glycerol-3-phosphate 3-phosphatidyltransferase, m	Pgs1		3035	10	10	10	22.4	22.4	22.4	62.488	0.0000	64.122	13837000	138	15.40	14.92	16.55	15.30	14.97	13.54	15.18	14.89
0.733	0.90	1.86	P60605	Ubiquitin-conjugating enzyme E2 G2	Ube2g2		1356	6	6	6	53.3	53.3	53.3	18.566	0.0000	70.447	14662000	106	16.85	17.04	15.40	16.90	16.32	16.07	15.91	14.30
2.584	0.90	1.86	P14824	Annexin A6	Anxa6		786	78	78	77	86.2	86.2	85.9	75.884	0.0000	323.31	821550000	2620	19.43	19.24	19.91	19.59	18.77	18.35	18.87	18.60
2.320	0.90	1.86	Q9CYN2	Signal peptidase complex subunit 2	Spcs2		4803	16	16	16	53.5	53.5	53.5	24.977	0.0000	222.82	77201000	424	17.46	17.09	17.92	17.61	16.56	16.47	16.95	16.52
0.552	0.89	1.86	P17665	Cytochrome c oxidase subunit 7C, mitochondrial	Cox7c		827	4	4	4	31.7	31.7	31.7	7.3325	0.0000	39.373	3648000	46	14.73	13.54	15.76	15.65	14.15	13.34	15.54	13.08
1.358	0.89	1.86	P63325	40S ribosomal protein S10	Rps10		1505	17	16	16	67.3	67.3	67.3	18.916	0.0000	107.02	168720000	491	19.04	18.96	20.41	19.32	18.61	18.24	18.75	18.55
0.826	0.89	1.86	Q9D1G2	Phosphomevalonate kinase	Pmvk		4912	6	6	6	40.6	40.6	40.6	21.916	0.0000	14.843	3066400	21	14.48	13.70	15.06	13.07	13.41	13.98	12.70	12.65
0.870	0.89	1.86	Q9CX13	Protein cornichon homolog 4	Cnih4		4744	1	1	1	14.4	14.4	14.4	16.089	0.0000	9.7567	2783400	27	14.45	15.37	13.35	13.70	14.00	13.46	12.88	12.96
2.165	0.89	1.86	Q9Z2X1	Heterogeneous nuclear ribonucleoprotein F;Heterogeneous nuclear ribo	Hnrnpf		5870	26	26	24	68	68	65.5	45.729	0.0000	323.31	163360000	689	18.83	19.44	18.87	19.38	17.94	18.45	18.03	18.53
0.625	0.89	1.85	Q9ERF3	WD repeat-containing protein 61;WD repeat-containing protein 61, N-ter	Wdr61		5308	6	6	6	38.7	38.7	38.7	33.772	0.0000	45.677	4149400	43	12.96	14.96	13.58	15.42	12.86	13.11	14.40	12.99
2.027	0.89	1.85	Q8CD15	Bifunctional lysine-specific demethylase and histidyl-hydroxylase MINA	Mina		3455	3	3	3	8.4	8.4	8.4	53.516	0.0000	21.866	2411200	26	14.30	13.85	14.59	14.62	13.36	13.76	13.05	13.62
1.664	0.89	1.85	O35286	Pre-mRNA-splicing factor ATP-dependent RNA helicase DHX15	Dhx15		352	43	43	42	53.5	53.5	53.5	91.006	0.0000	308.44	186960000	1076	17.80	17.41	18.63	17.85	17.13	16.75	17.37	16.88
0.831	0.89	1.85	Q8C7K6	Prenylcysteine oxidase-like	Pcyox1l		3412	6	6	6	16	16	16	54.874	0.0000	40.081	9575600	111	15.01	15.04	13.85	15.37	13.71	14.32	14.81	12.87
1.170	0.89	1.85	Q5SQX6	Cytoplasmic FMR1-interacting protein 2	Cyfip2		2132	41	18	18	35.9	17.4	17.4	145.66	0.0000	75.842	19104000	114	16.59	15.75	17.24	16.69	16.06	15.44	16.13	15.08
1.269	0.89	1.85	Q566J8	AarF domain-containing protein kinase 4	Adck4		2085	18	18	18	58	58	58	59.231	0.0000	279.28	27053000	248	16.13	15.57	16.69	16.38	15.98	15.35	15.30	14.58
2.633	0.89	1.85	Q9D8W5	26S proteasome non-ATPase regulatory subunit 12	Psmd12		5079	35	35	34	76.1	76.1	73.7	52.895	0.0000	323.31	118390000	839	17.65	17.54	18.26	17.90	17.08	16.73	17.00	16.99
1.661	0.89	1.85	P97823	Acyl-protein thioesterase 1	Lypla1		1679	13	13	13	59.6	59.6	59.6	24.687	0.0000	214.27	83388000	232	16.30	16.26	15.78	16.50	15.47	14.72	15.23	15.88
1.078	0.89	1.85	P06802	Ectonucleotide pyrophosphatase/phosphodiesterase family member 1;A	Enpp1		666	4	4	4	7.1	7.1	7.1	103.17	0.0000	50.356	3461700	58	14.90	13.40	14.62	14.73	13.21	13.67	13.04	14.18
0.379	0.89	1.85	O08523	Alpha-tectorin	Tecta		266	4	4	4	2.2	2.2	2.2	239.43	0.0015	3.3497	1430100	13	13.27	12.33	15.77	13.53	11.03	14.49	12.81	13.03
2.599	0.88	1.85	P97449	Aminopeptidase N	Anpep		1650	57	57	57	51.2	51.2	51.2	109.65	0.0000	323.31	683560000	2077	19.80	19.56	20.34	19.91	19.17	19.06	19.00	18.84
2.246	0.88	1.85	P61965	WD repeat-containing protein 5	Wdr5		1395	13	13	13	54.8	54.8	54.8	36.588	0.0000	49.696	24148000	161	16.20	15.89	16.54	16.35	15.35	15.31	15.78	15.00
1.724	0.88	1.85	P50136	2-oxoisovalerate dehydrogenase subunit alpha, mitochondrial	Bckdha		1175	11	11	11	30.3	30.3	30.3	50.37	0.0000	61.532	14169000	164	16.05	15.84	16.85	15.84	15.24	15.65	15.13	15.02
1.432	0.88	1.84	Q8VDW0	ATP-dependent RNA helicase DDX39A	Ddx39a		3975	27	27	11	65.6	65.6	39.6	49.067	0.0000	269.37	158020000	577	18.59	18.49	19.47	18.58	18.12	17.19	18.21	18.07
0.962	0.88	1.84	Q9QYF1	Retinol dehydrogenase 11	Rdh11		5571	14	14	14	38.9	38.9	38.9	35.147	0.0000	100.02	37154000	238	17.46	17.42	18.04	17.45	16.99	15.43	16.92	17.49
0.729	0.88	1.84	Q04207	Transcription factor p65	Rela		1730	12	12	10	29.7	29.7	27.7	60.211	0.0000	117.18	24004000	144	16.67	15.12	15.90	16.65	15.20	15.84	15.88	13.89
1.628	0.88	1.84	P97311	DNA replication licensing factor MCM6	Mcm6		1621	28	28	27	35.3	35.3	35.3	92.866	0.0000	119.26	42384000	312	16.65	16.11	17.50	16.67	15.76	15.77	15.94	15.95
1.726	0.88	1.84	P45952	Medium-chain specific acyl-CoA dehydrogenase, mitochondrial	Acadm		1100	26	26	26	61.5	61.5	61.5	46.481	0.0000	323.31	136340000	780	18.23	17.91	19.13	18.59	17.66	17.49	17.79	17.41
0.925	0.88	1.84	Q6DFW0	Protein C9orf72 homolog			2505	5	5	5	16.2	16.2	16.2	54.277	0.0000	11.801	4870400	37	15.31	13.78	15.20	15.00	13.64	14.06	14.81	13.27
0.932	0.88	1.84	P15532	Nucleoside diphosphate kinase A	Nme1		797	13	7	7	82.2	48.7	48.7	17.208	0.0000	33.751	136960000	276	19.38	18.98	18.10	19.31	18.06	17.07	18.24	18.89
1.177	0.88	1.83	O35704	Serine palmitoyltransferase 1	Sptlc1		400	17	17	17	46.3	46.3	46.3	52.534	0.0000	192.41	34782000	261	16.90	16.29	17.73	16.63	16.06	15.90	16.63	15.46
0.892	0.87	1.83	O88796	Ribonuclease P protein subunit p30	Rpp30		566	8	8	8	36.2	36.2	36.2	29.473	0.0000	33.431	6045300	83	14.66	14.83	14.92	14.95	14.36	12.54	14.78	14.18
0.982	0.87	1.83	B1AY13	Ubiquitin carboxyl-terminal hydrolase 24	Usp24		73	24	24	24	13.9	13.9	13.9	294	0.0000	110.64	18775000	193	14.89	14.71	16.27	14.96	14.60	13.91	15.01	13.82

1.409	0.87	1.83	Q9D5V5	Cullin-5	Cul5	4986	27	27	27	40.3	40.3	40.3	90.973	0.0000	92.756	24174000	199	15.67	15.25	16.50	15.87	15.03	14.45	15.44	14.88
0.884	0.87	1.83	P28293	Cathepsin G	Ctsg	948	10	10	10	33	33	33	29.095	0.0000	35.973	30968000	171	16.63	16.33	18.05	16.83	16.20	16.96	15.56	15.62
0.440	0.87	1.83	Q80ZJ1	Ras-related protein Rap-2a	Rap2a	2914	9	4	4	58.5	29.5	29.5	20.642	0.0000	21.515	18821000	112	17.01	16.84	14.94	16.79	16.42	13.31	16.36	15.97
1.425	0.87	1.83	Q99LD9	Translation initiation factor eIF-2B subunit beta	Eif2b2	4422	12	12	12	42.7	42.7	42.7	38.897	0.0000	66.297	15680000	172	15.23	15.47	16.18	15.67	14.85	14.80	15.32	14.08
1.393	0.87	1.83	P24547	Inosine-5-monophosphate dehydrogenase 2	Impdh2	899	31	31	29	57.8	57.8	56	55.814	0.0000	314.21	88459000	626	16.88	16.79	18.17	17.22	16.35	16.11	16.61	16.51
1.185	0.87	1.83	Q99JX3	Golgi reassembly-stacking protein 2	Gorasp2	4353	4	4	4	16	16	16	47.038	0.0000	31.033	7590300	79	15.68	15.84	14.42	15.89	14.40	15.03	14.29	14.63
1.048	0.87	1.83	Q8R2Y8	Peptidyl-tRNA hydrolase 2, mitochondrial	Ptrh2	3826	8	8	8	65.7	65.7	65.7	19.526	0.0000	107.07	55619000	291	17.22	16.97	18.66	17.38	16.86	16.18	17.14	16.57
0.763	0.87	1.82	Q3UHH8	Glucoside xylosyltransferase 1	Gxylt1	1973	6	6	6	14.6	14.6	14.6	46.49	0.0000	11.19	1893600	44	13.09	12.96	14.77	13.37	12.56	13.78	12.21	12.17
0.419	0.87	1.82	Q61133	Glutathione S-transferase theta-2	Gstt2	2256	4	4	4	19.7	19.7	19.7	27.634	0.0046	2.3758	8185600	15	15.85	16.19	13.30	16.81	15.47	13.36	15.39	14.47
0.335	0.86	1.82	Q8BNI4	Derlin-2	Derl2	3154	2	2	2	16.3	16.3	16.3	27.639	0.0000	33.822	8609100	40	13.12	15.90	15.54	16.06	16.46	14.60	13.76	12.34
1.327	0.86	1.82	A2BH40	AT-rich interactive domain-containing protein 1A	Arid1a	46	27	26	22	17.8	17.5	14.8	242.09	0.0000	118.54	22859000	242	15.35	14.92	16.15	15.85	14.29	14.43	15.24	14.85
0.810	0.86	1.82	O55229	Choline/ethanolamine kinase	Chkb	465	12	12	12	44.4	44.4	44.4	45.126	0.0000	96.94	14775000	157	15.55	15.42	14.87	15.66	15.03	13.07	15.29	14.68
0.615	0.86	1.81	Q3URF8	BTB/POZ domain-containing protein KCTD21	Kctd21	2023	4	4	4	30.4	30.4	30.4	29.623	0.0000	22.407	4350700	57	14.64	13.32	16.33	15.03	13.94	13.36	14.07	14.52
0.943	0.86	1.81	Q3U9N9	Monocarboxylate transporter 10	Slc16a10	1937	1	1	1	3.5	3.5	3.5	55.32	0.0000	50.222	10650000	70	16.07	15.38	16.14	16.55	15.29	14.47	16.24	14.71
0.860	0.86	1.81	Q9CR56	NF-kappa-B inhibitor-interacting Ras-like protein 2	Nkiras2	4668	2	2	2	12.6	12.6	12.6	21.494	0.0004	5.0157	6784300	58	15.10	15.11	15.95	15.71	15.25	14.03	15.51	13.65
0.534	0.86	1.81	Q7TSE6	Serine/threonine-protein kinase 38-like	Stk38l	2781	7	4	4	13.6	9.5	9.5	53.771	0.0000	13.945	2615700	32	14.48	14.26	17.23	14.66	14.74	13.55	14.33	14.58
0.367	0.86	1.81	Q9JLC3	Methionine-R-sulfoxide reductase B1	Msrb1	5476	3	3	2	36.2	36.2	15.5	12.788	0.0025	2.7961	7617300	44	15.55	15.37	14.32	15.45	12.98	12.32	16.05	15.90
1.560	0.86	1.81	Q6PGB6	N-alpha-acetyltransferase 50	Naa50	2631	9	9	9	46.2	46.2	46.2	19.414	0.0000	61.47	33131000	276	16.67	16.24	17.40	16.62	16.05	15.94	16.13	15.38
0.919	0.86	1.81	Q8R2V5	Arf-GAP with dual PH domain-containing protein 2	Adap2	3823	5	5	5	18.6	18.6	18.6	43.989	0.0000	13.747	3499700	21	14.66	14.12	12.98	14.78	13.11	12.69	13.58	13.74
2.348	0.85	1.81	Q8R307	Vacuolar protein sorting-associated protein 18 homolog	Vps18	3827	33	33	33	41	41	41	110.22	0.0000	122.29	40952000	400	16.58	16.05	16.86	16.52	15.73	15.48	15.88	15.49
1.333	0.85	1.81	Q924N4	Solute carrier family 12 member 6	Slc12a6	4298	8	8	6	11.3	11.3	9.1	127.53	0.0000	59.721	9992000	94	16.28	15.51	16.54	17.11	15.47	15.34	15.70	15.51
0.482	0.85	1.80	Q9JJA2	Conserved oligomeric Golgi complex subunit 8	Cog8	5421	10	10	10	29.2	29.2	29.2	71.601	0.0000	53.045	5428000	39	13.82	13.54	16.70	14.76	12.94	14.57	14.29	13.61
0.988	0.85	1.80	Q3B7Z2	Oxysterol-binding protein 1	Osbp	1808	21	21	21	31.8	31.8	31.8	88.796	0.0000	222.77	36195000	327	16.01	15.49	17.39	16.33	15.39	15.29	15.98	15.15
0.713	0.85	1.80	Q9CR89	Endoplasmic reticulum-Golgi intermediate compartment protein 2	Ergic2	4682	3	3	3	13.3	13.3	13.3	42.481	0.0000	16.663	2954000	57	14.42	13.59	14.04	14.06	13.54	11.53	13.83	13.81
1.034	0.85	1.80	O35623	BET1 homolog	Bet1	388	3	3	3	28	28	28	13.274	0.0000	24.85	3590400	64	14.46	13.29	14.56	14.44	14.17	12.73	13.21	13.26
0.565	0.85	1.80	Q8BH47	Vesicle-trafficking protein SEC22a	Sec22a	3010	3	3	3	13	13	13	35.024	0.0000	6.3211	1910000	38	13.33	13.34	15.26	13.65	11.58	13.14	14.11	13.35
0.679	0.85	1.80	P52479	Ubiquitin carboxyl-terminal hydrolase 10	Usp10	1218	6	6	6	12.4	12.4	12.4	87.021	0.0000	37.525	2813000	36	14.46	13.29	14.24	14.52	13.17	13.95	14.18	11.83
1.262	0.85	1.80	Q99LI7	Cleavage stimulation factor subunit 3	Cstf3	4433	19	19	19	35.6	35.6	35.6	82.876	0.0000	125.21	68020000	236	19.13	18.70	19.97	19.39	18.12	18.16	19.14	18.38
1.373	0.85	1.80	Q9DBB8	Trans-1,2-dihydrobenzene-1,2-diol dehydrogenase	Dhdh	5140	16	16	16	53.8	53.8	53.8	36.3	0.0000	224.29	29937000	282	15.78	15.42	16.91	16.04	15.17	14.97	15.23	15.40
0.673	0.85	1.80	Q9CZW4	Long-chain-fatty-acid--CoA ligase 3	Acsl3	4842	19	17	17	39	36.7	36.7	80.491	0.0000	149.27	20968000	183	15.96	15.58	17.39	16.90	14.57	15.28	16.60	16.01
1.170	0.85	1.80	O08739	AMP deaminase 3	Ampd3	292	26	26	23	41.3	41.3	37.3	88.651	0.0000	243.31	50484000	444	17.28	16.75	18.49	17.53	16.64	16.96	16.63	16.43
0.758	0.85	1.80	Q8BGC4	Zinc-binding alcohol dehydrogenase domain-containing protein 2	Zadh2	2976	11	11	11	47.7	47.7	47.7	40.528	0.0000	22.906	12263000	97	15.71	15.80	15.92	15.10	14.84	13.30	15.48	15.52
0.918	0.84	1.79	Q3UHC7	Disabled homolog 2-interacting protein	Dab2ip	1970	3	3	3	3.6	3.6	3.6	131.72	0.0066	2.0751	3872600	27	14.12	13.66	14.67	14.80	13.30	13.11	14.60	12.88
1.030	0.84	1.79	Q61878	Bone marrow proteoglycan;Eosinophil granule major basic protein	Prg2	2340	6	6	6	30	30	30	24.255	0.0000	49.768	119080000	151	20.33	20.41	20.90	19.21	19.73	19.78	18.92	19.05
0.832	0.84	1.79	Q8BMZ5	tRNA-splicing endonuclease subunit Sen34	Tsen34	3151	2	2	2	10.1	10.1	10.1	34.196	0.0004	4.4217	2169200	28	14.17	14.11	14.51	14.25	14.40	13.59	12.03	13.65
1.524	0.84	1.79	P23780	Beta-galactosidase	Glb1	889	23	23	22	35.2	35.2	33.4	73.12	0.0000	204.24	63321000	399	17.59	17.38	18.48	17.66	17.35	16.53	17.05	16.82
0.920	0.84	1.79	Q6KAR6	Exocyst complex component 3	Exoc3	2520	18	18	18	24.2	24.2	24.2	86.454	0.0000	91.653	15359000	157	15.28	14.72	16.84	15.85	14.73	15.00	15.02	14.57
2.078	0.84	1.79	P54775	26S protease regulatory subunit 6B	Psmc4	1257	26	26	26	81.1	81.1	81.1	47.408	0.0000	323.31	134060000	858	18.12	17.79	18.34	18.23	17.43	16.74	17.47	17.48
2.000	0.84	1.79	Q61881	DNA replication licensing factor MCM7	Mcm7	2342	35	35	35	55.2	55.2	55.2	81.21	0.0000	207.62	81082000	754	16.96	16.76	17.75	17.24	16.19	16.27	16.38	16.53
0.925	0.84	1.79	Q9QXG2	Rab proteins geranylgeranyltransferase component A 1	Chm	5540	5	5	5	15.2	15.2	15.2	73.976	0.0000	78.631	5839500	82	14.94	15.04	14.76	14.70	15.10	12.98	14.34	13.66
1.200	0.84	1.79	Q80U58	Pumilio homolog 2	Pum2	2820	10	6	6	9.8	5.7	5.7	114.31	0.0000	31.795	7364100	87	15.30	14.29	15.56	15.03	13.79	14.63	13.78	14.63
0.544	0.84	1.79	Q8JZK9	Hydroxymethylglutaryl-CoA synthase, cytoplasmic	Hmgcs1	3573	6	6	6	21.2	21.2	21.2	57.568	0.0000	26.945	4533900	51	14.23	14.80	15.19	15.62	13.23	15.73	14.61	12.92
0.921	0.84	1.78	Q9CR09	Ubiquitin-fold modifier-conjugating enzyme 1	Ufc1	4656	10	10	10	56.9	56.9	56.9	19.481	0.0000	60.684	29344000	189	16.37	16.17	18.08	16.66	16.38	15.62	16.11	15.84
2.553	0.84	1.78	Q91YX5	Acyl-CoA:lysophosphatidylglycerol acyltransferase 1	Lpgat1	4184	5	5	5	19.5	19.5	19.5	43.088	0.0000	26.035	4017700	53	14.69	14.22	14.66	14.57	13.84	14.00	13.45	13.50
1.652	0.84	1.78	Q8R3C0	Mini-chromosome maintenance complex-binding protein	Mcmbp	3840	9	9	9	22.1	22.1	22.1	72.89	0.0000	28.103	3606100	45	14.50	14.53	15.05	14.52	13.94	13.67	13.25	14.39
3.927	0.83	1.78	P16110	Galectin-3	Lgals3	805	16	16	16	45.1	45.1	45.1	27.515	0.0000	323.31	4239300000	4217	22.53	22.49	22.39	22.54	21.91	21.50	21.63	21.59
2.120	0.83	1.78	P25799	Nuclear factor NF-kappa-B p105 subunit;Nuclear factor NF-kappa-B p5(	Nfkb1	911	19	19	19	29.9	29.9	29.9	105.61	0.0000	208.29	28864000	252	16.48	16.42	17.22	16.85	15.92	15.65	16.14	15.92
1.447	0.83	1.78	Q8BH24	Transmembrane 9 superfamily member 4	Tm9sf4	3008	13	13	13	17.4	17.4	17.4	74.692	0.0000	79.273	45290000	297	17.31	16.87	18.22	17.32	16.48	16.45	16.96	16.51
0.478	0.83	1.78	Q91WM2	Cat eye syndrome critical region protein 5 homolog	Cecr5	4120	5	5	5	16.2	16.2	16.2	46.306	0.0000	22.316	5659800	81	14.97	14.71	14.79	15.32	14.78	14.91	14.98	11.79
0.607	0.83	1.78	Q9JJY4	Probable ATP-dependent RNA helicase DDX20	Ddx20	5439	11	11	11	21.9	21.9	21.9	91.709	0.0000	55	10344000	125	15.51	14.62	17.63	15.76	14.67	15.30	15.21	15.01
1.213	0.83	1.78	Q91YP2	Neurolysin, mitochondrial	Nln	4170	24	24	24	42.2	42.2	42.2	80.428	0.0000	134	44896000	306	16.69	16.46	17.94	16.83	16.27	16.08	16.47	15.78
1.108	0.83	1.78	Q99LC8	Translation initiation factor eIF-2B subunit alpha	Eif2b1	4418	7	7	7	31.5	31.5	31.5	33.816	0.0000	20.801	8740100	70	15.60	15.15	14.71	15.03	15.23	13.94	13.64	14.38
1.140	0.83	1.78	Q9CY27	Very-long-chain enoyl-CoA reductase	Tecr	4780	20	20	20	38.3	38.3	38.3	36.09	0.0000	89.824	119020000	517	18.54	18.11	18.46	18.63	17.82	16.56	18.24	17.82
0.659	0.83	1.78	Q80Y56	Rabenosyn-5	Rbsn	2895	4	4	4	9.7	9.7	9.7	88.49	0.0000	76.184	3600200	58	15.79	15.05	13.24	15.56	13.89	13.95	14.61	13.88
0.676	0.83	1.78	P11609;P11	Antigen-presenting glycoprotein CD1d1;Antigen-presenting glycoprotein	Cd1d1;Cd1d2	747	2	2	2	6.8	6.8	6.8	38.554	0.0000	8.8268	2101100	39	14.51	14.82	12.91	14.30	12.51	14.30	12.76	13.66
1.997	0.83	1.77	Q9EQU5	Protein SET	Set	5289	8	8	8	29.1	29.1	29.1	33.377	0.0000	91.015	12395000	102	16.12	16.34	16.41	16.66	15.16	15.32	15.71	16.02
0.922	0.83	1.77	O70572	Sphingomyelin phosphodiesterase 2	Smpd2	514	7	7	7	22	22	22	47.466	0.0000	16.442	4560400	34	14.55	13.75	14.95	14.07	13.24	13.18	14.61	12.99
2.804	0.83	1.77	Q8QZY1	Eukaryotic translation initiation factor 3 subunit L	Eif3l	3738	37	37	36	63.5	63.5	63.5	66.612	0.0000	323.31	165050000	847	17.86	17.71	18.29	17.75	17.20	16.93	17.20	16.97
2.902	0.82	1.77	P97363	Serine palmitoyltransferase 2	Sptlc2	1631	19	19	19	51.1	51.1	51.1	62.981	0.0000	220.82	44297000	314	17.39	17.03	17.58	17.48	16.64	16.31	16.67	16.56
1.733	0.82	1.77	Q9JJU8	SH3 domain-binding glutamic acid-rich-like protein	Sh3bgrl	5436	6	6	6	59.6	59.6	59.6	12.811	0.0000	137.49	17259000	183	16.49	16.91	17.17	17.05	15.67	16.45	15.76	16.44
0.644	0.82	1.77	Q8CHK3	Lysophospholipid acyltransferase 7	Mboat7	3525	4	4	4	13.1	13.1	13.1	53.435	0.0000	26.5	2515800	20	13.61	14.46	14.22	12.62	12.29	13.51	13.83	11.97
1.190	0.82	1.77	Q60790	Ras GTPase-activating protein 3	Rasa3	2208	29	29	29	37.4	37.4	37.4	95.986	0.0000	117.72	32224000	290	15.92	15.48	17.08	16.14	15.25	15.11	15.73	15.23
0.804	0.82	1.77	Q3U0M1	Trafficking protein particle complex subunit 9	Trappc9	1896	7	7	7	7.2	7.2	7.2	128.23	0.0000	16.347	3629500	38	13.70	13.77	15.51	13.99	13.42	12.97	14.20	13.09
1.611	0.82	1.77	P62827	GTP-binding nuclear protein Ran	Ran	1448	24	24	23	63.9	63.9	63.9	24.423	0.0000	186.34	504100000	751	20.44	20.13	21.27	20.72	19.88	19.43	20.04	19.89
0.478	0.82	1.77	Q99PG2	Opioid growth factor receptor	Ogfr	4505	8	8	8	15	15	15	70.678	0.0000	24.598	10245000	75	15.79	15.59	14.78	15.52	15.06	14.69	16.10	12.54
1.108	0.82	1.77	Q61334	B-cell receptor-associated protein 29	Bcap29	2292	16	16	16	36.7	36.7	36.7	27.964	0.0000	71.382	37425000	318	16.23	15.53	17.38	16.32	15.51	15.41	15.74	15.50
1.291	0.82	1.77	Q99PV0	Pre-mRNA-processing-splicing factor 8	Prpf8	4518	83	83	82	41.6	41.6	40.3	273.61	0.0000	323.31	395960000	1135	21.25	20.68	22.17	21.45	20.49	20.23	20.91	20.65
2.882	0.82	1.77	P49722	Proteasome subunit alpha type-2	Psma2	1170	17	17	17	68.4	68.4	68.4	25.926	0.0000	263.36	227680000	654	18.66	18.79	18.96	19.13	18.30	17.81	18.03	18.12
1.561	0.82	1.77	P06800	Receptor-type tyrosine-protein phosphatase C	Ptprc	664	49	49	49	36	36	36	144.84	0.0000	323.31	180420000	985	18.40	17.82	19.18	18.45	17.60	17.62	17.80	17.54
0.689	0.82	1.76	Q6ZWR6	Nesprin-1	Syne1	2705	14	14	14	2.5	2.5	2.5	1009.9	0.0000	41.886	6835000	42	15.43	14.63	16.41	15.21	14.11	15.04	15.60	13.65
0.966	0.82	1.76	Q8BGZ4	Cell division cycle protein 23 homolog	Cdc23	3003	6	6	6	14.6	14.6	14.6	68.561	0.0000	58.936	4799400	52	15.46	14.72	15.98	14.62	14.59	13.54	14.86	14.52
1.743	0.82	1.76	Q60737	Casein kinase II subunit alpha	Csnk2a1	2199	18	18	18	53.7	53.7	53.7	45.133	0.0000	323.31	81820000	501	17.24	16.89	17.75	17.58	16.82	16.26	16.85	16.25
1.072	0.82	1.76	Q9CQS2	H/ACA ribonucleoprotein complex subunit 3	Nop10	4627	5	5	5	87.5	87.5	87.5	7.7059	0.0000	37.971	8858700	72	15.43	15.21	15.81	16.05	15.23	15.18	15.06	13.77
1.216	0.82	1.76	Q8BKE6	Cytochrome P450 20A1	Cyp20a1	3100	10	10	10	25.3	25.3	25.3	52.149	0.0000	59.661	11741000	99	15.69	16.05	17.27	16.18	15.69	15.34	15.61	15.28
0.648	0.82	1.76	Q9D1H7	Golgi to ER traffic protein 4 homolog	Get4	4915	5	5	5	16.8	16.8	16.8	36.525	0.0000	42.367	5339200	50	15.70	15.49	16.19	14.35	15.40	14.36	15.29	13.42
2.848	0.82	1.76	Q60960	Importin subunit alpha-5;Importin subunit alpha-5, N-terminally processe	Kpna1	2228	15	11	11	39.2	32	32	60.182	0.0000	104	18461000	151	16.78	16.65	16.80	16.76	16.19	15.67	16.16	15.69
1.371	0.82	1.76	Q922R1	UPF0183 protein C16orf70 homolog		4274	4	4	4	16.4	16.4	16.4	47.416	0.0000	14.457	2724500	35	14.40	14.47	14.66	14.36	14.01	12.82	14.23	13.56
0.840	0.82	1.76	Q8CDM8	Protein FAM160B1	Fam160b1	3463	5	5	5	10.1	10.1	10.1	86.016	0.0000	22.691	4933900	63	14.52	14.02	15.10	14.88	14.14	12.57	14.50	14.05
1.767	0.81	1.76	Q8BKT7	THO complex subunit 5 homolog	Thoc5	3106	10	10	10	23.6	23.6	23.6	78.685	0.0000	80.253	6657700	74	15.29	15.22	15.36	15.35	14.25	15.21	14.10	14.41
1.033	0.81	1.76	Q8BWZ3	N-alpha-acetyltransferase 25, NatB auxiliary subunit	Naa25	3273	21	21	21	30.7	30.7	30.7	111.71	0.0000	69.589	18022000	215	15.58	15.33	16.78	15.63	14.80	15.39	15.47	14.41
1.835	0.81	1.76	Q99LL5	Periodic tryptophan protein 1 homolog	Pwp1	4439	5	5	5	15.4	15.4	15.4	55.586	0.0000	19.592	4401800	64	14.81	14.23	15.20	14.88	13.58	14.15	14.03	14.11
1.342	0.81	1.76	Q9WV98	Mitochondrial import inner membrane translocase subunit Tim9	Timm9	5742	2	2	2	32.6	32.6	32.6	10.344	0.0000	10.078	2983500	47	14.50	14.31	15.66	14.64	13.90	13.76	14.28	13.92
1.000	0.81	1.76	Q9WVS7	Dual specificity mitogen-activated protein kinase kinase 5	Map2k5	5761	3	3	3	9.4	9.4	9.4	50.104	0.0000	11.037	3704400	47	14.37	13.84	14.83	14.32	13.47	13.77	14.30	12.56
1.421	0.81	1.75	Q60823	RAC-beta serine/threonine-protein kinase	Akt2	2211	10	8	8	31	24.5	24.5	55.741	0.0000	46.55	6091600	65	14.63	13.87	14.56	15.09	13.70	14.07	13.88	13.26
1.111	0.81	1.75	O88587	Catechol O-methyltransferase	Comt	548	13	13	13	52.1	52.1	52.1	29.486	0.0000	216.16	55193000	347	17.67	17.55	16.89	17.41	17.30	15.66	16.61	16.71
3.291	0.81	1.75	Q9D8Y0	EF-hand domain-containing protein D2	Efhd2	5082	20	20	20	61.7	61.7	61.7	26.791	0.0000	123.55	49296000	430	17.25	17.43	17.61	17.65	16.62	16.87	16.50	16.72
1.493	0.81	1.75	P46737	Lys-63-specific deubiquitinase BRCC36	Brcc3	1112	9	9	9	36.1	36.1	36.1	33.34	0.0000	30.118	16743000	130	15.72	15.60	16.25	15.89	14.66	14.57	15.58	15.40
2.179	0.81	1.75	Q64324	Syntaxin-binding protein 2	Stxbp2	2430	45	45	45	74.5	74.5	74.5	66.357	0.0000	323.31	216800000	1226	18.00	17.79	18.64	18.26	17.31	17.18	17.54	17.42
0.820	0.80	1.75	Q9CQ26	STAM-binding protein	Stambp	4556	8	8	8	26.7	26.7	26.7	48.513	0.0000	23.25	7620400	92	15.52	14.84	15.39	15.16	15.04	13.20	15.25	14.20
1.691	0.80	1.75	P97742	Carnitine O-palmitoyltransferase 1, liver isoform	Cpt1a	1666	29	29	28	42.6	42.6	41.1	88.25	0.0000	309.87	77048000	482	17.46	17.32	17.97	16.92	16.86	16.79	16.55	16.25
1.212	0.80	1.75	Q8CFI7	DNA-directed RNA polymerase II subunit RPB2	Polr2b	3489	32	32	32	35.2	35.2	35.2	133.91	0.0000	291.89	51574000	558	16.63	15.93	17.32	16.98	15.85	15.70	16.46	15.64
0.493	0.80	1.74	P11440	Cyclin-dependent kinase 1	Cdk1	744	12	10	10	54.2	47.8	47.8	34.106	0.0000	40.695	11099000	99	15.04	15.41	13.73	15.50	15.18	12.33	14.55	14.42
2.324	0.80	1.74	Q6PD26	GPI transamidase component PIG-S	Pigs	2609	11	11	11	34.2	34.2	34.2	61.71	0.0000	115.2	14674000	108	15.61	15.97	16.25	15.77	15.09	14.76	15.26	15.28
0.783	0.80	1.74	Q9EPU4	Cleavage and polyadenylation specificity factor subunit 1	Cpsf1	5265	23	23	23	21.5	21.5	21.5	160.82	0.0000	100.41	15260000	194	15.35	15.13	17.02	15.40	14.84	14.26	15.48	15.13
1.911	0.80	1.74	Q11011	Puromycin-sensitive aminopeptidase	Npepps	1782	50	50	50	56.7	56.7	56.7	103.32	0.0000	323.31	144400000	1046	17.24	17.02	17.96	17.46	16.55	16.42	16.92	16.58
1.599	0.80	1.74	Q5SW19	Clustered mitochondria protein homolog	Cluh	2153	17	17	17	17.3	17.3	17.3	148.07	0.0000	43.816	12518000	129	15.20	14.54	15.02	15.16	14.17	13.89	14.82	13.85
1.184	0.80	1.74	Q91WK5	Glycine cleavage system H protein, mitochondrial	Gcsh	4117	2	2	2	13.5	13.5	13.5	18.637	0.0000	20.437	2109200	28	14.21	14.67	14.34	15.06	14.19	13.09	14.35	13.45
1.668	0.80	1.74	P35123	Ubiquitin carboxyl-terminal hydrolase 4	Usp4	1010	27	26	26	36.5	35.6	35.6	108.34	0.0000	166.72	73239000	440	19.07	18.87	19.96	19.00	18.36	18.25	18.62	18.48
0.928	0.80	1.74	Q9WVL3	Solute carrier family 12 member 7	Slc12a7	5756	10	10	10	12.4	12.4	12.4	119.48	0.0000	91.93	7727000	108	14.58	14.59	15.60	15.07	14.23	13.91	15.12	13.38
1.507	0.80	1.74	Q9DBG6	Dolichyl-diphosphooligosaccharide--protein glycosyltransferase subunit	Rpn2	5153	29	29	29	50.7	50.7	50.7	69.062	0.0000	323.31	254170000	912	18.81	18.72	19.76	18.90	18.23	17.94	18.68	18.15
2.126	0.80	1.74	Q6P1F6	Serine/threonine-protein phosphatase 2A 55 kDa regulatory subunit B a	Ppp2r2a	2554	18	18	13	50.1	50.1	38	51.691	0.0000	225.67	69068000	398	17.68	17.21	18.09	17.70	17.03	16.71	17.03	16.73
0.796	0.80	1.74	P27600	Guanine nucleotide-binding protein subunit alpha-12	Gna12	935	9	8	8	29.8	27.7	27.7	44.095	0.0000	19.197	4855800	40	14.57	13.76	16.00	14.35	13.52	14.24	13.93	13.79
0.888	0.80	1.74	Q9CR39	WD repeat domain phosphoinositide-interacting protein 3	Wdr45b	4664	4	4	4	10.8	10.8	10.8	38.02	0.0000	11.466	6573500	70	15.07	14.32	15.24	15.33	14.24	14.73	14.72	13.07
0.449	0.80	1.74	Q9CWZ3	RNA-binding protein 8A	Rbm8a	4740	4	4	4	32.2	32.2	32.2	19.889	0.0000	112.82	3093000	26	11.50	14.79	12.27	13.41	12.89	11.41	11.82	12.66
1.566	0.80	1.74	Q6PF93	Phosphatidylinositol 3-kinase catalytic subunit type 3	Pik3c3	2626	18	18	18	30.4	30.4	30.4	101.49	0.0000	78.232	14882000	184	15.19	14.76	15.80	15.54	14.38	14.74	14.83	14.15
1.173	0.79	1.74	Q9CQR6	Serine/threonine-protein phosphatase 6 catalytic subunit;Serine/threoni	Ppp6c	4626	15	15	15	60.3	60.3	60.3	35.159	0.0000	104.12	48077000	363	16.87	16.69	17.97	16.74	16.43	15.92	16.73	16.03
0.449	0.79	1.73	P19536	Cytochrome c oxidase subunit 5B, mitochondrial	Cox5b	848	7	7	7	43	43	43	13.813	0.0004	5.1791	8074300	26	15.84	16.14	17.29	15.81	16.23	16.51	13.38	15.77
1.654	0.79	1.73	P26516	26S proteasome non-ATPase regulatory subunit 7	Psmd7	926	16	16	16	50.5	50.5	50.5	36.539	0.0000	285.93	63018000	353	18.14	17.66	18.77	18.58	17.42	17.71	17.44	17.40

0.639	0.79	1.73	Q3UGC7;Q6	Eukaryotic translation initiation factor 3 subunit J-A;Eukaryotic translatio	Eif3j1;Eif3j2	1960	10	10	10	57.5	57.5	57.5	29.343	0.0000	144.42	6823600	80	14.84	15.14	16.34	14.57	14.55	14.78	15.24	13.16
0.741	0.79	1.73	P70426	GTP-binding protein Rit1	Rit1	1571	5	5	5	32.4	32.4	32.4	25.169	0.0000	20.434	2214600	41	13.15	13.26	15.26	14.21	13.29	12.74	13.10	13.60
0.835	0.79	1.73	Q7M753		Pank2	2738	5	5	5	14.7	14.7	14.7	50.212	0.0000	28.071	3720900	51	14.10	14.35	15.85	14.18	13.82	14.22	13.18	14.10
0.474	0.79	1.73	Q3TSG4	RNA demethylase ALKBH5	Alkbh5	1873	5	5	5	17.2	17.2	17.2	44.41	0.0000	132.75	9034400	103	14.82	14.78	15.93	15.25	15.14	14.07	15.83	12.59
0.921	0.79	1.73	Q61214	Dual specificity tyrosine-phosphorylation-regulated kinase 1A	Dyrk1a	2280	8	8	7	11.3	11.3	9.2	85.493	0.0000	20.427	3701700	38	14.07	13.36	14.56	14.04	13.01	14.28	12.81	12.77
0.745	0.79	1.72	Q8BQZ4	Ral GTPase-activating protein subunit beta	Ralgapb	3174	9	9	9	9.1	9.1	9.1	165.2	0.0000	24.901	3693900	39	14.71	14.26	15.86	14.54	14.77	13.00	14.38	14.08
1.008	0.78	1.72	Q80X90	Filamin-B	Flnb	2876	54	47	46	31.1	29.9	29.4	277.82	0.0000	192.54	46813000	424	16.06	15.74	17.25	16.46	15.27	15.19	16.20	15.72
1.697	0.78	1.72	Q62018	RNA polymerase-associated protein CTR9 homolog	Ctr9	2346	9	9	9	12.8	12.8	12.8	133.41	0.0000	48.939	5371200	87	14.32	14.02	15.00	14.96	13.86	13.79	13.92	13.60
0.963	0.78	1.72	P53995	Anaphase-promoting complex subunit 1	Anapc1	1244	9	9	9	8.2	8.2	8.2	215.99	0.0000	38.677	7654200	60	15.02	14.61	15.10	14.91	14.70	13.02	14.75	14.03
1.658	0.78	1.72	Q8VCM8	Nicalin	Ncln	3927	17	17	17	33.9	33.9	33.9	62.907	0.0000	181.82	34954000	300	16.80	16.18	17.40	16.81	16.06	16.12	16.02	15.86
1.197	0.78	1.72	Q9D5T0	ATPase family AAA domain-containing protein 1	Atad1	4985	9	9	9	31.9	31.9	31.9	40.744	0.0000	97.208	18570000	211	15.58	15.22	15.61	15.28	15.02	14.87	15.01	13.66
0.328	0.78	1.72	Q8BWY7	Zinc transporter ZIP11	Slc39a11	3271	3	3	3	8.8	8.8	8.8	35.466	0.0000	7.1718	8798100	72	16.22	15.46	13.43	15.58	15.66	12.69	15.90	13.31
1.041	0.78	1.72	Q8CJG1	Protein argonaute-1	Ago1	3572	14	9	7	22.4	15.3	12	97.213	0.0000	24.977	4970100	67	14.13	14.00	15.63	14.57	13.76	13.84	14.09	13.51
1.813	0.78	1.72	Q9JL56	Glycerophosphodiester phosphodiesterase 1	Gde1	5472	5	5	5	17.5	17.5	17.5	37.629	0.0000	9.6362	4802000	38	15.41	14.99	15.17	15.02	14.88	13.86	14.26	14.47
1.541	0.78	1.72	Q922Q9	Chitinase domain-containing protein 1	Chid1	4272	10	10	10	30	30	30	44.905	0.0000	30.398	13902000	133	15.86	15.21	15.40	15.86	15.13	15.20	14.30	14.59
1.552	0.78	1.72	A6H611	Mitochondrial intermediate peptidase	Mipep	61	6	6	6	10.1	10.1	10.1	80.851	0.0000	19.92	6390800	53	15.70	15.54	15.43	15.83	15.28	14.20	15.23	14.68
0.421	0.78	1.71	Q6DFX2	Anthrax toxin receptor 2	Antxr2	2507	3	3	3	11.3	11.3	11.3	53.184	0.0000	9.7942	1926100	24	13.30	12.61	15.76	13.87	13.81	11.75	13.24	13.62
1.753	0.78	1.71	Q91VJ4	Serine/threonine-protein kinase 38	Stk38	4065	23	23	20	46	46	41.9	54.174	0.0000	134.86	44681000	468	15.93	15.33	16.30	16.32	15.04	15.17	15.36	15.20
0.527	0.78	1.71	Q78HU3	Multivesicular body subunit 12A	Mvb12a	2721	4	4	4	19.2	19.2	19.2	28.705	0.0000	40.514	12318000	111	15.77	15.73	16.38	15.22	15.83	15.29	15.75	13.12
0.480	0.78	1.71	P51125	Calpastatin	Cast	1190	25	25	25	41.5	41.5	41.5	84.921	0.0000	323.31	25823000	227	15.74	16.54	16.70	15.71	16.32	15.10	16.59	13.59
1.119	0.77	1.71	Q8BMG7	Rab3 GTPase-activating protein non-catalytic subunit	Rab3gap2	3136	26	26	26	29.6	29.6	29.6	152.53	0.0000	136.12	26868000	343	15.60	14.94	16.60	15.71	14.96	14.60	15.12	15.07
1.113	0.77	1.71	P04441	H-2 class II histocompatibility antigen gamma chain	Cd74	642	13	13	13	42.7	42.7	42.7	31.557	0.0000	173.89	78060000	229	18.31	18.26	19.61	18.55	18.29	17.84	17.44	18.08
1.189	0.77	1.71	Q8VBT0	Thioredoxin-related transmembrane protein 1	Tmx1	3894	9	9	9	24.8	24.8	24.8	31.395	0.0000	35.203	39566000	251	17.08	16.41	17.67	17.25	16.58	16.27	16.74	15.74
0.706	0.77	1.71	P22315	Ferrochelatase, mitochondrial	Fech	873	9	9	9	36.9	36.9	36.9	47.13	0.0000	81.086	28311000	300	16.33	15.59	16.53	16.57	15.65	14.09	15.95	16.25
0.163	0.77	1.71	Q01149	Collagen alpha-2(I) chain	Col1a2	1705	5	5	5	5.2	5.2	5.2	129.56	0.0000	15.271	5083100	19	16.49	16.98	11.50	12.36	12.37	16.98	13.07	11.83
0.790	0.77	1.70	Q8BTY8	Sec1 family domain-containing protein 2	Scfd2	3213	9	9	9	23.2	23.2	23.2	74.75	0.0000	84.081	10207000	111	15.39	15.20	16.87	15.42	15.33	14.13	15.35	15.00
1.264	0.77	1.70	Q8R2N2	Cirhin	Cirh1a	3811	4	4	4	10.2	10.2	10.2	76.908	0.0000	22.249	2025200	36	14.38	13.69	15.11	14.50	13.85	13.38	13.44	13.94
0.354	0.77	1.70	P62309	Small nuclear ribonucleoprotein G	Snrpg	1419	5	5	5	77.6	77.6	77.6	8.496	0.0000	36.327	14939000	82	16.58	16.85	15.77	16.29	16.43	12.91	16.37	16.72
0.316	0.77	1.70	Q9JLC8	Sacsin	Sacs	5477	7	7	7	1.4	1.4	1.4	520.68	0.0011	3.6222	9963500	30	15.52	16.22	15.17	15.43	16.14	15.51	15.79	11.84
1.679	0.77	1.70	O88544	COP9 signalosome complex subunit 4	Cops4	544	28	28	28	80.5	80.5	80.5	46.284	0.0000	323.31	98937000	601	17.72	17.21	18.37	17.66	16.87	17.15	16.95	16.92
1.531	0.77	1.70	Q60759	Glutaryl-CoA dehydrogenase, mitochondrial	Gcdh	2202	9	9	9	37	37	37	48.605	0.0000	79.848	7499200	70	15.44	14.85	16.11	15.29	14.57	14.62	14.85	14.58
0.887	0.77	1.70	Q8BMQ8	Vacuolar fusion protein MON1 homolog B	Mon1b	3147	5	5	5	21.5	21.5	21.5	60.015	0.0000	8.6104	1269700	9	13.58	13.30	14.59	13.91	12.76	12.29	13.58	13.70
1.073	0.77	1.70	Q3UDE2	Tubulin--tyrosine ligase-like protein 12	Ttll12	1946	18	18	18	41	41	41	74.042	0.0000	185.36	36517000	291	16.58	16.05	17.48	17.05	15.95	15.59	16.59	15.97
1.533	0.77	1.70	Q9CR62	Mitochondrial 2-oxoglutarate/malate carrier protein	Slc25a11	4674	22	22	22	71	71	71	34.155	0.0000	238.4	138160000	584	18.59	18.31	19.40	18.91	18.33	17.87	17.77	18.18
1.224	0.76	1.70	Q9QZ88	Vacuolar protein sorting-associated protein 29	Vps29	5587	9	9	9	48.4	48.4	48.4	20.495	0.0000	116.87	86322000	258	18.20	18.41	17.81	19.06	17.87	17.46	17.10	17.99
1.612	0.76	1.70	Q52KI8	Serine/arginine repetitive matrix protein 1	Srrm1	2084	8	8	8	12.5	12.5	12.5	106.86	0.0000	90.967	14379000	129	17.16	16.59	16.70	17.42	16.38	16.41	16.33	15.71
0.778	0.76	1.70	P53690	Matrix metalloproteinase-14	Mmp14	1235	8	8	8	14.1	14.1	14.1	65.918	0.0000	15.721	6170600	71	14.91	14.74	13.33	14.38	13.62	14.09	13.96	12.63
0.476	0.76	1.69	Q4KML4	Costars family protein ABRACL	Abracl	2061	3	3	3	46.9	46.9	46.9	9.0303	0.0000	29.25	6622700	76	15.64	16.17	16.48	15.92	15.39	13.40	15.58	16.80
0.841	0.76	1.69	Q8BWM0	Prostaglandin E synthase 2;Prostaglandin E synthase 2 truncated form	Ptges2	3262	8	8	8	34.6	34.6	34.6	43.323	0.0000	145.95	12733000	137	16.06	15.62	14.90	16.26	15.12	14.32	15.83	14.52
1.423	0.76	1.69	Q91VB4	Hermansky-Pudlak syndrome 3 protein homolog	Hps3	4054	5	5	5	6.1	6.1	6.1	113.18	0.0000	10.764	3606300	70	14.09	13.47	14.57	14.15	13.01	13.00	13.60	13.64
0.589	0.76	1.69	E2JF22	Piezo-type mechanosensitive ion channel component 1	Piezo1	218	6	6	6	3.8	3.8	3.8	292	0.0000	23.026	4982100	52	14.62	14.29	13.61	13.97	14.58	12.53	14.07	12.27
2.118	0.76	1.69	Q8BJU0	Small glutamine-rich tetratricopeptide repeat-containing protein alpha	Sgta	3086	6	6	6	24.4	24.4	24.4	34.322	0.0000	9.968	8073600	73	14.95	15.40	15.83	15.50	14.80	14.51	14.71	14.62
1.213	0.76	1.69	Q8C2E7	WASH complex subunit strumpellin	Kiaa0196	3370	38	38	37	35	35	33.6	134.11	0.0000	165.31	67823000	620	16.30	16.09	17.30	16.75	15.99	15.48	16.33	15.61
1.272	0.76	1.69	P21956	Lactadherin	Mfge8	869	16	16	16	42.1	42.1	42.1	51.24	0.0000	112.09	58828000	393	17.65	17.46	18.26	17.66	17.48	17.42	16.61	16.47
0.631	0.76	1.69	Q64133	Amine oxidase [flavin-containing] A	Maoa	2420	20	20	20	40.9	40.9	40.9	59.601	0.0000	176.5	42252000	270	16.24	16.13	18.24	16.35	16.28	16.60	15.41	15.62
1.268	0.76	1.69	Q91VJ1	Interferon-inducible protein AIM2	Aim2	4064	9	9	9	41	41	41	40.155	0.0000	62.365	5788100	60	14.69	13.88	15.12	14.74	13.45	14.30	13.93	13.72
0.855	0.76	1.69	P62960	Nuclease-sensitive element-binding protein 1	Ybx1	1472	11	11	5	52.2	52.2	29.2	35.73	0.0000	323.31	35578000	272	17.65	17.28	19.17	17.57	16.79	17.36	17.12	17.36
0.519	0.76	1.69	Q9D6K9	Ceramide synthase 5	Cers5	5001	7	7	7	16.2	16.2	16.2	48.166	0.0000	58.647	13843000	78	15.65	15.15	16.97	15.74	15.41	13.52	15.52	16.03
1.804	0.76	1.69	Q9CZU6	Citrate synthase, mitochondrial	Cs	4840	27	27	27	59.9	59.9	59.9	51.736	0.0000	323.31	461630000	814	19.87	19.84	20.35	20.09	19.57	18.72	19.51	19.32
1.012	0.76	1.69	P18052	Receptor-type tyrosine-protein phosphatase alpha	Ptpra	833	15	14	14	28.8	26.7	26.7	93.696	0.0000	105.77	15395000	148	15.81	16.02	15.86	15.52	15.35	15.04	15.76	14.02
0.779	0.76	1.69	Q8K1X1	WD repeat-containing protein 11	Wdr11	3635	29	29	29	33.8	33.8	33.8	135.94	0.0000	256.77	26999000	322	16.06	15.82	16.37	16.07	15.70	13.94	16.00	15.64
2.070	0.76	1.69	Q91ZE0	Trimethyllysine dioxygenase, mitochondrial	Tmlhe	4194	12	12	12	31.4	31.4	31.4	49.609	0.0000	88.719	19451000	210	15.86	15.89	15.46	15.74	15.06	14.50	15.30	15.06
0.800	0.75	1.69	Q9QZC2	Plexin-C1	Plxnc1	5591	7	7	7	5.1	5.1	5.1	176.47	0.0000	15.225	7000700	77	15.00	13.97	15.39	14.88	14.86	13.80	13.20	14.37
0.868	0.75	1.69	B2RY04	Dedicator of cytokinesis protein 5	Dock5	88	19	18	17	15.7	15.1	14.2	214.43	0.0000	80.52	10946000	109	15.29	14.68	16.11	15.47	13.80	14.44	15.24	15.05
0.729	0.75	1.69	Q08024	Core-binding factor subunit beta	Cbfb	1759	12	12	12	63.1	63.1	63.1	22.03	0.0000	35.838	11158000	109	15.74	15.20	17.40	16.03	14.86	15.71	15.57	15.21
0.378	0.75	1.69	Q9QX60	Deoxyguanosine kinase, mitochondrial	Dguok	5533	4	4	4	22	22	22	32.281	0.0000	140.24	5615300	47	15.13	14.78	13.51	15.47	15.19	12.28	15.29	13.11
1.457	0.75	1.69	Q3UMB5	Smith-Magenis syndrome chromosomal region candidate gene 8 proteir	Smcr8	1998	13	13	13	17.6	17.6	17.6	104.96	0.0000	44.687	8543300	126	14.57	14.25	15.05	14.70	13.97	13.75	14.45	13.39
1.407	0.75	1.69	Q9DBL1	Short/branched chain specific acyl-CoA dehydrogenase, mitochondrial	Acadsb	5159	20	20	20	54.4	54.4	54.4	47.874	0.0000	323.31	48374000	374	17.17	16.76	17.93	17.23	16.66	16.41	16.85	16.16
2.026	0.75	1.69	Q60870	Receptor expression-enhancing protein 5	Reep5	2217	11	11	11	27	27	27	21.05	0.0000	240	113120000	507	18.08	17.60	18.02	18.39	17.31	17.20	17.56	17.00
1.084	0.75	1.69	Q3TCX3	UPF0469 protein KIAA0907	Kiaa0907	1830	3	3	3	7	7	7	64.562	0.0000	7.2271	1697200	24	13.93	13.74	13.95	13.76	12.45	14.09	12.74	13.07
1.675	0.75	1.69	Q60931	Voltage-dependent anion-selective channel protein 3	Vdac3	2223	22	22	22	73.1	73.1	73.1	30.752	0.0000	323.31	180230000	703	18.88	18.69	19.38	19.09	18.63	17.97	18.54	17.88
0.898	0.75	1.68	Q8CFI0	E3 ubiquitin-protein ligase NEDD4-like	Nedd4l	3487	10	10	10	15.3	15.3	15.3	115.42	0.0000	24.342	7237500	60	15.51	15.96	16.21	15.30	14.65	16.03	14.33	14.96
0.578	0.75	1.68	Q9CQN6	Transmembrane protein 14C	Tmem14c	4616	3	3	3	50	50	50	11.642	0.0000	16.032	4899400	62	14.19	15.45	15.43	15.81	14.75	14.17	13.30	15.66
1.541	0.75	1.68	Q9JL62	Glycolipid transfer protein	Gltp	5473	10	10	10	49.8	49.8	49.8	23.689	0.0000	61.053	79385000	350	18.83	18.71	19.78	19.28	18.41	18.33	18.66	18.21
1.029	0.75	1.68	B2RXR6	Serine/threonine-protein phosphatase 6 regulatory ankyrin repeat subur	Ankrd44	85	32	32	31	35.8	35.8	34.7	107.38	0.0000	156.1	49594000	551	16.50	15.96	17.59	16.60	15.82	15.76	16.39	15.66
0.459	0.75	1.68	P0C7N9	Proteasome assembly chaperone 4	Psmg4	708	4	4	4	44.7	44.7	44.7	13.974	0.0000	12.627	3430500	40	14.29	14.38	15.26	14.84	13.67	12.10	14.66	15.33
2.036	0.75	1.68	Q64521	Glycerol-3-phosphate dehydrogenase, mitochondrial	Gpd2	2453	45	45	45	65.6	65.6	65.6	80.953	0.0000	323.31	148980000	855	17.74	17.48	18.31	17.80	16.86	17.33	17.06	17.08
0.658	0.75	1.68	Q80TL7	Protein MON2 homolog	Mon2	2807	9	9	9	9	9	9	189.08	0.0000	58.786	9242400	83	15.36	15.14	16.95	14.95	14.46	14.71	15.74	14.50
1.415	0.75	1.68	Q920B9	FACT complex subunit SPT16	Supt16h	4209	39	39	39	40.8	40.8	40.8	119.82	0.0000	314.76	70212000	642	16.50	16.19	17.48	16.80	15.82	16.05	16.11	16.00
0.789	0.75	1.68	P58802	TBC1 domain family member 10A	Tbc1d10a	1324	5	5	5	13.4	13.4	13.4	56.202	0.0000	13.766	3416100	46	14.04	12.73	13.82	14.01	12.91	13.11	13.65	11.95
1.435	0.75	1.68	P97390	Vacuolar protein sorting-associated protein 45	Vps45	1643	16	16	16	33	33	33	65.052	0.0000	125.22	24545000	279	15.74	15.29	16.56	15.94	15.19	14.86	15.15	15.33
1.687	0.75	1.68	Q91YJ2	Sorting nexin-4	Snx4	4161	21	21	21	45.3	45.3	45.3	51.777	0.0000	149.75	42073000	401	16.38	15.80	16.84	16.47	15.70	15.38	15.88	15.54
0.861	0.75	1.68	Q7TNG5	Echinoderm microtubule-associated protein-like 2	Eml2	2762	16	16	16	36.1	36.1	36.1	70.733	0.0000	68.971	19991000	152	15.71	16.43	17.39	16.79	15.56	16.60	15.69	15.48
1.199	0.75	1.68	Q6ZPY7	Lysine-specific demethylase 3B	Kdm3b	2686	9	9	8	10.7	10.7	10	170.87	0.0000	47.317	7042300	53	15.19	15.31	16.46	15.82	14.79	14.71	15.41	14.87
2.544	0.75	1.68	Q68FD5	Clathrin heavy chain 1	Cltc	2473	133	133	133	68.2	68.2	68.2	191.55	0.0000	323.31	2046700000	5207	20.42	20.25	20.75	20.50	19.95	19.51	19.91	19.56
0.595	0.75	1.68	Q8C2K1	Differentially expressed in FDCP 6	Def6	3371	22	22	22	33.7	33.7	33.7	73.453	0.0000	48.214	18645000	204	15.05	14.84	17.38	15.48	14.76	15.18	15.10	14.73
1.095	0.74	1.68	Q99J31	Oligophrenin-1	Ophn1	4321	4	4	4	6.6	6.6	6.6	91.984	0.0002	5.8736	1901600	20	13.83	13.51	12.74	13.66	12.66	12.77	12.03	13.30
0.399	0.74	1.67	Q91YT7	YTH domain-containing family protein 2	Ythdf2	4179	10	5	4	16.2	9.3	7.4	62.279	0.0004	4.3449	5067500	32	14.96	15.05	12.67	14.77	14.29	14.92	12.84	12.42
1.403	0.74	1.67	P61982	14-3-3 protein gamma;14-3-3 protein gamma, N-terminally processed	Ywhag	1399	36	31	28	96	93.1	93.1	28.302	0.0000	323.31	741920000	1250	20.84	20.40	21.70	20.70	20.08	20.25	20.28	20.05
0.731	0.74	1.67	Q9DB60	Prostamide/prostaglandin F synthase	Fam213b	5129	5	5	5	26.9	26.9	26.9	21.67	0.0000	14.688	13875000	111	15.99	15.91	15.89	16.07	15.49	13.77	15.65	15.98
1.332	0.74	1.67	Q8C1E7	Transmembrane protein 120A	Tmem120a	3365	7	7	6	24.2	24.2	20.1	40.751	0.0000	51.563	16569000	163	15.69	15.28	15.78	15.67	15.41	14.94	15.00	14.10
2.803	0.74	1.67	Q6P5E4	UDP-glucose:glycoprotein glucosyltransferase 1	Uggt1	2574	76	76	76	61.1	61.1	61.1	176.43	0.0000	323.31	278110000	1526	18.64	18.32	18.86	18.77	17.92	17.83	18.09	17.79
0.812	0.74	1.67	Q9CWS4	Integrator complex subunit 11	Cpsf3l	4728	5	5	5	13.2	13.2	13.2	67.843	0.0000	21.505	3949500	29	13.85	14.21	15.86	14.35	13.98	13.75	13.98	13.59
1.187	0.74	1.67	P52825	Carnitine O-palmitoyltransferase 2, mitochondrial	Cpt2	1224	26	26	26	50.8	50.8	50.8	73.98	0.0000	220.3	42375000	396	16.45	16.18	17.53	16.61	16.14	15.81	16.26	15.61
2.439	0.74	1.67	Q99L04	Dehydrogenase/reductase SDR family member 1	Dhrs1	4402	31	31	31	85	85	85	34.005	0.0000	323.31	271520000	796	19.52	19.44	19.66	19.06	18.61	18.96	18.53	18.61
0.910	0.74	1.67	O55201	Transcription elongation factor SPT5	Supt5h	463	39	39	39	42.6	42.6	42.6	120.66	0.0000	247.47	50889000	501	16.03	15.65	17.43	16.02	15.31	15.41	15.87	15.59
0.527	0.74	1.67	O55091	Protein IMPACT	Impact	449	5	5	5	19.8	19.8	19.8	36.276	0.0000	22.39	3519600	48	13.78	14.02	15.89	14.37	13.60	13.52	12.96	15.02
0.594	0.74	1.67	Q9JJK2	LanC-like protein 2	Lancl2	5430	8	8	8	26.7	26.7	26.7	50.777	0.0000	89.079	19173000	144	15.95	15.96	15.88	16.32	15.72	15.57	16.25	13.61
0.936	0.74	1.67	Q8CGA0	Protein phosphatase 1F	Ppm1f	3502	8	8	8	28.8	28.8	28.8	49.61	0.0000	48.23	7276000	60	14.84	14.76	15.64	14.63	13.25	14.57	14.66	14.43
1.191	0.74	1.66	P26450	Phosphatidylinositol 3-kinase regulatory subunit alpha	Pik3r1	925	27	26	26	39.1	37.2	37.2	83.516	0.0000	137.4	47714000	324	17.89	17.66	19.11	18.17	17.41	17.39	17.68	17.41
2.022	0.73	1.66	P51660	Peroxisomal multifunctional enzyme type 2;(3R)-hydroxyacyl-CoA dehy	Hsd17b4	1199	39	39	39	58.8	58.8	58.8	79.481	0.0000	323.31	242740000	1093	18.47	18.37	19.07	18.31	18.04	17.86	17.73	17.65
1.247	0.73	1.66	Q9R1P1	Proteasome subunit beta type-3	Psmb3	5654	11	11	11	53.7	53.7	53.7	22.965	0.0000	169.58	125470000	413	19.50	19.43	20.74	19.73	19.14	19.30	18.97	19.06
0.884	0.73	1.66	Q5MJS3;Q8	Extracellular serine/threonine protein kinase FAM20C;Protein FAM20A	Fam20c;Fam20a	2114	2	2	2	6.9	6.9	6.9	65.765	0.0000	6.8122	920770	8	14.22	13.59	13.80	13.68	13.55	12.51	13.97	12.34
0.467	0.73	1.66	Q6PCP5	Mitochondrial fission factor	Mff	2606	10	10	10	56	56	56	32.931	0.0000	133.21	14398000	104	16.03	15.48	17.14	15.73	15.36	16.54	15.86	13.69
2.285	0.73	1.66	P97370	Sodium/potassium-transporting ATPase subunit beta-3	Atp1b3	1635	12	12	12	38.5	38.5	38.5	31.775	0.0000	41.707	63159000	216	17.83	17.92	17.92	18.10	17.33	16.73	17.38	17.41
1.487	0.73	1.66	Q9QY36;Q3	N-alpha-acetyltransferase 10;N-alpha-acetyltransferase 11	Naa10;Naa11	5561	8	8	8	39.1	39.1	39.1	26.519	0.0000	60.462	20001000	170	16.52	15.94	17.18	16.61	15.86	15.72	16.02	15.71
1.392	0.73	1.66	Q5U3K5	Rab-like protein 6	Rabl6	2162	7	7	7	13.9	13.9	13.9	79.83	0.0000	11.85	4612500	45	14.53	13.60	14.67	14.68	13.64	13.64	13.91	13.36
0.963	0.73	1.66	Q8R0G9	Nuclear pore complex protein Nup133	Nup133	3759	29	29	29	37.9	37.9	37.9	128.62	0.0000	276.28	33160000	298	17.15	16.64	18.15	17.28	16.07	17.06	16.85	16.33
1.480	0.73	1.66	Q9D554	Splicing factor 3A subunit 3	Sf3a3	4982	26	26	26	51.7	51.7	51.7	58.841	0.0000	115.61	69793000	431	17.42	17.35	18.27	17.50	16.47	17.15	16.92	17.09
0.782	0.73	1.66	Q9ET30	Transmembrane 9 superfamily member 3	Tm9sf3	5356	9	9	9	18.6	18.6	18.6	67.544	0.0000	73.059	29737000	177	16.24	16.30	18.04	16.10	15.86	15.86	16.08	15.97
1.001	0.73	1.66	B1AVY7	Kinesin-like protein KIF16B	Kif16b	70	9	9	9	8.8	8.8	8.8	150.06	0.0000	129.33	8889100	139	14.87	14.49	15.92	14.86	14.49	13.68	14.41	14.64
1.484	0.73	1.66	P19157	Glutathione S-transferase P 1	Gstp1	843	8	8	8	51.9	51.9	51.9	23.609	0.0000	224.21	87875000	291	18.13	18.19	17.86	18.72	17.68	16.96	17.85	17.50
1.294	0.73	1.65	Q9QUK4	Baculoviral IAP repeat-containing protein 1b	Naip2	5517	12	12	12	13.5	13.5	13.5	164.08	0.0000	53.067	9705900	97	14.56	14.35	15.48	14.88	14.01	13.92	14.58	13.83
1.399	0.73	1.65	P50431	Serine hydroxymethyltransferase, cytosolic	Shmt1	1183	11	10	10	32	29.1	29.1	52.6	0.0000	30.309	10398000	82	15.83	15.34	16.47	16.18	15.08	15.10	15.10	15.63
1.688	0.73	1.65	Q9QZE7	Translin-associated protein X	Tsnax	5596	22	22	22	79	79	79	32.926	0.0000	227.54	52044000	432	16.86	16.55	17.44	16.81	16.50	15.99	16.33	15.93
0.902	0.73	1.65	Q80X82	Symplekin	Sympk	2874	14	14	14	17.7	17.7	17.7	142.62	0.0000	88.8	10836000	152	15.17	15.02	16.42	15.22	14.39	14.82	15.40	14.32
0.449	0.73	1.65	Q5XG71	Small subunit processome component 20 homolog	Utp20	2170	12	12	12	6.3	6.3	6.3	317.74	0.0000	17.792	14107000	72	16.13	15.96	17.78	16.36	16.37	16.15	16.73	14.08
0.940	0.73	1.65	Q9CXE7	Transmembrane emp24 domain-containing protein 5	Tmed5	4753	4	4	4	17	17	17	26.172	0.0000	223.7	17076000	84	16.59	16.18	16.76	16.86	15.87	16.61	14.88	16.12

1.915	0.73	1.65	Q9JLI6	Selenocysteine lyase	Scly	5480	19	19	18	48.1	48.1	47.2	47.173	0.0000	168.39	31824000	378	15.84	15.43	16.28	16.12	15.17	15.15	15.44	15.02
0.859	0.72	1.65	Q99LB2	Dehydrogenase/reductase SDR family member 4	Dhrs4	4413	8	8	8	25.8	25.8	25.8	29.884	0.0000	17.097	12772000	147	15.25	14.90	16.71	15.03	14.86	14.54	14.79	14.79
2.129	0.72	1.65	Q9CQC6	Basic leucine zipper and W2 domain-containing protein 1	Bzw1	4582	22	22	21	58.7	58.7	57	48.043	0.0000	277.82	73775000	324	17.90	17.89	18.58	17.98	17.13	17.44	17.47	17.41
0.712	0.72	1.65	Q8VC03	Echinoderm microtubule-associated protein-like 3	Eml3	3903	17	17	17	29.9	29.9	29.9	95.694	0.0000	111.31	18365000	222	15.46	14.79	16.97	15.65	14.45	15.17	15.04	15.33
2.325	0.72	1.65	Q9Z2U0	Proteasome subunit alpha type-7	Psma7	5864	23	23	23	70.6	70.6	70.6	27.855	0.0000	323.31	226740000	663	19.06	18.96	19.27	19.07	18.57	17.95	18.61	18.33
0.367	0.72	1.65	P56391	Cytochrome c oxidase subunit 6B1	Cox6b1	1284	3	3	3	53.5	53.5	53.5	10.071	0.0004	4.8241	8940800	32	13.49	16.71	16.15	16.08	14.91	15.88	15.10	13.65
1.950	0.72	1.65	O55029	Coatomer subunit beta	Copb2	445	50	50	50	61.2	61.2	61.2	102.45	0.0000	323.31	240470000	963	18.92	19.00	19.66	18.94	18.52	18.40	18.56	18.15
0.845	0.72	1.65	Q91XD7	Cysteine-rich with EGF-like domain protein 1	Creld1	4143	3	3	3	7.6	7.6	7.6	45.717	0.0000	29.405	3595200	41	13.67	14.31	14.98	13.88	13.53	13.31	14.32	12.79
1.393	0.72	1.65	P97814	Proline-serine-threonine phosphatase-interacting protein 1	Pstpip1	1675	17	17	17	32.5	32.5	32.5	47.59	0.0000	29.465	34811000	246	16.71	16.28	17.16	16.59	16.25	16.38	15.70	15.52
1.057	0.72	1.65	Q8BLN5	Lanosterol synthase	Lss	3120	22	22	21	36.4	36.4	35.1	83.14	0.0000	75.892	36188000	286	16.16	15.94	16.49	16.79	15.64	14.83	16.30	15.72
2.250	0.72	1.65	Q91VC3	Eukaryotic initiation factor 4A-III;Eukaryotic initiation factor 4A-III, N-term	Eif4a3	4055	37	33	33	64.2	58.2	58.2	46.839	0.0000	323.31	136890000	776	18.22	18.23	18.93	18.50	17.66	17.73	17.85	17.74
1.017	0.72	1.65	Q8K274	Ketosamine-3-kinase	Fn3krp	3647	6	6	6	26.9	26.9	26.9	34.468	0.0000	29.815	7143500	88	15.63	15.02	16.06	15.28	15.07	15.32	14.72	13.99
2.810	0.72	1.65	Q91YH5	Atlastin-3	Atl3	4157	21	21	20	52.3	52.3	51	60.574	0.0000	323.31	64441000	411	17.77	17.60	17.93	17.70	16.96	17.01	17.34	16.81
0.322	0.72	1.65	Q8C0D4	Rho GTPase-activating protein 12	Arhgap12	3331	8	8	8	14.2	14.2	14.2	95.351	0.0000	16.856	9925300	31	15.80	14.87	16.33	15.82	14.90	12.47	16.26	16.31
0.787	0.72	1.65	Q99JF5	Diphosphomevalonate decarboxylase	Mvd	4337	12	12	12	47.4	47.4	47.4	44.072	0.0000	158.14	23278000	221	15.34	15.81	16.67	16.16	14.91	15.50	16.16	14.53
1.070	0.72	1.65	P49312	Heterogeneous nuclear ribonucleoprotein A1;Heterogeneous nuclear rib	Hnrnpa1	1160	23	23	23	79.7	79.7	79.7	34.196	0.0000	283.15	65335000	458	17.53	17.23	18.71	17.88	16.81	17.44	16.99	17.23
0.918	0.72	1.65	Q9CRA8	Exosome complex component RRP46	Exosc5	4686	8	8	8	39.6	39.6	39.6	25.194	0.0000	147.69	8244600	97	14.94	14.89	15.22	14.95	14.72	14.98	14.23	13.21
1.100	0.72	1.64	Q9CQG2	Methyltransferase-like protein 16	Mettl16	4598	6	6	6	10.3	10.3	10.3	62.34	0.0004	4.8804	2422800	22	13.79	13.48	14.99	13.92	13.49	13.36	13.39	13.08
1.194	0.72	1.64	P47934	Carnitine O-acetyltransferase	Crat	1131	21	21	20	37.5	37.5	35.8	70.839	0.0000	108.8	35158000	306	15.93	15.69	16.78	16.36	15.71	14.93	15.85	15.41
0.871	0.72	1.64	Q9D8S4	Oligoribonuclease, mitochondrial	Rexo2	5070	9	9	9	34.2	34.2	34.2	26.738	0.0000	35.032	14372000	83	16.46	16.58	16.20	16.89	16.46	14.76	15.70	16.35
1.351	0.72	1.64	Q8C172	Ceramide synthase 6	Cers6	3357	6	6	5	14.6	14.6	11.7	44.812	0.0000	46.264	19390000	149	16.12	15.75	17.04	16.42	15.70	15.42	15.58	15.76
1.954	0.72	1.64	Q9JKF7	39S ribosomal protein L39, mitochondrial	Mrpl39	5451	13	13	13	43.2	43.2	43.2	38.549	0.0000	90.861	17385000	210	15.67	15.23	15.83	16.05	14.92	15.03	15.21	14.75
1.252	0.72	1.64	Q99LC3	NADH dehydrogenase [ubiquinone] 1 alpha subcomplex subunit 10, mit	Ndufa10	4416	17	17	17	61.1	61.1	61.1	40.603	0.0000	133.08	44003000	388	16.29	15.97	17.14	16.35	15.90	15.24	15.73	16.02
1.016	0.71	1.64	Q9EQG9	Collagen type IV alpha-3-binding protein	Col4a3bp	5276	20	20	20	40.7	40.7	40.7	71.11	0.0000	121.86	25003000	287	16.02	15.32	16.92	16.13	15.19	15.38	15.14	15.82
1.012	0.71	1.64	Q8VI75	Importin-4	Ipo4	4030	16	16	15	19.3	19.3	19.3	119.27	0.0000	125.99	25319000	232	16.74	15.99	17.54	17.13	16.11	15.85	16.55	16.03
1.787	0.71	1.64	P68254	14-3-3 protein theta	Ywhaq	1515	31	24	24	85.3	78.8	78.8	27.778	0.0000	323.31	234060000	783	19.36	19.25	20.16	19.70	18.72	19.04	18.98	18.88
1.020	0.71	1.64	Q61249	Immunoglobulin-binding protein 1	Igbp1	2286	18	18	18	66.2	66.2	66.2	38.97	0.0000	279.97	21083000	164	16.36	15.85	17.22	16.21	15.44	16.23	15.84	15.27
0.698	0.71	1.64	Q9EPB4	Apoptosis-associated speck-like protein containing a CARD	Pycard	5250	7	7	7	48.7	48.7	48.7	21.458	0.0000	205.52	18467000	173	16.04	16.37	17.49	16.12	15.64	14.98	15.80	16.74
0.526	0.71	1.64	Q91WL8	WW domain-containing oxidoreductase	Wwox	4119	3	3	3	9.7	9.7	9.7	46.512	0.0000	7.5278	2812300	34	13.92	12.77	15.48	14.01	13.30	12.57	13.56	13.90
1.532	0.71	1.64	P70255	Nuclear factor 1 C-type	Nfic	1540	3	3	3	14.1	14.1	14.1	48.768	0.0002	6.1073	1774500	11	13.81	13.61	14.62	13.91	13.05	13.10	13.53	13.42
3.004	0.71	1.64	Q8R0X7	Sphingosine-1-phosphate lyase 1	Sgpl1	3768	48	48	48	62.1	62.1	62.1	63.676	0.0000	323.31	954320000	1981	20.33	19.95	20.21	20.23	19.55	19.32	19.68	19.33
1.521	0.71	1.64	Q9WV54	Acid ceramidase;Acid ceramidase subunit alpha;Acid ceramidase subur	Asah1	5731	29	29	29	54.8	54.8	54.8	44.669	0.0000	200.45	265670000	803	18.59	18.30	19.19	18.85	18.34	17.67	18.27	17.82
0.529	0.71	1.64	Q9R117	Non-receptor tyrosine-protein kinase TYK2	Tyk2	5646	9	8	8	10.5	9.5	9.5	133.31	0.0000	11.785	4801600	41	15.02	13.62	16.49	14.36	14.12	14.00	14.03	14.51
1.843	0.71	1.63	O88587	UDP-N-acetylglucosamine--peptide N-acetylglucosaminyltransferase 11	Ogt	3514	36	36	36	42.9	42.9	42.9	116.95	0.0000	249.94	50863000	569	16.10	15.89	16.77	16.42	15.63	15.57	15.77	15.38
0.974	0.71	1.63	Q64520	Guanylate kinase	Guk1	2452	8	8	7	48.5	48.5	40.4	21.918	0.0000	42.335	6052000	61	14.60	14.29	15.55	14.81	14.87	13.96	13.89	13.70
0.592	0.71	1.63	Q5SWU9	Acetyl-CoA carboxylase 1;Biotin carboxylase	Acaca	2157	48	48	41	28.9	28.9	25	265.25	0.0000	192.56	42133000	350	15.68	15.40	17.62	16.68	15.47	15.14	16.32	15.62
2.192	0.71	1.63	Q9DCH4	Eukaryotic translation initiation factor 3 subunit F	Eif3f	5214	15	15	15	48.2	48.2	48.2	37.984	0.0000	322.7	87371000	445	17.58	17.93	18.21	17.69	17.24	17.23	17.26	16.84
0.630	0.71	1.63	P39688	Tyrosine-protein kinase Fyn	Fyn	1054	7	6	3	15.3	15.3	8.4	60.674	0.0000	35.352	18855000	96	17.48	16.68	19.02	17.56	16.49	17.49	17.17	16.76
1.410	0.71	1.63	P53612	Geranylgeranyl transferase type-2 subunit beta	Rabggtb	1234	3	3	3	10.6	10.6	10.6	37.803	0.0000	8.1985	6979300	35	16.28	15.26	15.97	16.35	14.99	15.23	15.47	15.32
1.209	0.71	1.63	Q6P9J9	Anoctamin-6	Ano6	2586	17	17	17	21.3	21.3	21.3	106.25	0.0000	172.2	47598000	356	16.36	16.03	17.40	16.23	15.67	15.84	15.83	15.86
0.179	0.71	1.63	P40201	Chromodomain-helicase-DNA-binding protein 1	Chd1	1058	7	7	6	5.8	5.8	5.3	196.38	0.0000	8.4545	7186400	33	16.01	16.34	17.13	12.65	14.20	16.52	16.83	11.74
0.429	0.71	1.63	Q8QZS3	Folliculin	Flcn	3734	7	7	7	17.4	17.4	17.4	64.327	0.0000	19.394	9943500	36	16.42	16.09	16.95	14.98	15.76	13.75	16.64	15.46
0.941	0.71	1.63	Q920I9	WD repeat-containing protein 7	Wdr7	4211	15	15	15	16.7	16.7	16.7	163.45	0.0000	139.44	13246000	182	15.22	14.98	16.33	15.34	14.93	14.76	15.25	14.10
1.515	0.71	1.63	P35991	Tyrosine-protein kinase BTK	Btk	1039	27	27	26	46.7	46.7	45.5	76.437	0.0000	323.31	81213000	546	17.35	17.10	18.19	17.68	16.93	16.71	17.08	16.77
0.713	0.71	1.63	P43883	Perilipin-2	Plin2	1093	22	22	22	69.2	69.2	69.2	46.646	0.0000	283.67	20081000	224	15.50	14.99	17.03	15.84	15.42	15.19	15.42	14.51
1.429	0.70	1.63	P17710	Hexokinase-1	Hk1	828	57	57	50	53.8	53.8	48.4	108.3	0.0000	323.31	271750000	1314	17.90	17.76	18.78	18.04	17.55	17.05	17.67	17.40
1.676	0.70	1.63	Q9DBH5	Vesicular integral-membrane protein VIP36	Lman2	5157	13	13	13	48.6	48.6	48.6	40.429	0.0000	114.74	21002000	180	16.44	16.70	15.89	16.16	15.74	15.19	15.58	15.85
1.851	0.70	1.63	Q61335	B-cell receptor-associated protein 31	Bcap31	2293	22	22	22	52.7	52.7	52.7	27.956	0.0000	323.31	204690000	791	18.45	18.02	18.99	18.42	17.62	17.87	17.75	17.82
1.500	0.70	1.63	P09581	Macrophage colony-stimulating factor 1 receptor	Csf1r	699	26	26	26	25.1	25.1	25.1	109.18	0.0000	266.35	137830000	532	18.64	18.23	18.85	19.01	18.02	17.71	18.49	17.69
1.790	0.70	1.63	Q8CCJ3	E3 UFM1-protein ligase 1	Ufl1	3449	23	23	23	41.1	41.1	41.1	89.519	0.0000	202.6	32466000	352	16.43	15.97	16.75	16.57	15.84	15.76	15.97	15.35
0.904	0.70	1.63	P58137	Acyl-coenzyme A thioesterase 8	Acot8	1309	10	10	10	42.2	42.2	42.2	35.827	0.0000	84.919	10995000	87	15.07	14.73	14.31	15.73	14.65	14.15	14.65	13.59
0.393	0.70	1.63	Q3UUJ4	STE20-related kinase adapter protein alpha	Strada	2032	3	3	1	7	7	4.6	48.235	0.0000	7.1056	4849900	35	14.85	15.02	15.52	14.98	12.65	14.39	16.39	14.14
2.519	0.70	1.63	Q8R146	Acylamino-acid-releasing enzyme	Apeh	3776	45	45	45	69	69	69	81.58	0.0000	323.31	388940000	1037	17.65	17.65	18.21	17.74	17.04	17.14	17.27	16.99
1.076	0.70	1.63	Q8BK63	Casein kinase I isoform alpha	Csnk1a1	3094	8	8	8	23.1	23.1	23.1	38.914	0.0000	142.85	11750000	137	15.76	15.44	16.89	16.04	15.32	15.20	15.70	15.09
1.009	0.70	1.62	A3KGF7	1-phosphatidylinositol 4,5-bisphosphate phosphodiesterase beta-2	Plcb2	52	8	8	8	10.8	10.8	10.8	134.54	0.0000	57.611	4046400	65	13.86	13.48	14.56	13.69	13.25	12.47	13.78	13.29
0.552	0.70	1.62	Q8R1J9	Torsin-2A	Tor2a	3790	3	3	3	14	14	14	35.897	0.0000	42.155	1562600	12	14.16	13.10	15.50	13.78	12.58	13.96	13.77	13.44
1.849	0.70	1.62	P18242	Cathepsin D	Ctsd	836	27	27	25	56.6	56.6	55.1	44.953	0.0000	323.31	1295100000	1402	21.45	21.83	21.67	21.37	21.00	21.22	20.93	20.39
0.679	0.70	1.62	Q6R5N8	Toll-like receptor 13	Tlr13	2653	19	19	19	21.5	21.5	21.5	114.44	0.0000	160.93	47569000	300	17.57	17.36	18.89	17.75	17.93	16.35	17.63	16.86
0.687	0.70	1.62	Q8R0S2	IQ motif and SEC7 domain-containing protein 1	Iqsec1	3765	14	14	10	17.9	17.9	13.7	108.01	0.0000	29.592	12264000	107	14.94	15.28	16.83	15.55	14.76	14.89	15.70	14.45
2.112	0.70	1.62	Q3TXS7	26S proteasome non-ATPase regulatory subunit 1	Psmd1	1886	36	36	36	46.9	46.9	46.9	105.73	0.0000	323.31	95159000	834	16.95	16.78	17.50	17.20	16.30	16.29	16.64	16.41
1.072	0.70	1.62	Q9DCD2	Pre-mRNA-splicing factor SYF1	Xab2	5208	21	21	21	29.5	29.5	29.5	99.986	0.0000	84.03	19227000	178	15.21	14.77	16.20	15.35	14.49	14.39	15.08	14.77
0.621	0.70	1.62	Q8BKI2	Trinucleotide repeat-containing gene 6B protein	Tnrc6b	3102	6	6	6	5.2	5.2	5.2	191.96	0.0000	45.592	2124400	44	13.29	12.88	15.17	13.33	12.96	13.10	13.27	12.56
0.612	0.70	1.62	Q8K370	Acyl-CoA dehydrogenase family member 10	Acad10	3693	19	19	19	21.6	21.6	21.6	118.98	0.0000	52.965	38262000	143	18.04	17.86	18.72	17.38	18.09	15.99	17.78	17.35
0.674	0.70	1.62	Q8VCW8	Acyl-CoA synthetase family member 2, mitochondrial	Acsf2	3934	13	13	13	43.9	43.9	43.9	67.95	0.0000	139.26	16415000	89	16.29	16.19	16.79	16.08	15.51	16.53	16.16	14.37
1.123	0.70	1.62	Q8BVE3	V-type proton ATPase subunit H	Atp6v1h	3237	32	32	32	64.2	64.2	64.2	55.854	0.0000	323.31	279300000	1052	19.33	18.81	20.23	19.41	18.80	18.45	19.09	18.66
2.304	0.70	1.62	Q9ET01	Glycogen phosphorylase, liver form	Pygl	5353	66	66	54	74.5	74.5	64.4	97.462	0.0000	323.31	240710000	1362	18.07	17.95	18.59	17.95	17.46	17.45	17.55	17.32
0.798	0.69	1.62	Q6ZQH8	Nucleoporin NUP188 homolog	Nup188	2696	12	12	12	9.4	9.4	9.4	196.69	0.0000	25.574	6633300	68	14.88	14.03	15.50	14.89	13.65	14.40	14.88	13.59
1.316	0.69	1.62	Q8BK72	28S ribosomal protein S27, mitochondrial	Mrps27	3097	13	13	13	41.7	41.7	41.7	47.778	0.0000	78.977	16542000	161	15.41	15.28	16.42	15.66	14.67	15.01	15.19	15.13
1.272	0.69	1.62	Q64337	Sequestosome-1	Sqstm1	2432	11	11	11	36.9	36.9	36.9	48.162	0.0000	130.89	36478000	256	17.25	17.24	18.41	17.43	16.95	17.02	16.92	16.66
0.814	0.69	1.62	Q9ER41	Torsin-1B	Tor1b	5294	5	5	5	18.5	18.5	18.5	37.817	0.0000	52.215	3889800	46	14.31	14.68	14.45	13.83	13.76	13.31	14.61	12.81
1.265	0.69	1.62	Q9WUN2	Serine/threonine-protein kinase TBK1	Tbk1	5718	26	26	26	44	44	44	83.424	0.0000	291.95	50592000	365	17.00	16.76	18.05	17.15	16.42	16.48	16.74	16.55
1.568	0.69	1.61	Q9DCZ1	GMP reductase 1	Gmpr	5237	8	6	6	27.8	21.7	21.7	37.482	0.0000	34.573	7635600	48	15.40	15.58	15.51	15.64	14.81	15.49	14.40	14.66
1.288	0.69	1.61	P54823	Probable ATP-dependent RNA helicase DDX6	Ddx6	1261	22	22	22	65	65	65	54.191	0.0000	297	56170000	295	16.99	17.07	18.03	17.29	16.76	16.90	16.76	16.19
1.518	0.69	1.61	O70172	Phosphatidylinositol 5-phosphate 4-kinase type-2 alpha	Pip4k2a	475	23	23	16	52.3	52.3	42.7	46.151	0.0000	146.37	76937000	456	17.56	16.99	18.10	17.76	16.75	16.82	17.02	17.07
0.677	0.69	1.61	Q61136	Serine/threonine-protein kinase PRP4 homolog	Prpf4b	2257	15	15	15	18	18	18	116.97	0.0000	80.534	20018000	218	16.49	15.92	17.53	16.39	16.01	14.87	16.49	16.20
1.010	0.69	1.61	Q9JHS4	ATP-dependent Clp protease ATP-binding subunit clpX-like, mitochondr	Clpx	5372	18	18	18	42.7	42.7	42.7	69.228	0.0000	120.34	16260000	262	15.05	14.43	16.04	14.91	14.25	14.54	14.61	14.27
0.817	0.69	1.61	Q9QYF9	Protein NDRG3	Ndrg3	5572	6	6	5	23.5	23.5	22.4	41.555	0.0000	23.775	2625800	24	13.57	14.11	14.61	14.45	13.92	12.46	13.97	13.62
2.048	0.69	1.61	Q8BTV2	Cleavage and polyadenylation specificity factor subunit 7	Cpsf7	3208	13	13	13	35.7	35.7	35.7	52.01	0.0000	219.22	20126000	238	15.98	15.86	16.16	16.14	15.68	14.94	15.23	15.54
0.757	0.69	1.61	P16546	Spectrin alpha chain, non-erythrocytic 1	Sptan1	811	42	42	42	25.2	25.2	25.2	284.59	0.0000	299.32	31711000	407	15.62	15.00	17.04	15.58	14.95	15.22	14.94	15.40
0.686	0.69	1.61	Q9D0K0	TBC1 domain family member 7	Tbc1d7	4871	3	3	3	16.4	16.4	16.4	33.826	0.0000	10.028	2754300	29	14.17	13.45	15.23	13.42	13.37	13.84	13.57	12.75
0.552	0.69	1.61	Q4FK66	Pre-mRNA-splicing factor 38A	Prpf38a	2057	5	5	5	16	16	16	37.436	0.0000	28.254	7912200	68	15.35	15.00	17.28	16.00	15.12	15.89	14.52	15.36
2.661	0.68	1.61	P31750	RAC-alpha serine/threonine-protein kinase	Akt1	989	19	19	16	47.7	47.7	37.9	55.707	0.0000	151.04	39811000	288	16.64	16.29	16.74	16.62	16.06	15.89	15.96	15.63
1.383	0.68	1.61	Q9CPU0	Lactoylglutathione lyase	Glo1	4535	11	11	11	64.1	64.1	64.1	20.809	0.0000	76.317	15534000	147	16.37	16.59	16.63	16.65	15.54	15.82	15.53	16.62
0.626	0.68	1.61	Q6P3A9	ADP-ribosylation factor-like protein 11	Arl11	2560	2	2	2	17	17	17	19.212	0.0000	25.192	1823200	15	13.72	14.13	15.43	14.26	14.51	12.74	13.67	13.90
1.078	0.68	1.61	Q8C129	Leucyl-cystinyl aminopeptidase	Lnpep	3350	23	23	22	28.2	28.2	25.5	117.3	0.0000	157.96	41905000	326	16.91	16.23	17.56	16.48	15.99	15.77	16.50	16.18
1.117	0.68	1.61	Q61490	CD166 antigen	Alcam	2305	13	13	13	23.8	23.8	23.8	65.091	0.0000	82.758	33139000	197	17.48	17.07	18.27	17.18	16.92	17.24	16.63	16.46
1.429	0.68	1.61	P11499	Heat shock protein HSP 90-beta	Hsp90ab1	745	83	83	60	78.2	78.2	65.2	83.28	0.0000	323.31	1714500000	3337	20.43	20.11	21.28	20.69	19.80	20.01	20.08	19.88
0.699	0.68	1.60	Q9DCD6	Gamma-aminobutyric acid receptor-associated protein	Gabarap	5209	4	4	3	26.5	26.5	12	13.918	0.0000	8.5303	8154200	96	14.74	15.45	16.72	15.97	15.07	14.41	15.46	15.21
0.724	0.68	1.60	Q6DTY7	6-phosphofructo-2-kinase/fructose-2,6-bisphosphatase 4;6-phosphofruc	Pfkfb4	2511	13	13	13	38.4	38.4	38.4	54.066	0.0000	87.984	11045000	115	15.17	14.23	16.34	15.28	14.43	14.25	14.97	14.64
0.811	0.68	1.60	Q8BHB4	WD repeat-containing protein 3	Wdr3	3027	10	10	10	16.6	16.6	16.6	105.77	0.0000	21.597	8994000	79	14.96	14.46	15.74	15.13	14.04	14.12	15.36	14.04
1.009	0.68	1.60	O54692	Centromere/kinetochore protein zw10 homolog	Zw10	417	17	17	16	28.8	28.8	28.6	88.062	0.0000	156.02	14136000	172	15.68	15.25	16.18	15.88	15.05	14.48	15.85	14.88
0.263	0.68	1.60	Q62193	Replication protein A 32 kDa subunit	Rpa2	2368	6	6	6	40.7	40.7	40.7	29.718	0.0000	45.664	7932000	54	16.34	16.21	12.69	14.57	14.45	13.63	15.95	13.05
0.366	0.68	1.60	A3KMP2	Tetratricopeptide repeat protein 38	Ttc38	55	8	8	8	24.7	24.7	24.7	52.223	0.0000	43.197	9941400	78	15.80	15.69	13.92	15.86	15.68	14.02	15.78	13.07
0.587	0.68	1.60	Q8CFQ3	Intron-binding protein aquarius	Aqr	3491	15	15	15	16.1	16.1	16.1	170.29	0.0000	192.83	12187000	147	15.02	14.35	16.60	14.91	14.25	14.80	15.12	13.99
1.155	0.68	1.60	Q921G8	Gamma-tubulin complex component 2	Tubgcp2	4224	13	13	13	20	20	20	103.22	0.0000	36.167	8169400	113	14.10	14.18	15.34	14.42	13.79	13.52	14.10	13.90
1.559	0.68	1.60	P05132	cAMP-dependent protein kinase catalytic subunit alpha	Prkaca	648	25	25	13	54.7	54.7	30.2	40.57	0.0000	191.84	61700000	416	17.68	17.40	18.46	17.74	17.10	17.04	17.34	17.09
1.862	0.68	1.60	Q9DC23	DnaJ homolog subfamily C member 10	Dnajc10	5187	18	18	18	26.7	26.7	26.7	90.582	0.0000	157.13	28081000	301	16.12	16.25	16.74	16.55	15.92	15.35	15.72	15.95
1.334	0.68	1.60	P16125	L-lactate dehydrogenase B chain	Ldhb	806	16	14	14	54.2	46.7	46.7	36.572	0.0000	134.54	34373000	276	16.65	16.44	17.48	16.34	16.13	16.15	16.09	15.80
0.512	0.68	1.60	Q76N33	AMSH-like protease	Stambpl1	2720	8	8	8	22.5	22.5	22.5	49.639	0.0000	17.824	5677100	45	14.49	13.82	16.05	15.33	14.46	14.73	14.63	13.16
1.450	0.68	1.60	Q7TMB8	Cytoplasmic FMR1-interacting protein 1	Cyfip1	2740	63	63	40	52.4	52.4	35.8	145.24	0.0000	323.31	241380000	1232	18.51	18.20	19.30	18.70	17.99	17.85	18.28	17.87
1.326	0.68	1.60	Q8VDQ1	Prostaglandin reductase 2	Ptgr2	3967	22	22	22	68.9	68.9	68.9	38.015	0.0000	323.31	84315000	357	17.51	17.72	16.54	16.98	16.49	16.66	16.39	16.50
1.133	0.68	1.60	Q99K85	Phosphoserine aminotransferase	Psat1	4366	26	26	26	65.7	65.7	65.7	40.472	0.0000	223.76	87357000	612	17.00	16.73	18.12	17.46	16.69	16.45	16.80	16.65
0.591	0.68	1.60	Q99J93	Interferon-induced transmembrane protein 2	Ifitm2	4333	3	2	2	17.4	11.8	11.8	15.743	0.0000	16.2	5092600	70	15.11	14.45	14.16	16.11	14.71	13.67	14.96	13.78
3.426	0.68	1.60	D3Z7P3	Glutaminase kidney isoform, mitochondrial	Gls	215	30	30	30	53.3	53.3	53.3	73.963	0.0000	309.86	78178000	550	17.47	17.34	17.15	17.42	16.78	16.54	16.78	16.58
2.465	0.68	1.60	Q9EPL9	Peroxisomal acyl-coenzyme A oxidase 3	Acox3	5260	31	31	31	54.7	54.7	54.7	78.403	0.0000	224.46	103450000	611	17.60	17.43	17.77	17.48	17.18	16.93	16.87	16.58
1.663	0.68	1.60	P48025	Tyrosine-protein kinase SYK	Syk	1139	46	46	45	73.6	73.6	73.6	71.376	0.0000	323.31	353590000	1200	18.99	18.62	19.59	18.93	18.54	18.45	18.25	18.19
1.227	0.68	1.60	Q9CQH3	NADH dehydrogenase [ubiquinone] 1 beta subcomplex subunit 5, mitoc	Ndufb5	4599	6	6	6	30.7	30.7	30.7	21.71	0.0000	39.829	14856000	88	16.24	15.75	16.56	15.80	15.34	14.84	15.87	15.59
1.004	0.68	1.60	Q9D8V0	Minor histocompatibility antigen H13	Hm13	5077	4	4	4	17.2	17.2	17.2	41.747	0.0000	58.924	38310000	176	17.30	17.07	15.98	17.33	15.90	16.46	16.48	16.15

1.087	0.67	1.60	Q8CJ53	Cdc42-interacting protein 4	Trip10	3567	15	15	15	30.2	30.2	30.2	68.489	0.0000	148.24	11641000	122	15.41	15.36	16.24	15.69	14.74	14.79	14.72	15.75
0.708	0.67	1.59	F7BJB9		Morc3	258	10	10	10	18.2	18.2	18.2	106.52	0.0000	62.062	6017600	77	15.03	14.46	16.33	15.45	14.22	14.38	15.32	14.67
0.646	0.67	1.59	Q811U4	Mitofusin-1	Mfn1	2940	5	5	5	9.6	9.6	9.6	83.725	0.0000	29.84	2208900	19	14.12	13.39	14.97	14.21	13.23	14.20	14.02	12.56
0.535	0.67	1.59	Q9JL15	Galectin-8	Lgals8	5466	7	7	7	38	38	38	36.161	0.0000	70.616	4914400	44	13.71	14.41	15.78	14.64	13.71	12.97	14.55	14.62
1.223	0.67	1.59	P09671	Superoxide dismutase [Mn], mitochondrial	Sod2	701	12	12	12	46.4	46.4	46.4	24.603	0.0000	141.07	84057000	238	18.42	18.64	17.80	18.75	18.03	17.94	17.79	17.16
0.632	0.67	1.59	Q9DBF1	Alpha-aminoadipic semialdehyde dehydrogenase	Aldh7a1	5148	12	12	12	39.9	39.9	39.9	58.861	0.0000	50.117	10305000	108	15.19	14.88	15.29	15.24	14.66	15.01	15.23	13.02
0.654	0.67	1.59	P11531	Dystrophin	Dmd	746	6	6	6	2.5	2.5	2.5	425.83	0.0000	9.7677	7703900	31	15.44	14.85	15.32	16.04	15.03	13.95	15.82	14.18
1.619	0.67	1.59	P35831	Tyrosine-protein phosphatase non-receptor type 12	Ptpn12	1034	20	20	20	29.7	29.7	29.7	86.525	0.0000	90.103	29391000	259	15.92	15.45	16.50	16.19	15.31	15.35	15.42	15.30
1.608	0.67	1.59	O35864	COP9 signalosome complex subunit 5	Cops5	408	15	15	15	49.4	49.4	49.4	37.548	0.0000	155.69	18361000	243	15.89	15.80	15.26	15.82	15.28	14.69	15.35	14.77
1.474	0.67	1.59	P63330	Serine/threonine-protein phosphatase 2A catalytic subunit alpha isoform	Ppp2ca	1507	28	28	7	85.4	85.4	9.4	35.608	0.0000	323.31	113990000	636	17.95	17.78	18.20	17.84	17.71	16.66	17.48	17.24
0.885	0.67	1.59	P97872	Dimethylaniline monooxygenase [N-oxide-forming] 5	Fmo5	1683	4	4	4	11.3	11.3	11.3	60	0.0004	5.191	7241600	40	15.85	14.86	16.21	15.88	14.48	14.97	14.99	15.69
1.161	0.67	1.59	Q9DBR3	Armadillo repeat-containing protein 8	Armc8	5171	16	16	16	30	30	30	75.363	0.0000	36.697	9309700	114	15.26	14.87	16.19	15.14	14.48	14.75	14.93	14.61
1.021	0.67	1.59	O89023	Tripeptidyl-peptidase 1	Tpp1	596	8	8	8	23.1	23.1	23.1	61.341	0.0000	94.805	73061000	235	18.73	19.27	19.90	18.78	18.65	17.92	18.57	18.84
0.604	0.67	1.59	Q99LG0	Ubiquitin carboxyl-terminal hydrolase 16	Usp16	4426	5	5	5	8.6	8.6	8.6	93.433	0.0000	9.0463	3548200	21	14.15	13.81	15.56	13.78	14.46	12.97	13.75	13.45
1.212	0.67	1.59	E9Q912		Rap1gds1	251	23	23	22	47.3	47.3	46.3	66.076	0.0000	226.14	58364000	370	17.25	16.93	18.05	17.49	16.86	16.29	17.11	16.79
0.726	0.67	1.59	Q6PD19	UPF0668 protein C10orf76 homolog		2608	4	4	4	9.9	9.9	9.9	78.631	0.0002	6.0571	3676800	20	14.55	14.52	13.75	14.77	14.32	13.33	14.45	12.84
1.479	0.67	1.59	O08756	3-hydroxyacyl-CoA dehydrogenase type-2	Hsd17b10	295	13	13	13	64.4	64.4	64.4	27.418	0.0000	323.31	124960000	574	18.43	18.21	19.09	18.56	18.01	17.69	18.29	17.63
0.656	0.67	1.59	Q9JHP7	KDEL motif-containing protein 1	Kdelc1	5368	5	5	5	13.7	13.7	13.7	57.985	0.0000	52.766	3424500	54	14.53	14.73	15.35	13.69	14.04	14.60	14.06	12.95
0.778	0.66	1.59	P70261	Paladin	Pald1	1541	10	10	10	16.8	16.8	16.8	96.739	0.0000	48.271	9570400	122	15.03	14.82	15.44	15.34	13.31	15.00	14.75	14.91
1.768	0.66	1.59	Q9Z2G6	Protein sel-1 homolog 1	Sel1l	5851	13	13	13	23.2	23.2	23.2	88.339	0.0000	127.89	30930000	219	17.18	16.90	16.44	17.01	16.33	16.37	16.33	15.83
1.461	0.66	1.58	P42227	Signal transducer and activator of transcription 3	Stat3	1076	33	33	33	56	56	56	88.053	0.0000	323.31	86484000	657	16.79	16.37	17.29	16.94	16.17	15.88	16.60	16.09
0.743	0.66	1.58	Q3UFY7	7-methylguanosine phosphate-specific 5-nucleotidase	Nt5c3b	1958	6	6	6	30.7	30.7	30.7	34.425	0.0000	105.94	7266300	131	14.54	14.48	16.08	14.29	14.21	13.86	14.52	14.15
0.788	0.66	1.58	Q9D1M4	Eukaryotic translation elongation factor 1 epsilon-1	Eef1e1	4926	8	8	8	54.6	54.6	54.6	19.859	0.0000	125.17	23533000	152	16.08	16.46	17.65	16.61	15.50	16.41	15.76	16.50
0.657	0.66	1.58	Q80Y17	Lethal(2) giant larvae protein homolog 1	Llgl1	2894	7	7	7	12	12	12	112.62	0.0000	14.495	3015800	30	14.17	13.34	13.54	13.94	13.10	11.83	13.86	13.55
2.042	0.66	1.58	Q07797	Galectin-3-binding protein	Lgals3bp	1757	13	13	13	27.6	27.6	27.6	64.49	0.0000	323.31	78736000	453	17.22	17.28	17.39	17.67	16.84	16.54	17.08	16.46
2.189	0.66	1.58	P10852	4F2 cell-surface antigen heavy chain	Slc3a2	731	31	31	30	58.7	58.7	54.4	58.336	0.0000	323.31	171900000	695	18.66	18.69	19.16	18.57	18.07	18.37	17.94	18.05
1.205	0.66	1.58	Q9Z1T1	AP-3 complex subunit beta-1	Ap3b1	5822	51	51	51	46.3	46.3	46.3	122.74	0.0000	323.31	175240000	918	18.06	17.89	19.09	18.28	17.84	17.56	17.88	17.40
0.526	0.66	1.58	Q9EP72	ER membrane protein complex subunit 7	Emc7	5245	7	7	7	42.7	42.7	42.7	26.31	0.0000	100.73	17624000	132	15.68	16.14	16.80	16.09	15.92	13.98	16.40	15.77
1.381	0.66	1.58	Q8VHE0	Translocation protein SEC63 homolog	Sec63	4018	11	11	11	15.4	15.4	15.4	87.869	0.0000	82.615	31661000	252	15.78	15.22	16.42	15.94	15.02	15.23	15.31	15.17
1.776	0.66	1.58	P97310	DNA replication licensing factor MCM2	Mcm2	1620	36	36	36	47.1	47.1	47.1	102.08	0.0000	240	49955000	567	16.51	16.05	16.64	16.25	15.52	15.40	15.85	16.06
1.517	0.66	1.58	Q61207	Prosaposin	Psap	2277	9	9	9	19.9	19.9	19.9	61.422	0.0000	38.247	51786000	205	18.16	18.50	18.92	18.09	17.72	18.01	17.40	17.91
0.201	0.66	1.58	Q4VAA2	Protein CDV3	Cdv3	2067	11	11	10	48.4	48.4	46.3	29.729	0.0000	263.32	7775500	59	14.41	15.95	12.68	14.52	15.60	11.73	15.70	11.90
1.385	0.66	1.58	P51807	Dynein light chain Tctex-type 1	Dynlt1	1202	4	4	4	49.6	49.6	49.6	12.483	0.0000	281.93	20244000	150	16.79	16.76	17.61	16.82	16.22	16.19	16.78	16.14
1.093	0.66	1.58	Q9D0R2	Threonine--tRNA ligase, cytoplasmic	Tars	4883	45	45	45	56.4	56.4	56.4	83.355	0.0000	257.64	130280000	862	17.32	16.79	18.17	17.40	16.74	16.59	17.16	16.57
1.545	0.66	1.58	Q9JIF0	Protein arginine N-methyltransferase 1	Prmt1	5395	12	12	12	38.8	38.8	38.8	42.435	0.0000	132.69	52902000	319	17.37	17.39	17.49	17.74	16.93	16.24	17.25	16.94
0.490	0.66	1.58	Q9CY66	H/ACA ribonucleoprotein complex subunit 1	Gar1	4789	2	2	2	9.5	9.5	9.5	23.474	0.0004	4.9343	5165100	45	14.87	15.08	15.46	14.92	15.34	13.25	15.57	13.56
1.136	0.66	1.58	Q9CZP5	Mitochondrial chaperone BCS1	Bcs1l	4836	6	6	6	23.7	23.7	23.7	47.406	0.0000	25.817	3184900	24	14.71	13.86	14.19	14.84	14.30	13.76	13.40	13.52
1.364	0.66	1.58	O08688	Calpain-5	Capn5	285	14	14	14	25.6	25.6	25.6	72.954	0.0000	32.828	12926000	150	15.03	14.38	15.30	14.50	14.39	13.76	14.24	14.20
1.354	0.66	1.58	Q9CQF3	Cleavage and polyadenylation specificity factor subunit 5	Nudt21	4593	16	16	16	62.1	62.1	62.1	26.24	0.0000	173.97	49202000	294	17.20	17.08	17.97	17.22	16.67	17.17	16.43	16.58
0.821	0.65	1.57	O35134	DNA-directed RNA polymerase I subunit RPA1	Polr1a	341	8	8	8	7.3	7.3	7.3	194.11	0.0000	26.563	5280800	59	15.06	14.50	16.27	15.34	14.32	14.96	14.79	14.48
1.317	0.65	1.57	Q91X78	Erlin-1	Erlin1	4137	19	19	13	43.7	43.7	31.3	39.182	0.0000	85.506	105220000	340	19.84	19.25	20.44	19.60	19.15	18.89	19.22	19.27
1.221	0.65	1.57	Q8VDP3	Protein-methionine sulfoxide oxidase MICAL1	Mical1	3964	30	30	29	36.3	36.3	33	116.78	0.0000	145.02	47583000	489	16.35	16.02	17.27	16.35	15.82	15.63	16.06	15.86
1.594	0.65	1.57	P97372	Proteasome activator complex subunit 2	Psme2	1637	25	25	24	82.8	82.8	78.2	27.057	0.0000	323.31	243210000	663	18.66	18.26	19.14	18.91	18.20	17.74	18.26	18.15
1.636	0.65	1.57	P49717	DNA replication licensing factor MCM4	Mcm4	1168	30	30	30	36.2	36.2	36.2	96.735	0.0000	313.38	70365000	566	17.49	17.55	18.06	17.72	17.11	16.55	17.26	17.29
1.653	0.65	1.57	P06745	Glucose-6-phosphate isomerase	Gpi	661	47	47	37	71.1	71.1	58.8	62.766	0.0000	323.31	1188200000	2285	20.24	20.00	20.40	20.33	19.82	19.03	19.87	19.65
0.601	0.65	1.57	Q60949	TBC1 domain family member 1	Tbc1d1	2226	8	8	8	6.5	6.5	6.5	142.02	0.0000	14.327	4390700	33	13.95	13.67	15.18	15.47	13.63	13.36	14.17	14.49
2.354	0.65	1.57	Q9D2N9	Vacuolar protein sorting-associated protein 33A	Vps33a	4946	26	26	26	52	52	52	67.554	0.0000	232.63	45380000	397	16.99	16.79	16.98	16.89	16.37	15.88	16.26	16.53
0.658	0.65	1.57	O88876	Short-chain dehydrogenase/reductase 3	Dhrs3	580	6	6	6	21.5	21.5	21.5	33.652	0.0000	23.558	3265300	30	13.69	13.56	14.15	14.61	14.20	13.71	13.23	12.27
1.081	0.65	1.57	Q69ZK0	Phosphatidylinositol 3,4,5-trisphosphate-dependent Rac exchanger 1 pr	Prex1	2485	49	49	48	36	36	35.5	184.93	0.0000	323.31	66630000	745	15.64	15.38	16.69	16.17	15.10	15.38	15.63	15.16
2.008	0.65	1.57	Q3UPL0	Protein transport protein Sec31A	Sec31a	2015	49	49	49	39.2	39.2	39.2	133.57	0.0000	323.31	143540000	1058	17.58	17.41	18.20	17.83	17.06	17.20	17.06	17.11
2.402	0.65	1.57	Q9CQN1	Heat shock protein 75 kDa, mitochondrial	Trap1	4614	34	34	34	56.7	56.7	56.7	80.208	0.0000	323.31	142070000	930	18.33	18.07	18.39	18.56	17.69	17.68	17.94	17.45
1.274	0.65	1.57	O35954	Membrane-associated phosphatidylinositol transfer protein 1	Pitpnm1	413	8	8	8	10.5	10.5	10.5	134.94	0.0000	60.242	5312200	77	14.29	13.85	14.17	14.60	13.69	13.47	14.10	13.04
0.369	0.65	1.57	Q8CHQ0	F-box only protein 4	Fbxo4	3529	4	4	4	11.7	11.7	11.7	43.776	0.0000	9.4259	5760900	36	15.02	14.29	13.97	14.91	14.94	12.36	13.00	15.30
1.523	0.65	1.57	Q9Z210	Peroxisomal membrane protein 11B	Pex11b	5829	5	5	5	23.2	23.2	23.2	28.709	0.0000	23.684	9128800	108	14.93	14.64	14.74	15.29	14.44	14.29	14.54	13.73
0.631	0.65	1.57	Q8BGR2	Volume-regulated anion channel subunit LRRC8D	Lrrc8d	2989	9	9	9	17.7	17.7	17.7	98.112	0.0000	113.19	5464500	89	14.64	13.78	15.84	14.36	14.00	13.37	14.25	14.41
1.564	0.65	1.57	Q9CR11	YEATS domain-containing protein 4	Yeats4	4657	5	5	4	34.4	34.4	28.2	26.529	0.0000	27.281	2694800	33	14.42	14.32	13.82	14.45	13.60	13.20	14.03	13.60
0.918	0.65	1.57	Q9EP82	tRNA (guanine-N(7)-)-methyltransferase non-catalytic subunit WDR4	Wdr4	5246	4	4	4	12.1	12.1	12.1	45.755	0.0002	5.5103	5416100	48	14.24	14.34	15.20	15.12	14.28	13.60	14.70	13.71
2.423	0.65	1.57	Q99JI4	26S proteasome non-ATPase regulatory subunit 6	Psmd6	4340	22	22	22	57.1	57.1	57.1	45.536	0.0000	137.64	281210000	592	20.56	20.44	20.77	20.68	20.06	19.62	20.19	19.98
1.028	0.65	1.57	Q9QUJ7	Long-chain-fatty-acid--CoA ligase 4	Acsl4	5515	35	35	33	59.2	59.2	56.8	79.076	0.0000	253.05	95855000	645	18.03	17.85	18.85	18.64	17.34	17.32	18.22	17.89
0.495	0.65	1.57	Q8BHE8	Uncharacterized protein C2orf47 homolog, mitochondrial		3034	4	4	4	16.5	16.5	16.5	32.985	0.0000	10.749	5493600	28	14.92	15.73	14.97	13.68	13.54	13.88	15.41	13.87
0.791	0.65	1.57	P30115	Glutathione S-transferase A3	Gsta3	973	15	15	15	60.6	60.6	60.6	25.36	0.0000	98.472	40981000	206	17.36	16.78	16.76	16.07	16.25	16.83	15.95	15.35
1.115	0.65	1.56	Q8K1R7	Serine/threonine-protein kinase Nek9	Nek9	3633	19	19	19	22.3	22.3	22.3	107.14	0.0000	137.17	33430000	287	16.38	15.96	17.04	16.53	15.74	15.49	16.43	15.67
0.319	0.65	1.56	Q8K157	Aldose 1-epimerase	Galm	3616	9	9	9	36.3	36.3	36.3	37.798	0.0000	62.501	14780000	116	16.23	16.91	15.19	16.29	16.43	13.19	16.42	16.01
1.429	0.65	1.56	P63328	Serine/threonine-protein phosphatase 2B catalytic subunit alpha isoform	Ppp3ca	1506	25	25	16	48.9	48.9	29.9	58.643	0.0000	184.84	62502000	478	16.87	16.47	17.50	16.93	16.34	16.39	16.50	15.96
1.321	0.65	1.56	Q99L43	Phosphatidate cytidylyltransferase 2	Cds2	4407	3	3	3	11.7	11.7	11.7	51.313	0.0000	30.457	9911400	91	15.59	16.00	15.47	15.29	14.35	15.27	15.23	14.91
1.623	0.64	1.56	O88545	COP9 signalosome complex subunit 6	Cops6	545	12	12	12	49.4	49.4	49.4	35.88	0.0000	193.01	33157000	271	16.59	16.60	16.02	16.74	16.20	15.89	15.77	15.51
0.941	0.64	1.56	Q9DCT5	Stromal cell-derived factor 2	Sdf2	5231	2	2	2	18	18	18	23.159	0.0000	7.4174	1934500	41	13.99	14.17	14.37	14.12	12.64	13.32	14.12	13.99
0.741	0.64	1.56	Q9D6Y9	1,4-alpha-glucan-branching enzyme	Gbe1	5013	21	21	21	35.8	35.8	35.8	80.363	0.0000	250.17	49354000	346	16.64	16.36	18.15	16.60	16.11	16.64	16.23	16.21
0.613	0.64	1.56	Q9WU56	tRNA pseudouridine synthase A, mitochondrial	Pus1	5697	6	6	6	24.1	24.1	24.1	47.502	0.0000	9.2007	1665900	20	13.79	15.11	13.46	14.01	13.80	14.22	12.67	13.11
0.944	0.64	1.56	Q18PI6	SLAM family member 5	Cd84	1793	16	16	16	36.2	36.2	36.2	37.378	0.0000	159.71	31674000	236	17.44	16.86	18.35	17.05	16.67	16.60	17.08	16.77
0.592	0.64	1.56	Q8BYU6	Torsin-1A-interacting protein 2	Tor1aip2	3306	7	7	7	21.5	21.5	21.5	54.495	0.0000	50.776	5946800	56	14.15	14.14	12.56	14.14	12.60	14.05	12.80	12.96
0.961	0.64	1.56	Q9CQE1	Protein NipSnap homolog 3B	Nipsnap3b	4587	13	13	13	55.9	55.9	55.9	28.308	0.0000	60.537	51558000	186	17.62	17.76	18.01	17.14	17.39	16.61	16.38	17.57
0.902	0.64	1.56	Q8BG67	Protein EFR3 homolog A	Efr3a	2965	11	11	11	19.5	19.5	19.5	92.612	0.0000	80.265	11222000	154	15.15	14.33	15.91	15.12	14.07	14.85	14.57	14.45
3.846	0.64	1.56	Q91YR9	Prostaglandin reductase 1	Ptgr1	4176	22	22	22	72.3	72.3	72.3	35.56	0.0000	291.04	301900000	984	19.08	18.99	19.07	18.96	18.57	18.32	18.40	18.25
0.770	0.64	1.56	Q9JI10	Serine/threonine-protein kinase 3;Serine/threonine-protein kinase 3 36k	Stk3	5381	18	13	13	37.6	31.4	31.4	56.855	0.0000	55.507	11051000	138	14.92	14.43	15.62	15.04	14.09	15.17	14.55	13.62
0.572	0.64	1.56	Q9QXD8	LIM domain-containing protein 1	Limd1	5537	8	8	8	25.4	25.4	25.4	71.421	0.0000	119.22	9952900	104	14.96	14.72	15.45	15.14	14.88	14.34	15.42	13.07
0.841	0.64	1.56	Q00558	Factor VIII intron 22 protein	F8a1	1698	5	5	5	21.1	21.1	21.1	40.489	0.0000	44.363	16347000	112	16.21	15.99	17.12	16.37	15.90	16.33	15.95	14.95
1.188	0.64	1.56	Q8BU30	Isoleucine--tRNA ligase, cytoplasmic	Iars	3218	48	48	48	42.9	42.9	42.9	144.27	0.0000	323.31	106720000	752	17.25	16.81	18.06	17.67	16.66	16.66	17.01	16.89
1.288	0.64	1.56	Q8BK67	Protein RCC2	Rcc2	3096	29	29	29	62.7	62.7	62.7	55.983	0.0000	178.71	87742000	617	17.42	17.01	18.08	17.81	16.83	16.77	17.31	16.86
0.948	0.64	1.56	Q9Z2X2	26S proteasome non-ATPase regulatory subunit 10	Psmd10	5871	3	3	3	16.9	16.9	16.9	25.083	0.0000	7.7056	5332900	60	15.69	14.80	15.44	16.23	14.81	14.44	15.14	15.21
0.761	0.64	1.56	Q3URD3	Sarcolemmal membrane-associated protein	Slmap	2021	12	12	12	16.9	16.9	16.9	96.932	0.0000	39.099	3897000	72	14.19	13.39	14.77	14.44	12.74	13.65	14.12	13.72
0.701	0.64	1.56	Q8BYH7	TBC1 domain family member 17	Tbc1d17	3298	9	9	9	21.9	21.9	21.9	72.859	0.0000	19.553	9428900	81	14.87	14.84	15.87	14.90	14.89	13.40	14.98	14.66
0.641	0.64	1.56	Q8CIM8	Integrator complex subunit 4	Ints4	3560	11	11	11	18.2	18.2	18.2	108.19	0.0000	46.493	4701400	87	13.82	13.99	14.66	13.54	12.77	13.04	14.58	13.04
1.084	0.64	1.56	O35638	Cohesin subunit SA-2	Stag2	389	20	20	17	22.2	22.2	19.5	141.28	0.0000	90.742	16069000	174	15.47	15.28	16.21	15.77	14.79	14.52	15.42	15.44
0.518	0.64	1.56	Q99PP7	E3 ubiquitin-protein ligase TRIM33	Trim33	4512	3	3	3	4	4	4	123.84	0.0000	9.0553	1855900	29	14.36	12.89	15.47	14.31	13.51	13.15	13.68	14.14
0.781	0.64	1.56	Q9WUE3	Cytochrome b561 domain-containing protein 2	Cyb561d2	5710	2	2	2	9.5	9.5	9.5	24.187	0.0018	3.1786	1995200	19	14.45	13.36	14.26	14.04	13.52	13.56	12.47	14.01
0.418	0.64	1.56	Q14CH7	Alanine--tRNA ligase, mitochondrial	Aars2	1790	11	11	10	20.4	20.4	19.3	106.78	0.0000	36.172	17650000	80	16.95	16.29	16.94	16.64	16.60	16.72	16.87	14.10
1.778	0.64	1.56	Q61102	ATP-binding cassette sub-family B member 7, mitochondrial	Abcb7	2251	31	31	31	46.5	46.5	46.5	82.58	0.0000	249.75	50152000	496	16.01	15.59	16.45	16.09	15.32	15.22	15.53	15.53
1.482	0.64	1.56	Q8BML9		Qars	3144	46	46	45	57.3	57.3	57.2	87.676	0.0000	323.31	188870000	954	18.23	17.77	18.87	18.27	17.66	17.59	17.79	17.55
0.566	0.64	1.56	Q8VHI3	GDP-fucose protein O-fucosyltransferase 2	Pofut2	4020	4	4	4	10.3	10.3	10.3	49.429	0.0000	13.402	2074100	15	13.84	14.03	15.89	14.06	14.45	13.55	13.77	13.50
1.243	0.64	1.56	Q9CXR1	Dehydrogenase/reductase SDR family member 7	Dhrs7	4763	12	12	12	37.9	37.9	37.9	38.167	0.0000	62.047	21702000	160	16.49	15.91	17.17	16.61	15.90	15.73	16.12	15.88
0.806	0.64	1.55	Q921L5	Conserved oligomeric Golgi complex subunit 2	Cog2	4231	19	19	19	37.6	37.6	37.6	82.039	0.0000	171.24	19232000	126	15.63	16.19	16.97	16.15	15.79	15.71	16.10	14.81
1.493	0.64	1.55	Q78IK4	MICOS complex subunit Mic27	Apool	2723	12	12	12	56.2	56.2	56.2	29.26	0.0000	158.48	38082000	310	16.63	16.27	17.28	16.62	16.09	15.84	16.28	16.03
0.281	0.64	1.55	Q9D6N5	Dr1-associated corepressor	Drap1	5005	5	5	5	20	20	20	22.278	0.0000	11.511	3835600	39	14.98	13.73	16.16	15.84	13.01	15.23	13.58	16.35
0.769	0.63	1.55	Q9DC28	Casein kinase I isoform delta	Csnk1d	5188	9	9	5	25.1	25.1	13	47.316	0.0000	18.438	6817400	69	14.40	14.07	15.53	14.75	14.04	14.73	13.46	13.98
1.750	0.63	1.55	Q8BH59	Calcium-binding mitochondrial carrier protein Aralar1	Slc25a12	3015	41	41	35	71.9	71.9	65.6	74.569	0.0000	323.31	232860000	1068	18.56	18.45	19.27	18.71	18.10	18.14	18.30	17.93
0.528	0.63	1.55	Q9CQ54	NADH dehydrogenase [ubiquinone] 1 subunit C2	Ndufc2	4562	9	9	9	60	60	60	14.164	0.0000	36.309	24107000	147	16.77	16.21	16.00	17.29	16.42	14.86	16.98	15.48
2.064	0.63	1.55	Q8K2Q7	BRO1 domain-containing protein BROX	Brox	3668	19	19	19	60.3	60.3	60.3	46.201	0.0000	230.61	69681000	437	17.87	17.49	17.71	18.01	17.44	17.07	17.18	16.85
0.491	0.63	1.55	Q9D0M0	Exosome complex exonuclease RRP42	Exosc7	4877	6	6	6	36.8	36.8	36.8	31.825	0.0000	53.98	5286700	47	14.32	14.27	15.16	14.32	13.81	12.40	14.39	14.94
1.168	0.63	1.55	Q9D4H1	Exocyst complex component 2	Exoc2	4976	20	20	20	25.8	25.8	25.8	103.96	0.0000	123.93	28619000	335	15.77	15.43	16.46	16.01	15.38	15.25	15.70	14.82
2.423	0.63	1.55	Q62348	Translin	Tsn	2380	17	17	17	64.9	64.9	64.9	26.201	0.0000	235.19	66526000	390	17.00	16.86	17.23	17.20	16.20	16.37	16.71	16.48
0.807	0.63	1.55	Q810A3	Tetratricopeptide repeat protein 9C	Ttc9c	2924	4	4	4	22.8	22.8	22.8	19.997	0.0000	25.069	5873600	97	15.22	14.63	16.37	15.25	14.53	14.55	14.74	15.13
0.604	0.63	1.55	Q501J7	Phosphatase and actin regulator 4	Phactr4	2076	3	3	3	5.2	5.2	5.2	76.631	0.0025	2.806	1149300	5	13.79	12.73	13.74	14.58	12.53	13.94	12.67	13.18
0.733	0.63	1.55	P11928	2-5-oligoadenylate synthase 1A	Oas1a	752	21	21	21	56.4	56.4	56.4	42.428	0.0000	236.37	66143000	562	16.67	16.29	17.81	17.44	16.45	15.84	17.00	16.40
2.098	0.63	1.55	Q91YQ5	Dolichyl-diphosphooligosaccharide--protein glycosyltransferase subunit	Rpn1	4172	53	53	53	74.2	74.2	74.2	68.527	0.0000	323.31	580910000	1598	19.72	19.41	20.02	19.82	19.13	19.18	19.30	18.84
1.241	0.63	1.55	Q9CW03	Structural maintenance of chromosomes protein 3	Smc3	4708	69	69	69	57.4	57.4	57.4	141.55	0.0000	323.31	107730000	975	16.88	16.44	17.65	17.07	16.13	16.33	16.52	16.54
0.804	0.63	1.55	P46978	Dolichyl-diphosphooligosaccharide--protein glycosyltransferase subunit	Stt3a	1113	25	25	25	29.8	29.8	29.8	80.597	0.0000	170.61	85737000	381	17.57	17.81	19.28	18.02	17.41	17.52	17.48	17.76
0.781	0.63	1.55	Q8K1A5	Transmembrane protein 41B	Tmem41b	3620	2	2	2	9.6	9.6	9.6	32.429	0.0000	30.706	9615100	59	16.30	15.38	15.29	16.69	15.23	14.87	15.83	15.21

0.699	0.63	1.55	Q6PHQ8	N-alpha-acetyltransferase 35, NatC auxiliary subunit	Naa35	2641	11	11	11	20.8	20.8	20.8	83.305	0.0000	37.759	6055500	76	14.40	13.80	15.54	14.87	13.35	14.44	14.21	14.10
2.981	0.63	1.55	Q6ZQM8	UDP-glucuronosyltransferase 1-7C	Ugt1a7c	2702	26	26	17	56.1	56.1	38.2	59.757	0.0000	323.31	369020000	1036	19.57	19.45	19.84	19.54	19.03	18.86	19.12	18.87
0.394	0.63	1.55	O09126	Semaphorin-4D	Sema4d	325	8	8	8	13	13	13	95.639	0.0000	72.081	3998800	70	13.77	13.24	15.81	13.86	12.72	14.70	13.29	13.46
0.716	0.63	1.55	Q9ER88	28S ribosomal protein S29, mitochondrial	Dap3	5300	11	11	11	33.5	33.5	33.5	44.699	0.0000	80.966	12295000	118	15.55	15.25	17.04	15.70	14.99	15.55	15.51	14.97
0.598	0.63	1.55	Q60634	Flotillin-2	Flot2	2187	21	21	21	52.6	52.6	52.6	47.037	0.0000	132.34	24672000	236	15.65	15.36	17.07	15.93	15.25	15.76	15.97	14.51
0.815	0.63	1.54	Q80XI4	Phosphatidylinositol 5-phosphate 4-kinase type-2 beta	Pip4k2b	2882	20	14	14	49.3	38.2	38.2	47.318	0.0000	99.178	24752000	171	16.42	16.33	17.71	16.73	16.17	16.18	16.69	15.64
0.281	0.63	1.54	Q9D020	Cytosolic 5-nucleotidase 3A	Nt5c3a	4849	9	9	9	45	45	45	37.252	0.0000	110.7	21935000	121	16.75	17.34	16.78	16.60	17.26	13.67	17.76	16.27
2.223	0.63	1.54	P29416	Beta-hexosaminidase subunit alpha	Hexa	969	26	26	26	43.8	43.8	43.8	60.612	0.0000	323.31	173130000	671	19.17	19.07	19.22	19.28	18.58	18.26	18.94	18.46
0.412	0.63	1.54	Q9JHQ5	Leucine zipper transcription factor-like protein 1	Lztfl1	5369	2	2	2	16.7	16.7	16.7	34.773	0.0000	39.453	1085200	15	13.51	12.84	12.82	14.93	12.10	14.05	13.19	12.25
1.435	0.63	1.54	Q9D6Y7	Mitochondrial peptide methionine sulfoxide reductase	Msra	5012	10	10	10	55.4	55.4	55.4	25.988	0.0000	28.872	32122000	177	16.59	16.07	17.09	16.84	15.82	16.13	16.20	15.94
1.491	0.63	1.54	Q9CZ04	COP9 signalosome complex subunit 7a	Cops7a	4812	12	12	12	59.3	59.3	59.3	30.224	0.0000	160.28	29595000	313	16.66	16.48	17.41	16.58	16.16	16.28	15.95	16.24
1.579	0.62	1.54	Q9ERE7	LDLR chaperone MESD	Mesdc2	5306	6	6	6	27.7	27.7	27.7	25.206	0.0000	18.488	9529200	90	15.60	15.33	16.26	15.63	15.26	14.91	15.20	14.95
0.587	0.62	1.54	Q61211	Eukaryotic translation initiation factor 2D	Eif2d	2279	10	10	10	30.7	30.7	30.7	62.829	0.0000	40.428	9717600	90	15.71	15.31	17.51	16.26	15.50	15.72	15.89	15.18
0.545	0.62	1.54	Q9EPK2	Protein XRP2	Rp2	5254	7	7	7	21.9	21.9	21.9	39.376	0.0000	61.944	7658100	71	15.46	15.48	16.38	15.73	15.23	16.06	15.50	13.77
1.251	0.62	1.54	Q3TWW8	Serine/arginine-rich splicing factor 6	Srsf6	1884	14	14	11	37.2	37.2	32.4	39.025	0.0000	147.71	39258000	361	17.09	17.05	17.21	17.58	16.08	16.84	16.39	17.14
0.612	0.62	1.54	Q8BNV1	tRNA (uracil-5-)-methyltransferase homolog A	Trmt2a	3156	14	14	14	34.5	34.5	34.5	63.301	0.0000	57.679	12703000	161	15.35	14.49	16.35	15.34	14.48	15.59	14.75	14.22
1.234	0.62	1.54	Q8CIF6	SID1 transmembrane family member 2	Sidt2	3552	3	3	3	4.1	4.1	4.1	94.5	0.0000	7.7806	1885400	37	13.63	13.38	14.12	14.36	13.54	12.86	13.40	13.21
0.430	0.62	1.54	P19324	Serpin H1	Serpinh1	846	3	3	3	11.8	11.8	11.8	46.533	0.0000	50.303	1190400	35	12.80	13.04	11.34	13.73	11.98	11.18	13.14	12.12
0.412	0.62	1.54	Q3U1V6	Ubiquitin-conjugating enzyme E2 variant 3	Uevld	1905	9	9	9	31.8	31.8	31.8	51.681	0.0000	26.248	4092200	42	14.45	14.21	13.18	14.20	12.01	12.74	14.49	14.31
0.305	0.62	1.54	Q8R4V1	NFAT activation molecule 1	Nfam1	3874	6	6	6	25.4	25.4	25.4	29.987	0.0000	39.166	14343000	106	16.06	15.65	15.91	15.85	16.50	12.74	15.73	16.02
0.768	0.62	1.54	Q91WT8	RNA-binding protein 47	Rbm47	4128	14	14	14	45.4	45.4	45.4	64.061	0.0000	153.25	35592000	264	16.60	16.49	17.99	17.20	16.42	16.58	16.88	15.92
0.814	0.62	1.54	Q9DBT5	AMP deaminase 2	Ampd2	5177	21	21	21	31.5	31.5	31.5	92.023	0.0000	51.9	32608000	300	16.39	15.94	17.60	16.47	15.76	15.92	16.38	15.86
0.883	0.62	1.54	Q99LR1	Monoacylglycerol lipase ABHD12	Abhd12	4444	30	30	30	73.6	73.6	73.6	45.269	0.0000	323.31	168530000	668	18.22	17.66	19.26	18.33	17.72	17.83	18.01	17.43
2.214	0.62	1.54	P25206	DNA replication licensing factor MCM3	Mcm3	905	36	36	34	48.8	48.8	48.8	91.545	0.0000	323.31	55428000	589	16.43	16.00	16.55	16.52	15.54	15.86	15.76	15.86
1.934	0.62	1.53	Q8VDM4	26S proteasome non-ATPase regulatory subunit 2	Psmd2	3960	51	51	51	64.2	64.2	64.2	100.2	0.0000	323.31	193040000	995	18.85	18.80	19.49	18.95	18.41	18.22	18.55	18.44
2.671	0.62	1.53	P12382	ATP-dependent 6-phosphofructokinase, liver type	Pfkl	757	38	35	32	58.8	56.5	54.4	85.359	0.0000	323.31	146090000	887	17.77	17.65	18.13	18.03	17.39	17.16	17.27	17.30
0.603	0.62	1.53	Q8R420	ATP-binding cassette sub-family A member 3	Abca3	3864	13	13	13	10.9	10.9	10.9	191.97	0.0000	33.534	95833000	74	19.57	19.37	20.64	19.90	19.15	18.76	20.41	18.70
1.033	0.62	1.53	Q9ES00	Ubiquitin conjugation factor E4 B	Ube4b	5322	19	19	19	27.2	27.2	27.2	133.32	0.0000	169.64	16384000	178	14.97	14.64	15.84	15.55	14.27	14.79	14.95	14.52
0.478	0.62	1.53	P33609	DNA polymerase alpha catalytic subunit	Pola1	1002	10	10	10	14.2	14.2	14.2	167.34	0.0000	26.25	6739500	51	15.11	14.41	16.34	15.21	14.50	13.83	15.84	14.42
1.299	0.62	1.53	P61620	Protein transport protein Sec61 subunit alpha isoform 1	Sec61a1	1387	11	11	11	29.2	29.2	29.2	52.264	0.0000	128.35	61830000	322	17.59	17.73	18.70	18.09	17.44	17.49	17.26	17.46
0.918	0.61	1.53	Q8R242	Di-N-acetylchitobiase	Ctbs	3805	5	5	5	20.2	20.2	20.2	41.295	0.0000	90.108	7625300	115	14.81	15.42	15.90	15.34	15.25	14.04	14.85	14.88
1.611	0.61	1.53	P50247	Adenosylhomocysteinase	Ahcy	1177	29	29	29	65.3	65.3	65.3	47.688	0.0000	323.31	156610000	832	18.50	18.48	19.31	18.52	18.02	18.14	18.06	18.12
2.801	0.61	1.53	P14211	Calreticulin	Calr	778	31	31	31	59.1	59.1	59.1	47.994	0.0000	323.31	315410000	913	19.27	19.24	19.28	19.32	18.90	18.44	18.51	18.81
2.415	0.61	1.53	P51881	ADP/ATP translocase 2;ADP/ATP translocase 2, N-terminally processe	Slc25a5	1208	42	42	27	74.2	74.2	50.7	32.931	0.0000	323.31	1150500000	1648	21.18	21.04	21.60	21.28	20.81	20.61	20.53	20.70
0.468	0.61	1.53	Q5PRF0	HEAT repeat-containing protein 5A	Heatr5a	2123	16	16	16	12.4	12.4	12.4	219.89	0.0000	182.45	9875800	139	14.78	13.95	16.25	15.20	14.42	13.84	15.42	14.05
0.377	0.61	1.53	P63073	Eukaryotic translation initiation factor 4E	Eif4e	1486	7	7	7	27.6	27.6	27.6	25.053	0.0000	24.312	20154000	117	16.94	16.53	17.58	15.26	16.95	15.68	14.65	16.57
0.930	0.61	1.53	Q8VI63	MOB kinase activator 2	Mob2	4029	5	5	4	20	20	20	26.851	0.0000	9.5597	4890500	48	14.49	14.54	14.93	14.68	13.98	13.19	14.38	14.66
1.554	0.61	1.53	P60670	Nuclear protein localization protein 4 homolog	Nploc4	1357	25	25	25	49.8	49.8	49.8	68.016	0.0000	115.63	58369000	373	17.04	16.68	17.34	16.94	16.59	16.08	16.74	16.15
0.492	0.61	1.53	Q9Z2H5	Band 4.1-like protein 1	Epb41l1	5853	9	9	9	14.6	14.6	14.6	98.314	0.0000	42.498	5428900	82	14.84	14.79	15.35	14.94	14.30	12.88	15.47	14.83
1.450	0.61	1.53	P10605	Cathepsin B;Cathepsin B light chain;Cathepsin B heavy chain	Ctsb	724	23	23	23	62.8	62.8	62.8	37.279	0.0000	323.31	771270000	1535	21.15	21.12	21.50	21.29	21.15	20.23	20.41	20.83
1.331	0.61	1.53	P21981	Protein-glutamine gamma-glutamyltransferase 2	Tgm2	871	37	37	37	64.1	64.1	64.1	77.06	0.0000	323.31	330560000	1090	19.88	19.60	18.87	19.66	18.80	19.10	19.05	18.64
1.653	0.61	1.53	Q91W86	Vacuolar protein sorting-associated protein 11 homolog	Vps11	4096	36	36	36	44	44	44	107.72	0.0000	230.25	73749000	725	16.81	16.42	17.33	16.83	16.30	16.26	16.37	16.03
0.948	0.61	1.53	Q9R0A0	Peroxisomal membrane protein PEX14	Pex14	5619	8	8	7	35.4	35.4	26.6	41.207	0.0000	323.31	15284000	160	15.39	15.01	15.50	14.87	14.70	14.69	15.17	13.77
1.988	0.61	1.53	O35345	Importin subunit alpha-7	Kpna6	360	15	15	11	40.5	40.5	33.2	59.964	0.0000	149.04	29823000	314	16.51	16.69	17.00	16.51	15.98	16.18	16.33	15.78
2.197	0.61	1.52	P13439	Uridine 5-monophosphate synthase;Orotate phosphoribosyltransferase;	Umps	765	26	26	26	62.8	62.8	62.8	52.292	0.0000	250.66	51136000	409	16.87	16.78	17.15	16.94	16.44	16.09	16.63	16.15
0.570	0.61	1.52	Q9JJQ0	GPI mannosyltransferase 3	Pigb	5432	5	5	5	13.8	13.8	13.8	63.119	0.0000	26.339	3786700	25	14.45	14.16	13.28	14.70	13.74	14.56	12.81	13.04
0.665	0.61	1.52	Q9DC50	Peroxisomal carnitine O-octanoyltransferase	Crot	5193	12	12	11	28.1	28.1	24.7	70.264	0.0000	53.199	12076000	111	15.36	15.06	15.18	14.77	14.51	14.72	15.37	13.34
1.791	0.61	1.52	P82343	N-acylglucosamine 2-epimerase	Renbp	1599	20	20	20	38.8	38.8	38.8	49.771	0.0000	323.31	90722000	579	17.61	17.76	18.41	17.76	17.39	17.21	17.25	17.26
2.546	0.61	1.52	P09528	Ferritin heavy chain;Ferritin heavy chain, N-terminally processed	Fth1	698	15	15	15	78	78	78	21.066	0.0000	323.31	228540000	655	18.88	18.78	19.27	19.04	18.46	18.52	18.28	18.26
0.869	0.61	1.52	Q6ZQ08	CCR4-NOT transcription complex subunit 1	Cnot1	2688	31	31	31	17.7	17.7	17.7	266.8	0.0000	139.67	25157000	301	15.69	15.40	16.85	16.03	15.14	15.33	15.84	15.24
0.271	0.61	1.52	Q9CX84	Regulator of G-protein signaling 19	Rgs19	4749	5	5	5	34.7	34.7	34.7	24.677	0.0000	18.349	4195900	16	15.09	12.22	14.99	15.10	13.44	13.99	12.35	15.19
0.923	0.61	1.52	Q8CCM6	Mitochondrial import inner membrane translocase subunit Tim21	Timm21	3450	3	3	3	14.8	14.8	14.8	27.911	0.0000	7.3219	1665300	30	13.94	13.61	14.16	13.56	12.32	13.68	13.41	13.43
2.980	0.61	1.52	P47811	Mitogen-activated protein kinase 14	Mapk14	1126	21	21	21	80.6	80.6	80.6	41.287	0.0000	228.85	101210000	615	17.98	17.82	18.13	18.21	17.54	17.33	17.52	17.34
0.316	0.60	1.52	E9PYH6	Histone-lysine N-methyltransferase	Setd1a	223	8	8	8	7	7	7	186.06	0.0000	13.41	3970900	40	15.33	14.91	15.20	13.84	12.82	13.09	15.25	15.70
1.097	0.60	1.52	Q80WG5	Volume-regulated anion channel subunit LRRC8A	Lrrc8a	2861	7	7	7	11.2	11.2	11.2	94.118	0.0000	19.704	6520300	58	14.99	14.73	15.18	14.88	14.66	13.53	14.59	14.59
0.285	0.60	1.52	Q8BGS0	Protein MAK16 homolog	Mak16	2990	4	4	4	22.6	22.6	22.6	35.185	0.0000	30.384	9331000	107	15.16	14.49	16.74	15.37	15.13	12.74	16.28	15.20
2.308	0.60	1.52	Q8JZQ9	Eukaryotic translation initiation factor 3 subunit B	Eif3b	3579	37	37	37	56.2	56.2	56.2	91.369	0.0000	323.31	147200000	838	17.56	17.38	18.03	17.68	17.11	17.04	17.07	17.02
0.588	0.60	1.52	Q3UA06	Pachytene checkpoint protein 2 homolog	Trip13	1938	4	4	4	18.1	18.1	18.1	48.376	0.0000	17.86	2474200	32	13.39	14.40	13.51	14.52	13.43	13.95	12.25	13.78
0.904	0.60	1.52	Q60787	Lymphocyte cytosolic protein 2	Lcp2	2207	12	12	12	23.5	23.5	23.5	60.238	0.0000	32.264	24418000	189	16.40	16.37	17.67	16.62	15.85	16.01	16.34	16.45
1.962	0.60	1.52	O55234	Proteasome subunit beta type-5	Psmb5	466	12	12	12	47	47	47	28.532	0.0000	187.96	44091000	259	17.49	17.42	17.73	17.17	17.06	16.68	17.06	16.61
1.077	0.60	1.52	Q9WVQ5	Methylthioribulose-1-phosphate dehydratase	Apip	5759	15	15	15	68.5	68.5	68.5	26.949	0.0000	57.305	38741000	327	16.47	16.14	17.28	16.54	16.20	15.83	16.36	15.63
0.299	0.60	1.52	Q3U2C5	E3 ubiquitin-protein ligase RNF149	Rnf149	1910	5	5	5	17.8	17.8	17.8	42.552	0.0000	6.7526	4513200	57	14.06	13.40	15.90	14.42	13.65	15.59	13.72	12.41
0.499	0.60	1.52	Q8BYB9	Protein O-glucosyltransferase 1	Poglut1	3296	7	7	7	19.6	19.6	19.6	46.379	0.0000	14.095	10203000	91	15.48	15.45	15.50	15.91	15.09	13.43	15.80	15.62
0.500	0.60	1.52	A2ADA5	tRNA pseudouridine synthase-like 1	Pusl1	17	4	4	4	14.8	14.8	14.8	31.992	0.0000	32.243	3414600	39	14.14	13.09	15.20	14.28	13.53	14.35	13.71	12.71
1.385	0.60	1.52	Q3TDN2	FAS-associated factor 2	Faf2	1833	17	17	17	43.6	43.6	43.6	52.471	0.0000	290.46	33970000	301	16.30	16.11	16.74	16.25	15.66	15.27	16.14	15.94
0.837	0.60	1.52	E9Q3L2		Pi4ka	233	29	29	29	18.7	18.7	18.7	237.04	0.0000	125.64	27785000	296	15.68	15.20	16.79	15.52	15.13	15.06	15.49	15.10
0.816	0.60	1.52	Q8C0E2	Vacuolar protein sorting-associated protein 26B	Vps26b	3334	12	11	11	38.7	36.6	36.6	39.124	0.0000	33.515	20563000	124	15.94	15.93	17.30	15.84	15.48	15.95	15.47	15.72
2.772	0.60	1.52	Q99KK7	Dipeptidyl peptidase 3	Dpp3	4382	35	35	35	58.1	58.1	58.1	82.897	0.0000	323.31	132800000	610	18.47	18.56	18.33	18.59	18.11	17.66	17.96	17.83
0.328	0.60	1.51	Q6ZPR5	Sphingomyelin phosphodiesterase 4	Smpd4	2680	6	6	6	10.4	10.4	10.4	93.275	0.0000	27.803	8776000	49	15.90	15.16	14.18	15.61	15.05	15.40	15.40	12.59
2.430	0.60	1.51	P26040	Ezrin	Ezr	915	56	39	39	76.6	59	59	69.406	0.0000	323.31	236140000	1373	18.31	17.97	18.58	18.15	17.69	17.62	17.65	17.66
0.683	0.60	1.51	Q9ER69	Pre-mRNA-splicing regulator WTAP	Wtap	5295	4	4	4	15.2	15.2	15.2	44.176	0.0000	23.972	1873200	41	13.64	13.26	15.14	13.80	13.25	13.22	13.28	13.70
1.281	0.60	1.51	Q9QYB1	Chloride intracellular channel protein 4	Clic4	5566	20	20	20	88.9	88.9	88.9	28.729	0.0000	323.31	169020000	557	18.60	18.29	19.17	18.73	18.21	17.61	18.37	18.20
0.801	0.60	1.51	Q8CEE7	Retinol dehydrogenase 13	Rdh13	3473	9	9	9	38.9	38.9	38.9	36.464	0.0000	83.626	19061000	171	16.43	15.73	17.43	16.43	16.17	15.74	15.69	16.04
0.792	0.60	1.51	Q5DTM8	E3 ubiquitin-protein ligase BRE1A	Rnf20	2096	16	16	16	24.9	24.9	24.9	113.52	0.0000	80.923	15888000	99	16.15	15.82	17.48	16.51	15.88	15.60	16.06	16.04
0.720	0.60	1.51	O55022	Membrane-associated progesterone receptor component 1	Pgrmc1	442	10	10	10	32.8	32.8	32.8	21.694	0.0000	56.186	18398000	76	16.22	15.72	17.49	16.15	15.60	15.55	15.97	16.07
0.495	0.60	1.51	Q3UA37	Glutamine-rich protein 1	Qrich1	1939	6	6	6	11.8	11.8	11.8	86.555	0.0000	30.165	6605100	74	14.25	14.77	15.24	14.42	12.62	14.52	14.91	14.26
0.888	0.60	1.51	Q9EPB5	Serine hydrolase-like protein	Serhl	5251	3	3	3	18	18	18	35.31	0.0000	10.493	2197000	27	14.41	13.28	13.50	14.07	13.43	13.56	12.58	13.31
1.065	0.60	1.51	P48962	ADP/ATP translocase 1	Slc25a4	1152	36	21	21	70.5	46.3	46.3	32.904	0.0000	181.39	141290000	410	19.33	19.14	20.35	19.38	19.15	19.00	18.65	19.02
2.178	0.60	1.51	O09159	Lysosomal alpha-mannosidase	Man2b1	328	35	35	35	44.6	44.6	44.6	114.65	0.0000	323.31	264190000	982	18.98	18.78	19.14	18.97	18.64	18.28	18.52	18.07
1.068	0.59	1.51	Q3ULJ0	Glycerol-3-phosphate dehydrogenase 1-like protein	Gpd1l	1993	18	18	16	56.7	56.7	51.6	38.225	0.0000	120.9	45532000	410	16.63	16.70	17.68	17.11	16.16	16.17	16.75	16.66
1.432	0.59	1.51	Q7TSV4	Phosphoglucomutase-2	Pgm2	2789	22	22	22	49.2	49.2	49.2	68.747	0.0000	221.78	67493000	561	17.66	17.37	18.16	17.15	17.00	17.08	16.89	16.98
1.802	0.59	1.51	Q3U7R1	Extended synaptotagmin-1	Esyt1	1932	79	79	79	69.3	69.3	69.3	121.55	0.0000	323.31	751930000	2376	19.11	18.95	19.72	19.03	18.56	18.65	18.67	18.55
0.872	0.59	1.51	Q3UMB9	WASH complex subunit 7	Kiaa1033	1999	35	35	35	39	39	39	136.37	0.0000	305.48	74192000	501	17.89	17.84	19.00	17.96	17.70	17.17	18.08	17.37
1.360	0.59	1.51	Q8VC70	RNA-binding motif, single-stranded-interacting protein 2	Rbms2	3911	3	2	2	11	9.1	9.1	40.724	0.0008	4.1985	1013100	15	13.50	13.81	14.01	13.36	12.71	12.84	13.30	13.46
2.075	0.59	1.51	Q9CZD3	Glycine--tRNA ligase	Gars	4825	39	39	39	57.3	57.3	57.3	81.877	0.0000	323.31	138380000	924	17.45	17.17	17.56	17.58	16.78	16.65	17.20	16.76
1.971	0.59	1.51	P11688	Integrin alpha-5;Integrin alpha-5 heavy chain;Integrin alpha-5 light chair	Itga5	749	16	16	16	18	18	18	115.04	0.0000	217.49	28433000	218	16.52	16.00	16.67	16.31	15.87	15.61	15.92	15.73
0.796	0.59	1.51	Q8CBY8	Dynactin subunit 4	Dctn4	3441	14	14	14	35.8	35.8	35.8	53.056	0.0000	109.19	39263000	285	17.18	16.83	18.26	17.60	17.06	16.38	17.32	16.74
0.498	0.59	1.50	Q8JZN7	Mitochondrial Rho GTPase 2	Rhot2	3576	6	5	5	17.6	15.8	15.8	69.07	0.0000	15.468	2509300	23	12.98	12.56	14.47	14.39	13.40	12.32	13.12	13.20
0.509	0.59	1.50	O54916	RalBP1-associated Eps domain-containing protein 1	Reps1	432	11	11	11	18.9	18.9	18.9	86.518	0.0000	31.755	8496100	103	14.78	14.49	16.83	15.19	14.53	14.96	14.63	14.81
0.494	0.59	1.50	Q9JI90	E3 ubiquitin-protein ligase RNF14	Rnf14	5391	6	6	6	18.6	18.6	18.6	54.926	0.0000	26.98	3132900	33	14.37	13.87	12.91	14.50	12.68	12.70	13.51	14.40
0.594	0.59	1.50	P70182	Phosphatidylinositol 4-phosphate 5-kinase type-1 alpha	Pip5k1a	1530	6	5	5	17.2	14.7	14.7	60.484	0.0000	18.905	5004500	68	14.73	13.91	15.96	14.76	14.05	13.79	14.53	14.63
0.283	0.59	1.50	Q9DD03	Ras-related protein Rab-13	Rab13	5240	9	6	5	44.6	35.1	30.2	22.77	0.0000	6.4338	3378400	22	14.24	13.53	15.88	14.51	14.41	12.23	13.56	15.60
0.622	0.59	1.50	B9EJ80		Pdzd8	92	3	3	3	4.2	4.2	4.2	127.74	0.0000	22.431	1827600	19	14.02	13.67	14.31	13.75	12.67	14.02	14.15	12.56
1.835	0.59	1.50	P12265	Beta-glucuronidase	Gusb	755	37	37	37	49.7	49.7	49.7	74.194	0.0000	323.31	477460000	1070	19.93	20.08	20.54	19.93	19.70	19.52	19.64	19.26
0.538	0.59	1.50	D3Z4S3	Putative peptidyl-tRNA hydrolase PTRHD1	Ptrhd1	213	4	4	4	29.3	29.3	29.3	16.037	0.0000	12.369	10124000	55	15.62	15.74	16.42	15.54	15.41	16.04	15.61	13.90
1.106	0.59	1.50	Q5XKN4	Protein jagunal homolog 1	Jagn1	2174	4	4	4	14.8	14.8	14.8	21.102	0.0000	14.132	14069000	126	16.27	15.79	16.94	16.13	15.60	16.08	15.46	15.64
1.252	0.59	1.50	Q9R0E1	Procollagen-lysine,2-oxoglutarate 5-dioxygenase 3	Plod3	5623	22	22	22	38.7	38.7	38.7	84.921	0.0000	149.01	27642000	261	15.78	15.99	16.61	16.73	15.62	15.89	15.77	15.48
0.902	0.59	1.50	Q8R480	Nuclear pore complex protein Nup85	Nup85	3866	11	11	11	20.6	20.6	20.6	74.775	0.0000	86.218	12429000	132	15.70	15.64	16.63	15.62	15.22	15.74	15.54	14.72
0.291	0.59	1.50	P60762	Mortality factor 4-like protein 1	Morf4l1	1359	6	6	6	24.9	24.9	24.9	41.492	0.0000	10.871	4844900	27	15.29	13.87	16.82	15.60	15.19	14.85	15.98	13.21
1.610	0.59	1.50	Q8CIH5	1-phosphatidylinositol 4,5-bisphosphate phosphodiesterase gamma-2	Plcg2	3555	81	81	80	61.7	61.7	61.7	147.59	0.0000	323.31	365540000	1784	18.28	18.02	18.90	18.34	17.77	17.71	17.99	17.72
1.424	0.59	1.50	Q9R1T2	SUMO-activating enzyme subunit 1;SUMO-activating enzyme subunit 1	Sae1	5663	23	23	22	66.6	66.6	66.3	38.62	0.0000	176.25	55197000	389	16.48	16.18	17.13	16.91	16.09	16.19	15.92	16.15
0.755	0.59	1.50	Q810J8	Zinc finger FYVE domain-containing protein 1	Zfyve1	2928	11	11	11	21.5	21.5	21.5	86.939	0.0000	68.267	7636600	108	14.76	14.38	16.03	14.84	14.43	14.17	14.80	14.26
1.644	0.59	1.50	Q6P5F9	Exportin-1	Xpo1	2578	27	27	27	31.2	31.2	31.2	123.09	0.0000	306.22	96605000	571	17.67	17.64	18.03	17.85	17.37	16.71	17.45	17.31
0.848	0.59	1.50	Q6PHN9	Ras-related protein Rab-35	Rab35	2640	12	12	11	64.7	64.7	59.2	23.025	0.0000	151.9	47404000	340	16.93	16.32	17.89	17.28	16.37	16.33	16.82	16.55
1.001	0.59	1.50	P70280	Vesicle-associated membrane protein 7	Vamp7	1545	7	7	7	41.4	41.4	41.4	24.967	0.0000	174.8	15858000	150	16.18	15.65	16.79	15.96	15.95	15.09	15.68	15.50
2.866	0.59	1.50	P13020	Gelsolin	Gsn	763	62	62	24	75.3	75.3	34.5	85.941	0.0000	323.31	1259400000	2044	21.31	21.55	21.68	21.45	20.80	21.05	20.79	21.02
2.208	0.59	1.50	Q91V12	Cytosolic acyl coenzyme A thioester hydrolase	Acot7	4043	14	14	14	39.4	39.4	39.4	42.536	0.0000	83.054	73204000	310	17.44	17.27	17.49	17.44	16.53	17.08	17.03	16.66
0.996	0.59	1.50	O88448	Kinesin light chain 2	Klc2	531	13	6	6	27	16.5	16.5	66.662	0.0000	26.582	6356300	60	15.12	15.37	15.50	15.37	14.05	14.81	14.67	15.47

0.604	0.58	1.50	Q3TPX4	Exocyst complex component 5	Exoc5		1867	19	19	19	38	38	38	81.737	0.0000	130.82	18172000	228	15.71	15.02	17.13	15.74	14.99	15.52	15.45	15.30
2.150	0.58	1.50	P56395	Cytochrome b5	Cyb5a		1285	12	12	12	67.2	67.2	67.2	15.241	0.0000	323.31	133730000	477	18.34	18.09	18.27	18.34	17.59	17.34	17.83	17.94
1.209	0.58	1.50	P61164	Alpha-centractin	Actr1a		1373	29	29	16	70.2	70.2	42.3	42.613	0.0000	323.31	193400000	769	18.76	18.37	19.39	19.08	18.34	18.55	18.41	17.96
0.383	0.58	1.50	Q5NCF2	Trafficking protein particle complex subunit 1	Trappc1		2117	5	5	5	29.7	29.7	29.7	16.881	0.0002	5.3944	4424300	30	15.23	14.61	12.92	14.60	14.91	12.80	13.84	13.48
0.952	0.58	1.50	Q9Z2C5	Myotubularin	Mtm1		5840	24	24	24	47.1	47.1	47.1	69.558	0.0000	211.67	27418000	277	16.51	16.03	17.04	16.21	15.93	16.30	15.97	15.25
0.989	0.58	1.50	A2ADY9	Protein DDI1 homolog 2	Ddi2		18	19	19	19	58.4	58.4	58.4	44.59	0.0000	316.11	65085000	469	17.25	17.20	18.35	17.50	16.87	17.06	17.36	16.69
0.637	0.58	1.49	Q91VH2	Sorting nexin-9	Snx9		4061	18	18	18	46.1	46.1	46.1	66.545	0.0000	78.504	42059000	228	16.99	16.73	18.60	16.90	16.77	16.67	16.78	16.68
0.633	0.58	1.49	Q3UHA3	Spatacsin	Spg11		1968	12	12	12	7.4	7.4	7.4	273.93	0.0000	89.172	26211000	85	17.22	16.25	16.50	17.17	16.40	15.52	17.14	15.76
1.183	0.58	1.49	Q99P88	Nuclear pore complex protein Nup155	Nup155		4503	19	19	19	18.2	18.2	18.2	155.12	0.0000	323.31	31919000	370	16.24	16.28	17.28	16.40	15.77	16.02	16.15	15.94
0.854	0.58	1.49	Q8R418	Endoribonuclease Dicer	Dicer1		3863	5	5	5	4	4	4	216.82	0.0000	18.004	2567200	41	14.75	14.16	14.73	14.31	14.33	13.58	14.51	13.22
0.961	0.58	1.49	Q6PA06	Atlastin-2	Atl2		2593	12	12	10	24.5	24.5	21.1	66.223	0.0000	63.162	14806000	155	15.87	15.33	16.27	15.71	15.22	14.55	15.67	15.41
1.061	0.58	1.49	P47857	ATP-dependent 6-phosphofructokinase, muscle type	Pfkm		1128	16	10	10	26.8	20.1	20.1	85.268	0.0000	76.001	7793800	98	15.10	14.71	15.83	15.23	14.73	14.73	14.92	14.18
1.700	0.58	1.49	P14685	26S proteasome non-ATPase regulatory subunit 3	Psmd3		784	39	39	39	60.9	60.9	60.9	60.718	0.0000	323.31	154390000	978	17.77	17.46	18.32	17.98	17.25	17.26	17.35	17.36
1.865	0.57	1.49	Q7TSQ8	Pyruvate dehydrogenase phosphatase regulatory subunit, mitochondria	Pdpr		2787	10	10	10	16.1	16.1	16.1	99.229	0.0000	57.062	5787900	51	14.78	14.21	14.39	14.63	13.83	13.67	14.13	14.08
0.329	0.57	1.49	Q8VC31	Coiled-coil domain-containing protein 9	Ccdc9		3908	3	3	3	7.6	7.6	7.6	61.445	0.0000	6.3492	1179800	6	12.40	12.31	12.04	13.98	11.21	13.86	11.45	11.92
0.486	0.57	1.49	Q3UZ39	Leucine-rich repeat flightless-interacting protein 1	Lrrfip1		2046	9	8	8	18.9	17.3	17.3	79.248	0.0000	41.339	4674400	72	14.44	13.41	14.30	14.32	14.18	12.44	14.50	13.05
0.468	0.57	1.49	Q924T7	E3 ubiquitin-protein ligase RNF31	Rnf31		4301	14	14	14	20.2	20.2	20.2	119.31	0.0000	95.755	17989000	181	15.90	15.19	17.40	16.12	15.55	14.80	16.29	15.67
0.897	0.57	1.49	Q9D3U0	Putative tRNA pseudouridine synthase Pus10	Pus10		4970	8	8	8	20.7	20.7	20.7	59.71	0.0000	33.882	3845700	42	14.43	13.83	15.10	14.01	13.30	13.98	13.86	13.94
1.030	0.57	1.49	Q91V92	ATP-citrate synthase	Acly		4052	55	55	55	55.1	55.1	55.1	119.73	0.0000	323.31	269590000	1401	18.56	18.19	19.47	18.92	18.05	18.12	18.47	18.21
0.779	0.57	1.49	Q5SW28	Phosphoinositide 3-kinase regulatory subunit 5	Pik3r5		2154	7	7	7	16.3	16.3	16.3	96.953	0.0000	49.215	6109300	80	15.13	14.93	16.15	15.37	14.16	15.10	15.28	14.76
0.665	0.57	1.49	Q8BU31	Ras-related protein Rap-2c	Rap2c		3219	9	4	4	58.5	29.5	29.5	20.745	0.0000	21.197	18982000	115	16.58	16.03	17.28	16.47	16.20	15.08	16.55	16.24
0.873	0.57	1.49	Q8K1J6	CCA tRNA nucleotidyltransferase 1, mitochondrial	Trnt1		3629	18	18	18	50.9	50.9	50.9	49.895	0.0000	111.47	33020000	311	16.09	15.83	17.23	16.19	15.55	16.03	15.89	15.58
1.185	0.57	1.49	Q9CPQ8	ATP synthase subunit g, mitochondrial	Atp5l		4527	6	6	6	50.5	50.5	50.5	11.424	0.0000	70.611	95726000	225	19.16	19.23	19.45	18.64	19.10	18.27	18.35	18.47
2.666	0.57	1.49	Q61598	Rab GDP dissociation inhibitor beta	Gdi2		2317	58	58	51	95.3	95.3	83.4	50.537	0.0000	323.31	1315500000	2755	20.05	20.01	19.99	20.13	19.55	19.16	19.63	19.55
0.929	0.57	1.48	O08759	Ubiquitin-protein ligase E3A	Ube3a		296	15	15	15	25.7	25.7	25.7	99.818	0.0000	112.46	14874000	142	15.52	15.42	16.76	15.79	15.46	15.28	15.14	15.32
1.954	0.57	1.48	Q9R0X4;Q3	Acyl-coenzyme A thioesterase 9, mitochondrial;Acyl-coenzyme A thioes	Acot9;Acot10		5643	25	25	24	50.6	50.6	50.6	50.56	0.0000	129.22	84996000	550	17.48	17.24	17.91	17.31	16.95	17.03	16.86	16.82
1.467	0.57	1.48	Q61035	Histidine--tRNA ligase, cytoplasmic	Hars		2241	35	35	18	62.5	62.5	28.7	57.432	0.0000	323.31	139270000	784	17.55	17.46	18.35	17.89	17.10	17.22	17.37	17.27
1.776	0.57	1.48	O09106	Histone deacetylase 1	Hdac1		322	17	17	10	47.1	47.1	34.6	55.074	0.0000	265.86	116660000	403	18.75	18.60	19.25	18.69	18.13	18.09	18.29	18.51
1.608	0.57	1.48	O08800	Serpin B8	Serpinb8		301	14	14	14	53.7	53.7	53.7	42.15	0.0000	248.82	35383000	238	17.29	17.29	17.51	17.42	16.90	16.42	17.27	16.64
1.308	0.57	1.48	Q03347	Runt-related transcription factor 1	Runx1		1726	7	7	7	23.5	23.5	23.5	48.609	0.0000	33.2	7395600	56	15.41	14.97	15.81	15.67	14.53	15.17	14.82	15.06
1.088	0.57	1.48	Q9D824	Pre-mRNA 3-end-processing factor FIP1	Fip1l1		5048	13	13	13	29.6	29.6	29.6	64.958	0.0000	86.179	9022700	69	15.47	16.13	15.06	15.96	15.34	14.88	15.27	14.87
0.972	0.57	1.48	Q80XR2	Calcium-transporting ATPase type 2C member 1	Atp2c1		2891	15	15	15	24.6	24.6	24.6	100.3	0.0000	46.407	14449000	137	15.37	14.63	14.70	15.30	14.31	14.11	15.10	14.21
2.846	0.57	1.48	P24527	Leukotriene A-4 hydrolase	Lta4h		898	42	42	42	74.1	74.1	74.1	69.05	0.0000	323.31	204930000	926	18.34	18.12	18.31	18.44	17.73	17.75	17.92	17.54
0.983	0.57	1.48	Q925E7	Serine/threonine-protein phosphatase 2A 55 kDa regulatory subunit B d	Ppp2r2d		4305	11	6	6	31.1	16.1	16.1	51.957	0.0000	7.9746	2320700	15	13.42	14.20	14.68	13.79	13.50	13.41	13.74	13.18
0.752	0.57	1.48	Q8C4J7	Transducin beta-like protein 3	Tbl3		3386	8	8	7	16.2	16.2	15.1	88.265	0.0000	58.013	7397000	70	15.16	14.93	15.16	15.56	14.92	14.90	15.11	13.60
0.925	0.57	1.48	Q6PDI5	Proteasome-associated protein ECM29 homolog	Ecm29		2613	51	51	51	44.1	44.1	44.1	203.7	0.0000	323.31	91405000	799	17.37	16.97	18.39	17.63	17.02	16.94	17.27	16.87
1.436	0.57	1.48	Q60597	2-oxoglutarate dehydrogenase, mitochondrial	Ogdh		2182	54	54	54	52.3	52.3	52.3	116.45	0.0000	323.31	290220000	1361	18.41	18.02	18.99	18.55	17.88	17.81	18.13	17.88
2.274	0.57	1.48	Q9JIF7	Coatomer subunit beta	Copb1		5397	43	43	42	52.7	52.7	51.7	107.06	0.0000	323.31	143740000	870	18.70	18.50	18.82	18.80	17.94	18.26	18.39	17.97
0.858	0.57	1.48	Q80X50	Ubiquitin-associated protein 2-like	Ubap2l		2870	23	23	23	29.9	29.9	29.9	116.8	0.0000	301.15	37911000	412	16.43	16.14	17.24	16.30	15.88	16.57	15.50	15.89
0.228	0.57	1.48	Q9R1T4	Septin-6		38961	5664	18	9	8	38.2	21.9	18.7	49.619	0.0000	15.043	4177100	59	14.45	14.60	16.56	13.14	14.62	12.01	14.92	14.94
1.778	0.57	1.48	P35486	Pyruvate dehydrogenase E1 component subunit alpha, somatic form, m	Pdha1		1021	32	32	32	66.7	66.7	66.7	43.231	0.0000	129.66	105090000	638	17.32	16.78	17.59	17.29	16.67	16.60	16.76	16.69
1.046	0.57	1.48	P26043	Radixin	Rdx		917	57	40	37	75.1	57.3	54.9	68.542	0.0000	323.31	164250000	1047	17.15	16.94	18.17	17.24	16.62	16.88	16.92	16.82
0.451	0.56	1.48	Q9DC71	28S ribosomal protein S15, mitochondrial	Mrps15		5201	10	10	10	37.2	37.2	37.2	29.463	0.0000	16.428	5272700	74	14.32	14.03	16.13	14.16	13.96	14.66	14.34	13.42
0.856	0.56	1.48	Q6ZPF4	Formin-like protein 3	Fmnl3		2675	5	3	2	5.4	4.4	3.1	117.17	0.0022	3.0725	1797100	19	13.98	13.22	13.78	14.33	12.78	13.09	13.90	13.28
0.309	0.56	1.48	A0A0B4J1G0		Fcgr4		0	3	3	3	13.7	13.7	13.7	28.398	0.0000	11.71	4586100	45	14.99	13.51	15.74	14.22	14.11	14.38	15.29	12.42
0.378	0.56	1.48	Q8JZS9	39S ribosomal protein L48, mitochondrial	Mrpl48		3581	3	3	3	10.9	10.9	10.9	23.952	0.0009	3.7682	2548400	23	14.14	14.36	12.57	14.48	13.32	14.07	11.98	13.92
3.193	0.56	1.48	Q07076	Annexin A7	Anxa7		1753	33	32	32	52.7	52.7	52.7	49.925	0.0000	323.31	313780000	1119	19.00	18.93	18.94	18.90	18.56	18.47	18.30	18.18
0.570	0.56	1.48	Q99NB9	Splicing factor 3B subunit 1	Sf3b1		4497	49	49	49	42.1	42.1	42.1	145.81	0.0000	323.31	62711000	588	16.46	16.01	18.01	16.50	15.84	16.12	16.60	16.17
1.127	0.56	1.48	P60229	Eukaryotic translation initiation factor 3 subunit E	Eif3e		1352	25	25	25	55.7	55.7	55.7	52.22	0.0000	187.06	106230000	541	17.93	18.09	18.85	17.76	17.82	17.35	17.70	17.49
1.027	0.56	1.48	Q8K4X7	1-acyl-sn-glycerol-3-phosphate acyltransferase delta	Agpat4		3729	11	11	11	24.9	24.9	24.9	43.81	0.0000	26.729	11661000	97	14.94	15.09	15.69	15.76	14.49	15.24	15.03	14.48
0.804	0.56	1.48	Q8CFD4	Sorting nexin-8	Snx8		3480	13	13	13	36.6	36.6	36.6	52.058	0.0000	109.67	17582000	133	15.46	15.14	16.12	15.59	15.20	15.23	15.44	14.19
1.013	0.56	1.48	Q922F4	Tubulin beta-6 chain	Tubb6		4254	30	17	15	72.5	55.3	50.1	50.09	0.0000	220.1	47549000	338	17.26	17.11	17.64	17.24	17.14	16.49	17.23	16.14
0.365	0.56	1.48	Q64281	Leukocyte immunoglobulin-like receptor subfamily B member 4	Lilrb4		2426	5	5	5	26	26	26	37.544	0.0000	26.155	11635000	74	17.07	17.24	15.36	15.37	16.13	14.44	15.99	16.22
1.141	0.56	1.48	Q9Z0F8	Disintegrin and metalloproteinase domain-containing protein 17	Adam17		5767	23	23	23	34.1	34.1	34.1	93.055	0.0000	115.64	32172000	278	15.95	15.47	16.58	16.22	15.53	15.30	15.78	15.35
4.691	0.56	1.48	Q9DCD0	6-phosphogluconate dehydrogenase, decarboxylating	Pgd		5207	50	50	50	89.9	89.9	89.9	53.247	0.0000	323.31	1955300000	2821	21.24	21.18	21.33	21.27	20.74	20.76	20.65	20.61
1.801	0.56	1.48	Q61792	LIM and SH3 domain protein 1	Lasp1		2335	25	25	25	62	62	62	29.994	0.0000	315.31	456020000	1129	19.73	19.39	19.46	20.00	19.09	19.20	19.23	18.81
0.311	0.56	1.48	P55065	Phospholipid transfer protein	Pltp		1265	9	9	9	26.8	26.8	26.8	54.452	0.0000	86.953	12151000	108	15.18	15.25	15.45	15.90	15.28	14.49	16.66	13.10
0.456	0.56	1.48	Q3TUH1	Phosphatidate cytidylyltransferase, mitochondrial	Tamm41		1876	8	8	8	36.8	36.8	36.8	37.827	0.0000	46.946	5348100	48	14.15	14.04	15.76	13.93	14.02	13.11	14.78	13.73
1.003	0.56	1.48	O88879	Apoptotic protease-activating factor 1	Apaf1		582	28	28	28	27.5	27.5	27.5	141	0.0000	115.68	30372000	291	16.10	15.74	16.94	16.55	15.56	15.71	16.11	15.71
0.833	0.56	1.47	Q99K70;Q7	Ras-related GTP-binding protein C;Ras-related GTP-binding protein D	Rragc;Rragd		4364	14	14	14	34.7	34.7	34.7	44.12	0.0000	53.67	17672000	239	15.80	15.67	17.01	16.52	15.44	15.73	15.60	15.99
1.665	0.56	1.47	P46638	Ras-related protein Rab-11B	Rab11b		1107	19	19	4	70.6	70.6	17.9	24.489	0.0000	206.12	207410000	471	19.22	18.94	19.72	19.28	18.53	18.69	18.79	18.91
1.183	0.56	1.47	P36552	Oxygen-dependent coproporphyrinogen-III oxidase, mitochondrial	Cpox		1043	13	13	13	35.4	35.4	35.4	49.714	0.0000	43.683	13868000	140	15.05	14.50	15.29	15.31	14.73	14.77	14.32	14.10
0.671	0.56	1.47	P00405	Cytochrome c oxidase subunit 2	Mtco2		610	8	8	8	30.8	30.8	30.8	25.976	0.0000	182.2	369640000	531	20.57	20.08	19.52	20.61	19.66	19.34	20.49	19.05
0.787	0.56	1.47	Q8BX70	Vacuolar protein sorting-associated protein 13C	Vps13c		3279	116	116	116	41.1	41.1	41.1	420.08	0.0000	323.31	199780000	1792	17.46	17.15	18.49	17.64	16.84	16.78	17.68	17.20
0.469	0.56	1.47	P97864	Caspase-7;Caspase-7 subunit p20;Caspase-7 subunit p11	Casp7		1681	5	5	5	18.8	18.8	18.8	34.06	0.0000	23.143	5812800	71	14.42	14.65	16.67	15.37	14.84	15.17	14.52	14.35
0.513	0.56	1.47	Q8K4Q7	Ceramide kinase	Cerk		3726	5	5	5	14.3	14.3	14.3	59.842	0.0000	23.475	5035300	71	14.73	14.70	16.18	14.93	15.00	14.65	15.12	13.55
1.361	0.56	1.47	P62889	60S ribosomal protein L30	Rpl30		1463	13	13	13	82.6	82.6	82.6	12.784	0.0000	84.226	227260000	516	19.37	19.27	20.11	19.88	19.33	19.09	19.06	18.93
1.146	0.56	1.47	Q8BNU0	Armadillo repeat-containing protein 6	Armc6		3155	8	8	8	29.7	29.7	29.7	50.683	0.0000	67.483	12470000	132	15.43	15.19	16.12	15.65	15.20	14.57	15.30	15.09
1.018	0.56	1.47	O35350	Calpain-1 catalytic subunit	Capn1		361	32	32	32	50.1	50.1	50.1	82.105	0.0000	298.56	90110000	575	17.40	17.35	18.44	17.39	17.13	17.01	17.34	16.88
0.418	0.56	1.47	P01942	Hemoglobin subunit alpha	Hba		624	6	4	4	42.3	35.9	35.9	15.085	0.0000	14.277	11636000	71	15.91	15.01	14.65	14.64	15.55	14.51	14.80	13.12
0.329	0.56	1.47	Q8R0F8	Acylpyruvase FAHD1, mitochondrial	Fahd1		3757	3	3	3	28.2	28.2	28.2	25.172	0.0000	32.541	2157600	27	14.93	15.04	12.26	13.11	13.12	13.32	12.83	13.85
0.470	0.56	1.47	Q9JHS3	Ragulator complex protein LAMTOR2	Lamtor2		5371	6	6	6	70.4	70.4	70.4	13.48	0.0000	211.36	19603000	139	17.21	16.80	15.62	17.85	16.92	16.49	15.72	16.12
1.567	0.56	1.47	Q9CVB6	Actin-related protein 2/3 complex subunit 2	Arpc2		4706	35	35	35	90.3	90.3	90.3	34.357	0.0000	323.31	464170000	1489	19.15	19.10	19.45	19.40	18.94	18.22	18.80	18.92
1.042	0.56	1.47	Q9CYN9	Renin receptor	Atp6ap2		4804	9	9	9	31.4	31.4	31.4	39.091	0.0000	123.69	24846000	163	16.06	15.97	17.09	16.63	15.71	16.06	16.00	15.77
1.437	0.55	1.47	Q91V41	Ras-related protein Rab-14	Rab14		4046	25	25	25	87.4	87.4	87.4	23.897	0.0000	323.31	288280000	900	19.21	19.01	19.78	19.26	18.76	18.58	19.11	18.60
0.523	0.55	1.47	P04627	Serine/threonine-protein kinase A-Raf	Araf		643	11	7	6	25.3	18.2	15.6	67.581	0.0000	23.665	10799000	64	15.15	15.06	14.38	15.27	13.19	14.68	15.31	14.49
2.427	0.55	1.47	O70435	Proteasome subunit alpha type-3	Psma3		498	21	21	21	69.4	69.4	69.4	28.405	0.0000	271.11	308030000	525	20.40	20.25	20.61	20.57	19.84	19.92	20.14	19.72
1.087	0.55	1.47	Q6PDQ2	Chromodomain-helicase-DNA-binding protein 4	Chd4		2620	50	50	39	32.1	32.1	25.8	217.75	0.0000	323.31	72070000	674	16.21	15.92	17.05	16.63	15.96	15.62	16.03	16.00
0.307	0.55	1.46	Q8BGW1	Alpha-ketoglutarate-dependent dioxygenase FTO	Fto		2998	6	6	6	15.7	15.7	15.7	58.006	0.0000	14.802	9123300	49	16.11	16.43	14.83	16.57	16.06	13.69	16.65	15.34
1.028	0.55	1.46	P97371	Proteasome activator complex subunit 1	Psme1		1636	21	20	20	72.3	67.9	67.9	28.673	0.0000	193.22	53621000	446	16.96	17.19	16.32	17.27	16.57	15.86	16.58	16.52
0.427	0.55	1.46	Q922B6	E3 ubiquitin-protein ligase TRAF7	Traf7		4248	3	3	3	8.9	8.9	8.9	66.487	0.0000	8.9023	1961500	9	14.33	13.98	13.81	13.33	12.71	14.04	14.38	12.12
0.603	0.55	1.46	O08917	Flotillin-1	Flot1		312	19	19	19	52.8	52.8	52.8	47.513	0.0000	61.964	24446000	155	15.89	15.17	17.06	15.56	15.34	15.22	15.77	15.16
0.559	0.55	1.46	P36371	Antigen peptide transporter 2	Tap2		1040	15	15	15	29.2	29.2	29.2	77.444	0.0000	303.14	39051000	297	15.59	15.58	17.29	16.22	15.56	15.03	15.90	15.99
0.987	0.55	1.46	Q60676	Serine/threonine-protein phosphatase 5	Ppp5c		2193	19	19	19	43.1	43.1	43.1	56.876	0.0000	146.07	52535000	329	17.53	17.12	18.44	17.84	17.03	17.27	17.20	17.23
1.443	0.55	1.46	P05201	Aspartate aminotransferase, cytoplasmic	Got1		649	34	34	34	80.6	80.6	80.6	46.247	0.0000	323.31	264300000	805	18.76	18.54	19.39	18.93	18.30	18.36	18.60	18.16
0.869	0.55	1.46	Q9CZU3	Superkiller viralicidic activity 2-like 2	Skiv2l2		4839	35	35	35	39.8	39.8	39.8	117.64	0.0000	272.84	56433000	435	16.81	16.24	17.61	16.97	16.18	16.29	16.79	16.17
1.568	0.55	1.46	Q9QZD9	Eukaryotic translation initiation factor 3 subunit I	Eif3i		5594	21	21	21	70.5	70.5	70.5	36.46	0.0000	322.13	124840000	578	17.86	17.49	18.18	18.11	17.49	17.07	17.52	17.38
0.454	0.54	1.46	Q8VCI0	Phospholipase B-like 1;Phospholipase B-like 1 chain A;Phospholipase E	Plbd1		3923	18	18	18	36.4	36.4	36.4	62.998	0.0000	251.87	70800000	405	17.73	17.27	17.59	17.86	18.62	16.35	16.74	16.56
0.985	0.54	1.46	Q99M15	Proline-serine-threonine phosphatase-interacting protein 2	Pstpip2		4453	15	15	14	55.4	55.4	53.3	38.948	0.0000	141.47	43607000	340	17.19	16.64	17.87	17.59	16.87	16.54	16.98	16.72
0.146	0.54	1.46	P47955	60S acidic ribosomal protein P1	Rplp1		1133	3	3	3	79.8	79.8	79.8	11.475	0.0000	87.004	25982000	67	18.83	18.49	15.41	18.34	17.16	18.92	18.89	13.93
0.593	0.54	1.46	Q8BKT3	GC-rich sequence DNA-binding factor 2	Gcfc2		3105	5	5	5	9.8	9.8	9.8	87.441	0.0000	13.178	2762000	19	14.65	14.12	15.06	14.37	14.73	13.00	13.85	14.44
2.313	0.54	1.46	P62137	Serine/threonine-protein phosphatase PP1-alpha catalytic subunit	Ppp1ca		1404	22	22	5	63.3	63.3	13.9	37.54	0.0000	323.31	192260000	667	18.68	18.58	19.12	18.76	18.21	18.35	18.16	18.25
1.248	0.54	1.45	Q76MZ3	Serine/threonine-protein phosphatase 2A 65 kDa regulatory subunit A a	Ppp2r1a		2719	41	41	31	70.6	70.6	57	65.322	0.0000	323.31	421040000	1558	18.75	18.61	19.51	18.74	18.51	18.09	18.52	18.34
0.414	0.54	1.45	Q9CT10	Ran-binding protein 3	Ranbp3		4702	8	8	8	22.6	22.6	22.6	52.572	0.0000	42.038	8292700	103	15.15	15.06	16.84	14.93	14.44	15.67	14.24	15.47
1.053	0.54	1.45	P05213	Tubulin alpha-1B chain	Tuba1b		651	36	36	0	72.7	72.7	0	50.151	0.0000	323.31	2761500000	3433	22.52	22.37	21.83	22.55	22.14	21.21	22.03	21.73
1.269	0.54	1.45	Q99KF1	Transmembrane emp24 domain-containing protein 9	Tmed9		4373	14	14	12	49.8	49.8	39.6	27.127	0.0000	229.95	75405000	235	17.99	17.68	18.66	18.22	17.84	17.62	17.44	17.50
1.644	0.54	1.45	Q9WTR1	Transient receptor potential cation channel subfamily V member 2	Trpv2		5684	22	22	22	32.7	32.7	32.7	85.964	0.0000	252.58	82567000	525	17.20	16.98	17.61	17.05	16.66	16.48	16.98	16.57
0.588	0.54	1.45	Q8C0J2	Autophagy-related protein 16-1	Atg16l1		3338	15	15	15	32.8	32.8	32.8	68.171	0.0000	119.44	10250000	135	14.85	14.60	16.45	15.12	14.55	14.47	15.01	14.85
1.039	0.54	1.45	Q80U78	Pumilio homolog 1	Pum1		2824	15	15	11	16.8	16.8	13.2	126.62	0.0000	47.309	13490000	147	15.58	14.98	16.08	15.20	14.58	15.08	14.93	15.09
0.376	0.54	1.45	Q99LS3	Phosphoserine phosphatase	Psph		4445	11	11	11	54.7	54.7	54.7	25.096	0.0000	19.508	12852000	113	13.98	15.23	16.56	15.63	13.92	14.85	15.37	15.10
1.264	0.54	1.45	Q9WTU6	Mitogen-activated protein kinase 9	Mapk9		5689	4	4	2	9.9	9.9	5.7	48.189	0.0004	4.926	6219200	55	14.98	14.75	14.77	15.49	14.30	14.28	14.89	14.36
1.222	0.54	1.45	Q99N16	Leukotriene-B(4) omega-hydroxylase 2	Cyp4f3		4482	2	2	2	4.8	4.8	4.8	59.842	0.0015	3.3629	1977100	18	14.43	14.08	14.74	14.71	13.65	13.83	14.45	13.88
0.876	0.54	1.45	O88351	Inhibitor of nuclear factor kappa-B kinase subunit beta	Ikbkb		524	25	25	23	38.2	38.2	38.2	86.689	0.0000	104.58	27071000	250	15.87	15.18	16.60	15.66	15.51	15.10	15.23	15.33
1.187	0.54	1.45	Q8BH61	Coagulation factor XIII A chain	F13a1		3017	29	29	29	43.2	43.2	43.2	83.206	0.0000	122.93	86112000	369	17.63	17.38	18.04	17.29	17.17	17.43	16.94	16.65
0.914	0.53	1.45	Q3TIR1	Trafficking protein particle complex subunit 13	Trappc13		1848	6	6	6	22.3	22.3	22.3	46.476	0.0000	57.481	10301000	82	15.57	15.46	16.70	16.00	15.28	15.32	15.69	15.31
0.772	0.53	1.45	E9Q735	Ubiquitin conjugation factor E4 A	Ube4a		244	8	8	8	14.2	14.2	14.2	118.2	0.0000	81.573	3920600	36	14.99	13.61	14.55	14.78	14.03	13.72	14.36	13.69
0.791	0.53	1.45	E9Q137		Tex264		227	13	13	13	47.2	47.2	47.2	33.589	0.0000	95.981	25909000	157	16.74	16.08	16.53	16.53	15.99	16.16	16.52	15.08
1.212	0.53	1.45	P57780	Alpha-actinin-4	Actn4		1300	94	94	67	86.2	86.2	67.9	104.98	0.0000	323.31	715250000	2744	19.38	19.00	20.06	19.39	18.75	18.86	19.08	19.01

0.928	0.53	1.45	Q6P6M7	O-phosphoseryl-tRNA(Sec) selenium transferase	Sepsecs	2581	5	5	5	19.8	19.8	19.8	55.325	0.0000	31.756	4372300	52	14.30	14.30	15.46	14.99	14.03	14.23	14.38	14.27
1.859	0.53	1.45	P17047	Lysosome-associated membrane glycoprotein 2	Lamp2	817	10	10	10	14.2	14.2	14.2	45.681	0.0000	80.008	39421000	227	17.58	17.45	17.50	17.88	17.41	17.08	16.90	16.89
0.488	0.53	1.45	Q922E4	Ethanolamine-phosphate cytidylyltransferase	Pcyt2	4252	8	8	8	23.8	23.8	23.8	45.234	0.0000	27.084	8394800	65	15.72	15.43	16.44	15.69	15.47	13.97	15.78	15.93
1.239	0.53	1.45	Q9D906	Ubiquitin-like modifier-activating enzyme ATG7	Atg7	5089	33	33	33	54	54	54	77.519	0.0000	302.48	77517000	514	16.47	16.36	17.18	16.72	16.31	15.80	16.42	16.07
0.477	0.53	1.45	Q9CQU3	Protein RER1	Rer1	4635	6	6	6	34.7	34.7	34.7	22.988	0.0000	19.899	4460800	34	14.59	15.13	15.23	13.89	15.08	13.27	14.57	13.79
0.209	0.53	1.45	P11672	Neutrophil gelatinase-associated lipocalin	Lcn2	748	5	5	5	28.5	28.5	28.5	22.875	0.0000	10.456	3434200	30	13.25	12.84	15.69	15.73	15.65	13.93	12.97	12.84
0.328	0.53	1.44	P15535	Beta-1,4-galactosyltransferase 1;Lactose synthase A protein;N-acetylla	B4galt1	799	5	5	5	13.5	13.5	13.5	44.411	0.0000	11.029	5956600	34	15.01	15.09	12.79	14.44	14.37	12.54	14.37	13.94
0.410	0.53	1.44	Q9DBH0	NEDD4-like E3 ubiquitin-protein ligase WWP2	Wwp2	5156	4	3	3	6.3	5.4	5.4	98.76	0.0000	29.72	1723200	35	14.21	12.84	14.32	12.68	12.31	13.23	12.48	13.92
2.348	0.53	1.44	P60843	Eukaryotic initiation factor 4A-I	Eif4a1	1362	38	38	22	80.3	80.3	51.5	46.153	0.0000	323.31	883380000	2132	20.28	20.20	19.88	20.39	19.60	19.55	19.76	19.71
0.314	0.53	1.44	Q9JLZ3	Methylglutaconyl-CoA hydratase, mitochondrial	Auh	5494	6	6	6	20.4	20.4	20.4	33.395	0.0000	44.154	3485300	41	12.64	13.72	15.45	14.09	13.86	14.11	13.56	12.25
0.691	0.53	1.44	Q9CYH2	Redox-regulatory protein FAM213A	Fam213a	4797	5	5	5	17.4	17.4	17.4	24.394	0.0000	19.949	5429300	68	14.30	14.37	15.38	15.27	14.09	14.96	14.28	13.88
1.401	0.53	1.44	Q9JI11	Serine/threonine-protein kinase 4;Serine/threonine-protein kinase 4 37k	Stk4	5382	22	22	17	47.8	47.8	39.6	55.541	0.0000	178.14	67326000	559	17.06	16.76	17.14	17.13	16.29	16.28	17.03	16.37
0.615	0.53	1.44	P56542	Deoxyribonuclease-2-alpha	Dnase2	1289	4	4	4	17	17	17	38.81	0.0000	19.833	2391500	25	13.53	14.02	14.60	14.21	14.13	13.04	14.18	12.89
0.398	0.53	1.44	Q9CQ06	39S ribosomal protein L24, mitochondrial	Mrpl24	4551	6	6	6	43.5	43.5	43.5	24.944	0.0000	18.105	2782800	47	13.13	14.63	15.34	13.91	14.61	12.96	13.64	13.67
0.625	0.53	1.44	Q3U2S8	Voltage-gated hydrogen channel 1	Hvcn1	1913	8	8	8	34.6	34.6	34.6	31.242	0.0000	100.33	9528400	74	15.41	15.30	16.20	15.75	14.94	15.92	14.28	15.40
0.825	0.53	1.44	O09110	Dual specificity mitogen-activated protein kinase kinase 3	Map2k3	323	19	19	16	63.1	63.1	56.2	39.296	0.0000	127.14	31001000	245	16.51	16.31	17.61	16.79	16.06	16.01	16.55	16.50
1.038	0.53	1.44	Q9CXW3	Calcyclin-binding protein	Cacybp	4771	23	23	23	90.8	90.8	90.8	26.51	0.0000	323.31	74229000	598	16.79	16.30	17.55	16.77	16.20	16.40	16.32	16.37
0.716	0.53	1.44	Q69ZS7	HBS1-like protein	Hbs1l	2492	7	7	7	14.2	14.2	14.2	75.1	0.0000	22.163	4945400	59	14.74	13.90	15.26	14.05	13.73	14.29	14.21	13.61
0.657	0.53	1.44	Q8K245		Uvrag	3644	9	9	9	14.6	14.6	14.6	77.524	0.0000	15.236	4732400	52	13.90	14.09	13.69	14.98	13.88	13.40	14.23	13.05
2.265	0.53	1.44	Q8R1F1	Niban-like protein 1	Fam129b	3786	54	54	54	71.8	71.8	71.8	84.818	0.0000	323.31	382760000	1424	19.01	18.85	19.23	18.76	18.64	18.42	18.35	18.34
0.520	0.53	1.44	P31266	Recombining binding protein suppressor of hairless	Rbpj	986	15	15	15	32.1	32.1	32.1	58.537	0.0000	65.088	26345000	207	16.32	15.73	17.03	16.97	15.56	15.22	16.46	16.68
2.021	0.53	1.44	P83887;Q8V	Tubulin gamma-1 chain;Tubulin gamma-2 chain	Tubg1;Tubg2	1604	10	10	10	41	41	41	51.1	0.0000	108.45	8161500	99	14.94	14.68	14.98	15.01	14.36	14.65	14.08	14.41
0.768	0.53	1.44	Q8BN21	Serine/threonine-protein kinase VRK2	Vrk2	3152	10	10	10	23.9	23.9	23.9	58.118	0.0000	29.703	14006000	87	15.91	15.46	16.47	16.36	14.91	15.70	16.08	15.40
0.647	0.53	1.44	Q9D0G0	28S ribosomal protein S30, mitochondrial	Mrps30	4866	12	12	12	35.7	35.7	35.7	49.939	0.0000	97.177	15593000	116	15.99	15.86	17.35	15.77	15.73	15.55	16.02	15.57
0.518	0.53	1.44	Q9JJT0	RNA 3-terminal phosphate cyclase-like protein	Rcl1	5434	3	3	3	10.7	10.7	10.7	40.84	0.0000	7.6851	4876200	42	15.12	14.41	15.54	15.60	14.30	13.99	14.55	15.72
0.912	0.53	1.44	P62082	40S ribosomal protein S7	Rps7	1403	28	28	28	84	84	84	22.127	0.0000	323.31	873660000	1180	21.10	21.01	21.37	21.44	20.83	19.91	21.14	20.94
1.038	0.52	1.44	Q6P4T2	U5 small nuclear ribonucleoprotein 200 kDa helicase	Snrnp200	2566	70	70	70	43.5	43.5	43.5	244.54	0.0000	323.31	146540000	1067	17.53	17.46	18.50	17.52	17.01	17.25	17.38	17.27
0.509	0.52	1.44	Q64455	Receptor-type tyrosine-protein phosphatase eta	Ptprj	2445	15	15	15	13.1	13.1	13.1	136.77	0.0000	73.657	14105000	130	15.88	15.28	17.45	16.38	15.71	15.44	15.98	15.76
0.271	0.52	1.44	Q9Z0R4	Intersectin-1	Itsn1	5781	12	12	12	11.8	11.8	11.8	194.29	0.0000	31.723	11929000	72	16.25	14.96	17.99	15.98	16.55	16.32	15.85	14.37
1.101	0.52	1.44	B9EJ86	Oxysterol-binding protein	Osbpl8	93	44	44	42	50.4	50.4	49.5	101.27	0.0000	323.31	192960000	1091	18.01	17.63	18.74	18.17	17.56	17.75	17.76	17.39
1.967	0.52	1.44	Q91WK1	SPRY domain-containing protein 4	Spryd4	4115	5	5	5	27.1	27.1	27.1	23.275	0.0004	5.1022	1957300	18	14.02	13.58	13.66	13.99	13.22	13.13	13.55	13.26
1.281	0.52	1.44	Q91YE6	Importin-9	Ipo9	4155	19	19	19	28.8	28.8	28.8	116.05	0.0000	323.31	59831000	469	17.42	17.12	17.95	17.51	17.00	16.75	17.34	16.81
0.424	0.52	1.44	Q3UR70	Transforming growth factor-beta receptor-associated protein 1	Tgfbrap1	2020	6	6	6	10.7	10.7	10.7	97.302	0.0000	21.769	2668800	53	13.92	13.94	15.03	14.16	12.78	15.05	13.41	13.72
0.487	0.52	1.44	Q8R4N0	Citrate lyase subunit beta-like protein, mitochondrial	Clybl	3871	12	12	12	43.5	43.5	43.5	37.548	0.0000	52.248	7068300	101	14.57	14.28	16.06	15.11	14.37	15.24	14.52	13.81
0.802	0.52	1.44	Q8BK64	Activator of 90 kDa heat shock protein ATPase homolog 1	Ahsa1	3095	20	20	20	55.3	55.3	55.3	38.117	0.0000	323.31	104240000	514	18.16	17.67	19.15	18.23	17.66	17.66	18.07	17.74
0.709	0.52	1.44	O88597	Beclin-1	Becn1	549	6	6	6	20.3	20.3	20.3	51.588	0.0000	17.275	6261800	54	14.62	14.19	14.72	14.83	14.42	13.61	14.82	13.43
0.861	0.52	1.44	Q8BYM8	Probable cysteine--tRNA ligase, mitochondrial	Cars2	3304	2	2	2	5.8	5.8	5.8	61.271	0.0002	5.794	809090	20	13.19	12.81	13.46	13.07	12.50	13.37	12.51	12.06
0.619	0.52	1.44	Q8C7V3	U3 small nucleolar RNA-associated protein 15 homolog	Utp15	3416	7	7	7	18.8	18.8	18.8	59.374	0.0000	32.057	10047000	98	15.41	15.10	16.42	15.91	14.99	15.36	15.85	14.56
1.334	0.52	1.44	Q8K2K6	Arf-GAP domain and FG repeat-containing protein 1	Agfg1	3664	9	9	7	23.5	23.5	21.6	58.042	0.0000	69.107	13236000	150	15.62	15.36	16.04	15.85	15.09	15.54	15.31	14.85
2.587	0.52	1.43	P63101	14-3-3 protein zeta/delta	Ywhaz	1491	37	37	29	84.1	84.1	77.6	27.771	0.0000	323.31	1581100000	2128	21.53	21.49	21.91	21.57	21.03	21.20	21.15	21.04
0.669	0.52	1.43	Q8CGA3	Large neutral amino acids transporter small subunit 4	Slc43a2	3503	8	8	8	10.7	10.7	10.7	62.43	0.0000	38.472	13643000	149	15.57	15.01	16.52	15.36	14.60	15.53	15.17	15.07
1.008	0.52	1.43	Q9DAR7	m7GpppX diphosphatase	Dcps	5109	21	21	21	59.8	59.8	59.8	38.988	0.0000	113.83	54933000	293	17.08	16.75	17.38	17.24	15.92	16.77	16.93	16.76
1.419	0.52	1.43	Q9CZ42	ATP-dependent (S)-NAD(P)H-hydrate dehydratase	Carkd	4817	12	12	12	54.2	54.2	54.2	36.717	0.0000	165.84	39397000	320	16.96	16.87	17.26	16.88	16.59	16.45	16.84	16.01
0.750	0.52	1.43	B2RXC1	Trafficking protein particle complex subunit 11	Trappc11	84	24	24	24	32.7	32.7	32.7	128.39	0.0000	120.22	32634000	331	16.18	16.09	17.54	16.49	16.05	16.07	16.23	15.87
0.533	0.52	1.43	Q99KG3	RNA-binding protein 10	Rbm10	4374	11	10	10	19.9	18.7	18.7	103.49	0.0000	24.586	3990300	41	13.48	14.45	15.46	14.41	14.31	14.12	13.39	13.91
0.902	0.52	1.43	O70310	Glycylpeptide N-tetradecanoyltransferase 1	Nmt1	486	27	27	23	46.6	46.6	42.7	56.888	0.0000	323.31	90814000	558	17.51	17.23	18.52	17.63	17.26	17.13	17.41	17.01
1.565	0.52	1.43	Q7TN98	Cytoplasmic polyadenylation element-binding protein 4	Cpeb4	2757	13	13	9	25.1	25.1	17.7	80.121	0.0000	93.945	10911000	144	15.11	14.74	15.24	14.75	14.45	14.79	14.28	14.24
1.013	0.52	1.43	Q61510	E3 ubiquitin/ISG15 ligase TRIM25	Trim25	2307	23	23	23	42	42	42	71.726	0.0000	239.26	64923000	572	16.54	16.46	17.57	16.93	16.34	16.18	16.54	16.36
1.390	0.52	1.43	Q921F4	Heterogeneous nuclear ribonucleoprotein L-like	Hnrnpll	4221	31	31	31	64.6	64.6	64.6	64.124	0.0000	289.8	94525000	709	17.95	17.75	18.52	18.09	17.32	17.51	17.88	17.52
1.736	0.52	1.43	P47757	F-actin-capping protein subunit beta	Capzb	1121	30	30	30	81.6	81.6	81.6	31.345	0.0000	323.31	643890000	1148	20.15	19.66	20.20	20.35	19.70	19.46	19.66	19.47
1.380	0.52	1.43	D3Z6Q9	Bridging integrator 2	Bin2	214	26	26	24	56.9	56.9	55	52.553	0.0000	291.98	76745000	600	17.37	17.23	18.00	17.46	16.77	16.89	17.03	17.28
1.877	0.52	1.43	P47753	F-actin-capping protein subunit alpha-1	Capza1	1119	24	19	19	83.9	73.8	73.8	32.939	0.0000	323.31	240370000	641	19.54	19.30	19.89	19.71	19.05	19.33	18.98	19.01
0.870	0.52	1.43	Q99LI2	Chloride channel CLIC-like protein 1	Clcc1	4431	8	8	8	23.9	23.9	23.9	60.621	0.0000	41.397	3542300	41	13.58	12.56	13.35	12.53	12.17	12.60	12.39	12.79
0.548	0.52	1.43	Q9QZU9	Ubiquitin/ISG15-conjugating enzyme E2 L6	Ube2l6	5607	7	7	7	38.6	38.6	38.6	17.841	0.0000	11.207	3974900	24	13.42	12.74	14.20	14.06	13.67	13.31	12.32	13.04
0.409	0.52	1.43	Q8BXV2	BRI3-binding protein	Bri3bp	3290	4	4	4	20.6	20.6	20.6	28.263	0.0000	20.729	3999500	28	14.15	13.97	14.93	13.78	13.40	13.18	13.02	15.17
0.389	0.52	1.43	Q3THW5;P0	Histone H2A.V;Histone H2A.Z	H2afv;H2afz	705	9	7	7	64.8	57.8	57.8	13.509	0.0000	323.31	472960000	1246	20.18	19.87	22.00	21.00	20.50	19.54	19.89	21.05
0.243	0.52	1.43	P50637	Translocator protein	Tspo	1189	2	2	2	18.9	18.9	18.9	18.841	0.0000	170.46	1504100	29	13.89	12.61	16.15	13.11	12.83	13.57	14.40	12.89
0.817	0.52	1.43	Q925N2	Sideroflexin-2	Sfxn2	4310	7	7	7	29.2	29.2	29.2	36.141	0.0000	27.522	8868200	94	15.52	14.89	15.72	15.07	14.62	14.24	14.86	15.42
0.821	0.52	1.43	Q9CQE8	UPF0568 protein C14orf166 homolog		4591	12	12	12	54.1	54.1	54.1	28.152	0.0000	63.859	22395000	228	15.92	16.54	16.01	16.37	15.38	15.32	15.57	16.51
1.000	0.52	1.43	Q9DBR7	Protein phosphatase 1 regulatory subunit 12A	Ppp1r12a	5172	30	30	28	34.2	34.2	33.2	114.99	0.0000	136.05	20943000	159	16.88	16.61	17.77	16.93	16.66	16.41	16.70	16.35
2.405	0.52	1.43	P47226	Testin	Tes	1115	15	15	15	32.2	32.2	32.2	47.983	0.0000	197.61	48438000	376	17.17	16.94	17.39	17.13	16.67	16.75	16.69	16.45
1.002	0.52	1.43	Q9Z1K6	E3 ubiquitin-protein ligase ARIH2	Arih2	5811	10	10	10	30.7	30.7	30.7	57.696	0.0000	87.365	12501000	151	15.00	14.46	15.43	15.17	14.66	14.33	14.87	14.13
1.103	0.52	1.43	Q9EQH3	Vacuolar protein sorting-associated protein 35	Vps35	5278	50	50	50	68.3	68.3	68.3	91.712	0.0000	323.31	237250000	1034	19.05	18.93	19.98	19.22	18.84	18.88	18.76	18.64
0.494	0.52	1.43	Q9DBE0	Cysteine sulfinic acid decarboxylase	Csad	5145	11	11	11	42	42	42	55.144	0.0000	94.076	8738700	76	15.27	14.27	16.42	15.49	14.48	15.06	14.62	15.23
0.614	0.51	1.43	P35276	Ras-related protein Rab-3D	Rab3d	1012	6	5	4	33.8	30.1	21.5	24.416	0.0000	21.681	9082200	74	15.44	15.83	14.60	16.04	14.53	14.92	15.64	14.76
0.610	0.51	1.43	O88512	AP-1 complex subunit gamma-like 2	Ap1g2	537	9	9	9	15.4	15.4	15.4	87.862	0.0000	35.044	11144000	157	15.10	14.57	16.32	15.60	14.57	15.06	15.17	14.73
2.528	0.51	1.43	Q61425	Hydroxyacyl-coenzyme A dehydrogenase, mitochondrial	Hadh	2299	21	21	21	80.9	80.9	80.9	34.463	0.0000	323.31	113590000	539	18.01	17.90	18.21	18.11	17.76	17.46	17.58	17.37
1.412	0.51	1.43	Q9JHK5	Pleckstrin	Plek	5366	25	25	25	56.6	56.6	56.6	39.9	0.0000	305.44	96551000	489	17.83	17.74	18.47	18.02	17.45	17.77	17.53	17.24
0.665	0.51	1.43	Q7TMK6	Protein Hook homolog 2	Hook2	2745	5	5	5	12.7	12.7	12.7	83.365	0.0000	32.74	3260400	26	13.54	13.77	15.15	14.33	13.94	13.55	13.73	13.51
0.389	0.51	1.43	Q3UUG6	TBC1 domain family member 24	Tbc1d24	2031	4	4	4	9.8	9.8	9.8	63.235	0.0000	11.161	2967100	35	14.59	13.85	14.55	15.16	13.77	12.69	14.68	14.95
1.624	0.51	1.43	Q9EQH2	Endoplasmic reticulum aminopeptidase 1	Erap1	5277	27	27	27	31.4	31.4	31.4	106.6	0.0000	323.31	89830000	690	17.72	17.48	17.74	17.76	17.34	16.98	17.51	16.82
1.098	0.51	1.43	P56135	ATP synthase subunit f, mitochondrial	Atp5j2	1275	7	7	7	53.4	53.4	53.4	10.344	0.0000	20.357	105580000	203	19.09	18.78	18.86	19.13	18.63	17.84	18.92	18.40
0.625	0.51	1.43	Q8K221	Arfaptin-2	Arfip2	3642	4	4	4	15.8	15.8	15.8	37.772	0.0000	36.354	2576400	39	14.25	13.57	12.70	13.42	12.51	12.74	13.09	13.55
0.378	0.51	1.43	Q8C078	Calcium/calmodulin-dependent protein kinase kinase 2	Camkk2	3326	8	8	8	18.4	18.4	18.4	64.617	0.0000	26.601	6020700	63	13.66	14.00	15.33	15.38	13.78	13.15	14.86	14.53
0.995	0.51	1.42	Q3URE1	Acyl-CoA synthetase family member 3, mitochondrial	Acsf3	2022	12	12	12	30.7	30.7	30.7	65.076	0.0000	27.975	11130000	90	15.82	15.18	16.40	16.01	15.54	15.27	15.25	15.31
0.988	0.51	1.42	Q7TPR4	Alpha-actinin-1	Actn1	2771	72	46	45	77.7	58.6	57	103.07	0.0000	323.31	225670000	1167	18.41	18.09	19.25	18.21	17.90	18.11	17.99	17.92
2.345	0.51	1.42	Q80WC7	Arf-GAP domain and FG repeat-containing protein 2	Agfg2	2860	7	5	5	20	17.7	17.7	48.967	0.0000	34.835	10943000	109	15.43	15.16	15.67	15.60	14.89	14.93	15.02	14.98
1.199	0.51	1.42	Q3THS6	S-adenosylmethionine synthase isoform type-2	Mat2a	1847	19	19	19	43.3	43.3	43.3	43.688	0.0000	107.64	69173000	374	17.14	16.89	17.85	17.17	16.62	16.76	17.00	16.64
0.655	0.51	1.42	Q9CQZ5	NADH dehydrogenase [ubiquinone] 1 alpha subcomplex subunit 6	Ndufa6	4651	5	5	5	45.8	45.8	45.8	15.283	0.0000	37.458	8055700	84	15.19	15.38	16.63	15.79	15.36	15.16	14.75	15.68
0.476	0.51	1.42	Q80XP8	Protein FAM76B	Fam76b	2888	5	5	5	14.5	14.5	14.5	38.555	0.0000	8.8249	3569000	59	14.26	13.89	15.75	14.09	13.32	14.40	14.19	14.04
0.460	0.51	1.42	Q8K3A9	7SK snRNA methylphosphate capping enzyme	Mepce	3698	6	6	6	18.3	18.3	18.3	72.05	0.0000	40.228	4370900	55	14.67	14.44	15.58	14.74	14.29	13.15	14.89	15.06
1.421	0.51	1.42	Q9QXX4	Calcium-binding mitochondrial carrier protein Aralar2	Slc25a13	5554	40	34	34	72.6	66.7	66.7	74.466	0.0000	323.31	198520000	822	18.59	18.43	18.91	18.85	18.05	18.10	18.65	17.95
0.503	0.51	1.42	Q91X21	Uncharacterized protein KIAA2013	Kiaa2013	4133	5	5	5	12.6	12.6	12.6	69.424	0.0000	23.809	6143700	55	15.18	13.63	14.30	15.04	13.88	14.69	13.30	14.23
1.184	0.51	1.42	Q8BHS3	Pre-mRNA-splicing factor RBM22	Rbm22	3050	8	8	8	28.3	28.3	28.3	46.895	0.0000	24.332	8436200	82	15.13	15.34	16.02	15.11	14.85	15.09	14.74	14.88
0.749	0.51	1.42	Q3UKJ7	WD40 repeat-containing protein SMU1;WD40 repeat-containing protein	Smu1	1988	18	18	18	41.9	41.9	41.9	57.543	0.0000	131.58	40524000	332	16.55	16.35	15.92	16.68	16.14	15.65	16.50	15.18
1.419	0.51	1.42	Q3UMW7	MAP kinase-activated protein kinase 3	Mapkapk3	2004	17	16	16	64.8	64.8	64.8	43.293	0.0000	105	25825000	228	16.79	16.25	17.02	16.92	16.34	16.25	16.36	16.00
0.588	0.51	1.42	O08912	Polypeptide N-acetylgalactosaminyltransferase 1;Polypeptide N-acetylg	Galnt1	310	4	4	3	12.2	12.2	10.7	64.254	0.0000	16.108	1487000	12	14.26	13.63	15.13	14.17	14.36	13.50	14.08	13.22
0.727	0.51	1.42	Q3V3R1	Monofunctional C1-tetrahydrofolate synthase, mitochondrial	Mthfd1l	2054	36	35	35	47.2	46.2	46.2	105.73	0.0000	323.31	66153000	600	16.53	16.18	17.66	16.56	15.99	16.48	16.35	16.09
1.946	0.51	1.42	P37040	NADPH--cytochrome P450 reductase	Por	1046	55	55	55	73.3	73.3	73.3	77.043	0.0000	323.31	517180000	1608	19.19	18.98	18.93	18.86	18.82	18.48	18.36	18.27
4.511	0.51	1.42	Q922B2	Aspartate--tRNA ligase, cytoplasmic	Dars	4247	41	41	41	78.6	78.6	78.6	57.147	0.0000	323.31	189520000	1104	18.40	18.39	18.27	18.43	17.91	17.79	17.92	17.85
0.432	0.50	1.42	Q8C547	HEAT repeat-containing protein 5B	Heatr5b	3392	23	23	23	17.6	17.6	17.6	224.32	0.0000	81.041	11416000	159	14.44	14.04	15.65	15.04	14.53	13.90	15.24	13.48
0.505	0.50	1.42	P58854	Gamma-tubulin complex component 3	Tubgcp3	1325	9	9	9	14.4	14.4	14.4	103.47	0.0000	23.877	7261300	77	15.70	14.96	15.06	15.88	15.09	13.76	15.59	15.14
1.219	0.50	1.42	O54782	Epididymis-specific alpha-mannosidase	Man2b2	421	27	27	27	29.7	29.7	29.7	115.61	0.0000	280.68	68944000	533	17.43	17.05	16.68	17.48	16.89	16.74	16.64	16.36
0.503	0.50	1.42	Q8R313	Exocyst complex component 6	Exoc6	3829	9	8	8	15.2	14.1	14.1	93.076	0.0000	32.597	9201400	82	15.53	14.70	16.43	16.18	15.31	14.78	15.87	14.87
0.491	0.50	1.42	Q9D0M1	Phosphoribosyl pyrophosphate synthase-associated protein 1	Prpsap1	4878	16	14	14	58.1	54.2	54.2	39.431	0.0000	95.453	64687000	209	18.63	18.24	20.14	18.64	18.37	18.52	18.86	17.88
0.275	0.50	1.42	Q8C4X2	Casein kinase I isoform gamma-3	Csnk1g3	3389	7	7	4	17.9	17.9	11.1	48.937	0.0000	9.0168	3958700	20	13.89	11.60	14.56	14.19	13.38	12.01	13.33	13.51
1.089	0.50	1.42	Q78PY7	Staphylococcal nuclease domain-containing protein 1	Snd1	2728	70	70	70	78.9	78.9	78.9	102.09	0.0000	323.31	484700000	1686	19.71	19.56	20.64	19.97	19.40	19.42	19.55	19.50
2.062	0.50	1.42	Q9EQI8	39S ribosomal protein L46, mitochondrial	Mrpl46	5280	8	8	8	39.9	39.9	39.9	32.131	0.0000	70.24	19479000	224	15.63	15.69	15.99	16.03	15.45	15.31	15.47	15.10
0.479	0.50	1.42	Q923B1	Lariat debranching enzyme	Dbr1	4281	5	5	5	15.6	15.6	15.6	62.289	0.0000	57.271	4693800	27	13.87	14.36	15.61	14.91	13.50	14.24	14.94	14.06
0.539	0.50	1.42	Q3U319	E3 ubiquitin-protein ligase BRE1B	Rnf40	1916	16	16	16	22.9	22.9	22.9	113.97	0.0000	91.03	9028000	110	14.15	13.87	15.59	14.21	13.84	13.87	14.51	13.60
0.207	0.50	1.42	Q8C5L3	CCR4-NOT transcription complex subunit 2	Cnot2	3397	5	5	5	17.4	17.4	17.4	59.71	0.0000	19.177	8214700	60	15.54	16.03	15.02	15.14	15.43	16.28	15.85	12.17
0.667	0.50	1.42	Q9R0N0	Galactokinase	Galk1	5630	20	20	20	57.9	57.9	57.9	42.295	0.0000	216.76	53732000	393	17.24	16.78	18.43	17.24	16.77	17.03	17.10	16.78
1.152	0.50	1.42	Q9CPQ3	Mitochondrial import receptor subunit TOM22 homolog	Tomm22	4526	6	6	6	50	50	50	15.537	0.0000	104.83	75904000	201	18.58	18.08	18.51	18.84	17.87	17.78	18.50	17.85
1.854	0.50	1.41	P27612	Phospholipase A-2-activating protein	Plaa	937	35	35	35	50	50	50	87.22	0.0000	323.31	74411000	558	17.66	17.29	17.86	17.68	17.13	17.36	17.02	16.99
0.682	0.50	1.41	Q8VE62	Polyadenylate-binding protein-interacting protein 1	Paip1	3987	4	4	3	14	14	10.2	45.701	0.0000	18.054	3515800	32	13.73	14.18	14.15	13.94	12.65	13.84	14.21	13.30
1.248	0.50	1.41	P06151	L-lactate dehydrogenase A chain	Ldha	655	40	40	37	91	91	84.3	36.498	0.0000	323.31	1410900000	2137	20.92	21.00	21.78	21.10	20.72	20.56	20.91	20.61
0.366	0.50	1.41	Q8CG50	Ras-related protein Rab-43	Rab43	3498	7	6	6	43.8	37.6	37.6	23.263	0.0000	31.654	6989300	60	15.07	14.83	15.74	15.40	15.11	13.22	14.83	15.88
0.715	0.50	1.41	Q9D4H2	GRIP and coiled-coil domain-containing protein 1	Gcc1	4977	6	6	6	11.1	11.1	11.1	87.676	0.0000	14.75	3238800	15	13.80	13.73	14.34	13.88	13.10	12.82	13.58	14.24

0.387	0.50	1.41	Q80SY5	Pre-mRNA-splicing factor 38B	Prpf38b		2797	7	7	7	14.2	14.2	14.2	63.752	0.0000	91.736	10474000	82	16.33	15.29	17.24	16.93	15.92	14.94	16.64	16.29
0.515	0.50	1.41	Q3U829	AP-5 complex subunit zeta-1	Ap5z1		1935	10	10	10	19.8	19.8	19.8	89.406	0.0000	81.697	15428000	74	16.47	16.27	17.88	16.16	16.08	16.00	16.78	15.93
0.637	0.50	1.41	Q05512	Serine/threonine-protein kinase MARK2	Mark2		1740	15	15	11	25.8	25.8	20	86.305	0.0000	86.57	21568000	215	15.47	15.36	16.93	15.95	15.19	15.40	15.70	15.43
0.681	0.50	1.41	Q99LC2	Cleavage stimulation factor subunit 1	Cstf1		4415	10	10	10	34.6	34.6	34.6	48.381	0.0000	102.7	18786000	174	15.42	15.06	16.58	15.79	15.02	15.38	15.50	14.96
0.824	0.50	1.41	Q99J72	DNA dC->dU-editing enzyme APOBEC-3	Apobec3		4329	8	8	8	25.2	25.2	25.2	52.213	0.0000	52.384	6407600	88	15.06	14.58	14.55	15.15	14.53	13.82	14.96	14.04
1.079	0.50	1.41	Q8BXL7	ADP-ribosylation factor-related protein 1	Arfrp1		3288	4	4	3	31.8	31.8	29.4	22.659	0.0004	5.0351	3073200	37	15.04	15.16	15.92	15.17	14.84	14.87	15.12	14.48
0.448	0.50	1.41	Q9CQS4	Solute carrier family 25 member 46	Slc25a46		4628	5	5	5	23.7	23.7	23.7	46.224	0.0000	32.431	4435400	40	13.98	14.54	15.10	14.74	14.72	13.70	14.90	13.05
0.335	0.50	1.41	Q9JLJ5	Elongation of very long chain fatty acids protein 1	Elovl1		5484	4	4	3	10.8	10.8	10.8	32.677	0.0000	22.876	21419000	82	16.40	16.80	18.34	16.37	16.64	15.24	16.88	17.16
0.515	0.49	1.41	Q8BFZ3	Beta-actin-like protein 2	Actbl2		2954	21	5	5	46.8	18.1	18.1	42.004	0.0000	52.736	51789000	290	17.33	16.53	17.94	17.15	17.16	15.80	17.25	16.75
0.571	0.49	1.41	Q9DBE8	Alpha-1,3/1,6-mannosyltransferase ALG2	Alg2		5146	15	15	15	45.5	45.5	45.5	47.404	0.0000	77.366	19872000	169	15.62	15.16	15.81	14.83	15.32	14.33	15.56	14.23
0.500	0.49	1.41	Q9WTK5	Nuclear factor NF-kappa-B p100 subunit;Nuclear factor NF-kappa-B p52	Nfkb2		5676	22	22	22	35.8	35.8	35.8	96.831	0.0000	173.84	25286000	253	15.53	15.51	17.28	15.68	15.06	15.68	15.62	15.65
0.713	0.49	1.41	Q7TT50	Serine/threonine-protein kinase MRCK beta	Cdc42bpb		2793	39	38	38	27.4	27.4	27.4	194.75	0.0000	133.72	28611000	345	15.60	15.19	16.72	15.78	15.07	15.32	15.52	15.40
0.523	0.49	1.41	Q924L1	LETM1 domain-containing protein 1	Letmd1		4296	14	14	14	38.9	38.9	38.9	41.7	0.0000	57.028	17924000	140	14.81	14.21	15.37	15.43	14.51	14.70	15.10	13.54
1.758	0.49	1.41	P83940	Transcription elongation factor B polypeptide 1	Tceb1		1605	9	9	9	98.2	98.2	98.2	12.473	0.0000	104.49	37516000	230	17.51	17.45	17.88	17.89	17.18	16.94	17.41	17.23
1.716	0.49	1.41	Q9DB27	Malignant T-cell-amplified sequence 1	Mcts1		5121	12	12	12	71.3	71.3	71.3	20.555	0.0000	108.46	51914000	362	17.20	17.00	17.42	17.46	16.85	16.60	17.06	16.59
0.714	0.49	1.41	Q8C1B7	Septin-11		40787	3363	26	26	15	52.7	52.7	35.5	49.694	0.0000	323.31	93367000	491	17.54	17.52	18.90	17.95	17.37	17.75	17.35	17.46
1.168	0.49	1.41	Q571I9	Aldehyde dehydrogenase family 16 member A1	Aldh16a1		2092	19	19	18	31.9	31.9	30.7	84.755	0.0000	299.88	61750000	361	17.18	16.76	17.75	17.38	16.65	16.91	16.61	16.92
0.830	0.49	1.41	Q9D0W5	Peptidyl-prolyl cis-trans isomerase-like 1	Ppil1		4889	7	7	7	44.6	44.6	44.6	18.237	0.0000	39.31	31733000	203	16.80	16.71	17.85	16.82	16.20	16.52	16.75	16.73
0.492	0.49	1.41	Q9JL95	Proteoglycan 3	Prg3		5474	4	4	4	18.9	18.9	18.9	25.204	0.0000	6.9152	7877100	36	16.29	15.15	14.45	14.94	14.57	14.19	14.77	15.33
0.323	0.49	1.41	Q9Z2R6	Protein unc-119 homolog A	Unc119		5863	5	4	4	29.6	25.8	25.8	27.01	0.0000	22.925	3379000	29	14.30	14.18	12.87	14.72	13.96	12.26	14.64	13.23
0.430	0.49	1.41	Q9QXM0	Abhydrolase domain-containing protein 2	Abhd2		5546	4	4	4	14.8	14.8	14.8	48.377	0.0000	49.863	4484500	52	14.90	14.92	14.88	13.82	14.96	14.62	13.98	12.99
1.192	0.49	1.41	Q9QUM9	Proteasome subunit alpha type-6	Psma6		5519	23	23	23	69.5	69.5	69.5	27.372	0.0000	168.53	283110000	1083	19.07	18.60	19.51	19.20	18.54	18.73	18.81	18.32
0.733	0.49	1.41	Q6ZQ88	Lysine-specific histone demethylase 1A	Kdm1a		2692	14	14	14	26.4	26.4	26.4	92.849	0.0000	75.652	14421000	138	15.77	15.50	16.57	15.40	15.84	15.13	15.37	14.94
1.045	0.49	1.41	P59764	Dedicator of cytokinesis protein 4	Dock4		1344	17	17	16	12.1	12.1	11.4	226.55	0.0000	44.024	11875000	127	15.21	14.95	14.92	15.55	14.60	14.19	15.12	14.77
1.040	0.49	1.41	Q9EPU0	Regulator of nonsense transcripts 1	Upf1		5264	44	44	44	48.6	48.6	48.6	123.97	0.0000	323.31	108270000	835	17.24	16.92	17.99	17.08	16.73	16.89	16.95	16.68
0.902	0.49	1.41	Q9CQ62	2,4-dienoyl-CoA reductase, mitochondrial	Decr1		4566	13	13	13	47.2	47.2	47.2	36.213	0.0000	113.14	48916000	307	16.90	16.40	17.66	17.19	16.63	16.72	16.50	16.34
0.292	0.49	1.40	Q8VCC1	15-hydroxyprostaglandin dehydrogenase [NAD(+)]	Hpgd		3915	7	7	7	43.5	43.5	43.5	29.18	0.0000	39.868	5691100	47	14.08	14.66	16.19	14.23	13.94	13.76	15.82	13.68
1.157	0.49	1.40	Q6PGF7	Exocyst complex component 8	Exoc8		2635	23	23	23	33.1	33.1	33.1	81.034	0.0000	159.77	42112000	367	16.52	16.18	17.24	16.69	16.16	16.14	16.24	16.12
0.987	0.49	1.40	P48410	ATP-binding cassette sub-family D member 1	Abcd1		1143	22	22	22	32.5	32.5	32.5	81.858	0.0000	143.79	41061000	346	16.46	16.25	17.18	16.64	16.14	16.59	15.93	15.90
1.094	0.49	1.40	Q9WTX6	Cullin-1	Cul1		5692	31	31	31	41.8	41.8	41.8	89.69	0.0000	323.31	78550000	621	17.64	17.12	18.20	17.65	16.97	17.23	17.31	17.14
0.358	0.49	1.40	Q9R0Q6	Actin-related protein 2/3 complex subunit 1A	Arpc1a		5637	7	4	4	25.1	16.5	16.5	41.626	0.0000	20.053	2765700	40	14.03	13.17	15.26	13.57	14.35	12.59	13.31	13.83
1.427	0.49	1.40	Q9CZD5	Translation initiation factor IF-3, mitochondrial	Mtif3		4826	4	4	4	18.8	18.8	18.8	31.739	0.0000	16.264	2248600	43	13.58	13.54	14.05	13.47	12.96	13.46	13.31	12.95
0.899	0.49	1.40	Q91YI0	Argininosuccinate lyase	Asl		4158	32	32	32	64	64	64	51.739	0.0000	323.31	121490000	654	17.61	17.49	18.65	18.05	17.34	17.60	17.62	17.30
1.063	0.49	1.40	P30416	Peptidyl-prolyl cis-trans isomerase FKBP4;Peptidyl-prolyl cis-trans isom	Fkbp4		978	30	30	30	69.9	69.9	69.9	51.572	0.0000	267.15	68119000	507	16.94	16.70	17.74	17.18	16.50	16.89	16.68	16.54
0.633	0.49	1.40	Q99L48	60S ribosomal export protein NMD3	Nmd3		4410	6	6	6	16.3	16.3	16.3	57.611	0.0000	27.11	5895000	67	15.80	15.08	15.46	15.46	14.99	14.92	15.79	14.14
0.293	0.49	1.40	Q9JM90	Signal-transducing adaptor protein 1	Stap1		5500	8	8	8	38.7	38.7	38.7	34.627	0.0000	129.59	7745400	76	14.71	13.39	16.65	15.60	14.67	14.50	14.78	14.46
0.158	0.49	1.40	Q8VE95	UPF0598 protein C8orf82 homolog			3993	2	2	2	13.8	13.8	13.8	24.33	0.0000	13.696	4500900	13	12.21	15.89	15.41	15.51	15.93	13.14	15.38	12.62
0.431	0.49	1.40	Q99PG4	Regulator of G-protein signaling 18	Rgs18		4506	3	3	3	18.3	18.3	18.3	27.61	0.0000	11.738	3708300	69	13.90	13.95	14.96	14.34	14.18	12.49	14.37	14.17
0.565	0.49	1.40	O88738	Baculoviral IAP repeat-containing protein 6	Birc6		564	26	26	26	9.5	9.5	9.5	532.16	0.0000	151.08	31525000	271	16.82	16.40	17.77	17.12	16.48	16.66	17.20	15.83
0.877	0.49	1.40	Q8CG48	Structural maintenance of chromosomes protein 2	Smc2		3497	64	64	64	55.2	55.2	55.2	134.24	0.0000	294.97	76308000	671	16.18	15.94	17.06	16.47	15.54	16.16	16.10	15.89
0.245	0.49	1.40	Q8R4B8	NACHT, LRR and PYD domains-containing protein 3	Nlrp3		3867	6	6	6	6.7	6.7	6.7	118.27	0.0000	29.207	3292200	35	13.47	13.27	16.00	14.98	12.51	14.35	14.67	14.23
0.666	0.49	1.40	Q91X20	Set1/Ash2 histone methyltransferase complex subunit ASH2	Ash2l		4132	10	10	10	20.1	20.1	20.1	68.249	0.0000	24.399	11386000	71	16.69	16.56	17.73	16.69	16.41	17.01	15.92	16.39
0.584	0.49	1.40	Q922M3	BTB/POZ domain-containing adapter for CUL3-mediated RhoA degrada	Kctd10		4264	2	2	2	9.8	9.8	9.8	35.701	0.0000	22.504	2060600	39	13.79	13.25	14.94	14.30	13.13	13.63	13.84	13.73
1.458	0.49	1.40	P14483;P01	H-2 class II histocompatibility antigen, A beta chain;H-2 class II histocor	H2-Ab1		781	12	12	11	42.3	42.3	42.3	30.128	0.0000	270.72	113220000	310	18.80	18.52	19.00	18.62	18.64	18.02	18.28	18.06
1.266	0.48	1.40	Q9CQI6	Coactosin-like protein	Cotl1		4602	28	28	28	99.3	99.3	99.3	15.944	0.0000	323.31	607760000	1065	20.01	19.64	20.61	20.07	19.57	19.61	19.70	19.51
1.410	0.48	1.40	Q9Z1Z2	Serine-threonine kinase receptor-associated protein	Strap		5827	21	21	21	66.6	66.6	66.6	38.442	0.0000	323.31	119450000	574	17.87	17.64	18.39	17.97	17.34	17.54	17.74	17.32
1.034	0.48	1.40	Q61081	Hsp90 co-chaperone Cdc37;Hsp90 co-chaperone Cdc37, N-terminally p	Cdc37		2246	28	28	28	53.8	53.8	53.8	44.593	0.0000	323.31	139030000	751	18.15	17.75	18.82	18.25	17.79	17.77	17.99	17.49
1.140	0.48	1.40	Q8BIJ6	Isoleucine--tRNA ligase, mitochondrial	Iars2		3061	29	29	29	34.5	34.5	34.5	112.8	0.0000	239.49	70373000	627	16.90	16.58	17.43	17.15	16.71	16.36	16.78	16.27
0.313	0.48	1.40	Q60591	Nuclear factor of activated T-cells, cytoplasmic 2	Nfatc2		2180	9	9	9	18.2	18.2	18.2	100.02	0.0000	60.001	11253000	96	15.78	15.40	13.79	15.89	14.28	13.98	15.95	14.71
0.334	0.48	1.40	Q9CWG8	NADH dehydrogenase [ubiquinone] complex I, assembly factor 7	Ndufaf7		4714	8	8	8	29.6	29.6	29.6	48.384	0.0000	35.752	3133400	39	14.33	13.31	14.67	14.00	11.96	14.18	14.19	14.05
0.603	0.48	1.40	Q9D071	MMS19 nucleotide excision repair protein homolog	Mms19		4853	12	12	12	20.9	20.9	20.9	113.09	0.0000	150.33	23222000	214	16.08	15.86	16.45	16.26	15.93	14.66	15.82	16.32
0.371	0.48	1.40	Q8K4B2	Interleukin-1 receptor-associated kinase 3	Irak3		3713	5	5	5	8	8	8	68.455	0.0000	22.225	2150600	34	13.72	13.57	15.46	14.31	13.36	14.50	12.98	14.31
0.395	0.48	1.40	Q149L6	DnaJ homolog subfamily B member 14	Dnajb14		1786	8	8	8	23	23	23	42.325	0.0000	18.291	6509400	60	14.85	14.48	16.09	13.94	14.81	13.79	13.96	14.88
0.403	0.48	1.40	P04230	H-2 class II histocompatibility antigen, E-B beta chain	H2-Eb1		639	5	5	2	20.1	20.1	8	30.166	0.0000	13.12	6768500	31	14.40	13.52	15.43	14.95	14.40	13.10	14.49	14.39
1.337	0.48	1.40	Q9R0U0	Serine/arginine-rich splicing factor 10	Srsf10		5642	8	8	8	24.4	24.4	24.4	31.3	0.0000	39.163	26846000	134	17.28	17.40	17.36	17.78	17.09	17.22	16.52	17.06
0.161	0.48	1.40	Q9R1Z7	6-pyruvoyl tetrahydrobiopterin synthase	Pts		5666	6	6	6	41	41	41	16.188	0.0002	5.8078	9704200	29	15.48	14.78	14.35	16.06	14.53	11.70	15.83	16.69
1.150	0.48	1.40	P19783	Cytochrome c oxidase subunit 4 isoform 1, mitochondrial	Cox4i1		849	18	18	18	67.5	67.5	67.5	19.53	0.0000	181.22	382210000	658	20.00	19.71	20.66	20.02	19.47	19.46	19.82	19.71
0.340	0.48	1.39	Q3UHQ6	Protein dopey-2	Dopey2		1975	13	13	12	8.7	8.7	8.1	257.48	0.0000	62.909	8616800	57	15.09	14.51	16.17	15.75	14.32	15.51	15.90	13.87
1.372	0.48	1.39	Q9Z1F9	SUMO-activating enzyme subunit 2	Uba2		5806	29	29	29	59.9	59.9	59.9	70.568	0.0000	208.38	55257000	526	16.26	15.85	16.30	16.37	15.86	15.47	16.05	15.48
0.773	0.48	1.39	Q8C166	Copine-1	Cpne1		3355	20	19	19	47.2	45.5	45.5	58.886	0.0000	139.43	82054000	342	18.03	17.91	19.08	18.55	17.93	17.67	18.32	17.72
0.384	0.48	1.39	Q6NS46	Protein RRP5 homolog	Pdcd11		2523	14	14	13	12.8	12.8	12.4	207.78	0.0000	81.04	10727000	139	15.12	14.63	16.44	14.78	14.77	15.11	15.41	13.77
0.541	0.48	1.39	P49718	DNA replication licensing factor MCM5	Mcm5		1169	37	37	36	56.7	56.7	56.5	82.406	0.0000	275.41	82540000	561	16.47	16.30	17.99	16.51	16.05	16.47	16.57	16.26
1.285	0.48	1.39	Q9CXG3	Peptidyl-prolyl cis-trans isomerase-like 4	Ppil4		4755	10	10	9	29.7	29.7	25.2	57.23	0.0000	24.561	9837000	98	15.26	15.24	15.81	15.23	15.31	14.77	14.76	14.78
1.514	0.48	1.39	Q91YS8	Calcium/calmodulin-dependent protein kinase type 1	Camk1		4177	11	11	9	44.4	44.4	39	41.624	0.0000	57.48	7852900	106	15.25	15.81	15.56	15.70	14.75	15.24	15.23	15.21
0.340	0.48	1.39	Q6PCN7	Helicase-like transcription factor	Hltf		2605	9	9	9	13.3	13.3	13.3	113.32	0.0000	28.773	3643500	29	13.75	12.85	15.10	13.68	13.08	12.93	12.96	14.50
0.711	0.48	1.39	Q9CPQ1	Cytochrome c oxidase subunit 6C	Cox6c		4525	10	10	10	64.5	64.5	64.5	8.4689	0.0000	89.107	250040000	468	19.68	19.32	20.73	20.02	19.38	19.15	19.75	19.56
0.571	0.48	1.39	Q8VD65	Phosphoinositide 3-kinase regulatory subunit 4	Pik3r4		3942	26	26	26	27.5	27.5	27.5	152.6	0.0000	164.35	21307000	209	15.47	15.22	16.90	15.61	15.30	15.22	15.64	15.13
0.732	0.48	1.39	Q924C1	Exportin-5	Xpo5		4291	13	13	13	17.1	17.1	17.1	136.97	0.0000	57.282	6515000	99	14.63	14.18	15.39	14.72	14.19	14.32	14.73	13.77
1.184	0.48	1.39	Q80W54	CAAX prenyl protease 1 homolog	Zmpste24		2859	11	11	11	27.4	27.4	27.4	54.734	0.0000	158.58	50531000	288	17.29	16.82	16.44	17.14	16.67	16.54	16.28	16.29
1.027	0.48	1.39	P21279	Guanine nucleotide-binding protein G(q) subunit alpha	Gnaq		861	20	19	15	58.5	56.3	40.1	42.158	0.0000	239.18	64576000	479	16.73	16.46	17.31	17.13	16.60	16.63	16.02	16.48
0.967	0.47	1.39	P31938	Dual specificity mitogen-activated protein kinase kinase 1	Map2k1		991	25	25	17	62.8	62.8	48.6	43.474	0.0000	211.21	78235000	454	17.44	17.16	17.99	17.78	17.20	16.63	17.43	17.20
1.266	0.47	1.39	P63085	Mitogen-activated protein kinase 1	Mapk1		1488	24	18	18	70.1	60.3	60.3	41.275	0.0000	221.28	132410000	534	18.76	18.56	19.12	18.70	18.51	18.59	17.88	18.27
0.300	0.47	1.39	Q80VP1	Epsin-1	Epn1		2854	7	7	7	16.5	16.5	16.5	60.211	0.0000	24.024	7229600	82	14.59	14.30	16.10	15.52	14.46	15.77	13.33	15.05
0.499	0.47	1.39	Q2TBE6	Phosphatidylinositol 4-kinase type 2-alpha	Pi4k2a		1801	17	17	17	46.3	46.3	46.3	54.257	0.0000	136.13	32753000	265	16.28	15.86	17.77	16.77	16.38	16.47	16.05	15.89
1.695	0.47	1.39	P08113	Endoplasmin	Hsp90b1		680	73	71	71	66.3	64.6	64.6	92.475	0.0000	323.31	1570500000	3003	20.20	19.81	20.43	20.32	19.58	19.78	19.88	19.62
1.329	0.47	1.39	Q9QWR8	Alpha-N-acetylgalactosaminidase	Naga		5527	17	17	17	44.8	44.8	44.8	47.234	0.0000	70.782	80968000	289	18.48	18.57	19.18	18.86	18.50	18.36	18.00	18.33
1.276	0.47	1.39	Q9CWY8	Ribonuclease H2 subunit A	Rnaseh2a		4739	6	6	6	32.9	32.9	32.9	33.512	0.0000	20.558	8002800	93	15.39	15.42	15.26	15.37	14.81	15.12	15.25	14.38
0.786	0.47	1.39	Q9DCT2	NADH dehydrogenase [ubiquinone] iron-sulfur protein 3, mitochondrial	Ndufs3		5230	12	12	12	38.8	38.8	38.8	30.149	0.0000	40.965	30188000	147	16.90	17.09	17.78	17.69	16.78	17.16	17.26	16.37
0.849	0.47	1.39	Q8VD04	GRIP1-associated protein 1	Gripap1		3938	17	17	17	28.8	28.8	28.8	92.714	0.0000	132.87	7342000	91	14.79	14.27	14.60	14.90	14.39	14.25	14.56	13.47
0.729	0.47	1.39	P63154	Crooked neck-like protein 1	Crnkl1		1492	16	16	16	31.2	31.2	31.2	83.415	0.0000	91.859	18545000	221	15.85	15.46	16.81	16.02	15.16	15.65	15.71	15.74
0.382	0.47	1.39	Q8BXZ1	Protein disulfide-isomerase TMX3	Tmx3		3291	11	11	11	23.5	23.5	23.5	51.847	0.0000	42.849	46545000	153	17.11	16.62	15.97	17.62	16.60	15.27	17.23	16.33
0.330	0.47	1.39	Q80TI0	GRAM domain-containing protein 1B	Gramd1b		2805	2	2	2	4.7	4.7	4.7	85.354	0.0004	4.3144	1436800	3	13.80	13.68	14.19	14.11	13.29	13.63	12.04	14.94
0.318	0.47	1.39	O88379	Bromodomain adjacent to zinc finger domain protein 1A	Baz1a		525	6	6	6	6.1	6.1	6.1	178.46	0.0000	18.022	3455600	43	13.58	14.76	16.20	14.87	14.97	13.54	14.82	14.19
1.013	0.47	1.39	P62334	26S protease regulatory subunit 10B	Psmc6		1427	37	37	36	83.8	83.8	83.8	44.172	0.0000	323.31	242910000	1172	18.14	17.79	18.88	18.17	17.73	17.85	17.92	17.60
0.426	0.47	1.39	Q5SSH7	Zinc finger ZZ-type and EF-hand domain-containing protein 1	Zzef1		2136	21	21	21	10.8	10.8	10.8	328.31	0.0000	65.385	19442000	183	15.77	14.84	17.04	15.88	15.10	15.07	15.78	15.70
0.796	0.47	1.39	Q9QXB9	Developmentally-regulated GTP-binding protein 2	Drg2		5535	22	22	22	54.1	54.1	54.1	40.718	0.0000	180.68	51589000	383	16.77	16.54	17.54	17.21	16.58	16.87	16.73	16.01
1.164	0.47	1.39	Q8C0J6	Ankyrin repeat domain-containing protein SOWAHC	Sowahc		3339	8	8	8	21.9	21.9	21.9	54.937	0.0000	39.159	10195000	160	14.61	14.49	15.15	14.81	14.29	13.86	14.45	14.57
1.380	0.47	1.38	Q9QZE5	Coatomer subunit gamma-1	Copg1		5595	38	38	35	61.3	61.3	57.2	97.512	0.0000	323.31	198690000	1116	18.53	18.11	18.98	18.65	18.09	18.02	18.17	18.11
0.757	0.47	1.38	Q8JZN5	Acyl-CoA dehydrogenase family member 9, mitochondrial	Acad9		3575	18	18	18	41.8	41.8	41.8	68.721	0.0000	224.74	34406000	352	16.39	16.18	17.01	16.74	16.32	15.38	16.43	16.31
0.838	0.47	1.38	Q3TIU4	2,5-phosphodiesterase 12	Pde12		1850	11	11	11	28.1	28.1	28.1	67.513	0.0000	53.863	14863000	149	15.84	15.74	16.43	15.96	15.05	16.03	15.21	15.81
0.968	0.47	1.38	Q9CZ28	Vacuolar-sorting protein SNF8	Snf8		4815	10	10	10	51.2	51.2	51.2	28.886	0.0000	81.385	20674000	111	16.38	16.64	17.00	16.33	15.95	15.76	16.67	16.10
1.137	0.47	1.38	Q9QXK3	Coatomer subunit gamma-2	Copg2		5543	34	31	31	48.7	46.5	46.5	97.679	0.0000	323.31	84500000	522	17.81	17.54	18.45	17.81	17.45	17.68	17.41	17.21
0.958	0.47	1.38	Q9R0C8	Guanine nucleotide exchange factor VAV3	Vav3		5622	11	10	10	16.6	15.5	15.5	97.967	0.0000	61.044	6007200	59	15.65	15.18	15.96	15.16	14.70	15.21	15.36	14.81
1.404	0.47	1.38	Q8BZ20	Poly [ADP-ribose] polymerase 12	Parp12		3310	13	13	13	28.7	28.7	28.7	79.916	0.0000	61.12	11658000	146	15.08	14.70	15.33	15.27	14.65	14.61	14.89	14.36
0.222	0.47	1.38	Q3V4B5	COMM domain-containing protein 6	Commd6		2055	6	6	6	70.1	70.1	70.1	9.7951	0.0000	11.749	8567300	41	14.97	15.51	15.26	15.32	13.53	16.46	16.00	13.20
0.271	0.47	1.38	Q80TP3	E3 ubiquitin-protein ligase UBR5	Ubr5		2811	12	12	12	8.5	8.5	8.5	308.35	0.0000	80.171	6326300	85	14.62	14.00	15.40	14.03	13.81	15.00	15.01	12.35
3.103	0.47	1.38	Q8BT60	Copine-3	Cpne3		3198	23	23	21	44.8	44.8	43.2	59.584	0.0000	178.5	104210000	578	17.67	17.83	17.57	17.77	17.24	17.10	17.31	17.32
1.405	0.47	1.38	Q8BMP6	Golgi resident protein GCP60	Acbd3		3145	11	11	11	30.7	30.7	30.7	60.18	0.0000	186.49	29304000	213	16.53	16.41	16.93	16.67	16.08	16.15	16.55	15.89
2.194	0.47	1.38	Q91ZJ5	UTP--glucose-1-phosphate uridylyltransferase	Ugp2		4196	42	42	42	67.1	67.1	67.1	56.979	0.0000	323.31	209780000	1166	18.22	18.16	18.31	17.91	17.58	17.85	17.77	17.53
0.647	0.47	1.38	Q8R0F6	Integrin-linked kinase-associated serine/threonine phosphatase 2C	Ilkap		3756	9	9	9	32.9	32.9	32.9	42.774	0.0000	19.189	6223000	63	14.19	14.40	15.22	15.16	14.69	13.68	14.21	14.53
1.019	0.46	1.38	P12367	cAMP-dependent protein kinase type II-alpha regulatory subunit	Prkar2a		756	15	15	12	45.4	45.4	35.2	45.389	0.0000	175.76	28790000	361	15.84	15.71	16.21	16.39	15.54	15.09	15.83	15.83
1.037	0.46	1.38	Q8CAQ8	MICOS complex subunit Mic60	Immt		3430	58	58	58	69.6	69.6	69.6	83.899	0.0000	323.31	268290000	1595	18.16	17.90	18.87	18.16	17.70	18.07	17.86	17.60
0.392	0.46	1.38	Q8C5P5	5-nucleotidase domain-containing protein 1	Nt5dc1		3400	9	9	9	26.8	26.8	26.8	53.094	0.0000	34.761	3280300	39	14.57	13.68	15.18	14.04	13.60	13.37	15.11	13.55
1.132	0.46	1.38	P54822	Adenylosuccinate lyase	Adsl		1260	29	29	28	67.4	67.4	66.1	54.866	0.0000	323.31	104140000	585	17.39	17.22	17.48	17.54	17.17	16.35	17.01	17.23
0.534	0.46	1.38	Q91VE0	Long-chain fatty acid transport protein 4	Slc27a4		4058	18	18	17	37	37	35.8	72.318	0.0000	162.76	26924000	260	15.38	15.38	16.92	15.76	15.21	15.71	15.65	15.01
0.558	0.46	1.38	Q8BFQ4	WD repeat-containing protein 82	Wdr82		2942	11	11	11	26.5	26.5	26.5	35.079	0.0000	146.93	29508000	247	17.17	17.06	18.15	17.17	16.92	17.54	17.09	16.15
1.379	0.46	1.38	Q8BKC5	Importin-5	Ipo5		3098	49	49	49	60.2	60.2	60.2	123.59	0.0000	323.31	275480000	1470	18.92	18.76	19.37	19.20	18.71	18.36	18.87	18.46
0.375	0.46	1.38	P52019	Squalene monooxygenase	Sqle		1212	3	3	3	8.9	8.9	8.9	63.769	0.0000	25.084	2157800	27	13.79	12.54	14.21	13.80	13.59	13.72	13.20	11.98

0.507	0.46	1.38	Q62383	Transcription elongation factor SPT6	Supt6h	2383	27	27	27	22.2	22.2	22.2	199.08	0.0000	42.089	255010000	110	21.58	20.91	22.32	21.65	20.27	21.27	21.53	21.55
0.510	0.46	1.38	Q91YR5	Methyltransferase-like protein 13	Mettl13	4174	7	7	7	15.8	15.8	15.8	78.756	0.0000	54.309	3767700	61	14.57	14.68	14.23	14.96	13.84	14.14	15.22	13.41
0.522	0.46	1.38	Q91ZN5	Adenosine 3-phospho 5-phosphosulfate transporter 1	Slc35b2	4197	6	6	6	16.9	16.9	16.9	47.369	0.0000	21.026	5004000	36	14.80	14.52	14.34	14.06	14.33	14.35	12.84	14.35
0.291	0.46	1.38	Q9JJI8	60S ribosomal protein L38	Rpl38	5429	6	6	6	57.1	57.1	57.1	8.2038	0.0000	31.079	127650000	305	19.03	18.65	16.76	19.16	18.72	17.10	18.26	17.68
0.364	0.46	1.38	Q91ZT5	FYVE, RhoGEF and PH domain-containing protein 4	Fgd4	4200	13	13	13	24.2	24.2	24.2	86.54	0.0000	69.503	6606400	93	14.89	14.09	16.52	15.37	14.42	15.10	15.09	14.41
0.772	0.46	1.37	Q9EQ06	Estradiol 17-beta-dehydrogenase 11	Hsd17b11	5268	14	14	14	55	55	55	32.88	0.0000	239.59	72657000	372	17.34	17.88	18.37	18.07	18.00	17.19	17.13	17.51
1.271	0.46	1.37	P56546	C-terminal-binding protein 2	Ctbp2	1290	15	9	8	29.9	22.5	20.9	48.956	0.0000	60.155	18091000	123	16.37	16.04	16.76	16.27	15.72	16.11	16.09	15.67
0.609	0.46	1.37	Q8R164	Valacyclovir hydrolase	Bphl	3779	15	15	15	60.8	60.8	60.8	32.851	0.0000	70.509	23840000	203	15.70	15.49	16.95	15.89	15.88	15.65	15.50	15.16
0.669	0.46	1.37	Q9Z1K5	E3 ubiquitin-protein ligase ARIH1	Arih1	5810	17	17	17	36.8	36.8	36.8	64.016	0.0000	61.274	26100000	202	16.13	15.83	16.17	16.52	15.49	16.50	15.74	15.08
0.534	0.46	1.37	Q9JHI7	Exosome complex component RRP45	Exosc9	5362	3	3	3	7.8	7.8	7.8	48.936	0.0002	5.6968	2907700	20	13.88	14.44	14.00	14.10	14.18	13.17	14.40	12.85
0.229	0.46	1.37	Q8VCA8	Secernin-2	Scrn2	3912	3	3	3	14.6	14.6	14.6	46.6	0.0000	9.6669	954290	11	11.91	12.70	14.88	13.11	11.19	12.99	13.19	13.38
0.188	0.46	1.37	P56380	Bis(5-nucleosyl)-tetraphosphatase [asymmetrical]	Nudt2	1282	3	3	3	25.9	25.9	25.9	16.989	0.0008	4.232	5329400	34	15.53	14.92	12.07	15.73	14.82	13.05	14.83	13.72
0.783	0.46	1.37	Q99LE6	ATP-binding cassette sub-family F member 2	Abcf2	4424	23	23	23	35.7	35.7	35.7	71.781	0.0000	64.074	49674000	405	16.15	15.94	17.20	16.27	15.82	15.90	16.17	15.83
1.108	0.46	1.37	Q9R1C7	Pre-mRNA-processing factor 40 homolog A	Prpf40a	5650	24	24	24	30.7	30.7	30.7	108.48	0.0000	149.37	33266000	378	15.86	15.70	16.58	16.03	15.38	15.85	15.52	15.59
1.554	0.46	1.37	Q8BMS9	Ras association domain-containing protein 2	Rassf2	3150	16	16	14	56.7	56.7	48.8	37.973	0.0000	62.017	35078000	234	16.89	16.54	17.07	17.09	16.31	16.38	16.72	16.34
0.683	0.46	1.37	Q9D4F8	Gamma-tubulin complex component 4	Tubgcp4	4975	5	5	5	12.3	12.3	12.3	76.125	0.0000	10.517	2802200	21	13.84	13.65	14.57	13.83	13.41	13.78	14.02	12.86
0.709	0.46	1.37	Q8VDI9	Alpha-1,2-mannosyltransferase ALG9	Alg9	3955	5	5	5	11.8	11.8	11.8	69.56	0.0004	4.7415	2048100	18	13.11	13.64	13.78	13.30	12.37	12.74	13.58	13.32
0.560	0.46	1.37	Q8R2U0	Nucleoporin SEH1	Seh1l	3821	11	11	11	44.4	44.4	44.4	39.774	0.0000	69.365	13673000	129	15.38	15.35	16.91	15.83	15.23	15.27	15.73	15.42
0.832	0.46	1.37	Q3UCV8	Ubiquitin thioesterase otulin	Otulin	1945	9	9	9	34.7	34.7	34.7	40.32	0.0000	24.28	21603000	184	15.76	15.57	16.74	16.28	15.59	15.75	15.75	15.43
0.515	0.46	1.37	Q8VI36	Paxillin	Pxn	4027	18	18	18	50.1	50.1	50.1	64.476	0.0000	323.31	46747000	412	16.86	16.61	18.32	17.25	16.43	16.67	17.09	17.02
0.257	0.46	1.37	Q3TX08	tRNA (guanine(26)-N(2))-dimethyltransferase	Trmt1	1885	4	4	4	11.3	11.3	11.3	72.349	0.0000	30.064	4750100	47	15.49	14.03	14.72	14.94	14.53	15.05	15.35	12.42
1.000	0.46	1.37	Q922V4	Pleiotropic regulator 1	Plrg1	4279	18	18	18	48.7	48.7	48.7	56.937	0.0000	229.29	40336000	306	16.82	16.63	17.44	17.26	16.21	16.79	16.82	16.50
1.508	0.46	1.37	O35955	Proteasome subunit beta type-10	Psmb10	414	11	11	11	50.9	50.9	50.9	29.063	0.0000	196.08	104050000	364	18.04	18.18	18.57	17.92	17.56	17.63	17.93	17.77
1.229	0.46	1.37	Q8BYI6	Lysophosphatidylcholine acyltransferase 2	Lpcat2	3300	15	15	15	36.9	36.9	36.9	60.253	0.0000	100.88	45007000	265	16.94	16.64	17.35	16.90	16.32	16.24	16.77	16.68
1.441	0.46	1.37	Q01965	T-lymphocyte surface antigen Ly-9	Ly9	1713	18	18	18	31.2	31.2	31.2	73.142	0.0000	323.31	61907000	394	17.53	17.17	17.83	17.72	17.06	17.01	17.36	16.99
0.296	0.46	1.37	Q9CRC9	Glucosamine-6-phosphate isomerase 2	Gnpda2	4691	7	5	5	31.2	22.8	22.8	31.083	0.0000	13.501	3580000	39	14.63	14.48	13.55	14.01	12.07	13.68	14.86	14.24
0.183	0.46	1.37	P49070	Calcium signal-modulating cyclophilin ligand	Camlg	1154	4	4	4	23.8	23.8	23.8	32.542	0.0000	80.231	7273400	48	16.19	15.25	12.78	15.45	15.15	13.57	15.88	13.26
0.741	0.45	1.37	Q8C7X2	ER membrane protein complex subunit 1	Emc1	3418	27	27	27	36.7	36.7	36.7	111.6	0.0000	191.45	45701000	411	16.41	15.91	17.14	16.30	15.88	16.15	15.59	16.30
1.013	0.45	1.37	Q64669	NAD(P)H dehydrogenase [quinone] 1	Nqo1	2456	13	13	13	46.7	46.7	46.7	30.959	0.0000	36.503	28066000	218	17.03	16.82	16.22	16.89	16.27	16.66	15.94	16.26
0.716	0.45	1.37	Q9Z1T6	1-phosphatidylinositol 3-phosphate 5-kinase	Pikfyve	5823	22	22	22	15	15	15	236.87	0.0000	82.114	19894000	262	15.21	14.42	15.70	15.24	14.32	14.74	15.09	14.60
1.085	0.45	1.37	Q8JZQ2	AFG3-like protein 2	Afg3l2	3578	24	24	22	30.3	30.3	29.3	89.518	0.0000	94.562	55987000	328	16.54	16.13	17.09	16.35	15.99	16.22	16.17	15.91
0.661	0.45	1.37	P13597	Intercellular adhesion molecule 1	Icam1	767	8	8	8	12.8	12.8	12.8	58.844	0.0000	226.49	26084000	139	16.85	16.84	17.75	16.50	16.62	16.71	16.83	15.97
0.678	0.45	1.37	Q9CWU9	Nucleoporin Nup37	Nup37	4733	5	5	5	23	23	23	36.731	0.0000	17.641	3937000	35	15.14	14.38	13.96	14.79	14.00	14.18	14.61	13.67
0.444	0.45	1.37	P42669	Transcriptional activator protein Pur-alpha	Pura	1081	13	11	11	47.7	41.7	41.7	34.883	0.0000	228.92	30980000	193	17.01	16.70	18.71	17.24	16.78	17.22	16.97	16.88
0.660	0.45	1.37	Q791V5	Mitochondrial carrier homolog 2	Mtch2	2734	14	14	14	50.8	50.8	50.8	33.499	0.0000	203.1	157010000	471	19.73	19.34	20.71	19.82	19.48	19.58	19.72	19.00
0.377	0.45	1.37	Q61165	Sodium/hydrogen exchanger 1	Slc9a1	2265	8	8	8	15.7	15.7	15.7	91.467	0.0000	95.407	6639600	100	14.51	14.47	16.37	14.96	14.77	14.57	15.24	13.91
0.838	0.45	1.37	Q8CHT0	Delta-1-pyrroline-5-carboxylate dehydrogenase, mitochondrial	Aldh4a1	3531	13	13	13	26	26	26	61.84	0.0000	134.98	38914000	317	16.41	16.09	17.29	16.27	15.96	16.08	16.14	16.08
0.711	0.45	1.37	P53395	Lipoamide acyltransferase component of branched-chain alpha-keto aci	Dbt	1231	16	16	16	33.4	33.4	33.4	53.246	0.0000	64.447	24800000	223	15.73	15.14	16.57	15.84	15.55	15.38	15.44	15.09
2.508	0.45	1.37	Q9D6K5	Synaptojanin-2-binding protein	Synj2bp	4998	4	4	4	37.2	37.2	37.2	15.815	0.0000	36.644	7815900	106	14.91	14.80	14.93	15.00	14.53	14.21	14.61	14.48
0.543	0.45	1.37	P61957	Small ubiquitin-related modifier 2	Sumo2	1392	4	4	3	33.7	33.7	21.1	10.871	0.0000	14.2	44197000	92	18.53	18.30	19.94	18.56	18.19	18.53	18.54	18.26
0.822	0.45	1.37	Q6P9R1	ATP-dependent RNA helicase DDX51	Ddx51	2591	5	5	5	12.8	12.8	12.8	70.367	0.0000	19.432	2904900	47	13.87	13.26	14.41	13.83	13.43	13.48	13.65	12.99
0.869	0.45	1.37	P62869	Transcription elongation factor B polypeptide 2	Tceb2	1458	11	11	11	68.6	68.6	68.6	13.17	0.0000	67.803	35513000	192	17.29	16.93	17.79	17.85	16.96	16.99	17.40	16.70
1.719	0.45	1.37	Q921J2	GTP-binding protein Rheb	Rheb	4229	7	7	7	38.6	38.6	38.6	20.451	0.0000	60.555	25755000	153	16.99	17.24	17.48	17.20	16.66	17.08	16.70	16.68
0.571	0.45	1.37	Q80XN0	D-beta-hydroxybutyrate dehydrogenase, mitochondrial	Bdh1	2887	11	11	11	40.5	40.5	40.5	38.299	0.0000	61.064	16512000	101	15.31	15.30	16.34	16.52	14.92	15.47	15.66	15.63
1.269	0.45	1.36	P24270	Catalase	Cat	895	49	49	37	80.3	80.3	64.1	59.795	0.0000	323.31	878160000	2253	20.33	20.42	19.81	20.29	19.93	20.03	19.56	19.53
1.441	0.45	1.36	P54729	NEDD8 ultimate buster 1	Nub1	1254	27	27	27	47.7	47.7	47.7	70.306	0.0000	270.59	58885000	389	17.30	16.85	17.37	17.34	16.49	16.88	17.01	16.68
0.693	0.45	1.36	Q8CFE2	UPF0609 protein C4orf27 homolog		3481	10	10	10	35	35	35	39.291	0.0000	65.666	11221000	174	15.02	14.46	15.66	15.20	14.30	15.07	14.85	14.32
1.170	0.45	1.36	P27870	Proto-oncogene vav	Vav1	942	60	60	59	65.2	65.2	64	98.136	0.0000	323.31	154000000	975	17.73	17.52	18.37	18.04	17.42	17.56	17.27	17.62
0.903	0.45	1.36	Q80UP5	Ankyrin repeat domain-containing protein 13A	Ankrd13a	2835	9	9	9	19.2	19.2	19.2	67.177	0.0000	29.678	8624200	143	15.08	14.46	15.29	14.90	14.48	14.48	14.92	14.06
0.840	0.45	1.36	Q91YI4	Beta-arrestin-2	Arrb2	4159	19	19	18	63.7	63.7	61.7	46.313	0.0000	226.28	40578000	276	17.07	16.77	17.80	17.43	16.55	16.64	17.19	16.91
1.075	0.44	1.36	Q61699	Heat shock protein 105 kDa	Hsph1	2325	45	41	41	51.2	47.3	47.3	96.406	0.0000	323.31	63609000	606	16.33	15.98	16.96	16.22	15.85	16.08	15.91	15.87
0.253	0.44	1.36	Q8BJ64	Choline dehydrogenase, mitochondrial	Chdh	3074	2	2	2	6.2	6.2	6.2	66.414	0.0000	29.567	1848000	25	15.57	13.64	14.53	13.01	13.16	13.01	13.81	15.00
0.379	0.44	1.36	Q64735	Complement component receptor 1-like protein	Cr1l	2463	5	5	5	12.2	12.2	12.2	53.762	0.0000	87.021	7620900	65	15.59	15.52	15.41	15.10	15.39	13.48	15.64	15.34
0.361	0.44	1.36	Q3TLI0	Trafficking protein particle complex subunit 10	Trappc10	1860	11	11	11	13.9	13.9	13.9	141.49	0.0000	43.953	5511300	62	14.41	13.56	13.40	14.75	13.61	12.44	13.89	14.40
0.591	0.44	1.36	O35598	Disintegrin and metalloproteinase domain-containing protein 10	Adam10	383	9	9	9	14.6	14.6	14.6	83.967	0.0000	17.092	11702000	103	15.29	14.99	16.54	15.47	15.22	15.04	15.36	14.90
0.400	0.44	1.36	Q8CAS9	Poly [ADP-ribose] polymerase 9	Parp9	3431	25	25	25	43.9	43.9	43.9	96.658	0.0000	235.63	86341000	280	17.75	17.24	19.19	18.31	17.15	17.40	18.27	17.89
0.564	0.44	1.36	O88848	ADP-ribosylation factor-like protein 6	Arl6	576	1	1	1	9.7	9.7	9.7	20.959	0.0000	13.29	1305300	13	13.66	13.46	14.02	13.91	12.71	12.78	13.67	14.12
0.877	0.44	1.36	Q8VBZ3	Cleft lip and palate transmembrane protein 1 homolog	Clptm1	3902	14	14	14	27.7	27.7	27.7	75.29	0.0000	198.52	51895000	277	17.14	17.33	16.30	16.97	16.39	16.39	16.85	16.36
0.929	0.44	1.36	Q8BFQ8	Parkinson disease 7 domain-containing protein 1	Pddc1	2943	5	5	5	37.3	37.3	37.3	23.277	0.0000	168.96	14655000	114	15.80	15.55	15.96	16.21	15.86	15.33	14.94	15.63
1.321	0.44	1.36	Q9Z0S1	3(2),5-bisphosphate nucleotidase 1	Bpnt1	5784	19	19	19	64.6	64.6	64.6	33.196	0.0000	177.76	76999000	597	17.27	17.10	17.81	17.53	17.02	16.78	17.19	16.95
0.534	0.44	1.36	Q8K215	LYR motif-containing protein 4	Lyrm4	3641	2	2	2	23.1	23.1	23.1	10.854	0.0009	3.689	4134200	26	14.84	14.76	14.33	14.86	14.50	13.18	14.70	14.65
0.360	0.44	1.36	Q8K4Q0	Regulatory-associated protein of mTOR	Rptor	3725	6	6	6	7.7	7.7	7.7	149.47	0.0000	110.44	5964500	83	14.92	14.13	16.32	14.43	14.80	13.97	14.37	14.89
0.712	0.44	1.36	Q9CXD6	Mitochondrial calcium uniporter regulator 1	Mcur1	4752	8	8	8	24.7	24.7	24.7	37.849	0.0000	16.142	8199000	60	15.04	14.60	14.77	14.12	14.37	14.05	14.72	13.64
0.325	0.44	1.36	Q9DCM2	Glutathione S-transferase kappa 1	Gstk1	5223	8	8	8	37.2	37.2	37.2	25.704	0.0000	95.692	9778300	105	14.92	14.08	16.63	15.39	14.46	15.42	14.82	14.56
0.317	0.44	1.36	Q60766	Immunity-related GTPase family M protein 1	Irgm1	2204	22	22	22	56.5	56.5	56.5	46.551	0.0000	323.31	63359000	402	16.36	16.06	18.38	17.21	16.63	15.83	17.15	16.63
0.124	0.44	1.36	P46735	Unconventional myosin-Ib	Myo1b	1111	4	4	4	6.9	6.9	6.9	128.56	0.0000	22.553	8479300	14	16.60	12.19	16.46	16.25	16.52	13.78	16.05	13.38
0.405	0.44	1.36	Q91VT4	Carbonyl reductase family member 4	Cbr4	4077	8	8	7	44.9	44.9	39.8	25.414	0.0000	27.776	6164500	68	14.98	14.35	16.42	15.63	14.56	15.00	15.36	14.69
0.370	0.44	1.36	Q5H8C4	Vacuolar protein sorting-associated protein 13A	Vps13a	2106	11	11	11	5.9	5.9	5.9	359.4	0.0000	33.801	4382400	65	13.97	13.96	16.04	14.44	13.72	14.33	14.36	14.23
0.435	0.44	1.36	Q80Y81	Zinc phosphodiesterase ELAC protein 2	Elac2	2896	15	15	15	22.1	22.1	22.1	92.718	0.0000	70.363	13801000	118	15.06	15.08	16.60	15.08	14.92	14.80	15.71	14.64
0.311	0.44	1.35	A2RSJ4	UHRF1-binding protein 1-like	Uhrf1bp1l	47	6	6	6	6.3	6.3	6.3	161.94	0.0000	13.996	7116000	49	15.35	15.22	15.86	15.12	15.21	15.71	15.60	13.26
1.181	0.44	1.35	O70133	ATP-dependent RNA helicase A	Dhx9	470	56	56	56	40.9	40.9	40.9	149.47	0.0000	323.31	204580000	1291	18.02	17.85	18.64	18.15	17.50	17.66	17.78	17.96
0.408	0.44	1.35	Q9Z0U1	Tight junction protein ZO-2	Tjp2	5787	8	8	8	11	11	11	131.28	0.0000	25.459	8379000	53	15.75	15.78	17.59	15.75	15.65	16.16	15.67	15.65
0.624	0.44	1.35	Q9EPL8	Importin-7	Ipo7	5259	21	21	20	21.5	21.5	20.5	119.49	0.0000	160.2	51005000	373	16.77	16.78	17.96	17.04	16.37	17.05	16.98	16.41
1.455	0.44	1.35	Q99KN2	Probable cytosolic iron-sulfur protein assembly protein CIAO1	Ciao1	4385	9	9	9	32.2	32.2	32.2	37.632	0.0000	36.998	21508000	145	16.18	15.66	16.25	16.01	15.83	15.53	15.61	15.39
0.494	0.44	1.35	Q6YGZ1	Heparanase;Heparanase 8 kDa subunit;Heparanase 50 kDa subunit	Hpse	2673	14	14	14	37.9	37.9	37.9	60.065	0.0000	146.29	36182000	249	16.66	16.88	16.85	15.79	16.72	16.34	15.24	16.14
0.167	0.44	1.35	Q05117	Tartrate-resistant acid phosphatase type 5	Acp5	1738	3	3	3	15.6	15.6	15.6	36.807	0.0000	41.956	6101700	36	15.42	15.40	12.62	15.61	15.63	13.85	15.31	12.52
0.562	0.44	1.35	Q9WU28	Prefoldin subunit 5	Pfdn5	5695	8	8	8	57.1	57.1	57.1	17.356	0.0000	64.102	6014800	102	14.59	15.66	14.48	15.11	13.91	14.75	14.39	15.04
1.484	0.44	1.35	P40124	Adenylyl cyclase-associated protein 1	Cap1	1056	63	63	63	89.9	89.9	89.9	51.564	0.0000	323.31	2910800000	4020	22.13	21.88	22.44	22.30	21.66	21.70	22.05	21.60
1.631	0.44	1.35	Q9D662	Protein transport protein Sec23B	Sec23b	4990	38	38	35	64.8	64.8	61.1	86.436	0.0000	323.31	162520000	948	17.88	17.94	18.44	17.95	17.52	17.55	17.79	17.60
1.081	0.44	1.35	Q9DCS3	Trans-2-enoyl-CoA reductase, mitochondrial	Mecr	5227	17	17	17	68.9	68.9	68.9	40.342	0.0000	323.31	95646000	585	17.39	17.29	18.00	17.36	16.91	17.28	17.30	16.79
0.650	0.44	1.35	B1AXP6	Mitochondrial import receptor subunit TOM5 homolog	Tomm5	72	6	6	6	37.3	37.3	37.3	6.0372	0.0000	17.487	13751000	118	15.64	15.09	16.17	16.04	14.98	15.45	15.83	14.95
0.286	0.43	1.35	O35516	Neurogenic locus notch homolog protein 2;Notch 2 extracellular truncati	Notch2	378	6	6	5	4.2	4.2	4.2	265.63	0.0000	19.238	6994900	59	15.71	14.87	17.41	16.10	15.38	16.00	16.28	14.70
0.924	0.43	1.35	Q99N69	Leupaxin	Lpxn	4484	22	22	22	55.4	55.4	55.4	43.478	0.0000	323.31	139590000	674	17.92	17.66	18.68	18.20	17.56	17.49	17.93	17.74
0.237	0.43	1.35	Q00993	Tyrosine-protein kinase receptor UFO	Axl	1702	9	9	8	15.2	15.2	13.5	98.19	0.0000	26.476	3799700	31	13.14	12.82	15.90	13.86	13.73	13.48	14.01	12.76
0.792	0.43	1.35	P61028	Ras-related protein Rab-8B	Rab8b	1367	19	16	14	59.9	54.6	50.7	23.603	0.0000	188.29	120520000	420	18.65	18.12	19.41	18.91	18.25	18.42	18.31	18.37
0.294	0.43	1.35	P28843	Dipeptidyl peptidase 4;Dipeptidyl peptidase 4 membrane form;Dipeptidy	Dpp4	963	10	10	10	13.7	13.7	13.7	87.436	0.0000	28.209	7193100	63	15.50	15.89	15.11	13.83	15.39	14.04	15.38	13.81
1.879	0.43	1.35	O70145	Neutrophil cytosol factor 2	Ncf2	471	36	36	36	62.1	62.1	62.1	59.485	0.0000	323.31	250590000	1133	18.79	18.38	18.46	18.60	18.19	17.88	18.28	18.16
0.434	0.43	1.35	Q9QUK3	Protein CLN8	Cln8	5516	4	4	4	19.1	19.1	19.1	33.108	0.0000	53.759	3136800	35	14.33	15.31	15.14	14.89	15.21	14.64	14.72	13.38
0.275	0.43	1.35	Q8BZQ7	Anaphase-promoting complex subunit 2	Anapc2	3317	2	2	2	3.6	3.6	3.6	95.306	0.0000	10.186	2686500	56	13.44	12.90	15.19	13.46	14.45	12.45	13.21	13.15
0.372	0.43	1.35	Q9CWD8	Iron-sulfur protein NUBPL	Nubpl	4710	4	4	4	23.5	23.5	23.5	34.139	0.0000	31.185	2826200	43	15.14	14.45	14.04	13.26	14.33	13.06	13.47	14.30
0.213	0.43	1.35	P31725	Protein S100-A9	S100a9	988	4	4	4	32.7	32.7	32.7	13.049	0.0000	59.567	4990500	61	14.27	14.17	14.58	15.49	15.58	12.31	13.69	15.20
0.393	0.43	1.35	Q9CYR6	Phosphoacetylglucosamine mutase	Pgm3	4806	14	14	14	31.5	31.5	31.5	59.452	0.0000	26.581	11181000	116	14.57	15.05	15.84	15.51	14.72	15.16	13.76	15.60
0.524	0.43	1.35	Q6PAR5	GTPase-activating protein and VPS9 domain-containing protein 1	Gapvd1	2599	28	28	28	23.3	23.3	23.3	162.4	0.0000	91.637	53583000	326	17.81	17.59	19.03	18.11	17.51	17.63	18.30	17.38
1.117	0.43	1.35	Q60963	Platelet-activating factor acetylhydrolase	Pla2g7	2229	22	22	22	50.7	50.7	50.7	49.258	0.0000	203.42	109020000	570	17.54	17.60	18.17	17.44	17.11	17.48	17.44	17.01
0.484	0.43	1.35	Q3U2P1	Protein transport protein Sec24A	Sec24a	1912	8	8	8	11.2	11.2	11.2	118.78	0.0000	12.422	5831100	44	14.71	15.17	14.44	14.58	13.22	14.90	14.59	14.48
1.520	0.43	1.35	P07356	Annexin A2	Anxa2	670	63	63	63	94.1	94.1	94.1	38.676	0.0000	323.31	3252200000	3618	21.43	21.12	21.61	21.51	21.17	20.70	21.15	20.95
0.873	0.43	1.34	Q61093	Cytochrome b-245 heavy chain	Cybb	2250	27	27	27	42.5	42.5	42.5	65.304	0.0000	323.31	334160000	716	19.36	19.44	19.42	19.66	19.09	18.37	19.30	19.43
0.307	0.43	1.34	Q05920	Pyruvate carboxylase, mitochondrial	Pc	1744	30	30	30	35.8	35.8	35.8	129.68	0.0000	105.32	37456000	357	16.72	15.49	17.05	15.86	16.69	15.32	16.56	14.84
2.001	0.43	1.34	P51863	V-type proton ATPase subunit d 1	Atp6v0d1	1206	21	21	21	54.7	54.7	54.7	40.301	0.0000	323.31	237140000	690	19.46	19.66	19.96	19.55	19.33	19.20	19.26	19.15
1.780	0.43	1.34	Q99KI3	ER membrane protein complex subunit 3	Emc3	4378	7	7	7	28.4	28.4	28.4	29.98	0.0000	63.944	20136000	243	16.52	16.09	16.55	16.37	15.89	16.07	16.08	15.77
0.594	0.43	1.34	Q99KN9	Clathrin interactor 1	Clint1	4386	21	21	21	34.5	34.5	34.5	68.512	0.0000	135.26	50202000	354	16.67	16.86	18.00	17.00	16.24	16.90	16.72	16.96
0.406	0.43	1.34	Q8K3G9	DCC-interacting protein 13-beta	Appl2	3701	20	20	19	58.5	58.5	57.4	73.853	0.0000	323.31	20269000	194	16.12	15.79	17.68	16.73	16.03	16.00	16.74	15.84
0.686	0.43	1.34	P11276	Fibronectin;Anastellin	Fn1	741	12	12	12	7.1	7.1	7.1	272.53	0.0000	73.367	10080000	101	15.60	15.71	15.79	14.66	15.17	14.68	15.33	14.89
0.365	0.42	1.34	O55126	Protein NipSnap homolog 2	Gbas	456	11	11	11	39.1	39.1	39.1	32.932	0.0000	33.339	32284000	267	15.91	15.76	17.92	16.39	15.88	16.33	16.15	15.92
1.072	0.42	1.34	Q9Z1G3	V-type proton ATPase subunit C 1	Atp6v1c1	5807	39	39	39	87.4	87.4	87.4	43.887	0.0000	323.31	230220000	954	18.28	18.18	18.79	18.17	18.09	18.26	17.74	17.64
0.498	0.42	1.34	Q6P549	Phosphatidylinositol 3,4,5-trisphosphate 5-phosphatase 2	Inppl1	2568	41	39	37	40.1	38.7	36.9	138.97	0.0000	323.31	72598000	414	17.54	16.93	18.63	17.73	17.04	17.08	17.76	17.25
0.421	0.42	1.34	Q8BVU0	Leucine-rich repeat and calponin homology domain-containing protein 3	Lrch3	3247	13	13	13	23.8	23.8	23.8	86.34	0.0000	80.364	15653000	154	15.78	15.38	17.16	15.94	15.11	15.80	16.17	15.49
1.087	0.42	1.34	Q91VF2	Histamine N-methyltransferase	Hnmt	4060	6	6	6	32.9	32.9	32.9	33.664	0.0000	23.129	7429500	62	15.16	15.03	15.38	15.26	15.01	14.66	15.15	14.31

1.717	0.42	1.34	P97807	Fumarate hydratase, mitochondrial	Fh	1673	27	27	27	70.8	70.8	70.8	54.356	0.0000	323.31	188160000	762	18.92	18.57	19.08	19.00	18.26	18.60	18.52	18.50
0.737	0.42	1.34	P62301	40S ribosomal protein S13	Rps13	1416	21	21	21	72.2	72.2	72.2	17.222	0.0000	128.97	479710000	875	19.62	19.49	20.71	19.93	19.41	19.63	19.63	19.38
2.732	0.42	1.34	P08030	Adenine phosphoribosyltransferase	Aprt	675	18	18	17	96.7	96.7	96.1	19.724	0.0000	265.05	285310000	644	19.91	19.85	20.16	19.92	19.65	19.50	19.55	19.46
0.655	0.42	1.34	Q8CAA7	Glucose 1,6-bisphosphate synthase	Pgm2l1	3427	12	12	12	27.2	27.2	27.2	70.279	0.0000	284.1	20543000	170	16.10	16.53	17.12	16.20	16.12	16.05	16.55	15.54
0.710	0.42	1.34	Q9CQ80	Vacuolar protein-sorting-associated protein 25	Vps25	4572	13	13	13	77.3	77.3	77.3	20.747	0.0000	226.5	19544000	199	15.92	16.07	15.16	15.59	15.66	14.73	15.50	15.15
1.496	0.42	1.34	Q8BH58	TIP41-like protein	Tiprl	3014	13	13	13	60.1	60.1	60.1	31.253	0.0000	203.02	28982000	209	17.38	17.13	17.39	17.37	16.80	17.11	17.13	16.55
0.565	0.42	1.34	Q9WUL7	ADP-ribosylation factor-like protein 3	Arl3	5714	7	7	7	53.3	53.3	53.3	20.486	0.0000	38.699	10340000	119	15.54	15.12	16.63	15.53	15.16	15.55	15.46	14.97
1.403	0.42	1.34	O08810	116 kDa U5 small nuclear ribonucleoprotein component	Eftud2	304	41	39	39	56.6	55.5	55.5	109.36	0.0000	244.55	81746000	748	16.84	16.68	17.28	16.81	16.37	16.37	16.76	16.42
1.024	0.42	1.34	Q99P72	Reticulon-4	Rtn4	4502	25	25	25	32.4	32.4	32.4	126.61	0.0000	323.31	339280000	558	19.43	19.48	20.27	19.81	19.25	19.24	19.58	19.24
0.755	0.42	1.34	Q7TQ95	Protein lunapark	Lnp	2773	7	7	7	20.2	20.2	20.2	47.499	0.0000	25.415	13429000	131	15.33	15.04	14.73	15.55	14.54	15.17	15.00	14.26
0.546	0.42	1.34	Q8VEM8	Phosphate carrier protein, mitochondrial	Slc25a3	4015	22	22	22	54.1	54.1	54.1	39.632	0.0000	261.91	490090000	736	20.47	20.34	19.16	20.40	20.01	19.88	19.23	19.57
2.391	0.42	1.34	Q9QYI3	DnaJ homolog subfamily C member 7	Dnajc7	5573	22	22	22	50.2	50.2	50.2	56.475	0.0000	105.47	30132000	343	15.77	15.49	15.79	15.67	15.31	15.16	15.42	15.16
0.558	0.42	1.34	E9Q2M9		Wdfy4	230	39	39	39	17.4	17.4	17.4	354.33	0.0000	138.5	35948000	300	16.09	15.77	16.99	16.55	16.07	15.30	16.35	16.02
0.352	0.42	1.34	P17809	Solute carrier family 2, facilitated glucose transporter member 1	Slc2a1	831	5	5	5	11.2	11.2	11.2	53.984	0.0000	68.322	8924900	80	15.51	16.08	16.05	15.42	15.74	13.93	16.04	15.70
1.249	0.42	1.34	Q80U63	Mitofusin-2	Mfn2	2821	8	8	8	13.3	13.3	13.3	86.187	0.0000	9.8838	3449200	32	13.43	13.11	13.83	13.39	13.27	12.91	13.07	12.84
0.260	0.42	1.34	P42232	Signal transducer and activator of transcription 5B	Stat5b	1078	16	5	5	24.3	9.3	9.3	90.001	0.0000	11.998	6144900	72	15.15	14.17	16.18	14.95	13.61	15.64	14.04	15.49
0.530	0.42	1.34	P59016	Vacuolar protein sorting-associated protein 33B	Vps33b	1328	15	15	15	30	30	30	70.525	0.0000	87.843	20164000	184	16.11	15.89	17.35	16.01	15.96	16.04	16.14	15.55
1.427	0.42	1.34	P68510	14-3-3 protein eta	Ywhah	1522	31	25	25	84.1	81.3	81.3	28.211	0.0000	323.31	560210000	938	20.43	20.24	20.95	20.37	20.10	20.10	20.04	20.07
0.239	0.42	1.34	Q8BGY7	Protein FAM210A	Fam210a	3001	6	6	6	30	30	30	31.599	0.0000	22.493	4030500	43	14.04	12.92	13.04	13.85	14.93	12.80	12.00	12.44
1.481	0.42	1.33	Q8R1B4	Eukaryotic translation initiation factor 3 subunit C	Eif3c	3783	54	54	54	52.9	52.9	52.9	105.53	0.0000	323.31	197520000	1189	17.77	17.47	18.15	17.86	17.33	17.34	17.57	17.36
0.363	0.42	1.33	Q8C561	LMBR1 domain-containing protein 2	Lmbrd2	3393	8	8	8	14.3	14.3	14.3	81.1	0.0000	159.72	4103600	53	13.29	13.01	13.29	13.36	11.43	13.16	12.96	13.73
0.521	0.42	1.33	Q80YQ8	Protein RMD5 homolog A	Rmnd5a	2904	6	6	6	21.7	21.7	21.7	43.992	0.0000	15.66	5253100	89	14.26	13.54	13.24	14.41	13.03	13.46	14.10	13.18
0.431	0.42	1.33	Q9D6L8	Peptidyl-prolyl cis-trans isomerase-like 3	Ppil3	5002	2	2	2	20.5	20.5	20.5	18.127	0.0000	12.62	3244200	29	14.31	14.68	14.27	14.37	14.28	13.21	15.04	13.43
0.608	0.42	1.33	Q9Z0P5	Twinfilin-2	Twf2	5780	25	25	22	77.4	77.4	71.3	39.47	0.0000	323.31	147000000	642	18.16	17.99	19.37	18.32	17.78	18.06	18.19	18.15
1.437	0.42	1.33	Q9CQV8	14-3-3 protein beta/alpha;14-3-3 protein beta/alpha, N-terminally proces	Ywhab	4641	34	25	24	85.4	81.7	78.9	28.086	0.0000	323.31	366190000	897	19.95	19.85	20.27	19.57	19.37	19.64	19.50	19.46
1.342	0.41	1.33	Q9DBG3	AP-2 complex subunit beta	Ap2b1	5151	51	51	28	55.2	55.2	36.3	104.58	0.0000	323.31	201480000	1114	17.70	17.41	18.03	17.92	17.16	17.44	17.56	17.24
0.919	0.41	1.33	Q99K48	Non-POU domain-containing octamer-binding protein	Nono	4362	46	44	44	75.9	75.9	75.9	54.54	0.0000	323.31	252450000	1208	18.55	18.24	19.30	18.80	18.17	18.35	18.37	18.34
0.587	0.41	1.33	Q9CZ13	Cytochrome b-c1 complex subunit 1, mitochondrial	Uqcrc1	4813	30	30	29	59.6	59.6	59.6	52.851	0.0000	323.31	179090000	846	18.25	17.89	19.28	18.10	18.09	17.88	18.23	17.66
2.084	0.41	1.33	Q8BWG8	Beta-arrestin-1	Arrb1	3258	18	17	17	49.5	47.6	47.6	46.972	0.0000	138.38	39566000	347	16.81	16.56	16.90	16.88	16.30	16.51	16.49	16.20
0.950	0.41	1.33	Q9DB05	Alpha-soluble NSF attachment protein	Napa	5116	21	21	21	76.3	76.3	76.3	33.189	0.0000	302.15	198380000	760	18.53	18.35	19.26	18.44	18.26	18.20	18.43	18.05
0.590	0.41	1.33	Q6ZQK5	Arf-GAP with coiled-coil, ANK repeat and PH domain-containing protein	Acap2	2700	25	25	24	42.6	42.6	41.6	87.21	0.0000	146.38	43330000	450	16.18	15.61	17.08	16.18	15.73	15.84	16.21	15.62
0.795	0.41	1.33	P26443;P00	Glutamate dehydrogenase 1, mitochondrial	Glud1	924	56	56	56	73.5	73.5	73.5	61.336	0.0000	323.31	1083900000	2443	20.13	19.81	20.98	20.08	19.76	19.97	19.87	19.75
0.276	0.41	1.33	Q80Y98	Phospholipase DDHD2	Ddhd2	2898	7	7	7	13.9	13.9	13.9	79.576	0.0000	18.194	4740000	32	13.57	13.48	14.87	13.79	14.10	13.57	14.39	12.02
1.133	0.41	1.33	P84091	AP-2 complex subunit mu	Ap2m1	1608	30	30	30	62.5	62.5	62.5	49.654	0.0000	133.45	158780000	635	18.26	17.81	18.60	18.28	17.81	17.95	18.00	17.55
0.524	0.41	1.33	Q9Z2N8	Actin-like protein 6A	Actl6a	5860	18	18	17	56.2	56.2	55.2	47.447	0.0000	252.47	55776000	344	16.90	16.26	15.82	17.26	16.11	15.73	16.52	16.23
0.685	0.41	1.33	P70398	Probable ubiquitin carboxyl-terminal hydrolase FAF-X	Usp9x	1564	49	49	49	24.8	24.8	24.8	290.71	0.0000	248.34	65875000	618	16.29	16.00	17.18	16.40	16.02	15.88	16.48	15.86
0.304	0.41	1.33	Q99KU0	Vacuole membrane protein 1	Vmp1	4395	3	3	3	12.6	12.6	12.6	45.96	0.0000	38.841	9302700	60	15.85	15.97	14.11	16.56	15.31	14.72	15.71	15.11
0.617	0.41	1.33	Q91V61	Sideroflexin-3	Sfxn3	4047	24	24	23	71.7	71.7	65.7	35.406	0.0000	323.31	151060000	794	18.02	17.71	19.13	18.19	17.71	18.07	17.89	17.75
0.370	0.41	1.33	Q80YE7	Death-associated protein kinase 1	Dapk1	2902	19	19	19	19.2	19.2	19.2	161.44	0.0000	55.716	10651000	99	14.66	14.18	16.24	14.81	14.56	15.08	14.38	14.24
1.251	0.41	1.33	P45376	Aldose reductase	Akr1b1	1094	27	27	27	69.9	69.9	69.9	35.732	0.0000	323.31	689630000	1095	20.22	20.08	19.72	20.49	19.62	19.74	19.90	19.61
0.469	0.41	1.33	P62046	Leucine-rich repeat and calponin homology domain-containing protein 1	Lrch1	1400	7	7	7	13.5	13.5	13.5	79.075	0.0000	21.555	7874900	98	15.03	14.63	16.37	15.20	14.63	15.07	15.16	14.73
0.672	0.41	1.33	Q8C0C7	Phenylalanine--tRNA ligase alpha subunit	Farsa	3330	36	36	36	63.2	63.2	63.2	57.598	0.0000	323.31	160340000	721	18.70	18.42	19.73	18.75	18.38	18.50	18.65	18.43
1.057	0.41	1.33	P17439	Glucosylceramidase	Gba	825	24	24	24	51.1	51.1	51.1	57.621	0.0000	323.31	123530000	554	17.88	17.98	18.07	17.97	17.66	17.97	17.60	17.03
0.888	0.41	1.33	P62245	40S ribosomal protein S15a	Rps15a	1409	15	15	15	82.3	82.3	82.3	14.839	0.0000	111.44	177940000	509	18.89	18.74	19.71	19.35	18.86	18.72	18.58	18.89
0.237	0.41	1.33	Q8BIP0	Aspartate--tRNA ligase, mitochondrial	Dars2	3063	5	5	5	10.6	10.6	10.6	74.101	0.0000	9.2232	3860700	47	14.63	13.49	15.92	14.22	13.68	15.54	14.01	13.40
0.481	0.41	1.33	B2RY56	RNA-binding protein 25	Rbm25	89	26	26	26	32.6	32.6	32.6	99.551	0.0000	180.75	33380000	353	16.17	15.94	17.43	15.88	15.66	16.00	15.85	16.27
0.364	0.41	1.33	Q8CFE6	Sodium-coupled neutral amino acid transporter 2	Slc38a2	3485	4	4	4	12.5	12.5	12.5	55.503	0.0000	167.34	3159100	65	14.41	13.74	13.07	13.74	12.42	13.49	13.09	14.33
0.811	0.41	1.33	B1AVH7	TBC1 domain family member 2A	Tbc1d2	69	19	19	19	24.9	24.9	24.9	104.26	0.0000	41.354	16057000	115	15.67	15.42	16.29	15.77	15.70	14.91	15.55	15.35
0.873	0.41	1.32	Q99L27	GMP reductase 2	Gmpr2	4405	16	16	14	50.3	50.3	44.3	38.018	0.0000	99.388	38036000	249	16.66	16.22	17.19	16.75	16.15	16.65	16.11	16.30
0.717	0.41	1.32	Q810B6	Rabankyrin-5	Ankfy1	2926	40	40	40	46.8	46.8	46.8	128.65	0.0000	323.31	113880000	849	16.94	16.74	17.82	17.18	16.96	16.46	17.05	16.59
0.734	0.41	1.32	Q9QZ08	N-acetyl-D-glucosamine kinase	Nagk	5584	16	16	16	48.4	48.4	48.4	37.268	0.0000	63.926	37123000	324	16.72	17.05	17.73	17.08	17.00	17.05	16.53	16.38
1.354	0.41	1.32	Q8K2B3	Succinate dehydrogenase [ubiquinone] flavoprotein subunit, mitochondr	Sdha	3653	39	39	39	61.7	61.7	61.7	72.585	0.0000	323.31	182790000	962	18.21	17.74	18.37	18.31	17.67	17.61	17.93	17.81
0.452	0.40	1.32	P52623	Uridine-cytidine kinase 1	Uck1	1221	8	8	7	43	43	40.1	31.068	0.0000	33.196	7621500	86	15.09	14.43	14.55	14.67	14.80	14.18	14.89	13.25
1.684	0.40	1.32	Q99J36	THUMP domain-containing protein 1	Thumpd1	4322	10	10	10	32.9	32.9	32.9	38.884	0.0000	103.6	17528000	160	16.30	15.95	16.55	16.29	15.81	15.90	15.80	15.96
1.045	0.40	1.32	P55772	Ectonucleoside triphosphate diphosphohydrolase 1	Entpd1	1273	10	10	10	21.8	21.8	21.8	57.205	0.0000	119.19	24796000	138	16.75	16.48	17.27	16.91	16.67	16.45	16.54	16.13
0.687	0.40	1.32	O70252	Heme oxygenase 2	Hmox2	480	26	26	26	72.4	72.4	72.4	35.738	0.0000	323.31	141270000	702	17.92	17.49	18.82	18.22	17.72	17.56	17.86	17.69
0.336	0.40	1.32	Q8R5J9	PRA1 family protein 3	Arl6ip5	3890	15	15	15	39.9	39.9	39.9	21.557	0.0000	126.72	36792000	180	16.49	16.87	18.56	17.59	16.52	17.17	16.67	17.53
1.807	0.40	1.32	Q9D1E6	Tubulin-folding cofactor B	Tbcb	4908	18	18	18	80.7	80.7	80.7	27.385	0.0000	183.8	55696000	416	17.50	17.34	17.74	17.75	17.13	17.09	17.11	17.38
0.215	0.40	1.32	P34152	Focal adhesion kinase 1	Ptk2	1006	7	6	6	10.9	10.2	10.2	119.24	0.0000	40.006	2307000	35	12.45	12.46	14.92	14.08	13.88	12.68	13.67	12.08
1.078	0.40	1.32	Q80TY0	Formin-binding protein 1	Fnbp1	2815	28	28	27	47.1	47.1	47.1	71.343	0.0000	173.6	49960000	407	16.58	16.51	17.23	16.98	16.20	16.34	16.60	16.55
0.358	0.40	1.32	Q9D483	DNA-directed RNA polymerase III subunit RPC3	Polr3c	4972	4	4	4	13.5	13.5	13.5	60.705	0.0000	35.131	3153500	49	14.55	13.60	15.26	13.48	13.24	13.70	14.38	13.96
0.936	0.40	1.32	Q64442	Sorbitol dehydrogenase	Sord	2444	19	19	19	57.4	57.4	57.4	38.249	0.0000	155.49	54886000	317	17.23	16.98	17.83	16.94	16.87	16.81	17.02	16.68
0.561	0.40	1.32	Q80VI1	E3 ubiquitin-protein ligase TRIM56	Trim56	2850	9	9	9	19.8	19.8	19.8	79.512	0.0000	34.873	7428300	95	14.82	14.59	15.91	14.92	14.61	14.22	14.90	14.90
0.624	0.40	1.32	Q9Z0H7	B-cell lymphoma/leukemia 10	Bcl10	5772	4	4	3	35.2	35.2	24.5	25.948	0.0000	6.5683	2346100	28	13.50	13.91	14.90	14.00	13.81	13.69	13.75	13.45
0.189	0.40	1.32	Q6NSU3	Glycosyltransferase 8 domain-containing protein 1	Glt8d1	2527	3	3	3	8.1	8.1	8.1	41.99	0.0000	6.589	1891000	26	13.63	12.98	14.71	13.99	12.83	14.70	14.60	11.59
0.128	0.40	1.32	Q9WU81	Sugar phosphate exchanger 2	Slc37a2	5700	3	3	3	9.2	9.2	9.2	55.072	0.0000	13.323	10893000	78	15.26	16.65	14.37	15.70	16.58	12.17	16.83	14.80
0.605	0.40	1.32	O55143	Sarcoplasmic/endoplasmic reticulum calcium ATPase 2	Atp2a2	462	45	45	32	42.6	42.6	29.3	114.86	0.0000	323.31	271560000	1265	17.83	17.83	19.13	18.07	17.80	17.87	17.88	17.71
0.759	0.40	1.32	P06795	Multidrug resistance protein 1B	Abcb1b	662	33	33	13	33.2	33.2	13.3	140.99	0.0000	323.31	55450000	573	16.12	15.69	16.83	16.13	15.50	15.83	15.99	15.86
0.476	0.40	1.32	Q8CCB4	Vacuolar protein sorting-associated protein 53 homolog	Vps53	3445	16	16	16	24.8	24.8	24.8	94.422	0.0000	65.235	18697000	96	16.57	16.24	17.73	16.12	16.03	16.20	16.39	16.45
0.197	0.40	1.32	Q8BZ03	Serine/threonine-protein kinase D2	Prkd2	3309	3	3	3	4.9	4.9	4.9	96.541	0.0015	3.3737	1090500	16	13.20	12.84	15.32	13.49	12.32	14.88	13.43	12.63
0.306	0.40	1.32	Q6Q899	Probable ATP-dependent RNA helicase DDX58	Ddx58	2650	47	47	47	57.1	57.1	57.1	105.97	0.0000	287.09	82742000	581	16.46	15.77	17.80	17.22	16.14	15.74	17.25	16.53
0.380	0.40	1.32	Q7TNE3	Sperm-associated antigen 7	Spag7	2761	7	7	7	37.4	37.4	37.4	25.921	0.0000	39.888	2096800	15	12.57	13.77	12.83	13.23	11.61	12.83	13.16	13.21
0.664	0.40	1.32	P05063	Fructose-bisphosphate aldolase C	Aldoc	646	38	28	28	78.2	62.5	62.5	39.394	0.0000	323.31	122300000	574	17.77	17.80	18.74	18.15	17.27	17.67	18.11	17.83
0.689	0.40	1.32	Q9ERA6	Tuftelin-interacting protein 11	Tfip11	5301	17	17	17	22.4	22.4	22.4	96.304	0.0000	124.82	15212000	181	15.11	15.17	16.02	15.53	14.59	15.29	15.40	14.97
0.313	0.39	1.31	Q80UP3	Diacylglycerol kinase zeta	Dgkz	2834	13	13	11	17.2	17.2	14.5	104.03	0.0000	40.911	14482000	123	15.85	15.12	17.53	15.55	15.81	15.50	15.68	15.49
0.677	0.39	1.31	Q01730	Ras suppressor protein 1	Rsu1	1710	19	19	19	74.4	74.4	74.4	31.55	0.0000	223.25	73699000	308	17.96	17.85	17.90	17.68	17.54	16.79	17.36	18.13
0.564	0.39	1.31	Q9CPP6	NADH dehydrogenase [ubiquinone] 1 alpha subcomplex subunit 5	Ndufa5	4524	11	11	11	86.2	86.2	86.2	13.36	0.0000	125.51	16160000	148	16.05	15.70	17.12	16.54	15.77	16.03	15.81	16.23
0.335	0.39	1.31	O55128	Histone deacetylase complex subunit SAP18	Sap18	457	7	7	7	52.9	52.9	52.9	17.595	0.0000	77.541	17669000	139	16.27	15.78	15.11	16.62	14.42	15.84	16.02	15.93
0.686	0.39	1.31	Q9DBL7	Bifunctional coenzyme A synthase;Phosphopantetheine adenylyltransfe	Coasy	5161	13	13	13	35.7	35.7	35.7	62.022	0.0000	107.86	21084000	178	15.58	15.53	16.34	15.72	14.94	15.92	15.47	15.29
1.182	0.39	1.31	P30999	Catenin delta-1	Ctnnd1	982	44	44	44	58.5	58.5	58.5	104.92	0.0000	323.31	97535000	764	16.87	16.56	17.22	17.01	16.22	16.73	16.61	16.53
0.734	0.39	1.31	Q8R059	UDP-glucose 4-epimerase	Gale	3748	13	13	13	43.2	43.2	43.2	38.224	0.0000	137.25	14950000	164	15.66	15.29	16.06	16.10	15.13	15.03	15.69	15.70
1.431	0.39	1.31	P53026	60S ribosomal protein L10a	Rpl10a	1229	16	16	16	49.3	49.3	49.3	24.916	0.0000	133.04	280960000	650	19.38	19.01	19.42	19.56	18.95	19.10	19.05	18.71
0.297	0.39	1.31	Q8R0Y6	Cytosolic 10-formyltetrahydrofolate dehydrogenase	Aldh1l1	3769	21	21	19	35.4	35.4	33	98.708	0.0000	178.3	22743000	178	15.30	14.77	16.52	16.09	15.51	15.67	15.81	14.14
0.676	0.39	1.31	Q8BZN6	Dedicator of cytokinesis protein 10	Dock10	3316	56	56	56	31.6	31.6	31.6	245.76	0.0000	323.31	89185000	908	16.57	16.30	17.46	16.57	16.19	16.42	16.62	16.12
0.401	0.39	1.31	Q9ERU9	E3 SUMO-protein ligase RanBP2	Ranbp2	5320	71	71	70	29.8	29.8	29.5	341.12	0.0000	323.31	99914000	714	18.32	18.12	19.92	18.49	17.98	18.39	18.35	18.57
0.428	0.39	1.31	P63087	Serine/threonine-protein phosphatase PP1-gamma catalytic subunit	Ppp1cc	1489	21	4	3	63.2	12.7	9.3	36.983	0.0000	27.669	40884000	133	17.82	18.28	18.50	17.95	17.86	18.36	18.11	16.67
0.330	0.39	1.31	P97499	Telomerase protein component 1	Tep1	1665	16	16	16	10.1	10.1	10.1	291.46	0.0000	95.473	10900000	120	14.63	14.15	15.74	15.13	14.80	14.01	15.43	13.85
0.589	0.39	1.31	Q8BH65	Protein DENND6A	Dennd6a	3018	8	8	8	20.2	20.2	20.2	68.966	0.0000	10.98	8155000	43	15.88	15.91	16.43	15.74	15.65	16.03	15.90	14.83
0.487	0.39	1.31	Q0VGB7	Serine/threonine-protein phosphatase 4 regulatory subunit 2	Ppp4r2	1780	6	6	6	22.3	22.3	22.3	46.478	0.0000	32.65	7211500	65	14.86	14.18	14.27	15.25	14.26	13.76	14.96	14.02
1.167	0.39	1.31	P42932	T-complex protein 1 subunit theta	Cct8	1084	58	58	58	83.6	83.6	83.6	59.555	0.0000	323.31	598490000	1813	19.14	18.94	19.71	19.33	18.78	18.86	19.07	18.86
0.609	0.39	1.31	Q6PDG5	SWI/SNF complex subunit SMARCC2	Smarcc2	2611	27	27	18	26.5	26.5	20.4	132.6	0.0000	248.55	53120000	488	16.90	16.40	17.82	17.02	16.48	16.70	16.78	16.62
1.384	0.39	1.31	Q91W90	Thioredoxin domain-containing protein 5	Txndc5	4098	9	9	9	32.9	32.9	32.9	46.415	0.0000	25.202	15867000	143	15.61	15.84	15.85	16.07	15.63	15.12	15.46	15.60
0.882	0.39	1.31	Q8C854	Myelin expression factor 2	Myef2	3420	18	18	18	34.9	34.9	34.9	63.294	0.0000	89.675	39833000	270	17.03	16.69	17.45	16.70	16.20	16.75	16.62	16.75
1.255	0.39	1.31	Q11136	Xaa-Pro dipeptidase	Pepd	1783	30	30	30	57.4	57.4	57.4	55.028	0.0000	323.31	136510000	804	18.15	17.93	18.35	18.27	17.68	17.80	18.16	17.52
0.504	0.39	1.31	Q9JK23	Proteasome assembly chaperone 1	Psmg1	5442	12	12	12	43.6	43.6	43.6	33.104	0.0000	51.135	19620000	166	15.67	15.39	16.72	15.64	15.10	15.96	15.54	15.28
0.406	0.38	1.31	Q9D7I5	Phospholysine phosphohistidine inorganic pyrophosphate phosphatase	Lhpp	5031	4	4	4	26.7	26.7	26.7	29.144	0.0000	52.17	3529100	46	14.66	14.64	13.60	14.80	14.68	13.22	13.93	14.33
0.562	0.38	1.31	Q8C050	Ribosomal protein S6 kinase alpha-5	Rps6ka5	3324	19	19	19	30.9	30.9	30.9	96.582	0.0000	175.12	22607000	155	16.93	16.85	17.78	16.35	16.27	16.71	16.82	16.58
1.266	0.38	1.31	O70370	Cathepsin S	Ctss	491	21	21	21	59.4	59.4	59.4	38.474	0.0000	323.31	584330000	865	20.70	21.03	20.40	21.11	20.43	20.43	20.42	20.41
0.605	0.38	1.31	Q6A028	Switch-associated protein 70	Swap70	2496	41	41	41	62.9	62.9	62.9	68.995	0.0000	323.31	115450000	801	17.35	17.00	18.36	17.42	16.96	17.12	17.17	17.35
0.544	0.38	1.31	O35459	Delta(3,5)-Delta(2,4)-dienoyl-CoA isomerase, mitochondrial	Ech1	372	11	11	11	45.9	45.9	45.9	36.118	0.0000	112.9	17467000	159	16.42	16.26	17.06	15.85	16.12	16.48	15.98	15.47
0.981	0.38	1.31	Q9CXF4	TBC1 domain family member 15	Tbc1d15	4754	25	25	25	42.2	42.2	42.2	76.526	0.0000	107.65	38944000	258	16.36	16.35	16.88	16.46	15.87	16.00	16.59	16.05
1.195	0.38	1.30	Q9R1P0	Proteasome subunit alpha type-4	Psma4	5653	18	18	18	68.6	68.6	68.6	29.47	0.0000	258.47	69020000	407	17.40	17.94	17.42	17.35	16.85	17.22	17.24	17.27
0.212	0.38	1.30	Q9CQI7	U2 small nuclear ribonucleoprotein B	Snrpb2	4603	7	6	6	35.1	32	32	25.323	0.0000	40.296	7341500	84	14.44	15.58	13.79	15.22	14.47	12.68	15.42	14.93
1.522	0.38	1.30	P62259	14-3-3 protein epsilon	Ywhae	1410	32	29	29	85.5	85.5	85.5	29.174	0.0000	323.31	564100000	1397	20.44	20.42	20.90	20.72	20.05	20.22	20.38	20.29
0.477	0.38	1.30	Q9QWV9	Cyclin-T1	Ccnt1	5529	6	6	6	14.8	14.8	14.8	80.597	0.0000	110.82	4771500	62	14.54	14.54	15.33	14.59	13.85	13.87	15.08	14.68
0.938	0.38	1.30	O35075	Down syndrome critical region protein 3 homolog	Dscr3	333	6	6	6	30.3	30.3	30.3	32.97	0.0000	104.97	23637000	183	16.62	16.49	16.60	17.31	16.34	16.23	16.64	16.28
0.483	0.38	1.30	Q99JX7	Nuclear RNA export factor 1	Nxf1	4355	19	19	19	39.8	39.8	39.8	70.299	0.0000	84.122	29203000	276	15.87	15.90	17.23	16.21	15.58	16.14	16.25	15.71
0.185	0.38	1.30	Q9CRT8	Exportin-T	Xpot	4694	3	3	3	4.5	4.5	4.5	109.73	0.0000	29.154	4950000	58	15.26	15.14	13.52	15.07	14.70	12.42	15.71	14.62

1.289	0.38	1.30	Q07417	Short-chain specific acyl-CoA dehydrogenase, mitochondrial	Acads	1756	18	18	18	58.7	58.7	58.7	44.889	0.0000	323.31	131260000	608	18.52	18.31	18.48	18.53	18.15	18.19	18.32	17.64
1.225	0.38	1.30	Q5SSL4	Active breakpoint cluster region-related protein	Abr	2139	42	42	40	59.1	59.1	57	97.666	0.0000	307.91	114430000	714	17.76	17.49	17.05	17.59	17.01	17.18	17.22	16.96
0.604	0.38	1.30	O09012	Peroxisomal targeting signal 1 receptor	Pex5	317	8	8	8	20.5	20.5	20.5	70.755	0.0000	24.031	2172200	39	13.24	13.53	13.11	13.63	12.90	13.36	13.46	12.27
0.710	0.38	1.30	Q9EP97	Sentrin-specific protease 3	Senp3	5248	5	5	5	10.4	10.4	10.4	64.402	0.0000	15.882	4810800	73	14.81	14.97	15.54	14.85	14.59	14.98	14.94	14.12
0.583	0.38	1.30	Q5F2E7	Nuclear fragile X mental retardation-interacting protein 2	Nufip2	2103	12	12	12	23.6	23.6	23.6	75.656	0.0000	33.707	2826200	18	13.10	12.30	13.41	12.56	12.87	12.10	12.63	12.26
1.440	0.38	1.30	Q9Z0N1	Eukaryotic translation initiation factor 2 subunit 3, X-linked	Eif2s3x	5778	34	34	11	64.4	64.4	23.5	51.065	0.0000	323.31	223310000	1055	18.75	18.53	19.16	18.94	18.51	18.56	18.38	18.41
0.631	0.38	1.30	A2AN08	E3 ubiquitin-protein ligase UBR4	Ubr4	34	86	86	86	23.3	23.3	23.3	572.28	0.0000	323.31	115760000	1125	17.17	16.89	18.16	17.35	16.98	16.97	17.25	16.84
0.462	0.38	1.30	Q8CCH2	NHL repeat-containing protein 3	Nhlrc3	3447	5	5	5	17.9	17.9	17.9	38.195	0.0000	20.841	4974100	51	14.66	15.77	14.20	15.49	14.72	14.51	14.58	14.80
0.255	0.38	1.30	Q8CFA1	Interleukin-1 receptor-associated kinase-like 2	Irak2	3477	3	3	3	6.3	6.3	6.3	69.046	0.0010	4.013	1974300	11	14.30	14.16	12.61	13.99	14.53	12.34	13.65	13.03
1.153	0.38	1.30	Q9ES74	Serine/threonine-protein kinase Nek7	Nek7	5332	15	15	13	58.9	58.9	53.3	34.537	0.0000	90.715	34016000	340	16.26	15.95	16.52	16.56	15.88	16.07	16.14	15.70
0.956	0.38	1.30	Q99MK8	Beta-adrenergic receptor kinase 1	Adrbk1	4467	36	36	36	49.3	49.3	49.3	79.638	0.0000	279.03	85519000	828	17.22	16.92	17.59	17.37	16.94	16.48	17.09	17.09
0.523	0.38	1.30	Q8K2D3	Enhancer of mRNA-decapping protein 3	Edc3	3657	11	11	11	32.1	32.1	32.1	55.957	0.0000	30.791	7669900	113	14.27	14.27	15.25	14.68	13.59	14.35	14.75	14.27
1.139	0.38	1.30	Q9D0I9	Arginine--tRNA ligase, cytoplasmic	Rars	4869	51	51	51	68.6	68.6	68.6	75.673	0.0000	323.31	316650000	1459	18.96	18.64	19.15	19.23	18.41	18.50	18.94	18.62
1.044	0.38	1.30	P61290	Proteasome activator complex subunit 3	Psme3	1382	18	18	18	65	65	65	29.506	0.0000	82.102	59044000	237	17.25	16.98	17.54	17.51	16.74	17.29	17.01	16.72
0.960	0.38	1.30	P01899;P01	H-2 class I histocompatibility antigen, D-B alpha chain;H-2 class I histoc	H2-D1;H2-L	621	26	26	18	53.9	53.9	48.3	40.836	0.0000	222.05	309290000	670	19.45	19.29	20.14	19.39	19.10	19.09	19.31	19.25
0.968	0.38	1.30	Q8CG47	Structural maintenance of chromosomes protein 4	Smc4	3496	43	43	43	39.6	39.6	39.6	146.89	0.0000	213.12	43006000	518	16.44	16.00	16.83	16.59	15.83	16.11	16.30	16.12
0.647	0.38	1.30	O08547	Vesicle-trafficking protein SEC22b	Sec22b	270	20	20	20	79.1	79.1	79.1	24.74	0.0000	294.03	121490000	407	18.39	17.99	19.12	17.96	17.86	18.17	17.95	17.97
1.326	0.38	1.30	Q8JZU2	Tricarboxylate transport protein, mitochondrial	Slc25a1	3583	11	11	11	28.3	28.3	28.3	33.931	0.0000	27.184	33388000	244	16.58	16.47	17.01	16.92	16.21	16.27	16.47	16.53
0.672	0.37	1.30	Q99KH8	Serine/threonine-protein kinase 24;Serine/threonine-protein kinase 24 3	Stk24	4376	18	18	9	53.6	53.6	36.7	47.953	0.0000	112.65	39379000	305	16.71	16.85	17.74	16.88	16.31	16.78	16.92	16.67
0.752	0.37	1.30	Q3UH60	Disco-interacting protein 2 homolog B	Dip2b	1966	24	24	23	21.8	21.8	21.2	171.12	0.0000	146.38	23786000	302	15.72	15.46	16.33	15.42	15.20	15.57	15.56	15.09
0.598	0.37	1.29	Q9DCN2	NADH-cytochrome b5 reductase 3;NADH-cytochrome b5 reductase 3 m	Cyb5r3	5224	16	16	16	57.5	57.5	57.5	34.127	0.0000	323.31	252800000	597	19.98	19.54	20.80	19.69	19.49	19.77	19.48	19.79
0.420	0.37	1.29	Q9CQE3	28S ribosomal protein S17, mitochondrial	Mrps17	4588	2	2	2	15	15	15	13.382	0.0025	2.8818	2051900	37	13.70	13.27	13.10	14.55	12.63	13.39	13.45	13.65
0.377	0.37	1.29	O70161	Phosphatidylinositol 4-phosphate 5-kinase type-1 gamma	Pip5k1c	474	8	8	7	18.9	18.9	16.8	72.407	0.0000	51.756	8007100	88	15.61	15.40	15.60	15.31	15.67	13.84	15.45	15.46
0.970	0.37	1.29	Q9CX34	Suppressor of G2 allele of SKP1 homolog	Sugt1	4746	15	15	15	55.7	55.7	55.7	38.158	0.0000	220.1	40160000	323	16.98	17.03	17.47	17.35	16.42	16.94	16.80	17.17
0.352	0.37	1.29	P61750	ADP-ribosylation factor 4	Arf4	1388	13	9	7	67.8	43.9	27.8	20.396	0.0000	109.05	39940000	173	17.85	18.21	19.05	17.98	18.32	16.80	18.23	18.26
0.503	0.37	1.29	F6ZDS4	Nucleoprotein TPR	Tpr	257	50	50	50	27.8	27.8	27.8	273.99	0.0000	310.82	40299000	557	15.89	15.43	16.93	16.00	15.40	15.89	15.56	15.92
0.695	0.37	1.29	Q9Z2A0	3-phosphoinositide-dependent protein kinase 1	Pdpk1	5835	7	7	7	17.7	17.7	17.7	63.758	0.0000	39.56	4825600	71	15.36	14.88	14.39	14.79	14.31	14.61	14.13	14.88
0.400	0.37	1.29	P31324	cAMP-dependent protein kinase type II-beta regulatory subunit	Prkar2b	987	14	11	11	46.4	36.5	36.5	46.167	0.0000	60.451	9547900	128	16.00	15.42	14.47	15.00	14.99	15.42	14.23	14.77
0.435	0.37	1.29	Q8JZM7	Parafibromin	Cdc73	3574	16	16	16	32.8	32.8	32.8	60.576	0.0000	61.345	19060000	181	15.89	15.48	17.06	16.22	15.45	16.22	15.56	15.95
0.534	0.37	1.29	Q9JHF7	Hematopoietic prostaglandin D synthase	Hpgds	5358	9	9	9	56.8	56.8	56.8	23.227	0.0000	105.35	39718000	178	17.92	17.62	17.45	17.76	17.84	16.61	17.81	17.00
1.662	0.37	1.29	Q9Z0H4	CUGBP Elav-like family member 2	Celf2	5771	17	17	15	32.3	32.3	28.1	54.27	0.0000	162.27	79207000	573	17.00	17.07	17.44	17.24	16.81	16.63	16.97	16.86
0.773	0.37	1.29	Q6PE01	U5 small nuclear ribonucleoprotein 40 kDa protein	Snrnp40	2622	13	13	13	57.3	57.3	57.3	39.275	0.0000	318.87	26890000	236	16.03	15.80	16.40	16.01	15.88	15.72	16.04	15.13
0.748	0.37	1.29	P28352	DNA-(apurinic or apyrimidinic site) lyase;DNA-(apurinic or apyrimidinic s	Apex1	949	19	19	19	71.6	71.6	71.6	35.49	0.0000	196.43	80228000	507	16.91	16.94	17.85	17.21	16.71	16.65	17.01	17.07
0.723	0.37	1.29	Q91XB7	Protein YIF1A	Yif1a	4140	2	2	2	7.2	7.2	7.2	32.134	0.0000	10.094	6046600	41	15.00	14.57	15.49	14.52	14.53	14.33	14.82	14.42
0.715	0.37	1.29	Q9D8Z1	Activating signal cointegrator 1 complex subunit 1	Ascc1	5086	5	5	5	17.4	17.4	17.4	41.279	0.0009	3.9257	4674100	17	15.25	14.99	15.82	15.44	15.06	15.23	14.48	15.26
2.574	0.37	1.29	P50544	Very long-chain specific acyl-CoA dehydrogenase, mitochondrial	Acadvl	1187	30	30	30	61.4	61.4	61.4	70.875	0.0000	323.31	105580000	792	17.65	17.35	17.58	17.44	17.12	17.12	17.24	17.07
0.640	0.37	1.29	Q8K3H0	DCC-interacting protein 13-alpha	Appl1	3702	20	19	19	41.6	40.6	40.6	79.327	0.0000	104.42	21893000	205	15.98	15.69	16.89	16.03	15.58	15.88	15.99	15.67
3.501	0.37	1.29	Q9WU78	Programmed cell death 6-interacting protein	Pdcd6ip	5698	76	76	76	74.2	74.2	74.2	96.023	0.0000	323.31	534680000	2218	19.11	19.00	19.08	19.11	18.60	18.79	18.76	18.69
0.289	0.37	1.29	Q6PEB6	MOB-like protein phocein	Mob4	2624	9	9	9	59.6	59.6	59.6	26.032	0.0000	146.86	12588000	125	14.97	15.19	17.30	15.81	15.51	15.55	15.43	15.32
1.188	0.37	1.29	O08529	Calpain-2 catalytic subunit	Capn2	268	53	53	52	72.6	72.6	71.7	79.871	0.0000	323.31	244720000	1209	18.13	17.96	18.63	18.28	17.79	17.72	18.07	17.95
0.235	0.37	1.29	P36916	Guanine nucleotide-binding protein-like 1	Gnl1	1044	10	10	10	22.4	22.4	22.4	68.77	0.0000	20.445	6260800	45	14.70	14.55	15.16	14.84	14.77	15.36	15.02	12.64
0.476	0.37	1.29	Q3UPF5	Zinc finger CCCH-type antiviral protein 1	Zc3hav1	2013	30	30	30	41.2	41.2	41.2	106.69	0.0000	181.58	32599000	360	16.05	15.71	17.15	16.56	15.59	16.15	16.28	16.00
1.752	0.36	1.29	Q9DCL9	Multifunctional protein ADE2;Phosphoribosylaminoimidazole-succinocar	Paics	5221	32	32	32	70.8	70.8	70.8	47.006	0.0000	208.46	153500000	782	18.37	17.98	18.46	18.34	17.95	17.84	18.00	17.89
0.499	0.36	1.29	O88531	Palmitoyl-protein thioesterase 1	Ppt1	540	12	12	12	37.6	37.6	37.6	34.49	0.0000	285.44	77989000	331	17.71	17.58	18.84	17.63	17.43	18.02	17.44	17.41
1.553	0.36	1.29	Q8BJ71	Nuclear pore complex protein Nup93	Nup93	3075	32	32	32	52.7	52.7	52.7	93.28	0.0000	323.31	67897000	528	17.38	17.14	17.63	17.53	17.09	16.95	17.24	16.94
0.282	0.36	1.29	Q60692	Proteasome subunit beta type-6	Psmb6	2195	9	9	9	52.9	52.9	52.9	25.378	0.0000	240.82	99778000	226	18.59	18.69	19.34	19.00	18.75	19.41	17.08	18.93
0.568	0.36	1.29	Q68FL6	Methionine--tRNA ligase, cytoplasmic	Mars	2477	41	41	41	51.9	51.9	51.9	101.43	0.0000	323.31	115680000	803	17.23	17.04	18.36	17.46	16.98	17.16	17.26	17.24
0.698	0.36	1.29	Q3UQ44	Ras GTPase-activating-like protein IQGAP2	Iqgap2	2016	67	64	64	46.1	44.4	44.4	180.53	0.0000	323.31	135090000	1261	17.36	16.97	18.13	17.68	17.10	17.15	17.35	17.09
0.305	0.36	1.29	Q9D8X1	Copper homeostasis protein cutC homolog	Cutc	5080	7	7	7	46.7	46.7	46.7	29.003	0.0000	65.843	3694800	55	14.69	13.76	15.58	14.06	13.59	14.59	14.75	13.71
0.290	0.36	1.29	Q8K2J7	RELT-like protein 1	Rell1	3663	4	4	4	29.8	29.8	29.8	29.351	0.0000	18.216	2485700	32	14.28	13.00	14.79	15.15	13.53	14.51	13.64	14.09
0.491	0.36	1.29	Q9QY81	Nuclear pore membrane glycoprotein 210	Nup210	5563	22	22	22	17.1	17.1	17.1	204.1	0.0000	114.22	21851000	299	15.76	15.31	16.46	15.71	15.76	15.10	15.95	14.98
0.361	0.36	1.29	Q9ESE1	Lipopolysaccharide-responsive and beige-like anchor protein	Lrba	5336	9	9	8	4.3	4.3	3.7	317.06	0.0000	14.005	3305400	52	14.38	13.04	14.99	14.16	13.42	13.70	14.12	13.89
0.793	0.36	1.29	P41216	Long-chain-fatty-acid--CoA ligase 1	Acsl1	1066	37	37	35	50.8	50.8	49.5	77.951	0.0000	157.18	82367000	726	16.89	16.51	17.52	16.73	16.37	16.56	16.67	16.60
0.652	0.36	1.28	Q9JJ28	Protein flightless-1 homolog	Flii	5417	53	53	53	54.3	54.3	54.3	144.8	0.0000	323.31	175230000	1029	17.74	17.53	18.71	17.98	17.54	17.54	17.81	17.63
1.009	0.36	1.28	Q9CRB9	MICOS complex subunit Mic19	Chchd3	4689	14	14	14	47.6	47.6	47.6	26.334	0.0000	128.23	44327000	360	17.06	17.19	17.49	17.06	16.87	17.26	16.59	16.63
0.607	0.36	1.28	Q9CX00	IST1 homolog	Ist1	4742	16	16	16	46.4	46.4	46.4	39.468	0.0000	278.44	28260000	286	16.34	15.69	16.90	15.84	15.64	15.96	15.80	15.92
1.420	0.36	1.28	O09131	Glutathione S-transferase omega-1	Gsto1	327	29	29	29	74.2	74.2	74.2	27.497	0.0000	271	219320000	871	19.35	18.99	18.77	19.15	18.64	18.82	18.57	18.78
0.300	0.36	1.28	P59438	Hermansky-Pudlak syndrome 5 protein homolog	Hps5	1337	7	7	7	10.4	10.4	10.4	126.34	0.0000	17.397	3812000	35	13.83	12.88	14.39	13.90	12.56	13.06	14.41	13.51
1.049	0.36	1.28	P97770	THUMP domain-containing protein 3	Thumpd3	1669	9	9	9	21.4	21.4	21.4	56.431	0.0000	34.872	9861200	99	15.15	14.69	15.16	15.05	14.91	14.82	14.59	14.29
0.451	0.36	1.28	Q5U458	DnaJ homolog subfamily C member 11	Dnajc11	2164	18	18	18	34.3	34.3	34.3	63.232	0.0000	129.23	18097000	178	15.47	15.13	16.51	15.32	14.72	15.52	15.43	15.32
1.933	0.36	1.28	O89079	Coatomer subunit epsilon	Cope	600	19	19	19	60.7	60.7	60.7	34.567	0.0000	207.38	76489000	460	17.44	17.57	17.56	17.43	17.11	17.10	16.95	17.40
0.357	0.36	1.28	Q9CQC8	Maspardin	Spg21	4584	13	13	13	47.4	47.4	47.4	34.952	0.0000	97.67	26240000	173	16.90	16.58	16.80	16.60	16.73	16.32	17.19	15.20
2.348	0.36	1.28	Q91XD6	Vacuolar protein-sorting-associated protein 36	Vps36	4142	19	19	19	58	58	58	43.735	0.0000	128.41	31495000	248	16.30	16.08	16.32	16.20	15.86	16.04	15.81	15.77
0.271	0.36	1.28	Q8K4B0	Metastasis-associated protein MTA1	Mta1	3712	10	4	4	17.5	8.8	8.8	80.797	0.0000	14.493	1512800	36	13.39	13.39	11.73	13.55	11.88	12.55	13.51	12.70
0.390	0.36	1.28	Q8CIE4		Parp10	3549	6	6	6	10.9	10.9	10.9	103.68	0.0000	18.857	3247500	22	13.42	13.62	13.98	13.97	13.93	13.33	13.95	12.34
0.328	0.36	1.28	Q9JJA4	Ribosome biogenesis protein WDR12	Wdr12	5422	7	7	7	30	30	30	47.346	0.0000	45.657	8948300	116	15.30	15.55	14.61	14.15	14.72	14.33	15.37	13.78
0.646	0.36	1.28	O70480	Vesicle-associated membrane protein 4	Vamp4	504	4	4	4	39	39	39	16.353	0.0000	54.043	7611100	87	15.42	14.88	16.06	15.44	15.23	15.01	15.30	14.83
0.360	0.36	1.28	D3YZP9	Coiled-coil domain-containing protein 6	Ccdc6	211	5	5	5	15.6	15.6	15.6	52.938	0.0000	21.813	2858100	25	14.69	14.51	13.10	13.93	13.13	13.99	13.53	14.17
0.760	0.36	1.28	Q60854	Serpin B6	Serpinb6	2212	44	44	44	94.4	94.4	94.4	42.598	0.0000	323.31	563390000	2098	19.43	19.39	20.29	19.64	19.58	19.15	19.41	19.18
0.379	0.36	1.28	P0C6B7	Immunoglobulin superfamily member 6	Igsf6	706	7	7	7	24.1	24.1	24.1	26.354	0.0000	80.882	21114000	154	16.31	15.76	17.25	16.94	15.97	16.77	16.42	15.68
0.526	0.36	1.28	Q69ZR2	E3 ubiquitin-protein ligase HECTD1	Hectd1	2490	18	18	18	9.9	9.9	9.9	290.08	0.0000	41.257	25661000	78	18.01	17.48	18.47	17.62	18.04	17.12	17.78	17.22
1.511	0.36	1.28	Q69ZN7	Myoferlin	Myof	2488	133	133	132	66.4	66.4	66.4	233.32	0.0000	323.31	516740000	2971	18.19	17.96	18.39	18.02	17.86	17.87	17.87	17.55
0.647	0.35	1.28	Q6NZC7	SEC23-interacting protein	Sec23ip	2544	19	19	19	24.8	24.8	24.8	110.78	0.0000	66.056	27280000	215	16.08	15.82	16.88	15.91	15.54	16.03	15.85	15.84
0.863	0.35	1.28	P16879	Tyrosine-protein kinase Fes/Fps	Fes	816	25	25	24	36.9	36.9	35.5	93.778	0.0000	238.47	48422000	378	16.50	16.16	17.05	16.60	15.96	16.19	16.37	16.37
0.498	0.35	1.28	Q8BJS4	SUN domain-containing protein 2	Sun2	3084	40	40	39	63.9	63.9	62.8	81.604	0.0000	323.31	85062000	740	16.69	16.18	17.52	16.92	16.00	16.46	16.73	16.70
0.372	0.35	1.28	Q8K400	Syntaxin-binding protein 5	Stxbp5	3709	15	15	15	22	22	22	127.65	0.0000	96.131	35062000	264	16.70	16.67	17.99	16.07	16.49	16.43	16.75	16.35
2.932	0.35	1.28	Q91VR5	ATP-dependent RNA helicase DDX1	Ddx1	4073	39	39	39	61.5	61.5	61.5	82.499	0.0000	323.31	115830000	811	17.33	17.30	17.32	17.40	17.07	17.04	17.00	16.82
0.346	0.35	1.28	Q9DBD5	Proline-, glutamic acid- and leucine-rich protein 1	Pelp1	5144	5	5	5	8.8	8.8	8.8	118.07	0.0000	102.19	6158800	100	15.44	14.41	16.24	15.15	14.89	15.02	15.50	14.42
1.417	0.35	1.28	A2A432	Cullin-4B	Cul4b	5	13	9	9	20.2	15.2	15.2	110.7	0.0000	106.53	5350500	89	14.46	14.04	14.32	14.56	13.82	13.96	14.17	14.02
0.266	0.35	1.28	Q61235	Beta-2-syntrophin	Sntb2	2283	10	10	10	21.5	21.5	21.5	56.381	0.0000	28.839	14583000	132	16.11	15.87	14.56	16.44	15.64	15.62	15.96	14.34
0.534	0.35	1.28	P25911	Tyrosine-protein kinase Lyn	Lyn	912	36	36	34	72.3	72.3	69.9	58.812	0.0000	323.31	159530000	941	17.49	17.10	18.46	17.32	17.20	17.10	17.37	17.29
0.884	0.35	1.28	Q922H4	Mannose-1-phosphate guanyltransferase alpha	Gmppa	4258	17	17	17	41.4	41.4	41.4	46.244	0.0000	53.738	28330000	261	16.41	16.21	16.76	16.72	15.78	16.08	16.39	16.44
0.163	0.35	1.28	Q6ZWY8	Thymosin beta-10	Tmsb10	2711	2	2	2	36.4	36.4	36.4	5.0256	0.0004	4.5672	3260200	29	14.73	14.59	13.67	15.07	15.46	14.49	11.91	14.79
0.932	0.35	1.28	Q9JHU4	Cytoplasmic dynein 1 heavy chain 1	Dync1h1	5374	297	297	297	63.4	63.4	63.4	532.04	0.0000	323.31	1162200000	6469	19.16	18.95	19.77	19.29	18.83	18.91	19.18	18.84
0.244	0.35	1.28	P09925	Surfeit locus protein 1	Surf1	702	6	6	6	29.7	29.7	29.7	34.798	0.0000	34.801	9396200	72	15.16	14.94	17.19	14.75	15.04	15.35	15.46	14.78
0.816	0.35	1.28	Q9Z110	Delta-1-pyrroline-5-carboxylate synthase;Glutamate 5-kinase;Gamma-g	Aldh18a1	5795	28	28	28	45.8	45.8	45.8	87.265	0.0000	303.36	72541000	463	16.95	16.68	17.64	16.82	16.66	16.61	16.76	16.65
0.705	0.35	1.28	Q921E6	Polycomb protein EED	Eed	4219	5	5	5	12.9	12.9	12.9	50.197	0.0002	6.0124	2544600	25	13.70	13.42	14.47	13.85	13.55	13.26	13.75	13.47
0.853	0.35	1.28	Q9WVJ2	26S proteasome non-ATPase regulatory subunit 13	Psmd13	5750	25	25	25	60.9	60.9	60.9	42.809	0.0000	323.31	99701000	480	17.70	17.89	18.41	17.64	17.34	17.85	17.48	17.57
0.414	0.35	1.28	Q811J3	Iron-responsive element-binding protein 2	Ireb2	2937	8	7	7	14	13.2	13.2	104.92	0.0000	57.051	6051200	75	14.64	14.60	14.64	14.10	14.70	13.80	14.75	13.32
0.638	0.35	1.28	P08103	Tyrosine-protein kinase HCK	Hck	679	29	28	28	58.8	58.8	58.8	59.129	0.0000	224.05	116870000	612	17.72	17.33	18.45	17.93	17.29	17.47	17.42	17.85
1.070	0.35	1.28	Q8K1Z0	Ubiquinone biosynthesis protein COQ9, mitochondrial	Coq9	3637	8	8	8	34.5	34.5	34.5	35.082	0.0000	100.35	11241000	146	15.54	15.21	15.66	15.74	14.82	15.29	15.37	15.27
0.741	0.35	1.27	Q99LM2	CDK5 regulatory subunit-associated protein 3	Cdk5rap3	4440	11	11	11	26.2	26.2	26.2	56.99	0.0000	24.073	13779000	149	15.56	15.40	16.11	15.90	15.07	15.85	15.24	15.42
0.464	0.35	1.27	Q8VD66	Abhydrolase domain-containing protein 4	Abhd4	3943	4	4	4	16.1	16.1	16.1	38.86	0.0000	11.942	4463900	46	15.05	14.75	14.03	14.34	14.68	14.59	13.74	13.75
0.548	0.35	1.27	Q640Q5	PAB-dependent poly(A)-specific ribonuclease subunit PAN3	Pan3	2416	4	4	4	7	7	7	89.744	0.0000	12.212	3277200	45	14.32	14.02	15.32	14.27	13.99	14.33	14.03	14.19
0.415	0.35	1.27	Q8CH72	E3 ubiquitin-protein ligase TRIM32	Trim32	3519	5	5	5	10.1	10.1	10.1	72.057	0.0000	18.669	4381200	49	14.47	13.17	14.79	14.19	13.46	13.84	14.07	13.85
0.939	0.35	1.27	Q3UPH1	Protein PRRC1	Prrc1	2014	10	10	10	33.9	33.9	33.9	46.297	0.0000	149.69	25625000	166	16.50	16.18	16.61	16.61	15.69	16.38	16.08	16.35
0.451	0.35	1.27	Q921W4	Quinone oxidoreductase-like protein 1	Cryzl1	4243	13	13	13	46.8	46.8	46.8	38.725	0.0000	69.12	16365000	126	16.18	15.48	16.26	16.09	15.37	16.13	16.17	14.95
0.262	0.35	1.27	Q8VDS4	Regulation of nuclear pre-mRNA domain-containing protein 1A	Rprd1a	3970	2	2	2	10.9	10.9	10.9	35.7	0.0000	9.5828	1584400	35	13.19	13.02	15.26	13.28	13.07	13.70	13.44	13.16
1.333	0.35	1.27	Q99KP6	Pre-mRNA-processing factor 19	Prpf19	4388	24	24	24	55.8	55.8	55.8	55.238	0.0000	323.31	128250000	587	17.83	17.37	17.86	17.74	17.21	17.45	17.53	17.22
1.023	0.35	1.27	Q61187	Tumor susceptibility gene 101 protein	Tsg101	2270	18	18	18	53.2	53.2	53.2	44.123	0.0000	162	34855000	302	15.92	16.31	16.67	16.39	15.85	16.20	15.98	15.86
0.696	0.35	1.27	Q9EPE9	Manganese-transporting ATPase 13A1	Atp13a1	5252	31	31	31	31.3	31.3	31.3	132.39	0.0000	151.4	64149000	390	16.61	16.52	17.01	16.59	16.48	16.53	16.64	15.70
0.314	0.35	1.27	P50295	Arylamine N-acetyltransferase 2	Nat2	1178	9	9	9	46.2	46.2	46.2	33.701	0.0000	65.58	18901000	127	16.76	16.07	16.97	17.30	15.88	17.44	15.77	16.61
0.515	0.35	1.27	Q99NB1	Acetyl-coenzyme A synthetase 2-like, mitochondrial	Acss1	4495	8	8	8	17	17	17	74.622	0.0000	56.464	6917400	112	14.81	14.46	14.70	14.96	14.65	14.31	14.97	13.61
0.323	0.35	1.27	Q9CY97	RNA polymerase II subunit A C-terminal domain phosphatase SSU72	Ssu72	4792	6	6	6	36.6	36.6	36.6	22.517	0.0000	7.672	3548500	19	14.00	14.51	14.16	13.41	13.97	13.33	12.79	14.60
0.661	0.35	1.27	O55106	Striatin	Strn	454	20	20	19	47.8	47.8	46.5	85.965	0.0000	228.61	35636000	285	16.26	16.30	17.25	16.43	15.96	16.31	16.22	16.36
0.460	0.34	1.27	Q922H2	[Pyruvate dehydrogenase (acetyl-transferring)] kinase isozyme 3, mitocl	Pdk3	4257	10	10	10	34.2	34.2	34.2	47.922	0.0000	42.272	12342000	146	15.28	15.14	16.26	15.66	14.89	15.90	15.10	15.07
2.598	0.34	1.27	Q99KQ4	Nicotinamide phosphoribosyltransferase	Nampt	4389	39	39	39	68.6	68.6	68.6	55.446	0.0000	323.31	305610000	1175	18.82	18.85	18.77	18.81	18.34	18.63	18.52	18.38
0.955	0.34	1.27	Q63932	Dual specificity mitogen-activated protein kinase kinase 2	Map2k2	2408	17	9	9	30.9	16	16	44.402	0.0000	45.56	19320000	206	15.78	15.69	16.33	16.02	15.36	15.90	15.66	15.53

1.410	0.34	1.27	Q5EG47	5-AMP-activated protein kinase catalytic subunit alpha-1	Prkaa1		2101	19	19	19	39.2	39.2	39.2	63.928	0.0000	86.296	39699000	263	16.69	16.35	16.83	16.60	16.21	16.19	16.53	16.17
0.833	0.34	1.27	Q99KK9	Probable histidine--tRNA ligase, mitochondrial	Hars2		4383	15	11	11	33.3	27.7	27.7	56.985	0.0000	27.873	12767000	138	15.60	15.34	15.03	15.26	15.11	15.37	14.76	14.62
0.939	0.34	1.27	P09103	Protein disulfide-isomerase	P4hb		693	54	54	54	81.1	81.1	81.1	57.058	0.0000	323.31	431830000	1855	19.00	19.05	18.36	19.12	18.67	18.59	18.37	18.53
0.899	0.34	1.27	Q6ZQ38	Cullin-associated NEDD8-dissociated protein 1	Cand1		2689	51	51	50	47.1	47.1	46.3	136.33	0.0000	323.31	160400000	1105	17.76	17.51	18.22	18.05	17.41	17.43	17.89	17.44
0.307	0.34	1.27	Q921I2	Kelch domain-containing protein 4	Klhdc4		4227	4	4	4	13.7	13.7	13.7	64.86	0.0000	37.104	2097500	35	13.51	13.39	15.06	12.94	13.29	13.16	13.57	13.51
1.042	0.34	1.27	Q9WV32	Actin-related protein 2/3 complex subunit 1B	Arpc1b		5730	36	36	33	68.3	68.3	59.7	41.063	0.0000	323.31	771640000	1813	20.44	20.10	20.70	20.70	20.34	20.11	20.23	19.90
0.501	0.34	1.27	P80317	T-complex protein 1 subunit zeta	Cct6a		1594	37	37	32	61.4	61.4	58.2	58.004	0.0000	323.31	598070000	1903	19.18	18.97	20.34	19.34	19.07	19.24	19.21	18.93
0.208	0.34	1.27	Q9WUB4	Dynactin subunit 6	Dctn6		5707	2	2	2	14.7	14.7	14.7	20.67	0.0000	24.661	5600600	67	14.99	15.08	14.62	14.94	15.10	15.15	15.38	12.63
0.143	0.34	1.27	Q9WTK3	Glycosylphosphatidylinositol anchor attachment 1 protein	Gpaa1		5675	5	5	5	11	11	11	67.948	0.0000	27.356	12557000	85	15.70	15.73	13.49	15.65	15.44	12.66	15.82	15.28
0.937	0.34	1.27	Q921F2	TAR DNA-binding protein 43	Tardbp		4220	18	18	18	54.1	54.1	54.1	44.547	0.0000	323.31	119650000	495	18.36	17.62	17.94	18.33	17.57	17.78	17.84	17.69
0.729	0.34	1.27	Q920Q4	Vacuolar protein sorting-associated protein 16 homolog	Vps16		4213	27	27	27	41	41	41	94.927	0.0000	212.92	59140000	446	16.73	16.52	17.49	17.19	16.58	16.80	16.70	16.48
0.583	0.34	1.27	Q60973	Histone-binding protein RBBP7	Rbbp7		2232	11	11	4	29.4	29.4	19.1	47.789	0.0000	102.01	15592000	141	15.85	15.67	16.12	16.78	15.43	16.05	15.78	15.81
0.439	0.34	1.27	P54276	DNA mismatch repair protein Msh6	Msh6		1250	25	25	24	26.3	26.3	25.4	151.08	0.0000	62.213	61904000	166	18.77	18.66	19.88	18.92	18.11	18.85	18.99	18.93
0.564	0.34	1.27	P49138	MAP kinase-activated protein kinase 2	Mapkapk2		1156	26	26	25	61.4	61.4	58.3	44.049	0.0000	155.09	73545000	420	17.23	16.80	17.94	17.80	16.83	17.09	17.20	17.28
0.506	0.34	1.27	Q8K2T1	NmrA-like family domain-containing protein 1	Nmral1		3671	12	12	12	56.3	56.3	56.3	34.376	0.0000	137.12	25442000	218	16.04	15.98	15.69	16.04	15.54	14.81	15.85	16.20
0.273	0.34	1.26	P56959	RNA-binding protein FUS	Fus		1293	9	9	9	26.1	26.1	26.1	52.673	0.0000	65.936	17256000	101	15.93	16.29	17.53	16.07	15.14	16.18	16.28	16.86
0.465	0.34	1.26	A2AWA9	Rab GTPase-activating protein 1	Rabgap1		42	16	16	15	18.4	18.4	17.6	120.8	0.0000	98.549	15638000	158	14.97	14.92	15.95	14.92	14.87	14.26	15.23	15.05
0.278	0.34	1.26	Q8VC42	Uncharacterized protein C18orf8 homolog	Mic1		3909	8	8	8	18.6	18.6	18.6	74.922	0.0000	27.71	3994200	40	14.52	14.81	15.92	14.36	14.98	14.41	13.61	15.26
1.854	0.34	1.26	O88342	WD repeat-containing protein 1	Wdr1		523	52	52	52	79	79	79	66.406	0.0000	323.31	1206500000	2665	20.42	20.26	20.24	20.39	19.93	20.23	19.98	19.82
0.226	0.34	1.26	Q61470	Leukocyte antigen CD37	Cd37		2304	3	3	2	14.2	14.2	10	31.852	0.0000	25.986	3606700	38	13.61	13.81	12.81	15.26	13.78	13.83	12.59	13.92
0.581	0.34	1.26	Q80X95	Ras-related GTP-binding protein A	Rraga		2877	15	15	6	52.4	52.4	23	36.566	0.0000	117.78	26790000	250	16.57	16.45	15.59	16.39	15.98	15.65	16.33	15.70
0.121	0.34	1.26	Q9ESJ0	Exportin-4	Xpo4		5337	4	4	4	5.9	5.9	5.9	129.98	0.0000	16.175	4120400	48	14.66	12.47	16.88	14.01	13.59	14.48	15.39	13.23
0.302	0.33	1.26	Q9D0D5	General transcription factor IIE subunit 1	Gtf2e1		4860	5	5	5	17.3	17.3	17.3	49.593	0.0000	77.531	2494700	39	14.04	13.51	15.04	14.47	13.06	14.52	13.75	14.39
0.440	0.33	1.26	G5E829	Plasma membrane calcium-transporting ATPase 1	Atp2b1		262	43	43	30	38.2	38.2	26.2	134.75	0.0000	323.31	174300000	976	17.96	17.69	19.07	17.90	17.47	18.12	17.95	17.74
0.808	0.33	1.26	Q5M8N0	CB1 cannabinoid receptor-interacting protein 1	Cnrip1		2113	5	5	5	53.7	53.7	53.7	18.612	0.0000	49.026	2617400	29	14.02	14.08	14.04	14.07	14.30	13.35	13.53	13.70
0.527	0.33	1.26	P01901	H-2 class I histocompatibility antigen, K-B alpha chain	H2-K1		622	17	15	5	42.5	39.8	15.2	41.301	0.0000	88.172	472210000	349	22.29	22.10	22.77	22.78	22.46	21.45	22.35	22.37
0.206	0.33	1.26	Q921L3	Transmembrane and coiled-coil domain-containing protein 1	Tmco1		4230	8	8	8	31.9	31.9	31.9	21.175	0.0000	25.357	24678000	104	16.33	16.84	18.89	16.30	16.54	17.35	16.63	16.51
0.485	0.33	1.26	Q8BGB5	LIM domain-containing protein 2	Limd2		2973	10	9	9	59.4	53.9	53.9	14.237	0.0000	217.92	53571000	280	18.51	18.04	19.47	18.88	18.47	18.20	18.60	18.29
0.616	0.33	1.26	P21958	Antigen peptide transporter 1	Tap1		870	17	17	17	34.5	34.5	34.5	78.863	0.0000	323.31	70294000	326	17.32	16.55	17.10	17.26	16.60	16.29	17.18	16.83
0.234	0.33	1.26	Q9CQW9	Interferon-induced transmembrane protein 3	Ifitm3		4644	6	6	5	48.9	48.9	43.1	14.954	0.0000	205.56	151520000	393	18.62	17.92	20.33	19.66	18.76	18.41	19.38	18.66
0.681	0.33	1.26	O88643	Serine/threonine-protein kinase PAK 1	Pak1		553	15	9	8	31.9	22.2	20.6	60.736	0.0000	67.659	7336600	63	15.17	15.07	15.27	15.65	14.44	15.38	14.89	15.11
0.604	0.33	1.26	Q99N95	39S ribosomal protein L3, mitochondrial	Mrpl3		4493	8	8	8	43.1	43.1	43.1	39.109	0.0000	39.939	10014000	129	15.15	14.83	15.78	15.04	14.59	15.05	15.22	14.61
0.850	0.33	1.26	A2A8Z1	Oxysterol-binding protein-related protein 9	Osbpl9		13	17	17	17	26.8	26.8	26.8	83.108	0.0000	56.744	25486000	198	16.16	15.75	16.53	16.28	15.84	15.72	16.16	15.69
0.811	0.33	1.26	P56382	ATP synthase subunit epsilon, mitochondrial	Atp5e		1283	8	8	8	78.8	78.8	78.8	5.8378	0.0000	12.971	41423000	183	17.89	17.51	18.37	18.02	17.76	17.77	17.60	17.34
0.846	0.33	1.26	Q9D0L8	mRNA cap guanine-N7 methyltransferase	Rnmt		4876	21	21	21	48.6	48.6	48.6	53.291	0.0000	215.79	36940000	347	16.97	16.51	16.82	16.83	16.44	16.11	16.92	16.34
1.009	0.33	1.26	Q9EP89	Serine beta-lactamase-like protein LACTB, mitochondrial	Lactb		5247	20	20	20	40.1	40.1	40.1	60.705	0.0000	154.38	34815000	348	16.26	15.95	15.65	16.03	15.95	15.58	15.64	15.40
0.427	0.33	1.26	Q91YT0	NADH dehydrogenase [ubiquinone] flavoprotein 1, mitochondrial	Ndufv1		4178	21	21	21	61.4	61.4	61.4	50.834	0.0000	81.645	42011000	282	15.99	15.94	16.56	16.42	16.23	15.02	16.39	15.94
0.555	0.33	1.26	Q9Z1E4	Glycogen [starch] synthase, muscle	Gys1		5805	15	15	15	28.9	28.9	28.9	83.926	0.0000	48.787	9537300	90	14.19	14.15	15.18	14.24	14.00	13.89	14.47	14.08
0.232	0.33	1.26	Q9CQM5	Thioredoxin domain-containing protein 17	Txndc17		4612	4	4	4	28.5	28.5	28.5	14.015	0.0000	46.096	4606500	62	15.19	15.74	13.90	16.00	15.40	14.31	14.30	15.50
0.180	0.33	1.26	P35737	Class II histocompatibility antigen, M beta 1 chain	H2-DMb1		1030	2	2	2	9.2	9.2	9.2	28.899	0.0000	6.9792	4474900	30	14.96	14.77	13.81	14.89	15.28	12.77	15.49	13.58
0.624	0.33	1.26	P97492	Regulator of G-protein signaling 14	Rgs14		1662	11	11	11	32.2	32.2	32.2	59.846	0.0000	65.746	10090000	131	14.82	14.96	15.75	15.25	14.58	14.82	14.78	15.27
0.737	0.33	1.26	Q8CF89	TGF-beta-activated kinase 1 and MAP3K7-binding protein 1	Tab1		3476	7	7	7	21.1	21.1	21.1	54.616	0.0000	7.6827	3301400	23	14.47	14.52	14.32	14.60	14.05	14.70	14.15	13.68
1.012	0.33	1.26	Q9DB73	NADH-cytochrome b5 reductase 1	Cyb5r1		5130	14	14	14	46.9	46.9	46.9	34.134	0.0000	104.03	33915000	256	16.92	16.41	16.32	16.88	16.13	16.38	16.29	16.40
0.452	0.33	1.26	Q7TQK5	Coiled-coil domain-containing protein 93	Ccdc93		2778	34	34	34	53.6	53.6	53.6	72.602	0.0000	187.31	61938000	604	16.28	16.05	17.53	16.61	16.27	16.35	16.33	16.22
0.714	0.33	1.26	Q5SSZ5	Tensin-3	Tns3		2141	40	40	37	48	48	46.2	155.59	0.0000	323.31	109350000	758	17.31	16.79	17.69	17.37	16.91	17.07	17.22	16.64
0.301	0.33	1.26	Q60520	Paired amphipathic helix protein Sin3a	Sin3a		2178	24	24	24	20.2	20.2	20.2	145.09	0.0000	95.532	18822000	149	15.86	15.59	17.34	15.71	15.27	16.24	15.76	15.92
0.162	0.33	1.25	Q8CBA2	Schlafen family member 5	Slfn5		3435	30	30	30	47.1	47.1	47.1	100.82	0.0000	323.31	66655000	425	16.64	15.51	17.86	17.53	16.57	15.00	17.71	16.96
1.672	0.33	1.25	O09172	Glutamate--cysteine ligase regulatory subunit	Gclm		330	14	14	14	45.3	45.3	45.3	30.534	0.0000	323.31	157180000	533	18.74	18.89	18.59	18.56	18.44	18.54	18.26	18.24
2.435	0.33	1.25	P14901	Heme oxygenase 1	Hmox1		788	22	22	22	70.6	70.6	70.6	32.928	0.0000	192.86	128920000	472	18.34	18.24	18.32	18.15	17.95	18.09	17.87	17.84
0.305	0.33	1.25	Q80YV3	Transformation/transcription domain-associated protein	Trrap		2908	5	5	5	3	3	3	291.55	0.0000	14.262	3990700	55	14.44	14.07	14.92	14.70	13.23	14.42	15.19	13.98
0.240	0.33	1.25	Q921C5	Protein bicaudal D homolog 2	Bicd2		4217	8	8	8	12.8	12.8	12.8	93.39	0.0000	42.957	7765000	89	14.78	14.87	16.84	14.89	14.57	14.69	15.29	15.53
0.668	0.32	1.25	Q8C650	Septin-10		40422	3403	7	5	5	14.6	13.1	13.1	52.422	0.0000	16.329	6000300	72	14.98	14.48	14.73	15.16	13.99	14.65	14.83	14.57
0.183	0.32	1.25	Q8BPM0	Disheveled-associated activator of morphogenesis 1	Daam1		3165	13	13	12	16.9	16.9	15.9	123.37	0.0000	28.818	8284300	77	14.24	13.31	16.36	15.13	13.97	14.23	15.08	14.47
0.230	0.32	1.25	Q9WUR9	Adenylate kinase 4, mitochondrial	Ak4		5722	8	8	8	56.5	56.5	56.5	25.061	0.0000	17.166	6011500	64	13.75	13.96	13.14	14.62	14.18	12.70	14.56	12.74
0.213	0.32	1.25	Q8R5G7	Arf-GAP with Rho-GAP domain, ANK repeat and PH domain-containing	Arap3		3888	14	14	14	14.8	14.8	14.8	169.74	0.0000	56.643	9226600	69	15.30	15.56	14.99	15.61	15.45	15.27	16.09	13.35
1.022	0.32	1.25	Q921M3	Splicing factor 3B subunit 3	Sf3b3		4232	40	40	40	42.3	42.3	42.3	135.55	0.0000	271.78	95076000	523	17.19	17.43	17.85	17.27	16.96	17.29	17.14	17.05
0.515	0.32	1.25	Q8K2C9	Very-long-chain (3R)-3-hydroxyacyl-CoA dehydratase 3	Hacd3		3656	5	5	5	17.7	17.7	17.7	43.131	0.0000	46.94	28758000	83	17.64	17.35	17.43	17.08	16.88	16.89	17.82	16.61
0.213	0.32	1.25	Q69Z37	Sterile alpha motif domain-containing protein 9-like	Samd9l		2478	26	26	25	19.6	19.6	19	180.2	0.0000	85.874	14810000	105	14.20	14.40	16.18	15.23	14.00	14.04	15.67	15.00
0.185	0.32	1.25	Q3TJD7	PDZ and LIM domain protein 7	Pdlim7		1853	7	7	7	23.4	23.4	23.4	50.118	0.0000	22.286	6386500	70	14.47	14.53	16.25	13.01	13.77	14.28	14.38	14.53
1.331	0.32	1.25	P97429	Annexin A4	Anxa4		1648	39	39	38	87.5	87.5	87.5	35.915	0.0000	323.31	1054500000	1736	20.63	20.47	20.79	20.67	20.37	20.52	20.00	20.37
0.529	0.32	1.25	Q8K0V4	CCR4-NOT transcription complex subunit 3	Cnot3		3610	8	8	7	11.3	11.3	11.3	81.945	0.0000	13.394	7474900	72	14.72	14.49	15.45	14.61	14.32	14.85	14.74	14.09
0.343	0.32	1.25	P97434	Myosin phosphatase Rho-interacting protein	Mprip		1649	8	8	7	10.2	10.2	9.3	116.41	0.0000	12.553	4320500	46	14.39	14.01	14.72	14.53	13.13	13.89	14.58	14.77
0.642	0.32	1.25	Q9DC69	NADH dehydrogenase [ubiquinone] 1 alpha subcomplex subunit 9, mito	Ndufa9		5199	23	23	23	66	66	66	42.525	0.0000	244.89	51771000	420	17.07	16.63	17.69	17.25	16.75	16.62	17.00	16.98
0.511	0.32	1.25	Q8CI75	DIS3-like exonuclease 2	Dis3l2		3544	16	16	16	28.2	28.2	28.2	97.774	0.0000	68.658	16997000	181	16.04	15.63	16.83	15.99	15.66	15.49	16.11	15.95
0.570	0.32	1.25	Q6ZQA0	Neurobeachin-like protein 2	Nbeal2		2693	23	23	23	14.2	14.2	14.2	301.92	0.0000	101.09	13006000	181	14.93	14.55	15.22	14.92	14.44	14.48	15.23	14.19
0.716	0.32	1.25	Q8R180	ERO1-like protein alpha	Ero1l		3781	11	11	11	31.7	31.7	31.7	54.084	0.0000	105.75	19300000	141	16.56	17.04	17.24	16.73	16.73	16.11	16.69	16.76
0.199	0.32	1.25	Q7TNP2	Serine/threonine-protein phosphatase 2A 65 kDa regulatory subunit A b	Ppp2r1b		2763	22	12	11	49.1	34.6	28.1	65.934	0.0000	56.292	12099000	125	15.20	14.80	16.15	15.08	14.88	13.48	16.16	15.42
0.552	0.32	1.25	E9Q4N7		Arid1b		234	12	9	9	8.4	6.6	6.6	237.79	0.0000	136.6	10492000	137	15.53	14.95	14.82	15.64	14.40	15.08	15.18	15.00
0.151	0.32	1.25	Q8K385	Ferric-chelate reductase 1	FRRS1		3695	3	3	3	6.4	6.4	6.4	66.047	0.0000	7.675	4982800	56	15.49	15.55	14.14	15.19	15.33	12.60	15.92	15.22
0.335	0.32	1.25	Q8K273	Membrane magnesium transporter 1	Mmgt1		3646	3	3	3	42.7	42.7	42.7	14.677	0.0000	74.281	11132000	66	15.63	15.94	15.38	15.39	15.86	14.22	15.84	15.13
0.446	0.32	1.25	Q8R349	Cell division cycle protein 16 homolog	Cdc16		3836	5	5	5	9.4	9.4	9.4	71.459	0.0000	10.569	2712000	30	14.30	13.46	13.11	13.78	12.94	13.13	13.48	13.83
0.150	0.32	1.25	Q62073	Mitogen-activated protein kinase kinase kinase 7	Map3k7		2350	5	5	5	12.8	12.8	12.8	64.227	0.0000	19.395	1670600	23	14.51	13.02	15.54	13.18	12.32	13.45	14.75	14.46
0.532	0.32	1.25	O35382	Exocyst complex component 4	Exoc4		366	23	23	23	32.5	32.5	32.5	110.54	0.0000	189.12	31477000	303	15.98	15.75	16.90	15.94	15.58	16.01	15.98	15.72
0.370	0.32	1.25	Q60710	Deoxynucleoside triphosphate triphosphohydrolase SAMHD1	Samhd1		2196	51	51	51	68.7	68.7	68.7	75.892	0.0000	323.31	259260000	1353	17.88	17.45	19.08	18.37	17.92	17.53	18.19	17.87
0.325	0.32	1.25	Q8BFP9	[Pyruvate dehydrogenase (acetyl-transferring)] kinase isozyme 1, mitocl	Pdk1		2941	7	7	7	23.5	23.5	23.5	48.994	0.0000	31.795	10527000	106	15.20	14.80	14.91	15.48	14.82	13.67	15.24	15.37
0.415	0.32	1.25	Q60715	Prolyl 4-hydroxylase subunit alpha-1	P4ha1		2198	10	10	10	24.2	24.2	24.2	60.909	0.0000	49.83	7714000	76	15.17	14.49	15.47	15.63	14.62	14.40	15.06	15.42
1.767	0.32	1.25	P70318	Nucleolysin TIAR	Tial1		1553	15	15	12	46.7	46.7	38	43.388	0.0000	218.76	49091000	309	18.12	18.04	18.19	18.40	17.70	17.88	17.95	17.94
0.310	0.32	1.25	Q99M04	Lipoyl synthase, mitochondrial	Lias		4451	4	4	4	19	19	19	41.879	0.0000	11.531	2920000	25	14.20	13.68	13.47	14.17	14.21	13.34	14.15	12.55
0.359	0.32	1.25	Q3TMX7	Sulfhydryl oxidase 2	Qsox2		1863	7	7	7	15.3	15.3	15.3	77.774	0.0000	40.503	2543000	22	12.83	12.63	14.07	12.81	13.00	13.17	12.35	12.55
0.396	0.32	1.25	P15105	Glutamine synthetase	Glul		790	8	8	8	23.1	23.1	23.1	42.119	0.0000	73.774	6942200	61	15.18	14.68	16.33	15.43	14.99	15.16	15.23	14.95
0.597	0.32	1.25	Q8BJL0	SWI/SNF-related matrix-associated actin-dependent regulator of chrom	Smarcal1		3079	4	4	4	6.7	6.7	6.7	100.84	0.0002	5.7276	2768000	46	14.30	14.25	14.63	14.40	14.02	13.44	14.52	14.34
1.217	0.32	1.25	Q60770	Syntaxin-binding protein 3	Stxbp3		2205	30	30	29	54.2	54.2	53	67.942	0.0000	145.37	65371000	595	16.66	16.40	16.93	16.86	16.49	16.19	16.47	16.43
0.280	0.32	1.25	P62915	Transcription initiation factor IIB	Gtf2b		1469	8	8	8	39.2	39.2	39.2	34.819	0.0000	54.781	15509000	111	16.85	16.29	17.45	15.37	15.79	16.11	16.57	16.22
0.653	0.32	1.25	Q6VN19	Ran-binding protein 10	Ranbp10		2663	12	12	12	30.2	30.2	30.2	67.188	0.0000	113.44	21912000	205	16.10	15.88	16.66	16.45	15.57	16.18	16.22	15.84
0.371	0.32	1.25	Q6A0A9	Constitutive coactivator of PPAR-gamma-like protein 1	FAM120A		2501	31	31	31	36.2	36.2	36.2	121.64	0.0000	323.31	97432000	761	16.63	16.42	17.99	17.01	16.55	17.05	16.69	16.50
0.945	0.32	1.25	Q8C0Z1	Protein ITFG3	Itfg3		3349	6	6	6	17.7	17.7	17.7	60.575	0.0000	16.927	7349700	46	15.13	14.58	14.94	14.47	14.54	14.48	14.58	14.24
0.443	0.32	1.24	Q9JIW9	Ras-related protein Ral-B	Ralb		5408	12	12	7	51.5	51.5	27.2	23.349	0.0000	98.563	76931000	307	17.52	17.32	18.70	18.17	17.50	17.54	17.75	17.65
0.922	0.32	1.24	Q9DBS1	Transmembrane protein 43	Tmem43		5173	24	24	24	63.5	63.5	63.5	44.783	0.0000	323.31	124620000	675	17.53	17.26	17.95	17.75	17.14	17.16	17.48	17.45
0.338	0.32	1.24	Q9Z1J3	Cysteine desulfurase, mitochondrial	Nfs1		5809	16	16	15	48.8	48.8	46.2	50.569	0.0000	111.75	30472000	230	16.16	15.81	17.40	15.81	15.74	16.14	15.77	16.27
1.027	0.32	1.24	Q9QUN7	Toll-like receptor 2	Tlr2		5521	26	26	26	32.3	32.3	32.3	89.448	0.0000	165.23	78889000	508	17.44	16.96	17.55	17.40	17.05	16.88	17.27	16.89
0.354	0.31	1.24	Q8K2V1	Serine/threonine-protein phosphatase 4 regulatory subunit 1	Ppp4r1		3674	13	13	13	17.8	17.8	17.8	106.3	0.0000	31.539	21106000	195	16.43	16.13	17.62	16.76	16.02	16.18	16.94	16.54
0.711	0.31	1.24	Q80UM7	Mannosyl-oligosaccharide glucosidase	Mogs		2833	22	22	22	34.1	34.1	34.1	91.83	0.0000	158.53	31568000	300	16.22	15.96	16.62	16.26	15.60	16.09	16.35	15.77
0.245	0.31	1.24	Q8BVI5	Syntaxin-16	Stx16		3242	5	5	5	19.9	19.9	19.9	37.08	0.0000	135.99	5617000	73	14.87	14.52	16.53	14.91	15.54	14.79	14.25	14.99
0.355	0.31	1.24	P47713	Cytosolic phospholipase A2;Phospholipase A2;Lysophospholipase	Pla2g4a		1116	16	16	15	28.9	28.9	28.7	85.221	0.0000	80.446	20513000	173	15.42	16.01	15.59	16.47	15.79	15.59	16.13	14.72
1.352	0.31	1.24	O09061	Proteasome subunit beta type-1	Psmb1		321	16	16	15	73.8	73.8	62.1	26.372	0.0000	323.31	222310000	578	19.60	19.24	19.55	19.50	19.16	19.09	19.42	18.97
0.295	0.31	1.24	O89051	Integral membrane protein 2B;BRI2, membrane form;BRI2 intracellular	Itm2b		598	5	5	5	18.8	18.8	18.8	30.26	0.0000	15.702	7780200	69	15.23	15.50	16.73	15.39	15.80	14.64	15.84	15.32
0.216	0.31	1.24	Q62245	Son of sevenless homolog 1	Sos1		2372	7	7	5	8.4	8.4	6.1	150.88	0.0000	15.858	5518500	19	15.02	14.03	13.40	14.68	13.85	13.76	15.22	13.04
0.471	0.31	1.24	Q9DBG7	Signal recognition particle receptor subunit alpha	Srpr		5154	22	22	22	45	45	45	69.622	0.0000	245.63	50959000	505	16.40	16.28	17.57	16.72	16.45	16.24	16.41	16.62
0.960	0.31	1.24	P56399	Ubiquitin carboxyl-terminal hydrolase 5	Usp5		1286	48	48	48	66.1	66.1	66.1	95.832	0.0000	323.31	300370000	1217	19.33	19.10	19.81	19.37	19.01	18.98	19.31	19.06
0.946	0.31	1.24	Q80U38	Protein KHNYN	Khnyn		2817	5	5	5	12.2	12.2	12.2	74.564	0.0000	27.192	3575300	89	14.43	14.44	15.08	14.76	14.17	14.46	14.44	14.39
0.215	0.31	1.24	Q3TZZ7	Extended synaptotagmin-2	Esyt2		1893	24	24	24	38.1	38.1	38.1	94.138	0.0000	146.15	34612000	261	15.64	15.54	13.45	15.81	15.16	14.69	14.93	14.43
1.664	0.31	1.24	Q99PT1	Rho GDP-dissociation inhibitor 1	Arhgdia		4516	24	24	24	94.6	94.6	94.6	23.407	0.0000	323.31	1174400000	1400	21.26	21.16	21.32	21.10	20.99	20.83	21.09	20.69
0.325	0.31	1.24	E9Q5K9	YTH domain-containing protein 1	Ythdc1		239	9	9	9	12.4	12.4	12.4	85.648	0.0000	22.561	10081000	91	14.95	14.36	15.55	15.27	13.84	14.72	15.06	15.28
0.537	0.31	1.24	Q9QZB7	Actin-related protein 10	Actr10		5589	17	17	17	52.5	52.5	52.5	46.207	0.0000	33.536	24839000	141	15.94	15.82	16.90	15.93	15.78	15.81	16.10	15.66
0.494	0.31	1.24	P59997	Lysine-specific demethylase 2A	Kdm2a		1347	2	2	2	2.8	2.8	2.8	132.68	0.0000	7.0915	1995600	43	13.68	13.88	14.15	14.46	13.79	14.36	13.45	13.33
0.469	0.31	1.24	P36993	Protein phosphatase 1B	Ppm1b		1045	12	12	9	33.8	33.8	25.9	42.795	0.0000	20.801	8725800	95	15.40	15.45	14.76	15.88	15.22	14.57	15.47	14.98
1.834	0.31	1.24	Q9EP69	Phosphatidylinositide phosphatase SAC1	Sacm1l		5243	30	30	30	54.9	54.9	54.9	66.943	0.0000	323.31	106720000	721	17.43	17.41	17.58	17.41	17.25	17.23	17.20	16.90
1.425	0.31	1.24	Q8C6G8	WD repeat-containing protein 26	Wdr26		3405	15	15	15	37.3	37.3	37.3	70.543	0.0000	85.981	26713000	188	16.21	16.09	16.44	16.29	15.70	16.07	16.10	15.92

0.677	0.31	1.24	O88712	C-terminal-binding protein 1	Ctbp1	562	17	17	11	42	42	34.5	47.744	0.0000	119.89	42133000	312	16.73	16.62	17.46	17.09	16.39	16.93	16.64	16.70
0.326	0.31	1.24	O54833	Casein kinase II subunit alpha	Csnk2a2	428	23	23	23	64.3	64.3	64.3	41.215	0.0000	225.34	44896000	393	16.67	16.40	17.05	16.59	16.92	16.89	16.35	15.30
0.901	0.31	1.24	Q8R010	Aminoacyl tRNA synthase complex-interacting multifunctional protein 2	Aimp2	3742	11	11	11	54.1	54.1	54.1	35.377	0.0000	323.31	43628000	272	17.66	17.20	17.24	17.65	16.98	17.07	17.49	16.99
1.027	0.31	1.24	P48036	Annexin A5	Anxa5	1140	51	51	29	86.2	86.2	52.4	35.752	0.0000	323.31	2107300000	2678	21.57	21.61	21.84	21.44	21.28	20.96	21.56	21.43
0.498	0.31	1.24	Q8CIE6	Coatomer subunit alpha;Xenin;Proxenin	Copa	3550	73	73	73	61.8	61.8	61.8	138.43	0.0000	323.31	412070000	1819	19.02	18.73	20.03	19.11	18.91	18.96	18.98	18.81
1.446	0.31	1.24	P00493	Hypoxanthine-guanine phosphoribosyltransferase	Hprt1	612	20	20	20	71.1	71.1	71.1	24.57	0.0000	246.82	97595000	719	18.20	18.22	18.60	18.09	17.92	18.03	17.96	17.97
1.100	0.31	1.24	P35585	AP-1 complex subunit mu-1	Ap1m1	1027	21	21	21	55.1	55.1	55.1	48.542	0.0000	162.01	71593000	410	17.06	17.03	17.24	16.91	16.81	16.58	17.09	16.53
1.434	0.31	1.24	Q8VC28	Aldo-keto reductase family 1 member C13	Akr1c13	3906	21	21	21	77.4	77.4	77.4	37.057	0.0000	186.86	56711000	444	17.17	16.99	17.16	17.13	16.79	17.02	16.88	16.52
0.829	0.31	1.24	Q921S7	39S ribosomal protein L37, mitochondrial	Mrpl37	4239	16	16	16	45.9	45.9	45.9	48.34	0.0000	63.049	16108000	134	15.01	14.64	15.23	15.28	14.91	14.58	14.94	14.49
1.062	0.31	1.24	P80314	T-complex protein 1 subunit beta	Cct2	1591	62	62	62	91	91	91	57.477	0.0000	323.31	655340000	2289	19.60	19.36	20.03	19.60	19.21	19.47	19.39	19.29
0.319	0.31	1.24	Q8K0D5	Elongation factor G, mitochondrial	Gfm1	3604	19	19	19	36.2	36.2	36.2	83.549	0.0000	87.59	28322000	203	16.05	16.25	17.47	15.70	16.33	15.85	15.82	16.25
0.225	0.31	1.24	Q8CD92	Tetratricopeptide repeat protein 27	Ttc27	3457	6	6	6	9.9	9.9	9.9	96.43	0.0000	21.171	1991200	15	13.04	13.99	15.17	14.52	14.14	13.80	14.50	13.06
1.425	0.31	1.24	Q9CZ30	Obg-like ATPase 1	Ola1	4816	21	21	21	59.6	59.6	59.6	44.729	0.0000	215.42	76849000	459	17.55	17.59	18.01	17.66	17.36	17.40	17.52	17.30
0.991	0.31	1.24	Q9WUA3	ATP-dependent 6-phosphofructokinase, platelet type	Pfkp	5703	45	45	41	58.3	58.3	55.2	85.454	0.0000	323.31	273890000	1425	18.89	18.89	18.61	19.24	18.53	18.62	18.85	18.41
1.431	0.30	1.23	P68404	Protein kinase C beta type	Prkcb	1520	24	24	24	42.6	42.6	42.6	76.75	0.0000	66.789	56240000	390	17.39	17.01	17.13	17.40	16.89	16.95	17.09	16.79
0.967	0.30	1.23	P61087	Ubiquitin-conjugating enzyme E2 K	Ube2k	1370	17	17	17	79.5	79.5	79.5	22.406	0.0000	93.675	39018000	283	16.65	16.38	16.92	16.78	16.39	16.44	16.08	16.62
0.251	0.30	1.23	Q9WUD1	STIP1 homology and U box-containing protein 1	Stub1	5708	9	9	9	35.5	35.5	35.5	34.909	0.0000	12.108	8694900	53	15.12	14.84	16.74	15.20	14.89	14.72	15.80	15.29
0.407	0.30	1.23	B2RXS4	Plexin-B2	Plxnb2	86	48	48	47	30.8	30.8	30.2	206.23	0.0000	323.31	141470000	833	17.71	17.37	18.79	18.02	17.77	17.45	17.96	17.50
0.100	0.30	1.23	Q9JIG8	PRA1 family protein 2	Praf2	5399	3	3	3	21.3	21.3	21.3	19.478	0.0000	26.795	7546900	52	13.36	15.63	16.96	14.62	16.00	12.88	16.29	14.20
0.543	0.30	1.23	P23506	Protein-L-isoaspartate(D-aspartate) O-methyltransferase	Pcmt1	885	16	16	16	73.1	73.1	73.1	24.634	0.0000	309.04	187150000	676	19.25	19.68	20.23	19.32	19.61	19.26	19.40	19.01
1.820	0.30	1.23	Q61599	Rho GDP-dissociation inhibitor 2	Arhgdib	2318	22	22	22	93.5	93.5	93.5	22.851	0.0000	323.31	589710000	837	21.71	21.58	21.65	21.83	21.27	21.39	21.59	21.31
0.385	0.30	1.23	P47758	Signal recognition particle receptor subunit beta	Srprb	1122	16	16	16	62.8	62.8	62.8	29.579	0.0000	218.49	55836000	391	16.59	16.45	17.88	16.99	16.41	16.93	16.74	16.63
0.609	0.30	1.23	Q9D1I2	Bcl10-interacting CARD protein		4917	11	11	11	63.9	63.9	63.9	20.936	0.0000	21.15	11018000	92	15.94	15.84	16.61	16.35	16.07	16.15	15.83	15.49
0.270	0.30	1.23	Q8BX10	Serine/threonine-protein phosphatase PGAM5, mitochondrial	Pgam5	3276	7	7	7	25	25	25	31.994	0.0000	21.2	12161000	121	16.00	14.58	16.34	16.45	15.36	15.62	15.26	15.93
0.191	0.30	1.23	Q9Z1M4	Ribosomal protein S6 kinase beta-2	Rps6kb2	5813	4	4	4	8.7	8.7	8.7	53.538	0.0000	8.4627	1722200	28	12.89	14.07	14.33	13.44	12.51	13.60	12.64	14.78
0.653	0.30	1.23	Q8CI95	Oxysterol-binding protein-related protein 11	Osbpl11	3546	25	25	25	40.2	40.2	40.2	83.628	0.0000	123.93	37356000	315	16.60	16.24	17.22	16.72	16.32	16.58	16.48	16.19
0.865	0.30	1.23	Q9D0L7	Armadillo repeat-containing protein 10	Armc10	4875	10	10	10	40.2	40.2	40.2	33.31	0.0000	212.97	46769000	276	17.33	17.10	17.64	17.13	17.08	17.15	17.14	16.64
0.305	0.30	1.23	Q80U28	MAP kinase-activating death domain protein	Madd	2816	14	14	14	13.8	13.8	13.8	175.18	0.0000	87.9	13352000	133	14.65	14.79	15.86	14.83	14.17	15.27	15.25	14.24
0.917	0.30	1.23	Q9QVP9	Protein-tyrosine kinase 2-beta	Ptk2b	5524	53	53	52	61.3	61.3	60.6	115.79	0.0000	323.31	162910000	1227	17.73	17.32	18.05	17.73	17.25	17.43	17.60	17.37
0.145	0.30	1.23	Q9EPK6	Nucleotide exchange factor SIL1	Sil1	5255	4	4	4	9.9	9.9	9.9	52.429	0.0004	4.4515	4727300	22	12.98	15.12	15.19	15.40	12.75	14.98	15.01	14.75
0.203	0.30	1.23	P22907	Porphobilinogen deaminase	Hmbs	878	13	13	13	45.2	45.2	45.2	39.344	0.0000	185.73	28841000	253	16.50	15.88	17.99	16.63	16.05	15.60	17.01	17.14
0.923	0.30	1.23	O88958	Glucosamine-6-phosphate isomerase 1	Gnpda1	588	22	22	20	88.6	88.6	84.4	32.549	0.0000	323.31	150310000	592	18.62	18.52	18.05	18.39	18.10	18.28	18.22	17.80
0.409	0.30	1.23	Q91Z53	Glyoxylate reductase/hydroxypyruvate reductase	Grhpr	4187	8	8	8	40.5	40.5	40.5	35.328	0.0000	53.691	9217700	97	15.06	15.29	16.19	15.31	15.16	15.64	15.20	14.66
1.208	0.30	1.23	Q9JMA1	Ubiquitin carboxyl-terminal hydrolase 14	Usp14	5503	25	25	25	50.3	50.3	50.3	56.001	0.0000	323.31	113090000	589	17.85	17.92	17.36	17.57	17.39	17.34	17.39	17.40
0.303	0.30	1.23	Q8BGX2	Uncharacterized protein C19orf52 homolog		3000	5	5	5	30.5	30.5	30.5	29.415	0.0000	95.396	4579800	66	14.67	14.46	15.38	14.50	14.87	14.12	15.19	13.65
0.478	0.30	1.23	Q3V1V3	ESF1 homolog	Esf1	2050	3	3	3	3.7	3.7	3.7	98.047	0.0000	6.5781	986480	4	13.44	13.19	13.79	12.48	12.94	12.98	12.80	12.99
0.259	0.29	1.23	P43346	Deoxycytidine kinase	Dck	1091	8	8	8	45.8	45.8	45.8	30.367	0.0000	33.115	17768000	127	15.55	15.17	16.56	16.81	15.44	15.38	16.46	15.66
0.465	0.29	1.23	Q8BFR4	N-acetylglucosamine-6-sulfatase	Gns	2944	23	23	23	45.6	45.6	45.6	61.174	0.0000	194.94	101670000	386	18.07	18.28	19.00	18.01	18.28	18.40	17.69	17.82
0.797	0.29	1.23	Q9D3G5	Syntaxin-11	Stx11	4966	8	8	8	36.2	36.2	36.2	33.369	0.0000	27.106	18701000	110	16.46	16.07	16.76	16.50	16.20	16.00	16.46	15.95
0.441	0.29	1.23	Q8VCR7	Alpha/beta hydrolase domain-containing protein 14B	Abhd14b	3930	9	9	9	67.1	67.1	67.1	22.45	0.0000	63.852	14416000	149	16.19	16.07	16.56	15.38	15.97	15.34	16.07	15.64
0.399	0.29	1.23	Q8BLR5	PH and SEC7 domain-containing protein 4	Psd4	3122	5	5	5	7.2	7.2	7.2	112.73	0.0000	38.734	3860000	37	13.91	13.98	14.19	14.16	14.19	12.83	13.93	14.12
0.906	0.29	1.23	Q99020	Heterogeneous nuclear ribonucleoprotein A/B	Hnrnpab	4314	22	22	21	54.4	54.4	54.4	30.831	0.0000	207.81	60887000	411	17.53	17.54	18.00	17.76	17.26	17.62	17.15	17.63
0.329	0.29	1.22	Q9D0F9	Phosphoglucomutase-1	Pgm1	4865	37	37	37	66.4	66.4	66.4	61.417	0.0000	301.81	87643000	767	16.95	16.76	18.33	17.29	16.63	17.15	17.29	17.10
1.259	0.29	1.22	Q9JHW4	Selenocysteine-specific elongation factor	Eefsec	5377	11	11	11	31.4	31.4	31.4	63.538	0.0000	95.786	17864000	154	16.18	16.29	16.50	16.15	15.84	15.84	16.23	16.04
1.320	0.29	1.22	Q8K0C9	GDP-mannose 4,6 dehydratase	Gmds	3603	16	16	16	54.3	54.3	54.3	41.984	0.0000	144.16	27421000	240	16.05	16.17	16.56	16.26	15.88	15.91	16.02	16.08
0.468	0.29	1.22	Q9CQR4	Acyl-coenzyme A thioesterase 13;Acyl-coenzyme A thioesterase 13, N-	Acot13	4625	11	11	11	82.1	82.1	82.1	15.183	0.0000	61.82	42407000	209	17.04	17.48	17.90	17.11	16.87	16.71	17.62	17.17
0.237	0.29	1.22	Q9CY94	DNA replication complex GINS protein PSF3	Gins3	4791	4	4	4	26.9	26.9	26.9	24.576	0.0000	17.542	2487100	39	12.96	14.04	14.59	13.93	13.98	12.61	13.51	14.26
0.327	0.29	1.22	Q8BFS6	Serine/threonine-protein phosphatase CPPED1	Cpped1	2946	7	7	6	31.7	31.7	31.1	35.247	0.0000	47.133	10432000	120	15.06	14.95	16.05	15.65	14.43	15.05	15.33	15.74
0.288	0.29	1.22	Q8C156	Condensin complex subunit 2	Ncaph	3353	7	7	7	13.3	13.3	13.3	82.302	0.0000	24.701	3802200	75	13.98	13.53	14.01	14.32	12.98	14.61	13.99	13.11
0.184	0.29	1.22	A2AGH6	Mediator of RNA polymerase II transcription subunit 12	Med12	20	8	8	8	7.4	7.4	7.4	244.56	0.0000	16.744	3448300	56	13.99	12.62	14.28	14.17	12.17	13.48	13.87	14.38
1.946	0.29	1.22	Q91V76	Ester hydrolase C11orf54 homolog		4049	10	10	10	45.1	45.1	45.1	34.995	0.0000	294.59	38181000	268	16.76	16.74	16.83	16.72	16.41	16.55	16.64	16.30
0.253	0.29	1.22	Q6VNB8	WD repeat and FYVE domain-containing protein 3	Wdfy3	2664	27	27	27	11.1	11.1	11.1	392.33	0.0000	93.661	16395000	161	14.80	14.12	16.29	14.92	14.63	14.69	15.03	14.64
0.614	0.29	1.22	Q9WTP6	Adenylate kinase 2, mitochondrial;Adenylate kinase 2, mitochondrial, N-	Ak2	5681	17	17	17	72	72	72	26.468	0.0000	101.09	44029000	314	16.56	16.51	17.24	16.80	16.21	16.81	16.28	16.67
0.455	0.29	1.22	P10810	Monocyte differentiation antigen CD14	Cd14	729	14	14	14	54.1	54.1	54.1	39.203	0.0000	230.25	80178000	381	18.01	17.98	18.28	18.36	17.36	17.48	18.45	18.20
0.490	0.29	1.22	Q8BH57	WD repeat-containing protein 48	Wdr48	3013	11	11	11	24.7	24.7	24.7	76.006	0.0000	51.712	8446800	67	14.88	14.40	14.80	15.23	14.01	14.79	14.93	14.44
0.295	0.29	1.22	P51855	Glutathione synthetase	Gss	1204	15	15	15	45.1	45.1	45.1	52.246	0.0000	226.48	23121000	130	16.82	16.46	18.03	16.30	16.48	16.74	16.85	16.40
0.266	0.29	1.22	Q9WU40	Inner nuclear membrane protein Man1	Lemd3	5696	17	17	17	27.5	27.5	27.5	100.31	0.0000	125.09	19121000	140	15.62	15.16	16.70	16.08	15.19	16.15	16.07	15.00
0.593	0.28	1.22	P62313	U6 snRNA-associated Sm-like protein LSm6	Lsm6	1421	7	7	7	50	50	50	9.1275	0.0000	10.338	10196000	118	15.34	15.52	14.70	15.48	15.08	14.94	14.65	15.23
1.087	0.28	1.22	O08528	Hexokinase-2	Hk2	267	53	48	48	55.7	53.7	53.7	102.53	0.0000	323.31	153570000	1105	17.47	17.27	17.74	17.64	17.19	17.28	17.47	17.05
0.961	0.28	1.22	Q8BZA9	Fructose-2,6-bisphosphatase TIGAR	Tigar	3312	7	7	7	45.4	45.4	45.4	29.19	0.0000	124.85	21813000	117	16.37	16.41	16.47	16.44	15.78	16.39	16.38	16.00
0.987	0.28	1.22	P28867	Protein kinase C delta type;Protein kinase C delta type regulatory subur	Prkcd	964	51	51	49	67.8	67.8	66.5	77.546	0.0000	222.41	227380000	1018	18.17	18.01	18.13	18.24	17.94	17.61	18.21	17.65
0.906	0.28	1.22	Q9WVA3	Mitotic checkpoint protein BUB3	Bub3	5743	17	17	16	42.9	42.9	42.6	36.954	0.0000	173.92	51604000	310	17.44	17.38	17.75	17.02	17.00	17.12	17.26	17.07
0.169	0.28	1.22	Q99J87	Probable ATP-dependent RNA helicase DHX58	Dhx58	4332	30	30	30	45.7	45.7	45.7	76.708	0.0000	199.07	40493000	345	15.17	15.12	16.95	16.36	15.84	14.24	16.28	16.11
1.071	0.28	1.22	P97384	Annexin A11	Anxa11	1641	35	35	34	48.9	48.9	48.9	54.079	0.0000	323.31	178500000	966	17.99	17.98	17.88	17.90	17.62	17.86	17.86	17.29
0.844	0.28	1.22	Q8BGH2	Sorting and assembly machinery component 50 homolog	Samm50	2983	19	19	19	50.1	50.1	50.1	51.863	0.0000	69.963	39169000	298	16.18	16.26	15.81	16.12	16.10	15.56	15.98	15.61
0.659	0.28	1.22	Q8C147	Dedicator of cytokinesis protein 8	Dock8	3352	69	69	67	38.1	38.1	37.8	238.98	0.0000	323.31	130060000	1155	16.85	16.48	17.29	16.96	16.42	16.60	16.95	16.49
0.534	0.28	1.21	Q9JHW2	Omega-amidase NIT2	Nit2	5376	19	19	19	87.7	87.7	87.7	30.501	0.0000	156.72	40913000	293	16.54	16.46	17.41	16.55	16.38	16.73	16.39	16.32
0.738	0.28	1.21	P11031	Activated RNA polymerase II transcriptional coactivator p15	Sub1	735	6	6	6	42.5	42.5	42.5	14.427	0.0000	60.314	7044600	58	15.33	14.98	15.53	15.54	14.81	15.28	14.87	15.31
0.503	0.28	1.21	Q8BU33	Acetolactate synthase-like protein	Ilvbl	3220	18	18	18	43.2	43.2	43.2	68.155	0.0000	316.56	51536000	402	16.82	16.62	17.31	16.78	16.55	16.67	17.10	16.09
0.740	0.28	1.21	Q9JKR6	Hypoxia up-regulated protein 1	Hyou1	5458	50	50	50	51.4	51.4	51.4	111.18	0.0000	323.31	315180000	1485	19.22	18.86	19.68	19.17	18.87	19.11	19.05	18.79
1.627	0.28	1.21	Q8K411	Presequence protease, mitochondrial	Pitrm1	3711	42	42	42	56.3	56.3	56.3	117.37	0.0000	323.31	125970000	921	17.17	16.96	17.24	17.12	16.73	16.99	16.94	16.72
0.934	0.28	1.21	Q6PB44	Tyrosine-protein phosphatase non-receptor type 23	Ptpn23	2601	33	33	33	25.8	25.8	25.8	185.21	0.0000	146.14	54720000	384	17.05	16.92	17.36	17.21	16.51	17.05	16.94	16.92
0.214	0.28	1.21	Q7JJ13	Bromodomain-containing protein 2	Brd2	2736	10	10	9	19	19	17.4	88.066	0.0000	31.916	11733000	91	15.87	15.14	16.97	15.82	15.99	15.99	14.61	16.09
1.450	0.28	1.21	Q9JKB1;P58	Ubiquitin carboxyl-terminal hydrolase isozyme L3;Ubiquitin carboxyl-terr	Uchl3;Uchl4	5446	8	8	8	42.6	42.6	42.6	26.151	0.0000	33.781	13544000	153	15.93	16.16	16.16	16.07	15.77	15.88	15.57	15.98
0.399	0.28	1.21	Q99P91	Transmembrane glycoprotein NMB	Gpnmb	4504	23	23	23	36.8	36.8	36.8	63.675	0.0000	272.84	1296100000	1154	22.61	22.45	21.44	22.49	22.36	21.96	21.81	21.76
1.015	0.28	1.21	Q8C2Q3	RNA-binding protein 14	Rbm14	3373	26	26	26	44.8	44.8	44.8	69.448	0.0000	202.91	120240000	536	17.49	17.42	17.92	17.83	17.40	17.55	17.23	17.38
0.792	0.28	1.21	Q99KB8	Hydroxyacylglutathione hydrolase, mitochondrial	Hagh	4369	8	8	8	34	34	34	34.084	0.0000	84.058	23330000	189	16.26	16.36	16.92	16.43	16.01	16.25	16.43	16.17
0.266	0.27	1.21	P42859	Huntingtin	Htt	1082	26	26	26	13.5	13.5	13.5	344.69	0.0000	201.48	32102000	245	16.62	16.30	18.13	17.26	16.53	16.82	17.17	16.69
0.401	0.27	1.21	Q6ZPE2	Myotubularin-related protein 5	Sbf1	2674	19	19	19	17.1	17.1	17.1	208.69	0.0000	60.766	24494000	189	16.46	15.79	16.53	16.44	15.97	15.79	16.74	15.63
1.221	0.27	1.21	P70290	55 kDa erythrocyte membrane protein	Mpp1	1547	38	38	38	87.3	87.3	87.3	52.227	0.0000	323.31	225220000	1136	18.46	18.24	18.66	18.65	18.09	18.27	18.39	18.17
0.612	0.27	1.21	Q6NVF0	Inositol polyphosphate 5-phosphatase OCRL-1	Ocrl	2533	7	7	7	11.3	11.3	11.3	104.28	0.0000	29.857	7392300	89	14.64	14.90	14.78	14.87	14.18	14.21	15.05	14.65
0.304	0.27	1.21	O88520	Leucine-rich repeat protein SHOC-2	Shoc2	538	7	7	7	18.9	18.9	18.9	64.892	0.0000	68.84	9360500	124	15.21	14.89	16.55	15.53	15.05	15.46	15.50	15.09
0.836	0.27	1.21	Q8CJG0	Protein argonaute-2	Ago2	3571	20	20	14	29.4	29.4	20.3	97.303	0.0000	54.857	30161000	301	15.98	15.90	16.41	15.76	15.54	15.85	15.91	15.65
0.594	0.27	1.21	Q60855	Receptor-interacting serine/threonine-protein kinase 1	Ripk1	2213	23	23	23	38.7	38.7	38.7	74.854	0.0000	225.3	48928000	389	16.76	16.57	16.99	17.11	16.22	17.08	16.46	16.58
0.655	0.27	1.21	Q9Z1Q9	Valine--tRNA ligase	Vars	5819	61	61	61	59.3	59.3	59.3	140.21	0.0000	323.31	249030000	1361	18.70	18.50	19.36	18.96	18.44	18.52	18.74	18.73
0.487	0.27	1.21	Q9WUK2	Eukaryotic translation initiation factor 4H	Eif4h	5712	13	13	13	53.2	53.2	53.2	27.341	0.0000	93.209	19224000	85	15.76	16.71	16.02	16.45	15.86	16.29	15.64	16.07
0.209	0.27	1.21	P58749	Transmembrane 6 superfamily member 1	Tm6sf1	1322	4	4	4	14.6	14.6	14.6	41.662	0.0000	6.9371	3126100	25	14.79	13.77	15.43	13.21	13.71	14.41	14.01	13.99
0.491	0.27	1.21	B2RWS6	Histone acetyltransferase p300	Ep300	81	9	9	8	4.4	4.4	3.7	263.3	0.0000	16.679	5008600	68	13.95	13.69	14.45	14.01	13.92	13.18	14.07	13.85
0.350	0.27	1.21	O54734	Dolichyl-diphosphooligosaccharide--protein glycosyltransferase 48 kDa	Ddost	418	18	18	18	48.1	48.1	48.1	49.027	0.0000	323.31	150040000	490	18.64	18.73	17.51	18.13	18.40	17.54	17.86	18.12
0.381	0.27	1.21	Q3UW53	Protein Niban	Fam129a	2040	49	49	49	56.4	56.4	56.4	102.65	0.0000	323.31	183880000	824	19.38	18.77	20.03	19.44	18.68	19.09	19.53	19.23
0.541	0.27	1.21	Q8R3C6	Probable RNA-binding protein 19	Rbm19	3841	7	7	7	11.4	11.4	11.4	106.08	0.0000	18.794	5185500	74	14.38	14.30	14.79	14.18	14.41	14.53	13.74	13.89
0.534	0.27	1.21	Q9DBS5	Kinesin light chain 4	Klc4	5175	22	18	17	43.3	38.4	37	68.612	0.0000	87.15	16591000	156	16.47	16.06	15.87	15.83	15.55	16.33	15.57	15.70
0.499	0.27	1.21	Q9ESJ4	NCK-interacting protein with SH3 domain	Nckipsd	5338	10	10	10	16.9	16.9	16.9	78.572	0.0000	18.622	5610400	59	14.74	13.85	14.83	14.58	13.94	14.44	14.33	14.20
0.189	0.27	1.21	Q9JKY0	Cell differentiation protein RCD1 homolog	Rqcd1	5464	6	6	6	26.8	26.8	26.8	33.601	0.0000	72.456	11415000	141	15.75	15.09	17.63	15.73	15.78	15.53	15.75	16.06
0.476	0.27	1.21	Q80VQ0	Aldehyde dehydrogenase family 3 member B1	Aldh3b1	2855	22	22	16	59.6	59.6	42.3	52.292	0.0000	323.31	89522000	448	17.45	17.13	18.24	17.87	17.21	17.43	17.62	17.35
0.473	0.27	1.21	Q7M6Y3	Phosphatidylinositol-binding clathrin assembly protein	Picalm	2737	24	24	22	42.7	42.7	39.7	71.543	0.0000	323.31	101580000	542	18.77	18.61	19.64	19.03	18.51	18.92	19.00	18.55
0.211	0.27	1.21	Q8CAY6	Acetyl-CoA acetyltransferase, cytosolic	Acat2	3432	24	24	24	75.1	75.1	75.1	41.297	0.0000	323.31	103490000	498	17.53	17.25	19.03	18.72	17.27	17.59	18.31	18.28
0.292	0.27	1.21	Q925I1	ATPase family AAA domain-containing protein 3	Atad3	4307	32	32	32	51.4	51.4	51.4	66.741	0.0000	192.53	43267000	423	16.37	15.95	17.72	16.45	16.21	16.31	16.43	16.45
0.242	0.27	1.20	Q9JI78	Peptide-N(4)-(N-acetyl-beta-glucosaminyl)asparagine amidase	Ngly1	5390	5	5	4	14.9	14.9	10	74.274	0.0004	5.112	1617000	11	13.38	13.30	14.26	12.91	12.95	13.08	12.56	14.18
0.533	0.27	1.20	Q9D0L4	Uncharacterized aarF domain-containing protein kinase 1	Adck1	4874	5	5	5	15.4	15.4	15.4	59.735	0.0000	20.503	4915400	74	14.59	13.86	14.86	14.69	14.17	14.42	14.27	14.07
0.108	0.27	1.20	P21460	Cystatin-C	Cst3	865	3	3	3	26.4	26.4	26.4	15.531	0.0000	32.809	6307500	73	15.04	15.04	12.93	15.59	15.30	12.33	15.23	14.67
0.160	0.27	1.20	Q8C2K5	RAS protein activator like-3	Rasal3	3372	13	13	13	18.9	18.9	18.9	114.78	0.0000	45.547	15579000	143	15.41	15.09	17.73	15.47	15.16	15.60	16.17	15.70
0.644	0.27	1.20	P11152	Lipoprotein lipase	Lpl	739	7	7	7	19	19	19	53.109	0.0000	211.62	22688000	204	15.78	15.89	16.26	16.12	15.90	15.27	16.01	15.81
0.366	0.27	1.20	Q8CBE3	WD repeat-containing protein 37	Wdr37	3437	14	14	14	31.7	31.7	31.7	55.045	0.0000	103.88	17229000	158	15.74	14.92	16.34	15.73	15.14	15.61	15.63	15.27
1.490	0.27	1.20	P05202	Aspartate aminotransferase, mitochondrial	Got2	650	36	36	36	73	73	73	47.411	0.0000	323.31	431080000	1054	19.83	19.60	19.53	19.81	19.46	19.45	19.54	19.25
0.150	0.27	1.20	Q8R0F3	Sulfatase-modifying factor 1	Sumf1	3755	3	3	3	12.6	12.6	12.6	40.659	0.0000	8.15	4762200	36	14.88	15.22	13.93	15.48	12.97	14.89	14.83	15.75
0.727	0.27	1.20	O35375	Neuropilin-2	Nrp2	363	10	10	10	13.3	13.3	13.3	104.63	0.0000	56.296	16496000	132	16.23	16.15	15.81	16.15	15.72	15.79	16.24	15.52
0.551	0.27	1.20	Q9D819	Inorganic pyrophosphatase	Ppa1	5046	11	11	11	58.5	58.5	58.5	32.667	0.0000	35.713	17532000	95	15.41	16.11	16.21	16.32	15.66	15.91	15.87	15.54

0.737	0.27	1.20	Q8BPU7	Engulfment and cell motility protein 1	Elmo1	3168	45	45	39	59.4	59.4	55.6	83.935	0.0000	323.31	208650000	1032	18.14	18.03	18.73	18.39	17.87	18.21	18.19	17.95
0.196	0.26	1.20	Q8BIQ5	Cleavage stimulation factor subunit 2	Cstf2	3064	6	6	3	23.1	23.1	12.4	61.341	0.0000	36.868	3296000	42	14.02	14.04	12.53	14.14	13.56	12.34	13.83	13.94
0.247	0.26	1.20	O35130	Ribosomal RNA small subunit methyltransferase NEP1	Emg1	340	4	4	4	22.5	22.5	22.5	26.974	0.0000	9.8384	2589500	27	14.83	12.82	13.97	13.95	14.05	13.47	13.56	13.42
0.115	0.26	1.20	Q922J9	Fatty acyl-CoA reductase 1	Far1	4261	7	7	7	18.3	18.3	18.3	59.434	0.0000	16.113	9329100	58	15.45	12.16	15.27	15.07	14.32	14.76	14.63	13.19
0.729	0.26	1.20	O35166	Golgi SNAP receptor complex member 2	Gosr2	345	14	14	14	50	50	50	24.725	0.0000	119.73	24249000	268	15.66	15.59	16.29	15.82	15.46	15.53	15.81	15.51
0.804	0.26	1.20	Q9D0J4	ADP-ribosylation factor-like protein 2	Arl2	4870	11	11	11	71.7	71.7	71.7	20.864	0.0000	63.544	20119000	191	16.22	15.99	16.21	16.41	16.21	16.15	15.77	15.65
0.596	0.26	1.20	O08795	Glucosidase 2 subunit beta	Prkcsh	300	9	9	9	15.4	15.4	15.4	58.792	0.0000	22.439	24852000	167	17.03	16.93	16.31	17.01	16.87	16.33	16.44	16.59
0.369	0.26	1.20	Q9CZ44	NSFL1 cofactor p47	Nsfl1c	4818	9	9	9	38.1	38.1	38.1	40.709	0.0000	67.787	14392000	120	16.31	16.43	15.40	16.46	15.76	16.40	15.59	15.81
0.343	0.26	1.20	P61079	Ubiquitin-conjugating enzyme E2 D3	Ube2d3	1368	6	6	3	48.3	48.3	12.2	16.687	0.0000	229.02	83862000	238	18.59	18.78	17.80	17.79	18.28	17.41	18.04	18.18
0.119	0.26	1.20	P08508	Low affinity immunoglobulin gamma Fc region receptor III	Fcgr3	685	4	4	2	19.2	19.2	12.3	30.036	0.0000	26.34	11187000	71	16.61	16.50	14.55	14.91	16.51	15.23	16.09	13.70
0.557	0.26	1.20	Q99LD4	COP9 signalosome complex subunit 1	Gps1	4420	27	27	27	58.4	58.4	58.4	53.442	0.0000	120.31	41239000	415	16.78	16.47	16.47	17.34	16.39	16.67	16.59	16.36
0.322	0.26	1.20	Q9EQJ0	Two pore calcium channel protein 1	Tpcn1	5281	4	4	4	6.1	6.1	6.1	94.495	0.0000	7.9797	3736600	27	14.38	14.57	14.14	14.09	14.13	13.09	14.40	14.51
0.240	0.26	1.20	Q32NY4	Metal transporter CNNM3	Cnnm3	1807	5	5	5	12.8	12.8	12.8	76.278	0.0000	18.856	3789700	57	13.94	13.75	14.02	14.41	14.96	13.69	13.38	13.06
0.564	0.26	1.20	P28271	Cytoplasmic aconitate hydratase	Aco1	947	58	58	57	71.9	71.9	71	98.124	0.0000	323.31	178200000	1129	17.63	17.45	18.29	18.10	17.51	17.64	17.85	17.44
0.671	0.26	1.20	Q8BMD8	Calcium-binding mitochondrial carrier protein SCaMC-1	Slc25a24	3134	27	27	27	61.3	61.3	61.3	52.901	0.0000	323.31	175660000	773	18.69	18.41	18.00	18.52	18.20	17.91	18.44	18.04
0.093	0.26	1.20	Q8BW00	Probable peptidyl-tRNA hydrolase	Ptrh1	3252	5	5	5	40.2	40.2	40.2	22.16	0.0000	40.077	8688900	37	15.42	14.97	16.49	15.06	15.49	17.09	15.76	12.57
0.492	0.26	1.20	Q9ES52	Phosphatidylinositol 3,4,5-trisphosphate 5-phosphatase 1	Inpp5d	5328	69	69	67	56.5	56.5	55.7	133.54	0.0000	323.31	218900000	1316	17.87	17.84	18.76	17.91	17.57	17.92	17.98	17.86
0.262	0.26	1.20	Q9QZW0	Phospholipid-transporting ATPase 11C	Atp11c	5609	9	9	9	12.7	12.7	12.7	129.24	0.0000	25.015	4090600	45	14.32	14.22	15.74	14.33	14.19	14.01	14.79	14.57
1.226	0.26	1.20	P46467	Vacuolar protein sorting-associated protein 4B	Vps4b	1105	27	27	21	60.8	60.8	50.2	49.419	0.0000	323.31	106920000	576	17.29	17.12	17.60	17.45	17.03	17.05	17.17	17.19
0.153	0.26	1.20	Q6NWW9	Fibronectin type III domain-containing protein 3B	Fndc3b	2537	8	8	8	10.5	10.5	10.5	132.76	0.0000	38.087	4626500	40	15.24	13.12	14.43	13.35	13.50	14.21	14.65	12.75
0.359	0.26	1.20	Q8BTZ7	Mannose-1-phosphate guanyltransferase beta	Gmppb	3215	10	10	10	39.4	39.4	39.4	39.916	0.0000	75.679	28164000	169	16.41	15.90	16.95	16.79	16.09	16.15	15.93	16.85
0.227	0.26	1.19	Q8K1T1	Leucine-rich repeat-containing protein 25	Lrrc25	3634	7	7	7	32.7	32.7	32.7	32.673	0.0000	151.29	28228000	249	15.84	15.52	17.57	16.50	15.97	16.16	16.04	16.23
0.122	0.26	1.19	Q9JKN1	Zinc transporter 7	Slc30a7	5455	3	3	3	10.8	10.8	10.8	41.789	0.0000	12.115	10562000	97	15.81	15.42	13.43	15.63	15.30	15.18	13.17	15.59
0.145	0.26	1.19	Q99M31	Heat shock 70 kDa protein 14	Hspa14	4455	8	8	8	28.3	28.3	28.3	54.65	0.0000	92.764	7721700	68	15.33	12.97	15.97	15.09	14.18	15.03	14.63	14.50
0.952	0.26	1.19	Q8C3J5	Dedicator of cytokinesis protein 2	Dock2	3376	91	91	90	55.6	55.6	55.1	211.7	0.0000	323.31	305970000	2056	17.60	17.42	17.86	17.96	17.38	17.41	17.65	17.38
0.422	0.26	1.19	P50396	Rab GDP dissociation inhibitor alpha	Gdi1	1179	22	15	14	67.6	51.5	51.5	50.521	0.0000	142.36	21677000	170	15.85	16.41	16.99	16.44	16.01	16.37	16.42	15.86
0.809	0.26	1.19	P17563;Q63	Selenium-binding protein 1;Selenium-binding protein 2	Selenbp1;Selenbp	826	18	18	18	54.9	54.9	54.9	52.513	0.0000	128.85	25034000	225	16.33	16.18	16.76	16.08	16.15	16.03	16.18	15.96
0.419	0.26	1.19	P97377	Cyclin-dependent kinase 2	Cdk2	1639	12	11	9	48.6	46.2	39	38.978	0.0000	54.743	23298000	145	16.48	16.07	16.72	16.47	15.73	16.66	16.50	15.83
0.428	0.26	1.19	Q8BYA0	Tubulin-specific chaperone D	Tbcd	3295	21	21	21	24.8	24.8	24.8	133.32	0.0000	223	38690000	382	16.32	16.21	17.22	16.62	16.34	16.39	16.64	15.97
0.213	0.26	1.19	Q6Y685	Transforming acidic coiled-coil-containing protein 1	Tacc1	2671	4	4	4	7	7	7	83.951	0.0002	5.4664	1589200	36	14.05	13.51	14.90	13.66	12.79	13.96	13.83	14.52
0.301	0.25	1.19	Q6PAM1	Alpha-taxilin	Txlna	2597	18	18	17	41.3	41.3	39.9	62.368	0.0000	69.317	15806000	144	15.50	15.66	15.63	15.18	16.01	14.48	15.54	14.92
0.647	0.25	1.19	P17426	AP-2 complex subunit alpha-1	Ap2a1	822	46	46	38	58.4	58.4	48.5	107.66	0.0000	323.31	169650000	1144	18.21	18.33	18.74	17.99	18.21	17.82	18.26	17.96
0.412	0.25	1.19	Q9ERK4	Exportin-2	Cse1l	5313	26	26	26	31.8	31.8	31.8	110.45	0.0000	323.31	200390000	714	20.56	20.78	21.62	20.93	20.48	21.03	20.91	20.44
0.473	0.25	1.19	Q9JIG7	Coiled-coil domain-containing protein 22	Ccdc22	5398	36	36	36	67.5	67.5	67.5	70.843	0.0000	312.28	79701000	727	16.91	16.56	17.55	16.96	16.48	16.62	16.78	17.08
0.310	0.25	1.19	Q91WJ8	Far upstream element-binding protein 1	Fubp1	4113	18	15	15	37.2	33	33	68.539	0.0000	47.725	23106000	202	15.81	16.13	16.60	15.85	15.60	16.56	15.19	16.03
0.146	0.25	1.19	O35316	Sodium- and chloride-dependent taurine transporter	Slc6a6	356	4	4	4	7.9	7.9	7.9	69.855	0.0000	34.287	26750000	66	17.72	15.11	16.32	15.89	15.29	15.45	16.55	16.73
1.005	0.25	1.19	P04117	Fatty acid-binding protein, adipocyte	Fabp4	636	10	10	10	68.9	68.9	68.9	14.65	0.0000	83.553	50391000	326	17.38	17.02	16.94	17.40	17.05	16.86	16.99	16.84
1.156	0.25	1.19	P63168	Dynein light chain 1, cytoplasmic	Dynll1	1495	10	10	7	74.2	74.2	43.8	10.366	0.0000	191.42	51690000	321	17.48	17.25	17.77	17.55	17.22	17.38	17.25	17.20
0.332	0.25	1.19	Q80X73	Protein pelota homolog	Pelo	2872	11	11	11	33.5	33.5	33.5	43.349	0.0000	35.896	9697500	118	15.22	15.05	15.29	15.29	15.31	15.08	14.03	15.43
0.334	0.25	1.19	A2AQ19	RNA polymerase-associated protein RTF1 homolog	Rtf1	35	10	10	10	13.1	13.1	13.1	80.796	0.0000	18.289	5692900	106	14.24	14.18	15.32	14.22	13.79	14.32	14.29	14.56
0.460	0.25	1.19	P08775	DNA-directed RNA polymerase II subunit RPB1	Polr2a	689	46	46	46	35.7	35.7	35.7	217.17	0.0000	323.31	49219000	523	16.09	15.81	16.84	16.34	15.77	16.16	16.24	15.92
0.751	0.25	1.19	Q3UJU9	Regulator of microtubule dynamics protein 3	Rmdn3	1986	7	7	7	29.4	29.4	29.4	52.028	0.0000	78.21	12698000	184	15.05	14.96	15.39	15.05	15.13	14.84	14.98	14.50
0.428	0.25	1.19	Q8BVL3	Sorting nexin-17	Snx17	3244	16	16	16	43.2	43.2	43.2	52.797	0.0000	96.733	31058000	230	16.27	15.86	16.89	16.16	16.08	15.90	16.43	15.77
0.603	0.25	1.19	Q99KD5	Protein unc-45 homolog A	Unc45a	4371	17	17	17	21.3	21.3	21.3	103.45	0.0000	73.599	19020000	144	15.91	15.64	16.07	16.02	15.57	16.04	15.78	15.24
0.305	0.25	1.19	O89116	Vesicle transport through interaction with t-SNAREs homolog 1A	Vti1a	607	4	4	4	39.2	39.2	39.2	24.986	0.0000	99.06	10923000	124	15.06	15.41	15.86	15.64	15.41	14.40	15.77	15.40
0.282	0.25	1.19	Q8VE70	Programmed cell death protein 10	Pdcd10	3988	10	10	10	45.3	45.3	45.3	24.715	0.0000	262.7	33379000	214	16.47	16.84	17.51	16.49	16.52	17.16	16.78	15.87
0.832	0.25	1.19	Q9DC51	Guanine nucleotide-binding protein G(k) subunit alpha	Gnai3	5194	25	15	14	61.6	40.4	39.3	40.538	0.0000	147.46	32499000	331	16.05	15.93	16.48	16.06	15.73	16.04	16.03	15.73
0.867	0.25	1.19	Q99L45	Eukaryotic translation initiation factor 2 subunit 2	Eif2s2	4408	22	22	21	50.2	50.2	46.2	38.092	0.0000	123.73	75039000	517	16.90	16.72	17.14	17.04	16.63	16.94	16.81	16.43
0.211	0.25	1.19	E9PUQ8	Diacylglycerol kinase	Dgkd	219	2	2	2	2.5	2.5	2.5	135.21	0.0000	7.1612	670660	9	13.89	12.14	13.15	12.80	12.29	13.58	12.55	12.58
0.393	0.25	1.19	Q3UND0	Src kinase-associated phosphoprotein 2	Skap2	2010	18	18	18	52	52	52	40.712	0.0000	174.66	59641000	325	17.22	17.25	18.25	17.30	17.29	17.15	17.03	17.57
0.242	0.25	1.19	Q99KK2	N-acylneuraminate cytidylyltransferase	Cmas	4381	14	14	14	39.4	39.4	39.4	48.057	0.0000	74.031	33483000	199	16.97	16.82	18.36	17.23	17.27	17.65	16.76	16.72
0.307	0.24	1.19	Q9CZX8	40S ribosomal protein S19	Rps19	4846	23	23	23	73.1	73.1	73.1	16.085	0.0000	214.68	389850000	688	19.54	19.28	20.67	19.58	19.16	19.79	19.58	19.55
0.098	0.24	1.18	O55101	Synaptogyrin-2	Syngr2	452	2	2	2	12.9	12.9	12.9	24.778	0.0000	26.998	20013000	37	17.14	17.66	14.12	17.31	16.40	15.41	17.35	16.08
0.229	0.24	1.18	O35114	Lysosome membrane protein 2	Scarb2	337	9	9	9	25.9	25.9	25.9	54.043	0.0000	51.923	23010000	141	17.25	16.67	16.12	16.69	16.38	16.91	17.03	15.43
0.716	0.24	1.18	P52332	Tyrosine-protein kinase JAK1	Jak1	1215	26	26	24	29.2	29.2	27.6	133.37	0.0000	110.76	31170000	307	16.27	15.74	16.44	16.26	15.85	15.83	16.13	15.93
0.908	0.24	1.18	Q9JKC8	AP-3 complex subunit mu-1	Ap3m1	5449	17	17	14	56.9	56.9	47.4	46.936	0.0000	270.7	79185000	461	17.53	17.75	17.75	17.27	17.34	17.19	17.53	17.26
0.288	0.24	1.18	Q8K268	ATP-binding cassette sub-family F member 3	Abcf3	3645	14	14	14	30.7	30.7	30.7	79.864	0.0000	83.039	16371000	166	16.02	15.49	16.73	15.84	15.73	16.24	16.00	15.14
0.401	0.24	1.18	Q91WG4	Elongator complex protein 2	Elp2	4110	4	4	4	8.2	8.2	8.2	93.092	0.0000	17.333	2348300	57	14.08	13.86	13.53	13.93	13.69	13.08	14.23	13.44
0.756	0.24	1.18	P51174	Long-chain specific acyl-CoA dehydrogenase, mitochondrial	Acadl	1192	32	32	32	55.8	55.8	55.8	47.907	0.0000	317.86	255740000	1010	18.98	18.71	18.42	18.91	18.58	18.57	18.66	18.24
0.343	0.24	1.18	Q91W98	Solute carrier family 15 member 4	Slc15a4	4100	5	5	5	8.7	8.7	8.7	62.255	0.0000	32.557	20455000	123	16.25	15.79	16.97	16.57	16.24	15.65	16.40	16.33
0.110	0.24	1.18	Q80XC6	Protein NRDE2 homolog	Nrde2	2880	3	3	3	4.6	4.6	4.6	133.47	0.0000	17.435	2633800	37	14.61	14.53	14.33	12.89	14.49	14.18	14.94	11.78
0.834	0.24	1.18	Q9D1D4	Transmembrane emp24 domain-containing protein 10	Tmed10	4907	19	19	19	46.6	46.6	46.6	24.911	0.0000	172.99	318740000	742	19.18	18.73	18.91	19.16	18.84	18.62	18.98	18.57
0.171	0.24	1.18	Q9Z0J0	Epididymal secretory protein E1	Npc2	5773	2	2	2	25.5	25.5	25.5	16.442	0.0000	26.533	3012600	25	14.16	14.86	12.95	14.97	13.94	13.37	14.75	13.91
0.320	0.24	1.18	Q80UJ7	Rab3 GTPase-activating protein catalytic subunit	Rab3gap1	2829	28	28	28	35.7	35.7	35.7	110.2	0.0000	190.73	51588000	436	17.10	16.56	18.08	17.13	16.89	16.94	17.05	17.03
0.283	0.24	1.18	P97355	Spermine synthase	Sms	1629	10	10	10	54.6	54.6	54.6	41.313	0.0000	171.8	36608000	219	17.69	17.76	17.07	17.79	17.50	17.88	17.51	16.46
0.199	0.24	1.18	Q91X51	Golgi reassembly-stacking protein 1	Gorasp1	4134	2	2	2	8.1	8.1	8.1	46.882	0.0000	9.381	2324600	27	15.13	14.31	13.62	14.79	14.23	13.27	14.52	14.87
0.385	0.24	1.18	Q8CB96	Ras association domain-containing protein 4	Rassf4	3434	13	11	11	49.4	41.3	41.3	36.934	0.0000	28.702	10030000	51	15.70	14.83	15.06	15.88	15.28	14.90	15.31	15.02
0.244	0.24	1.18	Q6PNC0	DmX-like protein 1	Dmxl1	2648	16	16	16	8.8	8.8	8.8	336	0.0000	40.599	7006600	81	14.97	14.85	15.73	14.73	15.07	14.99	15.38	13.88
0.204	0.24	1.18	O55142	60S ribosomal protein L35a	Rpl35a	461	14	14	14	67.3	67.3	67.3	12.554	0.0000	49.023	84327000	275	18.05	18.06	19.83	19.31	18.50	18.80	18.37	18.64
0.440	0.24	1.18	P46664	Adenylosuccinate synthetase isozyme 2	Adss	1109	29	29	26	55.7	55.7	49.3	50.02	0.0000	245.34	182350000	726	18.82	18.73	19.56	18.74	18.55	18.89	19.01	18.45
0.190	0.24	1.18	Q9JIP4	Pannexin-1	Panx1	5405	2	2	2	9.9	9.9	9.9	48.167	0.0000	16.574	871710	20	13.75	13.21	13.56	14.29	12.67	14.49	13.88	12.83
0.090	0.24	1.18	Q8R0X2	CDK2-associated and cullin domain-containing protein 1	Cacul1	3767	5	5	5	23.9	23.9	23.9	42.106	0.0000	29.657	4656000	53	13.35	15.03	14.41	15.20	14.69	11.72	15.30	15.31
0.130	0.24	1.18	Q9D142	Uridine diphosphate glucose pyrophosphatase	Nudt14	4895	3	3	3	15.8	15.8	15.8	24.443	0.0000	7.2144	5325900	50	15.58	15.19	13.73	14.98	14.99	12.99	15.31	15.24
0.222	0.24	1.18	Q91X52	L-xylulose reductase	Dcxr	4135	14	14	14	69.7	69.7	69.7	25.746	0.0000	111.45	57029000	424	17.05	16.60	18.45	17.15	17.10	17.47	17.02	16.72
0.635	0.24	1.18	Q91VN6	Probable ATP-dependent RNA helicase DDX41	Ddx41	4071	14	14	13	30.9	30.9	29.6	69.819	0.0000	68.532	10557000	141	15.02	14.75	14.68	15.23	14.50	14.46	14.98	14.81
0.239	0.24	1.18	Q9ER81	Torsin-1A-interacting protein 2, isoform IFRG15	Tor1aip2	5299	2	2	2	25.2	25.2	25.2	15.278	0.0000	10.757	4492700	66	14.64	14.46	14.71	14.98	15.33	13.65	14.85	14.01
0.897	0.23	1.18	P41241	Tyrosine-protein kinase CSK	Csk	1068	31	31	30	74.2	74.2	72	50.716	0.0000	203.77	97358000	613	17.51	17.33	17.87	17.66	17.21	17.31	17.51	17.41
0.375	0.23	1.18	Q5F2E8	Serine/threonine-protein kinase TAO1	Taok1	2104	21	14	14	20.2	15.8	15.8	116.05	0.0000	36.162	7653200	90	14.49	15.05	15.07	14.35	14.69	13.93	14.59	14.80
0.877	0.23	1.18	Q6ZWX6	Eukaryotic translation initiation factor 2 subunit 1	Eif2s1	2709	30	30	30	93.3	93.3	93.3	36.108	0.0000	323.31	146810000	610	18.84	18.77	19.20	18.86	18.44	18.90	18.71	18.69
0.867	0.23	1.18	Q9JII6	Alcohol dehydrogenase [NADP(+)]	Akr1a1	5402	35	35	35	94.5	94.5	94.5	36.586	0.0000	323.31	1318500000	2017	21.01	20.86	21.12	20.83	20.98	20.83	20.65	20.43
0.351	0.23	1.18	Q6P8X1	Sorting nexin-6;Sorting nexin-6, N-terminally processed	Snx6	2585	33	31	30	69.7	67.7	64	46.648	0.0000	323.31	128150000	845	17.14	16.65	17.98	17.21	16.94	17.12	17.16	16.83
0.196	0.23	1.18	Q8BIH0	Histone deacetylase complex subunit SAP130	Sap130	3060	4	4	4	5	5	5	111.23	0.0029	2.6585	1526100	16	12.63	13.35	14.15	14.07	12.87	12.74	13.64	14.00
0.543	0.23	1.18	Q9D1M0	Protein SEC13 homolog	Sec13	4925	9	9	9	47.8	47.8	47.8	35.565	0.0000	250.91	51084000	186	17.83	18.31	17.82	18.01	17.67	18.13	17.88	17.36
0.269	0.23	1.18	Q64705	Upstream stimulatory factor 2	Usf2	2460	3	3	2	12.4	12.4	9	36.953	0.0002	5.8719	1434900	14	13.08	13.06	13.62	13.96	13.26	13.97	12.75	12.80
0.745	0.23	1.18	Q64378	Peptidyl-prolyl cis-trans isomerase FKBP5;Peptidyl-prolyl cis-trans isom	Fkbp5	2439	18	18	18	42.1	42.1	42.1	50.966	0.0000	23.362	16238000	141	15.78	15.37	15.17	15.19	15.20	15.01	15.08	15.28
0.328	0.23	1.17	Q8BSQ9	Protein polybromo-1	Pbrm1	3191	25	25	25	20.9	20.9	20.9	187.19	0.0000	79.074	16997000	133	15.60	15.15	16.53	15.54	15.34	15.40	15.50	15.67
0.230	0.23	1.17	P19096	Fatty acid synthase;[Acyl-carrier-protein] S-acetyltransferase;[Acyl-carri	Fasn	842	134	134	134	63	63	63	272.43	0.0000	323.31	639710000	3101	18.51	18.19	19.45	19.27	18.19	18.13	19.20	18.98
0.295	0.23	1.17	Q8CDA1	Phosphatidylinositide phosphatase SAC2	Inpp5f	3458	15	15	15	19.1	19.1	19.1	127.61	0.0000	48.151	6188100	67	14.34	13.94	15.33	14.51	14.02	14.72	14.12	14.34
0.501	0.23	1.17	Q9CQF9	Prenylcysteine oxidase	Pcyox1	4597	20	20	20	44.6	44.6	44.6	56.494	0.0000	242.02	64896000	406	17.18	17.22	16.41	17.27	16.89	16.66	16.85	16.75
0.284	0.23	1.17	Q64343	ATP-binding cassette sub-family G member 1	Abcg1	2434	11	11	11	20.9	20.9	20.9	74.032	0.0000	75.184	7237100	65	14.62	14.63	14.35	14.14	13.69	13.72	15.03	14.38
0.133	0.23	1.17	Q7TQK4	Exosome complex component RRP40	Exosc3	2777	7	7	7	36.1	36.1	36.1	29.546	0.0000	77.35	8556600	77	14.78	15.03	17.08	14.19	14.71	15.36	14.71	15.38
0.420	0.23	1.17	Q3TW96	UDP-N-acetylhexosamine pyrophosphorylase-like protein 1	Uap1l1	1880	38	37	36	69.6	69.6	67.1	56.613	0.0000	323.31	481710000	1297	19.30	19.22	20.01	19.63	19.63	19.23	18.88	19.49
0.900	0.23	1.17	Q9CQX2	Cytochrome b5 type B	Cyb5b	4645	9	9	8	70.5	70.5	70.5	16.318	0.0000	239.66	77632000	267	18.12	18.30	18.37	17.83	17.94	17.95	17.80	18.01
0.050	0.23	1.17	Q8CHP5	Partner of Y14 and mago	Wibg	3527	14	14	13	70.4	70.4	70.4	22.689	0.0000	141.08	11253000	95	13.06	15.03	14.63	12.72	16.57	10.85	15.94	11.16
0.120	0.23	1.17	Q60596	DNA repair protein XRCC1	Xrcc1	2181	3	3	3	9.8	9.8	9.8	68.97	0.0000	19.489	4247600	25	13.82	15.28	14.02	14.61	15.08	12.89	15.47	13.36
0.838	0.23	1.17	Q9CZN7	Serine hydroxymethyltransferase	Shmt2	4834	35	35	34	75	75	75	55.758	0.0000	323.31	169470000	893	17.83	17.53	17.46	17.94	17.28	17.42	17.59	17.56
0.362	0.23	1.17	Q569Z6	Thyroid hormone receptor-associated protein 3	Thrap3	2087	21	21	21	22.8	22.8	22.8	108.18	0.0000	53.307	44661000	309	16.79	16.68	17.58	17.21	16.48	16.86	16.69	17.32
0.371	0.23	1.17	A2AF47	Dedicator of cytokinesis protein 11	Dock11	19	43	43	42	25.5	25.5	25	237.77	0.0000	183.54	51875000	528	16.06	15.74	16.89	16.11	15.74	15.89	16.26	15.99
1.123	0.23	1.17	P53810	Phosphatidylinositol transfer protein alpha isoform	Pitpna	1240	29	29	27	86.3	86.3	83	31.893	0.0000	278.85	272300000	878	19.30	18.83	18.99	19.21	18.83	18.84	18.87	18.87
0.310	0.23	1.17	Q8C754	Vacuolar protein sorting-associated protein 52 homolog	Vps52	3409	17	17	17	32.4	32.4	32.4	82.043	0.0000	141.61	31805000	335	16.00	15.55	16.95	15.79	15.86	15.82	15.98	15.74
0.459	0.23	1.17	P55258	Ras-related protein Rab-8A	Rab8a	1270	19	14	14	60.9	51.7	51.7	23.668	0.0000	79.579	61220000	304	17.55	17.33	18.29	17.58	17.51	17.32	17.66	17.34
0.444	0.23	1.17	Q3U186	Probable arginine--tRNA ligase, mitochondrial	Rars2	1900	8	8	8	14.5	14.5	14.5	65.335	0.0000	20.358	6215300	86	14.32	14.38	15.10	14.60	14.28	14.80	14.18	14.22
0.208	0.23	1.17	Q9D666	SUN domain-containing protein 1	Sun1	4991	9	8	8	14.7	13.8	13.8	101.98	0.0000	55.847	6404700	115	14.29	13.86	15.61	14.66	14.21	13.87	14.90	14.55
0.276	0.23	1.17	Q3THG9	Alanyl-tRNA editing protein Aarsd1	Aarsd1	1844	11	11	10	40.8	40.8	39.1	44.97	0.0000	56.784	14381000	147	15.25	15.33	16.66	15.40	15.27	15.39	15.52	15.56
0.349	0.23	1.17	Q99KY4	Cyclin-G-associated kinase	Gak	4400	29	29	28	30.3	30.3	29.1	143.64	0.0000	323.31	55757000	423	16.57	16.16	17.40	16.89	16.32	16.47	16.54	16.78
0.271	0.23	1.17	Q6PB93	Polypeptide N-acetylgalactosaminyltransferase 2;Polypeptide N-acetylg	Galnt2	2603	15	15	15	33.2	33.2	33.2	64.514	0.0000	53.682	20843000	162	15.58	15.36	16.78	15.43	15.58	15.33	15.63	15.70
0.228	0.23	1.17	Q925Q3	Sodium/potassium/calcium exchanger 6, mitochondrial	Slc8b1	4311	2	2	2	3.1	3.1	3.1	64.364	0.0000	38.453	1391900	30	13.60	12.69	13.56	13.87	12.62	13.45	12.80	13.95
0.383	0.23	1.17	Q9Z0X1	Apoptosis-inducing factor 1, mitochondrial	Aifm1	5791	31	31	31	53.4	53.4	53.4	66.765	0.0000	295.92	101030000	560	17.23	17.32	18.24	17.47	17.09	17.65	17.28	17.32

0.417	0.23	1.17	Q9QZ05	Eukaryotic translation initiation factor 2-alpha kinase 4	Eif2ak4	5582	3	3	3	2.3	2.3	2.3	186.48	0.0000	8.3241	1672300	17	13.29	12.76	13.64	13.34	12.67	13.38	12.91	13.17
0.259	0.23	1.17	O88986	2-amino-3-ketobutyrate coenzyme A ligase, mitochondrial	Gcat	592	15	15	15	42.3	42.3	42.3	44.93	0.0000	196.99	38441000	292	16.26	15.99	17.09	17.00	15.84	16.12	16.85	16.63
0.096	0.22	1.17	Q3TWF6	WD repeat-containing protein 70	Wdr70	1881	8	8	8	15.7	15.7	15.7	73.118	0.0000	12.822	3230900	48	13.21	12.72	16.28	13.48	13.00	13.93	14.42	13.44
0.500	0.22	1.17	Q3UQN2	F-BAR domain only protein 2	Fcho2	2019	24	24	24	43.5	43.5	43.5	88.733	0.0000	323.31	34313000	318	16.29	15.98	16.66	16.52	15.93	16.14	16.53	15.96
0.129	0.22	1.17	P58682	Toll-like receptor 8	Tlr8	1320	4	4	4	5	5	5	119.36	0.0000	16.039	3927800	42	14.06	13.94	16.03	14.34	14.78	13.50	15.39	13.82
0.632	0.22	1.17	Q8R050;Q1	Eukaryotic peptide chain release factor GTP-binding subunit ERF3A;Eu	Gspt1;Gspt2	3747	18	18	18	37.1	37.1	37.1	68.625	0.0000	111.27	48390000	374	16.84	16.55	16.39	16.91	16.30	16.76	16.50	16.24
0.169	0.22	1.17	Q9DBN9	Probable ATP-dependent RNA helicase DDX59	Ddx59	5166	3	3	3	6.6	6.6	6.6	68.234	0.0000	9.4085	1254700	11	12.62	13.17	13.78	13.00	12.25	12.62	14.25	12.55
0.310	0.22	1.17	P70333	Heterogeneous nuclear ribonucleoprotein H2	Hnrnph2	1554	26	25	15	63.9	61.7	44.8	49.279	0.0000	323.31	173240000	604	19.69	20.20	18.91	19.94	19.29	19.38	19.39	19.80
0.144	0.22	1.17	A2RSY6	TRMT1-like protein	Trmt1l	49	12	12	12	22.7	22.7	22.7	80.86	0.0000	108.17	8189500	100	14.94	13.01	15.43	15.12	14.79	13.83	14.58	14.41
0.308	0.22	1.17	Q8BMK4	Cytoskeleton-associated protein 4	Ckap4	3142	48	48	48	74.6	74.6	74.6	63.691	0.0000	323.31	162360000	1168	16.87	16.54	17.88	16.73	16.68	16.86	16.88	16.72
1.182	0.22	1.17	O35639	Annexin A3	Anxa3	390	38	38	38	82.7	82.7	82.7	36.384	0.0000	323.31	416320000	1370	19.40	19.32	19.45	19.67	19.27	19.40	19.20	19.10
0.310	0.22	1.16	Q91VU6	DDB1- and CUL4-associated factor 11	Dcaf11	4079	7	7	7	18	18	18	61.991	0.0000	64.588	5212800	100	14.41	13.69	14.14	14.76	13.47	14.07	14.38	14.20
0.251	0.22	1.16	P97808	FXYD domain-containing ion transport regulator 5	Fxyd5	1674	5	5	5	38.2	38.2	38.2	19.454	0.0000	120.22	7360900	111	14.65	14.33	15.65	14.97	14.09	14.71	14.79	15.14
0.280	0.22	1.16	Q6NSR8	Probable aminopeptidase NPEPL1	Npepl1	2526	16	16	16	49.6	49.6	49.6	55.939	0.0000	96.435	27602000	206	16.56	16.65	17.68	16.55	16.38	16.32	17.03	16.83
0.272	0.22	1.16	Q9JJX6	P2X purinoceptor 4	P2rx4	5438	8	8	8	24.5	24.5	24.5	43.437	0.0000	123	21349000	171	16.06	15.83	17.24	16.70	16.14	16.12	16.19	16.51
0.376	0.22	1.16	Q9WTI7	Unconventional myosin-Ic	Myo1c	5673	65	65	64	55.6	55.6	54.8	121.94	0.0000	323.31	233080000	1328	17.98	17.85	18.93	18.17	17.91	18.01	18.22	17.92
0.164	0.22	1.16	O35492	Dual specificity protein kinase CLK3	Clk3	376	2	2	2	6	6	6	73.797	0.0000	40.813	864860	20	13.08	13.39	13.70	14.21	13.45	12.84	12.60	14.62
0.151	0.22	1.16	P42128	Forkhead box protein K1	Foxk1	1073	5	5	5	8.8	8.8	8.8	74.919	0.0000	16.314	4196500	41	13.74	12.96	15.47	14.35	14.26	13.82	14.01	13.57
0.276	0.22	1.16	P22366	Myeloid differentiation primary response protein MyD88	Myd88	874	12	12	12	46.3	46.3	46.3	33.753	0.0000	40.034	13271000	143	15.51	15.14	15.61	15.58	15.47	15.83	15.29	14.38
0.854	0.22	1.16	Q9EPK7	Exportin-7	Xpo7	5256	17	17	17	20.3	20.3	20.3	123.81	0.0000	62.467	24672000	216	16.18	16.47	16.26	16.43	16.14	15.95	16.42	15.97
0.116	0.22	1.16	A2ALW5	DnaJ homolog subfamily C member 25	Dnajc25	33	5	5	5	13.7	13.7	13.7	41.937	0.0000	30.855	2829100	39	13.51	12.09	13.21	13.08	12.07	14.61	12.12	12.22
0.629	0.22	1.16	Q9WTM5	RuvB-like 2	Ruvbl2	5679	33	33	33	72.6	72.6	72.6	51.112	0.0000	323.31	113950000	676	17.28	17.24	17.85	17.40	17.05	17.45	17.16	17.24
0.096	0.22	1.16	Q99N93	39S ribosomal protein L16, mitochondrial	Mrpl16	4491	7	7	7	34.7	34.7	34.7	28.803	0.0000	43.038	6332000	39	14.42	14.56	16.16	13.84	14.67	15.59	15.23	12.63
0.366	0.22	1.16	Q8R5A3	Amyloid beta A4 precursor protein-binding family B member 1-interactin	Apbb1ip	3882	18	18	18	28.2	28.2	28.2	74.318	0.0000	261.52	97435000	538	18.19	17.87	18.78	18.18	17.92	18.35	18.28	17.62
0.098	0.22	1.16	Q9EPR4	Solute carrier family 23 member 2	Slc23a2	5262	3	3	3	8.2	8.2	8.2	70.048	0.0000	18.335	2442100	31	13.57	12.33	15.28	14.19	12.92	14.91	14.00	12.68
0.368	0.22	1.16	Q8K1M6	Dynamin-1-like protein	Dnm1l	3630	38	38	38	60.5	60.5	60.5	82.657	0.0000	323.31	119400000	853	17.08	17.05	17.87	17.12	16.73	17.05	17.50	16.98
0.928	0.22	1.16	Q99JR1	Sideroflexin-1	Sfxn1	4344	18	17	17	64.3	58.4	58.4	35.649	0.0000	323.31	91882000	550	17.58	17.25	17.68	17.64	17.29	17.47	17.37	17.16
0.408	0.22	1.16	P60122	RuvB-like 1	Ruvbl1	1351	30	30	29	75.2	75.2	75.2	50.213	0.0000	256.59	103170000	673	17.08	17.10	17.93	17.23	16.79	17.33	17.22	17.14
0.598	0.21	1.16	P08003	Protein disulfide-isomerase A4	Pdia4	674	52	52	52	59.4	59.4	59.4	71.982	0.0000	323.31	312360000	1390	18.33	18.28	18.86	18.56	18.01	18.48	18.26	18.43
2.279	0.21	1.16	P08752	Guanine nucleotide-binding protein G(i) subunit alpha-2	Gnai2	687	36	36	20	78.3	78.3	49	40.489	0.0000	323.31	910920000	1830	20.65	20.78	20.70	20.80	20.57	20.42	20.57	20.51
0.837	0.21	1.16	Q9D024	Coiled-coil domain-containing protein 47	Ccdc47	4851	19	19	19	40.8	40.8	40.8	55.843	0.0000	122.81	66408000	353	17.53	17.49	17.50	17.81	17.36	17.13	17.63	17.36
0.374	0.21	1.16	Q3ULD5	Methylcrotonoyl-CoA carboxylase beta chain, mitochondrial	Mccc2	1991	18	18	18	43.9	43.9	43.9	61.378	0.0000	129.04	29215000	222	16.74	16.32	17.35	16.75	16.32	16.79	16.80	16.39
0.430	0.21	1.16	Q8BGQ1	Spermatogenesis-defective protein 39 homolog	Vipas39	2987	10	10	10	27.1	27.1	27.1	56.624	0.0000	87.858	10403000	104	15.43	14.68	14.81	15.49	14.86	14.75	15.08	14.88
0.241	0.21	1.16	Q6PAV2	Probable E3 ubiquitin-protein ligase HERC4	Herc4	2600	19	19	19	23.7	23.7	23.7	118.41	0.0000	92.589	11425000	125	14.99	15.24	15.73	14.65	14.74	14.70	15.76	14.55
0.183	0.21	1.16	Q8K212	Phosphofurin acidic cluster sorting protein 1	Pacs1	3640	10	10	10	18	18	18	104.83	0.0000	48.196	8398700	81	14.81	14.59	16.48	15.44	14.83	15.57	14.96	15.12
0.238	0.21	1.16	Q4VC33	Macrophage erythroblast attacher	Maea	2073	15	15	15	45.5	45.5	45.5	45.336	0.0000	88.476	22347000	211	15.61	15.09	16.72	15.65	15.26	15.53	15.70	15.74
0.202	0.21	1.16	Q8VDG7	Platelet-activating factor acetylhydrolase 2, cytoplasmic	Pafah2	3953	10	10	10	35.1	35.1	35.1	43.561	0.0000	22.493	3315800	26	14.32	14.27	15.57	13.93	13.73	14.48	14.34	14.69
0.302	0.21	1.16	P43406	Integrin alpha-V;Integrin alpha-V heavy chain;Integrin alpha-V light chai	Itgav	1092	16	16	16	19.6	19.6	19.6	115.36	0.0000	56.932	20604000	154	16.80	16.23	15.89	16.35	16.44	15.88	16.52	15.58
0.197	0.21	1.16	Q9JIY0	Pleckstrin homology domain-containing family O member 1	Plekho1	5411	12	12	12	30.4	30.4	30.4	45.996	0.0000	41.883	16827000	118	15.19	15.78	16.90	15.32	15.25	15.59	16.04	15.47
0.690	0.21	1.16	Q9QYR9	Acyl-coenzyme A thioesterase 2, mitochondrial	Acot2	5579	16	16	6	48.8	48.8	21.9	49.656	0.0000	156.24	42129000	381	16.30	16.11	16.64	16.42	15.95	16.18	16.42	16.09
0.227	0.21	1.16	Q61210	Rho guanine nucleotide exchange factor 1	Arhgef1	2278	45	45	45	47.4	47.4	47.4	102.8	0.0000	323.31	108880000	819	17.47	17.03	18.68	17.69	17.20	17.81	17.41	17.60
0.602	0.21	1.16	Q924T2	28S ribosomal protein S2, mitochondrial	Mrps2	4300	6	6	6	29.2	29.2	29.2	32.313	0.0000	70.838	12846000	112	16.20	15.77	15.87	16.33	15.62	15.72	16.07	15.92
0.454	0.21	1.16	Q9ESX5	H/ACA ribonucleoprotein complex subunit 4	Dkc1	5350	20	20	20	47.3	47.3	47.3	57.401	0.0000	187.03	39135000	354	16.42	16.12	16.81	16.79	16.22	16.10	16.68	16.29
0.888	0.21	1.16	Q99JI6	Ras-related protein Rap-1b	Rap1b	4341	20	20	8	85.9	85.9	38.6	20.825	0.0000	323.31	304390000	855	19.72	19.56	19.44	19.97	19.40	19.40	19.49	19.56
0.148	0.21	1.16	Q8R5F7	Interferon-induced helicase C domain-containing protein 1	Ifih1	3887	30	30	29	36.2	36.2	34.8	115.97	0.0000	84.3	31479000	182	16.55	16.00	15.85	17.50	16.17	15.42	17.31	16.16
0.334	0.21	1.16	Q8R4K2	Interleukin-1 receptor-associated kinase 4	Irak4	3870	13	13	13	29.6	29.6	29.6	50.871	0.0000	48.823	22798000	226	15.73	15.31	16.52	15.79	15.39	15.73	15.62	15.79
0.529	0.21	1.15	P70295	Ancient ubiquitous protein 1	Aup1	1548	15	15	15	38.5	38.5	38.5	46.121	0.0000	26.29	25524000	187	16.30	16.03	16.39	16.73	15.90	16.09	16.21	16.43
0.210	0.21	1.15	Q99LI8	Hepatocyte growth factor-regulated tyrosine kinase substrate	Hgs	4434	21	21	20	32.4	32.4	31.4	86.014	0.0000	139.57	24375000	185	16.34	15.93	17.37	15.61	15.92	16.36	16.05	16.10
0.116	0.21	1.15	Q3TKY6	Peptidyl-prolyl cis-trans isomerase CWC27 homolog	Cwc27	1857	7	7	7	24.3	24.3	24.3	53.542	0.0000	36.211	5516000	54	13.48	14.27	16.00	13.51	14.44	13.67	14.79	13.53
0.351	0.21	1.15	Q9D880	Mitochondrial import inner membrane translocase subunit TIM50	Timm50	5053	18	18	18	38	38	38	39.776	0.0000	170.07	46372000	331	16.75	16.52	17.47	16.85	16.26	16.73	16.82	16.95
0.367	0.21	1.15	Q8CHC4	Synaptojanin-1	Synj1	3521	32	32	32	30.2	30.2	30.2	172.62	0.0000	322.53	68612000	521	16.41	16.22	17.08	16.82	16.14	16.57	16.77	16.24
0.440	0.21	1.15	P49935	Pro-cathepsin H;Cathepsin H mini chain;Cathepsin H;Cathepsin H heav	Ctsh	1172	10	10	10	38.4	38.4	38.4	37.17	0.0000	29.898	24968000	149	17.23	17.58	17.38	17.15	17.33	16.78	16.86	17.55
0.133	0.20	1.15	Q61387	Cytochrome c oxidase subunit 7A-related protein, mitochondrial	Cox7a2l	2295	5	5	5	62.2	62.2	62.2	12.399	0.0000	58.859	4429300	39	13.68	13.84	13.92	12.25	12.62	12.39	13.75	14.13
0.233	0.20	1.15	Q8C4B4	Protein unc-119 homolog B	Unc119b	3385	5	5	4	27.1	27.1	23.5	28.302	0.0000	34.59	5281200	78	15.07	14.68	15.52	14.88	13.94	15.31	14.95	15.12
0.124	0.20	1.15	Q3TL44	NLR family member X1	Nlrx1	1858	14	14	14	18.3	18.3	18.3	107.83	0.0000	41.586	8997200	81	14.75	14.09	16.11	14.60	13.76	14.11	15.43	15.44
0.098	0.20	1.15	Q9EQU3	Toll-like receptor 9	Tlr9	5288	11	11	11	15.3	15.3	15.3	116.41	0.0000	49.093	7251400	49	13.73	14.21	16.46	14.51	13.42	15.50	15.07	14.11
0.251	0.20	1.15	Q8R1Q8	Cytoplasmic dynein 1 light intermediate chain 1	Dync1li1	3794	29	29	28	52.4	52.4	49.9	56.614	0.0000	323.31	109310000	596	17.85	17.71	19.10	18.17	17.72	18.20	17.99	18.12
1.416	0.20	1.15	Q93092	Transaldolase	Taldo1	4313	44	44	44	70.3	70.3	70.3	37.387	0.0000	323.31	1825900000	2338	21.41	21.25	21.09	21.21	21.04	20.95	21.15	21.02
0.206	0.20	1.15	Q60664	Lymphoid-restricted membrane protein;Processed lymphoid-restricted n	Lrmp	2190	9	9	9	27.1	27.1	27.1	59.587	0.0000	79.398	147010000	86	21.64	20.89	21.36	20.68	20.57	20.55	20.74	21.90
0.235	0.20	1.15	Q9DCJ1	Target of rapamycin complex subunit LST8	Mlst8	5216	8	8	7	39	39	31.3	35.851	0.0000	20.891	6235100	71	14.64	15.43	14.90	15.41	15.03	14.63	15.63	14.28
0.396	0.20	1.15	P62880	Guanine nucleotide-binding protein G(I)/G(S)/G(T) subunit beta-2	Gnb2	1462	20	12	10	65.3	44.1	40.3	37.331	0.0000	323.31	307080000	533	20.62	20.15	20.75	20.66	20.25	20.68	20.56	19.87
0.231	0.20	1.15	Q64373	Bcl-2-like protein 1	Bcl2l1	2437	3	3	3	13.7	13.7	13.7	26.132	0.0004	4.8422	4766600	46	14.88	14.48	13.66	14.02	14.49	13.40	14.18	14.17
0.208	0.20	1.15	P98078	Disabled homolog 2	Dab2	1685	19	19	19	33.4	33.4	33.4	82.311	0.0000	293.8	29339000	316	15.19	15.21	16.73	15.99	15.38	15.40	15.86	15.66
0.645	0.20	1.15	Q3UFK8	FERM domain-containing protein 8	Frmd8	1955	17	17	17	49.4	49.4	49.4	51.827	0.0000	220.42	52414000	383	17.99	17.68	18.01	17.80	17.39	17.52	17.86	17.91
0.542	0.20	1.15	B2RQC6	CAD protein;Glutamine-dependent carbamoyl-phosphate synthase;Asp	Cad	77	43	43	43	27	27	27	243.24	0.0000	323.31	75280000	637	16.52	16.38	17.03	16.82	16.46	16.42	16.75	16.33
0.570	0.20	1.15	Q9D8N0	Elongation factor 1-gamma	Eef1g	5065	34	34	34	62.9	62.9	62.9	50.06	0.0000	323.31	586990000	1119	20.48	20.26	19.81	20.35	20.25	19.92	20.01	19.92
1.254	0.20	1.15	O54984	ATPase Asna1	Asna1	439	19	19	19	68.1	68.1	68.1	38.822	0.0000	288.27	72323000	368	17.82	17.79	17.68	18.02	17.70	17.70	17.60	17.51
0.435	0.20	1.15	P70168	Importin subunit beta-1	Kpnb1	1529	41	41	40	51.1	51.1	51.1	97.183	0.0000	323.31	302790000	1009	19.01	18.91	19.78	19.21	18.86	19.06	19.16	19.03
0.254	0.20	1.15	Q8R5F3	O-acetyl-ADP-ribose deacetylase 1	Oard1	3886	3	3	3	22.4	22.4	22.4	17.107	0.0000	16.903	3007700	29	14.28	14.31	13.73	14.39	14.23	14.23	14.33	13.13
0.400	0.20	1.15	Q5SNZ0	Girdin	Ccdc88a	2131	13	13	13	10.2	10.2	10.2	215.92	0.0000	48.173	17747000	91	17.09	16.93	17.33	17.58	16.60	17.35	17.24	16.93
0.363	0.20	1.15	Q91WQ3	Tyrosine--tRNA ligase, cytoplasmic;Tyrosine--tRNA ligase, cytoplasmic,	Yars	4123	35	35	35	67.8	67.8	67.8	59.105	0.0000	239.69	122400000	767	17.63	17.73	18.50	17.91	17.41	18.05	17.68	17.84
0.516	0.20	1.15	Q9WVE8	Protein kinase C and casein kinase substrate in neurons protein 2	Pacsin2	5746	27	27	27	56.6	56.6	56.6	55.832	0.0000	165.61	81990000	517	17.38	16.99	17.84	17.49	17.18	17.25	17.28	17.19
0.334	0.20	1.15	O08848	60 kDa SS-A/Ro ribonucleoprotein	Trove2	307	7	7	7	15.2	15.2	15.2	60.123	0.0000	22.965	5924000	72	14.85	14.68	15.18	15.00	14.32	14.40	15.31	14.89
0.348	0.20	1.15	Q8K2I4	Beta-mannosidase	Manba	3662	16	16	16	21.4	21.4	21.4	100.83	0.0000	185.71	32073000	254	17.09	16.94	16.52	17.64	16.87	16.90	17.01	16.62
0.106	0.20	1.15	Q08122	Transducin-like enhancer protein 3	Tle3	1761	8	8	4	15.7	15.7	9.8	83.446	0.0000	21.187	20540000	58	18.57	16.53	17.77	17.78	19.05	17.30	16.63	16.87
0.158	0.20	1.15	P70451	Tyrosine-protein kinase Fer	Fer	1576	4	3	3	5.8	4.5	4.5	94.578	0.0000	63.348	1301200	10	13.19	13.91	12.79	13.77	13.49	14.24	12.49	12.66
0.133	0.20	1.15	Q91XV3	Brain acid soluble protein 1	Basp1	4150	10	10	10	51.8	51.8	51.8	22.086	0.0000	261.36	23943000	228	16.36	17.10	17.23	15.18	16.49	16.91	15.46	16.22
0.655	0.20	1.15	P11983	T-complex protein 1 subunit alpha	Tcp1	753	50	50	50	88.8	88.8	88.8	60.448	0.0000	323.31	734380000	2228	20.61	20.29	20.28	20.48	20.16	20.38	20.43	19.91
0.214	0.20	1.15	Q99LC9	Peroxisome assembly factor 2	Pex6	4419	3	3	3	7	7	7	104.55	0.0000	47.747	2056300	45	13.78	12.99	13.64	14.06	13.78	12.87	12.99	14.03
0.143	0.20	1.15	Q9D3S3	Sorting nexin-29	Snx29	4969	8	8	8	14.5	14.5	14.5	91.386	0.0000	7.2395	2373800	17	13.70	12.87	14.66	12.45	13.08	13.76	13.09	12.97
0.198	0.20	1.15	Q4VA53	Sister chromatid cohesion protein PDS5 homolog B	Pds5b	2066	25	25	25	23	23	23	164.42	0.0000	123.48	37546000	248	16.94	16.97	18.52	17.26	17.24	16.90	17.45	17.32
0.349	0.20	1.14	P50429	Arylsulfatase B	Arsb	1182	9	9	9	17	17	17	59.646	0.0000	18.37	18253000	117	16.42	16.74	17.39	17.28	16.77	16.98	16.65	16.65
0.146	0.20	1.14	Q8R3G1	Nuclear inhibitor of protein phosphatase 1	Ppp1r8	3846	5	5	5	21.4	21.4	21.4	38.527	0.0000	38.167	14487000	69	17.02	16.89	16.74	16.12	16.07	17.57	16.90	15.44
0.270	0.19	1.14	Q9JKX6	ADP-sugar pyrophosphatase	Nudt5	5463	7	7	7	36.2	36.2	36.2	23.984	0.0000	19.57	6463000	83	14.77	14.93	16.06	15.20	15.21	14.84	15.11	15.03
0.098	0.19	1.14	P62700	Protein yippee-like 5	Ypel5	1435	3	3	3	33.1	33.1	33.1	13.841	0.0000	34.357	2775200	35	14.72	14.70	14.58	12.39	12.59	14.49	14.30	14.23
0.154	0.19	1.14	Q8R3Y8	Interferon regulatory factor 2-binding protein 1	Irf2bp1	3860	5	4	4	12.5	11.3	11.3	61.75	0.0000	23.07	5377400	85	15.04	14.87	14.15	14.55	13.16	14.79	15.07	14.81
0.194	0.19	1.14	Q923W1	Trimethylguanosine synthase	Tgs1	4288	2	2	2	3.5	3.5	3.5	96.789	0.0004	4.435	767590	8	13.30	14.33	13.54	13.57	13.72	14.18	13.41	12.65
0.314	0.19	1.14	Q8C863	E3 ubiquitin-protein ligase Itchy	Itch	3421	8	8	6	9.6	9.6	6.8	98.992	0.0000	13.775	14759000	108	16.36	15.82	15.89	16.25	16.07	15.28	16.31	15.89
0.257	0.19	1.14	Q9QZ06	Toll-interacting protein	Tollip	5583	9	9	9	52.2	52.2	52.2	30.345	0.0000	123.39	20794000	184	16.86	16.87	16.06	16.96	16.52	16.88	16.70	15.87
0.144	0.19	1.14	Q921G6	Leucine-rich repeat and calponin homology domain-containing protein 4	Lrch4	4222	23	23	23	53.4	53.4	53.4	73.162	0.0000	241.83	76335000	404	17.78	17.79	19.81	18.38	17.81	18.39	18.55	18.24
0.445	0.19	1.14	Q9JHG7	Phosphatidylinositol 4,5-bisphosphate 3-kinase catalytic subunit gamma	Pik3cg	5359	17	17	16	20.3	20.3	18.6	126.4	0.0000	109.84	20546000	194	15.75	15.59	16.15	15.91	15.33	15.70	16.06	15.55
0.641	0.19	1.14	P53994	Ras-related protein Rab-2A	Rab2a	1243	18	18	12	75.5	75.5	47.2	23.547	0.0000	272.83	139920000	455	18.62	18.63	18.53	18.37	18.47	17.97	18.51	18.44
0.128	0.19	1.14	P59672	Ankyrin repeat and SAM domain-containing protein 1A	Anks1a	1340	13	13	13	18.6	18.6	18.6	125.24	0.0000	17.437	7911400	39	13.99	13.60	15.69	13.89	13.97	13.37	14.35	14.73
0.730	0.19	1.14	Q9EP71	Ankycorbin	Rai14	5244	13	13	13	20.1	20.1	20.1	108.85	0.0000	40.103	8994700	121	14.47	14.55	14.71	14.41	14.46	14.17	14.59	14.16
0.141	0.19	1.14	P70704	Phospholipid-transporting ATPase IA	Atp8a1	1589	11	11	11	12.8	12.8	12.8	131.41	0.0000	17.201	8451900	58	14.36	14.89	15.52	14.00	14.37	14.86	15.28	13.50
0.808	0.19	1.14	Q91VR2	ATP synthase subunit gamma, mitochondrial	Atp5c1	4072	22	22	22	58.7	58.7	58.7	32.886	0.0000	323.31	175060000	728	18.33	18.15	18.24	18.41	18.36	18.11	17.87	18.04
0.432	0.19	1.14	Q6Q477	Calcium-transporting ATPase	Atp2b4	2649	19	6	6	21.2	9.1	9.1	133.07	0.0000	15.28	3989500	31	14.36	14.19	13.73	14.36	13.99	13.61	14.18	14.11
0.083	0.19	1.14	Q91VW5	Golgin subfamily A member 4	Golga4	4082	10	10	9	6	6	5.4	257.56	0.0000	17.554	3346400	27	14.31	12.54	16.42	13.74	14.05	13.72	14.40	14.09
1.106	0.19	1.14	P58252	Elongation factor 2	Eef2	1312	91	91	89	82.6	82.6	81.4	95.313	0.0000	323.31	3129900000	5539	21.01	20.87	21.00	20.98	20.88	20.53	20.85	20.86
0.790	0.19	1.14	P80318	T-complex protein 1 subunit gamma	Cct3	1595	46	46	46	74.3	74.3	74.3	60.629	0.0000	323.31	704220000	1952	20.06	19.80	19.95	19.88	19.76	19.81	19.93	19.44
0.318	0.19	1.14	P14576	Signal recognition particle 54 kDa protein	Srp54	782	26	26	26	62.5	62.5	62.5	55.72	0.0000	260.59	66627000	462	17.30	17.20	17.79	17.35	17.10	17.58	17.53	16.68
0.145	0.19	1.14	Q61107	Guanylate-binding protein 4	Gbp4	2253	16	16	16	32.6	32.6	32.6	70.801	0.0000	74.575	13231000	127	15.26	14.32	16.42	15.66	15.08	14.96	15.87	14.99
0.089	0.19	1.14	Q8VBV3	Exosome complex component RRP4	Exosc2	3897	6	6	6	24.6	24.6	24.6	32.631	0.0000	25.784	5589900	59	14.10	15.02	12.39	14.92	14.24	14.60	14.28	12.57
0.223	0.19	1.14	Q8R3N6	THO complex subunit 1	Thoc1	3852	9	9	9	17.4	17.4	17.4	75.435	0.0000	45.897	12324000	98	15.34	14.77	15.50	15.33	15.10	14.23	15.27	15.61
0.260	0.19	1.14	Q61595	Kinectin	Ktn1	2316	18	18	18	19.9	19.9	19.9	152.59	0.0000	57.494	10503000	114	14.92	14.29	15.35	15.14	14.37	14.89	15.18	14.52
0.646	0.18	1.14	A1L314	Macrophage-expressed gene 1 protein	Mpeg1	4	24	24	24	33.9	33.9	33.9	78.39	0.0000	253.16	336230000	813	19.65	19.57	19.85	19.72	19.54	19.38	19.85	19.29
0.151	0.18	1.14	Q9D920	Loss of heterozygosity 12 chromosomal region 1 protein homolog	Loh12cr1	5090	5	5	5	40.5	40.5	40.5	22.12	0.0000	18.885	2776200	32	13.89	14.01	15.15	13.28	13.72	14.24	14.36	13.28
0.073	0.18	1.14	P23949	Zinc finger protein 36, C3H1 type-like 2	Zfp36l2	892	6	6	5	15.7	15.7	15.1	50.069	0.0000	21.836	6233400	87	15.44	15.73	14.25	15.72	16.07	12.61	15.76	15.96

0.438	0.18	1.13	Q8C1A5	Thimet oligopeptidase	Thop1		3362	27	27	27	47	47	47	78.025	0.0000	130.69	44336000	377	16.00	15.75	16.54	15.95	15.73	15.96	16.05	15.77
0.167	0.18	1.13	A6H5X4	PHD finger protein 11	Phf11		57	6	6	6	27.3	27.3	27.3	37.728	0.0000	64.233	1726500	18	12.53	13.86	13.72	12.69	13.33	13.41	13.01	12.33
0.489	0.18	1.13	P49442	Inositol polyphosphate 1-phosphatase	Inpp1		1162	12	12	12	37.4	37.4	37.4	43.346	0.0000	64.396	9912100	130	15.63	15.44	15.15	15.62	15.17	14.99	15.37	15.58
0.239	0.18	1.13	Q4LDD4	Arf-GAP with Rho-GAP domain, ANK repeat and PH domain-containing	Arap1		2063	53	53	53	43.6	43.6	43.6	162.27	0.0000	323.31	126300000	871	17.05	16.63	17.96	17.11	16.74	17.36	17.02	16.92
0.125	0.18	1.13	Q3TFD2	Lysophosphatidylcholine acyltransferase 1	Lpcat1		1837	5	5	5	19.1	19.1	19.1	59.743	0.0000	44.356	6072800	62	14.31	14.39	16.04	15.17	14.88	14.87	15.58	13.83
0.281	0.18	1.13	Q9ES28	Rho guanine nucleotide exchange factor 7	Arhgef7		5324	27	27	24	40.7	40.7	38.1	97.055	0.0000	113.82	54932000	416	16.64	16.11	17.34	16.65	16.26	16.54	16.54	16.68
0.097	0.18	1.13	Q99LJ1	Tissue alpha-L-fucosidase	Fuca1		4436	9	9	9	27.9	27.9	27.9	52.28	0.0000	113.14	19523000	117	16.35	15.81	14.52	15.72	14.57	14.41	16.70	15.99
0.265	0.18	1.13	P14131	40S ribosomal protein S16	Rps16		774	22	22	22	78.8	78.8	78.8	16.445	0.0000	48.605	247350000	655	19.17	18.91	20.10	19.53	19.20	19.49	18.98	19.32
0.379	0.18	1.13	Q3UMY5	Echinoderm microtubule-associated protein-like 4	Eml4		2006	13	13	13	18.4	18.4	18.4	110.03	0.0000	27.558	11247000	97	15.56	15.05	15.68	14.92	14.96	15.36	15.12	15.06
0.557	0.18	1.13	Q99MN1	Lysine--tRNA ligase	Kars		4468	37	37	37	47.6	47.6	47.6	67.839	0.0000	299.57	96053000	754	17.97	17.82	18.31	18.13	17.74	18.17	17.71	17.91
0.258	0.18	1.13	Q91WM3	U3 small nucleolar RNA-interacting protein 2	Rrp9		4121	8	8	8	19.8	19.8	19.8	52.106	0.0000	29.921	7643300	98	15.18	15.08	15.46	15.26	14.83	15.88	14.76	14.81
0.137	0.18	1.13	Q8K301	Probable ATP-dependent RNA helicase DDX52	Ddx52		3682	6	6	6	15.6	15.6	15.6	67.473	0.0000	83.499	3402800	34	14.17	13.21	14.23	14.15	14.15	13.40	14.72	12.79
0.296	0.18	1.13	Q91YD3	mRNA-decapping enzyme 1A	Dcp1a		4153	3	3	3	8.8	8.8	8.8	65.218	0.0000	10.689	6885800	64	14.62	15.17	14.62	15.21	14.44	14.92	15.16	14.40
0.151	0.18	1.13	Q9JLI8	Squamous cell carcinoma antigen recognized by T-cells 3	Sart3		5481	24	24	23	32.1	32.1	32	109.62	0.0000	186.31	30081000	323	15.81	15.80	17.49	15.75	15.80	16.41	15.90	16.04
0.065	0.18	1.13	Q9WVL2	Signal transducer and activator of transcription 2	Stat2		5755	17	17	17	19.2	19.2	19.2	105.42	0.0000	56.446	11195000	86	14.09	13.33	17.26	14.31	13.77	14.20	15.73	14.58
0.187	0.18	1.13	Q8BUR4	Dedicator of cytokinesis protein 1	Dock1		3229	22	21	20	15.1	14.6	14	215.08	0.0000	42.03	12822000	128	15.09	14.95	15.47	15.50	14.44	16.04	14.88	14.95
0.237	0.18	1.13	Q9WUP7	Ubiquitin carboxyl-terminal hydrolase isozyme L5	Uchl5		5719	17	17	17	63.8	63.8	63.8	37.616	0.0000	129.87	46220000	331	16.93	16.60	15.86	17.02	16.19	16.60	16.73	16.18
0.203	0.17	1.13	Q80TH2	Protein LAP2	Erbb2ip		2804	21	21	19	23.3	23.3	21.4	157.25	0.0000	94.214	33838000	276	16.59	16.22	17.40	16.44	16.04	16.19	16.99	16.72
0.403	0.17	1.13	P47199	Quinone oxidoreductase	Cryz		1114	6	6	6	29.3	29.3	29.3	35.268	0.0000	24.786	8607100	158	14.83	14.52	14.89	14.83	14.39	15.10	14.51	14.37
0.210	0.17	1.13	Q9Z120	tRNA (guanine-N(7)-)-methyltransferase	Mettl1		5796	2	2	2	10.8	10.8	10.8	30.603	0.0000	6.5996	890420	19	13.69	13.43	12.99	13.20	12.95	13.86	13.33	12.47
0.166	0.17	1.13	P53811	Phosphatidylinositol transfer protein beta isoform	Pitpnb		1241	15	13	13	53.1	46.9	46.9	31.487	0.0000	40.068	21397000	208	16.07	15.63	14.64	16.01	15.45	14.77	15.87	15.56
0.190	0.17	1.13	Q922P9	Putative oxidoreductase GLYR1	Glyr1		4266	13	13	13	31.5	31.5	31.5	59.715	0.0000	121.08	32777000	416	16.05	15.98	17.41	16.08	16.14	16.44	16.24	16.01
0.403	0.17	1.13	Q8CGK3	Lon protease homolog, mitochondrial	Lonp1		3509	46	46	46	63.1	63.1	63.1	105.84	0.0000	323.31	86030000	712	16.82	16.73	17.28	16.68	16.43	16.57	16.82	16.99
0.677	0.17	1.13	O54941	SWI/SNF-related matrix-associated actin-dependent regulator of chroma	Smarce1		435	9	9	9	29	29	29	46.638	0.0000	70.772	14119000	150	16.12	16.35	16.08	16.09	15.76	15.86	16.17	16.16
0.169	0.17	1.13	Q8CI59	Metalloreductase STEAP3	Steap3		3541	17	17	17	37.5	37.5	37.5	54.748	0.0000	104.34	54839000	347	16.76	16.26	18.06	17.06	16.65	16.97	17.08	16.77
0.228	0.17	1.13	Q4FZC9	Nesprin-3	Syne3		2058	18	18	18	28	28	28	112.03	0.0000	116.63	23153000	281	15.36	14.85	16.14	15.55	14.98	15.23	15.65	15.35
0.597	0.17	1.12	P49443	Protein phosphatase 1A	Ppm1a		1163	7	4	4	22	13.9	13.9	42.432	0.0000	21.232	6657300	88	15.41	15.06	15.14	15.47	15.13	14.85	15.27	15.15
0.145	0.17	1.12	Q9WUK4	Replication factor C subunit 2	Rfc2		5713	11	11	11	54.7	54.7	54.7	38.724	0.0000	51.62	6468300	70	14.70	14.32	16.01	14.26	14.89	14.81	14.78	14.13
0.395	0.17	1.12	P54071	Isocitrate dehydrogenase [NADP], mitochondrial	Idh2		1246	38	36	36	71.5	71.5	71.5	50.906	0.0000	323.31	323530000	1118	18.70	18.40	19.00	18.58	18.48	18.83	18.54	18.15
0.820	0.17	1.12	Q5FWK3	Rho GTPase-activating protein 1	Arhgap1		2105	22	22	22	61.3	61.3	61.3	50.41	0.0000	306.58	107950000	659	18.08	17.88	18.21	18.11	17.82	17.93	18.11	17.75
0.279	0.17	1.12	Q8K2Y7	39S ribosomal protein L47, mitochondrial	Mrpl47		3678	8	8	8	32.5	32.5	32.5	29.725	0.0000	48.688	10017000	144	14.92	14.40	14.36	14.86	14.65	14.17	14.96	14.09
0.293	0.17	1.12	Q8BJY1	26S proteasome non-ATPase regulatory subunit 5	Psmd5		3090	29	29	29	70.2	70.2	70.2	55.971	0.0000	323.31	113900000	733	18.06	18.15	17.28	17.98	17.60	17.87	17.97	17.36
0.165	0.17	1.12	O35083	1-acyl-sn-glycerol-3-phosphate acyltransferase alpha	Agpat1		334	4	4	4	21.4	21.4	21.4	31.709	0.0000	81.267	24370000	142	17.14	17.15	18.53	17.15	17.24	17.40	17.79	16.87
0.327	0.17	1.12	O08749	Dihydrolipoyl dehydrogenase, mitochondrial	Dld		294	25	25	25	56.4	56.4	56.4	54.272	0.0000	322.61	147840000	701	17.87	17.85	18.29	17.86	17.61	18.38	17.62	17.60
0.141	0.17	1.12	Q7TSI3	Serine/threonine-protein phosphatase 6 regulatory subunit 1	Ppp6r1		2786	12	12	12	27.7	27.7	27.7	94.526	0.0000	88.073	8551900	97	14.63	14.31	15.80	13.90	14.27	14.28	15.06	14.36
0.153	0.17	1.12	Q7TSI1	Pleckstrin homology domain-containing family M member 1	Plekhm1		2785	5	5	5	6.8	6.8	6.8	118.53	0.0000	20.721	4484200	66	13.56	13.25	14.55	14.09	13.18	14.05	14.39	13.16
0.785	0.17	1.12	Q9JLJ2	4-trimethylaminobutyraldehyde dehydrogenase	Aldh9a1		5483	40	40	40	76.7	76.7	76.7	53.514	0.0000	323.31	322850000	1317	18.82	18.56	18.91	18.54	18.40	18.64	18.54	18.57
1.078	0.17	1.12	Q62465	Synaptic vesicle membrane protein VAT-1 homolog	Vat1		2397	26	26	26	60.3	60.3	60.3	43.096	0.0000	323.31	655080000	1402	20.04	19.91	20.04	20.07	19.98	19.80	19.95	19.66
0.217	0.17	1.12	Q80YW0	Cytohesin-4	Cyth4		2910	14	14	14	41	41	41	45.284	0.0000	120.14	25624000	161	15.98	16.12	16.67	16.47	16.15	15.45	16.72	16.26
0.309	0.17	1.12	P16330	2,3-cyclic-nucleotide 3-phosphodiesterase	Cnp		808	13	13	13	37.6	37.6	37.6	47.123	0.0000	94.216	17881000	198	15.38	15.00	15.79	15.63	15.23	15.20	15.71	15.00
0.248	0.17	1.12	Q9EQ20	Methylmalonate-semialdehyde dehydrogenase [acylating], mitochondria	Aldh6a1		5269	15	15	15	37.6	37.6	37.6	57.915	0.0000	122.85	17464000	140	15.91	15.71	16.86	16.33	16.12	16.23	15.77	16.03
0.200	0.17	1.12	P61327;Q9C	Protein mago nashi homolog;Protein mago nashi homolog 2	Magoh;Magohb		1383	10	10	10	66.4	66.4	66.4	17.163	0.0000	69.555	52310000	203	17.35	17.07	18.41	17.75	17.02	17.64	17.58	17.66
0.251	0.17	1.12	Q3KNM2	E3 ubiquitin-protein ligase MARCH5	March5		1809	4	4	4	16.5	16.5	16.5	31.231	0.0000	8.0107	3859900	44	13.43	13.61	14.41	13.96	13.72	14.06	13.70	13.26
0.307	0.17	1.12	Q9QY76	Vesicle-associated membrane protein-associated protein B	Vapb		5562	18	17	17	60.9	60.9	60.9	26.946	0.0000	118.42	143800000	492	18.25	17.77	18.79	18.13	17.99	18.13	18.26	17.89
0.204	0.16	1.12	P97287	Induced myeloid leukemia cell differentiation protein Mcl-1 homolog	Mcl1		1617	11	11	11	51.1	51.1	51.1	35.217	0.0000	57.391	29590000	201	16.59	16.33	17.66	16.79	16.43	16.56	17.07	16.65
0.156	0.16	1.12	O70311	Glycylpeptide N-tetradecanoyltransferase 2	Nmt2		487	10	6	6	18.1	14.6	14.6	60.484	0.0000	47.951	4182100	59	14.73	14.33	15.77	14.83	14.62	14.83	15.43	14.14
0.656	0.16	1.12	Q9DBP5	UMP-CMP kinase	Cmpk1		5167	20	20	20	76.5	76.5	76.5	22.165	0.0000	323.31	178060000	715	18.12	18.18	17.85	18.15	17.67	18.12	17.89	17.97
0.133	0.16	1.12	Q5RKZ7	Molybdenum cofactor biosynthesis protein 1;Cyclic pyranopterin monop	Mocs1		2127	10	10	10	22.3	22.3	22.3	69.858	0.0000	57.286	8627300	116	14.52	14.17	15.74	15.03	14.50	13.89	15.24	15.17
0.130	0.16	1.12	A2A5R2	Brefeldin A-inhibited guanine nucleotide-exchange protein 2	Arfgef2		7	29	29	23	21.7	21.7	17	202.24	0.0000	276.91	21777000	249	15.25	14.86	16.65	14.59	14.92	15.15	15.50	15.13
0.329	0.16	1.12	Q80VP0	Tectonin beta-propeller repeat-containing protein 1	Tecpr1		2853	20	20	20	23.2	23.2	23.2	130.26	0.0000	57.631	19645000	189	15.57	15.38	14.87	15.54	15.08	15.55	15.16	14.92
0.100	0.16	1.12	Q91WG8	Bifunctional UDP-N-acetylglucosamine 2-epimerase/N-acetylmannosam	Gne		4112	6	6	6	13.7	13.7	13.7	79.198	0.0000	12.28	1984400	13	13.77	12.10	14.44	14.10	13.59	13.81	12.58	13.78
0.552	0.16	1.12	Q91W50	Cold shock domain-containing protein E1	Csde1		4091	27	27	27	32.6	32.6	32.6	88.79	0.0000	154.15	59729000	504	16.46	16.41	16.93	16.82	16.39	16.61	16.47	16.50
0.232	0.16	1.12	Q9JIY5	Serine protease HTRA2, mitochondrial	Htra2		5412	14	14	13	37.1	37.1	37.1	49.348	0.0000	120.87	36951000	213	16.12	16.88	16.79	15.88	15.89	16.45	16.45	16.23
0.102	0.16	1.12	Q2EMV9	Poly [ADP-ribose] polymerase 14	Parp14		1796	50	50	50	34.6	34.6	34.6	203.8	0.0000	286.34	48370000	407	15.56	15.01	17.38	16.52	15.42	15.96	16.65	15.79
0.289	0.16	1.12	Q8BY87	Ubiquitin carboxyl-terminal hydrolase 47	Usp47		3294	20	20	20	22.6	22.6	22.6	157.45	0.0000	51.601	18988000	181	15.75	15.35	15.07	15.84	15.11	15.27	15.78	15.21
0.186	0.16	1.12	Q8CFX1	GDH/6PGL endoplasmic bifunctional protein;Glucose 1-dehydrogenase	H6pd		3493	15	15	15	28.1	28.1	28.1	88.927	0.0000	147.25	40679000	240	17.93	18.18	16.83	18.21	17.85	17.47	17.73	17.46
0.155	0.16	1.12	Q9JJF9	Signal peptide peptidase-like 2A	Sppl2a		5426	5	5	5	13.4	13.4	13.4	58.128	0.0000	68.384	9578100	73	15.40	14.89	16.12	16.17	14.75	15.85	15.74	15.60
0.374	0.16	1.12	P14206	40S ribosomal protein SA	Rpsa		777	20	20	18	64.4	64.4	64.1	32.838	0.0000	323.31	469100000	741	20.60	20.48	21.19	20.52	20.47	20.80	20.46	20.42
0.157	0.16	1.12	Q8C7R4	Ubiquitin-like modifier-activating enzyme 6	Uba6		3414	25	25	24	29.4	29.4	27.6	117.96	0.0000	99.171	39601000	282	16.21	16.03	17.42	15.87	15.72	16.29	16.45	16.43
0.057	0.16	1.12	Q8CDJ3	Beclin 1-associated autophagy-related key regulator	Atg14		3461	3	3	3	8.1	8.1	8.1	55.388	0.0013	3.4959	3751300	20	14.84	11.90	15.07	15.16	15.05	15.22	12.70	13.34
0.113	0.16	1.12	Q78JE5	F-box only protein 22	Fbxo22		2725	6	6	6	18.9	18.9	18.9	44.202	0.0000	95.559	8552700	118	14.57	14.69	16.75	14.86	15.12	15.10	15.23	14.77
0.346	0.16	1.12	Q60932	Voltage-dependent anion-selective channel protein 1	Vdac1		2224	30	30	30	86.1	86.1	86.1	32.351	0.0000	323.31	452670000	1408	20.23	20.21	19.97	20.08	20.09	20.33	20.01	19.44
0.542	0.16	1.12	O88487	Cytoplasmic dynein 1 intermediate chain 2	Dync1i2		534	18	18	18	42.5	42.5	42.5	68.393	0.0000	323.31	138280000	626	18.60	18.46	18.91	18.71	18.34	18.73	18.61	18.35
1.001	0.16	1.12	Q9DBJ1	Phosphoglycerate mutase 1	Pgam1		5158	36	36	29	95.7	95.7	89.8	28.832	0.0000	323.31	2507300000	2081	22.21	22.17	22.52	22.31	22.20	22.14	22.10	22.15
0.263	0.16	1.12	P47809	Dual specificity mitogen-activated protein kinase kinase 4	Map2k4		1125	7	7	7	23.9	23.9	23.9	44.113	0.0000	57.418	20620000	189	15.96	15.81	16.36	16.56	15.89	16.30	16.30	15.56
0.379	0.16	1.12	Q3U9G9	Lamin-B receptor	Lbr		1936	22	22	22	31.8	31.8	31.8	71.439	0.0000	149	40757000	316	16.53	16.52	17.11	17.02	16.38	16.66	16.67	16.82
0.361	0.16	1.12	Q8R0H9	ADP-ribosylation factor-binding protein GGA1	Gga1		3760	16	16	15	33.2	33.2	32.1	69.971	0.0000	323.31	39672000	340	16.72	16.53	16.85	17.13	16.44	17.07	16.60	16.49
0.500	0.16	1.12	P32921	Tryptophan--tRNA ligase, cytoplasmic;T1-TrpRS;T2-TrpRS	Wars		998	24	24	24	62	62	62	54.357	0.0000	323.31	144330000	587	17.92	17.84	18.10	18.19	17.66	18.05	18.07	17.63
0.272	0.16	1.12	O35343	Importin subunit alpha-3	Kpna4		358	17	17	12	37.2	37.2	28.4	57.922	0.0000	249.57	39838000	324	16.77	17.39	17.87	17.22	17.03	17.07	17.36	17.15
0.210	0.16	1.12	E9PVA8		Gcn1l1		221	79	79	79	39.5	39.5	39.5	293.02	0.0000	323.31	124280000	1358	16.53	16.16	17.41	16.52	16.40	16.43	16.89	16.27
0.107	0.16	1.11	Q9D2E2	Target of EGR1 protein 1	Toe1		4941	3	3	3	11.7	11.7	11.7	56.87	0.0000	21.574	1965600	36	14.10	13.99	14.79	13.50	13.09	13.80	15.27	13.58
0.412	0.16	1.11	P62806	Histone H4	Hist1h4a		1445	24	24	24	77.7	77.7	77.7	11.367	0.0000	323.31	11310000000	7987	23.70	24.11	23.75	23.55	23.64	23.30	23.66	23.88
0.072	0.16	1.11	Q3UGY8	Brefeldin A-inhibited guanine nucleotide-exchange protein 3	Arfgef3		1964	6	6	6	4.6	4.6	4.6	240.09	0.0000	28.411	2112500	30	13.32	13.18	15.39	12.80	12.16	13.71	14.58	13.62
0.122	0.16	1.11	Q99M87	DnaJ homolog subfamily A member 3, mitochondrial	Dnaja3		4459	12	12	11	31.9	31.9	30.2	52.443	0.0000	62.281	19243000	149	16.10	16.25	17.40	15.14	16.15	16.32	15.80	16.01
0.194	0.16	1.11	Q8CHP8	Phosphoglycolate phosphatase	Pgp		3528	15	15	14	65.4	65.4	64.5	34.54	0.0000	191.06	64310000	337	17.66	17.82	19.02	17.87	17.78	17.96	17.97	18.03
0.553	0.15	1.11	Q6GQT9	Nodal modulator 1	Nomo1		2514	32	32	32	35.9	35.9	35.9	133.42	0.0000	247.28	94310000	564	17.66	17.58	17.64	17.76	17.79	17.48	17.56	17.19
0.127	0.15	1.11	Q78WZ7	DNA-directed RNA polymerase I subunit RPA43	Twistnb		2729	2	2	2	8.2	8.2	8.2	36.721	0.0004	4.6358	2775000	30	14.66	14.64	14.27	14.66	14.86	14.36	15.22	13.17
0.239	0.15	1.11	Q3TGW2	Endonuclease/exonuclease/phosphatase family domain-containing prot	Eepd1		1841	11	11	11	26.9	26.9	26.9	62.953	0.0000	93.367	26538000	169	17.32	17.12	17.47	17.48	16.89	17.70	17.51	16.66
0.454	0.15	1.11	Q6PIC6	Sodium/potassium-transporting ATPase subunit alpha-3	Atp1a3		2643	56	36	31	52.2	37.1	32.8	111.69	0.0000	323.31	100880000	654	17.57	17.34	17.98	17.69	17.39	17.40	17.70	17.48
0.201	0.15	1.11	Q8R4C2	RUN and FYVE domain-containing protein 2	Rufy2		3868	8	6	6	14.9	12.4	12.4	70.092	0.0000	19.505	3836100	62	14.15	13.64	14.35	15.07	14.07	14.10	14.35	14.07
0.078	0.15	1.11	Q8R0G7	Protein spinster homolog 1	Spns1		3758	5	5	5	21.2	21.2	21.2	56.708	0.0000	135.47	9315000	91	14.94	15.18	17.28	14.72	15.63	16.00	14.21	15.66
0.722	0.15	1.11	Q01768	Nucleoside diphosphate kinase B	Nme2		1711	16	16	10	86.8	86.8	53.3	17.363	0.0000	232.08	733750000	1016	20.82	20.98	20.85	20.73	20.56	20.96	20.58	20.67
1.007	0.15	1.11	Q8K1X4		Nckap1l		3636	41	41	39	42.6	42.6	41.8	128.9	0.0000	323.31	135770000	953	17.31	17.44	17.54	17.55	17.16	17.35	17.42	17.31
0.052	0.15	1.11	Q6NXJ0	Protein WWC2	Wwc2		2539	4	4	3	5.1	5.1	3.5	132.62	0.0025	2.8228	6361200	15	16.61	16.10	14.85	16.24	16.16	17.08	16.94	13.02
0.161	0.15	1.11	O08908	Phosphatidylinositol 3-kinase regulatory subunit beta	Pik3r2		309	4	4	3	5	5	3.7	81.265	0.0022	3.0546	3066900	39	14.13	14.17	13.43	14.77	13.37	14.18	14.48	13.88
0.413	0.15	1.11	Q3UM45	Protein phosphatase 1 regulatory subunit 7	Ppp1r7		1997	20	20	20	47.6	47.6	47.6	41.291	0.0000	178.66	36308000	335	16.52	16.60	17.06	17.01	16.42	16.75	16.63	16.80
0.196	0.15	1.11	O08582	GTP-binding protein 1	Gtpbp1		275	22	22	22	40.3	40.3	40.3	72.3	0.0000	150.25	42214000	355	16.79	16.43	17.81	17.17	16.73	16.89	17.08	16.90
0.250	0.15	1.11	Q8K183	Pyridoxal kinase	Pdxk		3617	20	20	20	62.2	62.2	62.2	35.015	0.0000	323.31	327760000	839	19.48	19.07	20.08	19.39	19.38	19.57	19.47	19.00
0.274	0.15	1.11	P59999	Actin-related protein 2/3 complex subunit 4	Arpc4		1348	22	22	22	89.9	89.9	89.9	19.667	0.0000	220.75	366760000	886	19.39	18.90	19.93	19.56	19.49	19.28	19.28	19.13
0.195	0.15	1.11	Q9R1P4	Proteasome subunit alpha type-1	Psma1		5656	22	22	22	76.8	76.8	76.8	29.546	0.0000	323.31	304380000	809	19.21	19.17	20.32	19.53	19.43	19.77	19.36	19.07
0.183	0.15	1.11	Q8BYK6	YTH domain-containing family protein 3	Ythdf3		3302	15	15	10	32	32	25	63.961	0.0000	48.973	24954000	185	16.25	15.97	15.27	16.13	15.27	16.36	15.74	15.65
0.115	0.15	1.11	Q3U1Y4	DENN domain-containing protein 4B	Dennd4b		1906	9	9	9	9.3	9.3	9.3	164.74	0.0000	25.709	7232800	86	14.06	13.68	15.70	14.78	13.91	14.37	14.72	14.62
0.110	0.15	1.11	Q9CX11	rRNA-processing protein UTP23 homolog	Utp23		4743	4	4	4	22.5	22.5	22.5	28.38	0.0000	13.071	4128600	26	14.37	14.43	14.85	15.12	15.01	15.27	13.20	14.70
0.150	0.15	1.11	Q9CZN8	Glutamyl-tRNA(Gln) amidotransferase subunit A, mitochondrial	Qrsl1		4835	8	8	8	28.4	28.4	28.4	56.782	0.0000	130.89	7560500	123	14.91	14.71	14.35	14.72	14.44	15.28	14.79	13.59
0.415	0.15	1.11	O88844	Isocitrate dehydrogenase [NADP] cytoplasmic	Idh1		574	52	52	50	89.6	89.6	87.4	46.674	0.0000	323.31	1272500000	2627	20.05	19.87	20.41	20.26	19.71	20.12	20.01	20.16
0.195	0.15	1.11	Q80ZW2	Protein THEM6	Them6		2923	2	2	2	12.6	12.6	12.6	23.802	0.0000	6.6899	2294600	27	13.98	13.41	13.41	14.28	13.42	13.16	14.02	13.89
0.218	0.15	1.11	P61759	Prefoldin subunit 3	Vbp1		1389	8	8	8	48.5	48.5	48.5	22.435	0.0000	323.31	12554000	138	15.35	16.14	15.03	15.57	15.52	15.34	15.02	15.62
0.152	0.15	1.11	Q6P5D8	Structural maintenance of chromosomes flexible hinge domain-containir	Smchd1		2573	38	38	38	26.7	26.7	26.7	225.65	0.0000	201.55	33288000	323	15.76	15.31	16.89	15.61	15.58	16.03	15.84	15.54
0.173	0.14	1.11	Q9JM13	Rab5 GDP/GTP exchange factor	Rabgef1		5496	16	16	16	37.7	37.7	37.7	56.868	0.0000	41.393	10707000	105	14.29	15.25	15.48	14.38	14.69	15.03	14.60	14.49
0.472	0.14	1.10	Q80UG5	Septin-9		40057	2828	28	28	28	39.1	39.1	39.1	65.574	0.0000	323.31	108900000	709	17.46	17.36	17.96	17.50	17.34	17.46	17.51	17.39
0.182	0.14	1.10	P24063	Integrin alpha-L	Itgal		894	34	34	34	30.3	30.3	30.3	128.33	0.0000	250.14	80717000	520	17.11	16.59	15.75	16.87	16.32	16.30	16.62	16.52
0.113	0.14	1.10	Q14C51	Pentatricopeptide repeat domain-containing protein 3, mitochondrial	Ptcd3		1788	10	10	10	21.5	21.5	21.5	77.795	0.0000	28.699	8018300	61	15.27	15.50	15.24	15.55	15.26	13.95	16.00	15.80
0.259	0.14	1.10	Q8K4I3	Rho guanine nucleotide exchange factor 6	Arhgef6		3717	23	20	20	32.4	29.4	29.4	87.051	0.0000	219.3	34973000	322	15.92	16.17	16.41	16.41	15.96	15.61	16.31	16.47
0.326	0.14	1.10	Q9CWJ9	Bifunctional purine biosynthesis protein PURH;Phosphoribosylaminoimi	Atic		4718	42	42	42	87.8	87.8	87.8	64.217	0.0000	323.31	395010000	1574	18.65	18.42	18.04	18.69	18.23	18.54	18.42	18.04
0.498	0.14	1.10	P70122	Ribosome maturation protein SBDS	Sbds		1526	18	18	18	68.8	68.8	68.8	28.78	0.0000	205.26	46726000	330	16.68	16.41	16.65	16.49	16.36	16.66	16.50	16.13
0.187	0.14	1.10	O35855	Branched-chain-amino-acid aminotransferase, mitochondrial	Bcat2		406	17	17	17	49.9	49.9	49.9	44.127	0.0000	153.79	115220000	480	18.51	18.30	18.23	18.48	17.89	19.10	17.98	17.97
0.520	0.14	1.10	Q3UHD6	Sorting nexin-27	Snx27		1971	22	22	22	50.5	50.5	50.5	60.988	0.0000	106.99	31659000	281	16.15	15.85	16.35	16.10	16.02	16.07	15.76	16.03
0.047	0.14	1.10	Q9CPZ6	ORM1-like protein 3	Ormdl3		4547	1	1	1	9.8	9.8	9.8	17.476	0.0000	35.502	8986000	73	16.24	13.03	16.91	16.00	15.93	13.68	16.22	15.79

0.791	0.14	1.10	P42208	Septin-2		37500	1074	24	24	24	71.2	71.2	71.2	41.525	0.0000	323.31	195720000	751	19.21	18.89	19.19	18.97	18.85	18.89	19.03	18.93
0.255	0.14	1.10	P52196	Thiosulfate sulfurtransferase	Tst		1213	6	6	6	25.3	25.3	25.3	33.466	0.0000	19.65	12334000	93	15.89	15.76	15.91	15.29	15.74	15.98	15.24	15.34
0.145	0.14	1.10	E9QAM5	Helicase with zinc finger domain 2	Helz2		253	34	34	34	15.1	15.1	15.1	331.56	0.0000	158.93	27853000	207	15.83	15.41	15.98	16.26	15.28	15.18	16.59	15.86
0.376	0.14	1.10	Q9R111	Guanine deaminase	Gda		5644	17	17	17	61.2	61.2	61.2	51.012	0.0000	169.5	54545000	321	17.09	17.19	17.21	17.43	17.33	17.35	16.90	16.79
0.366	0.14	1.10	Q99MN9	Propionyl-CoA carboxylase beta chain, mitochondrial	Pccb		4469	25	25	25	71.9	71.9	71.9	58.408	0.0000	308.83	91355000	476	18.23	18.15	18.60	18.14	18.07	18.49	18.09	17.92
0.565	0.14	1.10	P68040	Guanine nucleotide-binding protein subunit beta-2-like 1;Guanine nucle	Gnb2l1		1513	29	29	29	87.7	87.7	87.7	35.076	0.0000	323.31	602030000	1108	20.41	20.44	20.27	20.00	20.26	20.05	20.21	20.04
0.345	0.14	1.10	Q8VE11	Myotubularin-related protein 6	Mtmr6		3981	20	20	20	35.8	35.8	35.8	70.932	0.0000	74.136	24833000	303	15.63	15.41	16.04	15.83	15.51	15.78	15.74	15.33
0.631	0.14	1.10	P24369	Peptidyl-prolyl cis-trans isomerase B	Ppib		896	24	24	24	75.9	75.9	75.9	23.713	0.0000	254.98	304520000	793	19.01	18.93	18.92	19.20	18.80	19.10	18.89	18.73
0.382	0.14	1.10	Q924K8	Metastasis-associated protein MTA3	Mta3		4295	14	11	9	29.6	25.5	20.8	67.076	0.0000	80.674	20205000	190	16.61	16.15	16.37	16.70	16.19	16.18	16.59	16.30
0.157	0.14	1.10	Q9JJZ4	Ubiquitin-conjugating enzyme E2 J1	Ube2j1		5440	1	1	1	5.3	5.3	5.3	34.989	0.0000	15.298	2273600	67	14.30	13.75	12.97	14.19	13.30	13.73	13.99	13.63
0.124	0.14	1.10	Q4JIM5	Abelson tyrosine-protein kinase 2	Abl2		2060	3	3	2	5.5	5.5	4.3	128.19	0.0000	38.145	2563000	59	13.86	13.90	13.66	13.49	13.22	12.78	14.65	13.71
0.227	0.14	1.10	O08807	Peroxiredoxin-4	Prdx4		302	15	13	13	67.9	65.3	65.3	31.052	0.0000	198.9	393340000	476	21.93	21.84	22.43	22.24	22.17	21.38	22.19	22.16
0.090	0.14	1.10	Q5DU37	Zinc finger FYVE domain-containing protein 26	Zfyve26		2099	12	12	12	7.3	7.3	7.3	282.96	0.0000	46.254	5982800	65	13.69	13.59	15.64	13.99	14.29	13.36	14.67	14.03
0.201	0.14	1.10	Q8R1A4	Dedicator of cytokinesis protein 7	Dock7		3782	36	34	34	22.4	21.2	21.2	241.44	0.0000	146.95	46772000	463	15.91	15.67	16.67	16.20	15.59	15.93	16.37	16.01
0.288	0.14	1.10	Q8JZR0	Long-chain-fatty-acid--CoA ligase 5	Acsl5		3580	28	26	26	51.1	48.6	48.6	76.205	0.0000	193.32	48689000	381	16.17	16.15	16.57	16.13	16.08	15.68	16.27	16.45
0.303	0.14	1.10	O88630	Golgi SNAP receptor complex member 1	Gosr1		552	12	12	12	60.4	60.4	60.4	28.489	0.0000	109.93	25089000	175	16.63	16.32	17.01	17.01	16.34	16.66	16.76	16.67
0.212	0.14	1.10	Q8BUK6	Protein Hook homolog 3	Hook3		3226	21	21	21	39	39	39	83.217	0.0000	125.38	132230000	253	20.31	20.16	21.03	20.31	20.00	20.63	20.07	20.57
0.099	0.14	1.10	Q9JLN9	Serine/threonine-protein kinase mTOR	Mtor		5486	26	26	26	15.6	15.6	15.6	288.79	0.0000	137.44	21247000	250	15.11	14.71	16.95	15.39	15.20	15.55	15.64	15.24
0.192	0.14	1.10	P70372	ELAV-like protein 1	Elavl1		1561	16	16	16	54.9	54.9	54.9	36.169	0.0000	220.73	71470000	395	17.52	17.39	18.35	17.48	17.15	17.87	17.41	17.78
0.145	0.14	1.10	Q3UH93	Plexin-D1	Plxnd1		1967	14	14	14	9.2	9.2	9.2	211.61	0.0000	43.683	18333000	96	16.24	15.78	17.06	16.29	16.73	16.24	15.57	16.29
0.730	0.13	1.10	P68372	Tubulin beta-4B chain	Tubb4b		1518	39	39	2	77.3	77.3	3.6	49.83	0.0000	323.31	2639800000	3938	21.24	21.28	21.09	21.15	20.89	20.96	21.23	21.13
0.215	0.13	1.10	O08788	Dynactin subunit 1	Dctn1		299	56	56	56	49.2	49.2	49.2	141.67	0.0000	323.31	143300000	1079	17.19	17.05	17.93	17.30	16.97	17.68	17.18	17.09
0.445	0.13	1.10	Q8R317	Ubiquilin-1	Ubqln1		3830	7	7	5	25.9	25.9	21.6	61.976	0.0000	207.25	11255000	123	15.03	15.31	15.31	15.41	14.84	15.33	15.22	15.13
0.219	0.13	1.10	P40336	Vacuolar protein sorting-associated protein 26A	Vps26a		1061	22	22	21	70.9	70.9	68.8	38.113	0.0000	323.31	93534000	549	17.72	17.65	17.02	17.84	17.72	16.99	17.44	17.55
0.182	0.13	1.09	O88967	ATP-dependent zinc metalloprotease YME1L1	Yme1l1		589	20	20	20	33.6	33.6	33.6	80.027	0.0000	229.91	39202000	168	17.35	16.94	18.11	17.71	17.10	17.63	17.57	17.31
0.414	0.13	1.09	Q04899	Cyclin-dependent kinase 18	Cdk18		1737	15	14	11	35.3	33.5	26.8	51.847	0.0000	139.42	17989000	196	15.95	15.81	16.34	16.15	16.11	15.98	15.89	15.75
0.181	0.13	1.09	Q9CQC9	GTP-binding protein SAR1b	Sar1b		4585	13	9	9	57.6	46	46	22.382	0.0000	78.309	45409000	124	17.68	17.90	18.77	18.17	17.66	17.94	18.38	18.03
0.238	0.13	1.09	Q8BHZ0	Protein FAM49A	Fam49a		3053	14	12	12	45.5	38.7	38.7	37.342	0.0000	185.84	44913000	204	16.52	16.44	17.19	16.58	16.34	16.30	16.86	16.70
0.123	0.13	1.09	Q60809	CCR4-NOT transcription complex subunit 7	Cnot7		2210	3	3	3	15.8	15.8	15.8	32.718	0.0000	7.0855	2193100	15	14.18	13.74	14.16	14.13	12.90	14.71	13.99	14.10
0.169	0.13	1.09	Q8CIV8	Tubulin-specific chaperone E	Tbce		3563	10	10	10	31.3	31.3	31.3	59.085	0.0000	45.079	11860000	136	14.98	15.08	15.12	15.07	14.82	15.40	15.36	14.16
0.266	0.13	1.09	Q8K021	Secretory carrier-associated membrane protein 1	Scamp1		3596	10	10	10	36.4	36.4	36.4	38.028	0.0000	323.31	28814000	319	16.58	16.07	16.87	16.36	16.23	16.61	16.16	16.37
0.439	0.13	1.09	Q68FL4	Putative adenosylhomocysteinase 3	Ahcyl2		2476	23	23	14	40.5	40.5	26.8	66.899	0.0000	177.2	108130000	580	17.51	17.35	17.39	17.58	17.15	17.51	17.56	17.11
0.188	0.13	1.09	Q91XE8	Transmembrane protein 205	Tmem205		4145	4	4	4	21.2	21.2	21.2	21.18	0.0000	49.417	42392000	107	17.89	17.09	17.19	17.01	17.34	17.33	17.33	16.66
0.104	0.13	1.09	Q9WUA6	RAC-gamma serine/threonine-protein kinase	Akt3		5704	9	6	5	26.1	16.3	15.9	55.714	0.0000	19.579	1326000	24	13.70	12.82	13.76	13.36	14.31	12.88	12.51	13.43
0.132	0.13	1.09	Q9CPT3	N-acylneuraminate-9-phosphatase	Nanp		4532	6	6	6	31.9	31.9	31.9	27.808	0.0000	22.441	6825900	62	15.26	14.97	15.43	15.25	14.64	14.70	14.94	16.13
0.160	0.13	1.09	Q9JHI5	Isovaleryl-CoA dehydrogenase, mitochondrial	Ivd		5361	20	20	20	60.1	60.1	60.1	46.325	0.0000	165.62	87453000	525	17.69	17.45	16.42	17.45	17.11	17.43	17.03	16.92
0.216	0.13	1.09	Q9CWK8	Sorting nexin-2	Snx2		4719	39	39	37	58.2	58.2	54.1	58.47	0.0000	323.31	362350000	1251	18.78	18.41	19.41	18.70	18.49	18.81	18.92	18.58
0.105	0.13	1.09	Q9CZS1	Aldehyde dehydrogenase X, mitochondrial	Aldh1b1		4838	19	17	16	41.8	40.1	38.3	57.552	0.0000	100.26	31280000	270	16.38	16.09	14.99	15.39	16.02	15.57	16.04	14.73
0.110	0.13	1.09	P99024	Tubulin beta-5 chain	Tubb5		1691	37	7	6	73.2	20.5	17.1	49.67	0.0000	323.31	1078300000	824	22.16	21.96	20.59	21.92	21.81	20.88	21.82	21.62
0.098	0.12	1.09	Q8BMJ3	Eukaryotic translation initiation factor 1A, X-chromosomal	Eif1ax		3141	11	2	2	61.8	7.6	7.6	16.46	0.0008	4.1678	2012400	15	12.53	13.82	12.04	13.81	13.17	12.68	13.09	12.77
0.288	0.12	1.09	Q99KP3	Lambda-crystallin homolog	Cryl1		4387	21	21	21	90	90	90	35.208	0.0000	203.12	51094000	364	17.18	16.93	17.68	17.04	16.89	17.16	17.21	17.06
0.131	0.12	1.09	Q9R1R2	Tripartite motif-containing protein 3	Trim3		5660	7	7	7	16	16	16	80.774	0.0000	29.52	5068100	44	14.27	13.91	15.40	14.15	14.46	14.54	13.96	14.28
0.099	0.12	1.09	Q9Z130	Heterogeneous nuclear ribonucleoprotein D-like	Hnrnpdl		5798	12	10	10	52.5	48.2	48.2	33.558	0.0000	74.51	15793000	150	15.27	16.09	15.32	16.08	15.80	16.49	14.65	15.33
0.063	0.12	1.09	Q9Z321	DNA topoisomerase 3-beta-1	Top3b		5878	7	7	6	12.9	12.9	11.6	96.948	0.0000	20.786	4533300	47	13.74	13.47	15.51	14.50	13.83	12.90	14.91	15.09
0.226	0.12	1.09	Q8R3D1	TBC1 domain family member 13	Tbc1d13		3842	11	11	11	39.8	39.8	39.8	46.452	0.0000	47.531	40551000	296	16.72	16.39	16.45	16.77	16.38	16.03	16.97	16.48
0.184	0.12	1.09	Q8C4Q6	Axin interactor, dorsalization-associated protein	Aida		3387	6	6	6	28.5	28.5	28.5	34.888	0.0000	18.504	15125000	43	16.86	16.95	17.05	17.58	16.47	17.19	17.39	16.90
0.227	0.12	1.09	Q9DB25	Dolichyl-phosphate beta-glucosyltransferase	Alg5		5120	9	9	9	32.1	32.1	32.1	36.79	0.0000	58.919	29877000	195	16.79	16.73	16.40	17.19	16.75	16.61	16.97	16.29
0.127	0.12	1.09	Q6P1H6	Ankyrin repeat and LEM domain-containing protein 2	Ankle2		2555	13	13	13	22.2	22.2	22.2	106.2	0.0000	97.223	12144000	152	14.18	14.31	15.65	14.99	14.36	14.80	14.71	14.79
0.111	0.12	1.09	Q3UGR5	Haloacid dehalogenase-like hydrolase domain-containing protein 2	Hdhd2		1963	6	6	6	44.4	44.4	44.4	28.73	0.0000	56.04	21942000	168	17.17	16.60	15.74	16.98	16.65	16.52	17.00	15.84
0.178	0.12	1.09	Q8R3V5	Endophilin-B2	Sh3glb2		3858	19	18	18	57.8	57.8	57.8	44.503	0.0000	78.976	27387000	230	15.58	15.26	16.38	15.49	15.38	15.63	15.42	15.80
0.113	0.12	1.09	Q9EPJ9	ADP-ribosylation factor GTPase-activating protein 1	Arfgap1		5253	13	13	13	51	51	51	45.288	0.0000	131.12	25086000	214	16.18	15.78	17.44	16.01	15.93	16.56	16.22	16.22
0.837	0.12	1.09	Q02053	Ubiquitin-like modifier-activating enzyme 1	Uba1		1715	64	64	55	72.1	72.1	65.8	117.81	0.0000	323.31	944130000	2496	20.64	20.42	20.37	20.59	20.36	20.34	20.47	20.37
0.041	0.12	1.09	O35969	Guanidinoacetate N-methyltransferase	Gamt		415	9	9	9	52.5	52.5	52.5	26.336	0.0000	60.122	31695000	191	16.50	16.27	13.25	16.98	16.01	14.12	16.60	15.81
0.083	0.12	1.09	Q9D7H3	RNA 3-terminal phosphate cyclase	RtcA		5030	8	8	8	32.2	32.2	32.2	39.254	0.0000	51.267	6760700	81	13.93	14.72	16.17	14.50	14.42	14.35	14.92	15.14
0.088	0.12	1.09	O70494	Transcription factor Sp3	Sp3		508	3	2	2	3.3	2.2	2.2	82.361	0.0000	17.99	909160	38	13.30	12.80	13.59	14.02	12.32	13.59	13.06	14.28
0.123	0.12	1.08	Q8BMA6	Signal recognition particle subunit SRP68	Srp68		3130	35	35	35	60.3	60.3	60.3	70.573	0.0000	323.31	88518000	650	16.89	16.58	18.21	17.13	17.05	17.26	17.02	17.00
0.108	0.12	1.08	P06240	Proto-oncogene tyrosine-protein kinase LCK	Lck		656	2	1	1	2.8	2.8	2.8	57.942	0.0008	4.0969	1462500	27	13.70	12.07	12.96	12.91	12.45	12.55	13.43	12.75
0.239	0.12	1.08	O35857	Mitochondrial import inner membrane translocase subunit TIM44	Timm44		407	26	26	26	52.7	52.7	52.7	51.091	0.0000	137.99	67268000	601	16.39	16.35	17.04	16.53	16.13	16.70	16.49	16.52
0.141	0.12	1.08	Q99N94	39S ribosomal protein L9, mitochondrial	Mrpl9		4492	10	10	10	35.1	35.1	35.1	30.244	0.0000	23.118	13752000	118	15.26	15.55	14.99	15.96	15.53	14.63	15.47	15.68
0.379	0.12	1.08	Q8CGC7	Bifunctional glutamate/proline--tRNA ligase;Glutamate--tRNA ligase;Pro	Eprs		3507	111	111	111	74.7	74.7	74.7	170.08	0.0000	323.31	401510000	2651	17.70	17.54	18.02	18.00	17.65	17.66	17.89	17.60
0.141	0.12	1.08	A2AGT5	Cytoskeleton-associated protein 5	Ckap5		21	60	60	60	40.3	40.3	40.3	225.63	0.0000	323.31	74522000	711	15.79	15.86	16.88	16.30	15.69	15.93	16.56	16.17
0.219	0.12	1.08	Q9Z2A5	Arginyl-tRNA--protein transferase 1	Ate1		5836	12	12	12	27.9	27.9	27.9	59.146	0.0000	101.09	42147000	217	18.04	17.52	18.43	18.28	17.83	17.91	18.15	17.93
0.089	0.11	1.08	Q9CY21	Probable 18S rRNA (guanine-N(7))-methyltransferase	Wbscr22		4779	3	3	3	18.9	18.9	18.9	31.586	0.0000	44.267	4391900	57	14.40	14.56	16.29	15.09	14.48	15.02	15.40	14.98
0.262	0.11	1.08	O89103	Complement component C1q receptor	Cd93		603	6	6	6	10.7	10.7	10.7	69.354	0.0000	23.12	6009400	77	14.89	14.62	14.79	14.59	14.92	14.84	14.23	14.44
0.244	0.11	1.08	Q62167;P16	ATP-dependent RNA helicase DDX3X;Putative ATP-dependent RNA he	Ddx3x;D1Pas1		2360	52	51	20	78.9	78.9	37.9	73.101	0.0000	323.31	383660000	1355	19.21	19.01	19.76	19.57	19.22	19.48	19.07	19.33
0.160	0.11	1.08	Q8BVI4	Dihydropteridine reductase	Qdpr		3241	11	11	11	48.1	48.1	48.1	25.57	0.0000	241.64	117420000	449	18.29	17.98	17.38	18.27	17.83	18.11	18.13	17.39
0.132	0.11	1.08	O35711	Liprin-beta-2	Ppfibp2		401	18	18	17	27.9	27.9	26.5	98.752	0.0000	90.546	20461000	197	16.08	15.52	16.89	16.04	16.19	15.97	16.32	15.59
0.237	0.11	1.08	Q9ERG2	Striatin-3	Strn3		5310	11	10	8	16.2	14.9	13.6	87.149	0.0000	30.312	8013100	62	14.94	14.53	14.73	15.28	14.47	14.79	15.00	14.76
0.200	0.11	1.08	P58021	Transmembrane 9 superfamily member 2	Tm9sf2		1303	10	10	10	17.8	17.8	17.8	75.329	0.0000	38.027	13742000	124	15.73	15.04	15.02	15.52	14.94	15.21	15.58	15.13
0.253	0.11	1.08	P48193	Protein 4.1	Epb41		1141	26	26	23	30.1	30.1	27.7	95.91	0.0000	66.011	27489000	223	15.75	15.34	15.69	16.09	15.41	15.52	15.86	15.63
0.113	0.11	1.08	Q5SUF2	Luc7-like protein 3	Luc7l3		2146	16	16	16	32.4	32.4	32.4	51.45	0.0000	123.99	29109000	376	16.21	15.86	17.49	16.33	16.27	16.52	16.11	16.54
0.153	0.11	1.08	P16332	Methylmalonyl-CoA mutase, mitochondrial	Mut		809	20	20	20	47.2	47.2	47.2	82.843	0.0000	323.31	28162000	320	15.07	15.44	16.08	15.85	15.03	15.54	15.78	15.64
0.240	0.11	1.08	Q9CPX6	Ubiquitin-like-conjugating enzyme ATG3	Atg3		4541	11	11	11	37.6	37.6	37.6	35.796	0.0000	36.42	36175000	289	16.38	16.35	17.04	16.53	16.42	16.64	16.58	16.21
0.224	0.11	1.08	Q00612	Glucose-6-phosphate 1-dehydrogenase X	G6pdx		1699	56	56	44	79	79	68.7	59.262	0.0000	323.31	888940000	2038	20.21	20.21	19.62	20.52	19.86	20.12	20.08	20.06
0.138	0.11	1.08	Q9Z0Y1	Dynactin subunit 3	Dctn3		5792	13	13	12	61.8	61.8	61.8	20.978	0.0000	68.58	11096000	100	14.66	14.90	15.88	15.26	15.09	15.03	14.73	15.42
0.249	0.11	1.08	O88842	FYVE, RhoGEF and PH domain-containing protein 3	Fgd3		572	29	29	28	52.4	52.4	50.9	80.623	0.0000	323.31	76599000	584	17.08	16.68	16.94	17.12	16.51	16.68	17.08	17.12
0.082	0.11	1.08	O88622	Poly(ADP-ribose) glycohydrolase	Parg		550	5	5	5	6.1	6.1	6.1	109.32	0.0000	10.679	2371600	30	14.18	13.98	13.06	14.61	13.99	14.07	14.49	12.84
0.221	0.11	1.08	P47802	Metaxin-1	Mtx1		1124	11	11	11	39.7	39.7	39.7	35.623	0.0000	40.676	26014000	241	16.49	16.37	17.24	16.65	16.48	16.55	16.69	16.60
0.399	0.11	1.08	O55131	Septin-7		39326	458	30	30	29	56.9	56.9	56.9	50.549	0.0000	323.31	244110000	1255	17.78	17.54	18.00	17.81	17.55	17.88	17.65	17.62
0.138	0.11	1.08	Q8VD75	Huntingtin-interacting protein 1	Hip1		3944	40	40	39	51	51	50	115.2	0.0000	323.31	73852000	810	16.17	15.78	17.13	16.31	16.25	16.19	16.47	16.06
0.268	0.11	1.08	Q8C878	NEDD8-activating enzyme E1 catalytic subunit	Uba3		3422	16	16	16	51.3	51.3	51.3	51.737	0.0000	268.14	48214000	355	17.70	17.61	17.09	17.63	17.35	17.42	17.63	17.19
0.245	0.11	1.08	Q9Z1S8	GRB2-associated-binding protein 2	Gab2		5821	7	7	6	17.3	17.3	15	73.207	0.0000	40.424	7242200	97	15.11	14.84	14.67	15.18	14.64	15.08	15.06	14.58
0.077	0.11	1.08	Q9D0D3	Poly(A) RNA polymerase, mitochondrial	Mtpap		4859	4	4	4	14.4	14.4	14.4	65.229	0.0000	15.592	2042300	20	12.94	13.66	14.37	13.54	14.34	12.46	13.94	13.32
0.064	0.11	1.08	Q5SWT3	Solute carrier family 25 member 35	Slc25a35		2156	4	4	4	20.3	20.3	20.3	32.63	0.0000	37.571	1486400	24	13.53	13.93	15.18	13.51	13.04	14.35	14.98	13.36
0.147	0.11	1.08	Q9DB77	Cytochrome b-c1 complex subunit 2, mitochondrial	Uqcrc2		5131	27	27	27	52.8	52.8	52.8	48.234	0.0000	323.31	228330000	865	19.39	19.37	19.77	18.92	19.27	19.86	18.92	18.96
0.500	0.11	1.08	P39054	Dynamin-2	Dnm2		1051	52	52	38	55.1	55.1	44	98.144	0.0000	323.31	306160000	1468	18.88	18.80	18.73	19.05	18.55	18.87	18.82	18.78
0.673	0.11	1.08	P20060	Beta-hexosaminidase subunit beta	Hexb		852	28	28	27	48.5	48.5	47.2	61.115	0.0000	190.04	244440000	694	19.17	19.10	19.00	19.30	19.04	19.13	19.04	18.92
0.214	0.11	1.08	Q5SUA5	Unconventional myosin-Ig	Myo1g		2143	62	62	61	62.1	62.1	60.8	117.23	0.0000	323.31	349690000	1615	19.01	18.68	19.62	19.08	18.87	18.96	19.06	19.07
0.068	0.11	1.08	Q7TT37	Elongator complex protein 1	Ikbkap		2792	15	15	15	17.3	17.3	17.3	149.58	0.0000	67.699	14021000	145	15.07	14.93	16.85	14.45	14.92	15.51	15.57	14.86
0.548	0.11	1.08	Q9EQK5	Major vault protein	Mvp		5282	78	78	78	82.6	82.6	82.6	95.923	0.0000	323.31	457440000	1954	19.12	18.86	19.06	19.14	18.79	19.05	19.04	18.87
0.250	0.11	1.08	Q91V09	WD repeat-containing protein 13	Wdr13		4042	6	6	6	15.3	15.3	15.3	53.663	0.0000	17.733	5363200	98	14.82	14.11	14.69	14.52	14.20	14.45	14.57	14.49
0.155	0.11	1.08	Q3UJD6	Ubiquitin carboxyl-terminal hydrolase 19	Usp19		1984	21	21	21	29.6	29.6	29.6	150.55	0.0000	135.28	24951000	234	15.62	15.61	16.62	15.66	15.61	15.74	16.03	15.71
0.120	0.11	1.08	P62307	Small nuclear ribonucleoprotein F	Snrpf		1418	4	4	4	58.1	58.1	58.1	9.7251	0.0000	61.814	23341000	163	16.41	16.75	17.58	17.03	16.22	17.23	16.88	17.02
0.407	0.11	1.08	Q1HFZ0	tRNA (cytosine(34)-C(5))-methyltransferase	Nsun2		1795	31	31	31	47.7	47.7	47.7	85.451	0.0000	176.73	79640000	642	16.87	16.63	16.97	16.60	16.51	16.65	16.85	16.65
0.150	0.11	1.08	Q9DBS9	Oxysterol-binding protein-related protein 3	Osbpl3		5176	14	14	13	21.1	21.1	20	96.965	0.0000	50.922	10508000	111	14.49	14.71	15.51	15.15	14.69	14.56	15.18	15.01
0.041	0.11	1.08	Q9R0Q7	Prostaglandin E synthase 3	Ptges3		5638	6	6	5	39.4	39.4	32.5	18.721	0.0000	13.489	9969600	54	15.58	16.28	14.50	16.53	16.16	13.34	16.62	16.34
0.531	0.11	1.08	P40142	Transketolase	Tkt		1057	67	67	66	90.5	90.5	89.9	67.63	0.0000	323.31	4068900000	5169	22.21	22.03	21.99	22.30	21.92	22.00	22.18	22.01
0.245	0.11	1.08	Q8C708	Transmembrane protein C16orf54 homolog			3407	3	3	3	11.1	11.1	11.1	24.532	0.0000	8.2037	2484400	46	14.31	13.77	14.24	14.52	14.09	13.94	14.09	14.30
0.186	0.10	1.08	Q8CI71	Coiled-coil domain-containing protein 132	Ccdc132		3543	10	10	10	15	15	15	111.17	0.0000	97.473	17384000	176	15.55	15.36	16.00	15.90	15.28	15.35	15.87	15.89
0.040	0.10	1.08	Q80U95	Ubiquitin-protein ligase E3C	Ube3c		2827	5	5	5	7.7	7.7	7.7	123.97	0.0000	12.056	13068000	39	16.27	16.27	16.97	16.66	16.53	13.89	17.46	17.88
0.074	0.10	1.07	Q8CE50	Sorting nexin-30	Snx30		3467	16	16	16	51.5	51.5	51.5	49.52	0.0000	127.34	14962000	163	15.37	15.23	16.52	14.43	15.09	15.80	14.64	15.60
0.052	0.10	1.07	Q9D2R6	Cytochrome c oxidase assembly factor 3 homolog, mitochondrial	Coa3		4950	5	5	5	40.7	40.7	40.7	11.987	0.0000	21.38	13056000	68	16.50	15.40	17.34	15.16	14.57	16.48	16.32	16.62
0.258	0.10	1.07	Q99LF4	tRNA-splicing ligase RtcB homolog	Rtcb		4425	28	28	28	65.1	65.1	65.1	55.249	0.0000	323.31	159700000	887	17.82	17.55	17.87	17.72	17.71	17.91	17.72	17.21
0.134	0.10	1.07	Q64511	DNA topoisomerase 2-beta	Top2b		2448	45	45	35	32.7	32.7	26.9	181.91	0.0000	145	56095000	440	16.39	16.46	17.61	16.57	16.48	16.69	16.76	16.70
0.149	0.10	1.07	Q99PU8	Putative ATP-dependent RNA helicase DHX30	Dhx30		4517	18	18	18	19.1	19.1	19.1	136.67	0.0000	51.56	14488000	150	14.79	14.66	15.57	15.02	14.91	14.54	15.37	14.80
0.044	0.10	1.07	Q9Z183	Protein-arginine deiminase type-4	Padi4		5802	6	6	5	14.4	14.4	10.1	74.414	0.0000	56.309	6587100	46	12.30	15.49	15.47	15.16	14.71	15.05	14.28	13.96
0.056	0.10	1.07	Q9QXN3	Activating signal cointegrator 1	Trip4		5547	6	6	6	16.7	16.7	16.7	66.197	0.0000	33.93	4656600	56	14.26	14.36	15.88	13.96	14.81	13.18	15.49	14.58
0.300	0.10	1.07	P18654	Ribosomal protein S6 kinase alpha-3	Rps6ka3		840	32	19	18	50.7	35	33.2	83.693	0.0000	164.27	64341000	363	17.14	16.92	17.19	17.31	16.86	17.37	16.89	17.04

0.167	0.10	1.07	Q3TC93	HCLS1-binding protein 3	Hs1bp3	1825	12	12	12	48.4	48.4	48.4	43.691	0.0000	95.237	16284000	206	15.56	14.98	15.18	15.63	14.91	15.62	15.46	14.95
0.252	0.10	1.07	Q00262	Syntaxin-2	Stx2	1695	6	6	6	35.3	35.3	35.3	33.177	0.0000	285.76	14422000	184	15.98	15.74	15.73	16.02	15.43	15.63	15.89	16.10
0.202	0.10	1.07	Q9WTX2	Interferon-inducible double-stranded RNA-dependent protein kinase act	Prkra	5691	15	15	15	46	46	46	34.371	0.0000	46.063	28377000	202	16.48	16.09	16.54	16.40	16.37	16.71	15.92	16.11
0.028	0.10	1.07	A6H5Y3	Methionine synthase	Mtr	59	6	6	5	7.7	7.7	5.8	139.07	0.0000	24.006	5987600	24	15.38	15.96	12.71	15.01	15.65	15.09	16.19	11.73
0.086	0.10	1.07	Q9EPV8	Ubiquitin-like protein 5	Ubl5	5266	6	6	6	49.3	49.3	49.3	8.5468	0.0000	12.085	10098000	69	14.82	15.29	16.08	14.29	15.28	15.26	14.49	15.05
0.173	0.10	1.07	Q9CQZ0;Q9	ORM1-like protein 2;ORM1-like protein 1	Ormdl2;Ormdl1	4650	2	2	2	14.4	14.4	14.4	17.389	0.0000	42.929	20576000	100	17.25	17.07	17.99	17.34	17.10	17.44	17.53	17.20
0.171	0.10	1.07	P70303	CTP synthase 2	Ctps2	1551	24	24	22	50.3	50.3	46.8	65.514	0.0000	79.629	55084000	436	16.72	16.34	16.00	16.89	16.31	16.37	16.70	16.18
0.127	0.10	1.07	Q9JHU9	Inositol-3-phosphate synthase 1	Isyna1	5375	18	18	17	38.8	38.8	38.1	60.931	0.0000	295.85	87690000	387	18.33	17.79	17.20	18.03	17.73	17.77	18.15	17.31
0.371	0.10	1.07	Q9DBG5	Perilipin-3	Plin3	5152	33	33	33	90.4	90.4	90.4	47.262	0.0000	323.31	193150000	1036	18.21	18.28	18.43	18.37	17.93	18.30	18.23	18.43
0.116	0.10	1.07	P51175	Protoporphyrinogen oxidase	Ppox	1193	15	15	15	46.8	46.8	46.8	50.87	0.0000	142.53	11764000	138	14.32	14.46	15.58	14.83	14.40	15.02	14.86	14.51
0.045	0.10	1.07	P03921	NADH-ubiquinone oxidoreductase chain 5	Mtnd5	631	2	2	2	5.4	5.4	5.4	68.474	0.0002	5.8973	773950	8	12.59	11.88	15.16	12.46	12.93	13.55	12.82	12.39
0.166	0.10	1.07	O70469	Docking protein 2	Dok2	502	11	11	11	32	32	32	45.522	0.0000	14.118	15459000	83	16.03	15.63	16.44	16.47	15.85	16.24	16.25	15.84
0.052	0.10	1.07	B2RR83	Probable ATP-dependent RNA helicase YTHDC2	Ythdc2	78	6	6	6	7	7	7	161.09	0.0000	9.7909	2252300	23	13.27	13.01	15.18	13.53	12.66	13.95	13.26	14.72
0.113	0.10	1.07	P15920	V-type proton ATPase 116 kDa subunit a isoform 2	Atp6v0a2	803	5	5	5	6.7	6.7	6.7	98.144	0.0017	3.2905	1508100	11	13.00	13.20	13.87	13.52	13.30	13.57	12.58	13.76
0.349	0.10	1.07	Q3U6Q4	Phosphoinositide 3-kinase regulatory subunit 6	Pik3r6	1930	9	9	9	18.9	18.9	18.9	84.662	0.0000	31.625	12490000	64	15.90	15.76	15.68	15.90	15.77	15.55	15.99	15.54
0.083	0.10	1.07	Q6WKZ8	E3 ubiquitin-protein ligase UBR2	Ubr2	2666	8	8	8	5.9	5.9	5.9	199.15	0.0000	94.846	7449300	74	14.67	14.47	16.33	15.17	14.94	15.06	15.23	15.02
0.029	0.10	1.07	Q60972	Histone-binding protein RBBP4	Rbbp4	2231	11	4	4	29.6	19.3	19.3	47.655	0.0000	72.561	8681700	57	16.93	16.09	12.97	16.98	16.91	13.97	15.36	16.35
0.063	0.10	1.07	P09055	Integrin beta-1	Itgb1	692	16	16	16	20.2	20.2	20.2	88.231	0.0000	295.31	75682000	352	17.31	17.20	19.18	16.81	17.45	17.27	17.88	17.52
0.145	0.10	1.07	Q9CY64	Biliverdin reductase A	Blvra	4788	30	30	30	80.7	80.7	80.7	33.524	0.0000	323.31	270970000	913	19.45	18.99	18.40	19.35	19.00	19.02	19.08	18.71
0.064	0.10	1.07	Q9WVR4	Fragile X mental retardation syndrome-related protein 2	Fxr2	5760	11	9	9	22.4	18.4	18.4	73.742	0.0000	62.064	5863800	106	14.83	14.71	15.79	14.50	13.77	15.37	14.54	15.77
0.086	0.09	1.07	Q9ET26	E3 ubiquitin-protein ligase RNF114	Rnf114	5355	6	6	6	28.4	28.4	28.4	25.745	0.0000	40.509	8372900	83	14.66	14.63	16.14	14.78	14.62	14.75	15.18	15.29
0.148	0.09	1.07	Q9D7X3	Dual specificity protein phosphatase 3	Dusp3	5043	9	9	9	69.2	69.2	69.2	20.472	0.0000	323.31	120590000	426	19.25	19.02	18.42	19.20	18.84	19.11	19.08	18.47
0.206	0.09	1.07	Q9CXT8	Mitochondrial-processing peptidase subunit beta	Pmpcb	4766	19	18	18	50.1	48.7	48.7	54.614	0.0000	195.45	52843000	331	16.71	16.28	16.84	16.62	16.32	16.92	16.42	16.42
0.186	0.09	1.07	Q9ERS5	Pleckstrin homology domain-containing family A member 2	Plekha2	5318	8	8	8	24.7	24.7	24.7	47.379	0.0000	108.13	6224200	78	14.94	14.57	14.59	15.10	14.52	14.80	15.08	14.43
0.405	0.09	1.07	Q60864	Stress-induced-phosphoprotein 1	Stip1	2214	51	51	51	74.6	74.6	74.6	62.581	0.0000	323.31	244710000	1268	17.87	17.92	17.91	18.27	17.91	17.90	17.79	17.99
0.156	0.09	1.07	Q9ERL7	Glia maturation factor gamma	Gmfg	5314	10	8	8	58.5	48.6	48.6	16.748	0.0000	118.55	35550000	214	16.36	16.53	17.17	17.03	16.39	16.98	16.72	16.63
0.220	0.09	1.07	O35593	26S proteasome non-ATPase regulatory subunit 14	Psmd14	381	13	13	13	55.8	55.8	55.8	34.577	0.0000	322.53	144240000	531	18.63	18.59	18.31	18.42	18.42	18.72	18.45	17.99
0.054	0.09	1.07	Q8C3R1	BRCA1-associated ATM activator 1	Brat1	3379	4	4	4	8.8	8.8	8.8	89.085	0.0000	36.496	2504000	34	13.51	13.86	15.91	13.91	13.83	13.98	14.97	14.03
0.102	0.09	1.07	Q9JKY5	Huntingtin-interacting protein 1-related protein	Hip1r	5465	5	4	4	5.5	4.6	4.6	119.43	0.0064	2.0909	65444000	29	18.33	18.79	18.52	19.02	18.38	18.16	18.32	19.44
0.293	0.09	1.07	Q8BH04	Phosphoenolpyruvate carboxykinase [GTP], mitochondrial	Pck2	3005	36	36	35	68.4	68.4	67.2	70.527	0.0000	323.31	137210000	873	17.76	17.59	17.43	17.67	17.42	17.36	17.84	17.46
0.122	0.09	1.06	Q5Y5T1	Probable palmitoyltransferase ZDHHC20	Zdhhc20	2176	4	4	4	16.8	16.8	16.8	43.975	0.0000	45.802	6717600	77	15.07	14.38	14.87	14.97	14.42	14.27	15.25	14.98
0.061	0.09	1.06	P70671	Interferon regulatory factor 3	Irf3	1582	10	10	10	42.2	42.2	42.2	46.852	0.0000	88.753	8719100	92	15.42	15.47	13.77	14.57	14.14	14.97	15.55	14.21
0.153	0.09	1.06	Q6DFW4	Nucleolar protein 58	Nop58	2506	26	26	26	49.6	49.6	49.6	60.342	0.0000	301.66	86125000	627	16.93	16.73	17.58	16.65	16.93	16.71	17.08	16.81
0.190	0.09	1.06	Q8BYC6	Serine/threonine-protein kinase TAO3	Taok3	3297	60	60	53	49	49	45.1	105.33	0.0000	323.31	105930000	1078	16.55	16.71	17.33	16.63	16.66	16.61	16.83	16.76
0.923	0.09	1.06	P26041	Moesin	Msn	916	98	98	81	92.9	92.9	74.9	67.766	0.0000	323.31	5052600000	6590	21.48	21.40	21.38	21.35	21.24	21.42	21.32	21.27
0.095	0.09	1.06	Q7TSG2	RNA polymerase II subunit A C-terminal domain phosphatase	Ctdp1	2782	9	9	9	17.3	17.3	17.3	104.55	0.0000	72.164	6429000	92	14.09	14.03	15.18	13.85	13.73	14.27	14.45	14.34
0.210	0.09	1.06	P28474	Alcohol dehydrogenase class-3	Adh5	950	19	19	19	44.4	44.4	44.4	39.547	0.0000	199.56	123500000	555	17.34	17.24	17.58	17.24	17.25	17.57	17.35	16.87
0.116	0.09	1.06	P20491	High affinity immunoglobulin epsilon receptor subunit gamma	Fcer1g	856	4	4	4	32.6	32.6	32.6	9.6523	0.0000	77.006	240180000	223	20.13	19.45	19.54	20.45	19.48	19.73	20.20	19.82
0.104	0.09	1.06	P70206	Plexin-A1	Plxna1	1536	33	32	29	23	22.4	20.5	211.1	0.0000	223.13	36141000	327	16.30	15.97	17.24	16.40	16.73	16.18	16.57	16.08
0.090	0.09	1.06	A2AIV8	Caspase recruitment domain-containing protein 9	Card9	26	12	12	12	32.6	32.6	32.6	62.461	0.0000	77.716	19510000	144	16.11	16.04	17.35	16.47	15.89	16.74	16.42	16.58
0.129	0.09	1.06	Q6NVF9	Cleavage and polyadenylation specificity factor subunit 6	Cpsf6	2535	12	12	12	26.5	26.5	26.5	59.152	0.0000	323.31	52342000	378	17.53	17.59	18.29	17.55	17.75	17.15	17.74	17.96
0.085	0.09	1.06	Q64310	Surfeit locus protein 4	Surf4	2428	7	7	7	28.6	28.6	28.6	30.381	0.0000	172.66	25731000	197	16.46	16.36	17.43	16.17	16.13	16.54	17.17	16.24
0.248	0.09	1.06	Q8VDL4	ADP-dependent glucokinase	Adpgk	3958	11	11	11	32.1	32.1	32.1	53.902	0.0000	75.699	22349000	163	16.33	15.79	15.81	16.15	15.82	15.89	16.02	16.02
0.152	0.09	1.06	Q9DAA6	Exosome complex component CSL4	Exosc1	5106	4	4	4	28.2	28.2	28.2	21.423	0.0000	10.249	6443200	52	15.18	15.15	15.15	14.49	14.79	14.75	15.31	14.79
0.121	0.09	1.06	Q9R062	Glycogenin-1	Gyg1	5617	12	12	12	42	42	42	37.402	0.0000	264.98	60327000	313	17.91	17.85	16.86	17.92	17.67	17.52	17.51	17.50
0.162	0.08	1.06	Q8K3W0	BRCA1-A complex subunit BRE	Bre	3705	11	11	11	33.7	33.7	33.7	43.544	0.0000	82.551	22683000	136	16.53	16.09	16.35	16.69	15.99	16.57	16.62	16.14
0.046	0.08	1.06	P70227	Inositol 1,4,5-trisphosphate receptor type 3	Itpr3	1537	8	8	8	4.5	4.5	4.5	304.27	0.0000	62.845	5736000	67	15.27	14.84	13.06	15.04	13.93	13.92	15.61	14.41
0.172	0.08	1.06	E9PYK3		Parp4	224	19	19	19	15.2	15.2	15.2	216.13	0.0000	69.218	16761000	199	15.57	15.00	15.77	15.47	15.14	15.34	15.60	15.39
0.072	0.08	1.06	Q06180	Tyrosine-protein phosphatase non-receptor type 2	Ptpn2	1749	7	7	7	20.2	20.2	20.2	47.36	0.0000	44.3	4056100	44	13.68	14.49	13.58	13.60	12.74	13.78	14.20	14.29
0.078	0.08	1.06	Q8R2T8	General transcription factor 3C polypeptide 5	Gtf3c5	3820	6	6	6	14.4	14.4	14.4	60.501	0.0000	9.0236	2456600	33	13.58	13.10	14.04	13.76	13.10	14.46	13.55	13.03
0.053	0.08	1.06	O70293	G protein-coupled receptor kinase 6	Grk6	483	5	5	4	10.9	10.9	8.2	65.978	0.0000	47.872	6583500	77	15.42	14.97	16.89	15.87	14.84	15.59	16.62	15.77
0.079	0.08	1.06	E9Q612	Receptor-type tyrosine-protein phosphatase O	Ptpro	240	7	7	7	9.3	9.3	9.3	138.59	0.0000	71.17	10602000	92	15.52	15.43	16.61	15.79	15.49	15.47	16.55	15.51
0.174	0.08	1.06	Q9CRD2	ER membrane protein complex subunit 2	Emc2	4693	16	16	15	48.8	48.8	47.8	34.934	0.0000	112.05	41044000	251	16.59	16.63	17.06	16.64	16.50	17.08	16.44	16.58
0.139	0.08	1.06	Q60865	Caprin-1	Caprin1	2215	14	14	14	26	26	26	78.168	0.0000	121.42	19608000	217	15.57	16.02	15.93	15.64	15.67	16.04	15.17	15.95
0.108	0.08	1.06	P17225	Polypyrimidine tract-binding protein 1	Ptbp1	821	26	26	21	62.2	62.2	57.1	56.477	0.0000	323.31	345040000	1109	18.78	18.88	19.91	19.09	19.01	19.26	19.25	18.80
0.184	0.08	1.06	O55023	Inositol monophosphatase 1	Impa1	443	16	16	16	48.4	48.4	48.4	30.436	0.0000	123.7	116070000	412	18.46	18.30	19.07	18.60	18.48	18.64	18.56	18.44
0.101	0.08	1.06	Q922B9	Sperm-specific antigen 2 homolog	Ssfa2	4249	14	14	14	15.5	15.5	15.5	136.95	0.0000	32.451	10306000	109	14.41	14.54	14.96	13.84	14.00	14.30	14.85	14.27
0.178	0.08	1.06	O08992	Syntenin-1	Sdcbp	313	19	19	19	71.6	71.6	71.6	32.379	0.0000	227.1	65124000	408	17.04	17.27	16.70	17.06	16.56	17.12	16.98	17.09
0.138	0.08	1.06	Q8C8U0	Liprin-beta-1	Ppfibp1	3423	11	10	10	17	15.8	15.8	108.54	0.0000	85.138	5842400	74	14.93	14.23	14.35	14.51	14.14	14.82	14.50	14.23
0.101	0.08	1.06	Q8K1C9	Leucine-rich repeat-containing protein 41	Lrrc41	3624	5	5	5	9	9	9	88.179	0.0000	15.575	1993000	36	13.68	14.11	14.20	13.44	13.87	13.22	14.30	13.73
0.079	0.08	1.06	Q9D198	Pre-mRNA-splicing factor SYF2	Syf2	4902	9	9	8	46.3	46.3	45.9	28.712	0.0000	30.869	2378100	8	13.02	13.45	12.78	12.93	12.49	13.61	12.32	13.43
0.074	0.08	1.06	Q8VCX5	Calcium uptake protein 1, mitochondrial	Micu1	3936	11	11	11	27.5	27.5	27.5	54.352	0.0000	35.6	12843000	113	16.14	15.48	16.88	16.15	15.45	16.43	16.52	15.92
0.121	0.08	1.06	P84078;P61	ADP-ribosylation factor 1;ADP-ribosylation factor 3	Arf1;Arf3	1376	15	15	5	65.2	65.2	28.7	20.697	0.0000	323.31	213410000	574	19.43	19.80	19.74	19.02	19.51	18.99	19.42	19.76
0.252	0.08	1.05	P62196	26S protease regulatory subunit 8	Psmc5	1407	37	37	37	83.3	83.3	83.3	45.626	0.0000	323.31	205520000	1036	18.62	18.43	18.83	18.58	18.40	18.79	18.57	18.39
0.156	0.08	1.05	P59325	Eukaryotic translation initiation factor 5	Eif5	1334	21	21	21	45.7	45.7	45.7	48.968	0.0000	38.453	64459000	420	16.77	17.01	17.33	17.19	16.78	17.11	16.75	17.35
0.097	0.08	1.05	Q64430	Copper-transporting ATPase 1	Atp7a	2440	10	10	10	10.9	10.9	10.9	161.96	0.0000	104.92	8133400	147	15.07	14.63	14.58	14.97	14.65	15.13	15.13	14.03
0.084	0.08	1.05	Q8VDZ4	Palmitoyltransferase ZDHHC5	Zdhhc5	3977	4	4	4	7	7	7	77.5	0.0000	8.2244	1480600	21	13.17	13.13	14.28	12.87	13.52	13.43	13.09	13.11
0.122	0.08	1.05	Q9JM52	Misshapen-like kinase 1	Mink1	5498	20	20	10	19.4	19.4	12.3	147.29	0.0000	173.44	19606000	194	15.32	15.05	15.92	15.58	15.04	15.73	15.43	15.38
0.150	0.08	1.05	E9QAT4		Sec16a	254	23	23	23	17.4	17.4	17.4	254.2	0.0000	106.43	21990000	220	16.17	15.97	15.61	16.19	15.51	16.09	16.12	15.92
0.157	0.08	1.05	Q9CVD2	Ataxin-3	Atxn3	4707	6	6	6	22.5	22.5	22.5	40.533	0.0000	37.68	7811500	67	15.16	15.38	15.37	15.84	15.13	15.66	15.21	15.44
0.135	0.08	1.05	P27601	Guanine nucleotide-binding protein subunit alpha-13	Gna13	936	21	21	20	55.2	55.2	53.1	44.054	0.0000	218.56	179250000	517	19.84	19.69	19.15	19.93	19.58	19.45	19.90	19.38
0.072	0.07	1.05	P63028	Translationally-controlled tumor protein	Tpt1	1481	10	10	10	37.2	37.2	37.2	19.462	0.0000	70.794	41537000	224	17.11	17.78	16.17	17.36	17.12	17.33	16.66	17.00
0.216	0.07	1.05	O88543	COP9 signalosome complex subunit 3	Cops3	543	16	16	16	51.1	51.1	51.1	47.832	0.0000	323.31	36557000	303	16.75	16.46	16.81	16.73	16.47	16.84	16.77	16.37
0.098	0.07	1.05	Q3UFY8	Mitochondrial ribonuclease P protein 1	Trmt10c	1959	2	2	2	9.7	9.7	9.7	48.385	0.0000	47.374	2941200	50	13.74	14.55	14.32	14.39	13.64	14.24	14.69	14.14
0.032	0.07	1.05	Q6DFV7	Nuclear receptor coactivator 7	Ncoa7	2504	4	4	4	6.6	6.6	6.6	106.36	0.0000	6.3131	2807400	22	12.94	14.23	15.13	14.38	12.24	14.08	15.13	14.94
0.100	0.07	1.05	P70335	Rho-associated protein kinase 1	Rock1	1555	35	35	31	32.1	32.1	29.6	158.17	0.0000	207.31	35153000	533	15.28	15.30	16.38	15.51	15.38	15.67	15.54	15.59
0.035	0.07	1.05	Q8R3F9	Speckle targeted PIP5K1A-regulated poly(A) polymerase	Tut1	3845	5	5	5	11.6	11.6	11.6	94.602	0.0000	32.71	2772000	46	14.44	12.38	15.52	14.05	13.94	14.29	14.70	13.19
0.051	0.07	1.05	Q7M759	Alpha/beta hydrolase domain-containing protein 17B	Abhd17b	2739	3	3	3	19.4	19.4	19.4	32.2	0.0000	14.96	2942600	26	14.35	14.52	14.05	14.08	14.86	13.65	15.12	13.09
0.084	0.07	1.05	Q9JI75	Ribosyldihydronicotinamide dehydrogenase [quinone]	Nqo2	5389	12	12	12	41.1	41.1	41.1	26.248	0.0000	77.31	27001000	228	16.02	16.19	16.73	16.69	16.43	16.69	15.59	16.63
0.099	0.07	1.05	Q9CTG6	Probable cation-transporting ATPase 13A2	Atp13a2	4703	7	7	7	11.1	11.1	11.1	126.44	0.0000	96.804	8874900	61	14.77	15.00	14.46	14.85	14.89	14.88	15.04	13.99
0.086	0.07	1.05	P10630	Eukaryotic initiation factor 4A-II;Eukaryotic initiation factor 4A-II, N-termi	Eif4a2	725	23	7	7	52.6	23.8	23.8	46.402	0.0000	62.978	3608600	54	13.56	14.71	13.74	13.90	13.91	13.88	14.31	13.54
0.116	0.07	1.05	Q8BMS1	Trifunctional enzyme subunit alpha, mitochondrial;Long-chain enoyl-Co/	Hadha	3148	74	74	74	77.2	77.2	77.2	82.669	0.0000	323.31	522860000	2488	18.76	18.69	19.62	19.06	18.99	19.17	18.88	18.80
0.141	0.07	1.05	Q9QZQ1	Afadin	Mllt4	5603	39	39	39	30.4	30.4	30.4	206.5	0.0000	167.49	49910000	515	16.32	16.34	16.65	16.47	15.93	16.29	16.74	16.53
0.263	0.07	1.05	Q9JM76	Actin-related protein 2/3 complex subunit 3	Arpc3	5499	19	19	19	74.7	74.7	74.7	20.524	0.0000	245.03	253680000	649	19.81	20.15	19.69	20.07	19.88	19.87	19.82	19.89
0.054	0.07	1.05	E9Q555	E3 ubiquitin-protein ligase RNF213	Rnf213	236	149	149	149	35.8	35.8	35.8	584.78	0.0000	323.31	236780000	1777	16.62	16.31	18.18	17.18	16.74	16.68	17.39	17.20
0.038	0.07	1.05	Q9DBN5	Lon protease homolog 2, peroxisomal	Lonp2	5165	2	2	2	3.6	3.6	3.6	94.525	0.0002	6.1015	1850200	25	13.47	12.69	14.61	13.28	12.18	14.47	13.38	13.74
0.155	0.07	1.05	Q8BPB0;Q9	MOB kinase activator 1B;MOB kinase activator 1A	Mob1b;Mob1a	3163	8	8	8	37.5	37.5	37.5	25.091	0.0000	60.161	41792000	176	17.49	17.17	17.38	17.71	17.08	17.27	17.48	17.65
0.098	0.07	1.05	O70318	Band 4.1-like protein 2	Epb41l2	488	39	39	37	44.4	44.4	43.7	109.94	0.0000	278.5	94522000	626	16.90	16.82	17.83	16.96	16.87	17.29	17.08	17.00
0.040	0.07	1.05	Q8K2Y9	Cerebral cavernous malformations protein 2 homolog	Ccm2	3679	3	3	3	12.4	12.4	12.4	49.917	0.0000	6.6034	1900000	29	13.96	14.28	12.64	14.03	12.35	13.98	14.40	13.92
0.030	0.07	1.05	Q9D753	Exosome complex component RRP43	Exosc8	5020	6	6	6	29.7	29.7	29.7	29.949	0.0000	25.614	6160900	79	15.15	15.37	13.58	14.60	15.32	14.49	15.80	12.82
0.042	0.07	1.05	Q8BG07	Phospholipase D4	Pld4	2957	20	20	20	48.7	48.7	48.7	56.153	0.0000	194.84	188180000	581	19.55	19.80	17.59	19.61	19.37	18.49	19.16	19.27
0.159	0.07	1.05	O35295	Transcriptional activator protein Pur-beta	Purb	353	17	17	13	70.7	70.7	61.4	33.901	0.0000	301.33	62329000	446	17.60	17.37	17.93	17.88	17.38	17.70	17.63	17.82
0.130	0.07	1.05	Q8BUM3	Tyrosine-protein phosphatase non-receptor type 7	Ptpn7	3228	11	11	11	39.6	39.6	39.6	40.351	0.0000	86.873	22270000	159	16.51	16.46	17.17	16.60	16.46	16.75	16.46	16.82
0.074	0.06	1.05	Q9D2H6	Transcription factor Sp2	Sp2	4944	4	4	3	14.1	14.1	12.6	64.907	0.0000	29.01	2809200	44	14.55	14.64	14.86	15.07	13.88	14.80	15.23	14.95
0.070	0.06	1.05	P62305	Small nuclear ribonucleoprotein E	Snrpe	1417	5	5	5	59.8	59.8	59.8	10.803	0.0000	107.04	111310000	263	19.34	19.45	19.76	18.30	19.06	19.31	19.29	18.94
0.264	0.06	1.05	P52633	Signal transducer and transcription activator 6	Stat6	1222	24	24	24	49.6	49.6	49.6	93.496	0.0000	318.97	36951000	367	16.34	16.14	16.25	16.47	16.09	16.33	16.39	16.14
0.068	0.06	1.05	Q9WTX8	Mitotic spindle assembly checkpoint protein MAD1	Mad1l1	5693	9	9	9	17.3	17.3	17.3	83.54	0.0000	60.005	6467900	112	14.46	14.31	15.41	14.02	14.12	14.39	14.67	14.78
0.054	0.06	1.05	P35282	Ras-related protein Rab-21	Rab21	1015	14	14	14	56.3	56.3	56.3	24.106	0.0000	107.42	74175000	361	18.34	17.87	19.23	17.35	17.90	18.36	17.98	18.30
0.052	0.06	1.05	Q8K114	Integrator complex subunit 9	Ints9	3612	6	6	6	12.8	12.8	12.8	74.077	0.0000	13.897	2387100	21	13.16	12.96	14.65	13.18	13.71	13.54	13.56	12.87
0.113	0.06	1.05	Q8BG32	26S proteasome non-ATPase regulatory subunit 11	Psmd11	2962	34	34	34	78.7	78.7	78.7	47.436	0.0000	309.94	190220000	690	18.94	18.63	18.21	19.06	18.45	18.82	18.78	18.54
0.059	0.06	1.04	Q99KR6	E3 ubiquitin-protein ligase RNF34	Rnf34	4391	6	6	6	23.7	23.7	23.7	42.03	0.0000	19.865	2581900	22	13.65	13.51	14.94	14.04	13.65	14.20	14.41	13.63
0.128	0.06	1.04	Q5XG73	Acyl-CoA-binding domain-containing protein 5	Acbd5	2171	11	11	11	26.6	26.6	26.6	56.613	0.0000	41.914	15879000	106	15.33	15.13	15.68	15.56	14.96	15.55	15.41	15.54
0.119	0.06	1.04	Q6ZQI3	Malectin	Mlec	2697	17	17	17	51.2	51.2	51.2	32.342	0.0000	126.01	50723000	261	17.62	17.38	17.96	17.19	17.20	17.67	17.45	17.58
0.063	0.06	1.04	P49586	Choline-phosphate cytidylyltransferase A	Pcyt1a	1165	23	23	15	65.1	65.1	45.8	41.666	0.0000	156.04	57034000	529	16.23	16.42	17.68	16.37	16.44	16.74	16.51	16.76
0.124	0.06	1.04	Q9CQJ8	NADH dehydrogenase [ubiquinone] 1 beta subcomplex subunit 9	Ndufb9	4607	10	10	10	58.7	58.7	58.7	21.984	0.0000	52.294	18489000	123	15.44	15.51	15.95	15.92	15.56	15.37	16.00	15.64
0.066	0.06	1.04	Q64012	RNA-binding protein Raly	Raly	2412	25	25	25	56.4	56.4	56.4	33.188	0.0000	227.4	89783000	494	17.11	16.91	18.21	17.22	16.82	17.59	17.44	17.35
0.153	0.06	1.04	P61979	Heterogeneous nuclear ribonucleoprotein K	Hnrnpk	1398	37	37	35	72.8	72.8	67.2	50.976	0.0000	323.31	534240000	1400	19.85	20.43	20.37	20.16	19.97	20.21	20.06	20.32
0.061	0.06	1.04	Q9DCR2	AP-3 complex subunit sigma-1	Ap3s1	5225	7	7	7	37.8	37.8	37.8	21.732	0.0000	43.173	72934000	177	18.13	18.74	19.24	18.15	18.07	19.14	18.24	18.56

0.100	0.06	1.04	Q6P1B1	Xaa-Pro aminopeptidase 1	Xpnpep1	2552	30	30	30	64.5	64.5	64.5	69.59	0.0000	242.41	102480000	517	17.36	17.59	18.10	17.75	17.54	17.80	17.98	17.24
0.145	0.06	1.04	P57080	Ubiquitin carboxyl-terminal hydrolase 25	Usp25	1295	13	13	13	14.3	14.3	14.3	121.42	0.0000	64.067	12193000	188	15.07	15.14	15.14	15.32	14.72	15.18	15.07	15.45
0.049	0.06	1.04	P58501	PAX3- and PAX7-binding protein 1	Paxbp1	1318	5	5	5	8.8	8.8	8.8	104.83	0.0000	30.922	2410600	22	14.39	13.89	13.93	13.65	12.78	14.66	14.19	13.99
0.096	0.06	1.04	Q99LU0	Charged multivesicular body protein 1b-1	Chmp1b1	4447	6	6	2	20.6	20.6	5	22.124	0.0000	15.094	22317000	100	16.80	16.78	17.43	16.40	16.91	16.63	16.68	16.94
0.057	0.06	1.04	Q8BH95	Enoyl-CoA hydratase, mitochondrial	Echs1	3024	18	18	18	62.4	62.4	62.4	31.474	0.0000	323.31	209100000	806	18.17	18.27	19.00	17.95	18.67	18.89	17.72	17.87
0.046	0.06	1.04	P30993	C5a anaphylatoxin chemotactic receptor 1	C5ar1	981	4	4	4	7.4	7.4	7.4	39.023	0.0000	83.799	33417000	133	16.53	15.90	17.97	17.05	16.72	16.59	17.03	16.88
0.075	0.06	1.04	Q8CDG3	Deubiquitinating protein VCIP135	Vcpip1	3460	31	31	31	31.7	31.7	31.7	134.5	0.0000	180.18	47850000	438	15.99	15.68	16.84	16.29	15.91	16.43	16.33	15.88
0.076	0.06	1.04	Q9CQ22	Ragulator complex protein LAMTOR1	Lamtor1	4554	11	11	11	76.4	76.4	76.4	17.749	0.0000	277.03	77061000	372	17.72	17.20	18.37	18.11	17.60	17.79	18.11	17.65
0.143	0.06	1.04	Q91WS0	CDGSH iron-sulfur domain-containing protein 1	Cisd1	4125	6	6	6	42.6	42.6	42.6	12.097	0.0000	67.501	20745000	122	16.20	15.91	16.29	16.29	16.03	16.49	15.91	16.02
0.138	0.06	1.04	P18653	Ribosomal protein S6 kinase alpha-1	Rps6ka1	839	37	37	24	52.9	52.9	36.9	81.594	0.0000	234.71	91418000	731	16.71	16.73	16.66	17.22	16.62	17.04	16.72	16.71
0.194	0.06	1.04	Q9CXW4	60S ribosomal protein L11	Rpl11	4772	17	17	17	61.2	61.2	61.2	20.252	0.0000	171.44	430130000	530	20.45	19.90	20.29	20.31	20.22	20.15	20.21	20.13
0.183	0.06	1.04	Q9D0K2	Succinyl-CoA:3-ketoacid coenzyme A transferase 1, mitochondrial	Oxct1	4873	30	30	30	68.1	68.1	68.1	55.988	0.0000	323.31	420750000	1276	18.88	18.59	18.94	18.54	18.73	18.82	18.69	18.48
0.095	0.06	1.04	Q6PDL0	Cytoplasmic dynein 1 light intermediate chain 2	Dync1li2	2616	16	15	15	50.8	48.2	48.2	54.218	0.0000	190.58	37032000	289	16.18	16.41	16.96	16.57	16.08	16.79	16.58	16.44
0.028	0.06	1.04	Q80SU7	Interferon-induced very large GTPase 1	Gvin1	2794	86	86	86	41.3	41.3	41.3	280.81	0.0000	323.31	94693000	875	15.44	14.72	17.51	16.59	15.55	15.28	16.91	16.29
0.138	0.06	1.04	O88447	Kinesin light chain 1	Klc1	530	30	30	24	46.2	46.2	39.6	61.449	0.0000	135.45	36882000	405	15.99	15.91	16.40	15.92	15.75	15.98	16.01	16.25
0.031	0.06	1.04	Q4VBD2	Transmembrane anterior posterior transformation protein 1	Tapt1	2070	4	4	4	10.8	10.8	10.8	63.893	0.0002	6.1564	1539400	6	13.39	13.14	13.93	13.44	12.61	12.90	12.92	15.25
0.055	0.06	1.04	Q9ERG0	LIM domain and actin-binding protein 1	Lima1	5309	19	19	17	31.1	31.1	28.6	84.059	0.0000	44.918	22487000	206	16.33	16.09	17.57	16.11	16.26	16.54	16.57	16.49
0.060	0.06	1.04	Q99KX1	Myeloid leukemia factor 2	Mlf2	4399	4	4	4	23.5	23.5	23.5	28.055	0.0000	7.1161	2014300	18	14.05	13.47	14.13	13.30	13.48	14.46	13.39	13.39
0.079	0.06	1.04	Q9CPW4	Actin-related protein 2/3 complex subunit 5	Arpc5	4539	12	12	11	84.8	84.8	83.4	16.288	0.0000	323.31	192510000	460	18.81	19.11	19.57	19.20	19.10	19.68	18.92	18.77
0.137	0.06	1.04	P10107	Annexin A1	Anxa1	718	62	62	61	93.9	93.9	93.9	38.734	0.0000	323.31	3145400000	3743	22.34	22.16	22.78	22.55	22.44	22.59	22.30	22.28
0.270	0.05	1.04	P61161	Actin-related protein 2	Actr2	1372	45	45	44	80.2	80.2	80.2	44.76	0.0000	323.31	727520000	1648	20.44	20.17	20.45	20.44	20.33	20.22	20.45	20.27
0.097	0.05	1.04	Q7TQH0	Ataxin-2-like protein	Atxn2l	2774	12	12	12	17.6	17.6	17.6	110.65	0.0000	48.943	32251000	225	16.74	16.82	17.44	16.95	16.84	17.32	16.72	16.86
0.129	0.05	1.04	Q91VZ6	Stromal membrane-associated protein 1	Smap1	4086	9	8	8	18.4	16.6	16.6	47.66	0.0000	48.188	11150000	181	15.06	14.67	15.10	14.80	14.57	14.73	15.06	15.04
0.057	0.05	1.04	Q62261	Spectrin beta chain, non-erythrocytic 1	Sptbn1	2373	83	83	81	43	43	42.2	274.22	0.0000	323.31	131300000	1218	16.69	16.63	17.96	16.83	16.65	17.21	17.05	16.99
0.090	0.05	1.04	Q5SUR0	Phosphoribosylformylglycinamidine synthase	Pfas	2148	41	41	41	46.1	46.1	46.1	144.63	0.0000	323.31	98707000	913	16.98	16.78	17.63	17.12	16.81	17.15	17.37	16.97
0.027	0.05	1.04	Q9D6I7	Protein FAM69A	Fam69a	4994	3	3	3	9.6	9.6	9.6	48.936	0.0000	18.718	4125100	38	12.40	14.19	13.94	14.03	14.98	12.47	13.22	13.67
0.112	0.05	1.04	P46061	Ran GTPase-activating protein 1	Rangap1	1101	19	19	19	37	37	37	63.53	0.0000	169.66	37328000	324	16.50	16.21	16.47	15.96	16.02	16.20	16.57	16.13
0.035	0.05	1.04	Q8VD76	General transcription factor IIH subunit 3	Gtf2h3	3945	3	3	3	16.2	16.2	16.2	34.244	0.0000	9.4318	1156100	21	13.06	12.60	14.89	13.27	13.49	13.62	13.02	13.47
0.057	0.05	1.04	Q8BVW3	Tripartite motif-containing protein 14	Trim14	3250	21	21	21	51.8	51.8	51.8	49.64	0.0000	66.502	30606000	331	15.90	15.32	16.63	16.30	15.80	16.13	16.34	15.68
0.060	0.05	1.04	Q3TBD2	Minor histocompatibility protein HA-1	Hmha1	1819	57	57	57	47.2	47.2	47.2	122.9	0.0000	323.31	227150000	1475	17.55	17.50	18.55	17.71	17.50	18.24	17.78	17.57
0.081	0.05	1.04	Q8K337	Type II inositol 1,4,5-trisphosphate 5-phosphatase	Inpp5b	3687	5	5	5	6.7	6.7	6.7	112.76	0.0000	19.876	6953900	58	14.87	14.81	15.23	15.27	14.74	15.47	15.11	14.64
0.081	0.05	1.04	Q61542	StAR-related lipid transfer protein 3	Stard3	2308	7	7	7	27.6	27.6	27.6	50.469	0.0000	88.531	13160000	142	14.99	14.87	14.55	15.47	15.03	14.66	15.19	14.81
0.105	0.05	1.04	Q8CIB6	Transmembrane protein 230	Tmem230	3547	3	3	3	21.7	21.7	21.7	13.188	0.0000	8.3668	2666700	25	13.55	13.13	13.40	13.14	12.84	13.50	13.37	13.30
0.045	0.05	1.04	Q9ER73	Elongator complex protein 4	Elp4	5297	3	3	3	10.2	10.2	10.2	46.325	0.0000	57.774	4394400	83	15.05	13.94	14.35	15.18	14.49	14.23	15.34	14.27
0.057	0.05	1.04	Q9R061	Cytosolic Fe-S cluster assembly factor NUBP2	Nubp2	5616	6	6	5	32.7	32.7	26.2	29.518	0.0000	34.892	63192000	156	17.74	17.65	18.86	18.04	17.76	18.10	18.39	17.83
0.114	0.05	1.04	Q03265	ATP synthase subunit alpha, mitochondrial	Atp5a1	1724	69	69	69	79.7	79.7	79.7	59.752	0.0000	323.31	2838600000	4330	21.10	20.89	20.56	21.13	20.88	21.12	20.84	20.65
0.115	0.05	1.04	Q8R1V4	Transmembrane emp24 domain-containing protein 4	Tmed4	3802	10	8	8	48.5	37.9	37.9	26.022	0.0000	129.43	30032000	126	16.76	16.42	16.36	16.98	16.41	16.68	16.71	16.53
0.242	0.05	1.04	P62331	ADP-ribosylation factor 6	Arf6	1426	10	10	10	66.9	66.9	66.9	20.082	0.0000	156.51	40720000	299	16.79	16.74	16.70	16.82	16.50	16.69	16.83	16.83
0.039	0.05	1.03	Q9CZX0	Elongator complex protein 3	Elp3	4844	9	9	9	22.5	22.5	22.5	62.384	0.0000	25.11	10013000	106	15.48	15.24	16.97	15.91	16.17	15.41	16.28	15.54
0.039	0.05	1.03	P70444	BH3-interacting domain death agonist;BH3-interacting domain death ag	Bid	1575	5	5	5	34.4	34.4	34.4	21.951	0.0000	46.989	6643400	94	14.32	14.53	15.62	15.42	14.24	14.71	15.10	15.63
0.088	0.05	1.03	Q8BUB4	WD repeat and FYVE domain-containing protein 2	Wdfy2	3222	11	11	11	43.5	43.5	43.5	45.094	0.0000	54.28	13738000	114	16.11	15.83	15.45	15.73	15.39	15.98	15.57	15.98
0.040	0.05	1.03	Q9D1H8	39S ribosomal protein L53, mitochondrial	Mrpl53	4916	6	6	6	55.1	55.1	55.1	12.737	0.0000	41.869	9647600	103	15.22	15.58	16.63	15.79	15.08	16.36	15.44	16.16
0.170	0.05	1.03	Q8VDN2	Sodium/potassium-transporting ATPase subunit alpha-1	Atp1a1	3962	70	70	48	52.9	52.9	41.1	112.98	0.0000	323.31	860670000	2486	19.75	19.45	19.62	19.68	19.42	19.53	19.82	19.54
0.130	0.05	1.03	P38060	Hydroxymethylglutaryl-CoA lyase, mitochondrial	Hmgcl	1048	21	21	21	57.8	57.8	57.8	34.238	0.0000	323.31	119390000	617	19.06	19.03	19.58	19.10	19.12	19.18	19.24	19.03
0.054	0.05	1.03	P28741	Kinesin-like protein KIF3A	Kif3a	961	17	16	14	33.1	31.2	28.1	80.169	0.0000	169.48	24991000	237	16.14	16.01	17.16	15.81	16.17	16.21	16.20	16.35
0.071	0.05	1.03	P69566	Ran-binding protein 9	Ranbp9	1524	11	11	11	21.9	21.9	21.9	71.011	0.0000	75.449	19294000	164	15.78	15.65	16.00	15.36	15.56	15.97	15.92	15.14
0.058	0.05	1.03	P49962	Signal recognition particle 9 kDa protein	Srp9	1173	6	6	6	55.8	55.8	55.8	10.194	0.0000	10.554	7260000	33	14.93	16.18	15.88	15.89	15.51	15.86	15.61	15.71
0.026	0.05	1.03	Q9DCS9	NADH dehydrogenase [ubiquinone] 1 beta subcomplex subunit 10	Ndufb10	5228	7	7	7	38.1	38.1	38.1	21.024	0.0000	21.571	25402000	126	16.50	16.95	15.76	17.19	16.63	15.09	17.00	17.48
0.262	0.05	1.03	P80313	T-complex protein 1 subunit eta	Cct7	1590	47	47	47	77.2	77.2	77.2	59.652	0.0000	323.31	466630000	1631	18.51	18.38	18.66	18.43	18.41	18.56	18.40	18.42
0.042	0.05	1.03	Q8BSS9	Liprin-alpha-2	Ppfia2	3192	4	4	4	4.4	4.4	4.4	143.23	0.0002	5.6333	3119600	31	14.44	14.28	13.74	13.60	14.38	13.10	14.49	13.91
0.069	0.04	1.03	Q9Z0G0	PDZ domain-containing protein GIPC1	Gipc1	5768	8	8	8	30.9	30.9	30.9	36.129	0.0000	25.665	12469000	158	15.10	14.89	15.29	15.44	14.83	15.62	14.80	15.29
0.050	0.04	1.03	Q99104	Unconventional myosin-Va	Myo5a	4315	70	70	67	44.3	44.3	43.5	215.54	0.0000	323.31	128900000	1263	16.86	16.59	17.89	16.80	16.95	17.24	17.06	16.72
0.037	0.04	1.03	Q60902	Epidermal growth factor receptor substrate 15-like 1	Eps15l1	2220	16	16	16	27.7	27.7	27.7	99.307	0.0000	82.486	14381000	169	15.42	15.55	16.83	15.19	15.27	16.15	15.75	15.64
0.078	0.04	1.03	O08605	MAP kinase-interacting serine/threonine-protein kinase 1	Mknk1	281	8	8	8	26.2	26.2	26.2	47.914	0.0000	204.46	11815000	132	15.19	15.25	16.06	15.38	15.42	15.53	15.41	15.36
0.074	0.04	1.03	Q8R574	Phosphoribosyl pyrophosphate synthase-associated protein 2	Prpsap2	3881	18	18	16	60.2	60.2	56.4	40.88	0.0000	138.74	41018000	328	16.04	15.99	16.83	16.10	16.00	16.18	16.23	16.37
0.043	0.04	1.03	Q6QD59	Vesicle transport protein SEC20	Bnip1	2651	7	7	7	49.6	49.6	49.6	26.175	0.0000	103.54	16151000	139	15.66	15.74	16.49	15.16	15.33	16.19	16.03	15.34
0.078	0.04	1.03	Q9CR16	Peptidyl-prolyl cis-trans isomerase D	Ppid	4658	23	22	22	61.1	59.2	59.2	40.742	0.0000	149.69	82121000	622	17.33	17.24	18.01	17.44	17.21	17.61	17.46	17.58
0.030	0.04	1.03	Q3U214	Microtubule-associated serine/threonine-protein kinase 3	Mast3	1908	4	3	3	4.7	4.1	4.1	144.17	0.0000	9.7976	1159300	29	12.71	12.65	13.75	13.28	12.59	13.60	13.86	12.17
0.189	0.04	1.03	P05064	Fructose-bisphosphate aldolase A	Aldoa	647	52	52	12	98.9	98.9	30.5	39.355	0.0000	323.31	6032700000	5509	22.58	22.38	22.50	22.67	22.47	22.63	22.52	22.33
0.033	0.04	1.03	Q8BUV3	Gephyrin;Molybdopterin adenylyltransferase;Molybdopterin molybdenur	Gphn	3230	12	12	12	25.1	25.1	25.1	83.281	0.0000	62.68	15461000	146	15.07	15.11	16.81	15.18	15.22	15.85	15.45	15.47
0.043	0.04	1.03	O35099	Mitogen-activated protein kinase kinase kinase 5	Map3k5	336	5	5	3	5.1	5.1	3.3	154.51	0.0000	10.878	1863100	16	13.25	13.96	14.31	13.00	13.35	13.54	13.46	14.01
0.060	0.04	1.03	P59708	Splicing factor 3B subunit 6	Sf3b6	1341	5	5	5	37.6	37.6	37.6	14.585	0.0000	7.4121	10204000	71	15.50	16.13	15.16	15.72	15.61	15.68	15.19	15.86
0.053	0.04	1.03	Q8C570	mRNA export factor	Rae1	3394	11	11	11	35.1	35.1	35.1	40.965	0.0000	66.464	31999000	235	16.22	16.21	15.72	16.64	15.92	16.73	16.11	15.86
0.031	0.04	1.03	Q8VI93	2-5-oligoadenylate synthase 3	Oas3	4032	30	30	30	42.3	42.3	42.3	126.33	0.0000	323.31	121980000	607	17.65	17.26	18.64	18.39	17.61	17.18	18.62	18.37
0.052	0.04	1.03	Q9R233	Tapasin	Tapbp	5670	19	19	19	45.4	45.4	45.4	49.736	0.0000	232.77	124680000	538	17.65	17.33	18.53	17.96	17.73	17.56	17.96	18.05
0.060	0.04	1.03	Q8VC30	Bifunctional ATP-dependent dihydroxyacetone kinase/FAD-AMP lyase (	Dak	3907	10	10	10	31.1	31.1	31.1	59.69	0.0000	136.53	15875000	170	15.74	15.45	16.26	15.90	15.38	16.07	16.08	15.66
0.063	0.04	1.03	P62270	40S ribosomal protein S18	Rps18	1413	25	25	25	70.4	70.4	70.4	17.718	0.0000	323.31	526050000	1005	19.71	19.81	20.64	19.97	19.99	19.94	19.92	20.13
0.059	0.04	1.03	Q3UHJ0	AP2-associated protein kinase 1	Aak1	1974	16	16	15	30.9	30.9	30.2	103.35	0.0000	82.475	30755000	256	16.40	16.40	16.50	16.68	16.27	17.01	16.56	15.99
0.116	0.04	1.03	Q99JY9	Actin-related protein 3	Actr3	4358	44	44	44	85.2	85.2	85.2	47.357	0.0000	323.31	1385700000	2163	21.18	21.16	21.55	21.44	21.15	21.47	21.33	21.22
0.032	0.04	1.03	O08734	Bcl-2 homologous antagonist/killer	Bak1	290	6	6	6	40.7	40.7	40.7	23.294	0.0000	85.702	11028000	105	14.99	15.56	14.10	15.33	14.36	15.01	15.51	14.95
0.030	0.04	1.03	O70404	Vesicle-associated membrane protein 8	Vamp8	494	9	9	9	59.4	59.4	59.4	11.451	0.0000	120.87	114930000	272	17.77	17.50	19.38	17.88	17.99	18.12	18.13	18.15
0.032	0.04	1.03	Q8K019	Bcl-2-associated transcription factor 1	Bclaf1	3595	24	24	24	26.6	26.6	26.6	106	0.0000	48.928	43874000	405	16.35	16.22	17.15	16.52	16.06	16.94	15.87	17.22
0.034	0.04	1.03	P57746	V-type proton ATPase subunit D	Atp6v1d	1297	17	17	17	68	68	68	28.369	0.0000	323.31	88498000	404	17.92	18.37	19.35	17.73	18.45	18.30	18.35	18.13
0.033	0.04	1.03	Q61122	NGFI-A-binding protein 1	Nab1	2254	4	3	3	10.1	8.2	8.2	54.009	0.0000	7.6503	2303000	37	14.22	13.88	12.96	14.28	13.42	14.31	14.04	13.44
0.064	0.04	1.03	P62774	Myotrophin	Mtpn	1444	6	6	6	68.6	68.6	68.6	12.861	0.0000	198.68	24789000	233	16.86	17.06	16.43	17.15	16.70	16.84	16.64	17.18
0.026	0.04	1.02	Q9D0R4	Probable ATP-dependent RNA helicase DDX56	Ddx56	4884	8	8	8	24.5	24.5	24.5	61.211	0.0000	36.58	8928200	80	14.40	14.34	15.35	14.88	14.88	13.51	15.39	15.04
0.112	0.03	1.02	Q9D1A2	Cytosolic non-specific dipeptidase	Cndp2	4903	57	57	57	86.7	86.7	86.7	52.767	0.0000	323.31	2197000000	3173	20.85	20.78	20.47	20.89	20.74	20.69	20.86	20.55
0.073	0.03	1.02	Q9WUR2	Enoyl-CoA delta isomerase 2, mitochondrial	Eci2	5721	13	13	13	38.4	38.4	38.4	43.267	0.0000	58.743	14999000	144	15.67	15.96	15.56	15.90	15.55	16.00	15.45	15.97
0.191	0.03	1.02	Q3URS9	Coiled-coil domain-containing protein 51	Ccdc51	2025	8	8	8	24.6	24.6	24.6	45.132	0.0000	39.564	14587000	226	14.79	14.68	14.86	14.94	14.70	14.79	14.90	14.75
0.040	0.03	1.02	Q61550	Double-strand-break repair protein rad21 homolog	Rad21	2312	12	12	12	21.9	21.9	21.9	72.082	0.0000	59.918	23581000	245	15.60	15.45	16.48	15.85	15.39	15.78	16.28	15.80
0.112	0.03	1.02	Q9WV80	Sorting nexin-1	Snx1	5736	34	32	32	57.7	53.6	53.6	58.951	0.0000	323.31	199820000	823	18.20	18.03	17.99	18.33	17.92	18.27	18.05	18.17
0.153	0.03	1.02	Q6PHZ2	Calcium/calmodulin-dependent protein kinase type II subunit delta	Camk2d	2642	22	22	17	48.5	48.5	40.3	56.369	0.0000	296.59	104430000	529	18.00	17.75	18.03	17.72	17.82	17.88	17.84	17.84
0.060	0.03	1.02	Q3UEB3	Poly(U)-binding-splicing factor PUF60	Puf60	1954	15	15	15	46.3	46.3	46.3	60.248	0.0000	182	67210000	337	17.50	17.33	17.03	17.56	17.16	17.26	17.77	17.09
0.096	0.03	1.02	Q61166	Microtubule-associated protein RP/EB family member 1	Mapre1	2266	22	22	22	70.5	70.5	70.5	30.016	0.0000	323.31	171230000	704	18.32	18.44	18.15	18.61	18.17	18.50	18.46	18.26
0.034	0.03	1.02	Q8QZZ7	EKC/KEOPS complex subunit Tprkb	Tprkb	3739	5	5	5	45.7	45.7	45.7	19.556	0.0000	80.674	6296200	81	14.28	14.52	14.64	14.00	14.15	14.65	14.94	13.58
0.028	0.03	1.02	P70362	Ubiquitin fusion degradation protein 1 homolog	Ufd1l	1560	13	13	13	45.9	45.9	45.9	34.481	0.0000	48.029	38201000	208	16.81	16.84	18.10	16.38	16.93	17.36	17.02	16.70
0.069	0.03	1.02	O70493	Sorting nexin-12	Snx12	507	15	13	13	76.4	72.1	72.1	19.116	0.0000	112.77	83004000	375	17.60	17.50	17.84	17.59	17.69	17.71	17.19	17.82
0.042	0.03	1.02	P45377	Aldose reductase-related protein 2	Akr1b8	1095	21	21	17	64.9	64.9	56.3	36.12	0.0000	227.3	166980000	621	18.52	18.36	17.62	18.70	18.46	18.37	18.09	18.17
0.028	0.03	1.02	Q6GV12	3-ketodihydrosphingosine reductase	Kdsr	2516	4	4	4	14.8	14.8	14.8	35.955	0.0000	9.8791	12791000	69	15.39	14.82	16.47	15.63	15.27	15.48	15.78	15.66
0.053	0.03	1.02	P59017	Bcl-2-like protein 13	Bcl2l13	1329	5	5	5	18.7	18.7	18.7	46.719	0.0000	20.637	12931000	93	16.08	15.53	16.33	16.03	15.70	16.16	15.92	16.07
0.033	0.03	1.02	Q8C079	Striatin-interacting protein 1	Strip1	3327	6	6	6	12.7	12.7	12.7	95.584	0.0000	88.513	7492200	91	14.62	14.80	15.46	14.45	14.59	15.18	15.11	14.33
0.025	0.03	1.02	P51432	1-phosphatidylinositol 4,5-bisphosphate phosphodiesterase beta-3	Plcb3	1195	23	23	23	26.7	26.7	26.7	139.49	0.0000	81.994	21527000	197	15.31	15.11	16.76	15.51	15.32	15.93	15.69	15.64
0.097	0.03	1.02	Q91WD5	NADH dehydrogenase [ubiquinone] iron-sulfur protein 2, mitochondrial	Ndufs2	4106	15	15	15	44.3	44.3	44.3	52.625	0.0000	156.29	39572000	306	16.77	16.52	16.42	16.86	16.56	16.67	16.67	16.55
0.070	0.03	1.02	Q3TDQ1	Dolichyl-diphosphooligosaccharide--protein glycosyltransferase subunit	Stt3b	1834	18	18	17	22.2	22.2	22	93.245	0.0000	69.715	66506000	262	18.29	17.93	17.92	17.85	17.83	17.80	18.23	18.03
0.050	0.03	1.02	Q8VDV3	Guanine nucleotide exchange factor for Rab-3A	Rab3il1	3973	20	20	20	59.5	59.5	59.5	42.712	0.0000	258.44	27467000	317	16.05	15.74	16.51	15.93	16.11	16.07	16.18	15.77
0.037	0.03	1.02	Q921T2	Torsin-1A-interacting protein 1	Tor1aip1	4240	19	19	19	41.3	41.3	41.3	66.78	0.0000	304.81	78991000	420	17.40	17.14	18.18	17.40	17.15	17.62	17.68	17.56
0.036	0.03	1.02	P28656	Nucleosome assembly protein 1-like 1	Nap1l1	954	12	10	10	38.1	35.5	35.5	45.345	0.0000	120.88	62303000	311	17.71	17.85	18.55	17.55	17.70	17.64	18.11	18.11
0.029	0.03	1.02	Q9QX47	Protein SON	Son	5532	14	14	14	8	8	8	265.65	0.0000	43.305	16316000	134	15.36	15.23	16.37	15.15	15.47	15.11	15.73	15.69
0.105	0.03	1.02	Q9CPY7	Cytosol aminopeptidase	Lap3	4546	30	30	30	77.5	77.5	77.5	56.141	0.0000	323.31	159850000	927	17.66	17.47	17.62	17.71	17.40	17.77	17.60	17.60
0.007	0.02	1.02	Q6ZQK0	Condensin-2 complex subunit D3	Ncapd3	2699	4	4	4	5.4	5.4	5.4	169.43	0.0002	5.5244	26334000	45	17.22	13.67	16.45	16.77	16.21	13.47	16.59	17.74
0.026	0.02	1.02	Q91Z67	SLIT-ROBO Rho GTPase-activating protein 2	Srgap2	4188	39	39	35	42.4	42.4	40.3	120.8	0.0000	263.71	61761000	688	15.79	15.69	16.99	16.40	16.01	15.96	16.43	16.37
0.016	0.02	1.02	Q9D964	Glycine amidinotransferase, mitochondrial	Gatm	5097	17	17	17	55.8	55.8	55.8	48.297	0.0000	83.455	24088000	204	15.55	15.42	16.87	15.19	15.73	14.69	16.25	16.25
0.141	0.02	1.02	P35235	Tyrosine-protein phosphatase non-receptor type 11	Ptpn11	1011	25	25	25	46.4	46.4	46.4	68.034	0.0000	101.37	43099000	351	16.25	16.46	16.26	16.39	16.28	16.30	16.42	16.26
0.055	0.02	1.02	O35129	Prohibitin-2	Phb2	339	28	28	28	82.3	82.3	82.3	33.296	0.0000	323.31	305020000	851	19.03	18.77	19.16	19.18	19.07	19.28	18.87	18.84
0.026	0.02	1.01	Q9D8U8	Sorting nexin-5	Snx5	5076	49	49	47	85.1	85.1	83.7	46.797	0.0000	323.31	578660000	1635	20.61	20.30	21.58	20.73	20.62	20.84	20.91	20.77
0.054	0.02	1.01	P57776	Elongation factor 1-delta	Eef1d	1299	16	16	15	56.2	56.2	56.2	31.293	0.0000	323.31	95706000	428	17.77	17.69	17.81	17.73	17.48	18.03	17.51	17.88
0.040	0.02	1.01	Q571E4	N-acetylgalactosamine-6-sulfatase	Galns	2090	17	17	17	38.3	38.3	38.3	57.673	0.0000	246.09	65092000	396	17.35	17.31	17.16	17.30	17.76	17.23	17.12	16.93
0.033	0.02	1.01	Q4VBE8	WD repeat-containing protein 18	Wdr18	2071	10	10	10	28.3	28.3	28.3	47.211	0.0000	40.513	10879000	113	15.65	15.75	16.03	15.67	15.45	15.61	15.64	16.31

0.067	0.02	1.01	Q9R099	Transducin beta-like protein 2	Tbl2	5618	12	12	12	29.4	29.4	29.4	49.583	0.0000	72.98	19090000	240	15.75	15.54	15.72	15.77	15.49	15.70	15.91	15.60
0.025	0.02	1.01	O08600	Endonuclease G, mitochondrial	Endog	280	4	4	4	19	19	19	32.19	0.0000	11.942	3697100	30	14.43	14.67	14.07	14.04	14.30	14.50	14.65	13.68
0.061	0.02	1.01	Q64737	Trifunctional purine biosynthetic protein adenosine-3;Phosphoribosylam	Gart	2464	48	48	48	58.3	58.3	58.3	107.5	0.0000	323.31	183360000	1349	18.13	18.10	18.17	18.22	17.94	18.12	18.43	18.05
0.017	0.02	1.01	Q3UJB9	Enhancer of mRNA-decapping protein 4	Edc4	1983	23	23	22	26.9	26.9	26.6	152.48	0.0000	286.32	43908000	417	16.17	15.87	17.40	16.04	16.15	16.42	16.55	16.28
0.040	0.02	1.01	P22892	AP-1 complex subunit gamma-1	Ap1g1	877	16	16	16	24.6	24.6	24.6	91.349	0.0000	117.82	41284000	340	16.45	16.18	16.59	16.71	16.22	16.61	16.68	16.35
0.037	0.02	1.01	P61924	Coatomer subunit zeta-1	Copz1	1391	10	10	10	65.5	65.5	65.5	20.198	0.0000	255.35	76806000	360	18.21	18.12	18.44	17.76	18.06	18.32	18.17	17.91
0.022	0.02	1.01	Q8K136	Sodium channel modifier 1	Scnm1	3615	5	5	5	28.8	28.8	28.8	25.823	0.0000	12.788	3446600	34	14.47	14.30	14.61	14.06	14.30	13.78	14.36	14.93
0.017	0.02	1.01	Q8BGA9	Mitochondrial inner membrane protein OXA1L	Oxa1l	2971	7	7	7	13.9	13.9	13.9	48.219	0.0000	8.7987	7128200	105	14.49	14.60	15.89	14.54	14.90	14.78	14.99	14.78
0.012	0.02	1.01	P59279	Ras-related protein Rab-2B	Rab2b	1333	8	2	2	42.1	13.9	13.9	24.198	0.0000	9.4268	1056600	28	13.61	12.74	14.20	12.33	12.76	13.35	13.40	13.29
0.025	0.02	1.01	Q60631	Growth factor receptor-bound protein 2	Grb2	2186	22	22	22	94.5	94.5	94.5	25.238	0.0000	120.19	28312000	277	15.52	15.81	16.08	16.31	15.85	16.17	15.51	16.12
0.020	0.02	1.01	Q8VDM6	Heterogeneous nuclear ribonucleoprotein U-like protein 1	Hnrnpul1	3961	26	26	26	34.3	34.3	34.3	96.001	0.0000	204.83	56222000	485	16.22	15.99	17.03	16.39	16.03	16.48	16.35	16.70
0.042	0.02	1.01	Q9CZW5	Mitochondrial import receptor subunit TOM70	Tomm70a	4843	19	19	19	36.2	36.2	36.2	67.589	0.0000	212.27	29897000	330	15.85	15.96	15.90	15.76	15.78	15.76	16.21	15.66
0.013	0.02	1.01	Q8K2Q0	COMM domain-containing protein 9	Commd9	3667	6	6	6	51	51	51	21.85	0.0000	54.234	11382000	117	15.34	15.55	16.90	15.88	15.82	15.39	16.13	16.26
0.005	0.02	1.01	Q8BX90	Fibronectin type-III domain-containing protein 3A	Fndc3a	3281	10	10	10	11.6	11.6	11.6	131.96	0.0000	28.593	12556000	64	15.34	12.64	16.97	14.20	15.36	13.19	15.82	14.72
0.022	0.02	1.01	Q9QYJ0	DnaJ homolog subfamily A member 2	Dnaja2	5575	17	17	17	50.2	50.2	50.2	45.745	0.0000	96.681	42979000	357	16.17	16.33	16.74	15.61	16.12	16.30	16.04	16.33
0.046	0.02	1.01	Q59J78	Mimitin, mitochondrial	Ndufaf2	2094	7	7	7	51.8	51.8	51.8	19.628	0.0000	94.014	9179900	118	14.80	15.22	15.16	14.98	15.16	15.10	14.97	14.86
0.017	0.01	1.01	O89053	Coronin-1A	Coro1a	599	37	36	35	67.7	66.2	64.6	50.989	0.0000	323.31	873280000	1381	19.82	20.10	21.12	20.04	20.08	20.28	20.37	20.29
0.022	0.01	1.01	O35604	Niemann-Pick C1 protein	Npc1	385	11	11	11	8.2	8.2	8.2	142.88	0.0000	39.411	57186000	197	18.10	17.76	17.43	17.50	17.90	17.56	17.97	17.28
0.008	0.01	1.01	Q9D7A8	Armadillo repeat-containing protein 1	Armc1	5025	6	6	6	45	45	45	31.246	0.0000	130.33	10534000	92	16.05	15.98	14.68	14.86	15.42	14.04	16.06	15.99
0.020	0.01	1.01	P07091	Protein S100-A4	S100a4	667	13	13	13	64.4	64.4	64.4	11.721	0.0000	87.084	496300000	529	21.28	21.41	21.80	21.49	21.44	21.23	21.27	22.00
0.015	0.01	1.01	Q9Z2L7	Cytokine receptor-like factor 3	Crlf3	5858	9	9	9	31	31	31	49.558	0.0000	82.035	9415900	119	15.02	14.72	15.88	15.38	14.99	15.38	15.41	15.18
0.021	0.01	1.01	Q9R0P3	S-formylglutathione hydrolase	Esd	5631	29	29	29	96.1	96.1	96.1	31.319	0.0000	323.31	1515000000	1812	20.89	21.16	21.03	20.63	21.01	21.27	20.82	20.56
0.010	0.01	1.01	Q6A0D4	Raftlin	Rftn1	2502	25	25	25	51.3	51.3	51.3	61.536	0.0000	223.15	47251000	379	16.68	16.29	17.88	16.24	16.78	16.74	16.83	16.69
0.012	0.01	1.01	Q99JR8	SWI/SNF-related matrix-associated actin-dependent regulator of chroma	Smarcd2	4346	18	18	16	42.4	42.4	38.8	59.084	0.0000	99.723	34673000	252	16.11	16.00	17.02	15.68	16.07	16.14	16.30	16.25
0.025	0.01	1.01	P50516	V-type proton ATPase catalytic subunit A	Atp6v1a	1184	56	56	56	96.1	96.1	96.1	68.325	0.0000	323.31	1364500000	2659	20.63	20.46	20.22	20.51	20.61	20.20	20.63	20.35
0.004	0.01	1.01	Q8K3X4	Interferon regulatory factor 2-binding protein-like	Irf2bpl	3707	6	5	5	11.4	10.5	10.5	80.564	0.0000	20.402	7334000	77	15.10	15.39	13.26	15.15	14.90	15.18	15.62	13.17
0.017	0.01	1.01	P57784	U2 small nuclear ribonucleoprotein A	Snrpa1	1301	18	18	18	75.7	75.7	75.7	28.357	0.0000	323.31	38891000	280	16.55	16.19	16.79	16.12	16.39	16.64	16.46	16.13
0.011	0.01	1.01	Q9R257	Heme-binding protein 1	Hebp1	5671	8	8	8	71.6	71.6	71.6	21.067	0.0000	153.69	36223000	209	16.80	17.54	16.68	16.78	16.95	16.98	16.46	17.37
0.012	0.01	1.01	Q78IK2	Up-regulated during skeletal muscle growth protein 5	Usmg5	2722	4	4	4	44.8	44.8	44.8	6.3814	0.0000	21.442	12122000	112	15.53	16.02	14.93	15.79	15.32	15.81	15.61	15.49
0.009	0.01	1.01	Q9QYA2	Mitochondrial import receptor subunit TOM40 homolog	Tomm40	5565	9	9	9	36.3	36.3	36.3	37.895	0.0000	29.367	23668000	182	16.56	16.82	17.39	16.12	16.93	16.72	17.02	16.20
0.005	0.01	1.01	E9Q7D5		Arhgef5	246	4	4	4	2.8	2.8	2.8	176.66	0.0025	2.8085	29350000	46	16.25	18.07	18.25	18.88	17.54	18.41	17.45	18.02
0.006	0.01	1.01	Q3TVP5	Inactive ubiquitin thioesterase FAM105A	Fam105a	1879	5	5	5	11	11	11	41.576	0.0000	8.311	5101200	23	14.74	14.82	14.32	14.62	15.36	13.27	14.52	15.32
0.005	0.01	1.01	Q9CR95	Adaptin ear-binding coat-associated protein 1	Necap1	4683	5	5	5	24.4	24.4	24.4	29.639	0.0004	4.5864	5277600	27	14.41	15.20	13.26	15.19	14.84	13.30	15.02	14.86
0.005	0.01	1.01	P56671	Myc-associated zinc finger protein	Maz	1291	2	2	2	4.6	4.6	4.6	48.769	0.0000	6.2623	1507100	34	13.09	12.71	14.16	14.04	12.65	13.17	14.32	13.83
0.019	0.01	1.01	Q61655	ATP-dependent RNA helicase DDX19A	Ddx19a	2320	21	21	20	57.9	57.9	55.6	53.932	0.0000	274.42	70916000	448	17.16	17.06	17.15	17.41	17.00	17.40	17.37	16.97
0.017	0.01	1.01	Q9R0P5	Destrin	Dstn	5633	23	18	18	94.5	87.3	87.3	18.521	0.0000	91.881	69173000	388	18.18	17.84	18.17	17.79	17.86	18.29	17.75	18.05
0.006	0.01	1.01	Q8K330	Protein phosphatase Slingshot homolog 3	Ssh3	3686	13	13	13	32.2	32.2	32.2	72.227	0.0000	99.604	10938000	112	15.14	15.23	16.11	15.08	14.54	15.43	15.44	16.10
0.011	0.01	1.01	Q9D7B6	Isobutyryl-CoA dehydrogenase, mitochondrial	Acad8	5027	14	14	14	47.7	47.7	47.7	45.019	0.0000	105.8	31368000	256	16.16	16.14	15.75	16.23	16.06	16.30	16.36	15.53
0.008	0.01	1.00	Q8VDQ8	NAD-dependent protein deacetylase sirtuin-2	Sirt2	3968	18	18	17	52.2	52.2	48.3	43.256	0.0000	133.78	39199000	260	16.46	16.35	17.45	16.30	16.44	16.74	16.55	16.80
0.009	0.01	1.00	Q9CXY1	Transmembrane protein 175	Tmem175	4773	3	3	3	7.8	7.8	7.8	55.577	0.0000	44.838	2222500	39	13.41	12.90	12.33	13.13	13.22	12.76	12.93	12.84
0.023	0.01	1.00	P52480	Pyruvate kinase PKM	Pkm	1219	79	79	76	94.2	94.2	90	57.844	0.0000	323.31	8844600000	9714	23.15	23.00	22.91	23.23	23.10	22.86	23.16	23.13
0.017	0.01	1.00	Q8VDJ3	Vigilin	Hdlbp	3956	80	80	80	56.1	56.1	56.1	141.74	0.0000	323.31	327640000	1681	18.08	18.08	18.39	18.29	18.15	18.34	18.32	18.00
0.005	0.01	1.00	P28740	Kinesin-like protein KIF2A	Kif2a	960	19	19	18	30.6	30.6	29.4	79.755	0.0000	77.417	16098000	231	14.72	14.56	15.90	14.94	14.62	15.45	14.95	15.09
0.006	0.00	1.00	P70336	Rho-associated protein kinase 2	Rock2	1556	33	30	29	27.5	26.3	24.4	160.58	0.0000	102.15	46987000	346	16.57	16.46	17.57	16.78	16.58	17.11	16.99	16.68
0.006	0.00	1.00	Q8K310	Matrin-3	Matr3	3685	44	44	44	54.6	54.6	54.6	94.629	0.0000	323.31	210680000	1065	18.20	18.30	18.87	18.47	18.08	18.81	18.39	18.55
0.006	0.00	1.00	Q9Z1Q5	Chloride intracellular channel protein 1	Clic1	5818	24	24	24	79.7	79.7	79.7	27.013	0.0000	323.31	830680000	1199	21.29	21.08	20.75	21.18	20.97	21.15	21.37	20.80
0.008	0.00	1.00	Q58A65	C-Jun-amino-terminal kinase-interacting protein 4	Spag9	2093	38	38	30	33	33	28.4	146.22	0.0000	290	48435000	472	15.96	15.67	16.06	15.95	15.63	16.10	15.90	15.99
0.002	0.00	1.00	Q9CZR8	Elongation factor Ts, mitochondrial	Tsfm	4837	10	10	10	60.5	60.5	60.5	35.334	0.0000	166.76	16524000	135	16.07	16.23	14.96	16.52	16.37	16.73	16.29	14.37
0.001	0.00	1.00	Q9R049	E3 ubiquitin-protein ligase AMFR	Amfr	5613	9	9	9	21.3	21.3	21.3	73.104	0.0000	62.321	9293700	108	14.25	15.27	17.29	15.20	15.01	16.06	15.56	15.39
0.001	0.00	1.00	Q8BRG8	Transmembrane protein 209	Tmem209	3180	13	13	12	31.6	31.6	31.6	62.981	0.0000	55.374	11090000	111	15.79	15.30	14.36	15.79	14.98	15.46	15.55	15.24
0.001	0.00	1.00	P68368	Tubulin alpha-4A chain	Tuba4a	1516	33	9	9	58.7	16.5	16.5	49.924	0.0000	97.877	83518000	272	18.50	18.14	16.65	18.39	17.96	17.67	17.98	18.06
0.001	0.00	1.00	Q63829	COMM domain-containing protein 3	Commd3	2404	9	9	9	36.4	36.4	36.4	22.037	0.0000	66.616	16019000	166	16.00	16.61	15.87	16.46	16.14	15.99	16.22	16.60
0.000	0.00	1.00	Q9CYZ2	Tumor protein D54	Tpd52l2	4810	10	10	10	62.7	62.7	62.7	24.043	0.0000	65.213	19633000	128	16.50	16.85	16.47	16.96	16.61	17.02	15.79	17.35
0.000	0.00	1.00	Q8R2U4	N-terminal Xaa-Pro-Lys N-methyltransferase 1;N-terminal Xaa-Pro-Lys	Ntmt1	3822	3	3	3	15.7	15.7	15.7	25.42	0.0000	8.1252	6145400	85	15.45	15.60	13.53	12.13	14.80	12.20	15.85	13.86
0.003	0.00	1.00	P35564	Calnexin	Canx	1026	29	29	29	44.8	44.8	44.8	67.277	0.0000	284.93	228090000	533	19.15	18.99	18.53	18.95	18.92	18.71	19.15	18.83
0.000	0.00	1.00	Q9CY50	Translocon-associated protein subunit alpha	Ssr1	4784	3	3	3	17.1	17.1	17.1	32.065	0.0000	6.5913	4402400	47	14.33	16.24	12.56	13.25	14.89	13.61	14.76	13.12
0.002	0.00	1.00	Q9D187	Mitotic spindle-associated MMXD complex subunit MIP18	Fam96b	4901	6	6	6	57.1	57.1	57.1	17.667	0.0000	92.65	8879700	133	14.79	14.81	15.63	14.25	14.74	15.19	15.37	14.20
0.009	0.00	1.00	Q80U93	Nuclear pore complex protein Nup214	Nup214	2826	24	24	24	18.4	18.4	18.4	212.98	0.0000	144.68	31229000	413	15.83	15.79	16.14	15.94	15.59	16.05	16.07	16.00
0.003	0.00	1.00	Q99LB6	Methionine adenosyltransferase 2 subunit beta	Mat2b	4414	12	12	12	44	44	44	37.392	0.0000	79.441	23292000	162	15.68	16.04	17.46	15.97	16.05	16.49	16.29	16.34
0.005	0.00	1.00	Q9QYC0	Alpha-adducin	Add1	5568	17	17	16	30.6	30.6	29.5	80.646	0.0000	55.103	25075000	184	15.41	15.42	16.61	15.58	15.52	15.89	16.11	15.52
0.003	-0.01	1.00	Q80ZQ9	Protein Simiate	Fam206a	2919	5	5	5	46.6	46.6	46.6	21.597	0.0000	20.544	1162900	15	13.20	12.99	13.36	13.43	13.98	13.17	11.89	13.97
0.006	-0.01	1.00	Q9D7S7	60S ribosomal protein L22-like 1	Rpl22l1	5039	7	6	6	54.9	48.4	48.4	14.467	0.0000	100.15	32313000	163	16.54	16.68	17.62	16.87	16.69	16.79	17.07	17.19
0.006	-0.01	1.00	P47791	Glutathione reductase, mitochondrial	Gsr	1123	23	23	23	62.8	62.8	62.8	53.662	0.0000	307.74	180950000	488	20.17	19.94	19.85	19.91	19.90	20.71	19.89	19.38
0.011	-0.01	1.00	P08207	Protein S100-A10	S100a10	681	8	8	8	67	67	67	11.186	0.0000	68.213	127530000	357	18.51	18.55	18.87	18.65	18.67	18.23	18.81	18.90
0.005	-0.01	1.00	Q8CI32	BAG family molecular chaperone regulator 5	Bag5	3538	4	4	4	13.9	13.9	13.9	50.942	0.0000	19.078	4581100	76	14.27	14.75	14.33	14.70	14.50	13.73	15.31	14.53
0.003	-0.01	1.00	Q3U7U3	F-box only protein 7	Fbxo7	1933	3	3	3	10.9	10.9	10.9	57.648	0.0000	23.273	2393400	46	13.32	14.08	11.94	13.68	13.33	11.77	13.83	14.12
0.014	-0.01	1.00	Q9D358	Low molecular weight phosphotyrosine protein phosphatase	Acp1	4959	8	8	8	42.4	42.4	42.4	18.192	0.0000	82.917	22304000	172	16.37	16.31	16.52	16.33	16.23	16.83	16.29	16.21
0.013	-0.01	1.00	Q99J47	Dehydrogenase/reductase SDR family member 7B	Dhrs7b	4325	10	10	10	36.5	36.5	36.5	34.986	0.0000	67.839	25974000	276	15.80	15.70	15.97	15.79	16.14	15.87	15.91	15.36
0.003	-0.01	1.00	Q60996	Serine/threonine-protein phosphatase 2A 56 kDa regulatory subunit gar	Ppp2r5c	2236	10	8	8	23.5	17.9	17.9	60.824	0.0000	68.733	9164500	109	15.49	15.74	12.52	15.91	15.43	14.32	15.16	14.78
0.008	-0.01	0.99	Q61333	Tumor necrosis factor alpha-induced protein 2	Tnfaip2	2291	15	15	15	25.9	25.9	25.9	78.101	0.0000	100.43	33158000	276	16.54	16.23	15.58	16.53	16.46	16.60	16.29	15.57
0.015	-0.01	0.99	Q6Y5D8	Rho GTPase-activating protein 10	Arhgap10	2670	17	17	17	19.8	19.8	19.8	89.365	0.0000	33.322	8323000	83	15.49	15.03	15.79	15.32	15.43	15.60	15.38	15.25
0.011	-0.01	0.99	Q99J77		Nans	4330	25	25	25	73	73	73	40.024	0.0000	272.46	127790000	640	17.98	17.80	18.18	17.67	17.89	18.59	17.76	17.42
0.004	-0.01	0.99	Q64282	Interferon-induced protein with tetratricopeptide repeats 1	Ifit1	2427	29	29	28	66.1	66.1	65.9	53.737	0.0000	323.31	52123000	310	15.19	14.65	17.96	16.62	15.74	15.32	17.02	16.37
0.014	-0.01	0.99	Q9CPV4	Glyoxalase domain-containing protein 4	Glod4	4538	20	20	20	67.4	67.4	67.4	33.316	0.0000	269.38	92346000	586	16.79	17.07	17.81	17.11	16.93	17.23	17.12	17.54
0.021	-0.01	0.99	P48758	Carbonyl reductase [NADPH] 1	Cbr1	1149	25	24	24	87.4	84.5	84.5	30.641	0.0000	188.04	70303000	459	16.93	17.17	17.60	17.25	17.09	17.57	17.17	17.16
0.010	-0.01	0.99	Q8CHT3	Integrator complex subunit 5	Ints5	3532	2	2	2	2.5	2.5	2.5	108.35	0.0000	9.2417	1660400	17	12.80	13.26	14.09	13.54	13.36	13.91	13.74	12.73
0.010	-0.01	0.99	Q6P9R2	Serine/threonine-protein kinase OSR1	Oxsr1	2592	9	9	9	28.1	28.1	28.1	58.213	0.0000	134.92	21560000	180	15.78	16.17	15.72	14.62	15.70	15.28	16.15	15.21
0.008	-0.01	0.99	Q62418	Drebrin-like protein	Dbnl	2387	19	19	19	48.9	48.9	48.9	48.699	0.0000	162.64	52686000	314	18.25	18.32	16.33	18.52	17.77	18.00	17.77	17.93
0.018	-0.01	0.99	Q8BHY2	Nucleolar complex protein 4 homolog	Noc4l	3052	4	4	4	13.4	13.4	13.4	58.675	0.0000	18.542	1379100	24	13.47	14.25	14.08	13.83	13.70	14.38	13.98	13.63
0.008	-0.01	0.99	P61021	Ras-related protein Rab-5B	Rab5b	1364	11	7	7	66	47.9	47.9	23.707	0.0000	74.936	65302000	204	18.39	17.88	16.04	18.16	17.81	17.38	17.79	17.53
0.018	-0.01	0.99	Q7TMI3	E3 ubiquitin-protein ligase UHRF2	Uhrf2	2744	6	6	6	12.8	12.8	12.8	90.105	0.0000	29.682	8760700	55	15.52	15.58	16.39	15.52	15.85	15.45	16.02	15.74
0.008	-0.01	0.99	O88708	Origin recognition complex subunit 4	Orc4	561	2	2	1	6.9	6.9	3.7	49.981	0.0002	5.4064	1097100	16	13.23	13.09	14.32	13.34	13.58	14.49	13.67	12.29
0.005	-0.01	0.99	Q60649	Caseinolytic peptidase B protein homolog	Clpb	2189	7	7	7	14.6	14.6	14.6	76.003	0.0006	4.2979	2395500	18	14.04	13.95	14.63	11.47	12.85	14.55	14.24	12.50
0.017	-0.01	0.99	P63005	Platelet-activating factor acetylhydrolase IB subunit alpha	Pafah1b1	1478	27	27	27	65.4	65.4	65.4	46.67	0.0000	169.1	82874000	549	17.70	17.76	16.76	17.88	17.47	17.66	17.49	17.54
0.014	-0.01	0.99	Q9R0G7	Zinc finger E-box-binding homeobox 2	Zeb2	5625	14	14	14	16.9	16.9	16.9	136.61	0.0000	89.856	13854000	200	14.81	14.80	16.09	15.42	15.00	15.61	15.24	15.32
0.007	-0.01	0.99	Q9QXE7	F-box-like/WD repeat-containing protein TBL1X	Tbl1x	5539	7	5	5	24.3	16.5	16.5	56.802	0.0000	37.124	4639400	65	14.64	14.11	12.63	14.75	14.41	12.93	14.36	14.48
0.026	-0.01	0.99	Q9CZE3	Ras-related protein Rab-32	Rab32	4827	13	13	11	61	61	52.5	25.068	0.0000	323.31	113440000	488	18.17	18.41	17.59	18.11	17.86	18.23	18.12	18.14
0.024	-0.01	0.99	Q9EP53	Hamartin	Tsc1	5242	10	10	10	13.3	13.3	13.3	128.74	0.0000	17.613	4917500	51	14.57	14.35	14.55	14.59	14.71	14.39	14.99	14.03
0.016	-0.02	0.99	Q9D787	Peptidyl-prolyl cis-trans isomerase-like 2	Ppil2	5023	12	12	12	35.1	35.1	35.1	59.064	0.0000	72.429	16643000	175	15.53	15.40	14.45	15.95	15.10	15.33	15.45	15.51
0.037	-0.02	0.99	Q9CZY3	Ubiquitin-conjugating enzyme E2 variant 1	Ube2v1	4848	12	12	6	82.3	82.3	49	16.355	0.0000	201.03	131530000	351	18.94	18.63	18.31	18.79	18.62	18.81	18.75	18.57
0.023	-0.02	0.99	Q7TPH6	E3 ubiquitin-protein ligase MYCBP2	Mycbp2	2768	18	18	18	5.8	5.8	5.8	521.23	0.0000	47.665	16704000	133	16.31	16.03	16.87	16.49	16.34	16.76	16.60	16.06
0.018	-0.02	0.99	Q9QZD8	Mitochondrial dicarboxylate carrier	Slc25a10	5593	17	17	17	58.9	58.9	58.9	31.715	0.0000	116.3	37578000	179	16.85	16.95	16.38	16.77	16.75	16.06	16.82	17.39
0.038	-0.02	0.99	P39429	TNF receptor-associated factor 2	Traf2	1053	3	3	3	8.4	8.4	8.4	56.026	0.0000	33.883	9626600	53	15.78	15.67	16.31	16.06	15.82	16.01	16.01	16.05
0.009	-0.02	0.99	Q8BP92	Reticulocalbin-2	Rcn2	3161	2	2	2	6.2	6.2	6.2	37.27	0.0008	4.1802	834590	8	14.36	13.43	13.31	13.36	12.20	14.98	13.50	13.86
0.022	-0.02	0.99	Q9EQQ9	Protein O-GlcNAcase	Mgea5	5287	9	9	9	13.4	13.4	13.4	103.16	0.0000	40.826	10713000	104	15.71	15.33	15.25	16.27	15.82	15.91	15.70	15.20
0.007	-0.02	0.99	Q9CX30	Protein YIF1B	Yif1b	4745	5	5	5	35.4	35.4	35.4	33.982	0.0000	60.855	9865700	86	15.93	15.64	13.21	15.88	15.66	13.71	15.53	15.83
0.012	-0.02	0.99	Q921E2	Ras-related protein Rab-31	Rab31	4218	12	12	10	73.7	73.7	62.4	21.331	0.0000	168.58	38251000	255	17.37	17.07	15.71	16.66	17.26	15.85	17.43	16.35
0.018	-0.02	0.99	O35654	DNA polymerase delta subunit 2	Pold2	393	10	10	10	30.9	30.9	30.9	51.354	0.0000	44.819	7882500	95	15.18	14.74	15.87	16.09	15.49	15.78	15.61	15.06
0.034	-0.02	0.99	Q8VDC0	Probable leucine--tRNA ligase, mitochondrial	Lars2	3946	13	13	13	22.1	22.1	22.1	101.48	0.0000	83.804	10386000	117	14.31	14.05	14.28	14.62	14.08	14.33	14.78	14.15
0.022	-0.02	0.99	Q9Z2I0	LETM1 and EF-hand domain-containing protein 1, mitochondrial	Letm1	5855	25	25	25	37.9	37.9	37.9	82.988	0.0000	317.46	70968000	470	16.78	16.88	17.99	16.80	17.00	17.13	17.27	17.14
0.021	-0.02	0.99	Q8CBB9	Radical S-adenosyl methionine domain-containing protein 2	Rsad2	3436	8	8	8	27.9	27.9	27.9	41.523	0.0000	15.987	2567000	27	12.79	12.08	13.41	12.59	12.39	12.61	12.93	13.02
0.030	-0.02	0.99	Q64516	Glycerol kinase	Gk	2450	16	16	16	34.3	34.3	34.3	61.227	0.0000	66.337	17719000	142	15.34	15.34	16.23	15.72	15.60	15.87	15.49	15.75
0.028	-0.02	0.99	Q6NZM9	Histone deacetylase 4	Hdac4	2547	10	10	9	13.8	13.8	13.2	118.56	0.0000	27.361	5763100	48	14.13	14.44	14.08	14.64	14.23	13.79	14.73	14.62
0.030	-0.02	0.99	Q02105	Complement C1q subcomponent subunit C	C1qc	1716	6	6	6	29.7	29.7	29.7	25.991	0.0000	28.581	34675000	128	17.78	17.15	17.84	17.95	17.52	17.40	18.04	17.84
0.012	-0.02	0.99	Q91W39	Nuclear receptor coactivator 5	Ncoa5	4090	10	10	9	24	24	22.5	65.318	0.0000	21.478	9132200	99	14.08	14.70	16.75	14.75	15.11	15.46	14.67	15.12
0.032	-0.02	0.99	Q62318	Transcription intermediary factor 1-beta	Trim28	2379	36	36	36	46.6	46.6	46.6	88.846	0.0000	323.31	148910000	774	18.37	18.19	18.89	17.88	18.31	18.15	18.55	18.41
0.033	-0.02	0.99	Q6KCD5	Nipped-B-like protein	Nipbl	2522	16	16	15	8	8	6.6	315.45	0.0000	32.677	10698000	74	15.53	15.34	15.21	15.87	15.06	15.62	15.87	15.49
0.021	-0.02	0.98	Q9DBR0	A-kinase anchor protein 8	Akap8	5169	9	9	9	21.3	21.3	21.3	76.293	0.0000	41.582	31572000	66	17.99	17.78	16.79	18.29	17.33	17.87	18.13	17.61

0.024	-0.02	0.98	Q8BWU5	Probable tRNA N6-adenosine threonylcarbamoyltransferase	Osgep	3267	10	10	10	46.9	46.9	46.9	36.3	0.0000	67.894	18494000	184	16.20	15.87	15.04	16.33	15.72	16.28	16.01	15.52
0.032	-0.02	0.98	Q9R059	Four and a half LIM domains protein 3	Fhl3	5614	7	7	7	31.8	31.8	31.8	31.794	0.0000	11.865	10992000	65	15.63	16.07	16.69	16.05	16.01	16.45	15.86	16.21
0.034	-0.02	0.98	Q6P3D0	U8 snoRNA-decapping enzyme	Nudt16	2561	4	4	3	25.6	25.6	20	21.825	0.0000	17.761	2998500	39	12.97	13.94	13.94	13.93	13.68	13.85	13.68	13.65
0.067	-0.02	0.98	Q61990	Poly(rC)-binding protein 2	Pcbp2	2345	21	15	15	78.2	66.3	66.3	38.221	0.0000	264.14	112430000	395	17.71	17.95	18.16	18.06	17.83	18.11	18.16	17.87
0.023	-0.02	0.98	Q6P542	ATP-binding cassette sub-family F member 1	Abcf1	2567	32	32	31	44.4	44.4	44.4	94.944	0.0000	323.31	80225000	527	16.77	16.58	18.13	16.81	17.36	16.96	17.14	16.93
0.027	-0.03	0.98	Q3U0V2	Tumor necrosis factor receptor type 1-associated DEATH domain protei	Tradd	1898	6	6	6	37.1	37.1	37.1	34.577	0.0000	250.41	23856000	185	16.52	16.30	16.43	16.45	16.51	16.71	17.04	15.55
0.047	-0.03	0.98	P67778	Prohibitin	Phb	1508	32	32	32	94.5	94.5	94.5	29.82	0.0000	323.31	419520000	1111	18.67	18.38	19.06	18.77	18.55	18.92	18.99	18.52
0.012	-0.03	0.98	B8ZXI1	Queuine tRNA-ribosyltransferase subunit QTRTD1	Qtrtd1	91	5	5	5	17.3	17.3	17.3	46.288	0.0000	31.77	2213900	40	13.21	12.56	15.53	13.21	13.87	13.47	13.12	14.15
0.054	-0.03	0.98	Q8BRF7	Sec1 family domain-containing protein 1	Scfd1	3179	25	25	25	50.2	50.2	50.2	72.322	0.0000	202.58	78367000	571	16.67	16.66	16.39	16.82	16.37	17.01	16.77	16.50
0.033	-0.03	0.98	Q61712	DnaJ homolog subfamily C member 1	Dnajc1	2327	11	11	11	23.7	23.7	23.7	63.869	0.0000	32.258	8122400	75	14.25	14.37	13.53	14.13	13.51	14.16	14.36	14.35
0.021	-0.03	0.98	P47968	Ribose-5-phosphate isomerase	Rpia	1137	10	10	10	45.2	45.2	45.2	32.45	0.0000	74.886	14813000	144	15.71	15.64	17.08	15.16	15.64	16.16	16.05	15.85
0.066	-0.03	0.98	Q8BFR5	Elongation factor Tu, mitochondrial	Tufm	2945	35	35	35	77.2	77.2	77.2	49.508	0.0000	323.31	195990000	1130	17.76	17.99	18.11	17.62	17.80	18.01	17.67	18.12
0.034	-0.03	0.98	Q5NC05	Transcription termination factor 2	Ttf2	2116	10	10	10	12.8	12.8	12.8	125.53	0.0000	36.599	3795500	50	13.81	13.56	13.74	14.09	13.31	13.59	14.52	13.88
0.048	-0.03	0.98	P46460	Vesicle-fusing ATPase	Nsf	1104	49	49	49	69.4	69.4	69.4	82.613	0.0000	323.31	191710000	984	17.72	17.74	18.34	17.71	17.77	18.17	18.09	17.59
0.026	-0.03	0.98	O09047	C3a anaphylatoxin chemotactic receptor	C3ar1	320	5	5	5	10.3	10.3	10.3	53.575	0.0000	13.357	17388000	65	16.21	15.45	17.18	16.52	16.06	16.59	16.31	16.51
0.067	-0.03	0.98	Q9QXS1	Plectin	Plec	5548	472	472	466	78.1	78.1	77.2	534.18	0.0000	323.31	2618600000	12403	18.78	18.68	19.29	18.75	18.76	19.13	18.88	18.85
0.180	-0.03	0.98	P62874	Guanine nucleotide-binding protein G(I)/G(S)/G(T) subunit beta-1	Gnb1	1459	20	20	12	65.3	65.3	44.1	37.377	0.0000	323.31	339700000	911	19.32	19.46	19.27	19.34	19.31	19.51	19.28	19.42
0.023	-0.03	0.98	P62075	Mitochondrial import inner membrane translocase subunit Tim13	Timm13	1402	3	3	3	53.7	53.7	53.7	10.458	0.0000	44.621	2328900	20	12.93	14.01	15.00	14.51	13.83	14.33	14.16	14.26
0.055	-0.03	0.98	Q9CU62	Structural maintenance of chromosomes protein 1A	Smc1a	4704	78	78	78	58.9	58.9	58.9	143.23	0.0000	323.31	226530000	1423	18.03	17.99	18.77	18.30	18.02	18.37	18.42	18.40
0.021	-0.03	0.98	Q922J3	CAP-Gly domain-containing linker protein 1	Clip1	4260	12	12	10	10.4	10.4	9.3	155.81	0.0000	54.62	5278500	70	14.13	13.68	15.76	15.12	14.81	15.26	14.58	14.16
0.017	-0.03	0.98	Q921N8		Gps2	4236	4	4	4	12.8	12.8	12.8	36.737	0.0025	2.9007	8809200	53	15.51	14.82	15.33	13.64	14.64	13.52	15.74	15.53
0.013	-0.03	0.98	Q6PGH1	Protein BUD31 homolog	Bud31	2638	10	10	10	60.4	60.4	60.4	17	0.0000	19.105	13891000	73	16.83	16.68	14.65	16.49	16.68	17.12	17.02	13.97
0.029	-0.03	0.98	P52875	Transmembrane protein 165	Tmem165	1226	2	2	2	11.5	11.5	11.5	34.79	0.0000	15.74	6312100	72	15.20	15.03	13.53	14.79	14.72	14.55	15.06	14.37
0.061	-0.03	0.98	P05555	Integrin alpha-M	Itgam	652	45	45	45	40	40	40	127.48	0.0000	323.31	243250000	1075	18.17	18.11	18.60	18.41	18.71	18.02	18.57	18.12
0.036	-0.04	0.98	Q5U430	E3 ubiquitin-protein ligase UBR3	Ubr3	2163	5	5	5	3.4	3.4	3.4	212.75	0.0000	20.883	1206000	33	14.21	13.27	13.50	12.72	13.23	13.27	13.51	13.83
0.050	-0.04	0.98	Q6WVG3	BTB/POZ domain-containing protein KCTD12	Kctd12	2667	17	17	16	55.4	55.4	55.4	35.892	0.0000	322.4	156480000	624	18.94	18.73	17.93	18.63	18.62	18.69	18.81	18.26
0.060	-0.04	0.98	P48428	Tubulin-specific chaperone A	Tbca	1144	7	7	7	70.4	70.4	70.4	12.758	0.0000	57.961	18704000	146	16.20	16.16	16.85	16.55	16.37	16.57	16.16	16.80
0.020	-0.04	0.98	Q9CR76	Transmembrane protein 186	Tmem186	4679	2	2	2	13	13	13	24.631	0.0000	15.974	1018300	14	12.94	13.87	11.94	12.47	12.91	13.57	13.39	11.50
0.052	-0.04	0.97	Q8BSF4	Phosphatidylserine decarboxylase proenzyme;Phosphatidylserine deca	Pisd	3187	7	7	7	20.4	20.4	20.4	45.926	0.0000	28.85	4466600	46	13.58	13.88	13.23	13.85	13.67	13.33	13.47	14.22
0.032	-0.04	0.97	O35551	Rab GTPase-binding effector protein 1	Rabep1	380	5	5	4	8.6	8.6	6.7	99.523	0.0004	4.7729	1598900	13	14.27	12.63	13.82	13.35	13.02	13.61	13.81	13.78
0.036	-0.04	0.97	Q6NZJ6	Eukaryotic translation initiation factor 4 gamma 1	Eif4g1	2546	47	47	43	33.4	33.4	30.6	176.07	0.0000	295.24	152010000	1012	17.96	17.98	19.33	18.28	17.99	18.65	18.49	18.57
0.067	-0.04	0.97	Q8BP48	Methionine aminopeptidase 1	Metap1	3159	16	16	16	59.3	59.3	59.3	43.221	0.0000	93.344	58056000	273	17.42	17.16	17.16	17.59	17.31	17.78	17.43	16.96
0.049	-0.04	0.97	Q61549	EGF-like module-containing mucin-like hormone receptor-like 1	Emr1	2311	10	10	10	10	10	10	102.13	0.0000	197.52	62871000	322	16.97	16.48	17.70	17.21	17.27	16.94	17.30	17.00
0.024	-0.04	0.97	Q9WVL0	Maleylacetoacetate isomerase	Gstz1	5753	3	3	3	19.4	19.4	19.4	24.275	0.0000	157.71	6361600	50	15.34	15.09	13.30	15.06	14.92	14.54	15.30	14.18
0.080	-0.04	0.97	P56480	ATP synthase subunit beta, mitochondrial	Atp5b	1288	41	41	41	86.6	86.6	86.6	56.3	0.0000	323.31	1849000000	2950	21.44	21.53	20.81	21.05	21.20	21.22	21.27	21.29
0.053	-0.04	0.97	Q6P5D3	Putative ATP-dependent RNA helicase DHX57	Dhx57	2571	5	5	5	6.4	6.4	6.4	155.76	0.0000	8.7751	1554000	27	13.18	13.33	13.11	13.25	13.68	13.36	12.52	13.47
0.026	-0.04	0.97	Q8BMB3	Eukaryotic translation initiation factor 4E type 2	Eif4e2	3131	3	3	3	13.9	13.9	13.9	28.263	0.0000	8.3792	1732100	25	12.38	14.24	13.48	14.40	13.18	14.23	13.95	13.30
0.039	-0.04	0.97	Q9ES46	Beta-parvin	Parvb	5327	14	14	13	41.9	41.9	38.6	41.669	0.0000	82.632	35878000	187	17.16	17.70	16.25	17.62	16.93	17.10	17.41	17.45
0.035	-0.04	0.97	Q9JMH9	Unconventional myosin-XVIIIa	Myo18a	5512	51	51	51	30.8	30.8	30.8	232.75	0.0000	323.31	53586000	630	15.35	15.34	16.92	15.57	15.51	15.96	16.11	15.76
0.017	-0.04	0.97	Q9D9M5	Pyridoxal phosphate phosphatase PHOSPHO2	Phospho2	5102	2	2	2	13.7	13.7	13.7	27.562	0.0018	3.1189	538580	6	12.69	12.06	14.77	12.77	11.68	12.73	13.71	14.33
0.075	-0.04	0.97	Q80WQ2	Protein VAC14 homolog	Vac14	2864	20	20	20	30.3	30.3	30.3	88.047	0.0000	148.04	51152000	386	17.15	16.54	17.44	16.95	16.87	17.15	17.13	17.09
0.038	-0.04	0.97	Q61183;Q9\	Poly(A) polymerase alpha;Poly(A) polymerase beta	Papola;Papolb	2269	6	6	6	12.2	12.2	12.2	82.308	0.0000	18.805	12463000	81	15.69	15.55	16.84	15.24	15.41	16.19	15.88	16.01
0.061	-0.04	0.97	Q8K2X3	CST complex subunit STN1	Obfc1	3677	5	5	5	21.4	21.4	21.4	43.485	0.0000	9.0947	2596500	31	13.69	14.25	14.09	13.65	13.75	13.94	14.52	13.64
0.053	-0.04	0.97	Q9CPR5	39S ribosomal protein L15, mitochondrial	Mrpl15	4530	16	16	16	66.1	66.1	66.1	33.541	0.0000	125.63	35081000	329	15.69	15.72	16.69	16.22	16.09	16.36	16.36	15.68
0.021	-0.04	0.97	Q793I8	TRAF-interacting protein with FHA domain-containing protein A	Tifa	2735	5	5	5	27.7	27.7	27.7	21.56	0.0000	22.12	8119000	43	15.74	15.64	13.34	15.81	15.48	15.10	15.88	14.24
0.131	-0.04	0.97	Q9Z1G4	V-type proton ATPase 116 kDa subunit a isoform 1	Atp6v0a1	5808	26	26	25	27.8	27.8	26.9	96.466	0.0000	180.32	101430000	530	17.15	16.87	17.00	17.30	17.19	17.23	17.20	16.88
0.183	-0.04	0.97	Q9JKF1	Ras GTPase-activating-like protein IQGAP1	Iqgap1	5450	139	139	135	74.6	74.6	73	188.74	0.0000	323.31	2371900000	5676	20.32	20.25	20.58	20.34	20.36	20.51	20.52	20.28
0.096	-0.04	0.97	Q9Z2V5	Histone deacetylase 6	Hdac6	5866	8	8	8	9.7	9.7	9.7	125.79	0.0000	19.54	8813700	79	15.71	15.28	15.48	15.86	15.45	15.58	15.94	15.55
0.067	-0.04	0.97	Q504P2	C-type lectin domain family 12 member A	Clec12a	2077	10	10	10	34.5	34.5	34.5	30.757	0.0000	34.719	21510000	148	15.86	15.47	16.52	16.10	16.07	15.82	16.21	16.03
0.066	-0.04	0.97	P50580	Proliferation-associated protein 2G4	Pa2g4	1188	37	37	37	67	67	67	43.698	0.0000	323.31	211400000	936	18.39	18.28	19.08	18.12	18.45	18.84	18.35	18.40
0.102	-0.04	0.97	Q9QYE6	Golgin subfamily A member 5	Golga5	5570	34	34	34	56.5	56.5	56.5	82.367	0.0000	258.6	42744000	488	15.89	15.72	16.19	16.05	15.66	16.20	16.16	16.00
0.049	-0.04	0.97	P97315	Cysteine and glycine-rich protein 1	Csrp1	1623	8	8	8	59.1	59.1	59.1	20.583	0.0000	138.27	73794000	300	17.66	17.26	18.67	17.80	17.66	18.10	18.03	17.77
0.033	-0.04	0.97	Q8BYZ1	ABI gene family member 3	Abi3	3308	15	15	15	47.7	47.7	47.7	39.106	0.0000	189.5	28300000	245	16.66	17.26	18.19	17.56	16.97	18.45	17.05	17.40
0.039	-0.05	0.97	Q9WTZ1	RING-box protein 2	Rnf7	5694	3	3	3	37.2	37.2	37.2	12.707	0.0000	9.6279	1629300	19	12.88	14.36	13.45	13.45	13.14	14.22	13.15	13.82
0.033	-0.05	0.97	Q8C0L8	Conserved oligomeric Golgi complex subunit 5	Cog5	3343	15	15	15	26.8	26.8	26.8	91.39	0.0000	61.782	21589000	116	16.65	16.89	18.67	17.00	17.08	17.77	17.23	17.32
0.123	-0.05	0.97	Q9QUI0	Transforming protein RhoA	Rhoa	5514	18	18	10	67.9	67.9	39.9	21.782	0.0000	239.34	492120000	1176	19.20	18.99	18.87	19.47	19.10	19.10	19.19	19.31
0.050	-0.05	0.97	Q3U487	E3 ubiquitin-protein ligase HECTD3	Hectd3	1923	20	20	20	35.5	35.5	35.5	97.346	0.0000	61.948	25810000	216	15.73	15.12	16.57	15.76	15.48	16.05	15.93	15.91
0.061	-0.05	0.97	Q15843;P29	NEDD8	NEDD8;Nedd8	971	5	5	5	71.6	71.6	71.6	9.0714	0.0000	79.674	55180000	197	17.70	18.19	18.68	18.11	17.83	18.70	18.15	18.19
0.027	-0.05	0.97	Q62504	Msx2-interacting protein	Spen	2399	5	5	5	1.9	1.9	1.9	398.75	0.0000	12.694	1857500	16	12.22	14.79	13.80	13.44	13.81	12.94	14.10	13.60
0.107	-0.05	0.97	Q9Z2W0	Aspartyl aminopeptidase	Dnpep	5868	20	20	20	52.2	52.2	52.2	52.206	0.0000	323.31	90121000	494	17.34	17.27	16.79	17.13	17.02	17.07	17.50	17.12
0.215	-0.05	0.97	P10126	Elongation factor 1-alpha 1	Eef1a1	719	53	53	33	74.5	74.5	45.9	50.113	0.0000	323.31	6814600000	5720	23.39	23.66	23.29	23.48	23.51	23.55	23.56	23.38
0.063	-0.05	0.97	Q91ZR1	Ras-related protein Rab-4B	Rab4b	4198	12	12	11	70	70	63.4	23.629	0.0000	135.42	23147000	264	15.76	15.87	14.88	15.94	15.51	15.48	15.94	15.71
0.096	-0.05	0.97	Q8K297	Procollagen galactosyltransferase 1	Colgalt1	3651	17	17	17	32.1	32.1	32.1	71.06	0.0000	63.054	56244000	251	17.99	17.79	17.65	17.24	17.54	17.92	17.59	17.82
0.035	-0.05	0.97	Q9EQC5	N-terminal kinase-like protein	Scyl1	5275	19	19	19	34.9	34.9	34.9	89.159	0.0000	88.18	45884000	213	16.86	17.03	18.59	17.19	17.03	18.18	17.23	17.43
0.068	-0.05	0.97	P15307	Proto-oncogene c-Rel	Rel	794	11	10	10	22.8	21.3	21.3	64.959	0.0000	31.176	22202000	176	16.52	16.37	15.63	16.55	16.00	16.22	16.62	16.42
0.064	-0.05	0.97	Q8R1S0	Ubiquinone biosynthesis monooxygenase COQ6, mitochondrial	Coq6	3797	11	11	11	27.5	27.5	27.5	51.392	0.0000	53.218	17030000	125	17.13	16.38	16.75	16.60	16.69	16.18	16.99	17.19
0.023	-0.05	0.97	P30677	Guanine nucleotide-binding protein subunit alpha-14	Gna14	979	8	4	3	25.1	14.1	8.2	41.527	0.0000	27.343	1636000	50	13.49	13.36	12.52	13.65	11.97	15.06	12.47	13.70
0.095	-0.05	0.97	O89110	Caspase-8;Caspase-8 subunit p18;Caspase-8 subunit p10	Casp8	605	32	32	32	71.7	71.7	71.7	55.356	0.0000	181.6	88587000	749	16.82	16.89	17.53	16.84	16.94	17.17	16.92	17.25
0.147	-0.05	0.97	P47754	F-actin-capping protein subunit alpha-2	Capza2	1120	27	27	22	89.2	89.2	79	32.967	0.0000	323.31	445770000	1001	20.05	19.98	19.64	20.12	19.78	20.04	20.08	20.09
0.022	-0.05	0.97	Q8VCW4	Protein unc-93 homolog B1	Unc93b1	3933	5	5	5	8.9	8.9	8.9	66.98	0.0000	23.179	18756000	128	16.83	16.86	14.21	16.21	16.25	14.60	16.70	16.77
0.049	-0.05	0.97	Q8K4R4	Cytoplasmic phosphatidylinositol transfer protein 1	Pitpnc1	3728	4	4	4	13.9	13.9	13.9	38.383	0.0000	6.227	4478200	25	12.75	13.86	13.81	12.58	13.67	13.33	13.20	13.00
0.090	-0.05	0.96	P23492	Purine nucleoside phosphorylase	Pnp	884	25	25	25	79.2	79.2	79.2	32.277	0.0000	323.31	608620000	1283	20.16	20.31	19.61	20.57	20.11	20.19	20.33	20.22
0.038	-0.05	0.96	Q8BI84	Melanoma inhibitory activity protein 3	Mia3	3055	22	22	22	13.9	13.9	13.9	213.67	0.0000	45.563	24141000	170	16.01	16.04	17.87	16.40	16.07	16.90	16.67	16.89
0.190	-0.05	0.96	Q62426	Cystatin-B	Cstb	2391	12	12	12	93.9	93.9	93.9	11.045	0.0000	252.45	523680000	892	20.47	20.44	20.70	20.75	20.69	20.61	20.81	20.46
0.096	-0.06	0.96	Q03267	DNA-binding protein Ikaros	Ikzf1	1725	5	5	5	12.4	12.4	12.4	57.336	0.0000	11.083	10943000	36	16.35	16.23	16.97	16.28	16.41	16.36	16.87	16.40
0.067	-0.06	0.96	Q2TBA3	Mucosa-associated lymphoid tissue lymphoma translocation protein 1 h	Malt1	1800	7	7	7	13.5	13.5	13.5	93.215	0.0000	60.389	4609800	57	14.94	15.39	14.58	14.23	15.16	14.57	15.08	14.56
0.067	-0.06	0.96	Q9CR26	Vacuolar protein sorting-associated protein VTA1 homolog	Vta1	4661	11	11	11	39.5	39.5	39.5	33.913	0.0000	125.6	44259000	226	17.57	17.78	16.61	17.85	17.46	17.69	17.56	17.31
0.099	-0.06	0.96	Q8BMJ2	Leucine--tRNA ligase, cytoplasmic	Lars	3140	66	66	66	57.7	57.7	57.7	134.19	0.0000	323.31	175430000	1161	17.33	17.40	18.08	17.19	17.41	17.70	17.65	17.47
0.062	-0.06	0.96	Q8C1Y8	Vacuolar fusion protein CCZ1 homolog	Ccz1	3368	9	9	9	25.8	25.8	25.8	55.504	0.0000	36.494	12892000	170	15.68	14.92	16.23	15.24	15.49	15.99	15.60	15.22
0.145	-0.06	0.96	Q91ZW3	SWI/SNF-related matrix-associated actin-dependent regulator of chrom:	Smarca5	4204	41	41	30	39	39	29.1	121.63	0.0000	162.98	77487000	623	16.54	16.24	16.96	16.68	16.66	16.64	16.65	16.69
0.063	-0.06	0.96	Q9R0M6	Ras-related protein Rab-9A	Rab9a	5629	7	7	7	46.8	46.8	46.8	22.909	0.0000	101.85	19436000	137	16.09	16.38	17.31	16.34	16.10	16.53	16.93	16.79
0.061	-0.06	0.96	Q8R570	Synaptosomal-associated protein 47	Snap47	3880	5	5	5	18.2	18.2	18.2	46.524	0.0000	23.361	3628700	54	14.28	14.45	14.72	13.89	14.54	14.18	15.10	13.75
0.051	-0.06	0.96	Q9D855	Cytochrome b-c1 complex subunit 7	Uqcrb	5050	13	13	13	76.6	76.6	76.6	13.527	0.0000	118.26	30042000	182	15.98	16.81	16.83	15.95	16.15	17.02	15.73	16.90
0.061	-0.06	0.96	Q9Z2Z6	Mitochondrial carnitine/acylcarnitine carrier protein	Slc25a20	5874	9	9	9	42.5	42.5	42.5	33.026	0.0000	34.55	19327000	81	16.45	16.14	15.50	16.72	16.47	16.75	15.74	16.09
0.208	-0.06	0.96	P26638	Serine--tRNA ligase, cytoplasmic	Sars	927	46	46	46	69.5	69.5	69.5	58.388	0.0000	323.31	303710000	1413	18.33	18.31	18.07	18.30	18.11	18.36	18.55	18.21
0.127	-0.06	0.96	Q8C0I1	Alkyldihydroxyacetonephosphate synthase, peroxisomal	Agps	3337	31	31	31	56.3	56.3	56.3	71.683	0.0000	281.96	101300000	636	17.12	17.07	16.89	17.04	17.02	17.53	17.13	16.69
0.150	-0.06	0.96	Q9DC16	Endoplasmic reticulum-Golgi intermediate compartment protein 1	Ergic1	5186	10	10	10	30	30	30	32.562	0.0000	78.641	14195000	179	15.11	15.29	14.87	14.92	15.20	15.43	14.92	14.90
0.077	-0.06	0.96	Q9QZS3	Protein numb homolog	Numb	5605	13	13	13	22.5	22.5	22.5	70.812	0.0000	26.875	28461000	213	15.64	15.75	16.61	16.33	15.70	16.08	16.51	16.28
0.061	-0.06	0.96	Q9CWN7	CCR4-NOT transcription complex subunit 11	Cnot11	4722	3	3	3	12.1	12.1	12.1	54.959	0.0025	2.9065	3035200	35	14.78	14.16	13.50	14.14	13.51	14.79	14.28	14.26
0.103	-0.06	0.96	P97494	Glutamate--cysteine ligase catalytic subunit	Gclc	1663	23	23	23	44.4	44.4	44.4	72.571	0.0000	116.9	39585000	333	15.92	15.85	15.21	15.96	15.88	16.13	15.74	15.44
0.051	-0.06	0.96	Q8BW10	RNA-binding protein NOB1	Nob1	3253	3	3	3	12.7	12.7	12.7	45.463	0.0000	29.286	1844900	21	13.85	13.46	13.72	14.68	13.77	14.38	14.71	13.11
0.224	-0.06	0.96	Q5XJY5	Coatomer subunit delta	Arcn1	2173	44	44	44	71.2	71.2	71.2	57.229	0.0000	323.31	220050000	1199	17.67	17.56	17.53	17.86	17.60	17.98	17.68	17.62
0.150	-0.06	0.96	Q9DB29	Isoamyl acetate-hydrolyzing esterase 1 homolog	Iah1	5122	9	9	9	43	43	43	27.974	0.0000	164.94	33939000	246	16.46	16.34	15.87	16.37	16.33	16.08	16.32	16.56
0.103	-0.06	0.96	Q9D7N3	28S ribosomal protein S9, mitochondrial	Mrps9	5036	10	10	10	31.8	31.8	31.8	44.929	0.0000	72.551	46277000	118	18.85	18.52	18.26	18.90	18.75	18.70	19.09	18.24
0.273	-0.07	0.96	O35435	Dihydroorotate dehydrogenase (quinone), mitochondrial	Dhodh	369	16	16	16	55.7	55.7	55.7	42.699	0.0000	115.15	31153000	305	15.87	15.83	15.71	15.84	15.87	16.03	16.00	15.62
0.026	-0.07	0.96	Q8CGP1	Histone H2B type 1-K	Hist1h2bk	3511	27	2	0	96	13.5	0	13.92	0.0026	2.944	14130000	35	16.20	16.27	16.83	16.18	17.30	13.90	17.27	17.26
0.212	-0.07	0.96	P60867	40S ribosomal protein S20	Rps20	1363	13	13	13	79.8	79.8	79.8	13.373	0.0000	235.24	422050000	909	19.45	19.50	19.55	19.61	19.37	19.93	19.50	19.57
0.164	-0.07	0.96	Q8CG76	Aflatoxin B1 aldehyde reductase member 2	Akr7a2	3500	13	13	13	47.4	47.4	47.4	40.612	0.0000	114.83	50486000	273	16.74	16.79	16.90	16.69	16.85	17.25	16.75	16.54
0.095	-0.07	0.95	Q5SV85	Synergin gamma	Synrg	2150	4	4	4	4.1	4.1	4.1	139.61	0.0000	6.8095	1579300	15	13.05	13.20	13.66	13.04	13.93	13.06	13.22	13.01
0.138	-0.07	0.95	Q91YR1	Twinfilin-1	Twf1	4173	21	18	18	50.3	46.3	46.3	40.079	0.0000	323.31	124220000	527	17.53	17.49	17.36	17.78	17.36	18.06	17.62	17.39
0.117	-0.07	0.95	Q8BHD7	Polypyrimidine tract-binding protein 3	Ptbp3	3033	23	18	18	53.3	48.6	48.6	56.7	0.0000	323.31	140440000	472	18.16	18.18	17.59	18.56	18.04	18.08	18.28	18.35
0.220	-0.07	0.95	P42230	Signal transducer and activator of transcription 5A	Stat5a	1077	17	17	6	23	23	8.1	90.83	0.0000	90.869	24632000	251	15.81	15.55	15.30	15.51	15.44	15.73	15.57	15.70
0.029	-0.07	0.95	Q8R1K1	Ubiquitin-associated domain-containing protein 2	Ubac2	3791	5	5	5	18.6	18.6	18.6	39.056	0.0000	26.99	7535200	39	14.21	15.90	12.82	15.17	15.11	14.22	15.59	13.45
0.057	-0.07	0.95	Q8BL66	Early endosome antigen 1	Eea1	3110	65	65	65	51	51	51	160.91	0.0000	323.31	70811000	828	15.67	15.27	17.22	15.91	15.85	16.30	16.12	16.07
0.033	-0.07	0.95	Q8VEJ9	Vacuolar protein sorting-associated protein 4A	Vps4a	4009	15	9	9	40	28.4	28.4	48.906	0.0000	54.192	9063200	99	15.09	13.55	15.50	15.15	13.63	16.01	14.24	15.69
0.209	-0.07	0.95	P80316	T-complex protein 1 subunit epsilon	Cct5	1593	59	59	59	85.8	85.8	85.8	59.623	0.0000	323.31	559260000	2012	19.37	19.33	19.00	19.54	19.20	19.49	19.48	19.34
0.073	-0.07	0.95	E9Q5C9		Nolc1	238	9	9	8	13	13	12	73.697	0.0000	17.598	13545000	122	15.78	15.56	16.90	15.47	15.77	16.04	16.21	15.97
0.233	-0.07	0.95	Q9CWZ7	Gamma-soluble NSF attachment protein	Napg	4741	26	26	26	68.3	68.3	68.3	34.732	0.0000	204.46	53227000	470	16.48	16.85	16.72	16.44	16.76	16.84	16.50	16.67

0.094	-0.07	0.95	Q9JHK4	Geranylgeranyl transferase type-2 subunit alpha	Rabggta	5365	18	18	18	45.1	45.1	45.1	64.989	0.0000	231.53	34411000	387	16.12	15.99	16.59	16.13	15.98	16.64	16.73	15.77
0.195	-0.07	0.95	Q9DB20	ATP synthase subunit O, mitochondrial	Atp5o	5119	24	24	24	79.8	79.8	79.8	23.363	0.0000	323.31	443680000	982	19.52	19.54	19.91	19.30	19.63	19.84	19.56	19.53
0.054	-0.07	0.95	Q9D404	3-oxoacyl-[acyl-carrier-protein] synthase, mitochondrial	Oxsm	4971	5	5	5	21.4	21.4	21.4	48.627	0.0000	52.531	3925400	68	14.89	15.01	13.23	14.42	14.50	14.92	14.58	13.83
0.134	-0.07	0.95	Q9D3P8	Plasminogen receptor (KT)	Plgrkt	4968	9	9	9	41.5	41.5	41.5	17.261	0.0000	20.322	28815000	136	16.97	16.80	17.27	16.58	17.24	16.59	17.16	16.92
0.119	-0.07	0.95	Q60714	Long-chain fatty acid transport protein 1	Slc27a1	2197	13	12	12	27.2	26	26	71.275	0.0000	76.723	17295000	148	15.84	15.91	16.55	16.16	16.00	16.56	16.36	15.83
0.115	-0.07	0.95	Q8VCT3	Aminopeptidase B	Rnpep	3932	36	36	36	57.8	57.8	57.8	72.415	0.0000	323.31	209300000	906	18.27	18.33	17.36	18.22	17.94	18.14	18.22	18.17
0.138	-0.07	0.95	Q9D6R2	Isocitrate dehydrogenase [NAD] subunit alpha, mitochondrial	Idh3a	5007	21	21	21	51.6	51.6	51.6	39.638	0.0000	182.15	90883000	611	17.47	17.46	17.33	17.48	17.32	17.98	17.07	17.66
0.090	-0.07	0.95	P60766	Cell division control protein 42 homolog	Cdc42	1360	19	19	18	53.9	53.9	53.9	21.258	0.0000	255.26	1072900000	1512	20.75	20.31	21.66	20.93	20.76	20.85	21.11	21.22
0.113	-0.07	0.95	P18155	Bifunctional methylenetetrahydrofolate dehydrogenase/cyclohydrolase,	Mthfd2	834	17	17	17	53.1	53.1	53.1	37.863	0.0000	129.78	40830000	259	16.48	16.79	16.61	17.04	16.31	17.25	16.60	17.05
0.074	-0.07	0.95	Q80VC6	tRNA selenocysteine 1-associated protein 1	Trnau1ap	2848	2	2	2	10.1	10.1	10.1	32.423	0.0000	7.772	1047600	24	13.67	13.93	12.84	13.93	13.09	14.31	13.68	13.59
0.116	-0.08	0.95	Q00519	Xanthine dehydrogenase/oxidase;Xanthine dehydrogenase;Xanthine ox	Xdh	1697	57	57	55	47.3	47.3	46.6	146.56	0.0000	323.31	289430000	1532	18.00	17.87	18.76	18.15	18.39	18.03	18.62	18.04
0.335	-0.08	0.95	P29351	Tyrosine-protein phosphatase non-receptor type 6	Ptpn6	966	61	61	61	85.2	85.2	85.2	67.558	0.0000	323.31	831080000	1950	20.06	19.86	20.19	20.28	20.11	20.12	20.22	20.25
0.073	-0.08	0.95	Q8R366	Immunoglobulin superfamily member 8	Igsf8	3838	6	6	6	12.6	12.6	12.6	65.01	0.0000	45.641	4709600	39	13.88	13.90	14.84	14.10	13.36	14.64	14.47	14.56
0.450	-0.08	0.95	Q8R081	Heterogeneous nuclear ribonucleoprotein L	Hnrnpl	3750	31	31	31	61.6	61.6	61.6	63.963	0.0000	323.31	324740000	1095	18.94	19.06	19.02	18.84	18.89	19.14	19.01	19.13
0.086	-0.08	0.95	Q91YT8	CSC1-like protein 1	Tmem63a	4180	7	7	7	11.9	11.9	11.9	91.859	0.0000	59.194	10005000	114	15.65	15.76	15.61	14.67	15.63	15.21	16.01	15.15
0.178	-0.08	0.95	A6H8H2	DENN domain-containing protein 4C	Dennd4c	63	40	40	40	25.8	25.8	25.8	211.46	0.0000	235.04	56514000	494	15.88	15.67	16.25	15.95	15.90	16.28	16.14	15.74
0.047	-0.08	0.95	Q9Z0R9	Fatty acid desaturase 2	Fads2	5783	4	4	4	11.9	11.9	11.9	52.387	0.0000	6.9898	1395200	15	12.27	13.76	13.28	13.33	12.09	13.09	14.41	13.36
0.137	-0.08	0.95	Q99KE1	NAD-dependent malic enzyme, mitochondrial	Me2	4372	34	34	34	77.1	77.1	77.1	65.798	0.0000	323.31	126280000	725	17.45	17.49	16.80	17.46	17.35	17.66	17.48	17.03
0.043	-0.08	0.95	P33610	DNA primase large subunit	Prim2	1003	11	11	11	25.7	25.7	25.7	58.408	0.0000	19.816	4565700	39	13.87	12.82	15.37	14.44	13.80	13.91	15.25	13.85
0.073	-0.08	0.95	Q8R5C5	Beta-centractin	Actr1b	3884	24	11	11	78.7	46.8	46.8	42.281	0.0000	194.28	35737000	236	16.94	17.03	15.88	17.14	16.58	17.29	17.19	16.24
0.045	-0.08	0.95	P21855	B-cell differentiation antigen CD72	Cd72	868	15	15	15	44.6	44.6	44.6	40.347	0.0000	112.61	18691000	141	14.75	14.54	16.95	15.39	15.13	14.92	15.90	16.01
0.176	-0.08	0.95	Q99LJ0	CTTNBP2 N-terminal-like protein	Cttnbp2nl	4435	19	19	19	37.9	37.9	37.9	69.84	0.0000	54.139	14515000	165	15.00	15.16	15.61	15.16	15.47	15.54	15.07	15.15
0.205	-0.08	0.95	Q9CQF0	39S ribosomal protein L11, mitochondrial	Mrpl11	4592	10	10	10	59.4	59.4	59.4	20.68	0.0000	47.207	28152000	252	15.75	15.92	16.03	15.78	15.59	16.23	16.08	15.90
0.179	-0.08	0.95	Q99JB2	Stomatin-like protein 2, mitochondrial	Stoml2	4336	20	20	20	66.9	66.9	66.9	38.384	0.0000	323.31	62926000	475	16.84	16.48	16.68	17.02	16.54	17.10	17.00	16.71
0.160	-0.08	0.95	Q62425	Cytochrome c oxidase subunit NDUFA4	Ndufa4	2390	12	12	12	65.9	65.9	65.9	9.3267	0.0000	77.975	218640000	496	19.01	18.96	19.51	19.09	18.80	19.29	19.43	19.37
0.030	-0.08	0.95	Q8VE19	WD repeat-containing protein mio	Mios	3983	7	7	7	13	13	13	98.334	0.0000	29.589	7877700	25	16.26	15.85	14.15	15.02	16.27	12.97	16.38	15.98
0.141	-0.08	0.95	Q9WUM5	Succinyl-CoA ligase [ADP/GDP-forming] subunit alpha, mitochondrial	Suclg1	5717	15	15	15	38.2	38.2	38.2	36.154	0.0000	233.69	140850000	632	17.81	17.43	17.43	17.81	17.87	18.12	17.55	17.25
0.047	-0.08	0.95	O54950	5-AMP-activated protein kinase subunit gamma-1	Prkag1	436	12	12	11	49.1	49.1	46.1	37.52	0.0000	104.52	8867900	61	15.04	15.95	13.78	15.83	15.63	15.58	15.50	14.22
0.232	-0.08	0.94	P47740	Fatty aldehyde dehydrogenase	Aldh3a2	1118	19	19	18	46.7	46.7	44.2	53.97	0.0000	323.31	62865000	488	17.16	16.82	16.74	17.05	17.13	17.21	16.74	17.01
0.060	-0.08	0.94	Q3UQA7	Selenoprotein H	Selh	2018	5	5	5	63.8	63.8	63.8	12.974	0.0000	11.68	6766300	54	15.30	15.71	17.06	15.65	15.97	16.55	15.20	16.34
0.175	-0.08	0.94	Q8BFZ9	Erlin-2	Erlin2	2955	18	12	12	48.8	39.7	39.7	37.872	0.0000	200.39	40516000	270	16.87	17.01	16.49	16.39	16.77	17.08	16.68	16.57
0.102	-0.08	0.94	P70188	Kinesin-associated protein 3	Kifap3	1531	6	6	6	9.3	9.3	9.3	91.29	0.0000	45.752	3556200	78	14.01	13.37	14.54	13.84	13.73	13.80	14.53	14.05
0.043	-0.08	0.94	Q9D786	HAUS augmin-like complex subunit 5	Haus5	5022	8	8	7	19.4	19.4	18.1	69.571	0.0000	16.738	7015600	40	14.96	14.89	13.66	15.57	14.85	13.42	16.15	15.01
0.046	-0.08	0.94	Q99J62	Replication factor C subunit 4	Rfc4	4327	11	11	11	29.7	29.7	29.7	39.866	0.0000	42.8	13326000	117	15.43	15.25	17.62	14.96	15.34	16.19	16.01	16.07
0.087	-0.08	0.94	Q9DC70	NADH dehydrogenase [ubiquinone] iron-sulfur protein 7, mitochondrial	Ndufs7	5200	8	8	8	34.8	34.8	34.8	24.683	0.0000	92.219	18104000	127	15.94	16.54	16.61	16.67	16.90	16.41	17.09	15.69
0.064	-0.09	0.94	D3Z7Q2		Smim20	216	3	3	3	50.7	50.7	50.7	7.818	0.0002	5.4805	2307100	55	14.29	13.64	13.18	14.51	13.80	13.17	14.91	14.08
0.134	-0.09	0.94	Q9CQ65	S-methyl-5-thioadenosine phosphorylase	Mtap	4567	16	16	16	58.7	58.7	58.7	31.062	0.0000	252.43	101420000	431	18.39	18.32	18.32	18.13	18.32	18.48	18.92	17.78
0.109	-0.09	0.94	Q6ZPZ3	Zinc finger CCCH domain-containing protein 4	Zc3h4	2687	5	5	5	7.4	7.4	7.4	140.97	0.0000	17.335	3098800	49	14.41	13.88	14.78	14.21	14.32	13.96	15.03	14.33
0.056	-0.09	0.94	P34960	Macrophage metalloelastase	Mmp12	1009	6	6	6	15.6	15.6	15.6	54.97	0.0000	11.661	4924500	46	14.85	14.75	14.87	13.70	15.60	14.92	14.64	13.37
0.144	-0.09	0.94	Q9QXZ0	Microtubule-actin cross-linking factor 1	Macf1	5557	153	152	151	28.5	28.4	28.1	831.87	0.0000	323.31	171040000	1972	16.28	16.13	17.12	16.35	16.42	16.65	16.68	16.48
0.035	-0.09	0.94	Q61206	Platelet-activating factor acetylhydrolase IB subunit beta	Pafah1b2	2276	4	4	4	30.1	30.1	30.1	25.581	0.0000	48.951	10535000	83	15.43	15.90	12.95	16.05	16.10	14.01	15.27	15.30
0.149	-0.09	0.94	O70566	Protein diaphanous homolog 2	Diaph2	513	34	34	34	39	39	39	124.87	0.0000	308.47	80109000	527	17.10	17.03	17.96	17.23	17.30	17.38	17.59	17.40
0.191	-0.09	0.94	Q8BRN9	Coiled-coil and C2 domain-containing protein 1B	Cc2d1b	3183	22	22	22	41.3	41.3	41.3	93.09	0.0000	193.67	28434000	288	15.81	15.69	16.24	16.04	15.92	16.42	15.85	15.94
0.175	-0.09	0.94	Q8VHH5	Arf-GAP with GTPase, ANK repeat and PH domain-containing protein 3	Agap3	4019	8	8	6	12.9	12.9	10.2	97.964	0.0000	64.7	9859000	181	14.54	14.37	15.11	15.01	14.65	15.03	14.92	14.80
0.114	-0.09	0.94	O54825	Bystin	Bysl	426	6	6	6	18.8	18.8	18.8	49.783	0.0000	25.026	4590900	93	14.35	14.27	14.67	14.44	13.85	14.97	14.97	14.29
0.061	-0.09	0.94	Q9D154	Leukocyte elastase inhibitor A	Serpinb1a	4896	16	16	16	44.9	44.9	44.9	42.574	0.0000	25.593	17027000	118	15.13	15.61	13.72	15.55	15.40	14.53	15.65	14.78
0.272	-0.09	0.94	Q6PGG2	GEM-interacting protein	Gmip	2636	34	34	34	40.2	40.2	40.2	107.54	0.0000	266.87	82442000	797	16.68	16.52	17.04	16.73	16.62	16.81	16.99	16.90
0.120	-0.09	0.94	Q8CI11	Guanine nucleotide-binding protein-like 3	Gnl3	3537	6	6	6	17.5	17.5	17.5	60.786	0.0000	73.755	3353500	30	13.81	14.04	14.28	13.60	13.62	14.68	14.03	13.77
0.123	-0.09	0.94	P00375	Dihydrofolate reductase	Dhfr	609	15	15	15	75.9	75.9	75.9	21.606	0.0000	130.13	41741000	218	17.41	16.97	17.65	17.32	16.76	17.50	17.80	17.65
0.156	-0.09	0.94	Q64261	Cyclin-dependent kinase 6	Cdk6	2424	15	15	14	57.7	57.7	55.2	37.028	0.0000	144.39	35732000	311	16.95	16.53	17.30	16.78	16.67	16.84	17.05	17.35
0.145	-0.09	0.94	P61971	Nuclear transport factor 2	Nutf2	1397	5	5	5	40.2	40.2	40.2	14.478	0.0000	21.212	20809000	119	16.49	16.43	15.70	16.61	16.37	16.28	16.21	16.73
0.078	-0.09	0.94	P58466	Carboxy-terminal domain RNA polymerase II polypeptide A small phosp	Ctdsp1	1316	4	4	4	21.8	21.8	21.8	29.266	0.0000	70.75	13277000	119	16.27	15.66	17.46	16.33	16.23	16.62	16.99	16.24
0.067	-0.09	0.94	O35744	Chitinase-like protein 3	Chil3	404	22	22	22	57.3	57.3	57.3	44.458	0.0000	134.22	109870000	425	17.65	17.82	17.49	19.04	18.97	18.15	17.34	17.91
0.125	-0.09	0.94	Q8VE88	Protein FAM114A2	Fam114a2	3990	6	6	6	19.7	19.7	19.7	54.044	0.0000	104.97	10599000	162	14.84	14.68	15.67	15.20	14.86	15.66	15.13	15.13
0.140	-0.09	0.94	Q9Z160	Conserved oligomeric Golgi complex subunit 1	Cog1	5800	16	16	16	25.2	25.2	25.2	109.05	0.0000	136.35	20233000	263	15.46	15.75	16.34	15.48	15.85	16.20	15.86	15.50
0.093	-0.09	0.94	Q91Z96	BMP-2-inducible protein kinase	Bmp2k	4191	11	10	10	16.8	16	16	126.18	0.0000	21.209	13615000	97	16.13	15.86	16.67	14.96	15.78	16.18	15.98	16.04
0.243	-0.09	0.94	P28063	Proteasome subunit beta type-8	Psmb8	945	20	20	20	67.8	67.8	67.8	30.26	0.0000	236.09	122190000	504	18.40	18.54	17.89	18.42	18.40	18.48	18.51	18.24
0.066	-0.09	0.94	Q8BH60	Golgi-associated PDZ and coiled-coil motif-containing protein	Gopc	3016	7	7	7	15.3	15.3	15.3	50.662	0.0000	8.7531	4394800	43	14.66	14.50	13.95	13.45	14.78	15.13	13.57	13.46
0.067	-0.09	0.94	Q9ERB0	Synaptosomal-associated protein 29	Snap29	5302	5	5	5	20.4	20.4	20.4	29.572	0.0018	3.1996	1259600	8	13.64	13.82	12.53	13.89	14.60	13.66	13.01	12.97
0.085	-0.09	0.94	Q09200	Beta-1,4 N-acetylgalactosaminyltransferase 1	B4galnt1	1772	11	11	11	31.3	31.3	31.3	59.211	0.0000	51.919	8674100	133	14.43	14.03	12.93	13.98	13.61	14.35	14.33	13.46
0.197	-0.09	0.94	P70302	Stromal interaction molecule 1	Stim1	1550	22	22	22	31.8	31.8	31.8	77.566	0.0000	130.97	24575000	269	15.39	15.35	15.73	15.52	15.14	15.94	15.57	15.72
0.077	-0.09	0.94	Q8BVU5	ADP-ribose pyrophosphatase, mitochondrial	Nudt9	3248	6	6	6	18.9	18.9	18.9	38.604	0.0000	6.2519	1533500	20	13.38	13.06	14.17	14.08	13.36	14.02	13.07	14.62
0.080	-0.09	0.94	Q8CI04	Conserved oligomeric Golgi complex subunit 3	Cog3	3535	5	5	5	7.8	7.8	7.8	93.282	0.0000	8.3602	3691200	55	14.21	13.81	12.66	14.55	13.73	13.71	14.21	13.96
0.129	-0.09	0.94	Q6P4S8	Integrator complex subunit 1	Ints1	2564	10	10	10	8	8	8	245.16	0.0000	73.524	5933600	104	14.30	13.93	14.84	14.77	14.18	15.00	14.62	14.42
0.090	-0.10	0.94	P48024	Eukaryotic translation initiation factor 1	Eif1	1138	5	5	1	65.5	65.5	29.2	12.746	0.0000	134.84	19118000	122	16.36	17.03	17.93	16.40	16.92	16.80	17.35	17.04
0.039	-0.10	0.93	Q0KL02	Triple functional domain protein	Trio	1775	7	7	7	3.8	3.8	3.8	347.86	0.0000	58.286	3848900	34	13.56	11.99	15.70	13.75	12.91	14.57	14.52	13.38
0.302	-0.10	0.93	O35465	Peptidyl-prolyl cis-trans isomerase FKBP8	Fkbp8	373	16	16	16	54.5	54.5	54.5	43.528	0.0000	323.31	60114000	354	17.25	17.09	16.77	17.33	17.19	17.19	17.36	17.09
0.117	-0.10	0.93	Q5ND34	WD repeat-containing protein 81	Wdr81	2121	31	31	31	27.5	27.5	27.5	211.94	0.0000	323.31	44050000	378	16.11	15.99	17.21	15.97	16.31	16.53	16.64	16.20
0.097	-0.10	0.93	P98086	Complement C1q subcomponent subunit A	C1qa	1687	3	3	3	18	18	18	25.974	0.0000	25.19	2984700	41	14.06	14.43	13.88	14.57	14.70	13.46	14.15	15.03
0.143	-0.10	0.93	P50518	V-type proton ATPase subunit E 1	Atp6v1e1	1185	34	34	34	84.5	84.5	84.5	26.157	0.0000	323.31	526470000	1135	19.69	19.53	20.60	19.62	19.70	20.06	19.99	20.08
0.053	-0.10	0.93	P30355	Arachidonate 5-lipoxygenase-activating protein	Alox5ap	977	4	4	4	19.9	19.9	19.9	18.136	0.0000	11.942	63424000	88	18.26	17.67	20.53	18.24	18.36	19.09	18.53	19.13
0.272	-0.10	0.93	Q9D8S3	ADP-ribosylation factor GTPase-activating protein 3	Arfgap3	5069	20	19	19	41.7	39.8	39.8	57.455	0.0000	114.39	38987000	291	15.68	15.89	16.32	15.70	15.87	16.04	16.05	16.03
0.119	-0.10	0.93	Q3UIR3	E3 ubiquitin-protein ligase DTX3L	Dtx3l	1980	29	29	29	46.9	46.9	46.9	83.043	0.0000	269.61	61296000	559	16.23	15.84	16.70	16.86	16.00	16.33	17.01	16.70
0.099	-0.10	0.93	Q9D394	Protein RUFY3	Rufy3	4962	25	24	24	45.8	44.1	44.1	53.006	0.0000	66.804	38784000	213	16.31	16.34	17.79	16.25	16.55	16.92	16.74	16.89
0.356	-0.10	0.93	Q3TRM8	Hexokinase-3	Hk3	1872	55	54	52	69.6	68.3	66.8	100.1	0.0000	323.31	504320000	1766	19.31	19.33	19.23	19.27	19.73	19.18	19.39	19.25
0.390	-0.10	0.93	Q8CGF7	Transcription elongation regulator 1	Tcerg1	3508	41	41	40	38.6	38.6	38.4	123.79	0.0000	160.41	59994000	434	16.02	16.36	16.26	16.01	16.25	16.40	16.06	16.35
0.067	-0.10	0.93	Q99ME2	WD repeat-containing protein 6	Wdr6	4463	10	10	10	14	14	14	121.9	0.0000	13.346	4091900	31	14.60	13.40	15.79	13.72	14.56	14.64	14.62	14.11
0.396	-0.10	0.93	P23591	GDP-L-fucose synthase	Tsta3	886	17	17	17	46.1	46.1	46.1	35.877	0.0000	193.76	42643000	305	16.81	16.55	16.98	16.84	16.77	17.10	16.83	16.88
0.179	-0.10	0.93	Q61753	D-3-phosphoglycerate dehydrogenase	Phgdh	2332	30	30	30	47.5	47.5	47.5	56.585	0.0000	323.31	199310000	947	17.92	17.76	18.62	18.24	17.92	18.18	18.46	18.40
0.282	-0.10	0.93	Q569Z5	Probable ATP-dependent RNA helicase DDX46	Ddx46	2086	36	36	35	37.6	37.6	36.1	117.45	0.0000	236.23	76761000	642	16.48	16.65	16.98	16.89	16.54	16.96	16.96	16.96
0.144	-0.11	0.93	P98083	SHC-transforming protein 1	Shc1	1686	6	6	5	18	18	15.9	62.607	0.0000	60.045	10775000	98	16.26	15.78	15.28	16.39	15.81	16.29	16.18	15.85
0.060	-0.11	0.93	Q61216	Double-strand break repair protein MRE11A	Mre11a	2281	12	12	12	24.5	24.5	24.5	80.222	0.0000	46.333	10285000	93	13.40	14.45	16.10	14.35	14.29	14.10	15.06	15.27
0.131	-0.11	0.93	Q8CEC0	Nuclear pore complex protein Nup88	Nup88	3470	9	9	9	18.1	18.1	18.1	84.937	0.0000	75.411	11082000	125	15.32	15.54	15.45	14.27	15.50	15.25	15.17	15.10
0.163	-0.11	0.93	Q6P9Q6	FK506-binding protein 15	Fkbp15	2590	37	37	37	38.2	38.2	38.2	132.96	0.0000	323.31	84254000	725	16.77	16.56	17.57	16.99	16.83	17.41	17.19	16.91
0.570	-0.11	0.93	Q61233	Plastin-2	Lcp1	2282	73	73	67	92	92	90.7	70.148	0.0000	323.31	3779900000	5128	21.66	21.84	21.54	21.60	21.63	21.71	21.86	21.89
0.250	-0.11	0.93	P59729	Ras and Rab interactor 3	Rin3	1342	7	7	6	11.9	11.9	10.5	107.22	0.0000	46.128	7546600	124	15.09	14.93	14.57	15.24	14.97	15.17	15.29	14.82
0.068	-0.11	0.93	Q8R3L2	Transcription factor 25	Tcf25	3850	3	3	3	7.2	7.2	7.2	76.684	0.0000	7.3635	1862600	19	12.75	14.63	13.62	14.11	15.06	13.41	13.27	13.81
0.160	-0.11	0.93	P41105	60S ribosomal protein L28	Rpl28	1065	19	19	19	67.2	67.2	67.2	15.733	0.0000	179.58	410520000	646	18.92	18.77	19.78	19.32	18.95	19.61	19.26	19.41
0.049	-0.11	0.93	Q9JI99	Sphingosine-1-phosphate phosphatase 1	Sgpp1	5392	6	6	6	17.4	17.4	17.4	47.744	0.0000	18.214	13197000	100	15.54	15.42	13.30	15.41	14.82	13.48	16.12	15.70
0.258	-0.11	0.93	Q9CQI3	Glia maturation factor beta	Gmfb	4601	12	12	9	64.1	64.1	59.9	16.723	0.0000	84.669	26666000	206	16.17	16.68	16.10	16.35	16.32	16.59	16.16	16.67
0.259	-0.11	0.93	Q99LC5	Electron transfer flavoprotein subunit alpha, mitochondrial	Etfa	4417	31	31	31	79.6	79.6	79.6	35.009	0.0000	323.31	300480000	942	18.80	18.95	19.16	18.84	18.84	19.48	19.09	18.80
0.392	-0.11	0.92	Q9Z1Z0	General vesicular transport factor p115	Uso1	5826	29	29	29	40.6	40.6	40.6	106.98	0.0000	243.83	150200000	781	17.63	17.23	17.66	17.68	17.61	17.60	17.85	17.59
0.071	-0.11	0.92	P63280;P63	SUMO-conjugating enzyme UBC9	Ube2i;UBE2I	1502	8	8	8	54.4	54.4	54.4	18.007	0.0000	213.83	46838000	170	17.36	17.91	18.95	16.68	17.76	18.06	17.05	18.48
0.107	-0.11	0.92	O35601	FYN-binding protein	Fyb	384	28	28	28	38.1	38.1	38.1	90.054	0.0000	323.31	51254000	447	16.22	16.48	17.88	16.36	16.92	16.90	16.79	16.78
0.109	-0.11	0.92	Q3TFQ1	SPRY domain-containing protein 7	Spryd7	1839	6	6	6	35.7	35.7	35.7	21.681	0.0000	24.985	4796800	75	13.69	13.71	13.89	14.69	13.91	14.35	14.80	13.38
0.262	-0.11	0.92	Q91XI1	tRNA-dihydrouridine(47) synthase [NAD(P)(+)]-like	Dus3l	4147	11	11	11	25.1	25.1	25.1	71.078	0.0000	134.61	24574000	175	16.41	16.28	16.91	16.70	16.66	16.77	16.93	16.41
0.069	-0.11	0.92	Q8BMI0	F-box only protein 38	Fbxo38	3138	7	7	7	8.9	8.9	8.9	133.93	0.0000	22.409	5414100	42	14.54	12.43	14.20	14.73	13.90	13.66	14.87	13.93
0.099	-0.11	0.92	Q9D1K7	UPF0687 protein C20orf27 homolog		4923	5	5	5	44.8	44.8	44.8	19.476	0.0000	12.407	6084200	52	14.26	15.21	14.78	14.98	13.89	15.39	14.87	15.53
0.204	-0.12	0.92	Q8BGQ7	Alanine--tRNA ligase, cytoplasmic	Aars	2988	57	57	57	63.8	63.8	63.8	106.91	0.0000	323.31	298210000	1294	18.68	18.58	19.44	18.74	18.74	18.85	19.17	19.15
0.261	-0.12	0.92	P17427	AP-2 complex subunit alpha-2	Ap2a2	823	45	37	37	54.5	45.1	45.1	104.02	0.0000	323.31	105300000	766	17.22	17.16	16.51	17.24	17.12	17.24	17.21	17.02
0.131	-0.12	0.92	Q9Z172	Small ubiquitin-related modifier 3	Sumo3	5801	2	1	1	22.7	11.8	11.8	12.43	0.0027	2.6943	1690100	11	13.46	13.55	13.48	13.70	12.71	14.21	13.78	13.95
0.211	-0.12	0.92	Q99JW4	LIM and senescent cell antigen-like-containing domain protein 1	Lims1	4352	17	17	17	51.1	51.1	51.1	37.24	0.0000	77.39	58165000	584	16.45	16.13	15.72	16.63	16.22	16.43	16.57	16.17
0.377	-0.12	0.92	P46471	26S protease regulatory subunit 7	Psmc2	1106	34	34	34	78.8	78.8	78.8	48.647	0.0000	323.31	193710000	898	18.02	18.07	18.00	18.18	17.91	18.38	18.42	18.01
0.357	-0.12	0.92	Q8BJZ4	28S ribosomal protein S35, mitochondrial	Mrps35	3091	9	9	9	36.2	36.2	36.2	35.975	0.0000	84.356	15751000	119	15.88	16.07	15.66	16.05	16.00	16.14	16.24	15.75
0.188	-0.12	0.92	Q9DBW0	Cytochrome P450 4V2	Cyp4v2	5181	15	15	15	35.2	35.2	35.2	60.938	0.0000	71.145	18736000	171	15.50	15.33	16.30	15.86	16.16	15.56	15.92	15.81
0.116	-0.12	0.92	Q61696;P17	Heat shock 70 kDa protein 1A;Heat shock 70 kDa protein 1B	Hspa1a;Hspa1b	2324	33	25	19	52.7	41.7	32.1	70.078	0.0000	66.607	31779000	240	15.34	14.59	16.20	15.73	15.20	15.94	15.80	15.40
0.242	-0.12	0.92	Q8VE80	THO complex subunit 3	Thoc3	3989	6	6	6	16.5	16.5	16.5	38.737	0.0000	7.4402	7229500	69	15.53	15.65	14.81	15.41	15.44	15.32	15.67	15.45
0.076	-0.12	0.92	Q8C407	Protein YIPF4	Yipf4	3384	4	4	4	28.9	28.9	28.9	27.283	0.0000	17.617	8641300	67	15.12	14.74	13.38	15.01	14.66	15.05	15.43	13.60

0.244	-0.12	0.92	P47738	Aldehyde dehydrogenase, mitochondrial	Aldh2	1117	53	53	50	80.7	80.7	79.2	56.537	0.0000	323.31	1524400000	2402	20.91	21.04	20.25	20.64	20.90	21.09	20.71	20.64
0.152	-0.12	0.92	Q3UIU2	NADH dehydrogenase [ubiquinone] 1 beta subcomplex subunit 6	Ndufb6	1981	4	4	4	35.2	35.2	35.2	15.515	0.0000	26.109	22089000	105	16.08	16.33	16.86	16.76	16.46	16.25	17.34	16.47
0.390	-0.12	0.92	Q99KC8	von Willebrand factor A domain-containing protein 5A	Vwa5a	4370	49	49	49	55.6	55.6	55.6	87.142	0.0000	323.31	499770000	1873	19.57	19.53	19.06	19.51	19.60	19.54	19.67	19.35
0.196	-0.12	0.92	P70429	Ena/VASP-like protein	Evl	1572	23	23	23	63.8	63.8	63.8	44.337	0.0000	224.64	93860000	586	16.89	16.47	17.53	16.80	17.14	17.23	17.06	16.76
0.124	-0.12	0.92	A2AJL3	FGGY carbohydrate kinase domain-containing protein	Fggy	30	2	2	2	5.8	5.8	5.8	60.336	0.0000	17.266	767480	20	12.25	12.85	12.72	13.04	12.59	13.70	12.92	12.15
0.060	-0.12	0.92	Q9CZ83	39S ribosomal protein L55, mitochondrial	Mrpl55	4821	8	8	8	43.3	43.3	43.3	15.121	0.0000	23.497	12714000	78	15.05	16.20	18.03	15.55	15.65	16.76	15.90	17.03
0.191	-0.12	0.92	Q9CQC7	NADH dehydrogenase [ubiquinone] 1 beta subcomplex subunit 4	Ndufb4	4583	8	8	8	54.3	54.3	54.3	15.081	0.0000	77.319	31219000	199	16.57	16.17	16.65	15.91	15.91	16.71	16.70	16.49
0.574	-0.13	0.92	O88685	26S protease regulatory subunit 6A	Psmc3	558	36	36	36	86.7	86.7	86.7	49.548	0.0000	323.31	179930000	886	17.98	18.00	18.10	17.73	17.93	18.23	18.15	18.01
0.349	-0.13	0.92	Q8CH18	Cell division cycle and apoptosis regulator protein 1	Ccar1	3517	20	20	20	21.9	21.9	21.9	132.06	0.0000	188.54	33251000	302	16.52	16.50	16.13	16.77	16.44	16.60	16.83	16.56
0.176	-0.13	0.92	Q9ERS2	NADH dehydrogenase [ubiquinone] 1 alpha subcomplex subunit 13	Ndufa13	5317	12	12	12	60.4	60.4	60.4	16.859	0.0000	84.173	38221000	237	16.78	16.46	16.23	16.81	16.71	16.03	17.17	16.87
0.291	-0.13	0.92	Q9CWL8	Beta-catenin-like protein 1	Ctnnbl1	4720	14	14	13	26.5	26.5	26.5	64.979	0.0000	35.224	24350000	188	16.51	16.57	17.01	16.17	16.72	16.54	16.69	16.82
0.124	-0.13	0.92	Q99J09	Methylosome protein 50	Wdr77	4316	5	5	5	28.4	28.4	28.4	36.942	0.0000	63.285	3297700	37	14.81	15.34	15.06	13.87	15.45	14.53	15.02	14.60
0.318	-0.13	0.92	Q8CCN5	Breast carcinoma-amplified sequence 3 homolog	Bcas3	3451	11	11	11	18.1	18.1	18.1	101.02	0.0000	75.917	10227000	128	15.22	15.04	15.08	15.12	14.76	15.49	15.39	15.34
0.499	-0.13	0.92	Q61316	Heat shock 70 kDa protein 4	Hspa4	2290	69	69	64	71.6	71.6	67.7	94.132	0.0000	323.31	373430000	1895	17.97	17.87	17.95	17.85	17.81	18.35	17.97	18.02
0.408	-0.13	0.92	P27773	Protein disulfide-isomerase A3	Pdia3	940	59	59	59	73.3	73.3	73.3	56.678	0.0000	323.31	1713800000	2793	20.81	21.01	20.66	20.79	20.92	21.07	20.63	21.16
0.087	-0.13	0.92	Q8VCL2	Protein SCO2 homolog, mitochondrial	Sco2	3924	5	5	5	29.4	29.4	29.4	28.944	0.0000	16.626	7261300	66	15.03	13.09	15.21	15.25	14.83	14.58	15.08	14.59
0.226	-0.13	0.92	O88508	DNA (cytosine-5)-methyltransferase 3A	Dnmt3a	536	10	10	10	14.9	14.9	14.9	101.67	0.0000	48.94	13596000	169	14.95	14.91	15.70	15.16	15.03	15.40	15.64	15.15
0.281	-0.13	0.91	Q921Z5	Tumor necrosis factor alpha-induced protein 8	Tnfaip8	4245	10	10	10	51.5	51.5	51.5	22.96	0.0000	245.5	42390000	197	17.35	17.61	16.83	17.33	17.50	17.18	17.63	17.32
0.240	-0.13	0.91	Q9CQ60	6-phosphogluconolactonase	Pgls	4564	16	16	16	95.7	95.7	95.7	27.254	0.0000	323.31	279570000	768	18.92	19.18	19.20	19.35	19.19	19.36	18.83	19.79
0.159	-0.13	0.91	Q9R0T8	Inhibitor of nuclear factor kappa-B kinase subunit epsilon	Ikbke	5641	7	7	7	14.1	14.1	14.1	80.952	0.0000	39.318	3219100	29	13.79	13.10	13.60	14.14	13.55	13.79	14.41	13.39
0.139	-0.13	0.91	Q9CQ69	Cytochrome b-c1 complex subunit 8	Uqcrq	4568	9	9	9	80.5	80.5	80.5	9.7681	0.0000	19.738	23014000	128	16.27	16.26	16.49	16.91	16.64	17.44	16.47	15.91
0.533	-0.13	0.91	P70452	Syntaxin-4	Stx4	1577	14	14	14	55	55	55	34.165	0.0000	285.02	62632000	406	17.03	16.77	17.19	17.08	17.02	17.13	17.34	17.11
0.587	-0.13	0.91	Q9D2G2	Dihydrolipoyllysine-residue succinyltransferase component of 2-oxoglut	Dlst	4942	17	17	17	37.4	37.4	37.4	48.994	0.0000	173.5	74936000	326	17.95	17.70	17.91	17.54	17.84	17.85	18.02	17.89
0.171	-0.13	0.91	Q8CG72	Poly(ADP-ribose) glycohydrolase ARH3	Adprhl2	3499	9	9	9	37	37	37	39.414	0.0000	221.7	27986000	169	16.48	16.17	15.41	16.62	16.16	16.62	16.31	16.09
0.188	-0.13	0.91	Q60780	Growth arrest-specific protein 7	Gas7	2206	14	14	14	33	33	33	48.173	0.0000	135.53	20877000	259	15.65	15.41	16.03	16.39	15.54	15.96	16.23	16.27
0.054	-0.13	0.91	Q9JJZ6	Krueppel-like factor 13	Klf13	5441	5	5	4	23.5	23.5	16.6	31.136	0.0000	15.06	6480700	42	14.97	15.15	14.68	15.60	17.14	15.39	13.08	15.32
0.244	-0.13	0.91	P36536	GTP-binding protein SAR1a	Sar1a	1042	13	13	9	51.5	51.5	44.4	22.371	0.0000	124.75	89746000	256	18.38	19.03	18.12	18.46	18.43	18.84	18.48	18.77
0.166	-0.13	0.91	O35495	Cyclin-dependent kinase 14	Cdk14	377	6	5	5	13.6	11.9	11.9	52.995	0.0000	8.2641	2737900	30	13.74	13.72	13.91	14.21	13.63	14.01	13.63	14.83
0.142	-0.13	0.91	Q61584	Fragile X mental retardation syndrome-related protein 1	Fxr1	2315	20	20	19	35.9	35.9	34.7	76.221	0.0000	102.49	34005000	412	15.82	15.58	17.06	16.11	15.98	16.63	16.20	16.29
0.276	-0.13	0.91	Q3TKT4	Transcription activator BRG1	Smarca4	1856	37	37	27	24.4	24.4	18.5	181.43	0.0000	213.53	82767000	711	17.59	17.46	18.22	17.75	17.68	18.12	18.00	17.74
0.415	-0.13	0.91	O70475	UDP-glucose 6-dehydrogenase	Ugdh	503	22	22	22	58.6	58.6	58.6	54.831	0.0000	224	49581000	433	16.37	16.56	16.36	16.21	16.42	16.86	16.45	16.30
0.242	-0.13	0.91	Q6P5E6	ADP-ribosylation factor-binding protein GGA2	Gga2	2575	11	10	10	33.8	32.7	32.7	66.048	0.0000	187.55	20227000	183	16.20	15.90	15.82	15.56	15.91	16.51	15.92	15.67
0.243	-0.13	0.91	Q99JY0	Trifunctional enzyme subunit beta, mitochondrial;3-ketoacyl-CoA thiolas	Hadhb	4356	35	35	35	66.7	66.7	66.7	51.386	0.0000	305.99	193020000	768	18.05	17.75	17.35	18.22	17.90	18.32	17.82	17.87
0.137	-0.13	0.91	Q5SWD9	Pre-rRNA-processing protein TSR1 homolog	Tsr1	2155	10	10	10	17.3	17.3	17.3	92.104	0.0000	40.131	7235100	105	14.17	14.06	15.52	15.05	14.54	14.85	15.02	14.92
0.384	-0.13	0.91	Q8BRT1	CLIP-associating protein 2	Clasp2	3184	30	30	28	29	29	26.7	140.74	0.0000	246.69	42683000	464	15.65	15.47	15.90	15.86	15.55	16.03	16.02	15.82
0.356	-0.13	0.91	Q61074	Protein phosphatase 1G	Ppm1g	2245	9	9	9	20.1	20.1	20.1	58.727	0.0000	33.064	19318000	188	15.64	15.57	15.51	16.01	15.58	15.69	16.11	15.89
0.209	-0.13	0.91	Q9Z2I9	Succinyl-CoA ligase [ADP-forming] subunit beta, mitochondrial	Sucla2	5857	25	25	25	57.5	57.5	57.5	50.113	0.0000	219.39	85942000	461	17.59	17.80	17.06	17.16	17.44	18.00	17.60	17.11
0.399	-0.14	0.91	O55222	Integrin-linked protein kinase	Ilk	464	31	31	31	62.4	62.4	62.4	51.373	0.0000	238.21	165230000	761	18.01	17.81	17.70	17.84	17.91	18.23	18.15	17.62
0.285	-0.14	0.91	Q91YL3	Uridine-cytidine kinase-like 1	Uckl1	4165	12	12	12	34.7	34.7	34.7	60.841	0.0000	78.967	12053000	147	15.55	15.12	14.85	14.99	15.04	15.57	15.40	15.04
0.218	-0.14	0.91	Q9D0M3	Cytochrome c1, heme protein, mitochondrial	Cyc1	4879	9	9	9	39.1	39.1	39.1	35.327	0.0000	62.855	45544000	201	17.13	17.12	17.83	17.54	17.84	17.05	17.82	17.46
0.081	-0.14	0.91	Q6P069	Sorcin	Sri	2551	14	14	14	63.1	63.1	63.1	21.627	0.0000	104.54	58905000	224	17.98	18.33	15.70	17.88	17.39	17.86	17.29	17.90
0.067	-0.14	0.91	Q91WR3	Activating signal cointegrator 1 complex subunit 2	Ascc2	4124	6	6	6	13	13	13	85.652	0.0000	37.948	3752100	35	12.85	13.68	15.08	14.29	14.52	12.73	13.80	15.42
0.889	-0.14	0.91	Q8BU14	Translocation protein SEC62	Sec62	3217	7	7	7	15.6	15.6	15.6	45.58	0.0000	39.044	25954000	163	16.47	16.33	16.28	16.50	16.40	16.67	16.57	16.49
0.121	-0.14	0.91	Q6PGC1	ATP-dependent RNA helicase Dhx29	Dhx29	2632	28	28	28	24.6	24.6	24.6	153.97	0.0000	144.06	28833000	305	15.63	15.56	17.12	15.30	15.79	16.29	16.18	15.90
0.466	-0.14	0.91	Q6NV83	U2 snRNP-associated SURP motif-containing protein	U2surp	2530	20	20	19	26.6	26.6	25.7	118.26	0.0000	50.1	21338000	226	15.21	15.37	15.68	15.59	15.65	15.36	15.74	15.64
0.233	-0.14	0.91	Q03963	Interferon-induced, double-stranded RNA-activated protein kinase	Eif2ak2	1729	17	17	17	36.7	36.7	36.7	58.279	0.0000	209.53	48464000	450	16.73	16.23	17.23	16.96	16.59	17.05	17.11	16.96
0.105	-0.14	0.91	Q8BKX6	Serine/threonine-protein kinase SMG1	Smg1	3108	15	15	15	6.9	6.9	6.9	409.76	0.0000	40.918	6297900	43	14.25	13.26	14.86	14.01	14.25	13.29	14.38	15.03
0.169	-0.14	0.91	Q9DB43	Zinc finger protein-like 1	Zfpl1	5127	5	5	5	31.6	31.6	31.6	34.155	0.0000	46.507	4122100	40	14.14	14.03	14.29	15.23	14.08	14.57	14.82	14.77
0.882	-0.14	0.91	O08808	Protein diaphanous homolog 1	Diaph1	303	63	63	63	51.7	51.7	51.7	139.34	0.0000	323.31	155670000	1155	16.86	17.07	17.17	17.11	17.16	17.10	17.21	17.30
0.120	-0.14	0.91	Q8BTY2	Sodium bicarbonate cotransporter 3	Slc4a7	3212	5	5	5	9.4	9.4	9.4	116.51	0.0000	45.826	5712300	60	14.44	14.22	15.42	14.84	14.11	14.53	15.70	15.15
0.217	-0.14	0.91	Q9DAS9	Guanine nucleotide-binding protein G(I)/G(S)/G(O) subunit gamma-12	Gng12	5110	6	6	6	70.8	70.8	70.8	7.9972	0.0000	61.019	100570000	318	18.51	18.30	17.96	18.62	18.24	18.86	18.86	18.01
0.306	-0.14	0.91	O88384	Vesicle transport through interaction with t-SNAREs homolog 1B	Vti1b	526	14	14	14	56	56	56	26.713	0.0000	166.75	46969000	272	16.44	16.44	16.78	16.77	16.80	16.51	17.21	16.48
0.237	-0.14	0.91	Q9WV60	Glycogen synthase kinase-3 beta	Gsk3b	5733	11	9	9	41.4	35.7	35.7	46.71	0.0000	86.021	14553000	153	15.29	15.38	15.92	14.97	15.23	15.91	15.49	15.49
0.571	-0.14	0.91	Q9WUM3	Coronin-1B	Coro1b	5715	34	31	31	52.9	49.6	49.6	53.912	0.0000	323.31	213640000	752	18.97	18.97	19.44	19.07	19.31	19.25	19.15	19.31
0.333	-0.14	0.91	Q03958	Prefoldin subunit 6	Pfdn6	1728	6	6	6	50.4	50.4	50.4	14.454	0.0000	15.749	4731600	56	13.98	13.96	14.02	13.99	14.23	14.53	13.64	14.14
0.894	-0.14	0.90	Q9WUM4	Coronin-1C	Coro1c	5716	46	46	41	67.5	67.5	61.8	53.12	0.0000	323.31	547230000	1473	19.08	19.19	19.37	19.19	19.39	19.46	19.20	19.37
0.118	-0.14	0.90	Q9D706	RNA polymerase II-associated protein 3	Rpap3	5015	7	7	7	11.5	11.5	11.5	74.095	0.0000	31.696	4509800	47	13.86	15.09	13.58	14.96	14.76	14.76	14.77	13.77
0.069	-0.14	0.90	Q3TDX8	Cytochrome b5 reductase 4	Cyb5r4	1835	3	3	2	7.8	7.8	4.5	59.716	0.0002	5.9227	7027900	28	15.51	15.98	14.16	14.78	15.89	15.68	16.07	13.37
0.451	-0.14	0.90	Q9CSH3	Exosome complex exonuclease RRP44	Dis3	4698	22	22	22	35.3	35.3	35.3	108.84	0.0000	112.13	21888000	316	15.47	15.19	15.27	15.74	15.35	15.64	15.71	15.53
0.281	-0.15	0.90	Q80TQ5	Pleckstrin homology domain-containing family M member 2	Plekhm2	2813	5	5	5	7.6	7.6	7.6	112.73	0.0000	20.768	1408800	11	13.34	13.22	13.44	12.88	13.48	13.19	12.99	13.80
0.162	-0.15	0.90	Q8BFW4	Tripartite motif-containing protein 65	Trim65	2950	8	8	8	23	23	23	58.458	0.0000	140.82	8834000	98	15.08	14.91	14.08	15.36	14.92	15.47	15.13	14.49
0.098	-0.15	0.90	Q8K012	Formin-binding protein 1-like	Fnbp1l	3593	11	11	11	23.8	23.8	23.8	69.885	0.0000	47.143	7067500	87	13.71	13.05	15.37	14.70	13.97	14.65	14.63	14.17
0.129	-0.15	0.90	Q9CRB2	H/ACA ribonucleoprotein complex subunit 2	Nhp2	4687	3	3	3	23.5	23.5	23.5	17.247	0.0000	24.463	10764000	119	14.79	15.70	14.86	14.88	15.32	15.67	15.69	14.14
0.291	-0.15	0.90	Q9JIA7	Sphingosine kinase 2	Sphk2	5393	14	14	14	38.2	38.2	38.2	65.617	0.0000	172.7	23017000	201	16.19	15.74	16.13	16.05	16.50	15.81	16.48	15.91
0.110	-0.15	0.90	Q9JJF3	Bifunctional lysine-specific demethylase and histidyl-hydroxylase NO66	No66	5425	8	8	8	18.7	18.7	18.7	67.556	0.0000	16.468	3712200	39	13.91	13.01	14.71	14.27	13.31	13.83	14.67	14.67
0.408	-0.15	0.90	Q9Z1D1	Eukaryotic translation initiation factor 3 subunit G	Eif3g	5804	11	11	11	40.6	40.6	40.6	35.638	0.0000	83.783	28575000	228	16.50	16.88	16.23	16.55	16.86	16.45	16.74	16.69
0.526	-0.15	0.90	Q922R8	Protein disulfide-isomerase A6	Pdia6	4276	27	27	27	53.9	53.9	53.9	48.1	0.0000	323.31	453530000	956	19.35	19.39	18.83	19.27	19.31	19.32	19.44	19.36
0.276	-0.15	0.90	Q9WV55	Vesicle-associated membrane protein-associated protein A	Vapa	5732	26	26	25	81.5	81.5	81.5	27.855	0.0000	253.71	183440000	823	18.15	17.79	17.20	18.03	17.89	18.06	18.06	17.76
0.176	-0.15	0.90	Q9R0L7	A-kinase anchor protein 8-like	Akap8l	5628	8	8	8	17	17	17	71.453	0.0000	20.045	5365000	50	14.79	14.52	13.32	14.04	14.33	14.47	14.33	14.13
0.149	-0.15	0.90	Q8VE37	Regulator of chromosome condensation	Rcc1	3985	14	14	14	53	53	53	44.93	0.0000	323.31	42334000	279	16.86	17.09	18.33	17.27	16.96	17.83	17.55	17.81
0.090	-0.15	0.90	Q91VH6	Protein MEMO1	Memo1	4062	12	12	12	63.6	63.6	63.6	33.692	0.0000	117.58	30102000	282	16.48	16.17	14.16	16.64	16.04	16.51	15.81	15.69
0.109	-0.15	0.90	Q8BK08	Transmembrane protein 11, mitochondrial	Tmem11	3092	4	4	4	26.3	26.3	26.3	21.311	0.0000	11.545	3318300	46	14.37	13.65	13.42	12.52	14.48	13.26	13.86	12.96
0.124	-0.15	0.90	Q91VY9	Zinc finger protein 622	Znf622	4085	13	13	13	37	37	37	53.451	0.0000	65.582	12109000	113	14.73	14.76	16.31	14.68	14.97	15.86	14.89	15.34
0.520	-0.15	0.90	Q9CYG7	Mitochondrial import receptor subunit TOM34	Tomm34	4796	27	27	27	78	78	78	34.278	0.0000	262.76	143170000	696	18.03	18.01	18.09	18.12	18.01	18.58	18.23	18.03
0.123	-0.15	0.90	Q80XL6	Acyl-CoA dehydrogenase family member 11	Acad11	2885	10	10	10	18.4	18.4	18.4	87.365	0.0000	75.791	7086200	77	14.77	13.73	12.64	13.47	13.98	14.09	13.54	13.61
0.333	-0.15	0.90	O08915	AH receptor-interacting protein	Aip	311	15	15	15	51.8	51.8	51.8	37.605	0.0000	61.257	32254000	273	16.04	16.24	16.55	16.36	16.02	16.66	16.38	16.75
0.287	-0.15	0.90	Q61084	Mitogen-activated protein kinase kinase kinase 3	Map3k3	2248	9	9	6	19.8	19.8	13.6	70.775	0.0000	50.576	6811800	52	14.59	13.84	14.43	14.71	14.25	14.78	14.60	14.55
0.338	-0.15	0.90	Q8BH02	Torsin-4A	Tor4a	3004	12	12	12	39	39	39	47.585	0.0000	146.44	22049000	206	16.62	16.17	16.43	16.86	16.50	17.03	16.50	16.64
0.167	-0.15	0.90	P24668	Cation-dependent mannose-6-phosphate receptor	M6pr	902	7	7	7	28.1	28.1	28.1	31.172	0.0000	316.36	96721000	211	18.75	18.57	17.36	18.89	18.49	18.51	18.70	18.48
0.241	-0.15	0.90	Q99MR6	Serrate RNA effector molecule homolog	Srrt	4474	46	46	46	44.2	44.2	44.2	100.45	0.0000	258.49	79269000	631	17.37	17.55	17.90	17.28	17.21	17.96	17.41	18.15
0.139	-0.15	0.90	Q62311	Transcription initiation factor TFIID subunit 6	Taf6	2376	2	2	2	5.6	5.6	5.6	72.673	0.0000	14.221	1090500	18	13.66	12.87	14.15	12.90	13.20	13.24	13.36	14.39
0.141	-0.16	0.90	P68033;P68	Actin, alpha cardiac muscle 1;Actin, alpha skeletal muscle;Actin, gamm	Actc1;Acta1;Actg2	1511	39	11	10	66.6	35.8	31.6	42.019	0.0000	323.31	785630000	489	21.68	21.77	20.18	21.69	21.65	21.06	21.90	21.35
0.063	-0.16	0.90	Q9R210	Transcription factor EB	Tfeb	5669	5	3	3	11.2	7.8	7.8	52.614	0.0000	48.651	4596900	50	13.75	15.37	12.47	14.96	15.14	14.05	12.69	15.31
0.210	-0.16	0.90	Q8BGK6	Y+L amino acid transporter 2	Slc7a6	2986	3	3	2	9.7	9.7	8.3	56.77	0.0000	31.551	11291000	60	16.00	16.12	15.47	16.27	16.76	15.84	16.24	15.66
0.605	-0.16	0.90	Q9WV85	Nucleoside diphosphate kinase 3	Nme3	5738	7	7	7	63.3	63.3	63.3	19.099	0.0000	59.355	18159000	100	16.97	16.81	16.87	16.81	16.75	16.95	17.08	17.32
0.261	-0.16	0.90	Q7TMY8	E3 ubiquitin-protein ligase HUWE1	Huwe1	2754	37	37	37	15.2	15.2	15.2	482.63	0.0000	323.31	34529000	438	15.67	15.11	16.23	15.81	15.68	15.93	16.09	15.76
0.263	-0.16	0.90	Q9D2Y4	Mixed lineage kinase domain-like protein	Mlkl	4955	11	11	11	25	25	25	54.317	0.0000	38.651	12247000	120	15.60	15.24	15.24	14.97	15.42	15.83	15.60	14.84
0.123	-0.16	0.90	Q640M1	U3 small nucleolar RNA-associated protein 14 homolog A	Utp14a	2414	11	11	10	22.2	22.2	20.5	87.264	0.0000	29.682	2498300	24	13.36	13.02	14.86	13.72	14.25	14.05	13.09	14.21
0.483	-0.16	0.90	P97465	Docking protein 1	Dok1	1654	13	13	13	34.9	34.9	34.9	52.451	0.0000	93.984	35039000	298	16.28	15.84	15.81	16.18	16.01	16.40	16.27	16.07
0.111	-0.16	0.89	Q91WC0	Histone-lysine N-methyltransferase setd3	Setd3	4103	13	13	13	31.8	31.8	31.8	67.175	0.0000	111.72	17667000	214	15.70	15.78	14.30	15.87	15.54	16.14	16.10	14.51
0.199	-0.16	0.89	P42225	Signal transducer and activator of transcription 1	Stat1	1075	49	49	49	57.3	57.3	57.3	87.196	0.0000	323.31	208230000	1213	17.14	16.60	17.83	17.57	17.07	17.26	17.84	17.60
0.356	-0.16	0.89	Q9DCM0	Persulfide dioxygenase ETHE1, mitochondrial	Ethe1	5222	13	13	13	65.7	65.7	65.7	27.738	0.0000	84.124	31866000	280	16.14	16.17	15.36	15.95	15.94	16.17	16.02	16.12
0.108	-0.16	0.89	Q62191	E3 ubiquitin-protein ligase TRIM21	Trim21	2366	10	10	10	23	23	23	54.174	0.0000	55.282	8545700	108	14.26	14.28	16.05	14.83	14.84	14.33	16.02	14.88
0.093	-0.16	0.89	Q8R322	Nucleoporin GLE1	Gle1	3831	6	6	6	12	12	12	79.573	0.0000	13.241	5486100	30	14.52	14.72	15.61	14.27	15.89	13.99	15.95	13.94
0.224	-0.16	0.89	O08539	Myc box-dependent-interacting protein 1	Bin1	269	21	19	19	40	40	40	64.469	0.0000	206.57	51028000	447	16.90	17.29	16.79	16.88	17.28	17.51	16.34	17.38
0.166	-0.16	0.89	P14434;P14	H-2 class II histocompatibility antigen, A-B alpha chain;H-2 class II histo	H2-Aa	780	6	6	6	27	27	27	28.093	0.0000	116.82	21872000	125	16.16	16.72	16.67	15.23	16.77	16.31	16.33	16.03
0.339	-0.16	0.89	Q8BFY9	Transportin-1	Tnpo1	2953	22	22	15	38.2	38.2	29.4	102.36	0.0000	206.46	57330000	291	16.72	16.77	17.29	16.72	16.76	16.84	17.42	17.13
0.368	-0.16	0.89	Q9ER38	Torsin-3A	Tor3a	5292	11	11	11	33.8	33.8	33.8	43.813	0.0000	74.736	27922000	161	17.02	16.76	16.60	17.04	17.00	16.84	17.47	16.76
0.181	-0.16	0.89	Q8C5W3	Tubulin-specific chaperone cofactor E-like protein	Tbcel	3402	8	8	8	30.9	30.9	30.9	48.031	0.0000	28.095	7441000	91	15.43	15.46	14.54	14.24	15.43	14.66	15.16	15.06
0.134	-0.16	0.89	Q99PP6	Tripartite motif-containing protein 34A	Trim34a	4511	11	11	11	29.3	29.3	29.3	55.907	0.0000	71.578	4325300	35	12.73	12.82	14.06	12.84	12.50	12.94	13.89	13.77
0.067	-0.16	0.89	Q9D898	Actin-related protein 2/3 complex subunit 5-like protein	Arpc5l	5056	4	4	4	26.1	26.1	26.1	16.98	0.0000	22.113	9363400	51	16.14	15.08	13.51	14.54	14.54	15.72	13.31	16.36
0.460	-0.16	0.89	O54774	AP-3 complex subunit delta-1	Ap3d1	419	34	34	34	37.8	37.8	37.8	135.08	0.0000	323.31	111700000	763	17.59	17.70	18.15	17.68	17.91	17.98	18.17	17.71
0.631	-0.16	0.89	P09411	Phosphoglycerate kinase 1	Pgk1	696	62	62	54	99.5	99.5	97.4	44.55	0.0000	323.31	3891600000	4039	21.74	21.54	21.59	21.85	21.77	22.04	21.97	21.60
0.196	-0.16	0.89	Q8VEA8	Ras-related protein Rab-7b	Rab7b	3997	6	6	6	37.7	37.7	37.7	22.502	0.0000	36.459	8470500	91	14.24	14.68	15.51	14.14	14.92	15.01	14.78	14.52
0.489	-0.16	0.89	P35279;P61	Ras-related protein Rab-6A;Ras-related protein Rab-6B	Rab6a;Rab6b	1014	16	16	14	64.9	64.9	58.7	23.59	0.0000	139.85	185030000	452	20.03	19.99	19.53	19.71	19.78	20.11	19.85	20.18
0.393	-0.17	0.89	O35609	Secretory carrier-associated membrane protein 3	Scamp3	386	11	11	11	26.4	26.4	26.4	38.458	0.0000	293.2	77611000	368	17.69	17.64	17.45	17.75	17.66	18.23	17.89	17.42
0.100	-0.17	0.89	Q2YDW2	Protein misato homolog 1	Msto1	1804	9	9	9	25.9	25.9	25.9	61.23	0.0000	33.596	5353400	37	14.94	14.81	14.67	12.74	14.22	15.17	14.72	13.71
0.138	-0.17	0.89	Q9DBZ5	Eukaryotic translation initiation factor 3 subunit K	Eif3k	5185	10	10	10	50.5	50.5	50.5	25.086	0.0000	63.039	49424000	228	17.02	17.60	15.69	17.51	17.32	17.28	16.98	16.89

0.184	-0.17	0.89	Q80U87	Ubiquitin carboxyl-terminal hydrolase 8	Usp8	2825	25	25	25	33.7	33.7	33.7	122.61	0.0000	292.63	36406000	306	15.99	15.74	17.14	15.68	16.07	16.41	16.45	16.28
0.486	-0.17	0.89	Q9ER72	Cysteine--tRNA ligase, cytoplasmic	Cars	5296	46	46	46	68.1	68.1	68.1	94.859	0.0000	323.31	101720000	740	16.92	16.74	16.41	17.05	16.75	17.01	17.07	16.95
0.211	-0.17	0.89	E9Q1P8	Interferon regulatory factor 2-binding protein 2	Irf2bp2	228	12	12	11	28.9	28.9	27.7	59.291	0.0000	74.301	11372000	134	15.30	15.78	16.07	15.08	15.92	16.16	15.13	15.67
0.210	-0.17	0.89	Q8K2L8	Trafficking protein particle complex subunit 12	Trappc12	3665	8	8	8	16.2	16.2	16.2	87.693	0.0000	49.236	11535000	79	16.11	16.27	15.42	16.39	16.18	16.47	16.63	15.57
0.463	-0.17	0.89	Q9D1G1	Ras-related protein Rab-1B	Rab1b	4911	25	14	13	83.1	59.7	54.2	22.187	0.0000	323.31	243490000	698	18.89	18.82	19.34	18.85	18.85	19.20	19.37	19.14
0.212	-0.17	0.89	Q9CYR0	Single-stranded DNA-binding protein, mitochondrial	Ssbp1	4805	7	7	7	43.4	43.4	43.4	17.319	0.0000	106.78	24510000	171	16.91	17.31	16.03	16.64	17.02	17.27	16.76	16.51
0.269	-0.17	0.89	P53564	Homeobox protein cut-like 1	Cux1	1232	28	28	16	28.4	28.4	16.9	165.59	0.0000	169.48	32110000	354	15.81	16.37	16.50	15.66	15.93	16.58	16.05	16.46
0.413	-0.17	0.89	Q8K354	Carbonyl reductase [NADPH] 3	Cbr3	3690	27	27	26	84.5	84.5	81.6	30.953	0.0000	260.56	141670000	755	18.21	18.38	18.40	17.94	18.21	18.81	18.18	18.40
0.378	-0.17	0.89	Q9CQP0	39S ribosomal protein L33, mitochondrial	Mrpl33	4618	4	4	4	53.8	53.8	53.8	7.4158	0.0000	7.3798	6960300	80	15.19	14.91	14.92	15.42	15.26	15.29	15.66	14.92
0.638	-0.17	0.89	Q9JLQ2	ARF GTPase-activating protein GIT2	Git2	5488	36	36	32	67.2	67.2	63.8	78.765	0.0000	323.31	85527000	694	17.10	16.74	17.18	17.12	17.00	17.35	17.30	17.15
0.070	-0.17	0.89	Q6P2K6	Serine/threonine-protein phosphatase 4 regulatory subunit 3A	Smek1	2557	6	6	4	10.6	10.6	8	93.841	0.0000	21.423	11905000	66	16.66	16.53	13.44	16.31	16.59	15.93	14.73	16.38
0.527	-0.17	0.89	Q8VE47	Ubiquitin-like modifier-activating enzyme 5	Uba5	3986	12	12	12	42.9	42.9	42.9	44.789	0.0000	231.59	41472000	262	16.99	16.54	16.73	17.05	16.82	17.12	17.20	16.85
0.355	-0.17	0.89	Q9JL26	Formin-like protein 1	Fmnl1	5470	59	59	54	45.6	45.6	44.6	122.06	0.0000	323.31	187780000	1151	17.94	17.89	18.22	18.40	17.90	18.10	18.57	18.58
0.352	-0.17	0.89	Q61543	Golgi apparatus protein 1	Glg1	2309	28	28	28	28.8	28.8	28.8	133.73	0.0000	65.819	38166000	350	16.11	15.74	16.71	16.09	16.52	16.19	16.31	16.32
0.270	-0.17	0.89	P97452	Ribosome biogenesis protein BOP1	Bop1	1652	12	12	12	23.1	23.1	23.1	82.545	0.0000	100.51	14485000	198	15.21	14.78	14.11	15.09	14.88	14.93	15.27	14.81
0.396	-0.17	0.89	Q9CW46	Ribonucleoprotein PTB-binding 1	Raver1	4709	28	28	28	71.9	71.9	71.9	79.381	0.0000	323.31	40217000	320	16.08	16.13	16.78	16.42	16.34	16.81	16.58	16.39
0.191	-0.17	0.89	P28658	Ataxin-10	Atxn10	955	9	9	9	17.1	17.1	17.1	53.706	0.0000	111.76	15974000	83	16.47	16.91	15.49	15.60	16.26	16.04	16.56	16.32
0.784	-0.17	0.89	O08997	Copper transport protein ATOX1	Atox1	314	5	5	5	69.1	69.1	69.1	7.3384	0.0000	102.72	33510000	251	16.90	17.01	16.64	17.10	17.11	17.17	16.95	17.12
0.568	-0.18	0.89	Q3UVL4	Vacuolar protein sorting-associated protein 51 homolog	Vps51	2039	17	17	16	25.4	25.4	25.3	86.186	0.0000	145.47	20113000	218	15.53	15.27	15.45	15.81	15.48	15.91	15.73	15.64
0.075	-0.18	0.89	Q91V01	Lysophospholipid acyltransferase 5	Lpcat3	4039	3	3	3	7.8	7.8	7.8	56.146	0.0000	74.934	12372000	84	16.20	16.84	14.55	14.85	15.39	14.17	16.98	16.59
0.449	-0.18	0.89	Q9ERN0	Secretory carrier-associated membrane protein 2	Scamp2	5315	6	6	6	18.8	18.8	18.8	36.464	0.0000	196.41	33388000	140	17.05	17.47	17.19	17.69	17.29	17.73	17.42	17.66
0.350	-0.18	0.89	Q3U0V1	Far upstream element-binding protein 2	Khsrp	1897	37	37	34	51.2	51.2	47.6	76.775	0.0000	323.31	223730000	1145	17.95	18.52	17.92	17.99	18.39	18.53	17.80	18.37
0.256	-0.18	0.89	Q99KV1	DnaJ homolog subfamily B member 11	Dnajb11	4396	12	12	12	35.8	35.8	35.8	40.555	0.0000	107.63	47749000	229	17.42	17.62	17.60	17.05	17.33	18.26	17.12	17.68
0.315	-0.18	0.88	Q9R112	Sulfide:quinone oxidoreductase, mitochondrial	Sqrdl	5645	33	33	33	62.9	62.9	62.9	50.282	0.0000	323.31	170830000	778	18.11	17.71	18.76	18.18	18.61	18.26	18.42	18.18
0.403	-0.18	0.88	Q6PD03	Serine/threonine-protein phosphatase 2A 56 kDa regulatory subunit alpl	Ppp2r5a	2607	15	15	15	33.1	33.1	33.1	56.346	0.0000	41.9	15261000	221	14.88	14.96	14.60	15.24	15.09	14.70	15.29	15.31
0.186	-0.18	0.88	Q6P4S6	Serine/threonine-protein kinase SIK3	Sik3	2563	4	4	4	4.7	4.7	4.7	145.78	0.0000	13.482	1734000	24	13.37	12.73	13.53	13.19	12.45	14.03	13.52	13.52
0.377	-0.18	0.88	P11103	Poly [ADP-ribose] polymerase 1	Parp1	738	44	44	44	46.7	46.7	46.7	113.1	0.0000	224.92	123720000	925	17.82	17.84	18.66	18.25	18.15	18.44	18.40	18.30
0.169	-0.18	0.88	Q8K3G5	Inactive serine/threonine-protein kinase VRK3	Vrk3	3700	12	12	12	37.3	37.3	37.3	50.829	0.0000	24.916	7999300	89	14.32	13.91	12.87	14.31	13.99	14.32	14.44	13.39
0.177	-0.18	0.88	Q9QWY8	Arf-GAP with SH3 domain, ANK repeat and PH domain-containing prote	Asap1	5530	18	18	18	21.8	21.8	21.8	127.42	0.0000	133.11	32824000	270	16.33	16.21	17.80	16.76	16.50	17.25	17.10	16.97
0.104	-0.18	0.88	Q62230	Sialoadhesin	Siglec1	2370	38	38	38	28.6	28.6	28.6	182.98	0.0000	228.34	75068000	458	16.38	15.51	18.42	16.94	16.98	16.63	17.48	16.89
0.187	-0.18	0.88	Q8R151	NFX1-type zinc finger-containing protein 1	Znfx1	3778	43	43	43	25.8	25.8	25.8	218.83	0.0000	305.67	37743000	424	14.87	14.78	16.35	15.35	15.16	15.64	15.66	15.62
0.847	-0.18	0.88	Q99KR3	Beta-lactamase-like protein 2	Lactb2	4390	13	13	13	48.3	48.3	48.3	32.754	0.0000	45.509	41060000	311	17.07	17.14	16.87	16.83	16.99	17.07	17.24	17.33
0.182	-0.18	0.88	Q91VN4	MICOS complex subunit Mic25	Chchd6	4070	5	5	5	22	22	22	29.852	0.0000	13.553	3681000	14	13.72	15.26	14.42	13.75	14.45	14.62	14.07	14.74
0.111	-0.18	0.88	Q3UMU9	Hepatoma-derived growth factor-related protein 2	Hdgfrp2	2003	5	4	4	10.3	9.1	9.1	74.29	0.0000	29.467	2050200	40	13.09	13.16	13.90	13.54	12.04	13.52	14.09	14.76
0.671	-0.18	0.88	P29391;P49	Ferritin light chain 1;Ferritin light chain 2	Ftl1;Ftl2	968	19	19	18	69.4	69.4	69.4	20.802	0.0000	323.31	557210000	1034	19.92	19.92	19.78	19.86	20.28	20.14	20.11	19.69
0.338	-0.18	0.88	P14733	Lamin-B1	Lmnb1	785	79	77	70	83.2	83.2	75.9	66.785	0.0000	323.31	404700000	2053	18.18	18.09	18.87	18.47	18.30	18.87	18.35	18.82
0.351	-0.18	0.88	Q3TC46	Protein PAT1 homolog 1	Patl1	1823	10	10	10	16.6	16.6	16.6	86.769	0.0000	71.248	17111000	165	15.30	15.48	16.14	15.86	15.68	16.20	15.93	15.71
0.241	-0.18	0.88	Q9JHL0	Linker for activation of T-cells family member 2	Lat2	5367	4	4	4	30	30	30	22.876	0.0000	52.453	3040300	66	14.26	13.76	14.23	13.70	14.23	13.55	14.03	14.87
0.206	-0.19	0.88	Q8K2Q9	Shootin-1	Kiaa1598	3669	30	30	30	44.7	44.7	44.7	71.342	0.0000	115.59	37527000	308	15.92	16.35	17.09	16.11	16.27	17.02	16.00	16.93
0.555	-0.19	0.88	P38647	Stress-70 protein, mitochondrial	Hspa9	1050	50	50	50	67.2	67.2	67.2	73.46	0.0000	323.31	526750000	2045	18.55	18.92	18.79	18.56	18.68	19.20	18.68	19.00
0.183	-0.19	0.88	Q6ZWQ7		Spcs3	2704	7	7	7	27.8	27.8	27.8	20.313	0.0000	14.097	18940000	81	16.62	16.59	15.23	16.26	16.46	15.84	16.23	16.92
0.299	-0.19	0.88	Q60575	Kinesin-like protein KIF1B	Kif1b	2179	20	20	15	15.1	15.1	11.7	204.08	0.0000	95.719	25050000	240	15.84	15.61	16.36	15.89	15.52	16.50	16.27	16.15
0.708	-0.19	0.88	Q99JY4	TraB domain-containing protein	Trabd	4357	6	6	6	28.2	28.2	28.2	42.189	0.0000	72.763	15420000	100	16.05	15.79	16.20	15.81	16.15	16.14	16.36	15.95
0.289	-0.19	0.88	Q9EQW7	Kinesin-like protein KIF13A	Kif13a	5290	5	5	5	3.3	3.3	3.3	195.81	0.0000	23.227	5179000	65	15.24	15.09	14.56	15.79	15.38	15.62	15.16	15.27
0.218	-0.19	0.88	P97797	Tyrosine-protein phosphatase non-receptor type substrate 1	Sirpa	1671	11	11	11	34.9	34.9	34.9	56.413	0.0000	127.03	50425000	224	17.67	16.88	17.06	17.42	17.63	17.99	17.56	16.61
0.269	-0.19	0.88	Q5U5Q9	BRCA1-A complex subunit RAP80	Uimc1	2168	5	5	5	8.1	8.1	8.1	81.477	0.0000	12.979	1269700	13	12.29	12.33	12.63	12.29	13.07	11.79	12.78	12.65
0.713	-0.19	0.88	Q9JLV6	Bifunctional polynucleotide phosphatase/kinase;Polynucleotide 3-phosp	Pnkp	5492	28	28	28	63.6	63.6	63.6	57.223	0.0000	289.18	59987000	454	16.45	16.67	16.51	16.30	16.55	16.96	16.69	16.49
0.366	-0.19	0.88	Q9CRY7	Glycerophosphodiester phosphodiesterase domain-containing protein 1	Gdpd1	4695	9	9	9	27.1	27.1	27.1	35.866	0.0000	19.514	16941000	172	15.89	15.87	16.60	15.70	15.93	16.43	16.23	16.23
0.140	-0.19	0.88	Q9JM93	ADP-ribosylation factor-like protein 6-interacting protein 4	Arl6ip4	5501	3	3	3	22.3	22.3	22.3	25.524	0.0000	39.989	1821400	44	13.53	14.31	15.09	12.93	14.61	13.89	14.44	13.69
0.241	-0.19	0.88	Q99L00	HAUS augmin-like complex subunit 8	Haus8	4401	5	5	5	17.7	17.7	17.7	41.653	0.0000	18.949	2707300	18	13.91	13.42	13.03	14.03	14.01	13.28	13.58	14.29
0.087	-0.19	0.88	P26350	Prothymosin alpha;Prothymosin alpha, N-terminally processed;Thymosi	Ptma	922	2	2	2	15.3	15.3	15.3	12.254	0.0000	10.423	1633600	18	13.73	13.27	13.60	13.80	13.38	16.10	13.22	12.47
0.482	-0.19	0.88	Q63844	Mitogen-activated protein kinase 3	Mapk3	2405	25	25	19	70	70	60.8	43.066	0.0000	323.31	272570000	764	19.49	19.54	18.90	19.43	19.55	19.82	19.41	19.35
0.621	-0.19	0.87	Q9D0F3	Protein ERGIC-53	Lman1	4862	14	14	14	30.9	30.9	30.9	57.788	0.0000	149.13	47630000	298	16.57	16.21	16.44	16.72	16.81	16.59	16.88	16.43
0.734	-0.19	0.87	Q9DCT1	1,5-anhydro-D-fructose reductase	Akr1e2	5229	12	12	12	48.8	48.8	48.8	34.46	0.0000	75.477	21224000	190	15.84	15.82	15.48	15.75	15.83	16.21	15.77	15.85
0.101	-0.19	0.87	P45481	CREB-binding protein	Crebbp	1096	5	4	4	3.5	2.8	2.8	265.49	0.0000	57.92	3150500	41	13.08	13.68	16.28	14.11	14.29	14.37	14.57	14.69
0.075	-0.19	0.87	P35505	Fumarylacetoacetase	Fah	1023	5	5	5	20	20	20	46.175	0.0000	10.364	2547100	18	15.01	11.75	13.76	12.78	12.82	14.65	12.13	14.47
0.276	-0.19	0.87	P08905	Lysozyme C-2	Lyz2	690	9	9	4	54.7	54.7	41.2	16.689	0.0000	210.75	496610000	487	20.81	21.51	22.10	21.24	21.51	21.85	21.35	21.73
0.162	-0.19	0.87	P49290	Eosinophil peroxidase;Eosinophil peroxidase light chain;Eosinophil perc	Epx	1158	32	29	29	47.8	44.4	44.4	81.379	0.0000	126.21	111860000	534	18.22	18.10	16.65	16.93	17.95	18.10	17.07	17.55
1.444	-0.20	0.87	Q01853	Transitional endoplasmic reticulum ATPase	Vcp	1712	88	88	87	78.9	78.9	78.9	89.321	0.0000	323.31	1160100000	3210	20.34	20.47	20.55	20.33	20.55	20.71	20.51	20.70
0.351	-0.20	0.87	Q8R088	Golgi phosphoprotein 3-like	Golph3l	3752	8	7	7	33	28.4	28.4	32.905	0.0000	22.755	4417500	29	14.25	14.05	13.30	14.07	13.86	14.17	14.03	14.41
0.692	-0.20	0.87	Q8BH86	UPF0317 protein C14orf159 homolog, mitochondrial		3022	20	20	20	39.7	39.7	39.7	66.365	0.0000	162.61	34185000	320	15.94	15.95	15.54	16.18	16.12	16.13	16.16	16.00
0.225	-0.20	0.87	P14152	Malate dehydrogenase, cytoplasmic	Mdh1	776	27	27	27	75.1	75.1	75.1	36.511	0.0000	323.31	506610000	815	19.96	19.95	18.56	19.91	19.68	20.02	19.69	19.79
0.312	-0.20	0.87	P49446	Receptor-type tyrosine-protein phosphatase epsilon	Ptpre	1164	18	18	17	36.1	36.1	33.5	80.687	0.0000	130.12	22479000	258	15.86	16.08	14.95	15.25	15.75	15.81	15.57	15.80
0.232	-0.20	0.87	Q9JJL8	Serine--tRNA ligase, mitochondrial	Sars2	5431	10	10	10	25.7	25.7	25.7	58.316	0.0000	133.11	7633200	98	14.56	14.29	13.27	14.75	14.23	14.32	14.72	14.40
0.533	-0.20	0.87	Q8BHG2	UPF0587 protein C1orf123 homolog		3037	4	4	4	31.9	31.9	31.9	18.02	0.0000	15.059	11778000	138	15.04	15.32	15.69	15.38	15.45	15.83	15.63	15.32
0.068	-0.20	0.87	Q9JL16	Interferon-stimulated gene 20 kDa protein	Isg20	5467	3	3	3	13.7	13.7	13.7	32.503	0.0000	26.807	5198800	48	13.29	12.18	16.58	14.35	13.74	13.23	15.02	15.20
0.526	-0.20	0.87	P97376	Protein FRG1	Frg1	1638	12	12	12	37.2	37.2	37.2	29.127	0.0000	31.107	19157000	159	16.17	16.23	16.61	16.76	16.80	16.73	16.70	16.34
0.099	-0.20	0.87	Q8BHK9	DNA excision repair protein ERCC-6-like	Ercc6l	3043	6	6	6	7.2	7.2	7.2	138.85	0.0000	58.601	4973200	42	14.52	14.65	16.03	12.95	14.37	14.91	15.76	13.91
0.182	-0.20	0.87	Q8BWY3	Eukaryotic peptide chain release factor subunit 1	Etf1	3270	13	13	13	38.4	38.4	38.4	49.03	0.0000	267.46	72592000	351	17.95	18.04	16.28	17.89	17.45	17.98	17.76	17.78
0.387	-0.20	0.87	P54116	Erythrocyte band 7 integral membrane protein	Stom	1248	19	19	19	65.1	65.1	65.1	31.375	0.0000	323.31	469160000	1016	19.47	19.43	20.08	19.23	19.44	19.61	20.05	19.92
0.186	-0.20	0.87	Q9CZV8	F-box/LRR-repeat protein 20	Fbxl20	4841	6	6	6	13.8	13.8	13.8	48.396	0.0000	9.2105	2458600	22	13.33	13.73	14.58	12.66	13.66	14.07	13.99	13.39
0.592	-0.21	0.87	Q8BL80	Rho GTPase-activating protein 22	Arhgap22	3112	23	23	23	40.6	40.6	40.6	77.785	0.0000	113.74	44078000	382	15.96	15.78	15.95	16.00	15.74	16.19	16.07	16.50
0.115	-0.21	0.87	Q6ZQL4	WD repeat-containing protein 43	Wdr43	2701	7	7	7	14.5	14.5	14.5	75.38	0.0000	14.987	4962600	59	14.82	14.47	14.35	12.88	15.22	12.89	14.76	14.46
0.151	-0.21	0.87	P23950	Zinc finger protein 36, C3H1 type-like 1	Zfp36l1	893	2	2	2	6.2	6.2	6.2	36.384	0.0000	18.732	728380	42	12.64	13.63	14.15	13.82	13.88	14.81	13.58	12.80
0.118	-0.21	0.87	Q99JP6	Homer protein homolog 3	Homer3	4343	7	7	7	26.4	26.4	26.4	39.694	0.0000	13.478	3124000	29	15.01	14.30	13.27	13.00	14.25	15.26	13.84	13.06
0.235	-0.21	0.87	Q3THK7	GMP synthase [glutamine-hydrolyzing]	Gmps	1846	30	30	30	58.6	58.6	58.6	76.723	0.0000	317.57	65815000	528	17.01	16.84	15.61	17.17	16.91	16.69	16.91	16.95
0.375	-0.21	0.87	P08228	Superoxide dismutase [Cu-Zn]	Sod1	683	11	11	11	54.5	54.5	54.5	15.942	0.0000	113.21	28287000	304	16.94	17.23	16.64	17.04	17.41	17.26	16.55	17.45
0.056	-0.21	0.86	Q99LH2	Phosphatidylserine synthase 1	Ptdss1	4430	3	3	3	9.3	9.3	9.3	55.603	0.0002	5.7954	9239000	43	15.72	15.97	12.33	15.62	16.00	16.52	15.81	12.16
0.143	-0.21	0.86	P32883	GTPase KRas;GTPase KRas, N-terminally processed	Kras	997	13	6	5	77.2	32.8	24.9	21.656	0.0000	34.411	11192000	132	14.83	14.92	13.77	15.30	15.02	13.63	15.32	15.68
0.120	-0.21	0.86	Q8BH15	CCR4-NOT transcription complex subunit 10	Cnot10	3007	3	3	3	6.6	6.6	6.6	81.817	0.0000	32.47	1221800	44	13.17	12.09	14.50	13.35	13.76	13.81	12.24	14.14
0.122	-0.21	0.86	P57787	Monocarboxylate transporter 4	Slc16a3	1302	3	3	3	7.9	7.9	7.9	50.373	0.0000	14.643	3455500	29	14.52	13.80	12.75	11.87	13.84	12.59	13.61	13.76
2.026	-0.21	0.86	P60710	Actin, cytoplasmic 1;Actin, cytoplasmic 1, N-terminally processed	Actb	1358	59	59	3	99.5	99.5	7.5	41.736	0.0000	323.31	27455000000	13874	24.18	24.16	24.20	24.08	24.45	24.43	24.23	24.36
0.422	-0.21	0.86	Q8K4Z5	Splicing factor 3A subunit 1	Sf3a1	3731	32	32	31	44.6	44.6	43.4	88.544	0.0000	202.27	223090000	518	19.71	19.91	20.23	19.73	19.62	20.42	20.01	20.38
0.506	-0.21	0.86	P23116	Eukaryotic translation initiation factor 3 subunit A	Eif3a	879	99	99	99	58.6	58.6	58.6	161.93	0.0000	323.31	376990000	2049	17.49	17.48	18.27	17.65	17.78	17.98	17.98	18.02
0.969	-0.21	0.86	Q9CQM9	Glutaredoxin-3	Glrx3	4613	18	18	17	60.2	60.2	59.6	37.778	0.0000	232.42	62574000	479	17.31	17.46	17.27	17.26	17.34	17.59	17.42	17.80
0.106	-0.21	0.86	Q9Z127	Large neutral amino acids transporter small subunit 1	Slc7a5	5797	3	2	2	4.7	3.3	3.3	55.872	0.0000	9.179	3022000	35	14.72	13.62	12.01	15.10	13.61	13.74	14.82	14.13
0.089	-0.22	0.86	G3X9C2	F-box only protein 50	Nccrp1	259	2	2	2	6.8	6.8	6.8	30.407	0.0025	2.7587	3858100	45	13.53	16.08	13.31	12.95	13.19	15.24	13.43	14.87
0.280	-0.22	0.86	Q9CQY5	Magnesium transporter protein 1	Magt1	4648	10	10	9	22.7	22.7	22.7	37.969	0.0000	27.617	23829000	120	16.43	17.44	16.92	16.12	17.19	16.60	17.14	16.85
1.957	-0.22	0.86	Q8BTM8	Filamin-A	Flna	3204	184	184	175	70	70	68.4	281.22	0.0000	323.31	2804600000	6735	19.97	19.98	20.01	20.18	20.16	20.31	20.27	20.27
0.759	-0.22	0.86	P62821	Ras-related protein Rab-1A	Rab1A	1447	27	27	16	86.3	86.3	58	22.677	0.0000	323.31	666880000	1365	19.95	19.75	19.71	20.19	19.92	20.24	20.29	20.02
0.492	-0.22	0.86	Q8K224	N-acetyltransferase 10	Nat10	3643	27	27	27	36.6	36.6	36.6	115.42	0.0000	323.31	28055000	409	15.17	14.92	15.80	15.33	15.35	15.55	15.73	15.47
0.395	-0.22	0.86	P0DOV2			715	42	42	25	70.4	70.4	44.6	69.437	0.0000	323.31	196010000	939	18.40	17.98	18.46	18.85	18.46	18.34	19.09	18.67
0.358	-0.22	0.86	Q922Q8	Leucine-rich repeat-containing protein 59	Lrrc59	4271	17	17	17	48.9	48.9	48.9	34.877	0.0000	194.61	294440000	621	18.84	18.58	19.66	19.01	19.18	19.30	19.55	18.93
0.103	-0.22	0.86	Q9EQQ2	Protein YIPF5	Yipf5	5286	3	3	3	22.2	22.2	22.2	27.873	0.0000	96.637	9222900	81	16.07	15.94	13.01	16.15	15.09	15.49	15.96	15.51
0.636	-0.22	0.86	Q7TMM9	Tubulin beta-2A chain	Tubb2a	2747	37	9	2	73	28.8	3.6	49.906	0.0000	210.43	95885000	296	18.18	17.85	17.87	18.51	18.33	18.47	18.20	18.29
0.310	-0.22	0.86	Q9QY06	Unconventional myosin-IXb	Myo9b	5558	56	56	54	33.5	33.5	33.1	238.83	0.0000	261.52	74847000	651	16.08	16.02	17.25	16.35	16.40	16.81	16.68	16.69
0.210	-0.22	0.86	Q60604	Adseverin	Scin	2184	3	3	3	6.6	6.6	6.6	80.253	0.0000	12.149	1863300	28	12.96	14.46	13.66	13.15	13.60	13.59	13.40	14.52
0.283	-0.22	0.86	Q9ESW4	Acylglycerol kinase, mitochondrial	Agk	5348	9	9	9	30.2	30.2	30.2	46.975	0.0000	12.987	6138100	60	14.73	15.11	15.07	14.75	15.17	14.72	15.99	14.66
0.387	-0.22	0.86	Q99N57	RAF proto-oncogene serine/threonine-protein kinase	Raf1	4483	14	14	9	33.8	33.8	25.5	72.917	0.0000	154.12	25857000	168	16.46	16.30	15.49	16.23	16.13	16.10	16.51	16.61
0.304	-0.22	0.86	Q922Q4	Pyrroline-5-carboxylate reductase 2	Pycr2	4269	14	14	14	45.3	45.3	45.3	33.659	0.0000	60.447	52523000	351	16.30	16.29	17.27	16.39	16.75	17.30	16.35	16.73
0.509	-0.22	0.86	Q9Z1R2	Large proline-rich protein BAG6	Bag6	5820	23	23	23	31.9	31.9	31.9	121.04	0.0000	216.32	41630000	326	16.59	16.61	16.36	16.82	16.60	16.58	16.74	17.34
1.054	-0.22	0.86	Q99K01	Pyridoxal-dependent decarboxylase domain-containing protein 1	Pdxdc1	4359	27	27	27	38.5	38.5	38.5	87.334	0.0000	249.93	50631000	411	16.21	16.32	16.37	16.31	16.21	16.64	16.60	16.66
0.461	-0.22	0.86	P63094;Q6F	Guanine nucleotide-binding protein G(s) subunit alpha isoforms short;G	Gnas	1490	22	20	14	53.6	49.5	39.3	45.663	0.0000	122.95	58442000	351	17.26	16.81	16.40	17.27	17.02	17.33	17.15	17.13
0.287	-0.22	0.86	Q9D0R8	Protein LSM12 homolog	Lsm12	4885	6	6	6	32.8	32.8	32.8	21.701	0.0000	16.33	15610000	123	16.12	16.09	17.15	15.62	16.40	16.53	16.45	16.50
0.429	-0.22	0.86	Q9D2M8	Ubiquitin-conjugating enzyme E2 variant 2	Ube2v2	4945	11	5	5	78.6	44.8	44.8	16.367	0.0000	85.942	39002000	191	17.44	17.23	17.88	17.10	17.28	17.88	17.47	17.91
1.055	-0.22	0.86	Q9D6J5	NADH dehydrogenase [ubiquinone] 1 beta subcomplex subunit 8, mitoc	Ndufb8	4996	6	6	6	43	43	43	21.876	0.0000	52.811	21004000	150	16.00	15.99	15.99	16.09	15.96	16.20	16.38	16.43
0.325	-0.23	0.86	Q8BXC6	COMM domain-containing protein 2	Commd2	3285	5	5	5	30.7	30.7	30.7	22.848	0.0000	21.074	6298800	100	13.81	14.80	14.54	15.02	14.70	14.65	14.58	15.15
0.165	-0.23	0.85	Q9EQ28	DNA polymerase delta subunit 3	Pold3	5270	5	5	4	13.9	13.9	12.8	50.835	0.0000	10.335	1581200	39	13.07	12.73	13.09	13.72	13.30	13.11	12.39	14.72

0.134	-0.23	0.85	O54890	Integrin beta-3	Itgb3	431	3	3	3	5.2	5.2	5.2	86.738	0.0000	38.83	2802200	45	15.40	15.36	13.59	13.53	15.27	15.36	13.86	14.29
0.893	-0.23	0.85	Q8JZV7	Putative N-acetylglucosamine-6-phosphate deacetylase	Amdhd2	3584	18	18	18	62.6	62.6	62.6	43.5	0.0000	188.49	138380000	685	17.72	17.65	18.12	17.64	18.02	17.94	17.90	18.18
0.721	-0.23	0.85	Q62203	Splicing factor 3A subunit 2	Sf3a2	2369	11	11	11	27.8	27.8	27.8	49.911	0.0000	104.2	24505000	204	15.57	15.48	15.72	15.76	15.57	16.17	15.68	16.03
0.444	-0.23	0.85	Q9QZK7	Docking protein 3	Dok3	5599	15	15	15	55.2	55.2	55.2	48.027	0.0000	73.4	37997000	300	15.67	16.00	15.21	16.00	15.63	15.87	16.30	16.00
1.655	-0.23	0.85	P29452	Caspase-1;Caspase-1 subunit p20;Caspase-1 subunit p10	Casp1	970	29	29	29	60.7	60.7	60.7	45.64	0.0000	186.35	115900000	691	17.30	17.26	17.27	17.33	17.30	17.54	17.62	17.61
0.174	-0.23	0.85	Q922R0	cAMP-dependent protein kinase catalytic subunit PRKX	Prkx	4273	5	5	5	18.6	18.6	18.6	40.466	0.0000	13.585	3372300	42	14.17	13.13	13.04	14.09	13.02	13.65	13.69	14.99
0.618	-0.23	0.85	Q64704	Syntaxin-3	Stx3	2459	14	14	14	41.5	41.5	41.5	33.243	0.0000	236.06	26282000	191	16.25	16.11	15.83	16.43	16.08	16.38	16.68	16.41
0.425	-0.23	0.85	Q8BMI3	ADP-ribosylation factor-binding protein GGA3	Gga3	3139	12	11	11	22.1	21.2	21.2	77.972	0.0000	85.72	14473000	144	15.61	15.35	15.27	14.70	15.07	15.79	15.47	15.51
0.146	-0.23	0.85	Q673H1	Tumor suppressor candidate gene 1 protein homolog	Tusc1	2469	4	4	4	18	18	18	22.712	0.0000	22.836	4551100	61	13.16	15.31	15.07	15.46	14.38	14.68	15.57	15.30
0.171	-0.23	0.85	Q9DC48	Pre-mRNA-processing factor 17	Cdc40	5192	7	7	6	20.2	20.2	16.8	65.46	0.0000	34.003	3314200	33	14.10	13.05	12.53	14.12	13.89	13.74	14.37	12.73
0.350	-0.23	0.85	P58389	Serine/threonine-protein phosphatase 2A activator	Ppp2r4	1314	16	16	16	68.4	68.4	68.4	36.71	0.0000	323.31	63609000	388	17.34	17.62	16.30	17.04	17.30	17.31	17.35	17.27
0.552	-0.23	0.85	P97379	Ras GTPase-activating protein-binding protein 2	G3bp2	1640	15	12	12	33	29.5	29.5	54.087	0.0000	60.342	20168000	221	15.73	16.06	15.62	15.63	15.89	16.49	15.85	15.75
0.534	-0.23	0.85	Q8CCP0	Nuclear export mediator factor Nemf	Nemf	3452	24	24	24	26.8	26.8	26.8	121.19	0.0000	65.838	26964000	280	15.86	15.84	16.69	16.00	16.24	16.43	16.33	16.33
0.447	-0.24	0.85	Q8R5A6	TBC1 domain family member 22A	Tbc1d22a	3883	15	15	15	37.6	37.6	37.6	59.362	0.0000	98.294	20132000	168	15.89	16.08	15.42	15.18	15.79	15.77	16.21	15.73
0.335	-0.24	0.85	Q6ZWY3	40S ribosomal protein S27-like	Rps27l	2710	6	3	3	41.7	15.5	15.5	9.4771	0.0000	15.159	52000000	122	18.78	18.18	17.39	18.43	18.53	18.43	18.44	18.32
0.162	-0.24	0.85	Q8K2T8	RNA polymerase II-associated factor 1 homolog	Paf1	3673	4	4	4	11.2	11.2	11.2	60.518	0.0000	14.616	2773500	37	14.08	14.15	14.51	13.55	14.34	15.10	14.99	12.81
0.757	-0.24	0.85	P56477	Interferon regulatory factor 5	Irf5	1287	16	16	16	43.5	43.5	43.5	56.004	0.0000	80.705	33576000	293	15.86	15.94	16.13	16.18	15.87	16.36	16.40	16.44
0.744	-0.24	0.85	Q9D1B9	39S ribosomal protein L28, mitochondrial	Mrpl28	4904	13	13	13	51.4	51.4	51.4	30.169	0.0000	74.389	42045000	328	15.86	16.12	15.64	15.61	15.97	16.32	15.84	16.05
0.443	-0.24	0.85	Q91VR8	Protein BRICK1	Brk1	4074	10	10	10	96	96	96	8.7608	0.0000	37.598	34739000	127	16.74	17.24	17.39	17.19	16.88	17.56	17.28	17.80
0.330	-0.24	0.85	Q9CZM2	60S ribosomal protein L15	Rpl15	4833	17	17	17	59.8	59.8	59.8	24.146	0.0000	93.054	126080000	358	18.44	18.45	19.62	19.01	19.00	18.89	19.12	19.48
0.965	-0.24	0.85	Q7TMQ7	WD repeat-containing protein 91	Wdr91	2748	28	28	28	41.2	41.2	41.2	83.42	0.0000	212.02	54782000	500	16.51	16.34	16.51	16.78	16.53	16.91	16.78	16.88
0.316	-0.24	0.85	Q99L13	3-hydroxyisobutyrate dehydrogenase, mitochondrial	Hibadh	4403	18	18	18	63	63	63	35.44	0.0000	323.31	75549000	539	17.22	17.71	18.13	16.94	17.69	18.24	17.34	17.68
0.891	-0.24	0.85	O70378	ER membrane protein complex subunit 8	Emc8	492	8	8	8	52.2	52.2	52.2	23.348	0.0000	106.12	33064000	284	16.39	16.39	16.21	16.24	16.40	16.93	16.48	16.39
0.283	-0.24	0.84	Q9DAV9	Trimeric intracellular cation channel type B	Tmem38b	5113	4	4	4	13.7	13.7	13.7	32.64	0.0000	11.588	7771900	81	15.47	15.42	14.76	16.30	15.36	15.69	15.71	16.16
0.649	-0.24	0.84	Q8BX57	PX domain-containing protein kinase-like protein	Pxk	3278	11	11	11	26.8	26.8	26.8	65.23	0.0000	31.468	7964000	101	14.93	14.54	14.83	15.17	14.74	15.19	15.24	15.27
0.390	-0.24	0.84	P15379	CD44 antigen	Cd44	796	5	5	5	8.7	8.7	8.7	85.616	0.0000	40.254	57891000	174	18.38	18.30	17.34	18.45	18.39	18.19	18.27	18.58
0.576	-0.24	0.84	P63276	40S ribosomal protein S17	Rps17	1501	17	17	17	83.7	83.7	83.7	15.524	0.0000	323.31	532160000	813	20.29	20.02	19.69	20.36	20.18	20.18	20.71	20.27
0.772	-0.25	0.84	Q8CIN4	Serine/threonine-protein kinase PAK 2;PAK-2p27;PAK-2p34	Pak2	3561	40	40	29	76.1	76.1	57.3	57.93	0.0000	323.31	274290000	1154	19.02	19.07	18.66	19.31	19.12	19.48	19.15	19.28
0.417	-0.25	0.84	Q9CR51	V-type proton ATPase subunit G 1	Atp6v1g1	4667	10	10	10	55.1	55.1	55.1	13.724	0.0000	323.31	35276000	204	16.54	17.33	16.52	16.95	17.52	16.81	17.21	16.77
0.843	-0.25	0.84	O88456	Calpain small subunit 1	Capns1	533	26	26	26	66.5	66.5	66.5	28.463	0.0000	323.31	647540000	1424	19.66	19.85	19.92	19.59	19.82	19.89	19.92	20.37
0.174	-0.25	0.84	Q99M08	Uncharacterized protein C4orf3 homolog		4452	2	2	2	24.6	24.6	24.6	7.4043	0.0000	48.406	28367000	91	17.40	16.98	18.82	16.48	17.39	18.33	17.45	17.49
0.835	-0.25	0.84	Q8VD58	Protein EVI2B	Evi2b	3940	5	5	5	11	11	11	47.993	0.0000	19.254	10913000	134	15.03	15.18	14.81	14.82	15.24	14.92	15.50	15.18
1.027	-0.25	0.84	Q8BSY0	Aspartyl/asparaginyl beta-hydroxylase	Asph	3193	36	36	36	52.9	52.9	52.9	83.041	0.0000	314.68	88165000	761	17.17	16.89	17.07	17.28	17.15	17.23	17.53	17.48
0.293	-0.25	0.84	P70697	Uroporphyrinogen decarboxylase	Urod	1585	15	15	15	61	61	61	40.691	0.0000	170.06	27493000	191	16.51	16.64	15.58	16.68	16.61	15.92	17.00	16.87
0.133	-0.25	0.84	P04370	Myelin basic protein	Mbp	641	4	4	4	14.8	14.8	14.8	27.167	0.0000	11.048	3443900	45	13.55	14.13	13.63	14.47	12.73	14.01	14.05	15.99
0.287	-0.25	0.84	Q8K4J6	MKL/myocardin-like protein 1	Mkl1	3718	6	5	5	12.8	11.4	11.4	102.54	0.0000	39.183	2627400	50	13.93	14.64	14.40	14.53	15.40	13.81	14.60	14.69
0.754	-0.25	0.84	P97496	SWI/SNF complex subunit SMARCC1	Smarcc1	1664	13	4	4	13.4	6.7	6.7	122.89	0.0000	44.207	5854800	63	15.07	14.80	15.07	14.55	14.96	15.43	15.07	15.03
0.557	-0.25	0.84	Q78ZA7	Nucleosome assembly protein 1-like 4	Nap1l4	2732	16	16	14	56.3	56.3	53.6	42.679	0.0000	186.36	64991000	325	17.23	17.21	16.79	17.22	16.95	17.16	17.63	17.70
0.526	-0.25	0.84	P46062	Signal-induced proliferation-associated protein 1	Sipa1	1102	27	27	27	32.1	32.1	32.1	112.06	0.0000	155.61	54544000	404	16.89	16.90	16.22	17.13	16.78	17.14	17.25	16.97
0.635	-0.25	0.84	P19258	Protein Mpv17	Mpv17	845	6	6	6	34.7	34.7	34.7	19.686	0.0000	153.21	14984000	131	14.99	15.01	15.29	15.50	15.19	15.23	15.59	15.79
0.137	-0.25	0.84	Q9EPL0	Xylosyltransferase 2	Xylt2	5258	5	5	5	6.4	6.4	6.4	96.81	0.0000	8.9232	3086800	37	14.16	13.15	13.66	12.54	13.60	14.19	14.76	11.96
0.165	-0.25	0.84	Q6PGG6	Guanine nucleotide-binding protein-like 3-like protein	Gnl3l	2637	3	3	3	9	9	9	65.194	0.0000	10.726	3282800	15	14.37	14.53	14.33	14.11	15.05	15.87	14.32	13.11
0.590	-0.25	0.84	O08573	Galectin-9	Lgals9	273	12	12	12	48.7	48.7	48.7	40.035	0.0000	223.23	57999000	214	17.03	16.90	17.59	17.34	17.28	17.55	17.80	17.24
1.149	-0.25	0.84	Q3UDP0	WD repeat-containing protein 41	Wdr41	1949	12	12	12	38.3	38.3	38.3	51.51	0.0000	38.701	12123000	112	15.33	15.47	15.07	15.51	15.52	15.48	15.72	15.67
0.339	-0.25	0.84	Q9D2V5	Protein AAR2 homolog	Aar2	4952	3	3	3	13.3	13.3	13.3	43.408	0.0000	12.711	2052100	26	14.10	14.27	13.12	14.35	14.25	13.91	14.60	14.09
0.542	-0.25	0.84	Q8R326	Paraspeckle component 1	Pspc1	3833	12	12	12	34.2	34.2	34.2	58.758	0.0000	88.849	21582000	215	14.95	15.37	15.07	15.22	14.88	15.84	15.48	15.42
0.210	-0.26	0.84	Q9CQ75	NADH dehydrogenase [ubiquinone] 1 alpha subcomplex subunit 2	Ndufa2	4570	5	5	5	39.4	39.4	39.4	10.916	0.0000	31.991	43827000	92	17.17	17.59	19.01	17.22	17.80	18.64	17.98	17.61
0.207	-0.26	0.84	Q8BWG9	Calcium release-activated calcium channel protein 1	Orai1	3259	2	2	2	10.9	10.9	10.9	33.061	0.0000	22.65	2524100	28	13.56	13.79	13.91	13.16	13.35	14.96	14.25	12.88
0.441	-0.26	0.84	Q64727	Vinculin	Vcl	2461	69	69	69	69.6	69.6	69.6	116.72	0.0000	323.31	274040000	1600	17.68	17.45	18.52	17.72	17.76	18.25	18.26	18.14
1.668	-0.26	0.84	Q9Z0R6	Intersectin-2	Itsn2	5782	33	33	33	23.4	23.4	23.4	188.91	0.0000	232.1	37682000	371	15.43	15.61	15.78	15.63	15.79	15.84	15.99	15.86
0.498	-0.26	0.84	Q6PDG8	Vacuolar fusion protein MON1 homolog A	Mon1a	2612	4	4	4	11.7	11.7	11.7	62.131	0.0000	33.898	2517100	33	13.79	12.96	13.81	13.56	13.70	13.68	14.18	13.61
0.851	-0.26	0.84	Q9ESU6	Bromodomain-containing protein 4	Brd4	5346	10	9	5	9.9	9	6.2	155.89	0.0000	19.869	5120500	53	13.96	14.18	14.25	13.69	14.34	14.26	14.46	14.05
0.645	-0.26	0.83	Q8CFE4	SCY1-like protein 2	Scyl2	3483	16	16	16	29	29	29	103.32	0.0000	139.38	19157000	214	15.95	16.20	15.88	16.28	15.87	16.55	16.33	16.61
0.679	-0.26	0.83	Q08943	FACT complex subunit SSRP1	Ssrp1	1769	17	17	17	28.7	28.7	28.7	80.859	0.0000	122.33	26127000	240	16.15	16.45	16.40	15.68	16.26	16.54	16.44	16.49
1.034	-0.26	0.83	P32233	Developmentally-regulated GTP-binding protein 1	Drg1	996	16	16	16	44.4	44.4	44.4	40.512	0.0000	308.73	31232000	295	16.03	15.96	16.14	15.72	16.00	16.39	16.38	16.13
0.664	-0.26	0.83	Q62384	Zinc finger protein ZPR1	Zpr1	2384	6	6	6	20	20	20	50.714	0.0000	59.74	5833500	77	14.46	14.47	13.87	14.48	14.64	14.28	14.84	14.57
0.633	-0.26	0.83	Q8BSE0	Regulator of microtubule dynamics protein 2	Rmdn2	3186	2	2	2	6.8	6.8	6.8	47.016	0.0002	5.6962	4256500	56	14.93	14.49	14.13	14.21	14.47	14.78	14.77	14.79
0.207	-0.26	0.83	Q9D9V3	Ethylmalonyl-CoA decarboxylase	Echdc1	5103	8	8	8	39.4	39.4	39.4	35.467	0.0000	41.455	5954200	47	14.56	14.69	13.73	14.95	14.71	14.64	15.85	13.77
0.518	-0.26	0.83	P47856	Glutamine--fructose-6-phosphate aminotransferase [isomerizing] 1	Gfpt1	1127	11	11	11	24	24	24	78.538	0.0000	143.11	19633000	200	16.13	16.13	16.25	15.77	16.27	16.87	16.35	15.85
0.922	-0.26	0.83	O09044	Synaptosomal-associated protein 23	Snap23	319	18	18	18	85.2	85.2	85.2	23.261	0.0000	323.31	129520000	527	18.24	18.02	18.07	18.54	18.23	18.60	18.60	18.50
0.211	-0.27	0.83	Q9D125	28S ribosomal protein S25, mitochondrial	Mrps25	4893	4	4	4	21.6	21.6	21.6	19.92	0.0000	32.195	4116600	45	13.20	13.28	14.27	15.18	14.16	13.78	14.63	14.41
0.437	-0.27	0.83	Q8BIG7	Catechol O-methyltransferase domain-containing protein 1	Comtd1	3059	13	13	13	67.2	67.2	67.2	28.96	0.0000	178.93	35351000	254	16.36	16.36	17.19	16.79	17.17	16.41	16.98	17.21
0.482	-0.27	0.83	Q80X41	Serine/threonine-protein kinase VRK1	Vrk1	2869	18	18	18	45	45	45	49.74	0.0000	175.9	38151000	360	16.78	16.59	17.43	17.22	16.86	17.57	17.19	17.48
0.552	-0.27	0.83	Q9Z2U1	Proteasome subunit alpha type-5	Psma5	5865	10	10	10	56.8	56.8	56.8	26.411	0.0000	207.01	49333000	218	17.60	17.93	17.00	17.54	17.64	18.00	17.54	17.97
0.174	-0.27	0.83	Q9D287	Pre-mRNA-splicing factor SPF27	Bcas2	4938	4	4	4	30.7	30.7	30.7	26.131	0.0000	98.838	13459000	146	16.20	16.44	14.04	16.59	16.15	16.20	16.03	15.95
1.143	-0.27	0.83	Q8BM55	Transmembrane protein 214	Tmem214	3127	26	26	26	36.5	36.5	36.5	76.429	0.0000	233.52	35401000	340	15.87	15.92	16.25	15.70	16.08	16.27	16.25	16.21
1.005	-0.27	0.83	P63017	Heat shock cognate 71 kDa protein	Hspa8	1479	69	69	50	91	91	79.7	70.87	0.0000	323.31	3808000000	4527	21.51	21.74	21.64	21.63	21.60	22.21	21.80	21.99
0.745	-0.27	0.83	Q8R4R6	Nucleoporin NUP53	Nup35	3872	13	13	13	67.4	67.4	67.4	34.785	0.0000	87.458	30110000	224	16.25	16.11	16.29	15.88	16.11	16.80	16.23	16.48
0.670	-0.27	0.83	O70325	Phospholipid hydroperoxide glutathione peroxidase, mitochondrial	Gpx4	489	14	14	14	62.4	62.4	62.4	22.228	0.0000	112.55	98872000	515	17.72	17.67	17.91	17.83	18.23	17.95	18.45	17.59
0.864	-0.27	0.83	Q91VI7	Ribonuclease inhibitor	Rnh1	4063	43	43	43	92.1	92.1	92.1	49.816	0.0000	323.31	1307700000	2379	20.26	20.40	19.71	20.24	20.34	20.53	20.44	20.39
0.841	-0.27	0.83	P68037	Ubiquitin-conjugating enzyme E2 L3	Ube2l3	1512	20	20	20	89.6	89.6	89.6	17.861	0.0000	239.7	94728000	369	17.66	18.23	18.31	18.07	18.25	18.46	18.20	18.45
0.562	-0.27	0.83	B1AZI6	THO complex subunit 2	Thoc2	75	17	17	17	14.3	14.3	14.3	182.77	0.0000	92.387	14577000	173	15.48	15.98	15.56	15.71	15.84	16.19	16.34	15.45
0.557	-0.27	0.83	Q8C3Q9	Caspase-9;Caspase-9 subunit p35;Caspase-9 subunit p10	Casp9	3378	6	6	6	22.9	22.9	22.9	49.979	0.0000	104.2	10712000	134	15.23	14.85	15.76	14.82	15.36	15.50	15.58	15.30
1.287	-0.27	0.83	Q80VA0	N-acetylgalactosaminyltransferase 7	Galnt7	2847	15	15	15	27.1	27.1	27.1	75.418	0.0000	99.928	33298000	258	16.44	16.55	16.29	16.59	16.50	16.93	16.81	16.73
0.486	-0.27	0.83	Q91VM5	RNA binding motif protein, X-linked-like-1	Rbmxl1	4068	23	23	6	46.9	46.9	14.7	42.161	0.0000	100.79	55475000	417	16.45	16.37	17.07	16.71	16.59	17.23	16.57	17.33
0.164	-0.27	0.83	O35900	U6 snRNA-associated Sm-like protein LSm2	Lsm2	411	4	4	4	49.5	49.5	49.5	10.834	0.0000	33.215	18895000	121	16.53	16.61	15.34	17.31	16.69	17.97	16.70	15.52
0.741	-0.28	0.83	Q9D2V7	Coronin-7	Coro7	4953	42	42	42	71.4	71.4	71.4	100.81	0.0000	323.31	240490000	1014	18.11	18.14	18.60	17.89	18.23	18.70	18.58	18.35
0.980	-0.28	0.83	P51410	60S ribosomal protein L9	Rpl9	1194	18	18	18	75.5	75.5	75.5	21.881	0.0000	323.31	241160000	716	19.72	19.66	19.56	20.11	20.26	20.01	20.02	19.88
0.831	-0.28	0.83	Q8K1B8	Fermitin family homolog 3	Fermt3	3622	47	47	47	73.7	73.7	73.7	75.634	0.0000	323.31	321670000	1289	18.77	18.95	18.32	18.76	18.70	19.04	18.98	19.18
0.608	-0.28	0.83	Q76LS9	Protein FAM63A	Fam63a	2718	7	7	5	17.5	17.5	12.8	51.225	0.0000	9.8926	12034000	89	15.84	15.47	15.20	16.04	16.06	15.98	16.02	15.60
0.740	-0.28	0.82	P48678	Prelamin-A/C;Lamin-A/C	Lmna	1147	94	94	92	90.1	90.1	90.1	74.237	0.0000	323.31	862330000	2869	19.20	19.55	19.94	19.37	19.73	20.07	19.66	19.72
0.237	-0.28	0.82	Q05793	Basement membrane-specific heparan sulfate proteoglycan core protein	Hspg2	1741	7	7	7	2.9	2.9	2.9	398.29	0.0000	32.668	1963000	40	13.69	13.42	13.53	12.42	14.22	13.14	14.15	12.67
0.614	-0.28	0.82	P62835	Ras-related protein Rap-1A	Rap1a	1450	17	5	5	83.2	35.3	35.3	20.987	0.0000	33.606	19004000	164	16.34	16.40	15.53	15.93	16.23	16.32	16.21	16.55
0.128	-0.28	0.82	Q9R008	Mevalonate kinase	Mvk	5610	3	3	3	13.2	13.2	13.2	41.877	0.0000	7.8777	1090200	8	13.95	12.75	13.09	12.19	12.60	12.53	15.48	12.49
0.715	-0.28	0.82	P48722	Heat shock 70 kDa protein 4L	Hspa4l	1148	32	28	28	45.9	43	43	94.381	0.0000	109.26	29178000	257	15.61	15.63	15.20	15.14	15.28	15.72	15.94	15.77
0.343	-0.28	0.82	Q3ULW8		Parp3	1994	16	16	16	37.7	37.7	37.7	59.948	0.0000	98.377	14129000	121	15.31	14.60	15.08	15.87	15.45	14.98	16.13	15.42
0.072	-0.28	0.82	O70405	Serine/threonine-protein kinase ULK1	Ulk1	495	3	3	3	3	3	3	112.46	0.0000	9.3402	8950400	31	16.40	16.39	13.14	16.44	16.39	17.95	16.58	12.59
0.763	-0.29	0.82	Q9D8Y7	Tumor necrosis factor alpha-induced protein 8-like protein 2	Tnfaip8l2	5084	15	15	15	67.4	67.4	67.4	20.615	0.0000	129.14	93946000	345	18.48	18.33	17.72	18.28	18.30	18.68	18.48	18.49
0.201	-0.29	0.82	P15533	Tripartite motif-containing protein 30A	Trim30a	798	7	7	7	19.4	19.4	19.4	57.329	0.0000	21.242	2840000	41	12.74	12.13	14.44	13.37	13.93	13.05	13.91	12.94
0.105	-0.29	0.82	Q9CR70	EKC/KEOPS complex subunit Lage3	Lage3	4678	2	2	2	20.3	20.3	20.3	15.824	0.0000	165.67	5942800	53	15.24	11.96	16.45	15.86	15.00	14.81	15.53	15.33
0.134	-0.29	0.82	P70202	Latexin	Lxn	1535	4	4	4	25.7	25.7	25.7	25.492	0.0000	42.938	8686400	86	15.49	16.11	12.65	15.79	14.97	15.17	15.41	15.63
0.659	-0.29	0.82	P84104	Serine/arginine-rich splicing factor 3	Srsf3	1612	11	11	10	45.1	45.1	45.1	19.329	0.0000	120.85	174890000	451	18.57	18.90	18.68	19.22	19.06	19.38	18.72	19.37
0.233	-0.29	0.82	Q14AX6	Cyclin-dependent kinase 12	Cdk12	1787	5	4	4	5.1	4.6	4.6	163.68	0.0000	7.8156	2727200	48	12.96	13.10	15.05	14.00	13.94	14.05	14.46	13.82
1.037	-0.29	0.82	P62852	40S ribosomal protein S25	Rps25	1454	16	16	16	68.8	68.8	68.8	13.742	0.0000	160.92	215100000	449	18.85	19.37	19.08	18.93	19.09	19.41	19.47	19.44
0.960	-0.29	0.82	P16858	Glyceraldehyde-3-phosphate dehydrogenase	Gapdh	814	49	49	46	97.6	97.6	97.6	35.81	0.0000	323.31	6991100000	6082	22.57	22.74	22.07	22.64	22.83	22.90	22.68	22.77
0.222	-0.29	0.82	Q6PDY2	2-aminoethanethiol dioxygenase	Ado	2621	7	7	7	34.8	34.8	34.8	28.372	0.0000	13.023	13662000	76	15.69	16.22	14.44	16.45	16.34	16.10	15.17	16.37
1.181	-0.29	0.82	P18760	Cofilin-1	Cfl1	841	31	31	22	94.6	94.6	86.1	18.559	0.0000	323.31	1570800000	1815	21.69	22.07	21.46	21.78	22.05	22.11	21.95	22.07
0.500	-0.29	0.82	Q80XQ2	TBC1 domain family member 5	Tbc1d5	2889	16	16	16	32.1	32.1	32.1	91.836	0.0000	138.16	21324000	175	15.51	15.78	16.52	15.79	15.90	16.65	16.07	16.17
0.214	-0.29	0.82	Q91VC9	Growth hormone-inducible transmembrane protein	Ghitm	4056	7	7	7	14.5	14.5	14.5	37.275	0.0000	38.004	5948000	51	14.59	14.90	14.17	13.92	13.37	15.73	14.53	15.13
0.662	-0.29	0.82	Q8BWT1	3-ketoacyl-CoA thiolase, mitochondrial	Acaa2	3265	33	33	33	84.9	84.9	84.9	41.829	0.0000	323.31	363610000	1412	18.82	18.79	18.01	18.67	18.75	19.03	19.04	18.66
1.967	-0.30	0.81	O08579	Emerin	Emd	274	8	8	8	45.9	45.9	45.9	29.435	0.0000	147.67	48716000	319	17.13	16.92	17.09	17.12	17.19	17.51	17.38	17.36
0.788	-0.30	0.81	Q60668	Heterogeneous nuclear ribonucleoprotein D0	Hnrnpd	2191	23	23	22	47.9	47.9	45.6	38.354	0.0000	323.31	158740000	708	19.07	19.45	18.73	19.28	19.33	19.59	19.19	19.60
0.570	-0.30	0.81	Q8BGE6	Cysteine protease ATG4B	Atg4b	2979	9	9	9	38.7	38.7	38.7	44.375	0.0000	51.121	10116000	102	15.26	15.15	14.35	15.32	15.06	15.39	15.50	15.32
0.300	-0.30	0.81	Q99KN1	Arrestin domain-containing protein 1	Arrdc1	4384	2	2	2	9	9	9	46.329	0.0000	7.9014	2525500	14	14.12	14.08	13.63	13.23	13.74	13.48	15.10	13.93
0.527	-0.30	0.81	P97789	5-3 exoribonuclease 1	Xrn1	1670	16	16	16	12.7	12.7	12.7	194.31	0.0000	42.743	12956000	114	15.26	14.99	16.05	15.53	15.43	16.06	15.71	15.82
0.326	-0.30	0.81	Q9JM14	5(3)-deoxyribonucleotidase, cytosolic type	Nt5c	5497	8	8	8	54	54	54	23.076	0.0000	109.72	27120000	256	16.28	16.98	17.49	15.99	17.17	16.59	16.75	17.43
0.341	-0.30	0.81	Q3TAA7	Serine/threonine-protein kinase 11-interacting protein	Stk11ip	1813	6	6	6	8.1	8.1	8.1	118	0.0000	27.489	3748400	89	13.46	12.48	14.13	13.48	13.89	13.23	13.90	13.72
0.633	-0.30	0.81	Q9CSU0	Regulation of nuclear pre-mRNA domain-containing protein 1B	Rprd1b	4700	12	12	12	46.3	46.3	46.3	36.883	0.0000	147.04	19612000	202	16.14	16.07	15.42	15.76	15.72	16.45	16.24	16.18

0.954	-0.30	0.81	P11835	Integrin beta-2	Itgb2	750	37	37	37	45.1	45.1	45.1	85.025	0.0000	323.31	691180000	1282	20.55	20.24	20.85	20.33	20.89	20.79	20.95	20.55
0.617	-0.30	0.81	Q8BH74	Nuclear pore complex protein Nup107	Nup107	3020	12	12	12	20.7	20.7	20.7	106.72	0.0000	219.32	15221000	148	15.93	16.06	15.46	16.22	15.77	16.55	16.20	16.34
0.641	-0.30	0.81	P09405	Nucleolin	Ncl	695	36	36	36	42.1	42.1	42.1	76.722	0.0000	323.31	108680000	702	17.09	17.14	17.48	17.36	17.24	17.74	17.23	18.07
0.476	-0.30	0.81	P61222	ATP-binding cassette sub-family E member 1	Abce1	1379	37	37	37	64.8	64.8	64.8	67.314	0.0000	323.31	177700000	834	17.56	17.35	18.49	17.83	18.35	17.89	18.38	17.82
0.584	-0.30	0.81	Q3TDD9	Protein phosphatase 1 regulatory subunit 21	Ppp1r21	1831	41	41	41	67.1	67.1	67.1	88.337	0.0000	323.31	115580000	743	17.23	17.18	18.06	17.54	17.47	18.15	17.76	17.85
0.565	-0.30	0.81	P63037	DnaJ homolog subfamily A member 1	Dnaja1	1482	24	24	24	65	65	65	44.868	0.0000	260.39	74628000	485	17.16	17.53	16.91	17.32	17.05	17.85	17.31	17.94
0.343	-0.30	0.81	D0QMC3	Myeloid cell nuclear differentiation antigen-like protein	Mndal	207	21	10	5	39.8	18.6	12.6	60.526	0.0000	74.87	9416800	97	15.14	14.91	15.87	15.77	15.92	15.53	16.44	15.02
0.544	-0.31	0.81	Q9CQV7	Mitochondrial import inner membrane translocase subunit TIM14	Dnajc19	4640	10	10	10	78.4	78.4	78.4	12.436	0.0000	100.84	32884000	308	16.09	15.94	16.98	16.50	16.57	17.01	16.48	16.68
1.723	-0.31	0.81	Q3TCJ1	BRISC complex subunit Abro1	Fam175b	1827	17	17	17	48.9	48.9	48.9	46.942	0.0000	41.662	48612000	274	16.56	16.28	16.43	16.45	16.67	16.93	16.75	16.58
0.787	-0.31	0.81	P17918	Proliferating cell nuclear antigen	Pcna	832	20	20	20	80.5	80.5	80.5	28.785	0.0000	254.73	193580000	798	18.06	18.34	18.30	17.62	18.15	18.32	18.58	18.50
0.244	-0.31	0.81	Q9QYB5	Gamma-adducin	Add3	5567	9	9	9	19.3	19.3	19.3	78.776	0.0000	67.75	5857500	66	12.98	13.83	14.68	14.37	14.25	14.65	14.89	13.30
0.408	-0.31	0.81	A2AUY4		Baz2b	41	4	4	4	3.3	3.3	3.3	234	0.0000	21.261	2469400	41	13.69	14.45	13.47	13.39	13.97	14.36	14.45	13.45
0.517	-0.31	0.81	Q9D868	Peptidyl-prolyl cis-trans isomerase H	Ppih	5052	8	8	8	60.6	60.6	60.6	20.463	0.0000	60.608	16044000	160	16.08	15.54	15.03	15.42	15.34	15.94	16.09	15.93
0.405	-0.31	0.81	Q8BR65	Sin3 histone deacetylase corepressor complex component SDS3	Suds3	3176	4	4	4	12.2	12.2	12.2	38.107	0.0000	20.67	1448200	40	13.41	12.22	12.69	12.94	13.48	12.52	13.47	13.02
0.228	-0.31	0.81	Q6DVA0	LEM domain-containing protein 2	Lemd2	2512	7	7	7	20.5	20.5	20.5	57.506	0.0000	37.6	17640000	119	15.93	16.14	17.44	14.98	16.08	16.15	16.94	16.55
0.664	-0.31	0.81	Q9D0S9	Histidine triad nucleotide-binding protein 2, mitochondrial	Hint2	4886	6	6	6	47.9	47.9	47.9	17.32	0.0000	56.729	18991000	185	15.80	15.78	16.03	15.83	15.93	16.80	15.83	16.13
0.213	-0.31	0.81	Q02614	SAP30-binding protein	Sap30bp	1719	3	3	3	11	11	11	33.832	0.0010	4.0568	999610	3	13.03	11.20	12.97	13.24	11.93	13.03	13.35	13.38
0.319	-0.31	0.81	P03930	ATP synthase protein 8	Mtatp8	632	4	4	4	31.3	31.3	31.3	7.7662	0.0000	6.5461	30127000	84	17.13	16.89	18.10	18.60	17.71	18.03	18.16	18.07
0.431	-0.31	0.81	Q8BWW4	La-related protein 4	Larp4	3268	6	6	6	11.8	11.8	11.8	79.763	0.0000	29.883	4898100	54	13.93	14.02	14.26	14.44	14.25	15.01	13.73	14.90
0.942	-0.31	0.81	P62281	40S ribosomal protein S11	Rps11	1415	26	26	26	78.5	78.5	78.5	18.431	0.0000	139.02	575860000	1091	19.93	19.70	19.68	20.33	20.09	20.40	20.31	20.10
0.877	-0.31	0.80	Q8BP47	Asparagine--tRNA ligase, cytoplasmic	Nars	3158	33	33	33	54.9	54.9	54.9	64.279	0.0000	323.31	238080000	814	18.57	18.41	18.29	18.91	18.59	18.87	19.17	18.80
0.338	-0.31	0.80	P99027	60S acidic ribosomal protein P2	Rplp2	1693	7	7	7	79.1	79.1	79.1	11.651	0.0000	160.82	25281000	239	16.17	16.99	16.61	16.67	16.93	17.82	16.07	16.87
0.759	-0.31	0.80	Q80X85	28S ribosomal protein S7, mitochondrial	Mrps7	2875	11	11	10	50	50	47.1	28.062	0.0000	46.484	15422000	150	15.90	15.72	15.28	15.72	15.57	16.24	16.20	15.87
0.183	-0.32	0.80	Q9JJR8;Q9(	Transmembrane protein 9B;Transmembrane protein 9	Tmem9b;Tmem9	5433	4	4	4	25.6	25.6	25.6	22.607	0.0000	32.687	4583200	58	12.32	13.72	14.56	13.37	14.34	14.70	13.71	12.48
0.259	-0.32	0.80	Q5SVQ0	Histone acetyltransferase KAT7	Kat7	2151	4	4	4	7.2	7.2	7.2	70.641	0.0056	2.1789	5388400	15	15.37	13.45	15.44	15.33	15.27	15.36	15.39	14.83
0.269	-0.32	0.80	Q99LE1	RILP-like protein 2	Rilpl2	4423	14	14	13	78.2	78.2	78.2	22.393	0.0000	118.36	17370000	251	15.19	16.06	15.45	14.98	15.87	16.40	14.51	16.17
0.997	-0.32	0.80	Q09014	Neutrophil cytosol factor 1	Ncf1	1770	37	37	37	75.6	75.6	75.6	44.666	0.0000	323.31	278040000	1177	18.37	18.49	18.73	18.62	18.60	19.27	18.76	18.84
0.584	-0.32	0.80	O70551	SRSF protein kinase 1	Srpk1	512	7	5	5	12.8	10.2	10.2	73.088	0.0008	4.1129	1921400	24	13.79	13.79	13.66	13.83	13.36	14.50	14.34	14.14
0.313	-0.32	0.80	P62141	Serine/threonine-protein phosphatase PP1-beta catalytic subunit	Ppp1cb	1405	19	5	5	53.5	17.1	17.1	37.186	0.0000	36.361	25667000	155	16.80	16.75	16.17	15.55	17.02	15.98	16.23	17.30
0.711	-0.32	0.80	O88522	NF-kappa-B essential modulator	Ikbkg	539	4	4	4	13.8	13.8	13.8	47.972	0.0000	9.6973	7457500	18	15.13	15.55	15.60	15.98	15.58	16.20	15.78	15.96
0.975	-0.32	0.80	Q8BTI8	Serine/arginine repetitive matrix protein 2	Srrm2	3201	29	29	29	14.4	14.4	14.4	294.84	0.0000	188.2	136730000	620	18.11	17.96	17.73	18.43	18.14	18.49	18.37	18.50
0.509	-0.32	0.80	Q8C181	Muscleblind-like protein 2	Mbnl2	3359	15	5	5	38.3	16.9	16.9	40.156	0.0000	70.847	15544000	108	16.16	16.70	16.58	16.09	16.76	17.32	16.60	16.13
0.256	-0.32	0.80	P83741	Serine/threonine-protein kinase WNK1	Wnk1	1601	17	17	12	10.6	10.6	8	250.93	0.0000	27.525	9532700	94	14.45	14.51	16.29	14.05	14.93	15.45	15.00	15.18
1.279	-0.32	0.80	Q8K124	Pleckstrin homology domain-containing family O member 2	Plekho2	3613	33	33	33	63.4	63.4	63.4	53.872	0.0000	323.31	153680000	904	17.54	17.24	17.69	17.69	17.67	17.88	17.84	18.05
0.568	-0.32	0.80	Q07113	Cation-independent mannose-6-phosphate receptor	Igf2r	1755	17	17	17	8.3	8.3	8.3	273.81	0.0000	70.869	19213000	202	15.53	15.31	16.28	15.79	16.50	15.93	16.01	15.75
1.529	-0.32	0.80	Q8BJW6	Eukaryotic translation initiation factor 2A;Eukaryotic translation initiation	Eif2a	3089	30	30	30	64	64	64	64.403	0.0000	323.31	108980000	728	17.29	17.39	17.66	17.14	17.65	17.74	17.66	17.72
0.438	-0.32	0.80	Q6ZQB6	Inositol hexakisphosphate and diphosphoinositol-pentakisphosphate kin	Ppip5k2	2694	16	16	14	19.8	19.8	16.9	128.43	0.0000	123.18	16384000	231	15.08	15.04	16.37	15.25	15.56	15.86	15.62	15.99
1.343	-0.32	0.80	P62962	Profilin-1	Pfn1	1473	21	21	11	97.1	97.1	66.4	14.957	0.0000	323.31	3134700000	3656	22.50	22.31	21.90	22.32	22.58	22.60	22.61	22.53
1.692	-0.32	0.80	Q9JKP5	Muscleblind-like protein 1	Mbnl1	5456	20	20	7	52.5	52.5	25.2	36.975	0.0000	277.21	250970000	876	19.09	19.47	19.37	19.21	19.63	19.75	19.47	19.58
1.328	-0.32	0.80	P42567	Epidermal growth factor receptor substrate 15	Eps15	1080	13	13	13	24	24	24	98.47	0.0000	259.59	23019000	182	16.98	16.83	16.76	16.87	17.04	17.30	17.47	16.93
0.354	-0.32	0.80	P35278	Ras-related protein Rab-5C	Rab5c	1013	17	17	12	75	75	55.6	23.412	0.0000	259.78	245490000	726	18.99	18.78	17.42	18.94	18.99	18.54	18.80	19.10
0.777	-0.32	0.80	Q61735	Leukocyte surface antigen CD47	Cd47	2329	6	6	6	13.9	13.9	13.9	33.097	0.0000	133.3	39526000	131	17.61	17.09	17.20	17.87	17.80	17.61	18.03	17.63
0.474	-0.33	0.80	Q80YV2	Nuclear-interacting partner of ALK	Zc3hc1	2907	6	6	6	17.6	17.6	17.6	55.196	0.0000	14.903	6480000	74	15.16	14.61	13.74	14.81	14.68	15.00	15.03	14.91
0.248	-0.33	0.80	Q9JJ89	Coiled-coil domain-containing protein 86	Ccdc86	5420	4	4	4	9.4	9.4	9.4	46.486	0.0002	5.6567	1593700	6	11.59	13.14	13.26	14.13	13.58	13.17	13.30	13.38
0.336	-0.33	0.80	O35381	Acidic leucine-rich nuclear phosphoprotein 32 family member A	Anp32a	365	14	10	10	40.5	36.4	36.4	28.537	0.0000	42.021	14264000	127	15.26	15.71	15.00	16.10	15.05	15.72	15.94	16.68
0.442	-0.33	0.80	Q9CX86	Heterogeneous nuclear ribonucleoprotein A0	Hnrnpa0	4750	18	18	18	56.7	56.7	56.7	30.53	0.0000	90.438	56471000	349	17.11	16.98	16.10	17.32	16.98	17.73	16.85	17.26
0.589	-0.33	0.80	P52927	High mobility group protein HMGI-C	Hmga2	1228	4	4	4	41.7	41.7	41.7	11.819	0.0000	12.276	1436200	12	12.75	13.26	13.70	13.49	14.10	13.51	13.54	13.37
0.729	-0.33	0.80	Q99N96	39S ribosomal protein L1, mitochondrial	Mrpl1	4494	16	16	16	53	53	53	37.596	0.0000	275.6	30870000	392	15.54	15.37	15.65	15.35	15.43	16.38	15.82	15.59
0.205	-0.33	0.80	Q8C0T5	Signal-induced proliferation-associated 1-like protein 1	Sipa1l1	3348	4	4	3	3.8	3.8	3.1	197.03	0.0000	9.8859	1259500	34	12.16	11.93	14.01	12.78	11.97	14.04	13.27	12.93
0.531	-0.33	0.80	Q91W59	RNA-binding motif, single-stranded-interacting protein 1	Rbms1	4094	9	9	6	35.7	35.7	34	43.99	0.0000	93.467	14106000	156	14.99	15.67	14.90	15.71	16.00	15.91	15.20	15.46
1.342	-0.33	0.80	O35643	AP-1 complex subunit beta-1	Ap1b1	392	45	22	22	49	30.2	30.2	103.93	0.0000	323.31	81607000	403	17.65	17.59	17.51	17.64	17.57	18.10	18.11	17.93
0.282	-0.33	0.80	Q9QZ73	DCN1-like protein 1	Dcun1d1	5586	4	4	4	17.4	17.4	17.4	30.097	0.0000	33.289	11377000	73	15.88	15.99	14.20	16.24	15.69	15.67	16.22	16.05
0.247	-0.33	0.80	O35737	Heterogeneous nuclear ribonucleoprotein H;Heterogeneous nuclear ribo	Hnrnph1	403	23	11	11	61	35.6	35.6	49.199	0.0000	86.829	26312000	178	16.51	17.49	15.09	16.69	16.35	17.32	16.59	16.82
0.967	-0.33	0.80	Q8BMF4	Dihydrolipoyllysine-residue acetyltransferase component of pyruvate de	Dlat	3135	18	18	18	31.6	31.6	31.6	67.941	0.0000	142.01	87816000	401	17.96	18.17	17.71	17.75	18.14	18.26	18.59	17.93
0.825	-0.33	0.80	Q6A068	Cell division cycle 5-like protein	Cdc5l	2498	36	36	36	53.6	53.6	53.6	92.188	0.0000	255.25	58895000	519	16.31	16.71	16.99	16.28	16.66	17.18	16.89	16.89
0.505	-0.33	0.80	P70677	Caspase-3;Caspase-3 subunit p17;Caspase-3 subunit p12	Casp3	1583	9	9	9	40.1	40.1	40.1	31.474	0.0000	31.356	17693000	101	15.87	16.34	15.78	15.97	16.32	15.60	16.91	16.45
0.465	-0.33	0.80	Q9WV84	Nucleoside diphosphate kinase, mitochondrial	Nme4	5737	4	4	4	28	28	28	20.548	0.0000	6.9248	1967200	36	14.11	13.59	13.20	14.21	13.98	14.74	13.85	13.85
0.303	-0.33	0.80	Q920R0	Alsin	Als2	4214	5	5	5	5.5	5.5	5.5	182.56	0.0000	12.788	1163200	15	12.63	12.70	13.57	12.60	13.46	13.88	13.40	12.07
0.488	-0.33	0.79	Q6PIJ4	Nuclear factor related to kappa-B-binding protein	Nfrkb	2645	12	12	12	14.5	14.5	14.5	138.76	0.0000	50.446	7526700	85	14.71	14.74	15.46	14.23	14.65	15.49	15.19	15.14
0.565	-0.33	0.79	Q9WUA2	Phenylalanine--tRNA ligase beta subunit	Farsb	5702	32	32	32	60.1	60.1	60.1	65.696	0.0000	188.38	112990000	669	17.11	17.37	16.18	17.17	17.17	17.50	17.31	17.18
0.397	-0.33	0.79	Q8VBW1	Sodium- and chloride-dependent creatine transporter 1	Slc6a8	3899	3	3	3	5.9	5.9	5.9	70.999	0.0000	11.376	1433500	22	13.78	14.07	13.70	12.98	13.15	14.26	14.01	14.44
0.241	-0.33	0.79	P56371	Ras-related protein Rab-4A	Rab4a	1279	4	3	3	25.2	18.8	18.8	24.408	0.0000	25.87	2061100	22	13.66	12.98	13.59	13.72	12.96	13.00	15.21	14.12
0.318	-0.33	0.79	Q8BUV8	Protein GPR107	Gpr107	3231	3	3	3	5.3	5.3	5.3	62.055	0.0000	9.6631	1892600	28	13.80	13.32	12.31	14.09	13.84	13.50	14.25	13.26
0.188	-0.33	0.79	O35613	Death domain-associated protein 6	Daxx	387	4	4	4	7.6	7.6	7.6	81.488	0.0000	9.2296	1325000	13	14.26	13.17	14.78	11.99	13.35	14.77	13.97	13.45
0.156	-0.34	0.79	P08071	Lactotransferrin	Ltf	676	12	12	12	20.8	20.8	20.8	77.837	0.0000	58.549	19551000	85	16.25	15.37	17.72	17.52	18.23	17.78	16.68	15.51
0.363	-0.34	0.79	Q9R020	Zinc finger Ran-binding domain-containing protein 2	Zranb2	5611	3	3	3	10.3	10.3	10.3	37.35	0.0002	5.881	2273000	41	13.64	14.12	13.36	14.85	13.67	14.53	14.36	14.76
0.481	-0.34	0.79	P11881	Inositol 1,4,5-trisphosphate receptor type 1	Itpr1	751	5	5	5	2.7	2.7	2.7	313.16	0.0000	9.2333	2290500	25	13.51	12.51	12.28	13.03	12.99	13.03	13.64	13.01
1.323	-0.34	0.79	Q9CQD1	Ras-related protein Rab-5A	Rab5a	4586	12	7	6	67	47.4	44.2	23.598	0.0000	83.788	35613000	191	17.15	16.88	16.57	17.13	17.28	17.24	17.28	17.29
0.716	-0.34	0.79	Q3TIX9	U4/U6.U5 tri-snRNP-associated protein 2	Usp39	1852	11	11	11	28.9	28.9	28.9	65.146	0.0000	59.009	12036000	138	15.13	15.29	14.94	15.74	15.15	15.79	15.71	15.80
0.539	-0.34	0.79	Q9JIH2	Nuclear pore complex protein Nup50	Nup50	5400	11	11	11	30.3	30.3	30.3	49.485	0.0000	59.335	14205000	218	14.68	15.46	15.48	15.12	15.24	16.14	15.15	15.56
0.888	-0.34	0.79	P62717	60S ribosomal protein L18a	Rpl18a	1438	22	22	22	75	75	75	20.732	0.0000	132.18	204140000	770	19.15	19.11	18.97	19.47	19.45	19.85	19.09	19.67
0.859	-0.34	0.79	Q8K1A6	Coiled-coil and C2 domain-containing protein 1A	Cc2d1a	3621	12	12	12	17	17	17	103.7	0.0000	26.242	11687000	108	15.08	15.00	15.68	15.39	15.90	15.61	15.30	15.70
1.266	-0.34	0.79	P27659	60S ribosomal protein L3	Rpl3	938	45	45	45	76.2	76.2	76.2	46.109	0.0000	323.31	545920000	1621	19.21	19.11	19.50	19.59	19.53	19.92	19.61	19.73
0.309	-0.34	0.79	Q8BMC4	Nucleolar protein 9	Nop9	3133	12	12	12	23.3	23.3	23.3	70.046	0.0000	49.28	12870000	105	14.77	15.15	16.80	15.76	15.79	16.11	16.30	15.66
0.886	-0.34	0.79	O70305	Ataxin-2	Atxn2	484	5	5	5	6.5	6.5	6.5	136.48	0.0000	25.907	3957500	62	13.94	13.86	14.42	14.38	14.11	14.67	14.56	14.63
0.187	-0.34	0.79	Q80UU1	Ankyrin repeat and zinc finger domain-containing protein 1	Ankzf1	2837	6	6	6	12.4	12.4	12.4	82.975	0.0000	16.946	5013500	32	14.82	14.72	13.88	13.46	15.04	15.32	12.65	15.25
0.644	-0.34	0.79	O70274	Protein tyrosine phosphatase type IVA 2	Ptp4a2	481	7	7	5	38.3	38.3	34.1	19.127	0.0000	37.035	31137000	209	16.31	16.73	17.09	16.74	16.69	16.76	17.27	17.52
1.862	-0.34	0.79	Q05816	Fatty acid-binding protein, epidermal	Fabp5	1742	23	23	23	91.1	91.1	91.1	15.137	0.0000	323.31	734750000	1776	20.16	20.30	20.31	20.23	20.68	20.79	20.35	20.56
0.440	-0.35	0.79	Q9D8P4	39S ribosomal protein L17, mitochondrial	Mrpl17	5068	8	8	8	45.5	45.5	45.5	20.249	0.0000	13.162	15896000	127	15.16	15.13	16.17	15.58	15.41	16.21	15.43	16.37
0.249	-0.35	0.79	Q08857	Platelet glycoprotein 4	Cd36	1767	10	10	10	17.8	17.8	17.8	52.697	0.0000	69.66	28927000	153	17.34	17.56	16.12	16.37	18.35	17.32	16.86	16.24
0.736	-0.35	0.79	Q923D2	Flavin reductase (NADPH)	Blvrb	4282	19	19	19	85.9	85.9	85.9	22.197	0.0000	323.31	546370000	1335	19.64	20.16	19.52	19.67	19.90	20.62	19.81	20.05
0.124	-0.35	0.79	Q62293;Q3T9E4		Tgtp1	1811	9	9	9	28	28	28	47.12	0.0000	36.638	11499000	34	15.37	13.09	16.47	15.54	15.11	13.80	17.49	15.47
0.367	-0.35	0.79	P21550	Beta-enolase	Eno3	866	13	7	7	42.2	23.7	23.7	47.024	0.0000	12.572	6195000	71	15.10	14.65	13.39	14.87	14.49	14.93	15.20	14.78
0.900	-0.35	0.79	Q9DCC4	Pyrroline-5-carboxylate reductase 3	Pycrl	5204	11	11	11	48.9	48.9	48.9	28.721	0.0000	81.236	102370000	484	17.21	17.49	17.33	17.42	17.59	18.26	17.60	17.40
0.665	-0.35	0.79	P32020	Non-specific lipid-transfer protein	Scp2	993	23	23	22	32.5	32.5	32	59.125	0.0000	139.11	127940000	622	17.73	18.11	17.78	17.19	18.02	18.53	17.78	17.86
0.225	-0.35	0.78	Q5SFM8	RNA-binding protein 27	Rbm27	2130	10	10	9	10.6	10.6	10.6	118.55	0.0000	36.18	4127700	48	13.50	13.90	15.29	14.07	13.18	15.19	15.33	14.47
0.237	-0.35	0.78	Q8R043	Uncharacterized protein C6orf1 homolog		3746	2	2	2	19.6	19.6	19.6	11.553	0.0022	3.0483	1150600	23	13.65	13.09	13.07	12.27	13.52	13.30	14.63	12.04
1.277	-0.35	0.78	P70268	Serine/threonine-protein kinase N1	Pkn1	1542	32	32	31	42.6	42.6	41.8	104.41	0.0000	323.31	71043000	480	17.14	17.11	16.74	17.26	17.17	17.50	17.62	17.38
0.641	-0.35	0.78	Q9CR59	Growth arrest and DNA damage-inducible proteins-interacting protein 1	Gadd45gip1	4671	8	8	8	39.6	39.6	39.6	25.82	0.0000	47.463	24406000	145	15.73	16.77	15.85	16.34	16.36	16.88	16.44	16.43
0.239	-0.35	0.78	Q91XF0	Pyridoxine-5-phosphate oxidase	Pnpo	4146	4	4	4	20.7	20.7	20.7	30.114	0.0000	17.018	6120000	26	13.92	16.65	15.02	15.02	15.21	15.89	15.10	15.83
0.176	-0.35	0.78	P51859	Hepatoma-derived growth factor	Hdgf	1205	6	5	5	22.8	19.4	19.4	26.268	0.0000	18.572	8521100	80	15.25	16.56	12.83	14.70	15.45	15.12	14.90	15.29
1.459	-0.35	0.78	Q80ZV0	Ribonuclease H2 subunit B	Rnaseh2b	2922	11	11	11	38	38	38	34.729	0.0000	53.459	11144000	174	15.31	15.23	14.91	15.20	15.41	15.80	15.46	15.39
0.818	-0.36	0.78	O35685	Nuclear migration protein nudC	Nudc	398	12	12	12	34.9	34.9	34.9	38.358	0.0000	40.751	24228000	203	16.20	15.93	15.68	16.21	16.06	16.06	16.58	16.74
0.537	-0.36	0.78	Q91YJ5	Translation initiation factor IF-2, mitochondrial	Mtif2	4163	5	5	5	8.8	8.8	8.8	81.288	0.0000	10.819	4707900	32	14.80	15.38	14.62	14.61	15.55	15.38	15.44	14.47
0.243	-0.36	0.78	Q9Z0V7	Mitochondrial import inner membrane translocase subunit Tim17-B	Timm17b	5788	3	3	3	30.2	30.2	30.2	18.352	0.0000	20.052	5128400	47	14.44	14.90	14.74	14.64	15.65	15.74	15.48	13.28
1.089	-0.36	0.78	Q9WTP7	GTP:AMP phosphotransferase AK3, mitochondrial	Ak3	5682	17	17	17	74	74	74	25.426	0.0000	153.61	63149000	424	16.46	16.39	16.81	16.82	16.71	17.29	16.84	17.07
1.272	-0.36	0.78	Q8BYW1	Rho GTPase-activating protein 25	Arhgap25	3307	40	40	39	78.1	78.1	78.1	73.382	0.0000	323.31	87090000	671	17.19	17.00	17.34	16.94	17.37	17.78	17.23	17.52
0.208	-0.36	0.78	Q99M28	RNA-binding protein with serine-rich domain 1	Rnps1	4454	6	6	6	25.6	25.6	25.6	34.208	0.0000	37.781	21112000	120	16.39	16.01	17.11	15.33	16.46	17.36	14.99	17.47
0.949	-0.36	0.78	Q9DAZ9	Abscission/NoCut checkpoint regulator	Zfyve19	5115	6	6	6	21.3	21.3	21.3	43.272	0.0000	8.5019	3261300	42	14.26	14.08	13.56	13.98	14.19	14.70	14.24	14.18
0.681	-0.36	0.78	Q62189	U1 small nuclear ribonucleoprotein A	Snrpa	2365	10	10	9	35.9	35.9	35.9	31.835	0.0000	31.416	27630000	184	17.01	16.81	16.20	16.94	16.65	17.24	17.02	17.48
0.665	-0.36	0.78	Q6A0A2	La-related protein 4B	Larp4b	2500	7	7	7	13.9	13.9	13.9	81.626	0.0000	68.929	8605600	70	15.19	15.15	15.90	15.40	15.42	16.27	15.50	15.89
0.222	-0.36	0.78	P13808	Anion exchange protein 2	Slc4a2	769	8	8	8	10.3	10.3	10.3	136.81	0.0000	36.311	8780200	72	14.33	14.90	16.52	13.77	14.82	15.31	15.95	14.88
0.689	-0.36	0.78	P50171	Estradiol 17-beta-dehydrogenase 8	Hsd17b8	1176	9	9	9	53.3	53.3	53.3	26.588	0.0000	46.502	25226000	238	15.64	15.92	16.27	15.49	15.99	16.73	16.10	15.92
0.715	-0.36	0.78	Q8CI51	PDZ and LIM domain protein 5	Pdlim5	3540	26	26	26	49.7	49.7	49.7	63.299	0.0000	280.4	150810000	718	18.03	18.04	18.86	18.38	18.26	18.82	18.73	18.94
1.612	-0.36	0.78	Q99K28	ADP-ribosylation factor GTPase-activating protein 2	Arfgap2	4361	26	26	25	58.7	58.7	56.7	56.598	0.0000	254.77	65777000	490	16.50	16.65	16.90	16.66	16.80	17.12	17.01	17.22
0.541	-0.36	0.78	Q80UU9	Membrane-associated progesterone receptor component 2	Pgrmc2	2839	7	7	7	38.7	38.7	38.7	23.334	0.0000	68.638	13147000	114	14.85	16.09	15.38	15.60	15.32	16.12	15.98	15.96
0.229	-0.36	0.78	Q61151	Serine/threonine-protein phosphatase 2A 56 kDa regulatory subunit eps	Ppp2r5e	2260	10	10	8	27	27	20.8	54.713	0.0000	23.352	19560000	82	14.88	16.49	16.01	16.58	15.93	15.32	16.47	17.70
0.416	-0.36	0.78	Q9DB34	Charged multivesicular body protein 2a	Chmp2a	5124	18	18	18	50.5	50.5	50.5	25.134	0.0000	138.54	93218000	472	16.54	17.63	18.04	16.74	17.24	17.90	17.48	17.79

0.219	-0.37	0.78	Q9CYA6	Zinc finger CCHC domain-containing protein 8	Zcchc8	4793	5	5	5	11.3	11.3	11.3	78.025	0.0000	19.989	4999900	51	14.38	14.41	14.08	13.80	14.86	12.61	15.17	15.50
0.814	-0.37	0.77	Q7TMW6	Cytosolic Fe-S cluster assembly factor NARFL	Narfl	2751	12	12	12	45	45	45	53.107	0.0000	115.78	6996500	119	14.31	14.01	14.09	14.10	14.20	14.13	15.07	14.57
0.370	-0.37	0.77	Q8CD10	Calcium uptake protein 2, mitochondrial	Micu2	3454	7	7	7	22.2	22.2	22.2	49.475	0.0000	153.4	5137000	67	15.29	14.48	13.29	14.78	14.73	14.83	15.05	14.71
1.190	-0.37	0.77	P70460	Vasodilator-stimulated phosphoprotein	Vasp	1578	17	17	17	44.3	44.3	44.3	39.666	0.0000	192.42	114710000	529	17.72	17.86	17.28	17.75	17.76	18.05	18.02	18.26
0.401	-0.37	0.77	A2AB59	Rho GTPase-activating protein 27	Arhgap27	15	12	12	12	21.2	21.2	21.2	97.047	0.0000	70.654	9977300	77	15.06	14.78	13.74	15.32	14.67	15.15	15.64	14.91
0.736	-0.37	0.77	Q8K296	Myotubularin-related protein 3	Mtmr3	3650	9	9	9	13.7	13.7	13.7	133.84	0.0000	43.594	15033000	46	15.80	15.51	15.12	16.14	15.75	15.88	16.17	16.24
0.484	-0.37	0.77	Q61749	Translation initiation factor eIF-2B subunit delta	Eif2b4	2331	17	17	17	42.6	42.6	42.6	57.624	0.0000	97.038	23930000	241	15.42	15.61	16.69	15.15	15.86	16.19	16.14	16.16
0.674	-0.37	0.77	Q9D7N9	Adipocyte plasma membrane-associated protein	Apmap	5037	13	13	13	42.2	42.2	42.2	46.434	0.0000	191.83	37324000	325	16.58	16.55	15.87	15.56	16.49	16.32	16.72	16.50
1.137	-0.37	0.77	P47962	60S ribosomal protein L5	Rpl5	1134	35	35	35	67.7	67.7	67.7	34.4	0.0000	323.31	459640000	1555	18.88	18.65	19.35	19.12	19.20	19.45	19.30	19.53
0.310	-0.37	0.77	Q61830	Macrophage mannose receptor 1	Mrc1	2338	29	29	29	25.3	25.3	25.3	164.98	0.0000	151.27	29369000	245	15.70	14.91	16.77	16.20	17.00	15.77	16.57	15.73
1.250	-0.37	0.77	Q9JMH6	Thioredoxin reductase 1, cytoplasmic	Txnrd1	5511	49	49	47	65.9	65.9	63.5	67.083	0.0000	323.31	469310000	1451	19.74	19.78	19.75	19.71	20.59	19.93	19.98	19.98
0.560	-0.37	0.77	Q9JKK1	Syntaxin-6	Stx6	5453	5	5	5	27.5	27.5	27.5	28.996	0.0000	26.699	4424800	42	14.42	14.44	14.81	14.63	14.18	15.53	14.80	15.28
0.301	-0.37	0.77	Q3U821		Wdr75	1934	4	4	4	7.2	7.2	7.2	94.036	0.0000	53.898	3726400	42	14.22	13.91	14.41	12.21	13.95	13.89	14.48	13.94
0.126	-0.38	0.77	O54962	Barrier-to-autointegration factor;Barrier-to-autointegration factor, N-term	Banf1	437	5	5	5	46.1	46.1	46.1	10.102	0.0000	28.593	12686000	78	16.34	13.15	18.42	16.01	16.32	16.94	16.56	15.61
0.241	-0.38	0.77	P20934	Protein EVI2A	Evi2a	858	4	4	4	23.3	23.3	23.3	24.087	0.0000	31.459	4488100	44	13.18	13.18	15.20	14.04	14.18	14.99	14.80	13.14
1.640	-0.38	0.77	Q6ZWU9	40S ribosomal protein S27	Rps27	2706	6	6	3	41.7	41.7	15.5	9.461	0.0000	51.717	142890000	275	19.28	19.30	18.83	18.91	19.43	19.51	19.50	19.38
0.284	-0.38	0.77	Q91XB0	Three-prime repair exonuclease 1	Trex1	4139	13	13	13	52.2	52.2	52.2	33.675	0.0000	120.89	31379000	194	15.40	15.45	17.41	16.29	15.82	16.26	17.05	16.93
1.143	-0.38	0.77	Q9QZL0	Receptor-interacting serine/threonine-protein kinase 3	Ripk3	5600	15	15	15	39.3	39.3	39.3	53.322	0.0000	151.8	37977000	245	16.15	16.24	16.48	16.18	16.19	16.73	16.73	16.91
0.222	-0.38	0.77	Q8K0P3	TLD domain-containing protein 1	Tldc1	3608	2	2	2	7	7	7	50.844	0.0000	29.804	861570	25	12.49	12.74	15.03	12.45	12.95	14.15	13.94	13.19
1.309	-0.38	0.77	Q61768	Kinesin-1 heavy chain	Kif5b	2333	90	90	73	72.8	72.8	64.3	109.55	0.0000	323.31	337950000	2028	17.83	18.16	18.04	18.06	18.06	18.69	18.33	18.54
0.323	-0.38	0.77	Q63850	Nuclear pore glycoprotein p62	Nup62	2406	3	3	3	7	7	7	53.254	0.0000	7.3605	1759500	34	13.72	13.99	13.38	12.48	14.38	14.03	12.66	14.02
0.743	-0.38	0.77	Q9Z0H3	SWI/SNF-related matrix-associated actin-dependent regulator of chroma	Smarcb1	5770	12	12	12	43.4	43.4	43.4	44.141	0.0000	210.75	18032000	200	15.58	15.70	15.06	15.41	15.35	15.59	16.25	16.09
0.694	-0.38	0.77	Q5SSI6	U3 small nucleolar RNA-associated protein 18 homolog	Utp18	2137	13	13	13	33.9	33.9	33.9	61.217	0.0000	102.82	14407000	156	15.37	15.37	16.08	15.19	15.51	16.38	15.80	15.85
0.386	-0.38	0.77	Q91WZ8	Dysbindin	Dtnbp1	4131	4	4	4	11.9	11.9	11.9	39.651	0.0000	9.052	3384900	55	14.26	14.24	15.86	14.43	15.10	15.48	14.54	15.22
0.697	-0.39	0.77	Q08093	Calponin-2	Cnn2	1760	12	12	12	49.5	49.5	49.5	33.155	0.0000	51.134	24382000	133	16.36	15.76	16.59	16.99	16.83	16.73	16.67	17.02
1.137	-0.39	0.77	Q99KJ8	Dynactin subunit 2	Dctn2	4379	21	21	21	60.2	60.2	60.2	44.116	0.0000	181.17	55145000	444	16.17	16.52	16.10	16.38	16.43	16.96	16.41	16.91
0.212	-0.39	0.76	Q80ZS3	28S ribosomal protein S26, mitochondrial	Mrps26	2920	6	6	6	37	37	37	23.443	0.0000	25.695	6895800	71	14.80	13.63	16.07	14.52	14.06	16.57	15.01	14.91
0.772	-0.39	0.76	Q05D44	Eukaryotic translation initiation factor 5B	Eif5b	1747	43	43	43	48.8	48.8	48.8	137.61	0.0000	216.45	76055000	498	17.06	17.20	17.35	16.79	17.94	17.14	17.78	17.09
0.935	-0.39	0.76	P97820	Mitogen-activated protein kinase kinase kinase kinase 4	Map4k4	1676	16	7	6	15.7	8.9	8.4	140.6	0.0000	43.828	14089000	77	16.51	16.96	16.38	16.64	16.64	17.47	16.97	16.96
1.410	-0.39	0.76	Q6A026	Sister chromatid cohesion protein PDS5 homolog A	Pds5a	2495	33	33	33	37.5	37.5	37.5	150.33	0.0000	323.01	68400000	663	16.32	16.44	16.76	16.65	16.65	16.89	17.13	17.07
1.810	-0.39	0.76	Q8BHS6	Armadillo repeat-containing X-linked protein 3	Armcx3	3051	10	10	10	39.1	39.1	39.1	42.619	0.0000	131.73	15000000	146	16.30	16.07	16.04	16.24	16.40	16.78	16.38	16.65
0.918	-0.39	0.76	Q62433	Protein NDRG1	Ndrg1	2393	10	10	10	40.1	40.1	40.1	43.008	0.0000	93.979	31286000	154	16.87	17.04	16.78	17.21	17.01	17.36	17.19	17.91
0.299	-0.39	0.76	P10518	Delta-aminolevulinic acid dehydratase	Alad	722	9	9	9	49.7	49.7	49.7	36.023	0.0000	110.04	246280000	110	21.54	21.13	19.58	21.25	21.59	21.91	20.41	21.15
1.948	-0.39	0.76	Q505F5	Leucine-rich repeat-containing protein 47	Lrrc47	2082	27	27	27	48.5	48.5	48.5	63.589	0.0000	310.33	152370000	708	17.31	17.45	17.49	17.41	17.54	18.03	17.79	17.86
0.218	-0.39	0.76	P14869	60S acidic ribosomal protein P0	Rplp0	787	22	22	22	64.4	64.4	64.4	34.216	0.0000	323.31	375820000	1036	19.55	19.56	16.65	19.46	19.23	19.28	19.01	19.27
0.457	-0.39	0.76	Q9DB50	AP-1 complex subunit sigma-2	Ap1s2	5128	2	2	2	11.9	11.9	11.9	18.929	0.0013	3.5297	5687500	59	15.22	14.78	14.13	15.40	14.84	14.87	15.96	15.44
0.500	-0.39	0.76	Q9Z2C4	Myotubularin-related protein 1	Mtmr1	5839	4	4	4	8.8	8.8	8.8	75.313	0.0000	8.453	1097800	16	13.38	12.37	13.39	12.63	13.05	13.28	12.97	14.06
0.935	-0.40	0.76	O70496	H(+)/Cl(-) exchange transporter 7	Clcn7	509	15	15	15	24.4	24.4	24.4	88.712	0.0000	111.05	24490000	172	15.87	15.54	15.96	15.67	16.17	16.69	15.93	15.82
0.682	-0.40	0.76	Q9EQ61	Pescadillo homolog	Pes1	5272	10	10	10	17	17	17	67.795	0.0000	56.412	16081000	181	15.48	14.87	15.54	15.92	15.37	15.99	15.84	16.20
1.882	-0.40	0.76	O70492	Sorting nexin-3	Snx3	506	21	21	19	87.7	87.7	87.7	18.757	0.0000	250.88	260090000	549	19.63	19.87	19.74	19.49	20.12	20.16	19.85	20.19
0.744	-0.40	0.76	P04202	Transforming growth factor beta-1;Latency-associated peptide	Tgfb1	637	11	11	11	30	30	30	44.309	0.0000	41.037	13864000	130	14.95	15.14	15.08	15.14	15.04	16.20	15.21	15.46
0.160	-0.40	0.76	Q8BM85	TBC domain-containing protein kinase-like protein	Tbck	3129	8	8	8	18.5	18.5	18.5	86.37	0.0000	25.333	28024000	54	16.23	16.72	18.39	17.10	18.07	18.61	15.02	18.33
0.344	-0.40	0.76	Q9Z315	U4/U6.U5 tri-snRNP-associated protein 1	Sart1	5876	35	35	35	44.3	44.3	44.3	90.884	0.0000	235.16	28588000	243	15.41	16.25	16.33	15.72	17.37	15.49	16.76	15.68
0.602	-0.40	0.76	A6X919	Probable C-mannosyltransferase DPY19L1	Dpy19l1	65	5	5	5	10.9	10.9	10.9	84.186	0.0000	25.285	25299000	56	16.51	17.11	17.26	16.92	16.84	18.00	17.56	16.99
1.862	-0.40	0.76	Q8BI72	CDKN2A-interacting protein	Cdkn2aip	3054	14	14	14	33.6	33.6	33.6	59.744	0.0000	35.813	17811000	162	15.56	15.35	15.14	15.59	15.66	15.85	15.90	15.84
1.174	-0.40	0.76	Q9EST5	Acidic leucine-rich nuclear phosphoprotein 32 family member B	Anp32b	5345	15	15	11	32	32	25.4	31.078	0.0000	171.58	155240000	472	18.39	18.60	18.09	18.66	18.52	18.75	19.01	19.06
1.239	-0.40	0.76	P97868	E3 ubiquitin-protein ligase RBBP6	Rbbp6	1682	7	7	7	6.8	6.8	6.8	199.59	0.0000	24.638	4644400	79	14.04	13.99	14.38	14.37	14.65	14.20	14.83	14.70
0.399	-0.40	0.76	Q91WB4	Pleckstrin homology domain-containing family F member 2	Plekhf2	4102	7	7	7	52.6	52.6	52.6	27.754	0.0000	19.845	11794000	87	15.33	13.86	14.80	14.15	15.51	14.14	15.21	14.88
1.051	-0.40	0.76	Q61771	Kinesin-like protein KIF3B;Kinesin-like protein KIF3B, N-terminally proce	Kif3b	2334	29	29	24	42.8	42.8	36.5	85.287	0.0000	203.21	48075000	443	16.45	16.32	16.93	16.74	16.80	17.38	16.78	17.08
1.231	-0.40	0.76	Q6Y7W8	PERQ amino acid-rich with GYF domain-containing protein 2	Gigyf2	2672	16	16	16	17.7	17.7	17.7	149.19	0.0000	193.38	28645000	255	17.17	17.60	17.64	17.24	17.89	18.10	17.51	17.77
0.272	-0.40	0.76	P54751	CMP-N-acetylneuraminate-beta-galactosamide-alpha-2,3-sialyltransfera	St3gal1	1256	3	3	3	14.2	14.2	14.2	39.072	0.0034	2.5371	1903700	15	13.83	13.56	12.55	14.31	14.94	14.44	13.81	12.69
1.151	-0.40	0.76	P30204	Macrophage scavenger receptor types I and II	Msr1	974	11	11	11	31.4	31.4	31.4	50.169	0.0000	268.48	41070000	236	16.61	16.61	16.68	16.27	16.76	17.39	16.68	16.95
1.352	-0.41	0.76	Q8BV13	COP9 signalosome complex subunit 7b	Cops7b	3234	5	5	4	28	28	20.8	29.689	0.0000	83.065	8058200	124	14.98	14.88	14.61	15.15	15.08	15.57	15.17	15.42
0.450	-0.41	0.76	Q3UYC0	Protein phosphatase 1H	Ppm1h	2043	18	18	17	42.3	42.3	40.4	56.379	0.0000	56.925	22753000	187	15.80	15.78	14.17	15.67	15.65	15.89	15.90	15.60
0.969	-0.41	0.75	Q80WW9	DDRGK domain-containing protein 1	Ddrgk1	2867	9	9	9	44.8	44.8	44.8	35.977	0.0000	64.104	6706700	119	14.61	15.01	14.89	14.30	14.84	14.88	15.37	15.34
0.223	-0.41	0.75	P11438	Lysosome-associated membrane glycoprotein 1	Lamp1	743	7	7	7	12.3	12.3	12.3	43.865	0.0000	90.118	86716000	175	18.65	18.70	20.30	16.82	19.00	19.41	18.61	19.06
0.580	-0.41	0.75	Q91YE7	RNA-binding protein 5	Rbm5	4156	11	11	10	20.6	20.6	19.3	92.31	0.0000	67.676	6482500	102	14.22	14.71	15.41	14.18	14.77	15.34	14.74	15.29
0.837	-0.41	0.75	Q99MS7	EH domain-binding protein 1-like protein 1	Ehbp1l1	4476	21	21	21	18.6	18.6	18.6	184.83	0.0000	169.44	31198000	279	15.70	15.51	16.39	16.13	15.93	16.45	16.42	16.55
0.186	-0.41	0.75	Q9D8M4	60S ribosomal protein L7-like 1	Rpl7l1	5064	4	4	4	21.1	21.1	21.1	28.543	0.0000	12.566	4609900	68	13.69	13.97	14.27	14.23	15.30	14.73	15.77	11.98
0.126	-0.41	0.75	Q8K072	Receptor expression-enhancing protein 4	Reep4	3598	5	5	4	26.1	26.1	21	29.69	0.0000	7.3357	12427000	37	16.53	12.95	14.68	17.06	16.48	13.37	16.69	16.33
0.187	-0.41	0.75	Q91WK0	Leucine-rich repeat flightless-interacting protein 2	Lrrfip2	4114	13	13	12	32.8	32.8	29.9	47.147	0.0000	31.349	4491400	47	14.34	14.36	13.65	13.55	14.73	13.59	12.67	16.56
0.599	-0.41	0.75	P37913	DNA ligase 1	Lig1	1047	33	33	33	44	44	44	102.29	0.0000	152.32	63234000	649	15.48	15.79	16.31	16.12	15.66	16.18	16.86	16.66
0.697	-0.41	0.75	Q61739	Integrin alpha-6;Integrin alpha-6 heavy chain;Integrin alpha-6 light chain	Itga6	2330	9	9	9	12	12	12	122.16	0.0000	20.508	13071000	100	15.52	15.35	16.05	16.41	16.02	16.02	16.68	16.27
0.373	-0.42	0.75	Q8R2R9	AP-3 complex subunit mu-2	Ap3m2	3817	7	4	4	24.2	14.6	14.6	46.916	0.0000	12.978	2942500	39	13.82	14.69	14.09	12.61	14.31	14.10	14.73	13.74
0.674	-0.42	0.75	Q04447	Creatine kinase B-type	Ckb	1731	29	29	28	79.5	79.5	79.5	42.713	0.0000	323.31	605340000	1473	19.99	20.14	19.18	19.94	20.23	19.70	20.30	20.70
1.342	-0.42	0.75	P54103	DnaJ homolog subfamily C member 2;DnaJ homolog subfamily C meml	Dnajc2	1247	21	21	21	36.7	36.7	36.7	71.721	0.0000	44.107	14306000	132	14.90	14.95	15.37	15.18	15.81	15.31	15.31	15.63
1.186	-0.42	0.75	Q7TMK9	Heterogeneous nuclear ribonucleoprotein Q	Syncrip	2746	34	34	34	52.8	52.8	52.8	69.632	0.0000	323.31	151680000	827	17.81	17.93	17.86	17.97	17.92	18.67	18.09	18.55
0.258	-0.42	0.75	P40338	Von Hippel-Lindau disease tumor suppressor	Vhl	1062	4	4	4	21	21	21	20.769	0.0000	6.5018	3926800	32	12.44	13.91	14.30	14.20	13.91	14.57	12.84	15.19
0.864	-0.42	0.75	Q9CR88	28S ribosomal protein S14, mitochondrial	Mrps14	4681	7	7	7	35.9	35.9	35.9	14.92	0.0000	190.83	26785000	187	16.35	16.63	16.37	16.29	16.23	16.85	17.37	16.86
0.301	-0.42	0.75	P99026	Proteasome subunit beta type-4	Psmb4	1692	13	13	13	56.8	56.8	56.8	29.116	0.0000	323.31	207710000	520	19.12	18.93	16.64	18.72	18.96	18.53	18.86	18.73
0.632	-0.42	0.75	Q61029	Lamina-associated polypeptide 2, isoforms beta/delta/epsilon/gamma	Tmpo	2239	29	16	16	68.1	36.1	36.1	50.372	0.0000	165.53	131830000	446	17.33	17.61	18.68	17.76	17.98	18.16	18.34	18.58
1.107	-0.42	0.75	P42125	Enoyl-CoA delta isomerase 1, mitochondrial	Eci1	1072	12	12	12	35.3	35.3	35.3	32.25	0.0000	144.15	61385000	372	17.19	17.15	16.83	16.48	17.50	17.33	17.49	17.03
0.240	-0.42	0.75	Q8CDM1	ATPase family AAA domain-containing protein 2	Atad2	3462	9	9	9	14.1	14.1	14.1	117.94	0.0000	61.261	2953700	24	13.34	13.09	13.75	14.96	13.69	13.37	15.92	13.85
0.282	-0.42	0.75	Q9D2R0	Acetoacetyl-CoA synthetase	Aacs	4949	18	18	18	28.7	28.7	28.7	75.199	0.0000	125.19	50704000	334	16.57	16.52	14.86	17.32	16.26	16.08	17.55	17.09
1.979	-0.42	0.74	Q62422	Osteoclast-stimulating factor 1	Ostf1	2389	17	17	17	72.6	72.6	72.6	23.782	0.0000	257.33	104710000	487	17.48	17.92	17.42	17.72	18.10	18.09	17.99	18.06
0.756	-0.43	0.74	Q09XV5	Chromodomain-helicase-DNA-binding protein 8	Chd8	1773	8	8	7	6	6	5.6	290.84	0.0000	24.798	3860000	56	13.70	14.54	14.23	14.57	14.27	14.88	15.12	14.48
0.551	-0.43	0.74	Q8K3Z9	Nuclear envelope pore membrane protein POM 121	Pom121	3708	7	7	7	12.3	12.3	12.3	121.02	0.0000	48.023	4702900	80	14.13	14.67	15.69	14.79	14.85	15.14	15.49	15.50
0.448	-0.43	0.74	P98195	Probable phospholipid-transporting ATPase IIB	Atp9b	1689	10	10	10	11.8	11.8	11.8	129.02	0.0000	24.473	8462600	67	15.33	14.54	13.44	15.13	15.16	14.87	15.04	15.08
1.159	-0.43	0.74	P29341	Polyadenylate-binding protein 1	Pabpc1	965	43	43	43	67.1	67.1	67.1	70.67	0.0000	305.31	243420000	1003	17.68	17.96	18.24	17.99	18.09	18.55	18.19	18.75
0.711	-0.43	0.74	Q60749	KH domain-containing, RNA-binding, signal transduction-associated prc	Khdrbs1	2201	17	17	17	36.6	36.6	36.6	48.37	0.0000	104.41	57278000	346	16.93	17.33	17.92	17.66	17.34	18.19	17.81	18.21
1.815	-0.43	0.74	Q8VDD5	Myosin-9	Myh9	3948	254	254	216	80.8	80.8	73.5	226.37	0.0000	323.31	3082800000	9892	20.22	20.39	20.45	20.33	20.47	21.03	20.75	20.87
0.959	-0.43	0.74	Q9CQT2	RNA-binding protein 7	Rbm7	4633	8	8	8	47.2	47.2	47.2	30.148	0.0000	116.61	29793000	199	16.33	16.59	16.65	16.67	16.46	17.36	16.82	17.32
1.638	-0.43	0.74	Q9CQ56	Vesicle transport protein USE1	Use1	4563	7	7	7	28.1	28.1	28.1	30.593	0.0000	9.7614	6437600	52	14.78	14.48	14.66	14.48	14.76	15.29	15.18	14.90
0.667	-0.43	0.74	Q9Z0E6	Interferon-induced guanylate-binding protein 2	Gbp2	5765	24	24	23	59.4	59.4	56.2	66.739	0.0000	323.31	24421000	246	15.31	14.62	15.86	15.52	15.64	15.34	16.13	15.94
0.430	-0.43	0.74	Q8BLY7	Hermansky-Pudlak syndrome 6 protein homolog	Hps6	3125	9	9	9	19.8	19.8	19.8	87.34	0.0000	12.824	4480600	36	14.77	14.39	12.81	13.62	14.36	14.49	14.48	13.99
0.451	-0.43	0.74	O88845	A-kinase anchor protein 10, mitochondrial	Akap10	575	17	17	17	38.4	38.4	38.4	73.631	0.0000	99.141	41275000	313	16.67	16.51	15.02	16.67	16.53	17.12	16.60	16.36
1.243	-0.43	0.74	Q3TBT3	Stimulator of interferon genes protein	Tmem173	1820	11	11	11	31.7	31.7	31.7	42.829	0.0000	162.86	28186000	175	16.62	16.47	16.30	16.73	16.74	16.78	17.43	16.92
0.677	-0.44	0.74	Q68FF6	ARF GTPase-activating protein GIT1	Git1	2474	18	14	14	35.3	30.3	30.3	85.299	0.0000	96.015	11872000	138	15.63	15.23	15.65	14.53	15.86	16.08	15.38	15.46
0.435	-0.44	0.74	O35841	Apoptosis inhibitor 5	Api5	405	27	27	27	47.2	47.2	47.2	56.784	0.0000	323.31	93651000	640	16.80	16.98	17.28	17.41	16.84	16.80	18.35	18.22
0.494	-0.44	0.74	Q6GSS7;Q6	Histone H2A type 2-A;Histone H2A type 2-C	Hist2h2aa1;Hist2h	2515	19	13	4	77.7	66.2	28.5	14.095	0.0000	323.31	5547600000	1865	24.26	24.64	25.38	25.41	25.42	24.67	25.28	26.06
0.649	-0.44	0.74	Q8R3F5	Malonyl-CoA-acyl carrier protein transacylase, mitochondrial	Mcat	3844	6	6	6	27	27	27	41.928	0.0000	47.02	5236200	65	15.13	14.54	14.14	15.00	15.03	15.59	15.39	14.55
0.773	-0.44	0.74	Q8VCF0	Mitochondrial antiviral-signaling protein	Mavs	3918	10	10	10	32.8	32.8	32.8	53.398	0.0000	112.89	32455000	175	16.79	16.09	15.74	16.33	16.17	16.82	16.80	16.91
0.792	-0.44	0.74	P70349	Histidine triad nucleotide-binding protein 1	Hint1	1558	12	12	12	93.7	93.7	93.7	13.777	0.0000	220.69	106640000	236	18.91	19.26	18.71	18.99	19.24	20.14	19.14	19.09
0.468	-0.44	0.74	Q8VE22	28S ribosomal protein S23, mitochondrial	Mrps23	3984	10	10	10	62.1	62.1	62.1	20.348	0.0000	65.516	10628000	100	15.29	15.25	15.34	13.91	15.06	16.10	15.12	15.26
0.274	-0.44	0.74	Q8K2T4	Ubiquinol-cytochrome-c reductase complex assembly factor 3	Uqcc3	3672	3	3	3	36	36	36	9.5839	0.0009	3.7502	4715700	14	14.80	15.93	13.53	13.14	14.66	15.36	14.61	14.53
0.987	-0.44	0.74	Q61937	Nucleophosmin	Npm1	2343	17	17	16	59.9	59.9	51.7	32.56	0.0000	323.31	88123000	399	18.14	18.40	17.93	18.56	18.52	18.98	18.27	19.01
0.534	-0.44	0.74	Q9D1H6	NADH dehydrogenase [ubiquinone] 1 alpha subcomplex assembly facto	Ndufaf4	4914	3	3	3	22	22	22	20.082	0.0004	4.839	2226100	15	13.40	13.49	13.65	13.61	12.93	14.70	14.01	14.28
0.815	-0.44	0.74	Q9DB85	Ribosomal RNA-processing protein 8	Rrp8	5132	5	5	5	19.5	19.5	19.5	51.066	0.0000	19.385	3176400	39	14.25	13.88	14.60	14.10	14.67	15.26	14.40	14.26
1.166	-0.44	0.74	Q5RJG1	Nucleolar protein 10	Nol10	2126	6	6	6	13.1	13.1	13.1	80.076	0.0000	12.482	5331500	69	14.26	14.21	14.64	14.45	14.39	15.08	14.72	15.14
2.008	-0.44	0.74	Q61033	Lamina-associated polypeptide 2, isoforms alpha/zeta	Tmpo	2240	31	31	18	65.5	65.5	44.6	75.167	0.0000	323.31	134020000	714	18.05	18.24	18.43	18.37	18.52	18.88	18.63	18.83
1.766	-0.44	0.74	Q9ER00	Syntaxin-12	Stx12	5291	14	14	13	45.6	45.6	45.6	31.195	0.0000	283.75	69997000	407	17.02	16.91	17.30	17.15	17.25	17.66	17.73	17.53
0.296	-0.45	0.73	Q8K382	DENN domain-containing protein 1A	Dennd1a	3694	10	10	10	14.4	14.4	14.4	111.54	0.0000	31.938	7313100	45	13.05	13.65	15.36	14.49	14.03	14.20	15.69	14.40
0.584	-0.45	0.73	P49615	Cyclin-dependent-like kinase 5	Cdk5	1166	13	12	12	52.7	50	50	33.288	0.0000	42.999	19319000	161	16.29	16.36	14.92	15.90	16.13	16.60	16.51	16.02
0.764	-0.45	0.73	Q61462	Cytochrome b-245 light chain	Cyba	2301	2	2	2	19.8	19.8	19.8	20.748	0.0000	75.219	107530000	114	19.31	18.75	18.74	19.50	19.17	20.07	19.65	19.20
0.505	-0.45	0.73	Q80VD1	Protein FAM98B	Fam98b	2849	10	10	9	36.1	36.1	33.1	45.349	0.0000	107.17	20163000	191	15.71	15.76	16.24	15.36	16.33	17.12	15.37	16.02
0.652	-0.45	0.73	P20029	78 kDa glucose-regulated protein	Hspa5	851	68	66	66	69.5	69.5	69.5	72.421	0.0000	323.31	1749500000	2902	20.97	21.19	19.82	21.02	21.01	21.45	21.04	21.28
2.005	-0.45	0.73	P26369	Splicing factor U2AF 65 kDa subunit	U2af2	923	16	16	16	47.6	47.6	47.6	53.516	0.0000	260.47	78503000	398	17.97	18.19	17.67	18.09	18.45	18.48	18.30	18.46
1.455	-0.45	0.73	P61255	60S ribosomal protein L26	Rpl26	1381	22	22	21	77.2	77.2	72.4	17.258	0.0000	142.62	375250000	869	18.96	18.91	19.14	19.46	19.35	19.74	19.40	19.76

2.240	-0.45	0.73	Q9CQ10	Charged multivesicular body protein 3	Chmp3	4552	8	8	8	34.8	34.8	34.8	25.219	0.0000	20.34	21687000	137	16.02	16.11	15.73	15.75	16.32	16.46	16.24	16.38
0.728	-0.45	0.73	Q65Z40	Wings apart-like protein homolog	Wapal	2466	15	15	15	19.1	19.1	19.1	134.07	0.0000	35.873	15218000	100	16.05	16.15	15.90	15.52	15.87	16.98	16.63	15.94
1.392	-0.45	0.73	Q9EQ32	Phosphoinositide 3-kinase adapter protein 1	Pik3ap1	5271	16	16	16	24.4	24.4	24.4	90.927	0.0000	97.436	47811000	320	16.74	16.90	17.21	17.25	17.17	17.51	17.46	17.76
1.118	-0.45	0.73	Q3UE31	Uncharacterized protein KIAA0930 homolog		1952	12	12	12	39.4	39.4	39.4	45.96	0.0000	49.351	16963000	149	15.60	15.61	15.10	14.76	15.84	15.65	15.76	15.64
0.238	-0.45	0.73	Q64345	Interferon-induced protein with tetratricopeptide repeats 3	Ifit3	2435	32	32	32	70.7	70.7	70.7	47.222	0.0000	254.53	85299000	412	15.73	14.86	17.69	17.07	15.93	16.29	17.84	17.10
1.069	-0.45	0.73	Q3TZX8	Polynucleotide 5-hydroxyl-kinase NOL9	Nol9	1892	9	9	8	13.4	13.4	13.3	80.839	0.0000	15.783	12659000	127	15.64	14.84	15.14	15.62	15.53	15.77	15.71	16.04
0.948	-0.45	0.73	Q64311	Protein N-terminal asparagine amidohydrolase	Ntan1	2429	3	3	3	14.5	14.5	14.5	34.595	0.0000	15.372	4707400	58	14.49	14.40	13.56	14.56	14.56	14.86	14.78	14.62
0.625	-0.45	0.73	P21619	Lamin-B2	Lmnb2	867	59	50	49	80	72.8	70	67.317	0.0000	323.31	102620000	812	16.15	15.98	17.39	16.36	16.73	17.17	16.63	17.18
0.201	-0.45	0.73	Q80UW8	DNA-directed RNA polymerases I, II, and III subunit RPABC1	Polr2e	2840	4	4	4	16.2	16.2	16.2	24.57	0.0000	8.4697	5422700	55	12.35	15.22	16.57	15.08	15.07	15.36	15.05	15.56
1.001	-0.45	0.73	P10639	Thioredoxin	Txn	726	10	10	8	59	59	59	11.675	0.0000	55.891	122610000	429	17.58	18.22	17.94	18.03	18.03	18.90	18.17	18.48
1.190	-0.46	0.73	Q9Z1X4	Interleukin enhancer-binding factor 3	Ilf3	5825	22	22	22	27.6	27.6	27.6	96.02	0.0000	168.33	55039000	413	15.99	16.23	16.74	16.43	16.46	17.04	16.90	16.80
0.454	-0.46	0.73	A2AR02	Peptidyl-prolyl cis-trans isomerase G	Ppig	36	11	11	11	14.2	14.2	14.2	88.324	0.0000	32.054	14864000	169	14.89	15.78	16.78	15.47	16.20	16.28	15.61	16.65
0.188	-0.46	0.73	Q9CQS8	Protein transport protein Sec61 subunit beta	Sec61b	4630	3	3	3	37.5	37.5	37.5	9.9583	0.0000	11.734	28652000	125	17.17	17.38	19.74	15.11	17.74	17.94	17.49	18.07
1.056	-0.46	0.73	P61226	Ras-related protein Rap-2b	Rap2b	1380	11	11	6	66.7	66.7	43.7	20.504	0.0000	323.31	64622000	288	17.41	18.03	17.40	17.95	17.73	18.41	18.28	18.20
0.782	-0.46	0.73	Q8BH69	Selenide, water dikinase 1	Sephs1	3019	11	11	11	46.2	46.2	46.2	42.906	0.0000	217.69	41540000	220	17.54	18.08	17.10	17.16	18.20	17.65	18.30	17.58
0.807	-0.46	0.73	Q9CXY6	Interleukin enhancer-binding factor 2	Ilf2	4774	16	16	16	49.5	49.5	49.5	43.062	0.0000	107.94	43560000	283	17.19	17.41	16.52	17.35	17.48	18.14	17.48	17.21
1.106	-0.46	0.73	G5E870	E3 ubiquitin-protein ligase TRIP12	Trip12	263	26	26	26	20.9	20.9	20.9	224.13	0.0000	323.31	39501000	462	15.82	15.62	16.43	16.23	16.36	16.75	16.59	16.24
2.189	-0.46	0.73	Q9JK48	Endophilin-B1	Sh3glb1	5444	24	24	22	55.9	55.9	55.9	40.855	0.0000	323.31	96090000	484	17.29	17.23	16.91	17.14	17.52	17.78	17.44	17.69
1.165	-0.46	0.73	P67984	60S ribosomal protein L22	Rpl22	1510	9	9	8	68.8	68.8	62.5	14.759	0.0000	323.31	356070000	506	20.04	20.46	19.71	19.97	20.39	20.79	20.17	20.67
0.458	-0.46	0.73	Q8R310	Transmembrane and coiled-coil domains protein 3	Tmcc3	3828	5	5	5	14	14	14	53.721	0.0000	9.7722	2756200	30	13.23	13.60	13.96	14.28	13.61	14.23	15.34	13.73
0.873	-0.46	0.73	P06537	Glucocorticoid receptor	Nr3c1	659	11	11	11	22.3	22.3	22.3	86.052	0.0000	56.241	11883000	145	15.20	15.52	15.63	15.11	15.15	16.27	15.93	15.96
0.714	-0.47	0.72	P58742	Aladin	Aaas	1321	4	4	4	10.1	10.1	10.1	59.43	0.0000	39.849	4531200	66	14.15	14.67	13.62	13.41	14.25	14.81	14.48	14.16
2.115	-0.47	0.72	Q8K1E6	Alpha-ketoglutarate-dependent dioxygenase alkB homolog 3	Alkbh3	3626	7	7	7	32.9	32.9	32.9	33.061	0.0000	16.713	6228900	69	14.33	14.46	14.50	14.51	14.97	15.05	14.59	15.06
0.708	-0.47	0.72	Q9Z199;P63	Transcription elongation factor SPT4-B;Transcription elongation factor S	Supt4h1b;Supt4h1	1500	3	3	3	22.2	22.2	22.2	13.194	0.0009	3.7542	6913100	46	14.91	15.92	15.75	14.93	15.70	16.30	15.47	15.91
0.793	-0.47	0.72	Q6PGL7	WASH complex subunit FAM21	Fam21	2639	23	23	23	36.1	36.1	36.1	145.31	0.0000	158.92	14115000	139	15.12	15.17	16.28	15.42	15.67	16.08	15.91	16.18
1.572	-0.47	0.72	O08553	Dihydropyrimidinase-related protein 2	Dpysl2	272	47	47	42	80.6	80.6	78.5	62.277	0.0000	323.31	903250000	1997	19.50	19.71	19.39	19.65	19.71	19.89	20.34	20.19
0.847	-0.47	0.72	Q9DCX2	ATP synthase subunit d, mitochondrial	Atp5h	5236	19	19	19	91.3	91.3	91.3	18.749	0.0000	260.91	197080000	742	19.00	19.61	18.67	18.90	19.86	19.55	18.98	19.66
0.371	-0.47	0.72	Q9CQX8	28S ribosomal protein S36, mitochondrial	Mrps36	4646	5	5	5	70.6	70.6	70.6	11.101	0.0000	216.52	22715000	138	15.66	16.49	18.23	16.69	16.93	17.36	17.22	17.42
1.059	-0.47	0.72	P57759	Endoplasmic reticulum resident protein 29	Erp29	1298	21	21	21	63.7	63.7	63.7	28.823	0.0000	300.32	223410000	756	18.65	19.42	18.92	18.84	19.39	19.59	19.00	19.74
0.819	-0.47	0.72	Q810V0	U3 small nucleolar ribonucleoprotein protein MPP10	Mphosph10	2933	5	5	5	11.5	11.5	11.5	78.734	0.0000	18.622	11763000	22	15.98	16.17	16.58	16.12	16.92	16.20	17.29	16.32
1.099	-0.47	0.72	Q05CL8	La-related protein 7	Larp7	1746	22	22	22	44.9	44.9	44.9	64.802	0.0000	56.633	23866000	182	15.92	15.92	16.82	16.24	16.52	16.63	16.82	16.80
0.345	-0.47	0.72	Q3UH06	Ras-responsive element-binding protein 1	Rreb1	1965	7	7	7	8.3	8.3	8.3	184.15	0.0000	14.317	1751200	31	13.12	12.84	13.91	12.93	13.80	14.25	14.51	12.13
2.056	-0.47	0.72	Q921H8	3-ketoacyl-CoA thiolase A, peroxisomal	Acaa1a	4225	28	28	28	75.7	75.7	75.7	43.953	0.0000	323.31	628120000	1537	18.86	19.19	19.08	18.93	19.21	19.67	19.54	19.52
0.340	-0.47	0.72	Q8R2L5	28S ribosomal protein S18c, mitochondrial	Mrps18c	3809	3	3	3	35	35	35	16.275	0.0000	16.485	3900300	39	14.41	14.40	12.24	14.29	14.41	13.75	15.01	14.06
1.597	-0.47	0.72	P34022	Ran-specific GTPase-activating protein	Ranbp1	1005	11	11	11	52.2	52.2	52.2	23.596	0.0000	71.201	35928000	289	16.54	16.59	16.59	17.14	17.05	17.35	17.07	17.27
0.328	-0.47	0.72	Q8BS95	Golgi pH regulator	Gpr89a	3185	3	3	3	12.1	12.1	12.1	52.734	0.0000	102.02	5605100	51	12.80	14.02	13.75	13.05	14.38	12.48	15.01	13.64
0.750	-0.47	0.72	Q9BCZ4	Selenoprotein S	Vimp	4521	8	8	7	49.5	49.5	38.9	21.509	0.0000	83.492	12443000	108	14.92	14.83	15.37	15.66	15.90	14.97	16.04	15.75
0.619	-0.47	0.72	Q61578	NADPH:adrenodoxin oxidoreductase, mitochondrial	Fdxr	2314	11	11	11	35	35	35	54.201	0.0000	66.771	14940000	129	15.66	15.75	14.38	15.69	15.72	15.85	16.26	15.54
1.011	-0.47	0.72	Q8K1R3	Polyribonucleotide nucleotidyltransferase 1, mitochondrial	Pnpt1	3632	31	31	31	45.1	45.1	45.1	85.682	0.0000	102.41	46593000	273	16.12	15.98	16.48	16.25	16.54	17.30	16.28	16.62
0.244	-0.48	0.72	O88696	ATP-dependent Clp protease proteolytic subunit, mitochondrial	Clpp	559	10	10	10	38.2	38.2	38.2	29.8	0.0000	108.04	29706000	175	16.26	16.43	13.20	16.32	16.02	16.29	15.79	16.02
0.487	-0.48	0.72	Q9WUU7	Cathepsin Z	Ctsz	5723	6	6	6	17.6	17.6	17.6	33.996	0.0000	28.671	76798000	198	18.78	19.29	17.32	18.82	19.14	19.26	18.70	19.01
1.025	-0.48	0.72	P97821	Dipeptidyl peptidase 1;Dipeptidyl peptidase 1 exclusion domain chain;D	Ctsc	1677	8	8	8	16.7	16.7	16.7	52.376	0.0000	155.59	47981000	235	16.90	17.00	17.46	17.10	17.10	18.10	17.56	17.61
0.687	-0.48	0.72	Q8BQM4	HEAT repeat-containing protein 3	Heatr3	3171	6	6	6	11.8	11.8	11.8	74.305	0.0000	15.506	6911300	65	14.59	14.83	13.93	15.32	14.67	15.10	15.44	15.37
0.654	-0.48	0.72	Q3UQ84	Threonine--tRNA ligase, mitochondrial	Tars2	2017	10	10	10	16.3	16.3	16.3	81.699	0.0000	14.635	7084000	60	14.64	13.79	13.97	13.54	14.36	13.80	15.03	14.65
1.453	-0.48	0.72	O88746	Target of Myb protein 1	Tom1	565	18	18	18	60	60	60	54.325	0.0000	323.31	39894000	383	16.13	16.38	16.52	16.32	16.69	17.25	16.81	16.51
0.603	-0.48	0.72	Q8CDN6	Thioredoxin-like protein 1	Txnl1	3464	11	11	11	63	63	63	32.237	0.0000	153.21	32609000	204	16.16	16.84	16.08	15.06	16.31	16.63	16.52	16.59
1.153	-0.48	0.72	P62814	V-type proton ATPase subunit B, brain isoform	Atp6v1b2	1446	50	50	50	92.4	92.4	92.4	56.55	0.0000	323.31	1598500000	2916	20.69	20.58	19.80	20.62	20.98	21.03	20.77	20.82
0.800	-0.48	0.72	P56960	Exosome component 10	Exosc10	1294	16	16	16	24.6	24.6	24.6	100.94	0.0000	53.802	14827000	158	15.05	15.04	15.50	15.69	15.42	16.23	15.32	16.22
0.418	-0.48	0.72	P23249	Putative helicase MOV-10	Mov10	881	13	13	13	17.3	17.3	17.3	113.58	0.0000	29.226	8321600	75	14.44	14.28	13.49	14.39	14.58	14.95	15.59	13.40
1.029	-0.48	0.72	Q9CQ92	Mitochondrial fission 1 protein	Fis1	4576	11	11	11	53.3	53.3	53.3	17.008	0.0000	210.3	138050000	417	18.79	19.30	19.06	18.69	19.05	19.86	19.16	19.70
0.411	-0.48	0.72	Q8VE18	Protein SMG8	Smg8	3982	6	6	6	11.5	11.5	11.5	109.67	0.0000	68.774	4682400	84	12.84	13.74	15.07	13.81	14.21	14.99	14.37	13.82
2.782	-0.48	0.72	P26039	Talin-1	Tln1	914	230	230	230	83.2	83.2	83.2	269.82	0.0000	323.31	4504900000	10924	20.43	20.51	20.64	20.41	20.78	21.10	21.08	20.96
1.428	-0.48	0.72	P61089	Ubiquitin-conjugating enzyme E2 N	Ube2n	1371	14	14	14	79.6	79.6	79.6	17.138	0.0000	160.63	120760000	445	18.23	18.37	18.18	18.52	18.38	19.02	18.72	19.11
0.599	-0.48	0.71	Q8BKX1	Brain-specific angiogenesis inhibitor 1-associated protein 2	Baiap2	3107	11	11	11	35	35	35	59.236	0.0000	89.507	7303400	70	14.50	13.76	13.68	14.37	14.13	15.34	14.82	13.95
1.062	-0.48	0.71	P70195	Proteasome subunit beta type-7	Psmb7	1533	8	8	8	23.8	23.8	23.8	29.891	0.0000	226.12	117190000	412	19.09	18.99	18.46	19.15	19.18	19.93	19.34	19.19
1.384	-0.49	0.71	P58058	NAD kinase	Nadk	1305	16	16	16	28	28	28	48.597	0.0000	74.454	43506000	279	16.41	16.63	16.54	17.16	17.01	17.06	17.42	17.18
0.452	-0.49	0.71	O35239	Tyrosine-protein phosphatase non-receptor type 9	Ptpn9	350	6	6	6	13.3	13.3	13.3	67.969	0.0000	15.443	6124000	57	15.09	13.32	13.28	14.62	14.65	14.18	14.50	14.92
1.205	-0.49	0.71	Q60866	Phosphotriesterase-related protein	Pter	2216	6	6	6	25.5	25.5	25.5	39.218	0.0000	63.439	7926500	87	15.42	15.27	15.44	15.02	15.62	16.34	15.51	15.62
0.951	-0.49	0.71	P35293	Ras-related protein Rab-18	Rab18	1020	19	19	19	85	85	85	23.035	0.0000	173.38	72698000	418	17.19	17.14	16.41	16.42	17.16	17.61	17.42	16.94
1.536	-0.49	0.71	Q60875	Rho guanine nucleotide exchange factor 2	Arhgef2	2219	37	37	37	45.3	45.3	45.3	111.97	0.0000	310.11	113950000	661	18.05	17.80	18.36	18.06	18.34	18.88	18.62	18.37
0.292	-0.49	0.71	P62748;P84	Hippocalcin-like protein 1;Neuron-specific calcium-binding protein hippo	Hpcal1;Hpca	1441	8	8	8	42.5	42.5	42.5	22.338	0.0000	18.691	6926200	68	15.50	16.03	13.18	15.77	15.71	16.13	14.98	15.60
0.593	-0.49	0.71	Q9CRC8	Leucine-rich repeat-containing protein 40	Lrrc40	4690	22	22	22	50.8	50.8	50.8	68.075	0.0000	86.102	29685000	340	14.92	15.48	16.41	15.68	15.52	15.94	16.49	16.50
1.147	-0.49	0.71	Q9D6T0	Nitric oxide synthase-interacting protein	Nosip	5009	7	7	7	33.2	33.2	33.2	33.209	0.0000	41.716	7024900	69	15.25	15.47	14.87	14.96	15.98	15.58	15.79	15.16
1.294	-0.49	0.71	P62849	40S ribosomal protein S24	Rps24	1453	13	13	13	52.6	52.6	52.6	15.423	0.0000	203.15	248060000	520	19.59	19.75	19.00	19.64	20.14	19.67	19.98	20.15
0.386	-0.49	0.71	O09000	Nuclear receptor coactivator 3	Ncoa3	315	6	6	6	6.8	6.8	6.8	151.57	0.0000	35.541	4911100	76	14.91	14.57	12.66	15.04	14.74	14.81	14.92	14.67
0.344	-0.49	0.71	Q7TND5	Ribosome production factor 1	Rpf1	2760	2	2	2	11.5	11.5	11.5	40.036	0.0000	11.602	860570	26	13.29	12.67	11.15	12.93	12.36	14.11	12.61	12.93
1.015	-0.49	0.71	O54988	STE20-like serine/threonine-protein kinase	Slk	440	53	53	50	44.6	44.6	43	141.46	0.0000	323.31	110100000	1208	15.84	16.70	16.59	16.05	16.53	16.99	16.57	17.05
1.076	-0.49	0.71	Q8VIJ6	Splicing factor, proline- and glutamine-rich	Sfpq	4034	47	47	45	51.5	51.5	50.1	75.441	0.0000	323.31	242630000	992	17.80	18.10	18.53	18.25	18.22	18.86	18.53	19.04
0.643	-0.49	0.71	Q9CQF8	Ribosomal protein 63, mitochondrial	Mrpl57	4596	5	5	5	31.4	31.4	31.4	11.952	0.0000	12.393	15959000	134	15.05	15.73	15.47	15.40	16.04	14.91	16.29	16.37
0.225	-0.49	0.71	Q7TSH6		Scaf4	2784	6	6	5	7.9	7.9	7.4	129.05	0.0000	10.663	5878600	40	14.79	13.86	15.08	15.44	14.97	13.26	15.75	17.16
0.793	-0.49	0.71	Q99N89	39S ribosomal protein L43, mitochondrial	Mrpl43	4488	7	7	7	42.6	42.6	42.6	20.203	0.0000	25.236	17770000	106	15.57	15.59	15.73	15.39	15.62	16.94	15.74	15.96
2.750	-0.50	0.71	Q6ZWN5	40S ribosomal protein S9	Rps9	2703	25	25	25	66.5	66.5	66.5	22.591	0.0000	110.19	384080000	854	19.37	19.27	19.32	19.59	19.93	19.81	19.76	20.03
0.907	-0.50	0.71	Q6P9Q4	FH1/FH2 domain-containing protein 1	Fhod1	2589	22	22	22	28.2	28.2	28.2	129.6	0.0000	323.31	41198000	409	15.90	16.18	16.65	15.71	16.06	16.86	16.86	16.64
0.368	-0.50	0.71	Q9Z2V6	Histone deacetylase 5	Hdac5	5867	6	5	5	12.4	11.8	11.8	120.94	0.0000	28.63	2926500	37	14.14	13.40	13.33	14.74	14.38	13.05	15.11	15.05
0.776	-0.50	0.71	Q80SZ7	Guanine nucleotide-binding protein G(I)/G(S)/G(O) subunit gamma-5	Gng5	2798	5	5	5	51.5	51.5	51.5	7.3184	0.0000	9.9109	29548000	117	17.11	17.14	16.18	17.55	17.16	17.51	17.52	17.79
1.688	-0.50	0.71	Q9Z2M7	Phosphomannomutase 2	Pmm2	5859	15	15	15	50	50	50	27.656	0.0000	166.2	83662000	351	17.92	17.87	17.63	18.07	18.05	18.69	18.37	18.36
1.336	-0.50	0.71	P13864	DNA (cytosine-5)-methyltransferase 1	Dnmt1	770	36	36	36	30.2	30.2	30.2	183.19	0.0000	126.28	37291000	395	16.11	16.34	15.54	16.32	16.58	16.47	16.76	16.48
1.233	-0.50	0.71	O35226	26S proteasome non-ATPase regulatory subunit 4	Psmd4	348	10	10	10	40.7	40.7	40.7	40.703	0.0000	135.92	49883000	391	16.53	16.57	15.86	16.37	16.43	17.04	17.01	16.85
1.093	-0.50	0.71	Q921G7	Electron transfer flavoprotein-ubiquinone oxidoreductase, mitochondrial	Etfdh	4223	24	24	24	48.1	48.1	48.1	68.09	0.0000	61.45	36032000	258	16.06	16.17	17.06	16.12	16.81	16.88	16.81	16.88
1.053	-0.50	0.71	Q8VH51	RNA-binding protein 39	Rbm39	4016	20	20	20	37.9	37.9	37.9	59.406	0.0000	280.68	79249000	435	17.10	17.48	17.94	16.94	17.59	18.05	17.99	17.82
0.161	-0.50	0.71	P58059	28S ribosomal protein S21, mitochondrial	Mrps21	1306	4	4	4	49.4	49.4	49.4	10.561	0.0000	18.747	10140000	48	15.69	15.55	14.64	13.39	12.68	17.17	16.91	14.50
0.671	-0.50	0.71	P28650	Adenylosuccinate synthetase isozyme 1	Adssl1	953	29	26	26	69.1	62.8	62.8	50.254	0.0000	237.82	125790000	537	18.06	17.97	16.58	17.88	18.07	18.25	18.23	17.92
2.072	-0.50	0.71	Q05144	Ras-related C3 botulinum toxin substrate 2	Rac2	1739	19	18	9	63.5	63.5	32.3	21.441	0.0000	87.717	539890000	761	19.57	19.43	19.08	19.63	19.85	19.94	20.04	19.87
1.081	-0.50	0.71	P62315	Small nuclear ribonucleoprotein Sm D1	Snrpd1	1422	4	4	4	54.6	54.6	54.6	13.281	0.0000	70.875	148440000	199	20.48	20.38	19.94	20.12	21.06	20.18	20.64	21.03
0.978	-0.50	0.71	P63038	60 kDa heat shock protein, mitochondrial	Hspd1	1483	71	71	71	90.2	90.2	90.2	60.955	0.0000	323.31	1748600000	4305	20.82	21.34	20.34	20.53	21.10	21.63	20.96	21.33
1.447	-0.50	0.71	Q00PI9	Heterogeneous nuclear ribonucleoprotein U-like protein 2	Hnrnpul2	1703	24	24	24	39.1	39.1	39.1	84.939	0.0000	230.24	84807000	485	17.00	17.09	17.73	17.41	17.62	18.03	17.78	17.80
0.753	-0.50	0.71	Q9R060	Cytosolic Fe-S cluster assembly factor NUBP1	Nubp1	5615	5	5	5	24.7	24.7	24.7	34.085	0.0000	33.373	7161600	74	13.91	14.68	14.88	13.75	14.40	14.66	14.98	15.20
0.570	-0.51	0.70	Q9D6W8	Uncharacterized protein C17orf59 homolog		5011	4	4	4	18.9	18.9	18.9	38.011	0.0000	35.209	3521800	37	14.65	13.39	13.22	13.44	13.53	14.59	14.04	14.57
0.957	-0.51	0.70	Q99M51	Cytoplasmic protein NCK1	Nck1	4456	14	14	13	43.2	43.2	40.8	42.89	0.0000	34.524	11731000	99	14.81	15.25	14.13	15.03	15.31	15.33	15.01	15.60
1.320	-0.51	0.70	Q9D1P4	Cysteine and histidine-rich domain-containing protein 1	Chordc1	4930	11	11	11	41.4	41.4	41.4	37.35	0.0000	81.619	25968000	212	15.78	16.29	16.24	15.74	16.40	16.93	16.29	16.44
0.672	-0.51	0.70	Q8K4L3	Supervillin	Svil	3721	37	37	37	23.2	23.2	23.2	243.16	0.0000	216.17	29968000	339	14.75	15.29	16.36	15.62	15.61	16.24	16.16	16.04
0.509	-0.51	0.70	Q80SY3	V-type proton ATPase subunit d 2	Atp6v0d2	2796	10	10	10	42.3	42.3	42.3	40.493	0.0000	89.893	18667000	115	16.99	16.90	16.87	16.08	17.81	17.97	16.82	16.27
1.748	-0.51	0.70	Q9WVK4	EH domain-containing protein 1	Ehd1	5752	46	46	33	83.9	83.9	62.5	60.602	0.0000	323.31	346960000	1232	18.49	18.43	18.70	18.83	18.74	19.20	19.26	19.28
2.627	-0.51	0.70	Q99KI0	Aconitate hydratase, mitochondrial	Aco2	4377	52	52	52	62.8	62.8	62.8	85.462	0.0000	323.31	631830000	2050	19.21	19.04	19.01	19.21	19.60	19.76	19.75	19.40
0.378	-0.51	0.70	Q6P8I4	PEST proteolytic signal-containing nuclear protein	Pcnp	2583	7	7	7	45.5	45.5	45.5	18.963	0.0000	21.946	9546900	90	14.72	15.72	15.59	15.55	14.46	16.61	15.71	16.83
0.931	-0.51	0.70	O88532	Zinc finger RNA-binding protein	Zfr	541	16	16	16	21.9	21.9	21.9	116.86	0.0000	225.83	17268000	299	14.33	14.37	15.19	14.91	14.67	15.46	15.30	15.42
1.050	-0.51	0.70	Q8R361	Rab11 family-interacting protein 5	Rab11fip5	3837	23	23	23	49.1	49.1	49.1	69.552	0.0000	169.28	46578000	380	16.14	16.28	17.16	16.47	16.70	17.20	17.03	17.17
0.433	-0.51	0.70	Q8CAK1	Putative transferase CAF17 homolog, mitochondrial	Iba57	3429	6	6	6	19.8	19.8	19.8	38.399	0.0000	22.326	4293800	46	15.04	14.84	14.54	12.97	14.81	15.49	14.78	14.35
2.441	-0.51	0.70	P97461	40S ribosomal protein S5;40S ribosomal protein S5, N-terminally proces	Rps5	1653	19	19	19	54.4	54.4	54.4	22.889	0.0000	323.31	577400000	762	20.31	20.26	20.64	20.50	20.79	20.97	20.89	21.11
0.141	-0.51	0.70	Q8BHD0	Ras-related protein Rab-39A	Rab39a	3032	3	1	1	6.9	6	6	24.977	0.0017	3.2824	15053000	44	17.38	17.06	12.57	15.09	18.01	15.04	16.77	14.35
0.466	-0.51	0.70	Q8BL86	Metallo-beta-lactamase domain-containing protein 2	Mblac2	3113	3	3	3	15.1	15.1	15.1	31.205	0.0000	12.083	7135700	26	16.20	16.27	14.27	15.71	16.55	16.30	15.87	15.79
0.578	-0.52	0.70	Q9DC29	ATP-binding cassette sub-family B member 6, mitochondrial	Abcb6	5189	10	9	9	21	20.1	20.1	93.769	0.0000	175.96	14086000	102	15.19	15.91	15.84	14.43	15.96	15.37	16.47	15.63
1.328	-0.52	0.70	Q9D051	Pyruvate dehydrogenase E1 component subunit beta, mitochondrial	Pdhb	4852	19	19	19	51.8	51.8	51.8	38.937	0.0000	219.54	130680000	508	18.44	18.37	17.83	17.94	18.58	18.95	18.31	18.80
0.346	-0.52	0.70	P34884	Macrophage migration inhibitory factor	Mif	1007	7	7	7	60.9	60.9	60.9	12.504	0.0000	50.328	111070000	250	18.21	19.24	17.39	18.12	18.84	18.93	17.38	19.88
1.149	-0.52	0.70	Q8CIG9	F-box/LRR-repeat protein 8	Fbxl8	3554	4	4	4	14.2	14.2	14.2	41.144	0.0000	15.029	1612300	21	13.60	13.26	13.58	13.07	13.67	14.17	13.46	14.28
0.690	-0.52	0.70	Q6NXI6	Regulation of nuclear pre-mRNA domain-containing protein 2	Rprd2	2538	14	14	14	15	15	15	156.58	0.0000	103.42	12626000	204	14.51	14.67	15.85	14.97	15.16	15.90	15.15	15.85
0.332	-0.52	0.70	Q80W47	WD repeat domain phosphoinositide-interacting protein 2	Wipi2	2857	4	4	4	19.8	19.8	19.8	48.477	0.0000	129.38	4378100	30	14.10	14.32	13.98	13.41	15.07	15.95	13.72	13.15
0.478	-0.52	0.70	Q9DCJ9	N-acetylneuraminate lyase	Npl	5219	11	11	11	64.7	64.7	64.7	35.13	0.0000	139.45	13171000	146	14.48	14.87	16.38	14.25	15.30	15.32	15.66	15.78

1.253	-0.52	0.70	P39749	Flap endonuclease 1	Fen1	1055	18	18	18	48.4	48.4	48.4	42.314	0.0000	149.3	82708000	419	16.94	17.29	17.89	17.03	17.69	17.88	17.77	17.90
1.085	-0.52	0.70	Q9D620	Rab11 family-interacting protein 1	Rab11fip1	4988	9	9	9	16.4	16.4	16.4	70.683	0.0000	120.2	7790200	106	14.22	15.11	15.07	14.58	15.33	15.47	15.38	14.88
0.609	-0.52	0.70	P63254	Cysteine-rich protein 1	Crip1	1498	7	7	6	84.4	84.4	84.4	8.5497	0.0000	19.011	82817000	259	18.02	18.31	18.50	19.05	18.82	18.09	19.45	19.60
0.962	-0.52	0.70	Q8K2Z4	Condensin complex subunit 1	Ncapd2	3681	20	20	20	22.2	22.2	22.2	155.66	0.0000	123.43	16826000	158	14.59	15.15	15.76	15.11	15.34	15.63	16.03	15.69
1.479	-0.52	0.70	Q8C0E3	Tripartite motif-containing protein 47	Trim47	3335	12	12	12	31	31	31	69.912	0.0000	109.14	18401000	211	16.18	15.95	15.96	15.48	16.07	16.63	16.47	16.48
0.784	-0.52	0.70	Q61733	28S ribosomal protein S31, mitochondrial	Mrps31	2328	15	15	15	37.5	37.5	37.5	43.88	0.0000	177.21	26270000	313	15.73	16.66	15.99	15.77	16.16	16.89	16.10	17.09
1.386	-0.52	0.70	Q8K0Q5	Rho GTPase-activating protein 18	Arhgap18	3609	10	10	10	17.5	17.5	17.5	74.929	0.0000	33.145	13694000	140	15.84	15.21	15.10	15.37	15.76	15.64	16.10	16.12
0.647	-0.53	0.69	P97369	Neutrophil cytosol factor 4	Ncf4	1634	29	29	29	76.1	76.1	76.1	38.707	0.0000	323.31	264540000	935	18.34	18.52	16.91	18.33	18.25	18.55	18.74	18.67
1.108	-0.53	0.69	O35691	Pinin	Pnn	399	24	24	24	26.5	26.5	26.5	82.435	0.0000	132.43	54371000	412	15.47	15.93	15.59	15.95	15.62	16.56	16.41	16.46
0.908	-0.53	0.69	Q99KR7	Peptidyl-prolyl cis-trans isomerase F, mitochondrial	Ppif	4392	6	5	5	36.9	33.5	33.5	21.737	0.0000	16.546	6546700	42	15.11	15.97	14.92	15.46	16.22	16.14	15.80	15.42
0.961	-0.53	0.69	O70200	Allograft inflammatory factor 1	Aif1	477	5	5	5	37.4	37.4	37.4	16.91	0.0000	33.897	5939900	53	14.11	15.05	14.31	14.93	14.87	14.87	15.21	15.55
0.434	-0.53	0.69	Q8BG51	Mitochondrial Rho GTPase 1	Rhot1	2964	13	13	12	25.8	25.8	24.1	72.241	0.0000	73.893	11161000	118	15.05	15.34	13.09	15.06	15.33	14.88	15.53	14.91
0.558	-0.53	0.69	Q8BMS4	Ubiquinone biosynthesis O-methyltransferase, mitochondrial	Coq3	3149	4	4	4	15.4	15.4	15.4	40.956	0.0000	8.999	3356800	27	14.08	14.46	13.29	14.27	14.76	15.30	14.58	13.57
0.256	-0.53	0.69	Q3THE2;Q9	Myosin regulatory light chain 12B;Myosin regulatory light polypeptide 9	Myl12b;Myl9	1842	6	6	6	44.2	44.2	44.2	19.779	0.0000	96.621	13688000	75	16.02	17.22	13.47	15.25	15.92	16.35	15.17	16.63
0.400	-0.53	0.69	Q8VBV7	COP9 signalosome complex subunit 8	Cops8	3898	8	8	8	70.3	70.3	70.3	23.255	0.0000	35.57	56617000	128	18.44	18.26	16.23	18.79	18.34	18.64	18.32	18.54
1.341	-0.53	0.69	Q8K1E0	Syntaxin-5	Stx5	3625	11	11	11	44.2	44.2	44.2	39.713	0.0000	93.409	30866000	204	16.43	16.27	15.69	16.52	16.53	16.95	16.89	16.67
0.196	-0.53	0.69	Q64112	Interferon-induced protein with tetratricopeptide repeats 2	Ifit2	2418	15	15	15	36.7	36.7	36.7	55.02	0.0000	208.33	22477000	141	14.45	12.66	13.31	14.94	12.99	12.54	16.25	15.71
0.981	-0.53	0.69	Q922H9	Zinc finger protein 330	Znf330	4259	3	3	3	14.9	14.9	14.9	35.607	0.0000	33.445	2891700	64	14.39	13.91	13.52	14.24	14.71	15.02	14.10	14.36
0.567	-0.54	0.69	Q6ZQ58	La-related protein 1	Larp1	2690	13	13	13	20	20	20	121.12	0.0000	72.931	8170900	76	14.47	14.69	15.96	14.57	15.66	15.61	15.90	14.66
0.372	-0.54	0.69	Q8CGP0;Q9	Histone H2B type 3-B;Histone H2B type 3-A	Hist3h2bb;Hist3h2	3510	18	2	0	79.4	7.9	0	13.908	0.0025	2.9282	3927700	23	13.51	14.19	13.13	12.77	15.38	13.01	13.15	14.20
0.656	-0.54	0.69	E9Q634	Unconventional myosin-Ie	Myo1e	241	53	44	44	47.2	41.6	41.6	126.82	0.0000	313.77	78842000	573	16.01	15.97	17.56	16.86	16.86	17.11	17.27	17.29
1.958	-0.54	0.69	P80315	T-complex protein 1 subunit delta	Cct4	1592	44	44	44	87.4	87.4	87.4	58.066	0.0000	323.31	761510000	2046	19.62	19.77	19.63	19.89	19.98	20.51	20.48	20.10
0.351	-0.54	0.69	P33215	Protein NEDD1	Nedd1	1001	5	5	5	14.7	14.7	14.7	71.292	0.0000	32.41	6452100	79	14.79	14.60	12.35	15.00	14.48	14.98	15.24	14.20
0.838	-0.54	0.69	Q80X80	C2 domain-containing protein 2-like	C2cd2l	2873	5	5	5	10.9	10.9	10.9	76.328	0.0000	15.02	1705400	38	12.05	12.71	13.26	12.95	13.11	13.79	13.33	12.89
0.588	-0.54	0.69	P62274	40S ribosomal protein S29	Rps29	1414	6	6	6	67.9	67.9	67.9	6.6767	0.0000	47.096	221640000	335	19.36	19.63	18.24	19.65	19.62	19.12	19.87	20.43
1.364	-0.54	0.69	P17182	Alpha-enolase	Eno1	820	66	66	58	97.9	97.9	84.1	47.14	0.0000	323.31	8708300000	9696	21.88	22.14	21.25	21.92	22.10	22.53	22.32	22.40
0.782	-0.54	0.69	Q8BFW7	Lipoma-preferred partner homolog	Lpp	2951	9	9	9	24.3	24.3	24.3	65.89	0.0000	106.76	15941000	172	15.77	15.86	14.55	14.88	15.77	16.10	15.79	15.56
2.504	-0.54	0.69	P62702	40S ribosomal protein S4, X isoform	Rps4x	1436	34	34	34	79.5	79.5	79.5	29.597	0.0000	323.31	663610000	1226	19.43	19.40	19.31	19.72	20.18	19.83	20.03	19.99
0.289	-0.54	0.69	P09602	Non-histone chromosomal protein HMG-17	Hmgn2	700	5	5	5	60	60	60	9.4226	0.0000	25.991	1325500	20	11.87	13.22	13.94	14.55	14.52	13.12	15.14	12.97
0.231	-0.54	0.69	P35762	CD81 antigen	Cd81	1032	1	1	1	8.5	8.5	8.5	25.814	0.0000	69.602	7115100	53	16.18	16.37	13.70	12.74	14.61	15.65	15.84	15.07
0.365	-0.55	0.68	Q80Y14	Glutaredoxin-related protein 5, mitochondrial	Glrx5	2893	3	3	3	29.6	29.6	29.6	16.292	0.0000	9.865	4464700	52	15.10	14.99	14.93	12.82	14.47	16.01	14.88	14.65
1.578	-0.55	0.68	Q3UM18	Large subunit GTPase 1 homolog	Lsg1	1995	17	17	17	30.6	30.6	30.6	73.156	0.0000	91.325	14075000	178	15.13	15.69	15.20	15.51	15.74	15.79	15.86	16.33
0.572	-0.55	0.68	Q7TSC1	Protein PRRC2A	Prrc2a	2780	5	4	4	3.4	3.1	3.1	229.2	0.0000	23.349	2409900	38	13.19	13.69	15.12	13.88	14.48	14.74	14.01	14.84
1.148	-0.55	0.68	Q9D8T7	SRA stem-loop-interacting RNA-binding protein, mitochondrial	Slirp	5074	8	8	8	68.8	68.8	68.8	12.605	0.0000	108.84	51728000	299	16.56	17.00	15.94	16.36	16.84	17.32	16.80	17.10
0.857	-0.55	0.68	O55102	Biogenesis of lysosome-related organelles complex 1 subunit 1	Bloc1s1	453	6	6	6	74.4	74.4	74.4	14.281	0.0000	54.508	5956500	75	14.92	14.90	15.02	14.55	15.54	15.04	14.82	16.20
1.683	-0.55	0.68	P22682	E3 ubiquitin-protein ligase CBL	Cbl	876	15	15	13	22.3	22.3	20.5	100.56	0.0000	153.87	40444000	261	16.36	16.56	16.04	16.47	16.61	17.14	17.13	16.75
0.553	-0.55	0.68	Q9Z0L8	Gamma-glutamyl hydrolase	Ggh	5775	5	5	5	19.9	19.9	19.9	35.469	0.0000	15.865	43028000	97	16.92	18.27	17.37	16.21	17.58	17.58	18.25	17.57
1.073	-0.55	0.68	Q9Z2Y8	Proline synthase co-transcribed bacterial homolog protein	Prosc	5873	17	17	17	65	65	65	30.048	0.0000	126.8	135650000	463	18.06	18.35	17.34	18.12	18.38	18.97	18.32	18.41
0.536	-0.55	0.68	Q8VI84	Nucleolar complex protein 3 homolog	Noc3l	4031	7	7	7	11.2	11.2	11.2	93.21	0.0000	8.5874	2278900	19	13.51	13.04	13.94	13.82	12.90	14.29	14.43	14.91
1.481	-0.55	0.68	Q8BX80	Cytosolic endo-beta-N-acetylglucosaminidase	Engase	3280	5	5	5	10.1	10.1	10.1	82.944	0.0000	17.144	8958000	97	14.95	14.87	15.30	14.47	15.25	15.73	15.50	15.31
0.755	-0.55	0.68	Q9D1G5	Leucine-rich repeat-containing protein 57	Lrrc57	4913	10	10	10	55.2	55.2	55.2	26.76	0.0000	61.871	10374000	123	14.99	14.91	13.71	14.89	15.00	15.72	15.17	14.83
0.427	-0.55	0.68	Q3SXD3	HD domain-containing protein 2	Hddc2	1810	3	3	3	20.1	20.1	20.1	22.753	0.0000	6.8691	1333100	15	12.50	11.47	14.21	12.89	13.18	13.57	13.42	13.10
1.535	-0.55	0.68	Q9Z1M8	Protein Red	Ik	5814	19	19	19	42.4	42.4	42.4	65.615	0.0000	106.81	45252000	348	16.64	17.22	16.63	16.56	17.05	17.53	17.18	17.49
0.672	-0.55	0.68	O70152	Dolichol-phosphate mannosyltransferase subunit 1	Dpm1	473	12	12	12	47.7	47.7	47.7	29.174	0.0000	78.347	12633000	108	15.44	14.80	13.89	15.58	15.49	15.49	15.23	15.73
0.582	-0.56	0.68	Q68FH4	N-acetylgalactosamine kinase	Galk2	2475	23	23	23	56.3	56.3	56.3	50.503	0.0000	323.31	67146000	390	17.31	17.36	15.63	17.15	17.44	17.62	17.69	16.92
0.933	-0.56	0.68	Q9QXK7	Cleavage and polyadenylation specificity factor subunit 3	Cpsf3	5544	12	12	12	24.3	24.3	24.3	77.504	0.0000	90.465	19409000	85	16.34	16.08	16.14	16.11	15.97	17.18	17.22	16.51
0.854	-0.56	0.68	Q8BZT9	Laccase domain-containing protein 1	Lacc1	3319	6	6	6	23.5	23.5	23.5	47.514	0.0000	155.97	9112100	72	15.51	15.13	16.02	16.06	16.04	16.61	16.65	15.65
0.939	-0.56	0.68	Q9CQ79	Thioredoxin domain-containing protein 9	Txndc9	4571	5	5	5	31.4	31.4	31.4	26.259	0.0000	58.879	2219800	47	13.30	12.56	13.54	13.89	13.67	14.02	14.11	13.72
1.955	-0.56	0.68	Q501J6	Probable ATP-dependent RNA helicase DDX17	Ddx17	2075	48	36	36	54.8	44.3	44.3	72.399	0.0000	323.31	175920000	970	17.53	17.75	18.03	17.96	18.31	18.62	18.13	18.43
0.586	-0.56	0.68	O88878	AN1-type zinc finger protein 5	Zfand5	581	3	3	3	28.2	28.2	28.2	23.058	0.0000	14.116	1232100	31	12.57	12.64	12.34	12.27	13.12	13.60	11.74	13.59
1.714	-0.56	0.68	P62192	26S protease regulatory subunit 4	Psmc1	1406	35	35	35	71.6	71.6	71.6	49.184	0.0000	323.31	234470000	1095	17.72	18.03	17.89	17.77	18.15	18.49	18.17	18.84
0.650	-0.56	0.68	Q64433	10 kDa heat shock protein, mitochondrial	Hspe1	2441	15	15	15	99	99	99	10.963	0.0000	56.879	56801000	331	17.32	18.01	17.52	17.55	18.42	18.77	17.03	18.43
0.401	-0.56	0.68	Q9JLB0	MAGUK p55 subfamily member 6	Mpp6	5475	10	10	10	28.4	28.4	28.4	62.63	0.0000	38.706	7896200	98	14.86	14.45	15.26	13.16	13.92	14.76	15.62	15.67
0.638	-0.56	0.68	O55236	mRNA-capping enzyme;Polynucleotide 5-triphosphatase;mRNA guanyl	Rngtt	467	10	10	10	21.3	21.3	21.3	68.683	0.0000	10.129	5585500	40	14.17	12.90	13.06	14.43	13.86	14.41	13.98	14.54
0.393	-0.56	0.68	Q91YJ3	Thymocyte nuclear protein 1	Thyn1	4162	6	6	6	36.3	36.3	36.3	26.178	0.0000	28.43	6026800	62	13.81	15.15	15.19	13.19	15.80	15.24	14.15	14.41
1.237	-0.56	0.68	Q61687	Transcriptional regulator ATRX	Atrx	2323	10	10	10	5.5	5.5	5.5	278.58	0.0000	41.829	6752600	107	14.48	13.92	14.02	14.38	14.35	14.79	14.61	15.29
1.197	-0.56	0.68	Q9D8T2	Gasdermin-D	Gsdmdc1	5072	22	22	22	53.8	53.8	53.8	53.237	0.0000	321.69	113080000	548	17.69	17.85	16.94	17.62	18.02	18.51	17.89	17.93
0.386	-0.56	0.68	Q9Z0V8	Mitochondrial import inner membrane translocase subunit Tim17-A	Timm17a	5789	3	3	3	37.4	37.4	37.4	18.111	0.0000	38.573	1700100	13	13.65	11.47	13.84	13.80	13.21	13.89	13.45	14.46
1.126	-0.56	0.68	Q9CQA9	Cancer-related nucleoside-triphosphatase homolog	Ntpcr	4580	11	11	11	79.5	79.5	79.5	20.667	0.0000	104.02	46362000	264	16.65	16.87	15.90	16.91	16.91	17.44	17.20	17.04
0.438	-0.56	0.68	Q68ED3	Non-canonical poly(A) RNA polymerase PAPD5	Papd5	2471	2	2	2	5.1	5.1	5.1	69.703	0.0000	13.453	579770	35	12.09	12.06	12.92	12.76	13.45	12.11	12.20	14.32
1.762	-0.57	0.68	Q04750	DNA topoisomerase 1	Top1	1734	33	33	33	41.6	41.6	41.6	90.875	0.0000	130.77	57833000	548	16.03	15.71	15.68	15.82	16.42	16.71	15.96	16.40
1.114	-0.57	0.68	Q91ZX7	Prolow-density lipoprotein receptor-related protein 1;Low-density lipopro	Lrp1	4205	111	111	109	25.1	25.1	24.8	504.74	0.0000	323.31	577650000	2984	18.41	18.01	18.64	18.55	19.39	18.49	19.31	18.68
0.734	-0.57	0.68	Q8JZX4	Splicing factor 45	Rbm17	3586	8	8	8	24	24	24	45.303	0.0000	31.787	15830000	139	16.08	16.49	14.96	16.35	16.38	16.92	16.24	16.61
0.255	-0.57	0.68	P97364	Selenide, water dikinase 2	Sephs2	1632	8	8	8	37.6	37.6	37.6	47.833	0.0000	197.58	18888000	102	16.85	16.94	16.21	15.85	17.67	18.20	17.81	14.44
1.741	-0.57	0.68	Q9QXA5	U6 snRNA-associated Sm-like protein LSm4	Lsm4	5534	11	11	11	58.4	58.4	58.4	15.076	0.0000	56.37	52565000	273	16.49	17.19	16.75	16.45	17.16	17.37	17.31	17.31
1.134	-0.57	0.67	Q7TNV0	Protein DEK	Dek	2765	14	14	14	31.3	31.3	31.3	43.158	0.0000	52.303	43205000	276	16.63	17.34	17.69	17.23	17.49	17.86	17.66	18.15
0.135	-0.57	0.67	Q8BGS7	Choline/ethanolaminephosphotransferase 1	Cept1	2992	5	5	5	13.9	13.9	13.9	46.434	0.0000	22.71	15786000	64	16.94	12.70	15.65	17.12	12.62	17.52	17.94	16.61
1.828	-0.57	0.67	Q0GNC1	Inverted formin-2	Inf2	1774	25	25	25	30.5	30.5	30.5	138.56	0.0000	230.98	53267000	403	16.97	16.96	16.57	16.41	17.10	17.20	17.36	17.53
0.555	-0.57	0.67	Q9JLT4	Thioredoxin reductase 2, mitochondrial	Txnrd2	5490	12	10	10	36.1	33	33	56.602	0.0000	135.04	24578000	125	16.55	15.98	17.20	16.51	17.25	17.71	17.62	15.94
0.810	-0.57	0.67	Q811D0	Disks large homolog 1	Dlg1	2936	14	14	14	20.1	20.1	20.1	100.12	0.0000	74.109	32042000	152	16.39	16.57	15.96	15.62	16.81	17.07	15.89	17.06
0.468	-0.57	0.67	O55125	Protein NipSnap homolog 1	Nipsnap1	455	6	6	6	19.7	19.7	19.7	33.363	0.0000	8.4139	4995800	78	14.87	14.75	12.81	15.05	14.60	15.42	14.73	15.01
1.708	-0.57	0.67	P97351	40S ribosomal protein S3a	Rps3a	1627	39	39	39	75.8	75.8	75.8	29.885	0.0000	323.31	705210000	1848	19.33	19.25	18.84	19.66	19.72	20.02	19.85	19.78
1.227	-0.57	0.67	Q99NH0	Ankyrin repeat domain-containing protein 17	Ankrd17	4498	10	10	10	6.4	6.4	6.4	274.21	0.0000	42.194	8526900	132	15.12	15.19	15.78	14.65	15.51	15.86	15.79	15.89
0.403	-0.57	0.67	Q99JT1	Glutamyl-tRNA(Gln) amidotransferase subunit B, mitochondrial	Gatb	4347	5	5	5	15.1	15.1	15.1	62.118	0.0000	17.455	10727000	64	16.09	16.12	13.65	16.15	15.76	16.28	16.08	16.20
2.036	-0.58	0.67	B2RRE7	OTU domain-containing protein 4	Otud4	79	7	7	6	9.4	9.4	6.7	123.05	0.0000	16.01	3997700	71	13.69	14.01	13.98	14.22	14.31	14.80	14.62	14.47
1.440	-0.58	0.67	Q62448	Eukaryotic translation initiation factor 4 gamma 2	Eif4g2	2396	32	32	32	42.6	42.6	42.6	102.1	0.0000	323.31	115070000	780	17.05	17.18	16.42	17.27	17.29	17.67	17.53	17.72
1.732	-0.58	0.67	P25976	Nucleolar transcription factor 1	Ubtf	913	29	29	29	35.4	35.4	35.4	89.508	0.0000	129.12	40897000	356	15.76	15.90	15.76	15.48	15.94	16.24	16.32	16.70
0.679	-0.58	0.67	Q3UBG2	PTB-containing, cubilin and LRP1-interacting protein	Pid1	1941	11	11	10	55.8	55.8	52.1	24.784	0.0000	55.522	15003000	112	15.22	15.01	13.97	15.43	14.76	15.54	15.87	15.77
1.646	-0.58	0.67	Q8CCF0	U4/U6 small nuclear ribonucleoprotein Prp31	Prpf31	3446	19	19	19	41.7	41.7	41.7	55.429	0.0000	137.87	35066000	336	15.83	16.02	16.27	15.73	16.38	16.98	16.40	16.40
0.242	-0.58	0.67	Q99NH8	Triggering receptor expressed on myeloid cells 2	Trem2	4499	3	3	3	32.2	32.2	32.2	24.527	0.0000	29.906	9404100	84	15.90	15.54	12.03	14.72	15.38	14.59	16.18	14.35
0.926	-0.58	0.67	Q99MR8	Methylcrotonoyl-CoA carboxylase subunit alpha, mitochondrial	Mccc1	4475	23	23	23	44.6	44.6	44.6	79.343	0.0000	91.578	34850000	270	16.07	15.64	15.35	15.77	16.73	16.53	16.42	15.47
1.220	-0.58	0.67	Q9DBC0	Selenoprotein O	Selo	5141	14	14	14	31.8	31.8	31.8	74.22	0.0000	70.554	17457000	135	14.57	14.38	15.24	15.17	15.52	15.04	15.50	15.61
1.034	-0.58	0.67	Q61205	Platelet-activating factor acetylhydrolase IB subunit gamma	Pafah1b3	2275	11	11	11	64.2	64.2	64.2	25.853	0.0000	58.817	16208000	124	15.53	16.10	14.80	15.72	16.14	15.84	16.15	16.32
2.479	-0.58	0.67	Q9R190	Metastasis-associated protein MTA2	Mta2	5648	28	28	23	43.6	43.6	37.6	75.029	0.0000	231	72366000	580	16.97	16.84	17.17	17.13	17.34	17.64	17.66	17.79
1.632	-0.58	0.67	Q6ZWV3	60S ribosomal protein L10	Rpl10	2707	26	26	6	75.2	75.2	14.5	24.604	0.0000	323.31	374820000	1013	19.01	18.56	19.08	19.18	19.36	19.26	19.81	19.72
0.457	-0.58	0.67	Q9WUQ2	Prolactin regulatory element-binding protein	Preb	5720	13	13	13	38.4	38.4	38.4	45.437	0.0000	190.63	30619000	269	16.35	15.94	14.07	16.42	16.19	16.38	16.61	15.93
2.548	-0.58	0.67	Q9D6Z1	Nucleolar protein 56	Nop56	5014	31	31	31	66.9	66.9	66.9	64.464	0.0000	323.31	98856000	852	16.21	16.29	16.15	16.39	16.88	16.56	17.08	16.83
0.690	-0.58	0.67	Q60930	Voltage-dependent anion-selective channel protein 2	Vdac2	2222	24	24	24	78.3	78.3	78.3	31.732	0.0000	323.31	581200000	1185	20.32	20.21	18.67	20.25	20.46	20.59	20.55	20.18
0.190	-0.58	0.67	Q9D5R3	Centrosomal protein of 83 kDa	Cep83	4984	1	1	1	1.4	1.4	1.4	81.997	0.0095	1.8176	22604000	78	15.92	16.26	13.60	15.37	12.81	17.49	16.88	16.32
1.537	-0.58	0.67	Q9DBC7	cAMP-dependent protein kinase type I-alpha regulatory subunit;cAMP-d	Prkar1a	5143	27	27	20	60.9	60.9	49.3	43.185	0.0000	218.86	174730000	827	17.53	18.05	17.81	18.04	18.17	18.84	18.18	18.58
0.634	-0.58	0.67	Q9WV03	Protein FAM50A	Fam50a	5728	14	14	13	37.2	37.2	33.6	40.251	0.0000	28.288	10661000	93	13.56	15.32	13.62	14.73	14.66	14.99	14.90	14.99
2.060	-0.58	0.67	P10853;Q8C	Histone H2B type 1-F/J/L;Histone H2B type 1-P	Hist1h2bf;Hist1h2l	732	27	27	0	96	96	0	13.936	0.0000	323.31	7820800000	3130	23.31	23.49	23.89	23.65	24.25	24.00	24.05	24.39
1.668	-0.58	0.67	Q7TN29	Stromal membrane-associated protein 2	Smap2	2756	11	11	10	27.6	27.6	25.7	46.577	0.0000	57.201	23718000	155	16.16	16.24	15.88	15.80	16.68	16.34	17.01	16.38
1.383	-0.59	0.67	P99029	Peroxiredoxin-5, mitochondrial	Prdx5	1694	19	19	19	59.5	59.5	59.5	21.897	0.0000	323.31	405710000	934	19.73	20.29	20.23	19.71	20.53	21.02	20.53	20.24
1.394	-0.59	0.67	P63158	High mobility group protein B1	Hmgb1	1493	29	22	22	74	57.2	57.2	24.893	0.0000	207.94	371440000	935	19.09	19.85	19.17	19.39	20.26	19.87	19.59	20.12
2.508	-0.59	0.67	P70248	Unconventional myosin-If	Myo1f	1539	59	59	50	46.2	46.2	41.7	125.95	0.0000	323.31	314820000	1454	17.98	18.02	18.36	18.14	18.73	18.95	18.54	18.64
1.273	-0.59	0.67	Q8VCN9	Tubulin-specific chaperone C	Tbcc	3929	8	8	8	38.7	38.7	38.7	38.124	0.0000	57.772	20628000	200	16.05	16.08	15.61	16.15	16.33	17.07	16.72	16.12
0.886	-0.59	0.66	Q8BHL3	TBC1 domain family member 10B	Tbc1d10b	3044	13	13	13	24.6	24.6	24.6	87.274	0.0000	25.141	13038000	96	15.22	15.11	15.97	14.59	15.59	15.45	16.21	16.02
1.271	-0.59	0.66	Q810A7	ATP-dependent RNA helicase DDX42	Ddx42	2925	19	19	19	26	26	26	101.96	0.0000	87.165	17246000	184	15.52	15.72	15.08	15.67	15.95	15.72	16.03	16.65
1.909	-0.59	0.66	Q00899	Transcriptional repressor protein YY1	Yy1	1701	8	8	8	15.7	15.7	15.7	44.716	0.0000	46.454	5027100	108	14.12	14.30	13.56	14.00	14.50	14.66	14.48	14.70
1.590	-0.59	0.66	O70194	Eukaryotic translation initiation factor 3 subunit D	Eif3d	476	27	27	27	56.6	56.6	56.6	63.988	0.0000	323.31	251540000	1203	18.07	18.13	17.44	18.37	18.64	18.61	18.53	18.60
2.292	-0.59	0.66	Q8BIJ7	RUN and FYVE domain-containing protein 1	Rufy1	3062	34	34	31	47.2	47.2	46.2	80.376	0.0000	178.89	71223000	655	16.39	16.21	16.26	16.44	16.54	17.04	17.03	17.07
0.409	-0.60	0.66	Q80ZE4	Membrane progestin receptor alpha	Paqr7	2912	1	1	1	6.1	6.1	6.1	39.305	0.0000	7.569	540080	6	12.38	13.57	15.15	12.74	14.13	13.90	13.65	14.54
1.710	-0.60	0.66	O88983	Syntaxin-8	Stx8	591	9	9	9	37.3	37.3	37.3	26.925	0.0000	103.38	35869000	187	16.98	16.85	17.07	16.67	17.26	17.97	17.49	17.25
0.834	-0.60	0.66	Q8BKZ9	Pyruvate dehydrogenase protein X component, mitochondrial	Pdhx	3109	16	16	16	33.7	33.7	33.7	53.998	0.0000	136.27	24795000	255	15.38	16.28	15.50	14.71	15.91	16.40	15.69	16.27
0.554	-0.60	0.66	Q14DQ1	Protein FAM219B	Fam219b	1792	3	3	3	19.3	19.3	19.3	21.006	0.0000	50.556	2240500	47	13.54	13.41	11.71	13.91	13.57	13.96	13.51	13.94
1.142	-0.60	0.66	Q8BHJ5	F-box-like/WD repeat-containing protein TBL1XR1	Tbl1xr1	3040	9	9	7	33.3	33.3	25.3	55.66	0.0000	181.5	10277000	93	15.44	15.47	14.59	15.26	15.59	15.93	15.40	16.24
2.277	-0.60	0.66	Q9JIX8	Apoptotic chromatin condensation inducer in the nucleus	Acin1	5410	29	29	29	24.7	24.7	24.7	150.72	0.0000	203.16	29029000	266	15.27	15.33	15.56	15.24	16.00	16.28	15.79	15.75

0.725	-0.60	0.66	Q99J95	Cyclin-dependent kinase 9	Cdk9	4334	6	5	5	16.7	14.5	14.5	42.761	0.0000	20.327	4411200	72	14.23	14.38	12.97	14.43	14.30	15.22	14.48	14.40
0.223	-0.60	0.66	Q99PW4	TP53-regulating kinase	Tp53rk	4520	3	3	3	17.2	17.2	17.2	27.393	0.0000	49.229	7606700	70	15.44	15.85	11.83	15.24	16.09	13.58	15.70	15.39
1.067	-0.60	0.66	Q80WJ7	Protein LYRIC	Mtdh	2863	20	20	20	40.4	40.4	40.4	63.845	0.0000	323.31	95079000	620	16.97	16.94	18.14	17.14	17.75	18.04	18.00	17.81
2.332	-0.60	0.66	Q922D8	C-1-tetrahydrofolate synthase, cytoplasmic;Methylenetetrahydrofolate d	Mthfd1	4251	66	66	65	76.3	76.3	75.2	101.2	0.0000	323.31	286760000	1555	17.35	17.53	17.42	17.65	17.73	18.19	18.23	18.21
0.421	-0.60	0.66	Q8BGS2	BolA-like protein 2	Bola2	2991	7	7	7	77.9	77.9	77.9	10.214	0.0000	24.338	8532200	95	14.07	15.02	16.39	15.81	15.48	16.95	15.18	16.09
1.288	-0.60	0.66	Q8C052	Microtubule-associated protein 1S;MAP1S heavy chain;MAP1S light ch	Map1s	3325	26	26	26	31.8	31.8	31.8	102.94	0.0000	122.29	127530000	500	18.95	19.11	19.87	19.08	19.50	20.14	19.97	19.80
0.429	-0.60	0.66	Q00422	GA-binding protein alpha chain	Gabpa	1696	2	2	2	7.5	7.5	7.5	51.344	0.0000	45.005	3786400	50	14.83	13.73	12.65	14.71	13.59	14.59	15.35	14.80
0.779	-0.61	0.66	Q8CJF7	Protein ELYS	Ahctf1	3569	10	10	10	6.2	6.2	6.2	247.64	0.0000	12.647	2849500	25	12.57	13.17	13.25	13.99	13.28	14.37	13.60	14.16
1.328	-0.61	0.66	Q8VD12	Zinc finger protein 385A	Znf385a	3939	12	12	12	35.5	35.5	35.5	40.446	0.0000	107.88	14107000	181	15.08	15.18	14.84	14.66	15.21	16.12	15.61	15.24
0.640	-0.61	0.66	O88291	DBIRD complex subunit ZNF326	Znf326	521	9	9	9	22.4	22.4	22.4	65.225	0.0000	190.91	15667000	140	15.86	15.78	14.05	15.56	15.71	16.36	15.92	15.69
0.942	-0.61	0.66	Q8CGP5	Histone H2A type 1-F	Hist1h2af	3512	18	5	1	80	28.5	6.9	14.161	0.0000	154.48	2311700000	2259	22.49	22.56	22.99	22.04	23.53	22.39	23.09	23.51
0.697	-0.61	0.65	O35368	Interferon-activable protein 203	Ifi203	362	9	3	3	27.5	13	13	46.299	0.0002	5.878	1130200	12	13.29	12.85	13.84	14.21	13.88	13.62	15.02	14.13
0.278	-0.61	0.65	Q62241	U1 small nuclear ribonucleoprotein C	Snrpc	2371	2	2	2	13.2	13.2	13.2	17.364	0.0000	41.687	7192500	44	12.98	16.80	14.55	15.19	15.65	13.57	16.12	16.11
1.190	-0.62	0.65	Q99K90	TGF-beta-activated kinase 1 and MAP3K7-binding protein 2	Tab2	4367	9	9	9	21.9	21.9	21.9	76.441	0.0000	39.613	8694100	97	14.59	15.08	14.09	14.89	15.12	15.78	15.10	15.11
0.814	-0.62	0.65	Q91YP3	Deoxyribose-phosphate aldolase	Dera	4171	12	12	12	52.8	52.8	52.8	34.975	0.0000	155.04	29859000	241	16.54	16.57	15.22	16.54	16.33	17.17	16.87	16.97
1.377	-0.62	0.65	P31230	Aminoacyl tRNA synthase complex-interacting multifunctional protein 1;	Aimp1	984	17	17	17	70.3	70.3	70.3	33.997	0.0000	282.25	59019000	483	16.26	16.81	16.92	16.17	17.24	17.44	16.74	17.23
0.203	-0.62	0.65	Q9D6S7	Ribosome-recycling factor, mitochondrial	Mrrf	5008	8	8	8	46.2	46.2	46.2	29.05	0.0000	183.98	12519000	103	14.94	16.39	15.08	14.82	17.40	17.19	16.61	12.50
0.461	-0.62	0.65	O55013	Trafficking protein particle complex subunit 3	Trappc3	441	7	7	7	33.3	33.3	33.3	20.302	0.0000	16.325	15080000	124	15.52	16.17	13.48	15.54	16.11	15.69	16.00	15.41
0.533	-0.62	0.65	Q9CZX7	Type 2 phosphatidylinositol 4,5-bisphosphate 4-phosphatase	Tmem55a	4845	8	8	7	54.9	54.9	51	28.038	0.0000	117.5	33045000	117	17.17	16.45	15.51	15.89	17.18	17.09	17.52	15.71
0.844	-0.62	0.65	Q0VBL3		Rbm15	1777	12	12	12	18.8	18.8	18.8	105.72	0.0000	51.813	12191000	86	14.79	15.18	14.82	15.69	15.00	16.30	16.17	15.48
0.680	-0.62	0.65	P35700	Peroxiredoxin-1	Prdx1	1029	33	33	21	99.5	99.5	80.9	22.176	0.0000	323.31	3129500000	2868	22.41	22.95	20.93	22.20	23.07	22.70	22.66	22.54
0.233	-0.62	0.65	P56379	6.8 kDa mitochondrial proteolipid	Mp68	1281	2	2	2	34.5	34.5	34.5	6.6979	0.0009	3.7181	18471000	71	17.02	16.74	12.85	17.04	16.50	15.91	16.25	17.49
0.639	-0.62	0.65	P56183	Ribosomal RNA processing protein 1 homolog A	Rrp1	1276	4	4	4	16.6	16.6	16.6	54.776	0.0000	21.957	1020500	20	12.03	11.94	13.46	12.79	12.41	13.76	13.00	13.54
0.523	-0.62	0.65	Q8VEH8	Endoplasmic reticulum lectin 1	Erlec1	4006	3	3	3	6.4	6.4	6.4	54.906	0.0011	3.634	3276200	31	12.86	14.45	14.39	14.35	15.38	13.57	15.02	14.57
1.589	-0.62	0.65	Q99PL6	UBX domain-containing protein 6	Ubxn6	4508	21	21	21	55.7	55.7	55.7	49.795	0.0000	79.579	68308000	304	17.52	17.51	16.79	17.44	17.60	18.01	18.13	18.01
2.204	-0.63	0.65	Q3UIA2	Rho GTPase-activating protein 17	Arhgap17	1979	36	36	34	54.1	54.1	54.1	92.201	0.0000	323.31	115160000	679	17.21	17.29	17.80	17.19	17.86	18.09	18.01	18.02
0.707	-0.63	0.65	Q8BFU3	RING finger protein 214	Rnf214	2948	6	6	6	13	13	13	73.624	0.0000	13.299	3157700	28	13.75	13.81	15.17	13.43	14.43	15.24	14.53	14.45
1.475	-0.63	0.65	P62830	60S ribosomal protein L23	Rpl23	1449	16	16	16	77.1	77.1	77.1	14.865	0.0000	157.63	818850000	1007	20.66	21.10	20.60	20.47	20.94	21.75	21.52	21.12
0.458	-0.63	0.65	P18572	Basigin	Bsg	837	4	4	4	12.9	12.9	12.9	42.444	0.0000	8.3718	3665000	42	14.09	14.82	12.82	13.85	14.35	15.18	13.29	15.28
0.993	-0.63	0.65	Q9Z2E2	Methyl-CpG-binding domain protein 1	Mbd1	5847	2	2	2	4.2	4.2	4.2	70.022	0.0000	7.1024	719460	6	12.47	13.30	13.60	13.14	13.32	14.19	14.08	13.44
1.205	-0.63	0.65	Q9Z2I8	Succinyl-CoA ligase [GDP-forming] subunit beta, mitochondrial	Suclg2	5856	20	20	20	51	51	51	46.839	0.0000	256.78	80761000	550	17.11	17.27	16.39	17.15	17.23	18.12	17.62	17.46
0.562	-0.63	0.65	O54965	E3 ubiquitin-protein ligase RNF13	Rnf13	438	4	4	4	11.5	11.5	11.5	42.732	0.0000	18.104	3883300	37	13.59	14.42	12.85	13.82	14.80	13.73	15.18	13.49
0.639	-0.63	0.65	Q8WTY4	Anamorsin	Ciapin1	4036	5	5	5	27.2	27.2	27.2	33.429	0.0000	12.297	2570200	21	13.69	13.86	12.63	14.14	14.45	13.23	14.73	14.43
1.373	-0.63	0.65	Q8VBT9	Tether containing UBX domain for GLUT4	Aspscr1	3896	20	20	20	41.6	41.6	41.6	59.795	0.0000	151.92	37279000	251	16.27	16.85	16.11	16.67	16.62	17.15	17.46	17.20
1.552	-0.63	0.65	Q8C0D5	Elongation factor Tu GTP-binding domain-containing protein 1	Eftud1	3332	15	15	15	17.7	17.7	17.7	125.78	0.0000	59.071	15644000	169	14.51	14.44	15.22	14.68	14.97	15.53	15.50	15.36
1.467	-0.63	0.65	Q8BVY0	Ribosomal L1 domain-containing protein 1	Rsl1d1	3251	16	16	16	37.6	37.6	37.6	50.421	0.0000	226.52	35053000	173	16.58	16.44	17.31	16.32	17.14	17.43	17.31	17.29
1.180	-0.63	0.64	Q69ZA1	Cyclin-dependent kinase 13	Cdk13	2480	3	2	2	2.8	2.3	2.3	164.55	0.0000	46.912	1390700	58	13.45	13.32	13.46	13.53	14.31	14.32	13.24	14.42
1.007	-0.63	0.64	Q6ZQ73	Cullin-associated NEDD8-dissociated protein 2	Cand2	2691	2	1	1	1.9	1.2	1.2	135.63	0.0004	4.9232	5886700	81	15.29	14.40	13.79	14.30	14.92	14.97	15.32	15.10
1.033	-0.63	0.64	Q3UKU4	Protein FAM83F	Fam83f	1989	3	3	3	10.7	10.7	10.7	55.012	0.0000	63.221	1932000	40	13.43	13.31	14.53	13.33	14.18	14.25	14.62	14.08
0.897	-0.63	0.64	Q69ZQ2	Pre-mRNA-splicing factor ISY1 homolog	Isy1	2489	3	3	3	9.8	9.8	9.8	32.989	0.0000	32.098	5195500	104	14.42	15.31	14.96	14.49	15.67	15.76	14.56	15.73
0.429	-0.63	0.64	Q9QXG4	Acetyl-coenzyme A synthetase, cytoplasmic	Acss2	5541	8	8	8	16.4	16.4	16.4	78.861	0.0000	41.689	4237900	18	13.35	12.64	13.10	15.24	14.43	13.25	14.64	14.56
0.579	-0.64	0.64	Q9CQ48	NudC domain-containing protein 2	Nudcd2	4560	5	5	5	40.8	40.8	40.8	17.66	0.0000	19.958	3700400	26	13.50	14.17	13.48	14.32	14.71	13.21	14.66	15.43
0.360	-0.64	0.64	Q9CY16	28S ribosomal protein S28, mitochondrial	Mrps28	4777	5	5	5	32.8	32.8	32.8	20.52	0.0000	21.263	5654100	49	13.97	15.22	14.90	13.09	14.85	15.78	13.28	15.80
1.281	-0.64	0.64	Q99L47	Hsc70-interacting protein	St13	4409	16	16	16	35.6	35.6	35.6	41.655	0.0000	69.11	66067000	323	17.11	17.43	16.69	17.39	17.74	18.00	17.25	18.18
0.537	-0.64	0.64	P63044	Vesicle-associated membrane protein 2	Vamp2	1485	6	1	1	44.8	13.8	13.8	12.691	0.0000	12.483	3653400	40	14.53	15.09	14.97	13.27	15.37	14.10	15.79	15.15
0.809	-0.64	0.64	Q07813	Apoptosis regulator BAX	Bax	1758	9	9	9	47.4	47.4	47.4	21.394	0.0000	323.31	199600000	554	19.02	18.78	17.34	18.85	19.01	19.19	19.24	19.09
1.650	-0.64	0.64	Q922U1	U4/U6 small nuclear ribonucleoprotein Prp3	Prpf3	4278	24	24	24	41.9	41.9	41.9	77.454	0.0000	158.4	61316000	457	15.50	15.89	16.32	16.02	16.22	16.69	16.63	16.74
0.555	-0.64	0.64	Q9Z2X8	Kelch-like ECH-associated protein 1	Keap1	5872	8	8	8	14.7	14.7	14.7	69.552	0.0000	12.119	3875400	40	14.42	14.03	12.41	13.24	14.55	13.35	14.65	14.11
1.886	-0.64	0.64	P62908	40S ribosomal protein S3	Rps3	1467	36	36	36	93.8	93.8	93.8	26.674	0.0000	278.55	445340000	1176	19.60	19.54	18.91	19.27	20.07	19.95	19.71	20.14
0.451	-0.64	0.64	Q6SJQ0	CMRF35-like molecule 8	Cd300a	2657	6	6	6	18.6	18.6	18.6	35.629	0.0000	16.262	5320100	42	14.64	12.29	14.75	14.64	14.46	14.62	14.40	15.41
0.756	-0.64	0.64	P62743	AP-2 complex subunit sigma	Ap2s1	1439	5	5	5	27.5	27.5	27.5	17.018	0.0000	8.9936	10934000	106	14.89	16.17	15.04	15.61	16.35	16.28	15.17	16.47
0.858	-0.65	0.64	Q99ME9	Nucleolar GTP-binding protein 1	Gtpbp4	4464	15	15	15	24.8	24.8	24.8	74.112	0.0000	21.193	14642000	111	14.77	15.05	16.35	15.36	15.75	16.27	15.76	16.33
0.820	-0.65	0.64	Q9D6M3	Mitochondrial glutamate carrier 1	Slc25a22	5003	11	11	9	38.1	38.1	31.6	34.67	0.0000	31.752	16268000	98	15.30	15.59	13.85	14.73	15.29	15.41	15.64	15.70
0.889	-0.65	0.64	B2RUP2	Protein unc-13 homolog D	Unc13d	80	16	16	16	21	21	21	123.12	0.0000	73.081	25492000	191	16.90	16.75	15.71	17.15	16.76	17.66	17.33	17.34
0.243	-0.65	0.64	Q91YM4	Protein TBRG4	Tbrg4	4166	5	5	5	13.3	13.3	13.3	71.513	0.0000	8.0836	2821800	31	15.17	13.53	11.85	11.50	13.64	11.92	15.18	13.90
0.921	-0.65	0.64	Q9CZX9	ER membrane protein complex subunit 4	Emc4	4847	7	7	7	32.2	32.2	32.2	20.116	0.0000	30.724	11052000	96	15.04	15.60	14.12	15.19	15.29	15.89	15.39	15.98
0.511	-0.65	0.64	Q99LB0	Deoxynucleotidyltransferase terminal-interacting protein 1	Dnttip1	4412	7	7	7	30.5	30.5	30.5	36.852	0.0000	57.112	8053800	70	13.86	15.49	14.24	15.70	14.91	14.81	16.31	15.84
0.376	-0.65	0.64	P07339	Cathepsin D;Cathepsin D light chain;Cathepsin D heavy chain	CTSD	669	9	7	7	25.7	18.7	18.7	44.552	0.0000	13.487	5924600	56	14.80	14.73	12.34	14.02	13.88	14.98	15.34	12.88
1.025	-0.65	0.64	P51150	Ras-related protein Rab-7a	Rab7a	1191	25	25	25	87.9	87.9	87.9	23.489	0.0000	323.31	445930000	942	19.44	20.09	18.67	19.49	20.14	20.40	19.66	20.11
0.567	-0.65	0.64	Q8R332	Nucleoporin p58/p45	Nupl1	3834	6	6	5	12.8	12.8	10.7	59.445	0.0000	45.467	10453000	117	14.64	14.88	12.95	14.99	14.80	15.70	15.05	14.53
1.021	-0.65	0.64	Q9WV02	RNA-binding motif protein, X chromosome;RNA-binding motif protein, X	Rbmx	5727	20	3	3	39.1	7.2	7.2	42.3	0.0024	3.0055	771250	7	12.77	12.30	12.54	12.94	13.26	13.35	14.00	12.54
1.144	-0.65	0.64	P23298	Protein kinase C eta type	Prkch	882	4	4	4	7.5	7.5	7.5	77.918	0.0000	8.3028	2952600	44	13.47	13.03	13.25	13.71	13.63	13.75	14.79	13.90
0.305	-0.65	0.64	Q62087	Serum paraoxonase/lactonase 3	Pon3	2354	13	13	13	52	52	52	39.351	0.0000	224.9	110830000	406	18.51	19.06	15.23	18.66	18.40	18.99	18.22	18.47
0.523	-0.66	0.63	Q8VDP6	CDP-diacylglycerol--inositol 3-phosphatidyltransferase	Cdipt	3966	3	3	3	10.8	10.8	10.8	23.599	0.0000	6.7674	9519800	69	16.03	16.55	14.21	14.55	16.10	16.30	15.82	15.76
0.612	-0.66	0.63	P23198	Chromobox protein homolog 3	Cbx3	880	5	5	4	36.6	36.6	27.9	20.855	0.0000	19.461	12422000	118	15.51	16.04	13.90	15.77	15.75	16.33	15.60	16.17
1.354	-0.66	0.63	Q91WN1	DnaJ homolog subfamily C member 9	Dnajc9	4122	16	16	16	62.9	62.9	62.9	30.059	0.0000	109.91	21993000	261	15.45	16.30	15.49	15.25	16.41	16.35	15.93	16.45
0.738	-0.66	0.63	P52431	DNA polymerase delta catalytic subunit	Pold1	1216	28	28	28	37.4	37.4	37.4	123.79	0.0000	71.693	25988000	134	16.12	15.65	16.63	16.59	17.10	15.99	17.79	16.76
0.461	-0.66	0.63	Q3TB82	Pleckstrin homology domain-containing family F member 1	Plekhf1	1817	2	2	2	8.2	8.2	8.2	31.157	0.0022	3.0394	3958000	17	14.44	15.61	13.35	14.02	15.01	15.36	15.89	13.81
0.808	-0.66	0.63	Q9CR02	Translation machinery-associated protein 16	Tma16	4654	7	7	7	28.1	28.1	28.1	25.782	0.0000	23.363	7080300	75	15.14	15.35	14.68	14.32	16.08	15.54	14.59	15.93
0.928	-0.67	0.63	Q3UHX2	28 kDa heat- and acid-stable phosphoprotein	Pdap1	1977	11	11	11	41.4	41.4	41.4	20.605	0.0000	42.69	8709200	87	13.52	14.72	13.83	14.34	15.43	14.49	14.86	14.30
0.742	-0.67	0.63	Q61545	RNA-binding protein EWS	Ewsr1	2310	5	5	5	11.3	11.3	11.3	68.461	0.0000	95.176	18932000	148	15.88	16.16	14.67	16.20	16.19	15.82	16.54	17.01
1.696	-0.67	0.63	P27048;P63	Small nuclear ribonucleoprotein-associated protein B;Small nuclear ribo	Snrpb;Snrpn	933	8	8	8	30.7	30.7	30.7	23.656	0.0000	18.195	92489000	255	18.16	18.40	17.82	17.69	18.81	18.43	18.49	19.02
1.288	-0.67	0.63	P85094	Isochorismatase domain-containing protein 2A, mitochondrial	Isoc2a	1615	10	10	10	76.2	76.2	76.2	22.417	0.0000	189.76	55052000	224	16.91	17.30	16.62	17.48	17.66	17.81	18.24	17.29
0.644	-0.67	0.63	P12787	Cytochrome c oxidase subunit 5A, mitochondrial	Cox5a	758	7	7	7	59.6	59.6	59.6	16.101	0.0000	77.355	21985000	102	16.24	17.37	15.99	17.17	17.07	17.87	16.47	18.03
1.151	-0.67	0.63	P62878	E3 ubiquitin-protein ligase RBX1;E3 ubiquitin-protein ligase RBX1, N-te	Rbx1	1461	4	4	4	33.3	33.3	33.3	12.274	0.0000	48.521	23257000	165	16.81	17.07	16.29	15.88	16.83	17.48	17.03	17.37
1.007	-0.67	0.63	Q9QUN3	B-cell linker protein	Blnk	5520	8	8	8	20.8	20.8	20.8	50.67	0.0000	49.453	7963400	81	15.05	15.65	15.78	15.65	16.35	16.41	15.33	16.72
1.510	-0.67	0.63	P0DOV1			714	27	16	5	64.5	39.3	14.4	46.977	0.0000	180.86	53775000	382	16.78	16.59	17.14	17.14	17.24	17.28	18.04	17.78
0.644	-0.67	0.63	Q8K1H1	Tudor domain-containing protein 7	Tdrd7	3627	7	7	7	11.3	11.3	11.3	122.17	0.0000	43.644	6371500	62	14.44	14.05	12.63	14.92	14.45	14.82	14.72	14.74
1.252	-0.67	0.63	Q60817;P70	Nascent polypeptide-associated complex subunit alpha;Nascent polype	Naca	1581	6	6	6	28.8	28.8	28.8	23.384	0.0000	160.29	20276000	133	16.55	16.41	15.50	16.33	16.53	16.88	16.79	17.28
1.336	-0.67	0.63	P62320	Small nuclear ribonucleoprotein Sm D3	Snrpd3	1424	9	9	9	58.7	58.7	58.7	13.916	0.0000	143.76	117300000	339	18.85	18.89	18.35	19.37	19.40	20.03	19.26	19.56
0.473	-0.67	0.63	P51949	CDK-activating kinase assembly factor MAT1	Mnat1	1211	6	6	6	26.9	26.9	26.9	35.848	0.0000	17.264	2742600	42	12.43	14.33	13.58	12.02	13.75	12.85	14.66	13.79
0.624	-0.67	0.63	Q8VCH8	UBX domain-containing protein 4	Ubxn4	3922	9	9	9	23.3	23.3	23.3	56.471	0.0000	24.615	18473000	100	16.53	16.67	14.63	16.68	16.77	17.11	16.80	16.52
0.932	-0.68	0.63	P97386	DNA ligase 3	Lig3	1642	8	8	8	9.7	9.7	9.7	113.07	0.0000	16.727	7552000	51	15.22	15.07	14.27	15.61	15.21	16.10	16.15	15.42
3.056	-0.68	0.63	P32067	Lupus La protein homolog	Ssb	995	28	28	28	62.7	62.7	62.7	47.756	0.0000	198.05	118300000	749	16.98	17.10	17.26	17.27	17.67	17.97	17.69	17.98
1.493	-0.68	0.63	Q8CBW3	Abl interactor 1	Abi1	3439	25	25	18	50.3	50.3	43.5	52.287	0.0000	323.31	168570000	811	18.08	18.83	18.77	18.27	19.32	19.44	18.71	19.19
0.717	-0.68	0.63	Q689Z5	Protein strawberry notch homolog 1	Sbno1	2470	8	8	6	9.9	9.9	8.1	153.74	0.0000	22.285	4222200	54	13.75	14.18	12.65	14.24	13.92	14.26	14.18	15.18
0.623	-0.68	0.63	Q8R1S4	Metastasis suppressor protein 1	Mtss1	3798	14	14	14	28.5	28.5	28.5	82.407	0.0000	59.936	11814000	106	14.65	14.70	16.44	15.04	15.15	15.68	16.16	16.55
2.143	-0.68	0.63	Q9QYY8	Spastin	Spast	5581	20	20	20	44.1	44.1	44.1	66.455	0.0000	152.98	33581000	296	15.33	15.90	15.84	15.94	16.40	16.65	16.20	16.47
4.227	-0.68	0.63	P25444	40S ribosomal protein S2	Rps2	907	25	25	25	63.5	63.5	63.5	31.231	0.0000	242.4	503740000	937	19.85	19.81	19.66	19.85	20.40	20.57	20.37	20.54
0.840	-0.68	0.62	Q9CQW1	Synaptobrevin homolog YKT6	Ykt6	4642	19	19	19	85.4	85.4	85.4	22.314	0.0000	262.84	32124000	231	16.14	15.07	16.75	16.40	16.37	17.16	16.57	16.98
0.464	-0.68	0.62	O08691	Arginase-2, mitochondrial	Arg2	286	3	3	3	12.4	12.4	12.4	38.878	0.0000	7.1375	4324300	15	14.33	14.99	12.21	13.57	14.64	14.63	13.63	14.93
0.235	-0.68	0.62	Q5NBU8	XIAP-associated factor 1	Xaf1	2115	8	8	8	43.2	43.2	43.2	31.117	0.0000	27.147	10111000	91	12.19	13.11	16.18	14.80	15.36	13.61	16.65	13.39
0.411	-0.68	0.62	P69905	Hemoglobin subunit alpha	HBA1	1525	6	2	2	42.3	18.3	18.3	15.257	0.0002	5.3565	1957500	15	13.06	14.43	14.90	12.13	15.34	13.58	14.13	14.20
2.392	-0.68	0.62	P63323	40S ribosomal protein S12	Rps12	1504	12	12	12	72.7	72.7	72.7	14.525	0.0000	323.31	207020000	627	19.52	19.90	19.93	19.83	20.30	20.69	20.25	20.67
0.670	-0.68	0.62	P17156	Heat shock-related 70 kDa protein 2	Hspa2	819	26	8	8	42	17.5	17.5	69.641	0.0000	17.593	6599600	68	14.93	14.88	13.09	14.64	14.39	15.20	15.25	15.42
0.598	-0.69	0.62	Q8BJ34	Meiosis arrest female protein 1	Marf1	3070	7	7	7	4.9	4.9	4.9	192.06	0.0000	8.1017	2827200	26	13.23	13.73	13.63	12.94	12.98	13.97	15.44	13.88
0.466	-0.69	0.62	Q61301	Catenin alpha-2	Ctnna2	2289	4	2	2	7	3.4	3.4	105.28	0.0000	9.626	2942300	33	13.00	13.84	13.75	13.87	13.35	14.83	15.84	13.18
0.411	-0.69	0.62	Q9WTQ8	Mitochondrial import inner membrane translocase subunit Tim23	Timm23	5683	4	4	4	37.8	37.8	37.8	21.978	0.0000	105.28	10148000	64	16.75	15.97	13.83	14.42	15.78	15.20	16.13	16.61
1.480	-0.69	0.62	P35550	rRNA 2-O-methyltransferase fibrillarin	Fbl	1025	17	17	16	58.1	58.1	54.7	34.306	0.0000	134.48	59194000	397	16.53	16.86	17.31	16.90	17.10	17.88	17.47	17.90
0.368	-0.69	0.62	P0DJE0	Protein PET100 homolog, mitochondrial	Pet100	711	3	3	3	28.9	28.9	28.9	9.4097	0.0000	6.4546	10986000	50	17.04	16.31	13.55	16.78	16.36	16.86	16.62	16.59
0.664	-0.69	0.62	Q9CQZ6	NADH dehydrogenase [ubiquinone] 1 beta subcomplex subunit 3	Ndufb3	4652	5	5	5	42.3	42.3	42.3	11.692	0.0000	10.34	13581000	50	16.18	16.07	14.37	14.79	15.85	16.47	15.55	16.29
0.584	-0.69	0.62	P61963	DDB1- and CUL4-associated factor 7	Dcaf7	1394	4	4	4	12.6	12.6	12.6	38.926	0.0000	7.3118	4570900	54	14.96	15.22	13.63	12.92	15.06	14.99	14.85	14.59
1.052	-0.70	0.62	Q9QZ15	C-type lectin domain family 4 member A	Clec4a	5585	3	3	3	15.5	15.5	15.5	27.323	0.0000	56.048	6187300	46	14.53	14.44	15.61	14.51	15.40	15.84	14.94	15.68
0.321	-0.70	0.62	Q9JI13	Something about silencing protein 10	Utp3	5383	3	3	3	9.6	9.6	9.6	53.398	0.0000	8.6125	2569600	21	15.25	14.18	13.01	11.46	14.53	14.78	14.51	12.89
0.624	-0.70	0.62	Q0P678	Zinc finger CCCH domain-containing protein 18	Zc3h18	1776	5	5	5	6.2	6.2	6.2	105.69	0.0000	15.371	2654300	31	13.28	12.53	14.50	14.46	13.81	14.22	14.69	14.86
0.315	-0.70	0.62	Q9WTU0	Lysine-specific demethylase PHF2	Phf2	5687	4	3	3	4.4	3.1	3.1	120.81	0.0002	5.8266	3339800	16	13.11	14.08	14.09	12.22	15.16	14.98	14.52	11.64
0.435	-0.70	0.62	Q9JKV1	Proteasomal ubiquitin receptor ADRM1	Adrm1	5459	7	7	7	19.9	19.9	19.9	42.06	0.0000	65.122	31485000	118	17.19	17.04	14.41	17.44	17.05	17.48	17.36	16.99
0.948	-0.70	0.62	Q61160	FAS-associated death domain protein	Fadd	2262	9	9	9	50.2	50.2	50.2	22.96	0.0000	171.26	17985000	230	15.21	15.28	14.95	15.18	15.04	15.94	15.64	16.82

1.857	-0.70	0.61	P84228	Histone H3.2	Hist1h3b	1613	22	22	2	88.2	88.2	24.3	15.388	0.0000	323.31	2035400000	1151	22.18	22.40	22.89	22.95	23.36	23.35	23.07	23.44
0.456	-0.70	0.61	Q8K2H2	OTU domain-containing protein 6B	Otud6b	3659	8	8	8	32	32	32	33.758	0.0000	45.768	3704400	25	12.72	11.38	14.28	13.01	12.97	12.95	14.40	13.89
0.923	-0.70	0.61	Q9QZH3	Peptidyl-prolyl cis-trans isomerase E	Ppie	5597	4	4	4	15	15	15	33.448	0.0004	4.5204	2060900	39	12.46	13.86	13.42	12.93	13.38	13.58	14.07	14.45
0.889	-0.70	0.61	P48771	Cytochrome c oxidase subunit 7A2, mitochondrial	Cox7a2	1150	5	5	5	59	59	59	9.2908	0.0000	117.3	22155000	131	15.57	16.64	15.97	16.84	16.44	17.41	16.54	17.44
1.055	-0.70	0.61	Q6A065	Centrosomal protein of 170 kDa	Cep170	2497	19	19	19	18.7	18.7	18.7	175.05	0.0000	78.582	12656000	143	14.20	14.84	15.12	14.78	14.80	15.51	15.28	16.18
0.540	-0.70	0.61	Q9D975	Sulfiredoxin-1	Srxn1	5099	5	5	5	50.7	50.7	50.7	14.149	0.0000	43.041	20051000	181	16.71	17.47	15.14	15.54	17.14	17.08	16.10	17.38
0.771	-0.70	0.61	Q9DB15	39S ribosomal protein L12, mitochondrial	Mrpl12	5118	9	9	9	60.7	60.7	60.7	21.708	0.0000	109.92	11908000	140	15.48	15.84	15.13	14.97	15.82	16.93	15.04	16.45
0.716	-0.71	0.61	Q8BVK9	Sp110 nuclear body protein	Sp110	3243	15	15	15	35.1	35.1	35.1	50.14	0.0000	141.87	25379000	218	16.54	16.04	18.01	16.84	17.06	17.26	17.87	18.07
1.938	-0.71	0.61	Q8VDP2	UPF0428 protein CXorf56 homolog		3963	5	5	5	26.1	26.1	26.1	25.594	0.0000	10.416	4260200	56	14.45	14.40	13.63	13.99	14.84	14.90	14.88	14.69
0.570	-0.71	0.61	P84096	Rho-related GTP-binding protein RhoG	Rhog	1609	14	13	13	63.4	57.6	57.6	21.308	0.0000	256.67	335320000	573	19.39	19.18	17.11	19.59	19.39	19.74	19.32	19.65
1.000	-0.71	0.61	P26883	Peptidyl-prolyl cis-trans isomerase FKBP1A	Fkbp1a	929	6	6	6	59.3	59.3	59.3	11.922	0.0000	33.774	31960000	137	16.85	18.14	18.20	17.09	18.10	18.49	18.10	18.44
0.314	-0.71	0.61	Q9D958	Signal peptidase complex subunit 1	Spcs1	5096	3	3	3	13	13	13	18.186	0.0000	18.236	6345200	52	16.31	15.99	14.92	12.19	15.61	16.06	15.13	15.45
0.484	-0.71	0.61	Q8VCB1	Nucleoporin NDC1	Ndc1	3913	8	8	8	21.2	21.2	21.2	75.409	0.0000	66.266	20963000	120	16.27	16.41	13.73	15.84	16.04	16.53	16.81	15.71
1.218	-0.71	0.61	Q7TMF3	NADH dehydrogenase [ubiquinone] 1 alpha subcomplex subunit 12	Ndufa12	2743	13	13	13	88.3	88.3	88.3	17.086	0.0000	80.009	64198000	360	16.88	17.30	17.11	17.55	17.22	17.76	18.45	18.25
0.817	-0.72	0.61	P83870	PHD finger-like domain-containing protein 5A	Phf5a	1602	6	6	6	49.1	49.1	49.1	12.405	0.0000	23.853	15601000	166	14.82	15.27	13.60	15.29	15.37	15.79	15.72	14.98
0.716	-0.72	0.61	P58468	Protein FAM207A	Fam207a	1317	9	9	9	47	47	47	24.814	0.0000	20.39	12197000	76	14.43	15.03	15.56	14.85	14.74	16.29	15.19	16.53
0.951	-0.72	0.61	Q8K3J1	NADH dehydrogenase [ubiquinone] iron-sulfur protein 8, mitochondrial	Ndufs8	3703	12	12	12	45.8	45.8	45.8	24.038	0.0000	45.874	32180000	311	16.37	15.96	17.49	17.15	17.76	17.00	17.59	17.50
2.753	-0.72	0.61	A2AAY5	SH3 and PX domain-containing protein 2B	Sh3pxd2b	14	23	23	23	35.4	35.4	35.4	101.52	0.0000	121.06	65141000	562	15.85	16.05	16.08	16.07	16.43	16.92	16.63	16.95
1.943	-0.72	0.61	O35972	39S ribosomal protein L23, mitochondrial	Mrpl23	416	7	7	7	47.9	47.9	47.9	17.121	0.0000	70.336	23719000	136	15.77	16.21	16.42	15.81	16.50	17.08	16.85	16.67
0.288	-0.72	0.61	P61514	60S ribosomal protein L37a	Rpl37a	1386	8	8	8	73.9	73.9	73.9	10.275	0.0000	133.91	74001000	258	18.09	18.17	14.33	18.34	18.05	18.68	16.85	18.23
0.707	-0.72	0.61	Q99MR3	Solute carrier family 12 member 9	Slc12a9	4473	7	7	7	12.1	12.1	12.1	96.329	0.0000	143.22	17329000	137	16.40	14.48	14.64	16.07	16.04	16.17	16.33	15.94
3.462	-0.72	0.61	P35922	Fragile X mental retardation protein 1 homolog	Fmr1	1035	13	12	11	30.3	29	25.9	68.988	0.0000	103.18	13355000	185	14.99	15.20	15.14	14.98	15.70	15.95	15.62	15.94
0.981	-0.72	0.61	O88736	3-keto-steroid reductase	Hsd17b7	563	4	4	4	21.3	21.3	21.3	37.316	0.0000	64.009	6168500	65	14.80	15.07	13.95	15.02	14.86	15.38	15.30	16.18
0.794	-0.73	0.60	Q3UDR8	Protein YIPF3;Protein YIPF3, N-terminally processed	Yipf3	1950	7	7	7	23.3	23.3	23.3	37.998	0.0000	28.135	7553900	43	14.93	15.18	13.37	14.80	15.16	15.08	15.90	15.05
1.012	-0.73	0.60	P53702	Cytochrome c-type heme lyase	Hccs	1236	5	5	5	26.8	26.8	26.8	30.977	0.0000	20.695	3948300	47	14.26	13.39	14.30	13.66	14.99	13.98	15.25	14.31
1.168	-0.73	0.60	P60335	Poly(rC)-binding protein 1	Pcbp1	1354	23	23	17	94.1	94.1	82	37.497	0.0000	323.31	664580000	1379	20.14	20.29	19.00	20.19	20.34	20.94	20.67	20.58
0.724	-0.73	0.60	P50427	Steryl-sulfatase	Sts	1180	18	18	18	31.1	31.1	31.1	66.59	0.0000	92.507	39256000	218	17.40	17.46	15.53	17.34	17.41	18.08	17.48	17.67
1.042	-0.73	0.60	Q8R0A0	General transcription factor IIF subunit 2	Gtf2f2	3753	13	13	13	52.6	52.6	52.6	28.381	0.0000	215.02	89164000	383	17.23	18.11	17.24	16.99	17.60	18.75	17.78	18.37
2.668	-0.73	0.60	P63024	Vesicle-associated membrane protein 3	Vamp3	1480	8	8	3	67	67	32	11.48	0.0000	197.3	64606000	349	17.81	17.68	17.74	17.54	18.27	18.71	18.57	18.14
1.951	-0.73	0.60	P0C0A3	Charged multivesicular body protein 6	Chmp6	704	6	6	6	29.5	29.5	29.5	23.415	0.0000	43.543	21639000	161	16.06	15.58	15.82	15.79	16.04	16.64	16.66	16.85
1.268	-0.73	0.60	Q6R891	Neurabin-2	Ppp1r9b	2654	15	15	15	30	30	30	89.519	0.0000	170.53	17114000	230	14.91	15.34	15.71	15.30	15.92	16.55	15.38	16.36
1.552	-0.73	0.60	Q62419	Endophilin-A2	Sh3gl1	2388	25	25	25	58.2	58.2	58.2	41.518	0.0000	198.71	74183000	411	16.87	17.60	17.38	16.90	18.07	18.07	17.38	18.16
1.479	-0.74	0.60	Q9DCF9	Translocon-associated protein subunit gamma	Ssr3	5211	6	6	6	20	20	20	21.064	0.0000	215.16	37449000	108	17.83	17.96	17.09	16.98	18.37	18.31	18.19	17.93
0.337	-0.74	0.60	Q9CR27	WASH complex subunit CCDC53	Ccdc53	4662	5	5	3	35.1	35.1	22.7	21.092	0.0000	6.5824	6286400	34	15.38	12.02	16.19	15.49	15.50	15.48	15.63	15.42
0.798	-0.74	0.60	Q8VDC1	FYVE and coiled-coil domain-containing protein 1	Fyco1	3947	5	5	4	5.2	5.2	4.5	162.33	0.0000	38.132	2045400	37	13.37	13.44	12.35	13.75	13.50	13.27	14.71	14.37
0.403	-0.74	0.60	Q8JZY2	COMM domain-containing protein 10	Commd10	3587	6	6	6	35.1	35.1	35.1	22.812	0.0000	13.434	12217000	77	15.56	15.44	18.21	14.66	16.05	17.13	16.69	16.96
1.395	-0.74	0.60	Q9QUH0	Glutaredoxin-1	Glrx	5513	12	12	12	95.3	95.3	95.3	11.871	0.0000	323.31	113980000	359	18.34	18.90	19.01	17.94	18.93	19.23	19.45	19.54
0.329	-0.74	0.60	Q9CQV4	Protein FAM134C	Fam134c	4637	4	4	4	17.4	17.4	17.4	51.637	0.0000	65.233	19836000	115	16.57	16.40	12.88	16.69	16.45	16.69	16.80	15.57
1.942	-0.74	0.60	Q9JIK5	Nucleolar RNA helicase 2	Ddx21	5403	36	36	36	53.1	53.1	53.1	93.55	0.0000	189.68	70790000	635	16.07	16.20	16.90	16.49	17.00	17.34	16.99	17.31
0.550	-0.74	0.60	Q8R1X6	Spartin	Spg20	3803	8	8	7	20.3	20.3	17.9	72.654	0.0000	31.108	4202500	66	12.19	12.65	14.75	13.11	13.05	14.15	14.15	14.33
0.997	-0.75	0.59	Q8R1T1	Charged multivesicular body protein 7	Chmp7	3799	8	8	8	25.7	25.7	25.7	50.632	0.0000	67.12	7570600	93	14.41	14.31	12.92	13.85	14.24	15.12	14.64	14.50
2.186	-0.75	0.59	Q8K363	ATP-dependent RNA helicase DDX18	Ddx18	3692	13	13	13	27.1	27.1	27.1	74.18	0.0000	34.467	12521000	115	14.62	14.97	15.24	14.53	15.33	15.65	15.69	15.68
1.181	-0.76	0.59	Q8C163	Nuclease EXOG, mitochondrial	Exog	3354	14	14	13	50.8	50.8	48.6	41.383	0.0000	100.84	14872000	177	14.94	15.22	14.18	15.11	15.16	15.25	16.12	15.94
1.553	-0.76	0.59	P40630	Transcription factor A, mitochondrial	Tfam	1063	7	7	7	36.6	36.6	36.6	27.987	0.0000	50.005	4924200	85	13.87	14.51	14.10	14.34	14.80	15.15	14.44	15.47
0.962	-0.76	0.59	Q99MR1	PERQ amino acid-rich with GYF domain-containing protein 1	Gigyf1	4472	4	4	4	6.3	6.3	6.3	116.24	0.0000	17.48	3223800	33	14.05	14.55	14.17	13.52	14.09	15.72	14.60	14.90
1.024	-0.76	0.59	Q9R207	Nibrin	Nbn	5668	4	4	4	6.7	6.7	6.7	83.794	0.0000	22.445	4898200	67	14.51	14.91	13.27	14.78	15.15	15.06	14.98	15.32
1.165	-0.76	0.59	Q9CQA3	Succinate dehydrogenase [ubiquinone] iron-sulfur subunit, mitochondria	Sdhb	4578	19	19	19	46.8	46.8	46.8	31.814	0.0000	97.322	131240000	571	17.54	16.99	18.16	17.85	18.46	17.73	18.85	18.55
2.053	-0.76	0.59	Q91YX0	Protein THEMIS2	Themis2	4183	19	19	19	45.2	45.2	45.2	74.377	0.0000	188.87	37600000	259	16.07	16.03	16.34	16.61	16.69	16.96	17.01	17.41
0.637	-0.76	0.59	P70406	Mitochondrial uncoupling protein 2	Ucp2	1568	2	2	2	14.2	14.2	14.2	33.373	0.0000	18.869	533210	11	11.49	13.63	12.50	12.64	13.03	14.05	12.43	13.80
1.925	-0.76	0.59	D3YXK2	Scaffold attachment factor B1	Safb	210	22	22	18	28.4	28.4	21.1	105.1	0.0000	262.51	21204000	249	14.55	15.04	15.06	14.95	15.20	15.70	15.70	16.07
1.270	-0.77	0.59	Q8CI08	SLAIN motif-containing protein 2	Slain2	3536	10	10	9	20.3	20.3	20.3	62.377	0.0000	19.523	4670700	23	13.63	13.83	12.60	13.31	13.75	14.02	14.58	14.08
0.463	-0.77	0.59	Q9ERI6	Retinol dehydrogenase 14	Rdh14	5312	7	7	7	33.5	33.5	33.5	36.365	0.0000	13.467	4010100	20	13.32	12.69	15.72	12.54	14.23	14.67	14.31	14.14
1.831	-0.77	0.59	P63321	Ras-related protein Ral-A	Rala	1503	10	5	5	51	26.7	26.7	23.553	0.0000	26.016	25829000	172	15.88	16.19	15.32	16.29	16.68	16.82	16.72	16.52
1.182	-0.77	0.59	O08709	Peroxiredoxin-6	Prdx6	288	22	22	22	82.6	82.6	82.6	24.87	0.0000	120.21	140420000	555	18.14	18.57	17.37	18.17	18.97	19.27	18.17	18.91
0.811	-0.77	0.59	Q8BPG6	Sulfatase-modifying factor 2	Sumf2	3164	3	3	3	13.6	13.6	13.6	34.737	0.0000	15.254	9776500	82	15.54	16.16	14.25	15.23	16.19	16.52	15.33	16.21
2.558	-0.77	0.59	Q60675	Laminin subunit alpha-2	Lama2	2192	5	5	5	2.5	2.5	2.5	343.81	0.0059	2.097	11051000	21	16.09	16.43	16.25	16.18	16.59	17.19	17.12	17.14
0.940	-0.77	0.59	P70441	Na(+)/H(+) exchange regulatory cofactor NHE-RF1	Slc9a3r1	1574	18	18	18	61.7	61.7	61.7	38.6	0.0000	113.01	22676000	277	15.21	15.70	16.42	15.03	16.22	16.89	15.63	16.70
0.918	-0.77	0.59	Q8CFB4	Guanylate-binding protein 5	Gbp5	3478	10	9	9	30	26.8	26.8	66.929	0.0000	22.033	3520600	39	12.58	12.71	13.37	13.50	13.58	13.06	14.78	13.83
0.782	-0.77	0.59	P54923	[Protein ADP-ribosylarginine] hydrolase	Adprh	1262	15	15	15	58.6	58.6	58.6	40.068	0.0000	167.14	40939000	205	16.85	17.56	15.82	15.74	17.52	17.75	16.86	16.91
1.066	-0.77	0.59	Q56A08	G patch domain and KOW motifs-containing protein	Gpkow	2088	15	15	15	37.1	37.1	37.1	53.831	0.0000	35.408	17841000	163	14.79	15.46	13.91	15.20	15.40	16.07	15.61	15.37
1.400	-0.77	0.58	P62317	Small nuclear ribonucleoprotein Sm D2	Snrpd2	1423	14	14	14	70.3	70.3	70.3	13.527	0.0000	186	185460000	438	18.63	19.22	18.96	18.29	19.15	19.92	19.20	19.93
0.346	-0.78	0.58	Q61176	Arginase-1	Arg1	2268	3	3	3	11.5	11.5	11.5	34.807	0.0002	5.2487	4039200	11	13.57	15.67	13.39	13.10	12.65	15.85	14.46	15.88
0.819	-0.78	0.58	Q9D832	DnaJ homolog subfamily B member 4	Dnajb4	5049	6	5	5	28.8	25.2	25.2	37.781	0.0000	11.215	8238600	49	14.31	16.27	15.09	14.56	16.23	15.34	15.94	15.83
0.692	-0.78	0.58	Q3TWL2	Type 1 phosphatidylinositol 4,5-bisphosphate 4-phosphatase	Tmem55b	1882	5	4	4	21.5	18	18	30.047	0.0000	31.298	8913000	89	15.34	15.57	14.41	13.82	16.35	15.09	15.97	14.83
1.195	-0.78	0.58	Q64213	Splicing factor 1	Sf1	2423	24	24	24	31.2	31.2	31.2	70.402	0.0000	183.7	104770000	592	16.89	17.67	16.99	16.64	17.76	18.45	17.17	17.92
2.379	-0.78	0.58	Q640N3	Rho GTPase-activating protein 30	Arhgap30	2415	20	20	19	27.5	27.5	26.9	120.11	0.0000	85.156	25242000	230	15.37	15.30	14.96	15.44	15.75	16.19	15.90	16.35
0.809	-0.78	0.58	Q3UMT1	Protein phosphatase 1 regulatory subunit 12C	Ppp1r12c	2002	14	14	14	28	28	28	84.684	0.0000	27.179	5494500	28	13.23	14.21	14.56	14.20	14.82	13.78	15.12	15.60
0.352	-0.78	0.58	Q9JLQ0	CD2-associated protein	Cd2ap	5487	14	14	13	34.5	34.5	31.4	70.449	0.0000	56.774	3779500	25	11.22	12.09	12.50	11.30	12.73	11.57	15.02	10.91
1.254	-0.78	0.58	Q8C3P7	N6-adenosine-methyltransferase subunit METTL3	Mettl3	3377	3	3	3	7.1	7.1	7.1	64.615	0.0000	8.0361	4409500	40	15.43	15.75	14.27	15.36	15.89	16.20	15.85	15.99
1.137	-0.78	0.58	Q8R550	SH3 domain-containing kinase-binding protein 1	Sh3kbp1	3879	24	24	24	37.1	37.1	37.1	78.169	0.0000	122.6	21342000	215	14.70	15.54	15.96	14.70	16.32	16.02	16.17	15.53
0.938	-0.78	0.58	Q06185	ATP synthase subunit e, mitochondrial	Atp5i	1750	7	7	7	67.6	67.6	67.6	8.2355	0.0000	28.136	71789000	159	18.25	18.28	16.71	18.13	18.39	19.19	18.38	18.55
2.053	-0.78	0.58	O88939	Zinc finger and BTB domain-containing protein 7A	Zbtb7a	585	4	4	4	10.4	10.4	10.4	60.28	0.0000	87.153	9307600	90	15.44	15.56	14.91	15.77	16.10	16.33	15.99	16.39
1.630	-0.78	0.58	P61358	60S ribosomal protein L27	Rpl27	1384	14	14	14	60.3	60.3	60.3	15.798	0.0000	225.62	374280000	648	19.29	19.62	18.88	19.98	19.97	20.16	20.50	20.27
2.572	-0.79	0.58	Q9Z0H1	WD repeat-containing protein 46	Wdr46	5769	8	8	8	17.8	17.8	17.8	69.047	0.0000	58.189	5702600	106	14.35	14.38	14.43	14.03	14.72	15.33	15.23	15.06
2.030	-0.79	0.58	P62897	Cytochrome c, somatic	Cycs	1465	14	14	8	67.6	67.6	41	11.605	0.0000	172.43	59130000	289	16.67	16.89	17.15	17.08	17.87	17.55	17.36	18.17
1.708	-0.79	0.58	Q91ZA3	Propionyl-CoA carboxylase alpha chain, mitochondrial	Pcca	4192	48	48	48	69.5	69.5	69.5	79.921	0.0000	323.31	176390000	1070	17.33	16.95	16.83	17.02	18.36	17.72	17.94	17.27
1.762	-0.79	0.58	Q62351	Transferrin receptor protein 1	Tfrc	2381	26	26	26	45.6	45.6	45.6	85.73	0.0000	176.95	54471000	297	16.48	16.24	16.94	16.85	17.75	17.60	17.40	16.93
1.024	-0.79	0.58	P63242;Q8E	Eukaryotic translation initiation factor 5A-1;Eukaryotic translation initiatic	Eif5a;Eif5a2	1497	13	13	13	77.3	77.3	77.3	16.832	0.0000	216.41	45973000	292	16.03	17.31	17.10	17.40	17.31	18.38	17.42	17.90
1.575	-0.79	0.58	Q8BH43	Wiskott-Aldrich syndrome protein family member 2	Wasf2	3009	25	25	25	37.8	37.8	37.8	54.073	0.0000	190.35	163500000	691	17.39	18.17	17.91	17.64	18.32	18.87	18.10	18.99
0.363	-0.79	0.58	Q9DCC7	Isochorismatase domain-containing protein 2B, mitochondrial	Isoc2b	5205	2	2	2	9	9	9	23.151	0.0000	6.2435	363180	6	11.30	13.27	15.08	11.71	13.45	14.74	13.44	12.90
0.583	-0.79	0.58	Q8VDD9	PH-interacting protein	Phip	3950	7	7	7	4.6	4.6	4.6	206.72	0.0000	14.054	11630000	52	15.98	16.39	14.01	16.59	16.05	17.18	16.59	16.32
0.962	-0.79	0.58	Q80TE0	RNA polymerase II-associated protein 1	Rpap1	2802	7	7	7	6.7	6.7	6.7	155.27	0.0000	47.053	4565600	58	14.40	14.24	13.29	14.92	14.59	15.11	15.67	14.64
2.357	-0.79	0.58	P51880	Fatty acid-binding protein, brain	Fabp7	1207	5	5	5	45.5	45.5	45.5	14.893	0.0000	13.053	9114800	110	15.02	15.31	15.34	14.63	15.74	16.07	15.78	15.89
0.708	-0.80	0.58	Q61048	WW domain-binding protein 4	Wbp4	2243	7	7	7	24.5	24.5	24.5	42.136	0.0000	50.369	84338000	52	18.64	20.05	18.78	18.63	20.94	20.02	19.21	19.12
2.004	-0.80	0.58	Q3TN34	MICAL-like protein 2	Micall2	1864	10	10	10	15.9	15.9	15.9	108.29	0.0000	23.121	3203100	46	13.93	13.37	13.64	13.45	13.87	14.50	14.62	14.58
2.383	-0.80	0.58	Q8VEK3	Heterogeneous nuclear ribonucleoprotein U	Hnrnpu	4011	43	43	43	46.5	46.5	46.5	87.917	0.0000	323.31	550140000	1607	18.92	19.21	19.21	19.34	19.57	20.09	19.90	20.29
0.513	-0.80	0.57	Q8R404	Protein QIL1	Qil1	3861	6	6	6	86.6	86.6	86.6	13.373	0.0000	225.69	29793000	210	16.63	16.61	16.39	13.98	17.13	15.89	16.55	17.24
0.557	-0.80	0.57	Q9D3D9	ATP synthase subunit delta, mitochondrial	Atp5d	4964	2	2	2	13.7	13.7	13.7	17.6	0.0004	4.7723	4285100	42	14.36	15.40	13.88	14.38	15.73	16.64	14.96	13.87
1.249	-0.80	0.57	P26187	Methylated-DNA--protein-cysteine methyltransferase	Mgmt	919	5	5	5	30.3	30.3	30.3	22.434	0.0000	10.916	3064900	53	13.29	14.43	13.83	13.28	14.25	14.49	14.21	15.09
1.463	-0.80	0.57	Q8VDP4	Cell cycle and apoptosis regulator protein 2	Ccar2	3965	31	31	31	41.1	41.1	41.1	103	0.0000	220.89	60400000	487	16.13	16.45	15.21	16.29	16.54	16.94	16.89	16.92
1.571	-0.80	0.57	P26231	Catenin alpha-1	Ctnna1	920	24	24	22	43	43	39.2	100.11	0.0000	260.51	20505000	219	14.74	14.73	14.69	15.34	15.00	15.86	15.94	15.92
1.689	-0.80	0.57	P35980	60S ribosomal protein L18	Rpl18	1038	15	15	15	52.7	52.7	52.7	21.644	0.0000	111.05	182830000	420	19.13	19.04	20.02	19.77	20.29	20.06	20.34	20.48
0.362	-0.81	0.57	Q9Z103	Activity-dependent neuroprotector homeobox protein	Adnp	5793	8	8	8	9.3	9.3	9.3	124.31	0.0000	9.0247	6397200	49	12.16	14.92	15.46	13.92	13.29	16.28	14.70	15.42
0.314	-0.81	0.57	P56213	FAD-linked sulfhydryl oxidase ALR	Gfer	1278	3	3	2	16.2	16.2	10.6	22.876	0.0000	8.1701	2900300	38	13.98	14.40	13.08	14.31	15.91	16.27	11.71	15.10
0.359	-0.81	0.57	Q64092	Transcription factor E3	Tfe3	2413	8	8	4	13.5	13.5	7.5	61.535	0.0000	9.8491	11468000	60	15.50	16.34	12.63	14.92	16.22	15.23	14.31	16.88
2.019	-0.81	0.57	Q4KMM3	Oxidation resistance protein 1	Oxr1	2062	16	16	16	26.6	26.6	26.6	95.911	0.0000	160.86	10938000	151	14.86	14.88	14.11	14.65	15.33	15.80	15.28	15.34
1.260	-0.81	0.57	Q3U0J8	TBC1 domain family member 2B	Tbc1d2b	1895	9	9	9	9.8	9.8	9.8	109.95	0.0000	19.851	8944500	74	14.68	14.18	14.58	15.63	15.48	15.74	15.90	15.21
1.191	-0.81	0.57	O35344	Importin subunit alpha-4	Kpna3	359	16	11	11	41.8	33	33	57.772	0.0000	201.6	20523000	119	15.95	17.10	15.98	17.02	16.98	17.63	17.61	17.08
1.157	-0.81	0.57	Q922Y1	UBX domain-containing protein 1	Ubxn1	4280	12	12	11	42.8	42.8	42.1	33.572	0.0000	186.62	25527000	167	16.33	16.85	15.86	16.42	17.28	17.43	16.30	17.71
1.667	-0.82	0.57	Q3TIV5	Zinc finger CCCH domain-containing protein 15	Zc3h15	1851	10	10	10	21.1	21.1	21.1	48.327	0.0000	20.268	8888400	120	13.76	14.75	14.53	14.59	15.26	15.46	14.80	15.37
1.379	-0.82	0.57	Q64191	N(4)-(beta-N-acetylglucosaminyl)-L-asparaginase;Glycosylasparaginase	Aga	2422	7	7	7	30.9	30.9	30.9	37.022	0.0000	109.75	21388000	172	15.34	16.70	15.94	16.35	16.97	17.03	16.53	17.05
1.074	-0.82	0.57	Q8C0G2	TRAF3-interacting JNK-activating modulator	Traf3ip3	3336	8	8	8	20.3	20.3	20.3	58.563	0.0000	14.646	9567400	29	15.89	16.54	16.63	15.57	16.29	16.96	16.89	17.76
1.195	-0.82	0.57	P08249	Malate dehydrogenase, mitochondrial	Mdh2	684	34	34	34	75.4	75.4	75.4	35.611	0.0000	323.31	1314700000	1758	20.37	20.41	19.16	20.10	20.89	21.39	20.61	20.42
0.458	-0.82	0.57	Q9CXZ1	NADH dehydrogenase [ubiquinone] iron-sulfur protein 4, mitochondrial	Ndufs4	4776	8	8	8	53.1	53.1	53.1	19.784	0.0000	20.09	4511800	35	13.04	16.46	13.81	13.67	14.45	15.61	15.42	14.77
1.000	-0.82	0.57	Q9D172	ES1 protein homolog, mitochondrial	D10Jhu81e	4899	17	17	17	69.2	69.2	69.2	28.09	0.0000	238.25	92740000	461	17.46	18.02	16.10	17.59	17.91	18.27	18.16	18.10
2.069	-0.82	0.57	Q9CY58	Plasminogen activator inhibitor 1 RNA-binding protein	Serbp1	4787	17	17	17	43.2	43.2	43.2	44.714	0.0000	267.23	67104000	297	17.08	17.67	17.02	17.11	17.92	18.49	17.91	17.85
3.645	-0.82	0.57	P14148	60S ribosomal protein L7	Rpl7	775	31	31	31	61.5	61.5	61.5	31.419	0.0000	141.86	424640000	881	19.29	19.31	19.60	19.43	20.18	20.05	20.28	20.41
1.736	-0.83	0.56	Q6P5B0	RRP12-like protein	Rrp12	2569	17	17	17	17.9	17.9	17.9	143.13	0.0000	91.951	13531000	145	14.79	14.83	14.26	15.09	15.53	15.05	15.84	15.87

0.509	-0.83	0.56	A2ASS6	Titin	Ttn		37	25	25	25	0.9	0.9	0.9	3906.4	0.0015	3.3944	109690000	136	17.48	17.87	19.49	16.13	18.98	19.10	17.99	18.21
3.114	-0.83	0.56	Q62523	Zyxin	Zyx		2401	9	9	9	27	27	27	60.545	0.0000	35.76	23305000	164	16.26	16.58	16.42	16.44	17.38	17.44	16.93	17.25
0.994	-0.83	0.56	Q922D4	Serine/threonine-protein phosphatase 6 regulatory subunit 3	Ppp6r3		4250	4	4	4	6.8	6.8	6.8	94.652	0.0000	16.464	15790000	41	16.96	16.97	15.23	16.70	17.09	17.27	17.57	17.25
1.415	-0.83	0.56	Q9D6J6	NADH dehydrogenase [ubiquinone] flavoprotein 2, mitochondrial	Ndufv2		4997	4	4	4	25.4	25.4	25.4	27.285	0.0000	27.903	2858600	39	12.95	13.97	13.71	14.06	14.38	14.12	14.50	15.01
0.543	-0.83	0.56	Q8BTW3	Exosome complex component MTR3	Exosc6		3209	10	10	10	44	44	44	28.37	0.0000	159.82	15029000	132	16.04	15.91	13.29	15.95	15.89	16.82	15.84	15.94
0.953	-0.83	0.56	Q8CH25	SAFB-like transcription modulator	Sltm		3518	15	15	15	15.3	15.3	15.3	116.92	0.0000	42.657	21399000	178	15.25	15.93	17.25	15.83	16.55	17.01	16.81	17.22
1.423	-0.83	0.56	Q9CR00	26S proteasome non-ATPase regulatory subunit 9	Psmd9		4653	8	8	7	45.5	45.5	38.7	24.72	0.0000	14.044	5821600	64	14.62	14.46	13.76	14.86	15.59	15.03	14.79	15.62
0.645	-0.83	0.56	E9Q6J5		Bod1l		242	8	8	8	3.3	3.3	3.3	327.45	0.0000	23.852	15061000	42	14.81	16.12	13.56	16.10	15.75	15.99	16.17	16.02
0.397	-0.84	0.56	Q9Z1Q2	Abhydrolase domain-containing protein 16A	Abhd16a		5817	16	16	16	36	36	36	63.085	0.0000	83.382	19348000	178	14.70	14.42	11.02	14.59	14.98	13.97	14.95	14.18
2.274	-0.84	0.56	Q9D883	Splicing factor U2AF 35 kDa subunit	U2af1		5054	14	14	10	56.9	56.9	43.9	27.815	0.0000	223.73	96165000	492	17.19	17.91	17.19	17.44	18.10	18.56	18.24	18.18
0.580	-0.84	0.56	Q3U308	Cytoplasmic tRNA 2-thiolation protein 2	Ctu2		1915	5	5	5	16.5	16.5	16.5	56.104	0.0000	36.117	14172000	73	16.79	16.26	13.76	16.09	16.28	16.67	16.78	16.53
0.779	-0.84	0.56	Q8BVF2	Phosducin-like protein 3	Pdcl3		3238	3	3	3	15	15	15	27.581	0.0000	12.659	5172000	33	14.58	13.53	15.34	15.94	15.39	15.93	15.60	15.83
1.531	-0.84	0.56	Q64674	Spermidine synthase	Srm		2457	14	14	14	62.3	62.3	62.3	33.995	0.0000	190.92	31995000	275	16.15	16.98	15.69	16.01	17.01	17.22	16.76	17.20
0.869	-0.84	0.56	O70439	Syntaxin-7	Stx7		499	11	11	11	51.7	51.7	51.7	29.82	0.0000	247.03	124720000	406	18.45	18.22	16.42	18.31	18.47	18.91	18.66	18.72
1.607	-0.84	0.56	Q8CHI8	E1A-binding protein p400	Ep400		3524	10	10	10	5.1	5.1	5.1	337.18	0.0000	34.668	5846300	88	13.55	13.96	14.69	14.20	14.62	15.24	14.74	15.16
0.658	-0.84	0.56	Q9CQJ4	E3 ubiquitin-protein ligase RING2	Rnf2		4605	4	4	3	17	17	12.8	37.623	0.0000	13.064	4194400	41	13.75	14.80	12.19	14.56	14.91	14.41	15.02	14.33
1.002	-0.85	0.56	Q8BX17	Gem-associated protein 5	Gemin5		3277	8	8	8	7.9	7.9	7.9	166.59	0.0000	34.591	11065000	69	15.03	15.64	13.97	15.83	15.65	15.96	16.14	16.10
1.328	-0.85	0.56	Q922Q1	Mitochondrial amidoxime reducing component 2	Marc2		4267	16	16	16	54.4	54.4	54.4	38.194	0.0000	102.51	42624000	269	16.39	16.42	15.05	16.23	17.00	16.61	16.98	16.89
1.895	-0.85	0.56	Q14CH1	Molybdenum cofactor sulfurase	Mocos		1789	32	32	32	54.9	54.9	54.9	95.012	0.0000	323.31	61334000	690	16.37	16.00	16.03	16.32	17.45	16.54	17.36	16.77
2.020	-0.85	0.56	Q69Z38	Pseudopodium-enriched atypical kinase 1	Peak1		2479	18	18	18	14.8	14.8	14.8	191.09	0.0000	36.037	10039000	99	14.06	13.48	14.34	13.92	14.39	14.93	14.86	15.02
1.106	-0.85	0.56	Q8R4Y4	Stabilin-1	Stab1		3876	21	21	21	10.6	10.6	10.6	276.25	0.0000	79.591	19707000	221	14.93	14.69	16.25	15.86	16.24	15.98	16.70	16.21
3.554	-0.85	0.56	O55098	Serine/threonine-protein kinase 10	Stk10		450	44	41	41	43.3	41	41	111.9	0.0000	198.76	60888000	550	16.10	16.46	16.14	16.38	16.95	17.11	17.13	17.29
1.687	-0.85	0.55	P27661	Histone H2AX	H2afx		939	21	21	8	81.1	81.1	47.6	15.142	0.0000	323.31	4640700000	2863	22.88	23.56	23.53	23.63	23.70	24.38	24.22	24.70
0.311	-0.85	0.55	A2A4P0	ATP-dependent RNA helicase DHX8	Dhx8		6	7	6	6	9	8.3	8.3	142.57	0.0000	10.048	14697000	33	16.99	16.29	13.68	16.09	17.50	14.07	18.22	16.67
1.625	-0.86	0.55	B2RX14	Terminal uridylyltransferase 4	Zcchc11		83	6	6	6	4.7	4.7	4.7	184.65	0.0000	25.522	3840200	61	13.65	13.58	13.12	14.13	14.51	14.48	14.94	13.99
2.079	-0.86	0.55	Q7TPV4	Myb-binding protein 1A	Mybbp1a		2772	55	55	55	46.4	46.4	46.4	152.04	0.0000	323.31	142510000	1255	16.73	16.85	17.34	17.44	17.58	17.95	18.08	18.20
0.456	-0.86	0.55	P70296	Phosphatidylethanolamine-binding protein 1;Hippocampal cholinergic ne	Pebp1		1549	9	9	9	85.6	85.6	85.6	20.83	0.0000	116.53	34555000	173	17.17	18.37	14.59	15.50	17.05	17.41	17.11	17.51
0.956	-0.86	0.55	P17751	Triosephosphate isomerase	Tpi1		830	34	34	34	93.2	93.2	93.2	26.712	0.0000	323.31	1546800000	2082	21.04	21.51	19.53	20.76	21.62	21.93	21.05	21.70
2.119	-0.87	0.55	Q8QZT1	Acetyl-CoA acetyltransferase, mitochondrial	Acat1		3735	31	31	31	72.4	72.4	72.4	44.816	0.0000	323.31	264330000	981	18.34	18.89	18.22	18.57	18.96	19.75	19.38	19.39
1.429	-0.87	0.55	Q61191	Host cell factor 1;HCF N-terminal chain 1;HCF N-terminal chain 2;HCF I	Hcfc1		2272	38	38	38	27.3	27.3	27.3	210.43	0.0000	286.01	103050000	694	16.88	17.26	16.17	17.53	17.52	18.18	17.81	17.81
0.899	-0.87	0.55	P50543	Protein S100-A11	S100a11		1186	5	5	5	51	51	51	11.083	0.0000	72.168	35048000	216	17.08	17.19	16.48	16.02	17.85	17.61	16.43	18.34
2.731	-0.87	0.55	Q9EQP2	EH domain-containing protein 4	Ehd4		5285	43	39	35	81.1	77.6	73.6	61.48	0.0000	323.31	558670000	1342	19.24	19.31	18.81	19.40	19.82	19.99	20.28	20.14
0.483	-0.87	0.55	Q8R0J7	Vacuolar protein sorting-associated protein 37B	Vps37b		3761	2	2	2	10.2	10.2	10.2	31.055	0.0000	23.262	4094900	38	12.02	15.34	13.71	15.07	15.28	15.17	15.18	13.99
0.739	-0.87	0.55	Q8VDV8	MIT domain-containing protein 1	Mitd1		3974	3	3	3	17.3	17.3	17.3	28.847	0.0000	9.5053	1761100	24	13.70	12.63	13.05	13.40	15.57	14.05	13.25	13.40
1.462	-0.87	0.55	Q8VCD5	Mediator of RNA polymerase II transcription subunit 17	Med17		3916	4	4	4	12.6	12.6	12.6	72.461	0.0000	29.518	1054800	30	13.35	13.00	13.33	12.94	13.75	14.76	13.37	14.22
2.603	-0.87	0.55	P84099	60S ribosomal protein L19	Rpl19		1610	19	19	19	56.1	56.1	56.1	23.466	0.0000	155.11	318280000	752	20.13	20.21	20.24	20.42	21.00	20.81	21.09	21.59
1.133	-0.88	0.55	Q61193	Ral guanine nucleotide dissociation stimulator-like 2	Rgl2		2273	11	11	11	23.4	23.4	23.4	83.825	0.0000	60.953	9141300	76	14.40	15.03	16.12	15.48	16.00	15.79	16.10	16.63
0.495	-0.88	0.54	Q8BHL5	Engulfment and cell motility protein 2	Elmo2		3045	23	17	17	42.2	38.1	38.1	83.886	0.0000	138.62	24623000	218	16.17	15.87	13.06	16.54	16.20	16.68	16.30	15.96
2.350	-0.88	0.54	Q9ERR7	15 kDa selenoprotein		42248	5316	6	6	6	30.2	30.2	30.2	17.806	0.0000	71.215	13516000	106	15.96	16.14	16.27	16.14	16.72	17.54	16.78	16.98
0.221	-0.88	0.54	Q9CQJ2	PIH1 domain-containing protein 1	Pih1d1		4604	6	6	5	24.1	24.1	21.7	32.208	0.0000	9.5625	25438000	30	15.86	18.40	13.96	14.00	18.54	13.02	17.60	16.59
0.630	-0.88	0.54	Q9JK81	UPF0160 protein MYG1, mitochondrial	Myg1		5445	14	14	14	46.1	46.1	46.1	42.722	0.0000	80.81	66371000	286	17.24	17.07	14.63	17.48	17.35	17.44	17.62	17.54
1.202	-0.88	0.54	Q8R035	Peptidyl-tRNA hydrolase ICT1, mitochondrial	Ict1		3745	9	9	9	55.3	55.3	55.3	23.477	0.0000	46.432	18715000	157	15.59	15.82	15.02	14.27	15.62	16.32	15.92	16.37
2.013	-0.89	0.54	Q60872	Eukaryotic translation initiation factor 1A	Eif1a		2218	11	11	2	61.8	61.8	7.6	16.502	0.0000	176.94	57202000	280	16.80	17.57	16.93	17.32	17.98	18.39	17.64	18.16
1.453	-0.89	0.54	Q8VEJ4	Notchless protein homolog 1	Nle1		4008	6	6	6	16.9	16.9	16.9	53.133	0.0000	36.132	4005900	67	14.66	14.42	13.45	13.84	14.88	15.37	15.13	14.54
2.209	-0.89	0.54	P27546	Microtubule-associated protein 4	Map4		934	18	18	18	26	26	26	117.43	0.0000	119.31	27982000	300	15.41	16.10	16.01	15.37	16.45	16.89	16.60	16.50
0.419	-0.89	0.54	Q60953	Protein PML	Pml		2227	10	10	10	16.5	16.5	16.5	98.241	0.0000	57.576	3143600	35	10.22	12.62	13.89	13.56	12.46	13.32	14.64	13.43
0.835	-0.89	0.54	O88952	Protein lin-7 homolog C	Lin7c		587	7	7	7	39.6	39.6	39.6	21.834	0.0000	14.873	10012000	125	15.15	15.88	16.25	15.03	16.27	17.14	15.28	17.19
1.331	-0.89	0.54	Q5PSV9	Mediator of DNA damage checkpoint protein 1	Mdc1		2124	4	4	4	3.7	3.7	3.7	184.67	0.0004	4.3099	1009000	20	11.06	12.38	11.98	11.65	12.06	12.58	12.99	13.03
1.108	-0.90	0.54	O70591	Prefoldin subunit 2	Pfdn2		517	3	3	3	22.7	22.7	22.7	16.534	0.0000	11.828	2877600	38	13.30	14.45	14.85	13.55	14.85	15.31	14.38	15.20
0.753	-0.90	0.54	O54790	Transcription factor MafG	Mafg		424	6	4	4	36.4	27.8	27.8	17.876	0.0000	8.817	3669000	42	13.09	14.88	15.47	14.05	14.85	14.76	15.87	15.62
1.386	-0.90	0.53	Q3UDK1	TRAF-type zinc finger domain-containing protein 1	Trafd1		1948	7	7	7	15.7	15.7	15.7	64.282	0.0000	22.342	6208700	78	14.77	14.86	13.92	15.49	15.39	16.01	15.57	15.67
2.000	-0.90	0.53	Q62036	Centrosomal protein of 131 kDa	Cep131		2348	5	5	5	6.9	6.9	6.9	120.31	0.0000	14.271	3076000	46	13.16	13.94	13.51	14.04	14.33	14.91	14.38	14.65
1.182	-0.90	0.53	Q6PAQ4	RNA exonuclease 4	Rexo4		2598	5	5	5	14.1	14.1	14.1	47.598	0.0000	9.6187	2474900	27	13.72	13.53	13.38	14.09	14.14	15.68	14.10	14.42
1.884	-0.91	0.53	P06797	Cathepsin L1;Cathepsin L1 heavy chain;Cathepsin L1 light chain	Ctsl		663	8	8	8	24	24	24	37.547	0.0000	76.164	14757000	128	15.41	16.04	15.60	14.97	16.46	16.15	16.26	16.77
3.613	-0.91	0.53	P12970	60S ribosomal protein L7a	Rpl7a		761	26	26	26	60.2	60.2	60.2	29.976	0.0000	323.31	465520000	828	19.00	19.25	19.52	19.34	20.14	20.32	20.15	20.14
1.368	-0.91	0.53	A2AWP8	Rho guanine nucleotide exchange factor 10-like protein	Arhgef10l		43	17	17	17	24	24	24	139.99	0.0000	107.79	23184000	192	15.40	15.52	14.28	15.93	16.06	16.33	16.16	16.21
1.952	-0.91	0.53	Q8BQ30	Phostensin	Ppp1r18		3170	17	17	17	29.1	29.1	29.1	65.629	0.0000	57.829	47880000	202	16.57	17.03	16.73	16.17	17.47	18.02	17.16	17.49
1.377	-0.91	0.53	G3X9J0		Sipa1l3		260	5	5	5	4.6	4.6	4.6	195.06	0.0000	14.975	1723100	35	12.31	13.32	12.99	12.65	12.96	14.03	14.23	13.70
1.663	-0.91	0.53	Q8VDD8	WAS protein family homolog 1	Wash1		3949	12	12	12	38.1	38.1	38.1	51.658	0.0000	98.808	19636000	186	15.33	15.48	16.11	14.98	15.87	16.66	16.44	16.58
1.804	-0.91	0.53	Q6PFQ7	Ras GTPase-activating protein 4	Rasa4		2628	28	28	28	46.3	46.3	46.3	90.059	0.0000	313.91	61063000	480	16.42	16.14	15.37	15.66	16.59	16.63	17.20	16.83
1.090	-0.92	0.53	Q8CC12	Codanin-1	Cdan1		3442	6	6	6	8.7	8.7	8.7	135.88	0.0000	68.247	10271000	105	15.18	14.88	16.20	15.01	15.48	16.07	17.02	16.36
1.239	-0.92	0.53	O54879	High mobility group protein B3	Hmgb3		429	17	15	15	58	53	53	23.01	0.0000	91.998	13268000	121	14.66	16.35	15.40	15.90	16.48	16.52	16.12	16.85
1.057	-0.92	0.53	P20664	DNA primase small subunit	Prim1		857	6	6	6	16.5	16.5	16.5	49.295	0.0000	7.0226	1913800	15	12.95	12.89	13.09	13.21	12.81	13.75	14.87	14.38
0.894	-0.92	0.53	Q3UMC0	Spermatogenesis-associated protein 5	Spata5		2000	13	13	13	21.1	21.1	21.1	97.255	0.0000	56.74	31962000	116	16.91	18.02	18.54	16.67	18.40	19.23	18.02	18.17
2.173	-0.92	0.53	P83882	60S ribosomal protein L36a	Rpl36a		1603	15	15	15	63.2	63.2	63.2	12.441	0.0000	32.767	132830000	468	18.76	19.06	18.67	19.20	19.62	19.62	19.72	20.40
1.155	-0.92	0.53	O35326	Serine/arginine-rich splicing factor 5	Srsf5		357	10	9	9	28.3	24.9	24.9	30.891	0.0000	77.042	29918000	258	15.57	16.34	16.77	16.12	17.27	17.63	16.14	17.44
1.798	-0.92	0.53	Q9Z204	Heterogeneous nuclear ribonucleoproteins C1/C2	Hnrnpc		5828	25	25	25	58.5	58.5	58.5	34.384	0.0000	131.49	164770000	512	18.38	18.63	17.60	18.42	19.13	19.48	18.76	19.36
0.519	-0.92	0.53	P08074	Carbonyl reductase [NADPH] 2	Cbr2		677	11	11	11	62.7	62.7	62.7	25.958	0.0000	164.27	6432400	68	13.33	12.94	16.22	14.08	15.07	15.69	14.04	15.46
0.404	-0.92	0.53	Q4VBF2	R3H domain-containing protein 4	R3hdm4		2072	3	3	3	17.9	17.9	17.9	29.659	0.0000	24.826	2140100	53	14.10	13.90	10.85	14.61	15.06	15.33	13.64	13.12
1.874	-0.92	0.53	Q62376	U1 small nuclear ribonucleoprotein 70 kDa	Snrnp70		2382	23	23	23	45.3	45.3	45.3	51.991	0.0000	150.02	52438000	393	16.57	17.28	16.24	16.69	17.68	17.24	17.56	17.98
0.565	-0.92	0.53	Q6PDN3	Myosin light chain kinase, smooth muscle;Myosin light chain kinase, sm	Mylk		2619	6	6	6	3.7	3.7	3.7	212.92	0.0027	2.6964	43992000	48	17.67	18.70	15.40	18.45	18.09	18.57	18.80	18.45
0.741	-0.93	0.53	Q8C0L6	Peroxisomal N(1)-acetyl-spermine/spermidine oxidase	Paox		3342	9	9	9	22.4	22.4	22.4	55.446	0.0000	56.023	10347000	110	14.71	15.28	12.80	15.36	15.31	15.51	15.84	15.21
3.131	-0.93	0.53	Q8BUH8	Sentrin-specific protease 7	Senp7		3224	4	4	4	5.3	5.3	5.3	116.34	0.0000	8.7879	3090400	20	13.41	13.17	12.78	13.10	13.89	14.23	14.03	14.02
1.110	-0.93	0.53	Q62432	Mothers against decapentaplegic homolog 2	Smad2		2392	7	7	6	22.9	22.9	20.6	52.265	0.0000	204.61	18092000	88	16.56	16.72	16.77	15.41	16.86	17.73	17.89	16.71
0.529	-0.93	0.52	Q8BG15	CTD small phosphatase-like protein 2	Ctdspl2		2958	3	3	3	6.9	6.9	6.9	52.812	0.0017	3.2981	2167600	17	13.61	12.78	13.96	14.40	15.29	15.36	12.41	15.40
1.520	-0.93	0.52	Q5BLK4	Terminal uridylyltransferase 7	Zcchc6		2095	19	19	19	17	17	17	169.1	0.0000	54.583	13271000	151	14.05	14.41	15.33	14.64	15.21	15.22	15.93	15.81
1.586	-0.93	0.52	P52293	Importin subunit alpha-1	Kpna2		1214	12	12	12	29.5	29.5	29.5	57.927	0.0000	129.25	19843000	218	14.87	15.39	15.92	15.67	15.81	16.33	16.85	16.60
1.595	-0.93	0.52	Q8VI33	Transcription initiation factor TFIID subunit 9	Taf9		4026	7	7	7	37.1	37.1	37.1	28.979	0.0000	34.103	12557000	81	15.15	15.85	15.28	14.50	16.35	16.43	15.93	15.81
1.112	-0.93	0.52	Q6NVE8	WD repeat-containing protein 44	Wdr44		2531	5	5	5	6.2	6.2	6.2	101.55	0.0000	6.8243	2953500	30	14.40	12.45	12.92	13.70	14.12	14.29	14.52	14.28
0.749	-0.93	0.52	Q04857	Collagen alpha-1(VI) chain	Col6a1		1735	5	5	5	7.4	7.4	7.4	108.49	0.0000	37.919	4404800	69	14.77	14.75	12.69	13.18	13.91	15.00	14.99	15.23
0.789	-0.94	0.52	Q80UZ2	Protein SDA1 homolog	Sdad1		2843	4	4	4	7	7	7	79.558	0.0000	11.531	3866800	39	14.49	14.82	13.94	15.14	15.98	15.05	14.35	16.77
1.818	-0.94	0.52	Q9ESV0	ATP-dependent RNA helicase DDX24	Ddx24		5347	8	8	8	13.3	13.3	13.3	96.428	0.0000	14.806	4939700	76	13.84	12.98	13.39	14.08	14.18	14.46	14.85	14.54
0.612	-0.94	0.52	Q8CHW4	Translation initiation factor eIF-2B subunit epsilon	Eif2b5		3533	13	13	13	23	23	23	80.085	0.0000	37.785	15445000	72	14.68	16.30	16.87	14.00	16.10	17.17	16.42	15.93
2.199	-0.94	0.52	P63001	Ras-related C3 botulinum toxin substrate 1	Rac1		1477	18	8	5	64.1	33.9	24.5	21.45	0.0000	35.548	267020000	512	19.21	19.10	18.64	18.26	19.87	19.73	19.55	19.82
2.485	-0.95	0.52	Q6PFD9	Nuclear pore complex protein Nup98-Nup96;Nuclear pore complex prot	Nup98		2627	44	44	44	32.8	32.8	32.8	197.24	0.0000	323.31	121540000	795	16.82	17.22	16.53	17.25	18.10	17.95	17.61	17.95
2.453	-0.95	0.52	P59759	MKL/myocardin-like protein 2	Mkl2		1343	10	10	9	13.1	13.1	11.9	117.55	0.0000	25.122	4337100	33	13.68	14.45	14.47	14.26	15.35	15.11	15.24	14.95
1.569	-0.95	0.52	Q9Z2B9	Ribosomal protein S6 kinase alpha-4	Rps6ka4		5838	11	11	11	18.6	18.6	18.6	85.651	0.0000	24.323	13469000	103	15.71	15.63	14.53	15.97	16.39	16.27	16.41	16.57
[truncated: 1,078,156 more chars]
